# Error Tolerance of Machine Learning Algorithms across Contemporary Biological Targets

**DOI:** 10.3390/molecules24112115

**Published:** 2019-06-04

**Authors:** Thomas M. Kaiser, Pieter B. Burger

**Affiliations:** 1 St Peter’s College, University of Oxford, New Inn Hall St, Oxford OX1 2DL, UK; 2 Department of Drug Discovery and Biomedical Sciences, College of Pharmacy, Medical University of South Carolina, 280 Calhoun St. MSC 141, Charleston, SC 29425-1410, USA; 3 Department of Chemistry, Emory University, 201 Dowman Drive, Atlanta, GA 30322, USA

**Keywords:** machine learning, error, FEP, anaplastic lymphoma kinase (ALK), Naïve Bayes Network, Random Forest, drug discovery, cheminformatics, Neural Network

## Abstract

Machine learning continues to make strident advances in the prediction of desired properties concerning drug development. Problematically, the efficacy of machine learning in these arenas is reliant upon highly accurate and abundant data. These two limitations, high accuracy and abundance, are often taken together; however, insight into the dataset accuracy limitation of contemporary machine learning algorithms may yield insight into whether non-bench experimental sources of data may be used to generate useful machine learning models where there is a paucity of experimental data. We took highly accurate data across six kinase types, one GPCR, one polymerase, a human protease, and HIV protease, and intentionally introduced error at varying population proportions in the datasets for each target. With the generated error in the data, we explored how the retrospective accuracy of a Naïve Bayes Network, a Random Forest Model, and a Probabilistic Neural Network model decayed as a function of error. Additionally, we explored the ability of a training dataset with an error profile resembling that produced by the Free Energy Perturbation method (FEP+) to generate machine learning models with useful retrospective capabilities. The categorical error tolerance was quite high for a Naïve Bayes Network algorithm averaging 39% error in the training set required to lose predictivity on the test set. Additionally, a Random Forest tolerated a significant degree of categorical error introduced into the training set with an average error of 29% required to lose predictivity. However, we found the Probabilistic Neural Network algorithm did not tolerate as much categorical error requiring an average of 20% error to lose predictivity. Finally, we found that a Naïve Bayes Network and a Random Forest could both use datasets with an error profile resembling that of FEP+. This work demonstrates that computational methods of known error distribution like FEP+ may be useful in generating machine learning models not based on extensive and expensive in vitro-generated datasets.

## 1. Introduction

Pharmaceutical development demands new approaches capable of confronting the ever-increasing cost of drug discovery and drug development. With the rise of PubChem, ChEMBL, and additional sources of information, pharmaceutical research has moved into the realm of big data analytical techniques [1,2]. Several machine learning methods have emerged as robust platforms for big data and cheminformatics like the Support Vector Machine (SVM), Naïve Bayes Network (NBN), Random Forest (RF), neural net, and deep learning methods. Early work addressing the task of increasing efficiency in drug development through the use of machine learning and big data techniques is encouraging. Guangli et al. reported a foundational study exploring the utility of SVM techniques in predicting Caco-2 properties, and the team achieved modest success in predicting this key pharmacological parameter for drug development [3]. Additionally, Kortagere and coworkers explored the utility of SVM algorithms trained on molecular descriptors from *Shape Signatures* and the Molecular Operating Environment (MOE) to predict pregnane X receptor activation and found an accuracy of 72–81% could be achieved [4]. With regard to potency on a desired biological target, we reported preliminary success in using NBNs prospectively against a desired target [5]. Our work is part of a significant body of work emerging which shows that machine learning has a high degree of prospective predictive utility in the drug development process when optimizing for potency against a desired target or off target [6,7,8]. Finally, work has emerged which uses metadata constructed on selectivity indices for enzyme isoforms or viral mutants, and techniques are being developed which allow for the prediction of a biological target, given some query small molecule structure [9,10].

However, the success of machine learning in these drug development applications is reliant on preexisting experimental information in a research group or on large databases of experimental data. The fundamental limitation of machine learning has been the necessity of biological activity data generated from benchtop experiments. Technological advances in computing power and improvements to techniques like the Free Energy Perturbation method (FEP/FEP+) are poised to alleviate this need [11,12,13,14]. FEP and other techniques are an appealing format for generating virtual biological data on which to train machine learning algorithms as these techniques can explore 100’s to 1000’s of candidate molecules and they possess a high degree of accuracy (on the order of <1 kcal/mol) [11]. The potential opportunity for machine learning is to use techniques like FEP+ to create virtual data sets of 100’s of compounds in a much shorter timeframe than wet lab experimental work and then use the significantly quicker machine learning techniques trained on those 100’s of compounds to explore 10’s of millions of possible synthetic targets. The reason for such a hybrid approach is that it is not currently feasible to explore the millions of synthetic candidates for a given scaffold using FEP alone due to computational cost [15,16]. Additionally, the success of FEP may only be limited to the target on which the FEP calculations were conducted. The set of compounds explored by FEP may have other hurdles in the development process that were not ascertainable at the time of FEP calculation. However, we envision the data produced from FEP being used to construct machine learning algorithms which can explore the 10’s to 100’s of millions of synthetically accessible and drug-like compounds in the chemical space of interest. These millions of compounds can then be optimized for on target potency, off target potency, resistance susceptibility for infection or cancer, and many other properties now being predicted with machine learning. However, the initial hurdle to addressing this research direction was to determine the amount of error contemporary machine learning algorithms could accommodate. We therefore set out to discover the error profiles of a Naïve Bayes Network, a Random Forest, and a Probabilistic Neural Network trained across ten contemporary biological targets.

## 2. Results and Discussion

### 2.1. Selection of Targets and Machine Learning Methods

**Figure 1:**
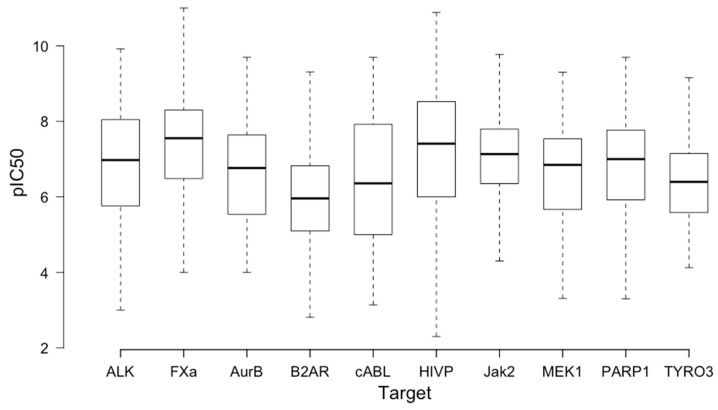
Summary of the activity distribution (pIC_50_) for the ten targets investigated. (B2AR: β-2 adrenergic receptor and HIVP: HIV protease).

We identified a series of contemporary biological targets that were either known to have produced a drug or are currently being explored in drug discovery with well-distributed activity data, and N is equal to the number of molecules used for each target (Table 1 and Figure 1, data cleaning details below). Our selection parameters were that the target must have data in the ChEMBL database and must have a single pocket of drug–protein interaction (details for molecular biology on each target in the Supporting Information). The ChEMBL database was selected as our data source due to the rigorous curation process activity data undergo before being incorporated [17].

**Figure 2:**
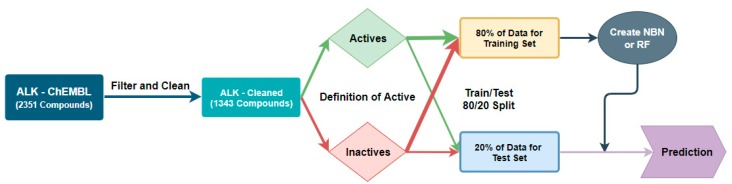
Workflow for classification threshold evaluation in the Naïve Bayes Network (NBN) and Random Forest (RF) algorithms.

**Figure 3:**
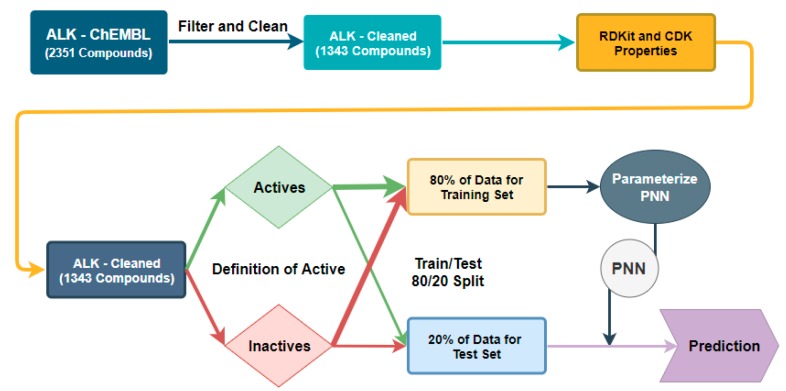
Workflow for classification threshold evaluation in the Probabilistic Neural Network (PNN) algorithm.

Our design principles for our machine learning workflow revolved around creating a workflow that would have the best accuracy characteristics at a given threshold of potency (IC_50_ value), and this method is represented graphically in Figure 2; Figure 3 with anaplastic lymphoma kinase (ALK) is an example case. We used the KNIME Analytics Platform as our means of manipulating our data and generating our machine learning algorithms [18]. The data were cleaned for missing IC_50_ values, duplicate entries, and entries that did not have units in nM. Mutant enzyme data were removed in the case of JAK2 (V617F) and HIV protease (strain V18, strain NL4-3 or any mutant). We used classification algorithms for the NBN, RF, and Probabilistic Neural Network (PNN) algorithms in KNIME due to the decreased computational cost associated with classification [19]. Additionally, we explored the Support Vector Machine method initially as well. However, we were unsuccessful in building a predictive algorithm with either a polynomial kernel, hypertangent kernel, or a radial basis function kernel using similar techniques to the PNN method discussed below (Supporting Information). The classifier method only gives a score which corresponds to the likelihood of whether the compound in question is good (an IC_50_ < a predefined threshold) or bad (an IC_50_ > a predefined threshold) [20,21]. The training set for each algorithm consisted of 80% of the data defined as active for a given threshold (e.g., IC_50_ < 20 nM as active) and 80% if the data defined as inactive for the same threshold [19]. The test set was the remaining 20% of each category. Our inputs for training the NBN and RF were extended connectivity fingerprints (ECFP-4) as the independent variable and active/inactive as the dependent variable due to the rapid computation associated with ECFP [5,9,22]. We initially only investigated ECFP-4 as the independent variable, and, if inadequate predictive capability was encountered, we would use additional calculated properties from the molecular structure as independent variables on which machine learning could take place.

For the PNN, we were unable to build an algorithm that had any predictive capacity using ECFP-4 as the algorithm requires variable inputs to be in numerical format and ECFP-4 is a bit array. Therefore, we used 46 calculable properties as our independent variables, which were calculated using the RDKit and CDK toolkit modules in KNIME, and the same active/inactive classification was our dependent variable [23,24].

The performance of each model was evaluated in training using a leave-one-out cross validation method for the NBN, while we used the standard algorithm for RF in KNIME and we used a 5-fold cross validation for parameterization of the PNN [19]. We explored the active/inactive threshold by stepwise increasing the IC_50_ value from 2.5 to 50 nM. The IC_50_ value that was capable of giving the best ROC score, mean top 10% IC_50_, enrichment characteristics, sensitivity, and precision overall was used as the definition of active/inactive for the error analysis.

### 2.2. Evaluation of Error Tolerance in ALK

**Figure 4:**
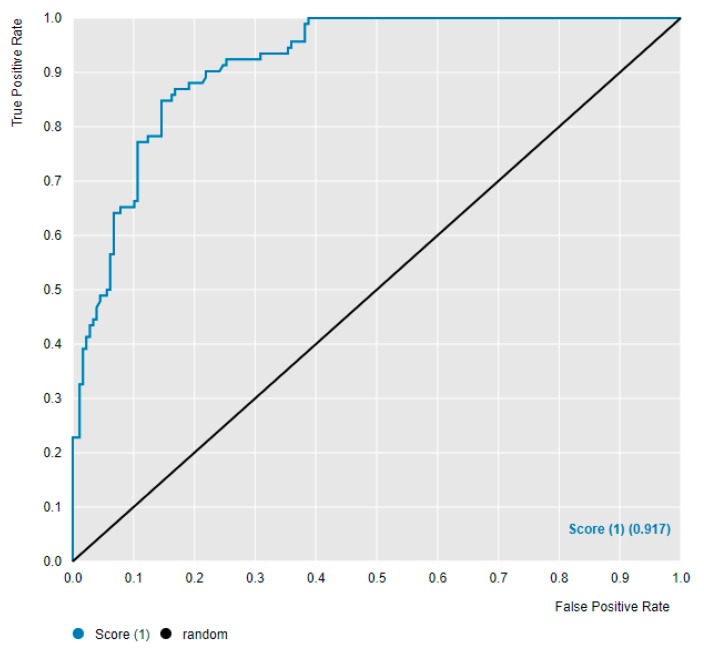
ROC for the NBN trained on the ALK dataset with a <20 nM classification.

**Figure 5:**
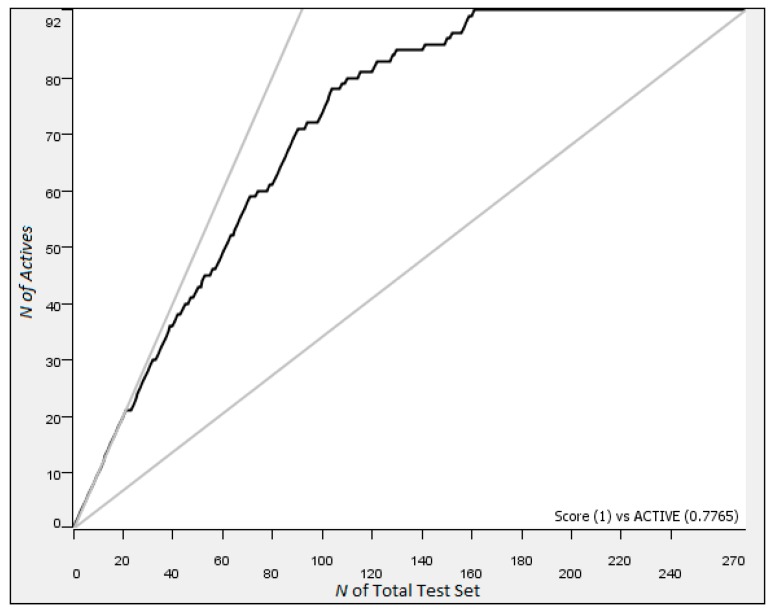
Enrichment plot for the NBN Trained on the ALK dataset with a <20 nM classification.

We began our work with the anaplastic lymphoma kinase (ALK) dataset from ChEMBL and sought to create control algorithms against which we could compare error introduced into the dataset. We scanned the classification threshold from 5 to 25 nM, and we found that using a definition of good as <20 nM gave us an NBN which, when tested on the 270 compounds in the 20% test set, had a ROC AUC of 0.917, a mean top 10% IC_50_ of 6.7 nM, and good enrichment characteristics (Figure 4 and Figure 5; additional data in ALK Supporting Information). The other cutoffs explored gave less satisfactory performances when NBNs trained at that threshold made predictions on the test set (Supporting Information). Another contender for the best algorithm was the <5 nM classification threshold; however, this algorithm only gave a ROC AUC of 0.874 and moderate enrichment despite a mean top 10% IC_50_ of 5.3 nM. We therefore selected the classification threshold (the definition of good) as <20 nM for the NBN in ALK. 

We repeated this analysis of the ALK dataset using a RF algorithm and found that the <20 nM decision value gave the best top 10% IC_50_ at 3.3 nM, gave a ROC AUC of 0.913 and had excellent enrichment characteristics (ALK Supporting Information). Again, we repeated this analysis for the ALK dataset using the workflow in Figure 3 for a PNN algorithm and again found that the <20 nM decision value gave the best performance. The ROC AUC was 0.782 for a PNN trained with a <20 nM decision value, and the model had a top 10% IC_50_ mean of 34.7 nM and modest enrichment characteristics (ALK Supporting Information). These data are summarized in Table 2. As both definitions of good in the RF model and the PNN model mirrored the NBN definition of good and the NBN experiment to find the classification value was much faster than the RF or PNN experiments, we devised a system wherein we would find the classification value in an NBN and confirm that this value performed well in a RF or PNN. In subsequent cases where the classification value from the NBN performed well with a RF or PNN, we would not explore all possible classification thresholds for an RF or PNN thus enabling us to compare error tolerance on the same dataset across the three algorithm types.

**Figure 6:**
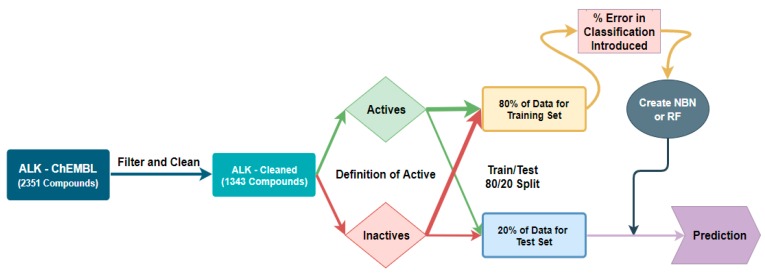
Workflow for error tolerance evaluation in the NBN and RF algorithms.

After identifying the classification definition of good for ALK to be <20 nM, we turned our attention to the task of designing a workflow capable of introducing error at specified populations into our training data. Our experimental setup would be to create three random splits, one of which would be the same as the control, and evaluate how introducing error into the three training sets degraded retrospective predictivity on the unaltered test sets. The initial type of error chosen was a general classification error where an active would be switched inappropriately and intentionally to an inactive and/or vice versa. We would scan the percent error introduced from 5% error in the training set increasing by 5% error in the training set up to the point of failure which was defined as either the ROC AUC dropping below 0.7 or the top 10% mean IC_50_ exceeding 750 nM. With those clear objectives defined, we envisioned the workflow as follows in Figure 6.

**Figure 7:**
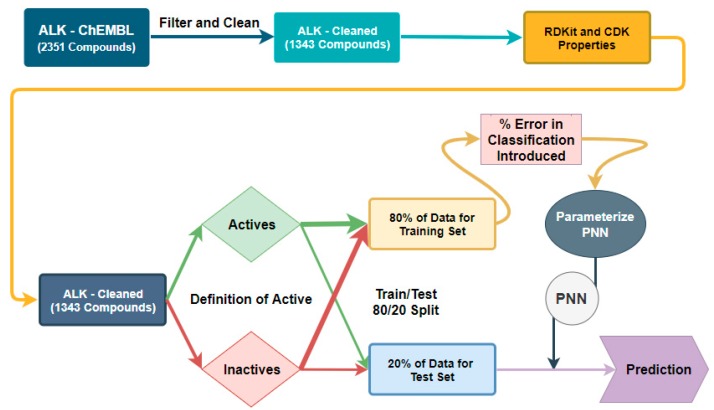
Workflow for error tolerance evaluation in the PNN algorithm.

A similar process would be used for the exploration of classification error in a PNN, and the following workflow would be used (Figure 7). The compounds would be cleaned, and molecular properties would be generated as in the control case for PNN generation. However, percent error would be introduced in the training set as a classification error before the parameterization step. This training set containing error would also be used to generate the PNN and the algorithm would be used to make predictions about the unadulterated, error-free test data.

Using the workflows outlined in Figure 6 and Figure 7, we explored the error tolerance of the NBN on the ALK dataset. In three different random splits, we found that the step-wise increase in error from 5–50% percent classification error in the training set led to a failure of predictivity at 45%, 45% and 50% error (Table 3 and ALK Supporting Information). As compared to the control, each split with the indicated level of error had a significant decline in ROC AUC as well as a severe loss of predictivity in the top 10%. The mean IC_50_ in the top 10% increased from 6.7 nM to 1400 nM in split 1 as the error reached 45% in the training set. Additionally, the fold difference between the bottom 10% mean IC_50_ and the top 10% IC_50_ decreased from 2500 to 3.5. This represents a significant loss of predictive power and increasing the error to 45% of the training set in the first random split resulted in an algorithm no longer useful for prioritizing candidates for synthesis. Similar results were seen in the second and third random splits with the third random split at 50% error suffering a reversal in potency as the top 10% of compounds were less potent on average than the bottom 10%.

It can be seen that these levels of error reported in Table 3 are the points of failure in each random split as we have included the performance statistics of the three splits at the error threshold just before failure in Table 4 (i.e., 40%, 40%, and 45%; the representative set of statistics for each error percentage can be found in the Supporting Information). While Table 4 represents a series of NBNs with diminished predictive power as compared to the control, the ROC AUC is still acceptable for each. Additionally, the enrichment capacity for actives at the top of the list is still largely preserved in each random split with the indicated error. It is important to note that even at error percentages of 40%, 40% and 45%, useful NBNs can be generated which retain predictive power against a test set.

Encouraged by the high tolerance for error seen in an NBN trained for ALK potency, we used a similar approach to evaluate the performance of an RF as error was introduced to the dataset. In three different random splits used to train an RF algorithm, we found that the step-wise increase in error from 5–50% percent classification error in the training set led to a failure of predictivity at 30%, 40%, and 40% error (Table 5 and ALK Supporting Information).

Similar to the NBN results, each percent error failure threshold was said to be reached when there was a significant decline in ROC AUC as well as a severe loss of predictivity in the top 10% as compared to the control performance (failure to tolerate error was defined as a ROC AUC < 0.7 or a mean top 10% IC_50_ > 750 nM). The mean IC_50_ in the top 10% increased from 3.33 nM to 1800 nM in split 1 as the error reached 30% classification error in the training set. Additionally, the fold difference between the bottom 10% mean IC_50_ and the top 10% IC_50_ decreased from 4800 to 4.2. This represents a significant loss of predictive power and increasing the error to 30% of the training set in the first random split resulted in an algorithm no longer useful for prioritizing candidates for synthesis. Similar results were seen in the second and third random splits; however, the enrichment in split 3 was still moderately useful, even in the face of a loss of general accuracy, as the fold difference between top 10% and bottom 10% was 45-fold. The overall failure of the algorithm in split 3 is due to a significant contamination of the top compounds with inactive compounds of >1000 nM IC_50_. Once again, we investigated the performance statistics of the three splits at the error threshold just before failure, and these statistics are reported in Table 6 (i.e., 25%, 35%, and 35%; the representative set of statistics for each error percentage can be found in the Supporting Information). We found that the random forests generated on the three splits with 25%, 35%, and 35% classification error had diminished predicative power as compared to the control; nevertheless, the ROC AUC is still acceptable for each and the enrichment capacity for actives at the top of the list was still largely preserved as in the NBN experiment. 

Finally, we evaluated the tolerance for error seen in a PNN trained for ALK potency using the workflow outlined in Figure 7. In three different random splits used to train a PNN algorithm, we found that the step-wise increase in error from 5–50% percent classification error in the training set led to a failure of predictivity at 20%, 30%, and 15% error (Table 7 and Table 8; ALK Supporting Information). We initially used the parameters identified on the unaltered training set as the parameters used in all PNNs created with increasing error in each split. Once a threshold was identified, the model was reparametrized on the training data with the introduced error, and the performance was reevaluated to confirm a lack of rescue through reparameterization. Reparameterization did not change the percent error tolerated in the training set (Supporting Information). Of note is the significantly reduced error tolerance that a PNN has for its training set as compared to an NBN and a RF trained on the same data. The average point of failure of error introduced to a training set for a Probabilistic Neural Network was 22% error as compared to an average of 37% for a Random Forest and an average of 47% error for a Naïve Bayes Network. We turned our attention to exploring this initial result across the remaining nine biological targets.

### 2.3. Evaluation of Error Tolerance in Ten Biological Targets

We applied the above methods developed on ALK to the remaining nine targets. The percentage of classification error that lead to a failure of the predictive algorithm on an unaltered test set (a ROC AUC < 0.7 or a mean top 10% IC_50_ > 750 nM) is reported for each algorithm on three different random splits of the data for each biological target (Table 9). The specific details for each algorithm generated can be found in the Supporting Information for each biological target. Several surprising findings emerged from this exploration of error. The first was that it was not always possible to parameterize a PNN to where the algorithm could make predictions in the control test. This was the case when we used the data from Aurora B kinase, JAK2 kinase, and TYRO3 kinase. We cannot currently comment as to the reason for the failure of a PNN for these three targets except that it is likely not due to enzyme type (the three are from separate biological families) nor is it due to data size as these datasets were either the smallest dataset (TYRO3), largest dataset (JAK2), or intermediate in size (Aurora B) as can be seen in Table 1. The second finding was that reparameterization did alter the percent error that led failure in three cases. This is in contrast to our initial work in ALK where parameterization on the original dataset was all that was needed to find the point of error for the error-containing data. Once that error threshold was found in ALK, reparameterization did not rescue the predictivity of the models. However, the point of error shifted dramatically in random split 1 in PARP1. In this split, reparameterization rescued predictivity at 5% error and moved the point of failure to 25% error. Split 3 had a modest change in percent error that led to failure as did split 3 in MEK1 (Supporting Information). Finally, there were two targets which had a random split fail in the control stage: the NBN on split 3 for JAK2 and the RF on split 3 for TYRO3. In both cases, a new random seed number was used, and that split was employed in the error tolerance analysis (Supporting Information).

After this analysis, we were curious as to the average percent classification error that would lead to predictive failure in each algorithm category (Table 10). The NBN had an average threshold resulting in failure of 39% classification error introduced to the training set, while an RF had a 29% error threshold and the PNN had a 20% error threshold. These results suggest that the NBN is the most tolerant of error of the three algorithms explored.

### 2.4. Use of Error Profile in FEP+ on the ALK Dataset with an NBN and RF

Encouraged by the high degree of error tolerance by an NBN and a RF, we were curious to apply the specific error profile observed in FEP+ to the ALK dataset in an effort explore preliminarily the utility of FEP+ in generating datasets. FEP+ and other relative binding free energy (RBFE) calculations allow for the computation of ΔΔG_A,B_, or the relative binding energy difference between a known inhibitor, A, and candidate molecule of unknown potency, B [14,15]. The accuracy of these methods has been well explored, and Abel et al. have shown that 73% of data have a calculated relative energy of binding < 1 kcal/mol away from the experimentally observed value [14]. However, medicinal chemists frequently use IC_50_ rather than energy of binding as a decision parameter because it is often an easier datapoint to attain, requiring roughly only 20% of the datapoints needed to acquire a *K_i_* value [25]. This is in contrast to the inhibition constant, *K_i_*, which requires time-intensive kinetic experiments or the enthalpy of binding which requires a surface plasmon resonance approach or an isothermal titration calorimetry approach [15]. However, there is a relationship between *K_i_* and IC_50_: the Cheng–Prusoff equation Equation (1). We have only included the relationship between IC_50_ and *K_i_* for competitive inhibitors below as we restricted our analysis to ALK, an enzyme for which most inhibitors compete with ATP for the ATP-binding pocket of ALK [26].

(1) $${\mathrm{IC}}_{50}=K_{i}\left(1+\frac{\left[S\right]}{K_{m}}\right)$$

Equation (1): The Cheng–Prusoff Equation relating *K_i_* and IC_50_ for competitive inhibitors.

As we stated above, RBFE calculations give the ΔΔG_A,B_ value between two compounds, one of which is known to inhibit the desired target. We can use the molecule, A, with a known *K_i_* as a reference point to calculate the computationally determined ΔG_A_, given we know *K_i_* for A and, thus, the Gibbs free energy of A from Equation (2) [15].

(2) $${\Delta G}_{A}=RT\ln K_{i}$$

Equation (2): Relationship between Gibbs free energy of binding and *K_i_.*

Therefore the relationship between the calculated property in RBFE calculations, ΔΔG_A,B_, the experimentally determined *K_i_* of A and the theoretical *K_i,theor_* of molecule B is as follows Equation (3). This is because ΔG_B_ is equal to the difference of ΔG_A_ and ΔΔG_A,B_.

(3) $${\Delta G}_{B}={\Delta G}_{A}-{\Delta \Delta G}_{A,B}\;{\Delta G}_{B}=RT\ln K_{i,theor}\;K_{i,theor}=e^{\left(\frac{{\Delta G}_{B}}{RT}\right)}\;؞\;K_{i,theor}=e^{\left(\frac{{\Delta G}_{A}-{\Delta \Delta G}_{A,B}}{RT}\right)}$$

Equation (3): The relationship between the relative binding free energy (RBFE)-derived ΔΔG_A,B_ and the theoretical *K_i,theor_* for B.

Now that we had a way to relate the computationally derived ΔΔG_A,B_ to a theoretical IC_50_ with Equations (1) and (3), we could turn our attention to designing an initial retrospective experiment to introduce error into the ALK dataset resembling the error found in FEP+. We needed to make two assumptions in order to construct our analysis. The first assumption was that the error introduced by our workflow would meaningfully reflect the real error generated by FEP+ or another RBFE in such an analysis. In this situation, the error profile reported for FEP+ (27% with an energy of binding > 1 kcal/mol from the experimentally observed value) would be maximally damaging if it were introduced as categorical error around the definition of good used in the NBN or RF. Therefore, we desired to introduce 27% error to molecules that fell within an IC_50_ range that was ±1 kcal/mol of the energy of binding associated with that IC_50_ value used as the definition of good (in the case of ALK, 20 nM). This would effectively shift these compounds into the >1 kcal/mol error category. The logic of this can be seen in that if a molecule was found experimentally to have an IC_50_ of 300 nM when FEP+ predicted a *K_i,theor_* that gave a theoretical IC_50_ of 10,000 nM, the overall result of this error is not relevant to the classification algorithm. This is because both a 300 nM and a 10,000 nM compound would be classified as inactive for a definition of good of <20 nM. However, if a molecule with an experimental value of 100 nM were incorrectly predicted to have an IC_50_ of 2 nM, this mistake would disrupt the predictivity of the machine learning algorithm. Thus, the most damaging error would be error that led to a blurring of <20 nM and >20 nM when learning took place on the training data.

The second assumption was the nature of the relationship between the IC_50_’s in our dataset and their *K_i_*’s. We assumed relative uniformity in the concentration of ATP, [*S*], used in the ChEMBL dataset as there are frequently used standard assay conditions in the determination of IC_50_’s for a given target [27]. We used an ATP concentration of 300 µM as [*S*] given the work of Gunby and coworkers in establishing a protocol for the ELISA-ALK assay often employed in the literature [28]. Finally, the ALK *K*_m_ for ATP was found to be 134 µM according to the work of Bresler et al [29]. With these assumptions, we could find the window of IC_50_’s in which we would need to introduce 27% categorical error. The first step in this process was to relate the 20 nM definition of good to an energy of binding and then back calculate the IC_50_ values that were ±1 kcal/mol from that calculated energy of binding (Supporting Information). This gave us an energy of binding of −11.19 kcal/mol for a 20 nM IC_50_ and an energy range of −10.19 kcal/mol to −12.19 kcal/mol corresponding to an IC_50_ range of 109 nM to 3.7 nM. It was into this IC_50_ range that we could introduce 27% classification error in the training sets for NBN and RF algorithms.

The results of this error exploration are reported in Table 11 and show that the classification error of 27% introduced into the training set compounds with IC_50_’s between 109 and 3.7 nM did degrade the predictivity of the NBN, but not to a point where the algorithm lost utility. The worst performance was when split 3 was evaluated, and the mean top 10% IC_50_ was found to have a significantly reduced potency. However, this was due to the second-to-last molecule ranked in the top 10% which had an IC_50_ of 2400 nM. These results suggest that a useful NBN can be generated for a dataset generated by FEP+.

We reproduced the above experiment but with the RF algorithm (Table 12). The classification error of 27% introduced into the compounds with IC_50_’s between 109 and 3.7 nM did not significantly degrade the predictivity of the RFs trained on the dataset. In all three cases, useful algorithms were generated that possessed a high degree of decision-making concerning active and inactive compounds. These results suggest that a useful RF can be generated for a dataset generated by FEP+. Additionally, the RF outperformed the NBN in this task with the RF averaging a ROC AUC of 0.92, a top 10% mean IC_50_ of 10.1 nM and a fold difference between mean top 10% IC_50_ and mean bottom 10% IC_50_ of 1800-fold. The NBN averaged a ROC AUC of 0.90, a top 10% mean IC_50_ of 46.8 nM and a fold difference between mean top 10% IC_50_ and mean bottom 10% IC_50_ of 460-fold.

## 3. Materials and Methods 

The literature data were acquired from the ChEMBL database by performing a target search for each of the ten targets. These targets were evaluated for a single pocket of inhibition by performing a literature review and a summary for each target is reported in the Supporting Information [30,31,32,33,34,35,36,37,38,39,40,41,42,43,44,45,46,47,48,49,50,51,52,53]. All cheminformatics processing and analysis, as well as machine learning, was performed in KNIME 3.7.0 and KNIME 3.7.1. The software was run on a Razer Blade 15 with an 8th Gen Intel core i7-8750H 6 core and 16 Gb of DDR4 system RAM. The first step involved filtering the data to ensure all compounds had explicitly defined activities that were reported as nM. Next, molecules that had an exact value for the IC_50_, a value reported as <10 nM or a value >999 nM were retained as these molecules were either exactly known for IC_50_ or were considered potent (<10 nM) or lacking potency (>999 nM). All molecules were sorted in order of increasing IC_50_ and duplicate entries were removed. The molecular structure was generated using the CDK community expansion for KNIME and the ECFP-4 fingerprints were calculated from the CDK structure. The molecules were then split according to whether they were <X where X is an IC_50_ value that defines active and inactive categorically (5, 10, 15, 20, 25, etc. nM). The actives and inactives were each split into an 80% training set and a 20% test set. An NBN or RF was generated using the independent variable as the ECFP-4 and the category active/inactive as the dependent variable. The algorithm was then fed into the predictor module for the corresponding algorithm, and the performance of the NBN or RF was evaluated on the test set using the ROC Curve (Java Script) and Enrichment Plotter modules. Rule based modules were used to sort out the true positive/true negative/false positive/false negative statistics. The definition of good was selected in accordance with which definition of good performed best in the ROC AUC, sensitivity, specificity, top 10% mean IC_50_ and enrichment characteristics.

For the PNN control, the training data generated as above were fed into a 5-fold cross validation where Theta minus and Theta plus were parametrized using accuracy as the scoring metric. These parameters were used in the PNN algorithm tested on the reserved 20% test data. The values for learning were calculated from the CDK molecular structure through the use of the RDKit Descriptor Calculation and the CDK Molecular Properties modules. All IC_50_ values, publication data, and other non-molecular data were removed from the training set after the active/inactive split so as to remove confounding variables. A key feature for the control experiments was that once the definition of good for an NBN was found, this value was used for the RF and PNN algorithms.

For the error tolerance experiments, the definition of good was used to split the data into active and inactive sets using the previously identified definition of good, and each set was then split into an 80% training set and a 20% test set as above. With the 80% training data, a variable percentage was removed, shuffled, and split into actives/inactives where the class was inverted (e.g., a compound defined as active had this definition inverted to inactive). These errors were then reintroduced to the training set, and an NBN or RF was generated. The percent error was increased until the machine learning algorithm had a ROC AUC < 0.7 or a mean top 10% IC_50_ > 750 nM. This was done in triplicate with the actives partitioning, inactives partitioning and test error partitioning modules each using a random seed of 1515533876005, 429, or 121783. In the cases where 121783 failed, 12178 was used instead.

For the PNN error tolerance experiments, the introduction of error was performed as was done for the RF and NBN while using the parameters from the PNN control step. Once the error threshold was identified, the model was reparametrized on the training set containing the percent error and the model with the corrected parameters was evaluated for a ROC AUC < 0.7 or a mean top 10% IC_50_ > 750 nM. This was done in triplicate with the actives partitioning, inactives partitioning, and test error partitioning modules each using a random seed of 1515533876005, 429, or 121783. In the cases where 121783 failed, 12178 was used instead.

For the error tolerance experiments using an error profile resembling FEP+, the definition of good was used to split the data into active and inactive sets using the previously identified definition of good, and each set was then split into an 80% training set and a 20% test set as above. With the 80% training data, those compounds that had an IC_50_ between 109 nM and 3.7 nM were removed, and 27% of those compounds were shuffled, and split into actives/inactives where the class was inverted (e.g., a compound defined as active had this definition inverted to inactive). These errors were then reintroduced to the training set, and an NBN or RF was generated. The performance of the NBN or RF was evaluated using the same techniques in the RF or NBN control experiments.

## 4. Conclusions

We explored the behavior of nearly 600 machine learning algorithms generated with various types of error on ten contemporary biological targets representing common target types pursued in drug discovery and drug development. The categorical error tolerance was quite high for a Naïve Bayes Network algorithm averaging 39% error in the training set required to lose of predictivity on the test set (defined as a ROC AUC < 0.7 or a mean top 10% IC_50_ > 750 nM). This average was the result of three random splits applied to the biological data for ten targets downloaded from the ChEMBL database. Additionally, a Random Forest tolerated a significant degree of categorical error introduced into the training set with an average error of 29% required to lose predictivity. However, we found the Probabilistic Neural Network algorithm to be difficult to work with, and it did not tolerate as much categorical error requiring an average of 20% error to lose predictivity. Additionally, the PNN required a computationally expensive 5-fold cross validation parameterization step for each algorithm generated. Finally, we explored the possibility of using FEP+ as a means to rapidly generate a dataset for machine learning thereby increasing the number of molecules explorable from 100’s to 10’s of millions. We trialed a 27% classification error within the 1 kcal/mol range of our IC_50_ category decision value (20 nM) and found that both the NBN and RF retained a high degree of retrospective predictivity in the face of this error. We found that the Random Forest, while less tolerant of general error in the ten targets we explored, had a superior performance to the Naïve Bayes Network when exposed to the 27% error in the 1 kcal/mol window. Together, these error tolerance results suggest that contemporary methods for calculating relative binding energies may be a method by which initial data may be rapidly generated for 100’s of compounds for the purpose of using machine learning to quickly explore 10’s of millions of possible synthetic candidates for a given target. Additionally, FEP+ may be used as a means to increase data size by 100’s of compounds for isoforms of a given enzyme family. This potentially could increase the likelihood of success for machine learning to optimize a given scaffold for desired isoform selectivity.

## Figures and Tables

**Table 1:** Summary of the ten targets investigated.

Target	Protein Family	Species	*N*	Drug Stage
Anaplastic lymphoma kinase (ALK)	Receptor Tyr Kinase	*H. sapiens*	1343	Phase IV
Aurora B	Ser/Thr Kinase	*H. sapiens*	1481	Phase III
β-2 Adrenergic Receptor	GPCR	*H. sapiens*	641	Phase IV
c-Abl	Tyr Kinase	*H. sapiens*	1439	Phase IV
Factor Xa	Protease	*H. sapiens*	1657	Phase IV
HIV Protease	Protease	HIV	2544	Phase IV
JAK2	Non-receptor Tyr Kinase	*H. sapiens*	3624	Phase IV
MEK1	MAP Kinase Kinase	*H. sapiens*	823	Phase IV
PARP1	Polymerase	*H. sapiens*	1933	Phase IV
TYRO3	Receptor Tyr Kinase	*H. sapiens*	277	none

**Table 2:** Summary of performance for the NBN, RF, and PNN on the ALK dataset.

Model	ROC AUC	Top 10% Mean IC_50_ (nM)	Sensitivity	Precision	Enrichment Factor at 10%
ALK < 20 nM NBN	0.917	6.70	0.891	0.678	2.717
ALK < 20 nM RF	0.913	3.30	0.739	0.739	2.935
ALK < 20 nM PNN	0.782	34.7	0.533	0.645	1.789

**Table 3:** Performance statistics for failed NBNs generated with the specified error in each split.

Model	%Error Failure	ROC AUC	Top 10% Mean IC_50_ (nM)	Mean IC_50_ (nM)	Bottom 10% Mean IC_50_ (nM)	Fold Difference in Mean Top 10% IC_50_
ALK < 20 nM NBN	Control	0.917	6.70	4200	17,000	2500
Split 1 Error	45	0.674	1400	4200	4900	3.5
Split 2 Error	45	0.632	900	3300	8100	9.0
Split 3 Error	50	0.539	2400	4100	1100	0.46

**Table 4:** Performance statistics for NBNs with retained predictivity generated with the specified error in each split.

Model	%Error Before Failure	ROC AUC	Top 10% Mean IC_50_ (nM)	Mean IC_50_ (nM)	Bottom 10% Mean IC_50_ (nM)	Fold Difference in Mean Top 10% IC_50_
ALK < 20 nM NBN	Control	0.917	6.70	4200	17,000	2500
Split 1 Pre-failure	40	0.742	53.5	4200	3100	58
Split 2 Pre-failure	40	0.747	275	3300	9200	33
Split 3 Pre-failure	45	0.703	130	4100	4500	35

**Table 5:** Performance statistics for failed RFs generated with the specified error in each split.

Model	%Error Failure	ROC AUC	Top 10% Mean IC_50_ (nM)	Mean IC_50_ (nM)	Bottom 10% Mean IC_50_ (nM)	Fold Difference in Mean Top 10% IC_50_
ALK < 20 nM RF	Control	0.913	3.33	4200	16,000	4800
Split 1 Error	30	0.762	1800	4200	7500	4.2
Split 2 Error	40	0.677	383	3300	8500	22
Split 3 Error	40	0.691	113	4100	5100	45

**Table 6:** Performance statistics for RFs with retained predictivity generated with the specified error in each split.

Model	%Error Before Failure	ROC AUC	Top 10% Mean IC_50_ (nM)	Mean IC_50_ (nM)	Bottom 10% Mean IC_50_ (nM)	Fold Difference in Mean Top 10% IC_50_
ALK < 20 nM RF	Control	0.913	3.33	4200	16,000	4800
Split 1 Pre-failure	25	0.828	362	4200	6400	18
Split 2 Pre-failure	35	0.739	111	3300	5600	50
Split 3 Pre-failure	35	0.746	19.5	4100	7600	390

**Table 7:** Performance statistics for failed PNNs generated with the specified error in each split.

Model	%Error Failure	ROC AUC	Top 10% Mean IC_50_ (nM)	Mean IC_50_ (nM)	Bottom 10% Mean IC_50_ (nM)	Fold Difference in Mean Top 10% IC_50_
ALK < 20 nM PNN	Control	0.782	34.7	4200	19,000	550
Split 1 Error	20	0.654	726	4200	12,000	17
Split 2 Error	30	0.615	287	3300	7600	26
Split 3 Error	15	0.635	1200	4100	3300	2.8

**Table 8:** Performance statistics for PNNs with retained predictivity generated with the specified error in each split.

Model	%Error Before Failure	ROC AUC	Top 10% Mean IC_50_ (nM)	Mean IC_50_ (nM)	Bottom 10% Mean IC_50_ (nM)	Fold Difference in Mean Top 10% IC_50_
ALK < 20 nM PNN	Control	0.782	34.7	4200	19,000	550
Split 1 Pre-failure	15	0.701	14.0	4200	2700	190
Split 2 Pre-failure	25	0.706	637	3300	5500	8.6
Split 3 Pre-failure	10	0.736	375	4100	7400	20

**Table 9:** Summary of points of failure for each algorithm, random split and target.

Target	Algorithm	Split 1 Percent Error of Failure	Split 2 Percent Error of Failure	Split 3 Percent Error of Failure	Mean Percent Error of Failure
ALK	NBN	45	45	50	47
	RF	30	40	40	37
	PNN	20	30	50	33
Aurora B	NBN	30	30	35	32
	RF	20	20	25	22
	PNN	-	-	-	-
β-2	NBN	50	50	50	50
	RF	35	25	45	35
	PNN	10	20	5	12
c-Abl	NBN	45	50	45	47
	RF	35	25	35	32
	PNN	30	25	15	23
Factor Xa	NBN	35	45	45	42
	RF	30	30	30	30
	PNN	20	10	30	20
HIV Protease	NBN	50	45	50	48
	RF	35	20	15	23
	PNN	25	15	25	22
JAK2	NBN	40	30	40 *	35
	RF	40	30	35	35
	PNN	-	-	-	-
MEK1	NBN	40	30	40	37
	RF	30	35	25	30
	PNN	5	5	25 **	12
PARP1	NBN	5	45	50	33
	RF	40	25	40	35
	PNN	25 **	5	25 **	18
TYRO3	NBN	30	10	5	15
	RF	20	5	5 *	10
	PNN	-	-	-	-

* failed in the control step, therefore another random split was used. ** reparameterization shifted the point of failure.

**Table 10:** Average percent classification error that leads to failure.

Model	Average %Error Threshold
NBN	39
RF	29
PNN	20

**Table 11:** Performance statistics for NBNs with retained predictivity generated with 27% classification error in molecules between 109 nM and 3.7 nM.

Model	ROC AUC	Top 10% Mean IC_50_ (nM)	Mean IC_50_ (nM)	Bottom 10% Mean IC_50_ (nM)	Fold Difference in Mean Top 10% IC_50_
ALK < 20 nM NBN	0.917	6.70	4200	17,000	2500
Split 1 Error	0.916	18.0	4200	17,000	940
Split 2 Error	0.897	22.5	3300	7000	310
Split 3 Error	0.887	99.8	4100	14,000	140

**Table 12:** Performance statistics for RFs with retained predictivity generated with 27% classification error in molecules between 109 nM and 3.7 nM.

Model	ROC AUC	Top 10% Mean IC_50_ (nM)	Mean IC_50_ (nM)	Bottom 10% Mean IC_50_ (nM)	Fold Difference in Mean Top 10% IC_50_
ALK < 20 nM RF	0.913	3.33	4200	16,000	4800
Split 1 Error	0.911	4.87	4200	17,000	3500
Split 2 Error	0.920	15.6	3300	12,000	770
Split 3 Error	0.930	9.74	4100	12,000	1200

## Supplementary Materials

The following are available online, Supporting Information—Target Molecular Biology, Activity Distribution, Support Vector Machine Experiments and Calculations; Supporting Information—Performance Statistics of Machine Learning Algorithms; Excel of Retrospective Predictive Performance for All Algorithms.
