# Supplementary material for: Error Tolerance of Machine Learning Algorithms across Contemporary Biological Targets
Source: Molecules. 2019 Jun 4;24(11):2115. doi: 10.3390/molecules24112115 (PMC6601015; doi:10.3390/molecules24112115)
Supplement: Supplementary file 1 [file molecules-24-02115-s001.zip › Supporting Information - Performance Statistics of Machine Learning Algorithms.pdf]

Error Tolerance of Machine Learning Algorithms across Contemporary Biological Targets

Supporting Information - Performance Statistics of Machine Learning Algorithms

Thomas M. Kaiser [1,*] and Pieter B. Burger [2,3,*]

[1] St Peter’s College, University of Oxford, New Inn Hall St, Oxford OX1 2DL , United Kingdom

[2] Department of Drug Discovery and Biomedical Sciences, College of Pharmacy, Medical University of South Carolina, 280 Calhoun St. MSC 141 Charleston, SC 29425-1410, United States

[3] Department of Chemistry, Emory University, 201 Dowman Drive, Atlanta, GA 30322, United States

*Correspondence: thomas.kaiser@spc.ox.ac.uk (T.M.K.); pieter.burger@gmail.com (P.B.B.)

ALK FEP Experiments

ALK Algorithm Design

- Rules - $RELATION$ = "=" OR $IC50$ > 999 OR $IC50$ < 10 => TRUE
- Sort ascending and remove duplicates
- DefGood was used as 20 nM
- 80-20 split for both actives and inactives
- NBN – ECFP4 used Classifier where Prediction was Active = True
- 1343 Compounds made it through the filter

NBN Error Tolerance- <20 nM DefGood in AKL

- 27% error confined to the range of 3.7 – 109 nM

<20 nM DefGood in AKL, 27% error (FEP interval); Random seed = 1515533876005

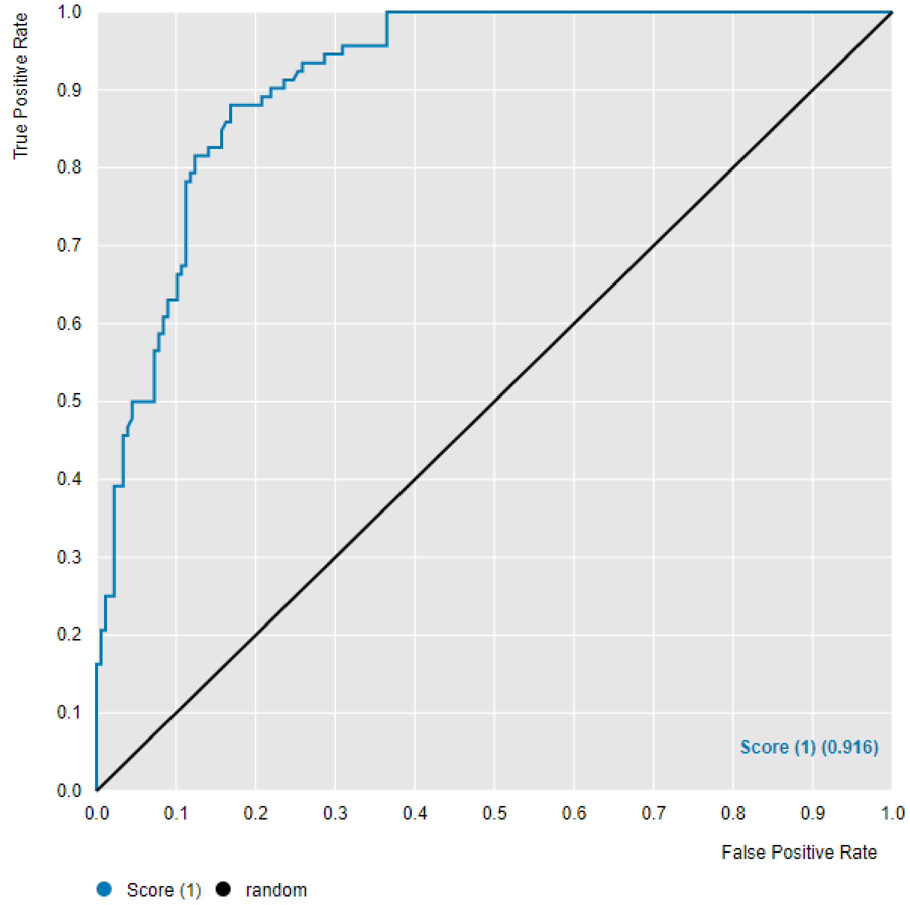

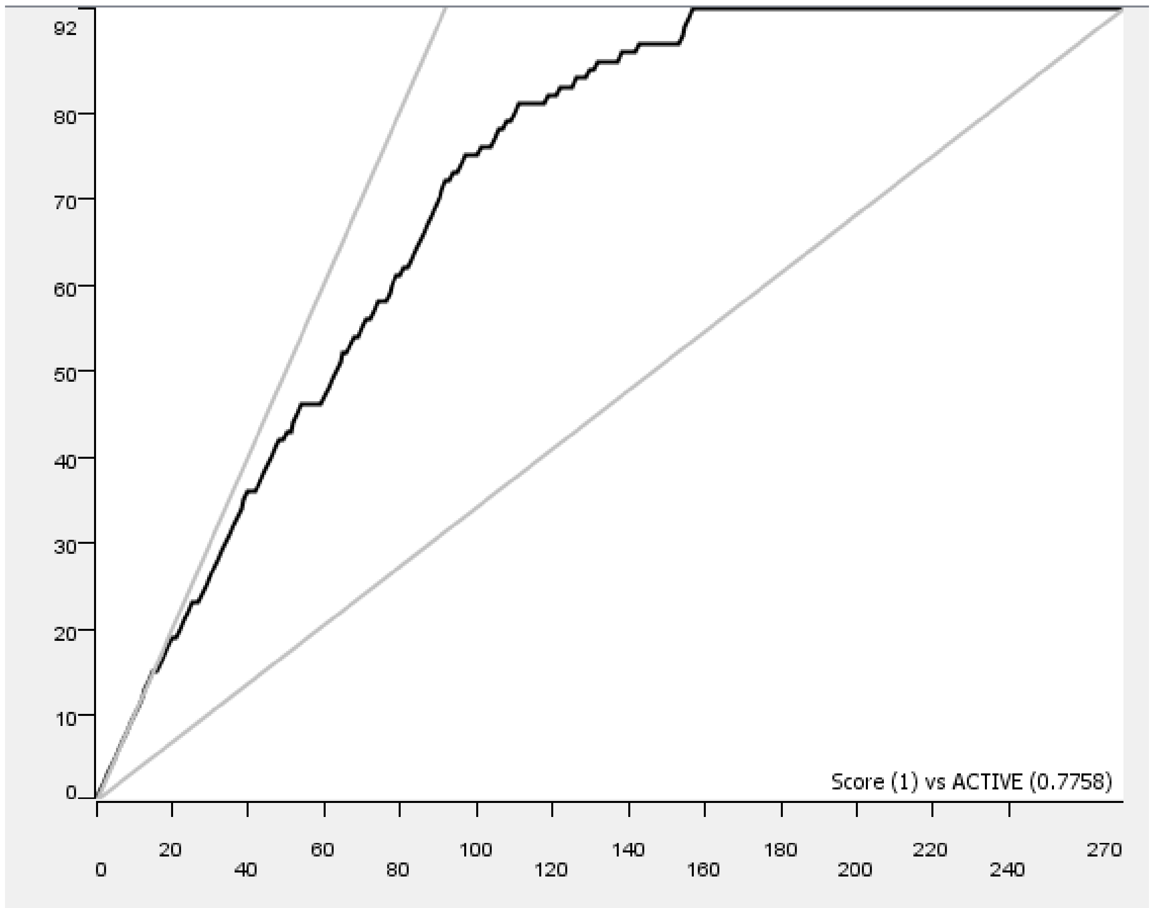

N=270

Top 10% Mean IC50	18.0 nM
Bottom 10% Mean IC50	17,000 nM

	Predicted Active	Predicted Inactive
Active	79	13
Inactive	29	149

<20 nM DefGood in AKL, 27% error (FEP interval); Random seed = 429

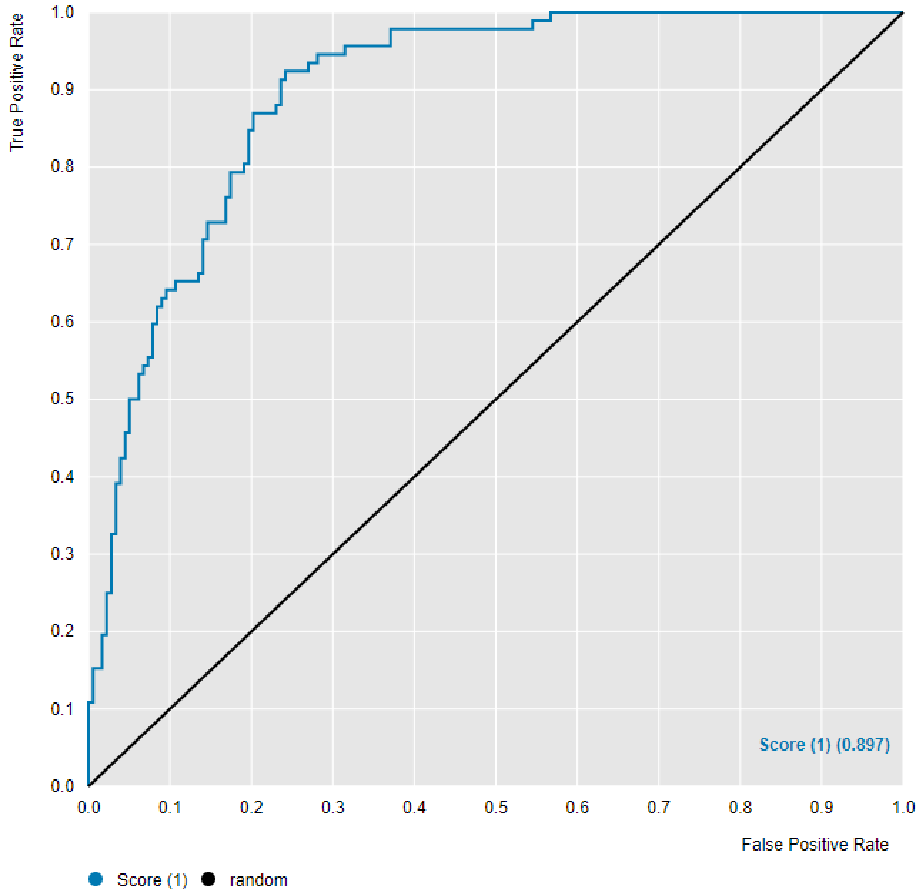

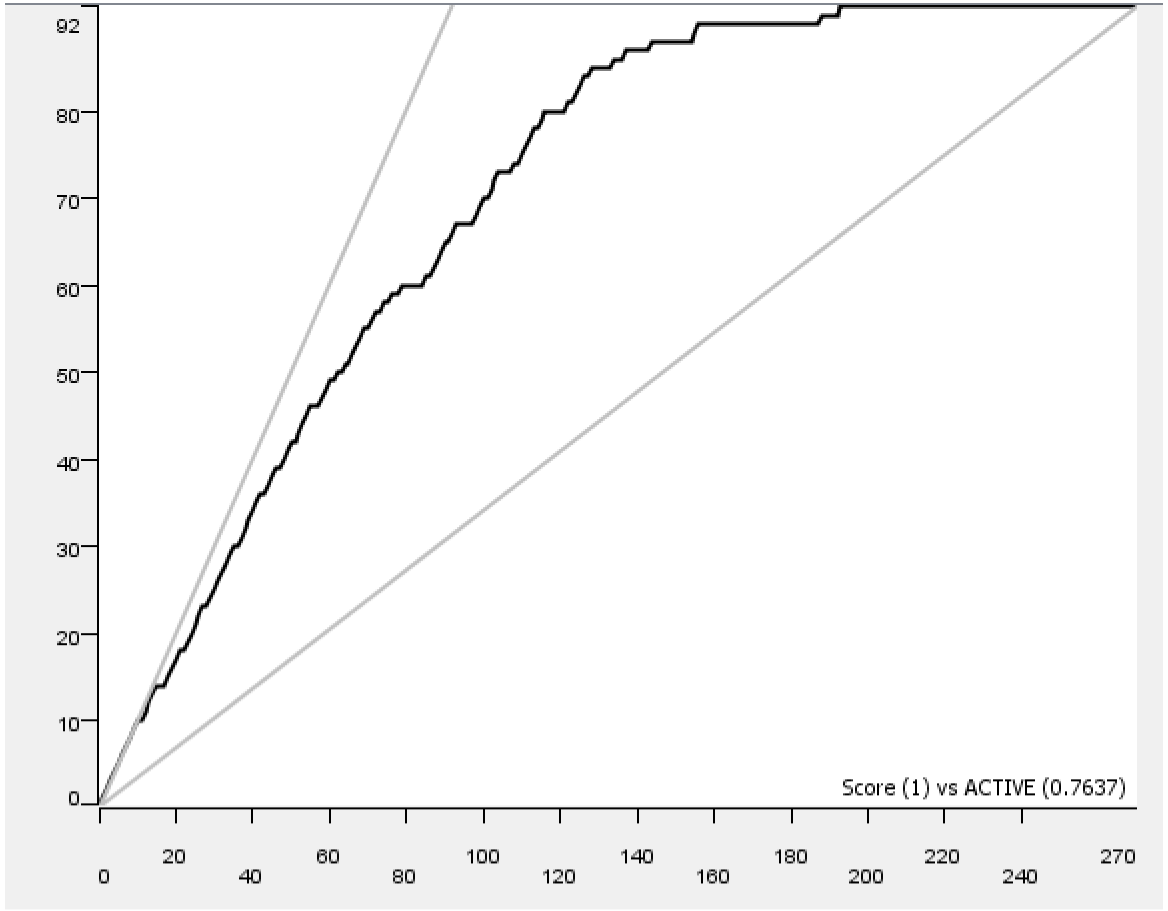

N=270

**Top 10% Mean IC50**	**22.5 nM**
Bottom 10% Mean IC50	7,000 nM

	**Predicted Active**	**Predicted Inactive**
Active	85	7
Inactive	45	133

<20 nM DefGood in AKL, 27% error (FEP interval): Random seed = 121783

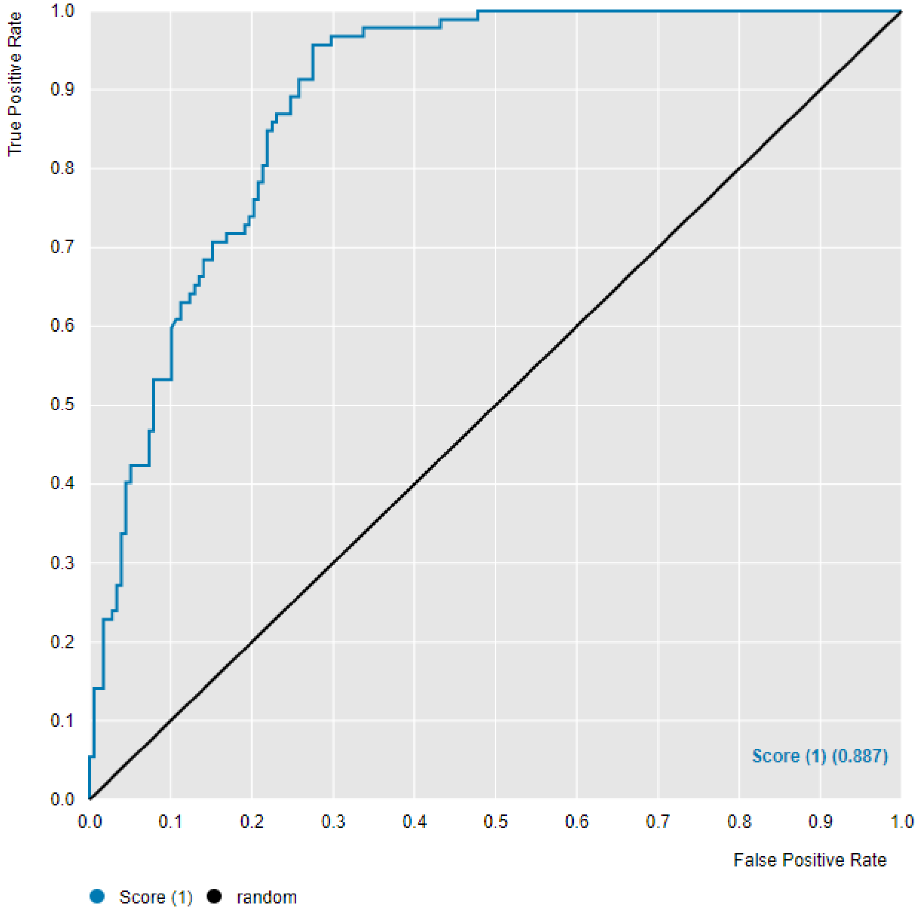

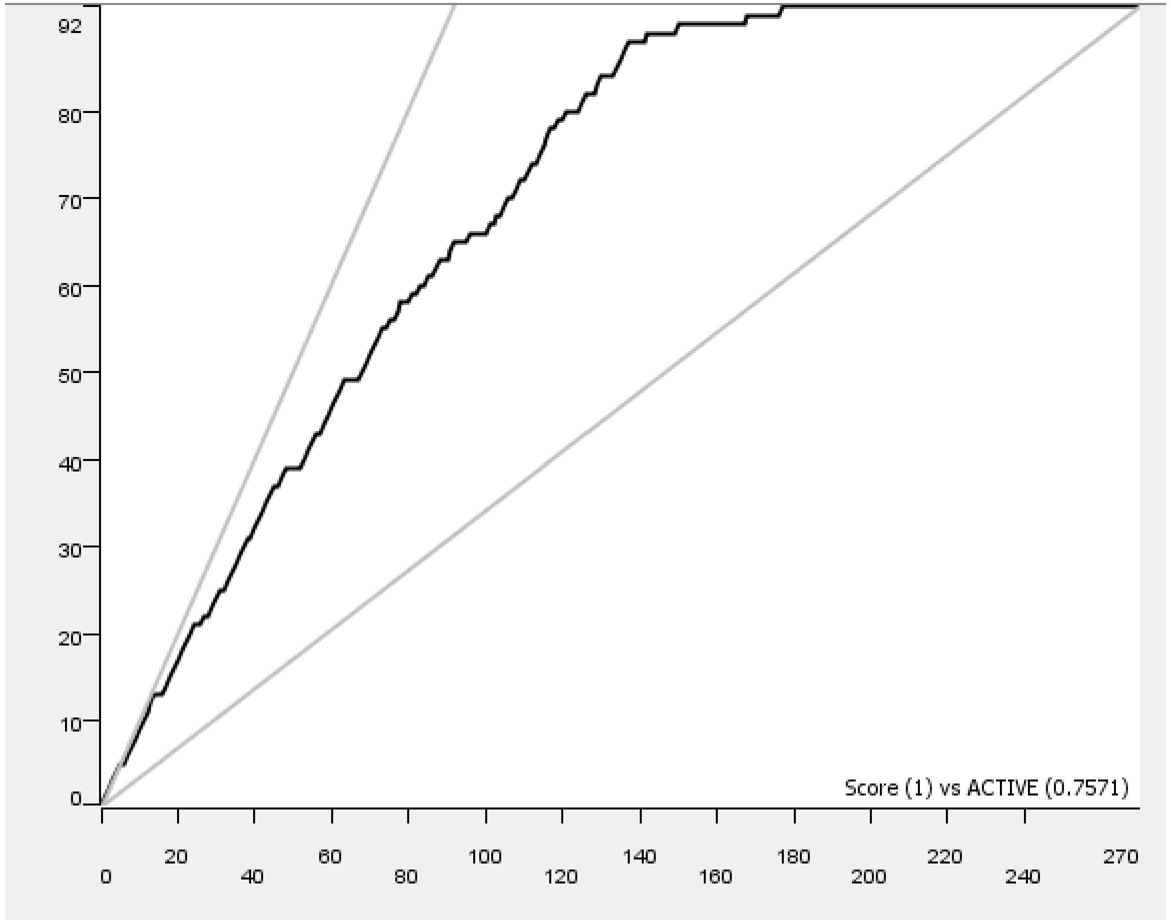

N=270

**Top 10% Mean IC50**	**99.8 nM**
Bottom 10% Mean IC50	14,000 nM

	Predicted Active	Predicted Inactive
Active	80	12
Inactive	42	136

Random Forrest Error Tolerance- <20 nM DefGood in AKL

- 27% error confined to the range of 3.7 – 109 nM

RF <20 nM DefGood in AKL, 27% error (FEP interval); Random seed = 1515533876005

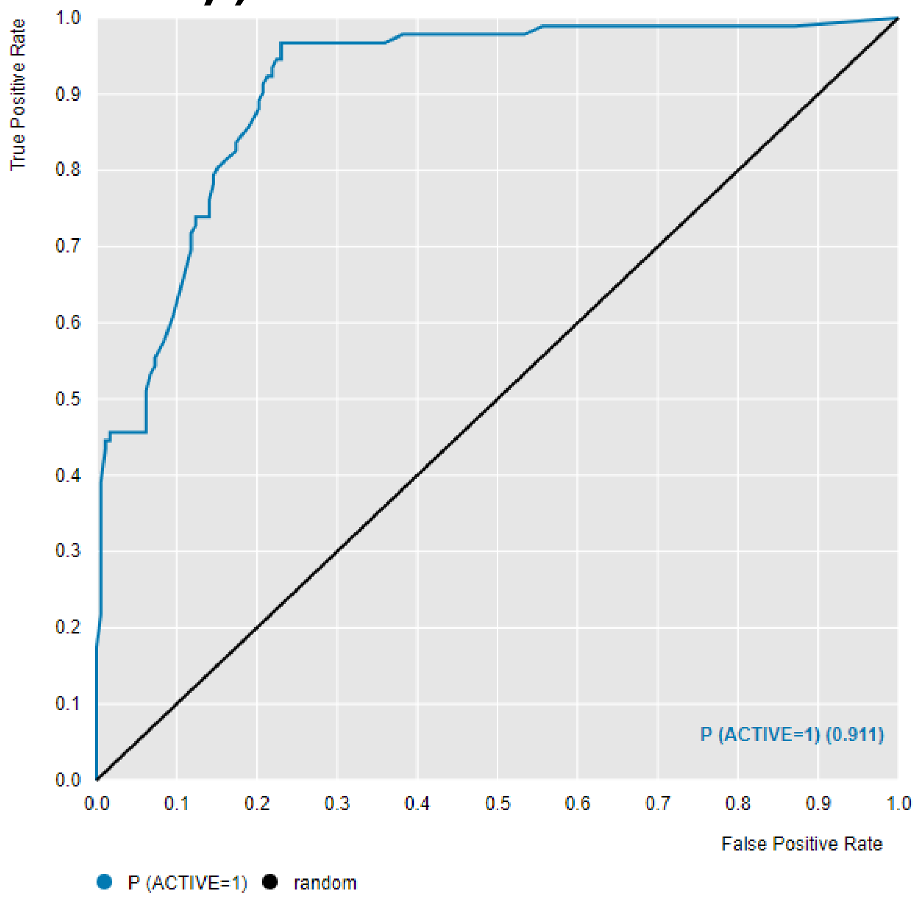

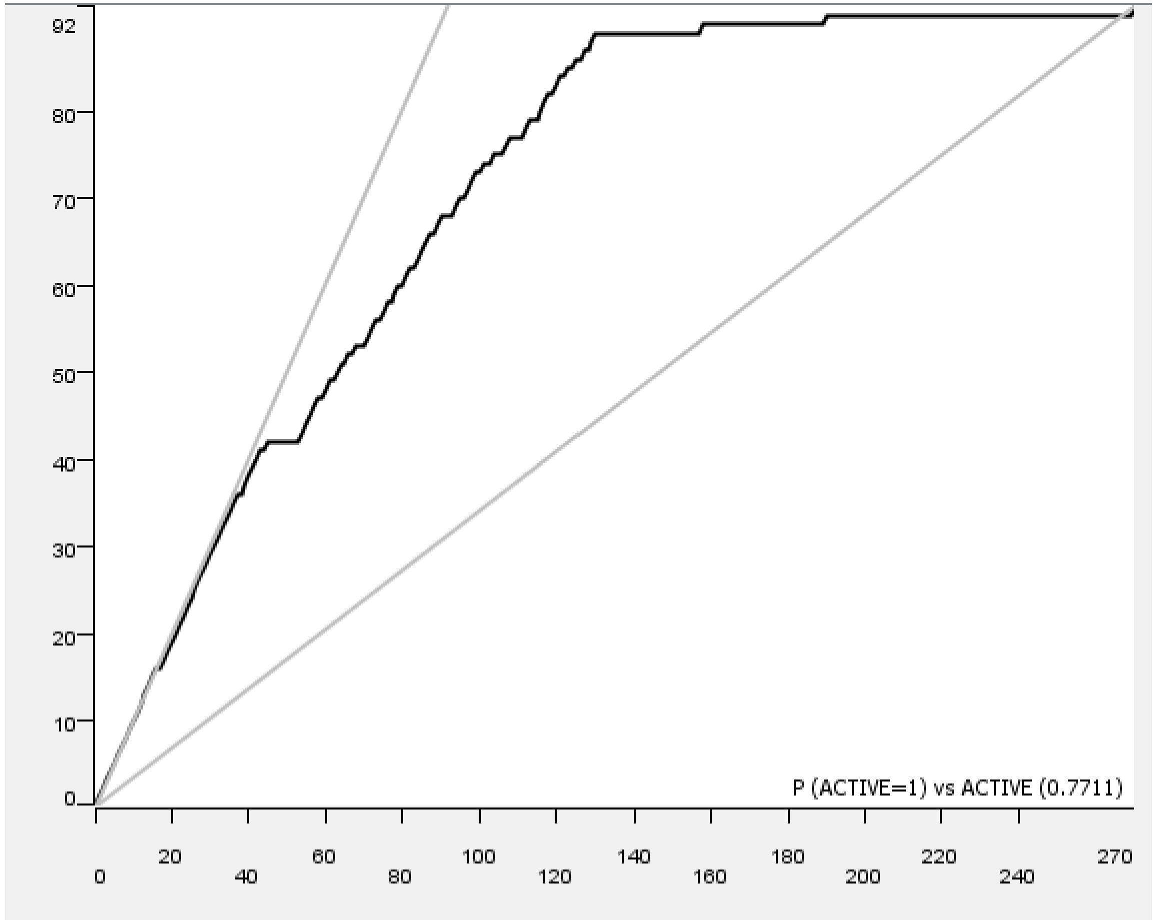

N=270

Top 10% Mean IC50	4.87 nM
Bottom 10% Mean IC50	17,000 nM

	Predicted Active	Predicted Inactive
Active	68	24
Inactive	22	156

RF <20 nM DefGood in AKL, 27% error (FEP interval); Random seed = 429

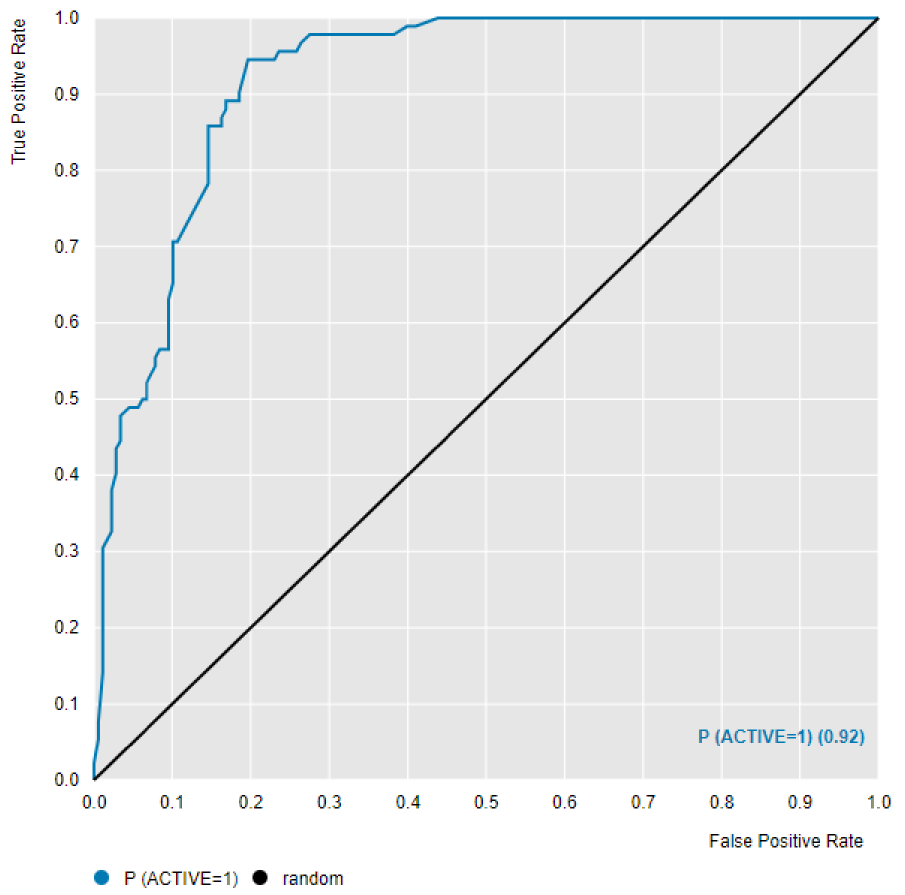

N=270

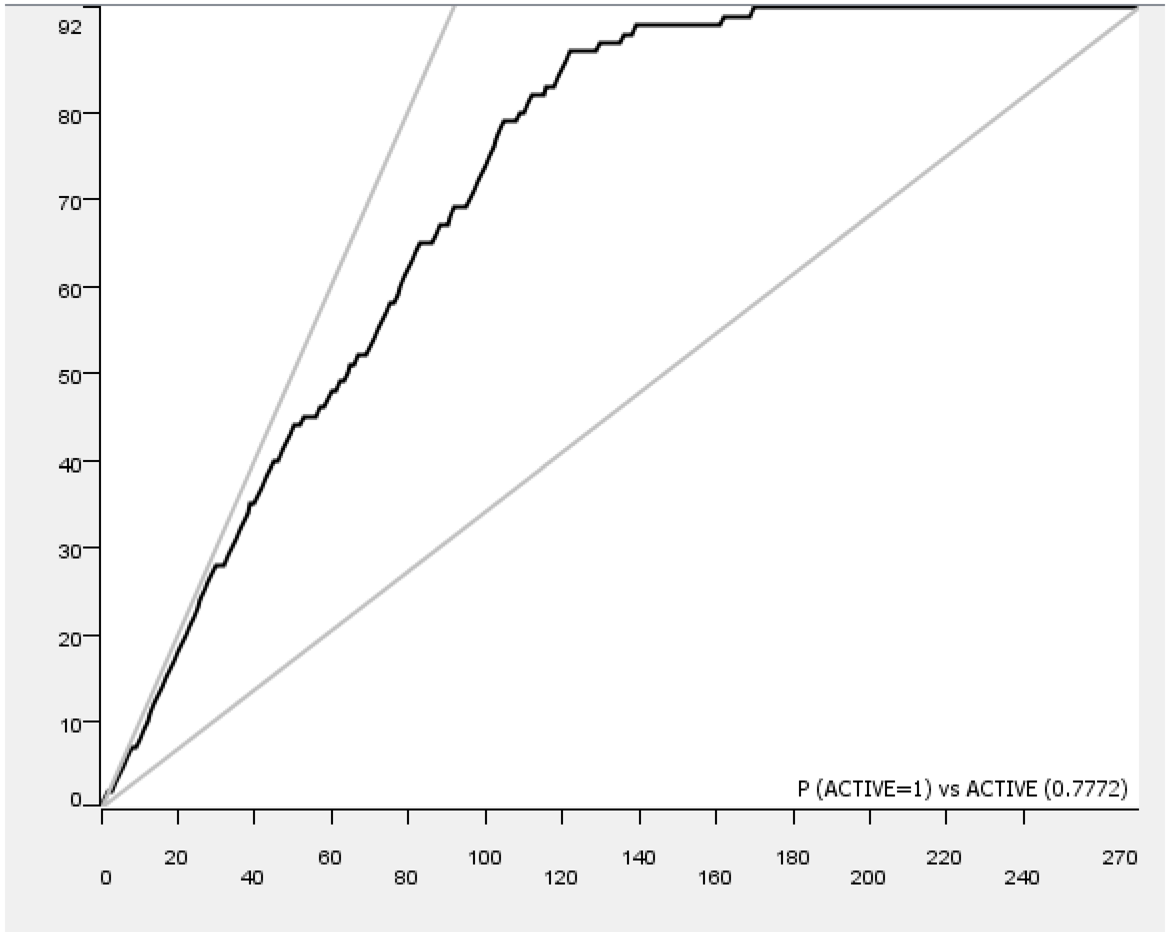

Top 10% Mean IC50	15.6 nM
Bottom 10% Mean IC50	7,000 nM

	Predicted Active	Predicted Inactive
Active	79	13
Inactive	28	150

RF<20 nM DefGood in AKL, 27% error (FEP interval); Random seed = 121783

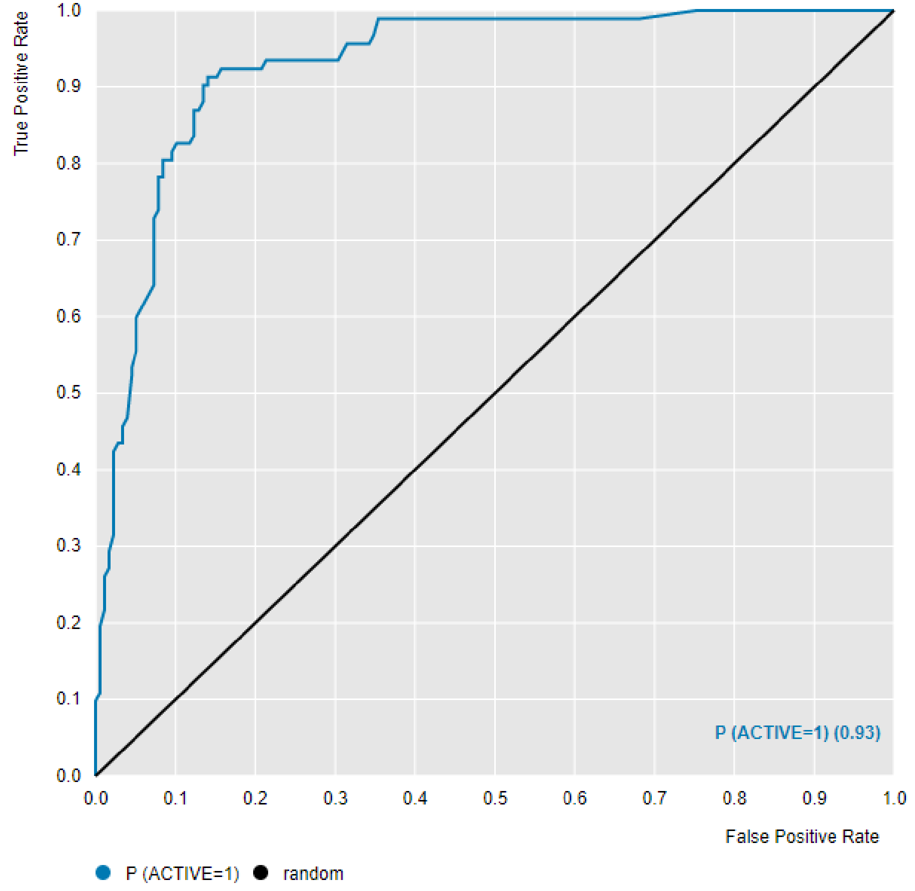

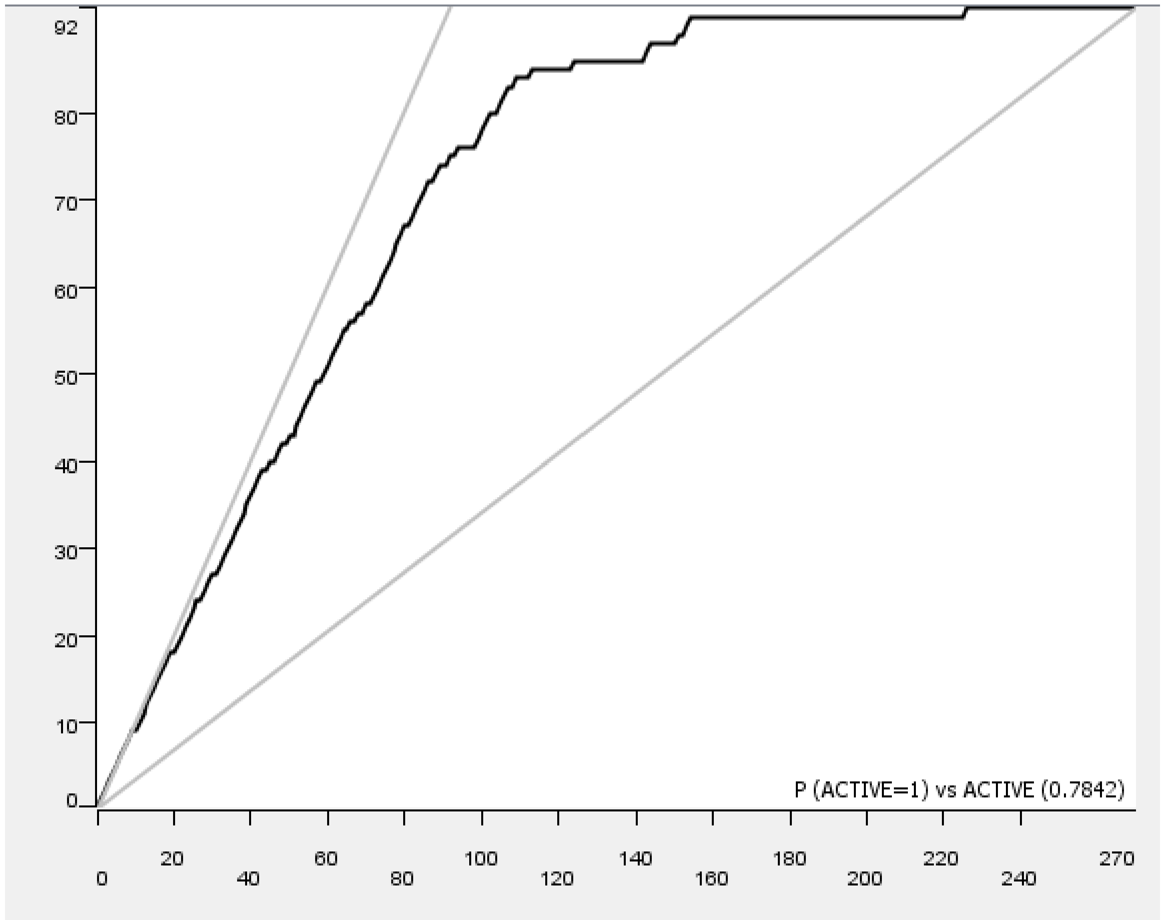

N=270

**Top 10% Mean IC50**	**9.74 nM**
Bottom 10% Mean IC50	12,000 nM

	Predicted Active	Predicted Inactive
Active	75	17
Inactive	17	161

ALK

ALK NBN Design

- Rules - $RELATION$ = "=" OR $IC50$ > 999 OR $IC50$ < 10 => TRUE
- Sort ascending and remove duplicates
- DefGood scanned between 5 and 25 nM
- 80-20 split for both actives and inactives
- NBN – ECFP4 used Classifier where Prediction was Active = True
- 1343 Compounds made it through the filter

<5 nM DefGood in ALK

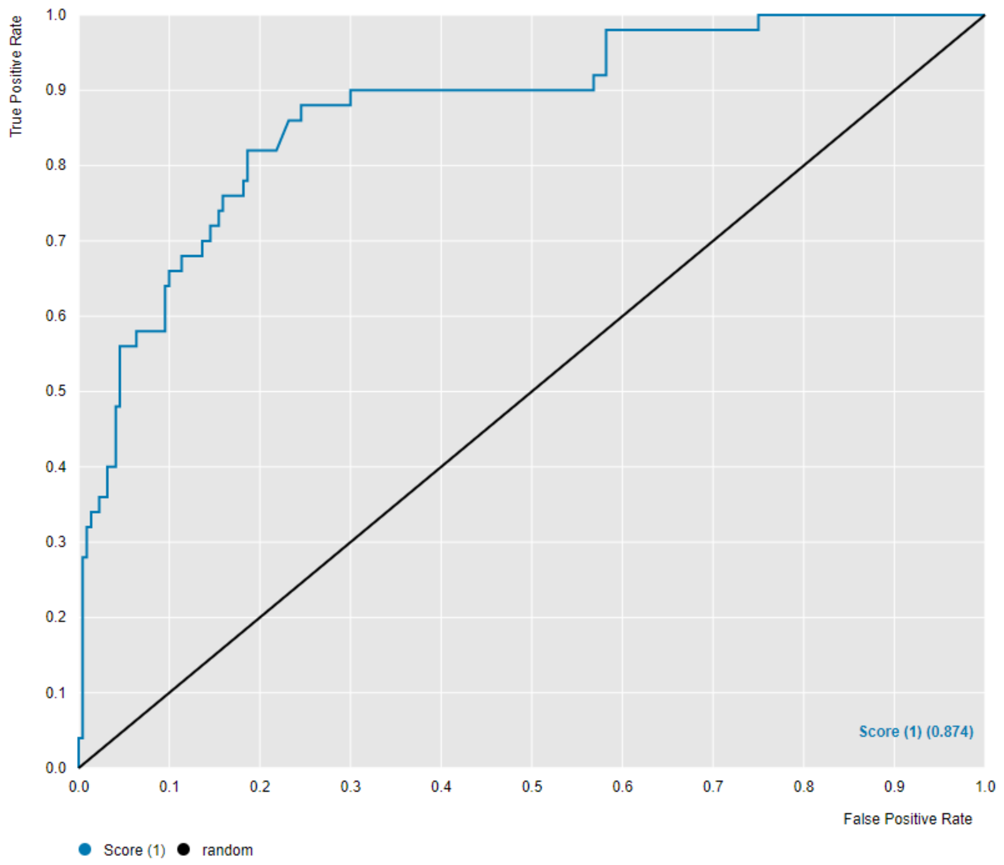

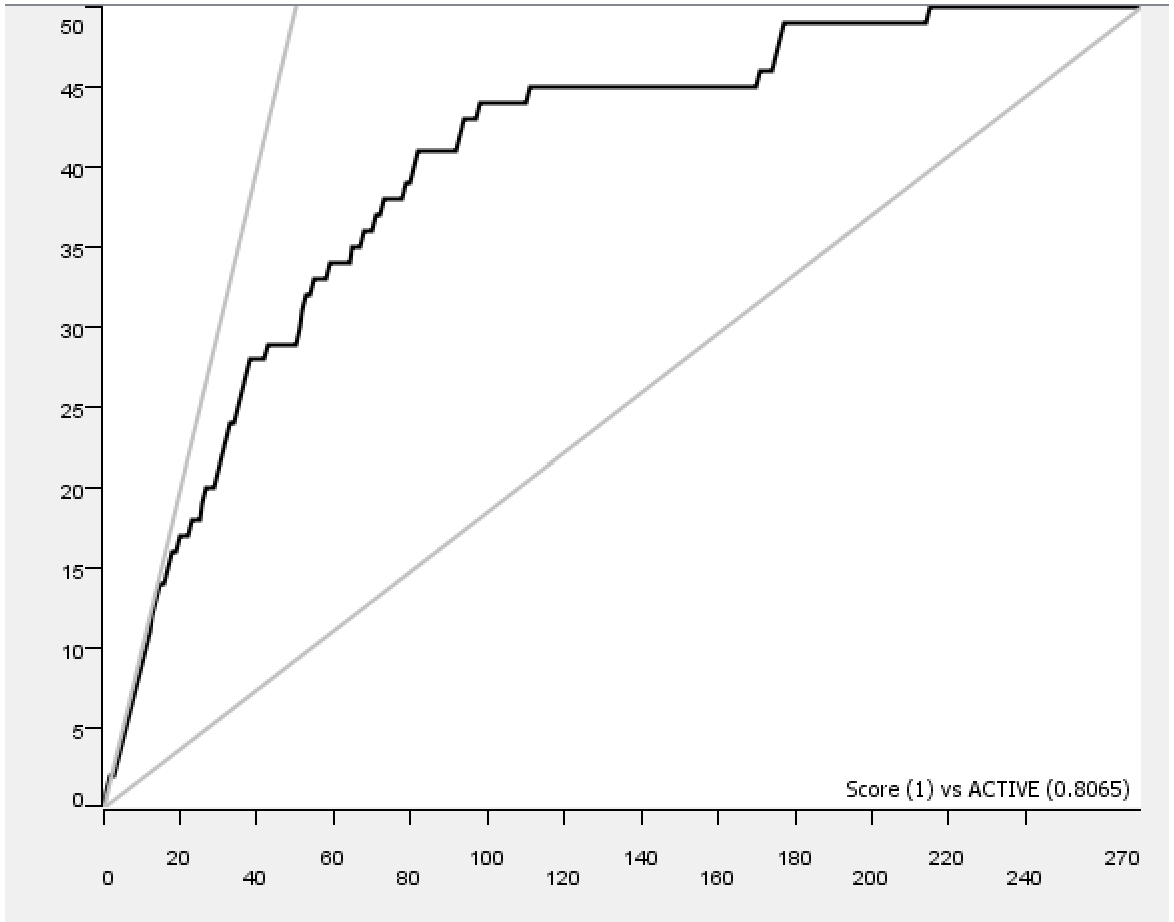

N=270

**Top 10% Mean IC50**	**5.3 nM**

	**Predicted Active**	**Predicted Inactive**
Active	44	6
Inactive	55	165

<10 nM DefGood in ALK

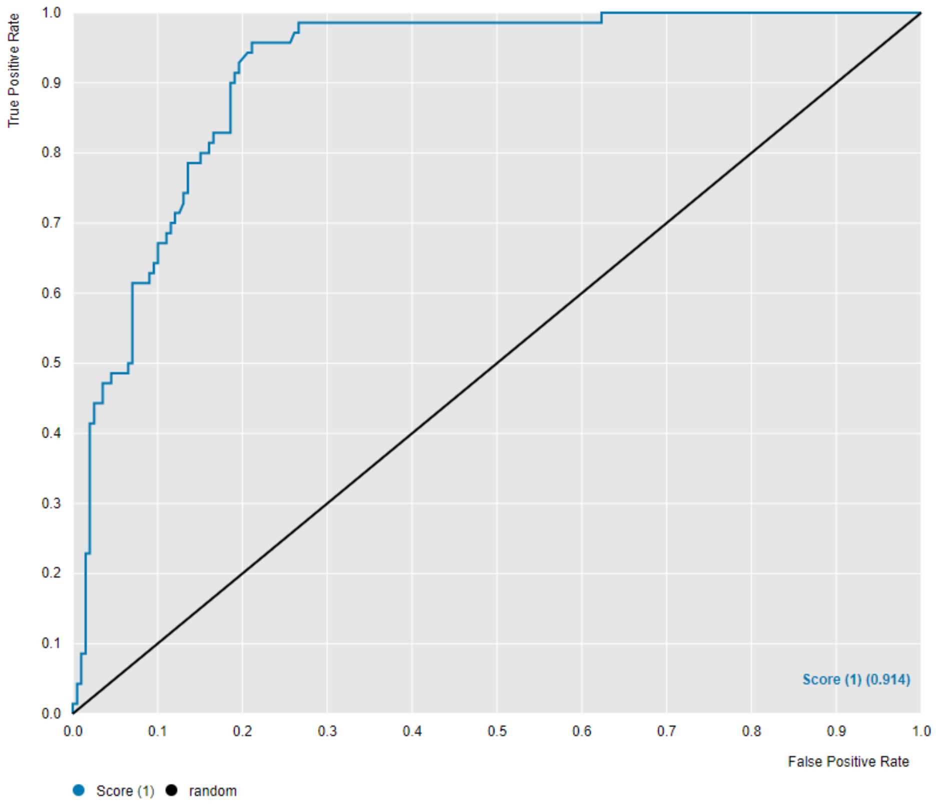

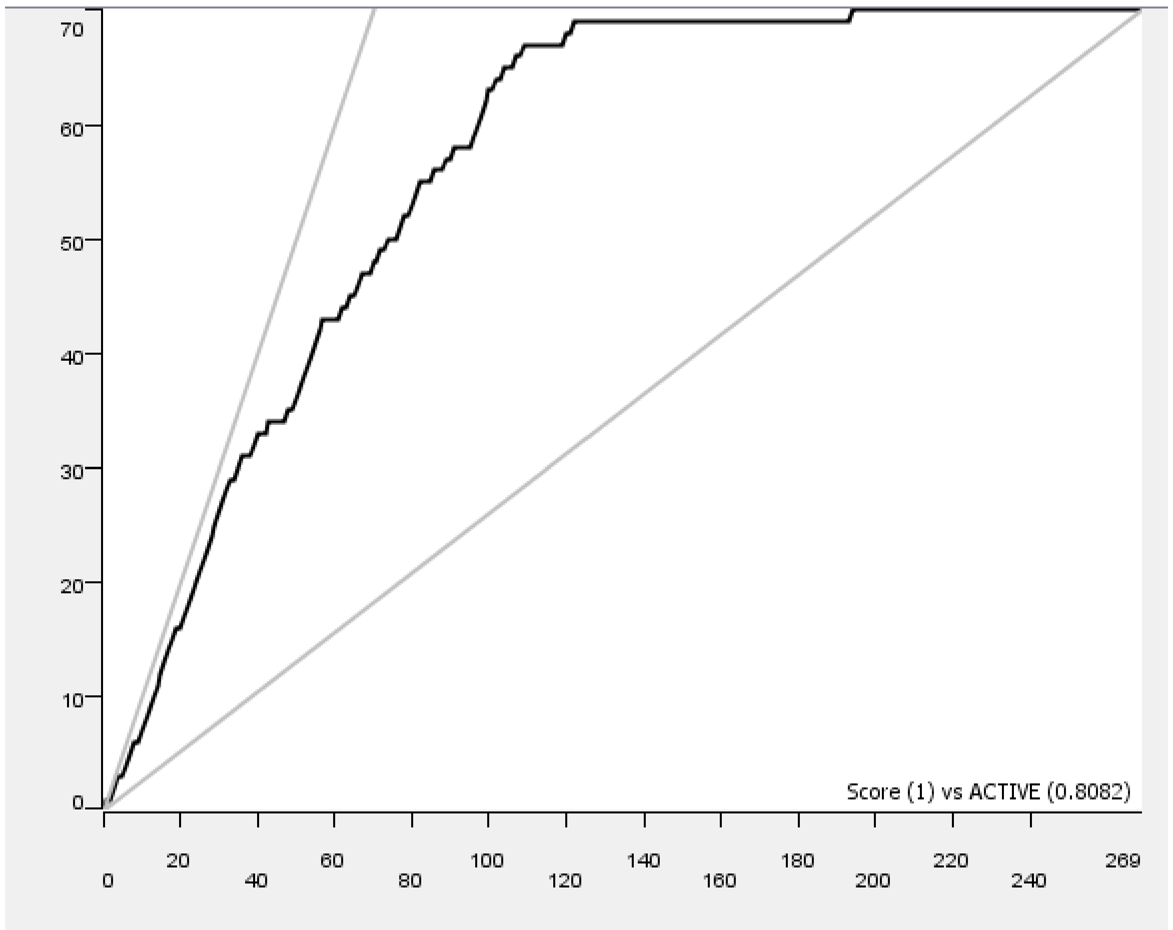

N=269

Top 10% Mean IC50	14.6 nM

	Predicted Active	Predicted Inactive
Active	67	3
Inactive	45	154

<15 nM DefGood in ALK

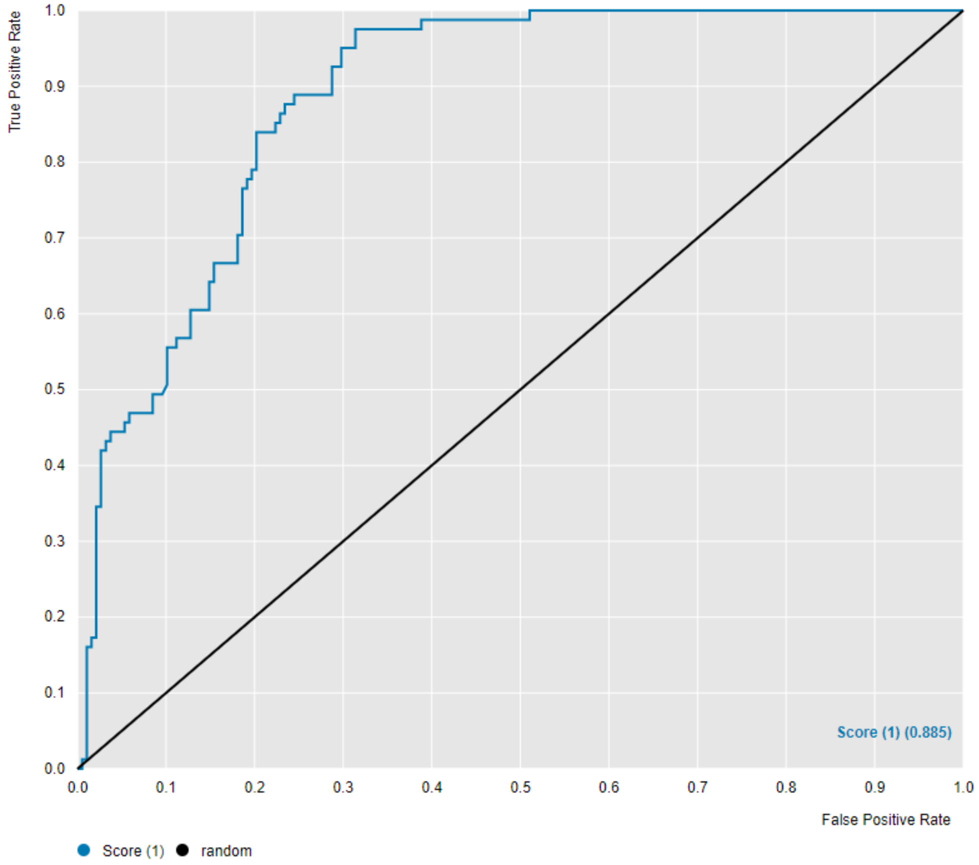

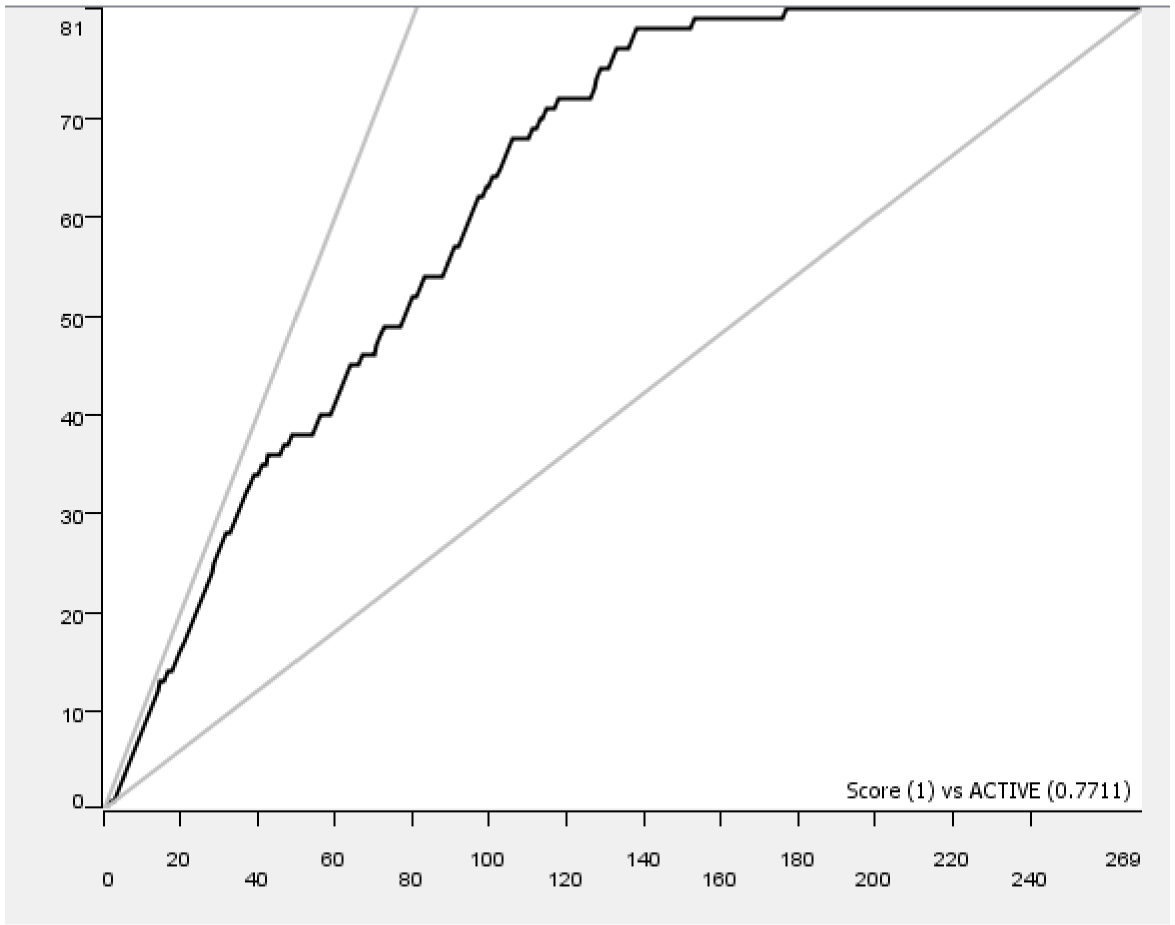

N=269

Top 10% Mean IC50	7.3 nM

	Predicted Active	Predicted Inactive
Active	71	10
Inactive	46	142

<20 nM DefGood in ALK

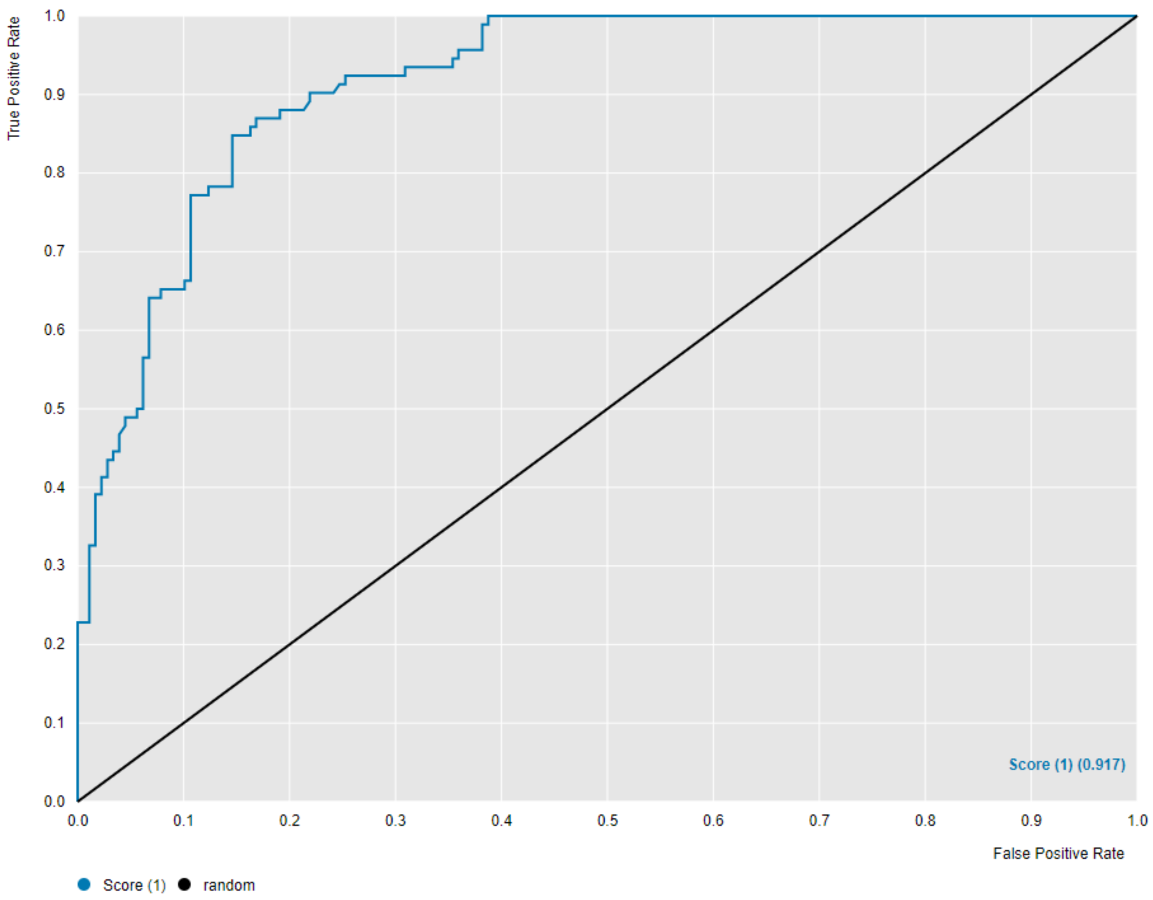

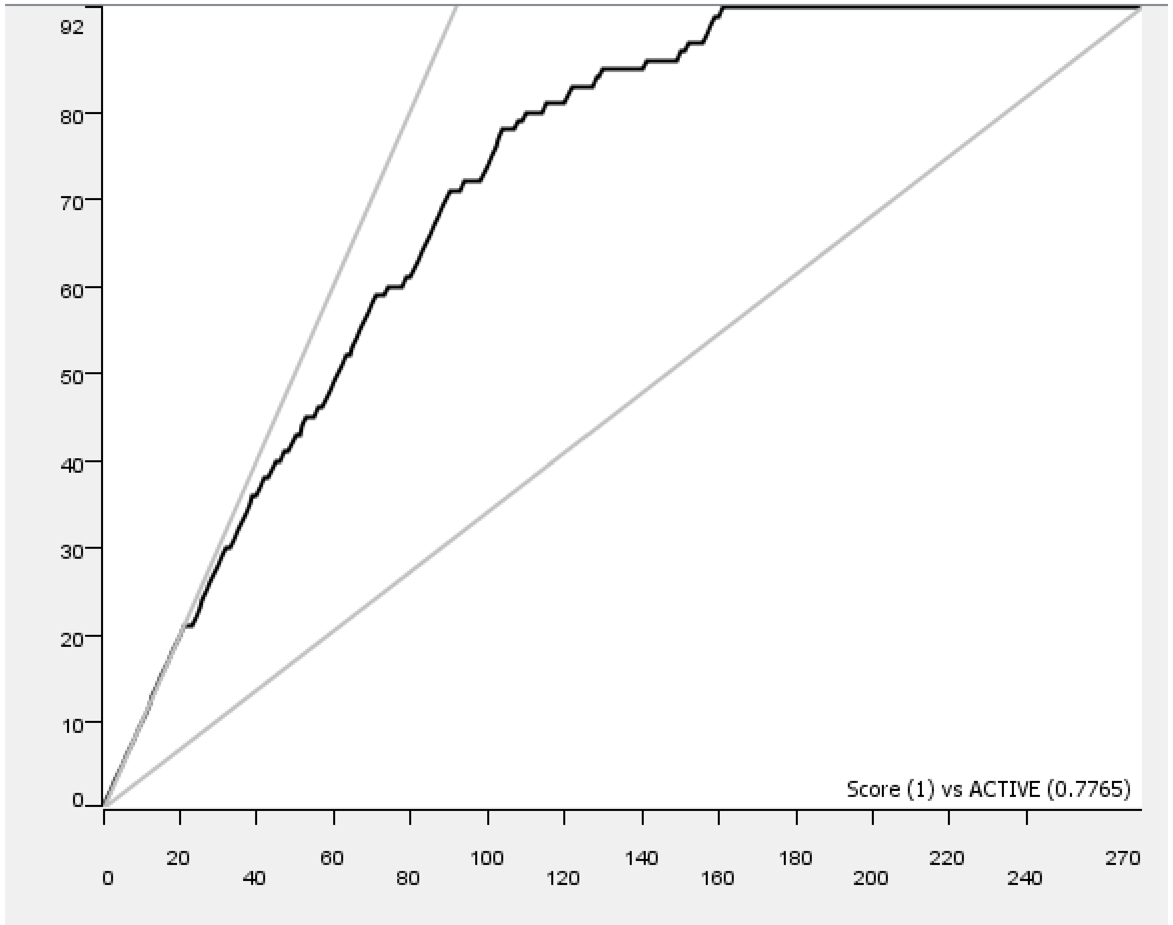

N=270

**Top 10% Mean IC50**	**6.7 nM**

	Predicted Active	Predicted Inactive
Active	82	10
Inactive	39	139

<25 nM DefGood in ALK

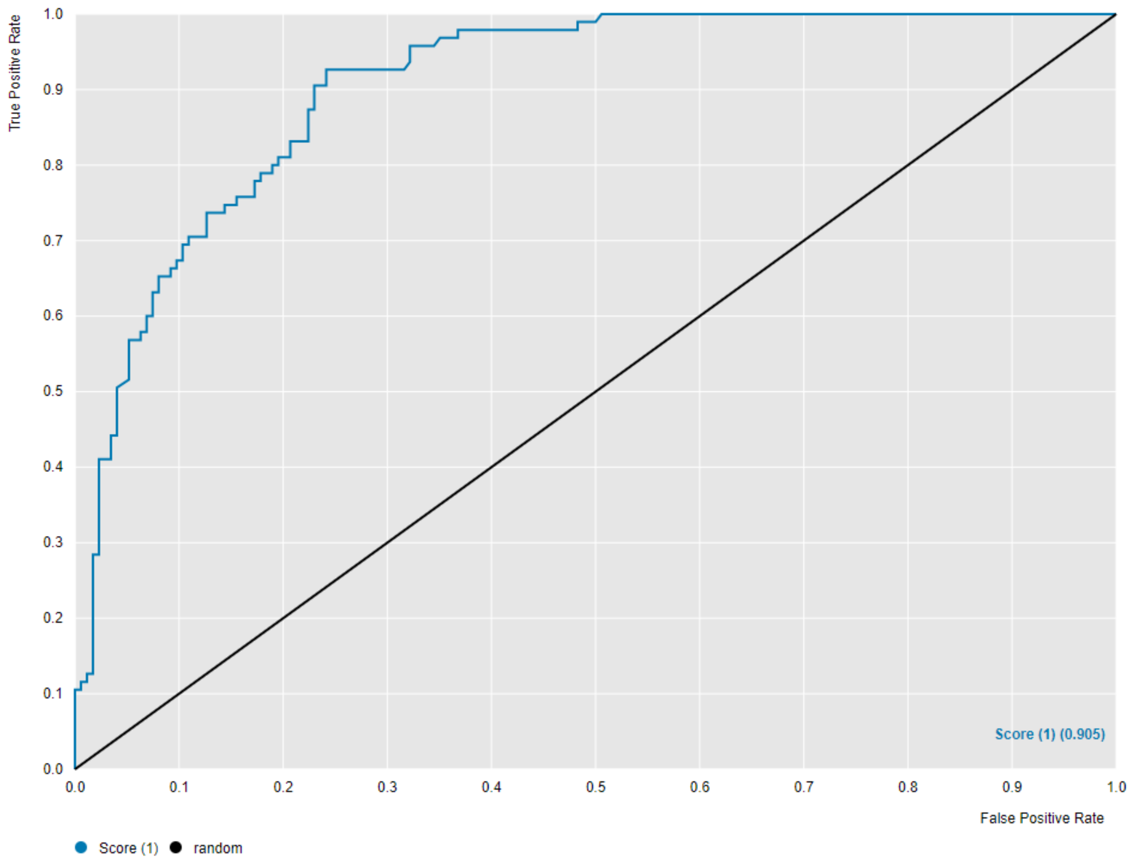

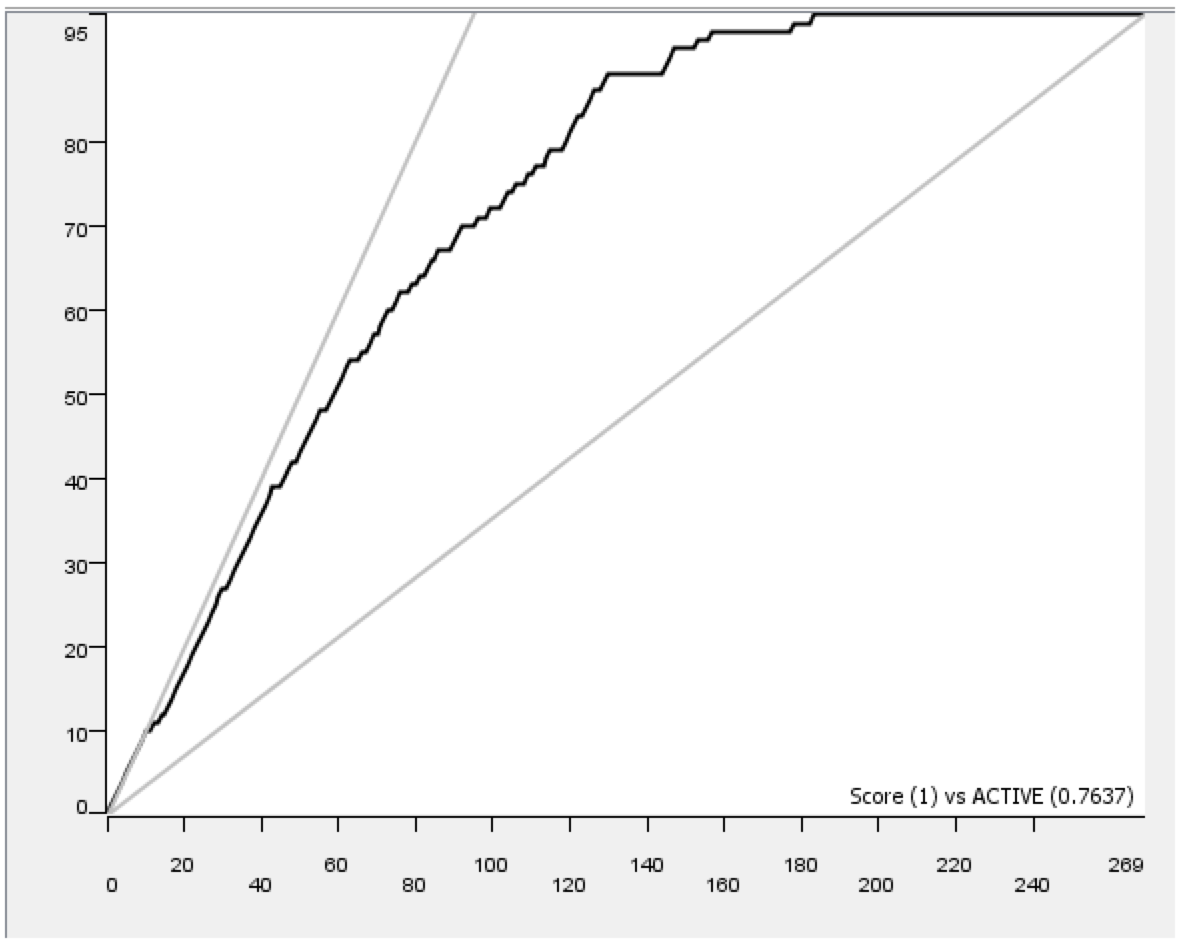

N=269

Top 10% Mean IC50	19.1 nM

	Predicted Active	Predicted Inactive
Active	88	7
Inactive	44	130

Decision on DefGood

- <20 nM has the best performance overall. <20 nM may not have the best top 10% IC50, but it is second best at 6.7 and the chemical space defined as good is much larger than the <5 nM NBN without losing significant potency on average. Also the specificity is much higher.

RF - <15 nM DefGood in ALK

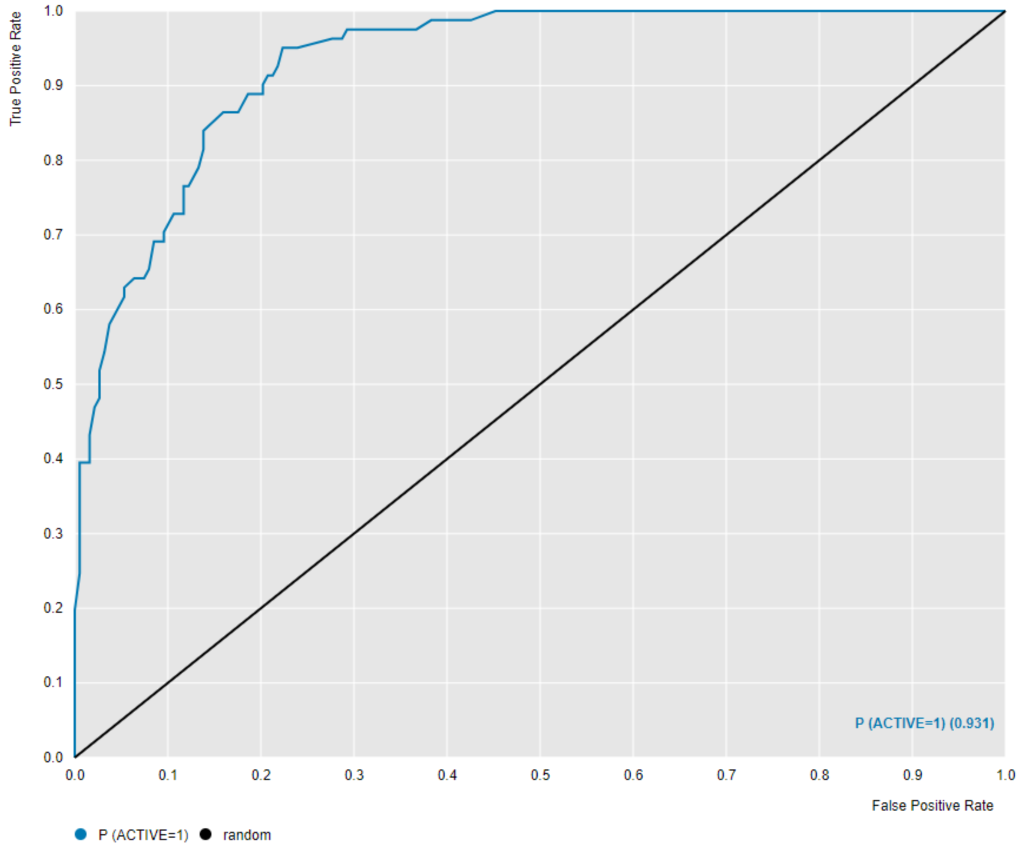

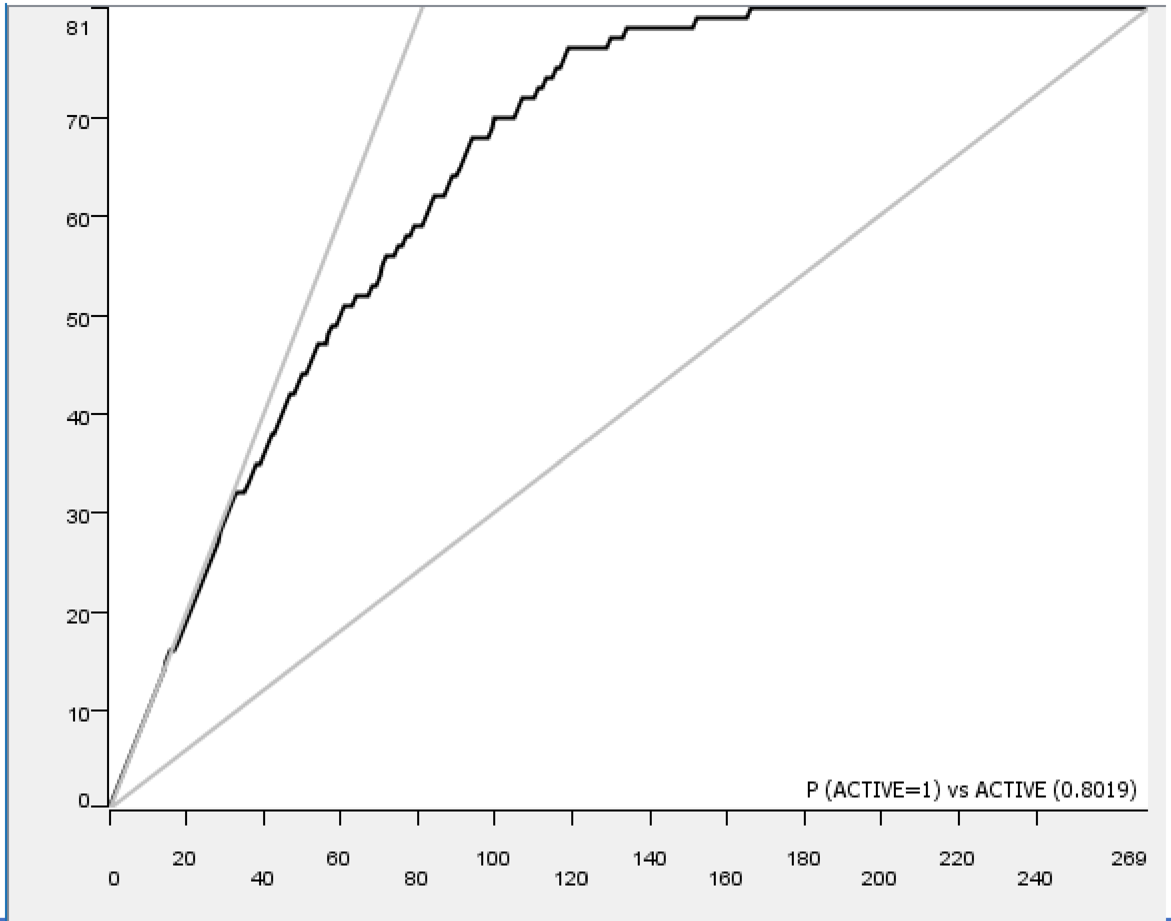

N=269

**Top 10% Mean IC50**	**3.9 nM**

	Predicted Active	Predicted Inactive
Active	61	20
Inactive	22	166

RF - <20 nM DefGood in ALK

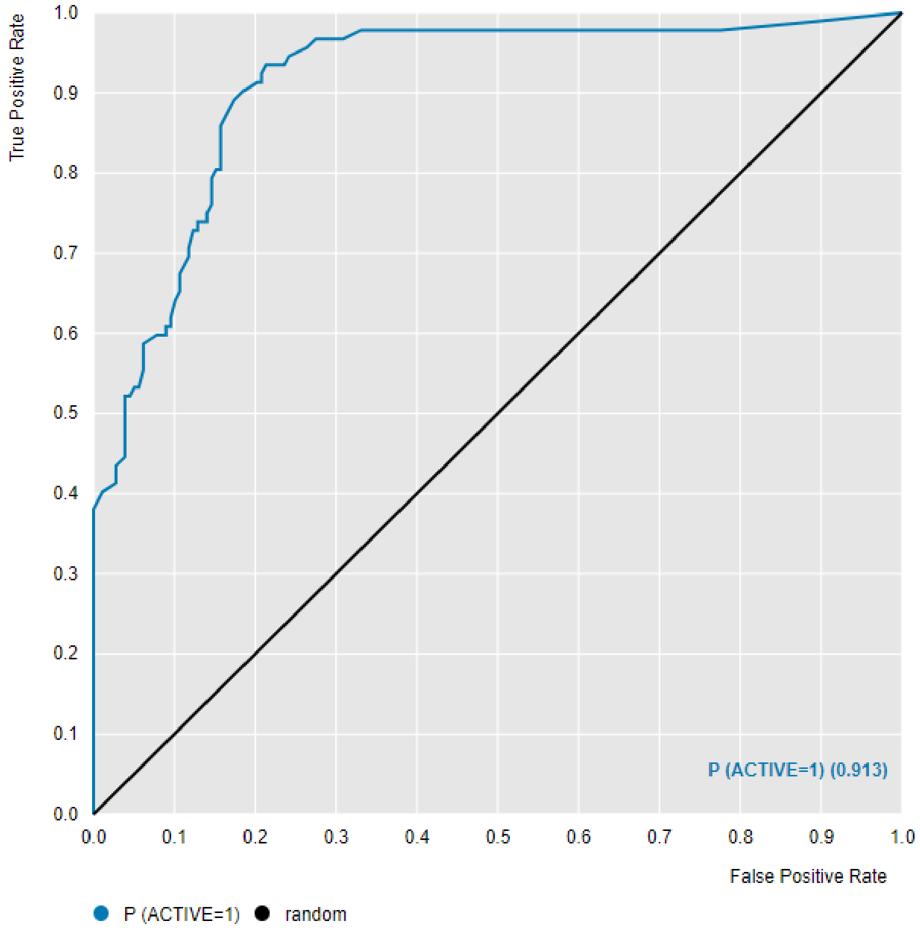

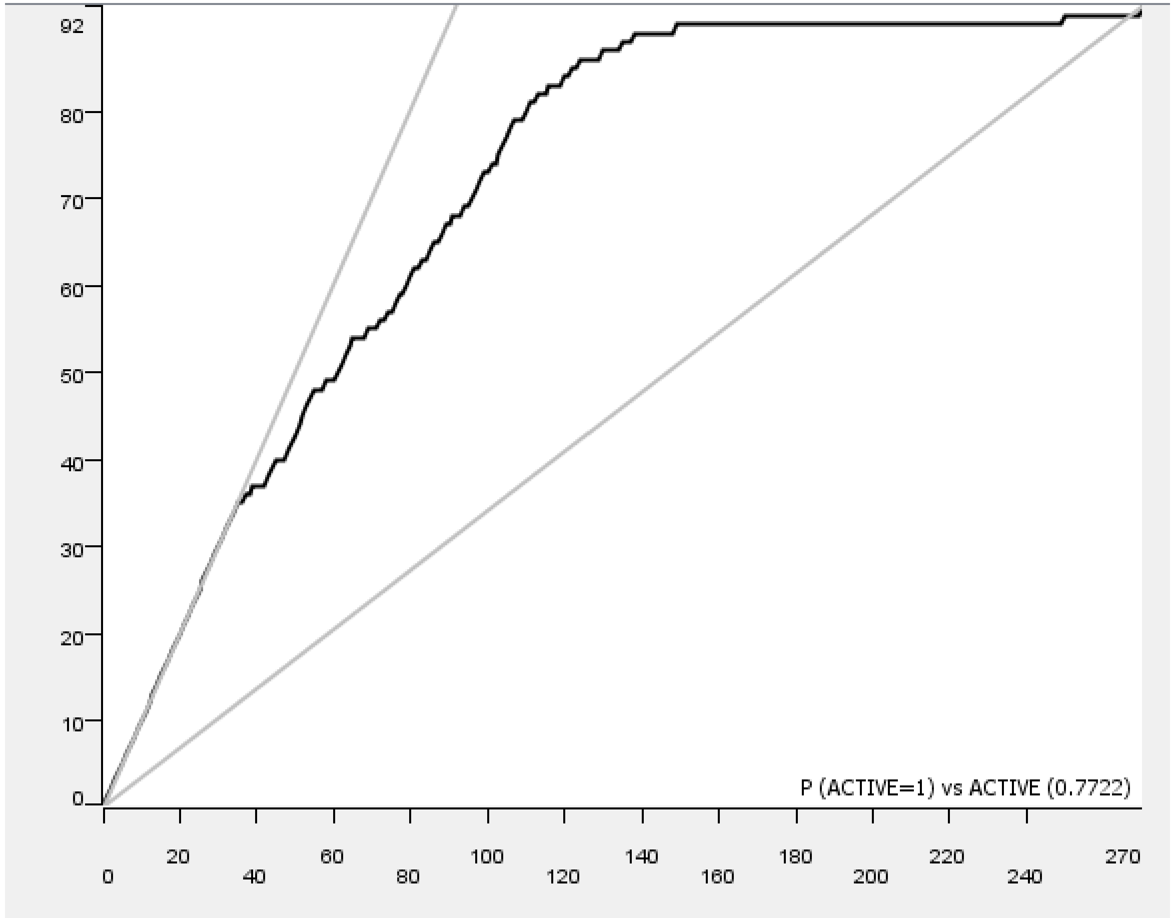

N=269

Top 10% Mean IC50	3.3 nM

	Predicted Active	Predicted Inactive
Active	68	24
Inactive	24	154

RF - <25 nM DefGood in ALK

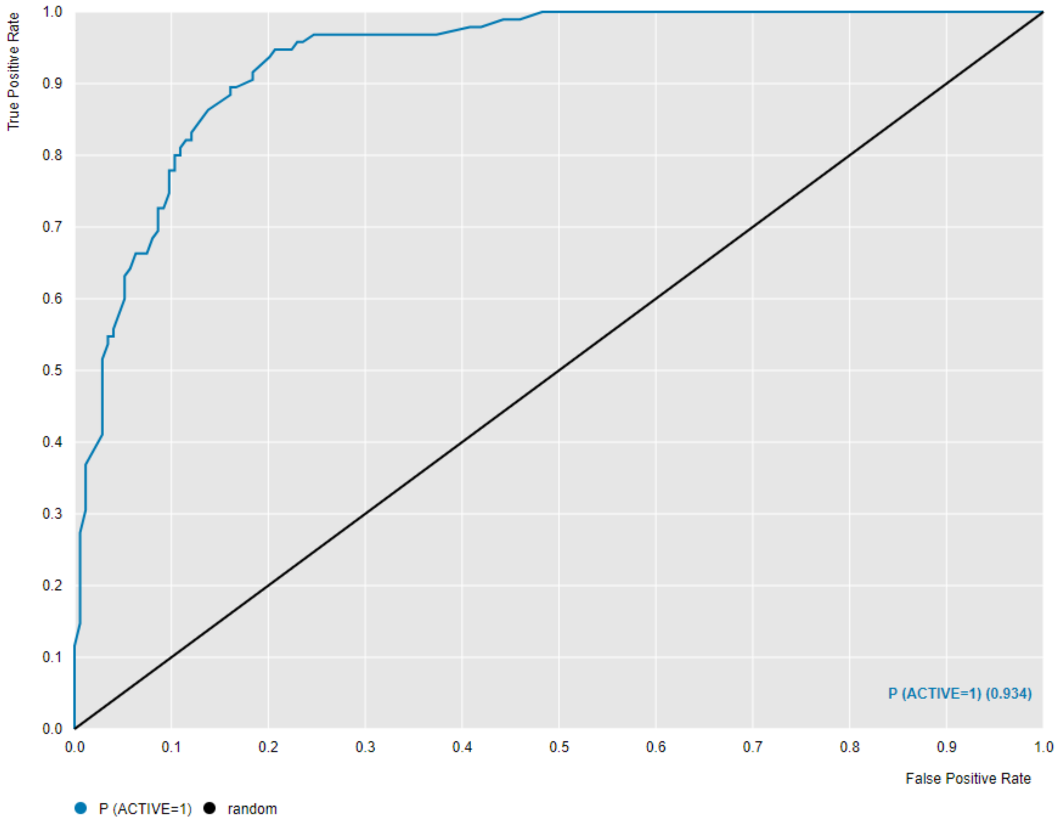

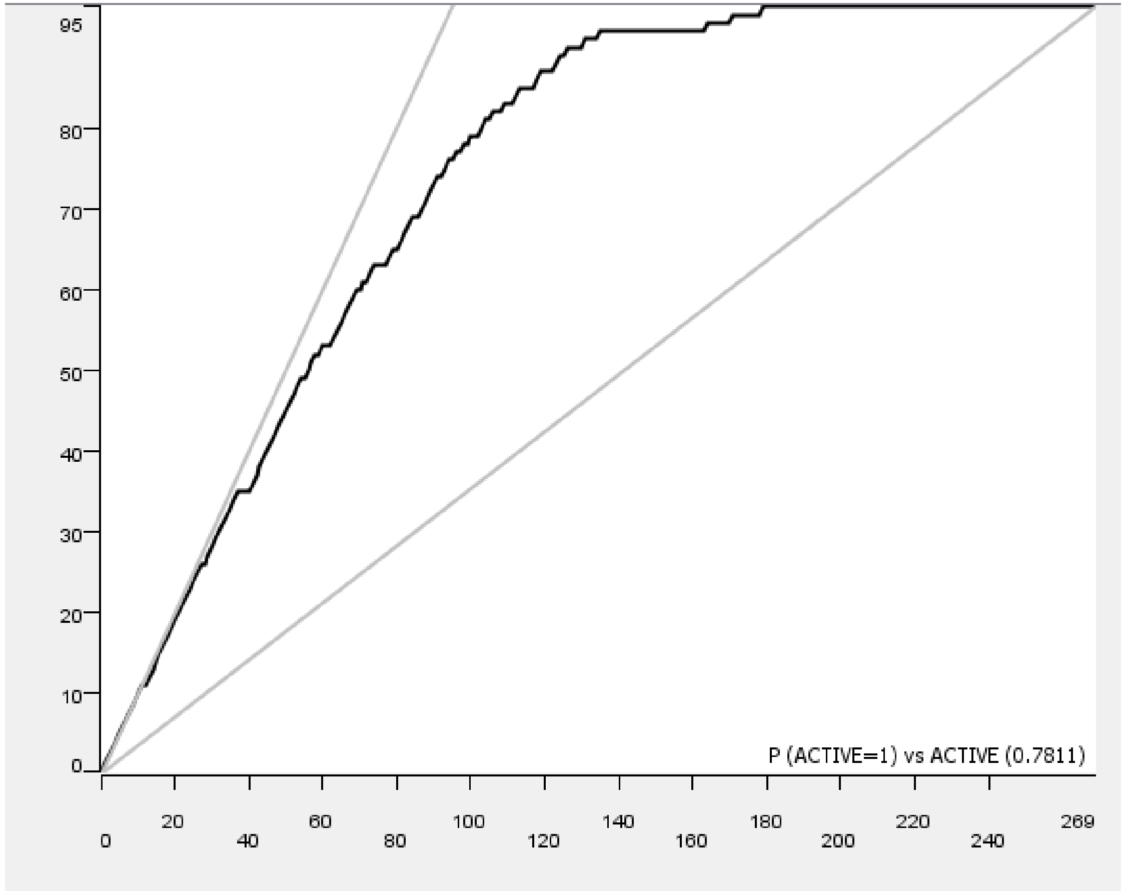

N=269

**Top 10% Mean IC50**	**14.5 nM**

	Predicted Active	Predicted Inactive
Active	76	19
Inactive	19	155

Decision on DefGood - RF

- <20 nM has the best performance overall. This mirrored the NBN DefGood, and the DefGood for the NBNs in the future will be used for the RFs as well.

PNN - <20 nM DefGood in AKL

- Properties List (46 calculatable properties were used)
- Parameterization (Global Accuracy for Activity at a 20 nM decision value was used, and a 5-fold stratified sampling cross validation was used to parameterize theta minus and theta plus) Theta Minus = 0.35 and Theta Plus = 0.65

PNN - <20 nM DefGood in AKL

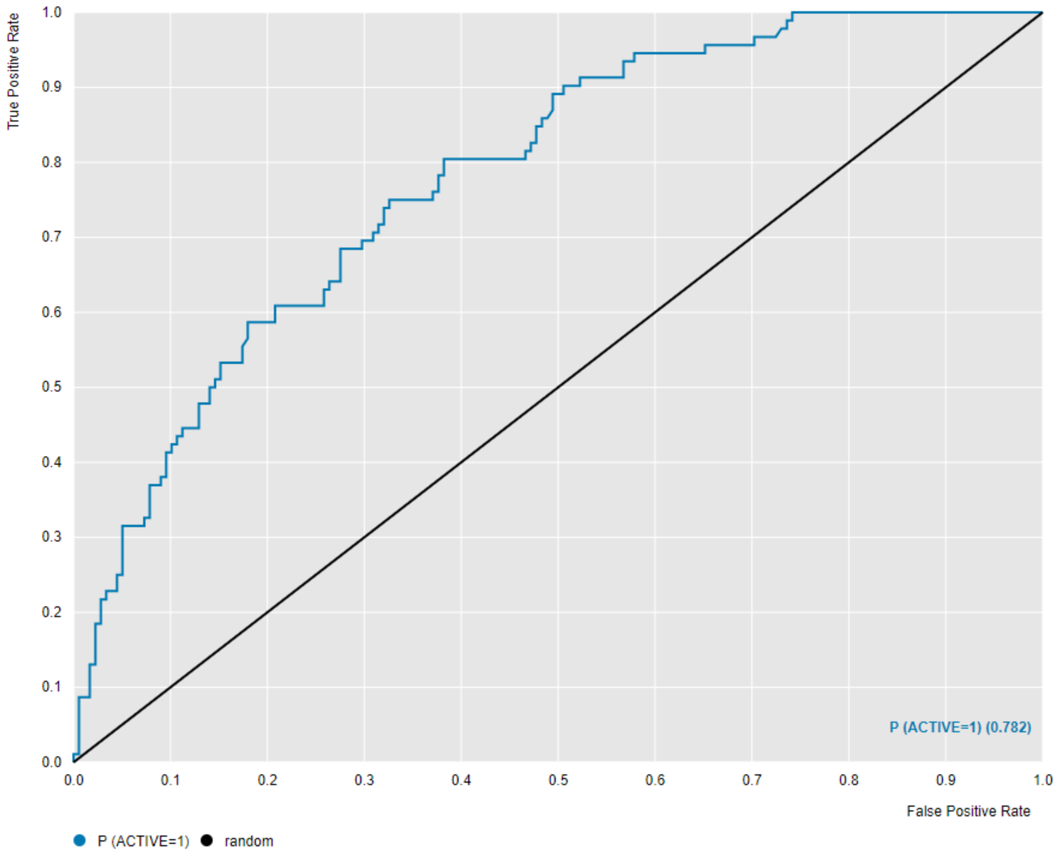

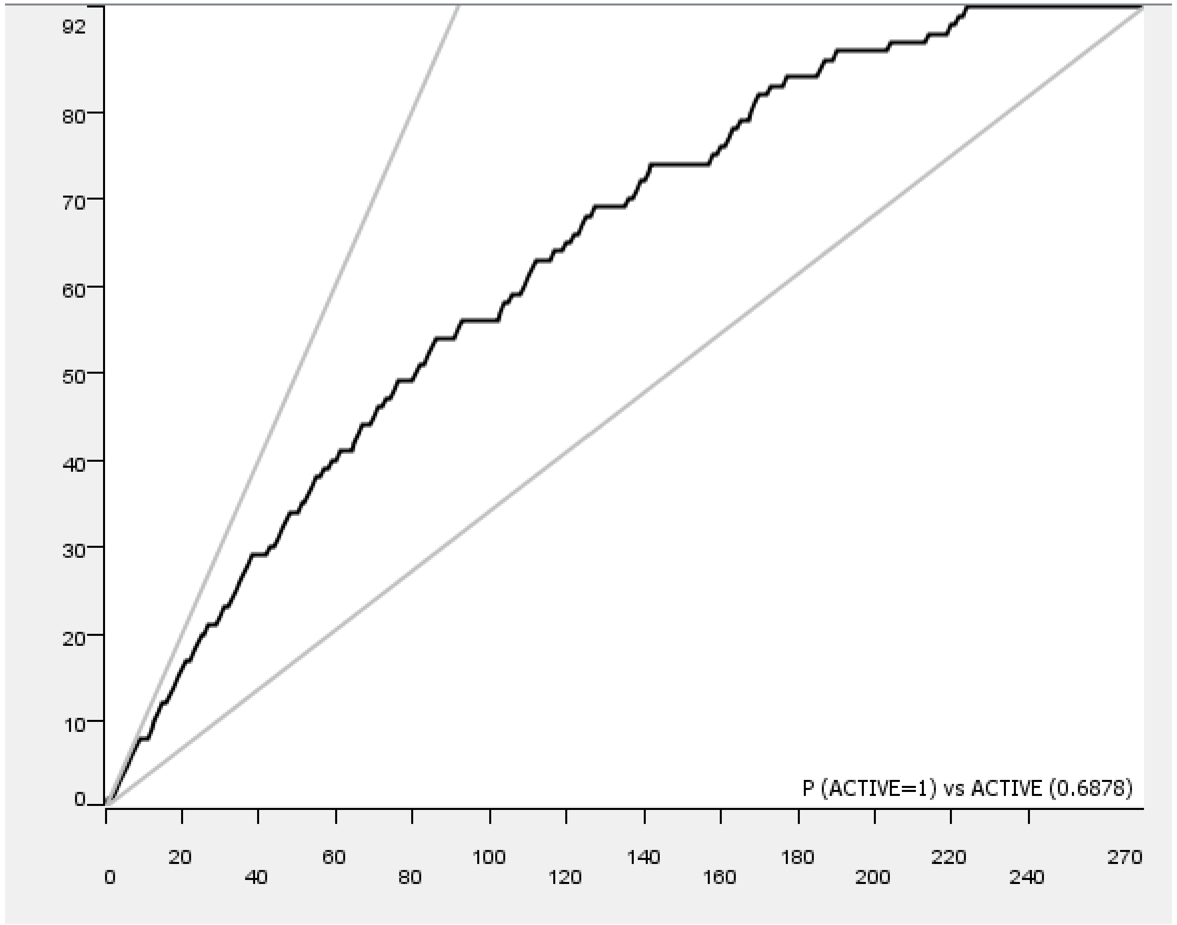

N=270

Top 10% Mean IC50	34.7 nM

	Predicted Active	Predicted Inactive
Active	49	43
Inactive	27	151

PNN - <15 nM DefGood in AKL (unparameterized)

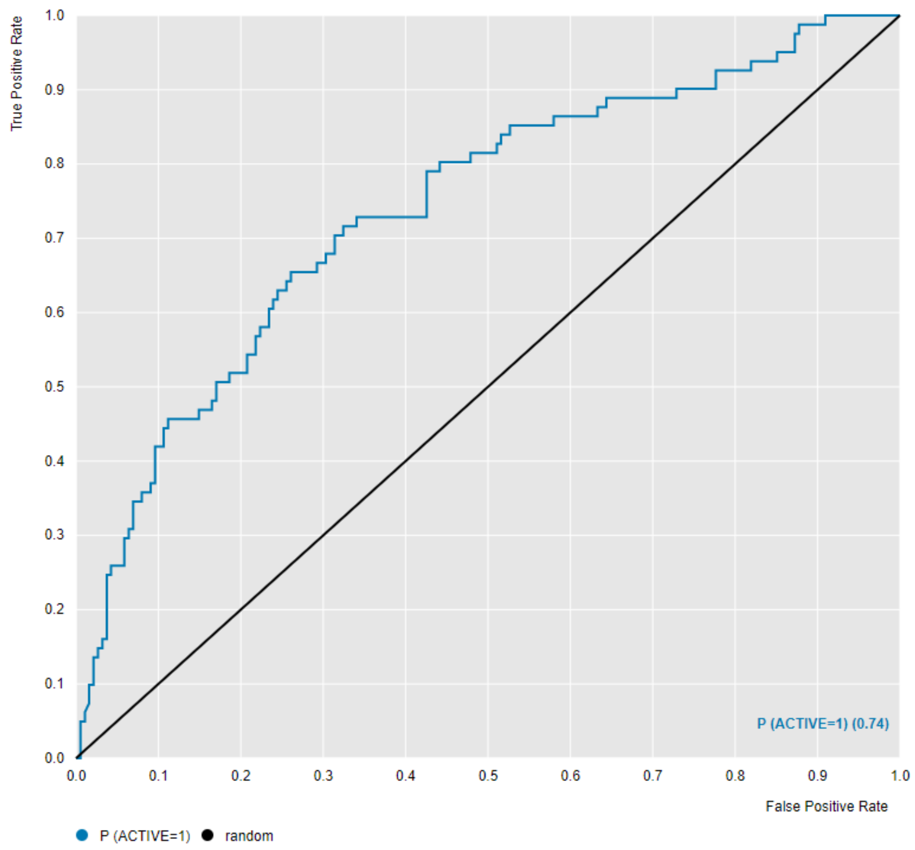

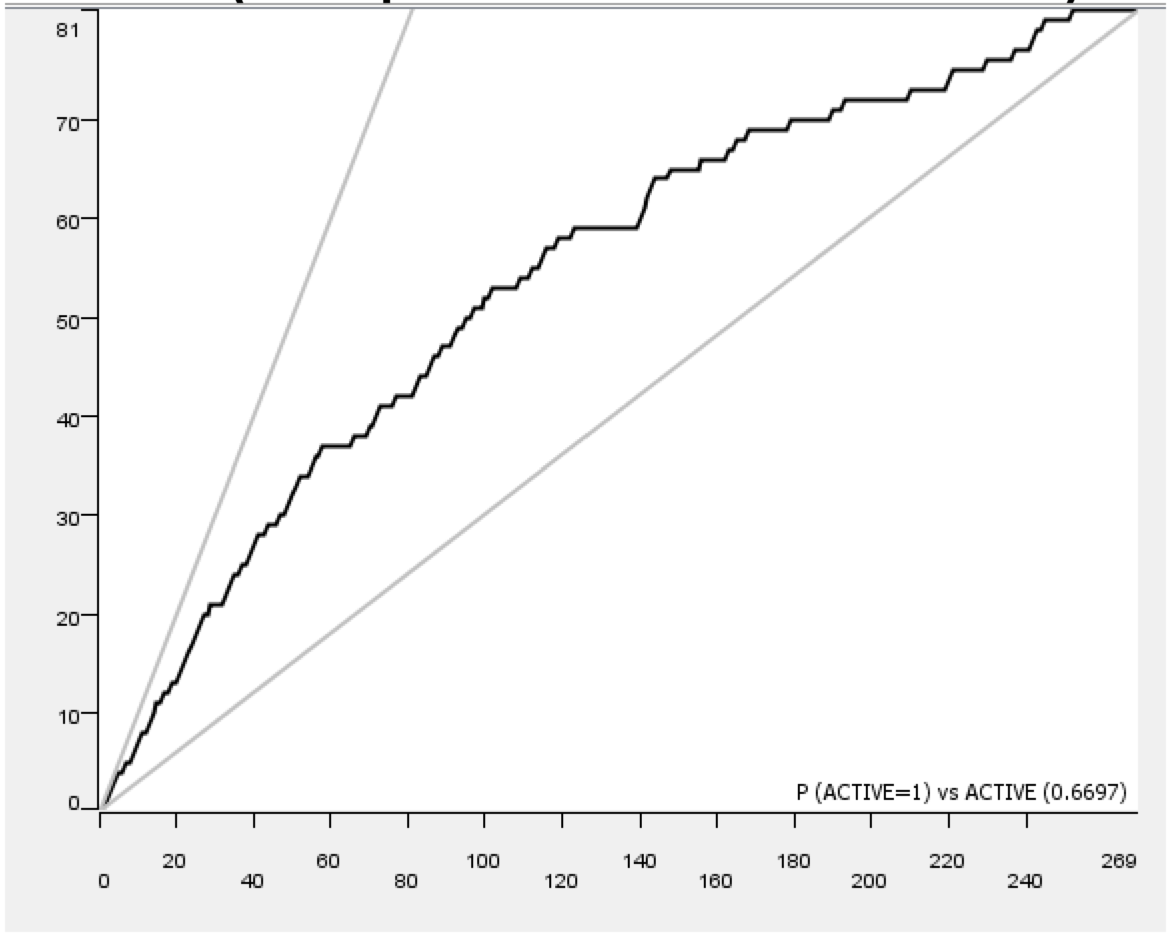

N=269

**Top 10% Mean IC50**	**1,200 nM**

	Predicted Active	Predicted Inactive
Active	18	63
Inactive	7	181

PNN - <25 nM DefGood in AKL (unparameterized)

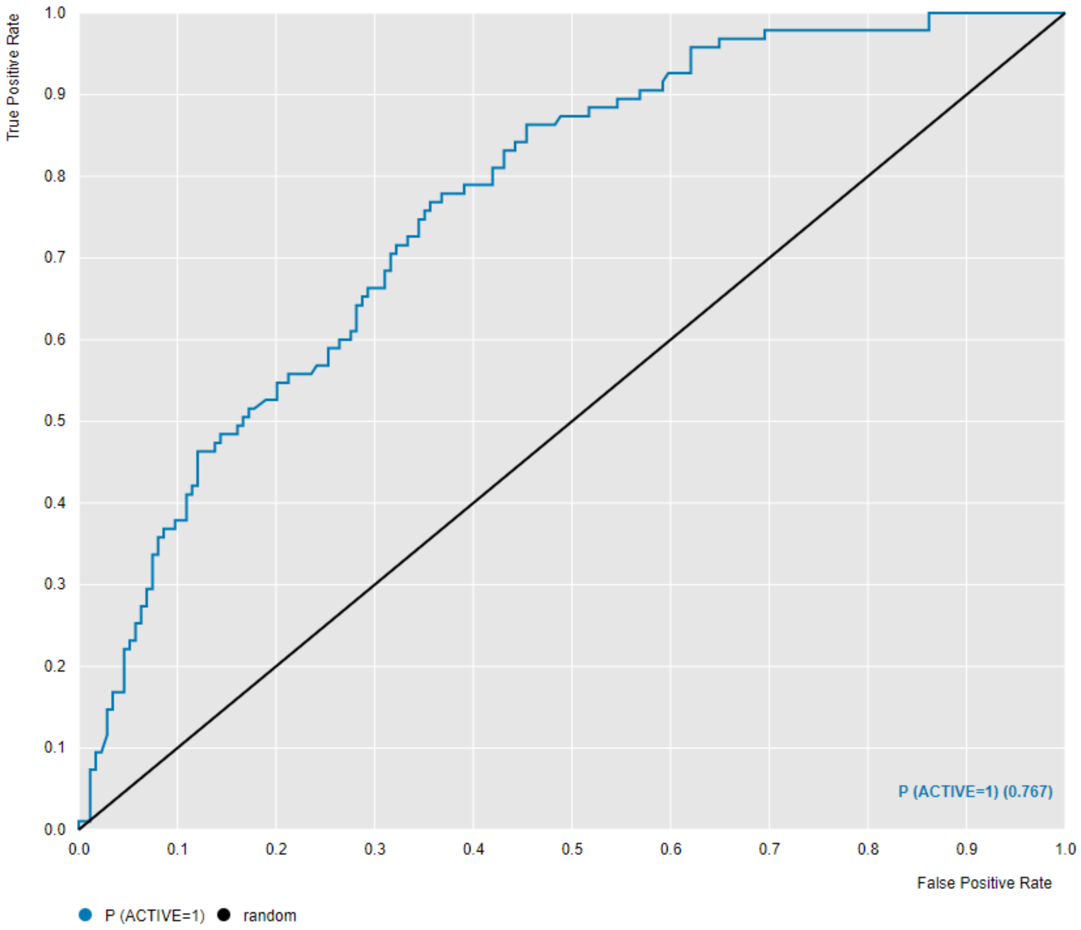

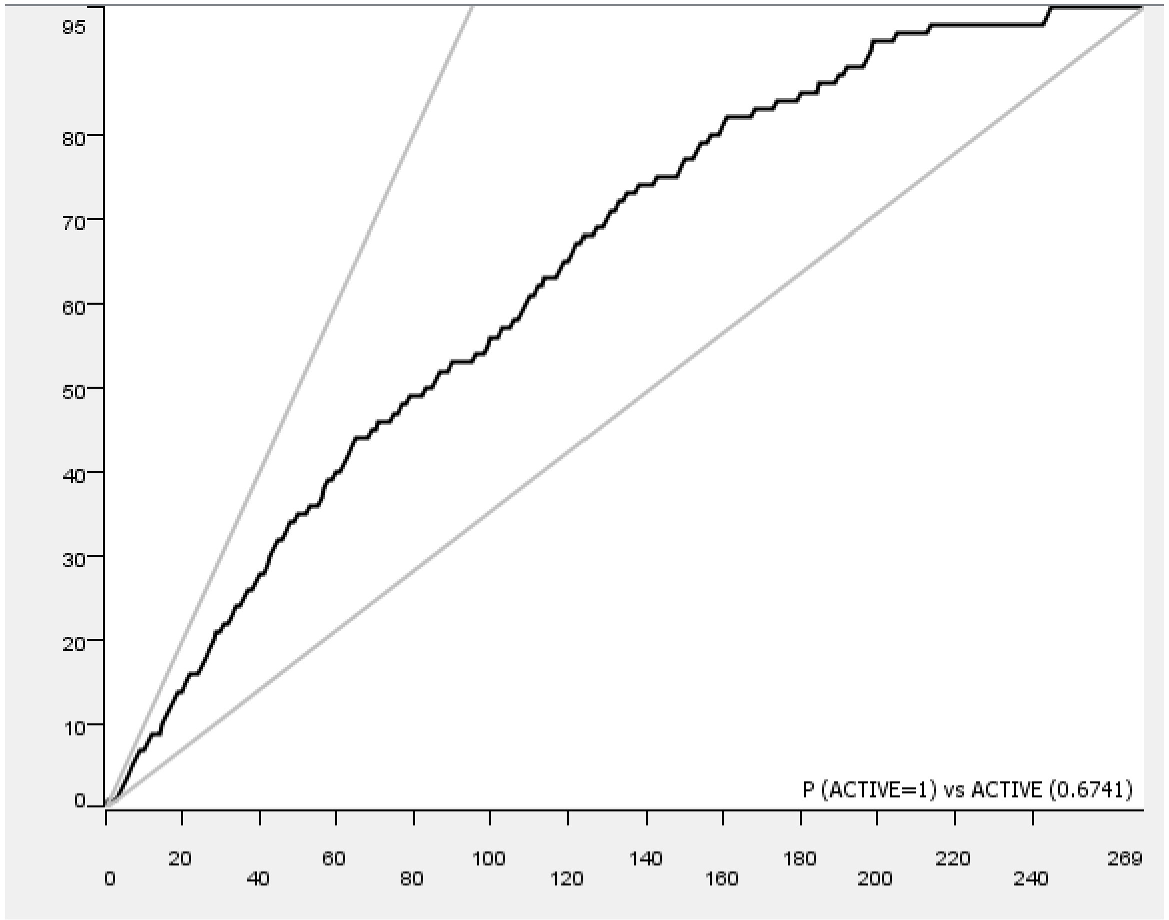

N=269

Top 10% Mean IC50	1,100 nM

	Predicted Active	Predicted Inactive
Active	47	48
Inactive	29	145

PNN - <15 nM DefGood in AKL (parameterized (theta minus = 0.15; theta plus = 0.55)

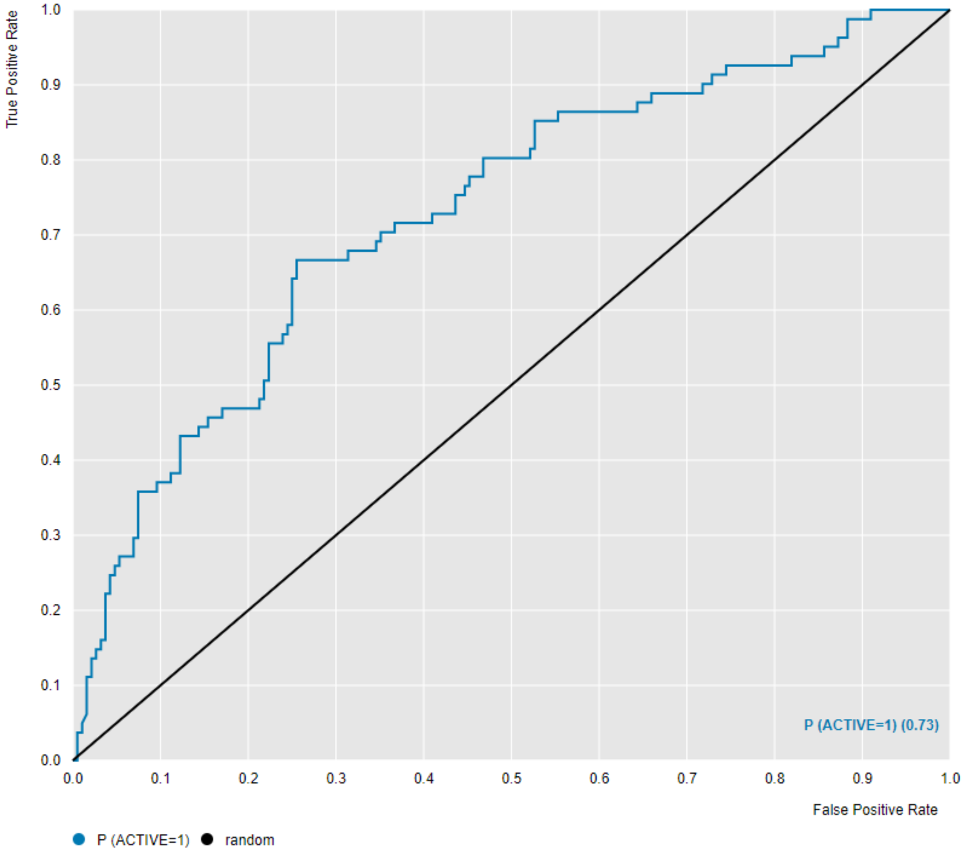

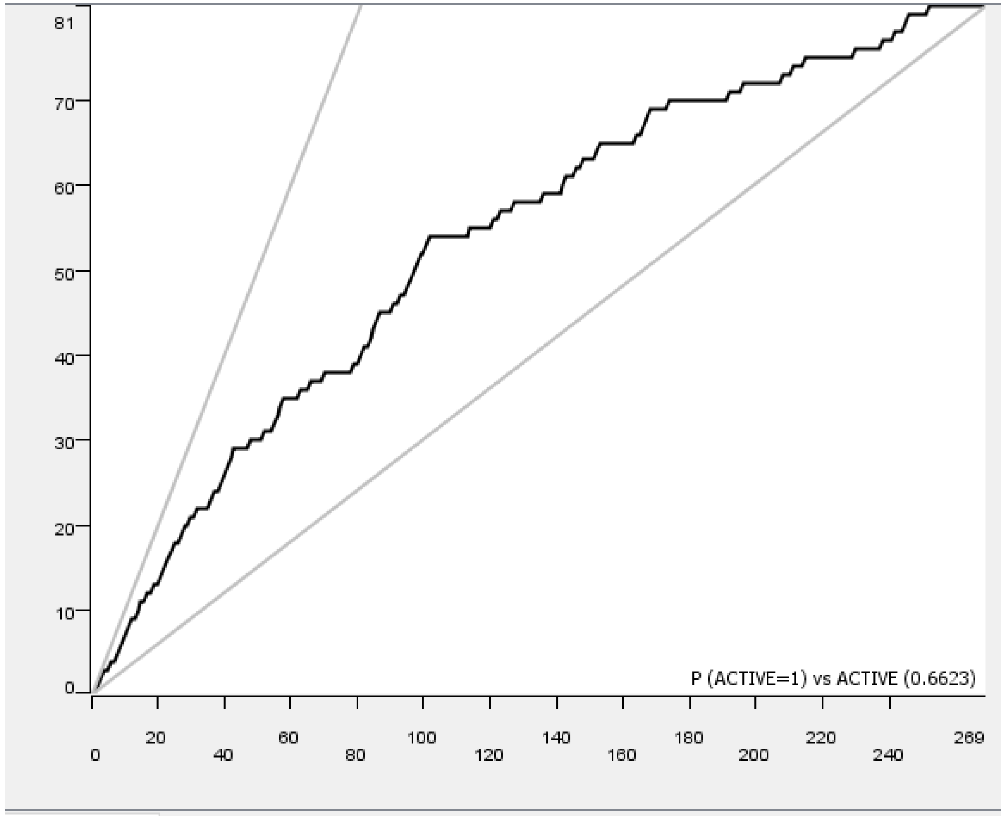

N=269

Top 10% Mean IC50	1,200 nM

	Predicted Active	Predicted Inactive
Active	24	57
Inactive	14	174

PNN - <25 nM DefGood in AKL (parameterized (theta minus = 0.45; theta plus = 0.45)

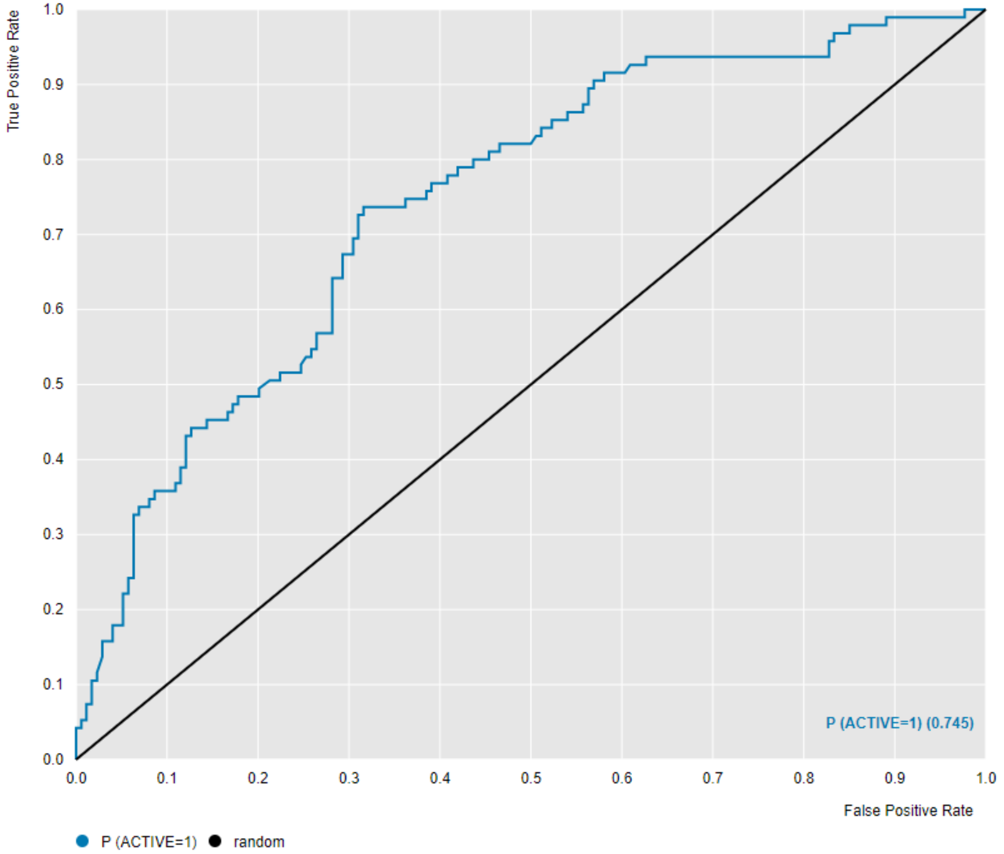

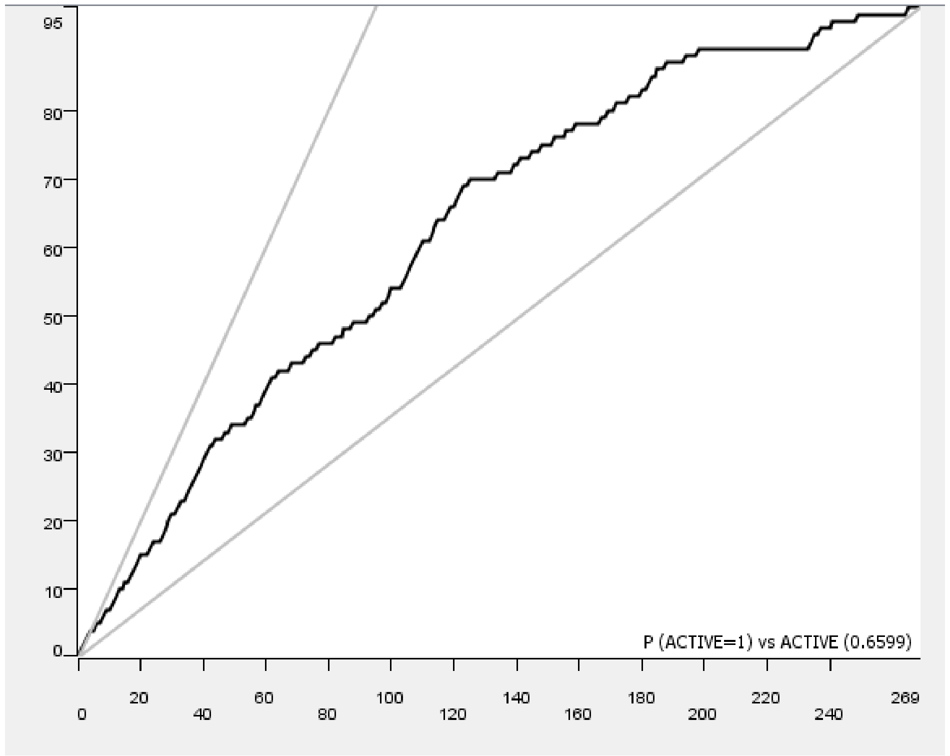

N=269

Top 10% Mean IC50	340 nM

	Predicted Active	Predicted Inactive
Active	42	53
Inactive	23	151

Decision on DefGood - PNN

- <20 nM has the best performance overall. This mirrored the NBN DefGood, and the DefGood for the NBNs in the future will be used for the RFs as well. Parameterization for each model adds significant time to the process. A rough benchmark can be applied where point of failure is discovered and then the model is parameterized to ensure that the situation is optimal for the PNN.

NBN Error Tolerance- <20 nM DefGood in AKL

- 0-50% absolute error

<20 nM DefGood in AKL, 5% error; Random seed = 1515533876005

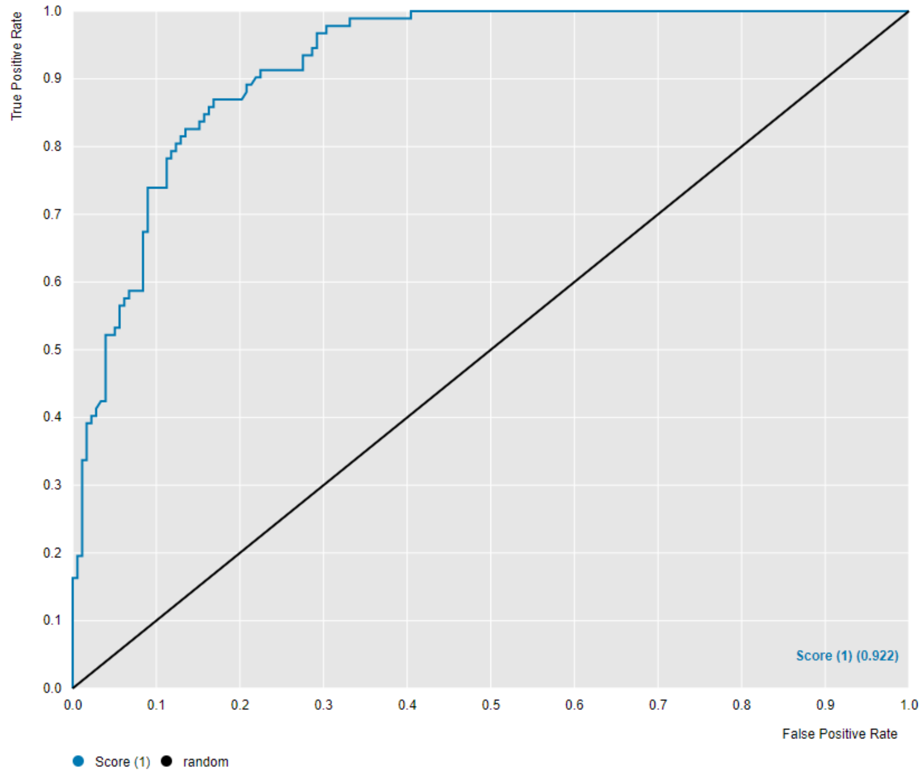

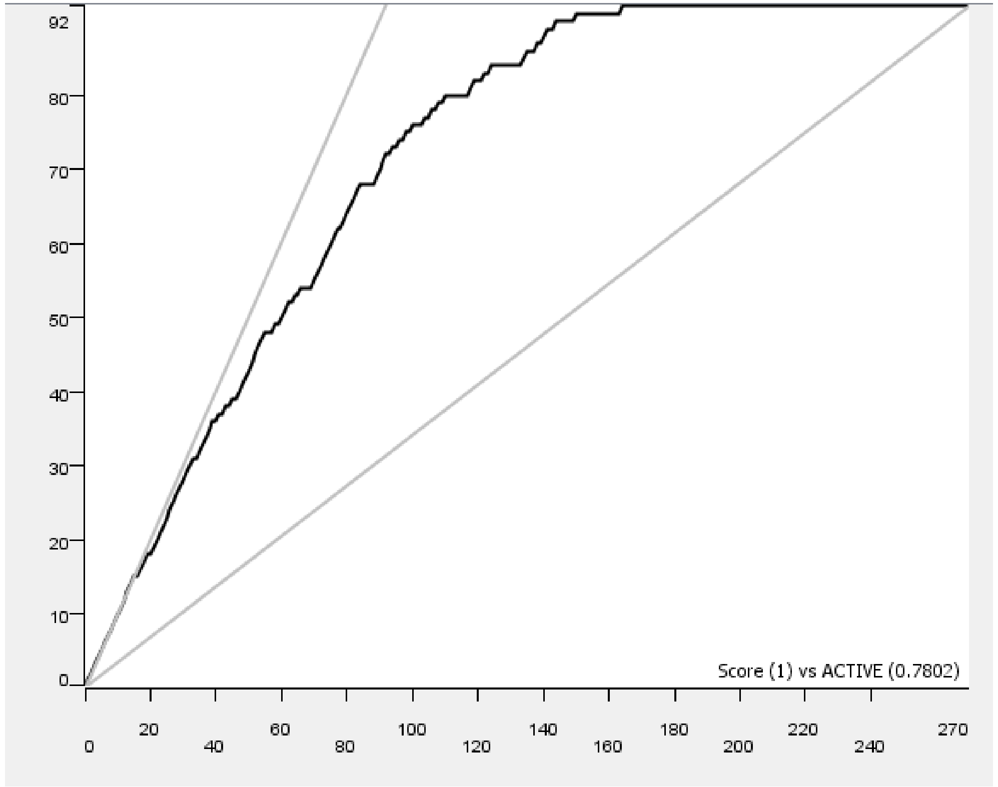

N=270

**Top 10% Mean IC50**	**6.9**

	Predicted Active	Predicted Inactive
Active	80	12
Inactive	35	143

<20 nM DefGood in ALK, 10% error

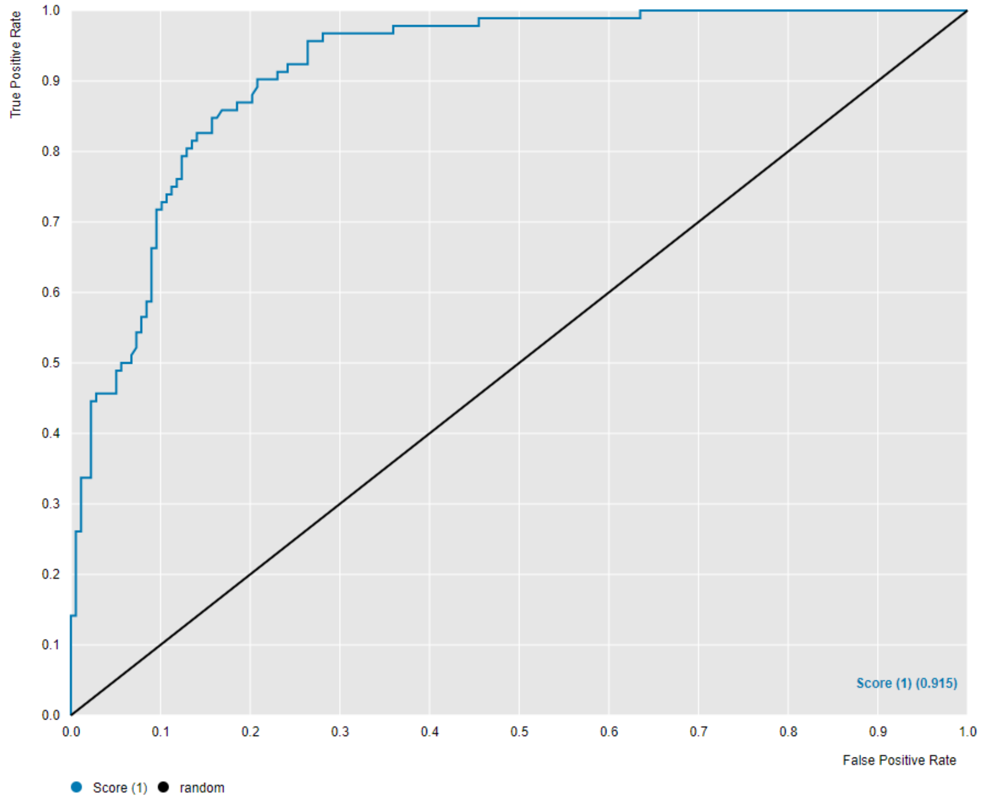

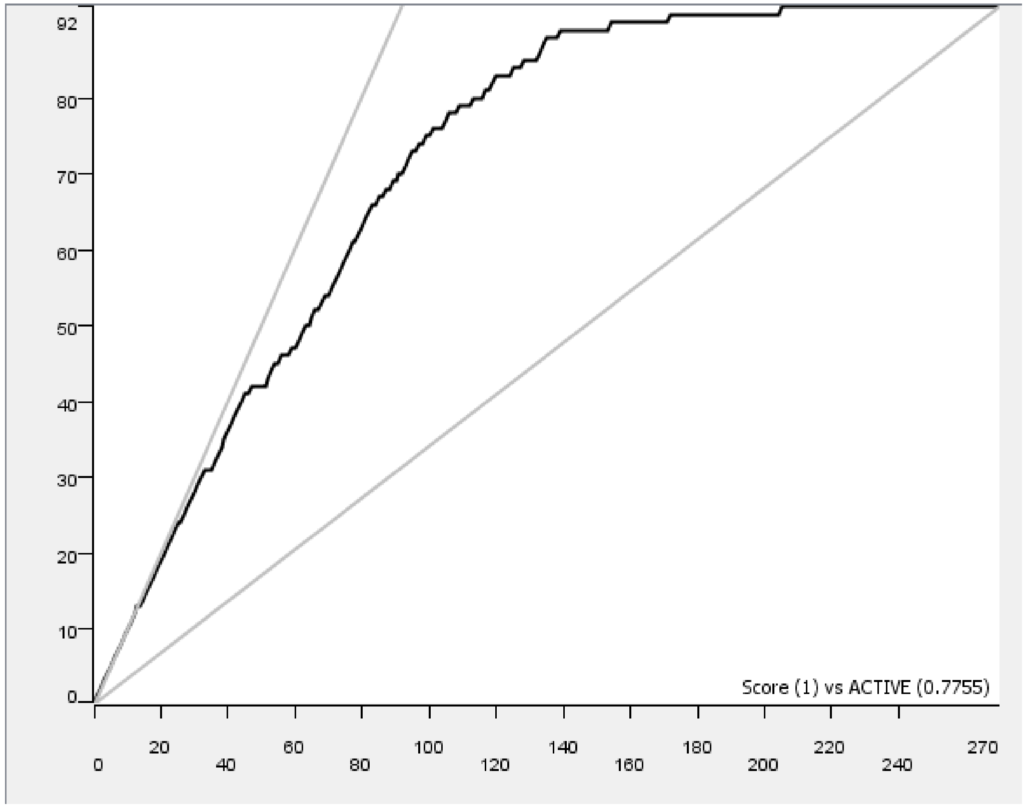

N=270

**Top 10% Mean IC50**	**7.3 nM**

	Predicted Active	Predicted Inactive
Active	83	9
Inactive	37	141

<20 nM DefGood in ALK, 15% error

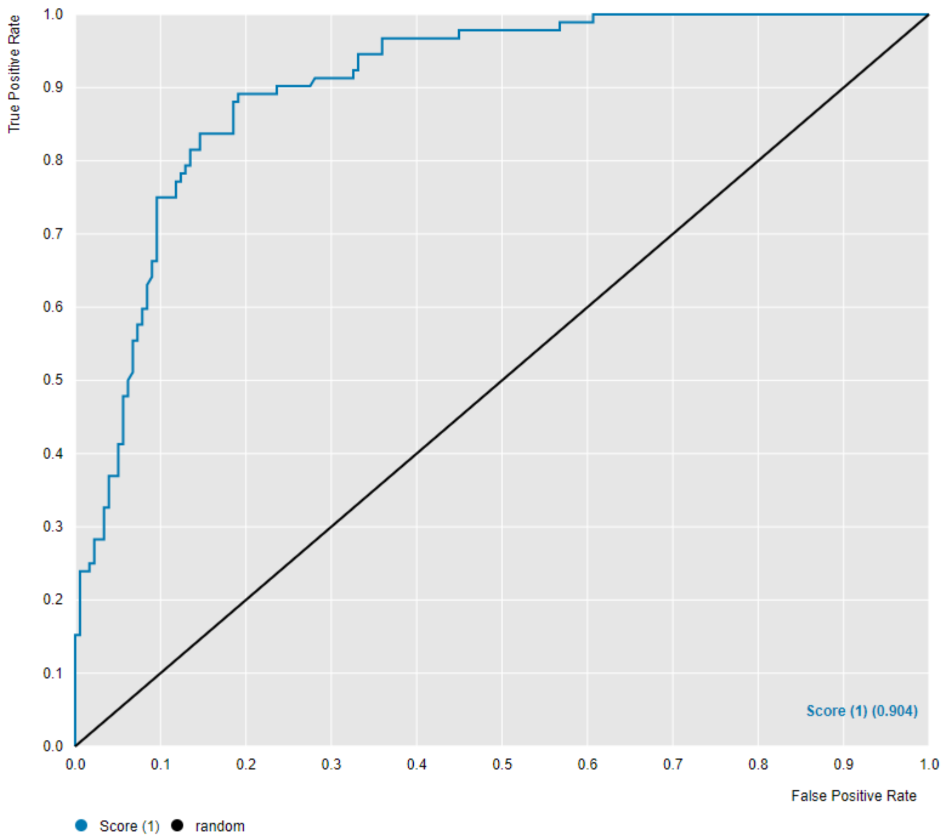

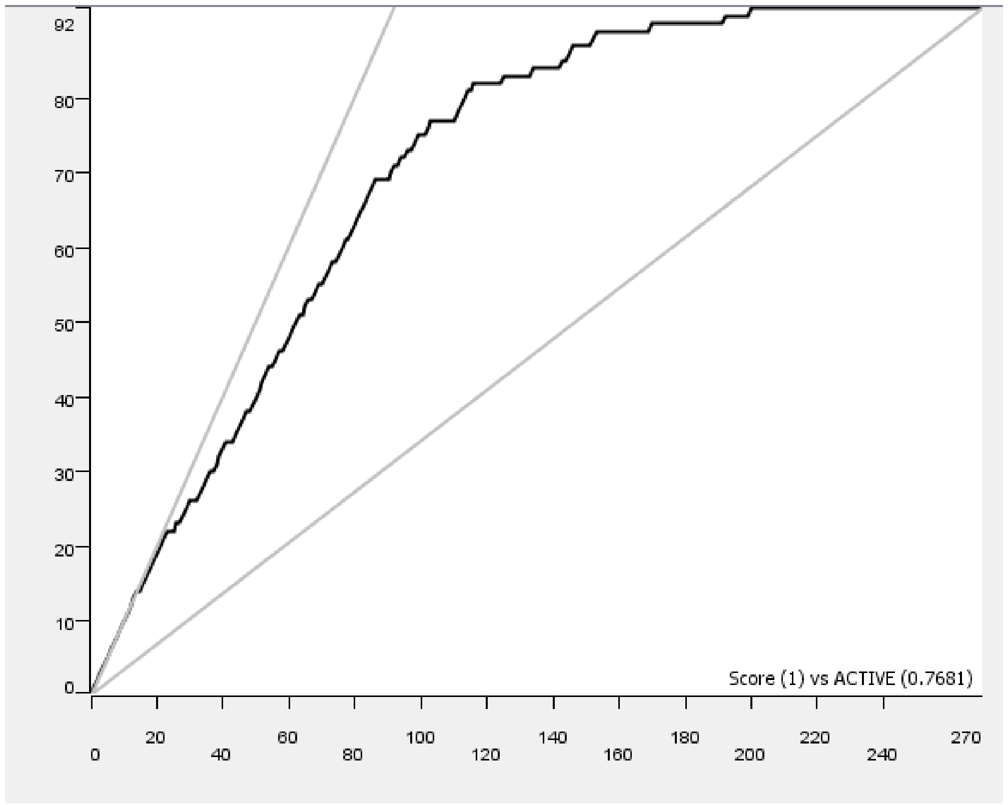

N=270

Top 10% Mean IC50	20.1 nM

	Predicted Active	Predicted Inactive
Active	82	10
Inactive	40	138

<20 nM DefGood in ALK, 20% error

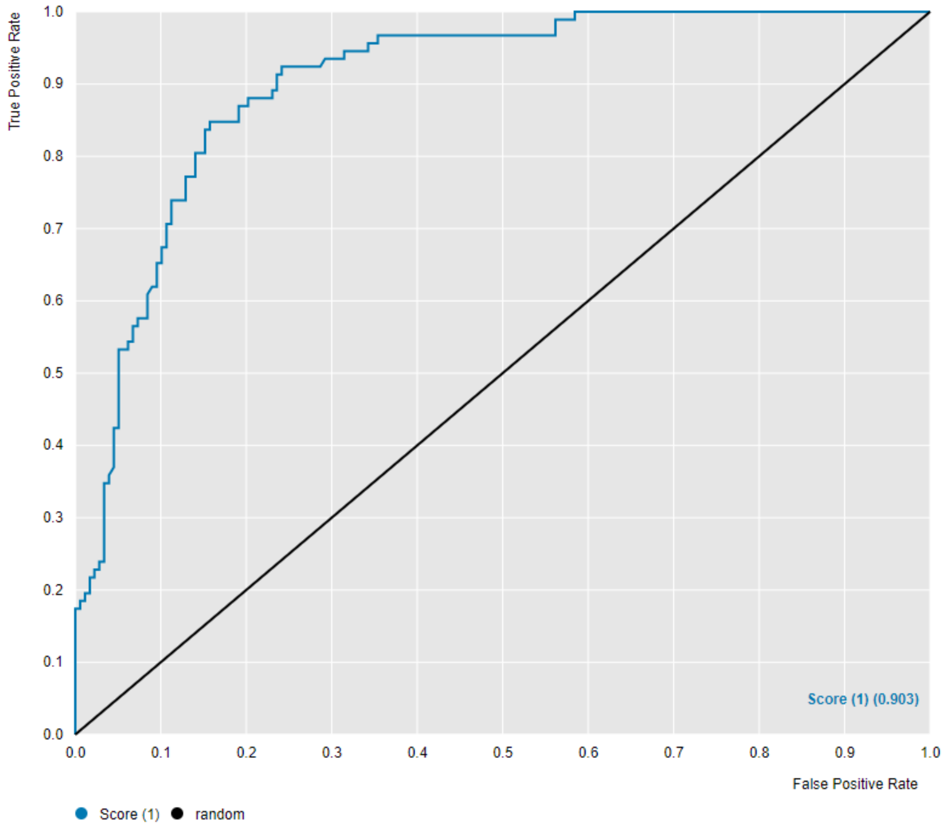

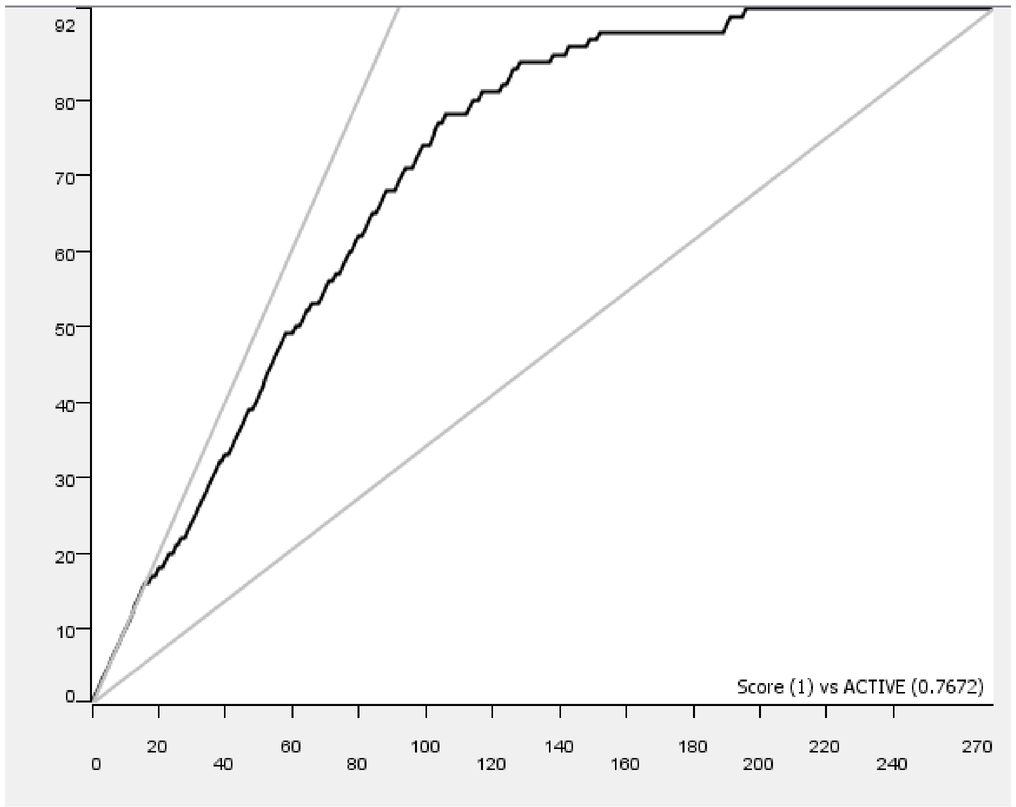

N=270

Top 10% Mean IC50	13.0 nM

	Predicted Active	Predicted Inactive
Active	82	10
Inactive	42	136

<20 nM DefGood in ALK, 30% error

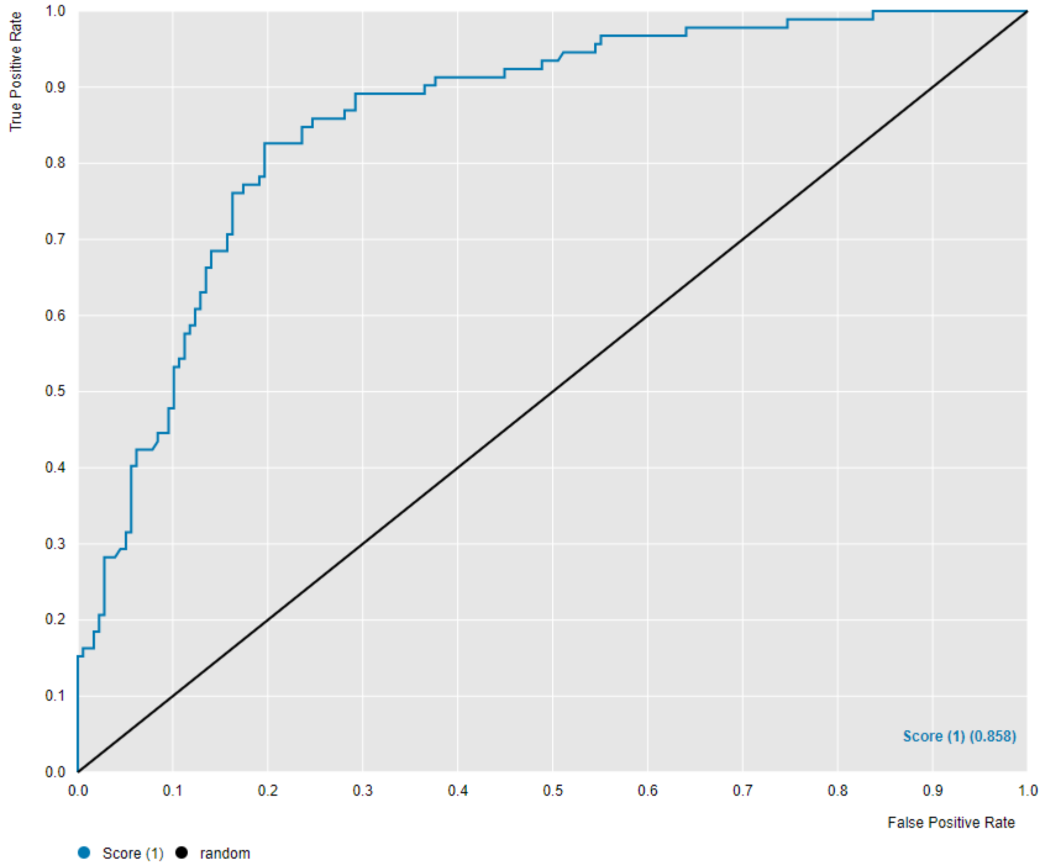

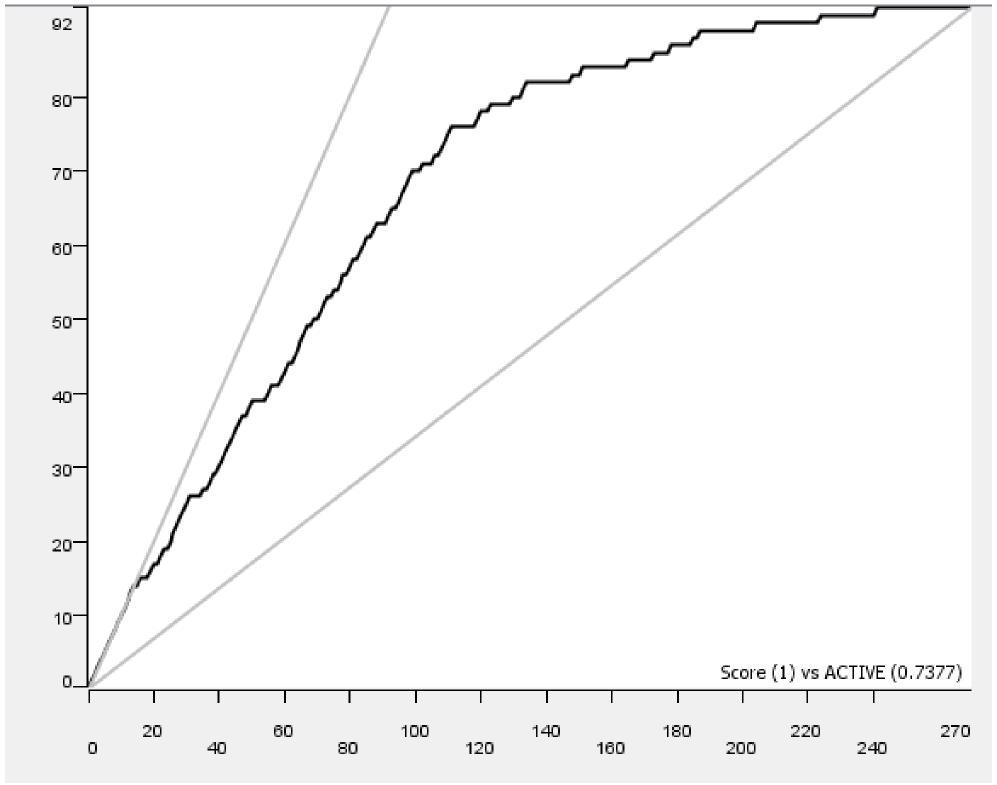

N=270

Top 10% Mean IC50	13.8 nM

	Predicted Active	Predicted Inactive
Active	78	14
Inactive	43	135

<20 nM DefGood in ALK, 40% error

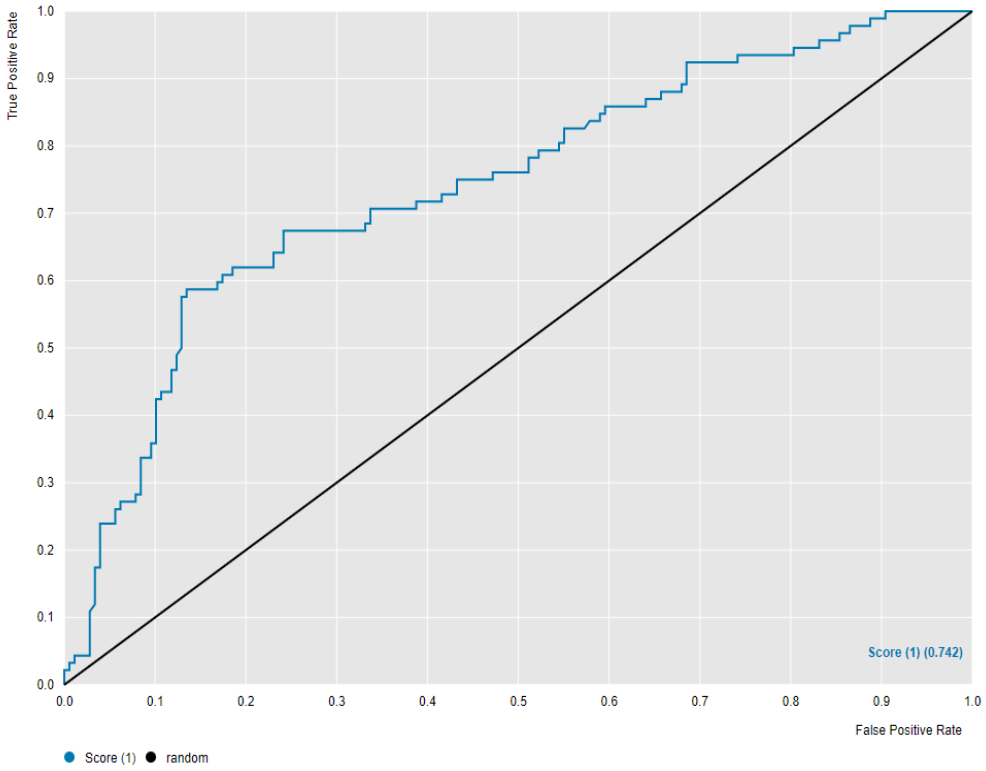

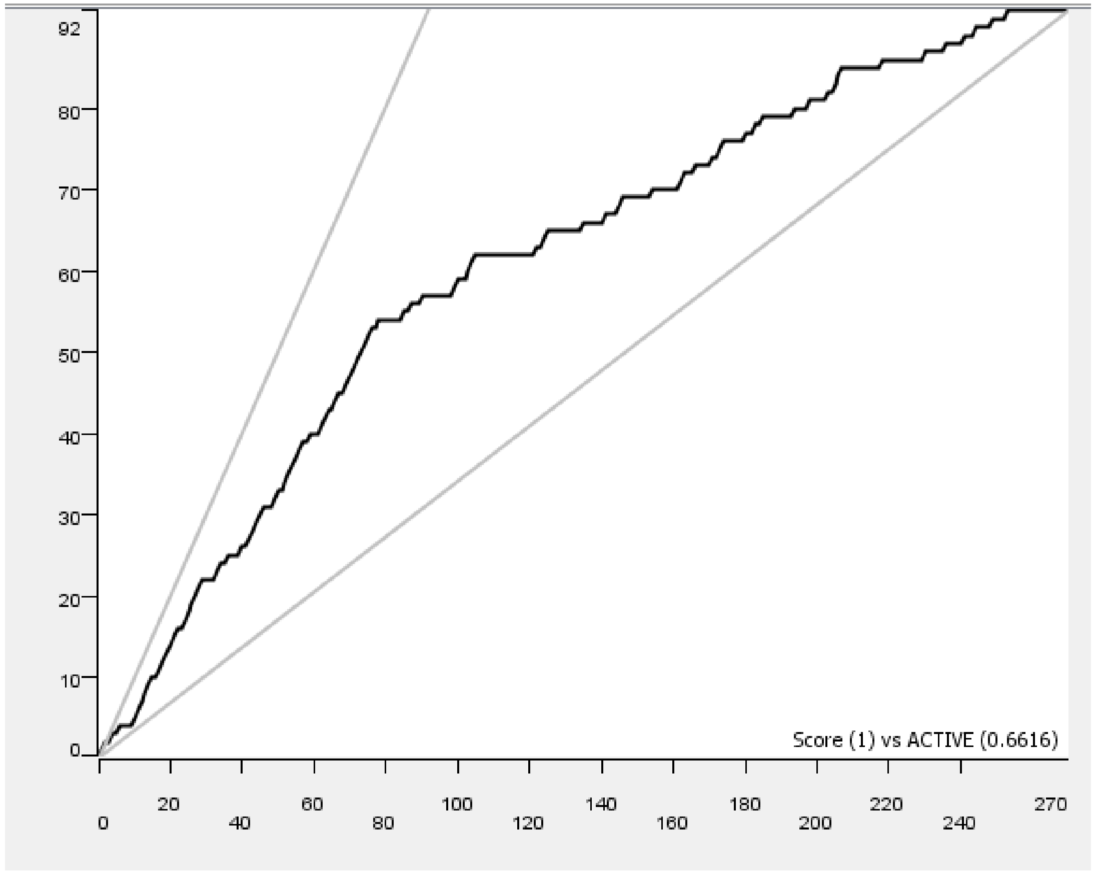

N=270

**Top 10% Mean IC50**	**53.5 Nm**

	**Predicted Active**	**Predicted Inactive**
Active	82	30
Inactive	55	123

<20 nM DefGood in ALK, 45% error

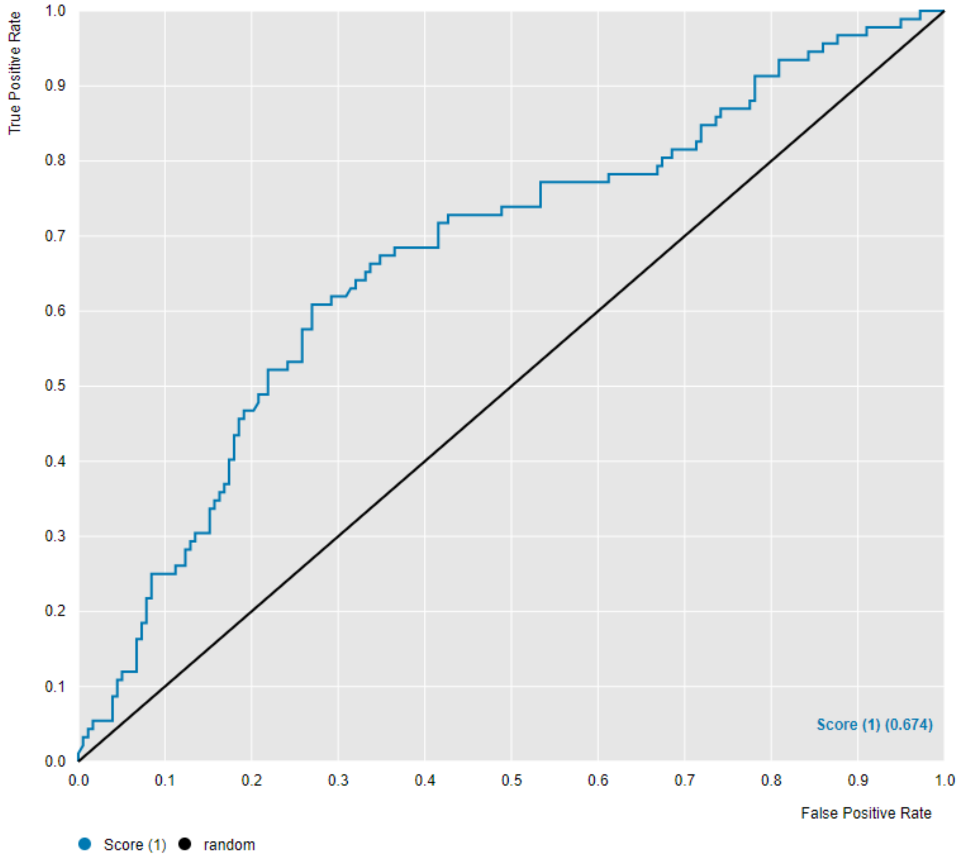

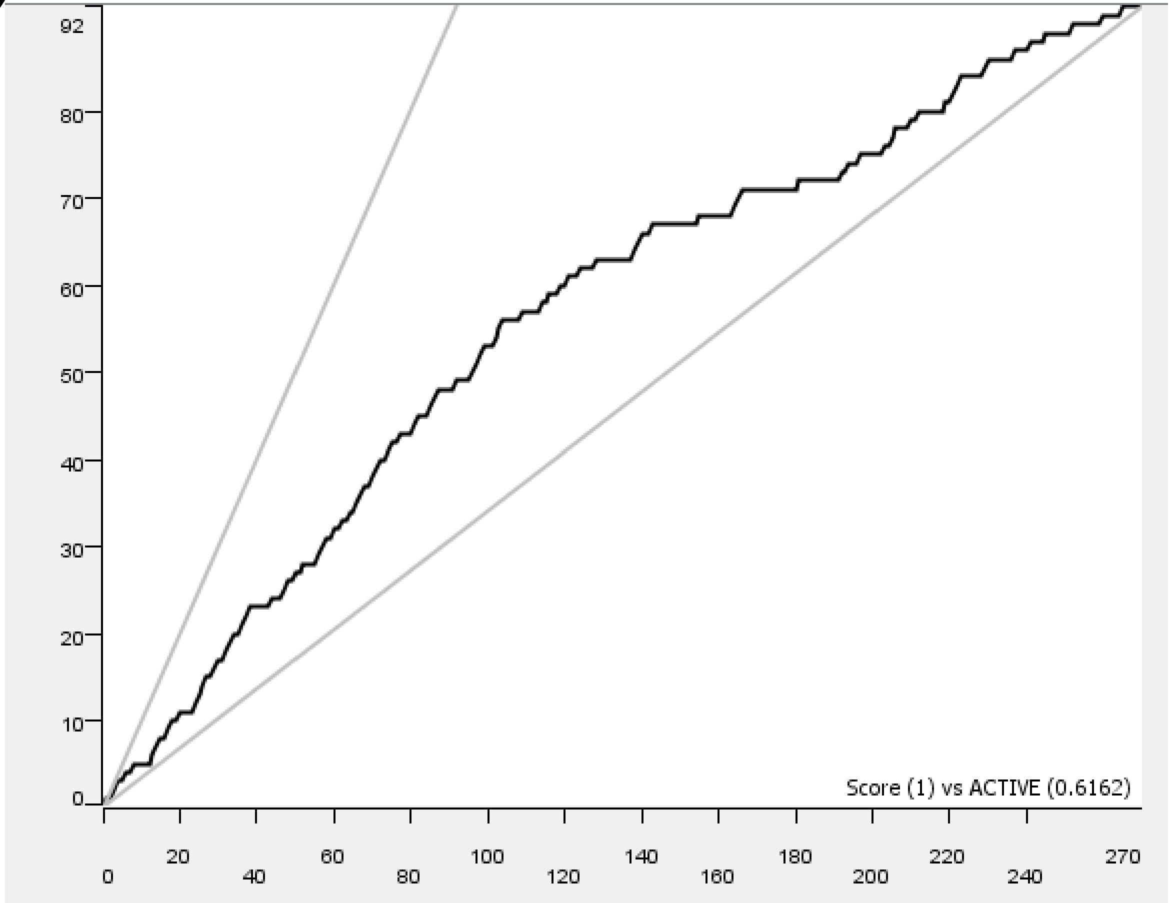

N=270

Top 10% Mean IC50	1,400 Nm

	Predicted Active	Predicted Inactive
Active	71	21
Inactive	96	82

<20 nM DefGood in ALK, 10% error; Random seed = 429

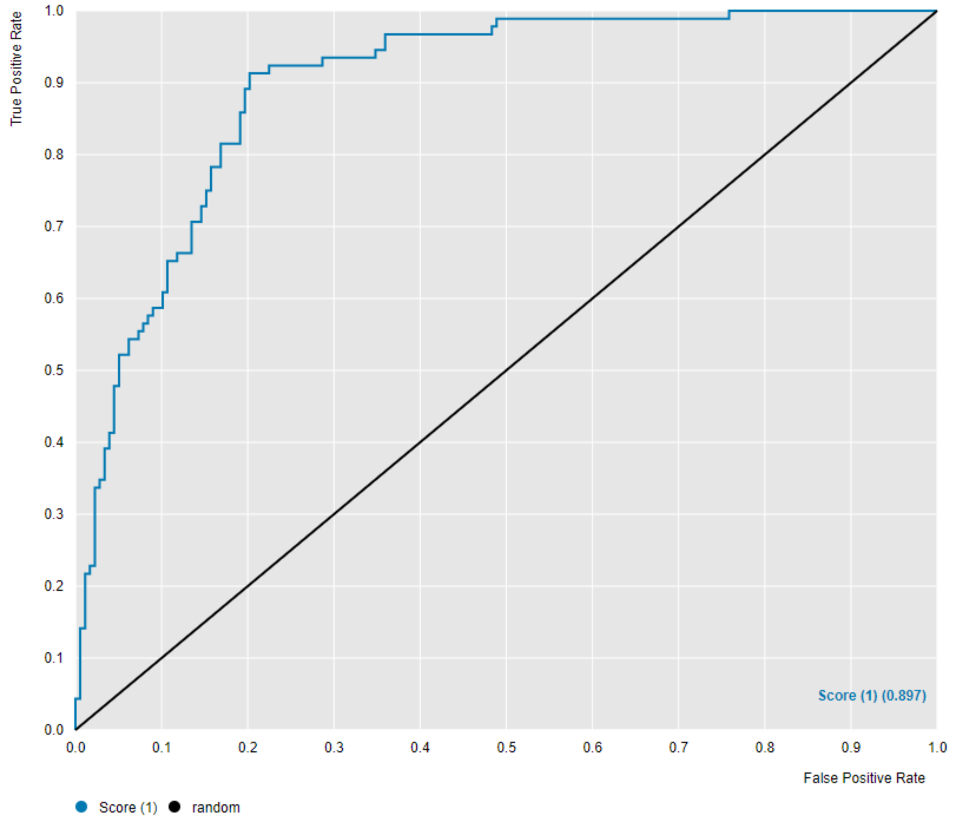

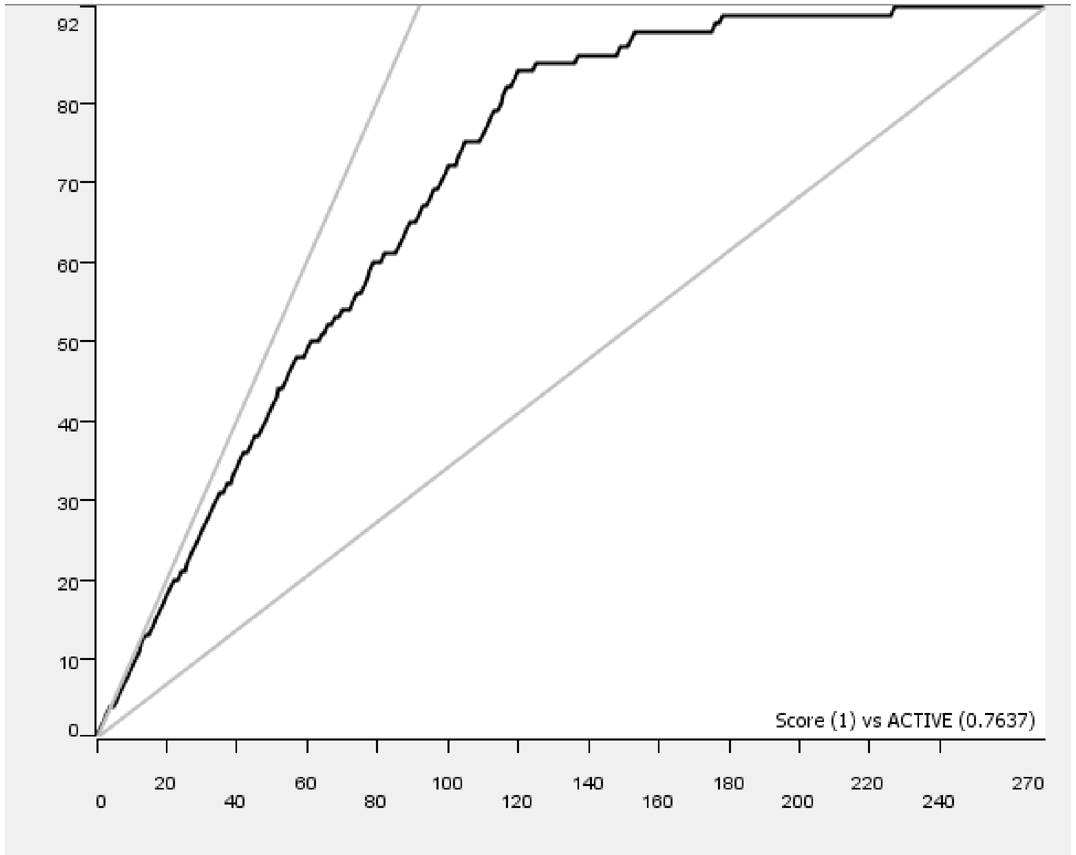

N=270

Top 10% Mean IC50	22.3 nM

	Predicted Active	Predicted Inactive
Active	83	9
Inactive	36	142

<20 nM DefGood in ALK, 20% error; Random seed = 429

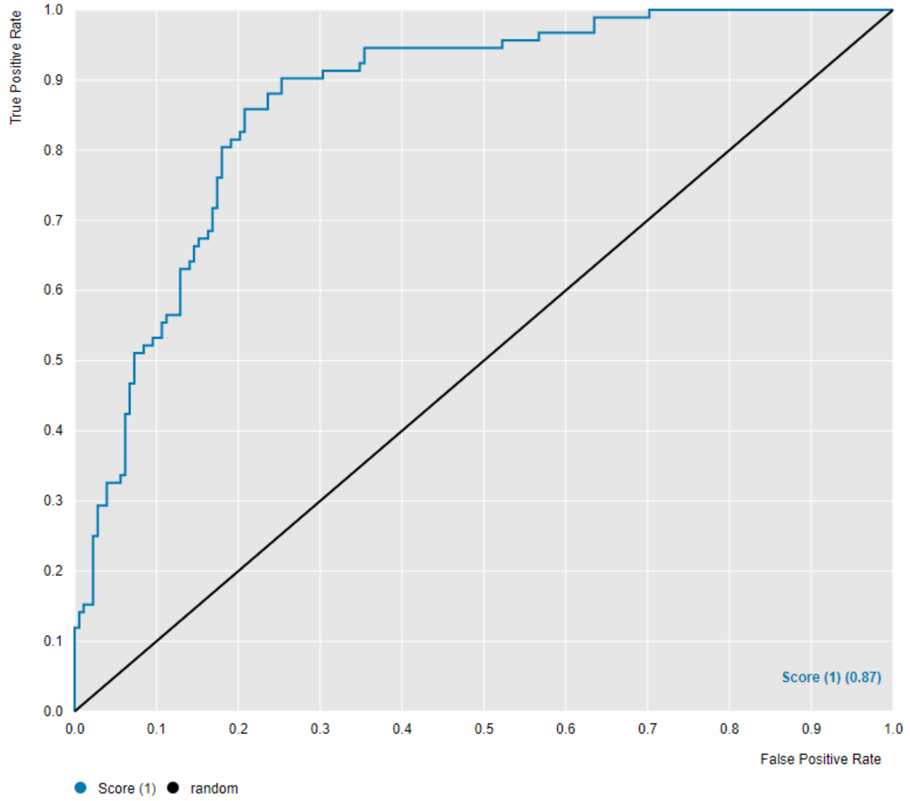

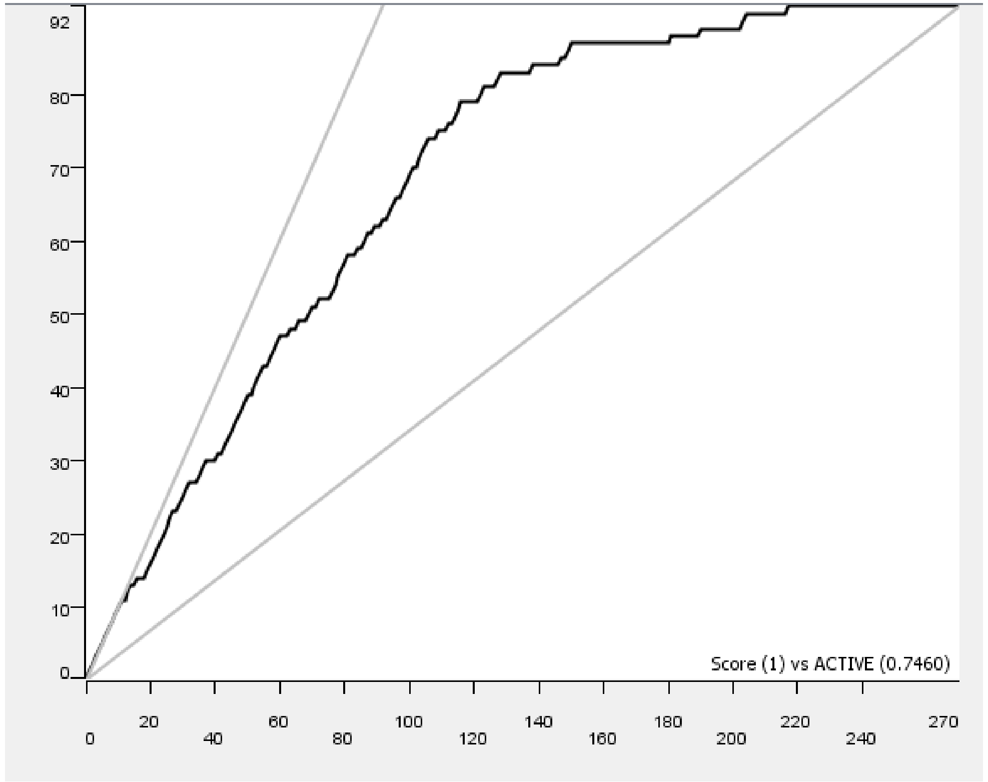

N=270

**Top 10% Mean IC50**	**22.9 nM**

	Predicted Active	Predicted Inactive
Active	81	11
Inactive	44	134

<20 nM DefGood in ALK, 30% error; Random seed = 429

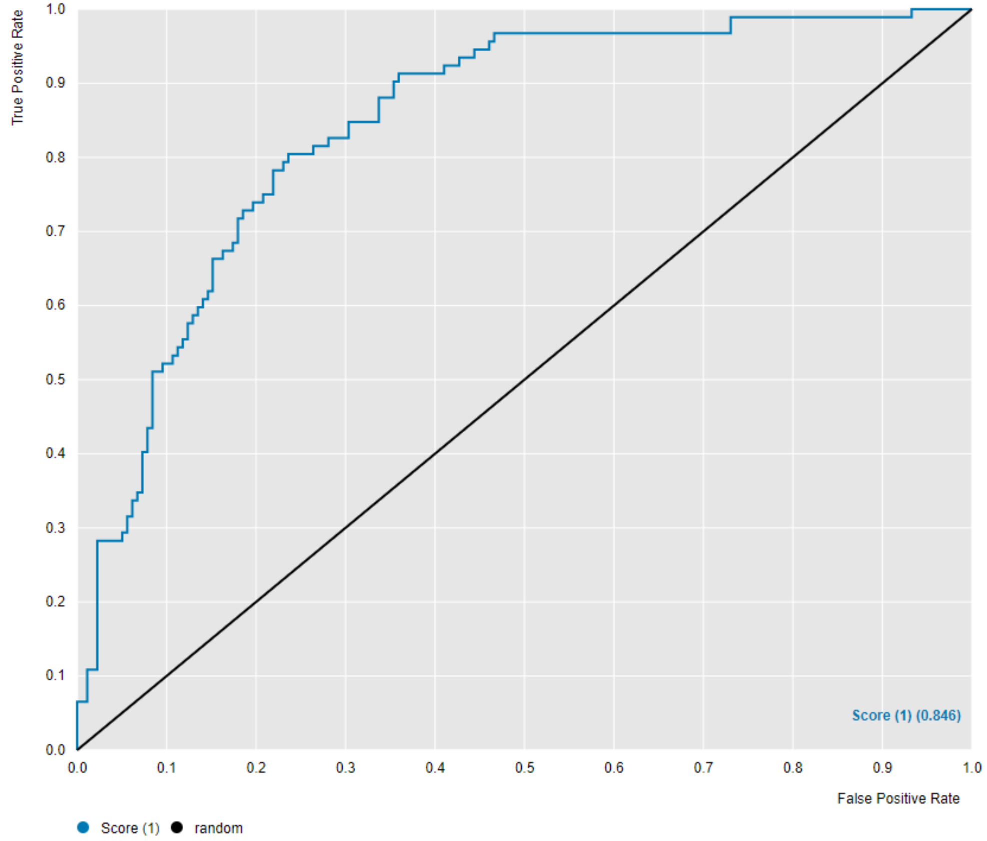

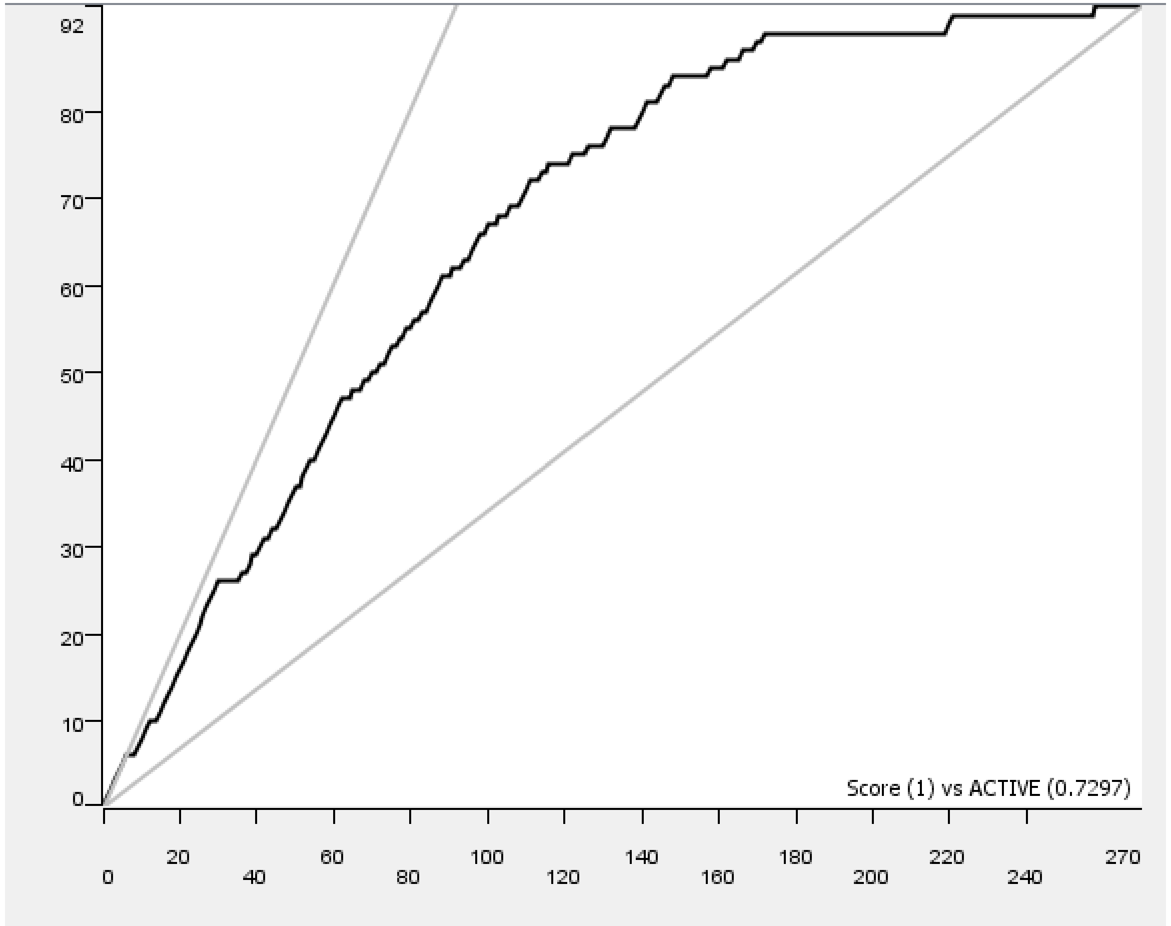

N=270

Top 10% Mean IC50	22.4 nM

	Predicted Active	Predicted Inactive
Active	69	23
Inactive	38	140

<20 nM DefGood in ALK, 40% error; Random seed = 429

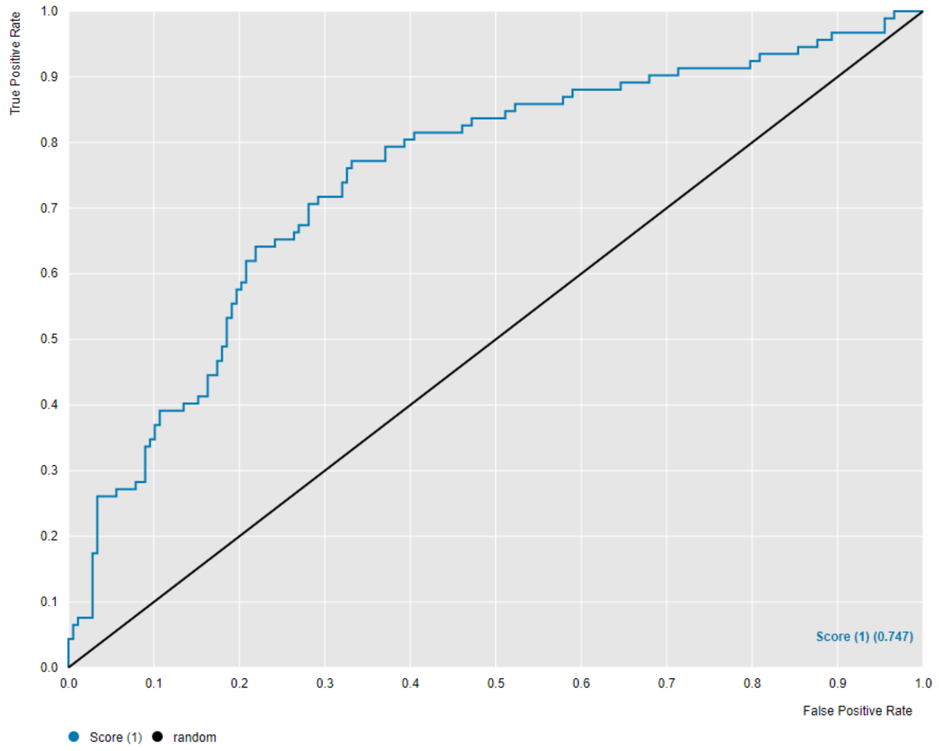

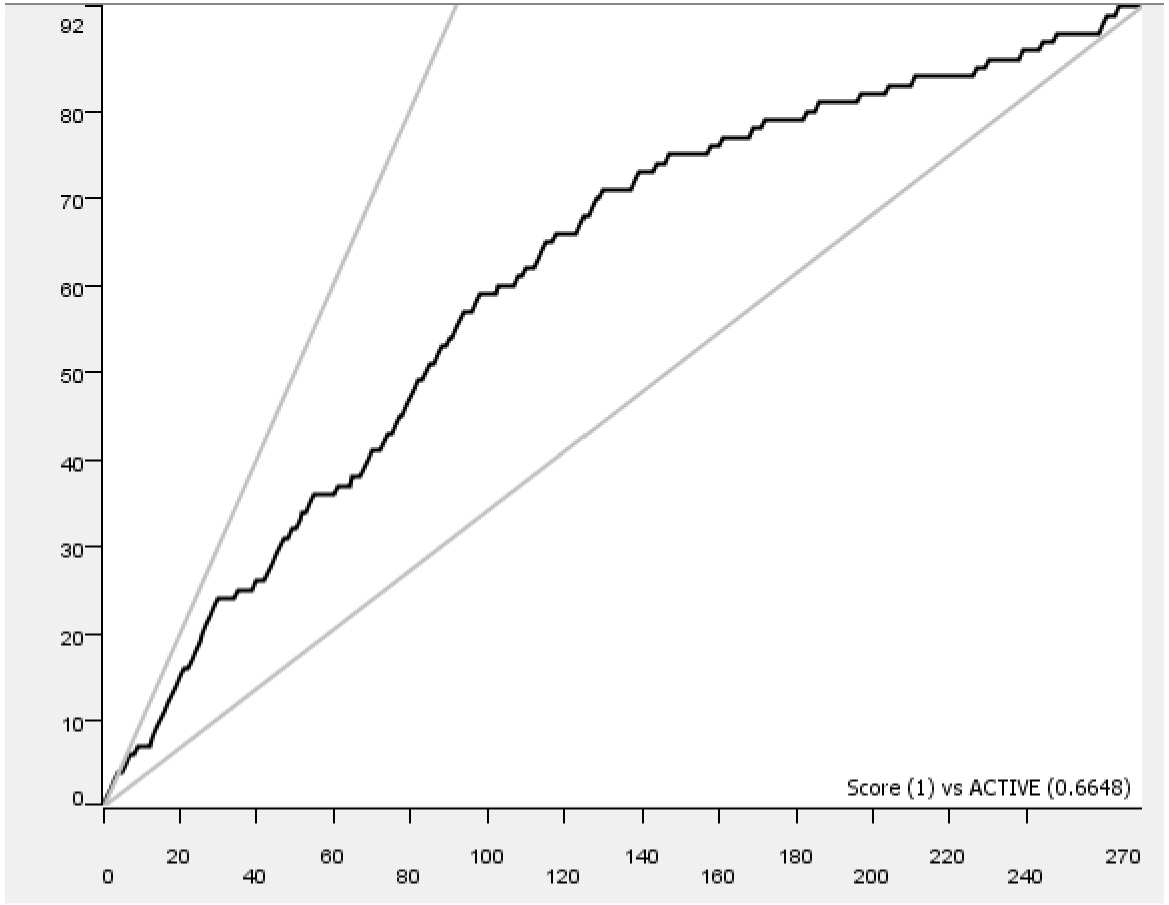

N=270

Top 10% Mean IC50	280 nM (one 6,000 nM mistake

	Predicted Active	Predicted Inactive
Active	32	60
Inactive	17	161

<20 nM DefGood in ALK, 45% error; Random seed = 429

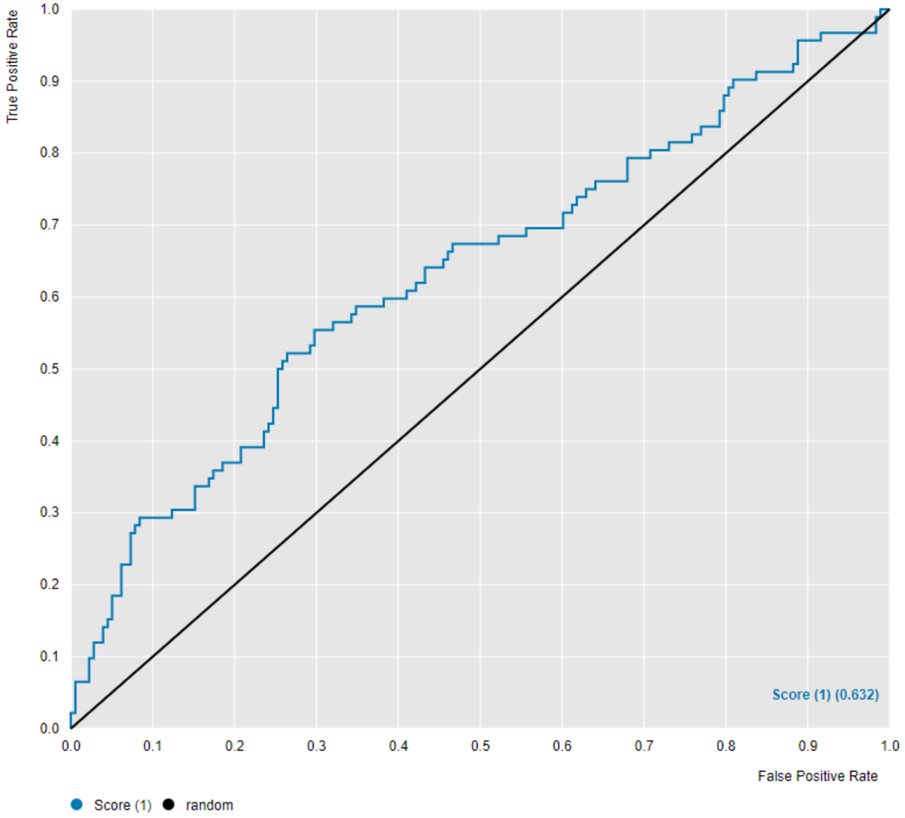

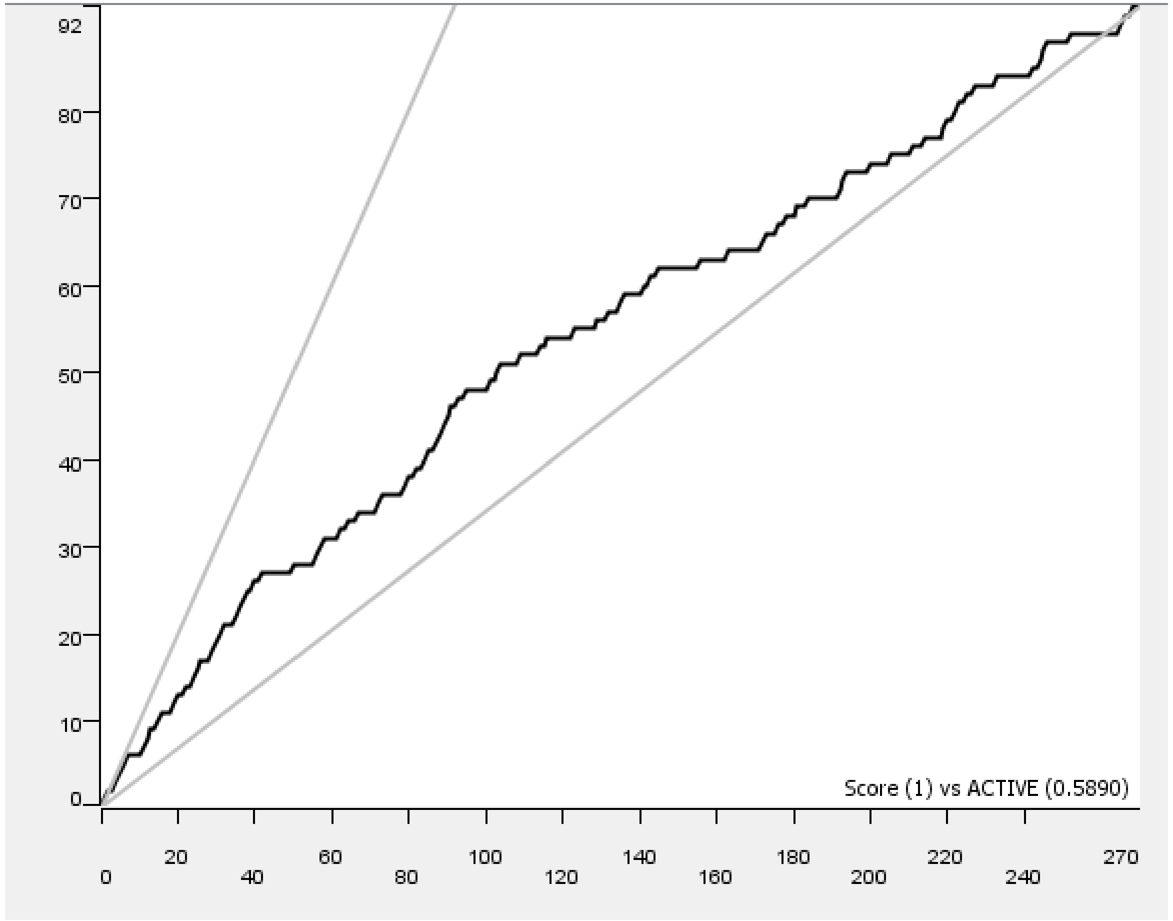

N=270

**Top 10% Mean IC50**	**900 nM Several severe mistakes**

	Predicted Active	Predicted Inactive
Active	17	75
Inactive	10	168

<20 nM DefGood in ALK, 10% error; Random seed = 121783

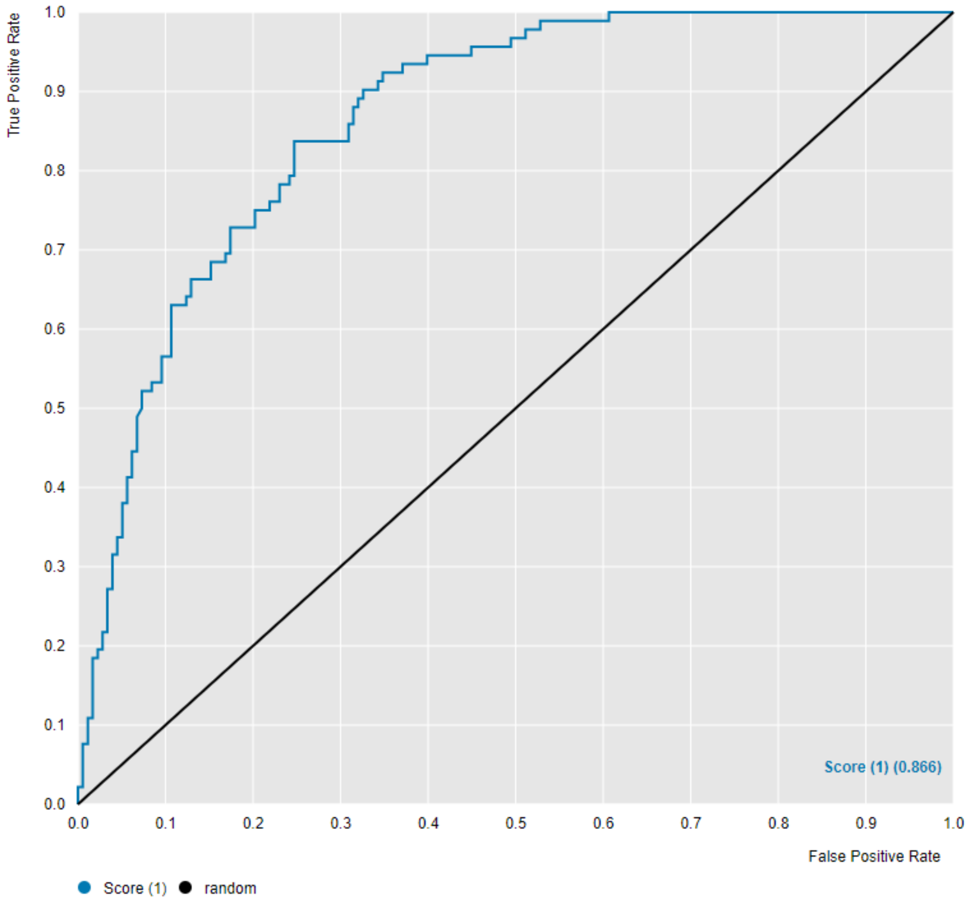

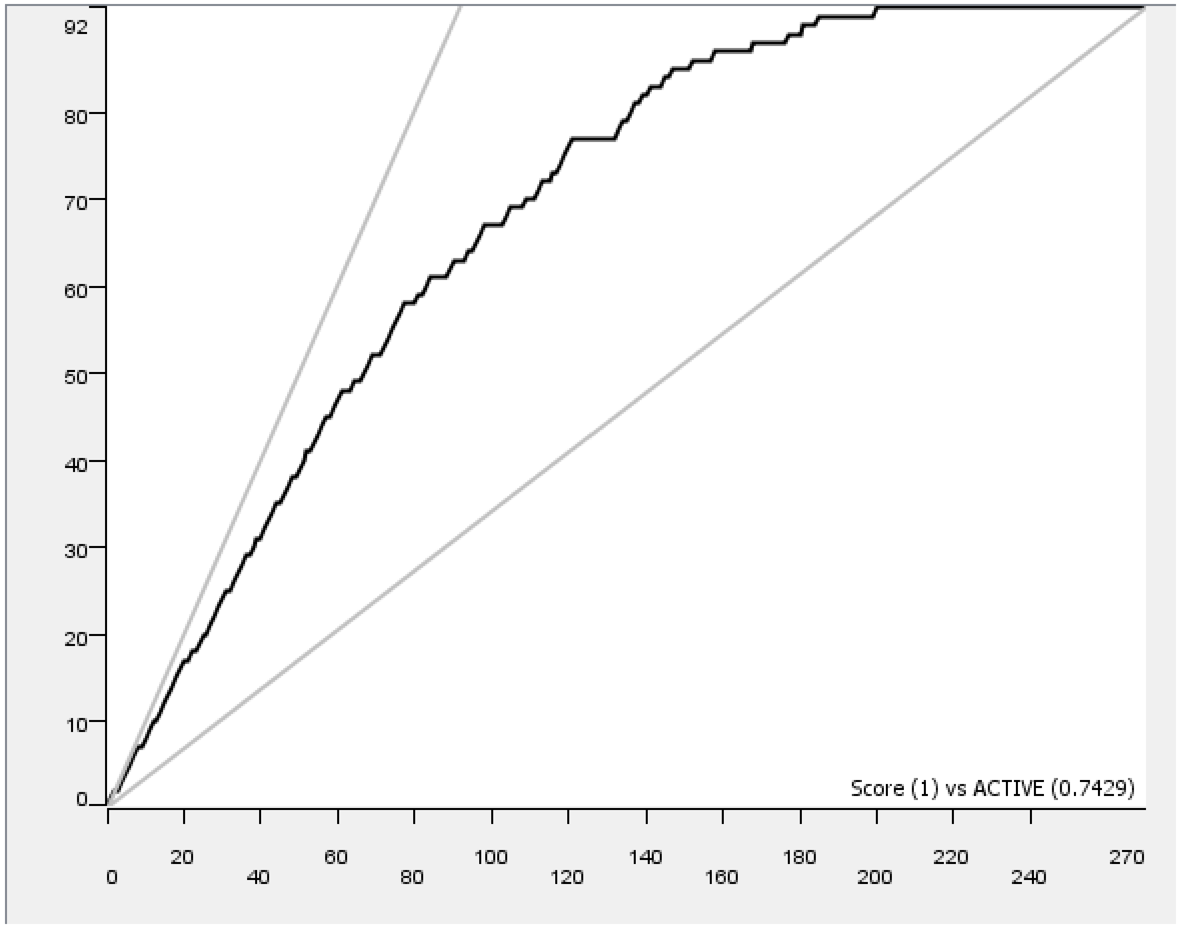

N=270

**Top 10% Mean IC50**	**21 nM**

	Predicted Active	Predicted Inactive
Active	81	11
Inactive	56	122

<20 nM DefGood in ALK, 20% error; Random seed = 121783

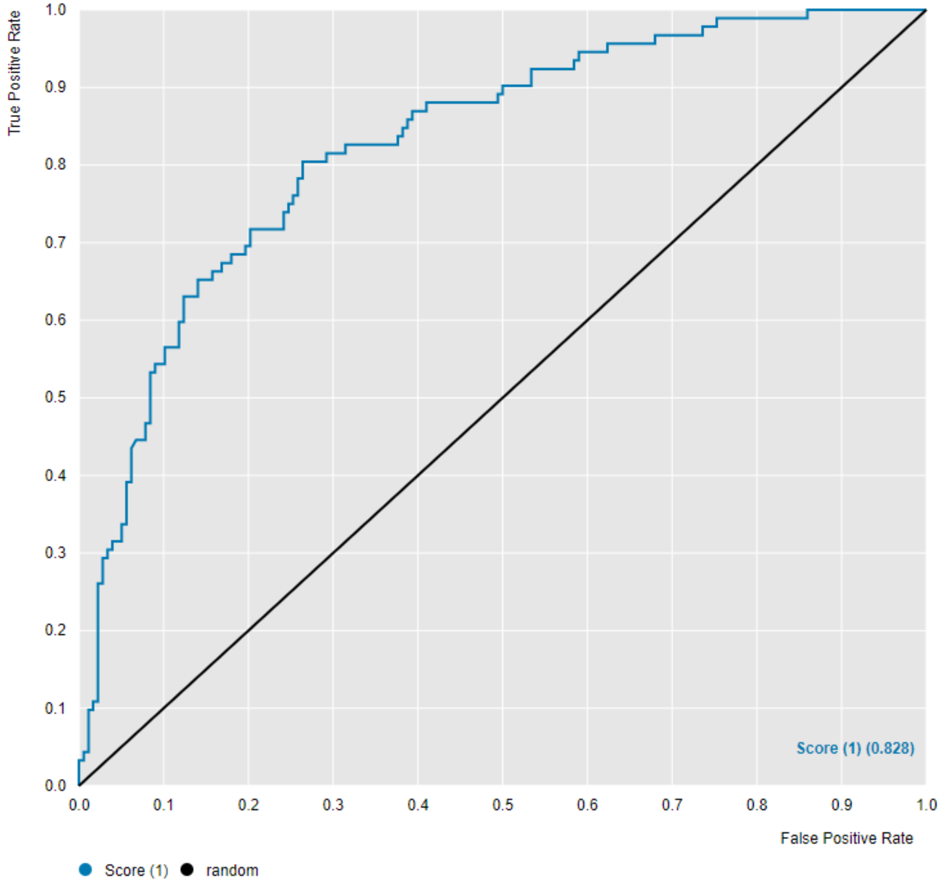

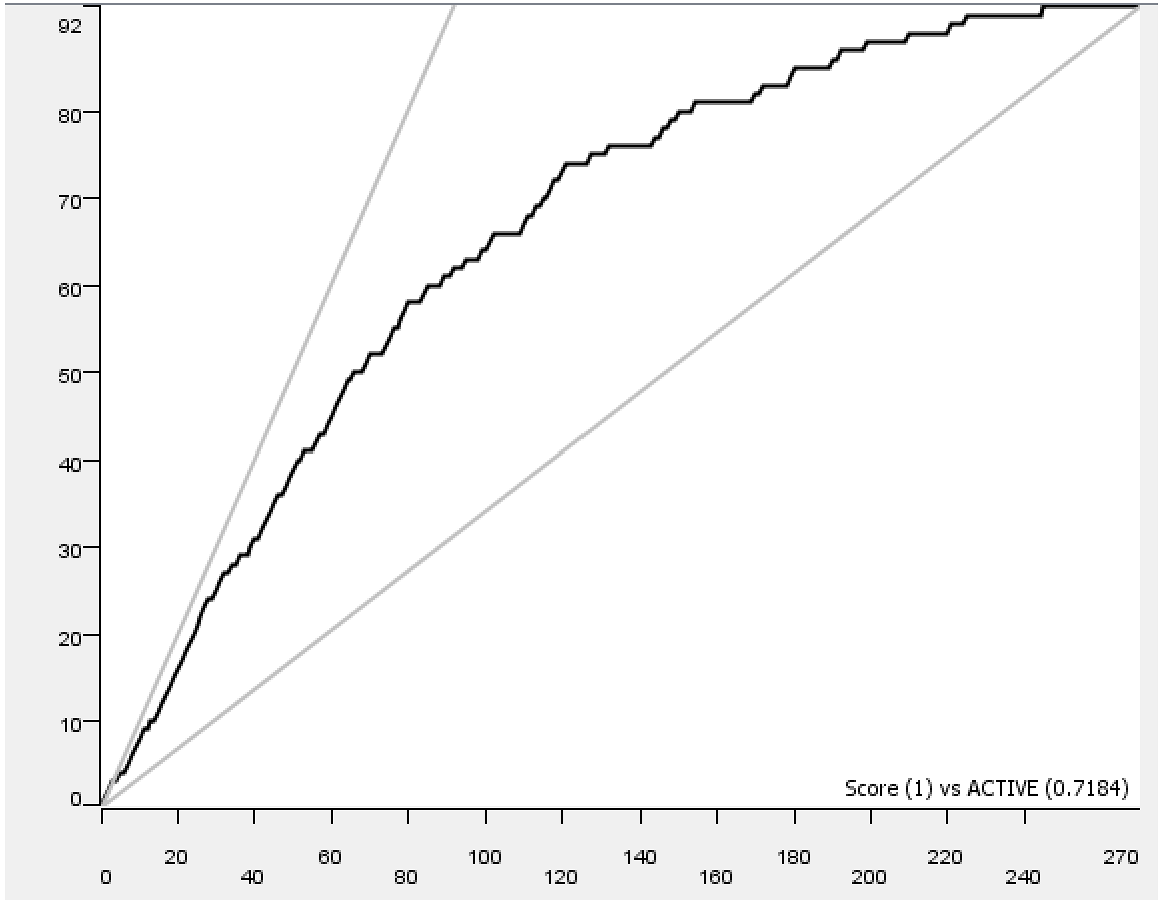

N=270

Top 10% Mean IC50	18.4 nM

	Predicted Active	Predicted Inactive
Active	74	18
Inactive	50	128

< 20 nM DefGood in ALK, 30% error; Random seed = 121783

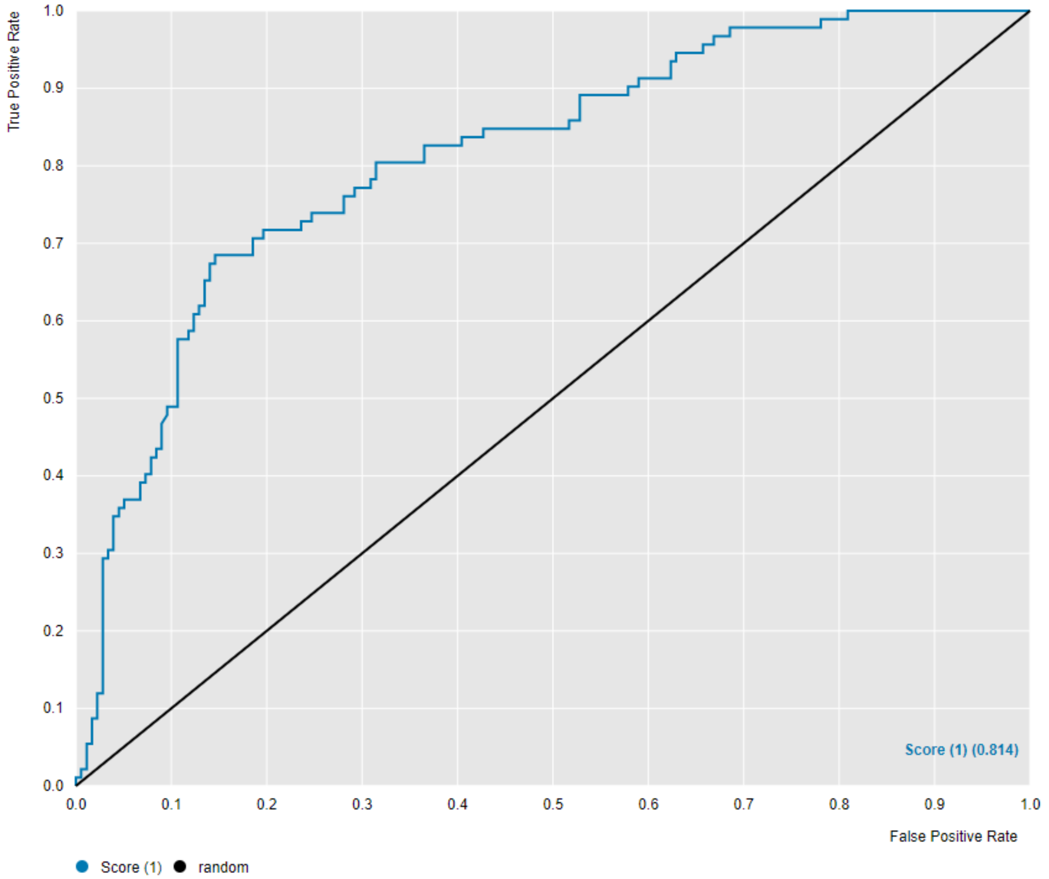

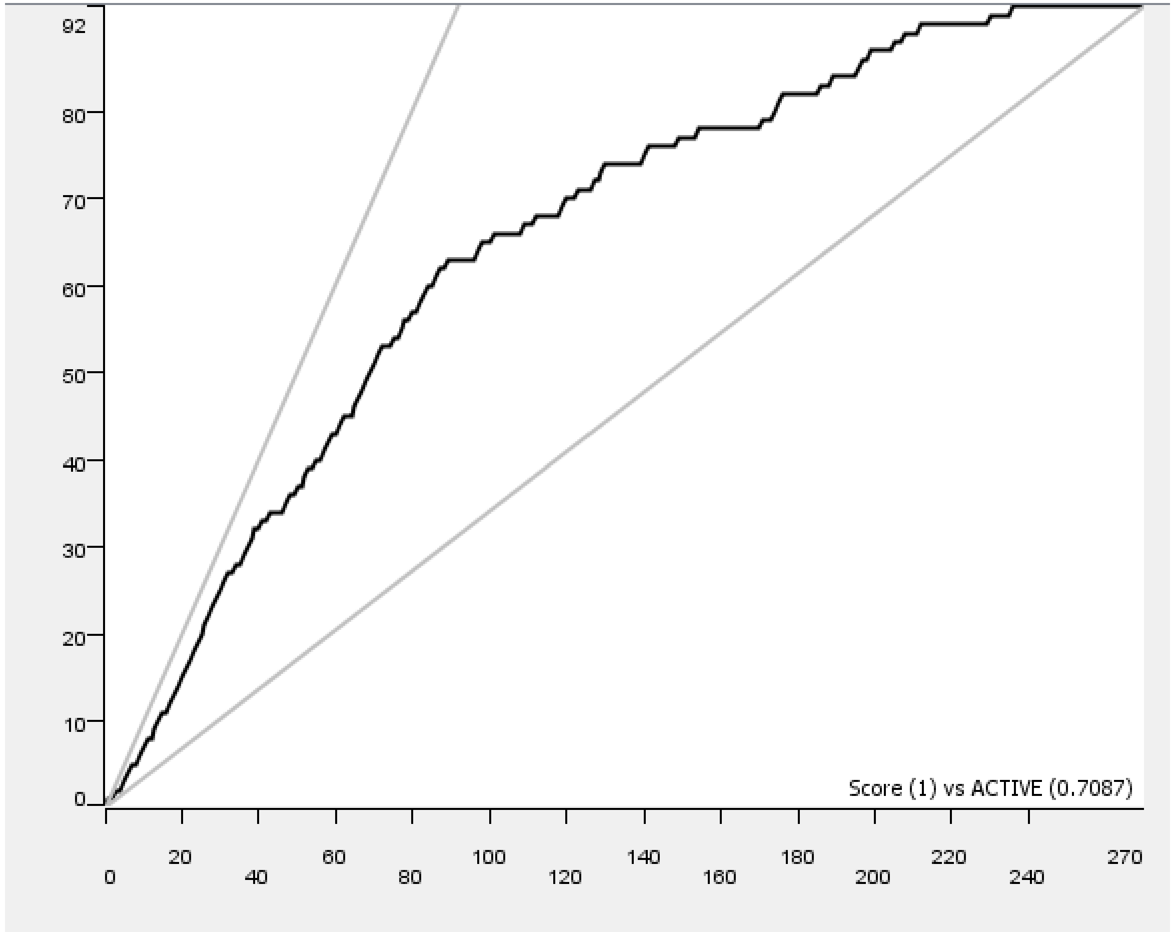

N=270

Top 10% Mean IC50	20.4 nM

	Predicted Active	Predicted Inactive
Active	63	29
Inactive	31	147

<20 nM DefGood in ALK, 40% error; Random seed = 121783

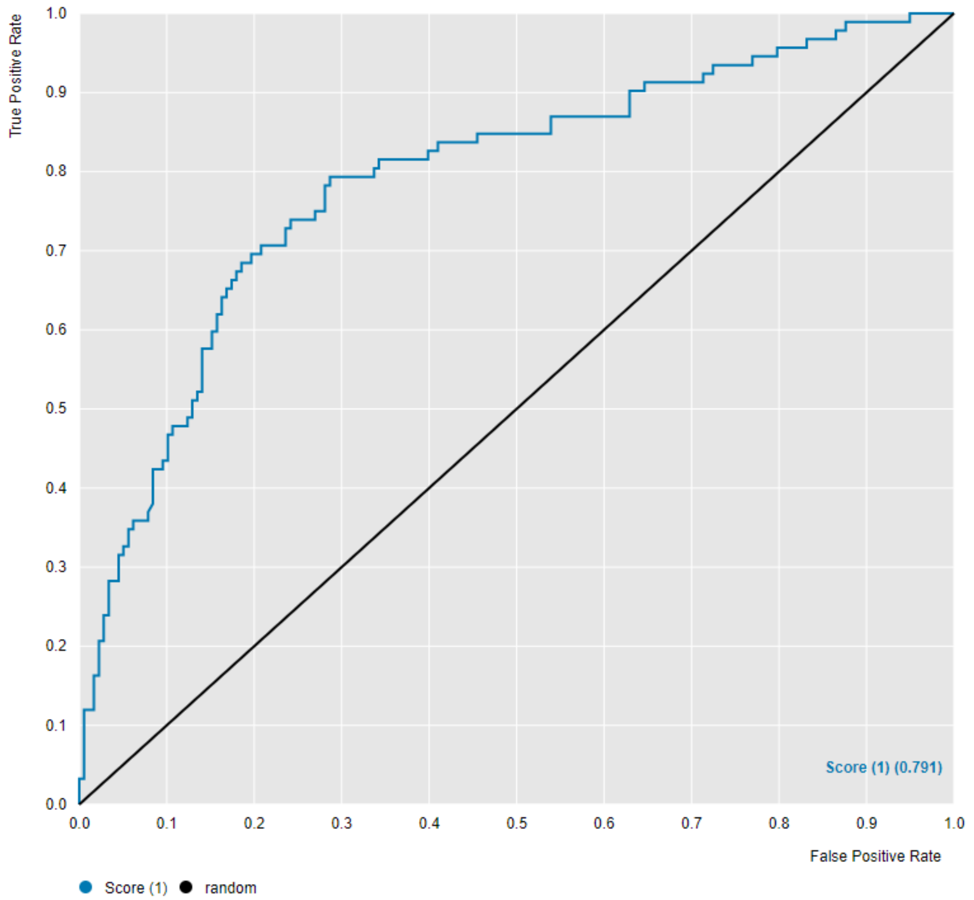

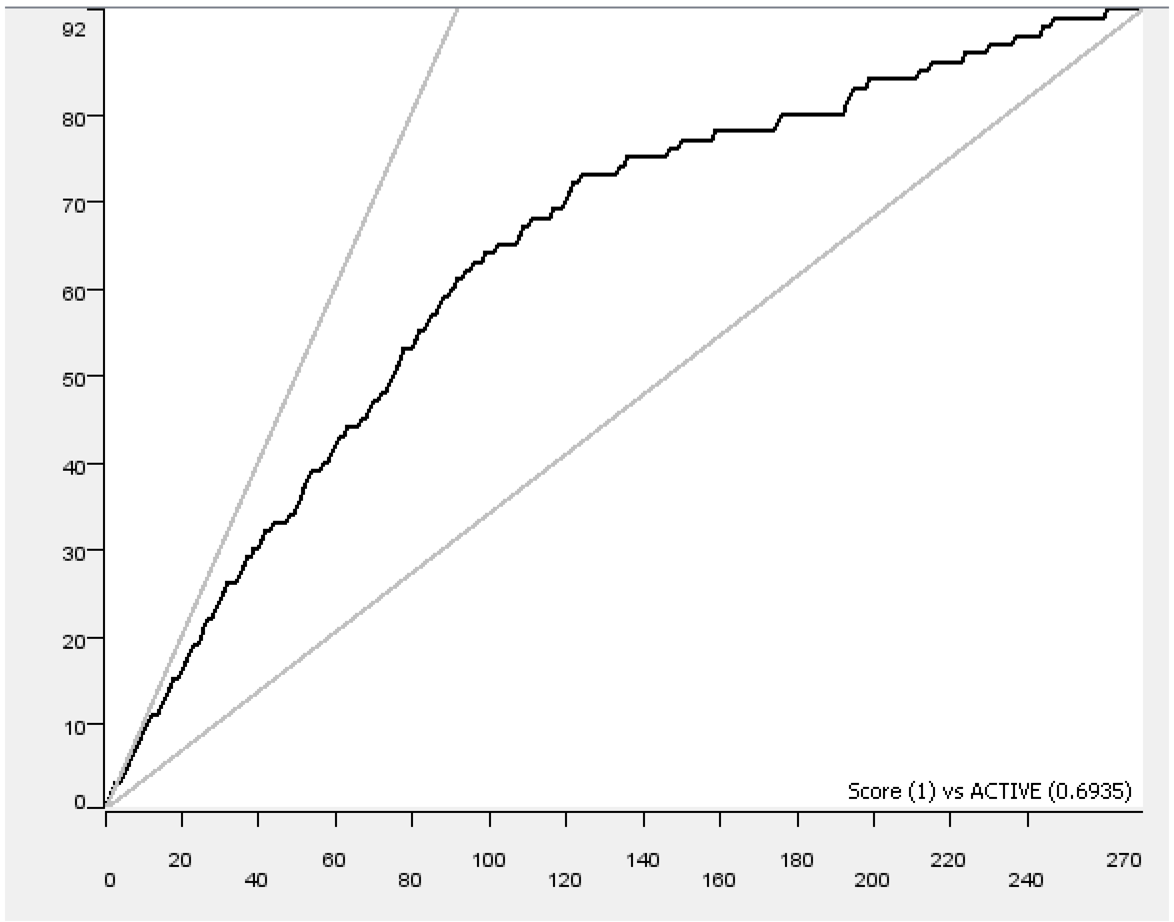

N=270

Top 10% Mean IC50	20.0 nM

	Predicted Active	Predicted Inactive
Active	57	35
Inactive	28	150

<20 nM DefGood in ALK, 45% error; Random seed = 121783

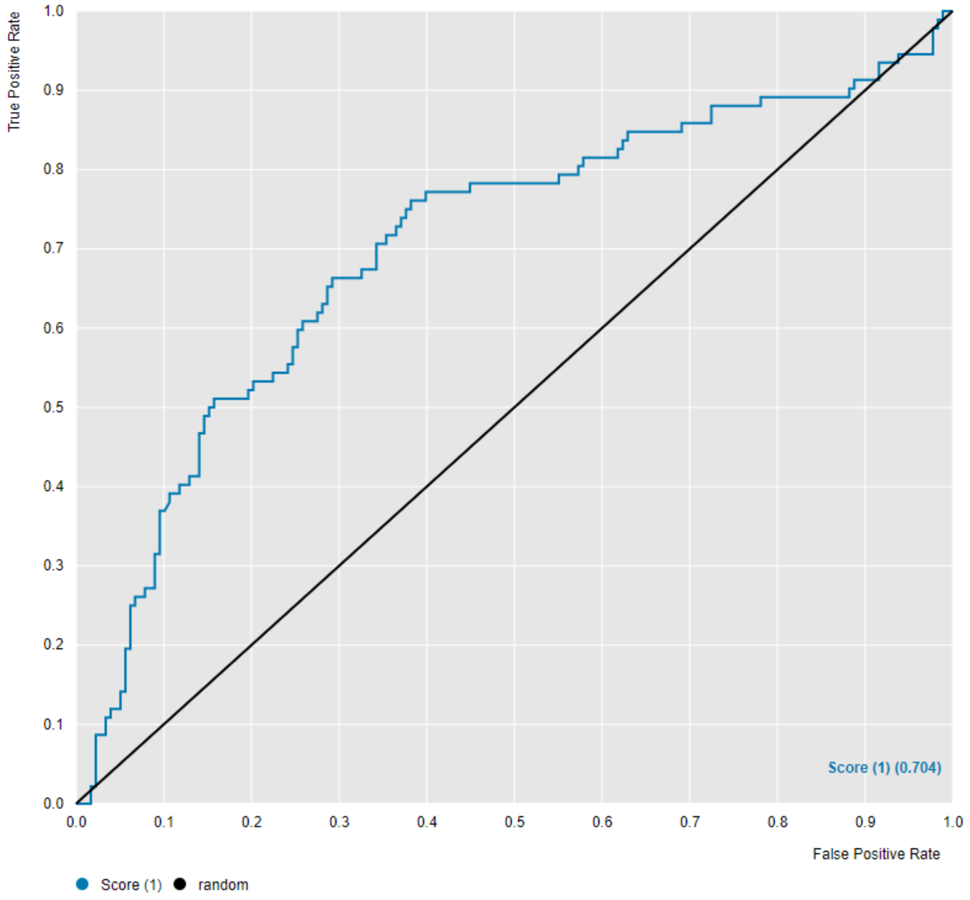

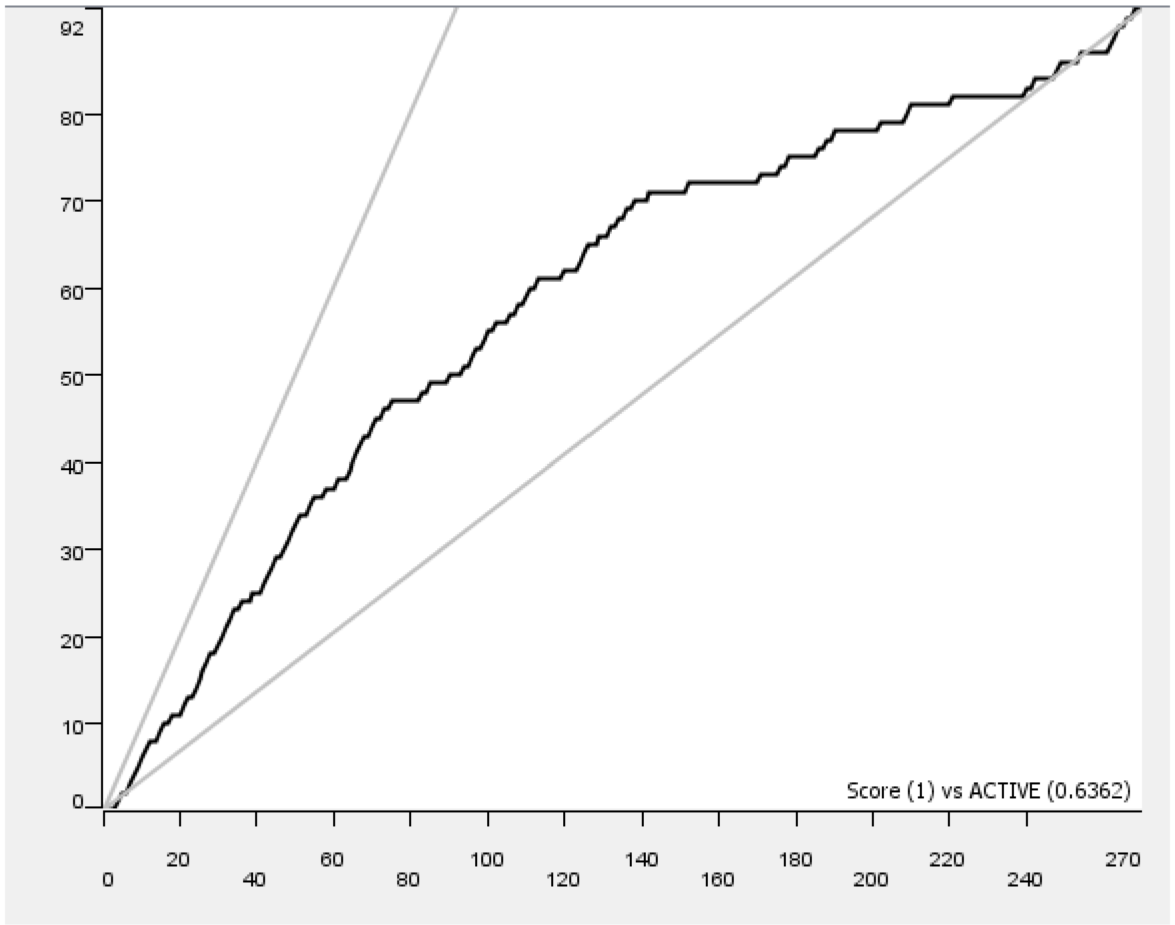

N=333

**Top 10% Mean IC50**	**130 nM**

	**Predicted Active**	**Predicted Inactive**
Active	47	45
Inactive	28	150

<20 nM DefGood in ALK, 50% error; Random seed = 121783

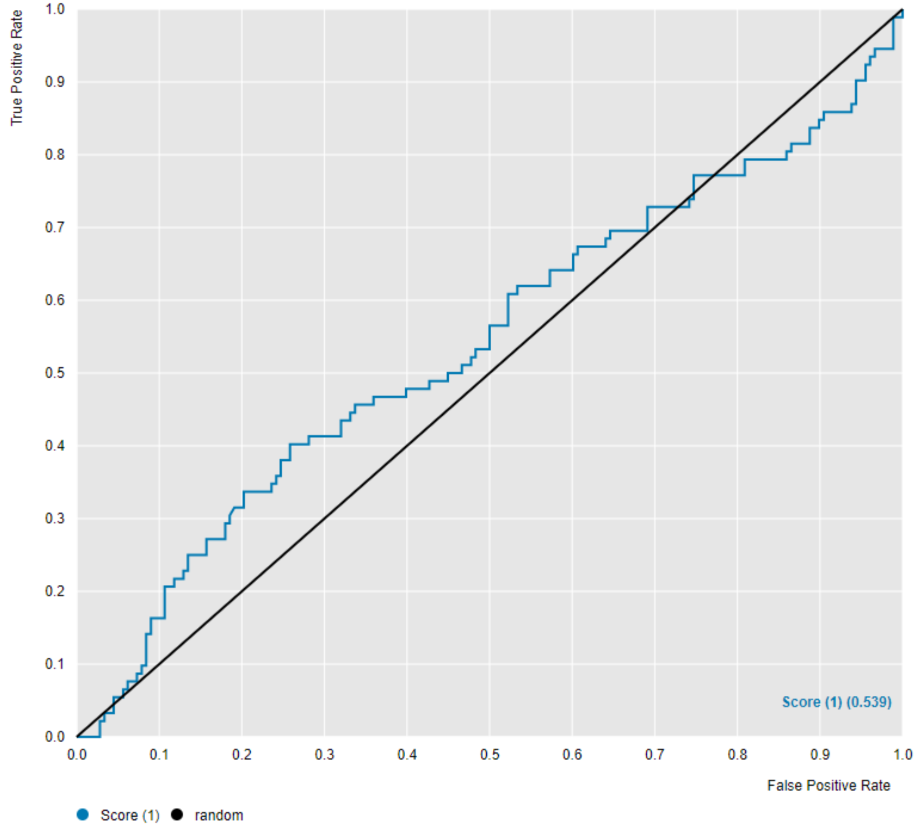

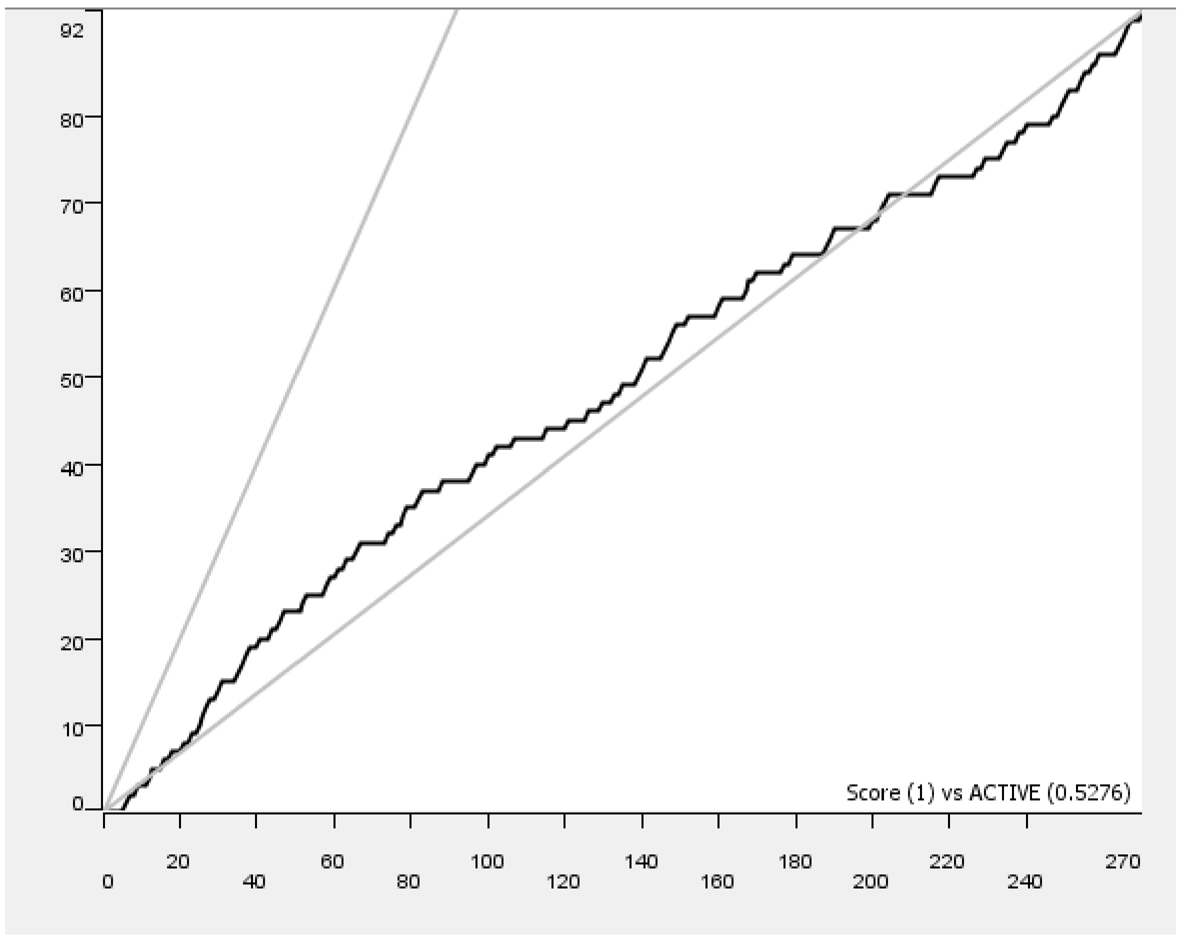

N=270

Top 10% Mean IC50	2,400 nM

	Predicted Active	Predicted Inactive
Active	67	25
Inactive	123	55

Conclusion - NBN

- An NBN could be generated for ALK with a decision value of <20 nM as defined as active. The predictivity of the algorithm could be preserved in three random train/test cycles. The point of failure was 45%, 45% and 50% error.

RF Error Tolerance- <20 nM DefGood in ALK

- 0-50% absolute error

RF- < 20 nM DefGood in ALK, 5% error; Random seed = 1515533876005

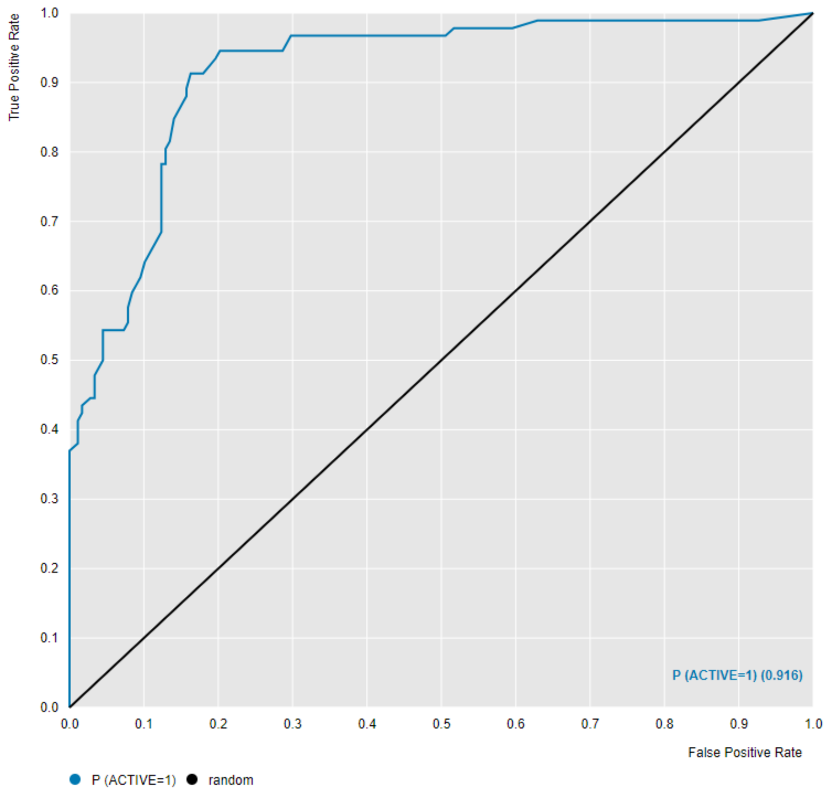

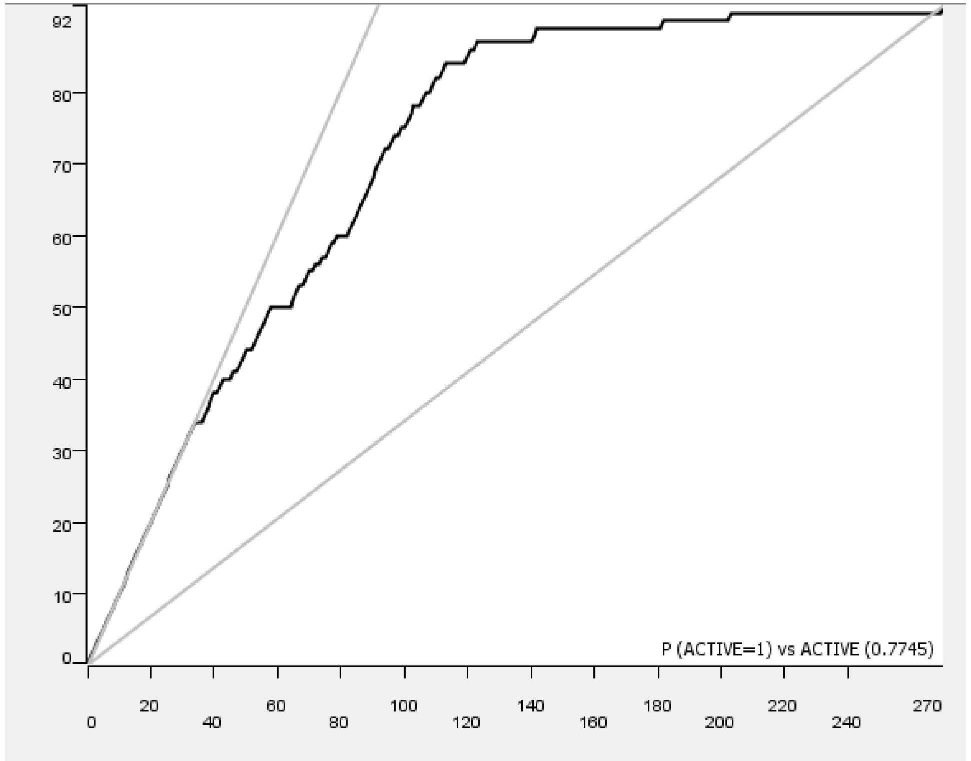

N=270

Top 10% Mean IC50	2.8 nM

	Predicted Active	Predicted Inactive
Active	66	26
Inactive	22	156

RF-< 20 nM DefGood in ALK, 10% error

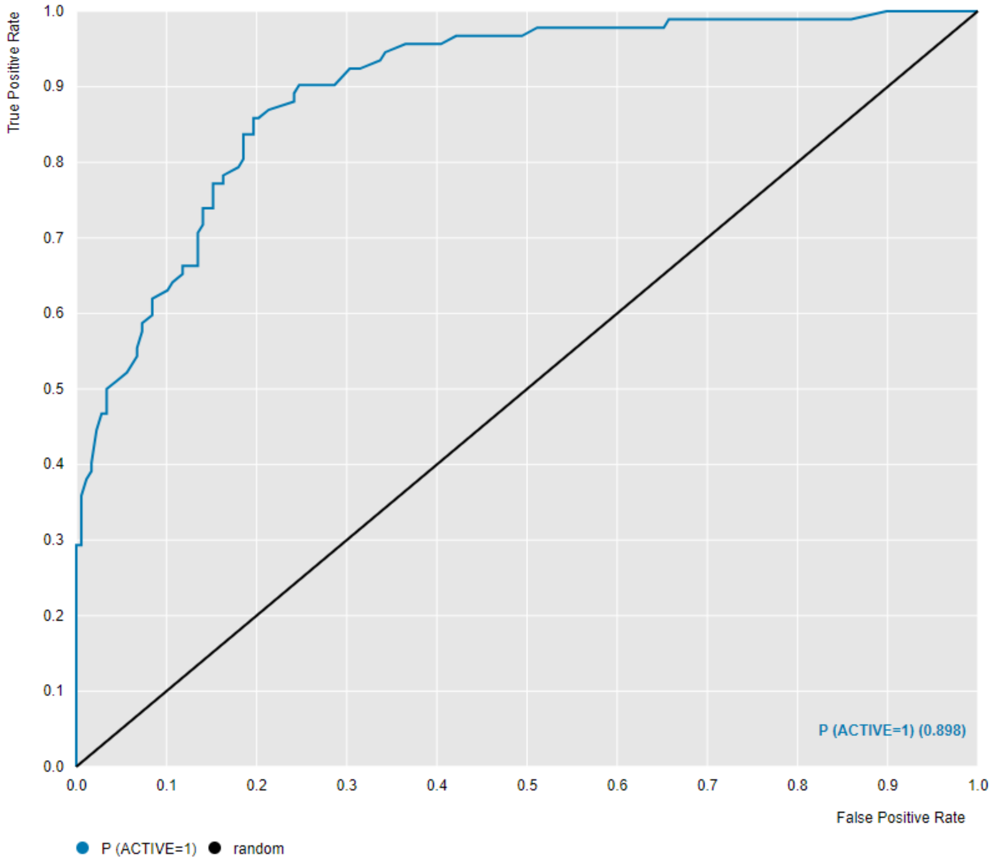

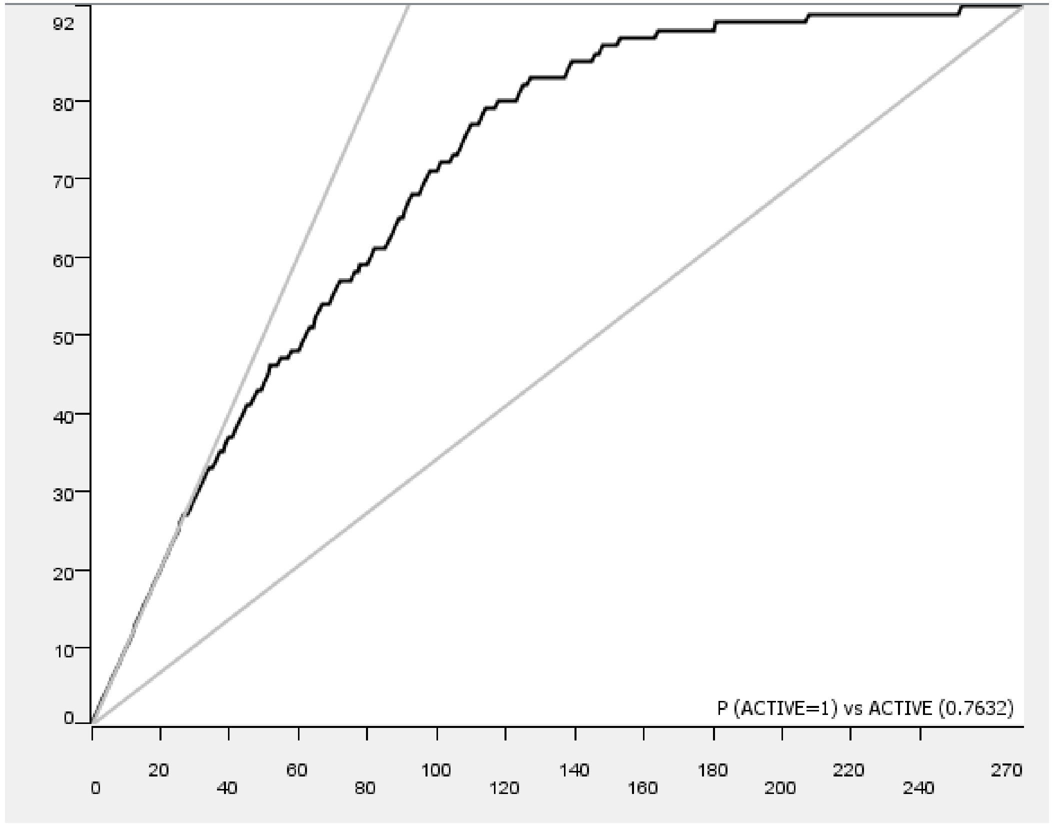

N=270

Top 10% Mean IC50	3.5 nM

	Predicted Active	Predicted Inactive
Active	68	24
Inactive	26	152

RF-< 20 nM DefGood in ALK, 20% error

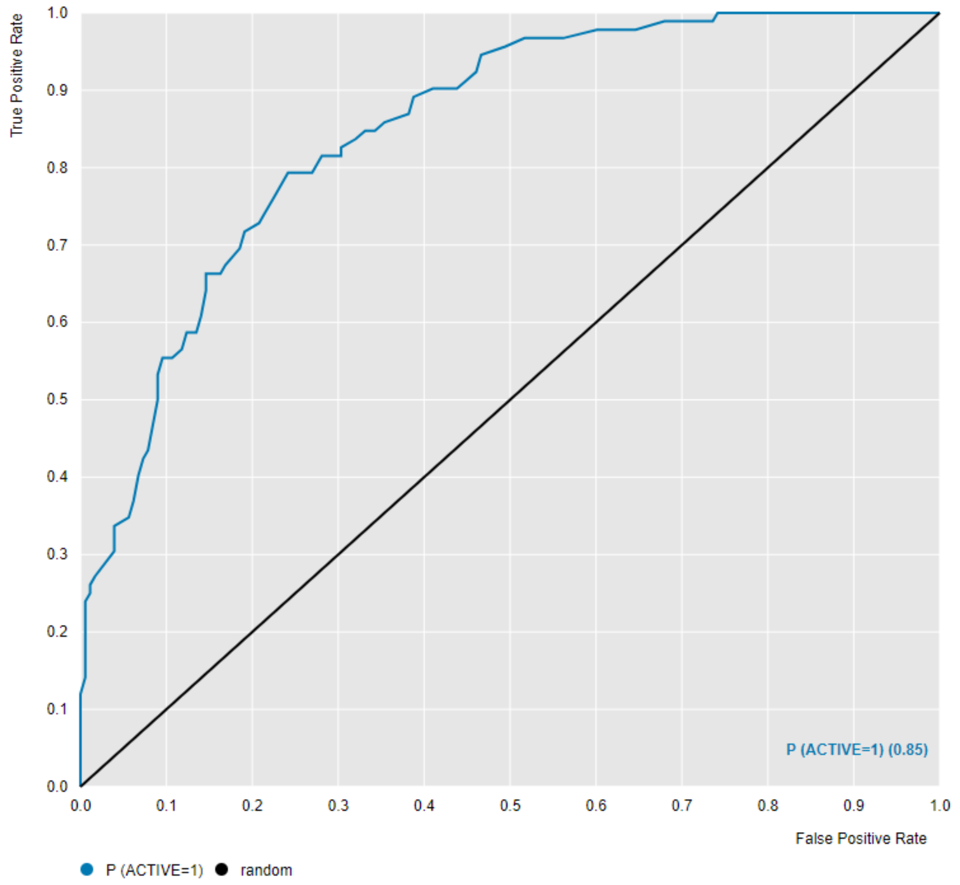

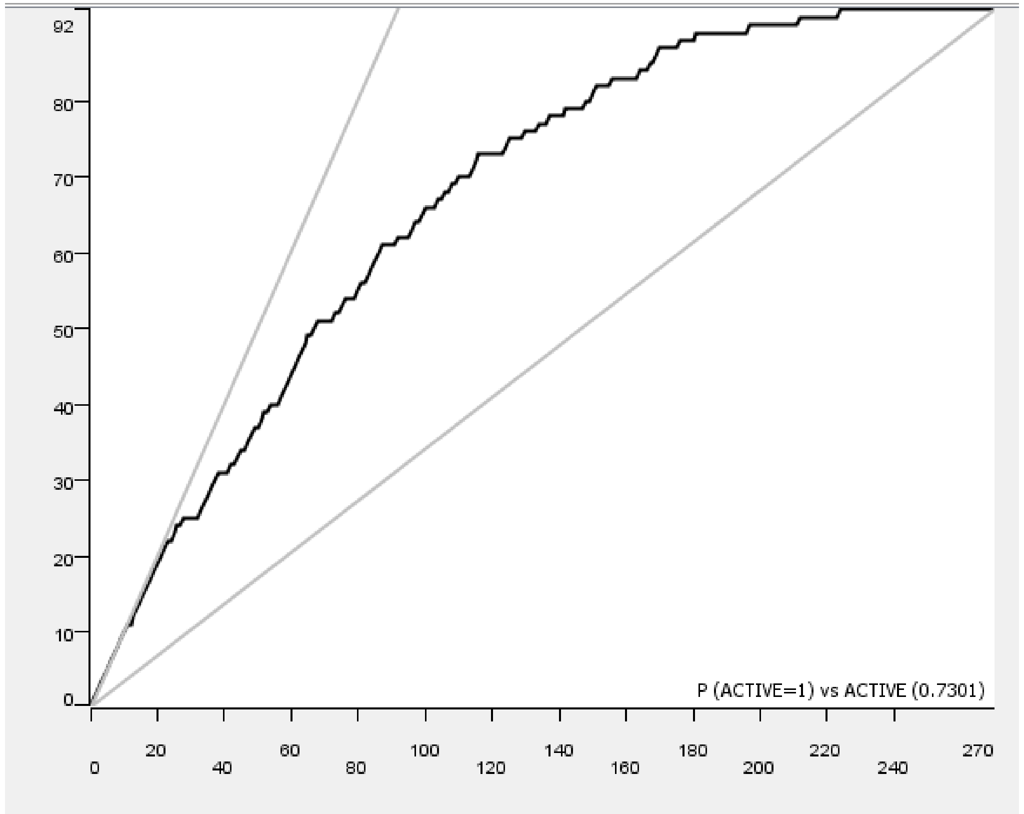

N=270

Top 10% Mean IC50	6.8 nM

	Predicted Active	Predicted Inactive
Active	62	30
Inactive	30	148

RF-< 20 nM DefGood in ALK, 25% error

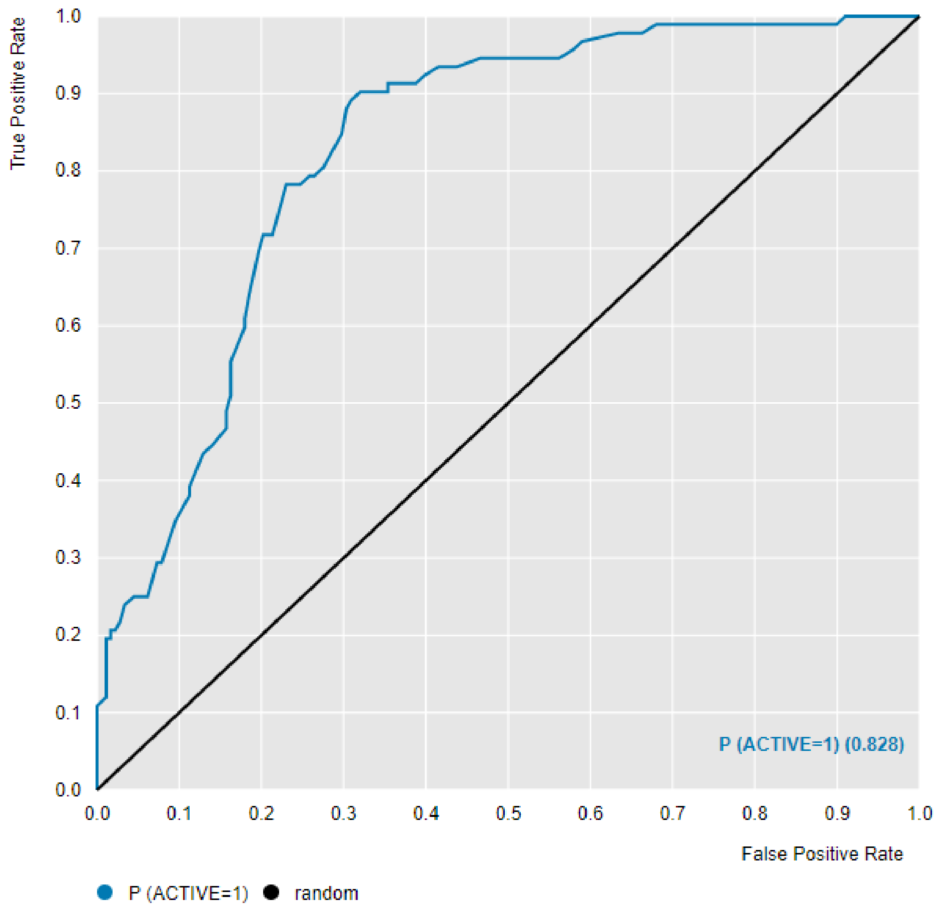

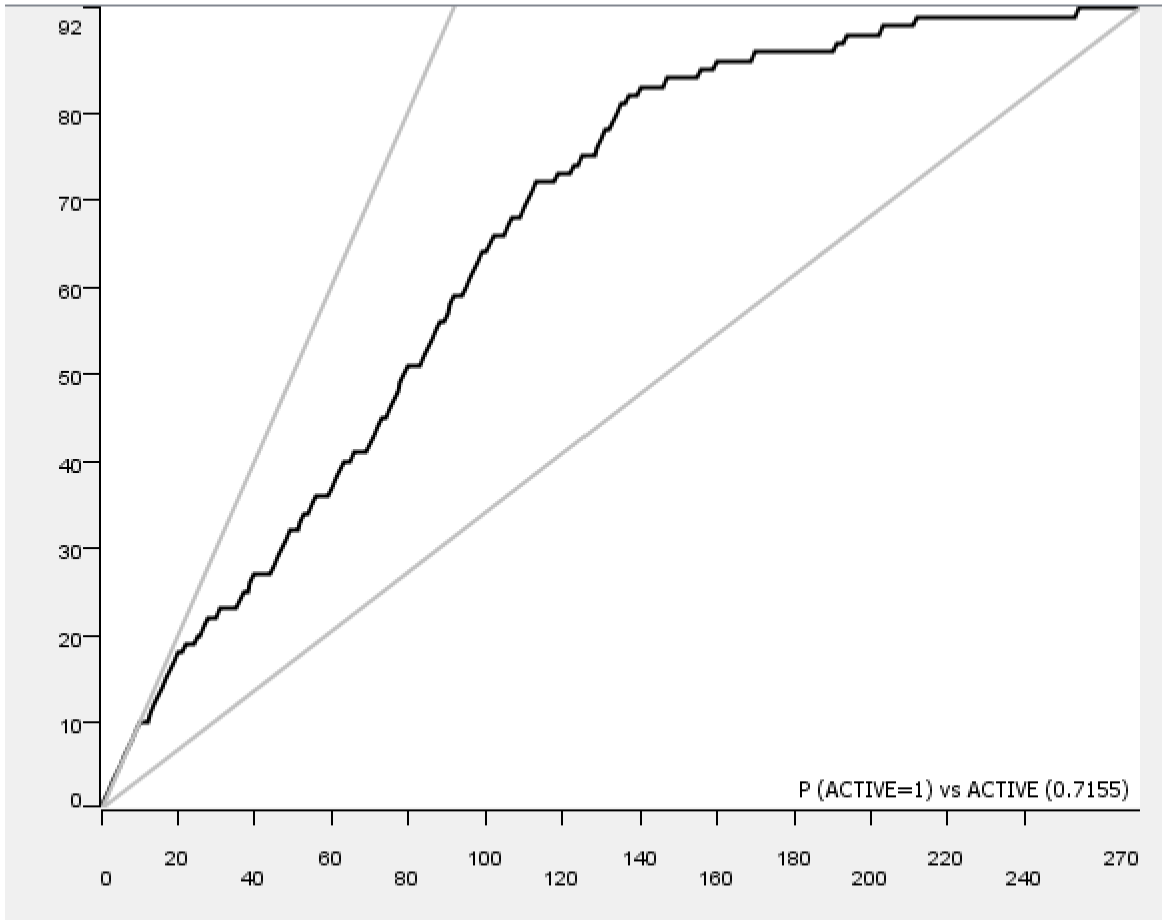

N=270

Top 10% Mean IC50	362 nM

	Predicted Active	Predicted Inactive
Active	66	26
Inactive	38	140

RF-<20 nM DefGood in ALK, 30% error

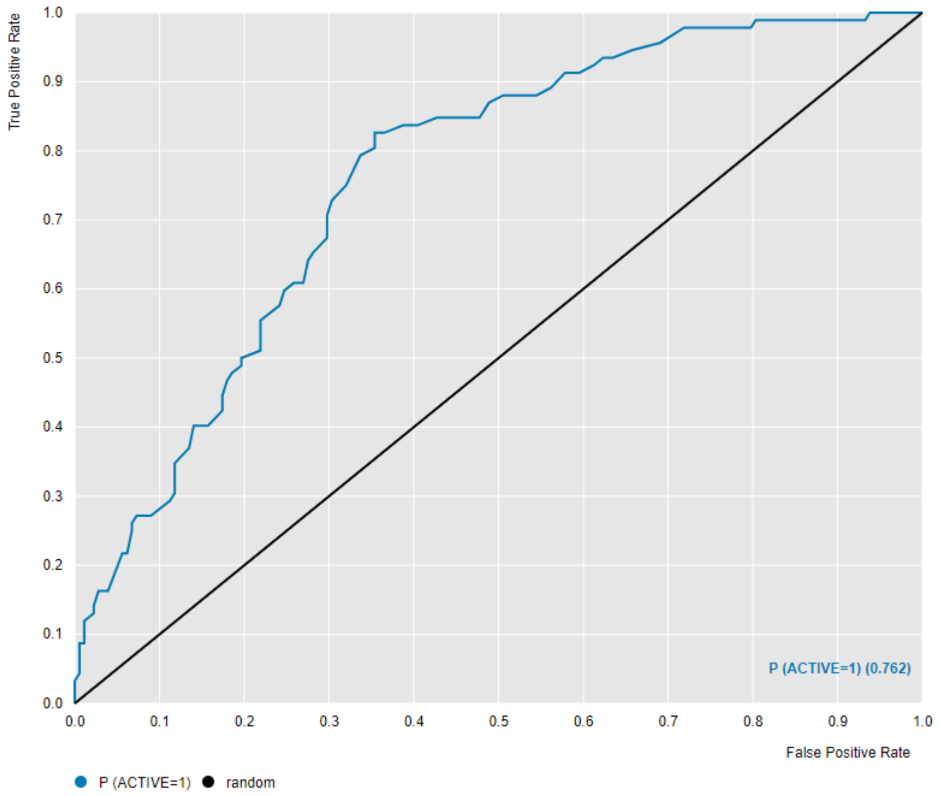

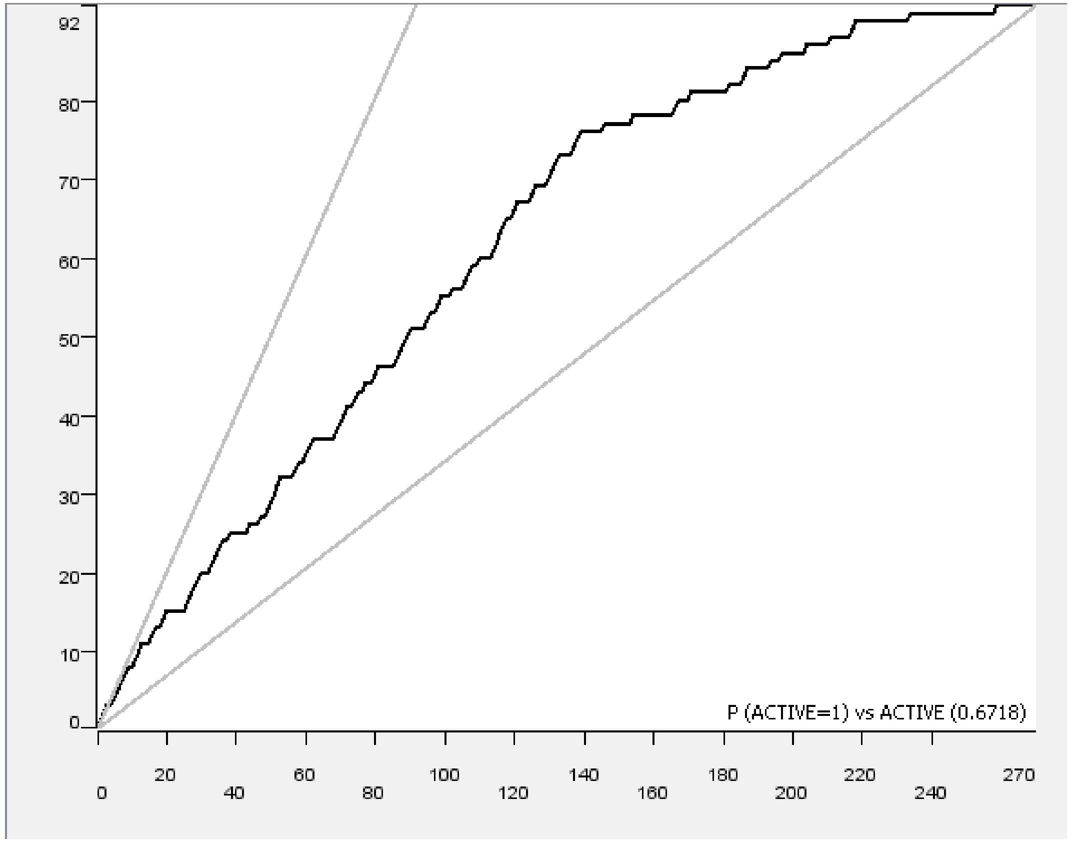

N=270

Top 10% Mean IC50	1,800 nM

	Predicted Active	Predicted Inactive
Active	56	36
Inactive	46	132

< 20 nM DefGood in ALK, 10% error; Random seed = 429

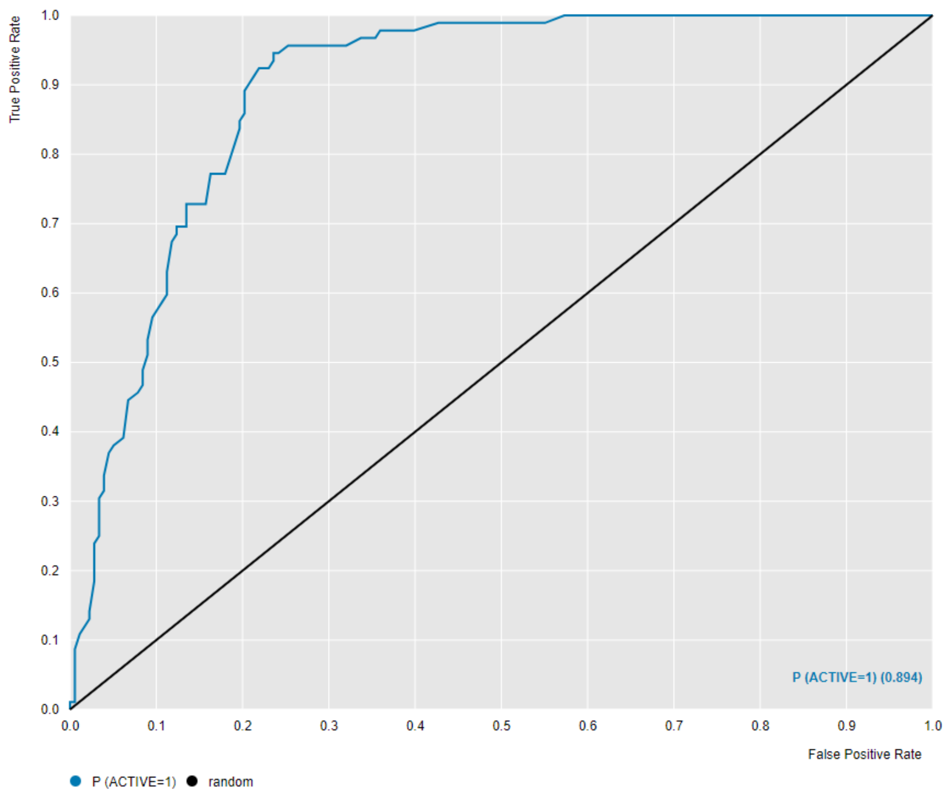

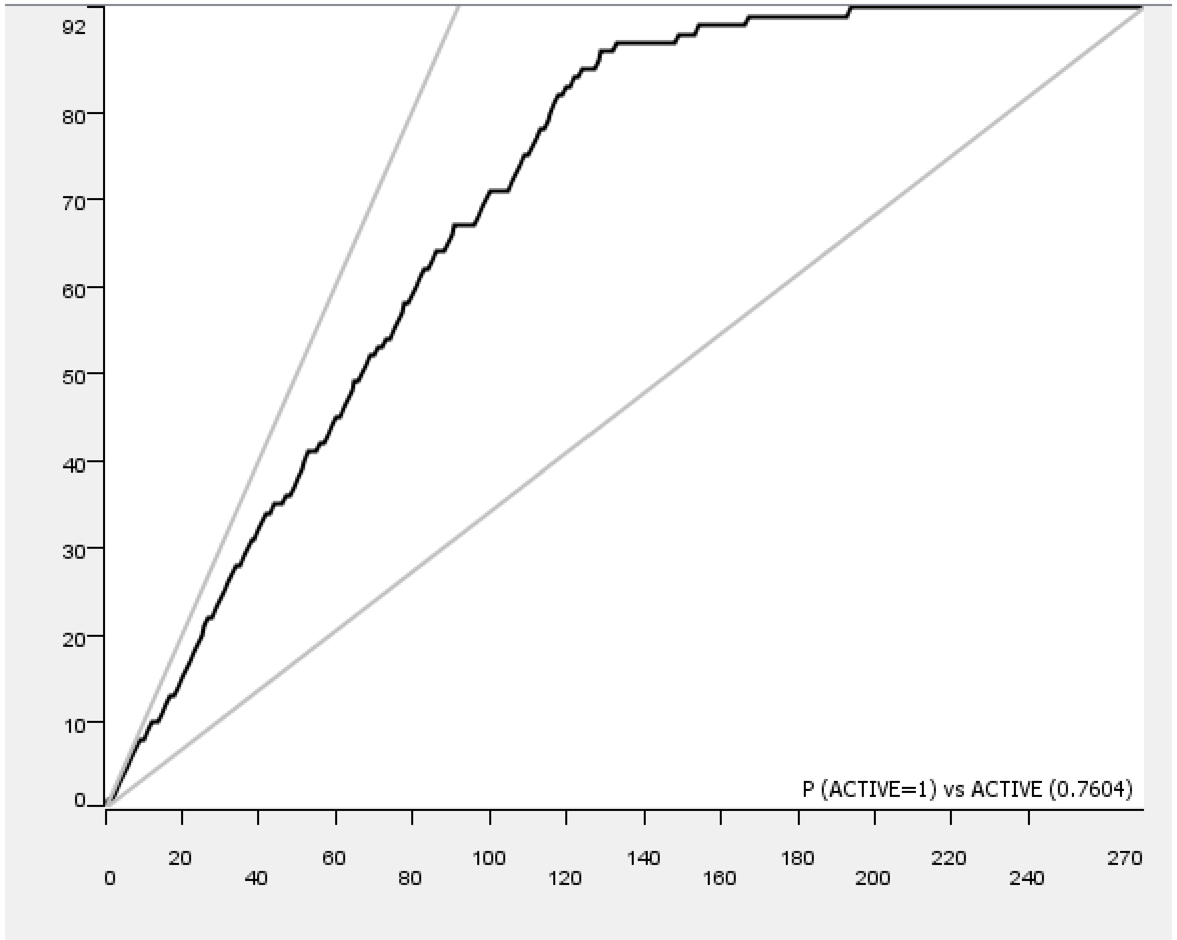

N=270

Top 10% Mean IC50	16.2 nM

	Predicted Active	Predicted Inactive
Active	67	25
Inactive	28	150

< 20 nM DefGood in ALK, 20% error; Random seed = 429

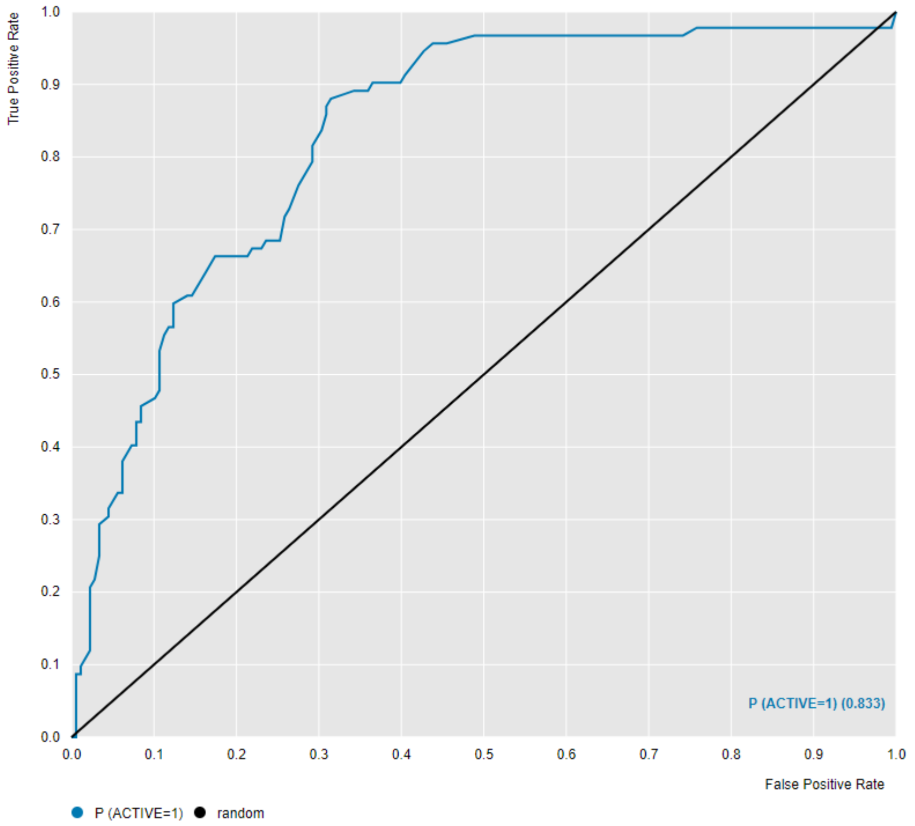

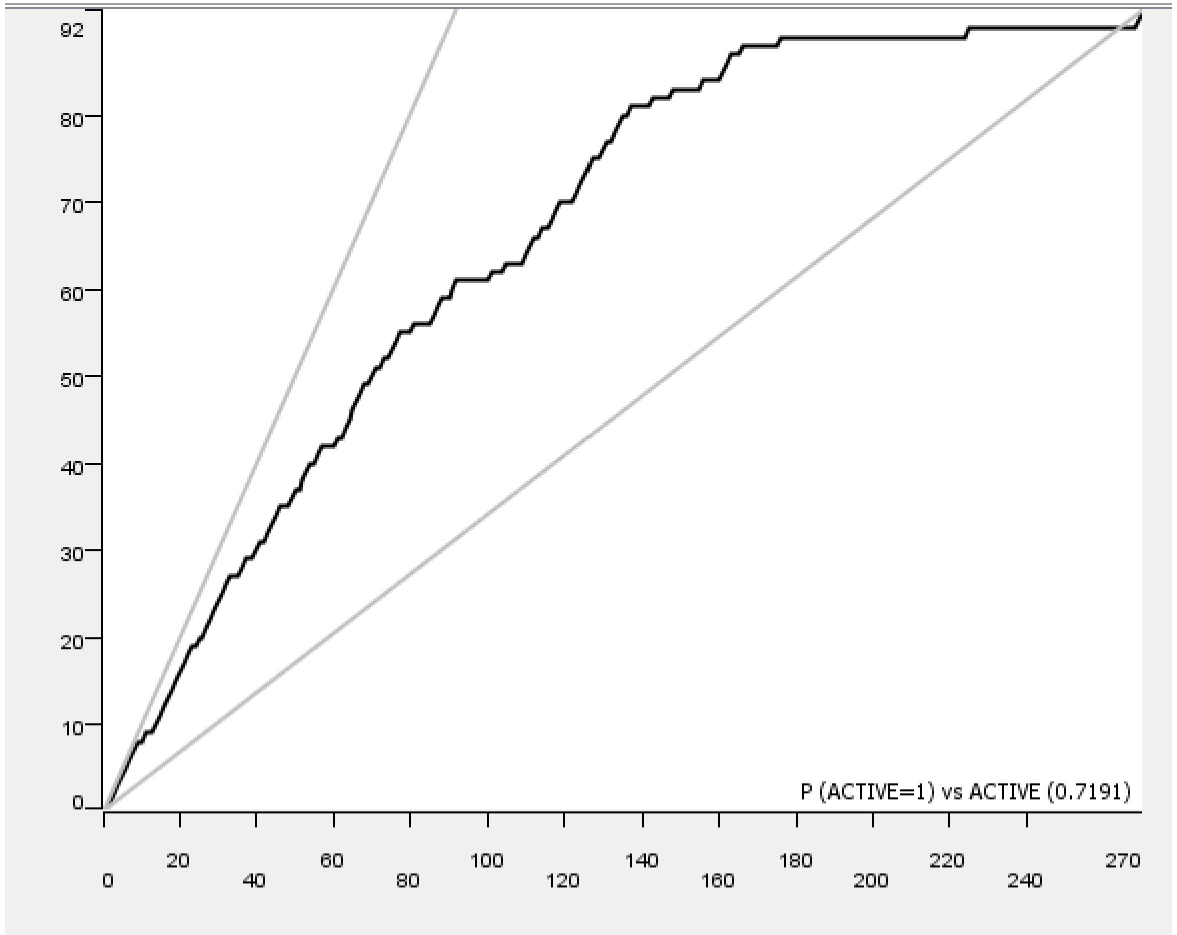

N=270

Top 10% Mean IC50	139 nM

	Predicted Active	Predicted Inactive
Active	62	30
Inactive	39	139

< 20 nM DefGood in ALK, 25% error; Random seed = 429

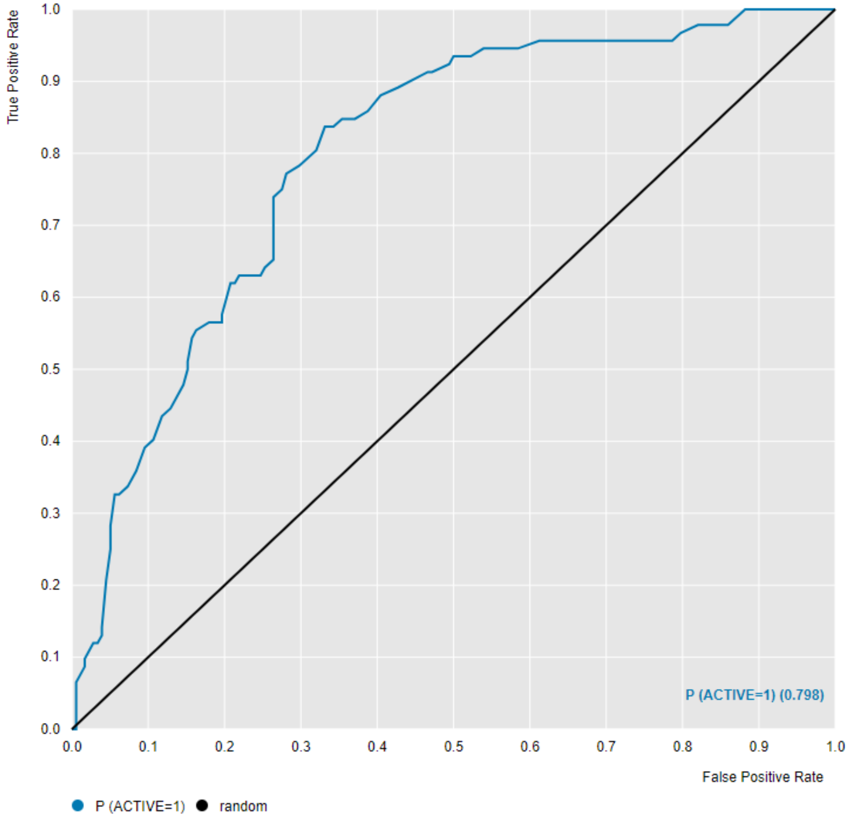

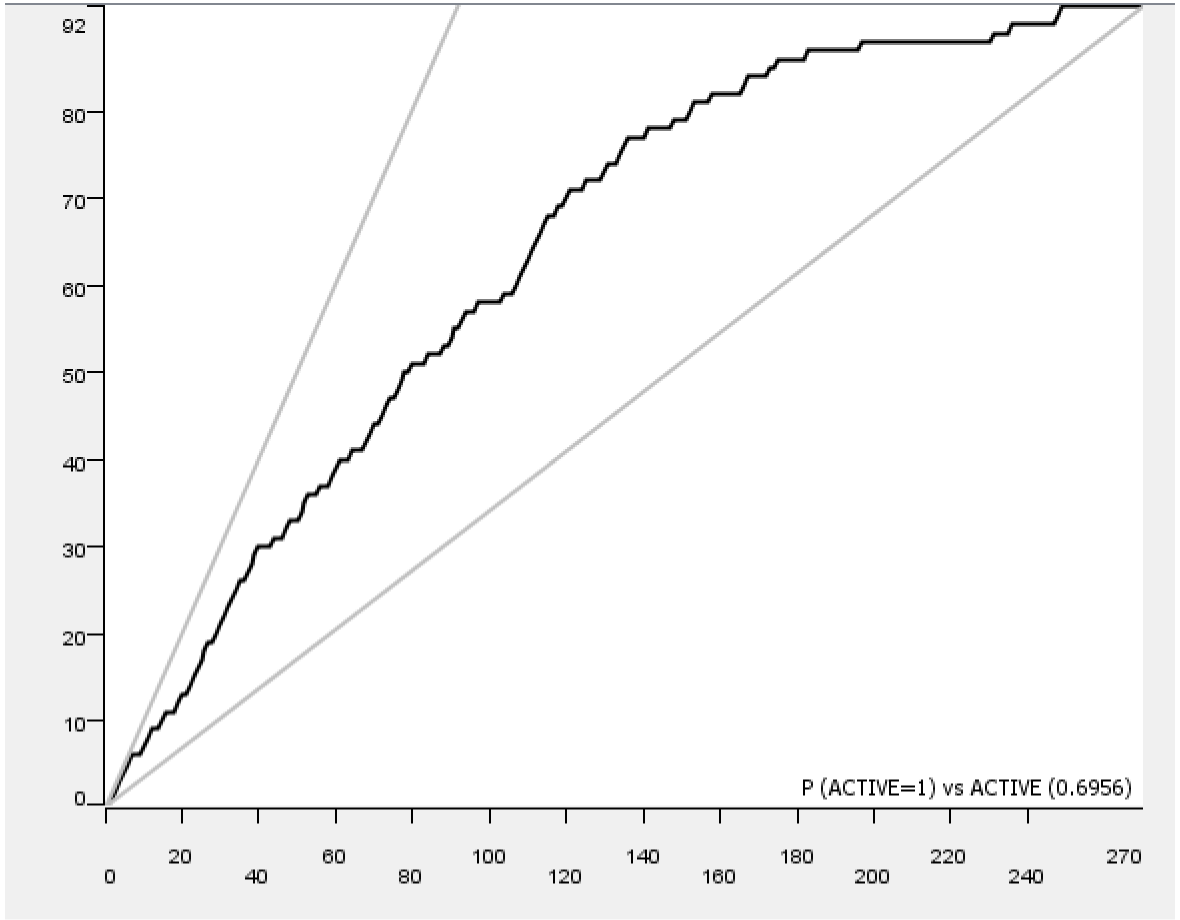

N=270

Top 10% Mean IC50	233 nM

	Predicted Active	Predicted Inactive
Active	58	34
Inactive	43	135

< 20 nM DefGood in ALK, 35% error; Random seed = 429

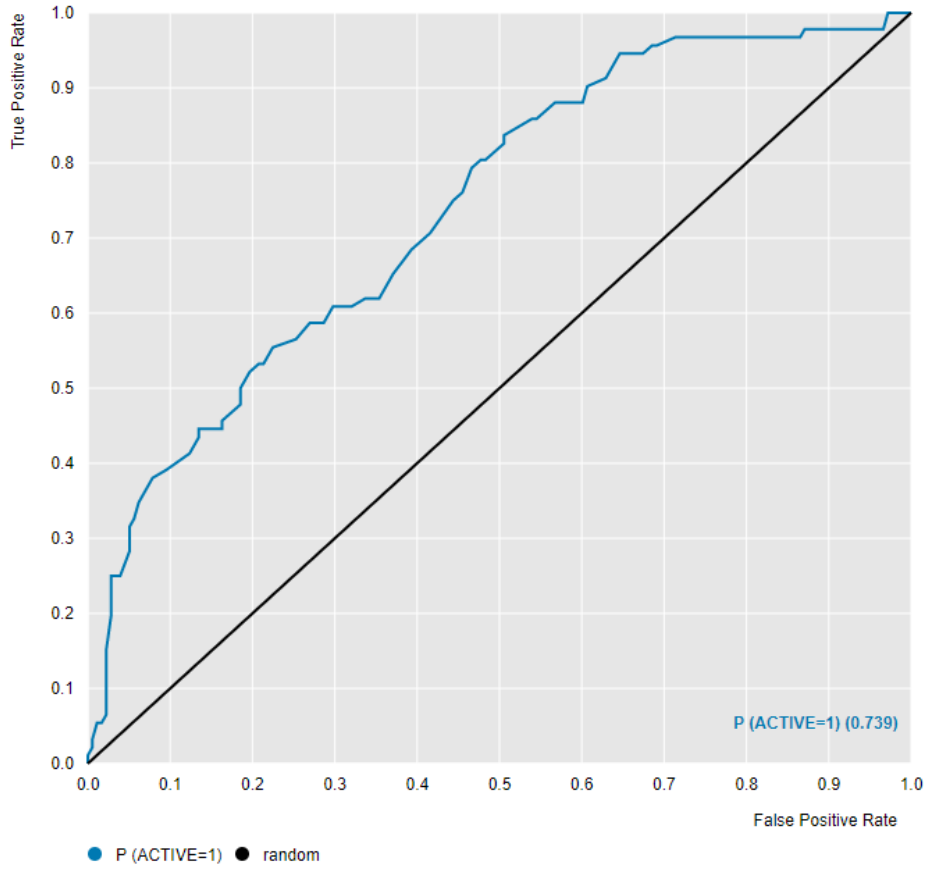

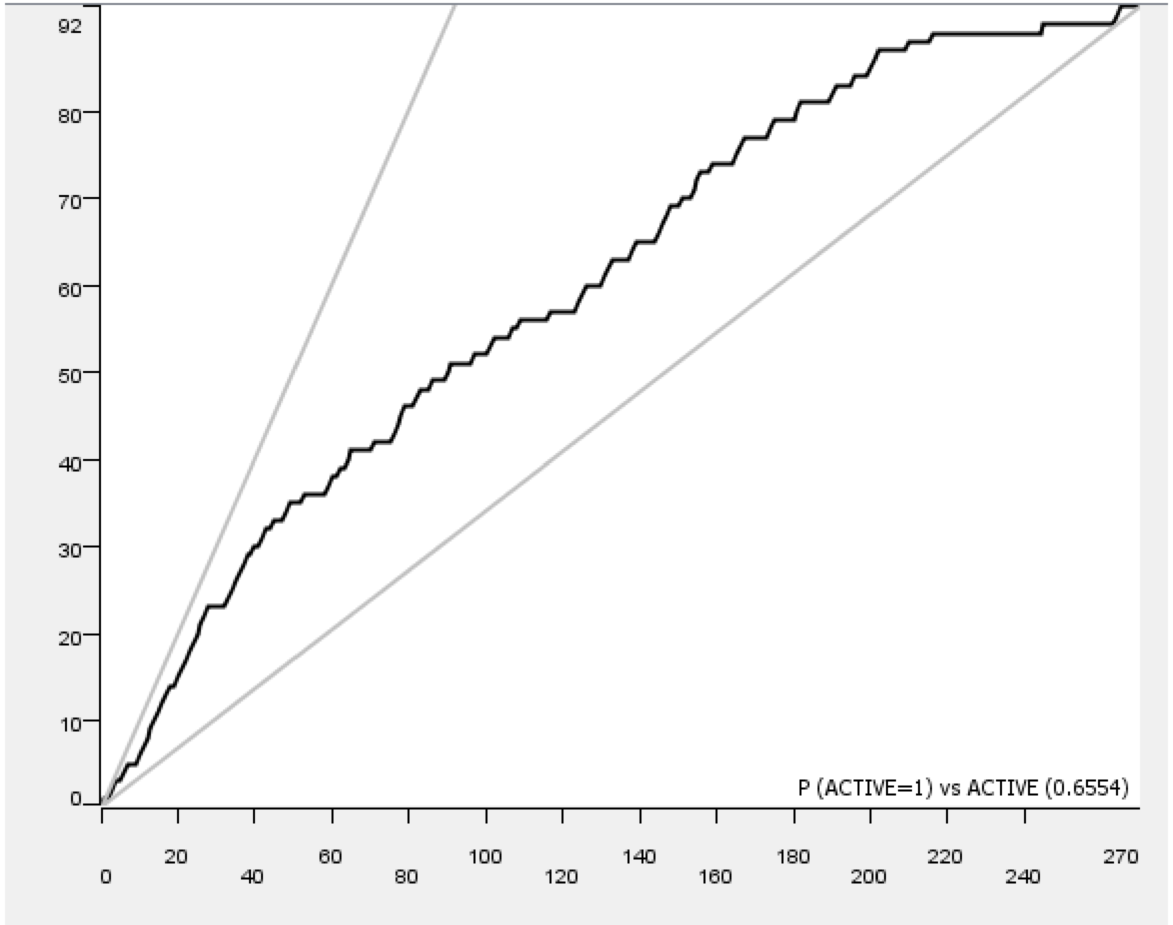

N=270

**Top 10% Mean IC50**	**111 nM**

	**Predicted Active**	**Predicted Inactive**
Active	55	37
Inactive	52	126

< 20 nM DefGood in ALK, 40% error; Random seed = 429

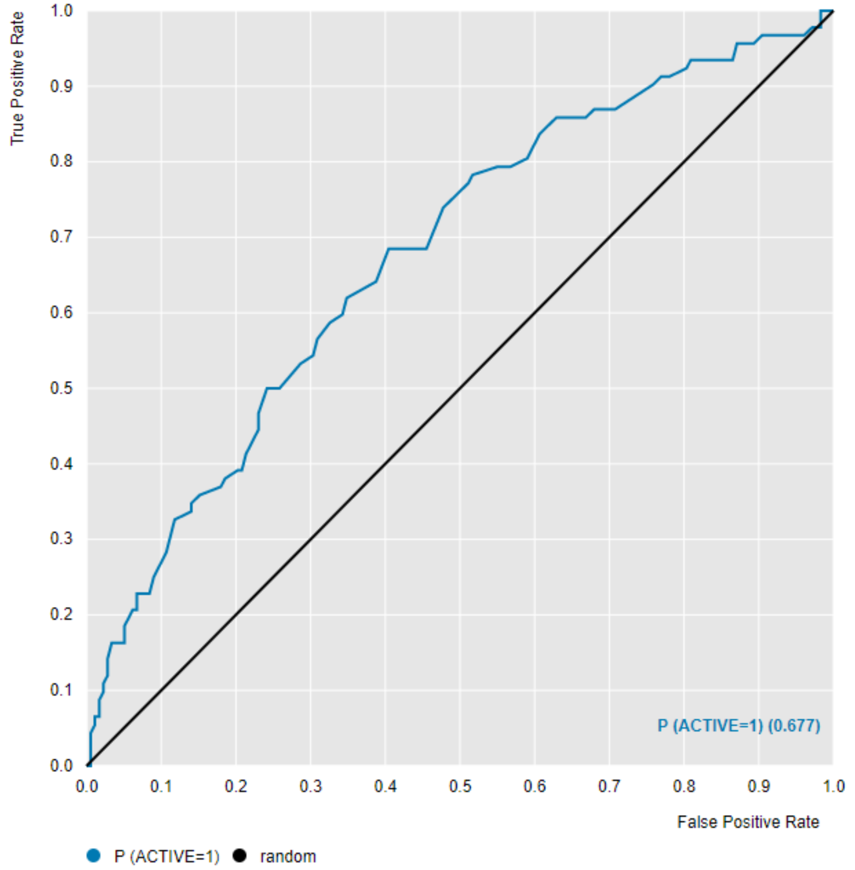

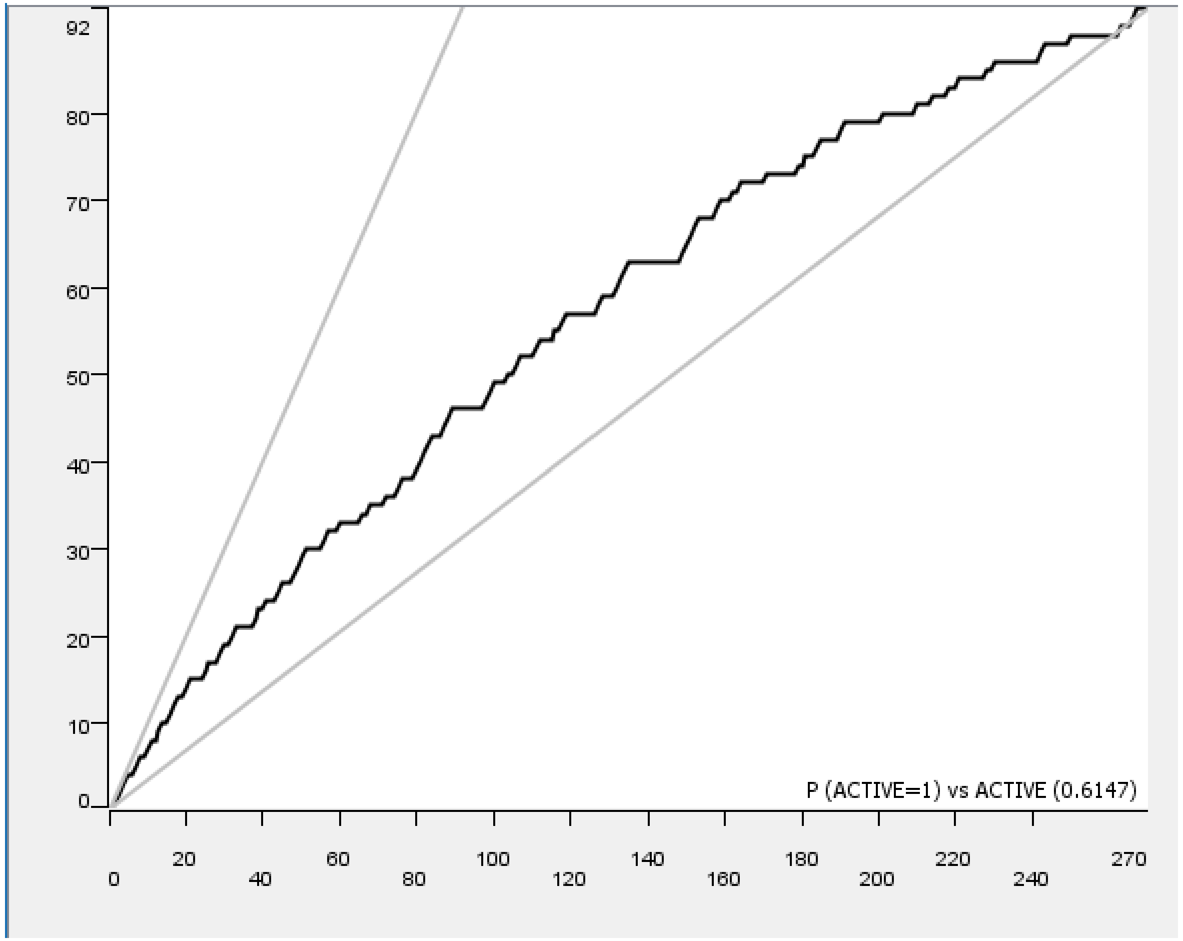

N=270

Top 10% Mean IC50	383 nM

	Predicted Active	Predicted Inactive
Active	50	42
Inactive	54	124

< 20 nM DefGood in ALK, 10% error; Random seed = 121783

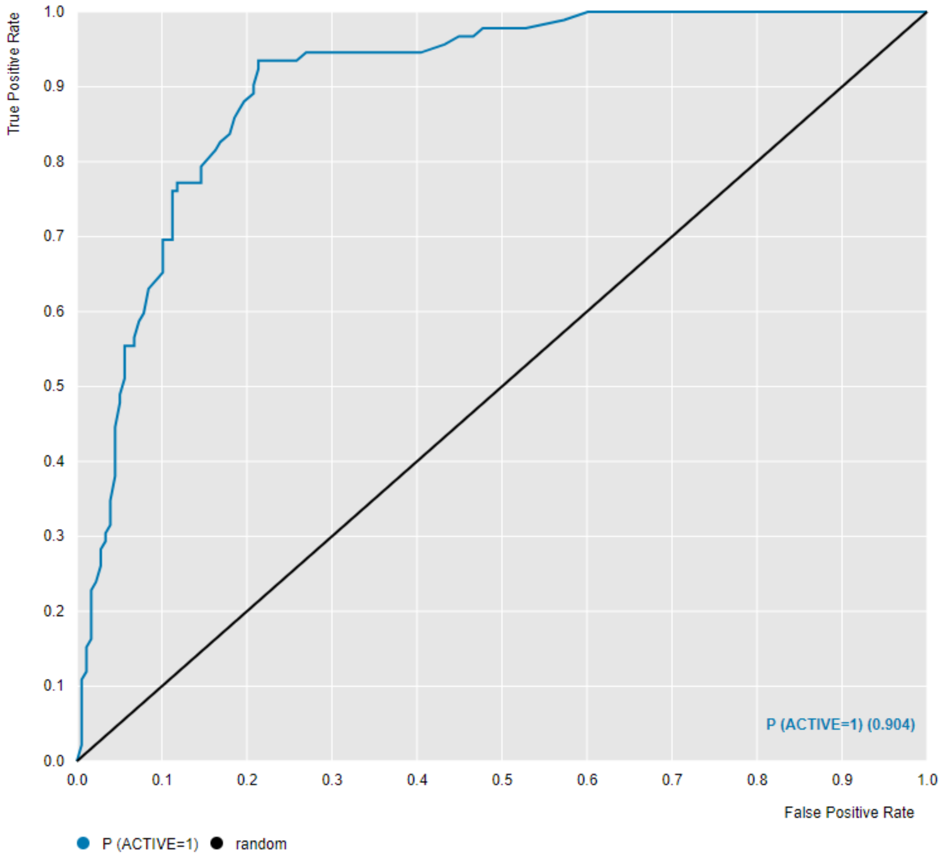

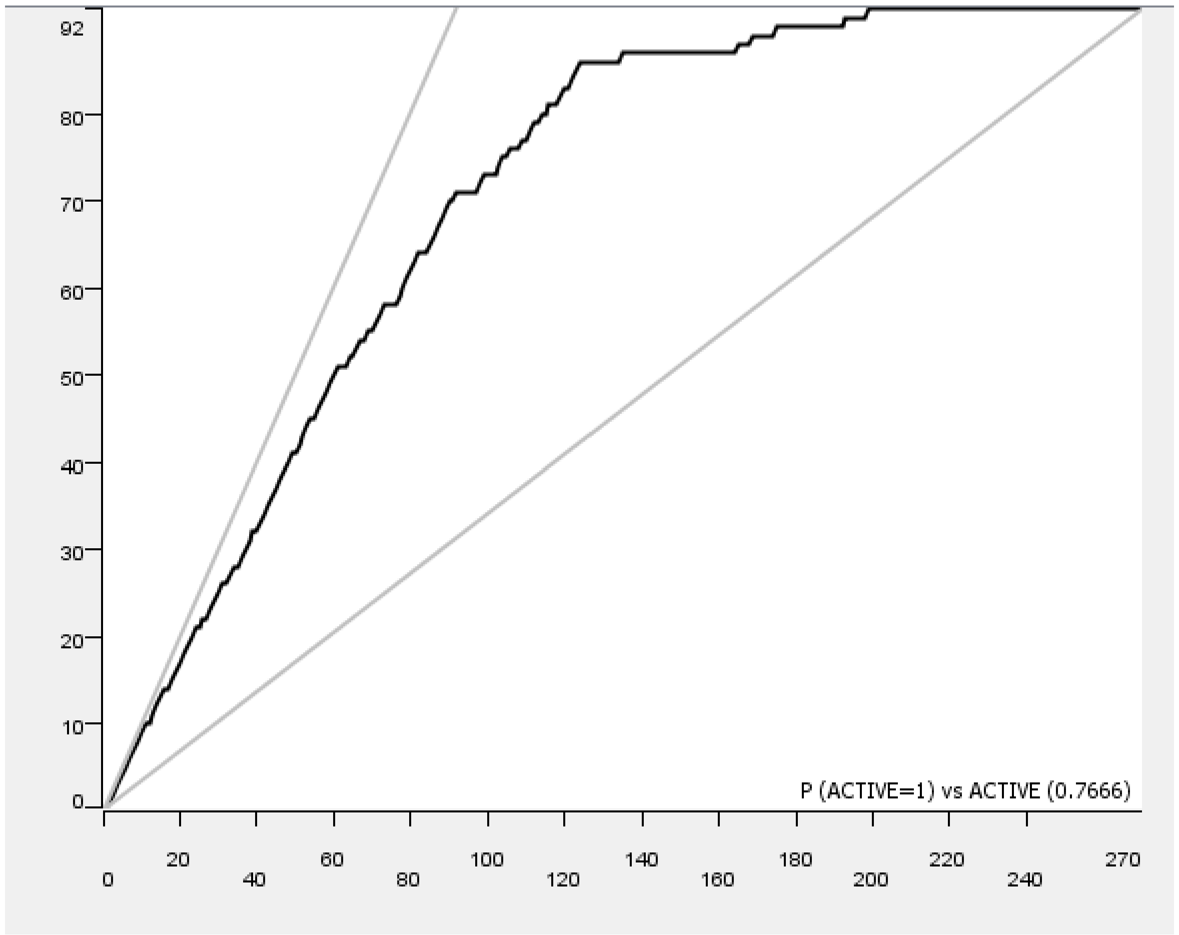

N=270

Top 10% Mean IC50	13.4 nM

	Predicted Active	Predicted Inactive
Active	71	21
Inactive	21	157

< 20 nM DefGood in ALK, 20% error; Random seed = 121783

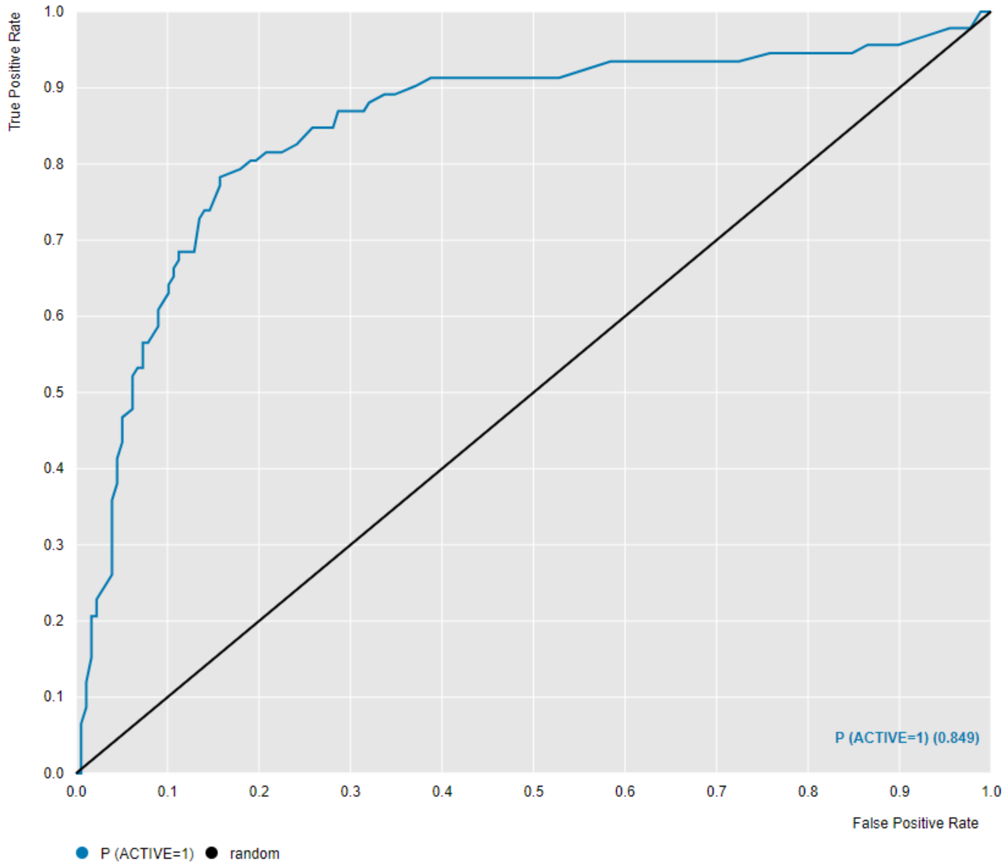

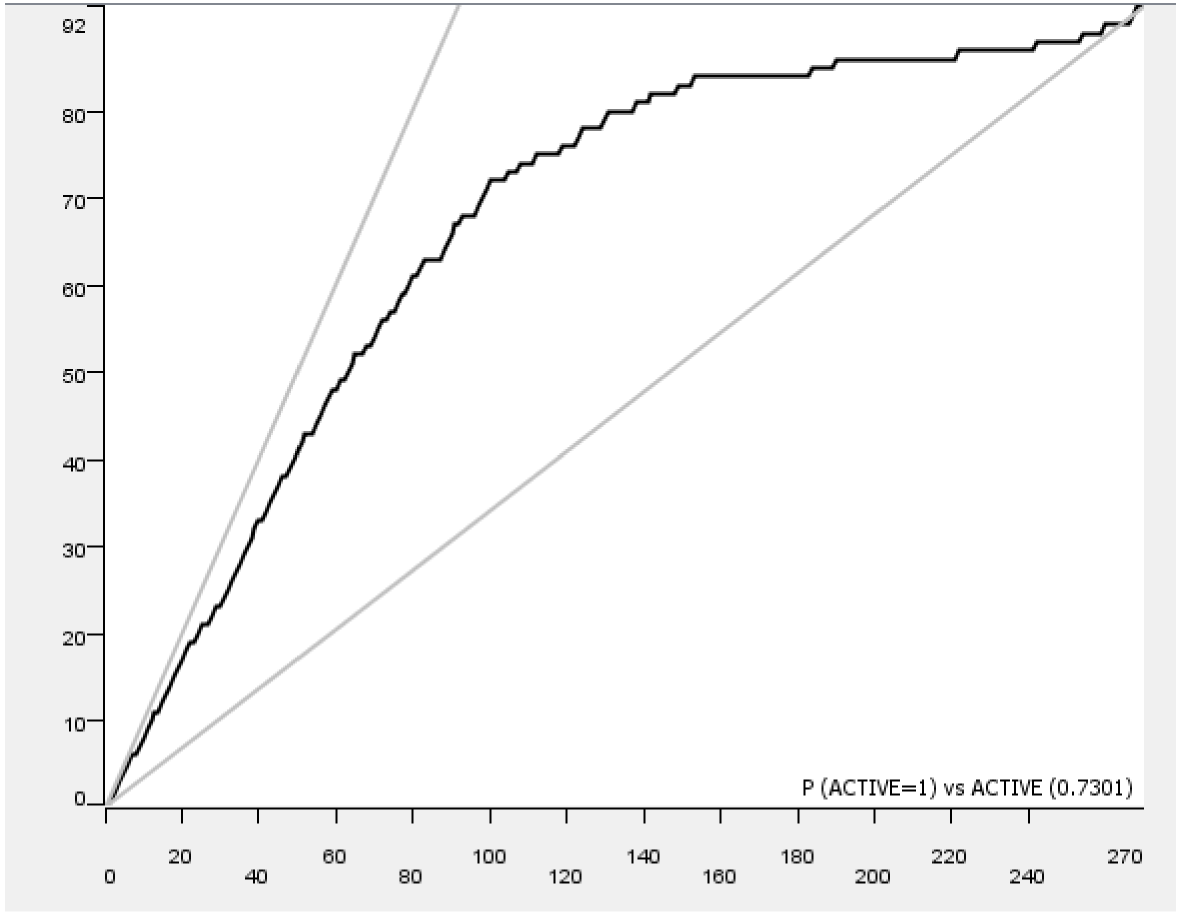

N=270

**Top 10% Mean IC50**	**15.9 nM (2 micromolar mistakes)**

	Predicted Active	Predicted Inactive
Active	63	29
Inactive	22	156

< 20 nM DefGood in ALK, 30% error; Random seed = 121783

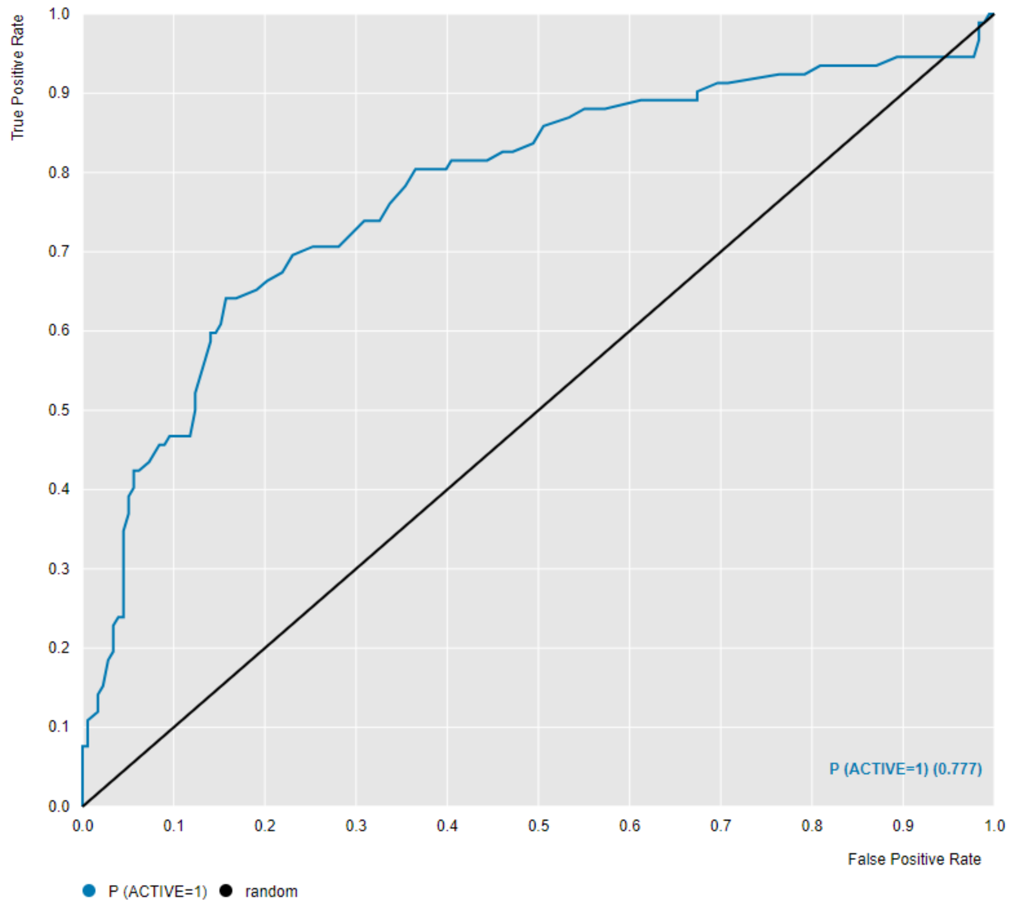

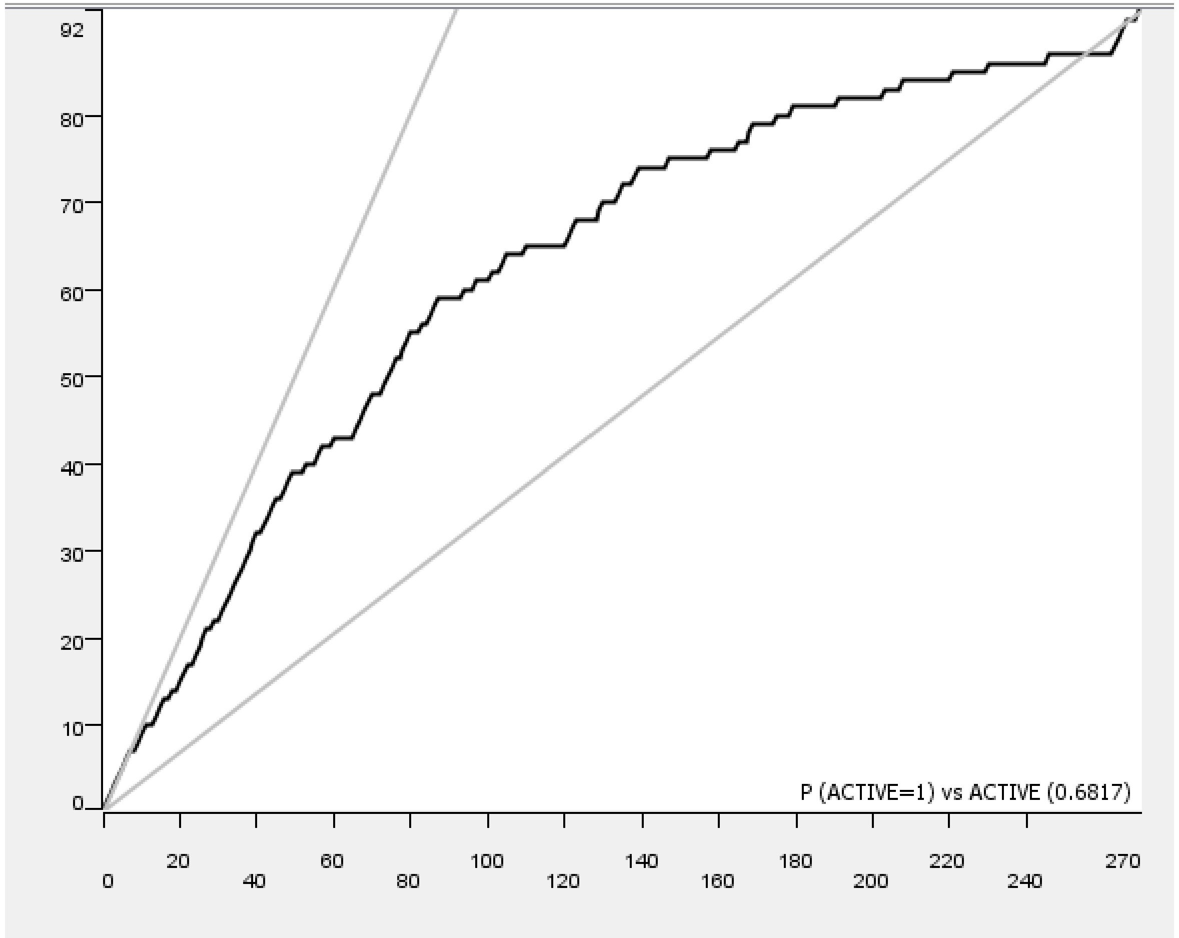

N=270

Top 10% Mean IC50	19.0 nM

	Predicted Active	Predicted Inactive
Active	56	36
Inactive	27	151

< 20 nM DefGood in ALK, 35% error; Random seed = 121783

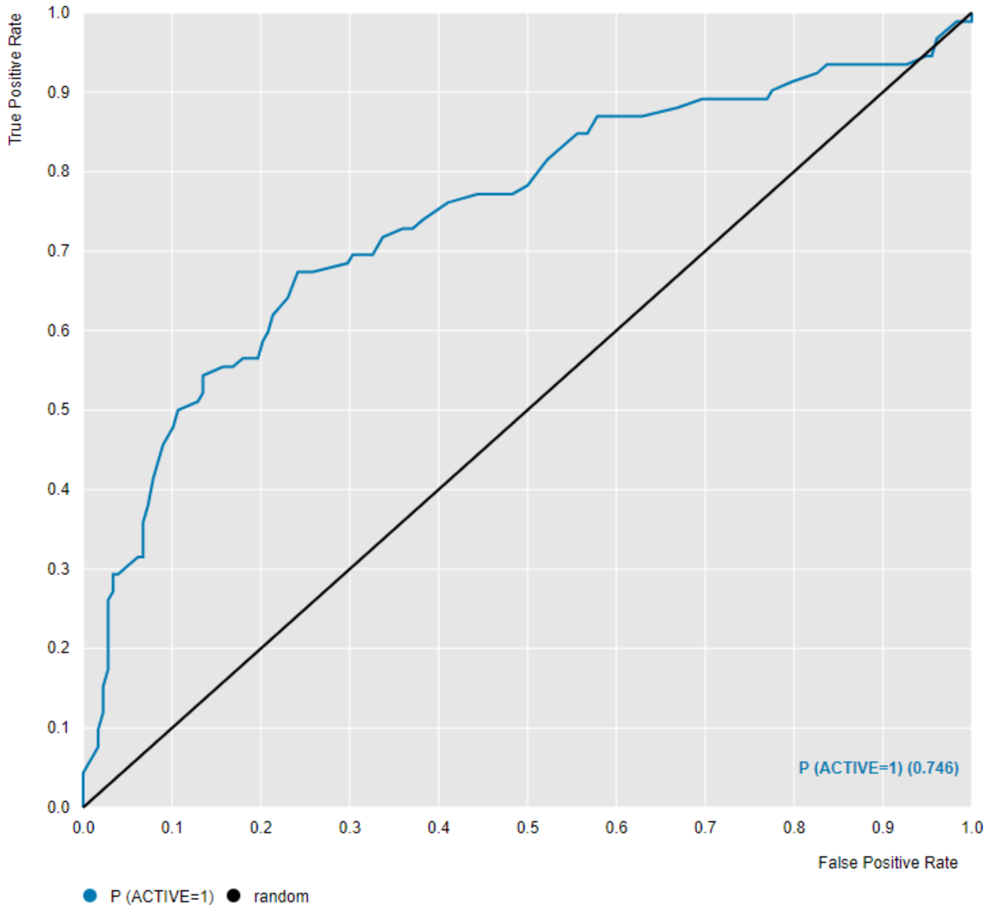

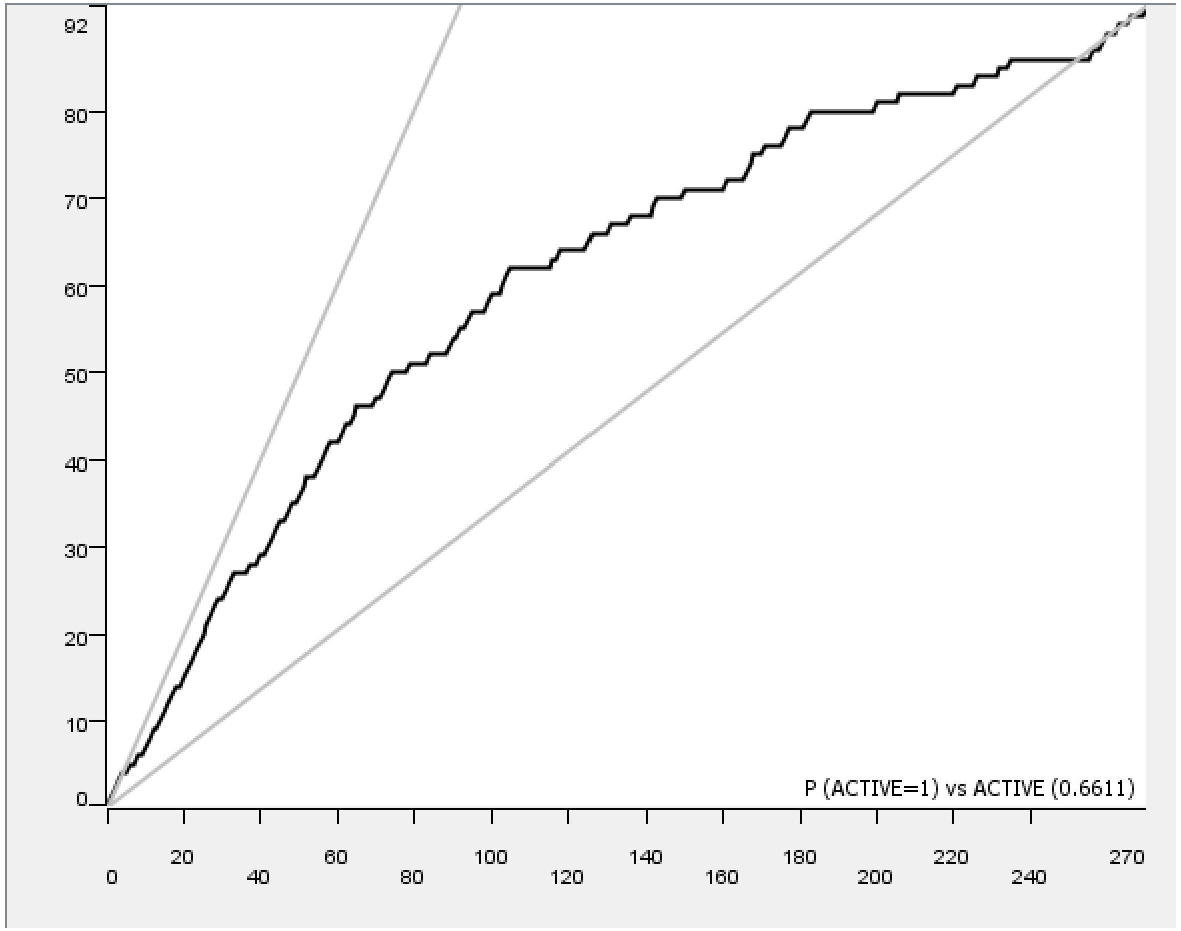

N=270

Top 10% Mean IC50	19.5 nM

	Predicted Active	Predicted Inactive
Active	54	38
Inactive	36	142

< 20 nM DefGood in ALK, 40% error; Random seed = 121783

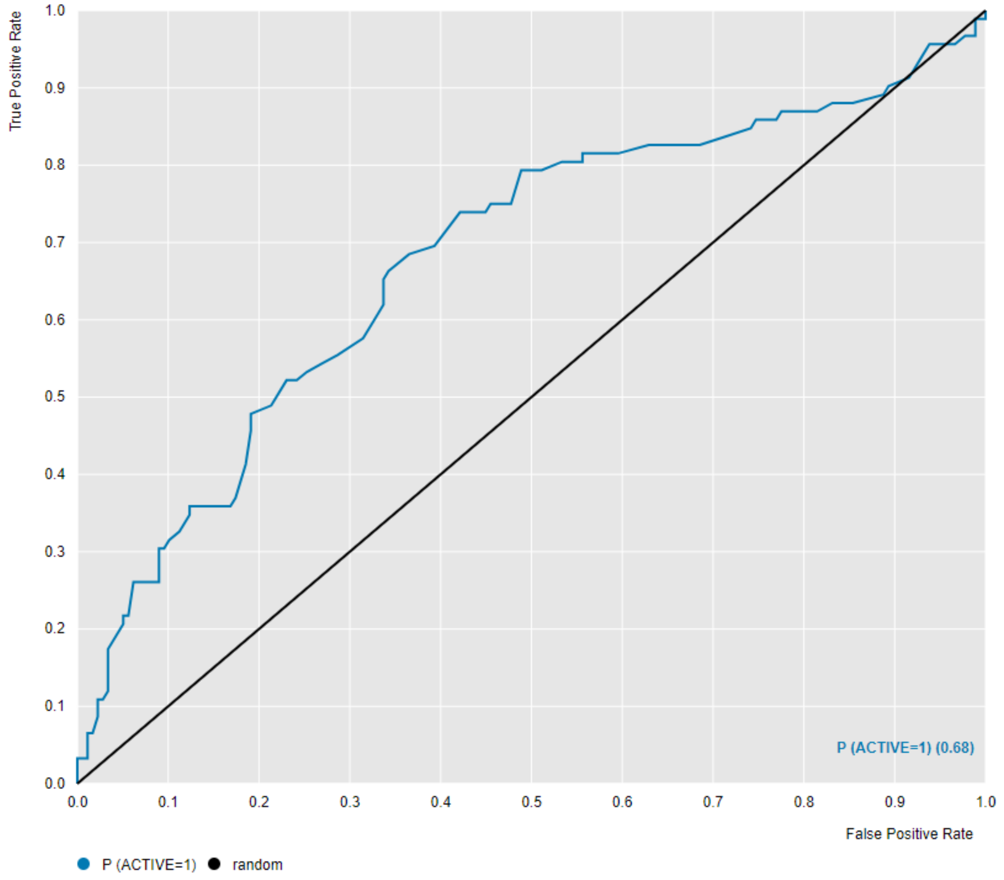

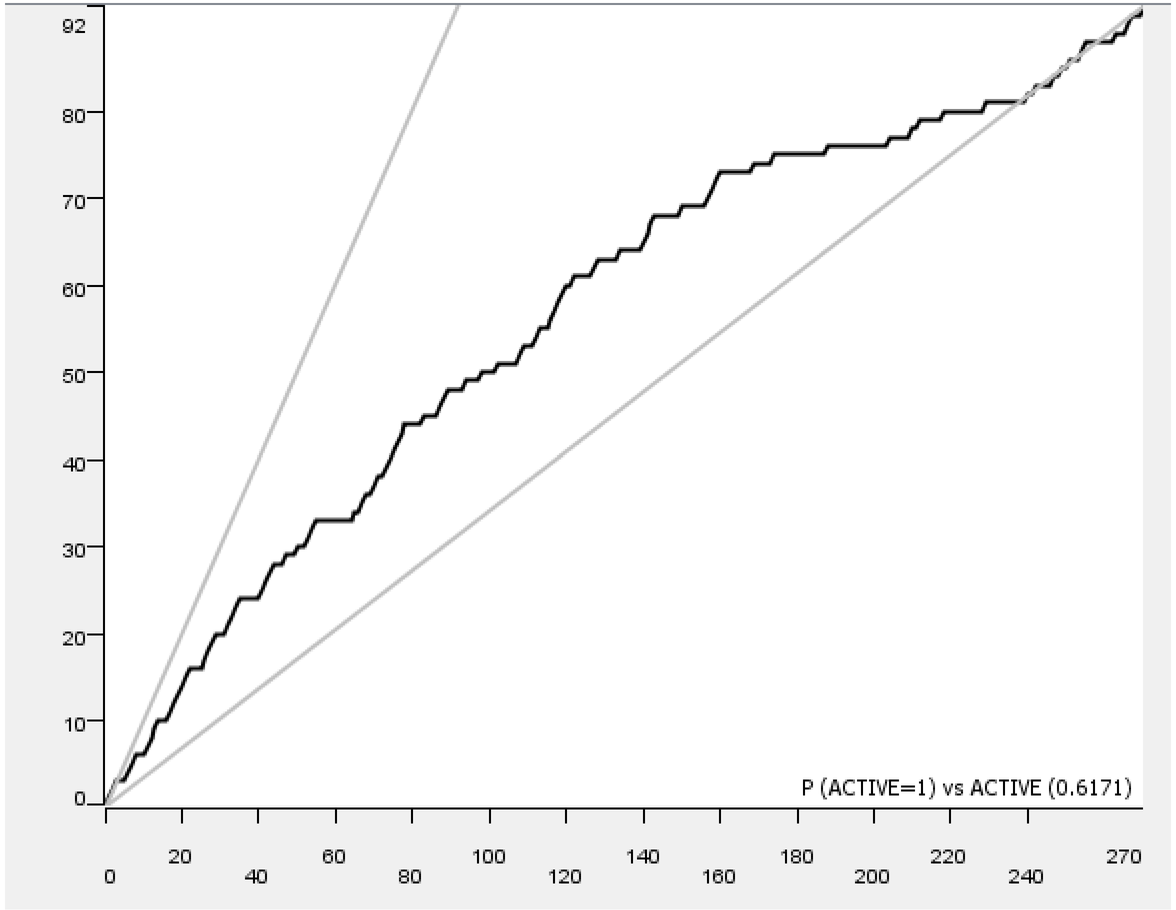

N=270

**Top 10% Mean IC50**	**128 nM**

	Predicted Active	Predicted Inactive
Active	53	39
Inactive	56	122

Conclusion - RF

- A Random Forrest could be generated for ALK with a decision value of <20 nM as defined as active. The predictivity of the algorithm could be preserved in three random train/test cycles as error was introduced in the training set. The point of failure was 30%, 35% and 40% error.

PNN - <20 nM DefGood in ALK, 5% error ; Random seed = 1515533876005

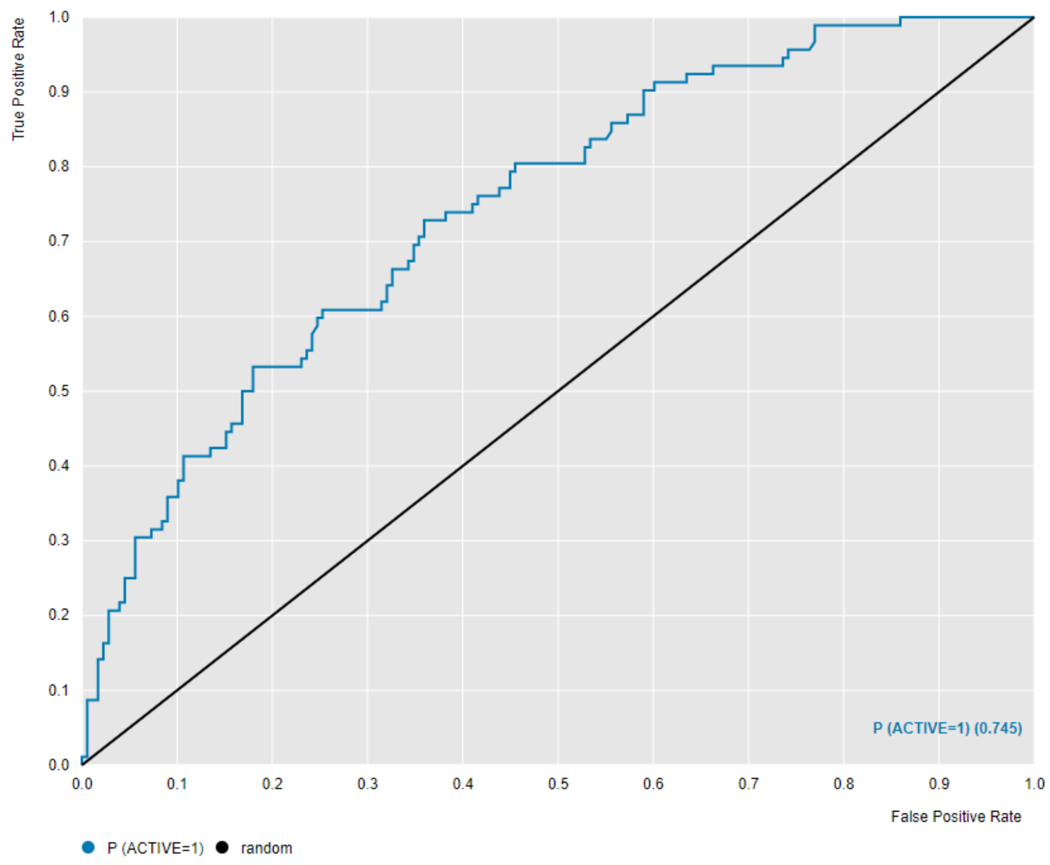

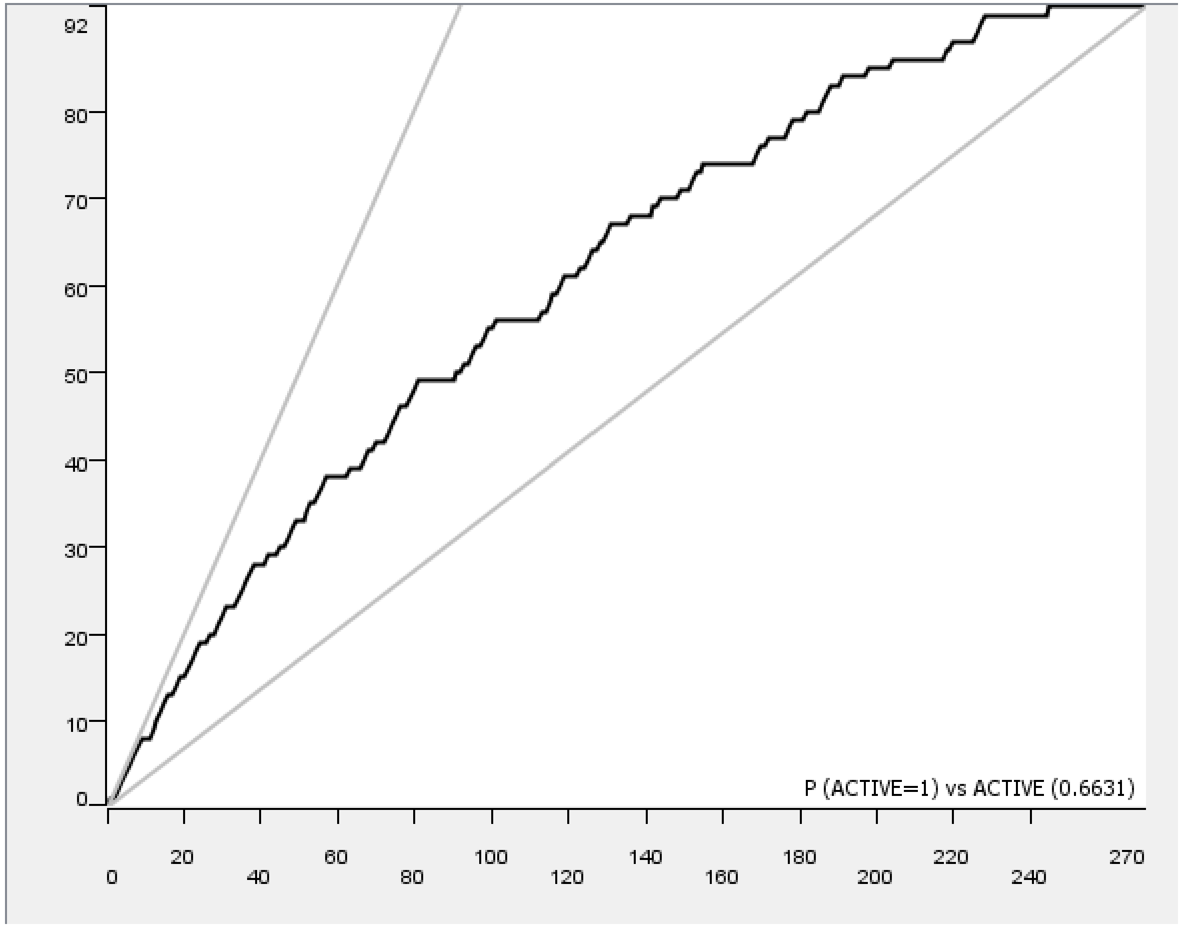

N=270

Top 10% Mean IC50	223 nM

	Predicted Active	Predicted Inactive
Active	49	43
Inactive	33	145

PNN - < 20 nM DefGood in ALK, 10% error

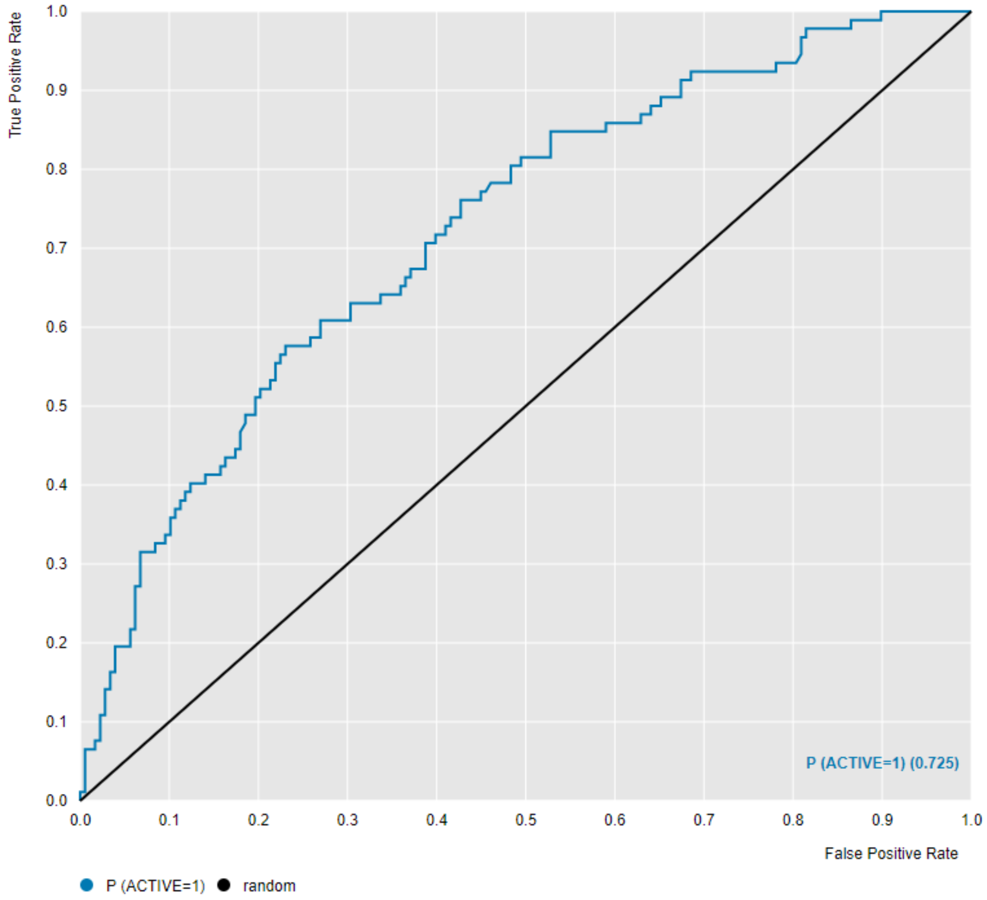

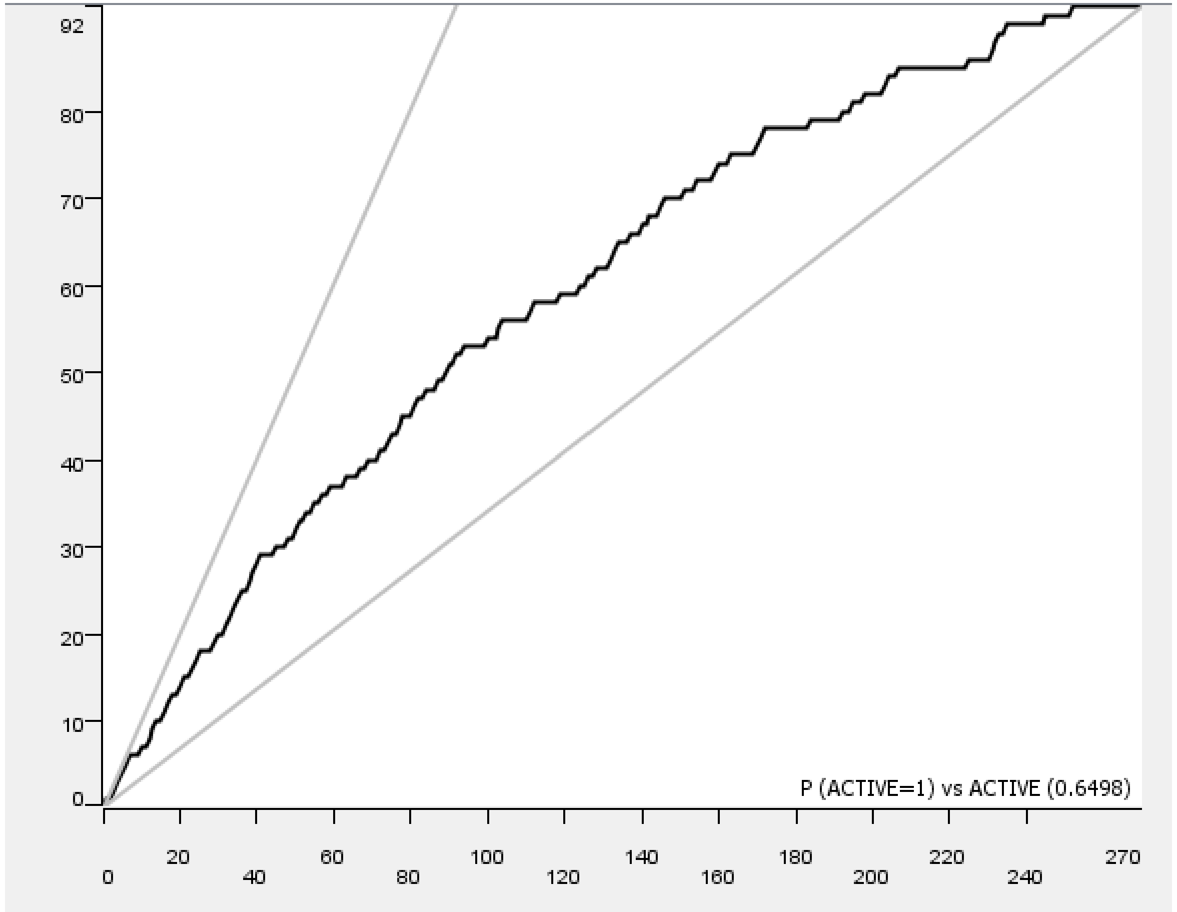

N=270

Top 10% Mean IC50	440 nM

	Predicted Active	Predicted Inactive
Active	51	41
Inactive	40	138

PNN - < 20 nM DefGood in ALK, 15% error

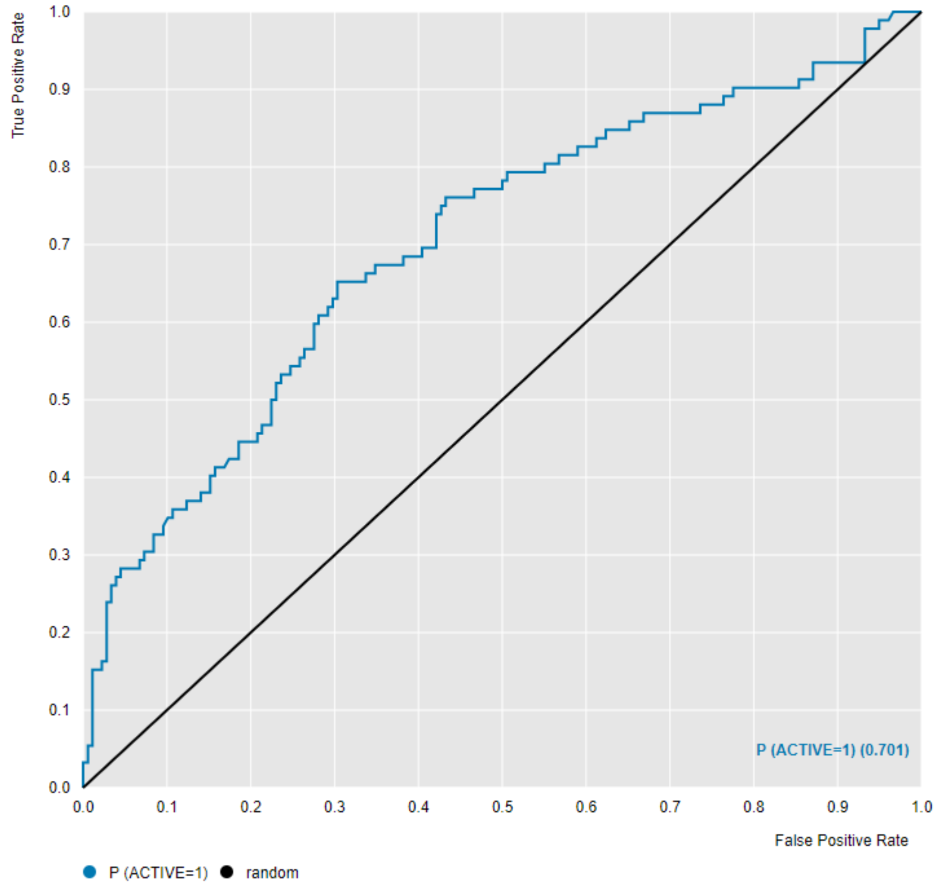

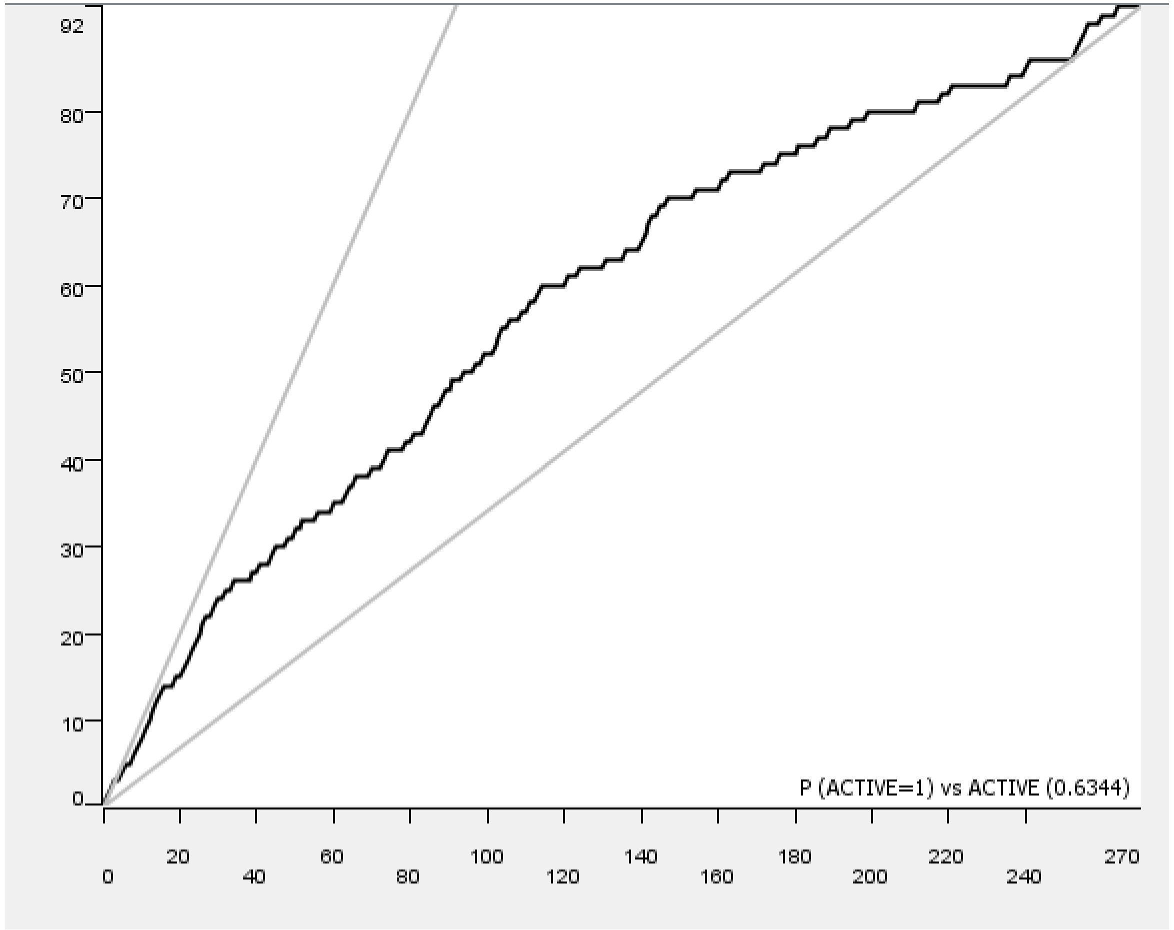

N=270

**Top 10% Mean IC50**	**14 nM**

	Predicted Active	Predicted Inactive
Active	33	59
Inactive	22	156

PNN - < 20 nM DefGood in ALK, 20% error

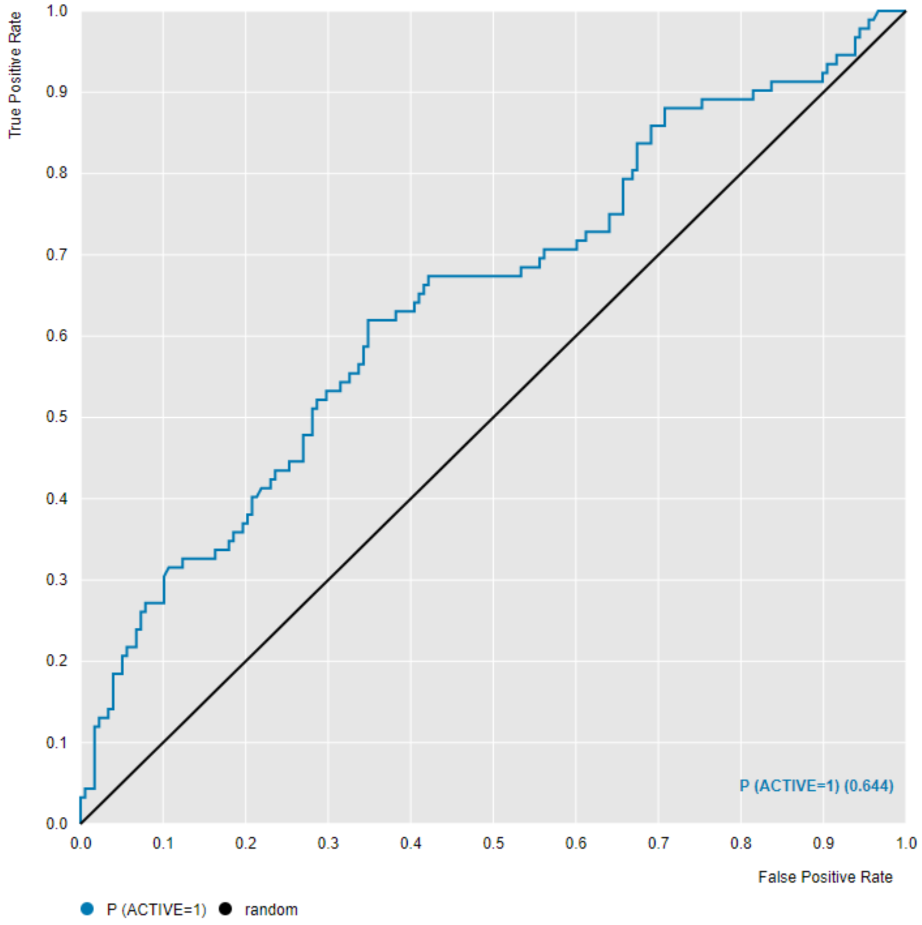

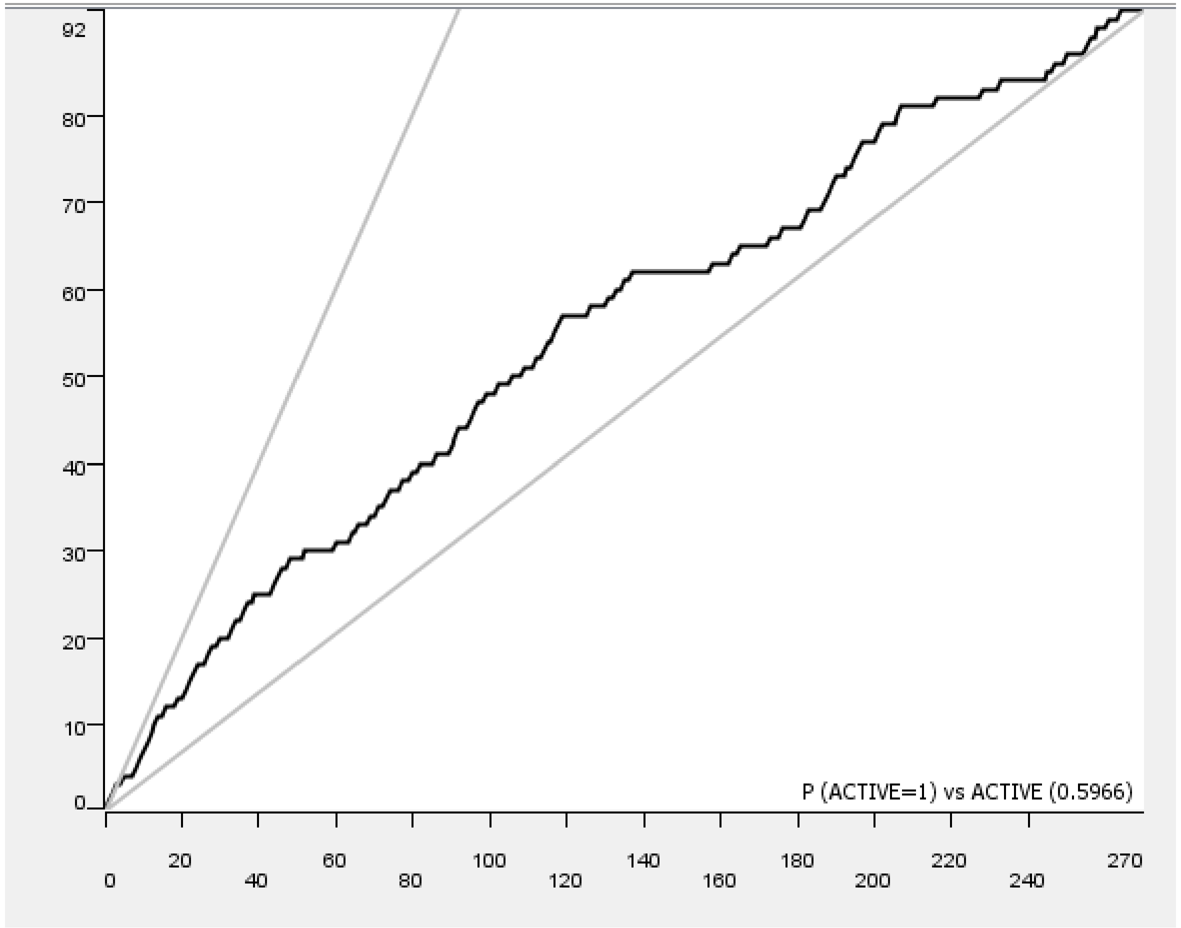

N=270

**Top 10% Mean IC50**	**1,400 nM**

	Predicted Active	Predicted Inactive
Active	32	60
Inactive	33	145

PNN - < 20 nM DefGood in ALK, 20% error (reparametrized; (0.95, 0.85) (Theta minus and plus)

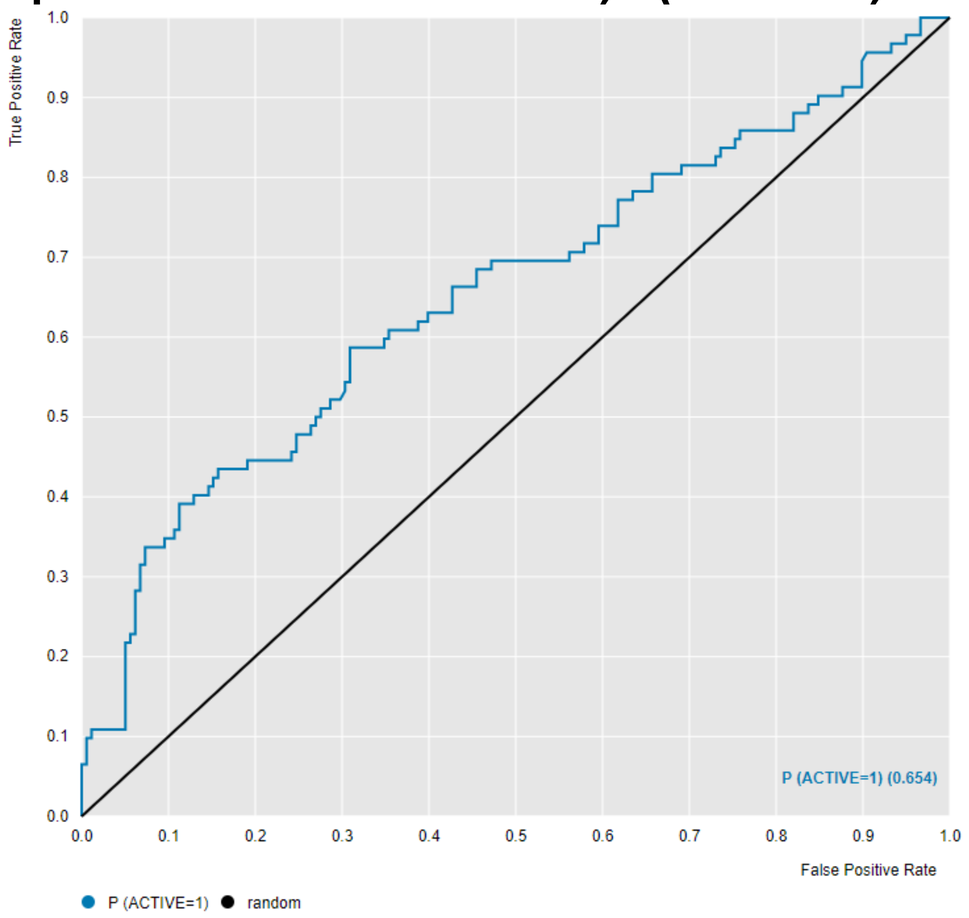

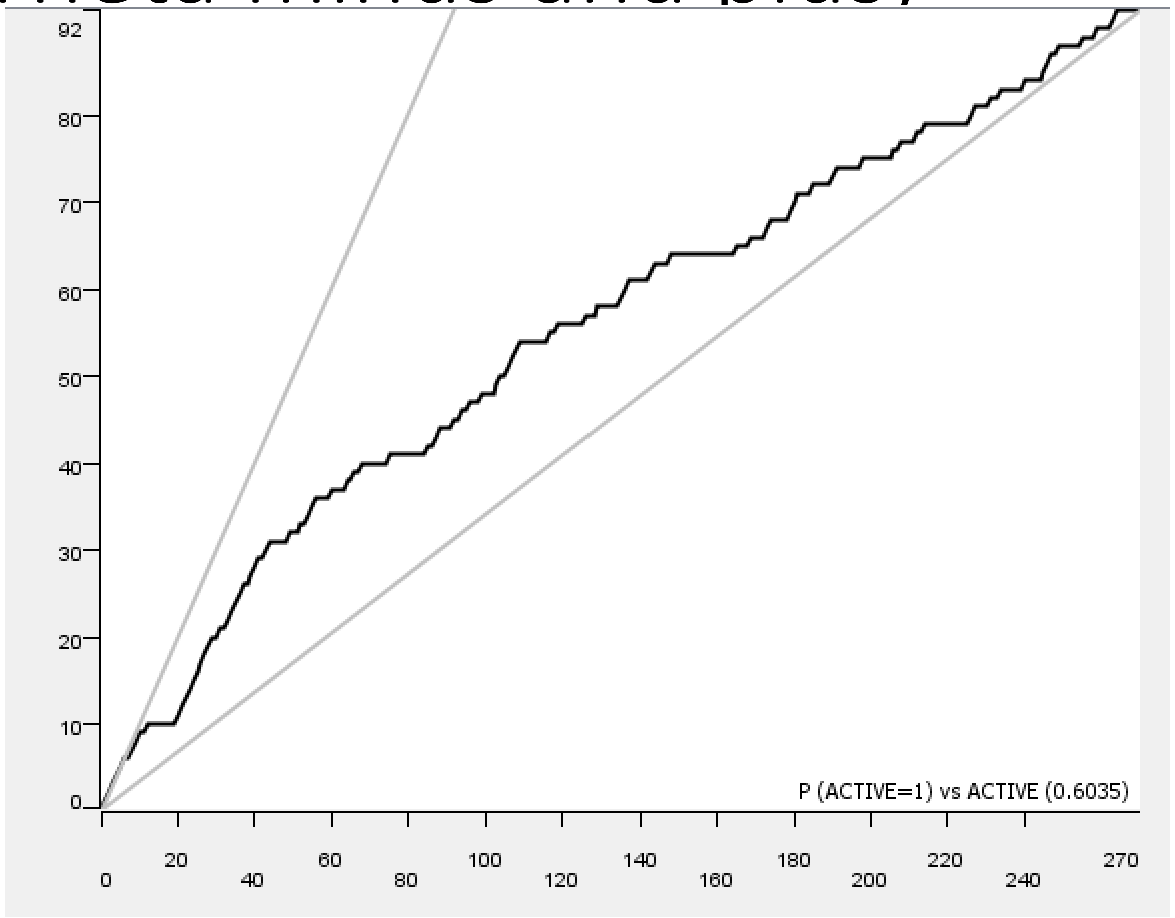

N=270

Top 10% Mean IC50	779 nM

	Predicted Active	Predicted Inactive
Active	25	67
Inactive	11	167

PNN - < 20 nM DefGood in ALK, 15% error; Random seed = 429

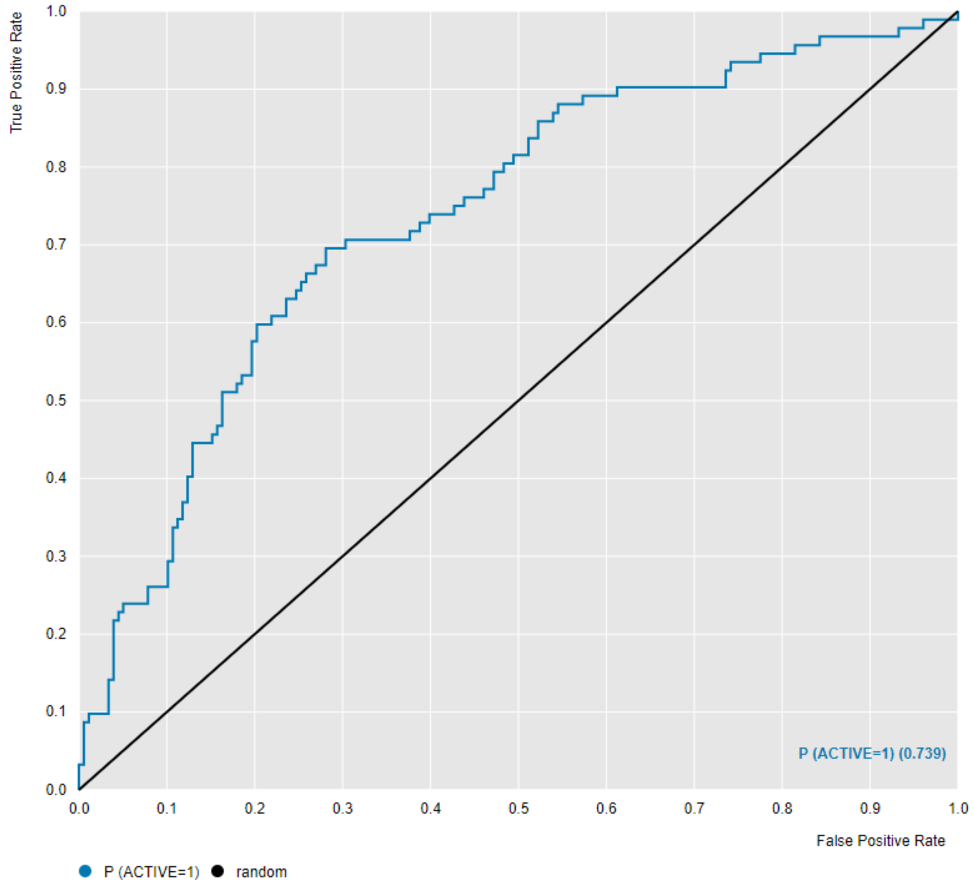

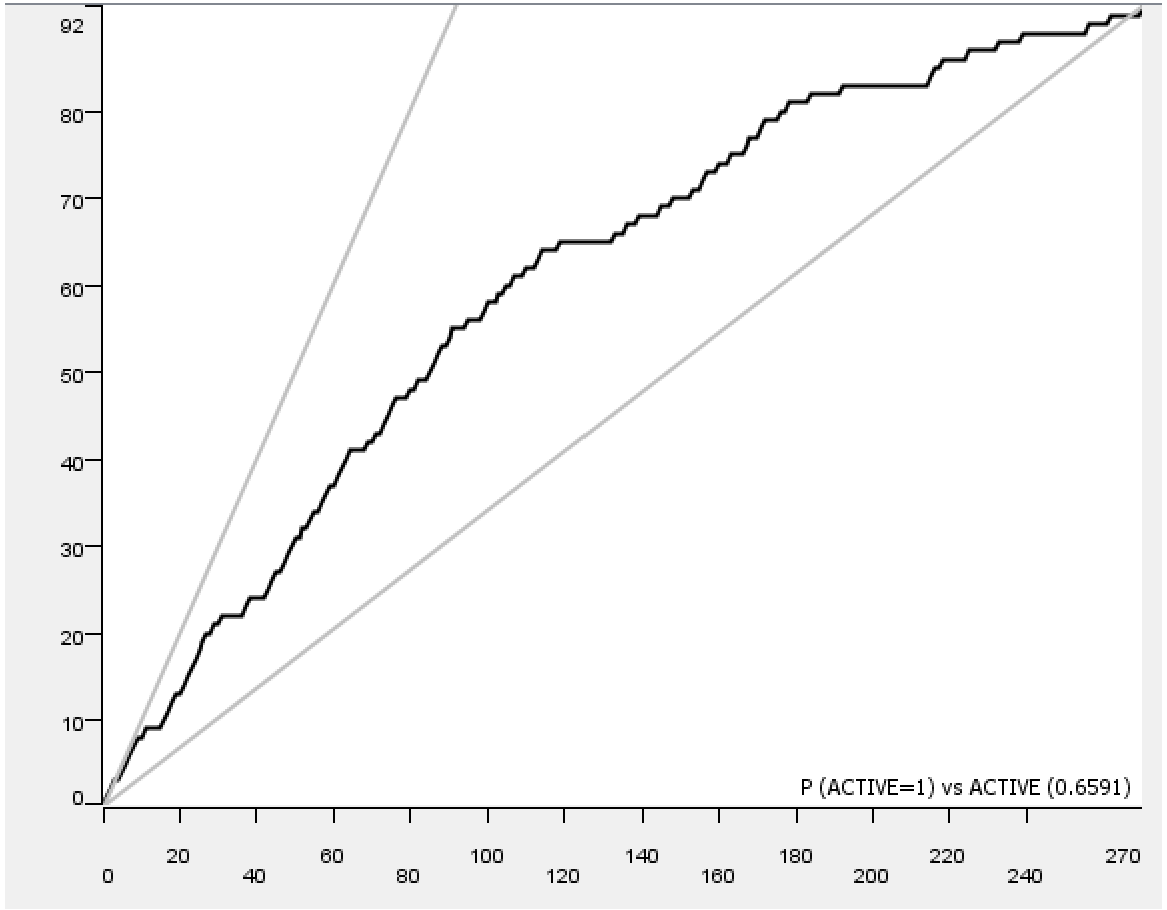

N=270

Top 10% Mean IC50	552 nM

	Predicted Active	Predicted Inactive
Active	45	47
Inactive	29	149

PNN - < 20 nM DefGood in ALK, 20% error ; Random seed = 429

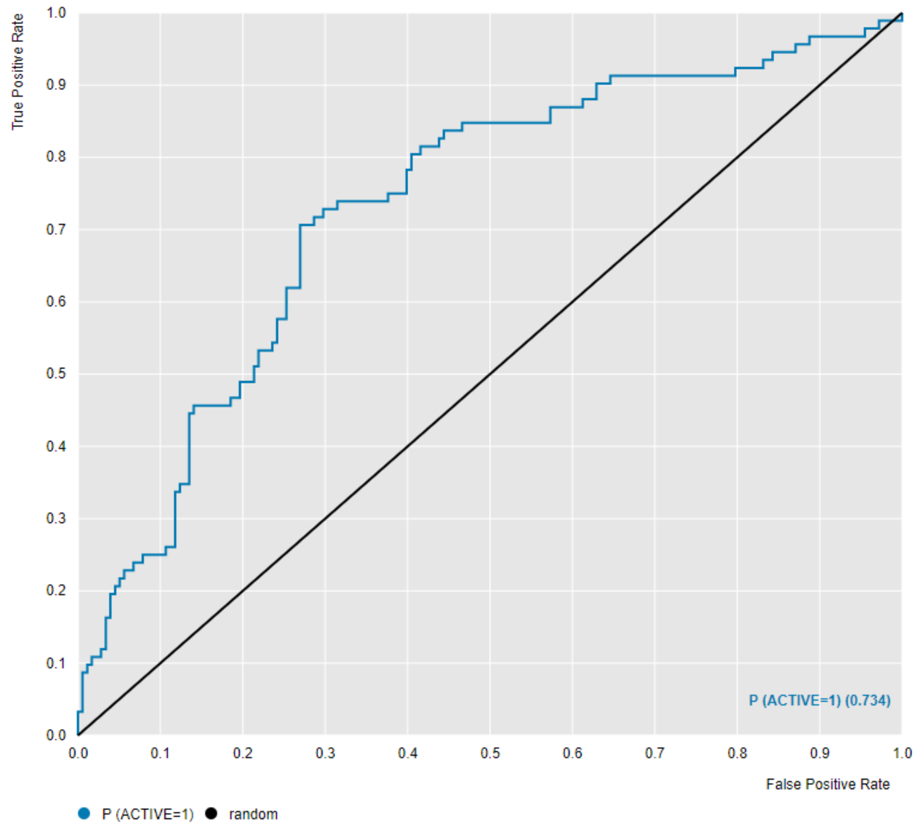

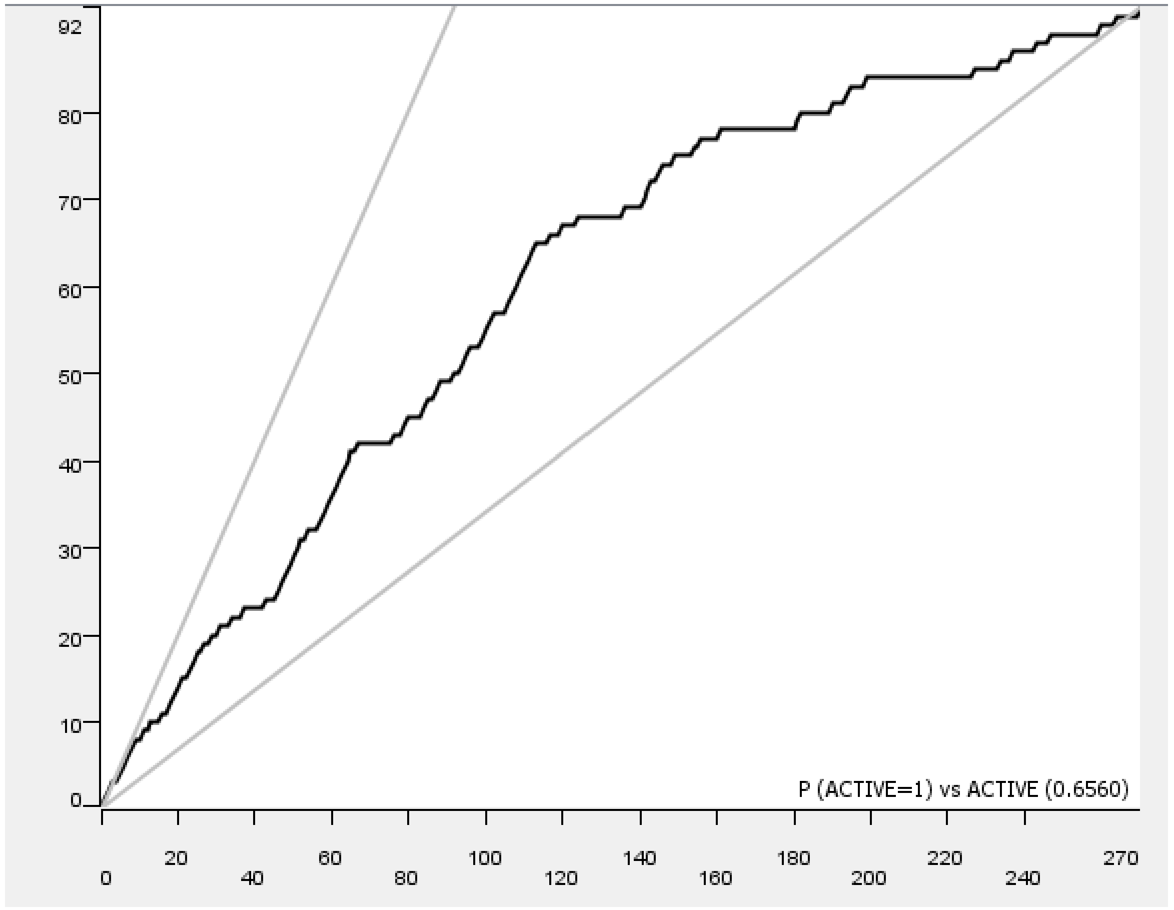

N=270

Top 10% Mean IC50	600 nM

	Predicted Active	Predicted Inactive
Active	45	47
Inactive	35	143

PNN - < 20 nM DefGood in ALK, 25% error ; Random seed = 429

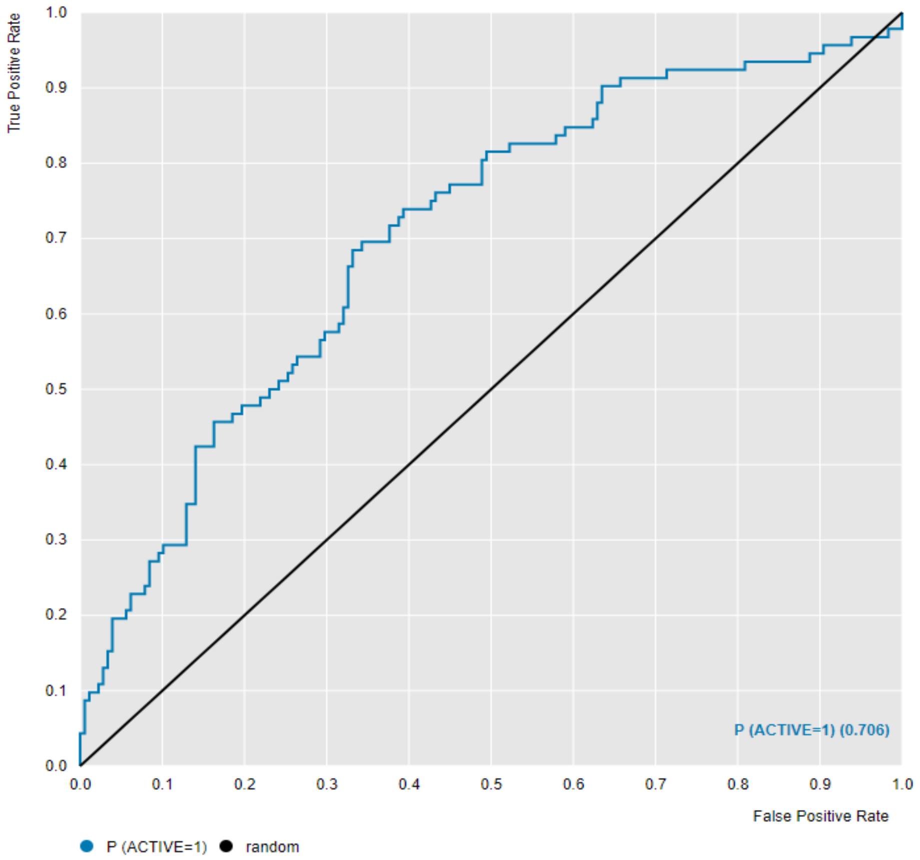

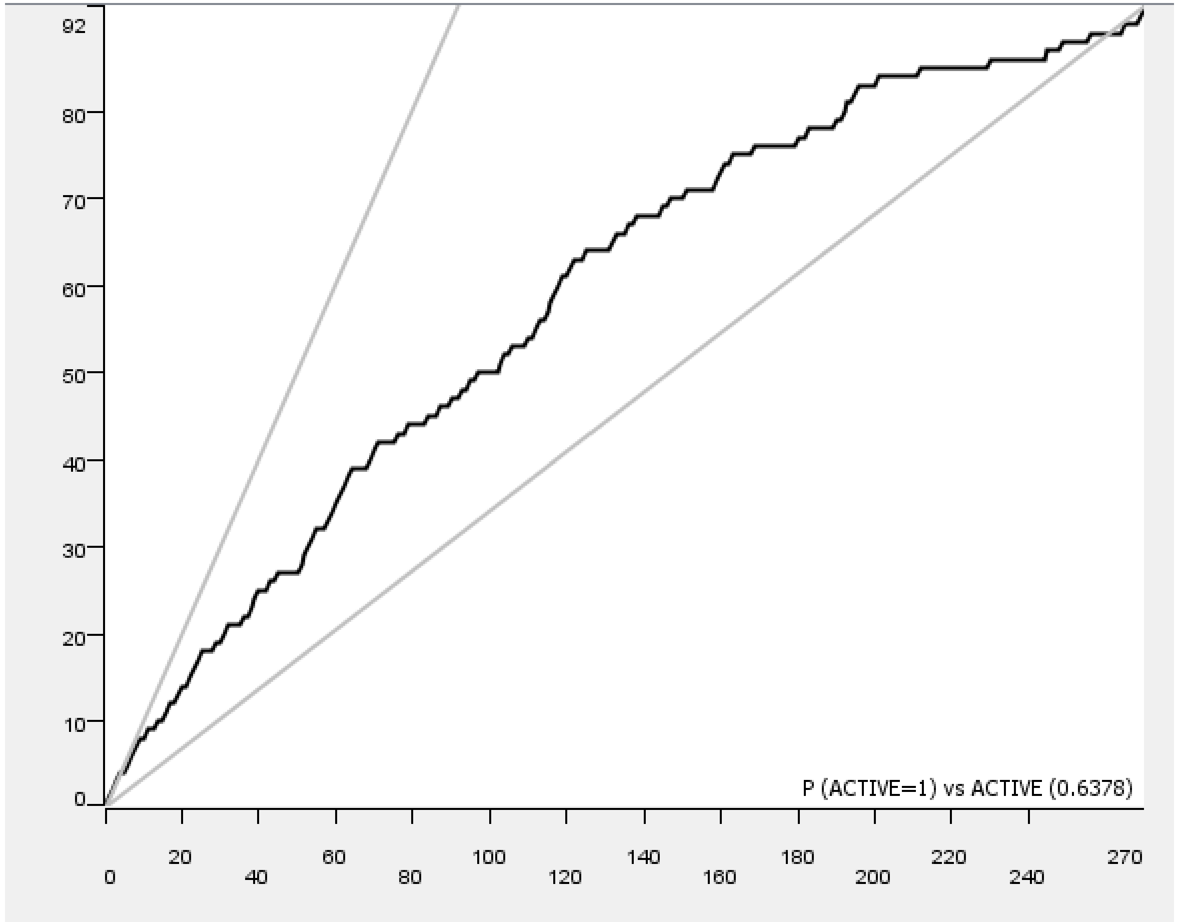

N=270

**Top 10% Mean IC50**	**637 nM**

	Predicted Active	Predicted Inactive
Active	46	46
Inactive	41	137

PNN - < 20 nM DefGood in ALK, 30% error ; Random seed = 429

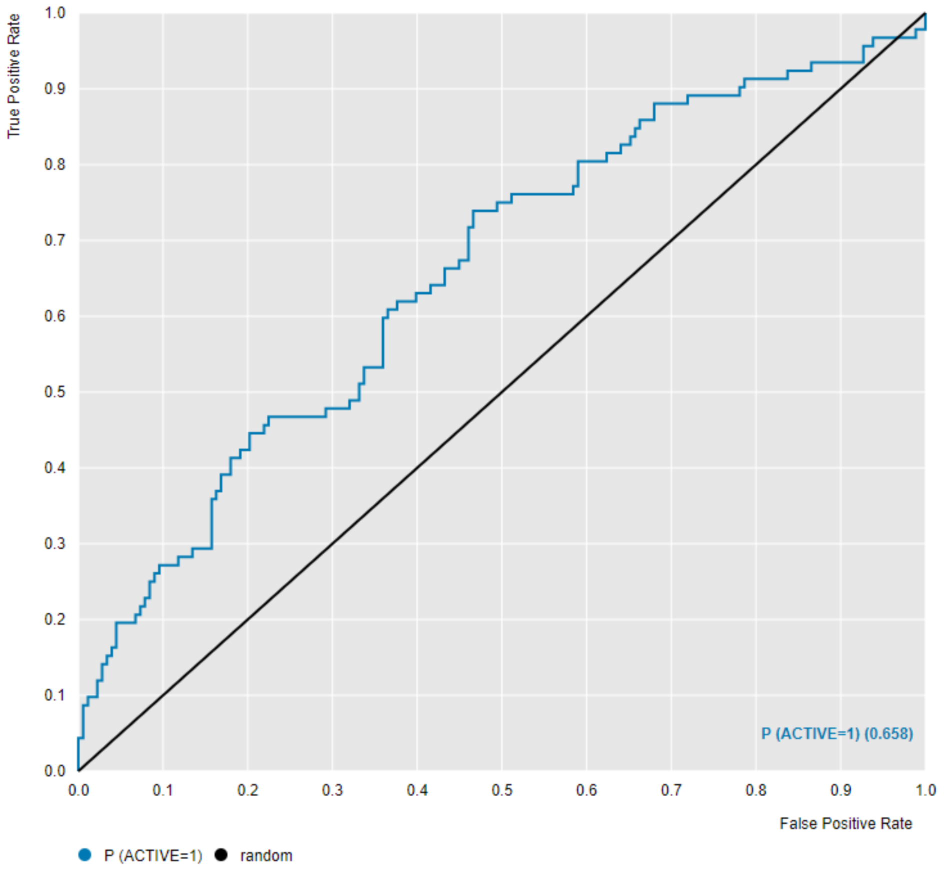

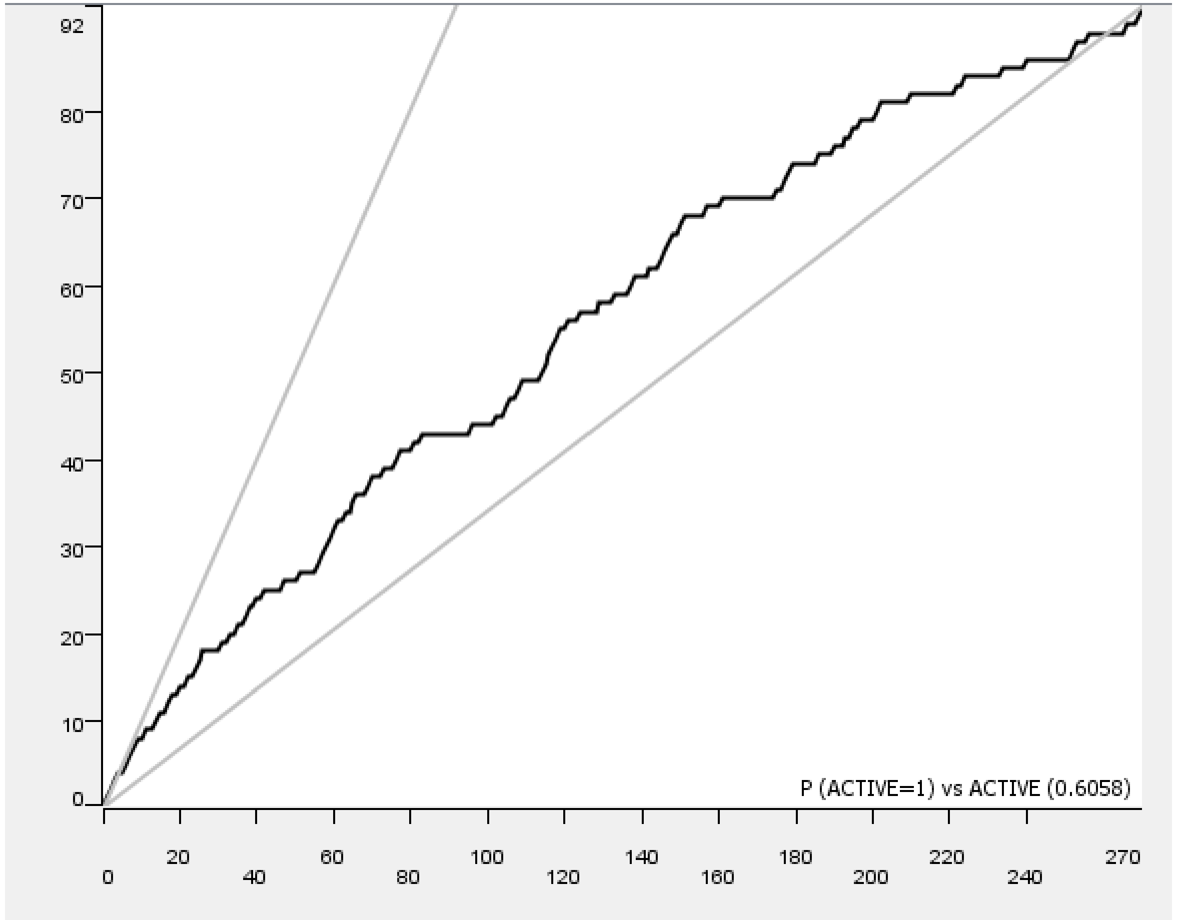

N=270

Top 10% Mean IC50	680 nM

	Predicted Active	Predicted Inactive
Active	44	48
Inactive	55	123

PNN - < 20 nM DefGood in ALK, 30% error ; Random seed = 429 (reparametrized) Theta minus = 0.75; Theta plus = 0.65

N=270

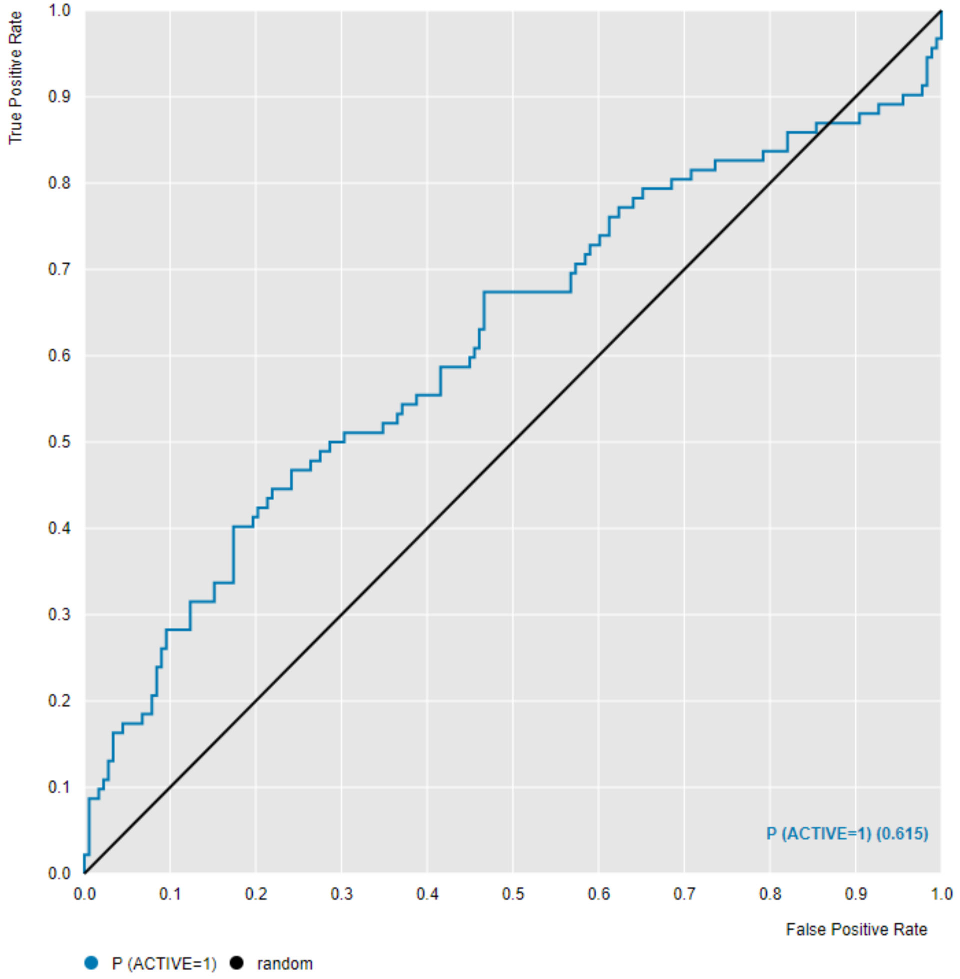

Top 10% Mean IC50	320 nM

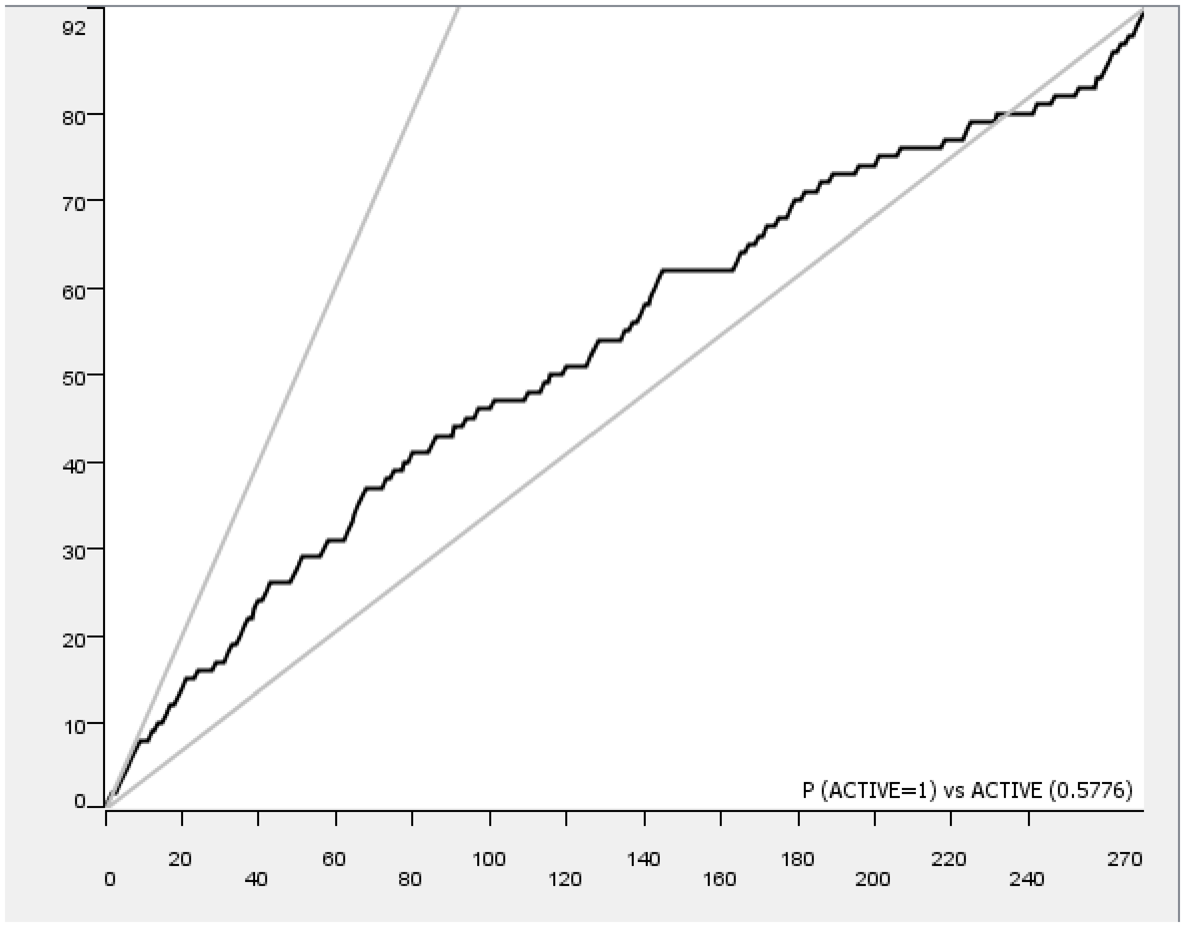

	Predicted Active	Predicted Inactive
Active	37	55
Inactive	32	146

PNN - < 20 nM DefGood in ALK, 10% error; Random seed = 121783

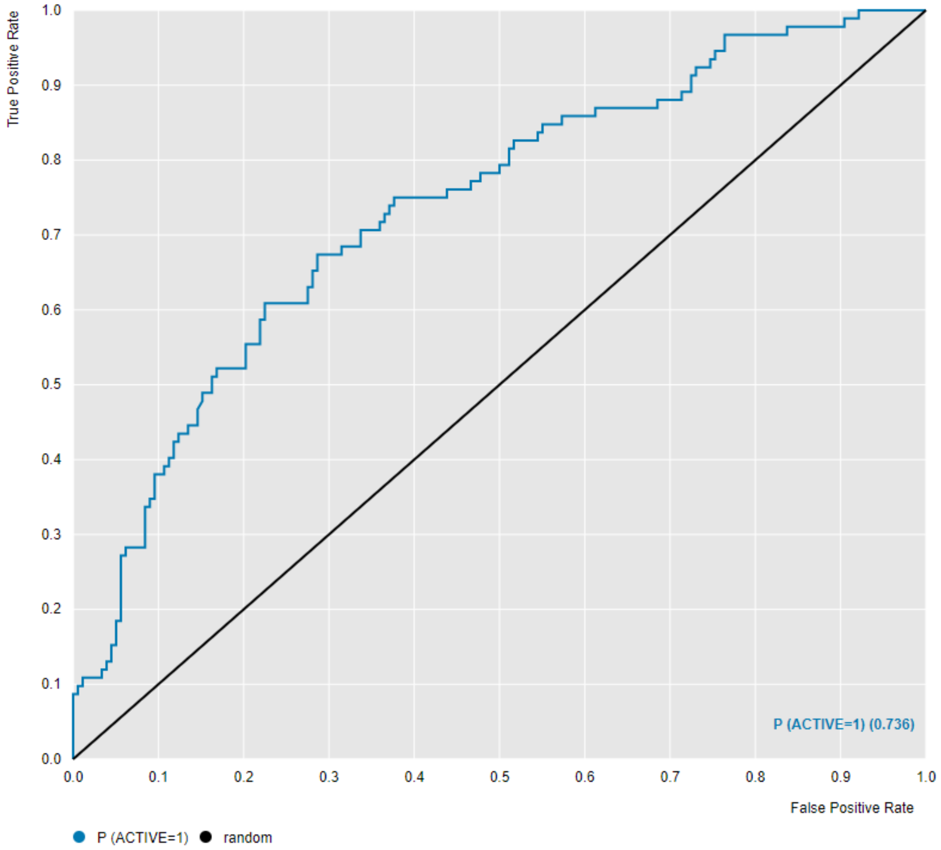

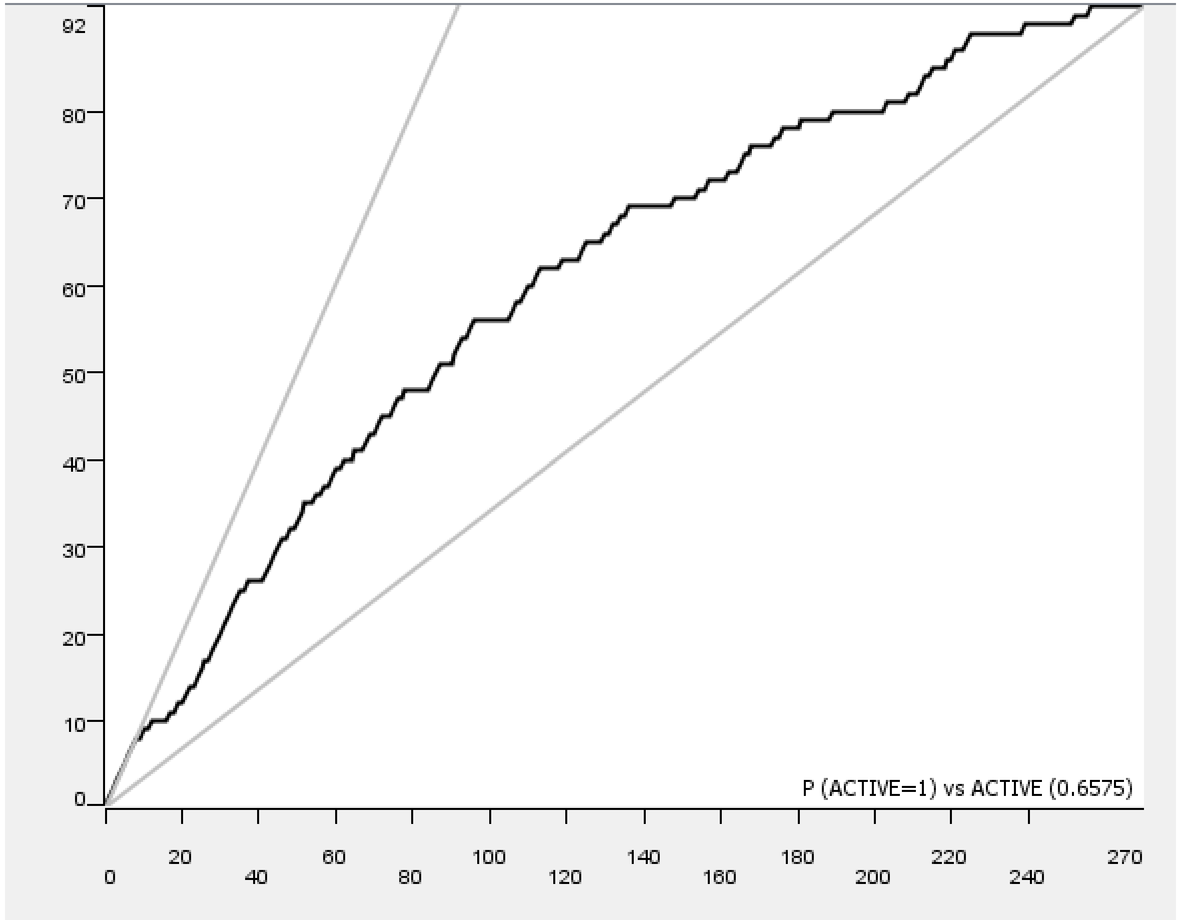

N=270

Top 10% Mean IC50	375 nM

	Predicted Active	Predicted Inactive
Active	44	48
Inactive	27	151

PNN - < 20 nM DefGood in ALK, 15% error; Random seed = 121783

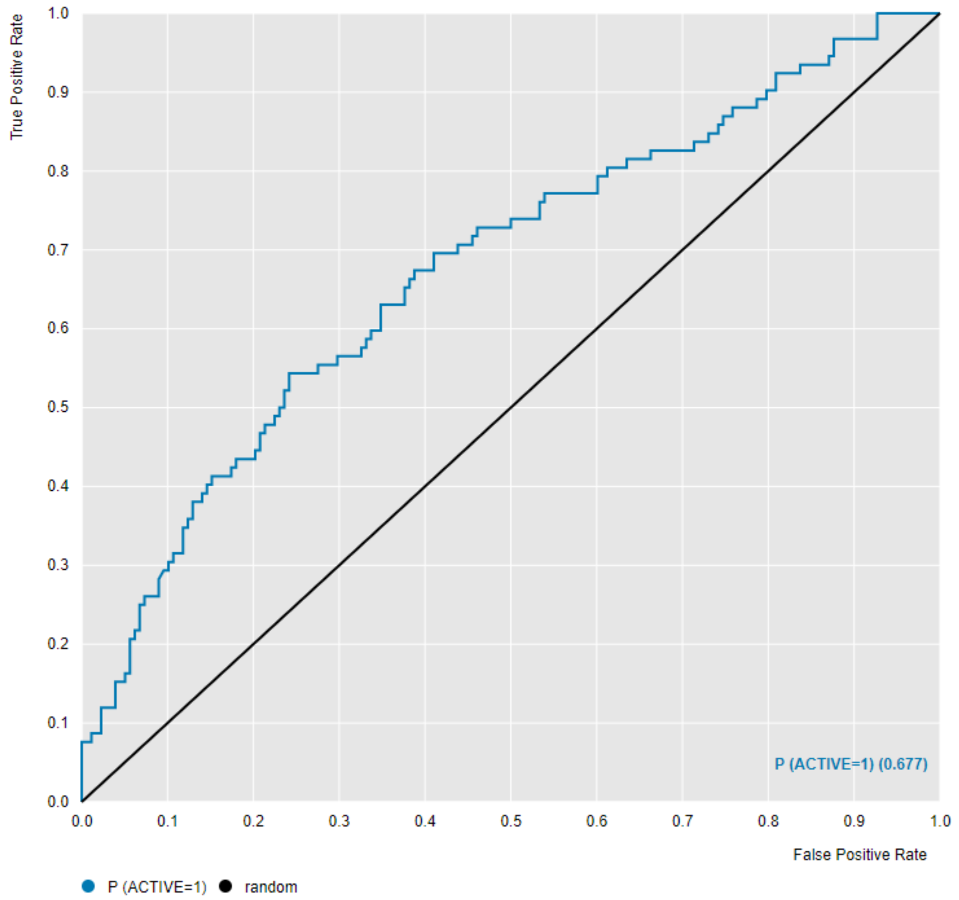

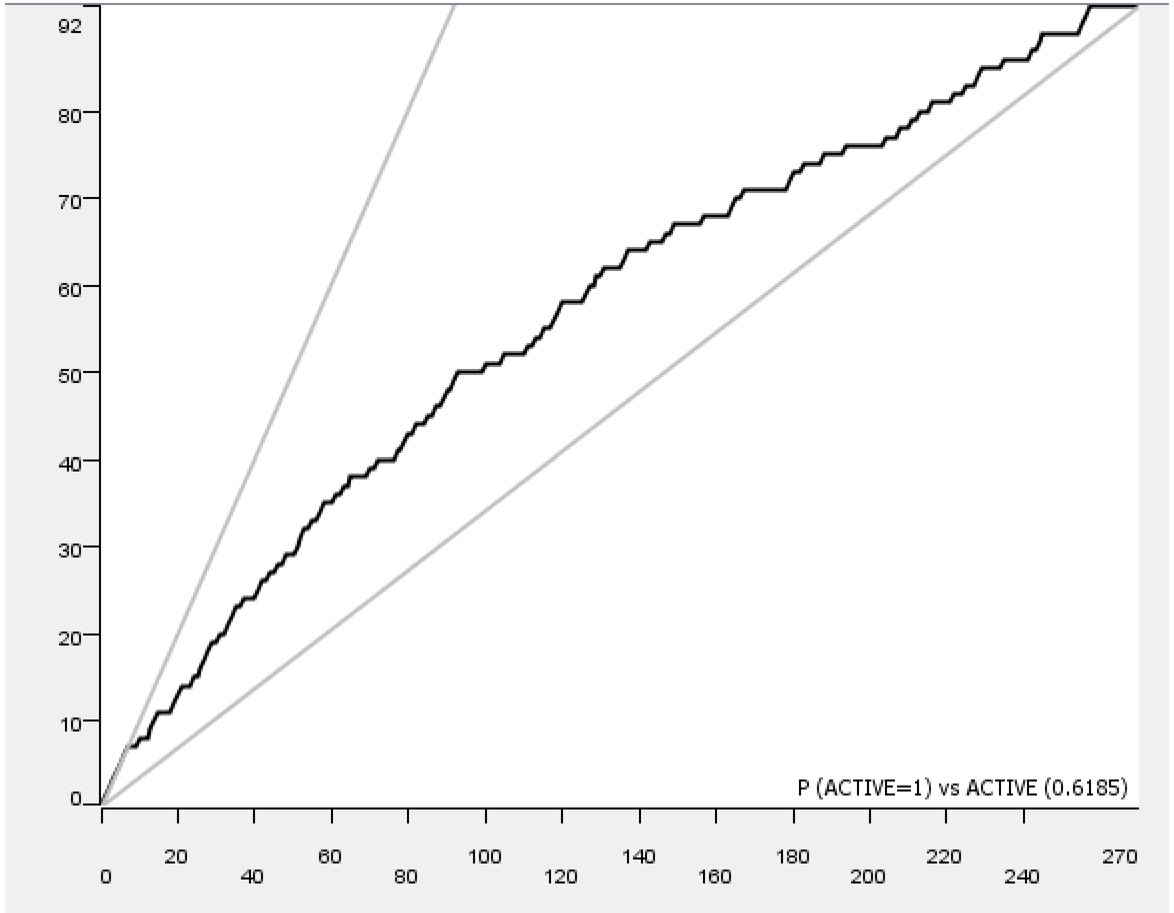

N=270

Top 10% Mean IC50	115 nM

	Predicted Active	Predicted Inactive
Active	37	55
Inactive	26	152

PNN - < 20 nM DefGood in ALK, 20% error; Random seed = 121783

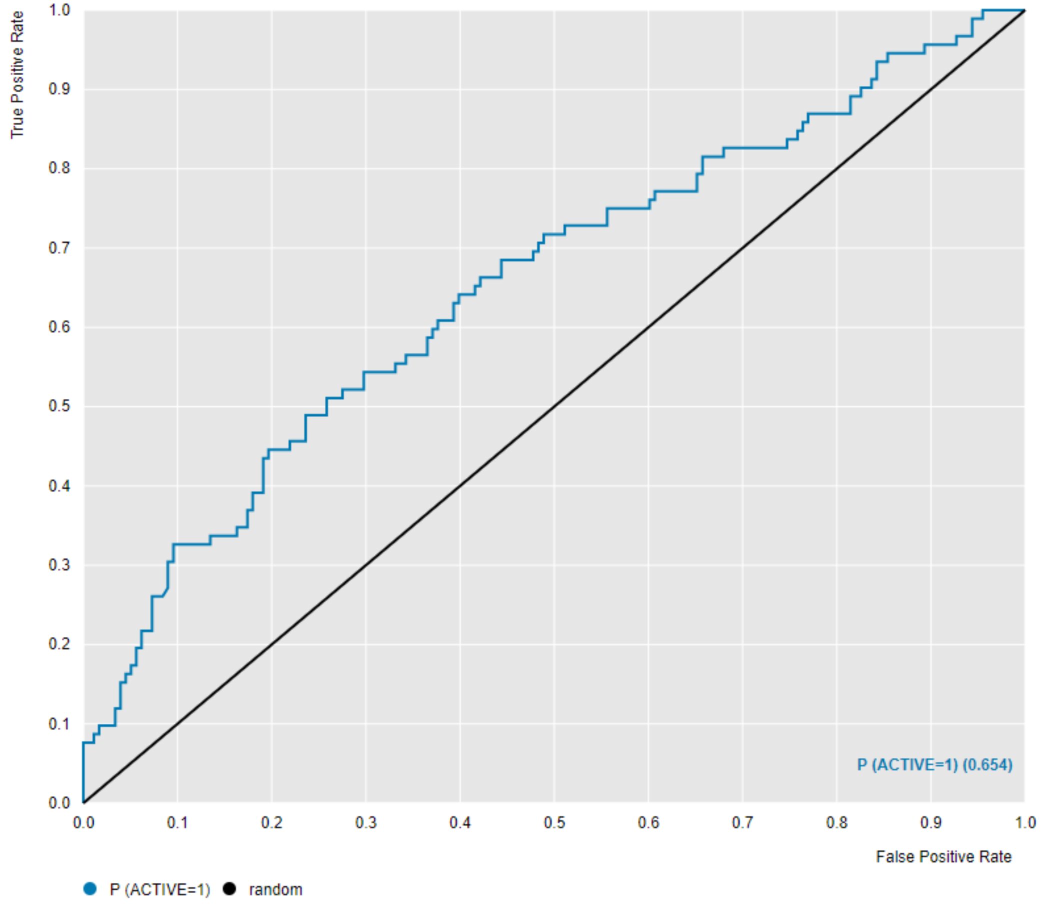

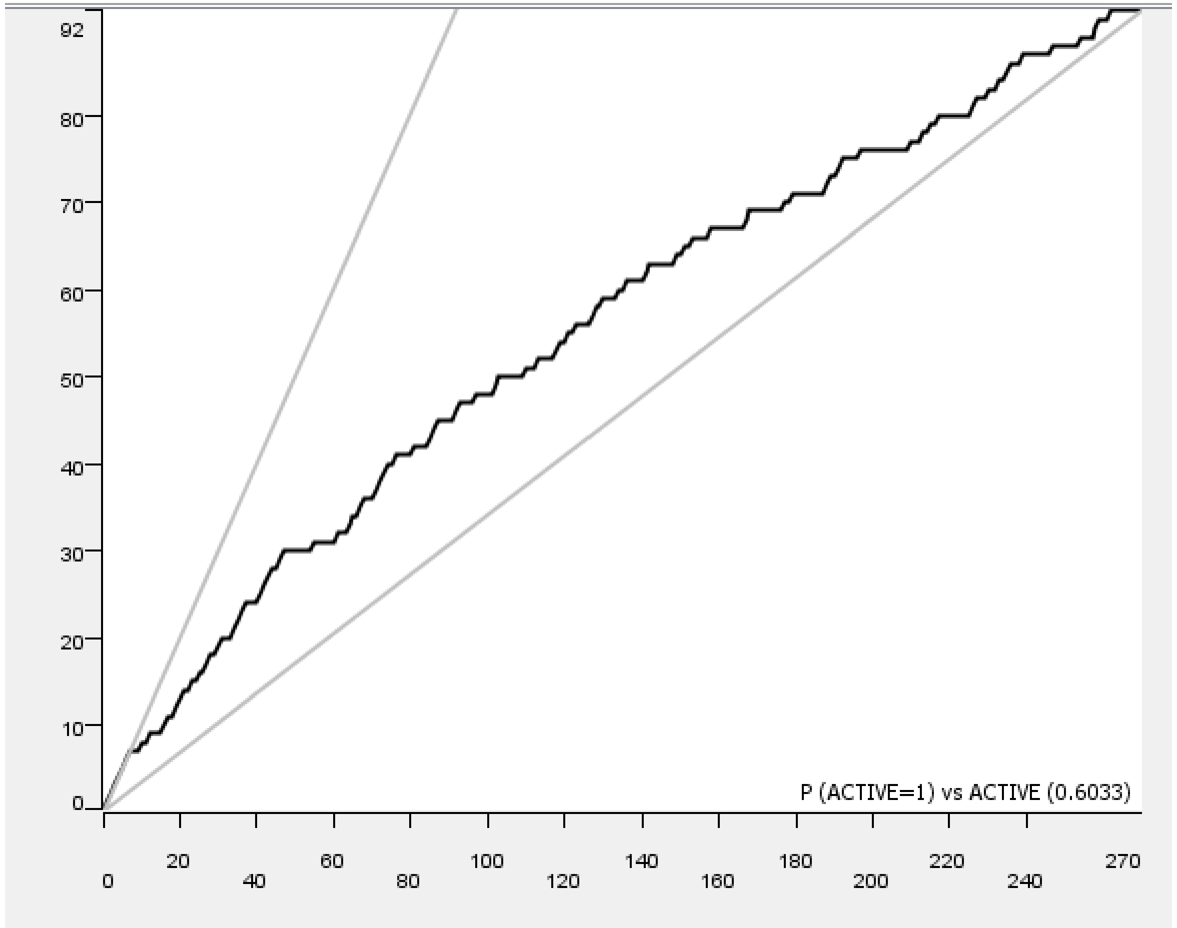

N=270

Top 10% Mean IC50	115 nM

	Predicted Active	Predicted Inactive
Active	34	58
Inactive	31	147

Conclusion - PNN

- Across three random seeds, the % error that lead to a significant enrichment failure and/or top 10% IC50 increase in the retrospective test was 20%, 30% and 15% for each unique random seed.
- Finally, reparameterization needs to be undertaken with each dataset. It did not significantly alter the outcomes of when failure was encountered as a result of error. In the future, a model will be parameterized on the original dataset and unaltered for the error experiment.

Aurora B Kinase

Aurora B Kinase NBN Design

- Rules - $RELATION$ = "=" OR $IC50$ > 999 OR $IC50$ < 10 => TRUE
- Sort ascending and remove duplicates
- DefGood scanned between 5 and 25 nM
- 80-20 split for both actives and inactives
- NBN – ECFP4 used Classifier where Prediction was Active = True
- Resulted in 1481 compounds after cleaning the data

<5 nM DefGood in Aurora B

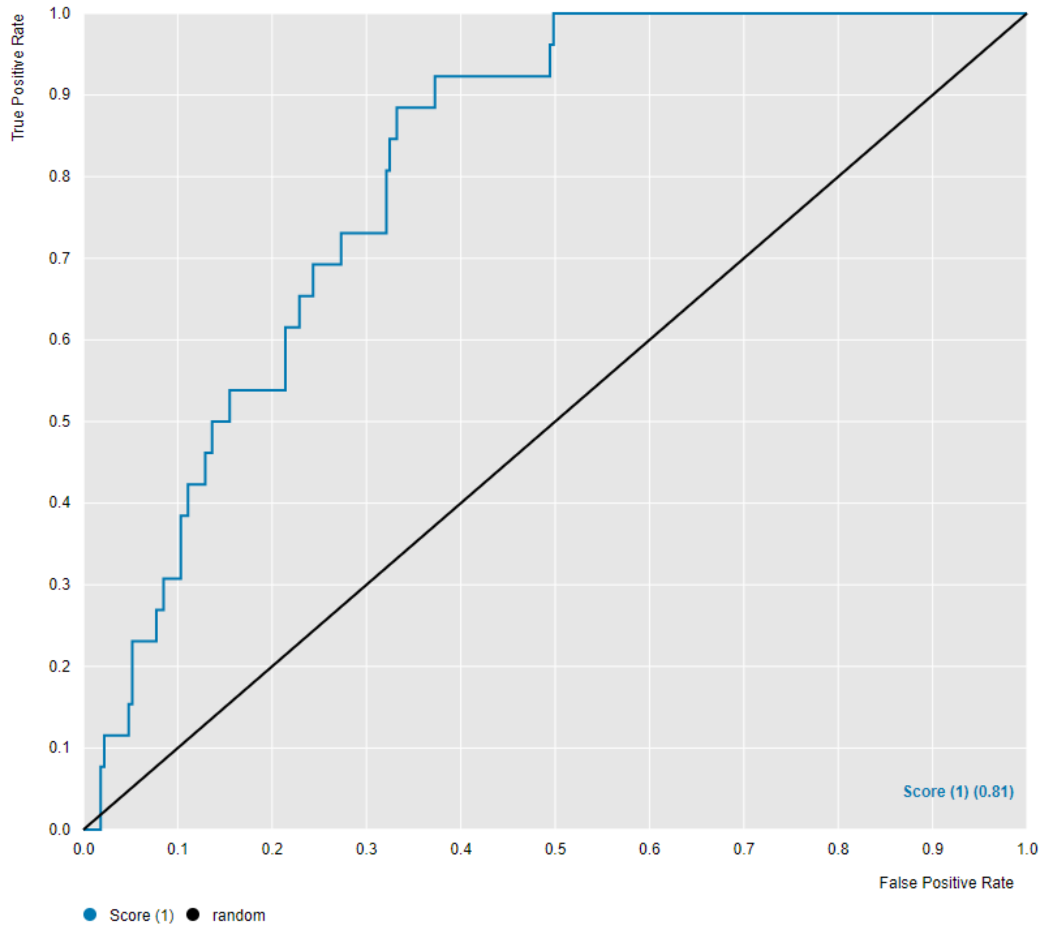

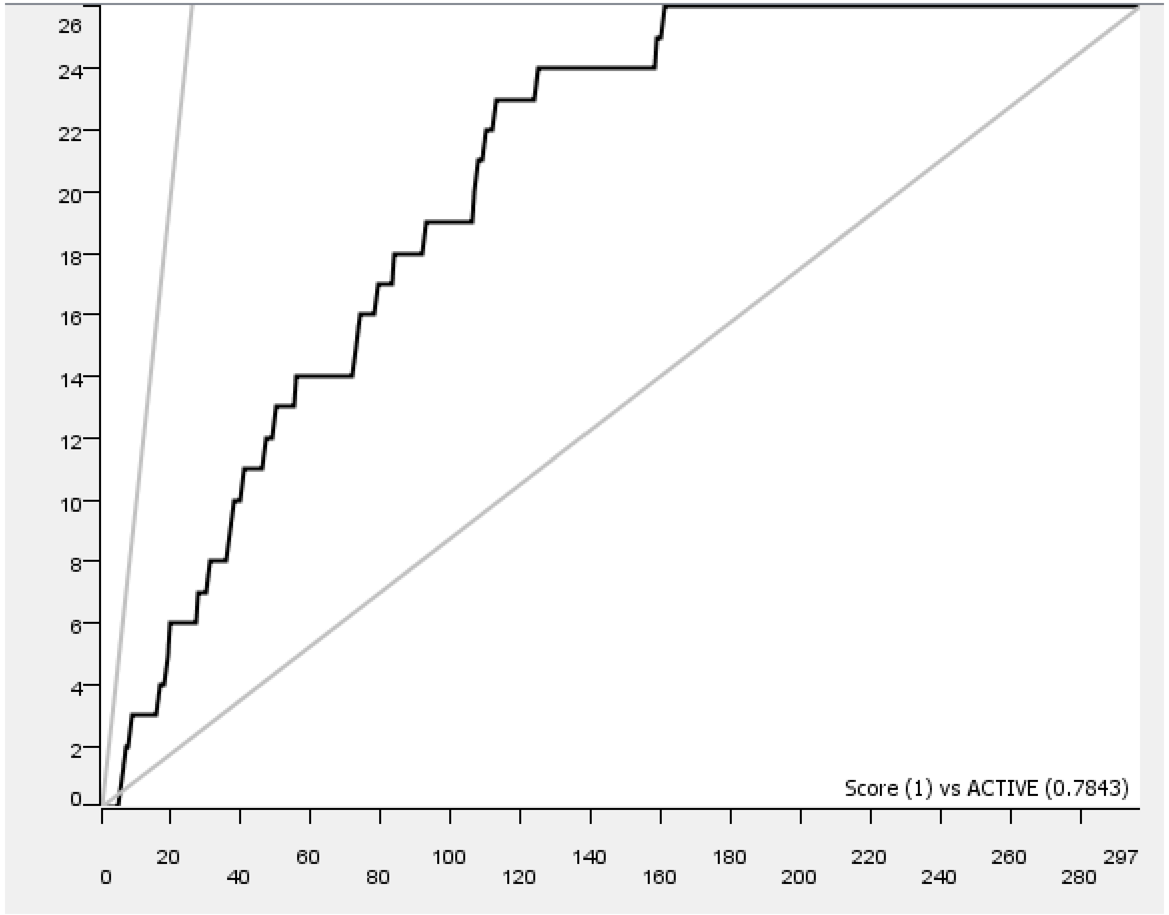

N=297

Top 10% Mean IC50	157 nM

	Predicted Active	Predicted Inactive
Active	16	10
Inactive	61	210

<10 nM DefGood in Aurora B

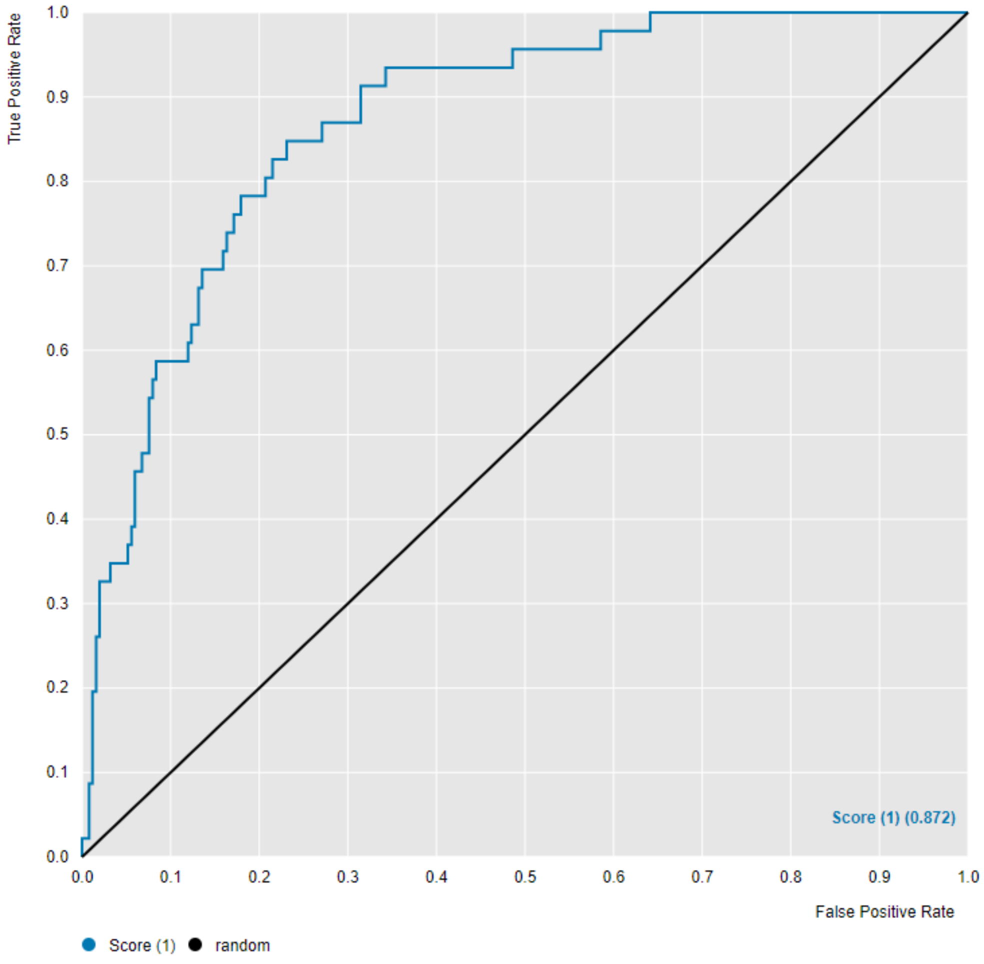

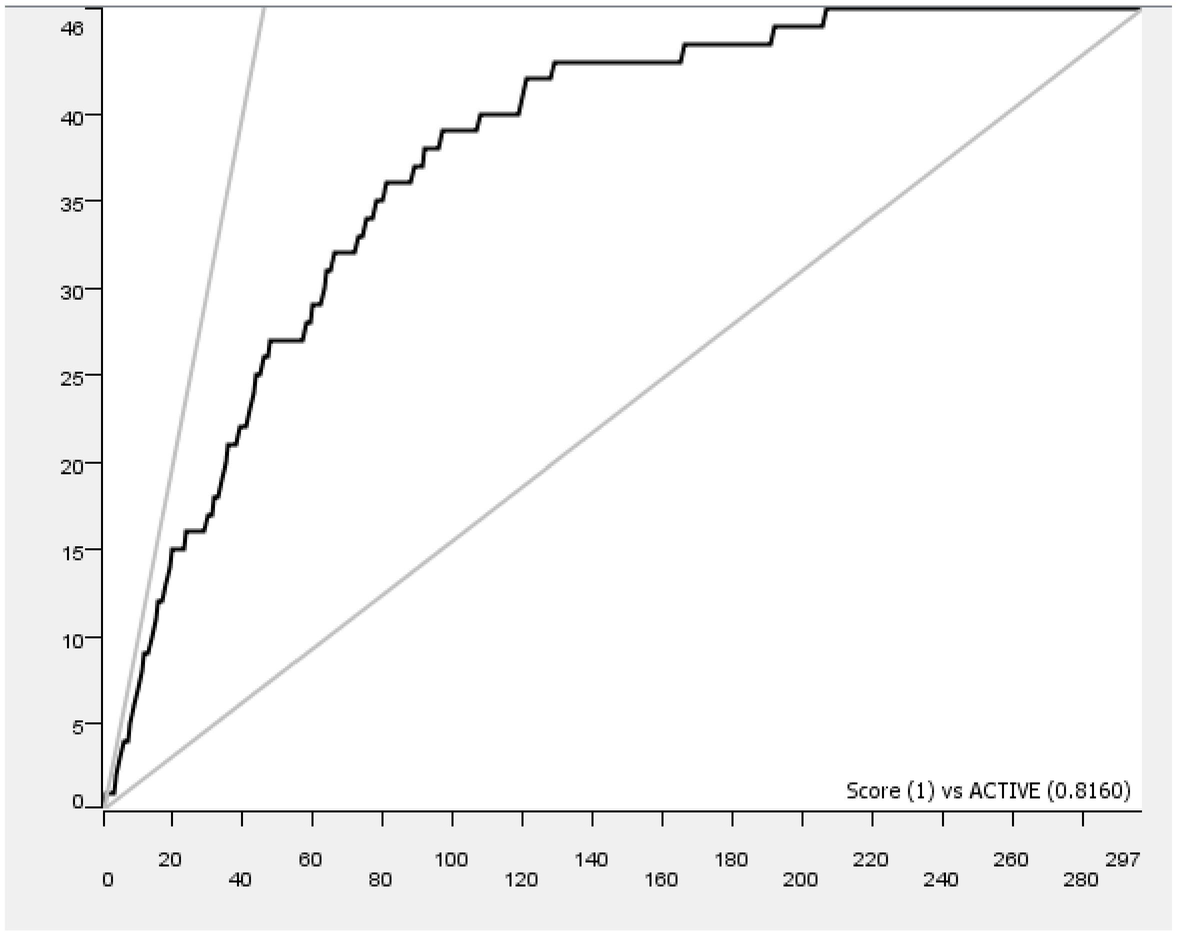

N=297

**Top 10% Mean IC50**	**30.6 nM**

	Predicted Active	Predicted Inactive
Active	39	7
Inactive	64	187

<15 nM DefGood in Aurora B

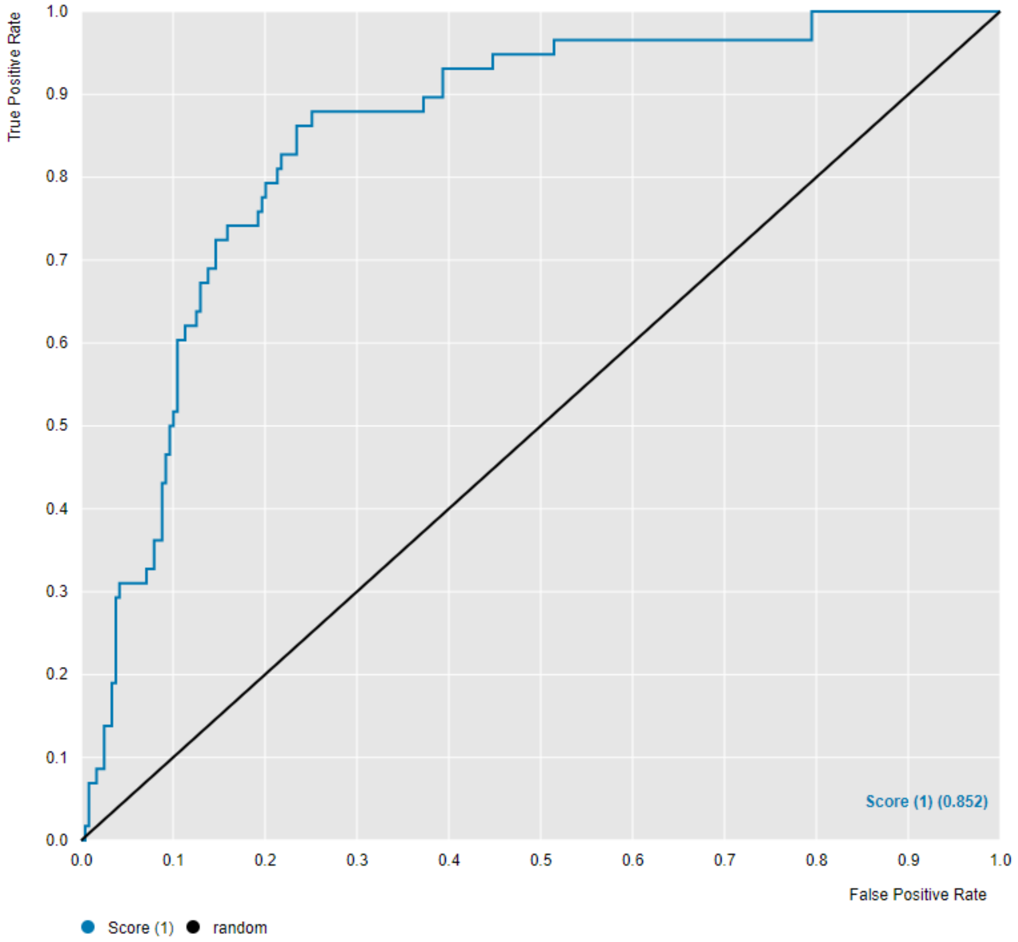

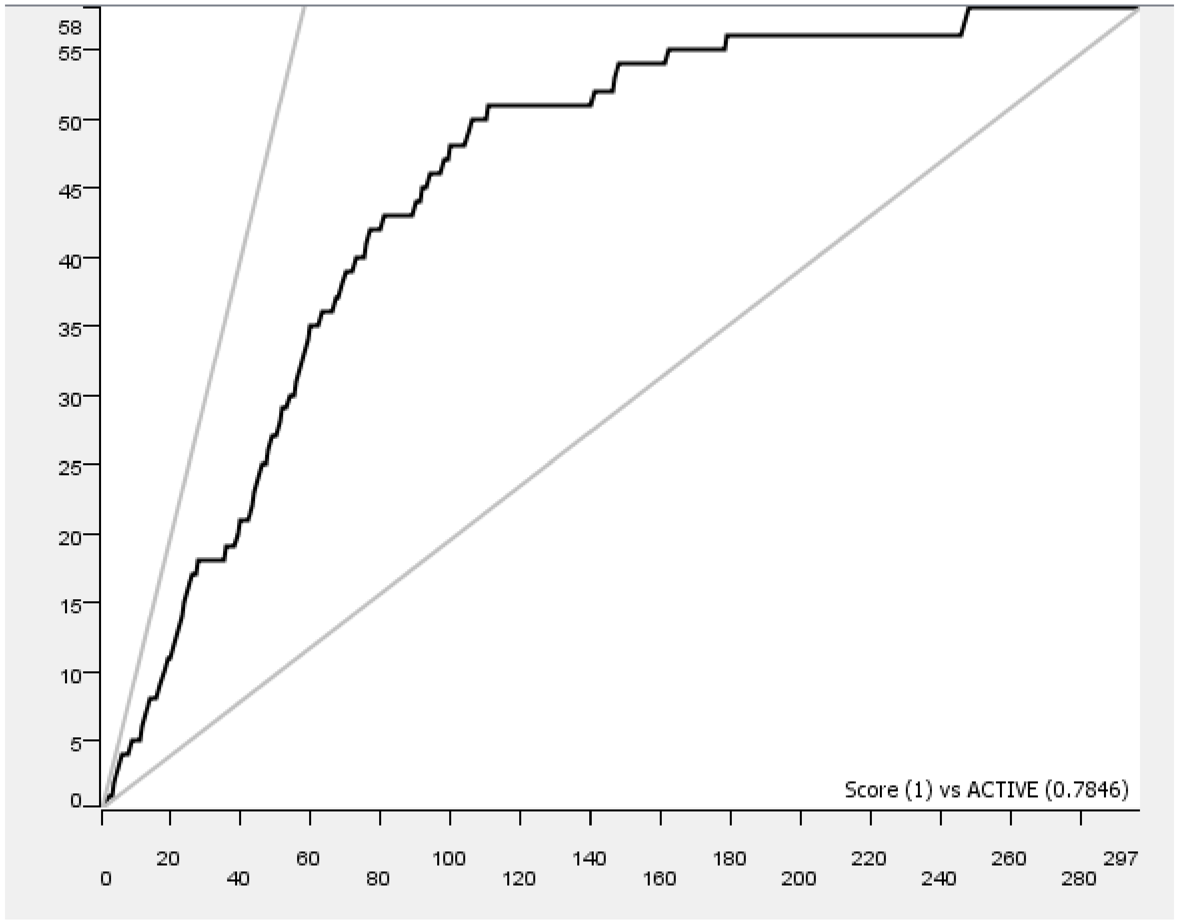

N=297

**Top 10% Mean IC50**	**53.1 nM**

	Predicted Active	Predicted Inactive
Active	51	7
Inactive	66	173

<20 nM DefGood in Aurora B

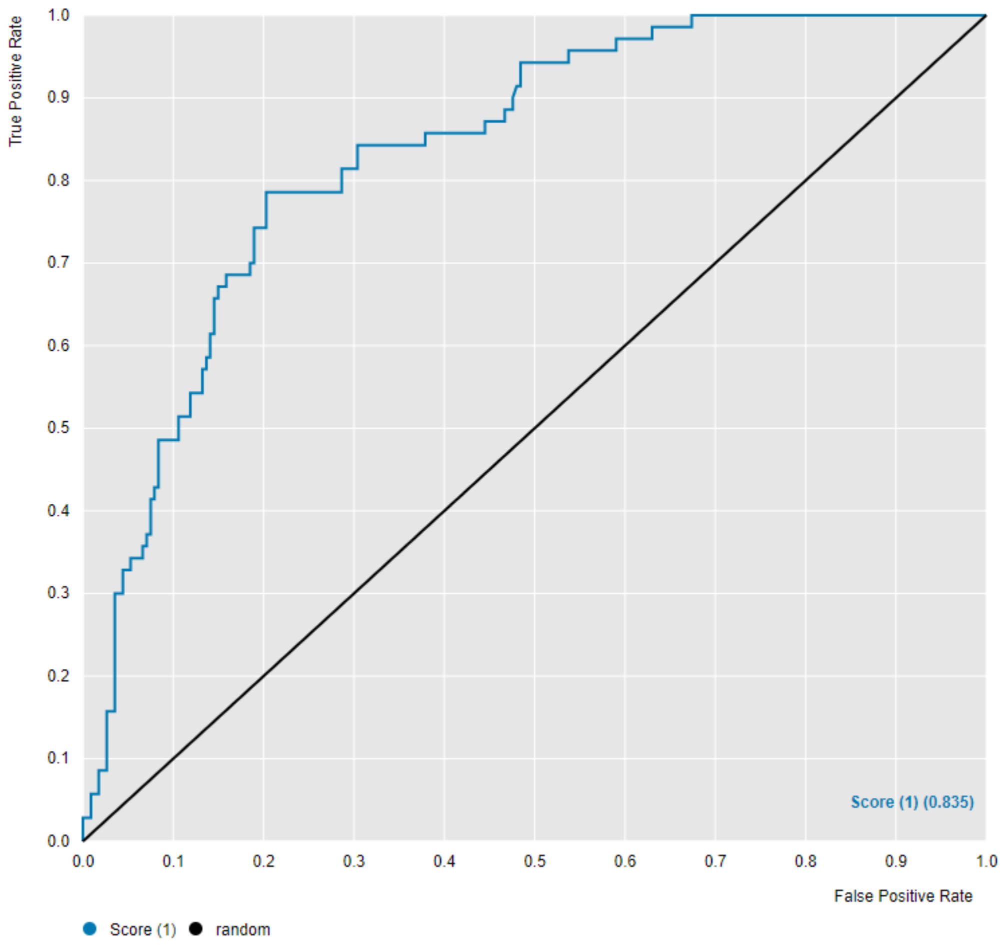

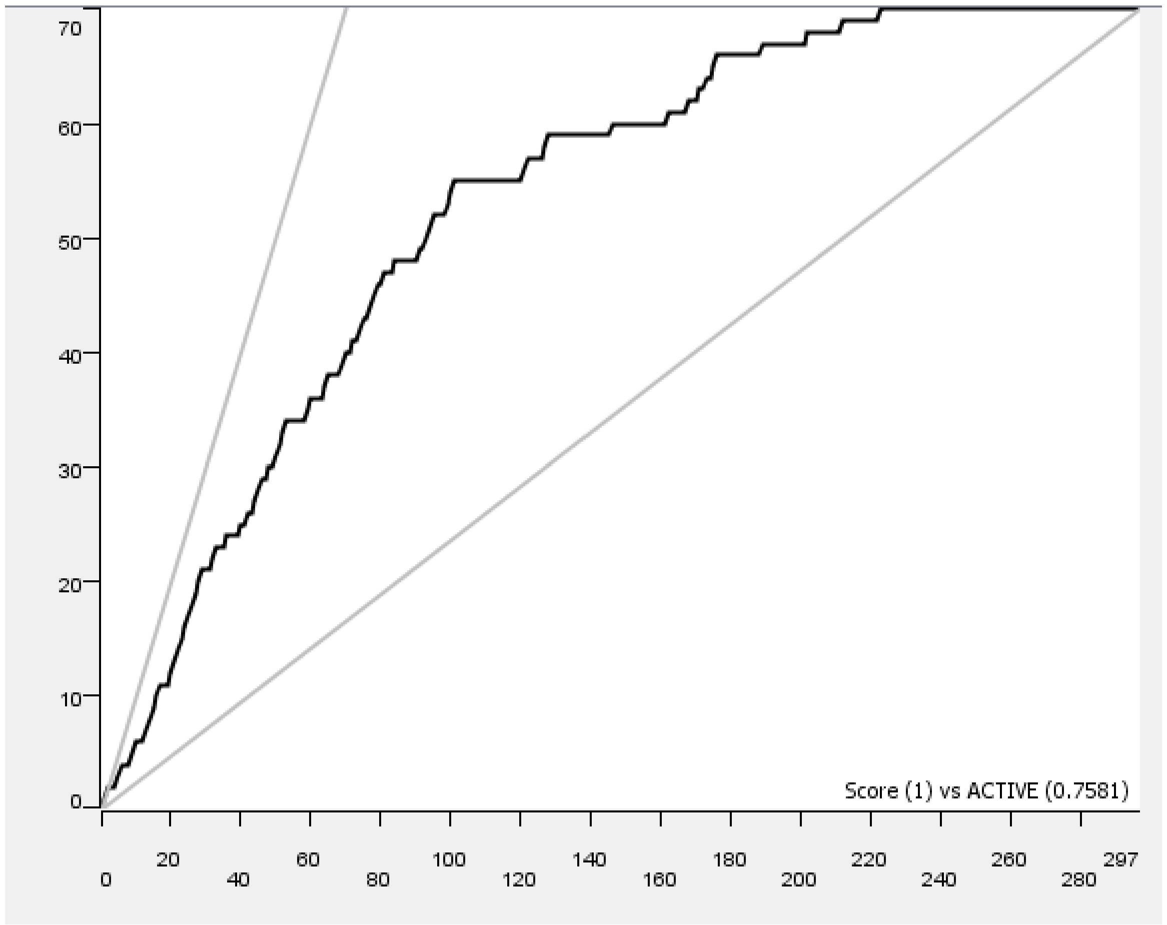

N=297

**Top 10% Mean IC50**	**32.4 nM**

	Predicted Active	Predicted Inactive
Active	52	18
Inactive	44	183

<25 nM DefGood in Aurora B

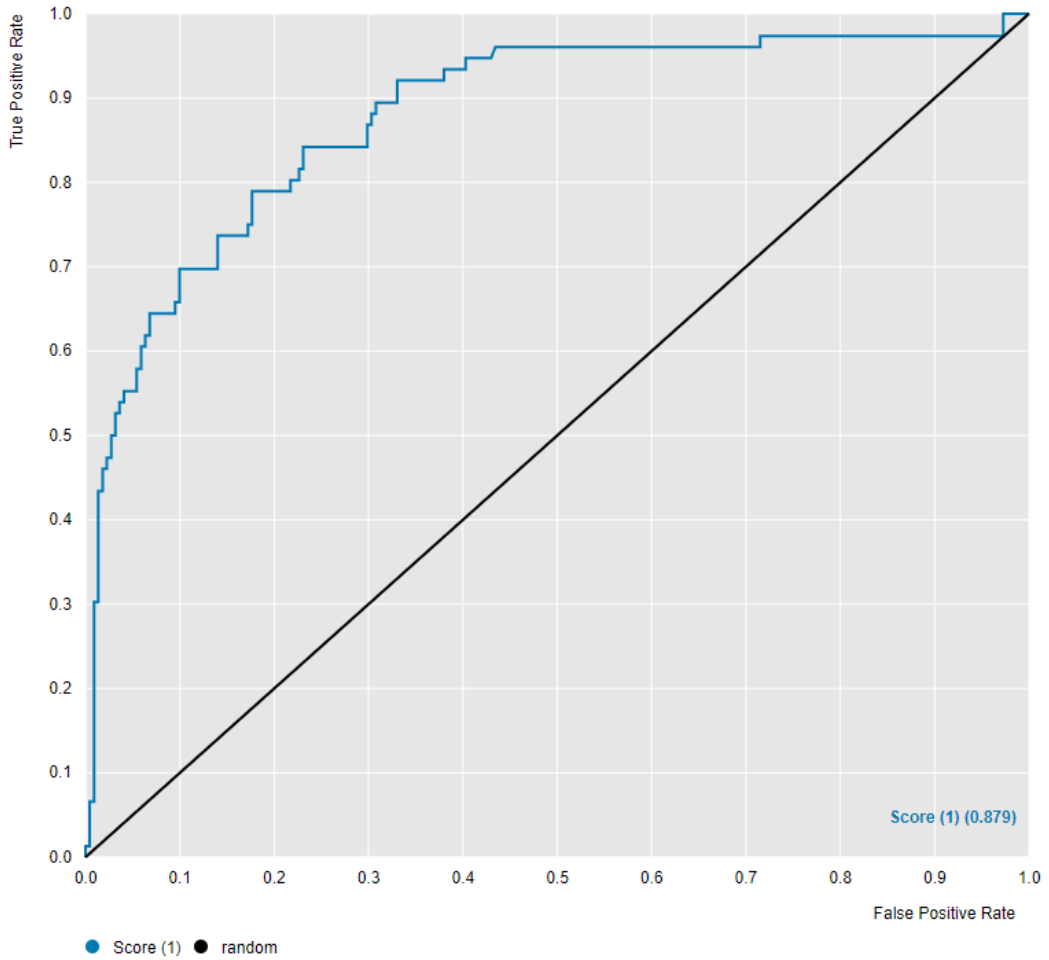

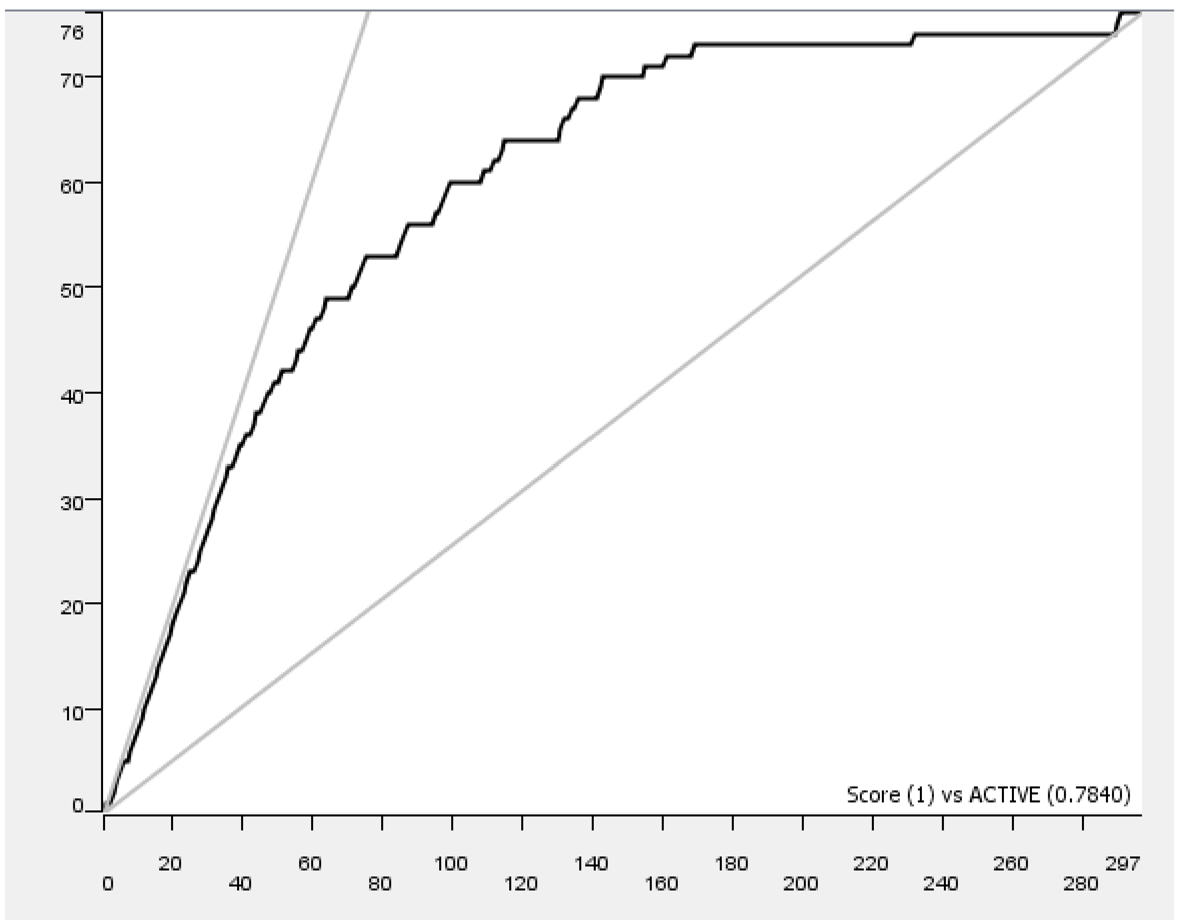

N=297

**Top 10% Mean IC50**	**391 nM**

	**Predicted Active**	**Predicted Inactive**
Active	60	16
Inactive	40	181

Decision on DefGood

- <10 nM has the best performance with regard to enrichment and mean top 10% IC50.

RF - <10 nM DefGood in Aurora B

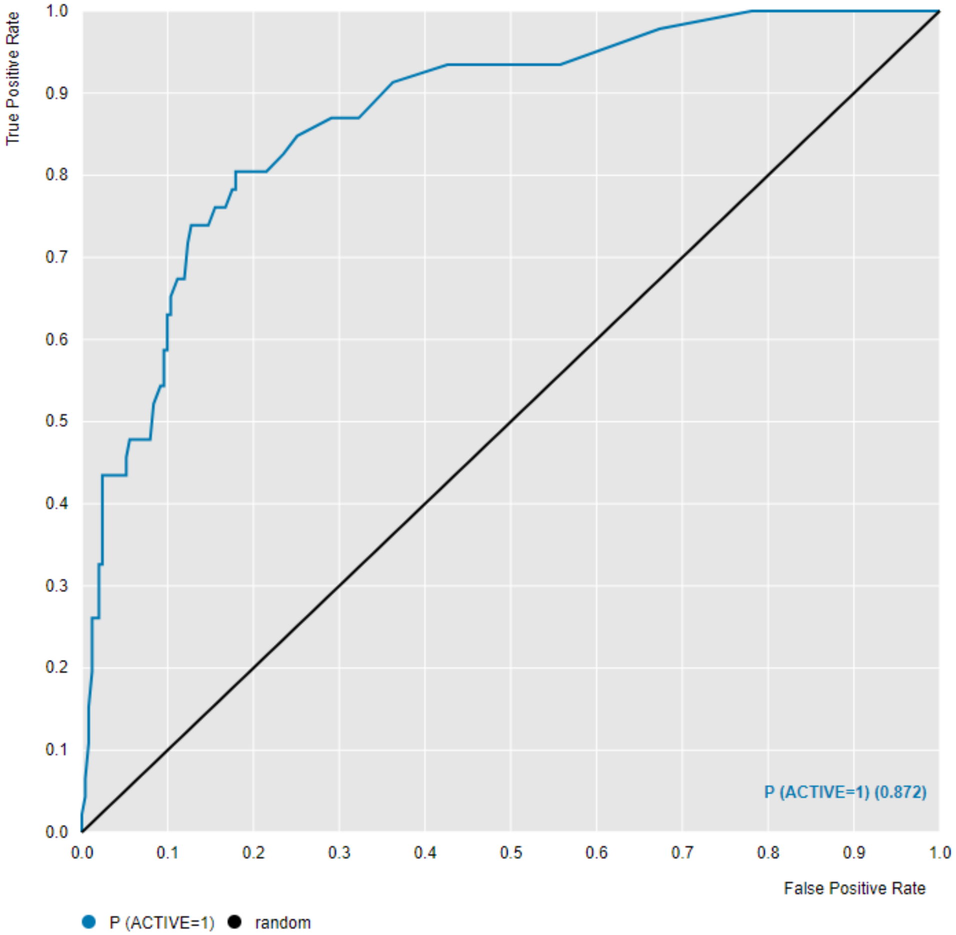

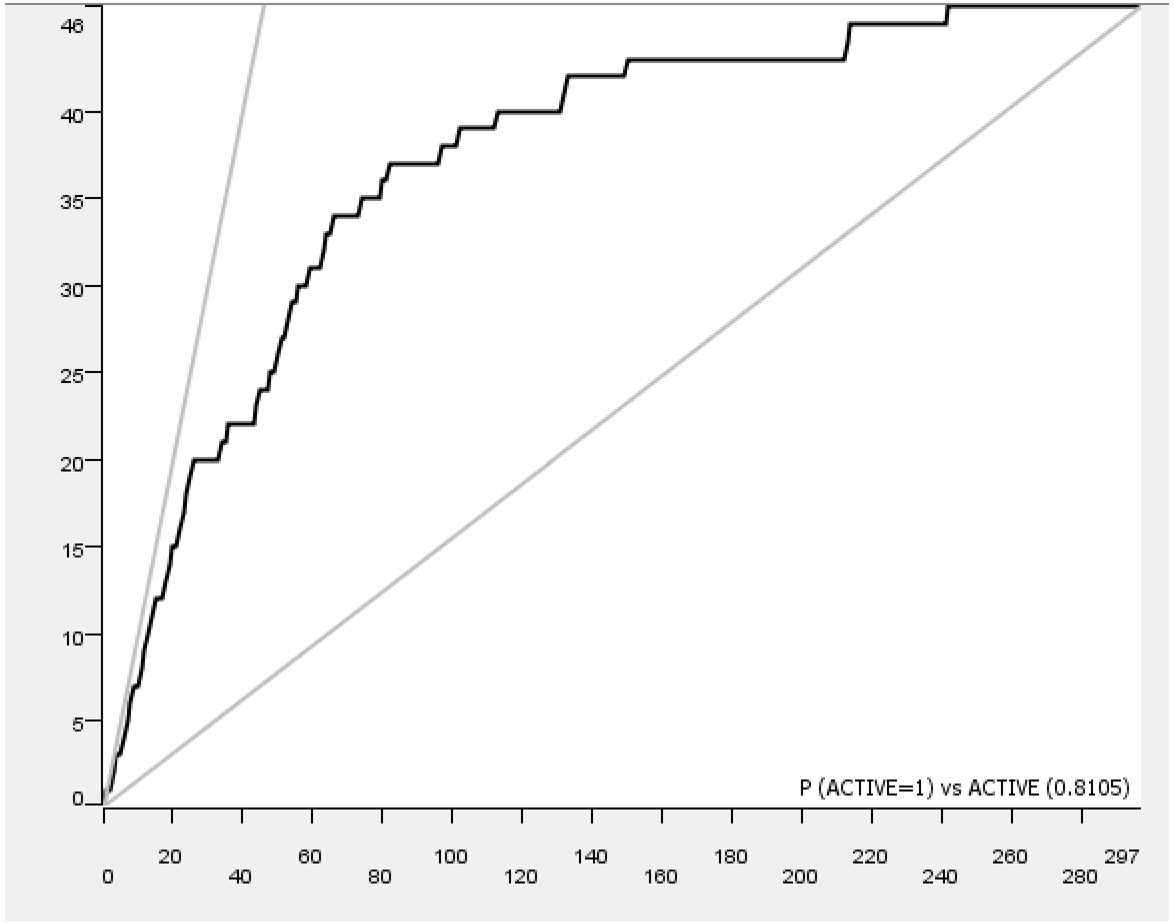

N=297

Top 10% Mean IC50	32.6 nM

	Predicted Active	Predicted Inactive
Active	20	26
Inactive	9	242

Decision on DefGood - RF

- Work in ALK showed DefGood in the NBN was similar to DefGood in an RF. Therefore, this value for DefGood from the NBN design was used as DefGood.

PNN - <10 nM DefGood in Aurora B

- Properties List (46 calculatable properties were used)
- Parameterization (Global Accuracy for Activity at a 10 nM decision value was used, and a 5-fold stratified sampling cross validation was used to parameterize theta minus and theta plus) Theta Minus = 0.05 and Theta Plus = 0.45

PNN - <10 nM DefGood in Aurora B

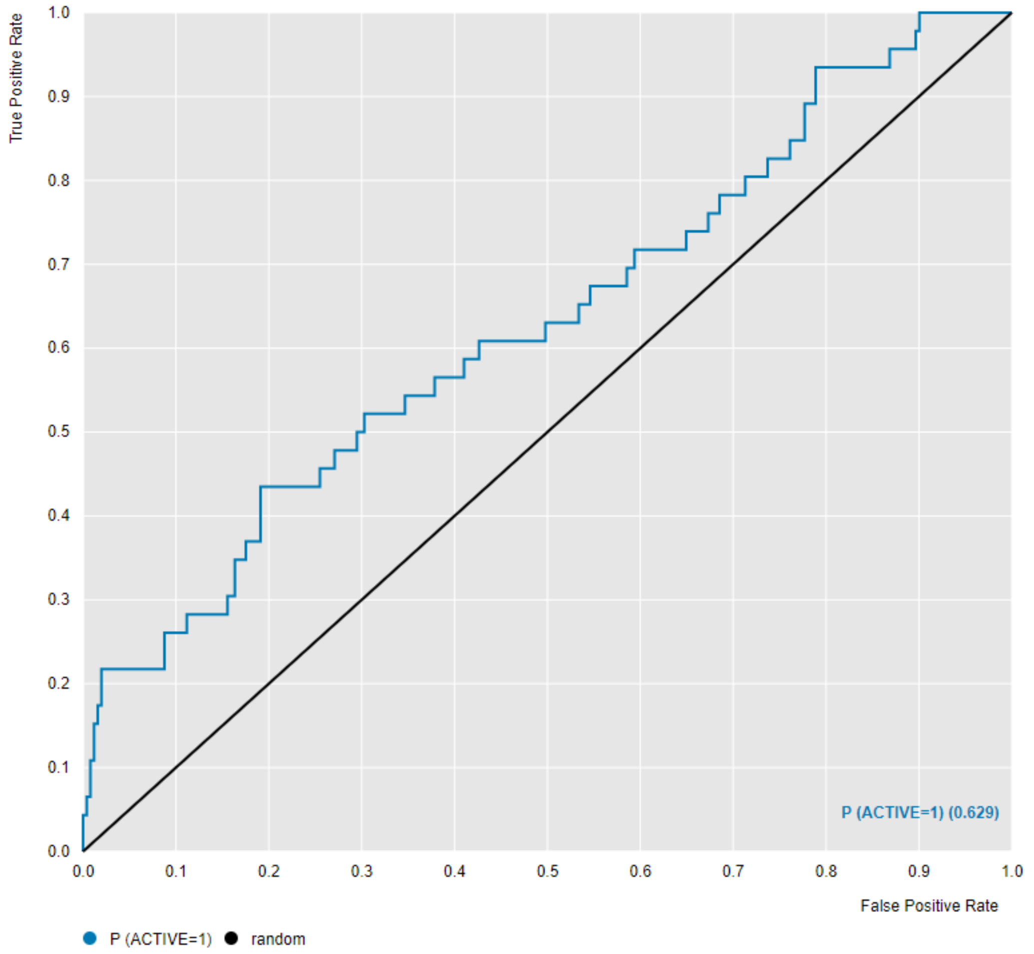

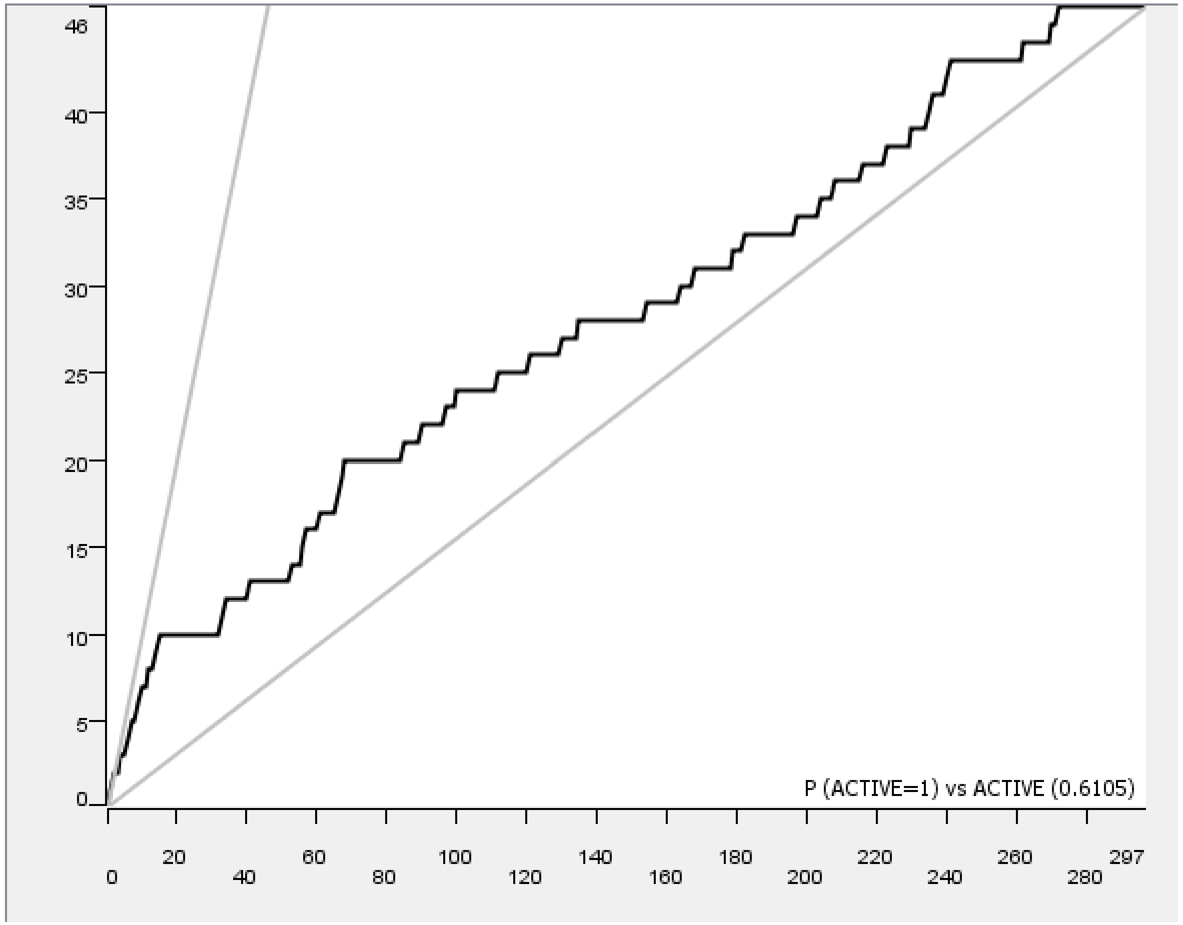

N=297

Top 10% Mean IC50	5,900 nM

	Predicted Active	Predicted Inactive
Active	3	43
Inactive	1	250

PNN - <20 nM DefGood in Aurora B

- Initial DefGood failed to generate a useful model. Therefore, 20 nM was evaluated as the decision value
- Properties List (46 calculatable properties were used)
- Parameterization (Global Accuracy for Activity at a 20 nM decision value was used, and a 5-fold stratified sampling cross validation was used to parameterize theta minus and theta plus) Theta Minus = 0.05 and Theta Plus = 0.65

PNN - <20 nM DefGood in Aurora B

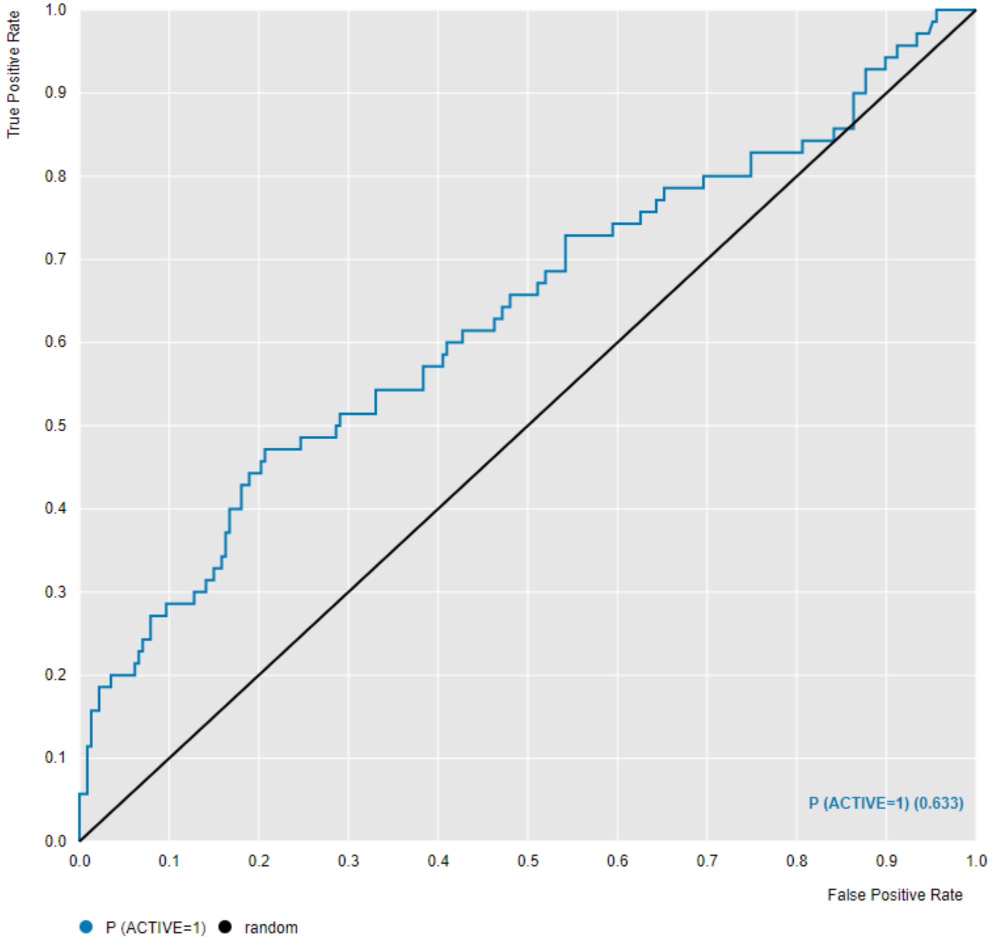

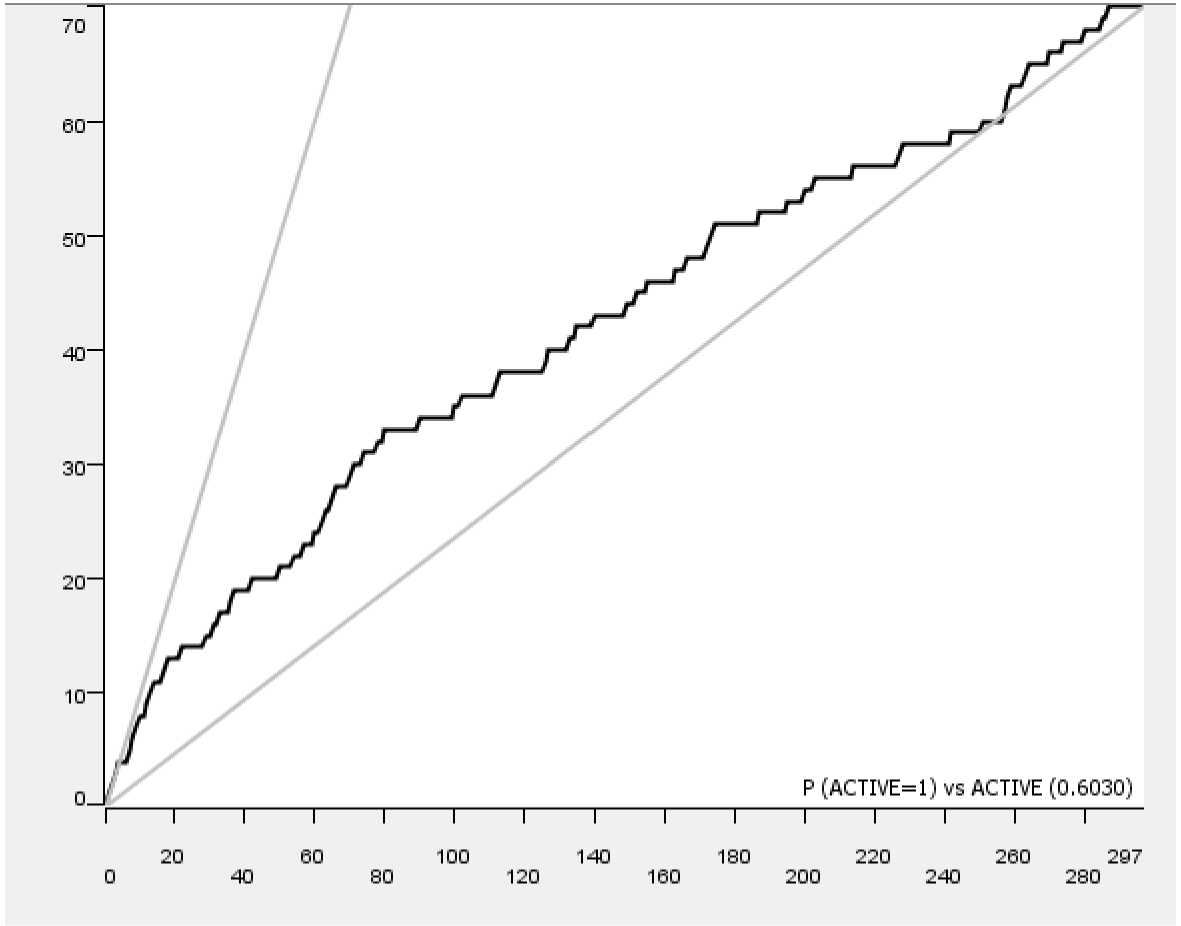

N=297

**Top 10% Mean IC50**	**2,300 nM**

	Predicted Active	Predicted Inactive
Active	4	66
Inactive	0	227

Decision on DefGood - PNN

- The PNN failed to generate a useful predictive algorithm with a definition of good at <10 nM or at <20 nM
- A PNN will not be used for aroura B kinase

NBN Error Tolerance- <10 nM DefGood in Aurora B

- 0-50% absolute error

<10 nM DefGood in aurora B, 5% error; Random seed = 1515533876005

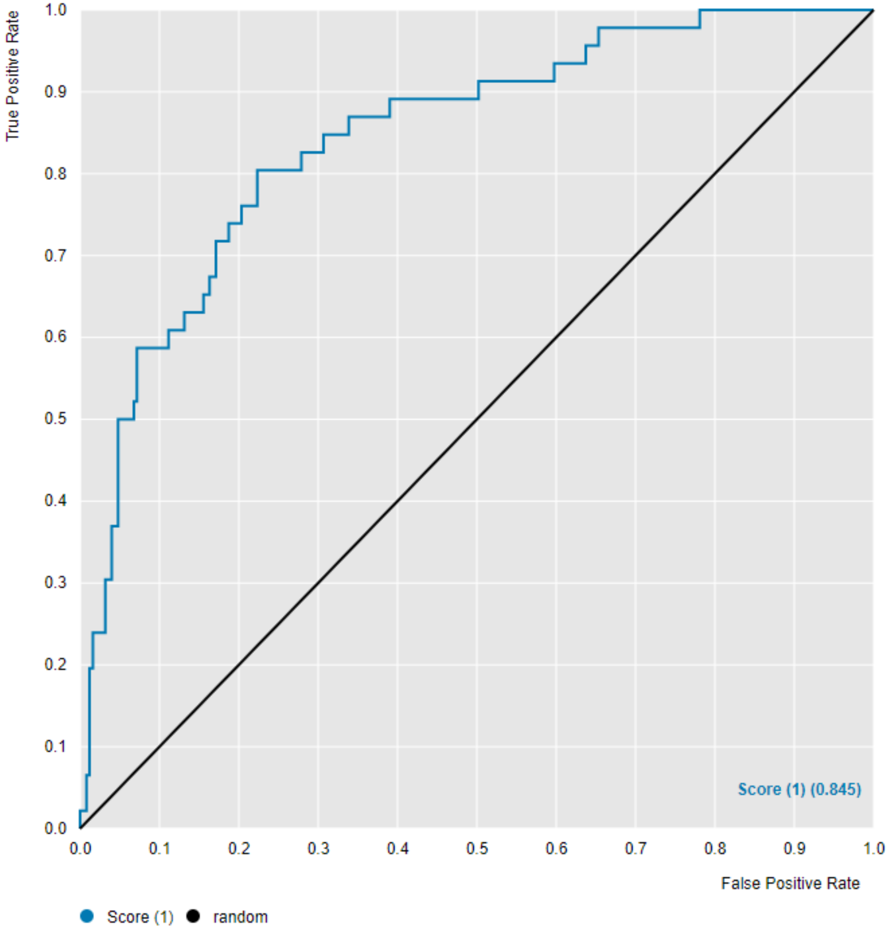

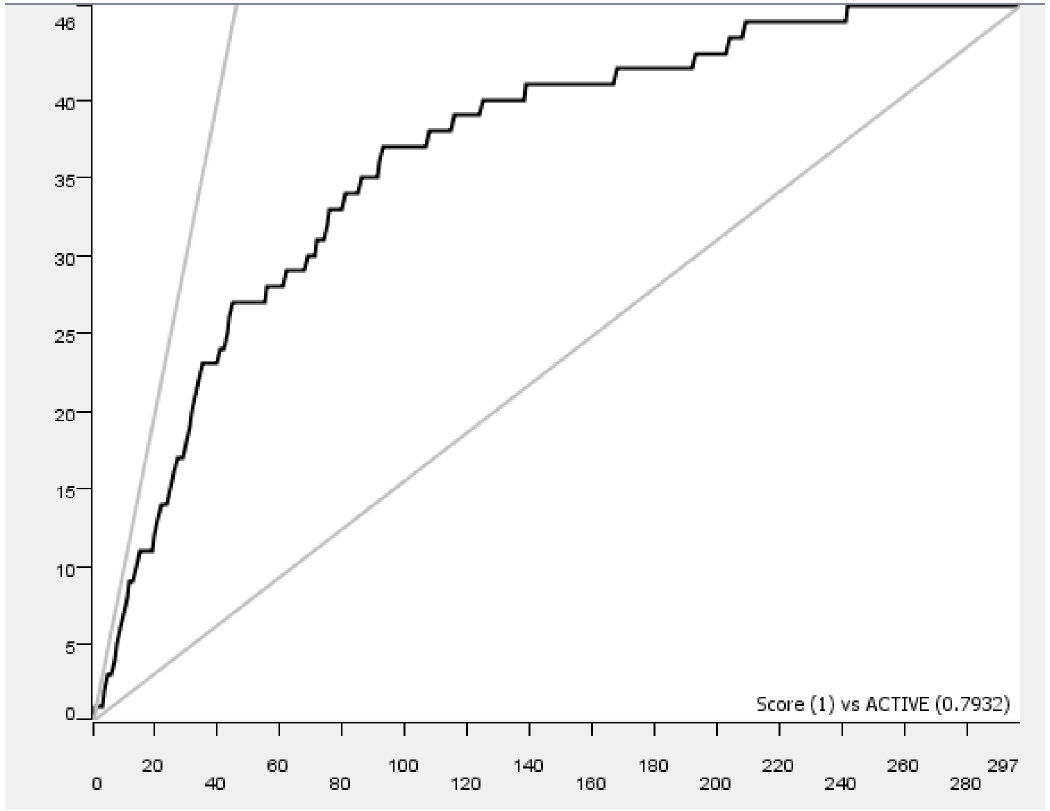

N=297

**Top 10% Mean IC50**	**405 nM (one micromolar and one >10000 nM)**

	Predicted Active	Predicted Inactive
Active	37	9
Inactive	63	188

<10nM DefGood in aurora B, 10% error

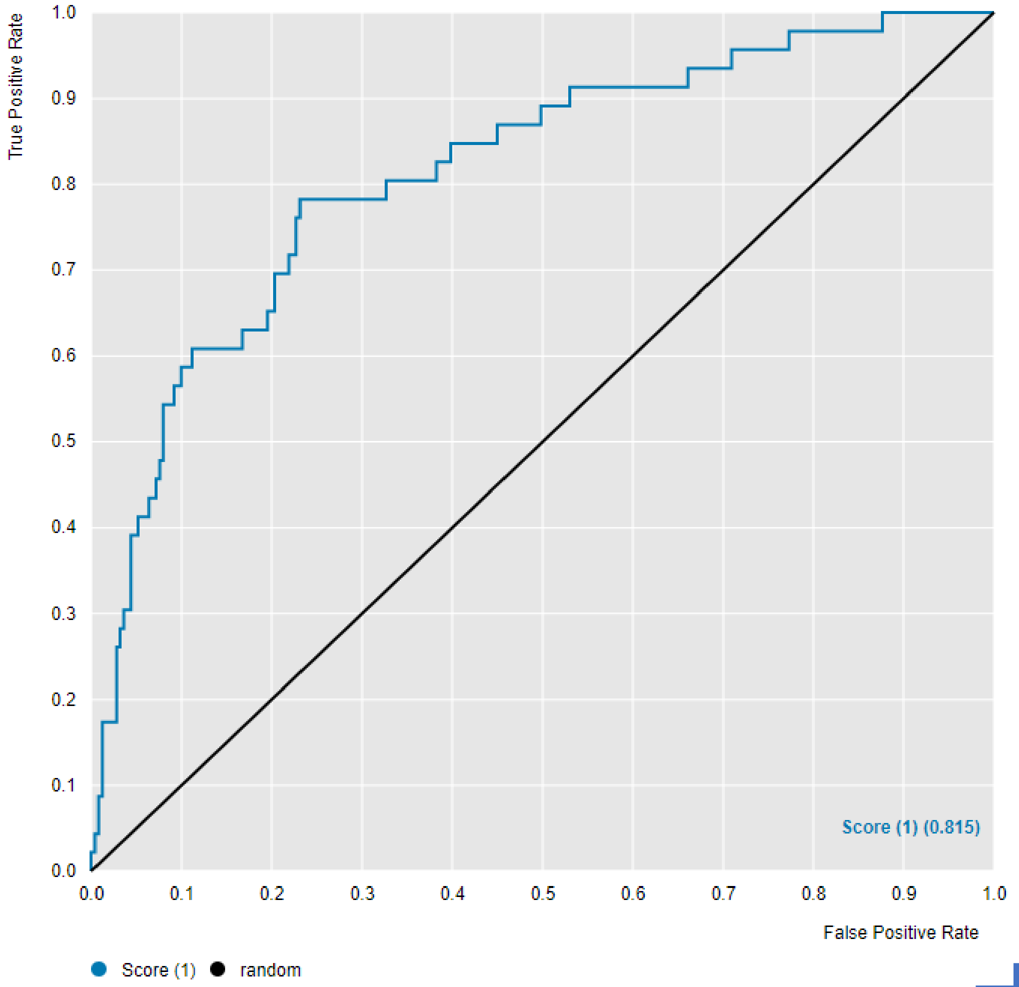

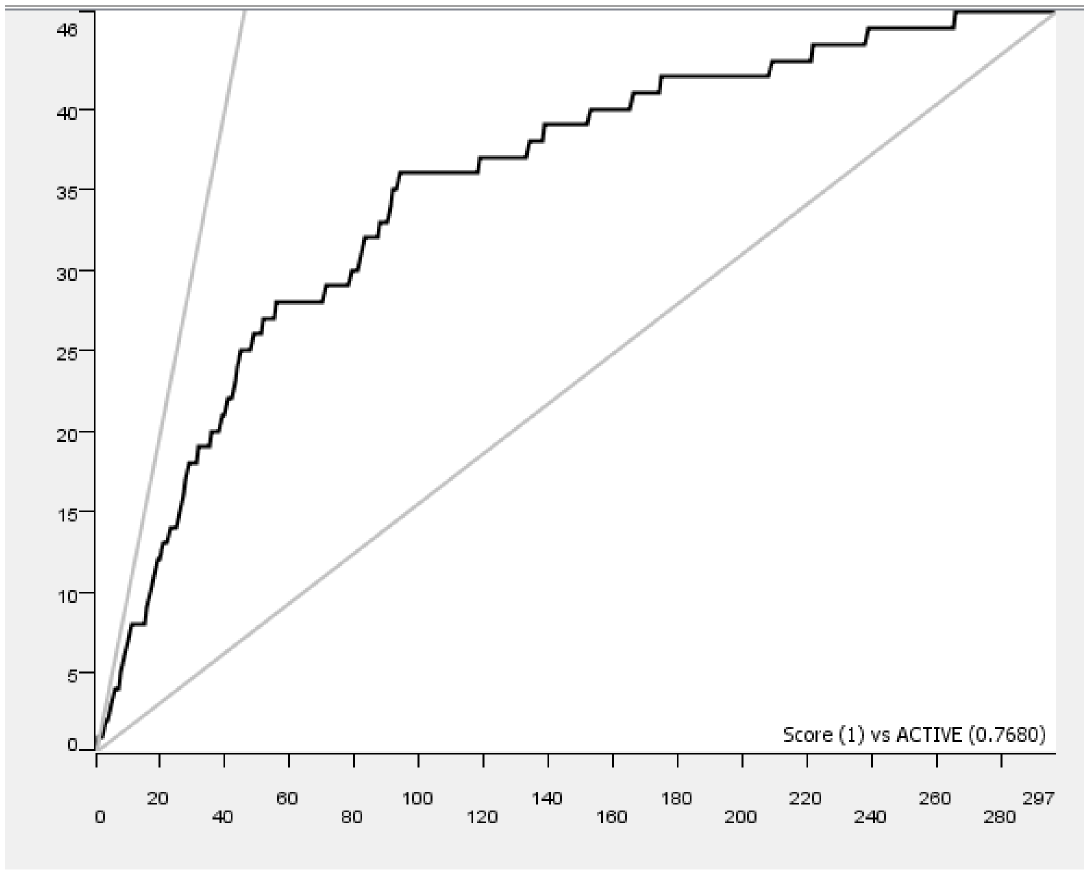

N=297

**Top 10% Mean IC50**	**403 nM (one micromolar and one >10000 nM)**

	Predicted Active	Predicted Inactive
Active	28	18
Inactive	41	210

<10nM DefGood in aurora B, 15% error

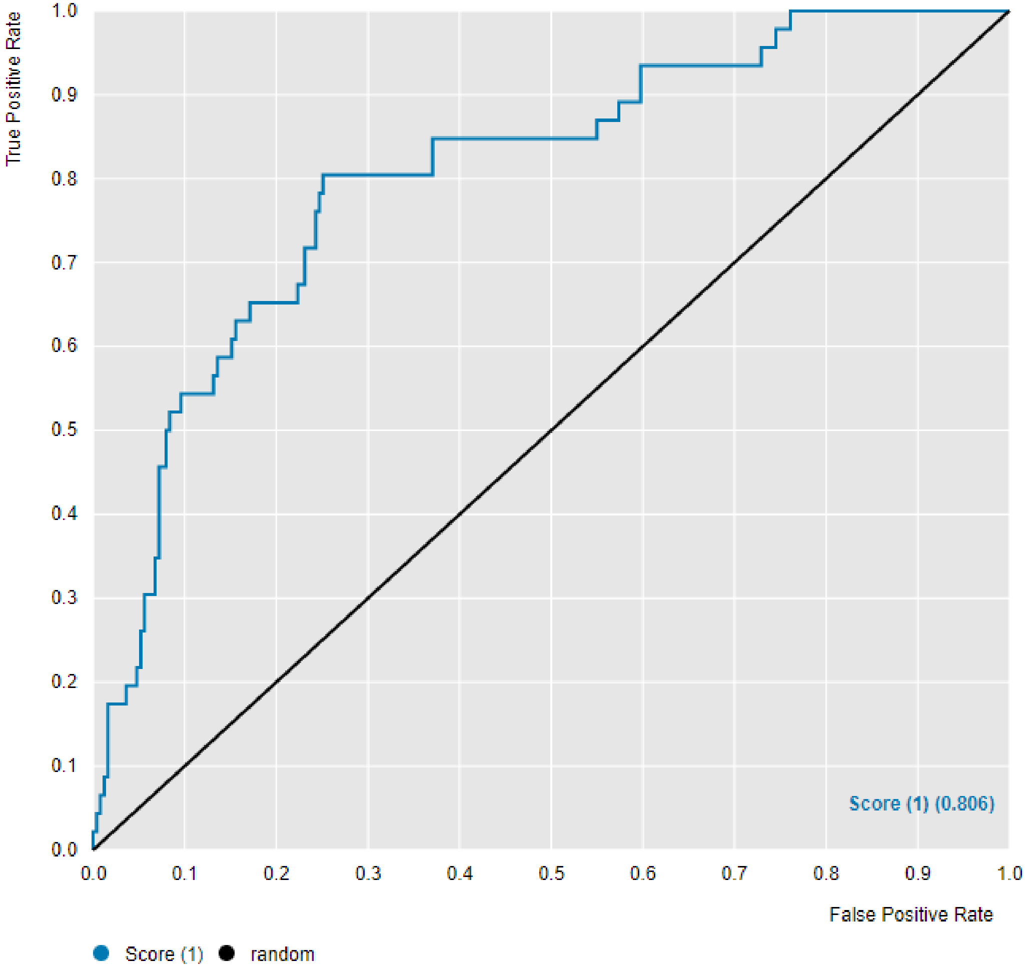

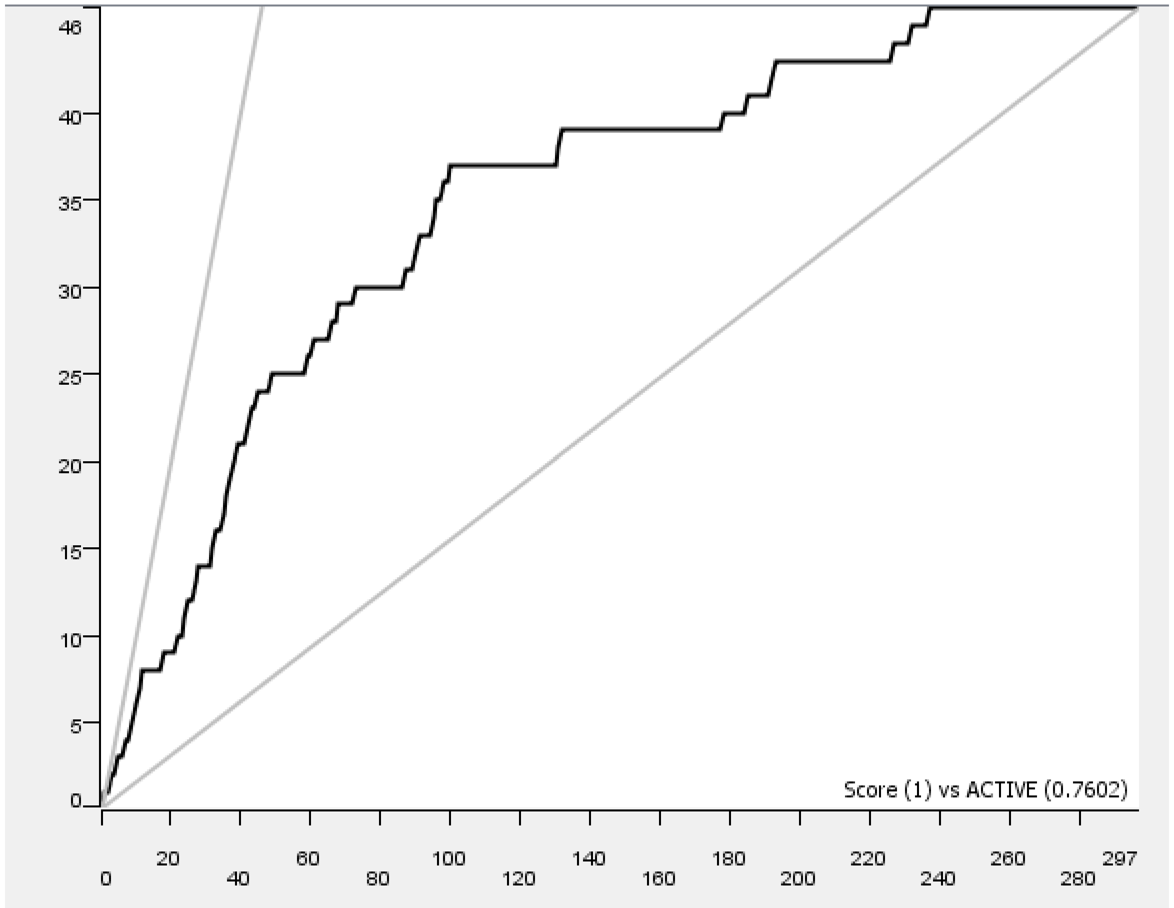

N=297

**Top 10% Mean IC50**	**426 nM**

	Predicted Active	Predicted Inactive
Active	26	20
Inactive	33	218

<10nM DefGood in aurora B, 20% error

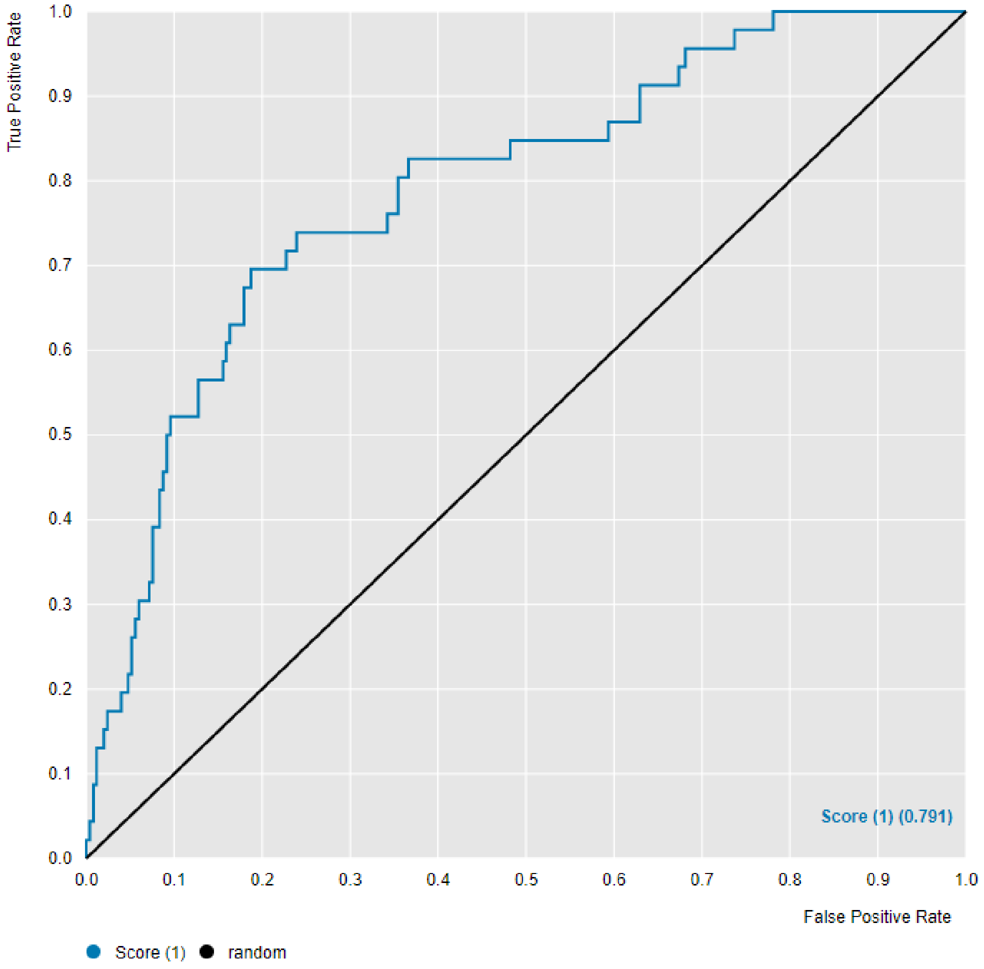

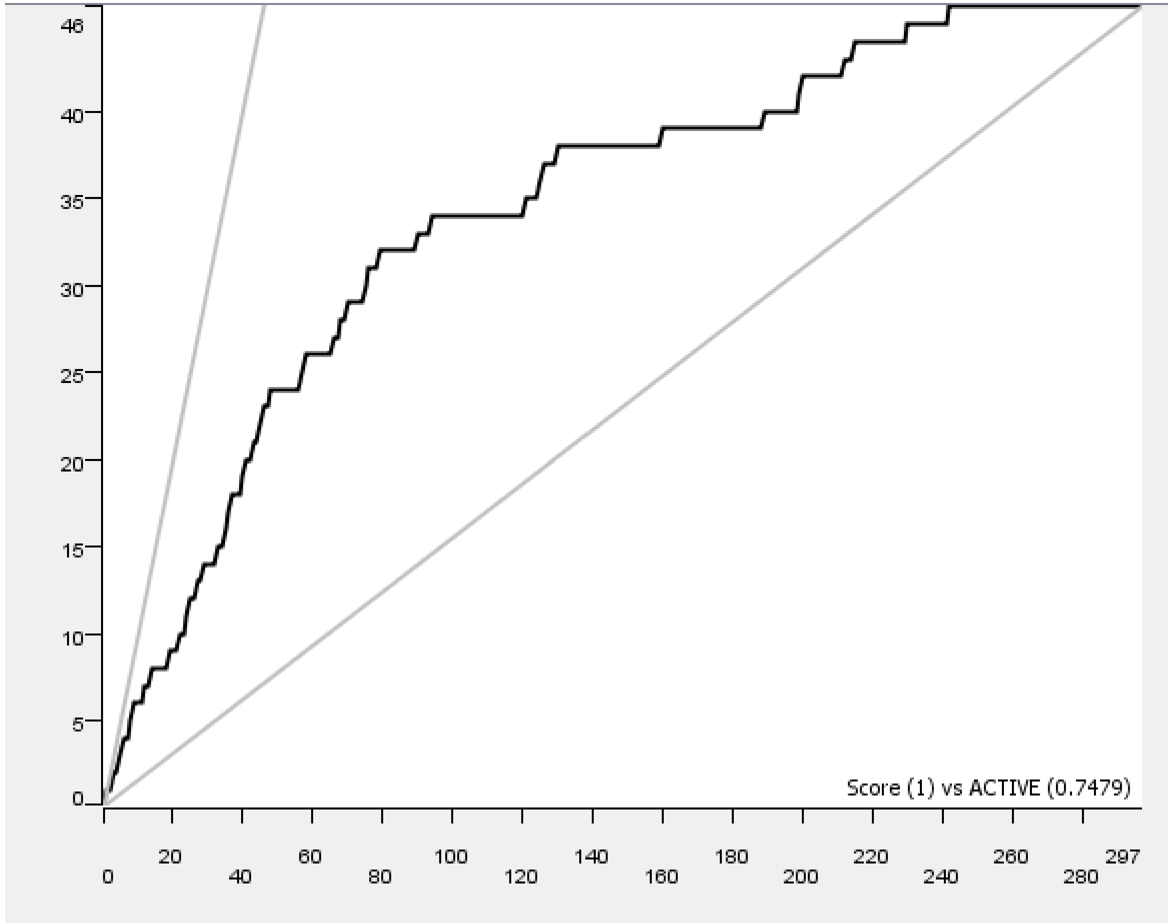

N=297

Top 10% Mean IC50	427 nM

	Predicted Active	Predicted Inactive
Active	33	13
Inactive	57	194

<10nM DefGood in aurora B, 25% error

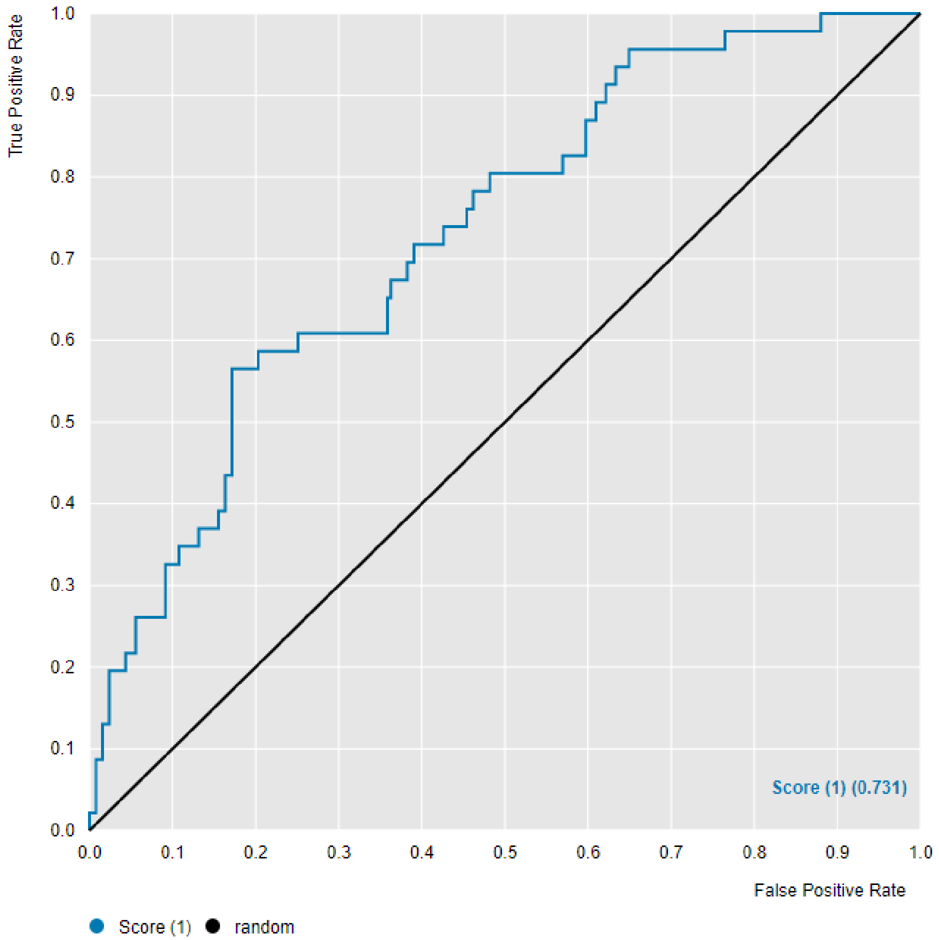

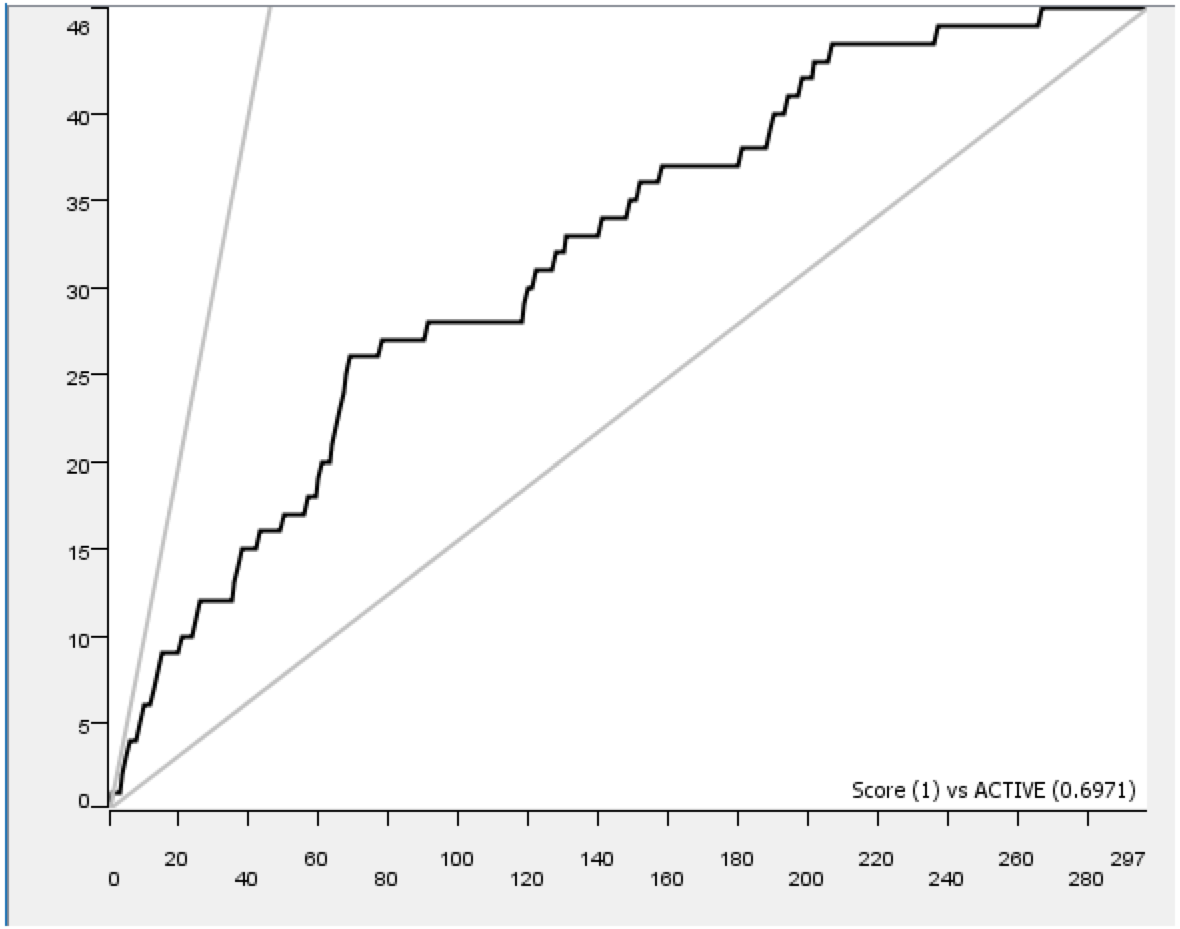

N=297

**Top 10% Mean IC50**	**435 nM**

	Predicted Active	Predicted Inactive
Active	28	18
Inactive	80	171

<10nM DefGood in aurora B, 30% error

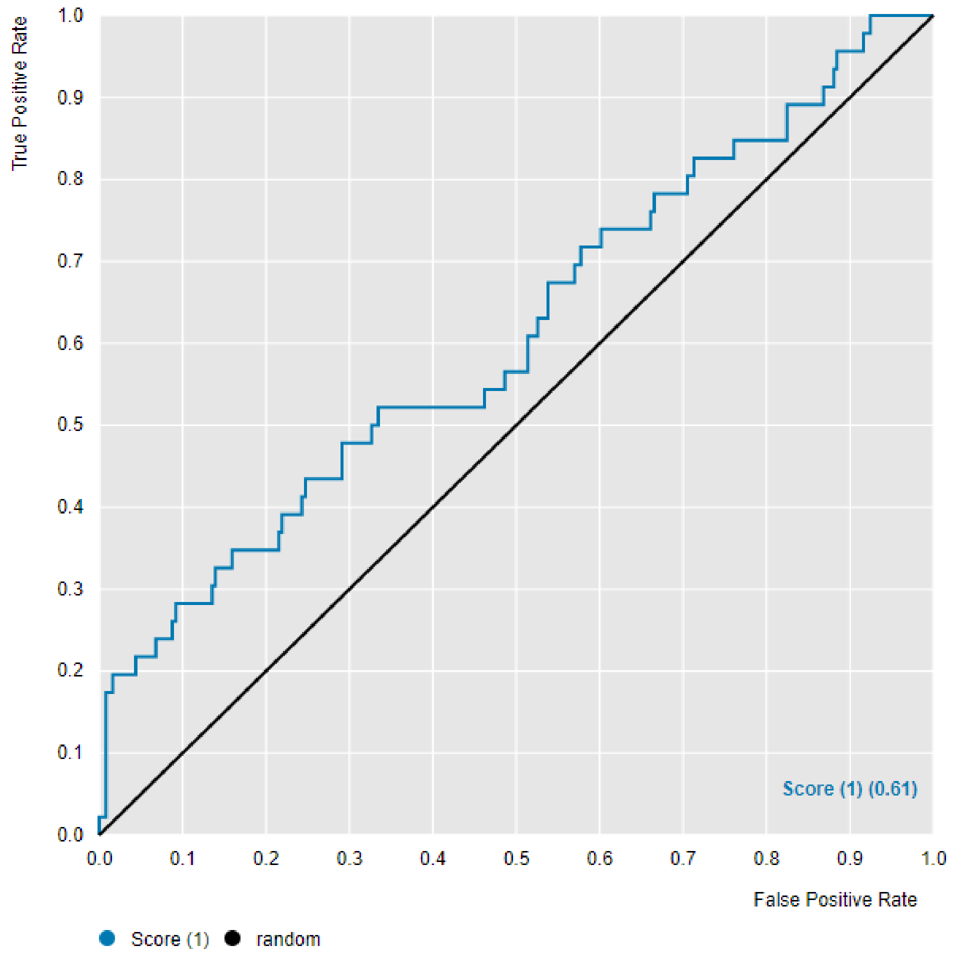

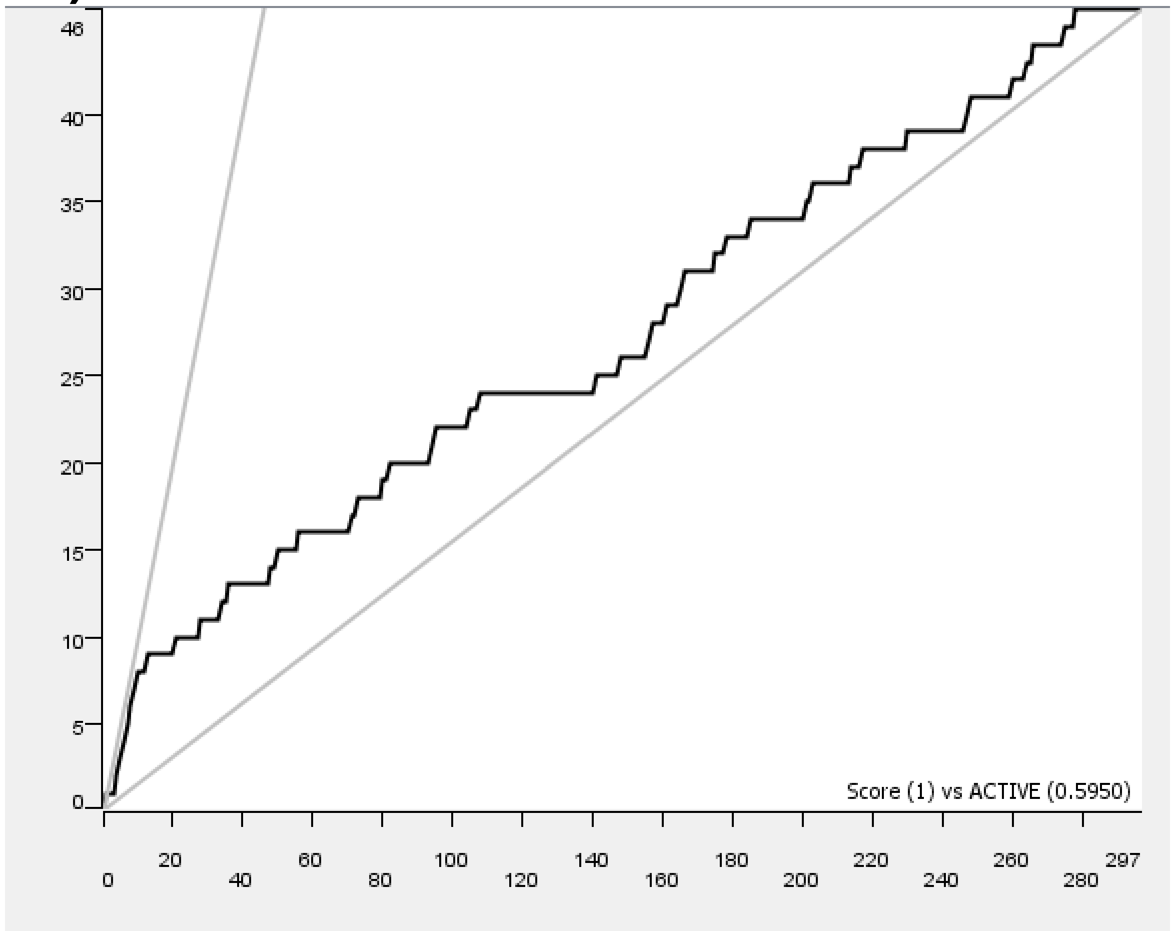

N=297

**Top 10% Mean IC50**	**925 nM**

	**Predicted Active**	**Predicted Inactive**
Active	24	22
Inactive	98	153

<10nM DefGood in aurora B, 10% error; Random seed = 429

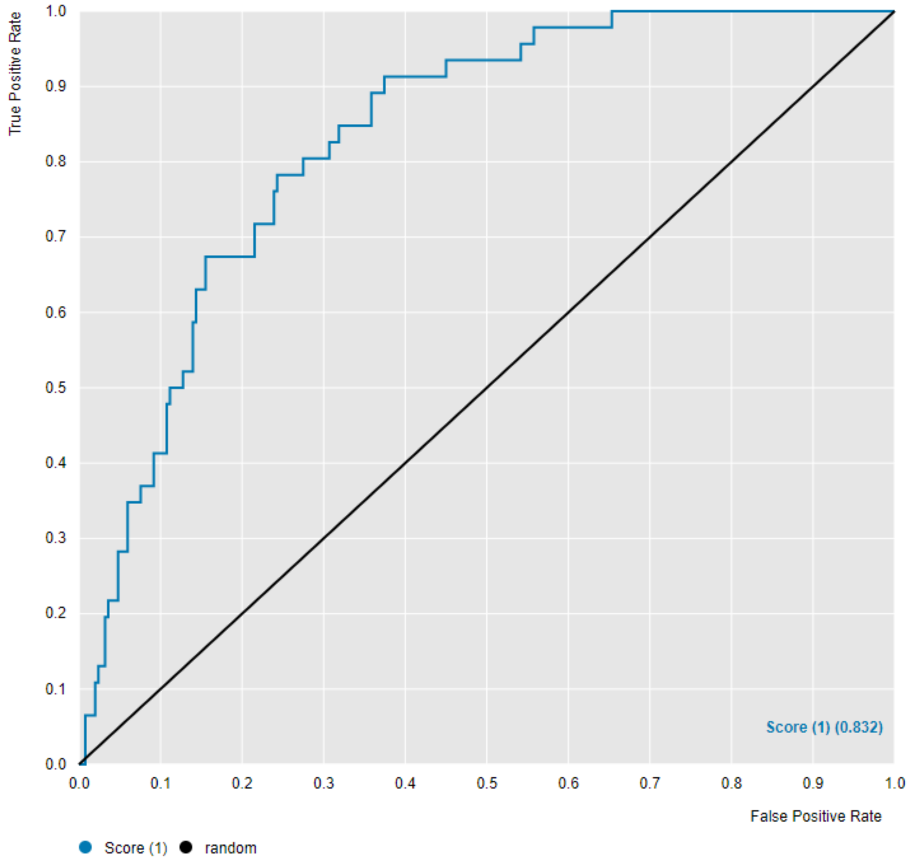

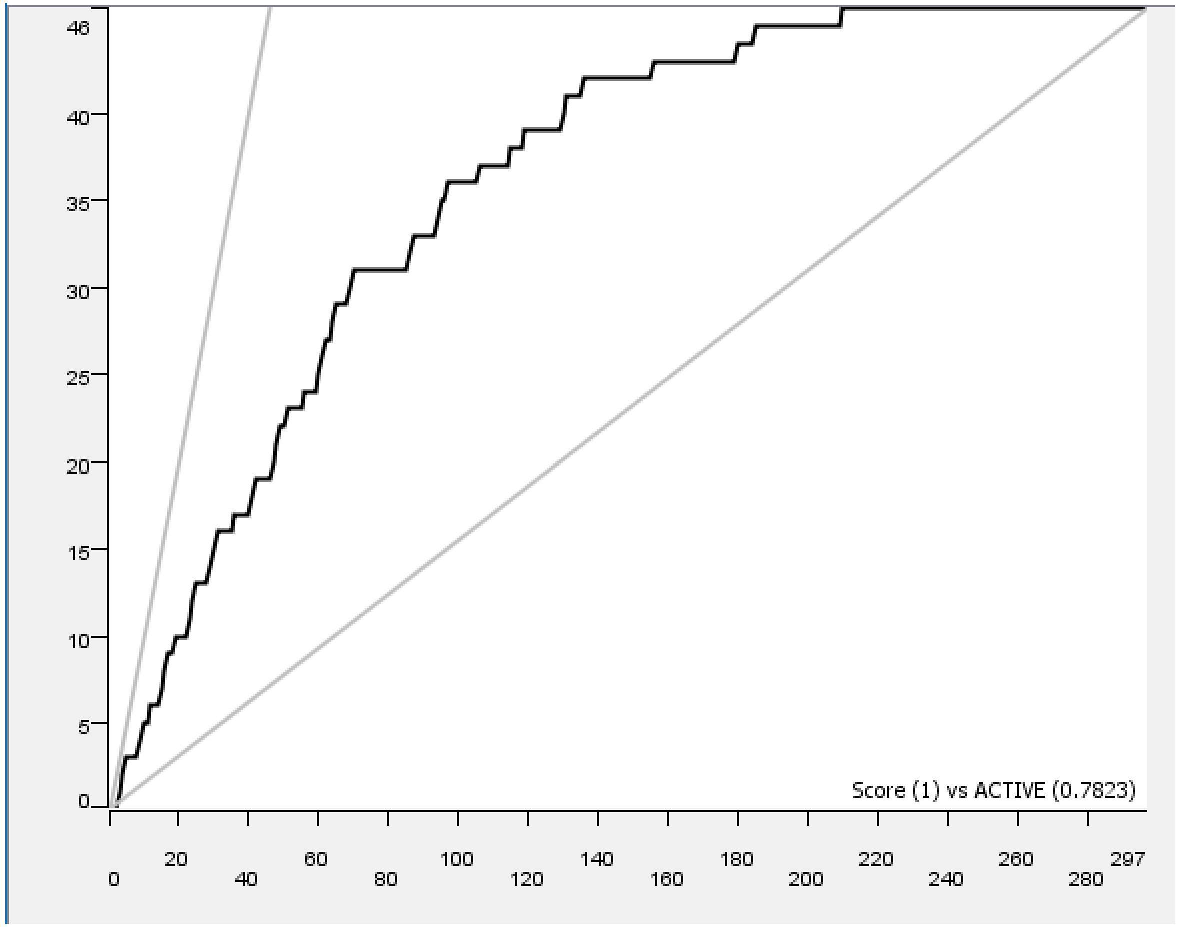

N=297

Top 10% Mean IC50	385 nM (one > 10,000 nM compound)

	Predicted Active	Predicted Inactive
Active	36	10
Inactive	61	190

<10nM DefGood in aurora B, 20% error; Random seed = 429

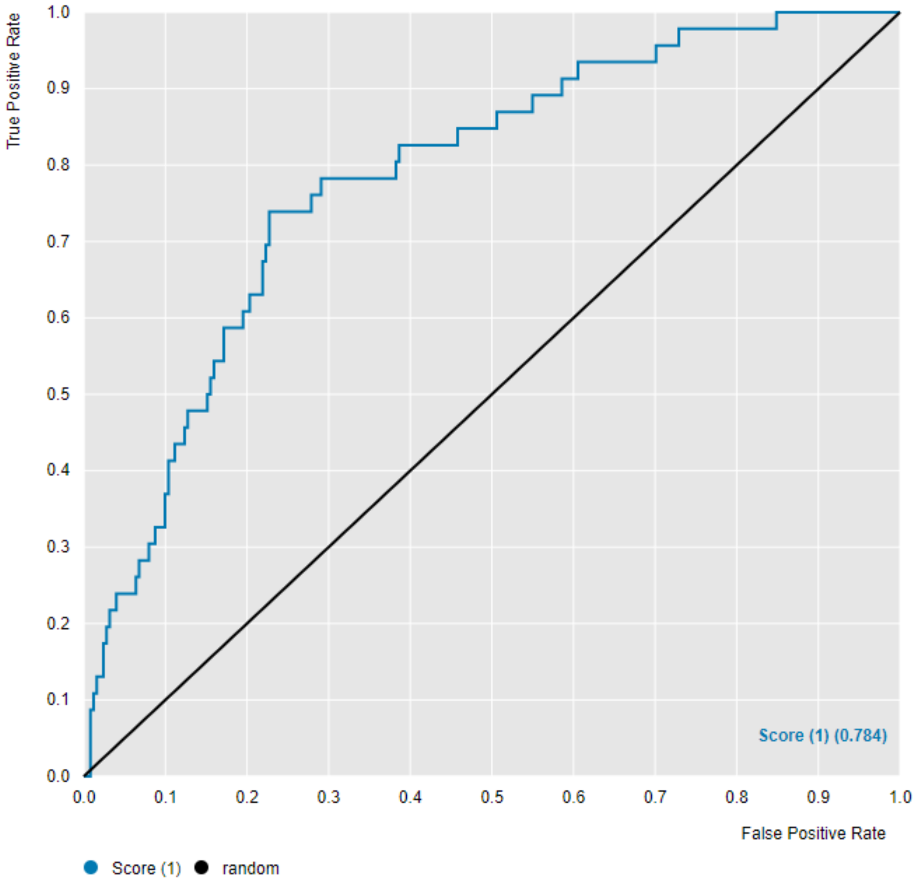

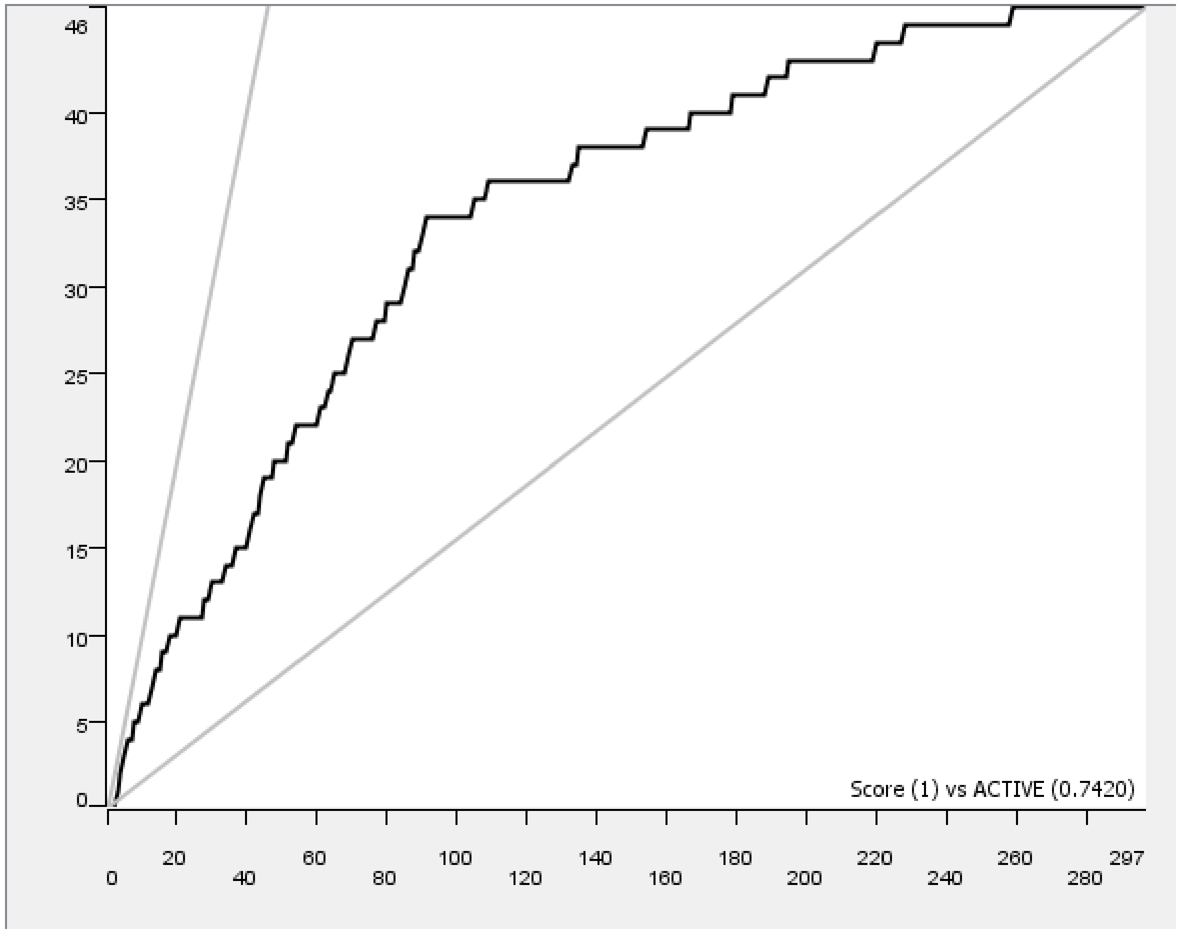

N=297

Top 10% Mean IC50	391 nM

	Predicted Active	Predicted Inactive
Active	24	22
Inactive	40	211

<10nM DefGood in aurora B, 25% error; Random seed = 429

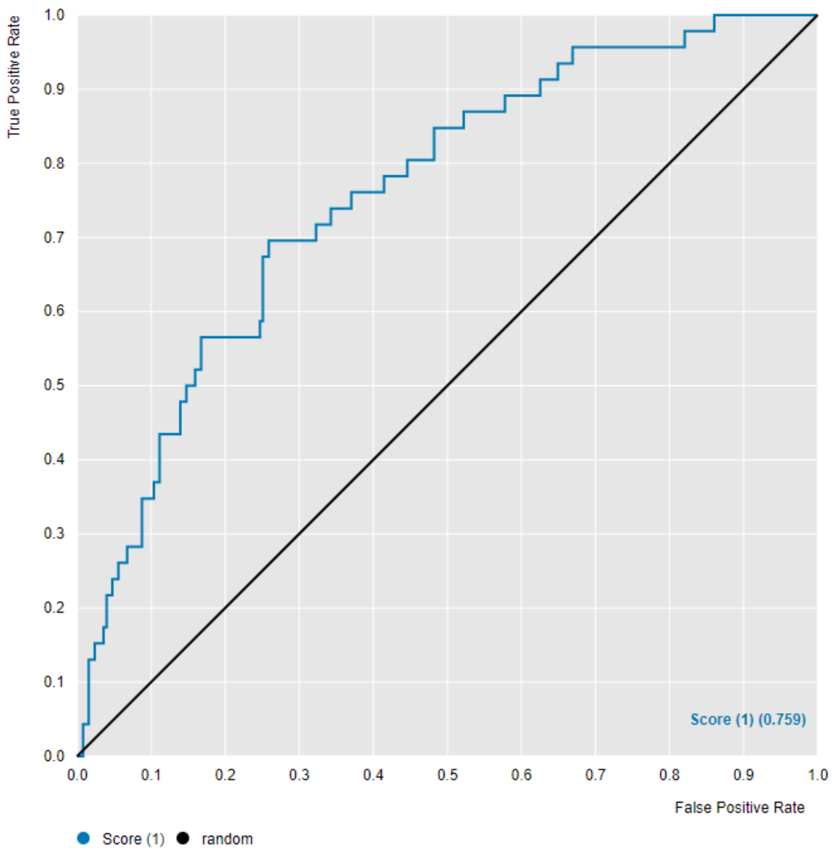

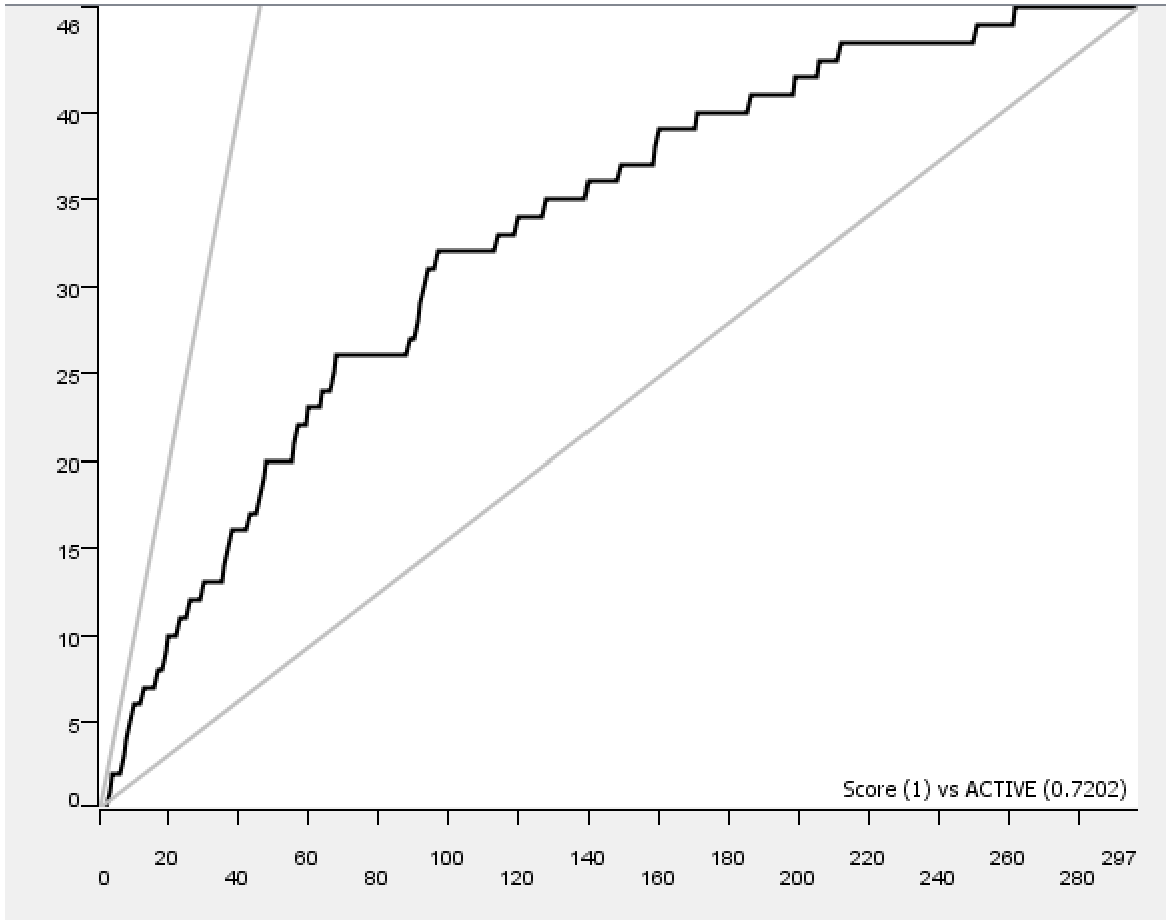

N=297

Top 10% Mean IC50	407 nM

	Predicted Active	Predicted Inactive
Active	26	20
Inactive	47	204

<10nM DefGood in aurora B, 30% error; Random seed = 429

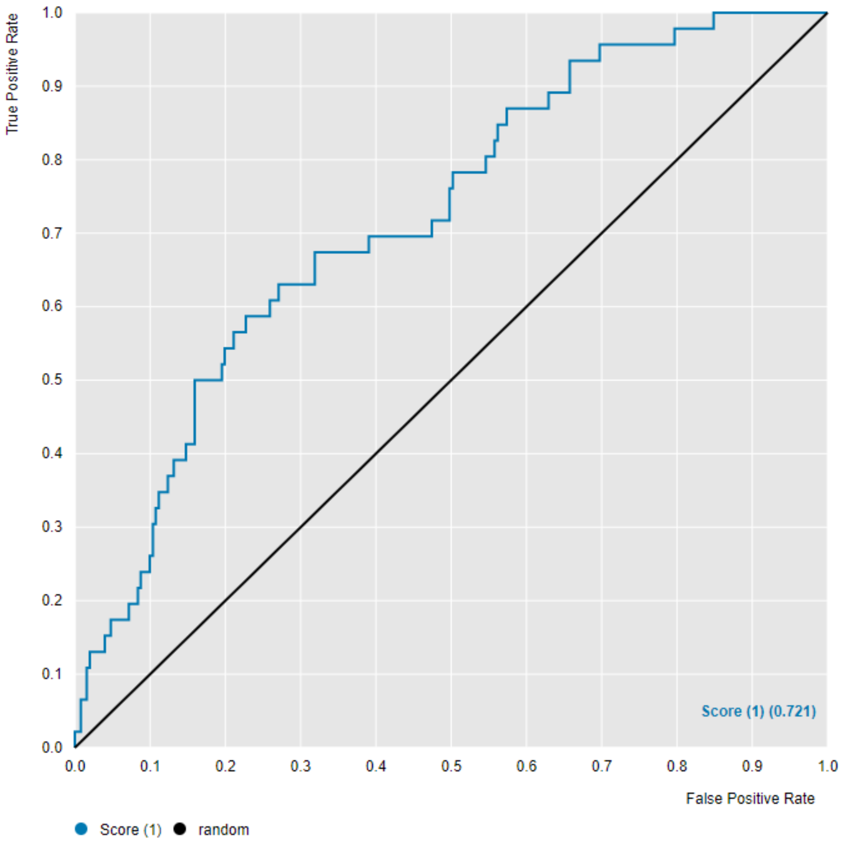

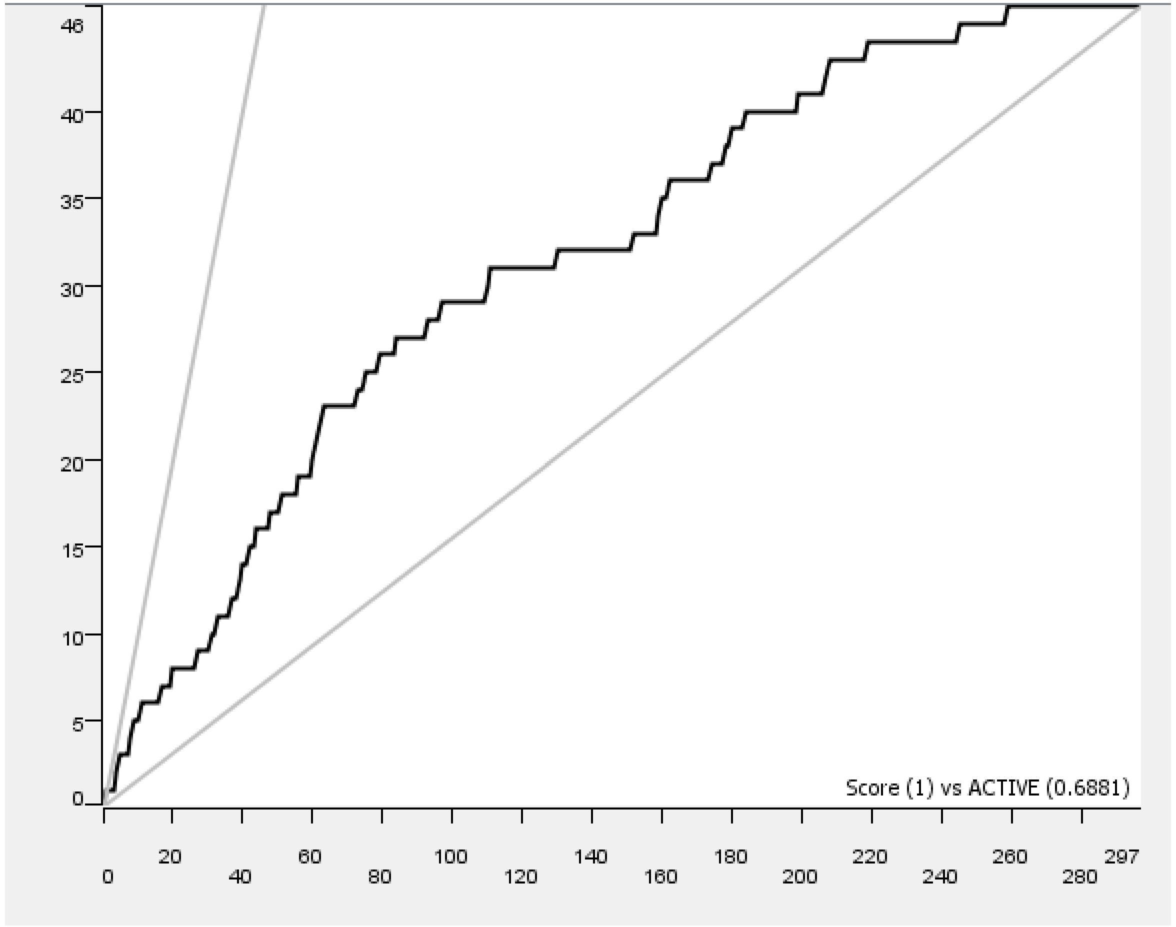

N=297

Top 10% Mean IC50	1,000 nM

	Predicted Active	Predicted Inactive
Active	23	23
Inactive	46	205

<10nM DefGood in aurora B, 10% error; Random seed = 121783

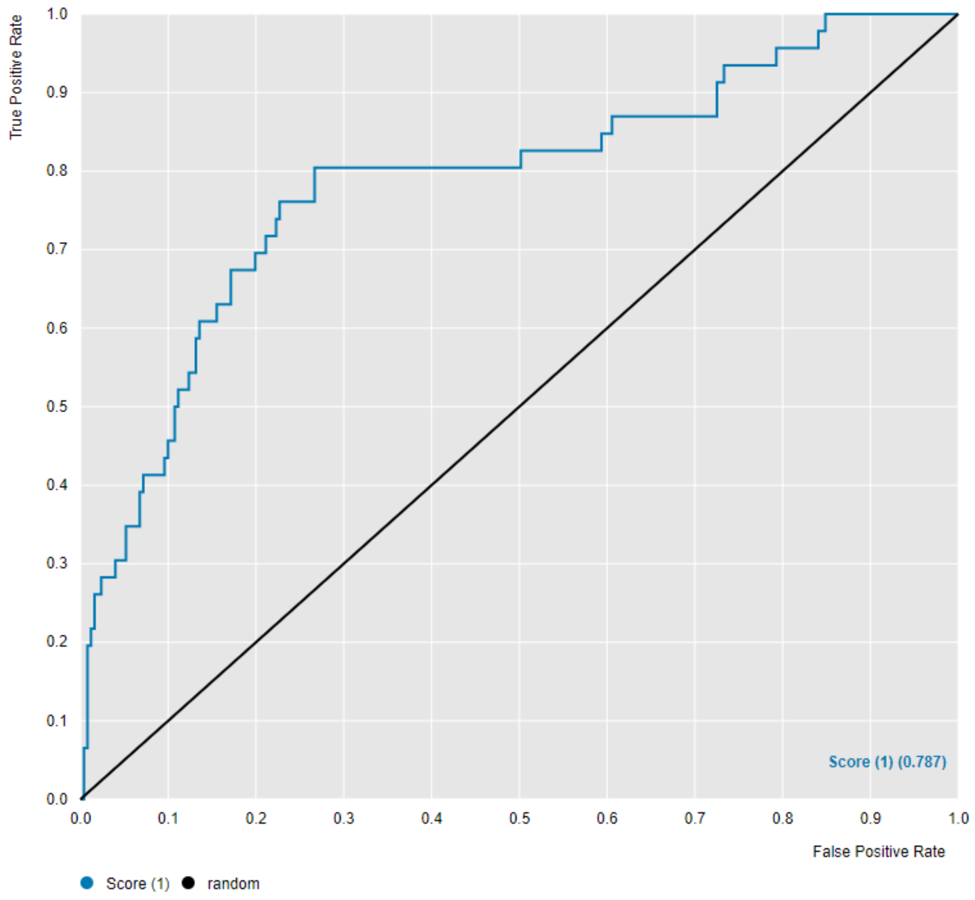

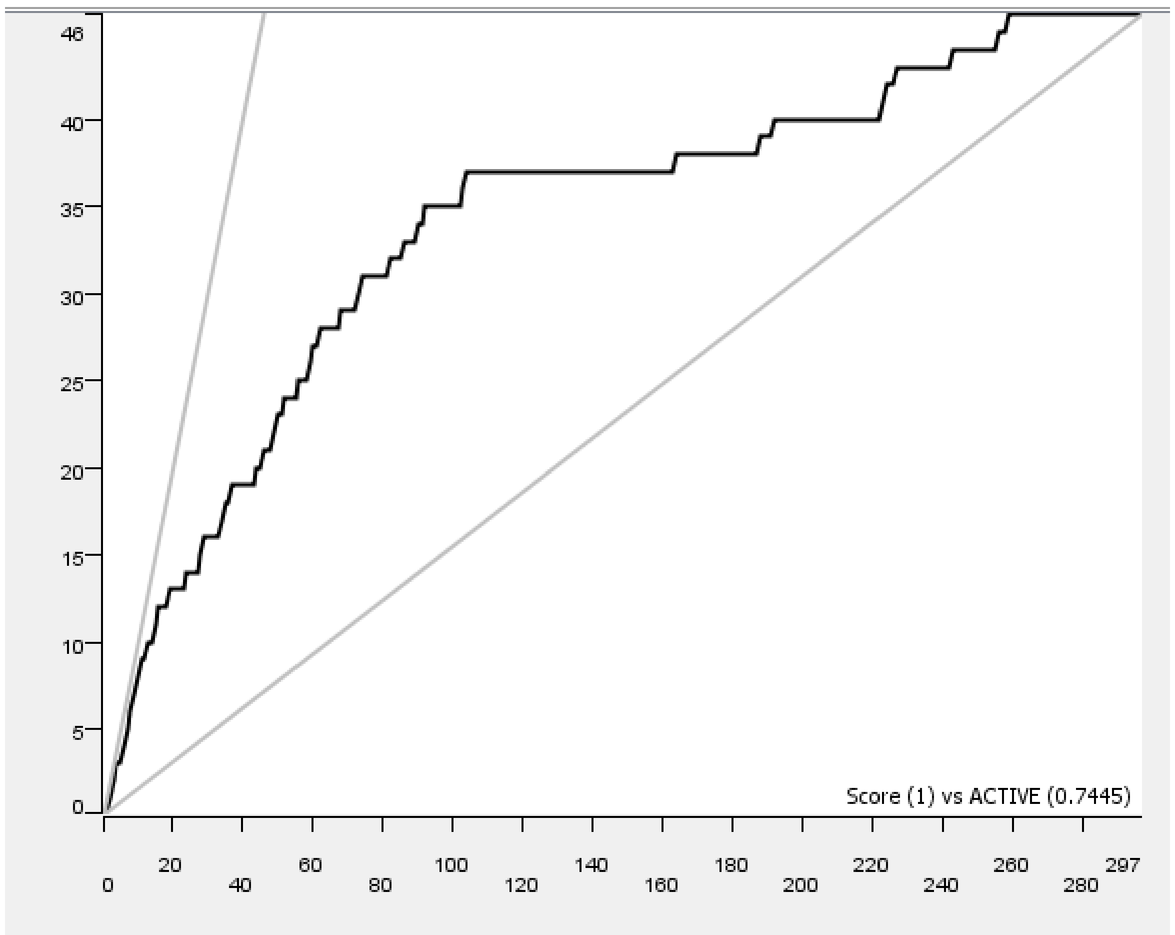

N=297

Top 10% Mean IC50	42.7 nM

	Predicted Active	Predicted Inactive
Active	23	23
Inactive	28	223

<10nM DefGood in aurora B, 20% error; Random seed = 121783

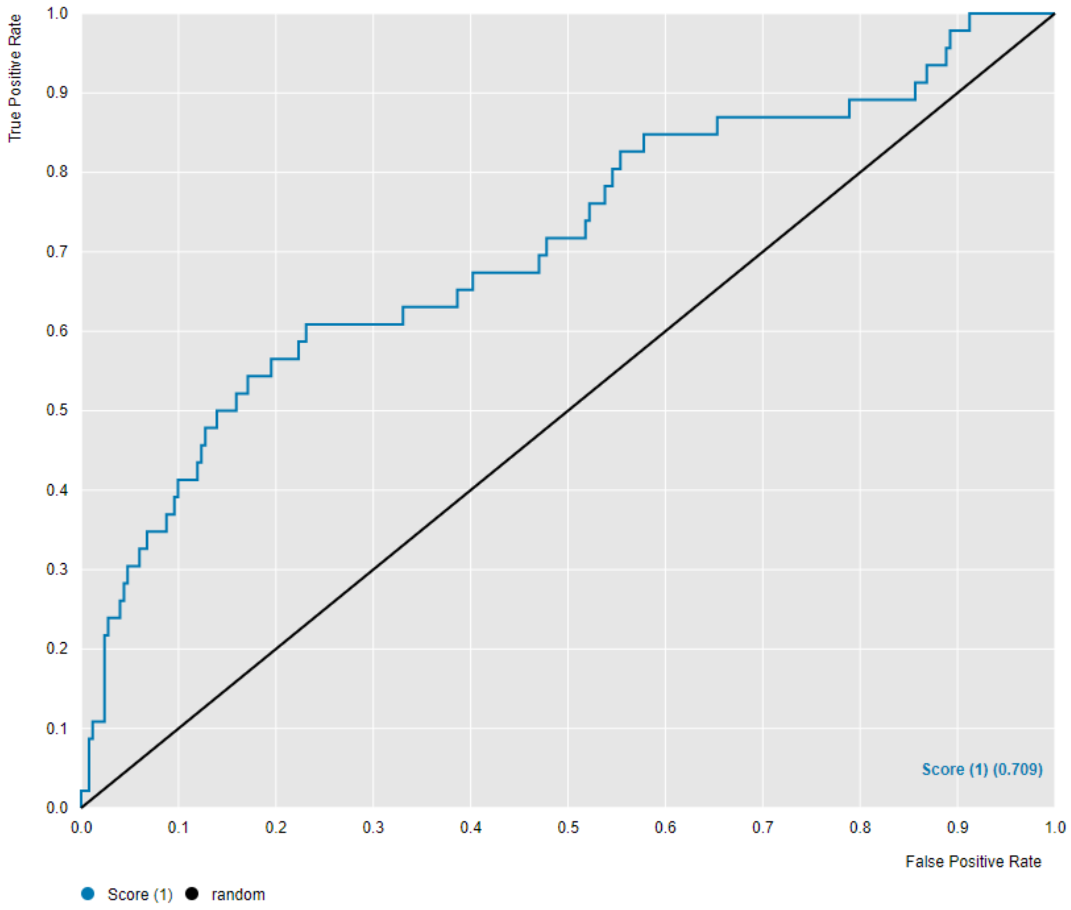

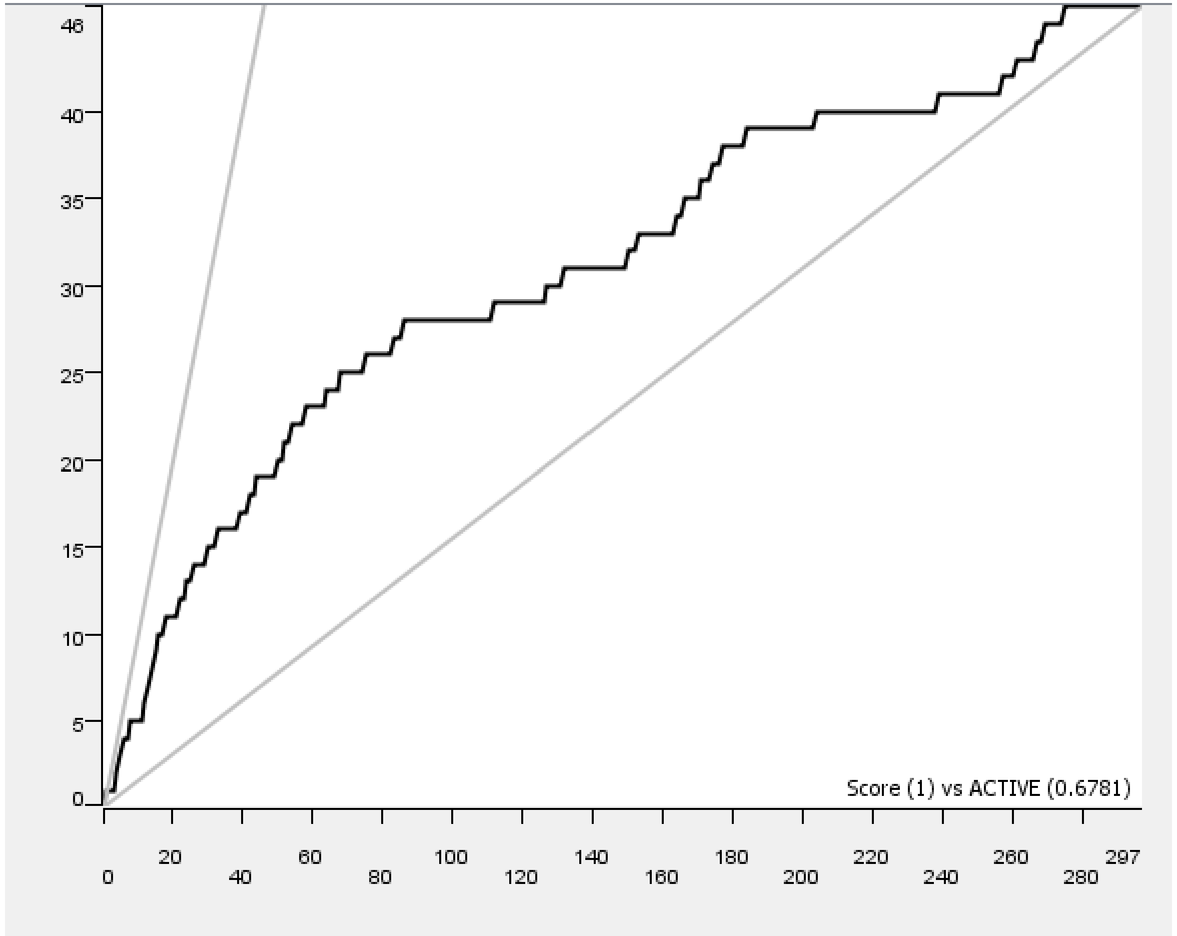

N=297

Top 10% Mean IC50	53.9 nM

	Predicted Active	Predicted Inactive
Active	27	19
Inactive	58	193

<10nM DefGood in aurora B, 25% error; Random seed = 121783

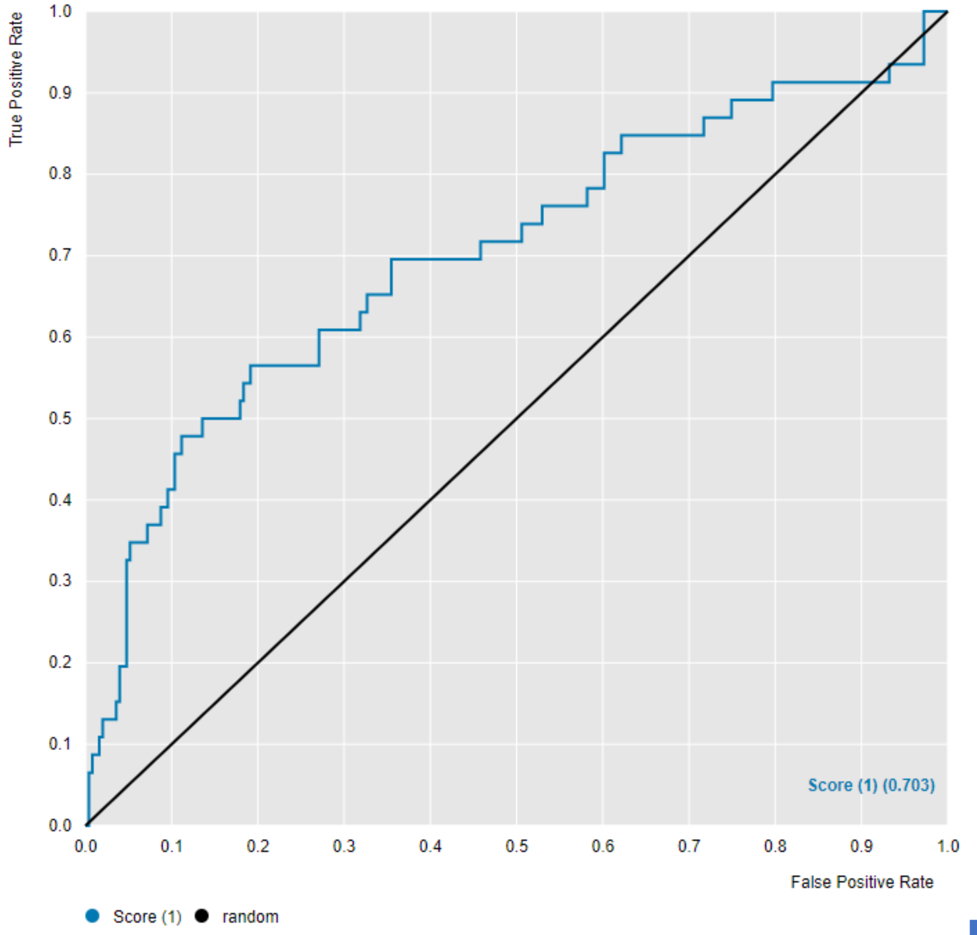

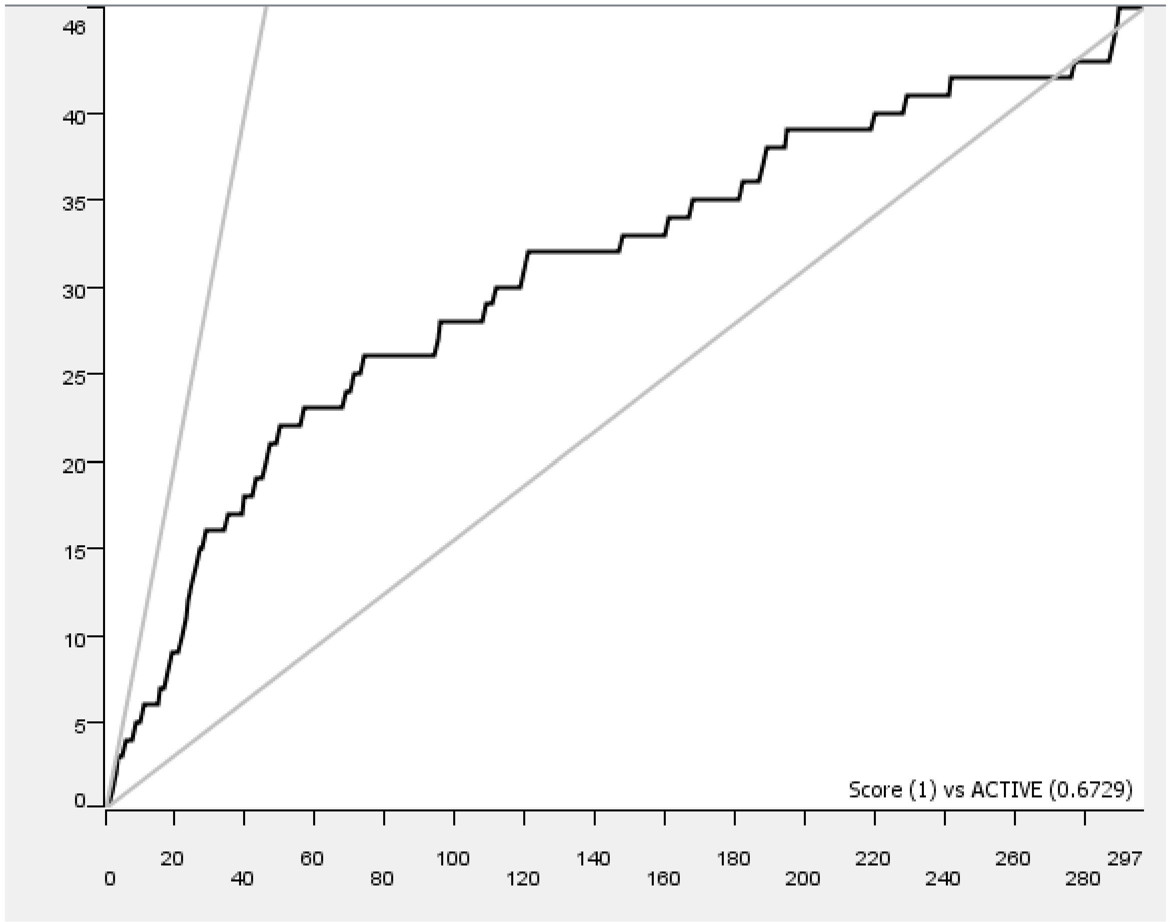

N=297

Top 10% Mean IC50	83.4 nM

	Predicted Active	Predicted Inactive
Active	26	20
Inactive	53	198

<10nM DefGood in aurora B, 30% error; Random seed = 121783

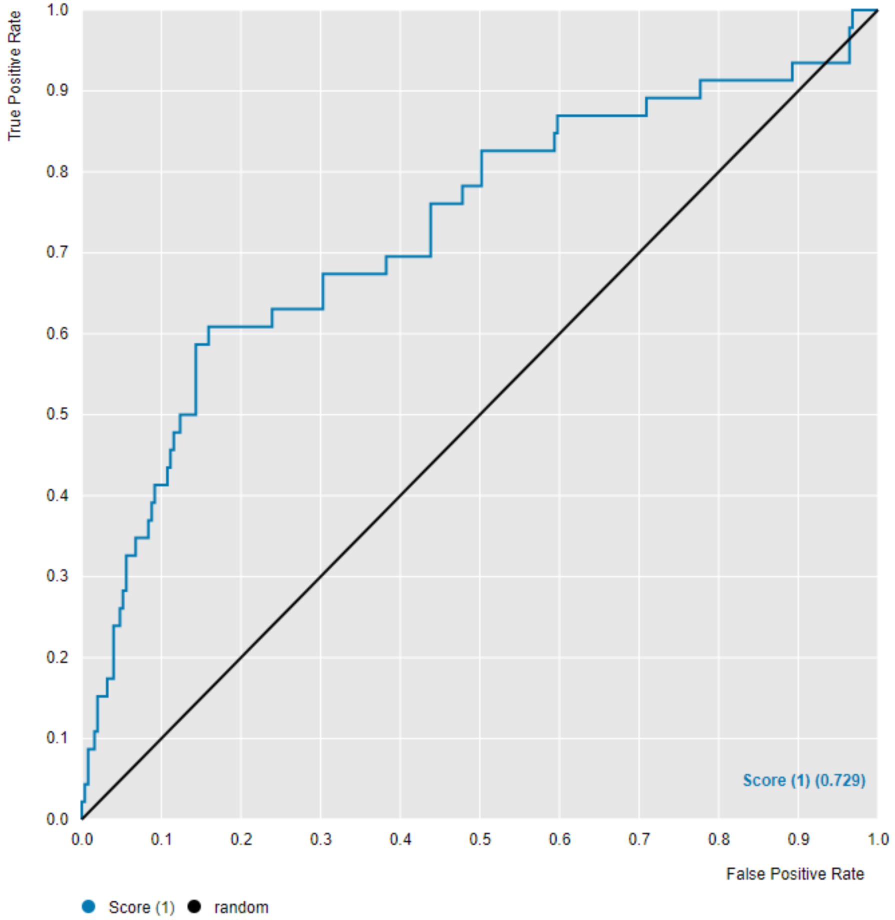

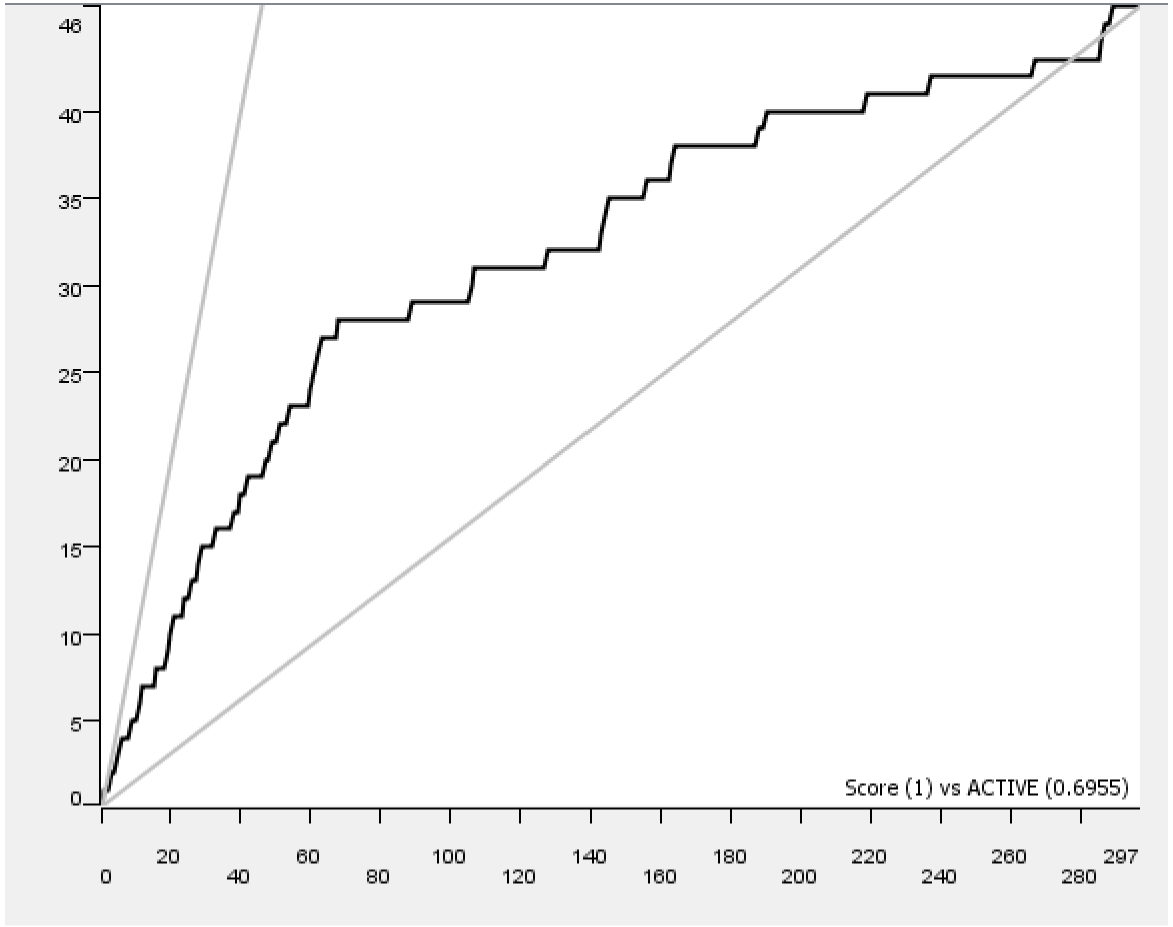

N=297

Top 10% Mean IC50	67.4 nM

	Predicted Active	Predicted Inactive
Active	28	18
Inactive	52	199

<10nM DefGood in aurora B, 35% error; Random seed = 121783

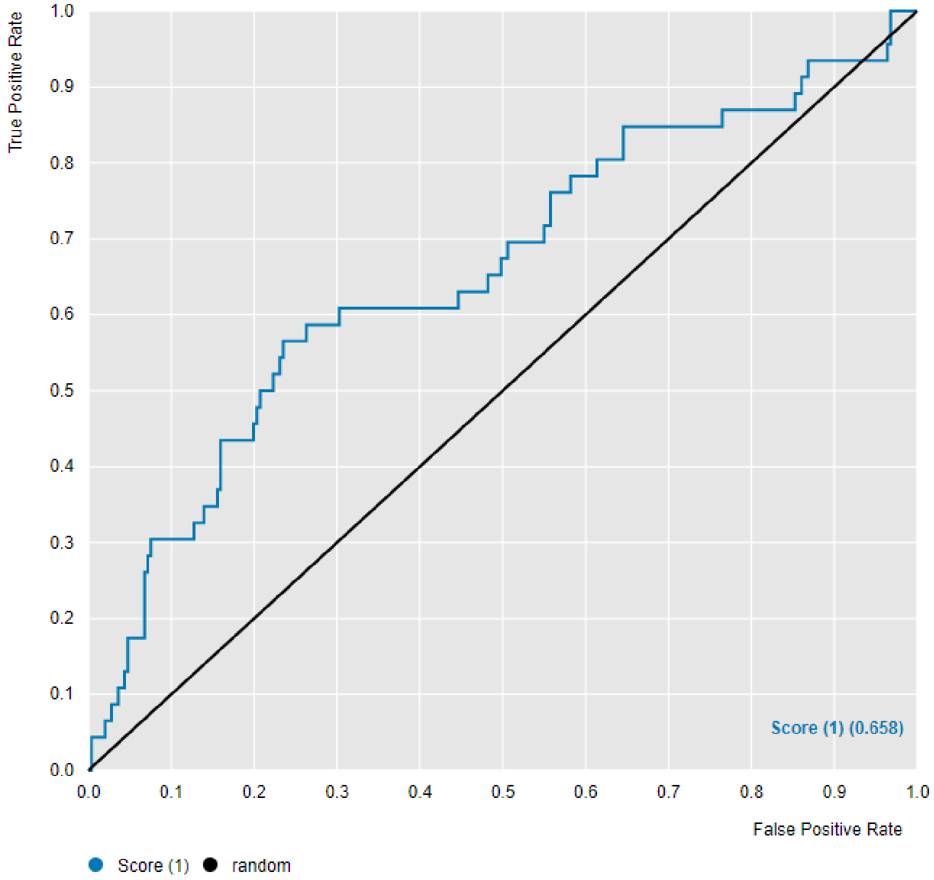

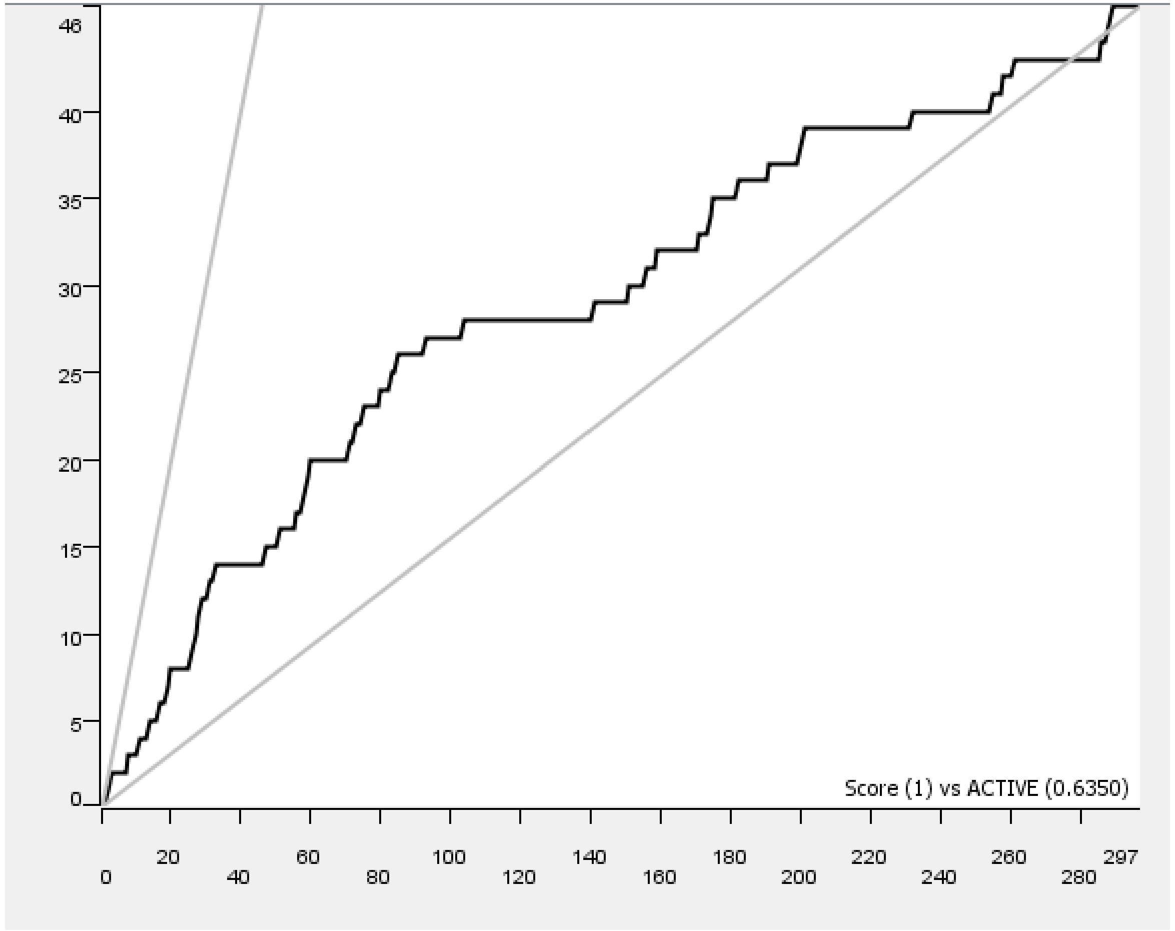

N=297

Top 10% Mean IC50	67.4 nM

	Predicted Active	Predicted Inactive
Active	28	18
Inactive	52	199

Conclusion - NBN

- An NBN could be generated for aurora B kinase with a decision value of <10 nM as defined as active. The predictivity of the algorithm could be preserved in three random train/test cycles. The point of failure was 30%, 30% and 35% error.

RF Error Tolerance- <10 nM DefGood in Aurora B

- 0-50% absolute error

RF- <10nM DefGood in aurora B, 10% error

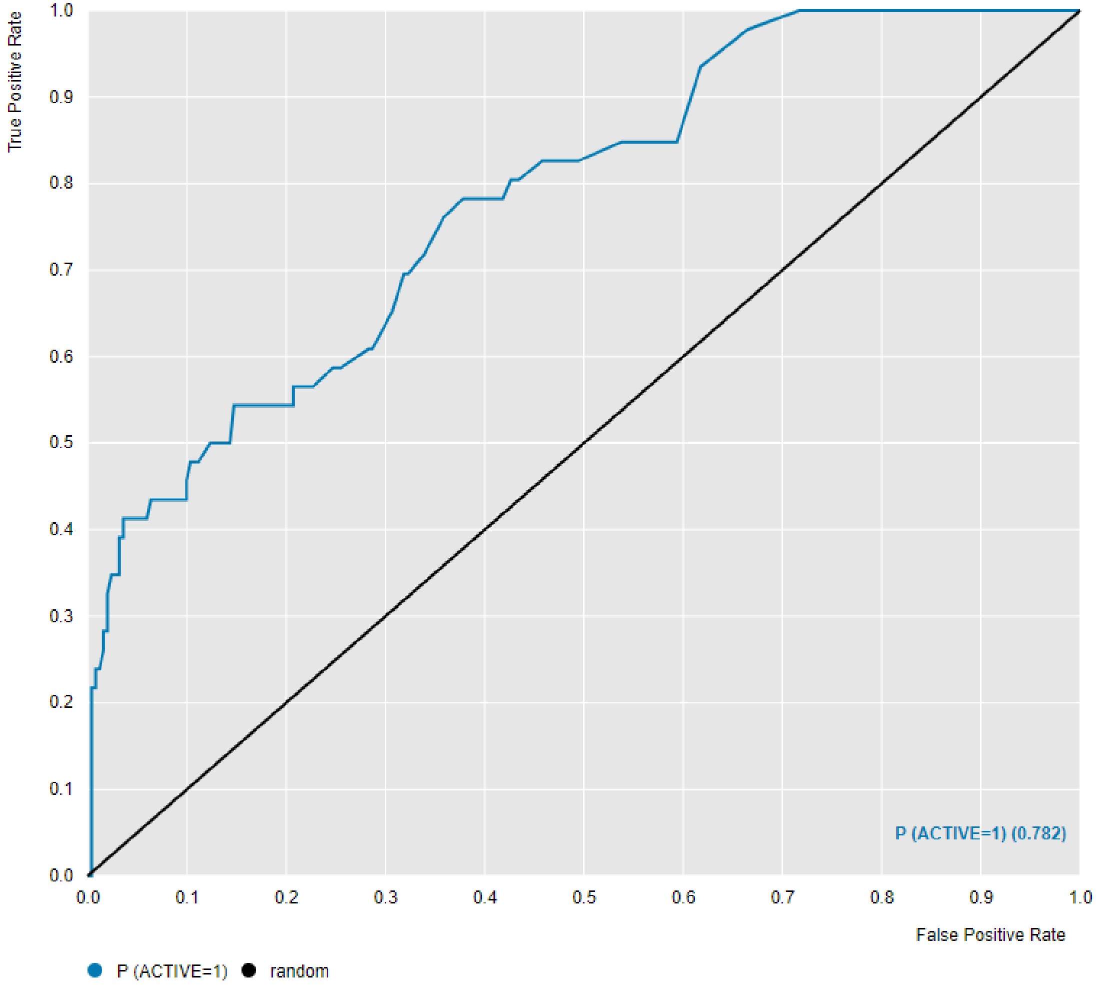

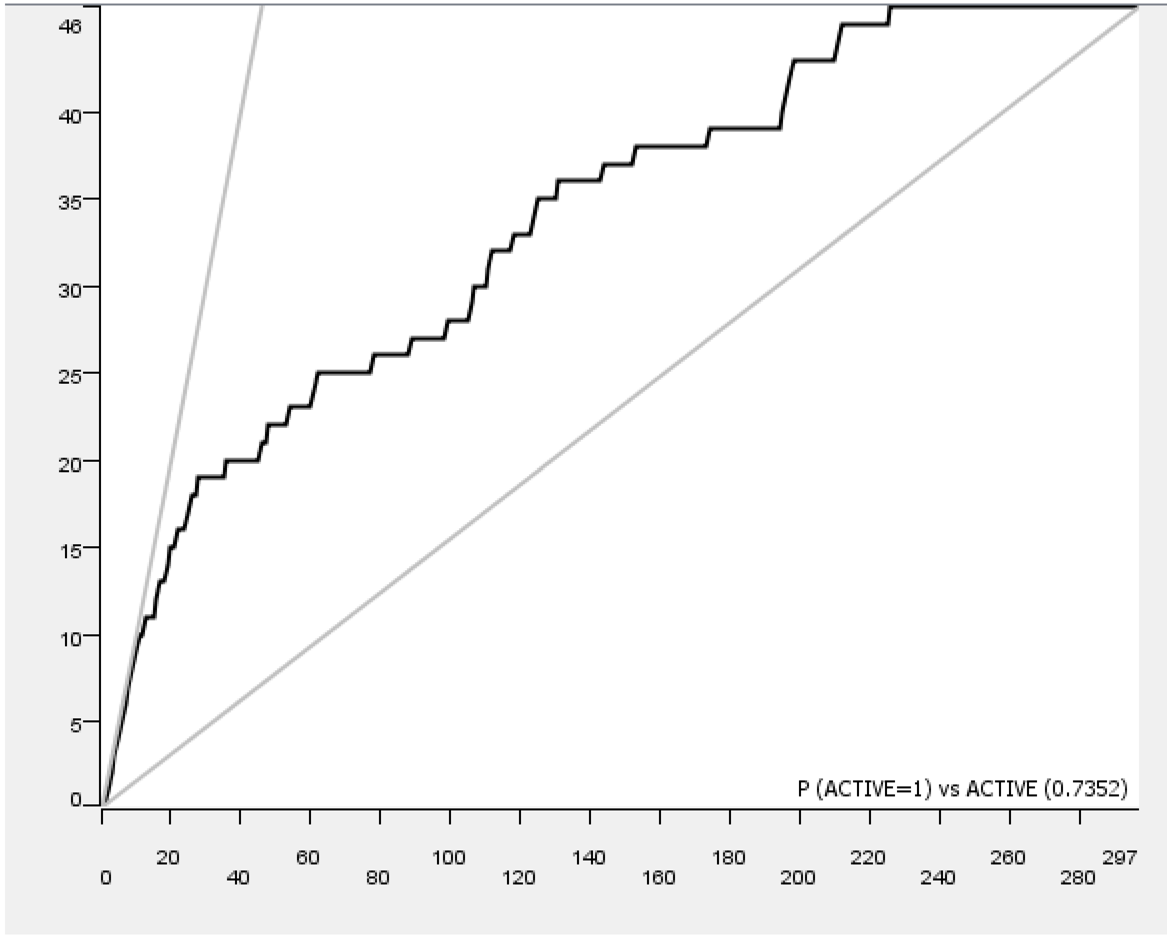

N=297

Top 10% Mean IC50	93.1 nM

	Predicted Active	Predicted Inactive
Active	19	27
Inactive	15	236

RF- <10nM DefGood in aurora B, 15% error

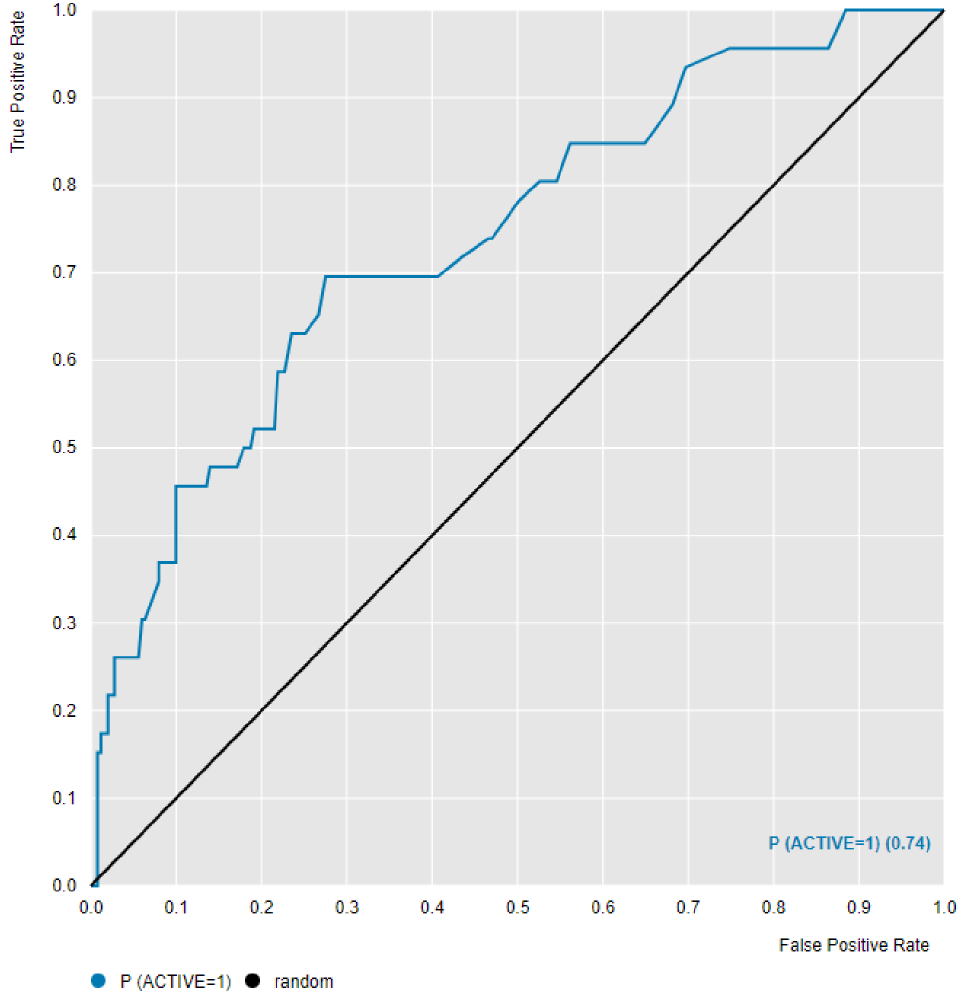

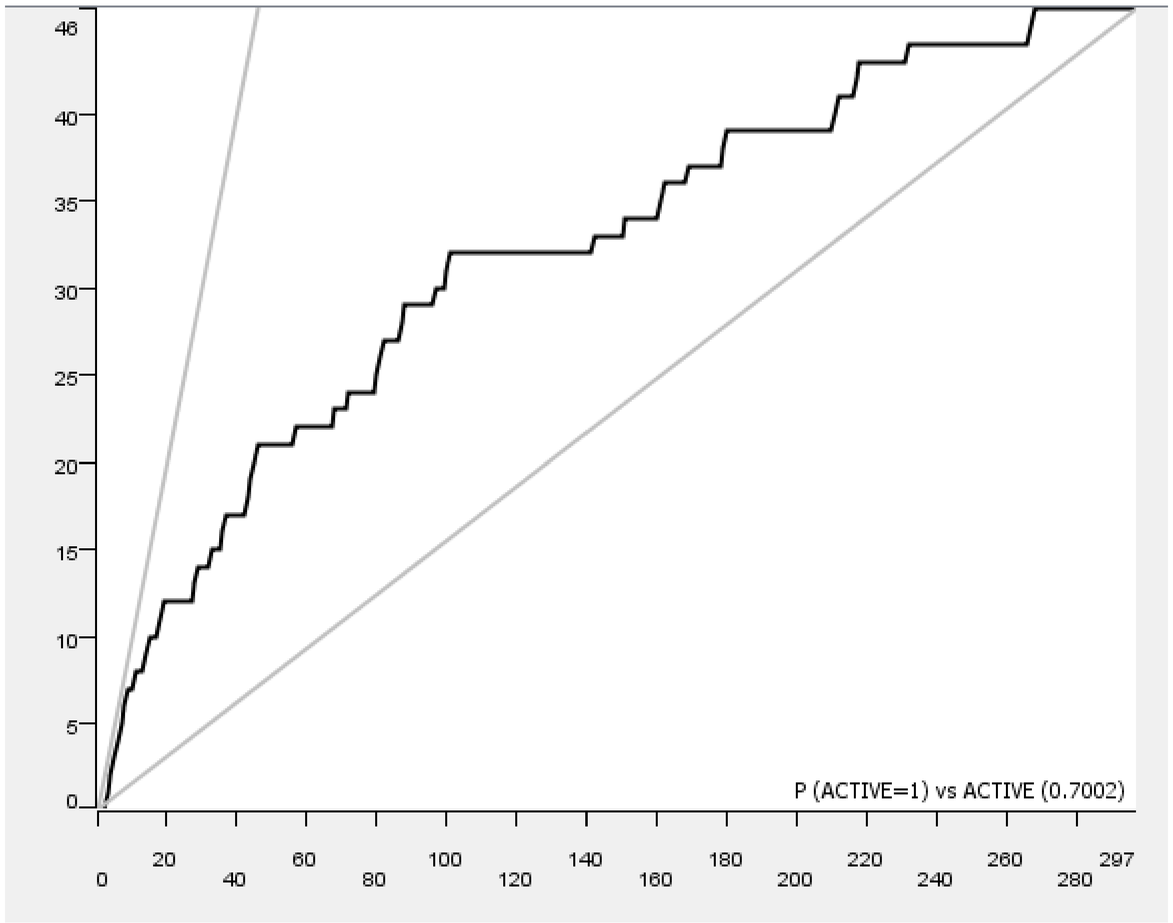

N=297

**Top 10% Mean IC50**	**128 nM**

	Predicted Active	Predicted Inactive
Active	17	29
Inactive	24	227

RF- <10nM DefGood in aurora B, 20% error

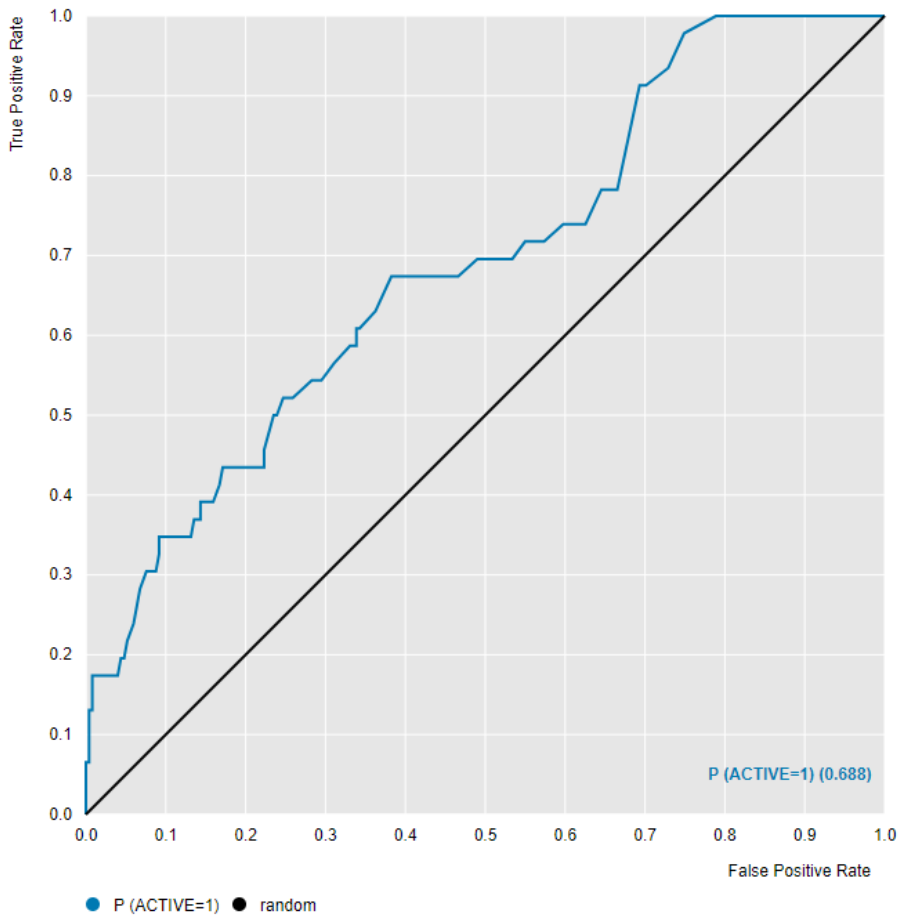

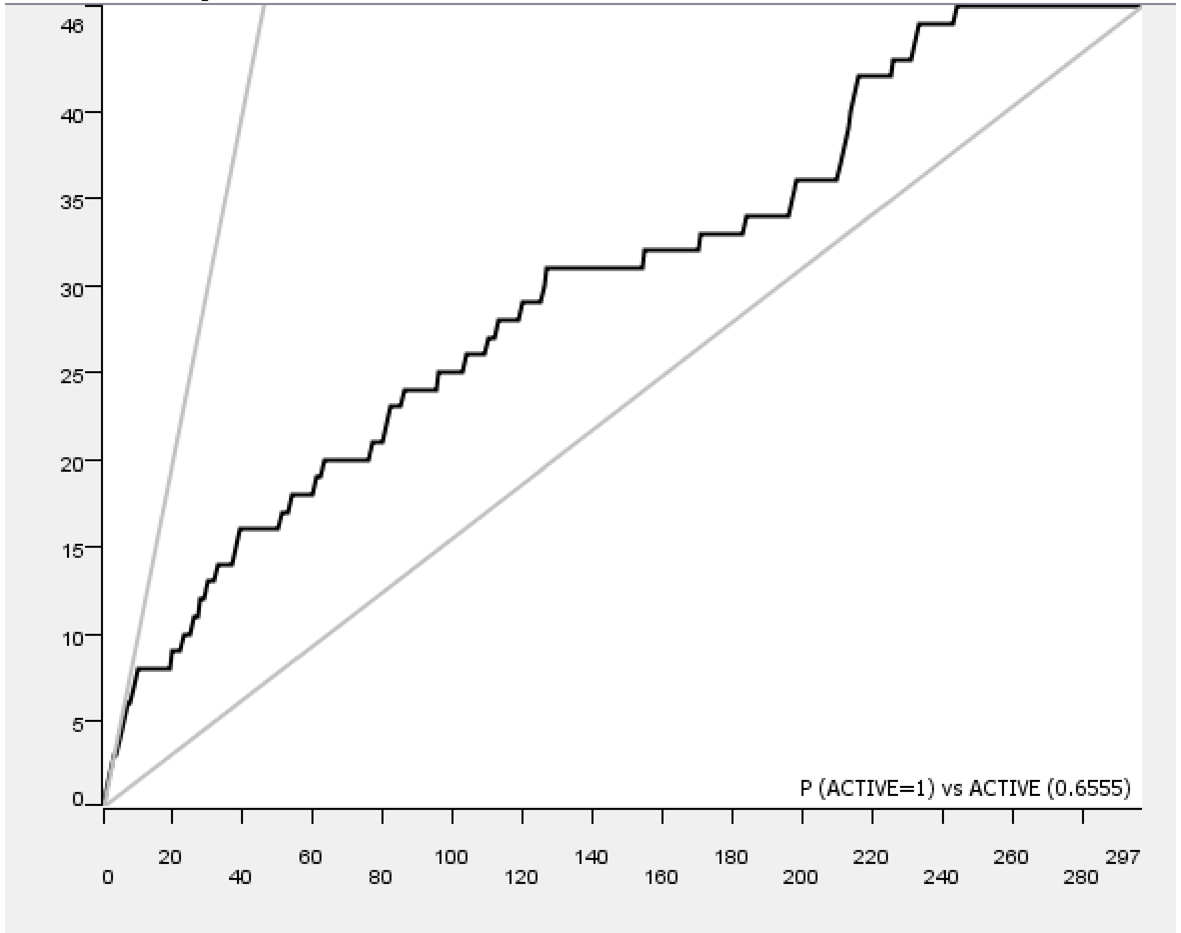

N=297

Top 10% Mean IC50	534 nM

	Predicted Active	Predicted Inactive
Active	17	29
Inactive	36	215

<10nM DefGood in aurora B, 10% error; Random seed = 429

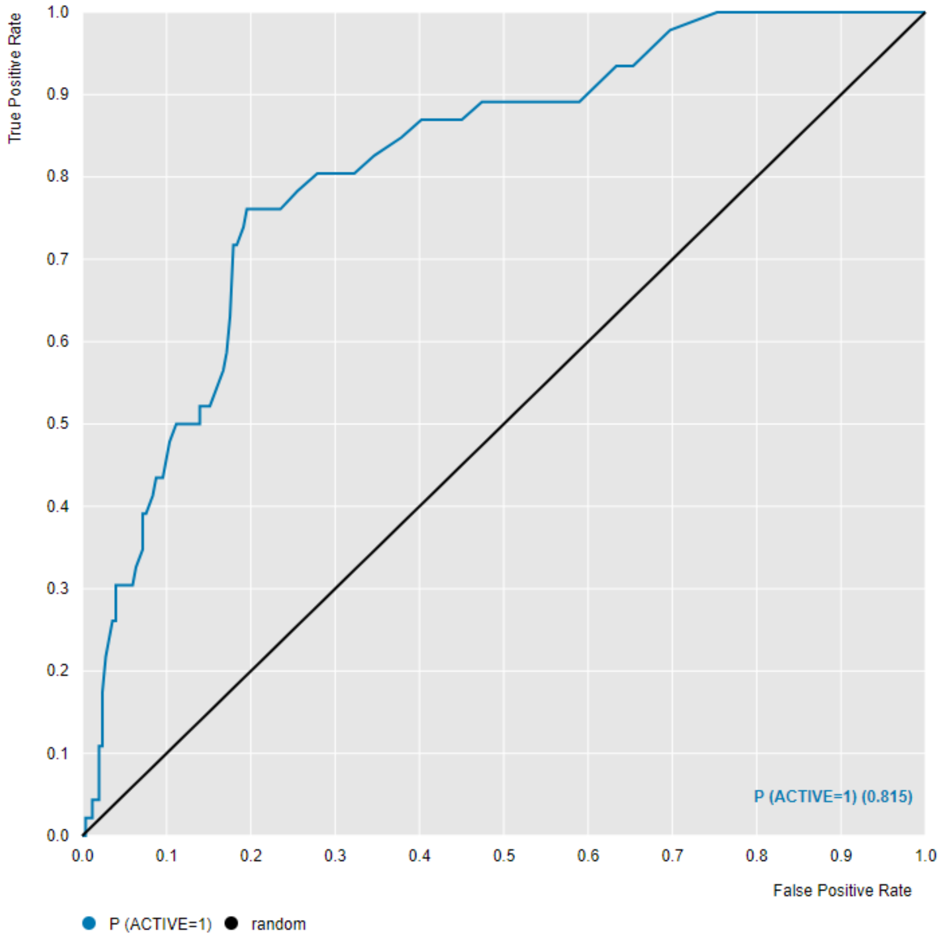

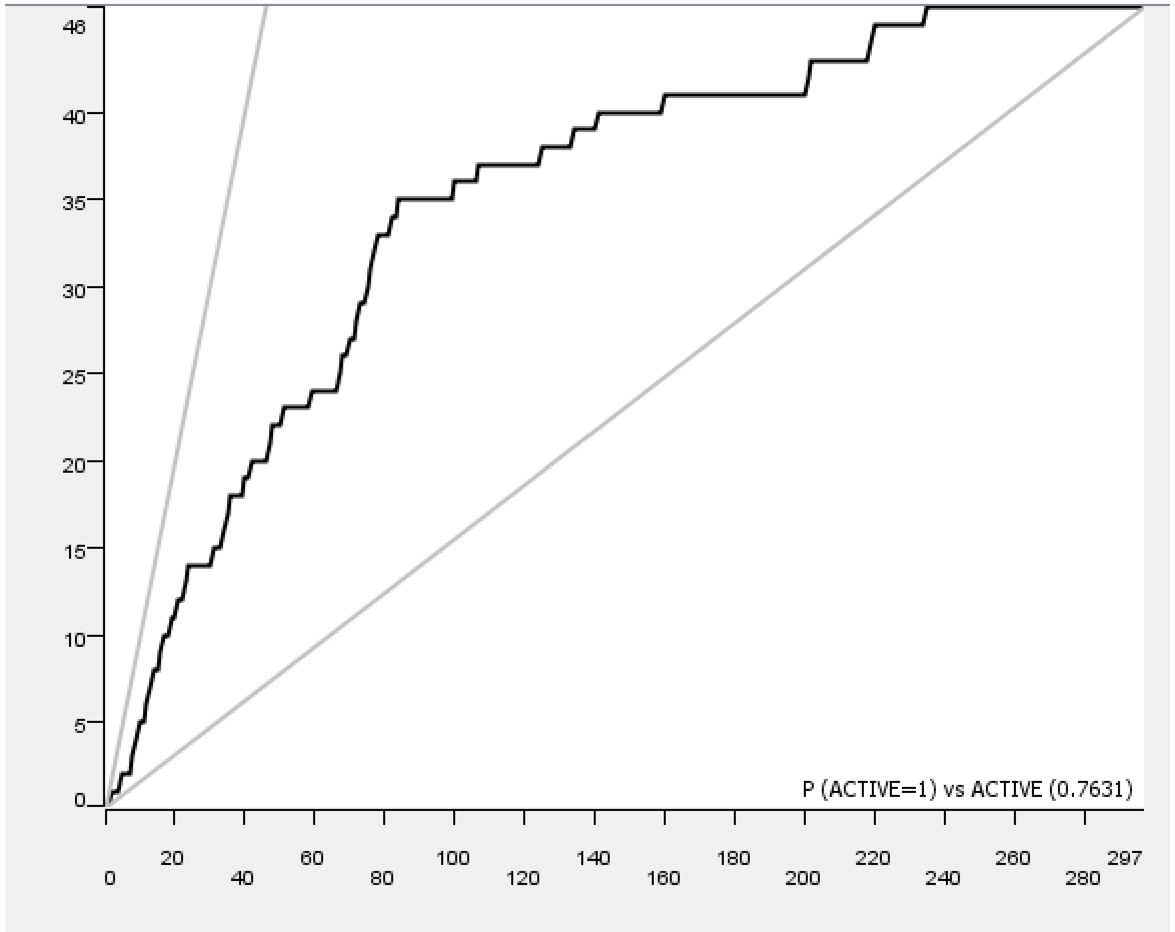

N=297

Top 10% Mean IC50	365 nM (one > 10,000 nM compound)

	Predicted Active	Predicted Inactive
Active	19	27
Inactive	21	230

<10nM DefGood in aurora B, 15% error; Random seed = 429

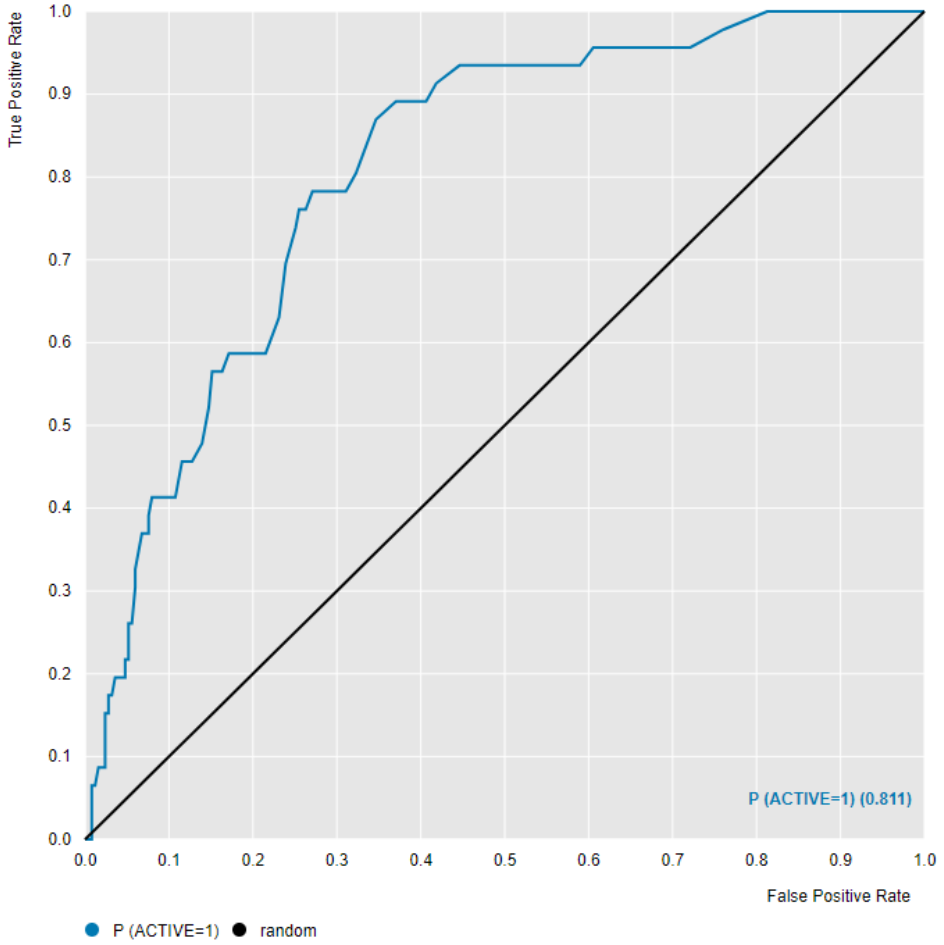

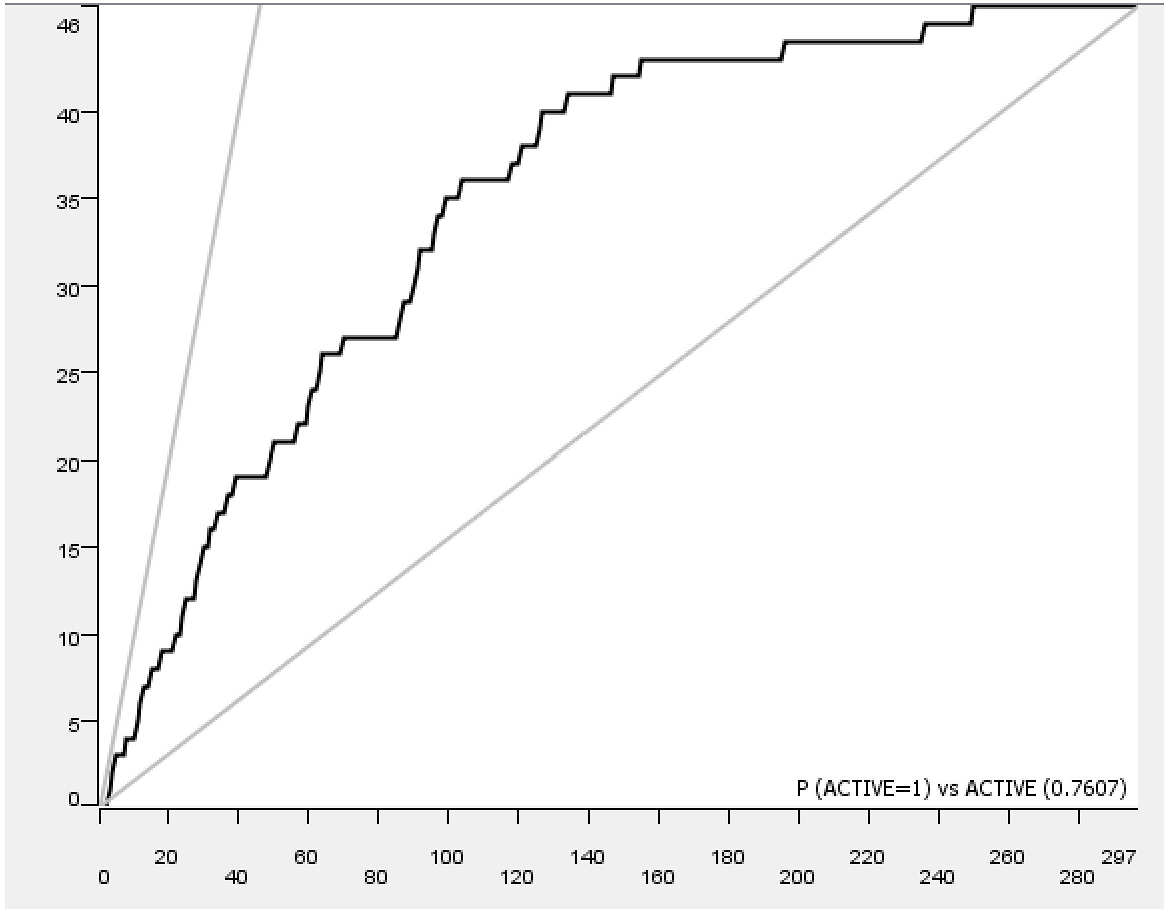

N=297

Top 10% Mean IC50	656 nM

	Predicted Active	Predicted Inactive
Active	19	27
Inactive	24	227

<10nM DefGood in aurora B, 20% error; Random seed = 429

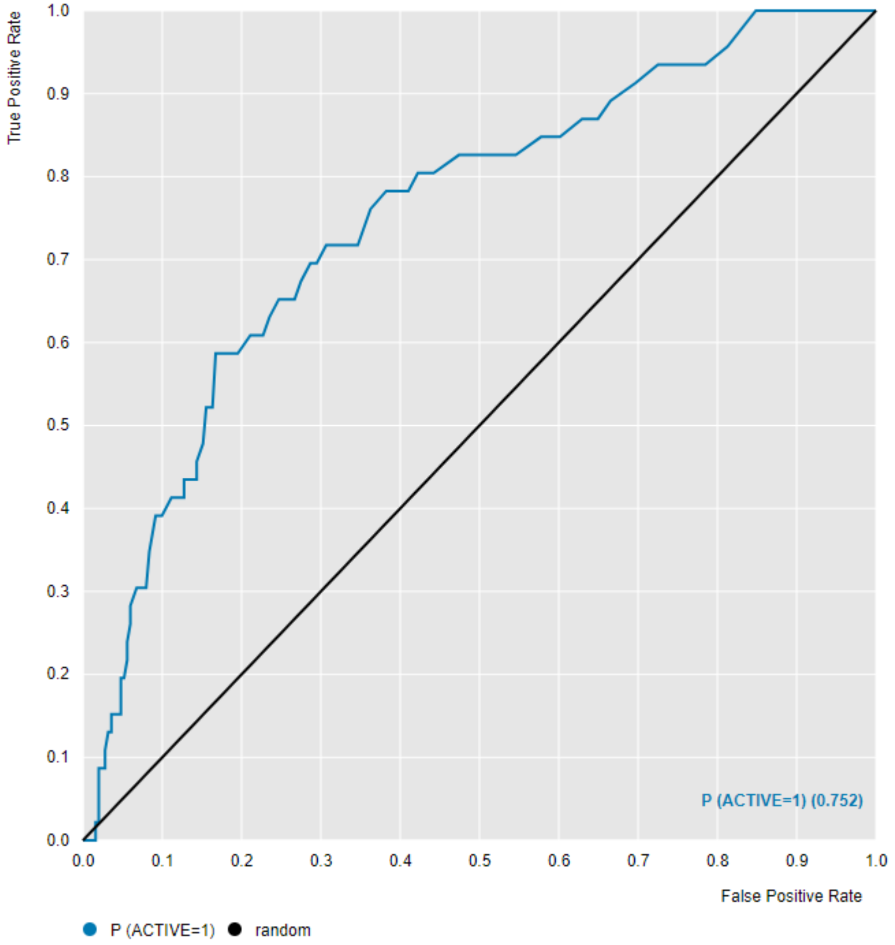

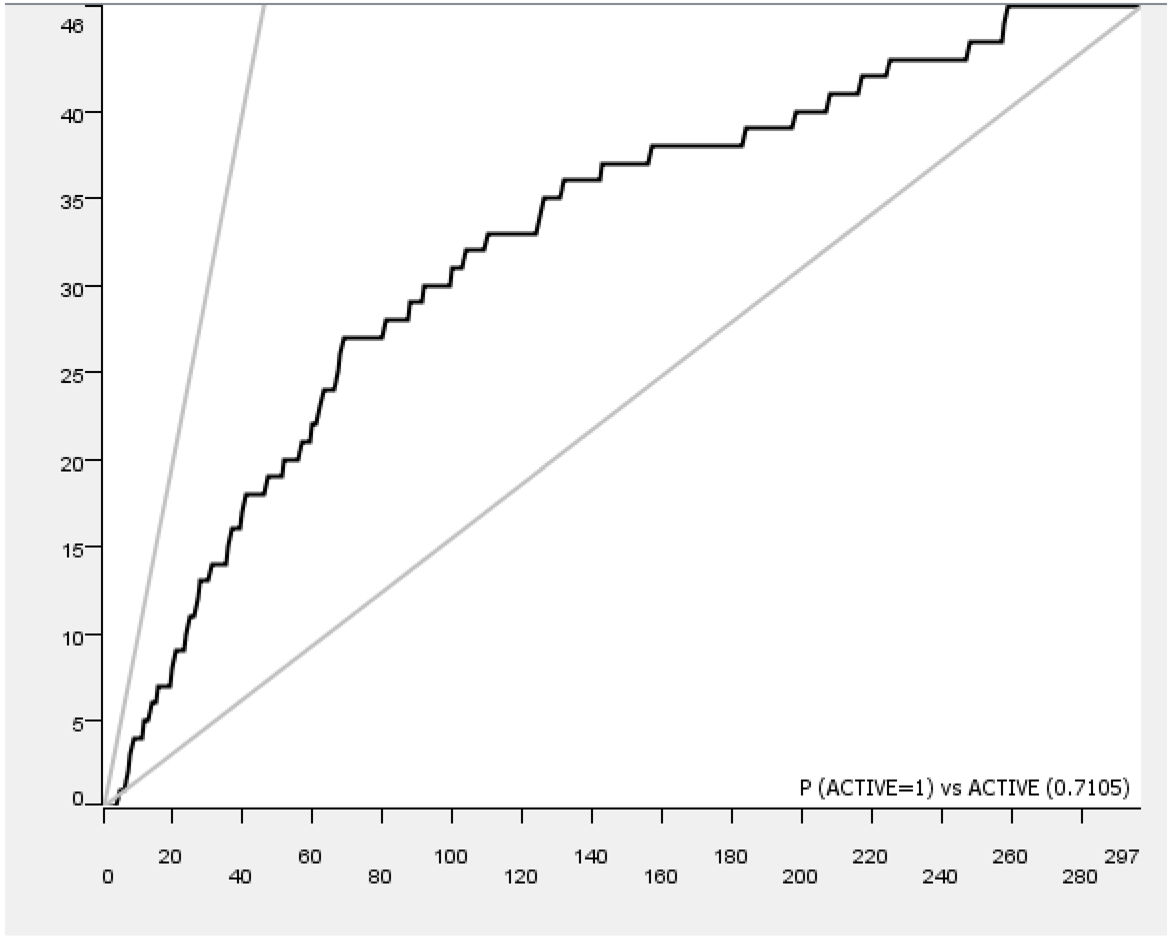

N=270

**Top 10% Mean IC50**	**1,000 nM**

	Predicted Active	Predicted Inactive
Active	20	26
Inactive	35	216

<10nM DefGood in aurora B, 10% error; Random seed = 121783

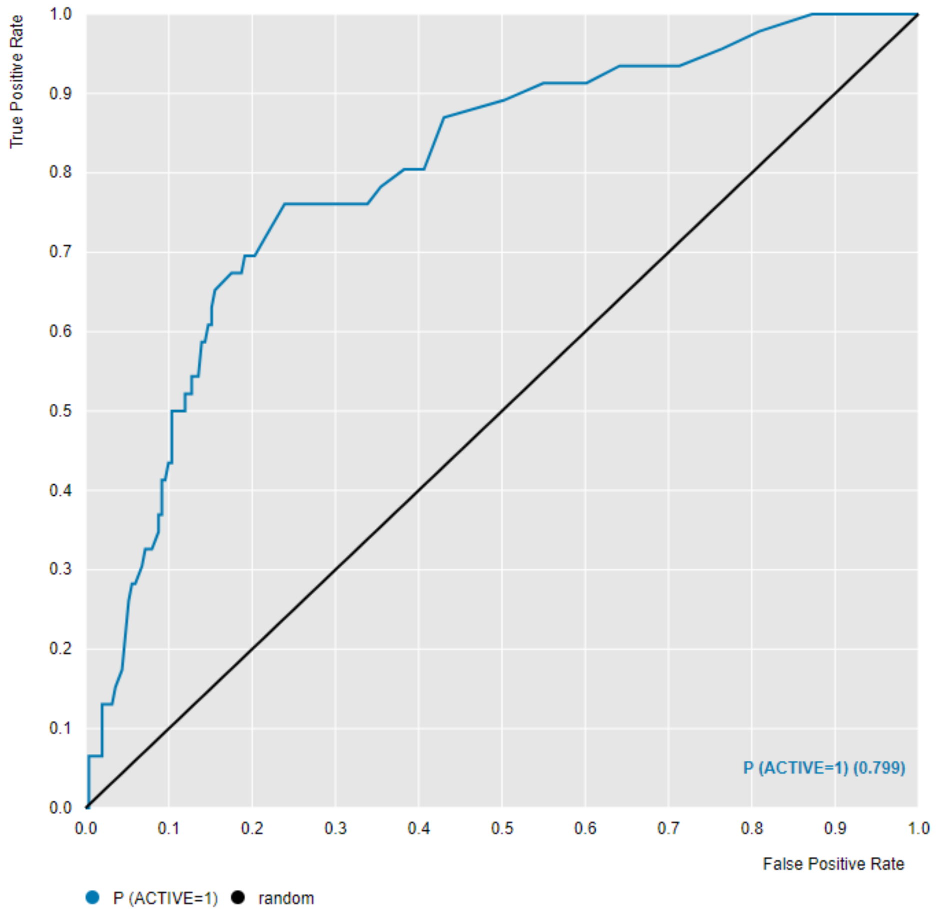

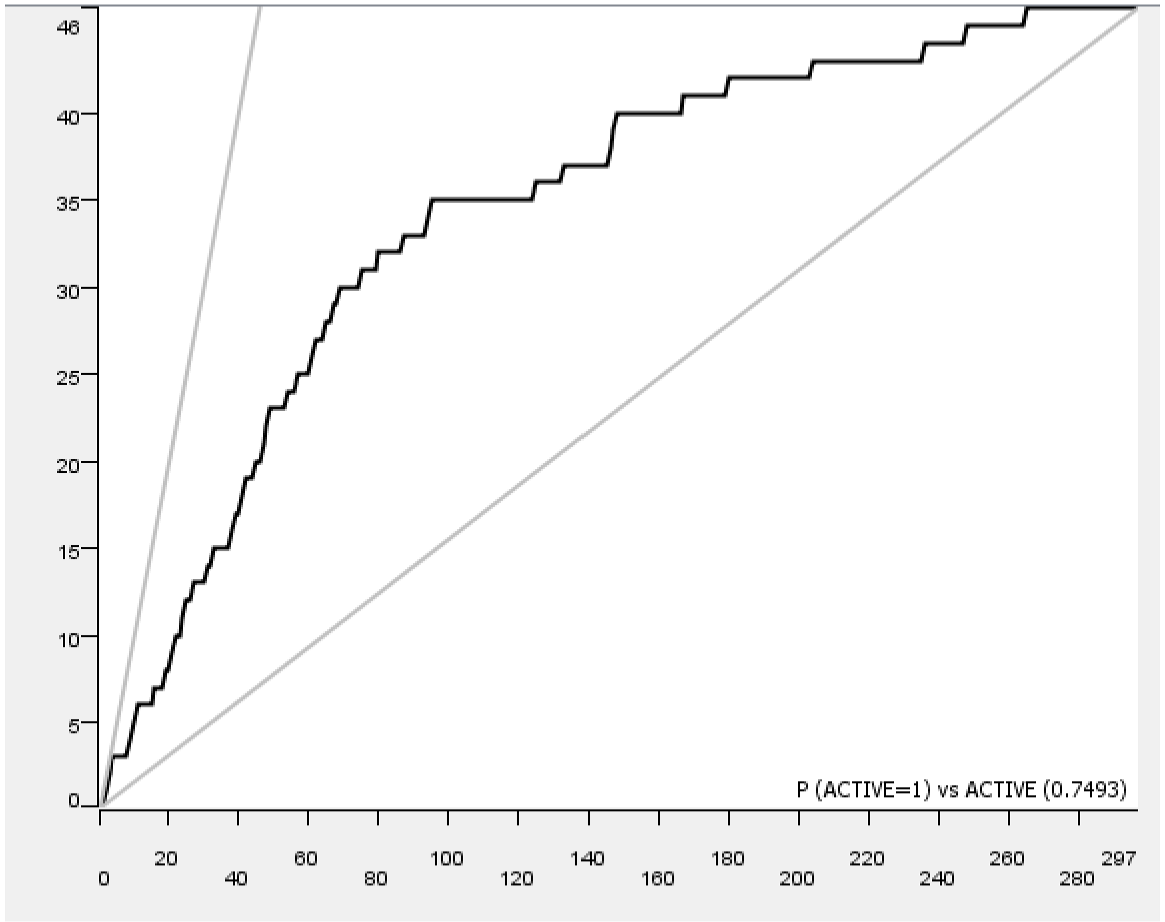

N=297

**Top 10% Mean IC50**	**221 nM**

	**Predicted Active**	**Predicted Inactive**
Active	17	29
Inactive	22	229

<10nM DefGood in aurora B, 20% error; Random seed = 121783

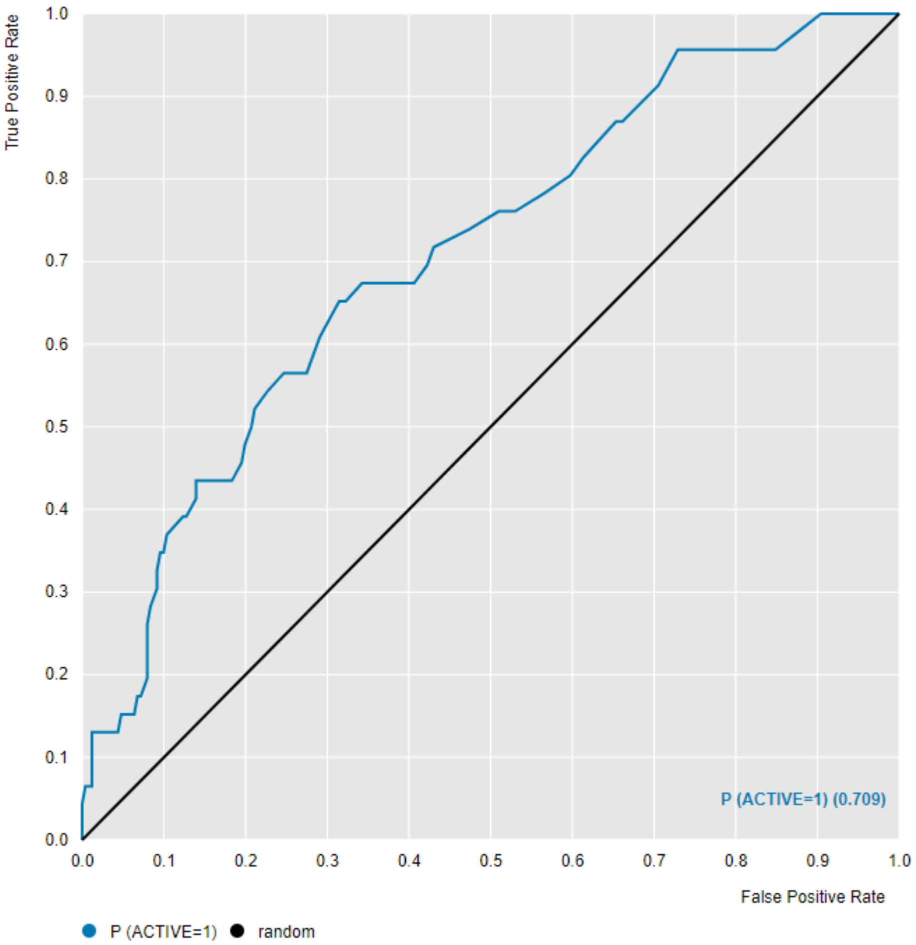

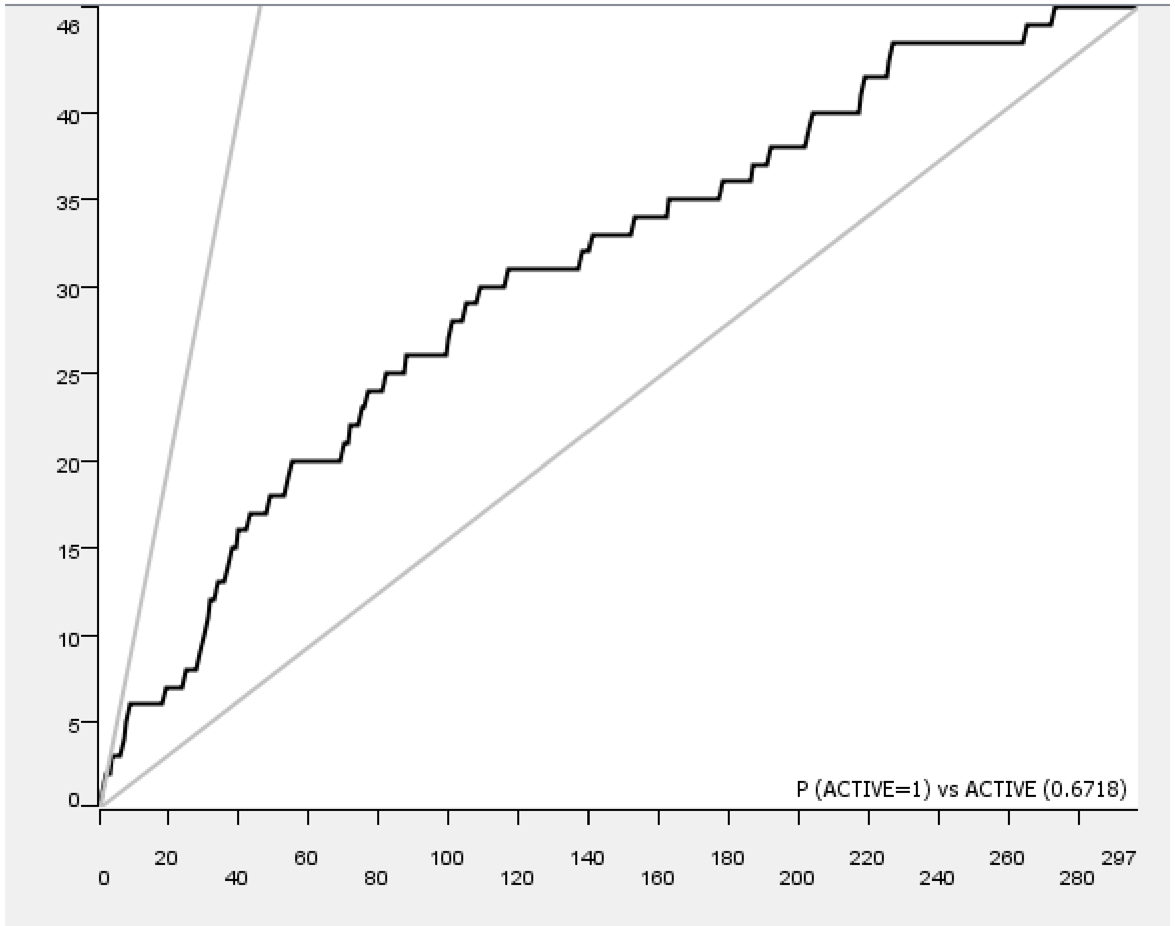

N=297

Top 10% Mean IC50	395 nM

	Predicted Active	Predicted Inactive
Active	15	31
Inactive	23	228

<10nM DefGood in aurora B, 25% error; Random seed = 121783

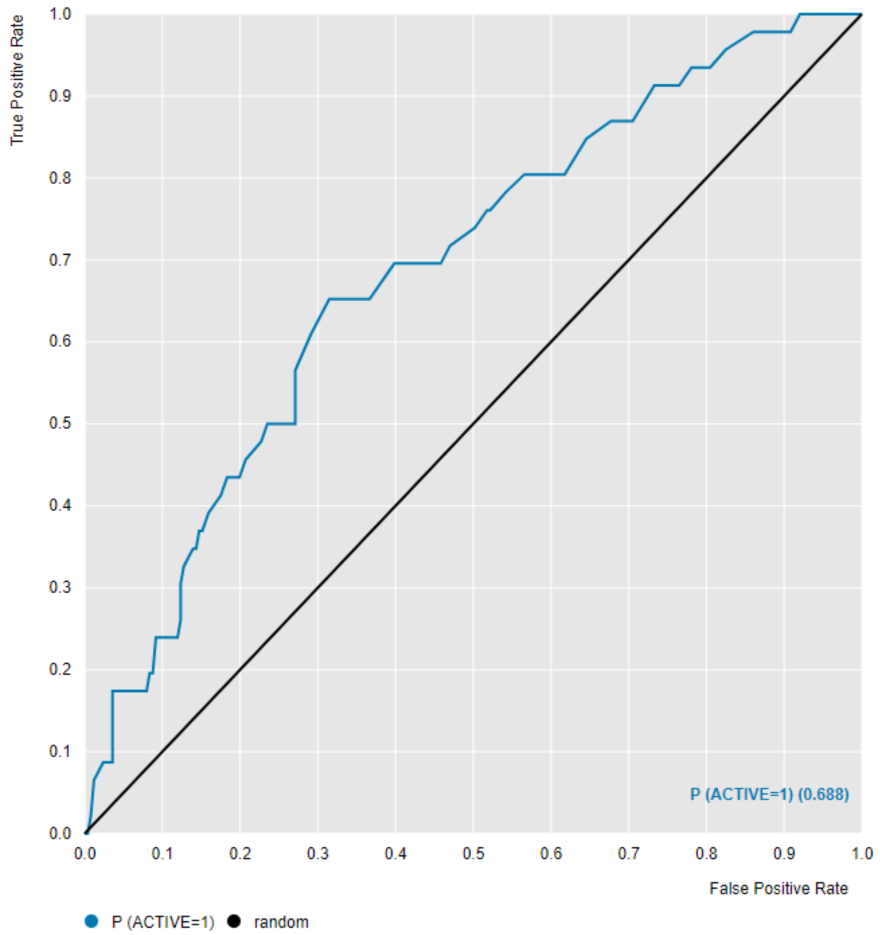

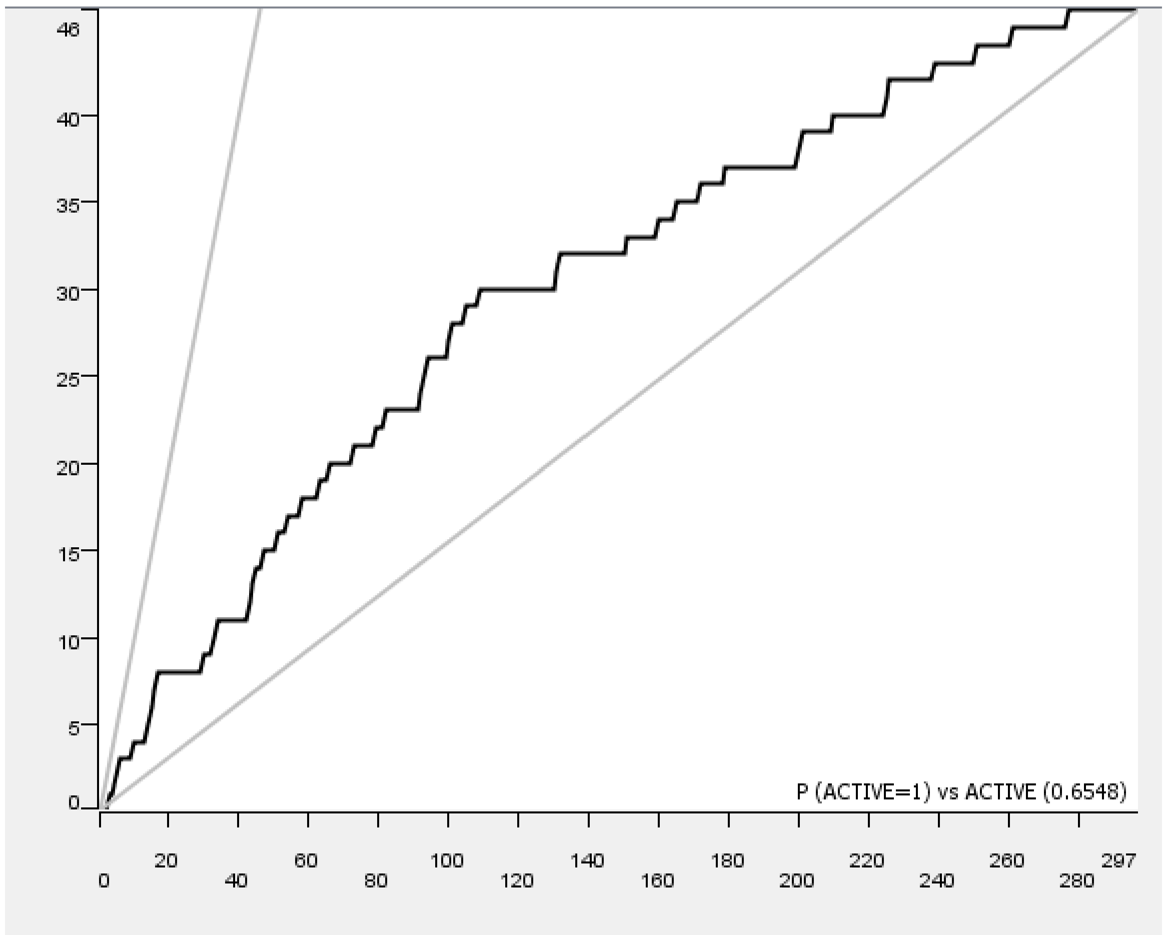

N=297

Top 10% Mean IC50	1,980 nM

	Predicted Active	Predicted Inactive
Active	14	32
Inactive	31	220

Conclusion - RF

- A Random Forrest could be generated for aurora B kinase with a decision value of <10 nM as defined as active. The predictivity of the algorithm could be preserved in three random train/test cycles as error was introduced in the training set. The point of failure was 20%, 20% and 25% error.

Conclusion - PNN

- Not done due to early failure on control set

β2 Adrenergic Receptor

β2 Adrenergic Receptor NBN Design

- Rules - $RELATION$ = "=" OR $IC50$ > 999 OR $IC50$ < 10 => TRUE
- Sort ascending and remove duplicates
- DefGood scanned between 5 and 25 nM
- 80-20 split for both actives and inactives
- NBN – ECFP4 used Classifier where Prediction was Active = True
- Resulted in 641 compounds after cleaning the data

<5 nM DefGood in beta-adren

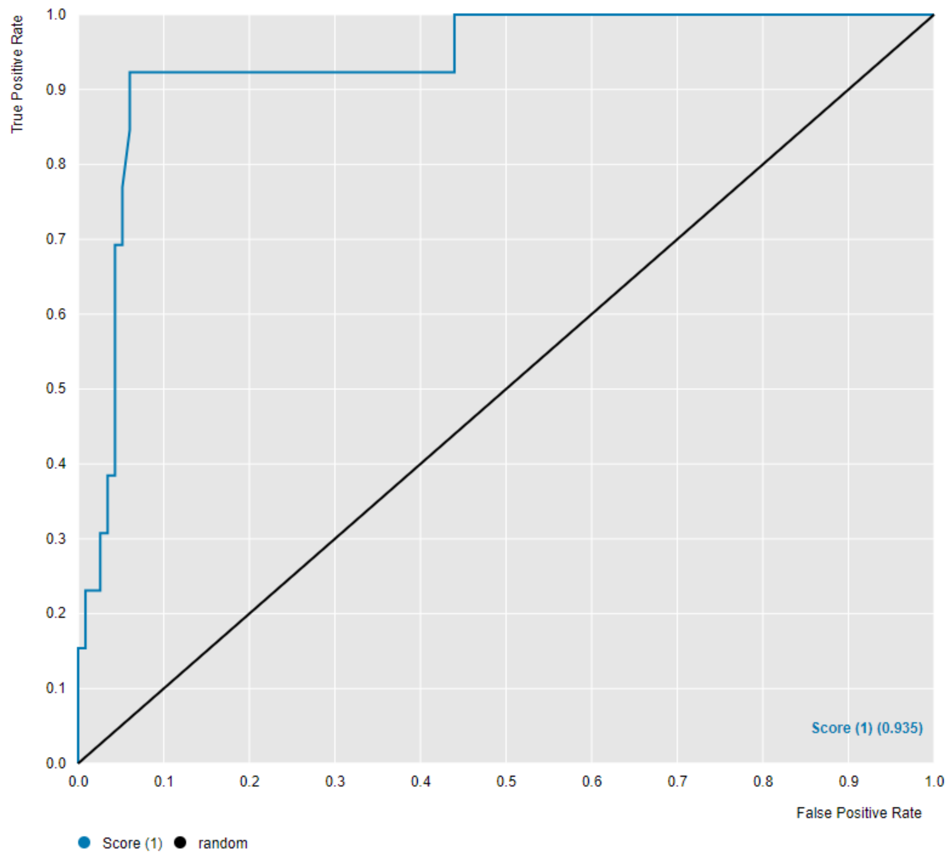

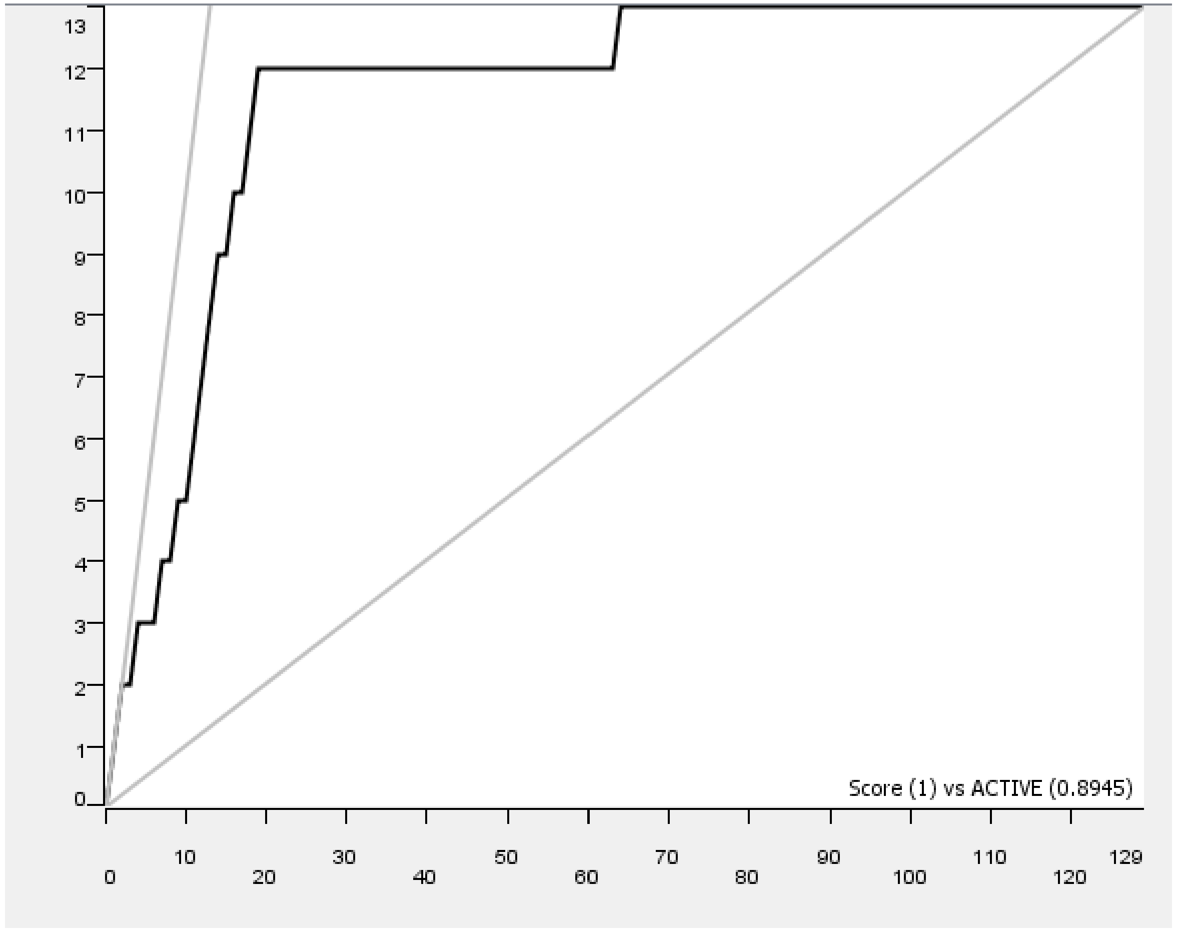

N=129

Top 10% Mean IC50	63.0 nM

	Predicted Active	Predicted Inactive
Active	12	1
Inactive	11	105

<10 nM DefGood in beta-adren

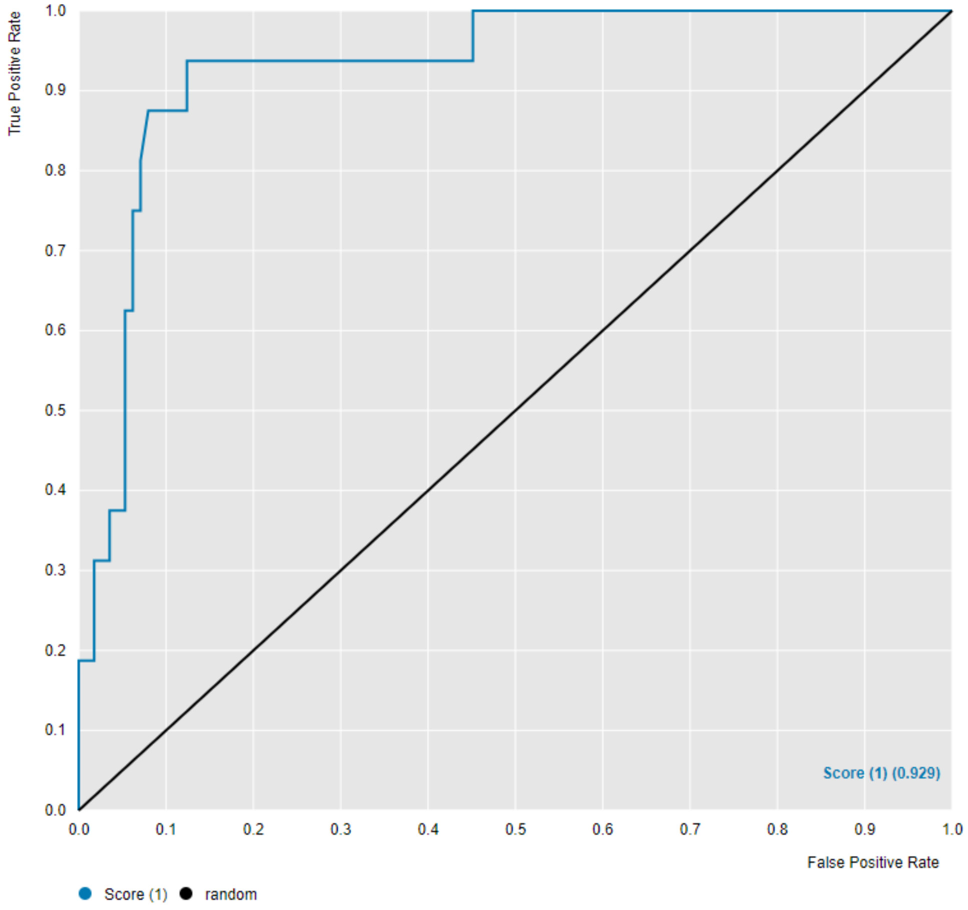

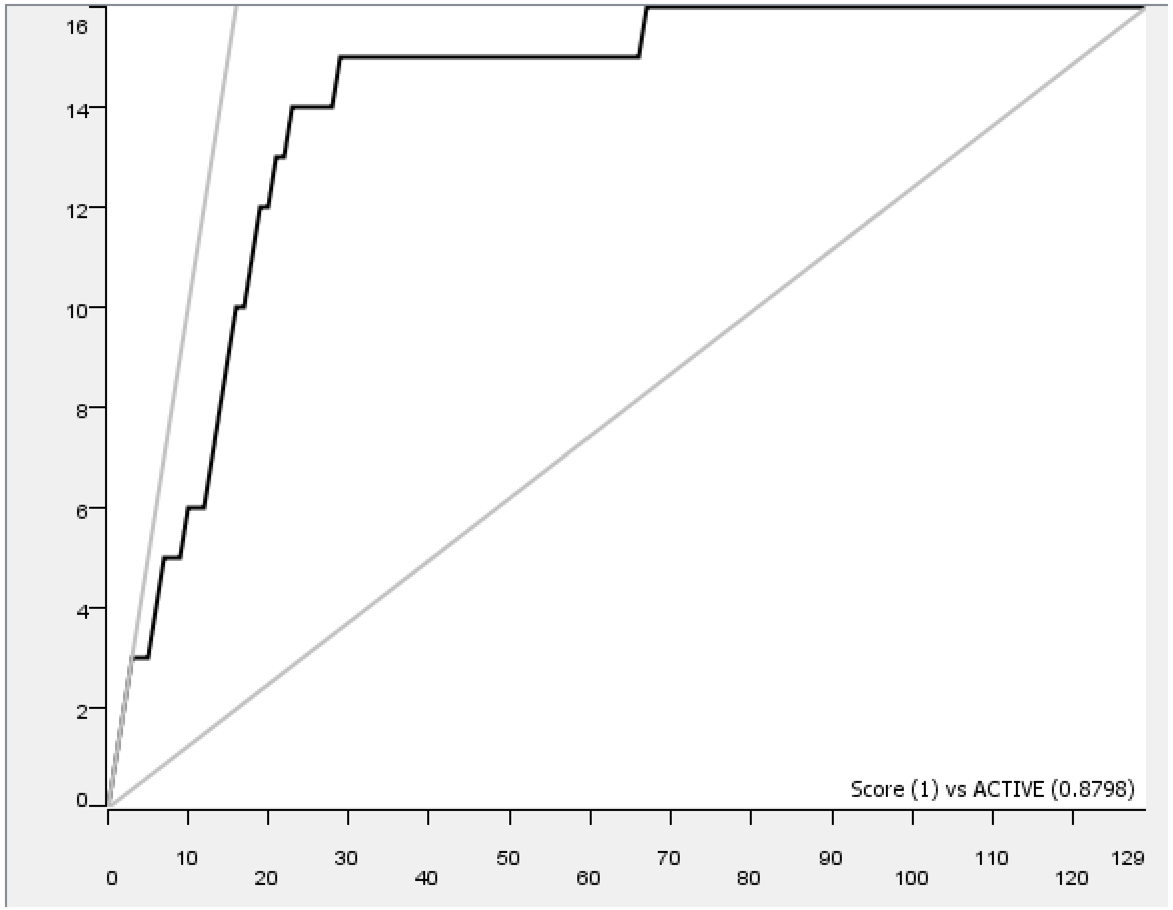

N=129

Top 10% Mean IC50	33.7 nM

	Predicted Active	Predicted Inactive
Active	15	1
Inactive	17	96

<15 nM DefGood in beta-adren

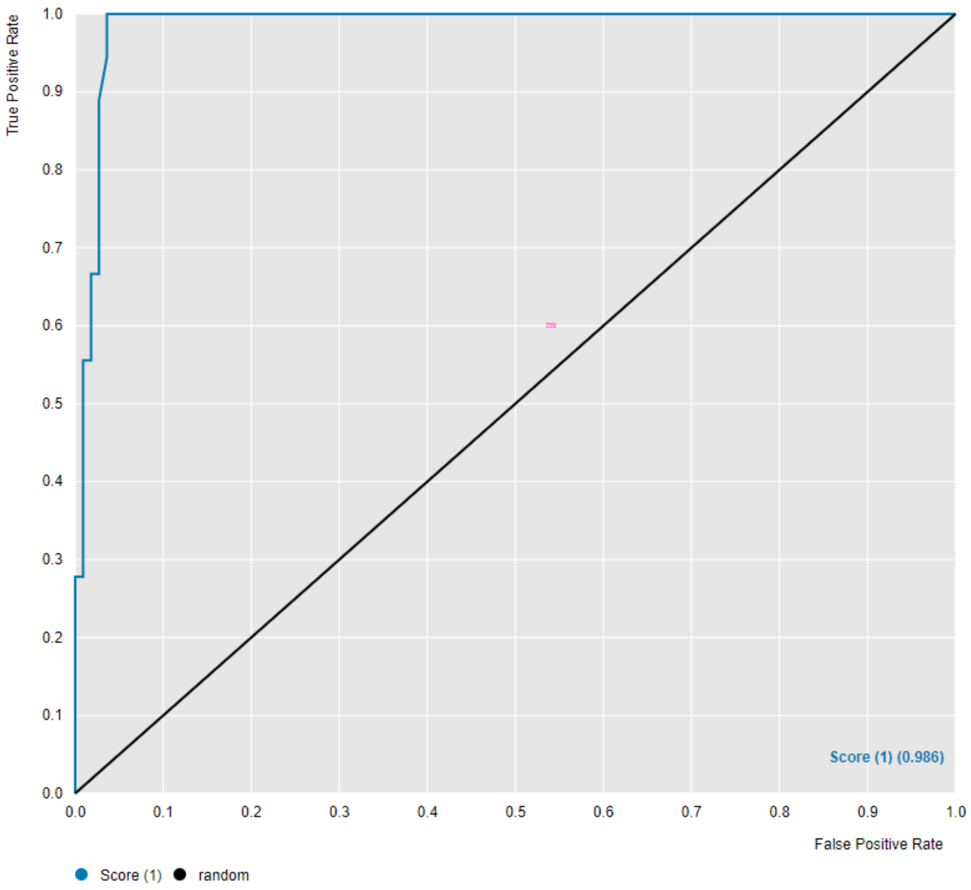

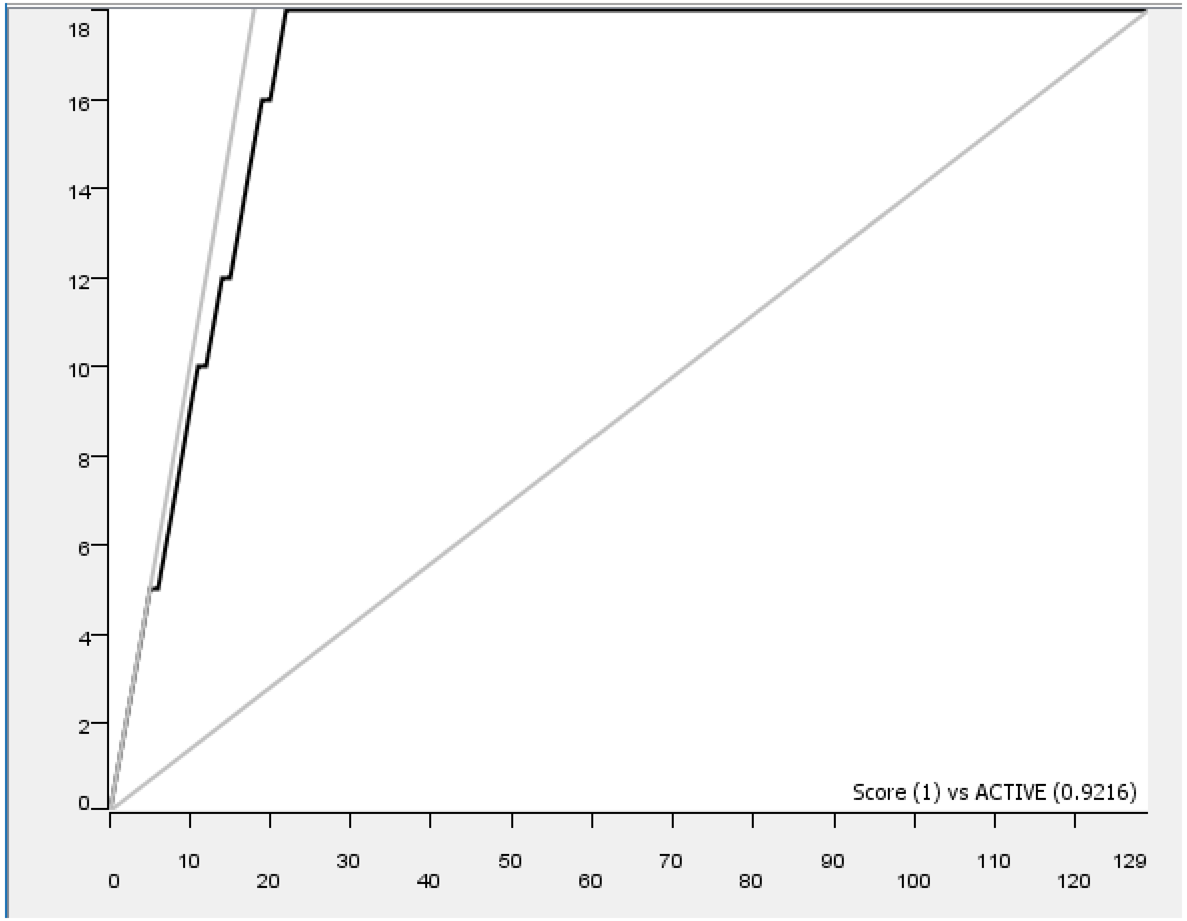

N=129

Top 10% Mean IC50	21.7 nM

	Predicted Active	Predicted Inactive
Active	18	0
Inactive	9	102

<20 nM DefGood in beta-adren

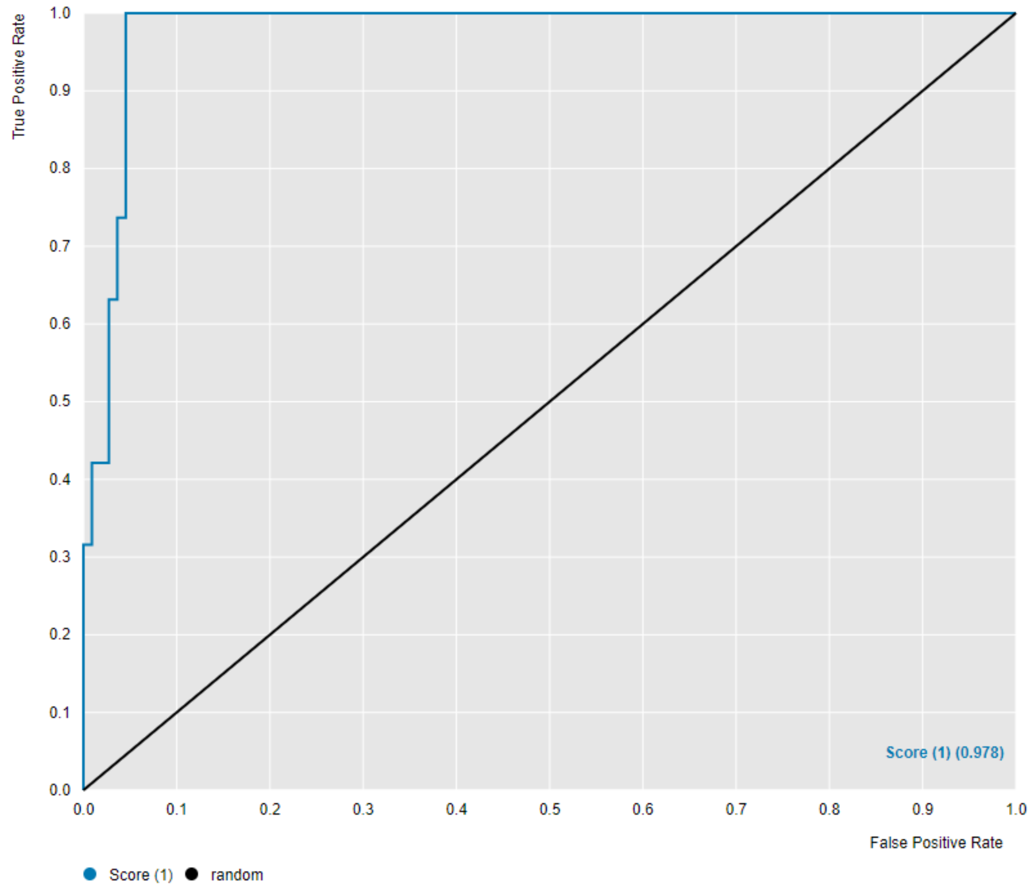

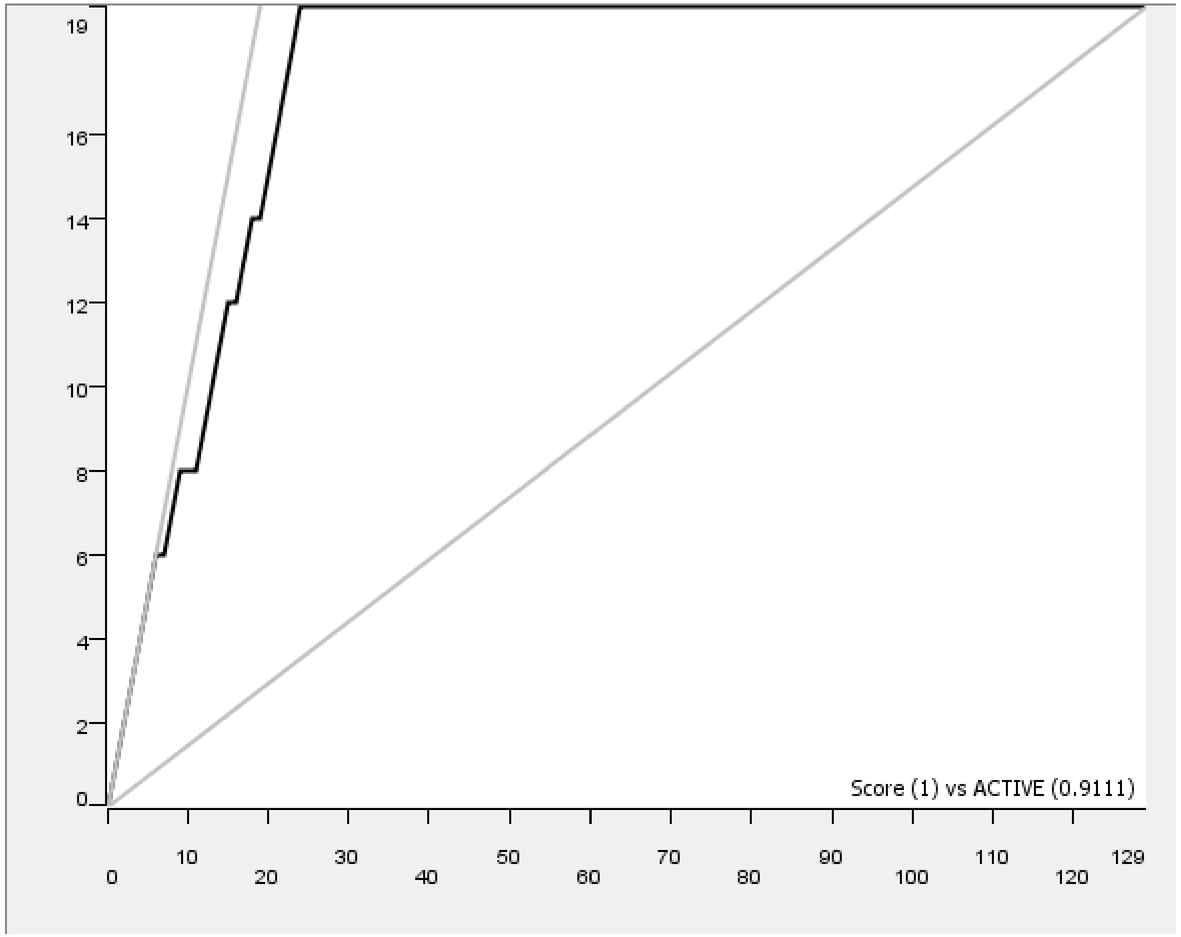

N=129

**Top 10% Mean IC50**	**32.2 nM**

	Predicted Active	Predicted Inactive
Active	19	0
Inactive	15	95

<25 nM DefGood in beta-adren

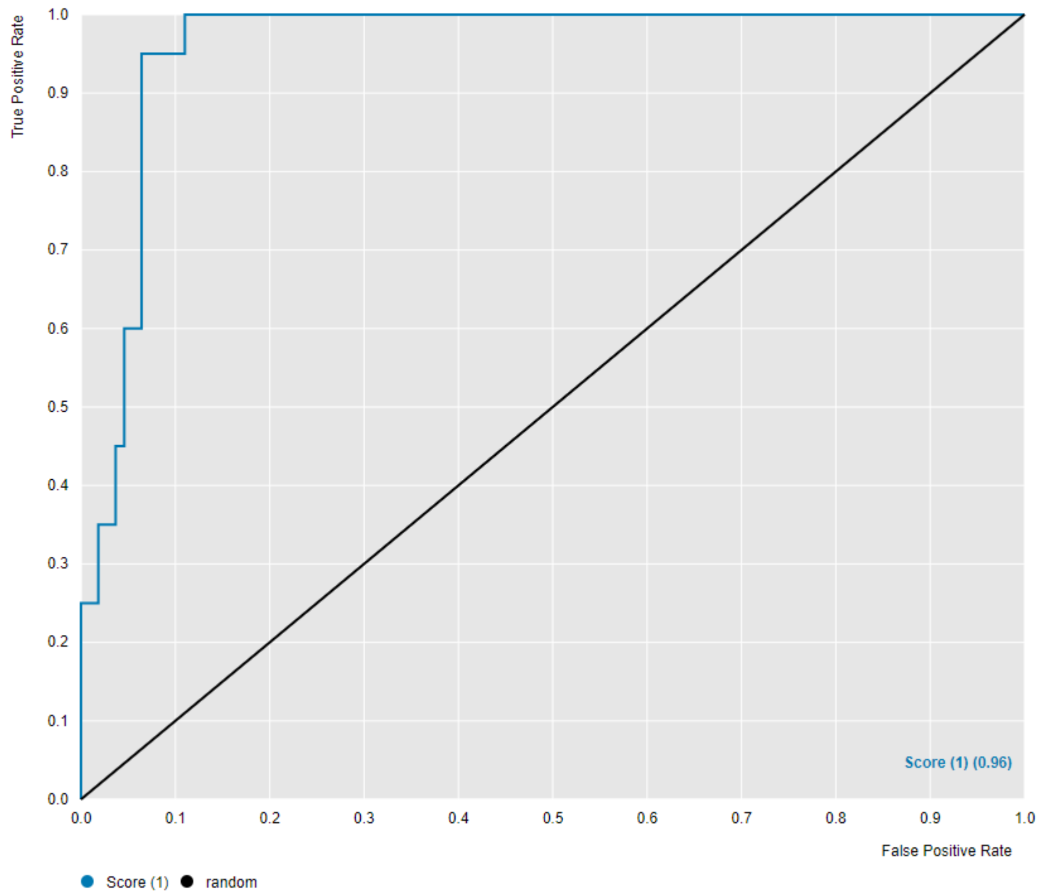

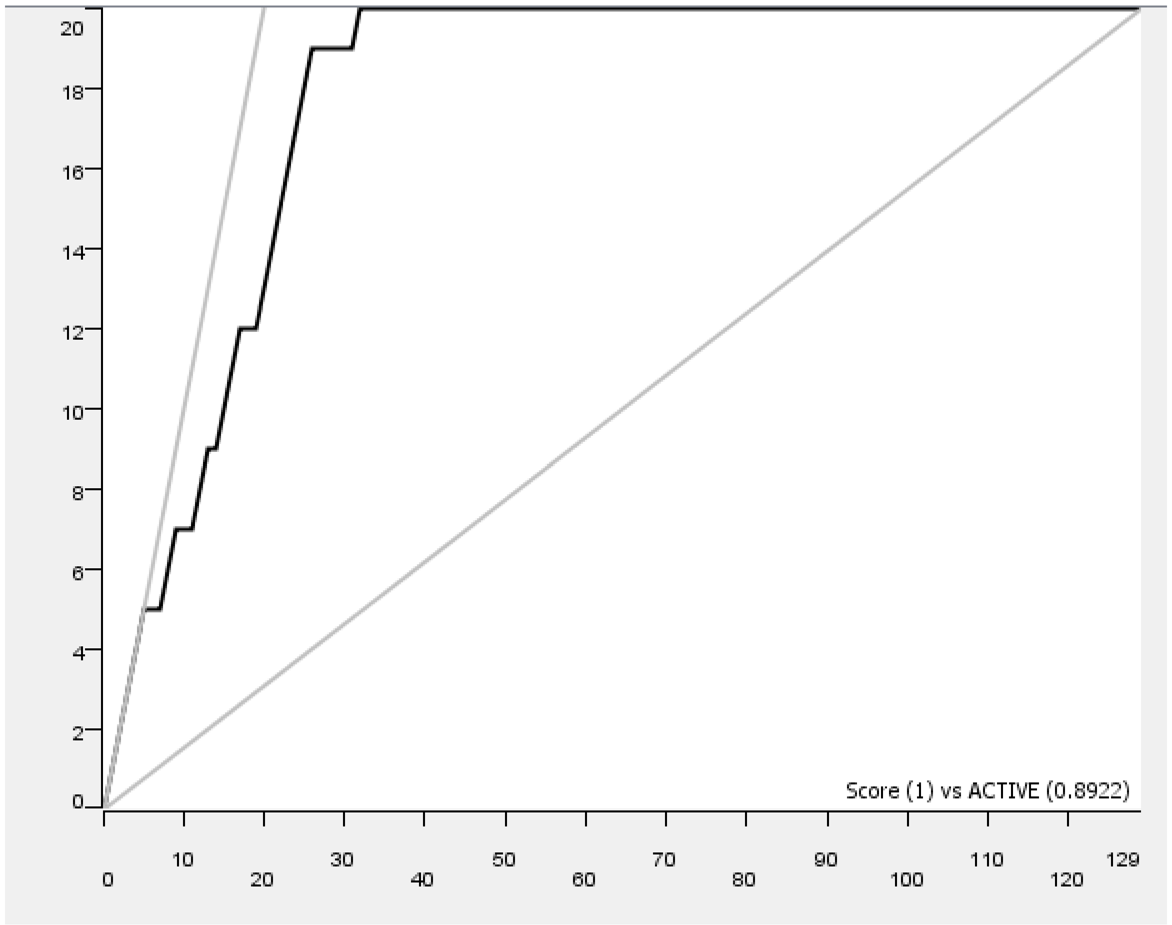

N=129

Top 10% Mean IC50	37.1 nM

	Predicted Active	Predicted Inactive
Active	19	1
Inactive	10	99

Decision on DefGood

- <15 nM has the best performance overall by far.

RF - <15 nM DefGood in beta-adren

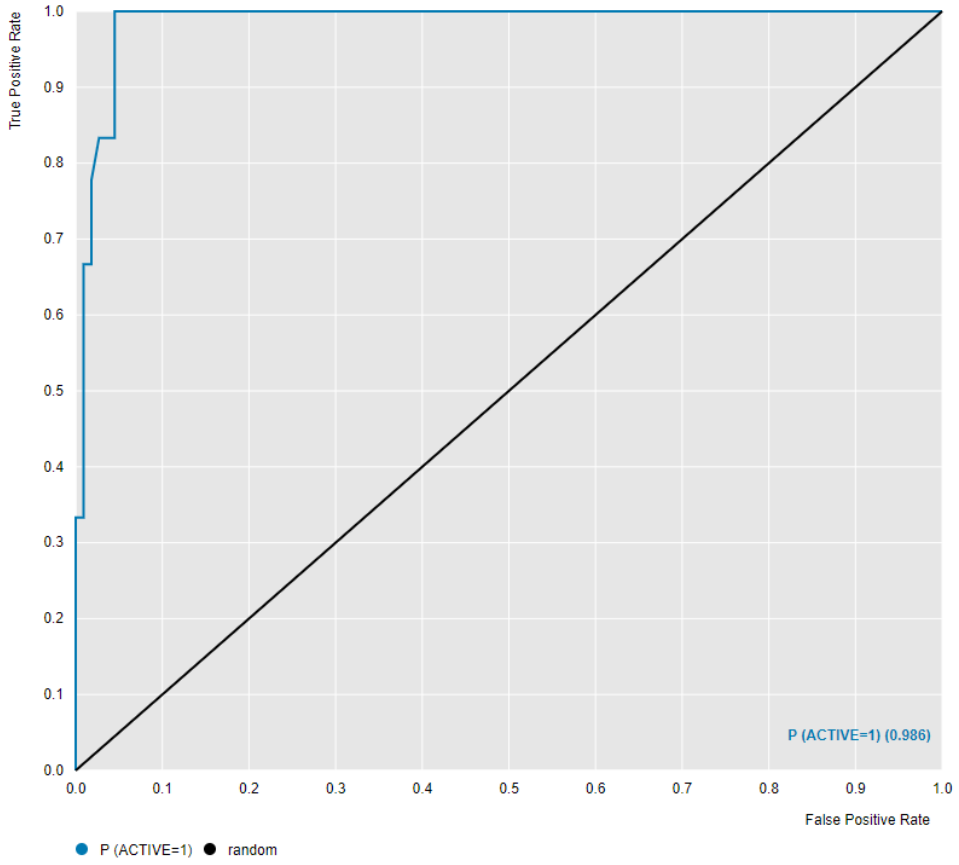

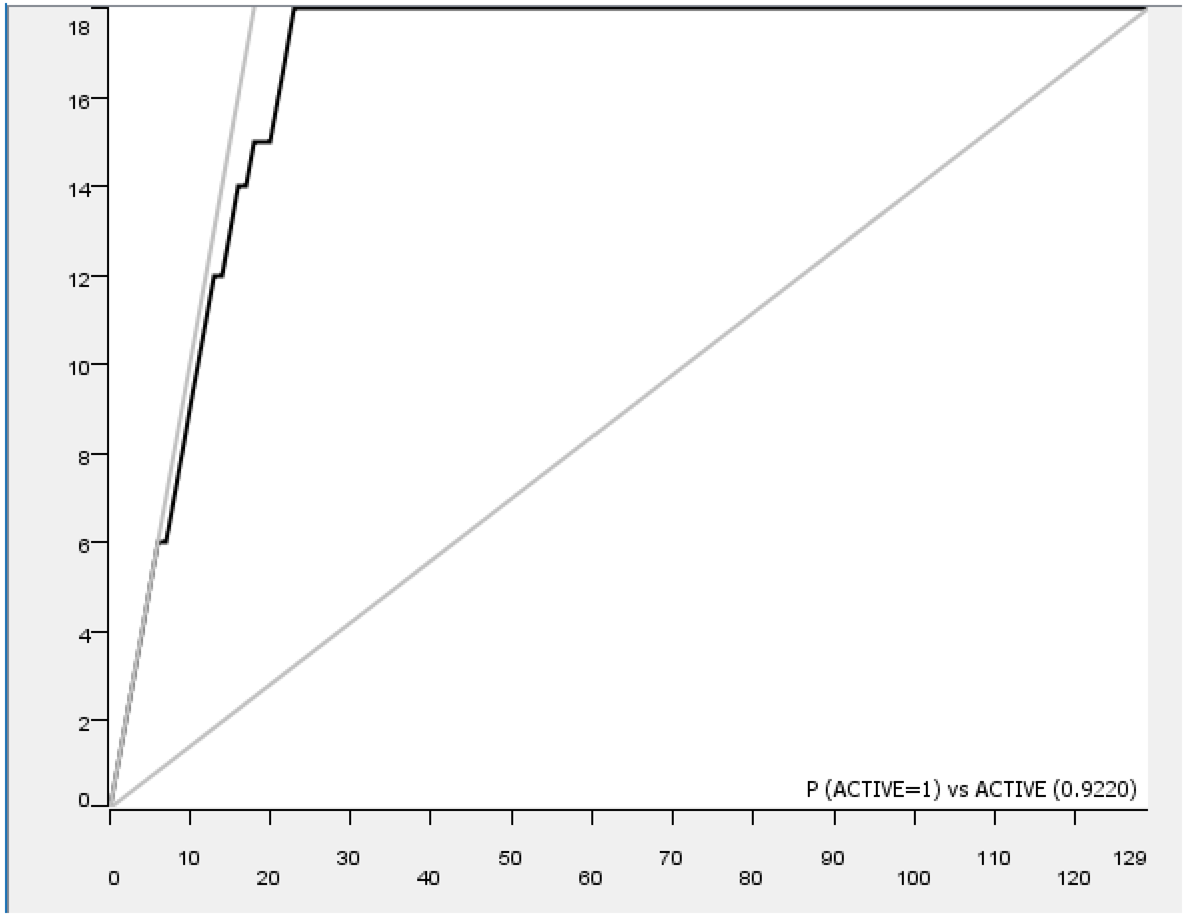

N=129

Top 10% Mean IC50	3.8 nM

	Predicted Active	Predicted Inactive
Active	15	3
Inactive	3	108

Decision on DefGood - RF

- Work in ALK showed DefGood in the NBN was similar to DefGood in an RF. Therefore, this value for DefGood from the NBN design was used as DefGood.

PNN - <15 nM DefGood in beta-adren

- Properties List (46 calculatable properties were used)
- Parameterization (Global Accuracy for Activity at a 15 nM decision value was used, and a 5-fold stratified sampling cross validation was used to parameterize theta minus and theta plus) Theta Minus = 0.05 and Theta Plus = 0.25

PNN - <15 nM DefGood in beta-adren

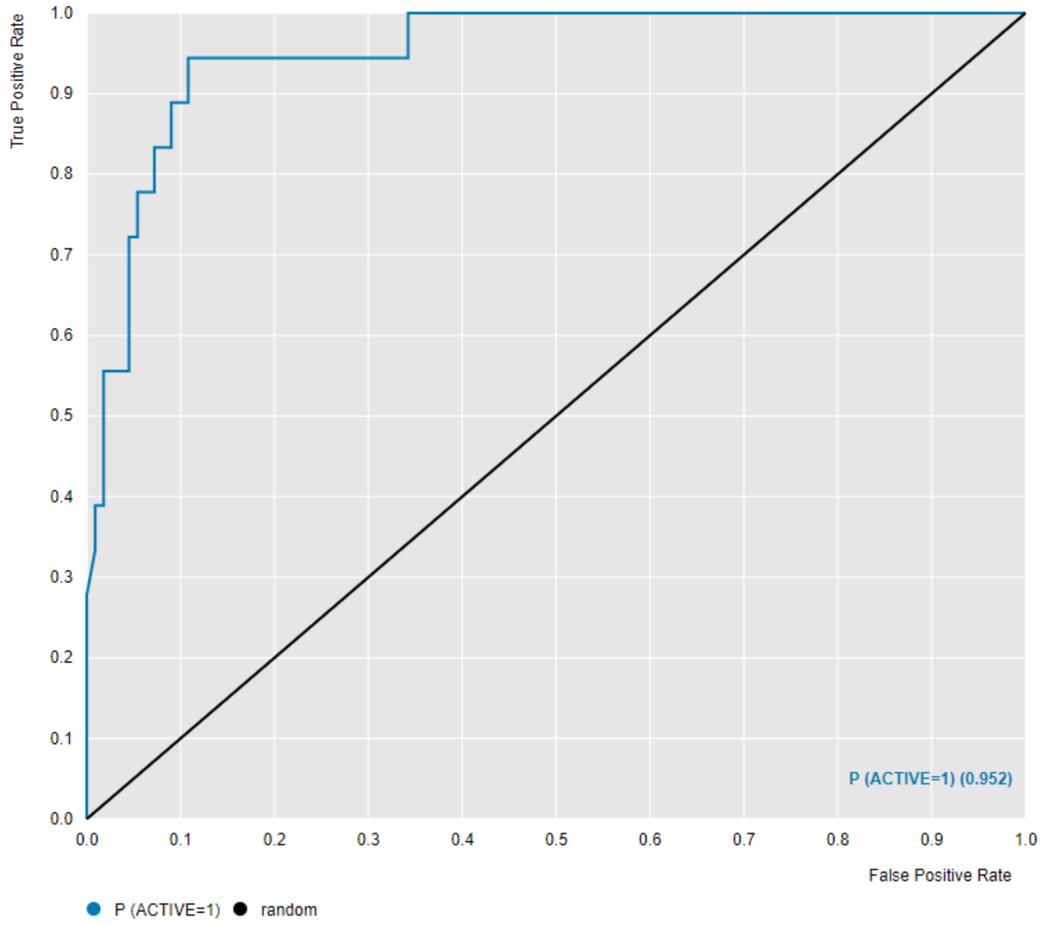

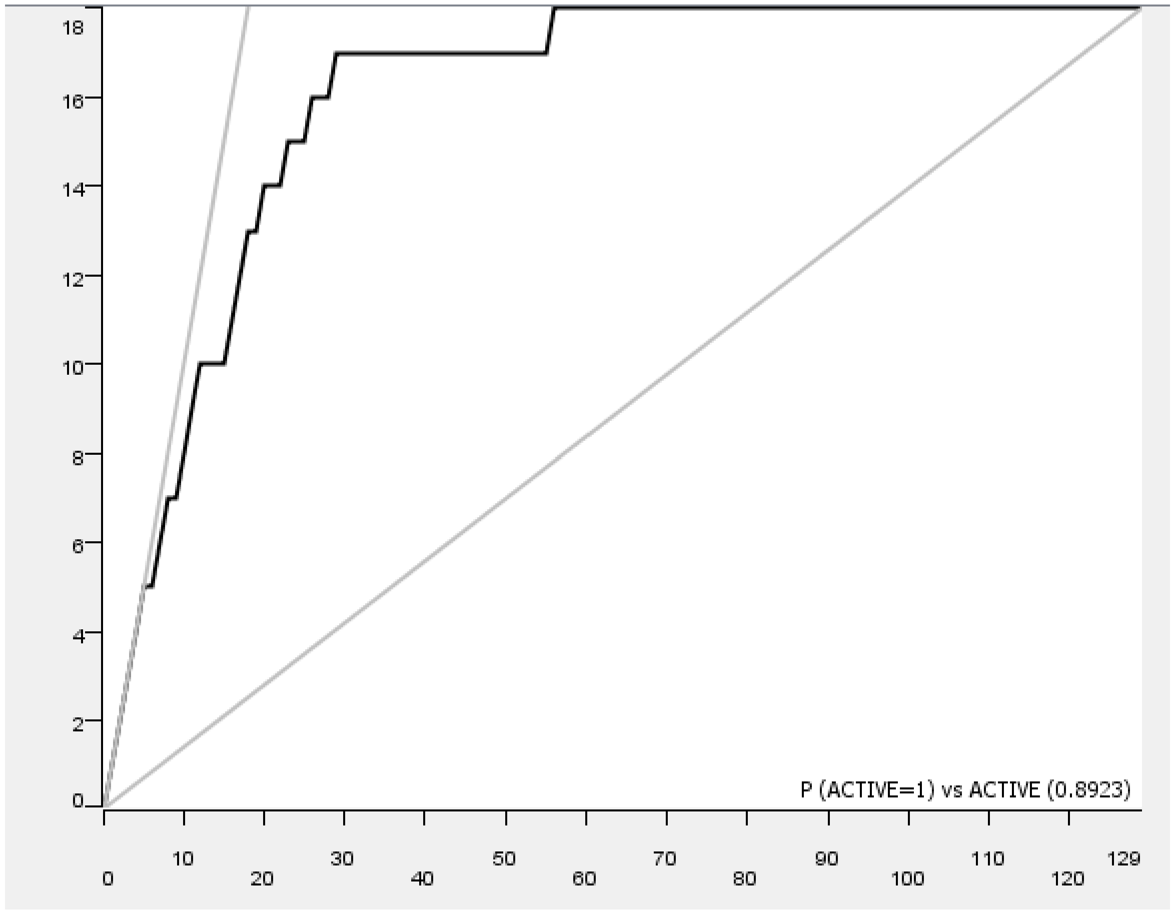

N=129

Top 10% Mean IC50	13.1 nM

	Predicted Active	Predicted Inactive
Active	9	9
Inactive	2	109

Decision on DefGood - PNN

- In ALK, the DefGood for a PNN mirrored those of the RF and the NBN. Therefore, DefGood from the NBN was used as DefGood for the PNN. Parameterization for each model adds significant time to the process. A rough benchmark can be applied where point of failure is discovered and then the model is parameterized to ensure that the situation is optimal for the PNN. This will be applied to the discovery of the error tolerance threshold for the PNN in the beta 2 adrenergic receptor space.

NBN Error Tolerance- <15 nM DefGood in beta-adren

- 0-50% absolute error

<15 nM DefGood in beta-adren, 5% error; Random seed = 1515533876005

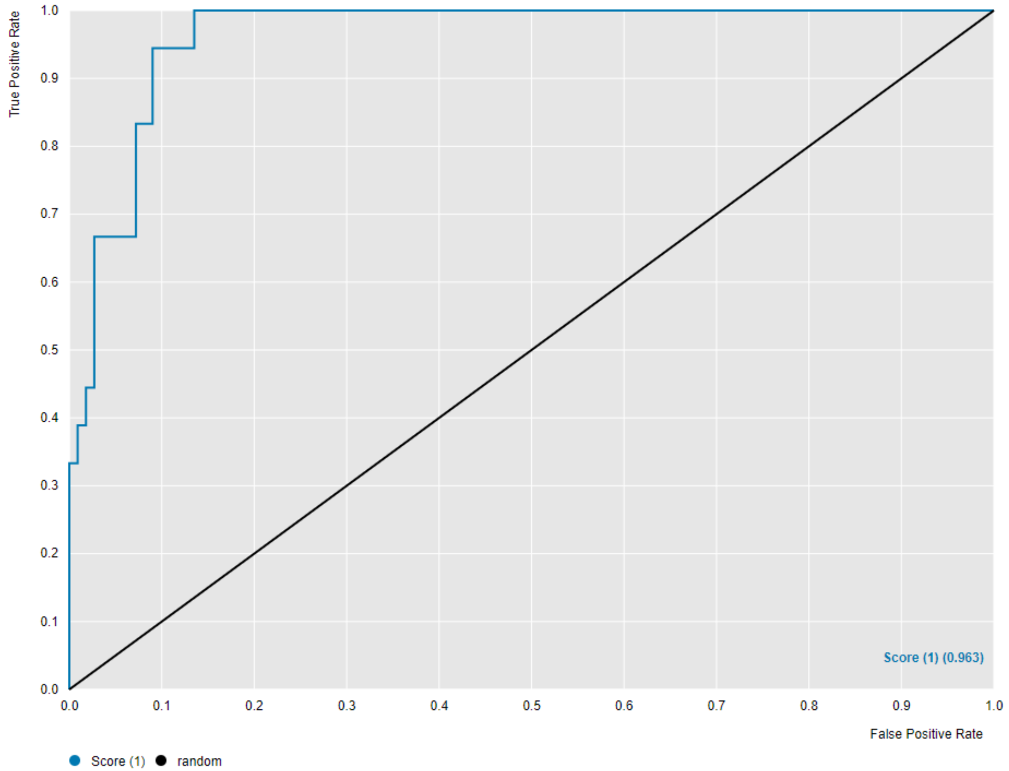

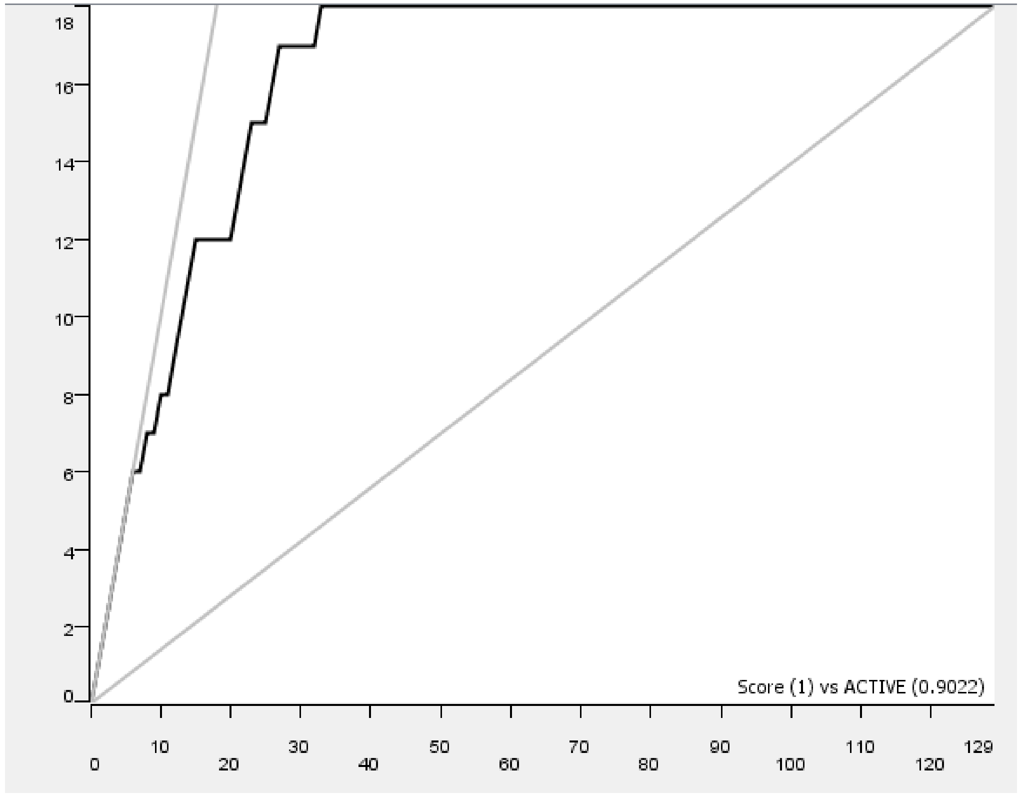

N=129

Top 10% Mean IC50	24.5 nM

	Predicted Active	Predicted Inactive
Active	12	6
Inactive	8	103

<15nM DefGood in beta-adren, 10% error

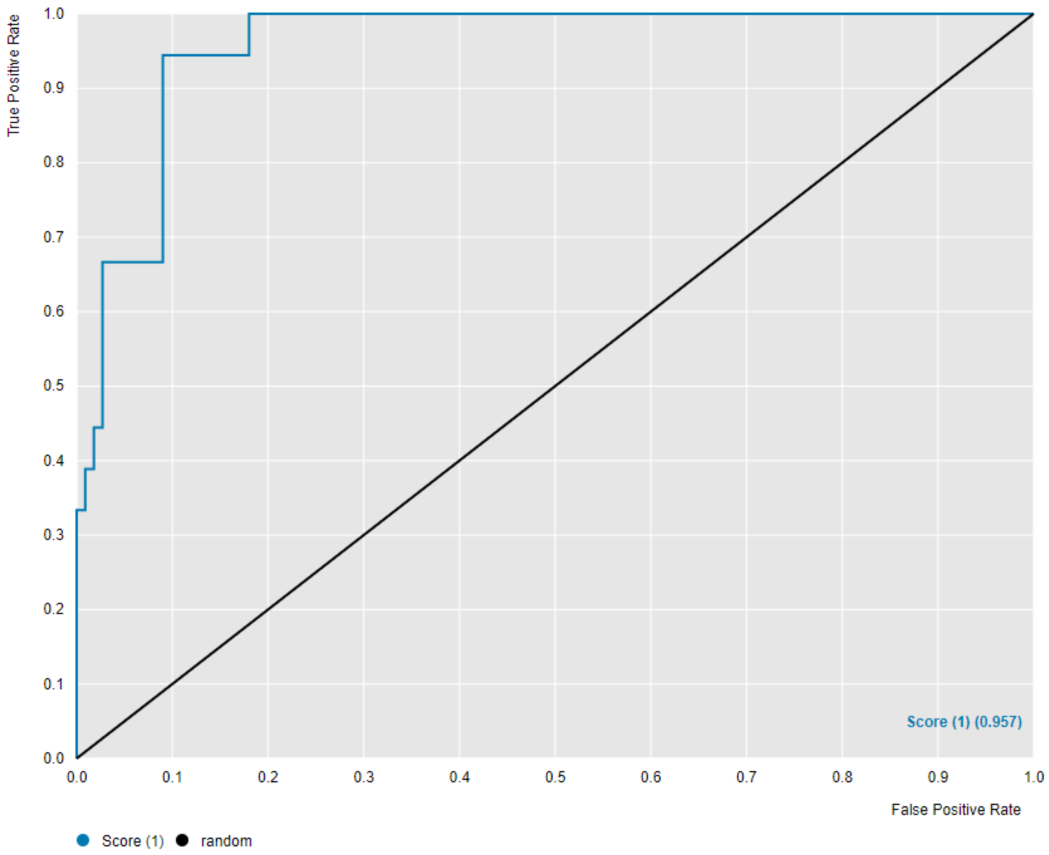

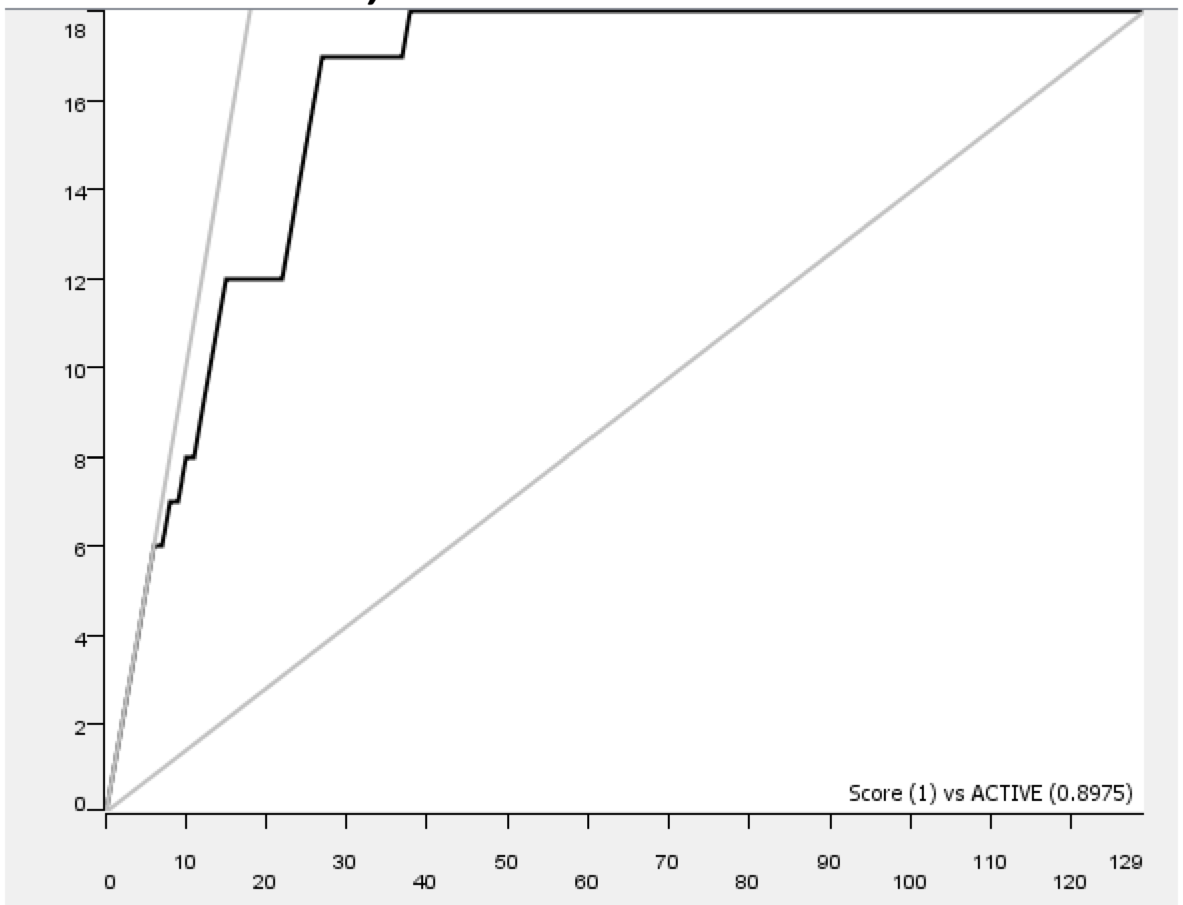

N=129

**Top 10% Mean IC50**	**24.5 nM**

	**Predicted Active**	**Predicted Inactive**
Active	12	6
Inactive	8	103

<15nM DefGood in beta-adren, 15% error

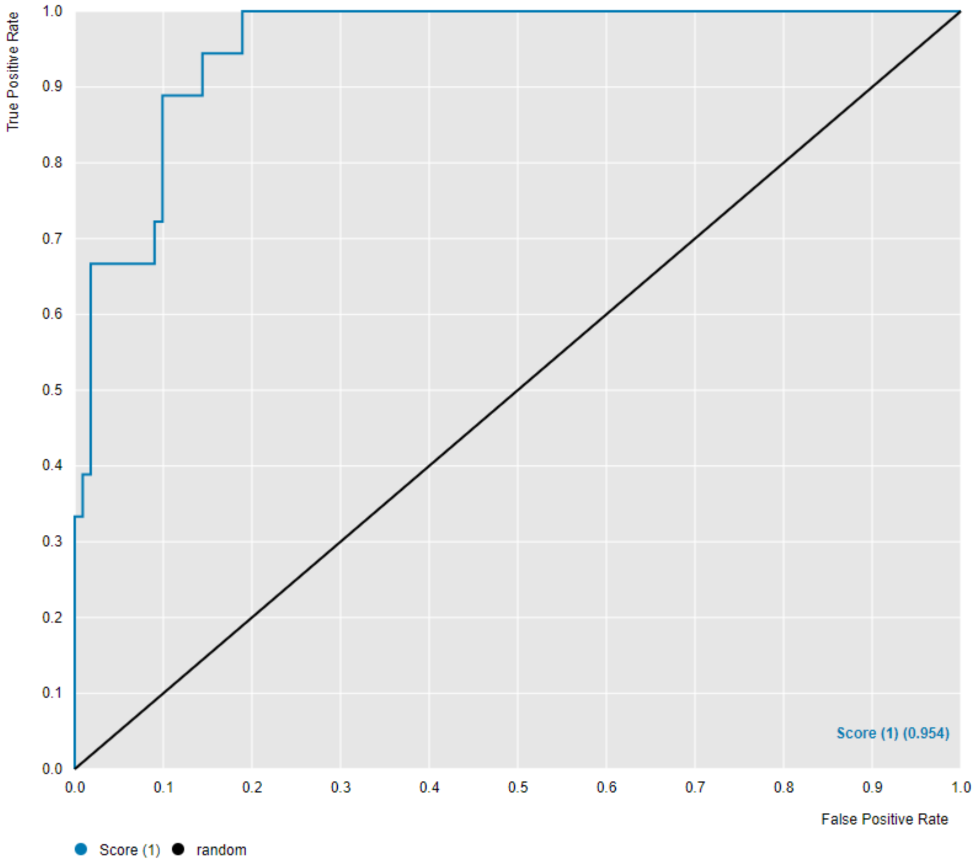

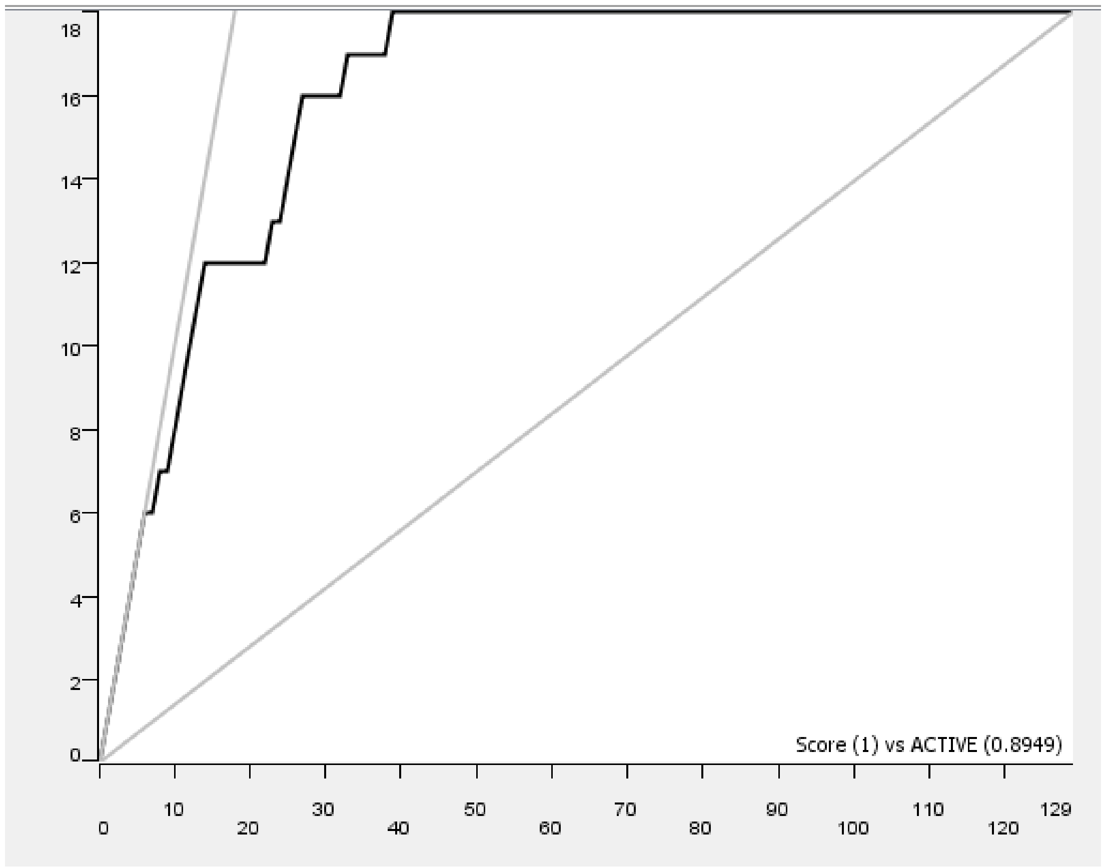

N=129

**Top 10% Mean IC50**	**21.9 nM**

	**Predicted Active**	**Predicted Inactive**
Active	16	2
Inactive	12	99

<15nM DefGood in beta-adren, 20% error

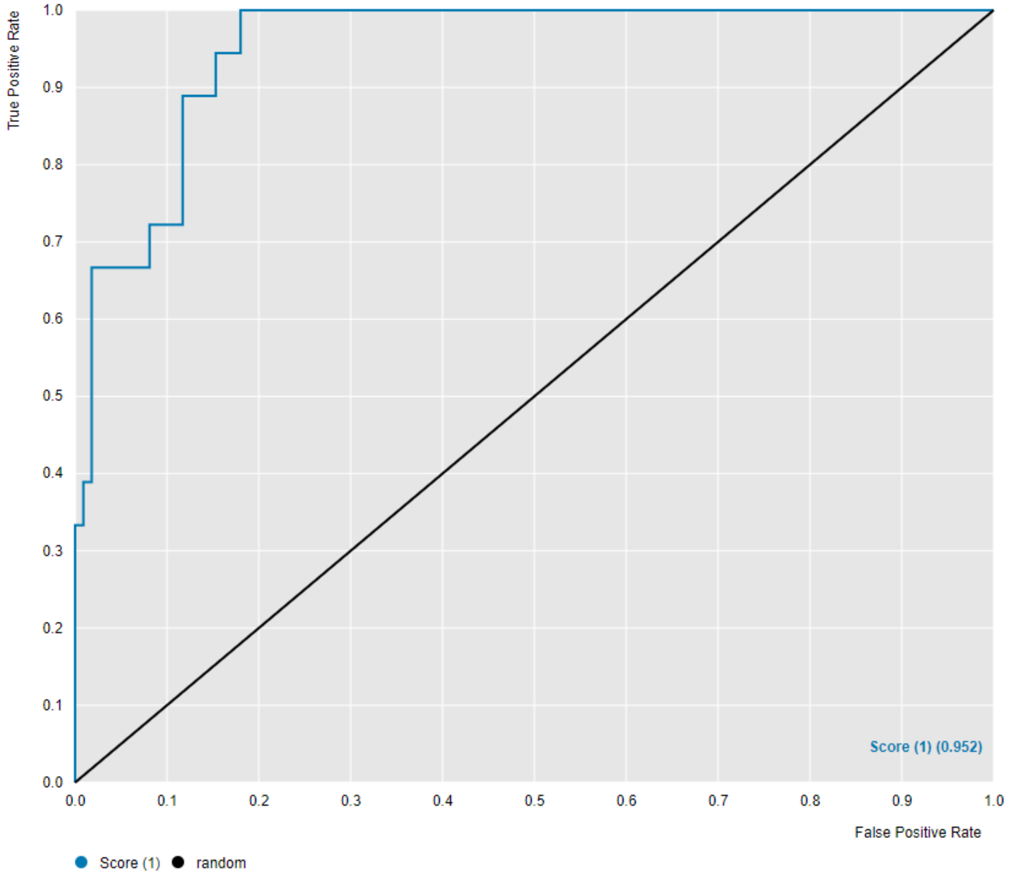

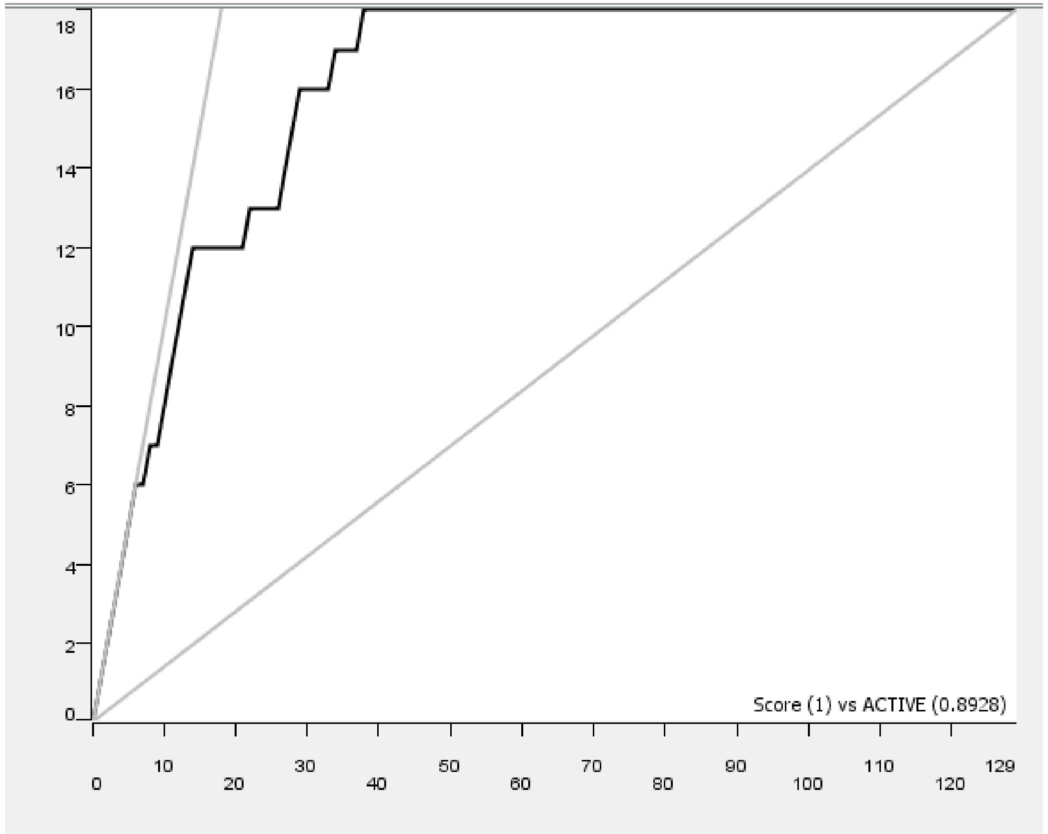

N=129

**Top 10% Mean IC50**	**21.1 nM**

	**Predicted Active**	**Predicted Inactive**
Active	13	5
Inactive	11	100

<15nM DefGood in beta-adren, 30% error

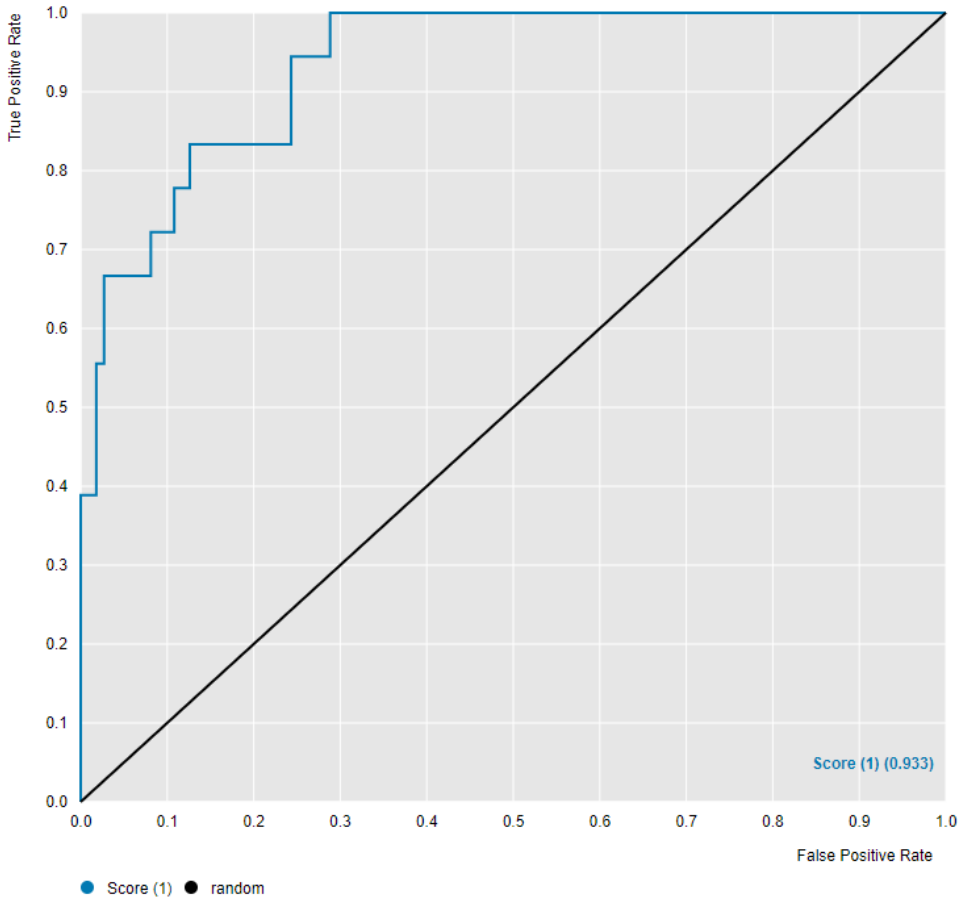

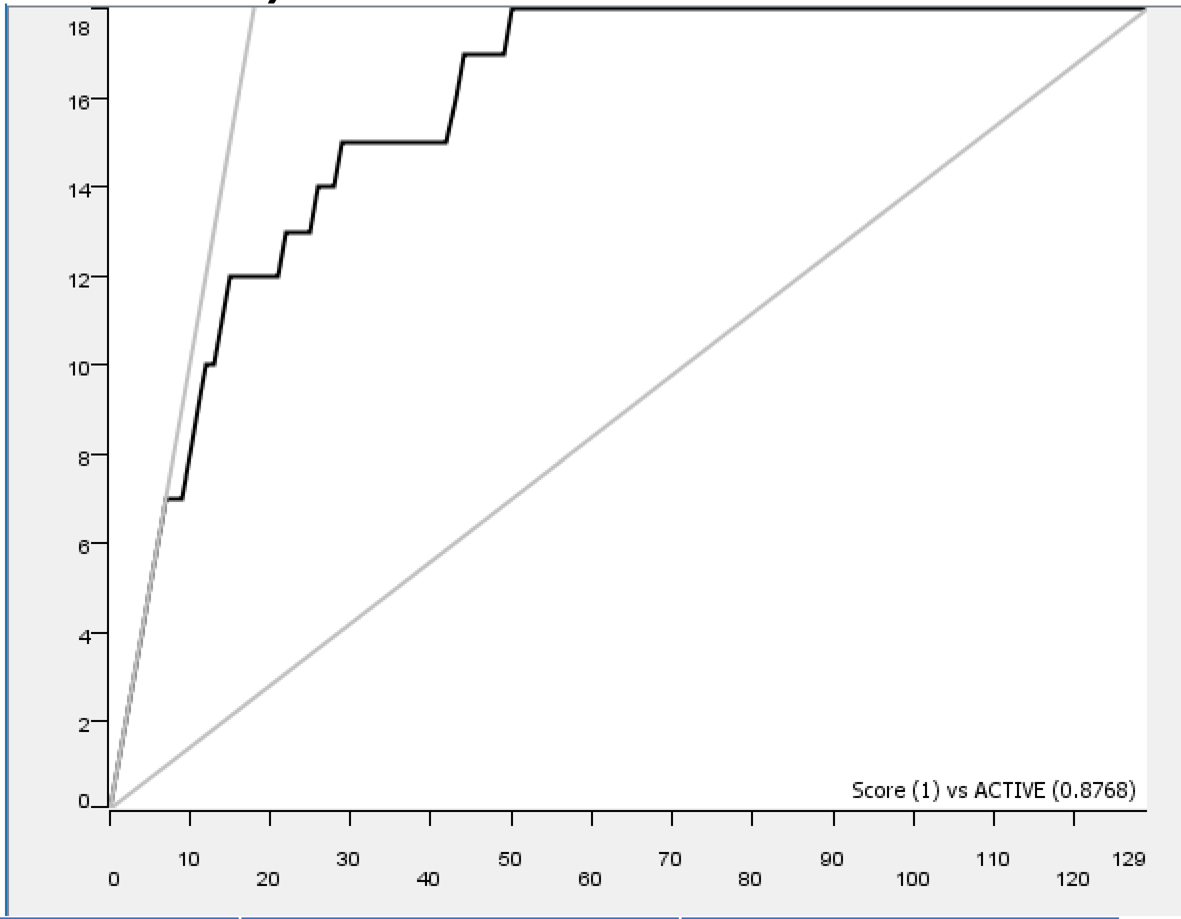

N=129

**Top 10% Mean IC50**	**21.9 nM**

	**Predicted Active**	**Predicted Inactive**
Active	13	5
Inactive	11	100

<15nM DefGood in beta-adren, 40% error

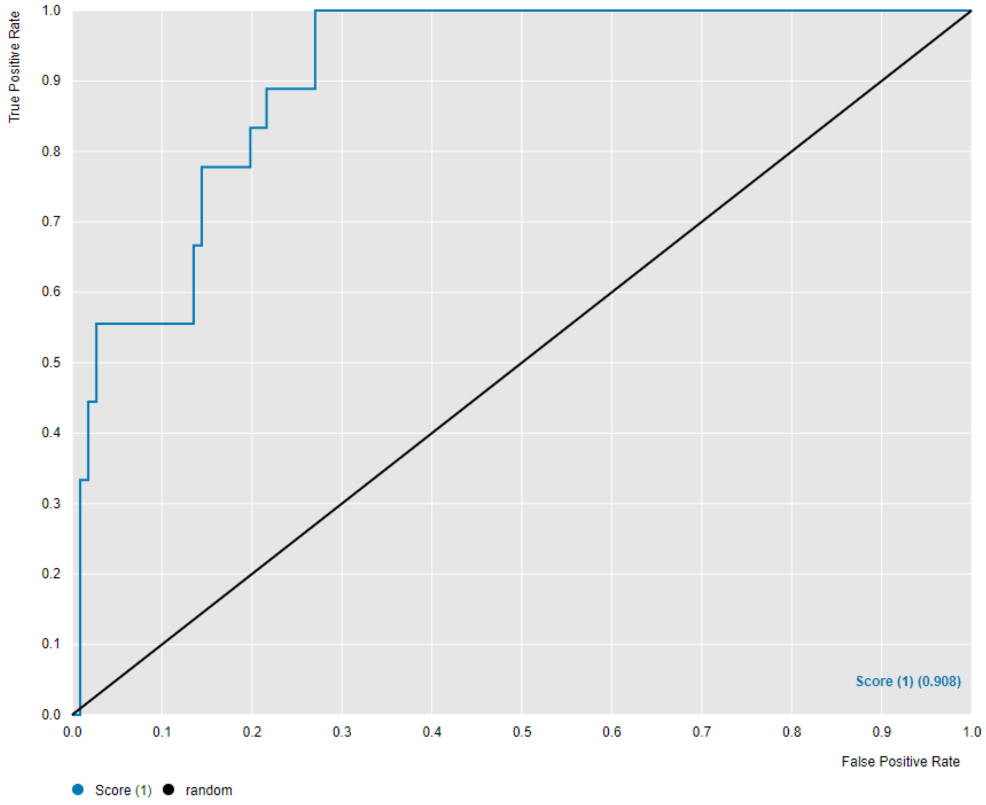

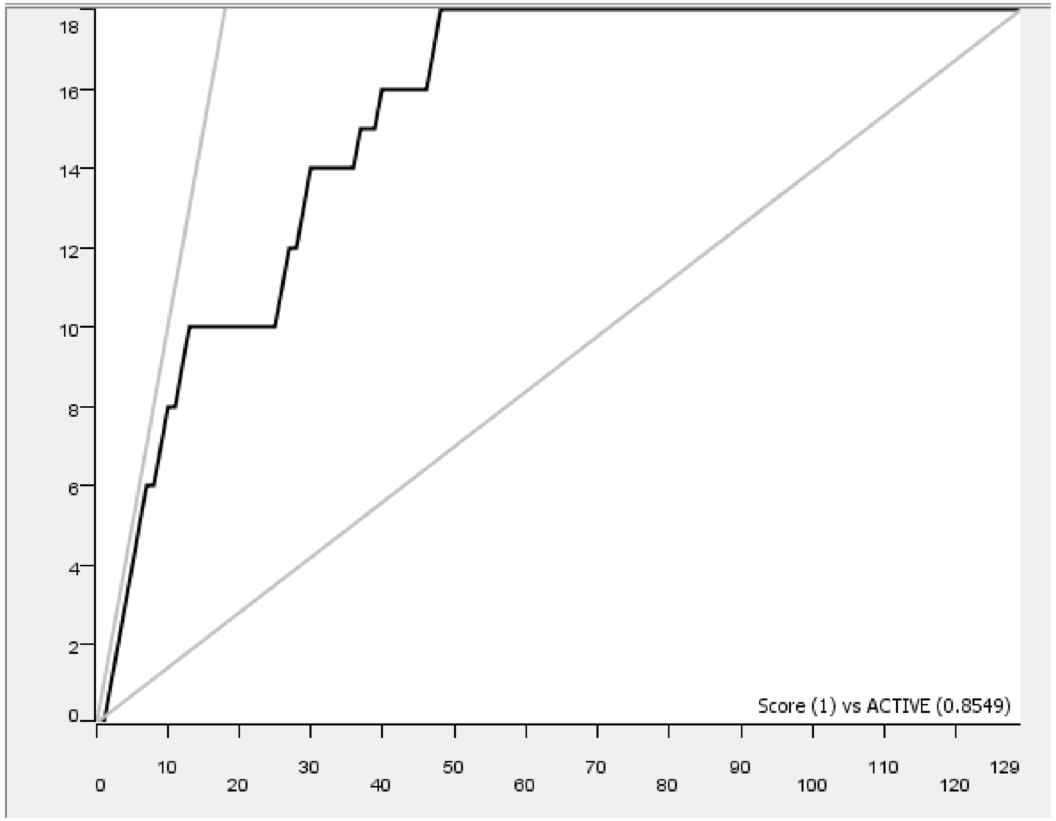

N=129

**Top 10% Mean IC50**	**24.5 Nm**

	**Predicted Active**	**Predicted Inactive**
Active	10	8
Inactive	6	105

<15nM DefGood in beta-adren, 45% error

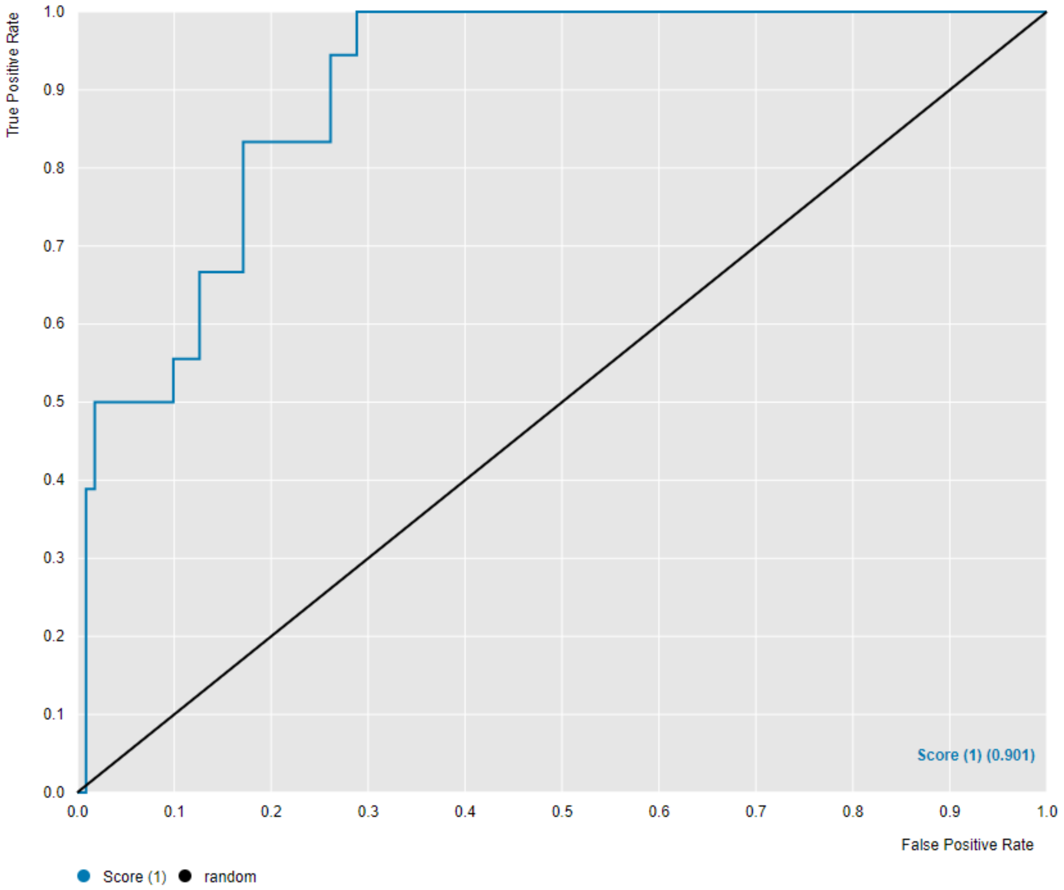

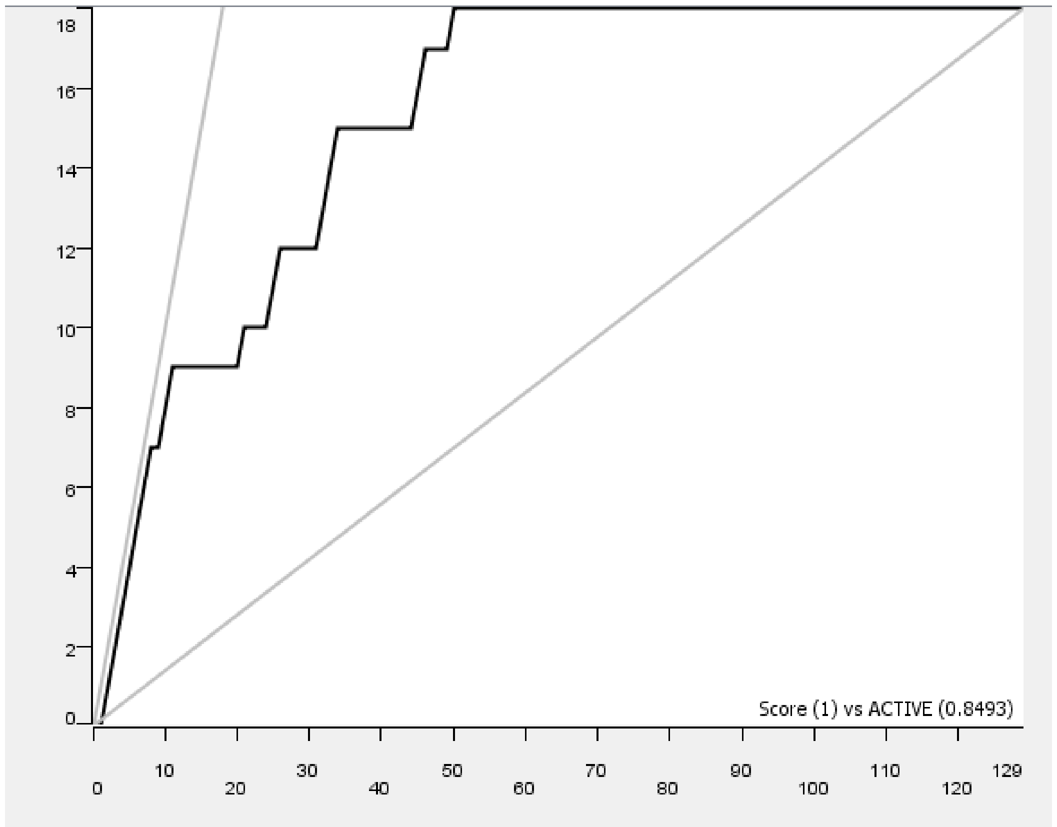

N=129

Top 10% Mean IC50	24.5 Nm

	Predicted Active	Predicted Inactive
Active	18	0
Inactive	77	34

<15nM DefGood in beta-adren, 50% error

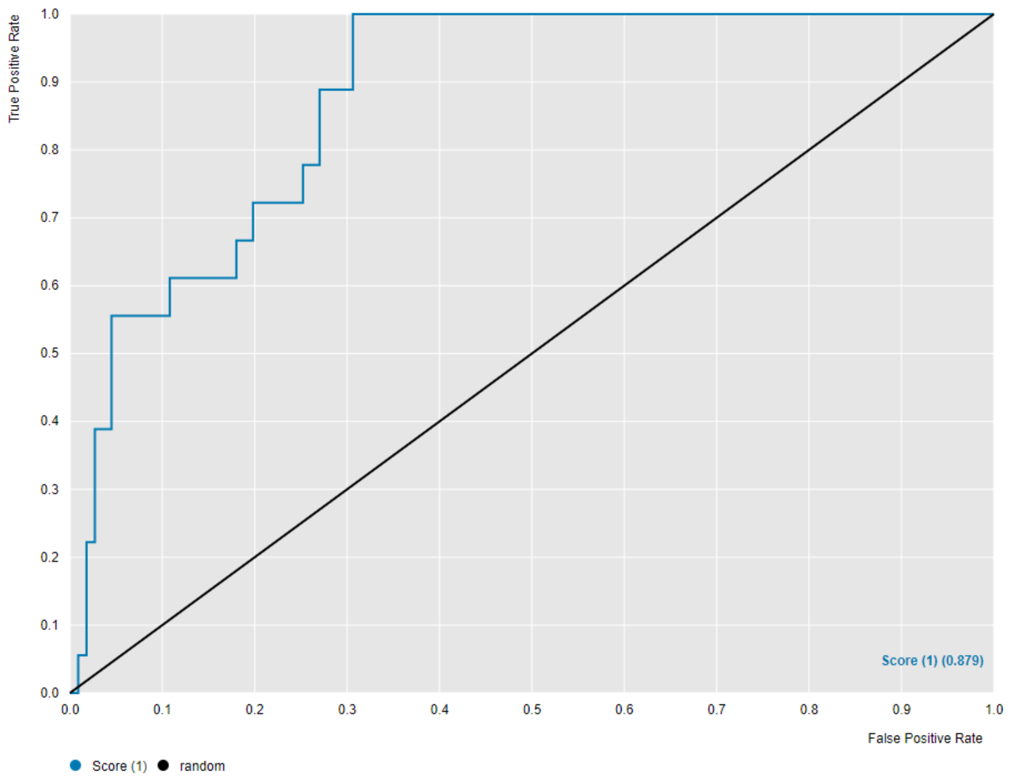

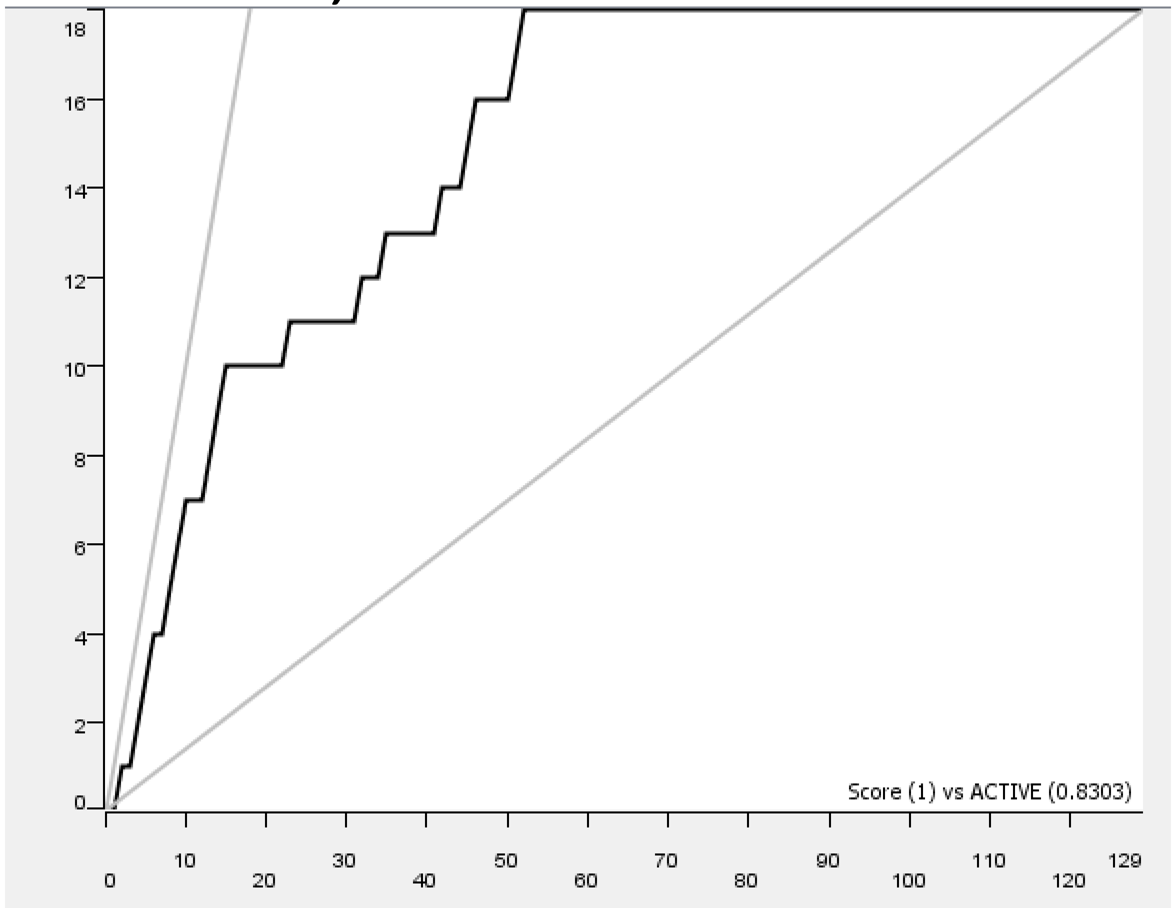

N=129

Top 10% Mean IC50	2,500 Nm

	Predicted Active	Predicted Inactive
Active	18	0
Inactive	77	34

<15nM DefGood in beta-adren, 10% error; Random seed = 429

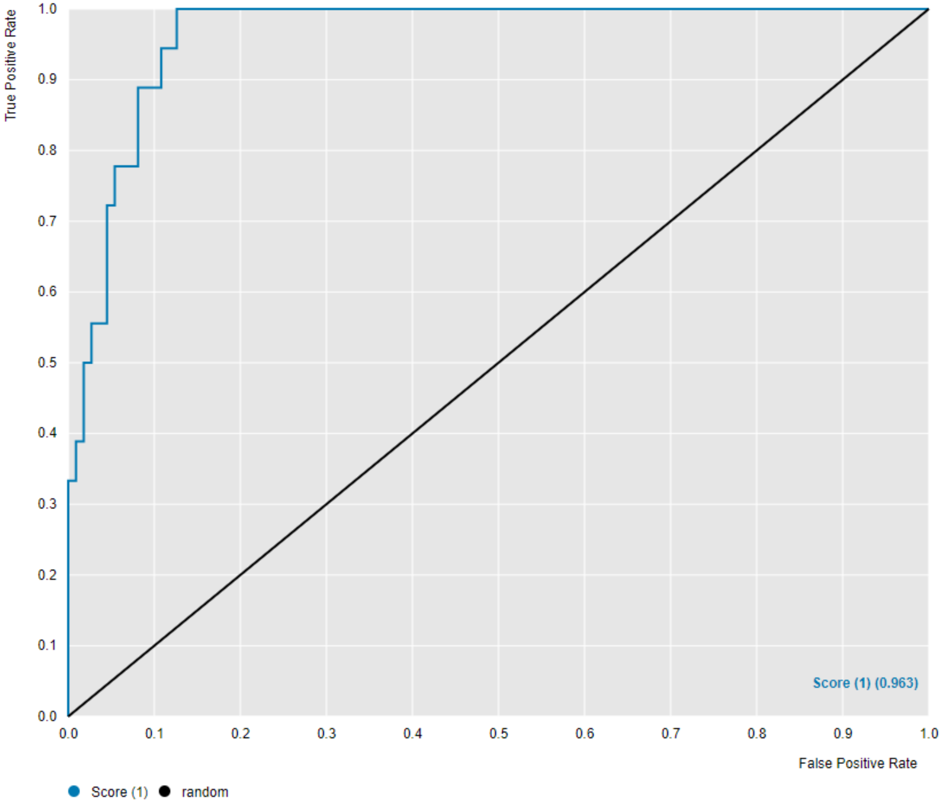

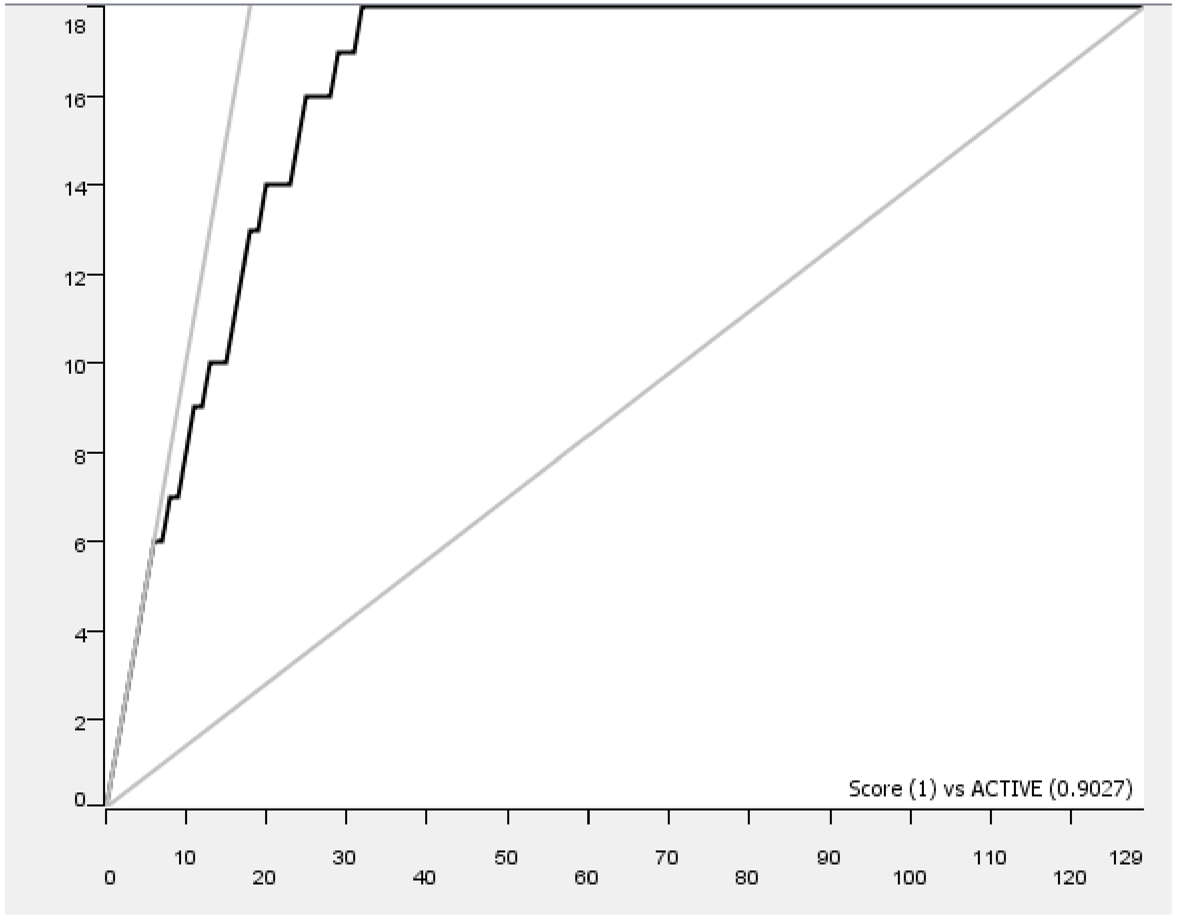

N=129

Top 10% Mean IC50	17.9 nM

	Predicted Active	Predicted Inactive
Active	16	2
Inactive	9	102

<15nM DefGood in beta-adren, 20% error; Random seed = 429

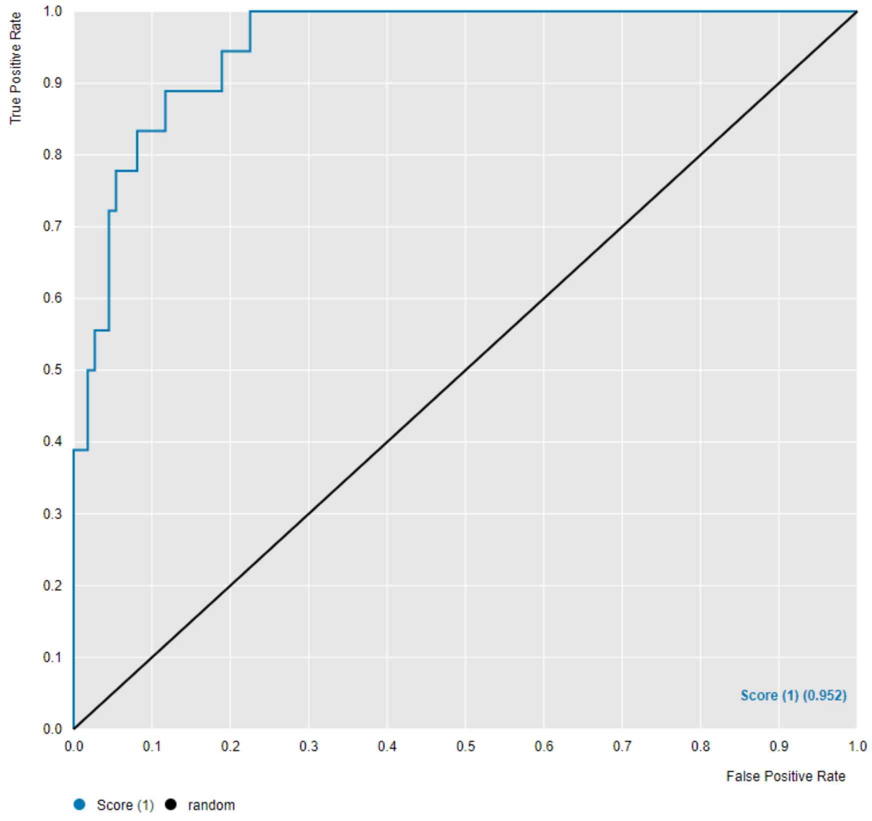

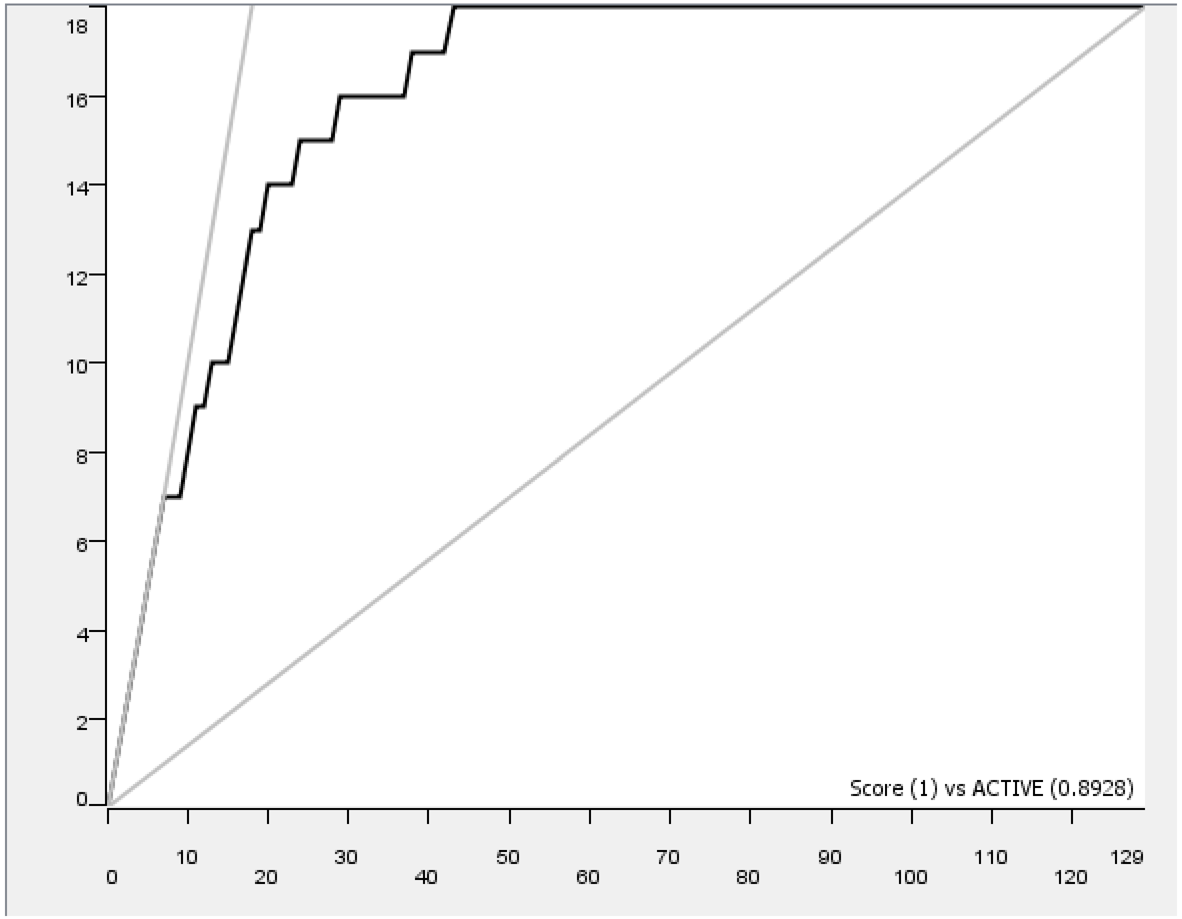

N=129

**Top 10% Mean IC50**	**19.7 nM**

	Predicted Active	Predicted Inactive
Active	15	3
Inactive	11	100

<15nM DefGood in beta-adren, 30% error; Random seed = 429

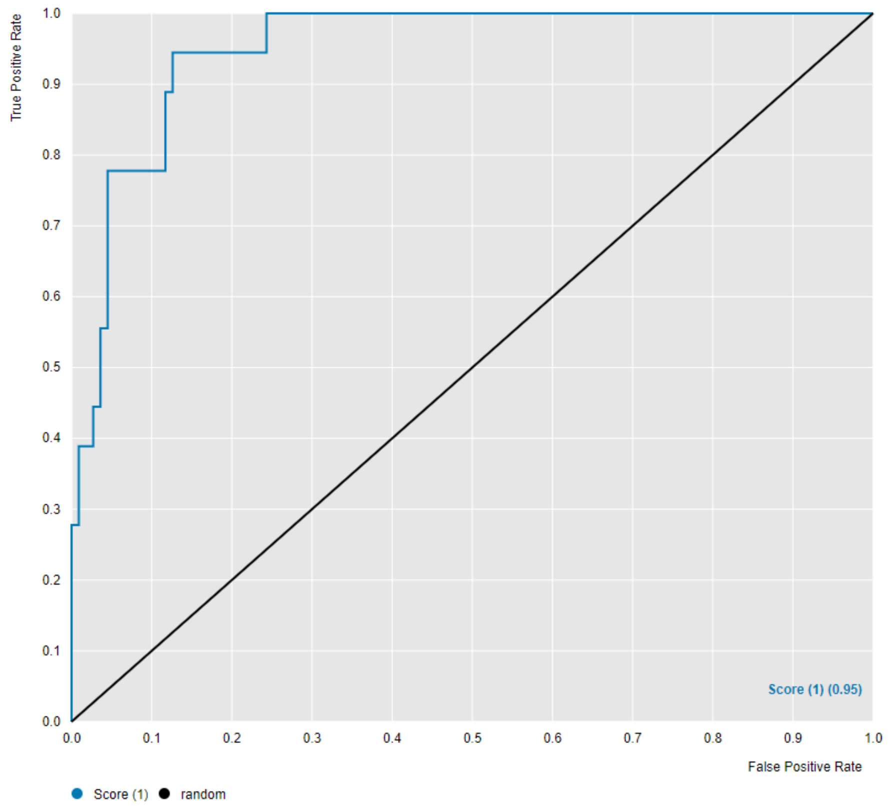

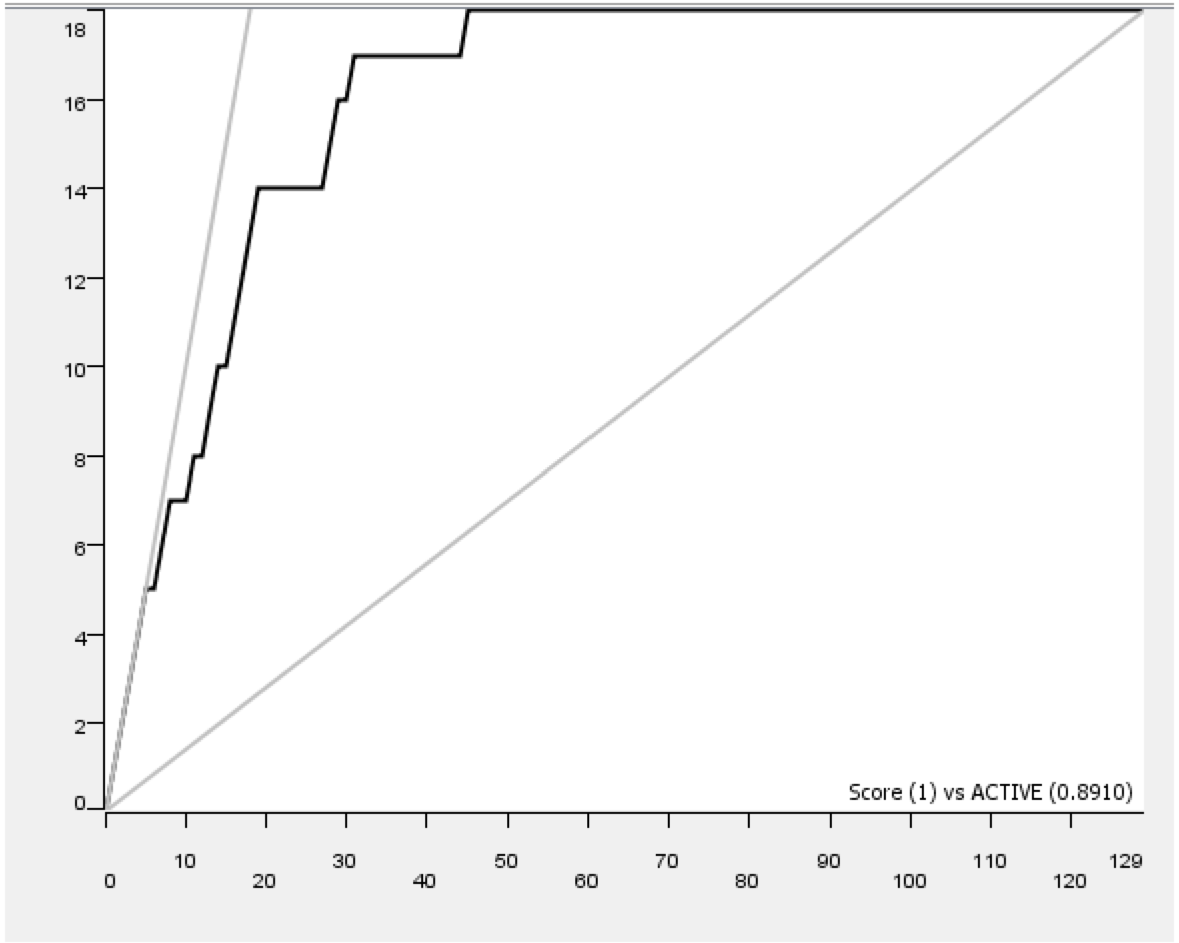

N=129

Top 10% Mean IC50	30.0 nM

	Predicted Active	Predicted Inactive
Active	17	1
Inactive	20	91

<15nM DefGood in beta-adren, 40% error; Random seed = 429

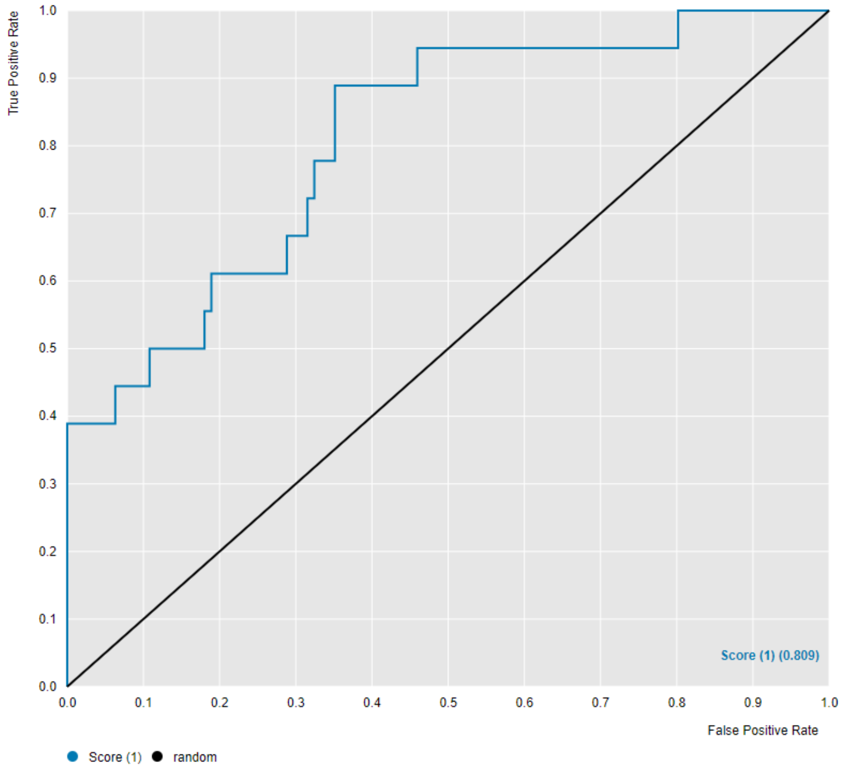

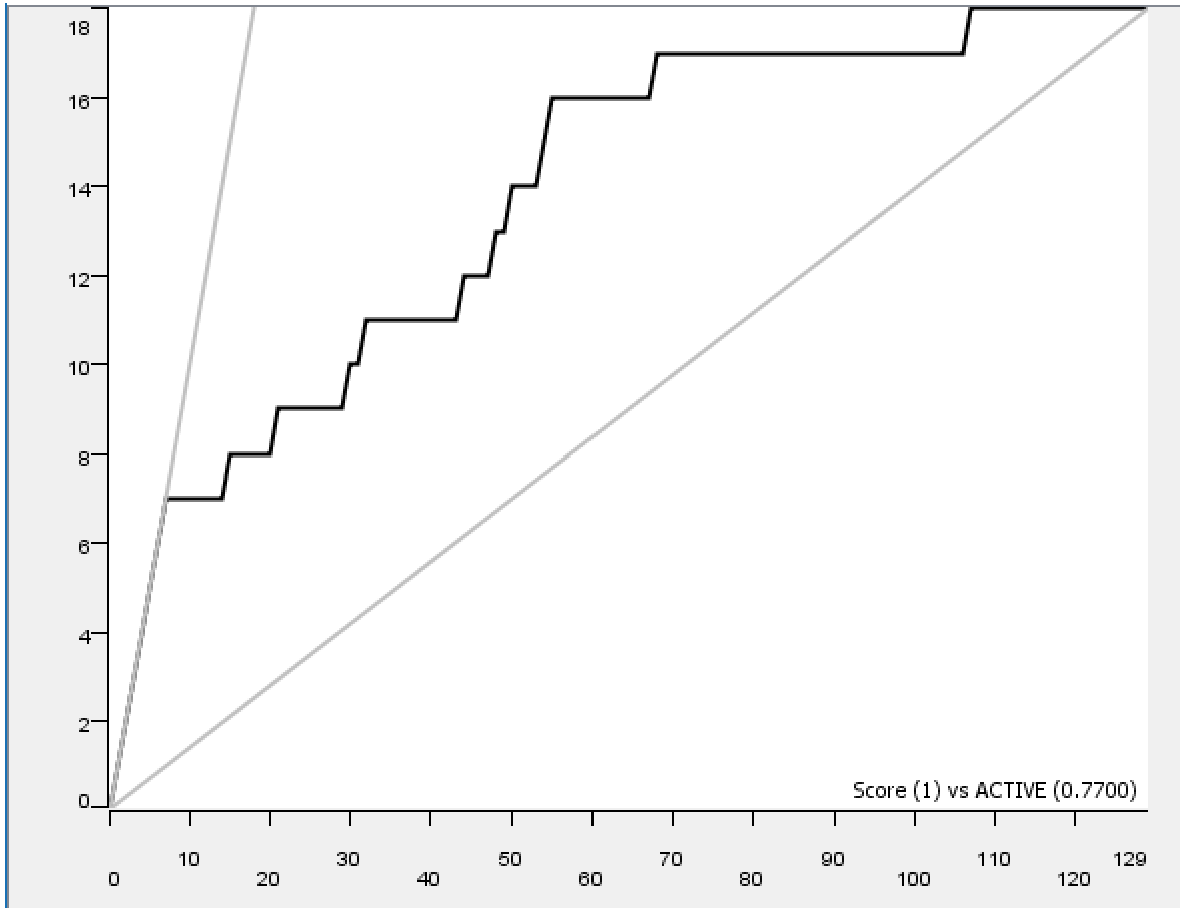

N=129

Top 10% Mean IC50	109 nM (one 900 nM mistake)

	Predicted Active	Predicted Inactive
Active	17	1
Inactive	67	44

<15nM DefGood in beta-adren, 45% error; Random seed = 429

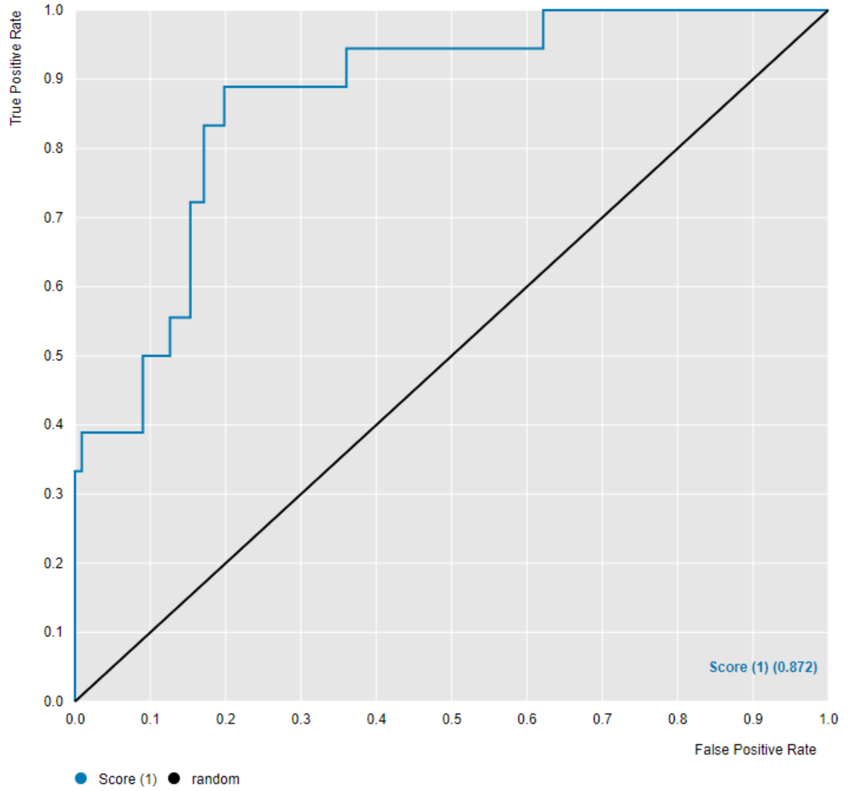

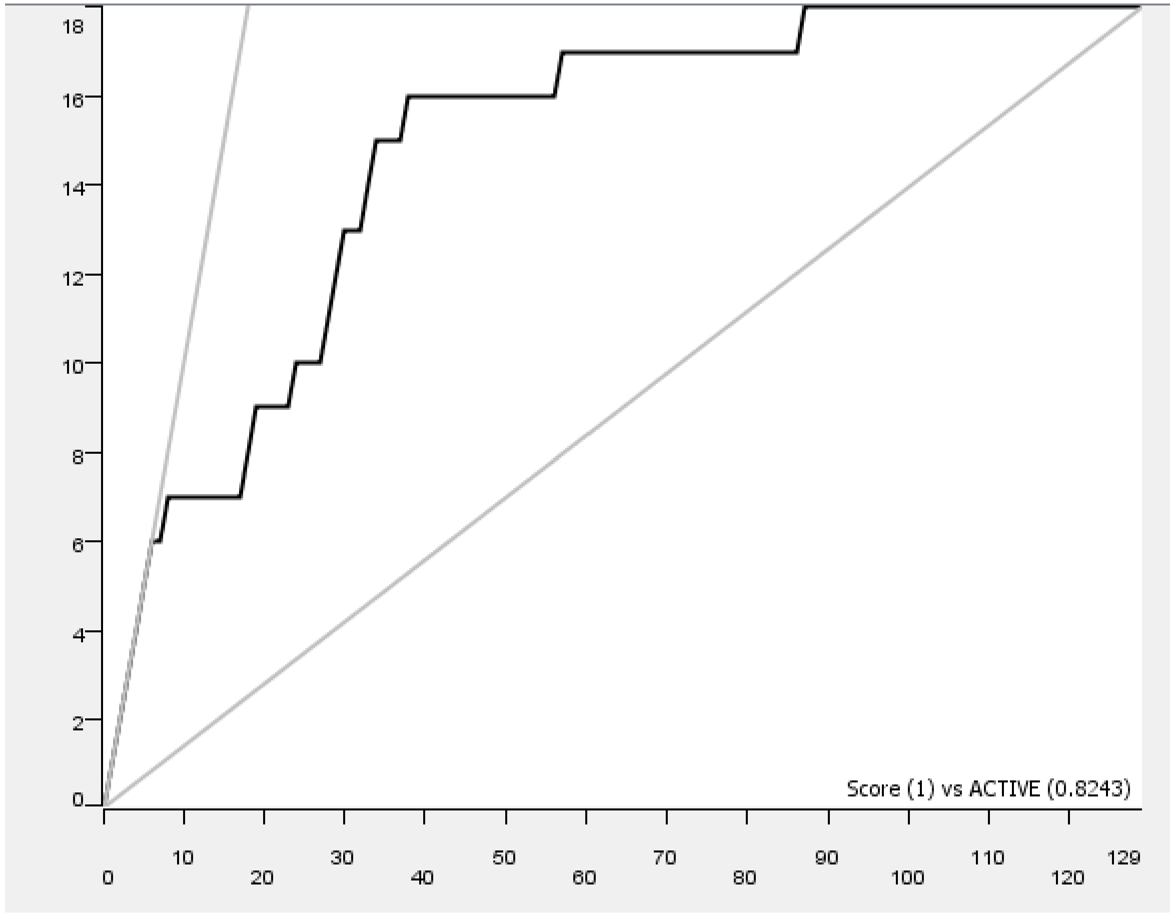

N=129

Top 10% Mean IC50	105 nM (one 900 nM mistake)

	Predicted Active	Predicted Inactive
Active	7	11
Inactive	2	109

<15nM DefGood in beta-adren, 50% error; Random seed = 429

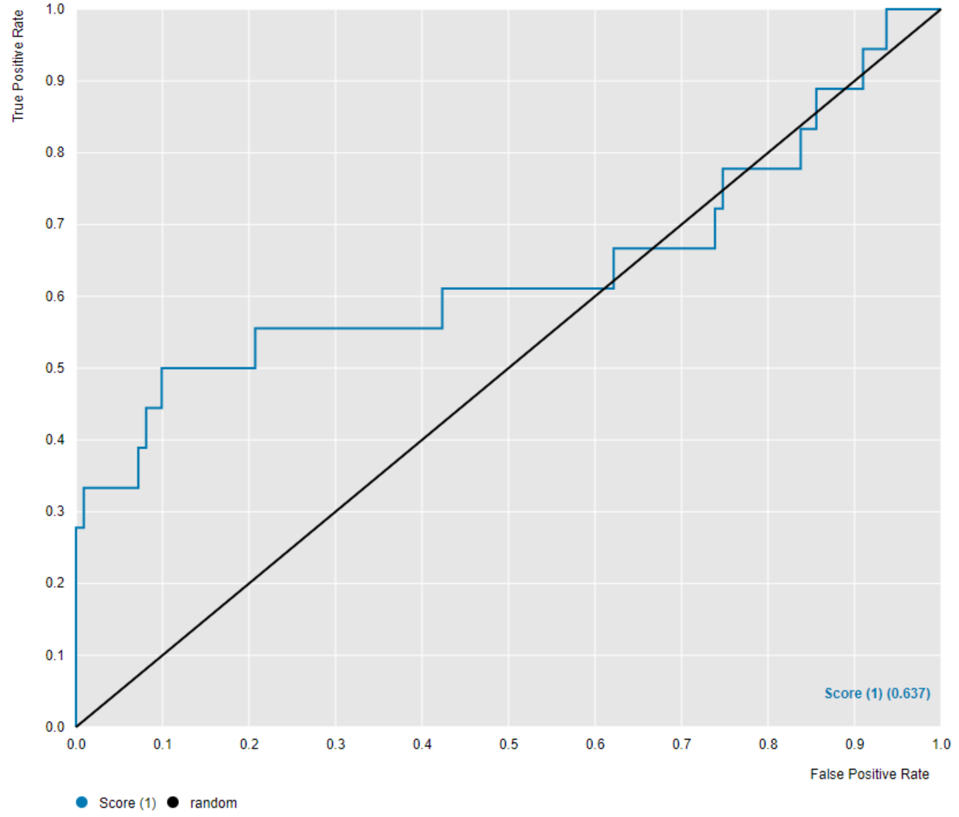

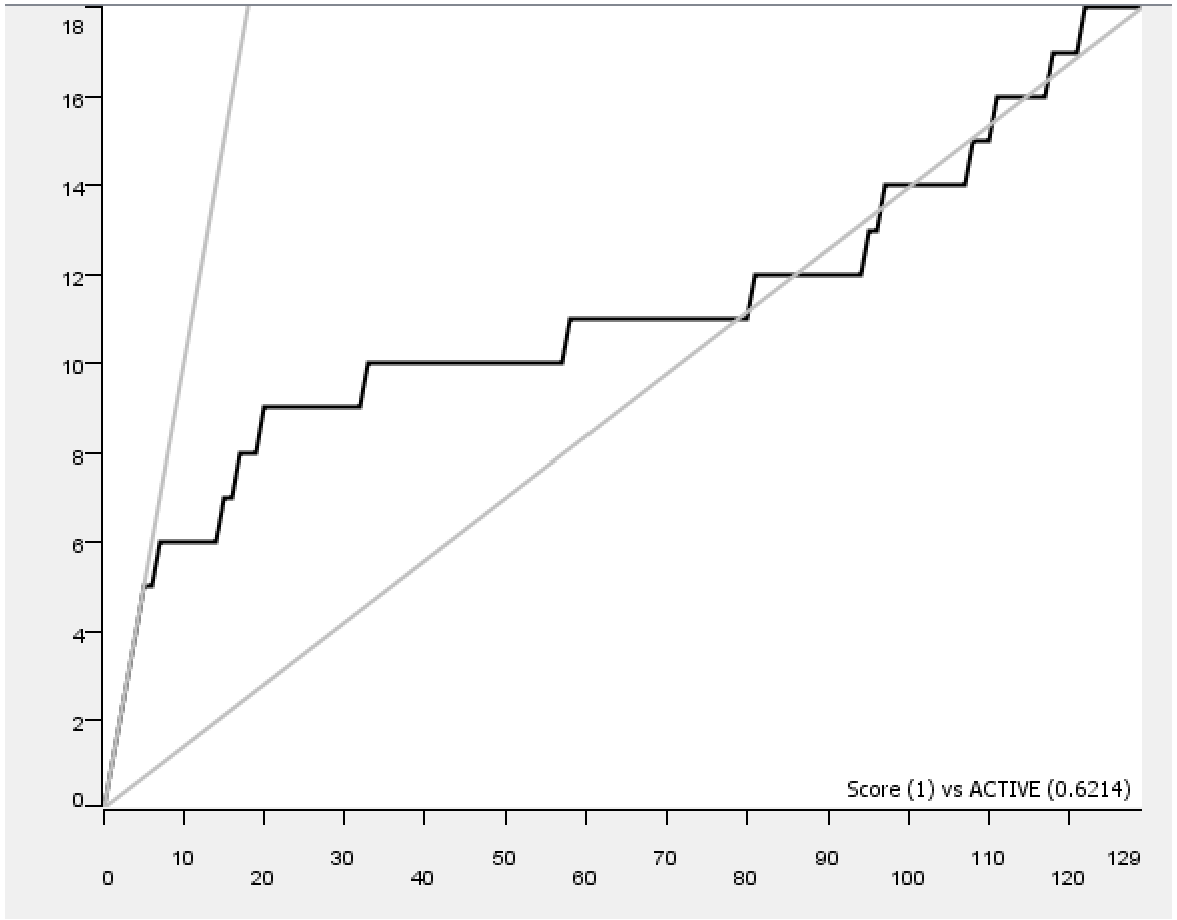

N=129

Top 10% Mean IC50	1,900 nM

	Predicted Active	Predicted Inactive
Active	8	10
Inactive	11	100

<15nM DefGood in beta-adren, 10% error; Random seed = 121783

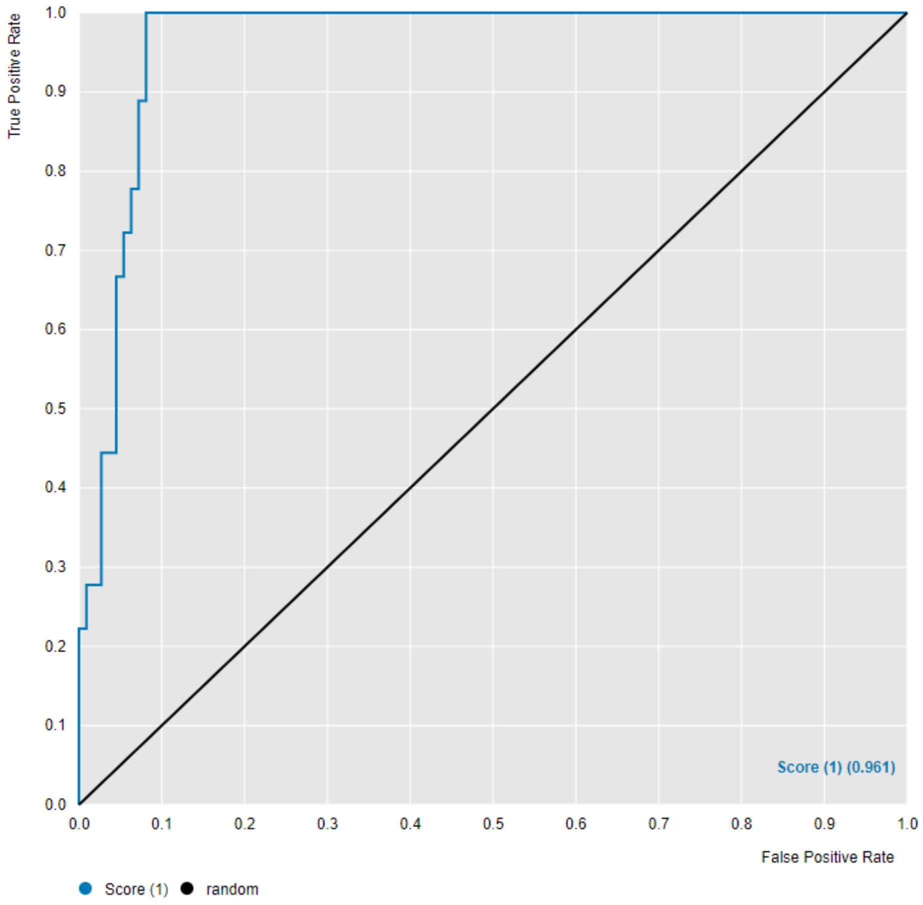

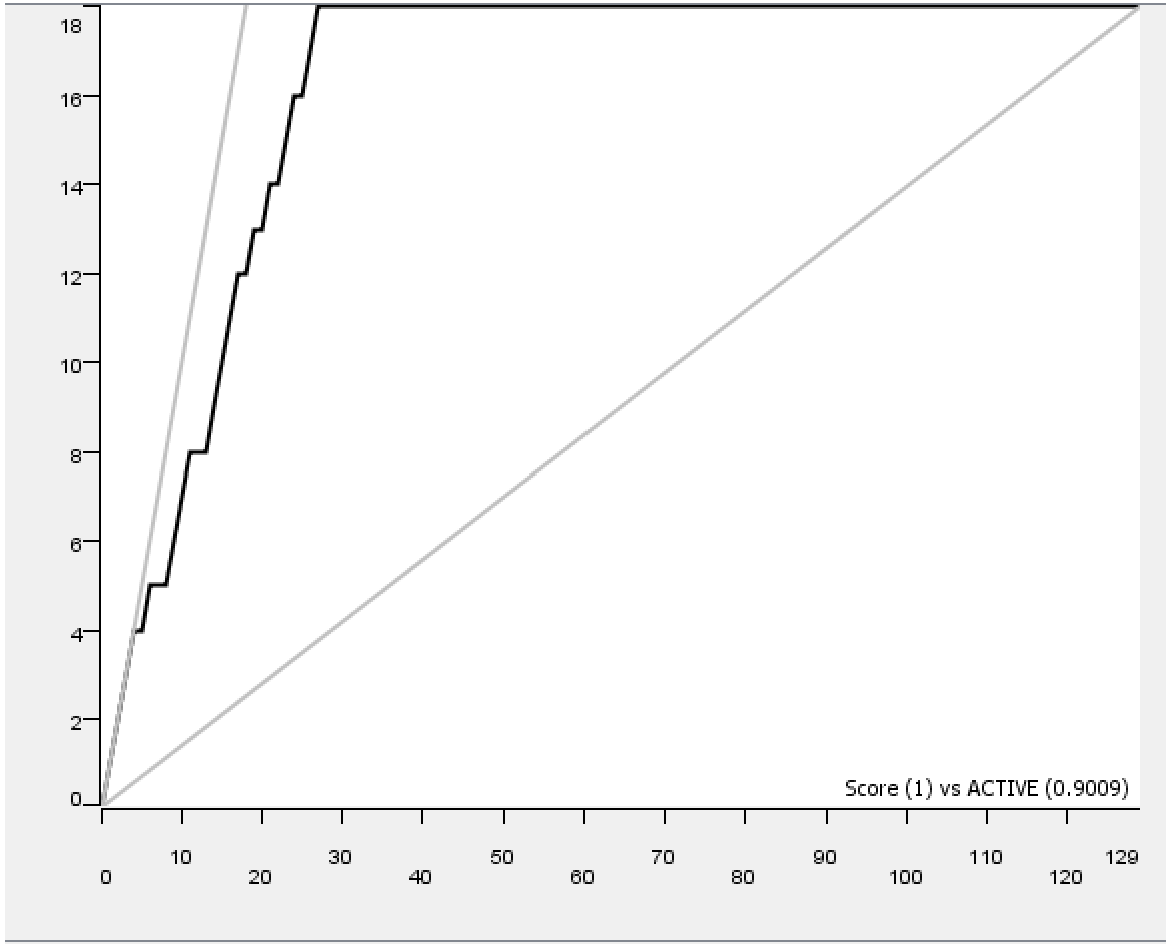

N=129

**Top 10% Mean IC50**	**18.5 nM**

	**Predicted Active**	**Predicted Inactive**
Active	18	0
Inactive	12	99

<15nM DefGood in beta-adren, 20% error; Random seed = 121783

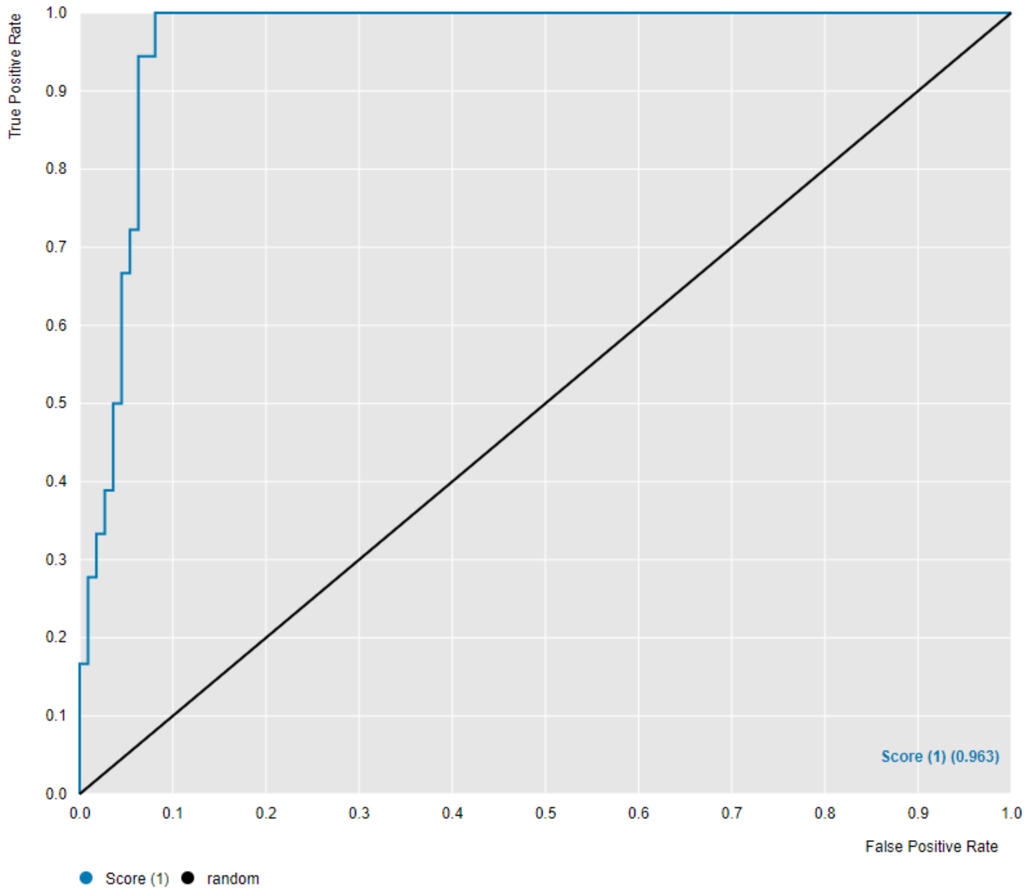

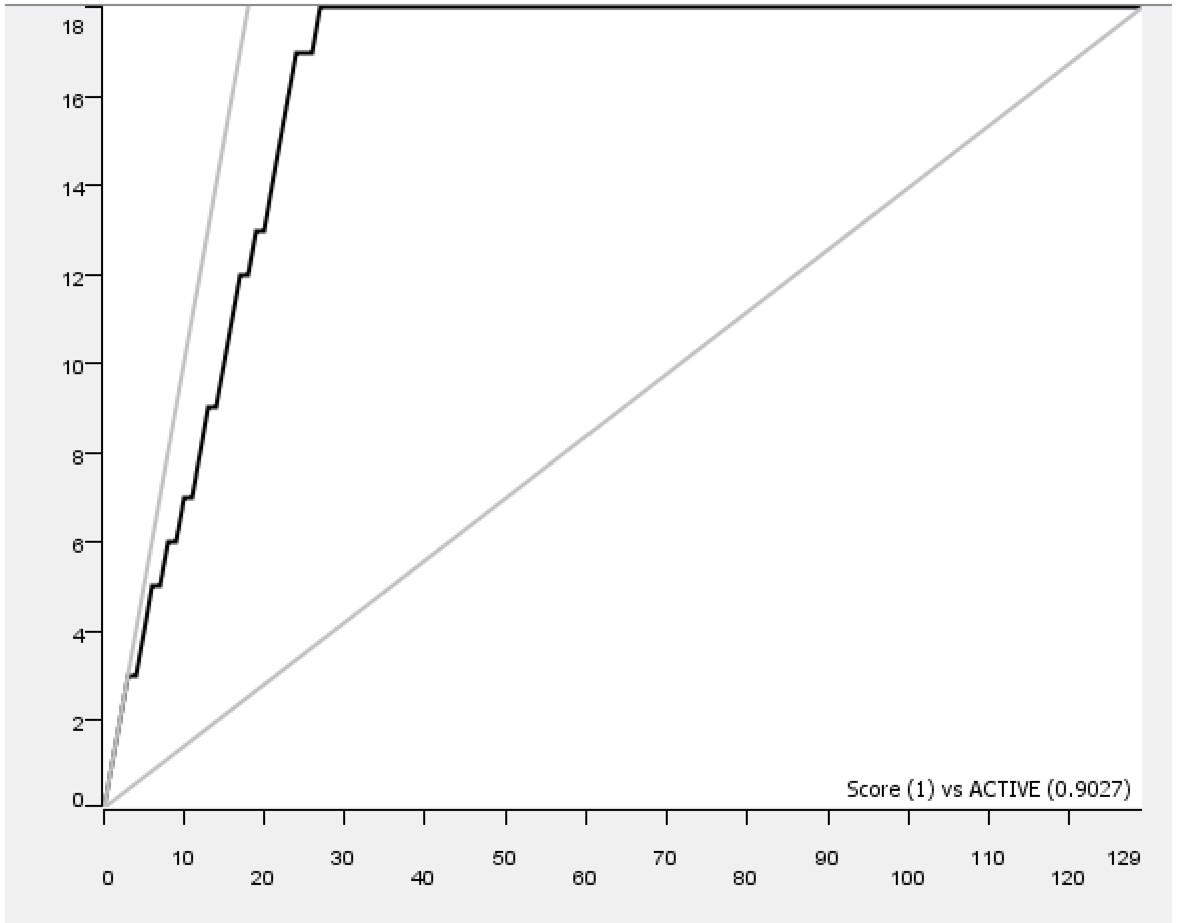

N=129

Top 10% Mean IC50	18.5 nM

	Predicted Active	Predicted Inactive
Active	18	0
Inactive	12	99

<15nM DefGood in beta-adren, 30% error; Random seed = 121783

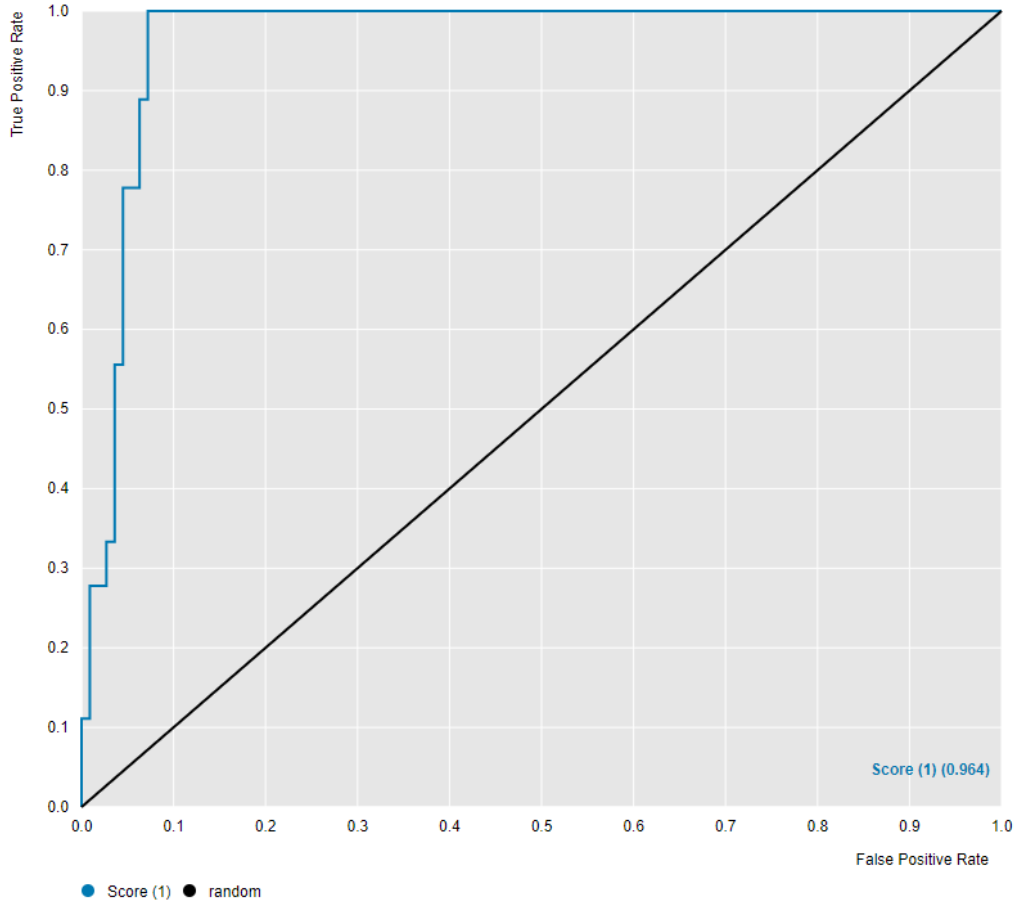

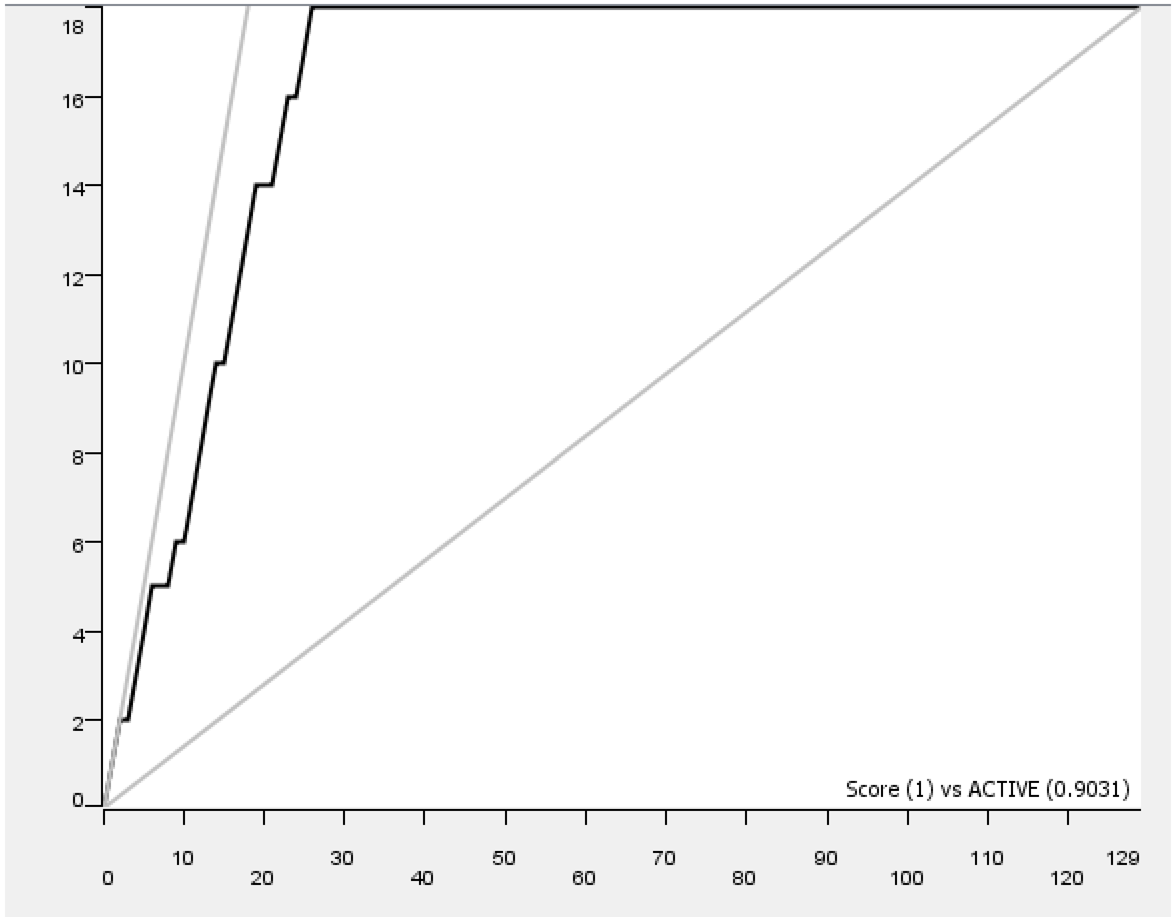

N=270

Top 10% Mean IC50	18.4 nM

	Predicted Active	Predicted Inactive
Active	18	0
Inactive	20	91

<15nM DefGood in beta-adren, 40% error; Random seed = 121783

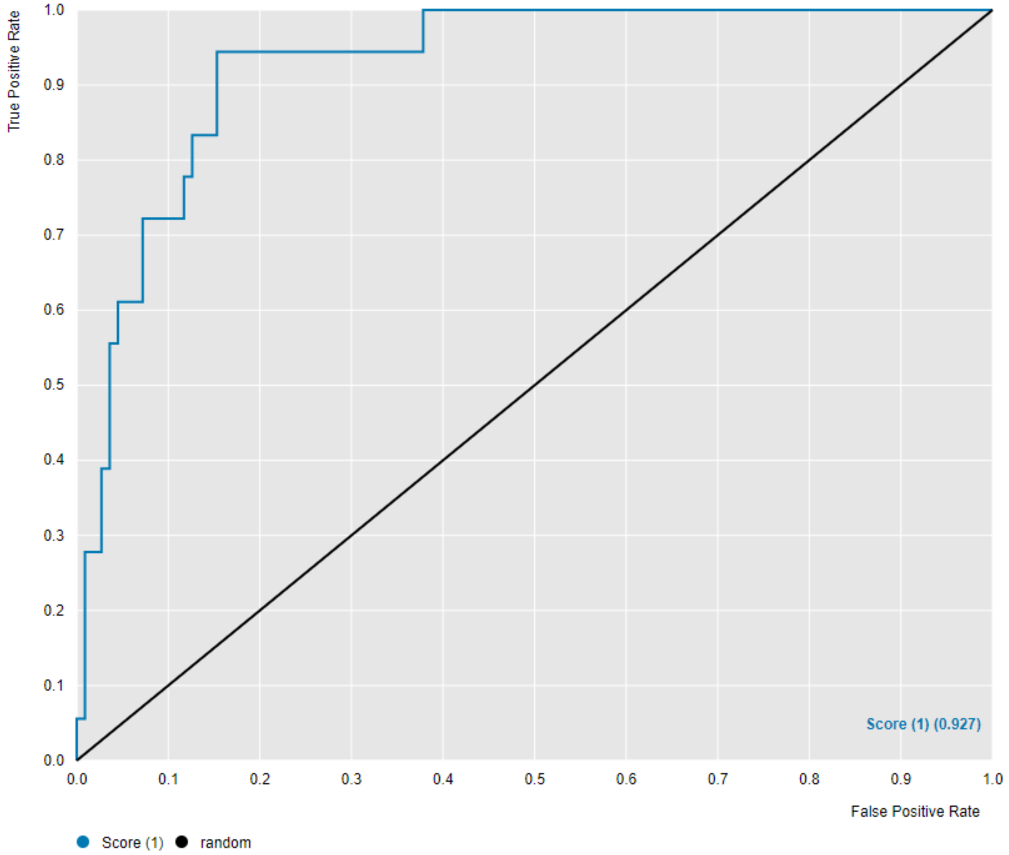

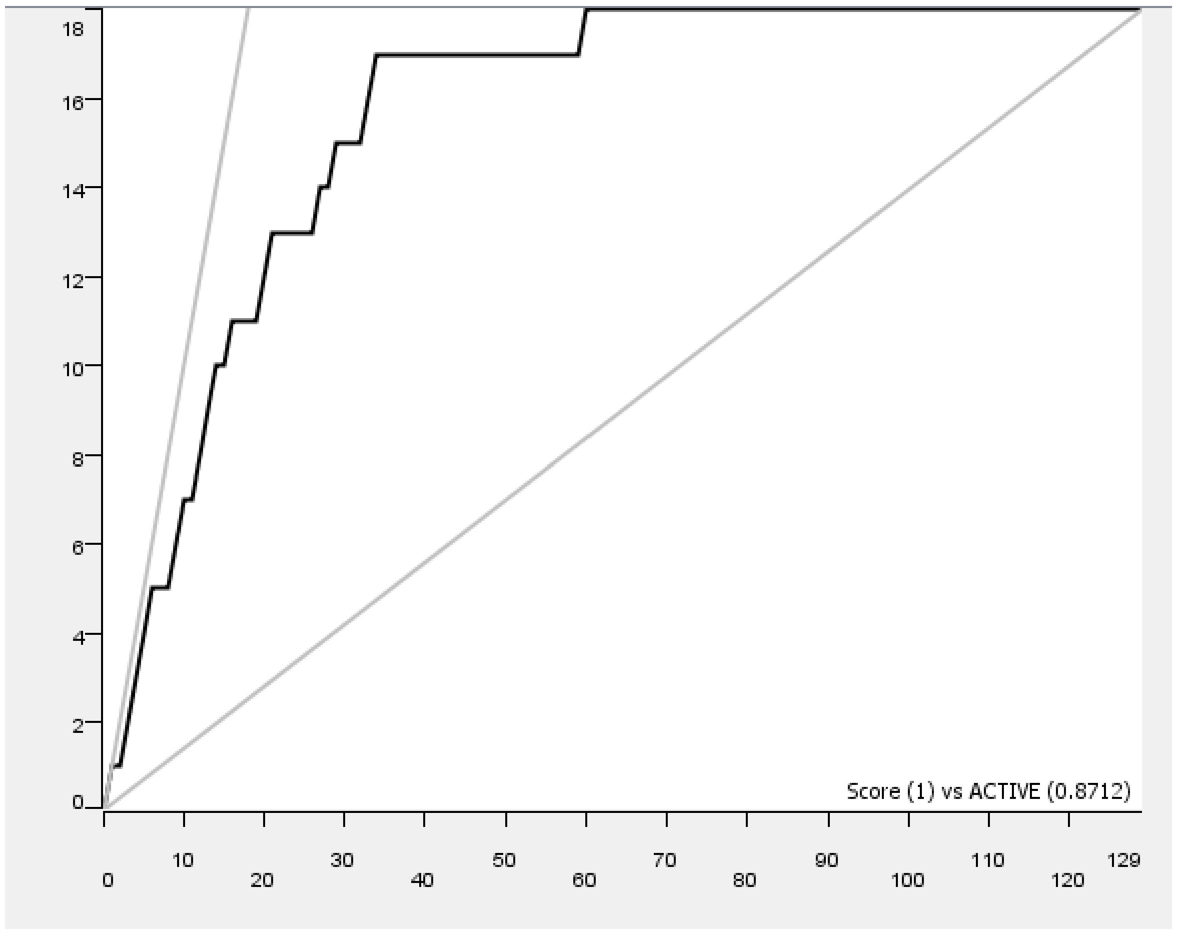

N=129

Top 10% Mean IC50	18.4 nM

	Predicted Active	Predicted Inactive
Active	17	1
Inactive	31	80

<15nM DefGood in beta-adren, 45% error; Random seed = 121783

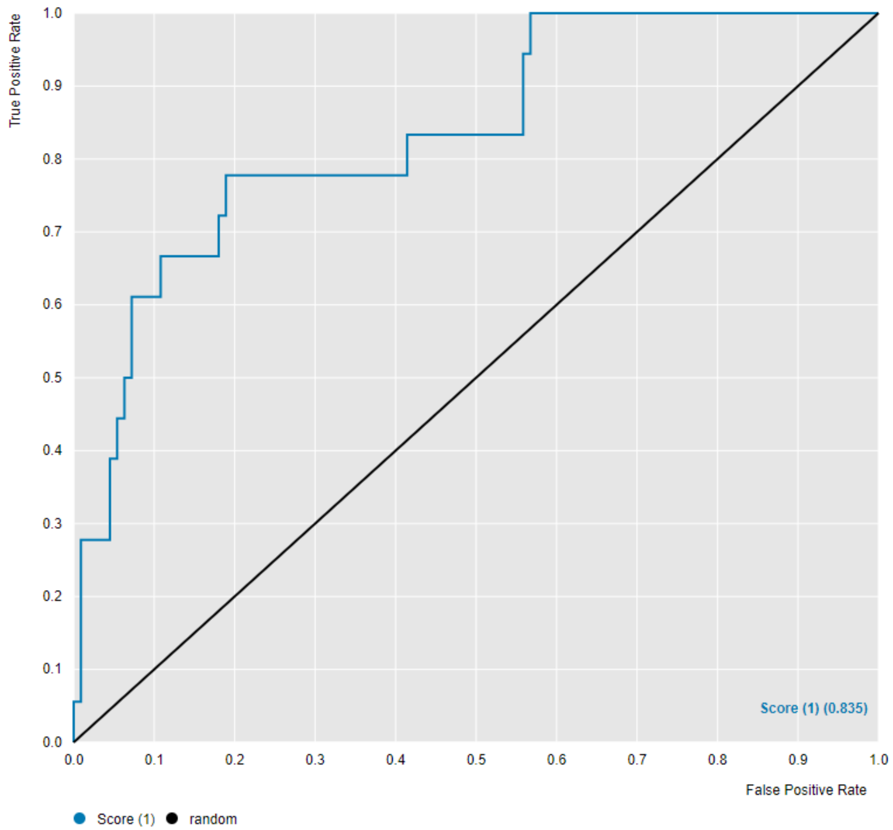

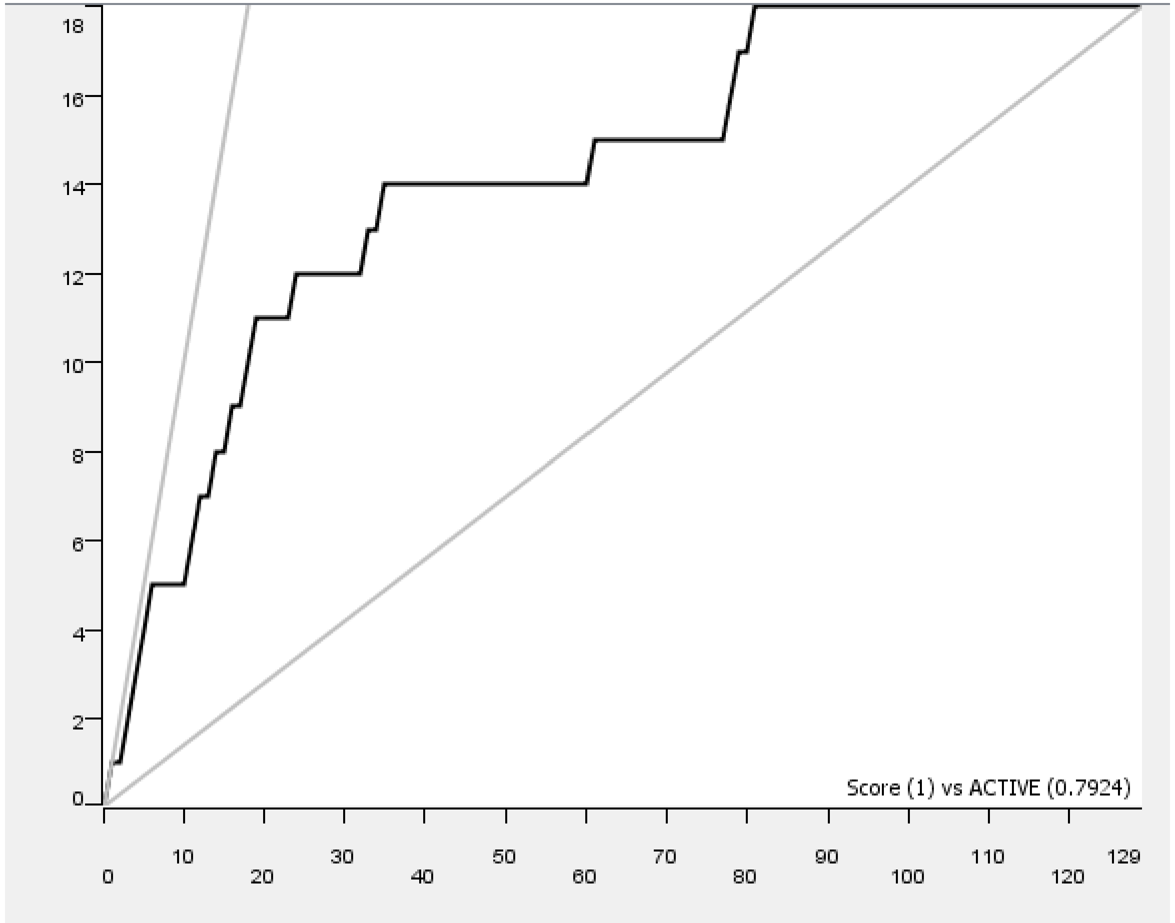

N=129

Top 10% Mean IC50	33.1 nM

	Predicted Active	Predicted Inactive
Active	14	4
Inactive	30	81

<15nM DefGood in beta-adren, 50% error; Random seed = 121783

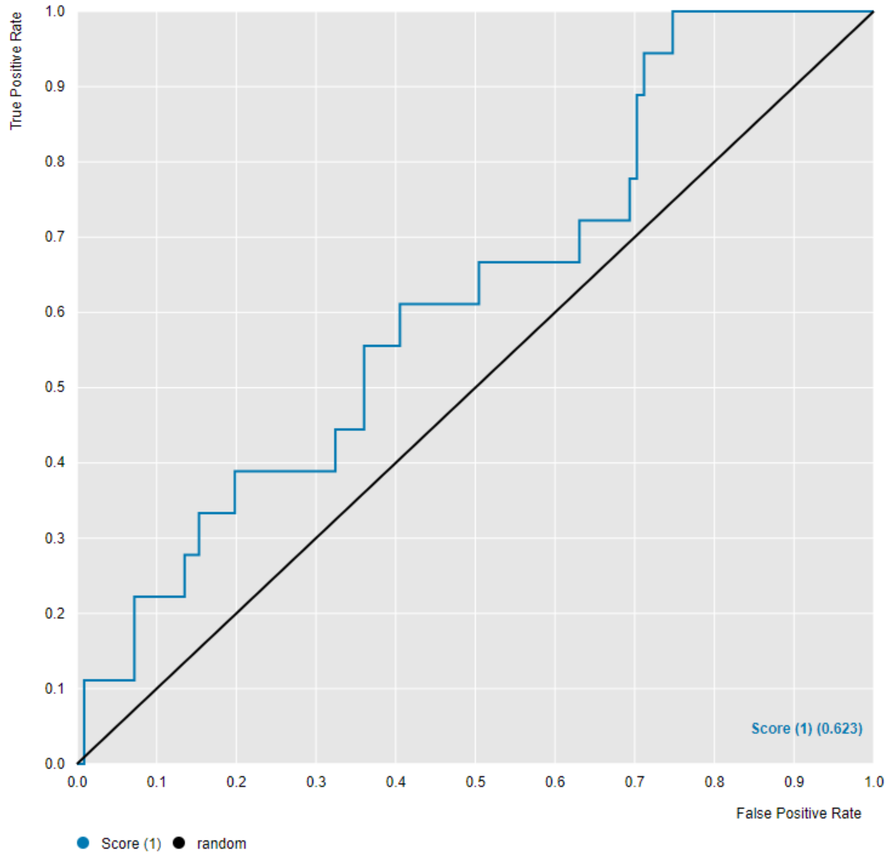

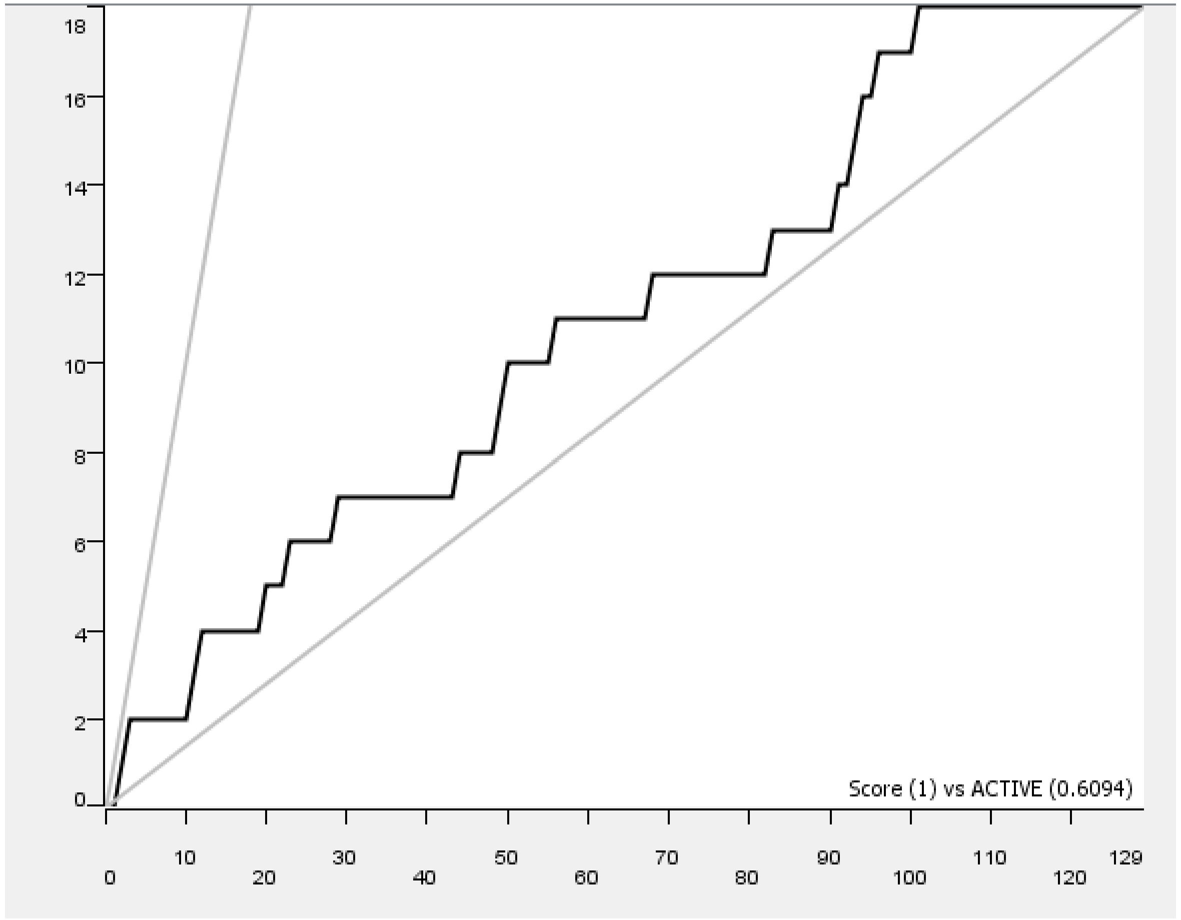

N=129

Top 10% Mean IC50	250.5 nM

	Predicted Active	Predicted Inactive
Active	10	8
Inactive	42	69

Conclusion - NBN

- An NBN could be generated for the β2 adrenergic receptor with a decision value of <15 nM as defined as active. The predictivity of the algorithm could be preserved in three random train/test cycles. The point of failure was 50%, 50% and 50% error.

RF Error Tolerance- <15 nM DefGood in Beta Adrenergic Receptor

- 0-50% absolute error

RF- <15nM DefGood in beta-adren, 5% error; Random seed = 1515533876005

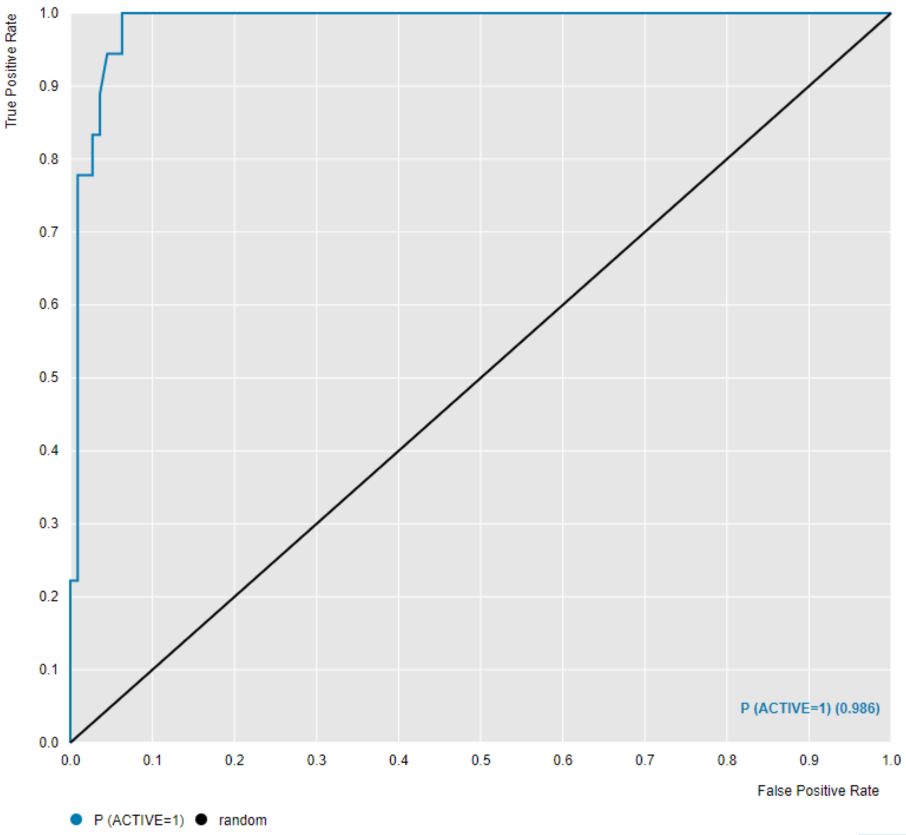

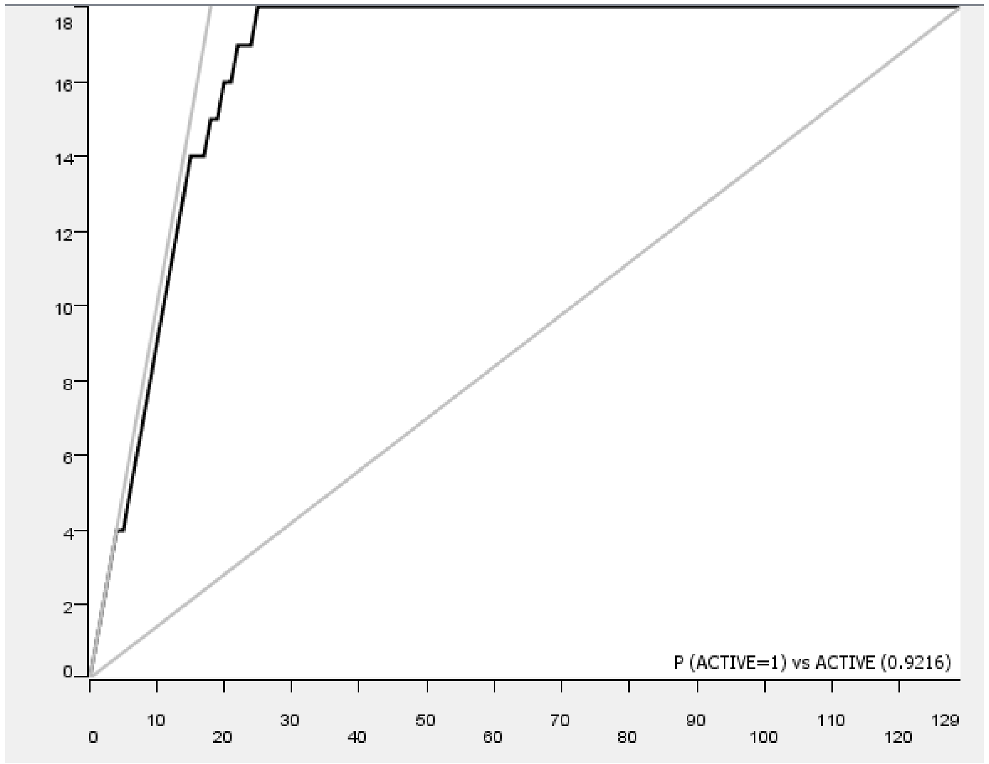

N=129

Top 10% Mean IC50	4.8 nM

	Predicted Active	Predicted Inactive
Active	15	3
Inactive	3	108

RF- <15nM DefGood in beta-adren, 10% error

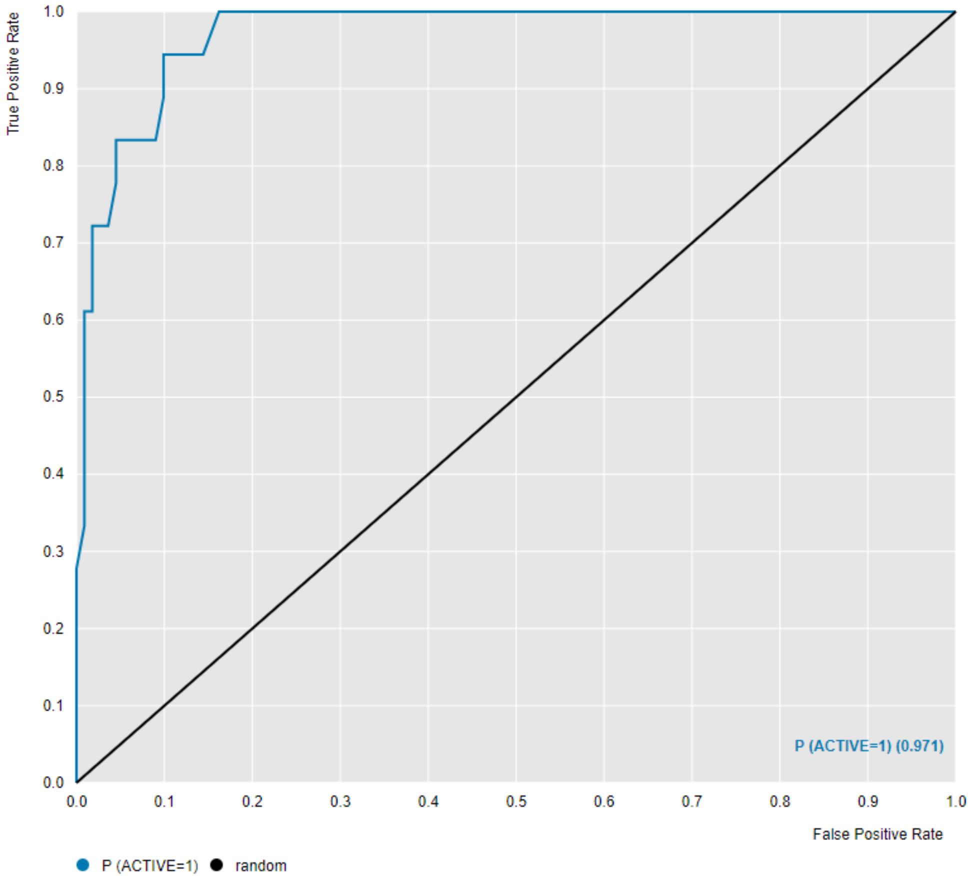

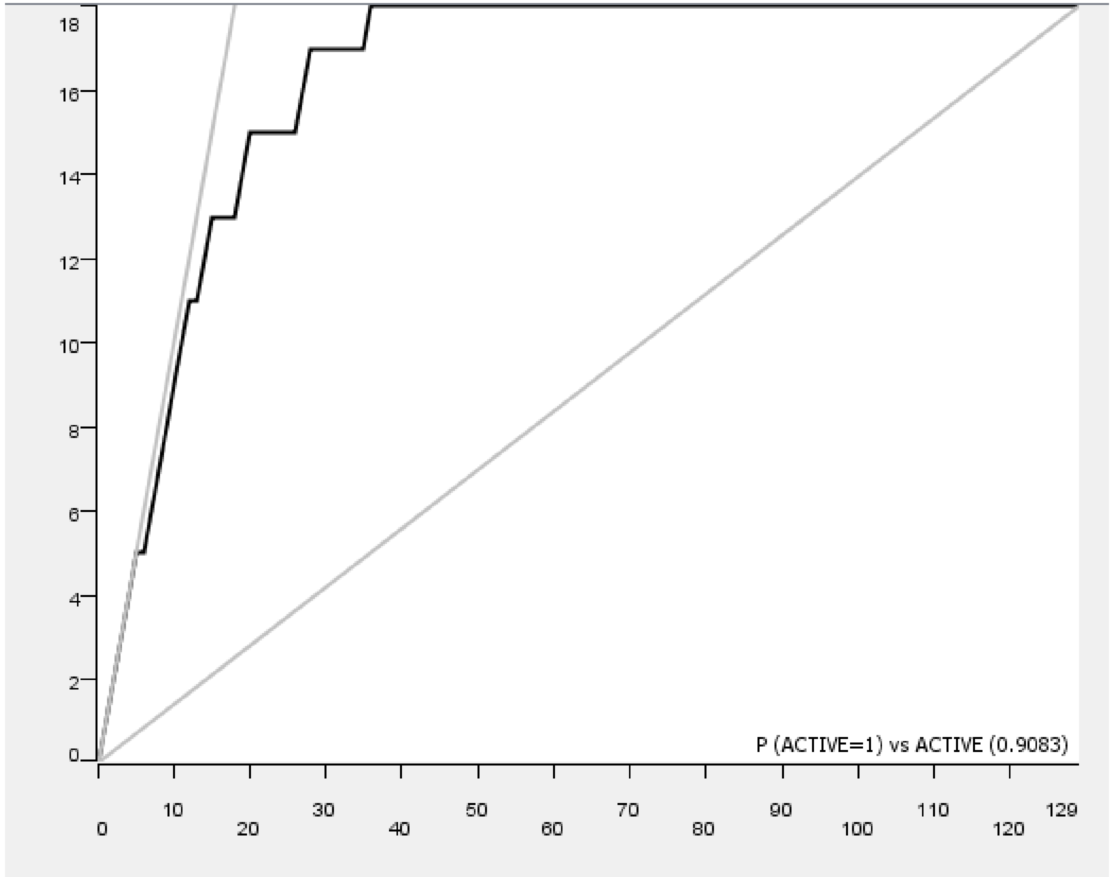

N=129

Top 10% Mean IC50	4.7 nM

	Predicted Active	Predicted Inactive
Active	15	3
Inactive	6	105

RF- <15nM DefGood in beta-adren, 20% error

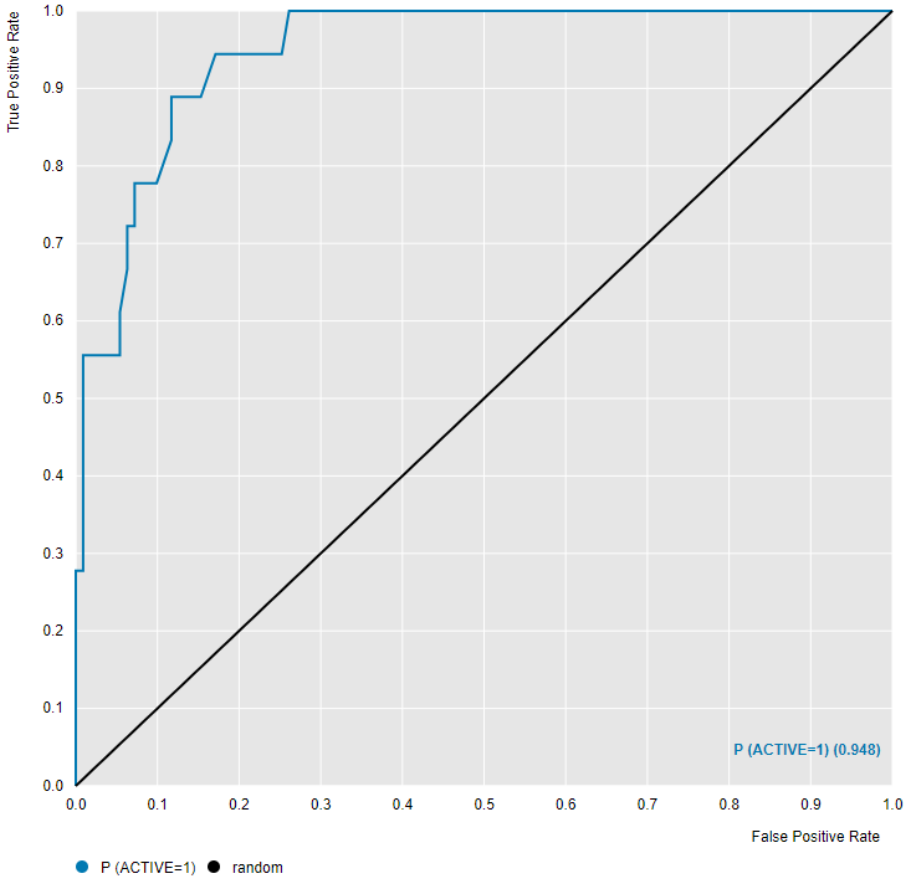

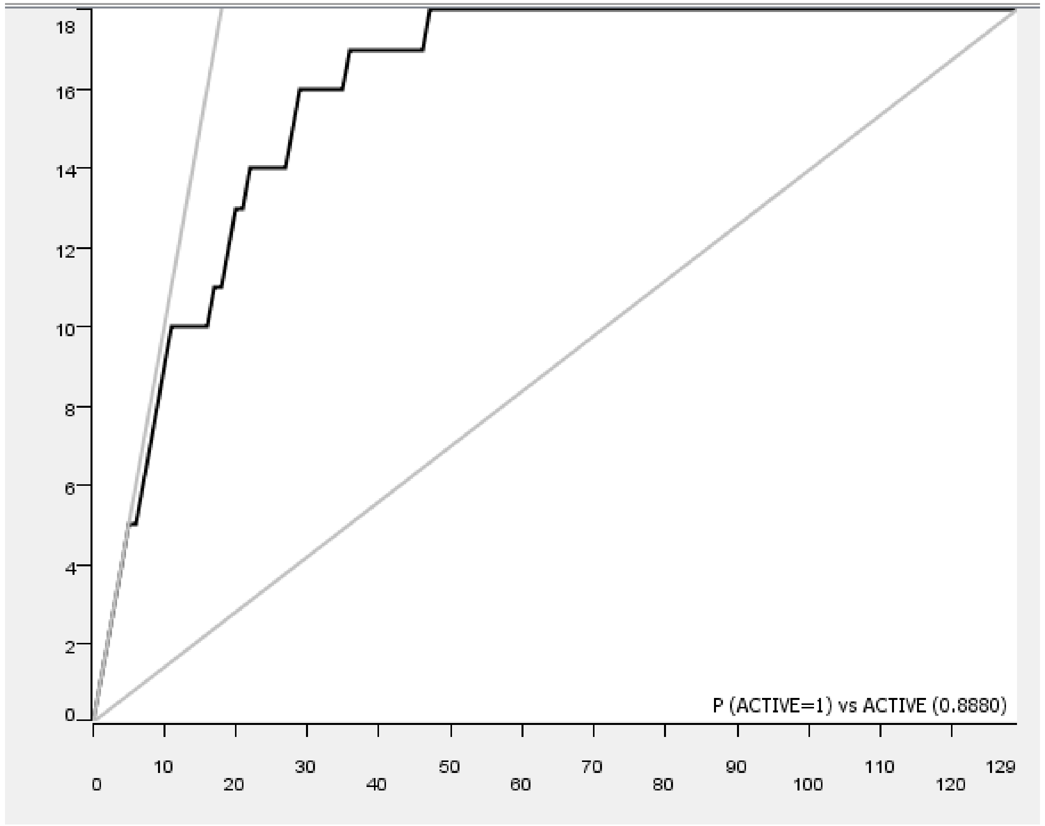

N=129

Top 10% Mean IC50	95 nM

	Predicted Active	Predicted Inactive
Active	14	4
Inactive	8	103

RF- <15nM DefGood in beta-adren, 30% error

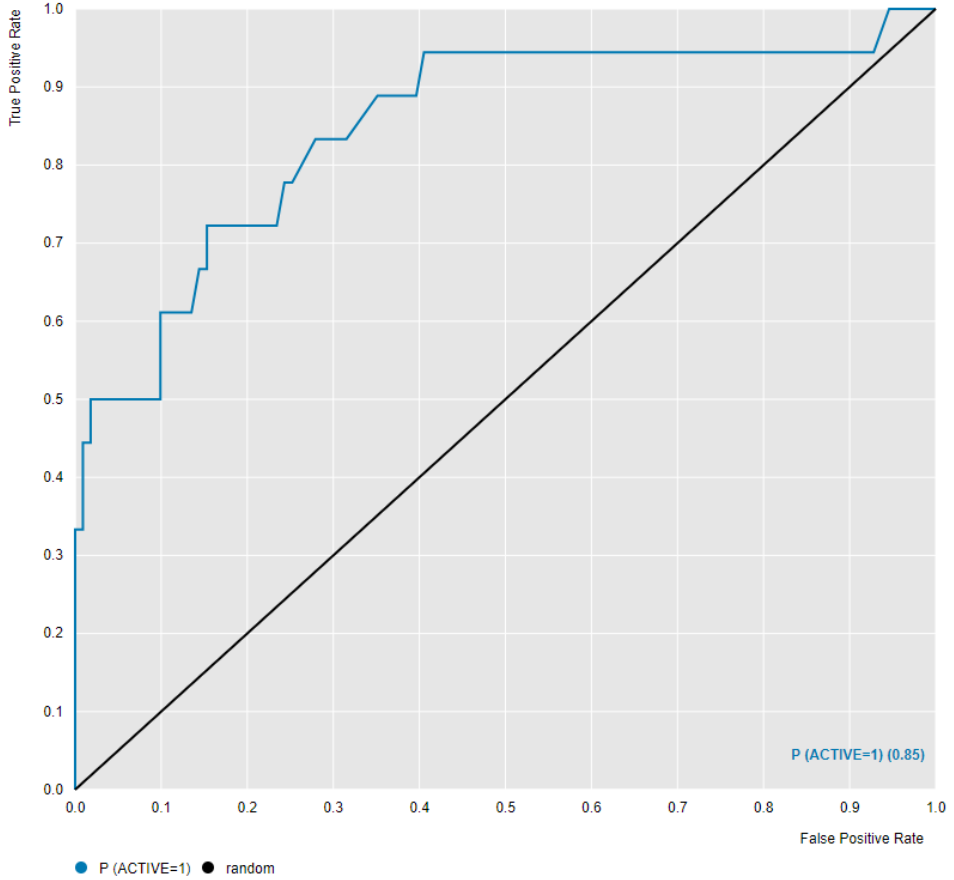

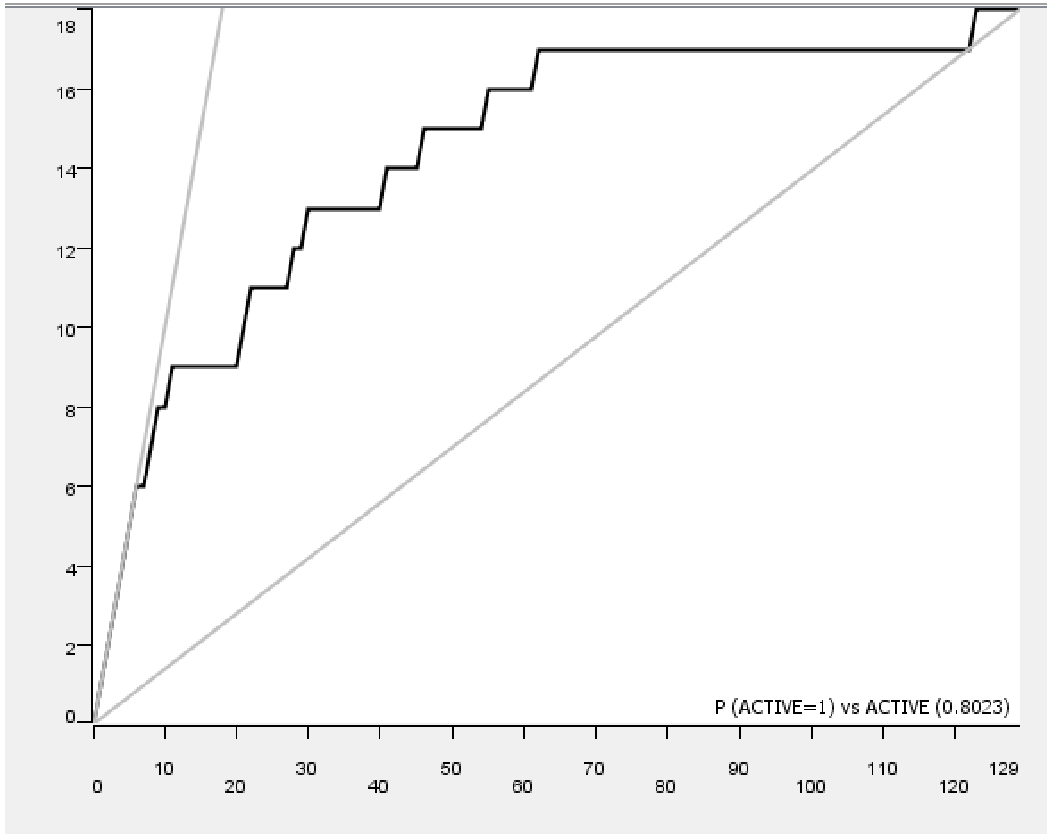

N=129

**Top 10% Mean IC50**	**130 nM**

	Predicted Active	Predicted Inactive
Active	13	5
Inactive	19	92

RF- <15nM DefGood in beta-adren, 35% error

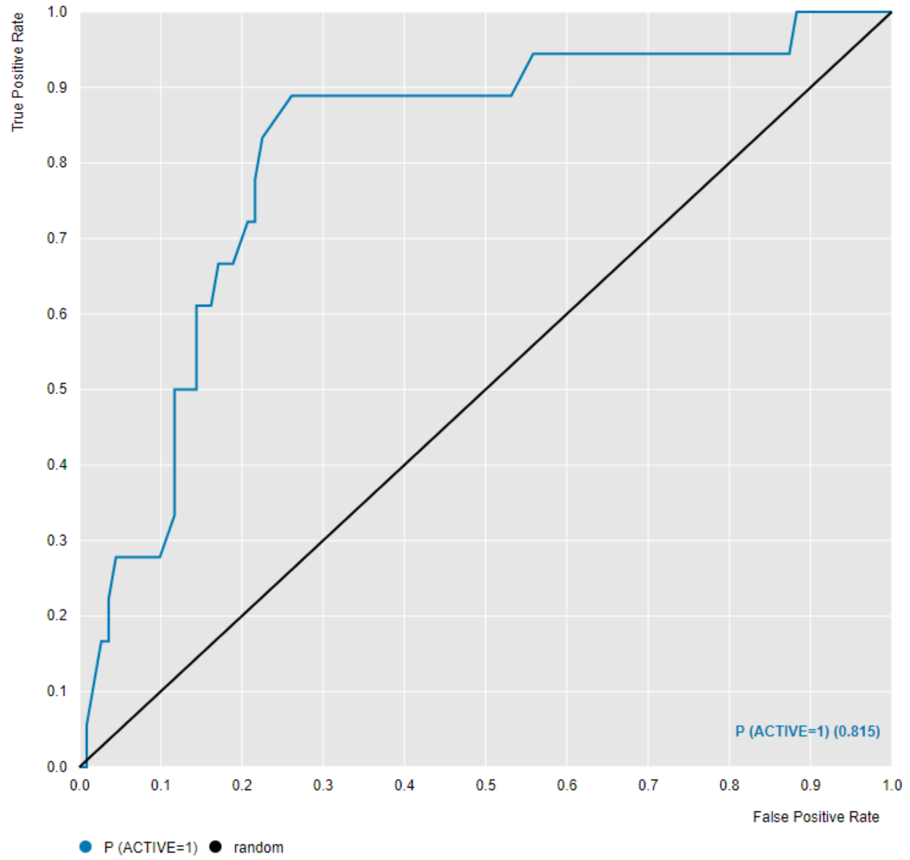

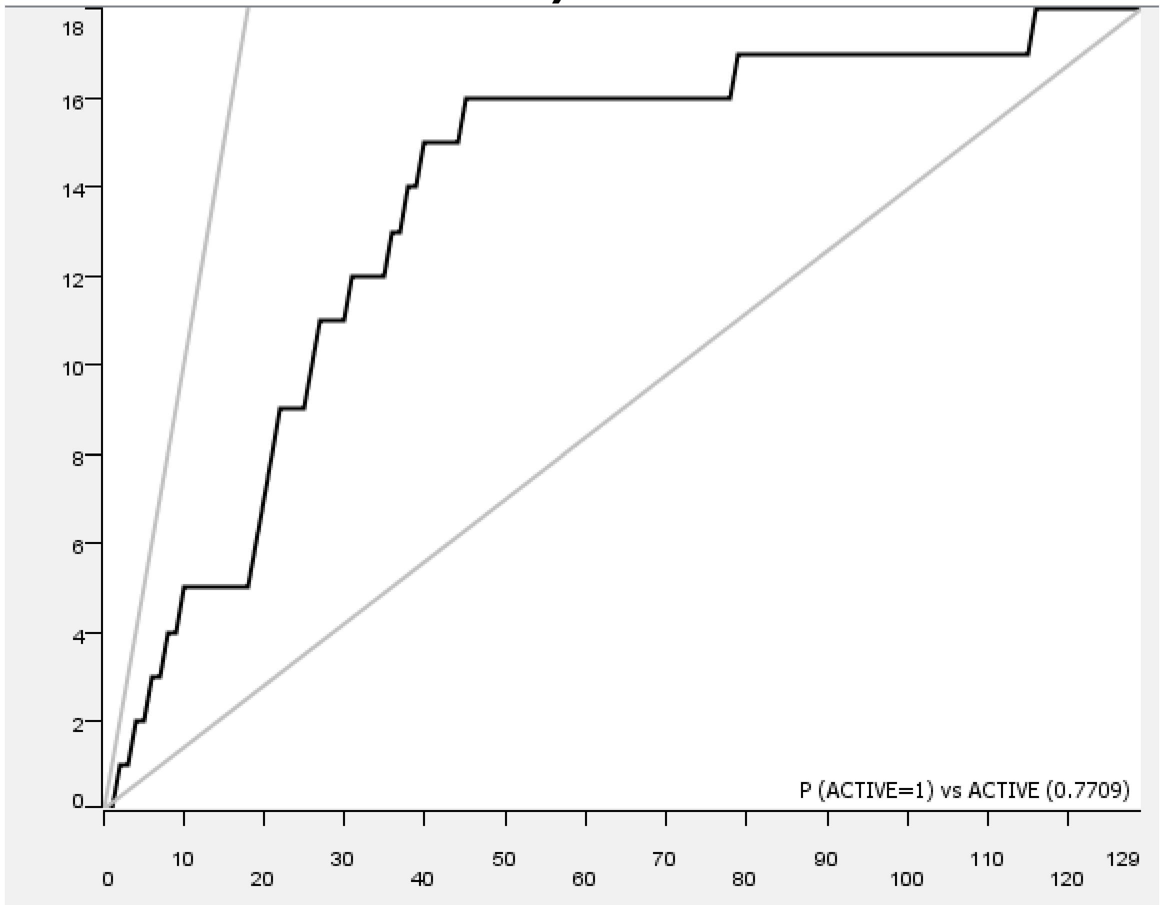

N=129

Top 10% Mean IC50	3,700 nM

	Predicted Active	Predicted Inactive
Active	13	5
Inactive	24	87

<15nM DefGood in beta-adren, 10% error; Random seed = 429

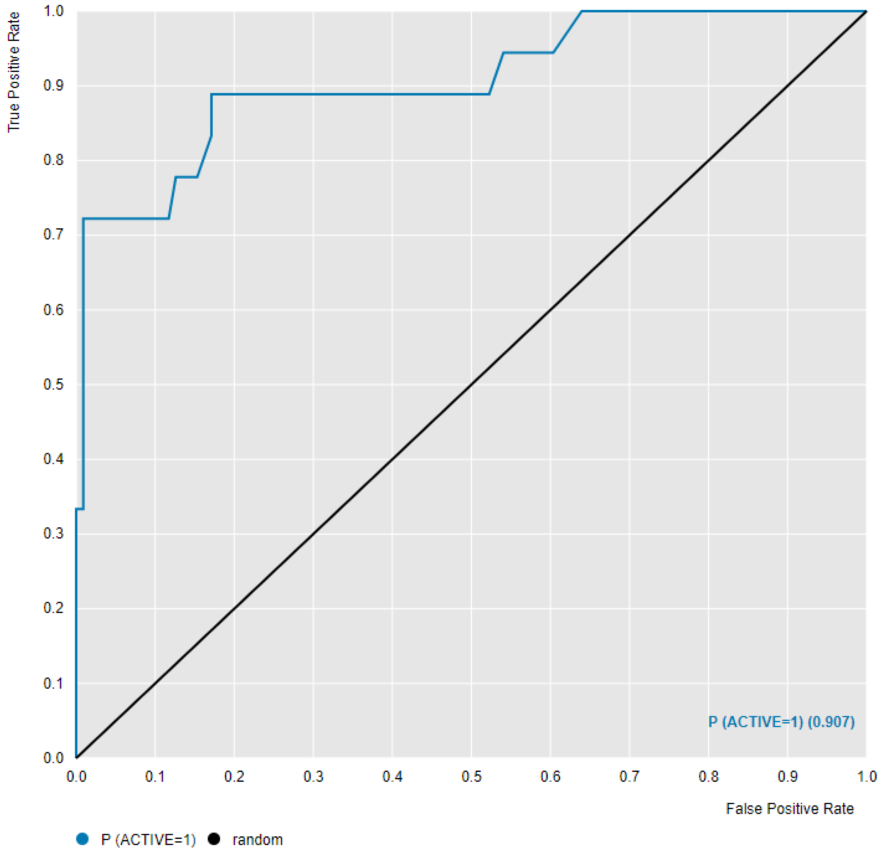

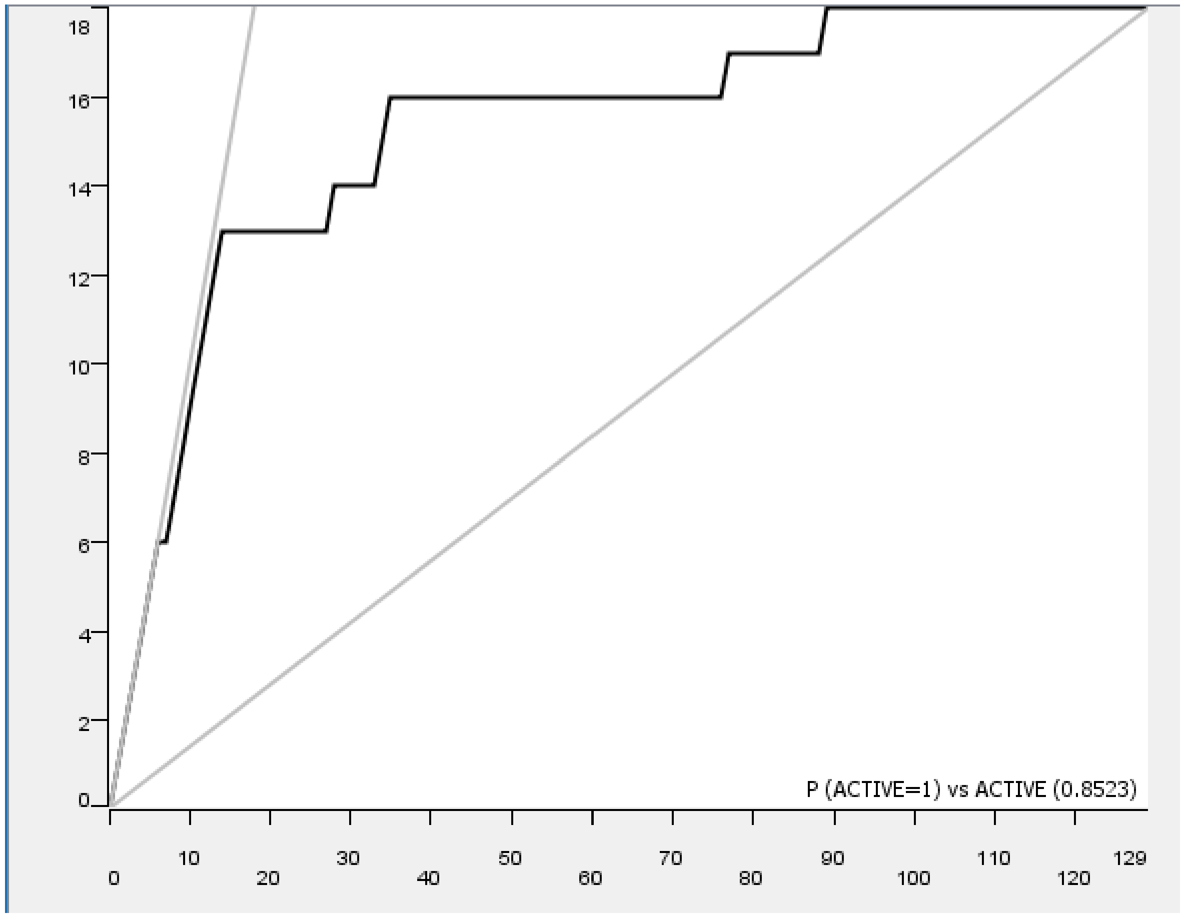

N=129

Top 10% Mean IC50	7.9 nM

	Predicted Active	Predicted Inactive
Active	13	5
Inactive	6	105

<15nM DefGood in beta-adren, 20% error; Random seed = 429

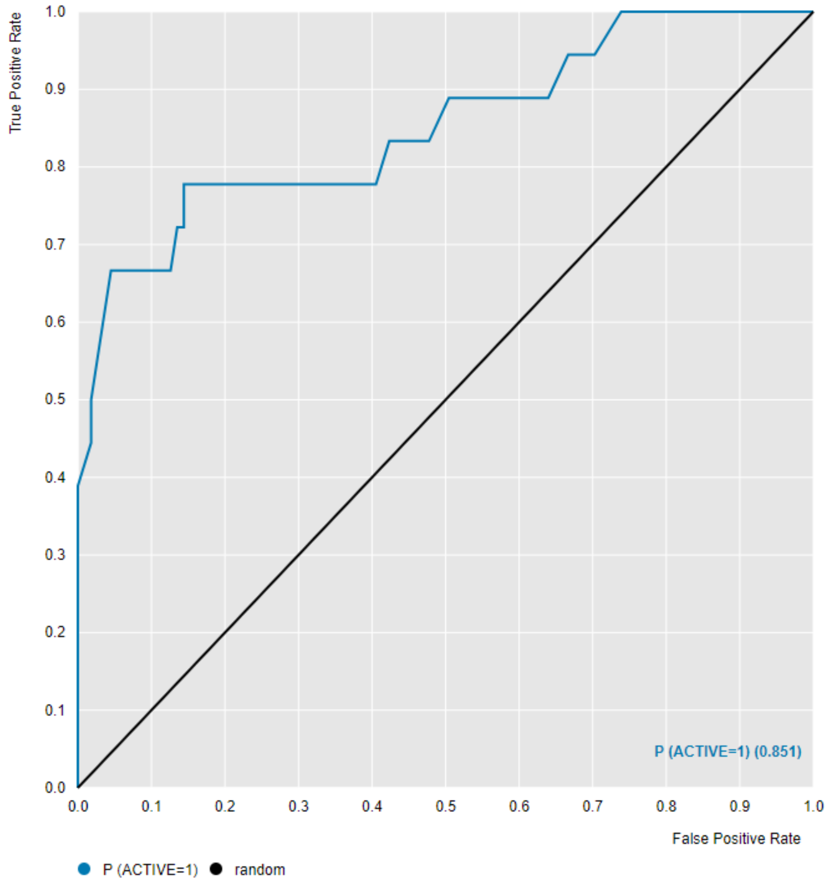

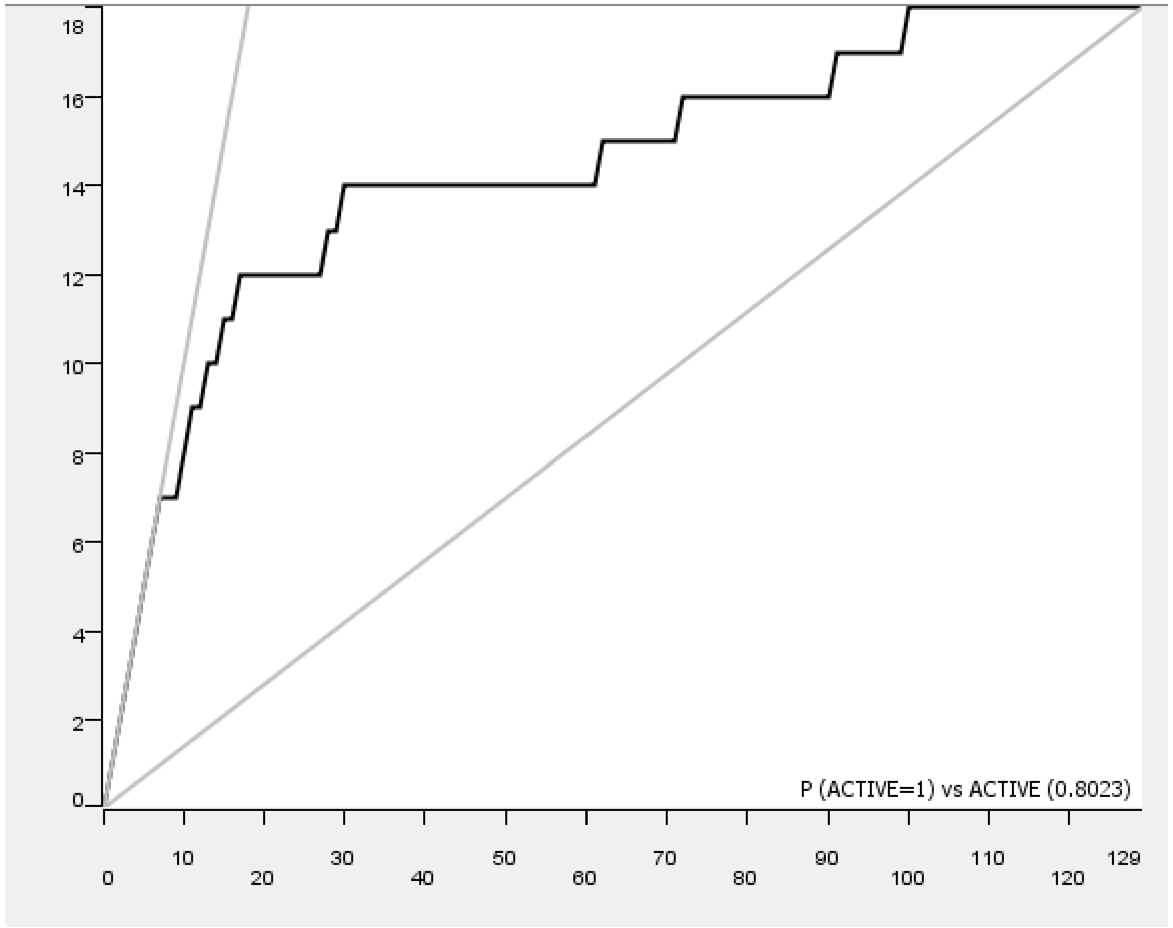

N=129

**Top 10% Mean IC50**	**8.4 nM**

	**Predicted Active**	**Predicted Inactive**
Active	12	6
Inactive	9	102

<15nM DefGood in beta-adren, 25% error; Random seed = 429

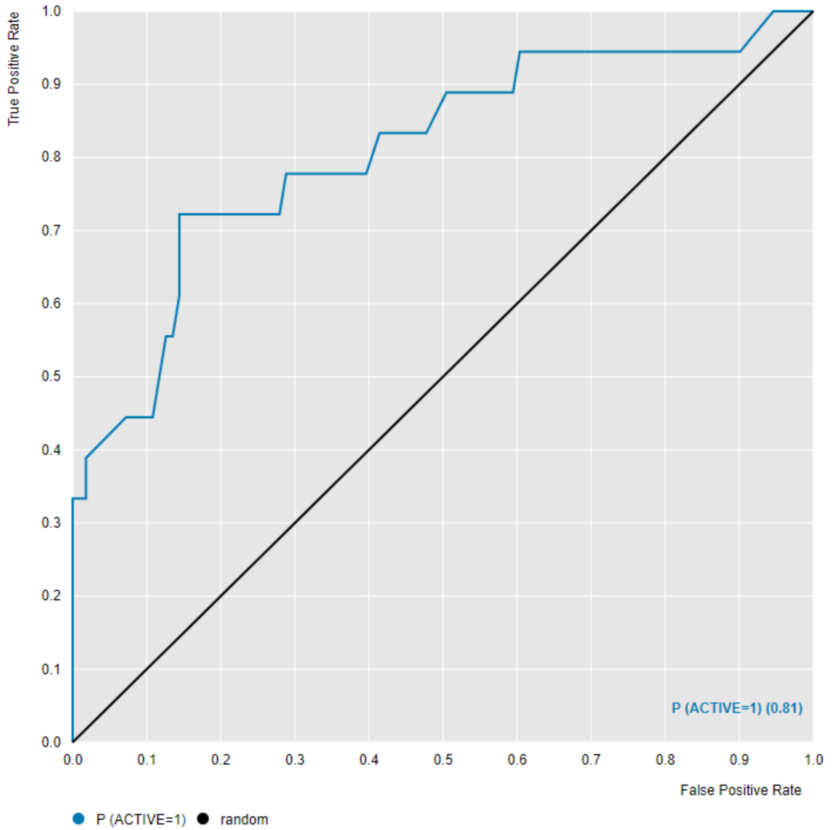

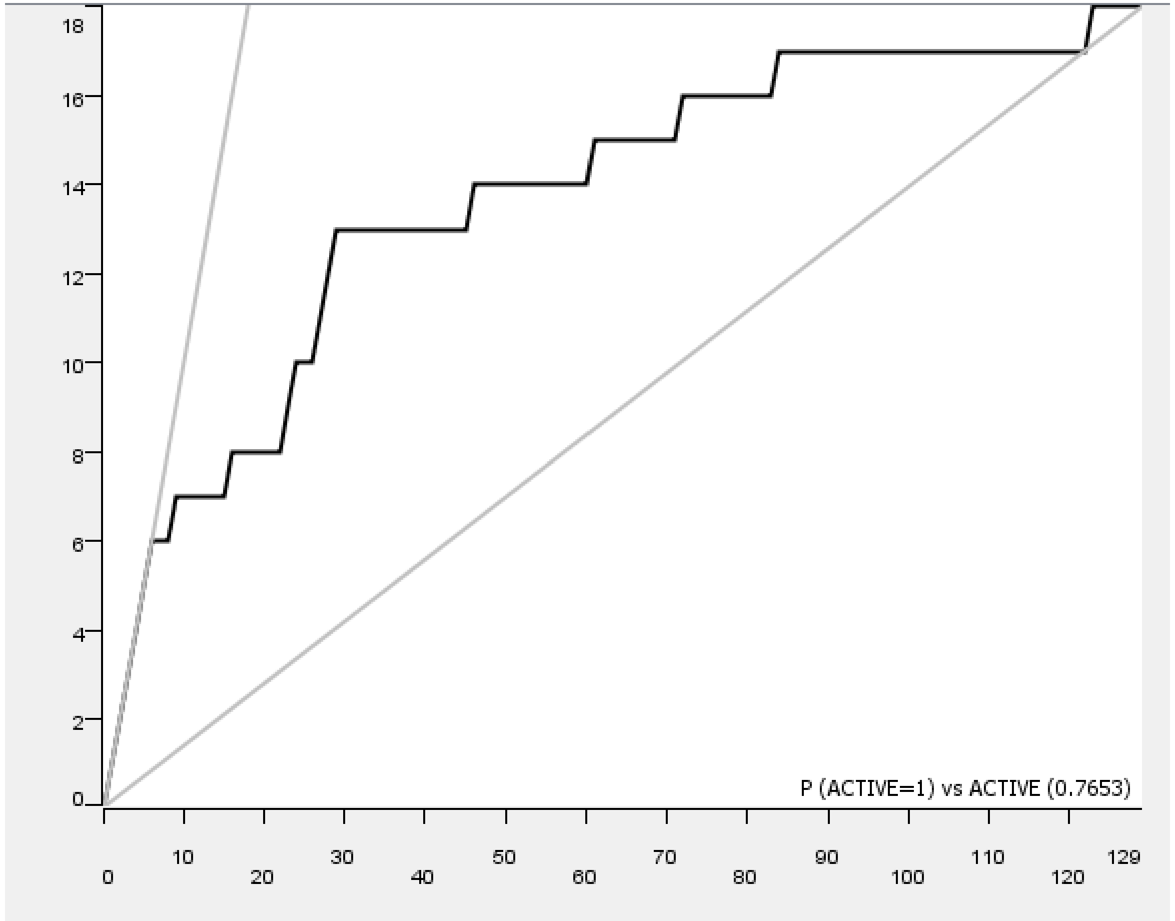

N=129

Top 10% Mean IC50	1,800 nM

	Predicted Active	Predicted Inactive
Active	13	5
Inactive	17	94

<15nM DefGood in beta-adren, 10% error; Random seed = 121783

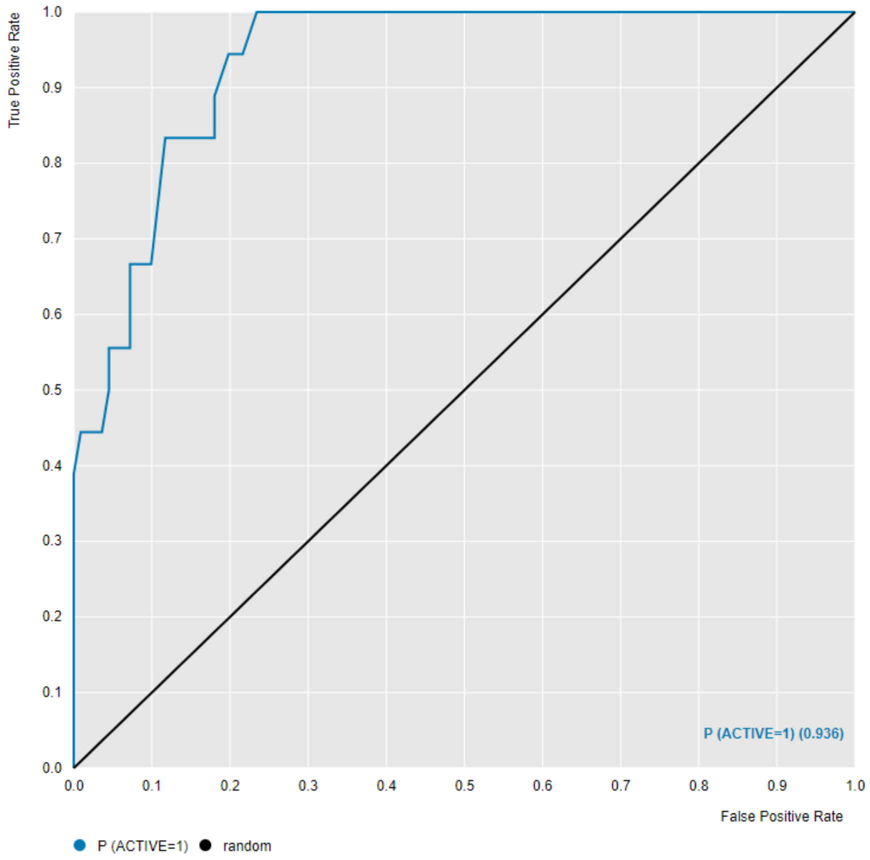

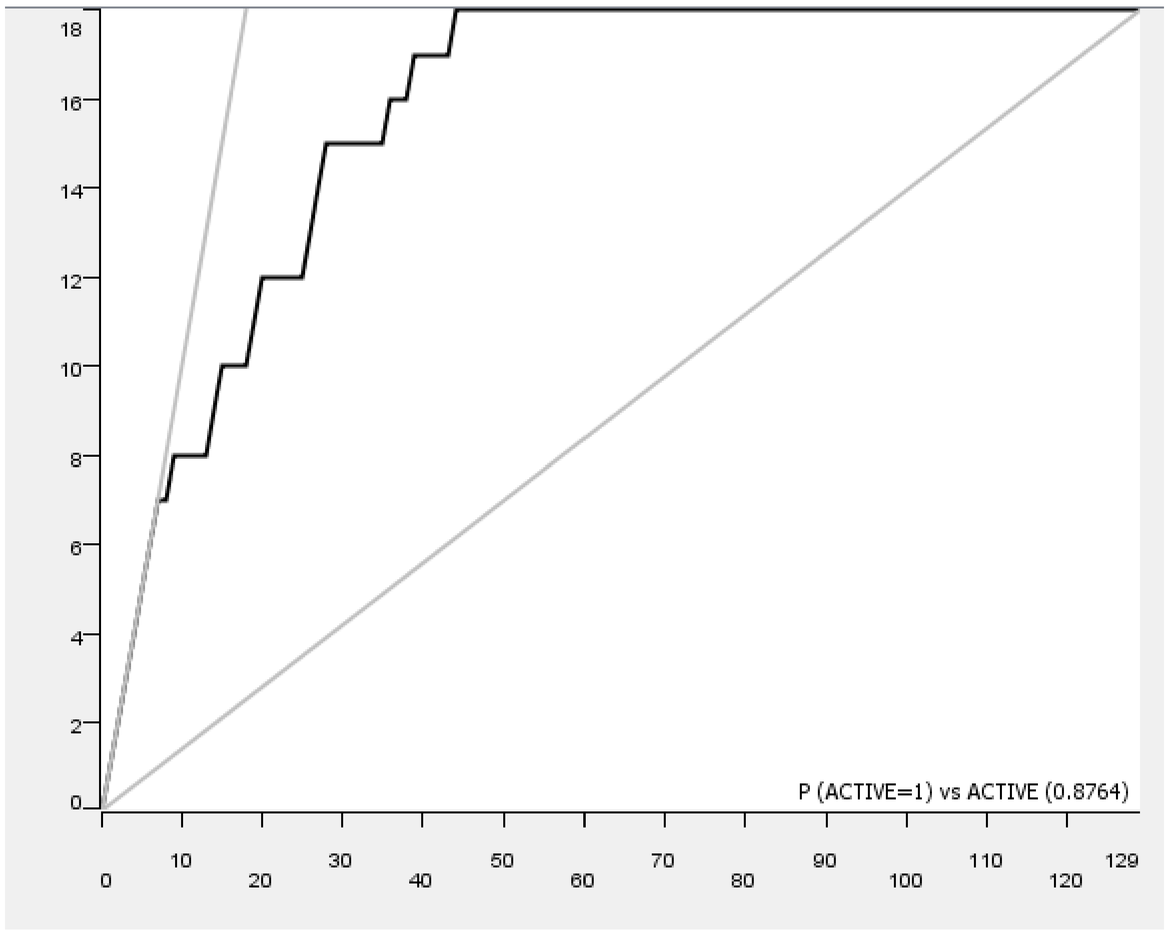

N=129

Top 10% Mean IC50	32.7 nM

	Predicted Active	Predicted Inactive
Active	10	8
Inactive	7	104

<15nM DefGood in beta-adren, 20% error; Random seed = 121783

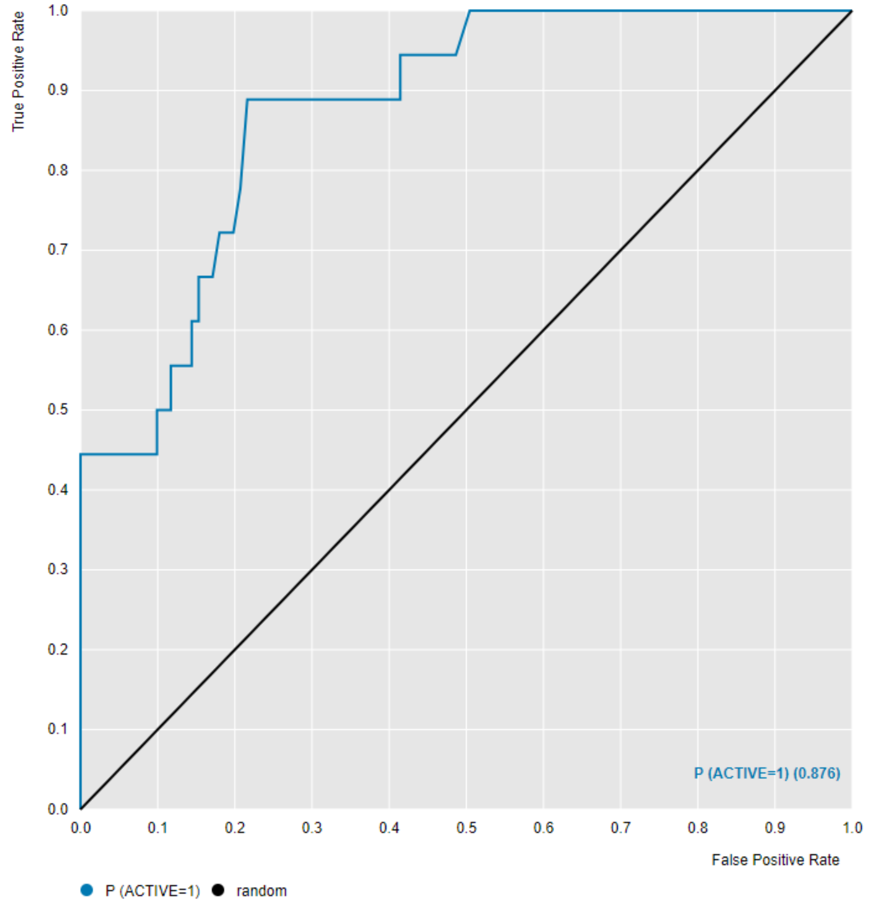

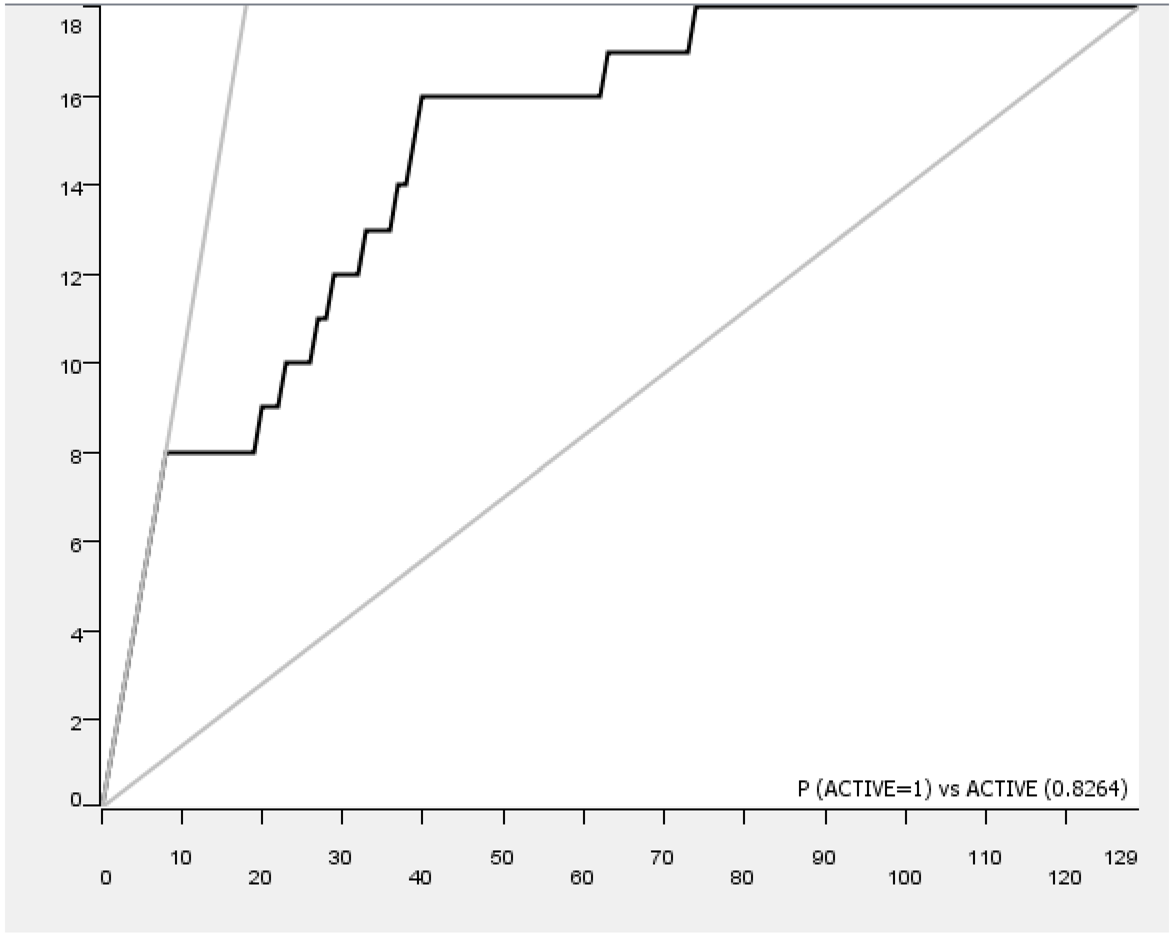

N=129

Top 10% Mean IC50	91 nM

	Predicted Active	Predicted Inactive
Active	10	8
Inactive	13	98

<15nM DefGood in beta-adren, 30% error; Random seed = 121783

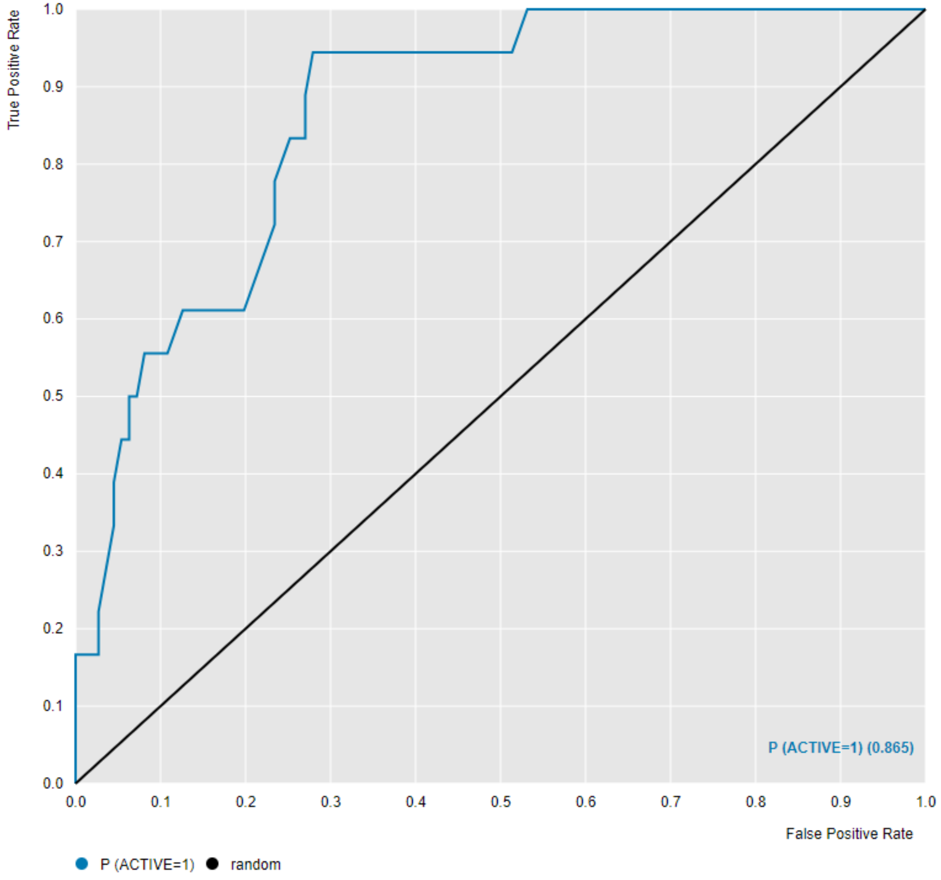

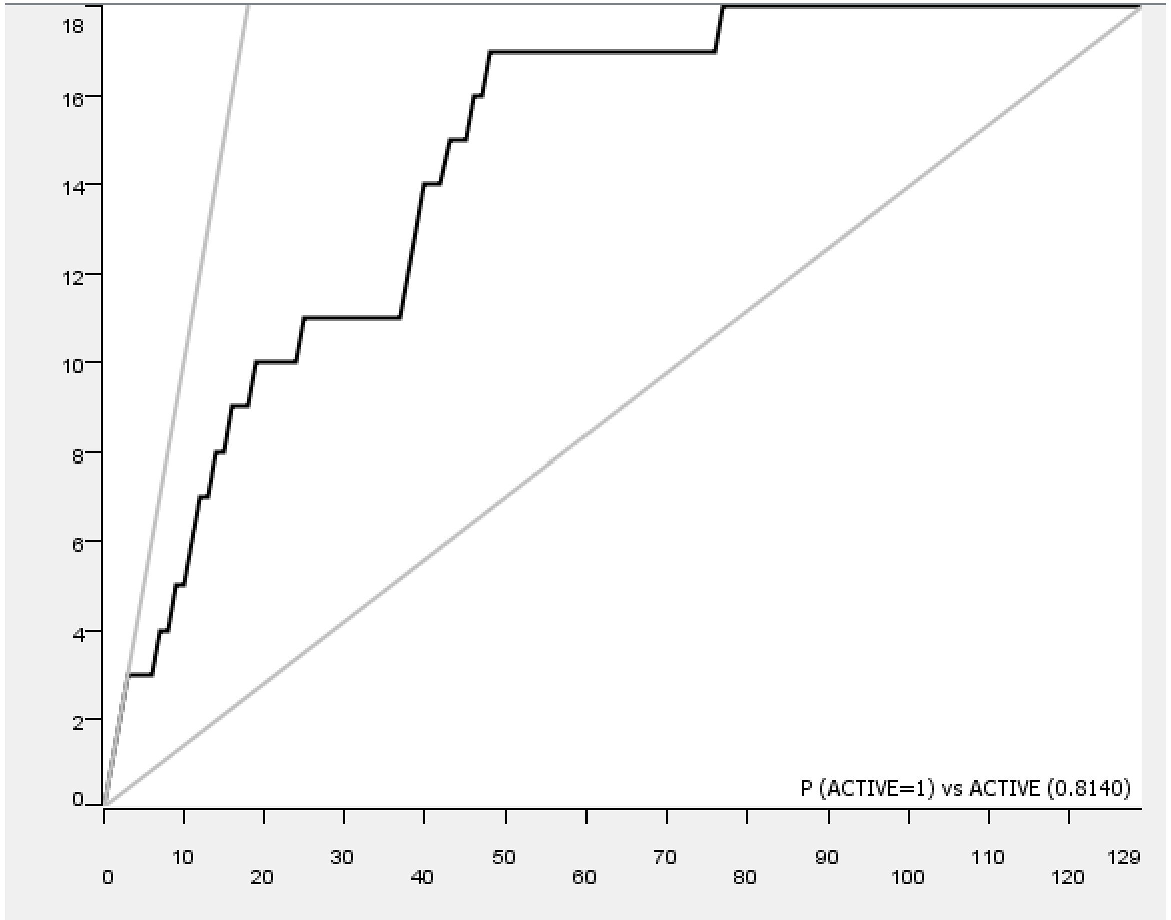

N=129

Top 10% Mean IC50	63 nM

	Predicted Active	Predicted Inactive
Active	11	7
Inactive	15	96

<15nM DefGood in beta-adren, 35% error; Random seed = 121783

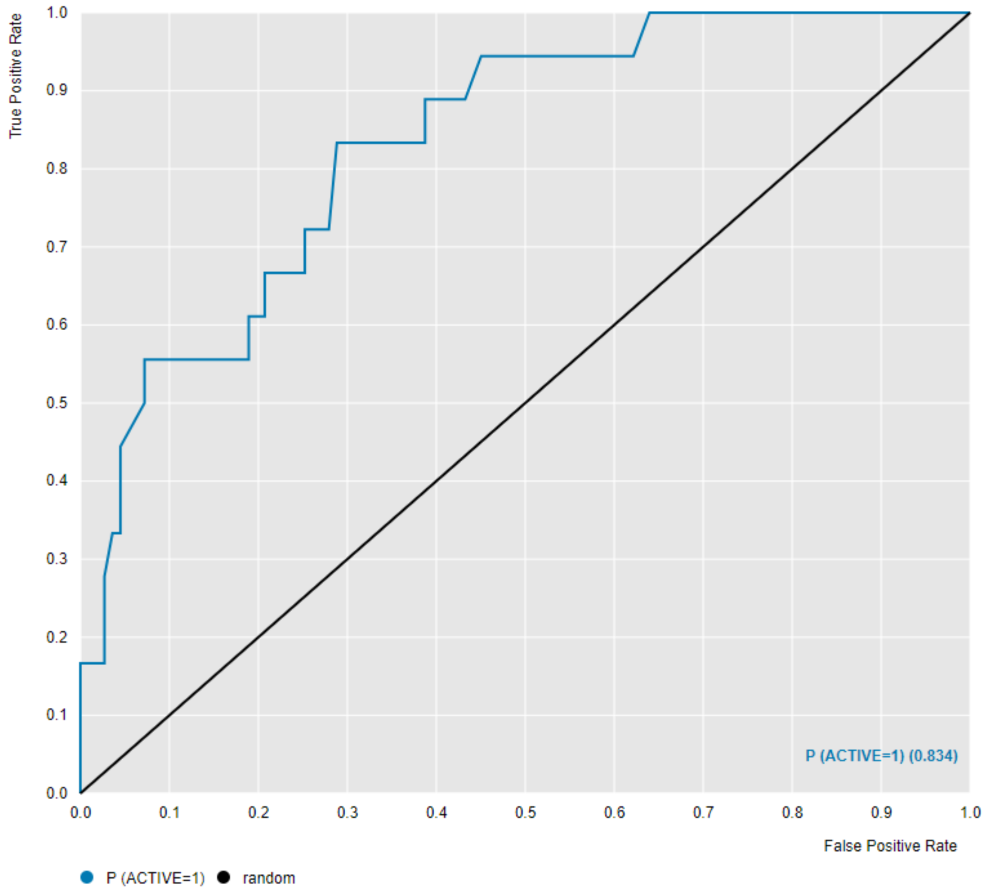

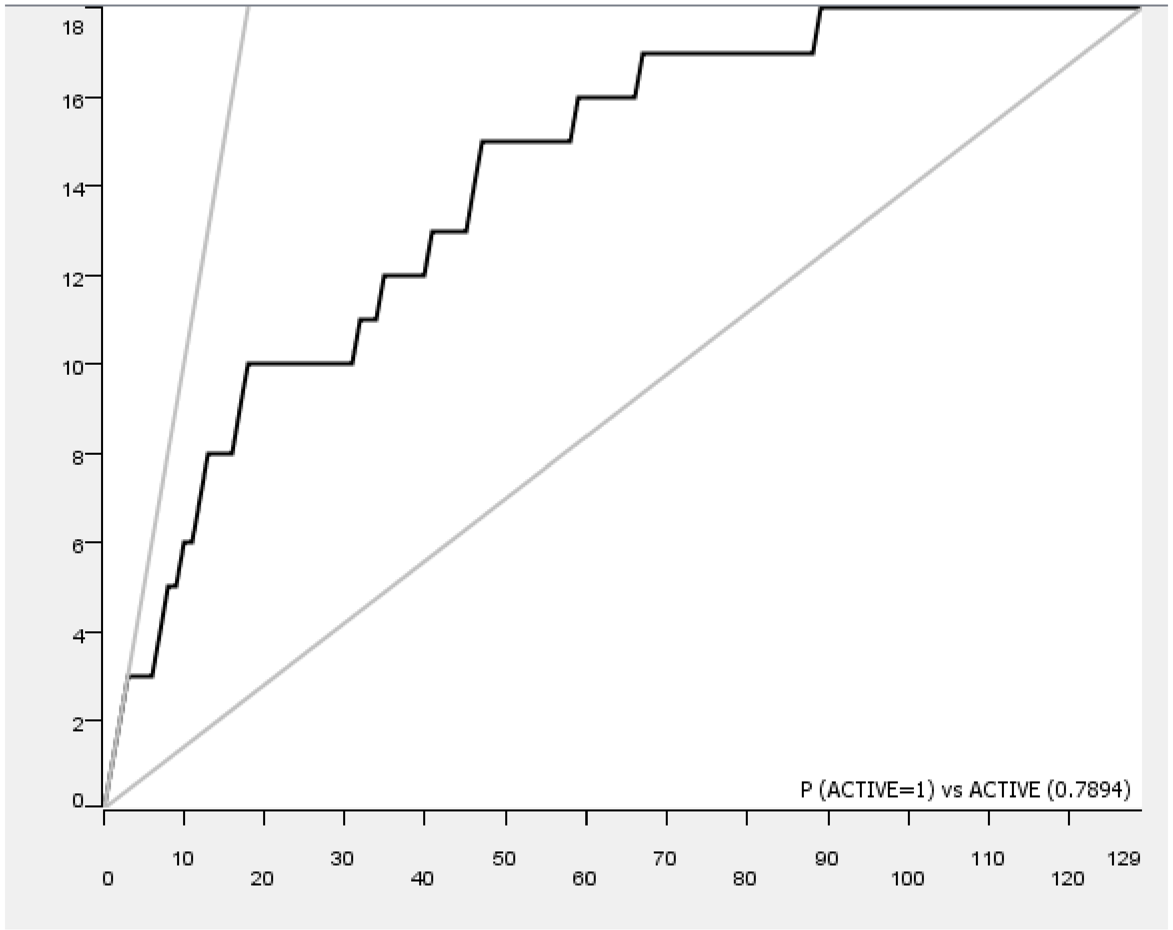

N=129

Top 10% Mean IC50	110 nM

	Predicted Active	Predicted Inactive
Active	12	6
Inactive	26	85

<15nM DefGood in beta-adren, 40% error; Random seed = 121783

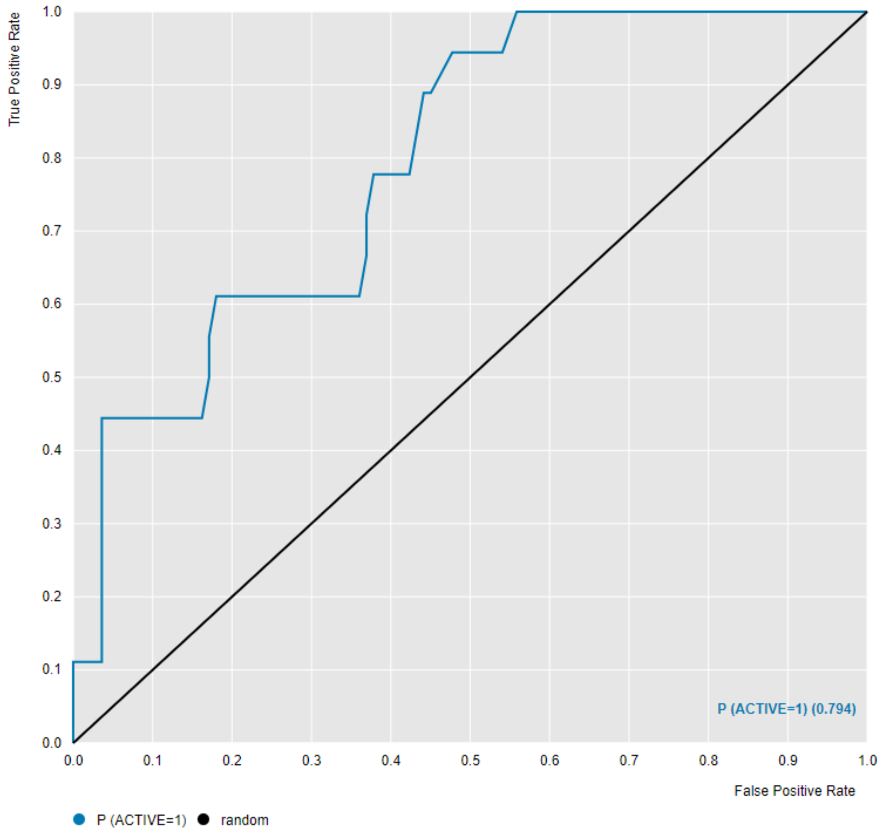

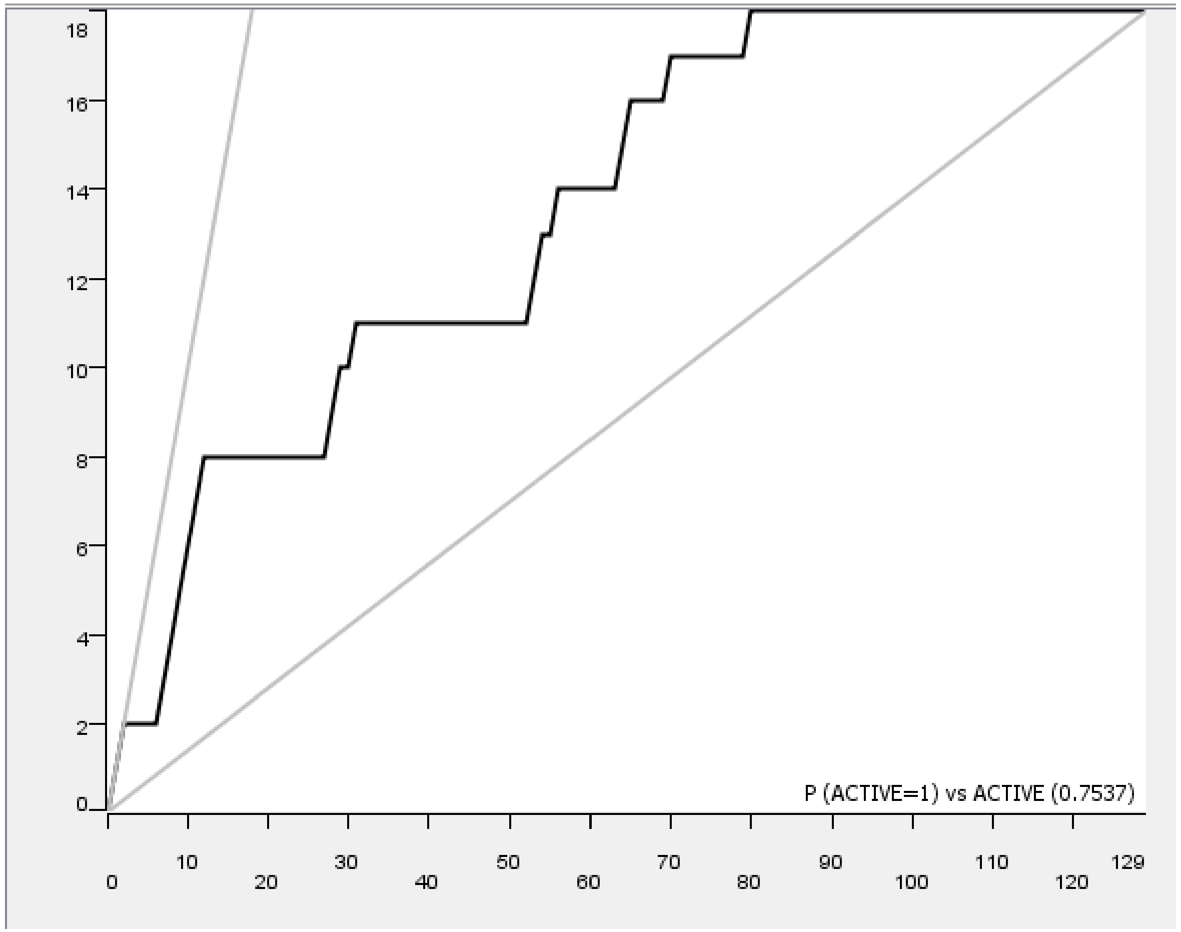

N=129

**Top 10% Mean IC50**	**58.2 nM**

	**Predicted Active**	**Predicted Inactive**
Active	11	7
Inactive	26	85

<15nM DefGood in beta-adren, 45% error; Random seed = 121783

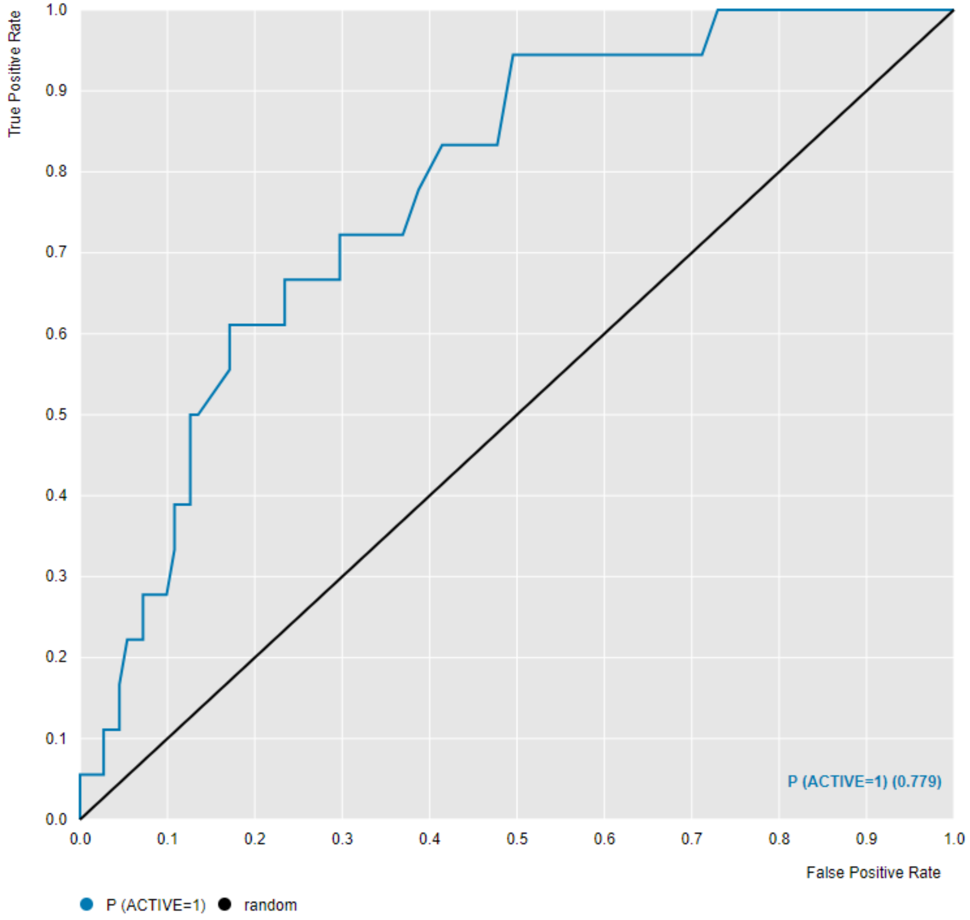

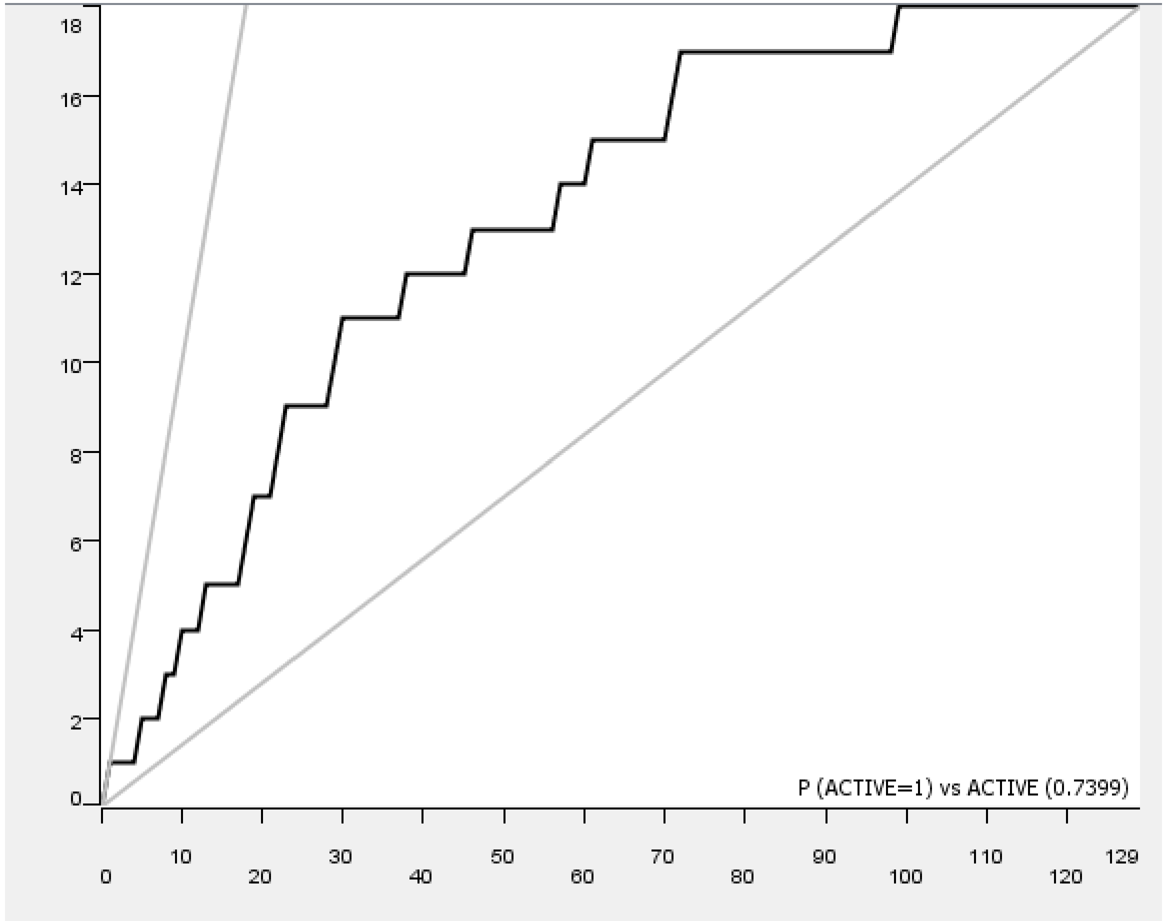

N=129

**Top 10% Mean IC50**	**771 nM**

	Predicted Active	Predicted Inactive
Active	12	6
Inactive	33	78

Conclusion - RF

- A Random Forrest could be generated for β2 adrenergic receptor with a decision value of <15 nM as defined as active. The predictivity of the algorithm could be preserved in three random train/test cycles as error was introduced in the training set. The point of failure was 35%, 25% and 45% error.

PNN- <15nM DefGood in beta-adren, 5% error ; Random seed = 1515533876005

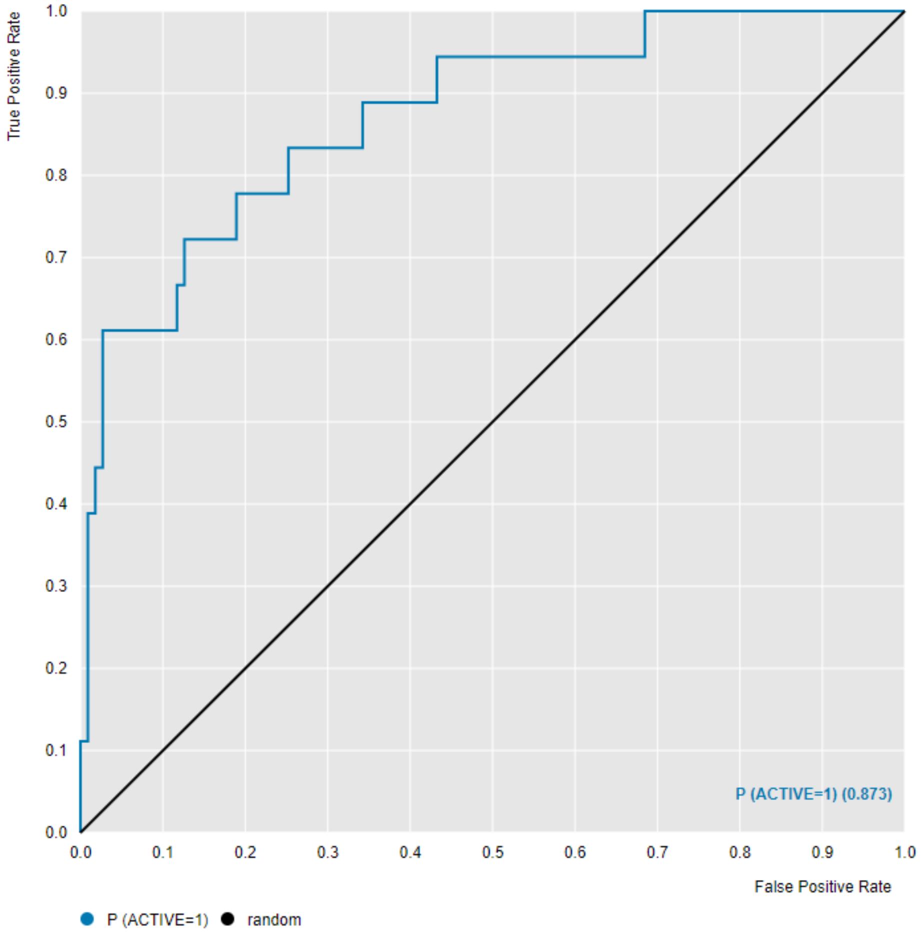

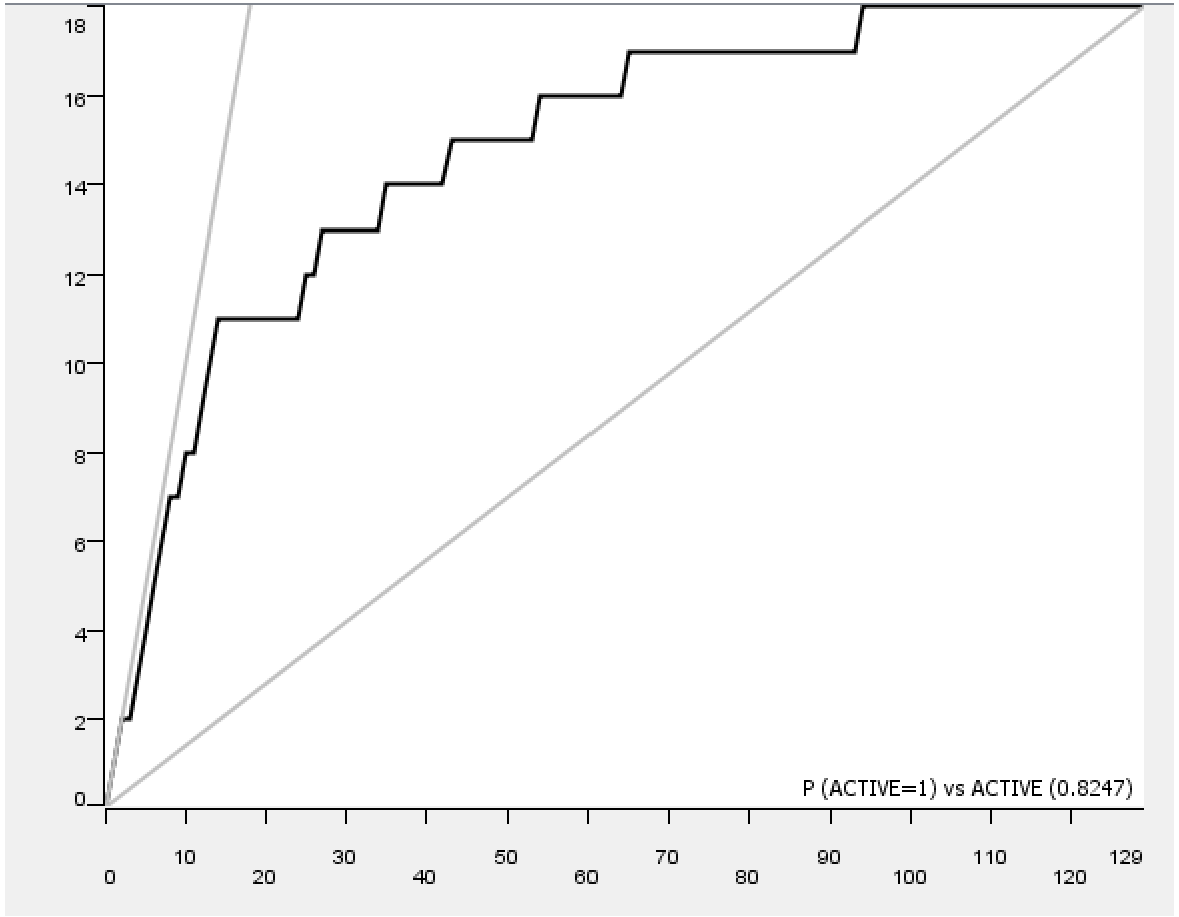

N=129

Top 10% Mean IC50	26.5 nM

	Predicted Active	Predicted Inactive
Active	10	8
Inactive	3	108

PNN- <15nM DefGood in beta-adren, 10% error ; Random seed = 1515533876005

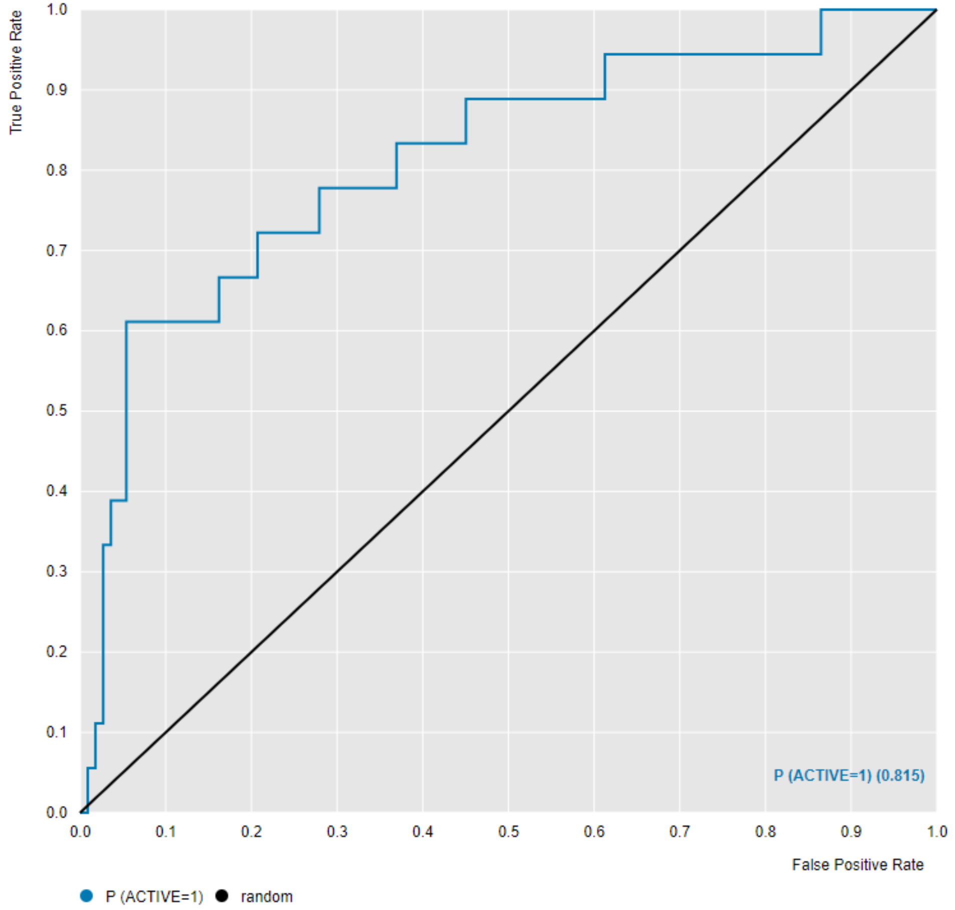

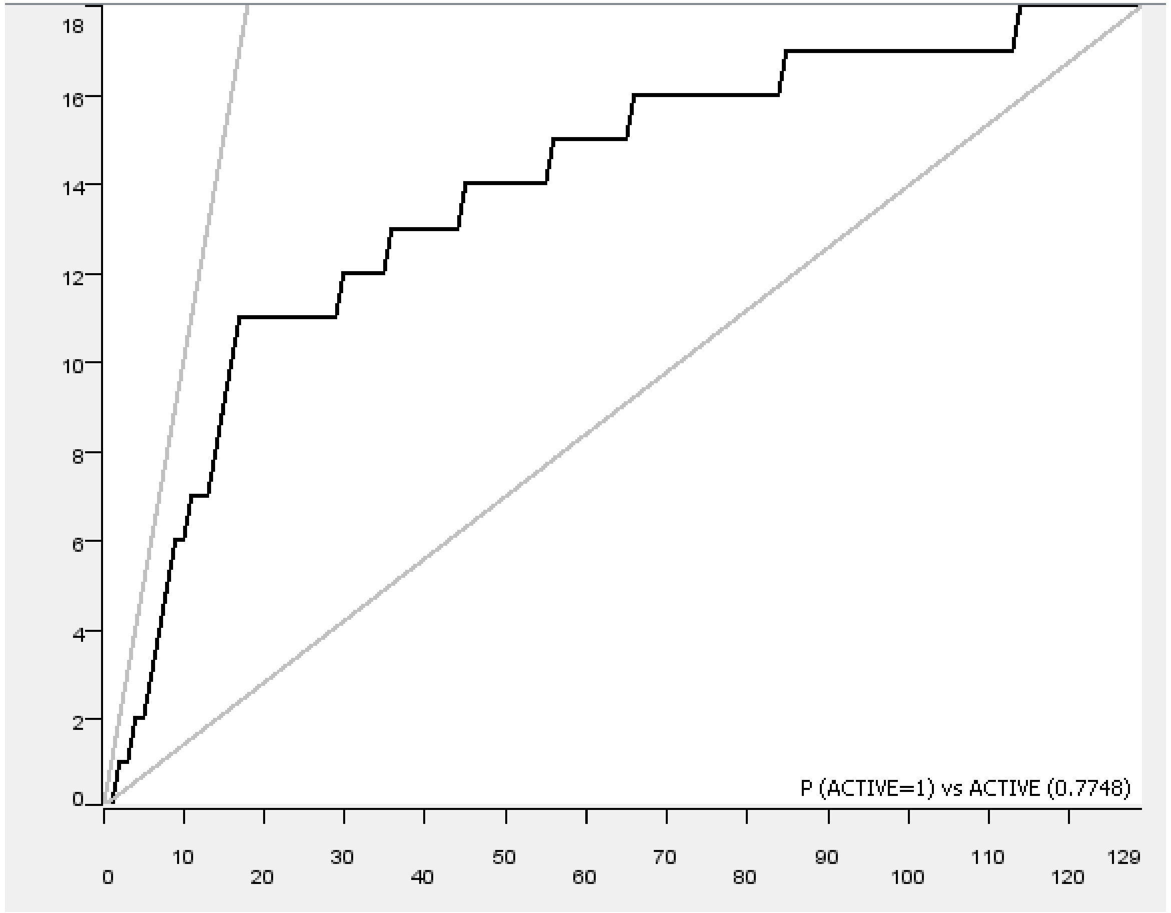

N=129

Top 10% Mean IC50	1,100 nM

	Predicted Active	Predicted Inactive
Active	10	8
Inactive	6	105

PNN- <15nM DefGood in beta-adren, 5% error ; Random seed = 429

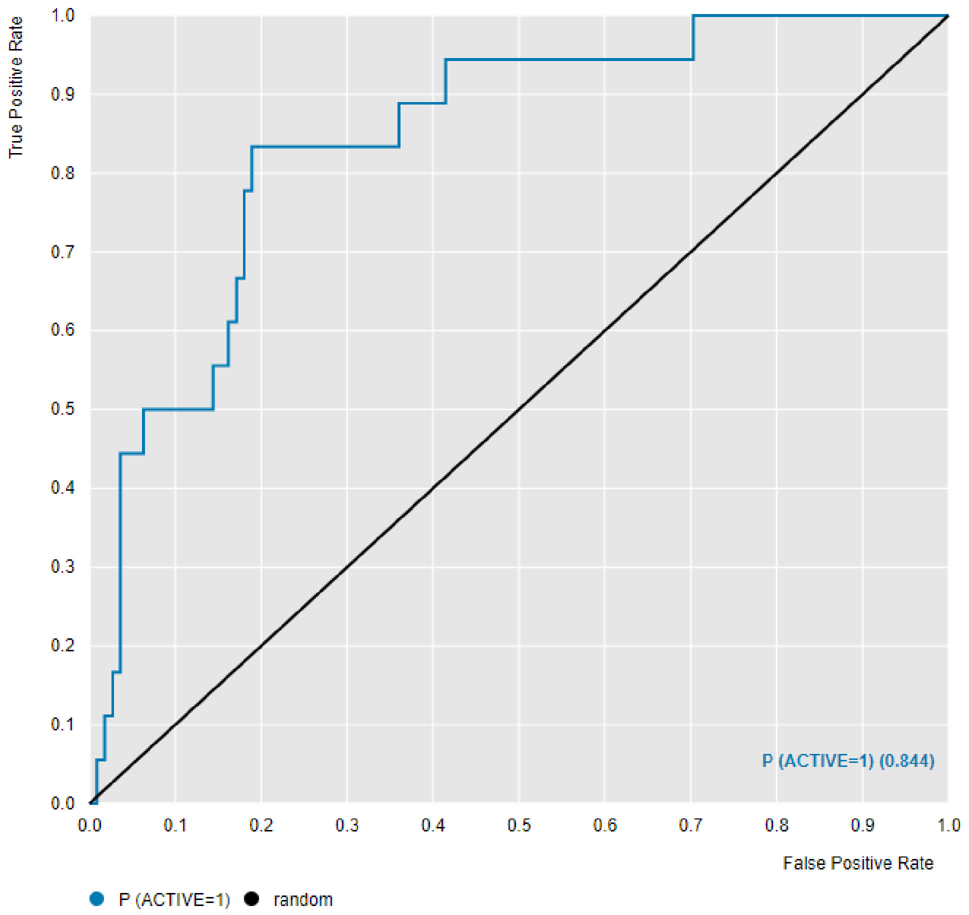

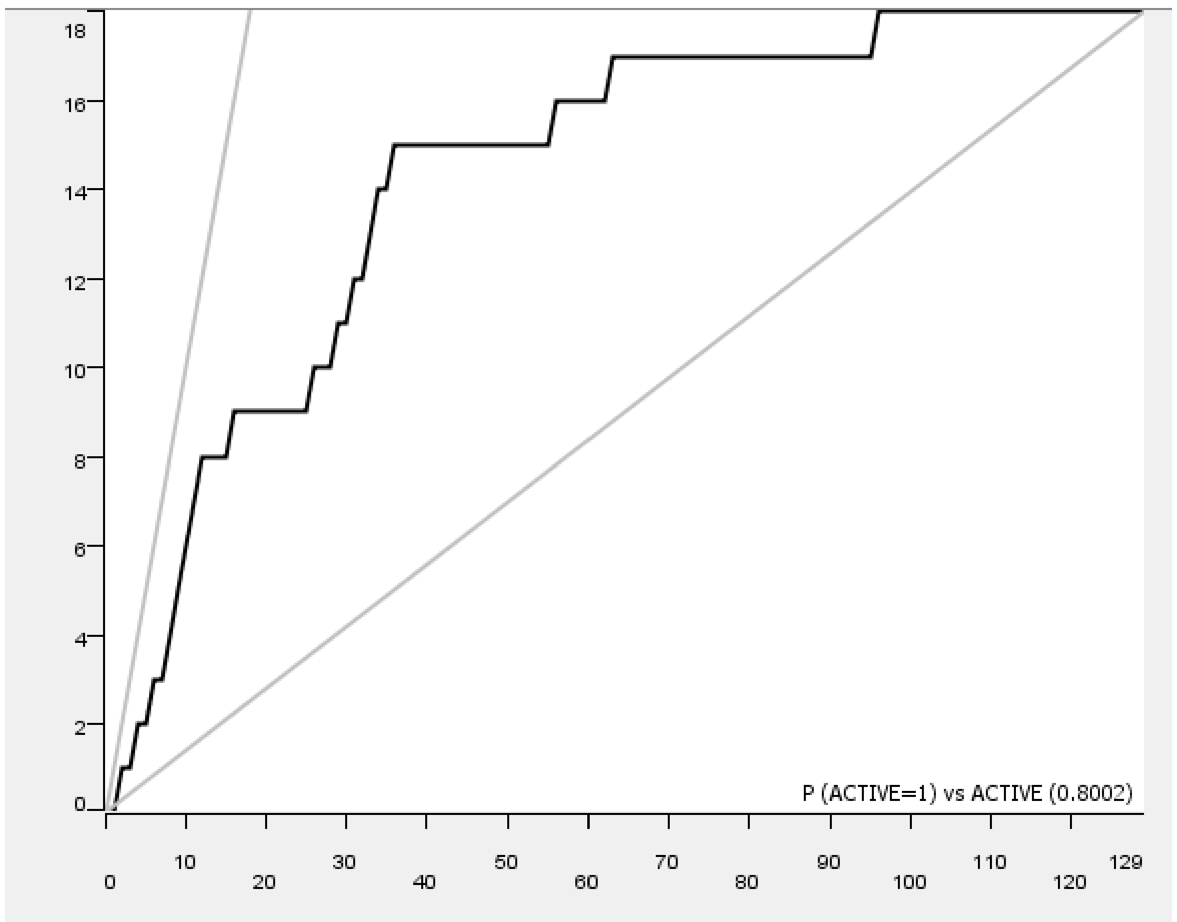

N=129

**Top 10% Mean IC50**	**21.7 nM**

	Predicted Active	Predicted Inactive
Active	8	10
Inactive	7	104

PNN- <15nM DefGood in beta-adren, 10% error ; Random seed = 429

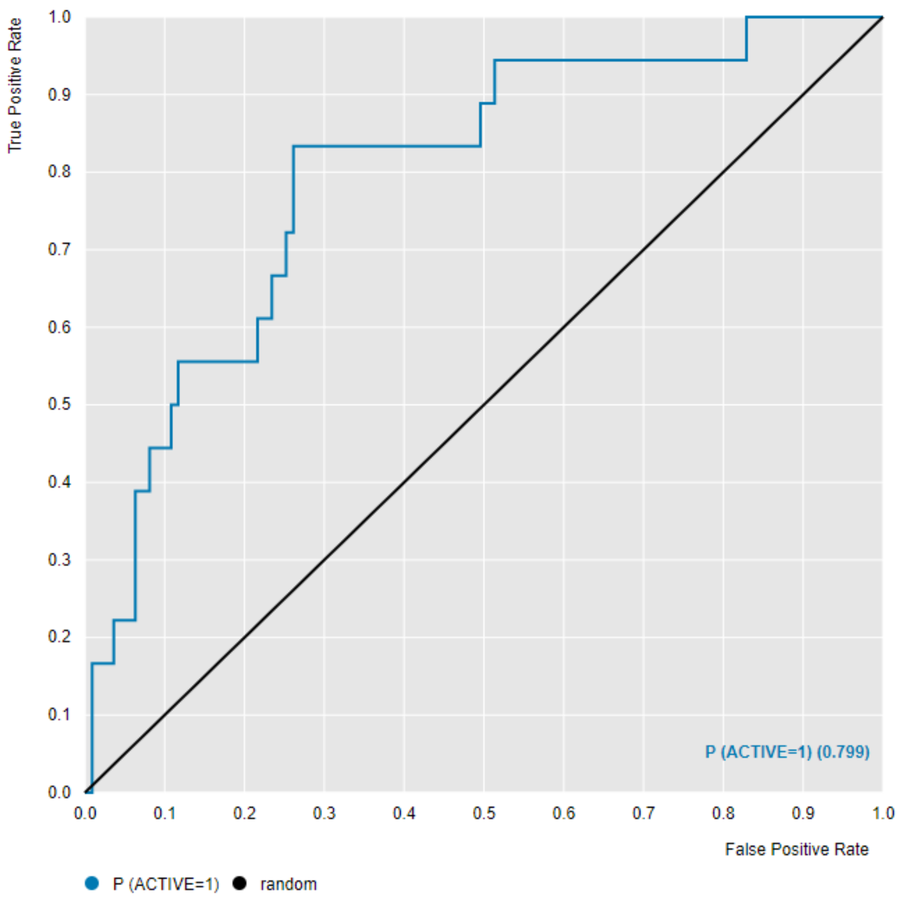

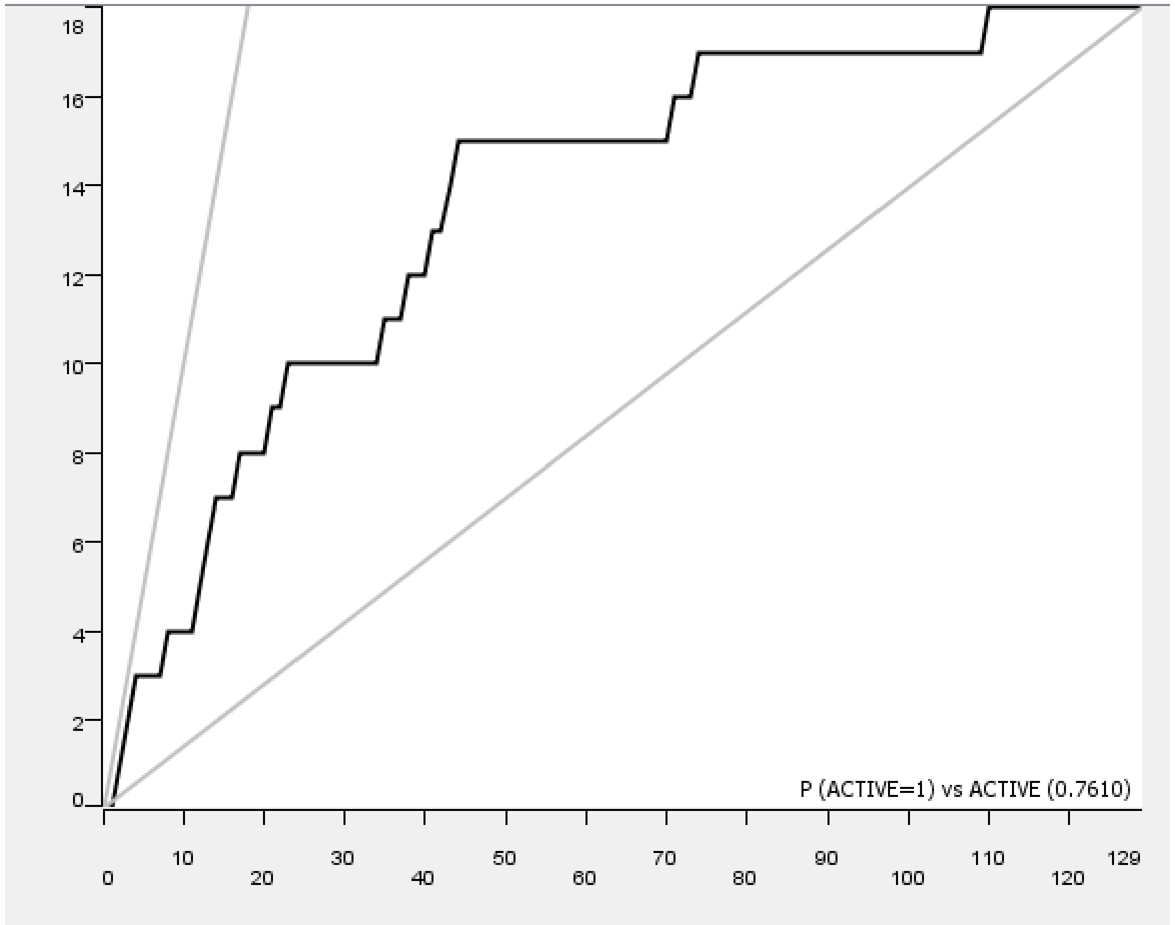

N=129

Top 10% Mean IC50	150 nM

	Predicted Active	Predicted Inactive
Active	8	10
Inactive	11	100

PNN- <15nM DefGood in beta-adren, 15% error; Random seed = 429

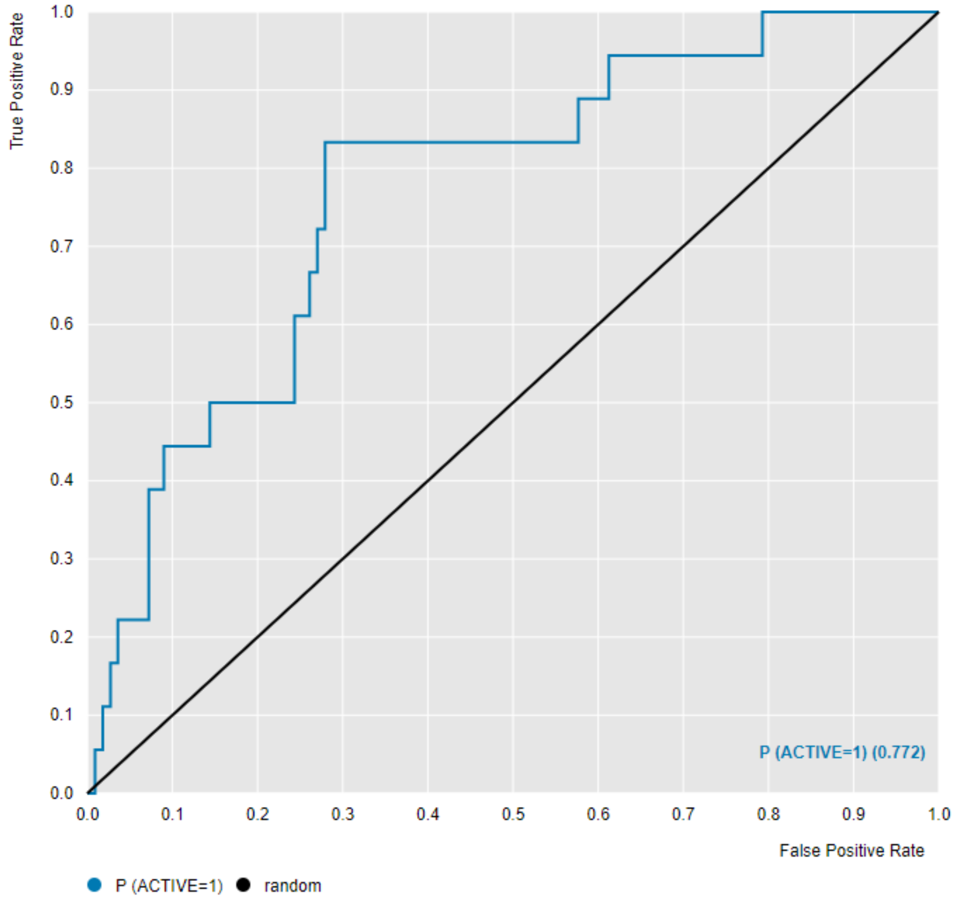

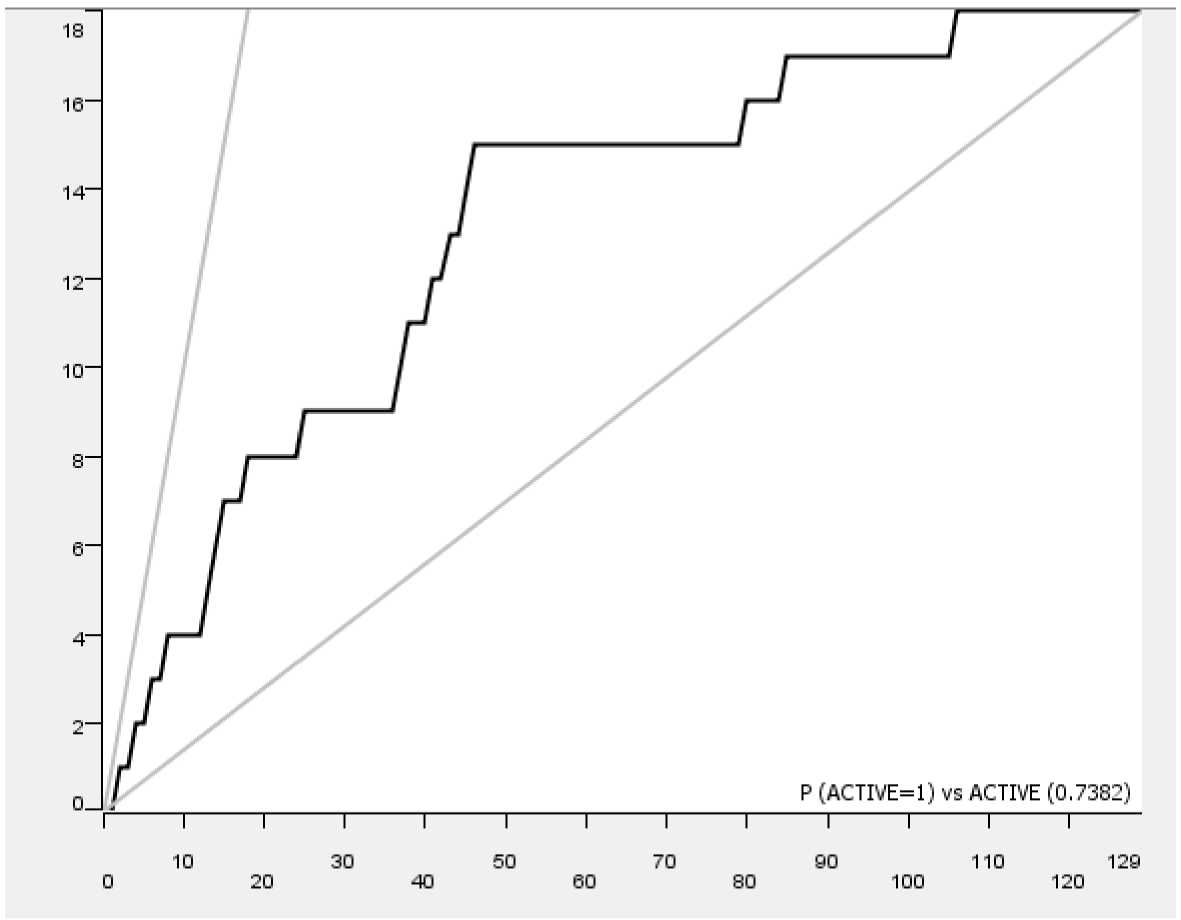

N=129

Top 10% Mean IC50	184 nM

	Predicted Active	Predicted Inactive
Active	8	10
Inactive	12	99

PNN-<15nM DefGood in beta-adren, 20% error ; Random seed = 429

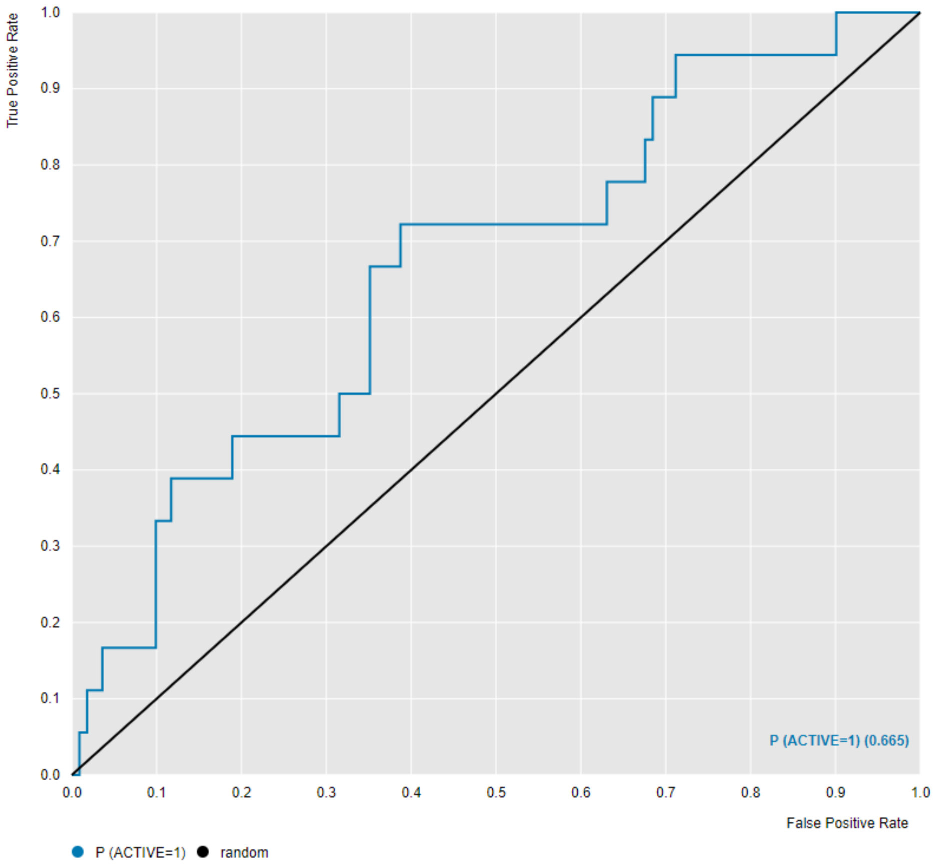

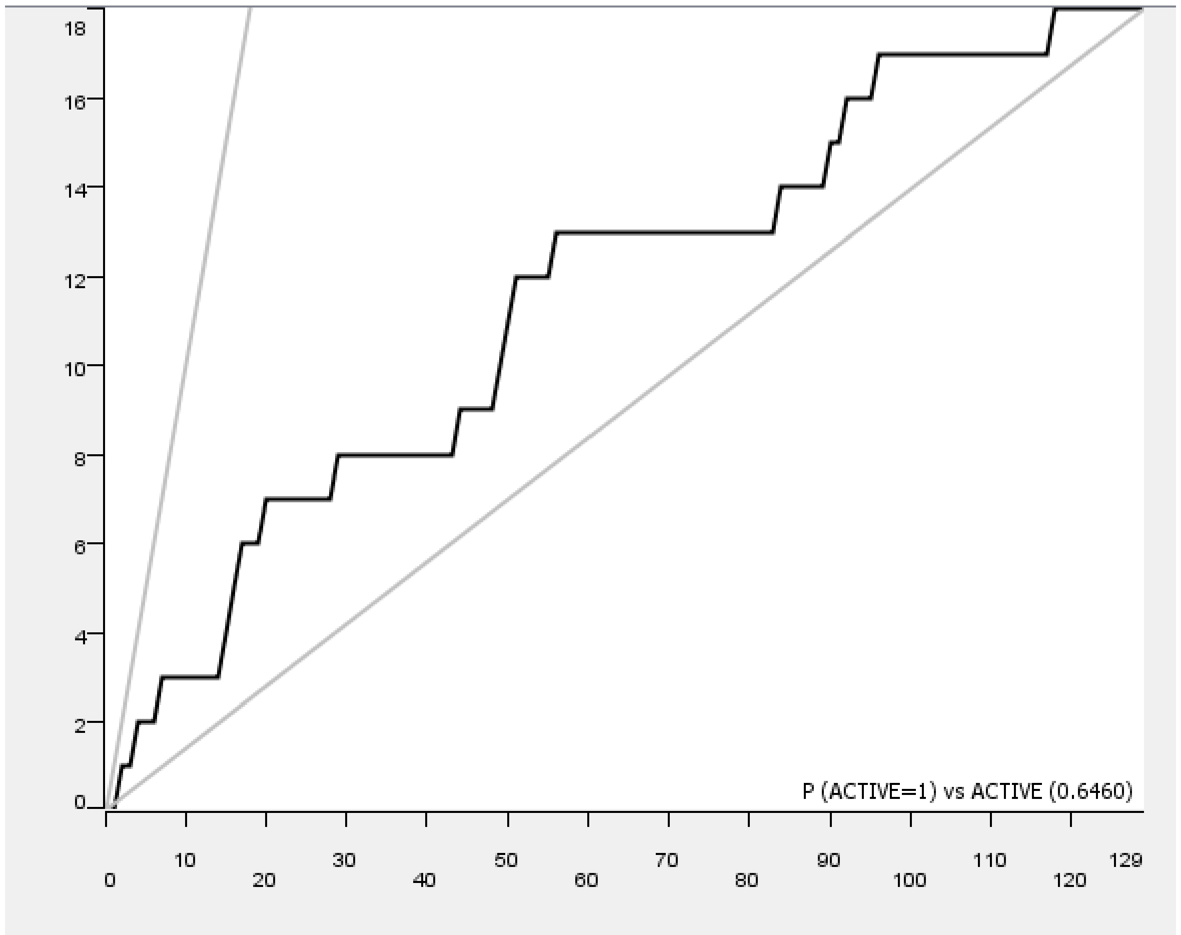

N=129

Top 10% Mean IC50	130,000 nM

	Predicted Active	Predicted Inactive
Active	7	11
Inactive	16	95

PNN - <15nM DefGood in beta-adren, 5% error; Random seed = 121783

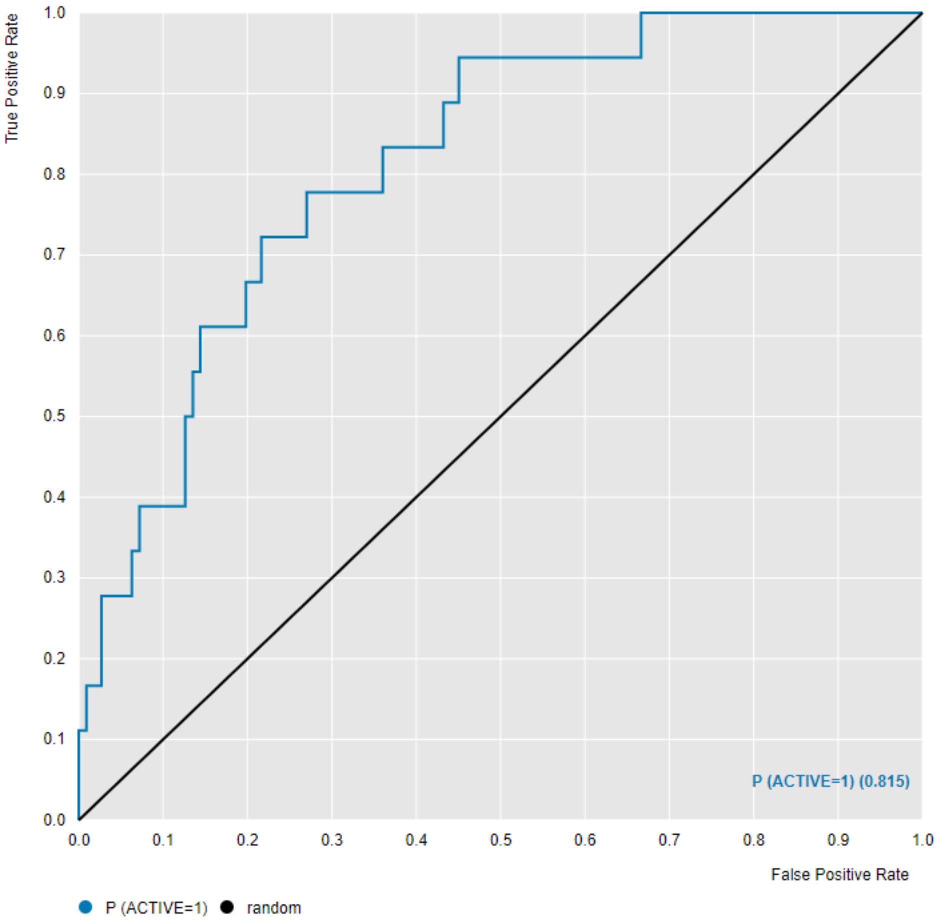

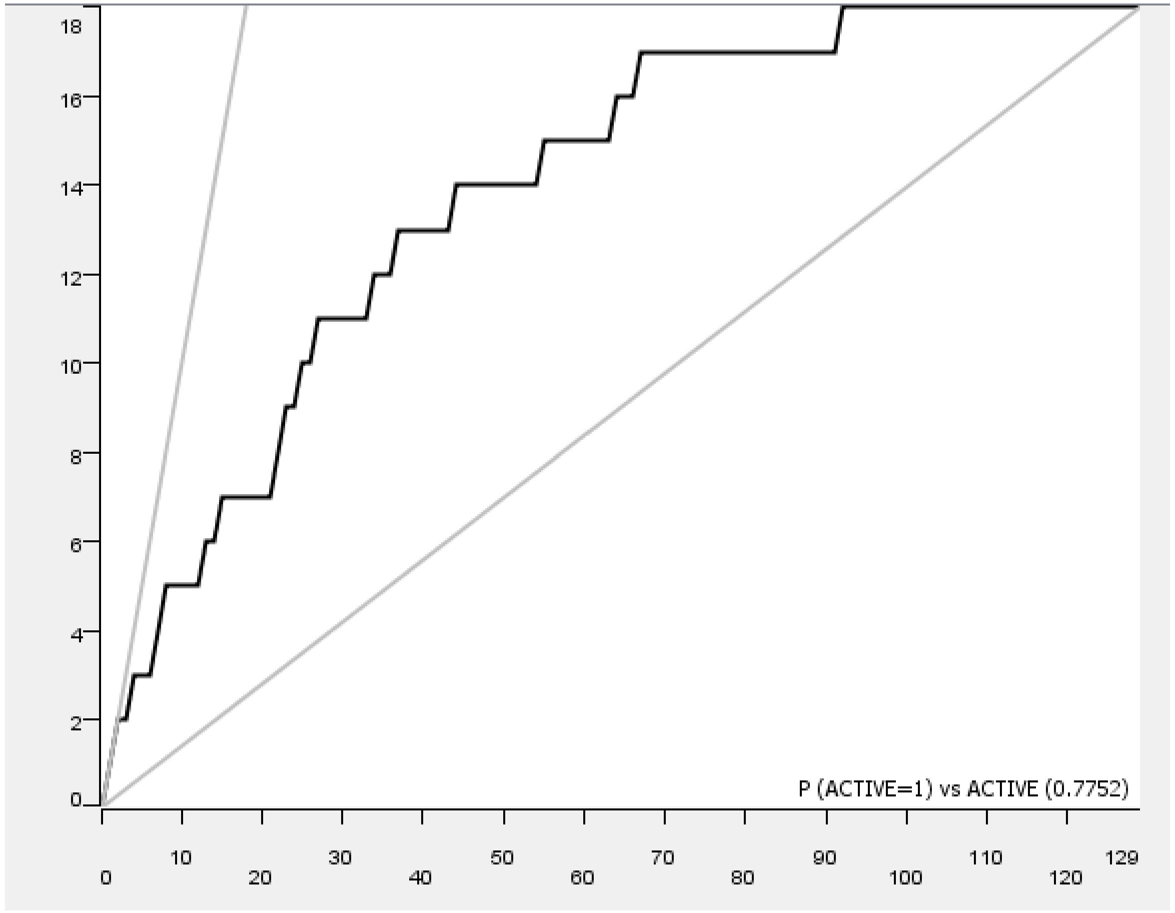

N=129

Top 10% Mean IC50	1,900 nM

	Predicted Active	Predicted Inactive
Active	7	11
Inactive	11	100

PNN - <15nM DefGood in beta-adren, 5% error; Random seed = 121783 (reparameterized Theta minus = 0.55; theta plus = 0.95)

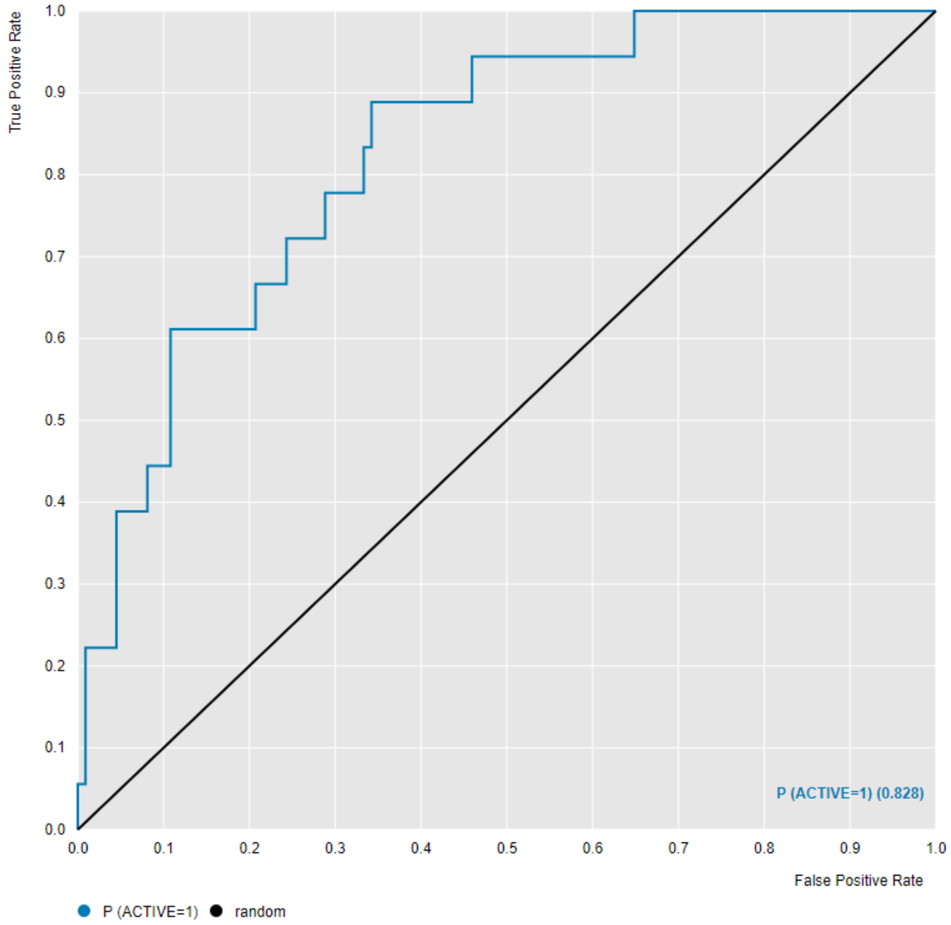

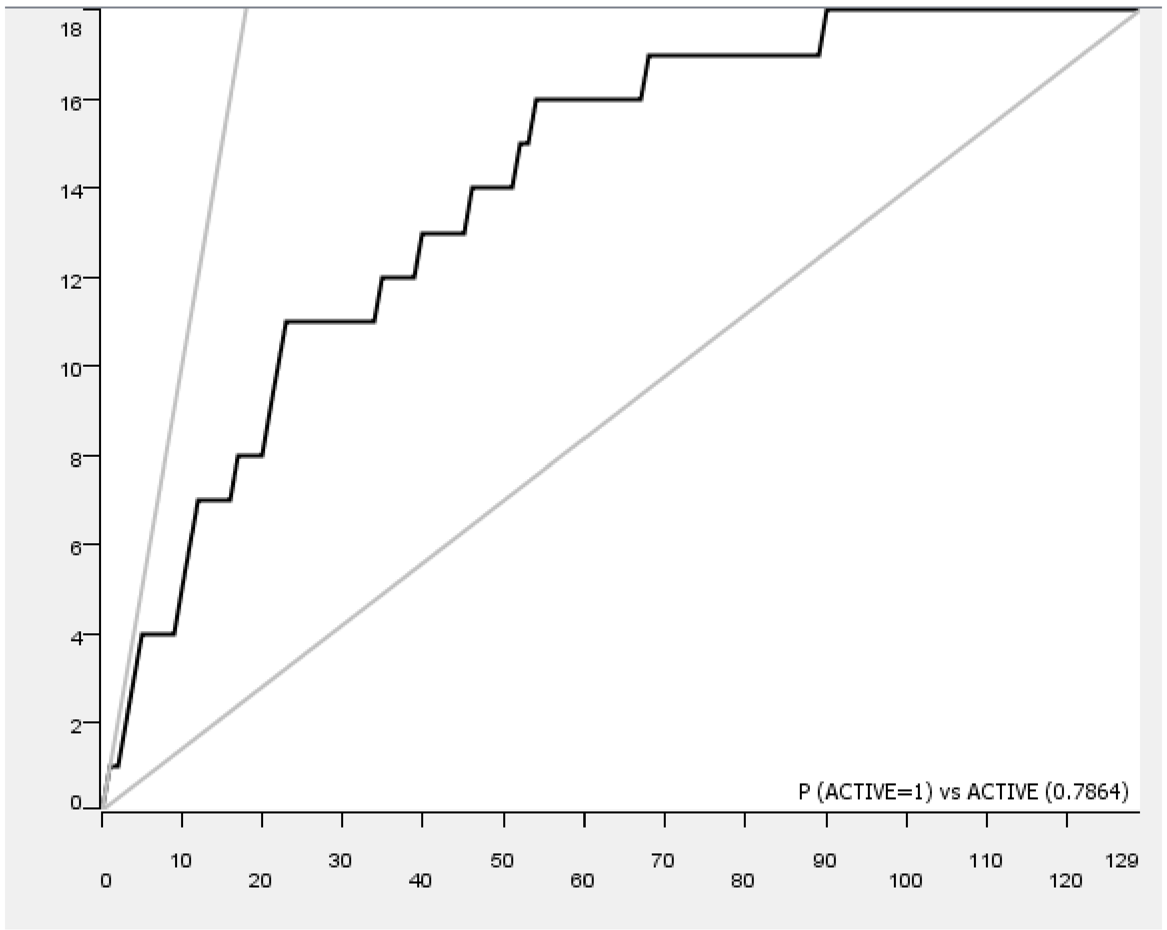

N=129

Top 10% Mean IC50	1,100 nM

	Predicted Active	Predicted Inactive
Active	7	11
Inactive	6	105

PNN - <15nM DefGood in beta-adren, 10% error; Random seed = 121783

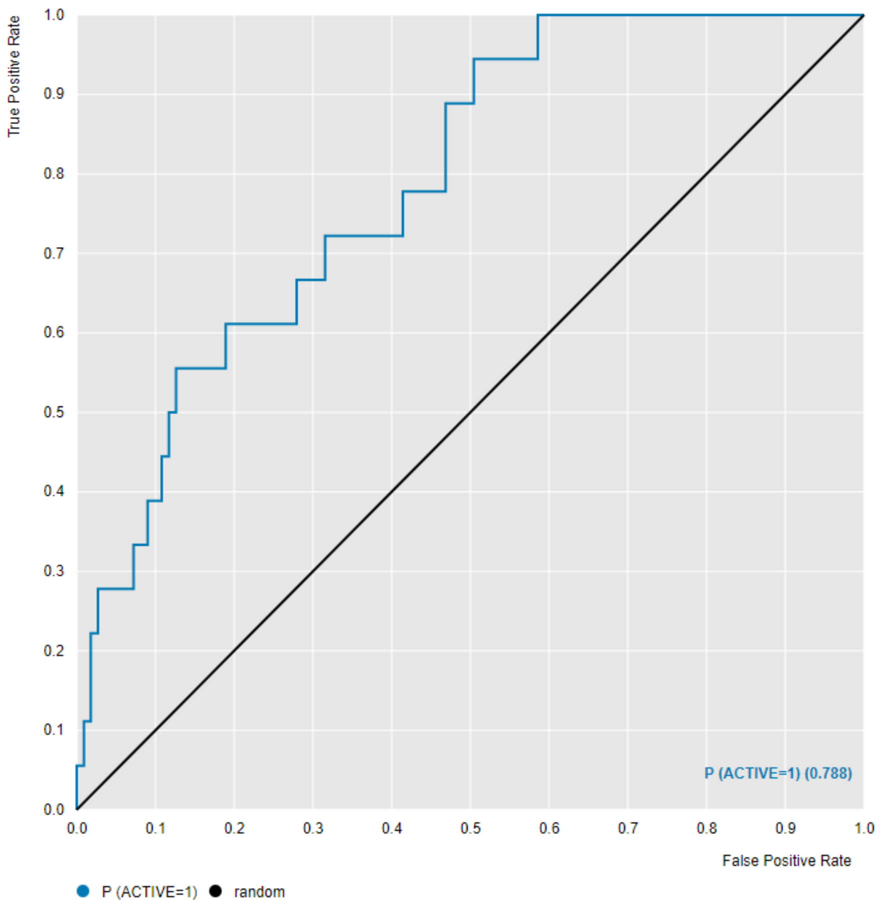

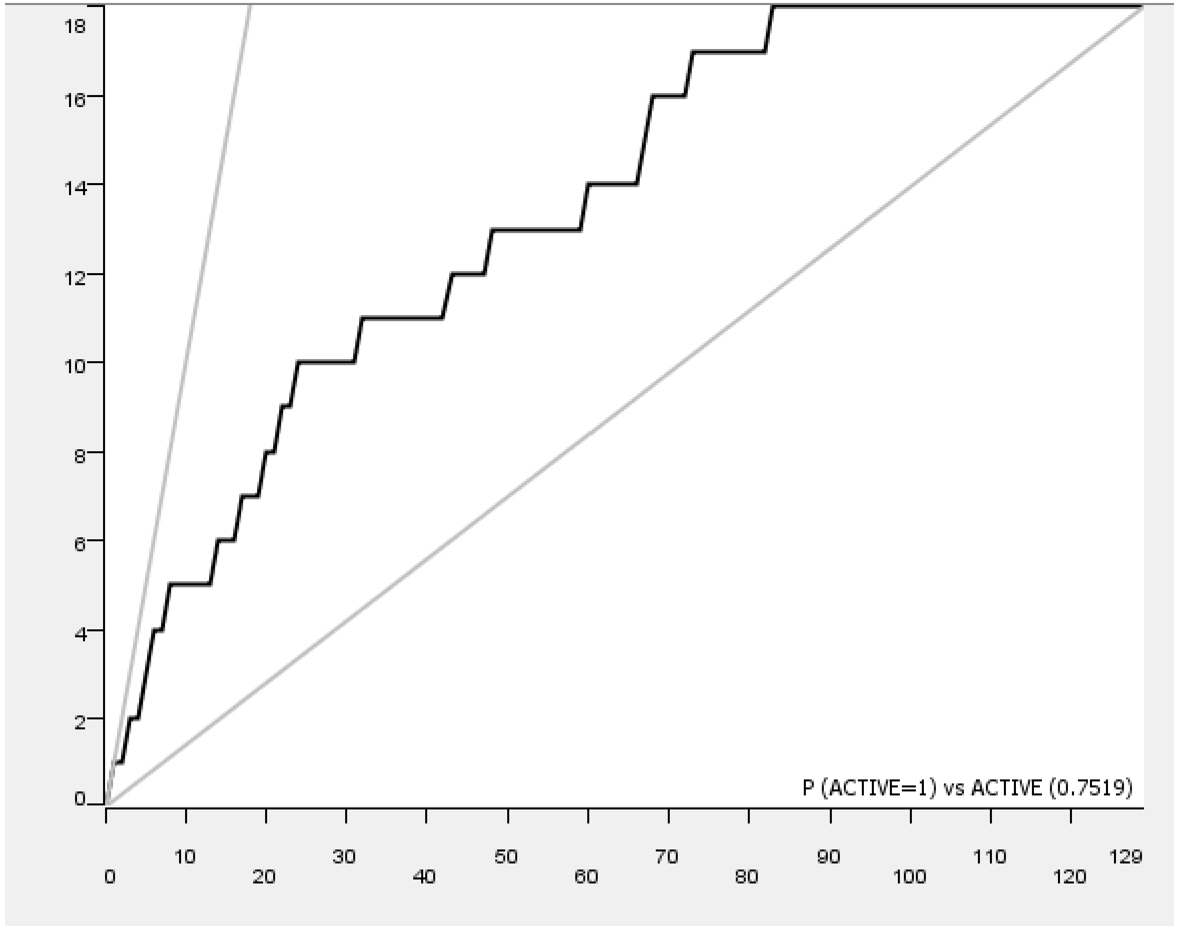

N=129

**Top 10% Mean IC50**	**1,200 nM**

	**Predicted Active**	**Predicted Inactive**
Active	7	11
Inactive	10	101

Conclusion - PNN

- Across three random seeds, the % error that lead to a significant enrichment failure and/or top 10% IC50 increase in the retrospective test was 10%, 20% and 5% for each unique random seed.

c-Abl

C-Abl NBN Design

- Rules - $RELATION$ = "=" OR $IC50$ > 999 OR $IC50$ < 10 => TRUE
- Sort ascending and remove duplicates
- DefGood scanned between 5 and 25 nM
- 80-20 split for both actives and inactives
- NBN – ECFP4 used Classifier where Prediction was Active = True
- Resulted in 1439 compounds after cleaning

<5 nM DefGood in c-Abl

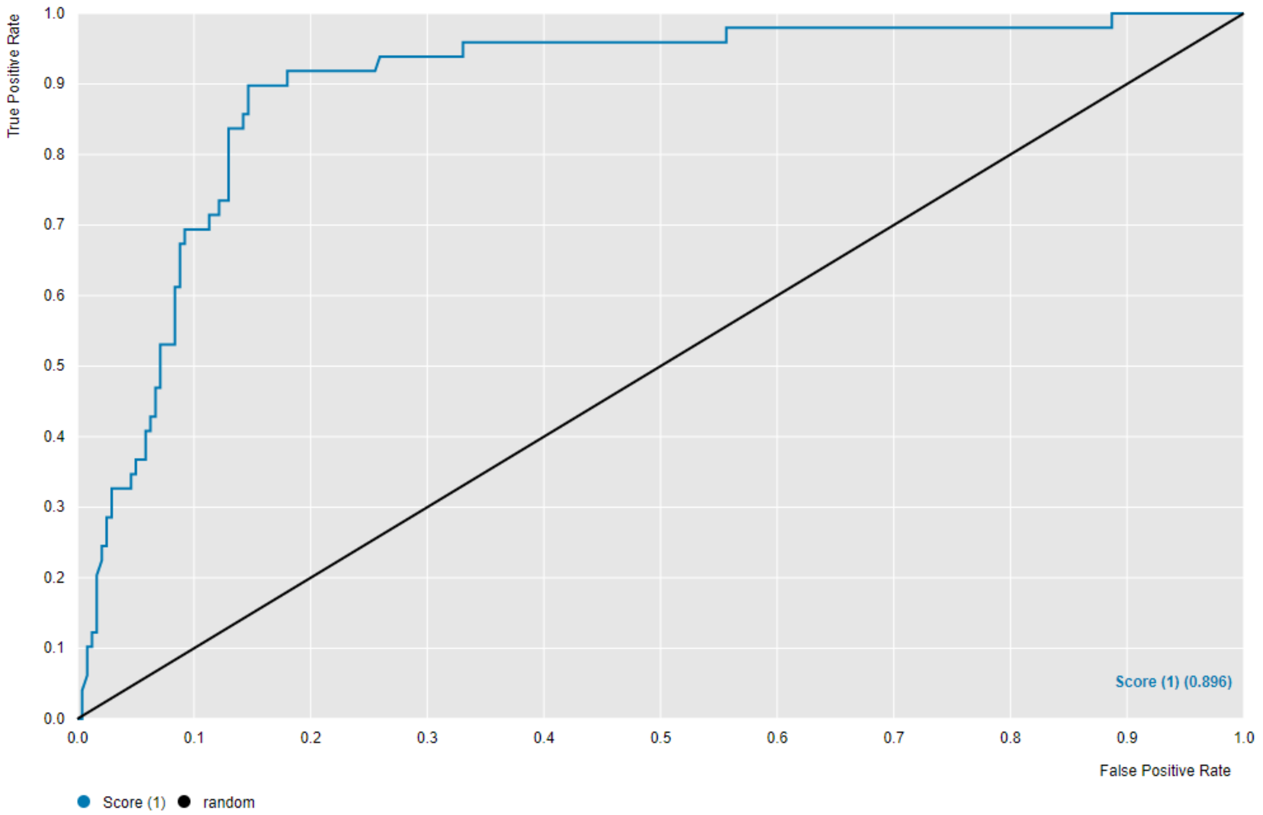

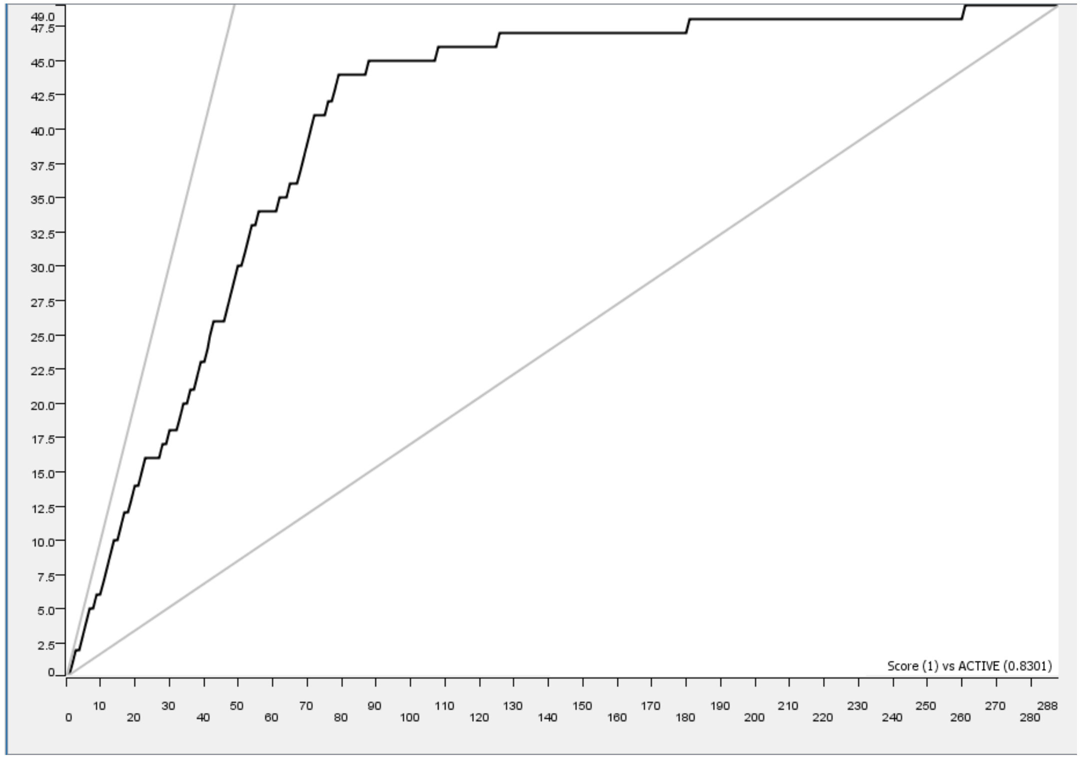

N=289

Top 10% Mean IC50	8.0 nM

	Predicted Active	Predicted Inactive
Active	44	5
Inactive	43	196

<10 nM DefGood in c-Abl

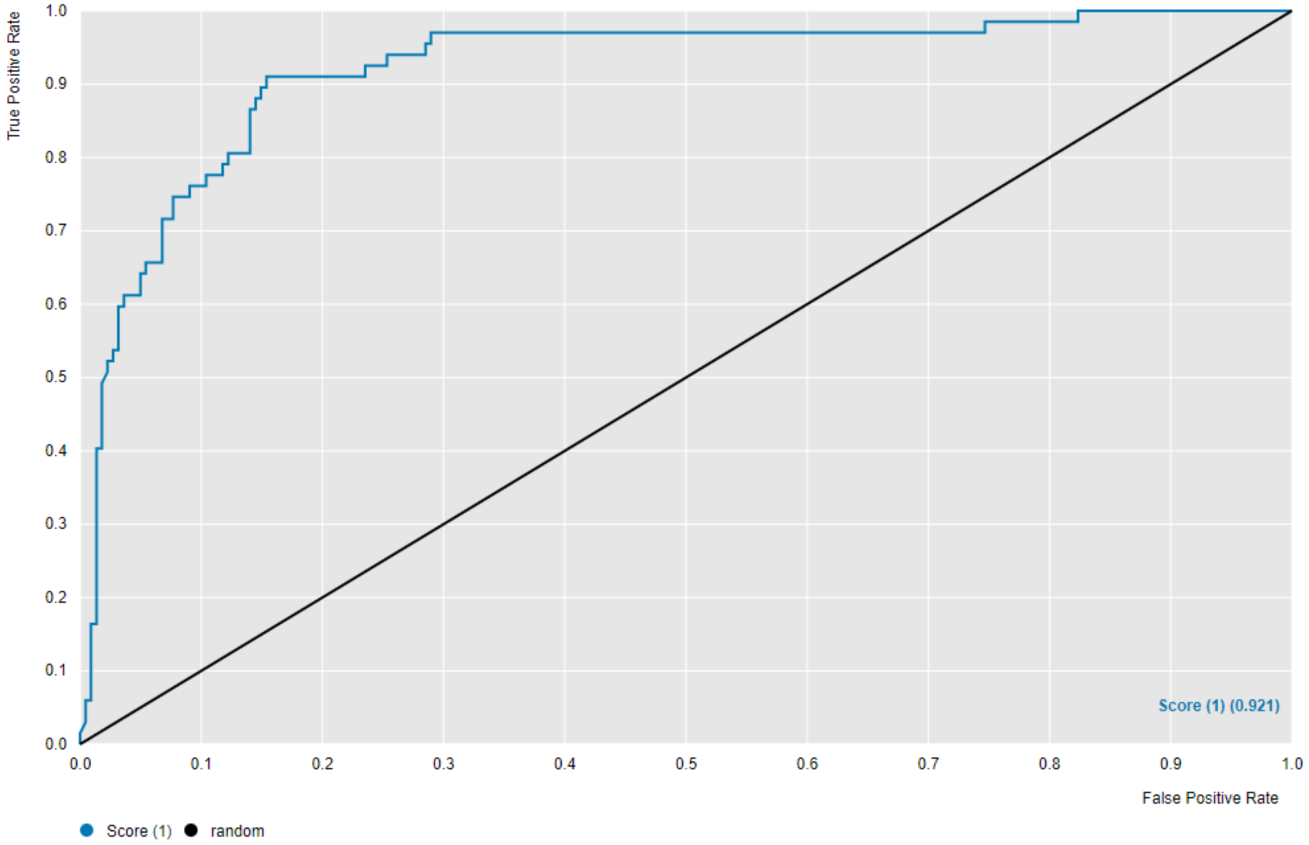

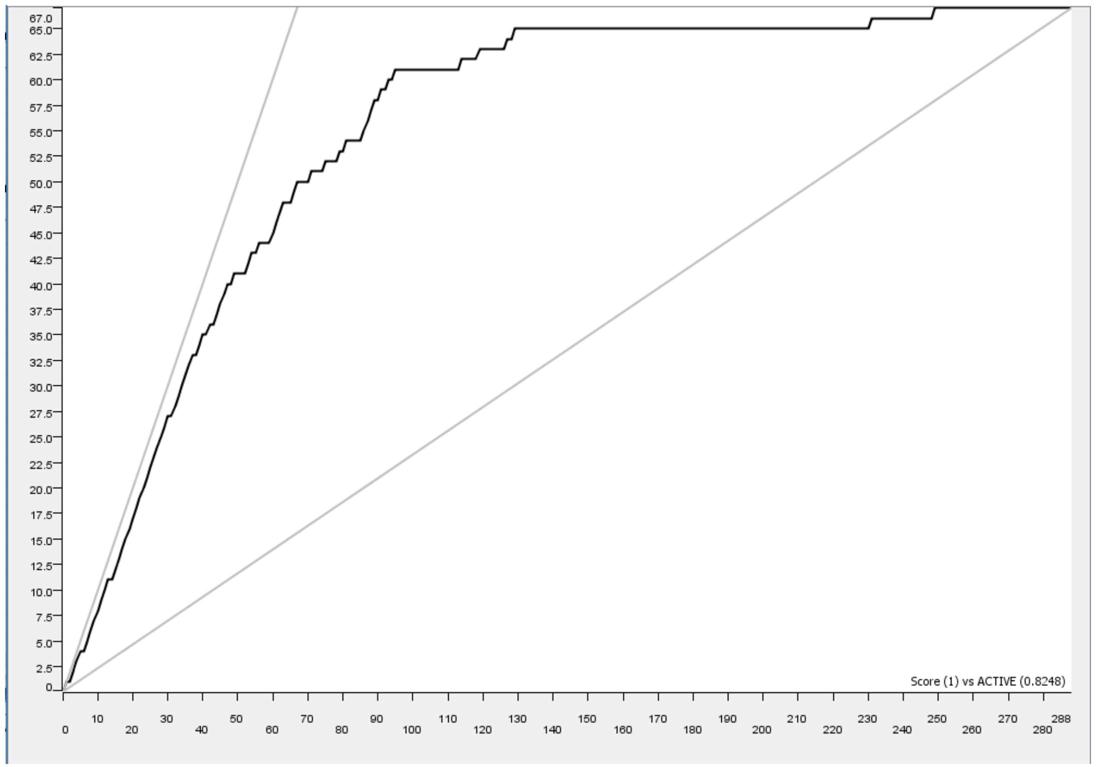

N=289

**Top 10% Mean IC50**	**26.5 nM (one 554 nM Compound)**

	**Predicted Active**	**Predicted Inactive**
Active	61	6
Inactive	43	178

<15 nM DefGood in c-Abl

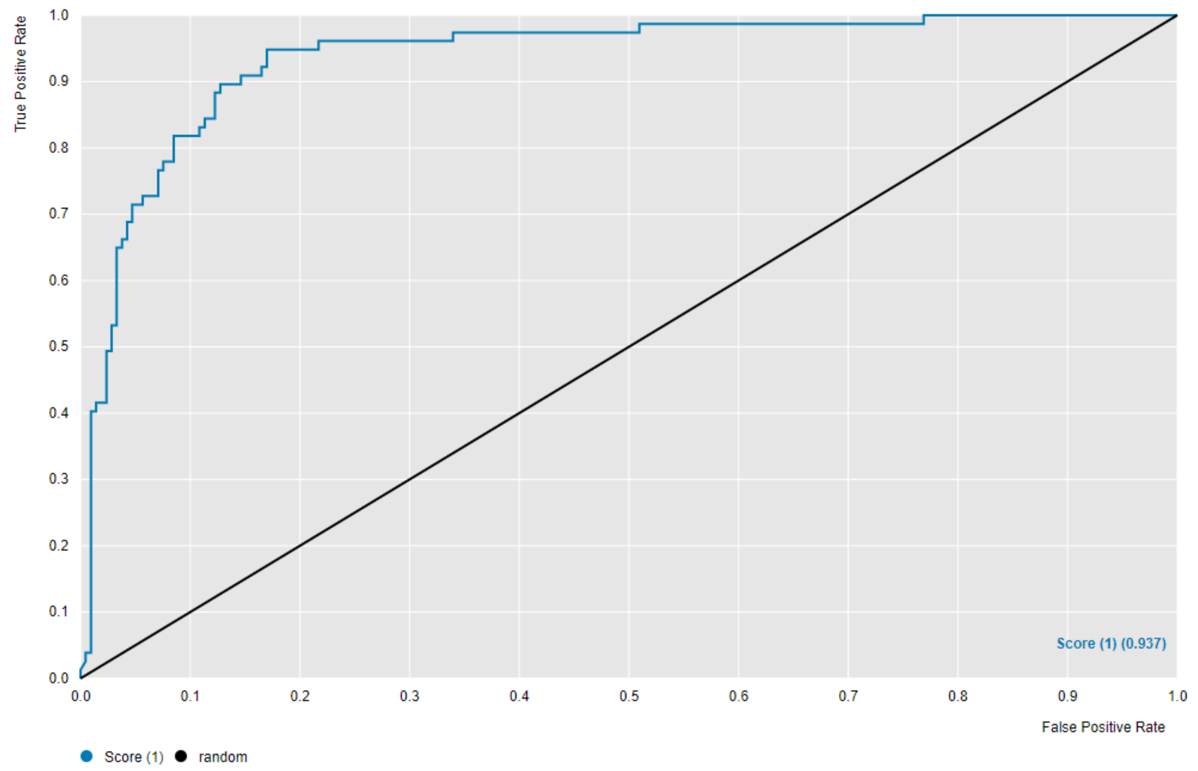

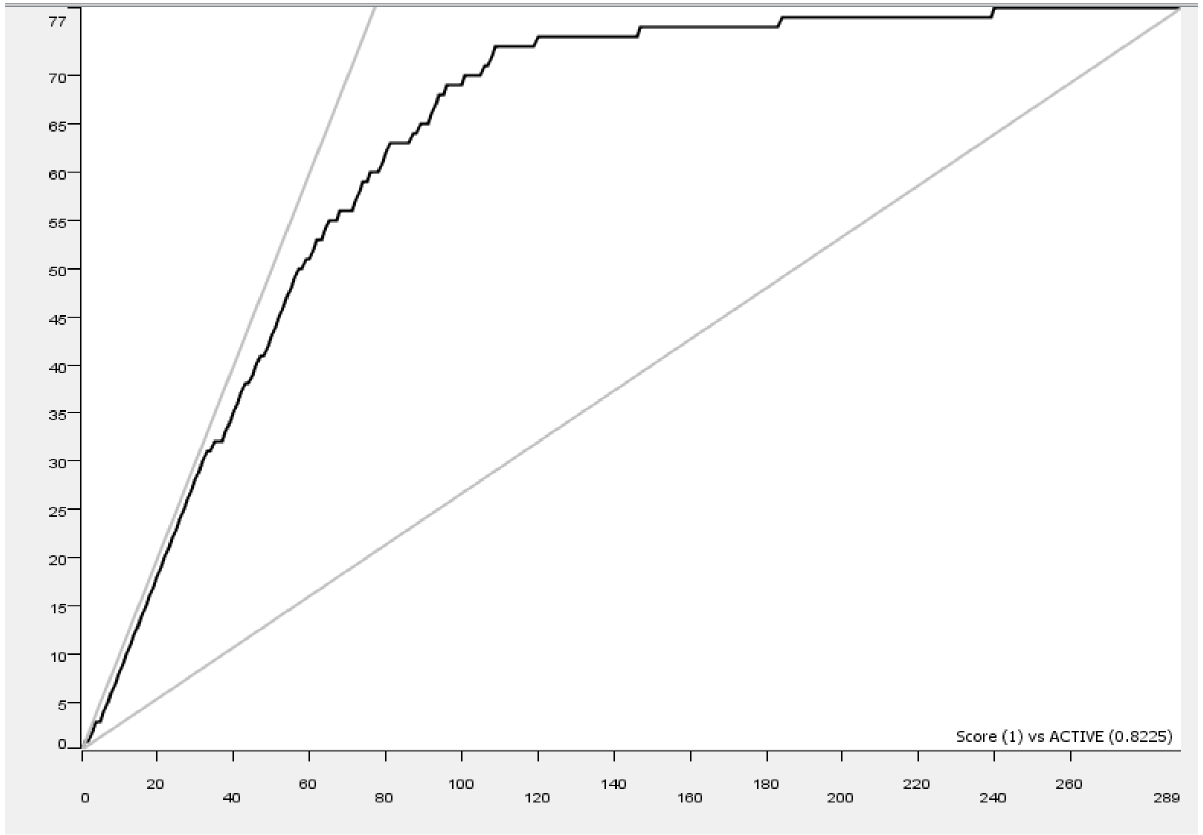

N=289

Top 10% Mean IC50	35.4 nM (one 800 nM compound)

	Predicted Active	Predicted Inactive
Active	71	6
Inactive	35	177

<20 nM DefGood in c-Abl

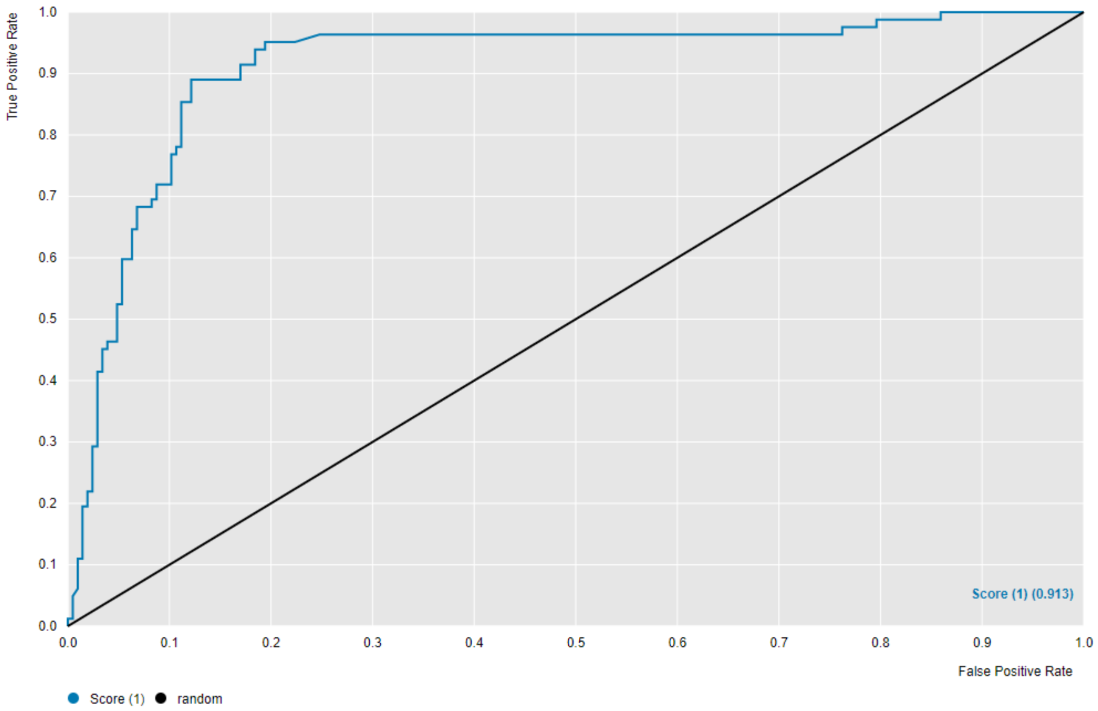

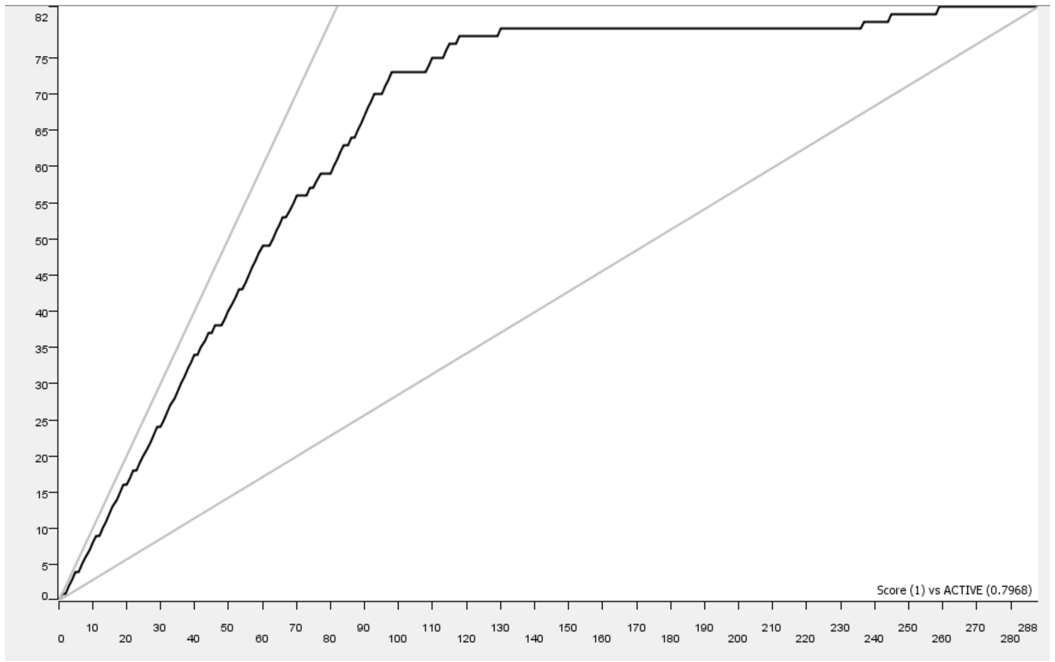

N=289

**Top 10% Mean IC50**	**77.4 nM (several >100 nM mistakes)**

	**Predicted Active**	**Predicted Inactive**
Active	73	9
Inactive	33	173

<25 nM DefGood in c-Abl

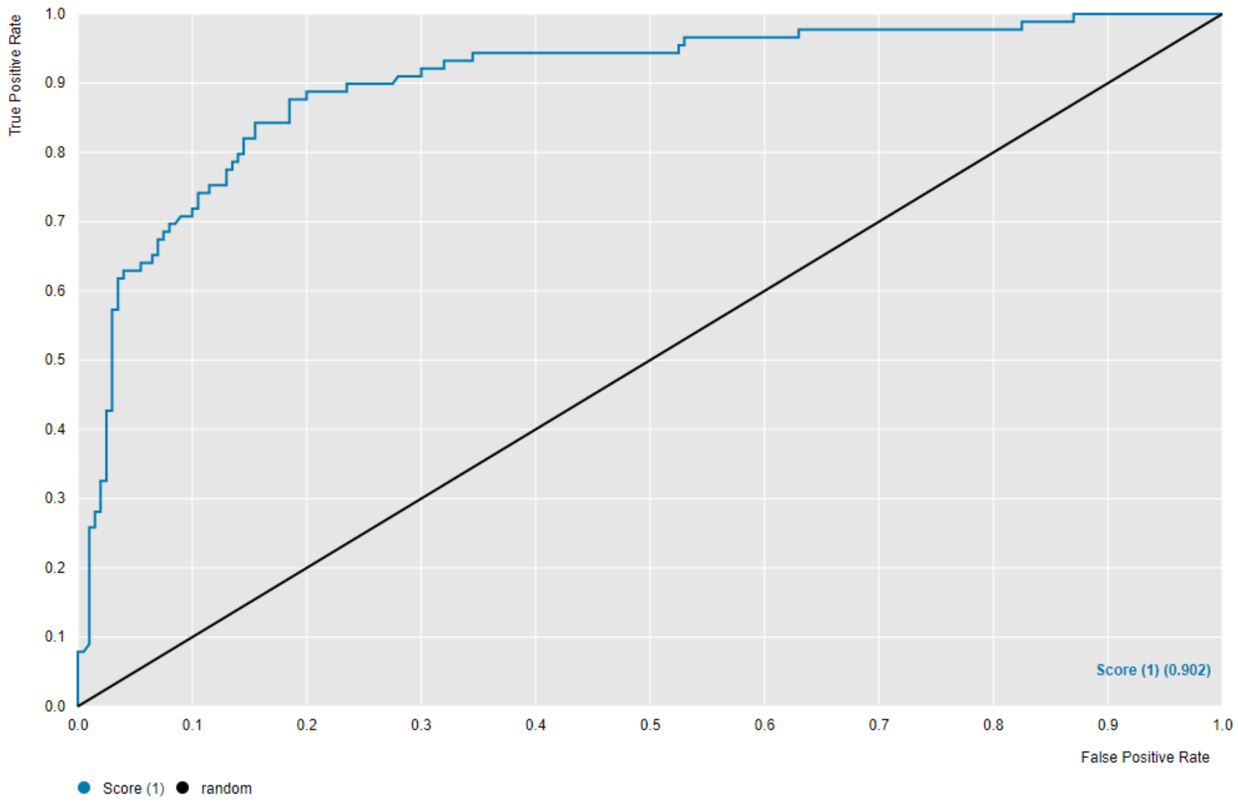

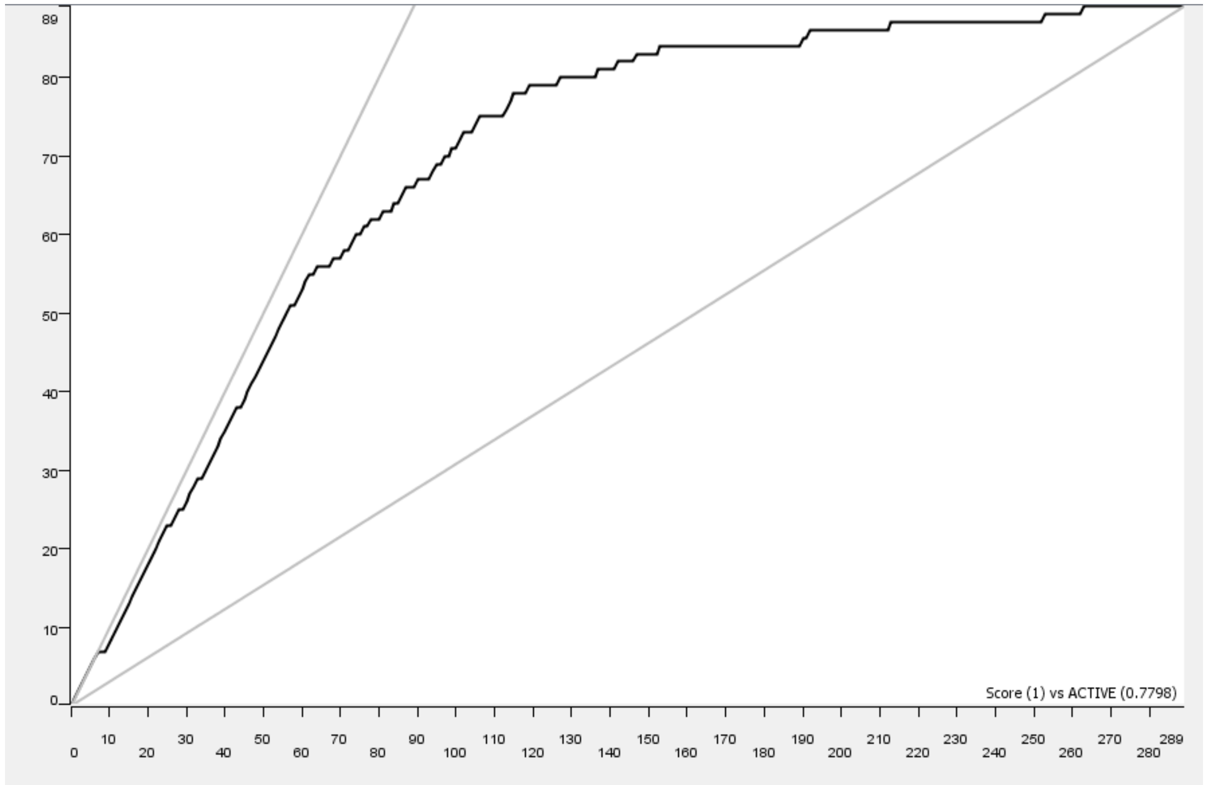

Top 10% Mean IC50	82.7 nM (several >100 nM mistakes)

	Predicted Active	Predicted Inactive
Active	70	19
Inactive	27	173

Decision on DefGood

- <15 nM has the best specificity and enrichment. Not the best mean IC50, but that is because of one significant mistake (800 nM).

SVM

- Doesn’t work (Support vectors could not be generated)

RF - <15 nM DefGood in c-Abl

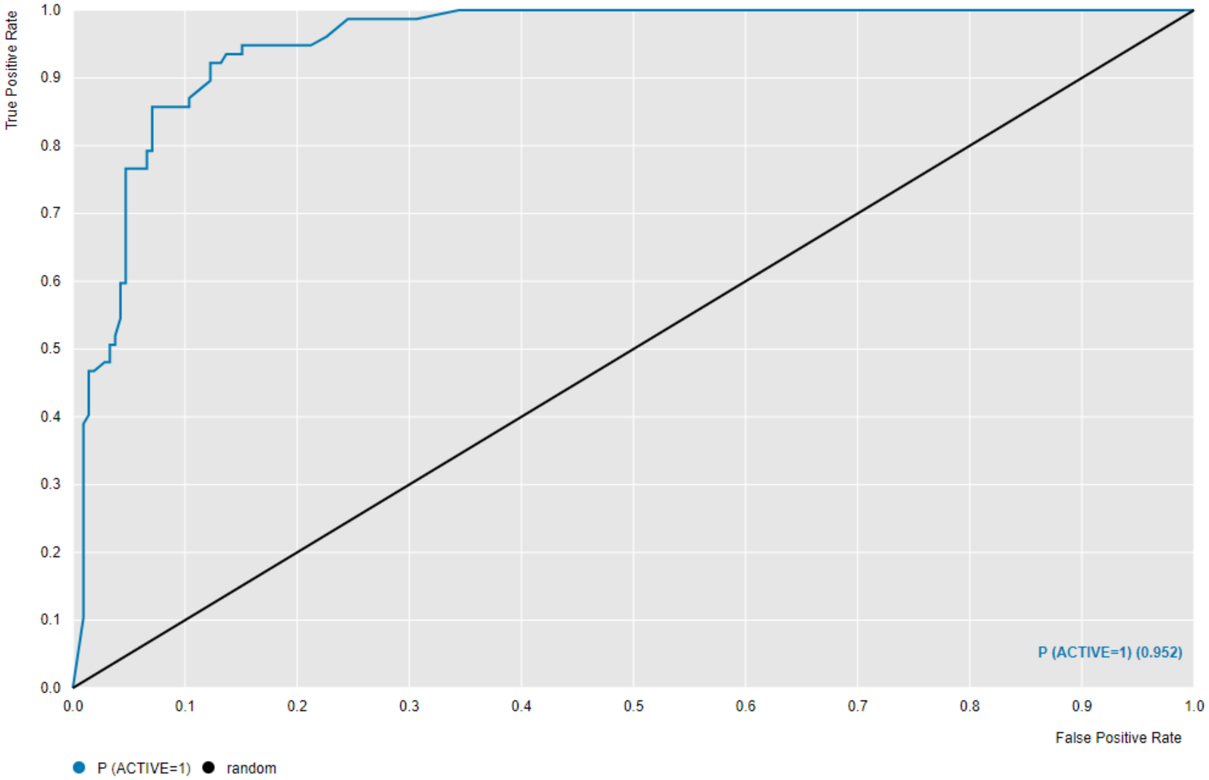

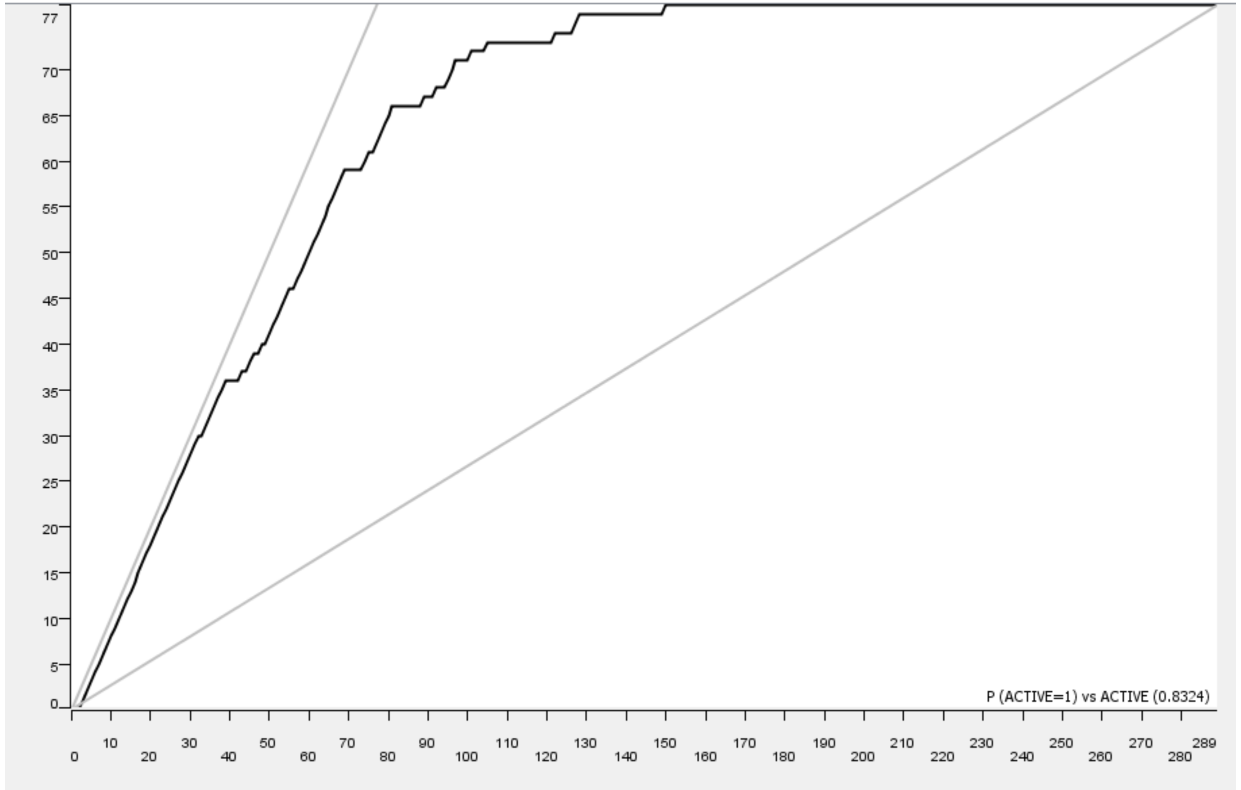

N=289

**Top 10% Mean IC50**	**35.0 nM (one 800 nM compound)**

	Predicted Active	Predicted Inactive
Active	66	11
Inactive	15	197

PNN - <15 nM DefGood in c-Abl

- Properties List (46 calculatable properties were used)
- Parameterization (Global Accuracy for Activity at a 15 nM decision value was used, and a 5-fold stratified sampling cross validation was used to parameterize theta minus and theta plus) Theta Minus = 0.05 and Theta Plus = 0.65

PNN - <15 nM DefGood in c-Abl

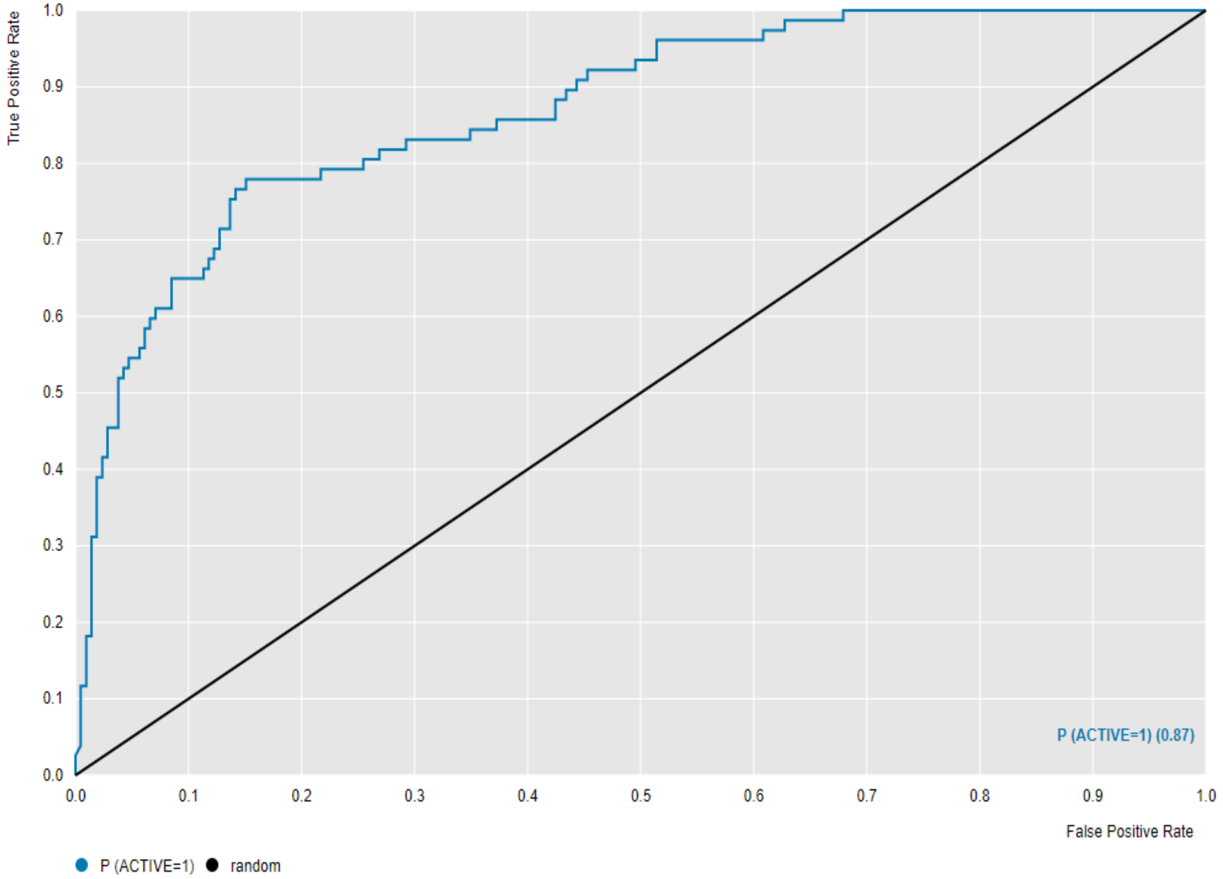

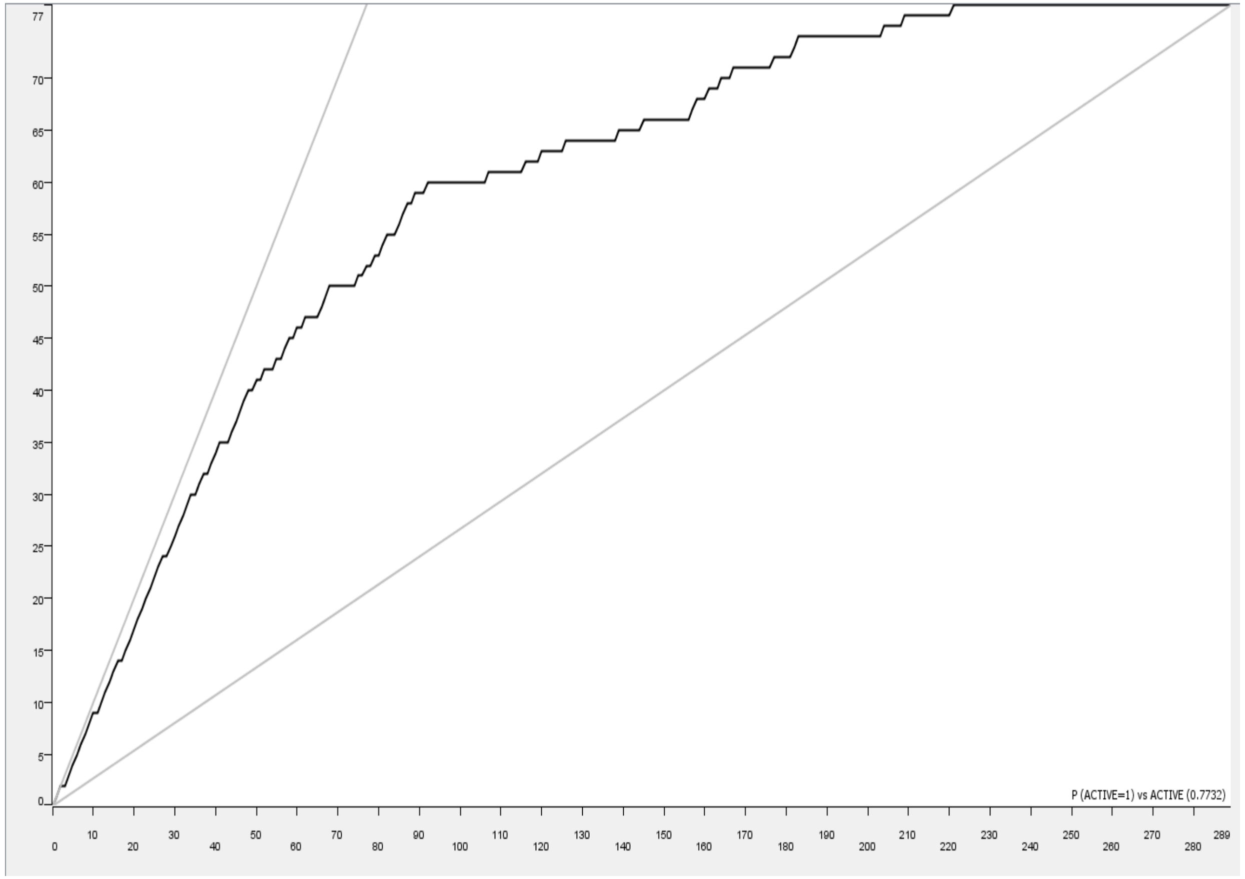

N=289

**Top 10% Mean IC50**	**41.8 nM (one 800 nM compound)**

	**Predicted Active**	**Predicted Inactive**
Active	27	50
Inactive	4	208

NBN Error Tolerance- <15 nM DefGood in c-Abl

- 0-50% absolute error

<15 nM DefGood in c-Abl, 5% error; Random seed = 1515533876005

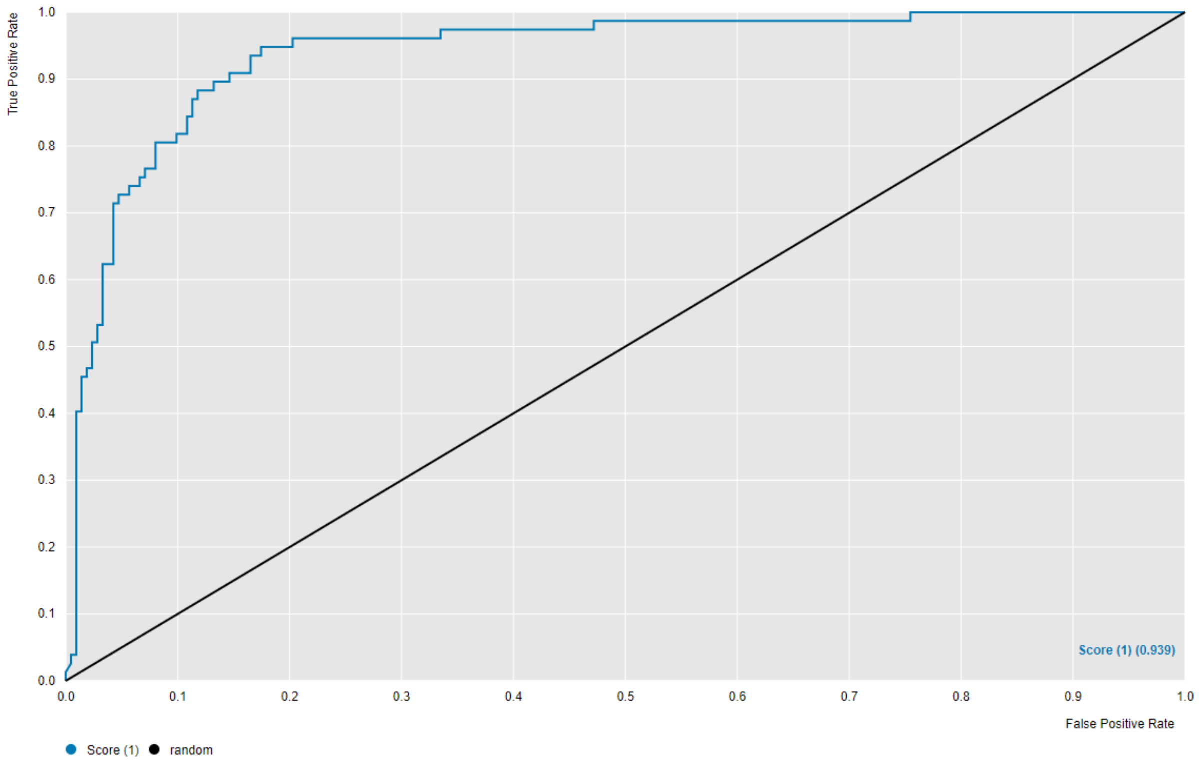

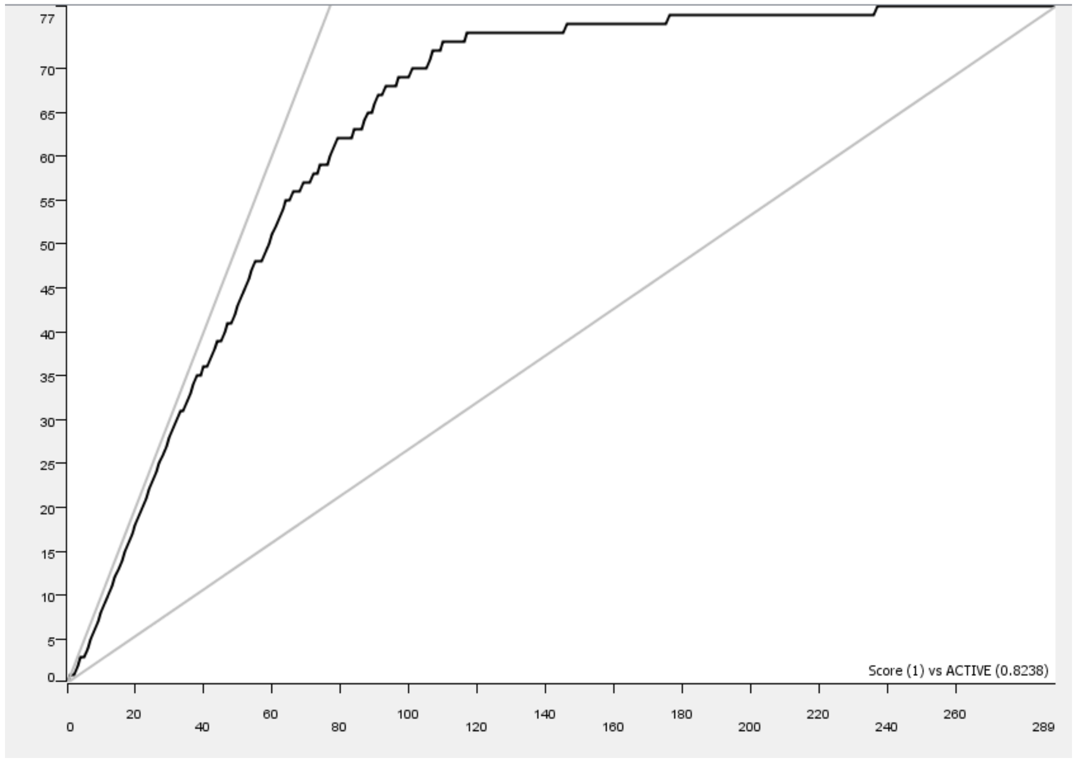

N=289

**Top 10% Mean IC50**	**35.7 nM (one 800 nM compound)**

	Predicted Active	Predicted Inactive
Active	70	7
Inactive	31	181

<15 nM DefGood in c-Abl, 10% error

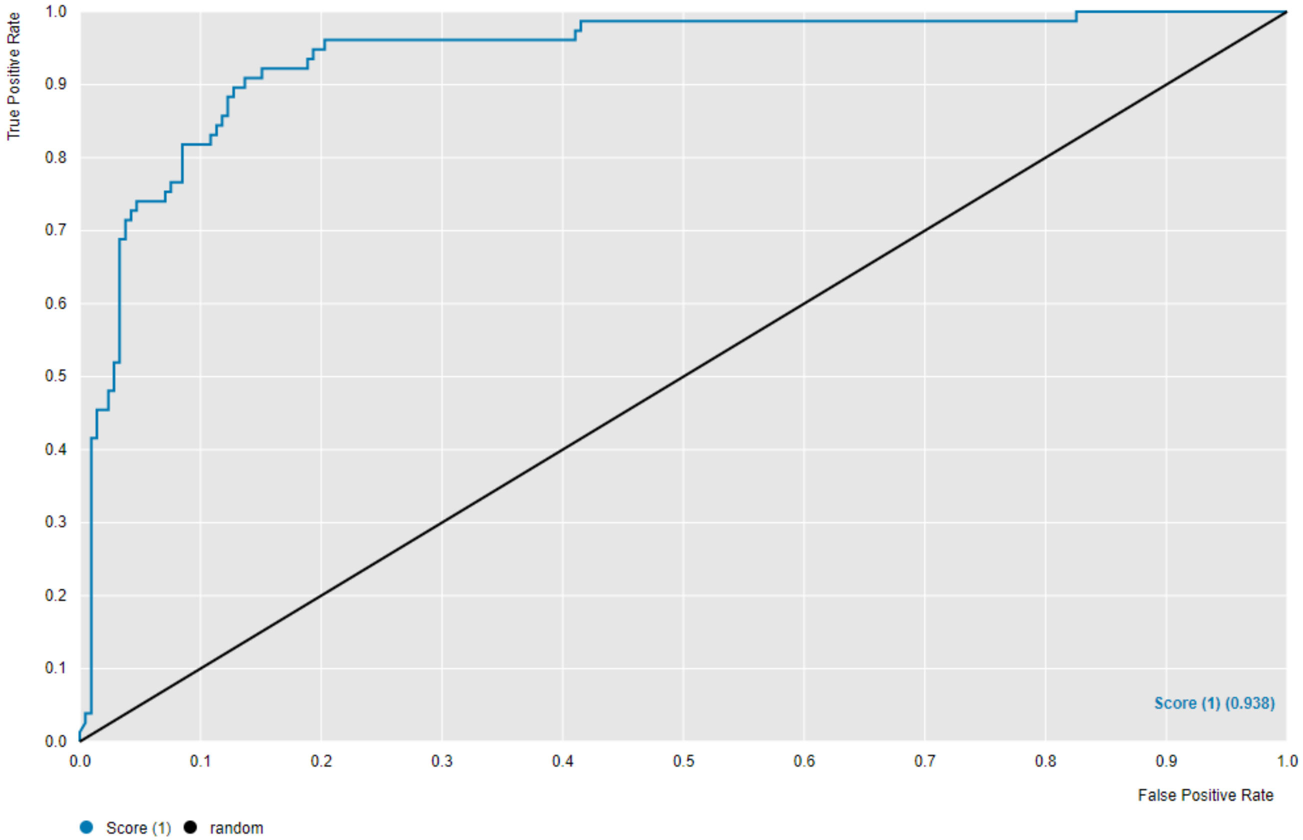

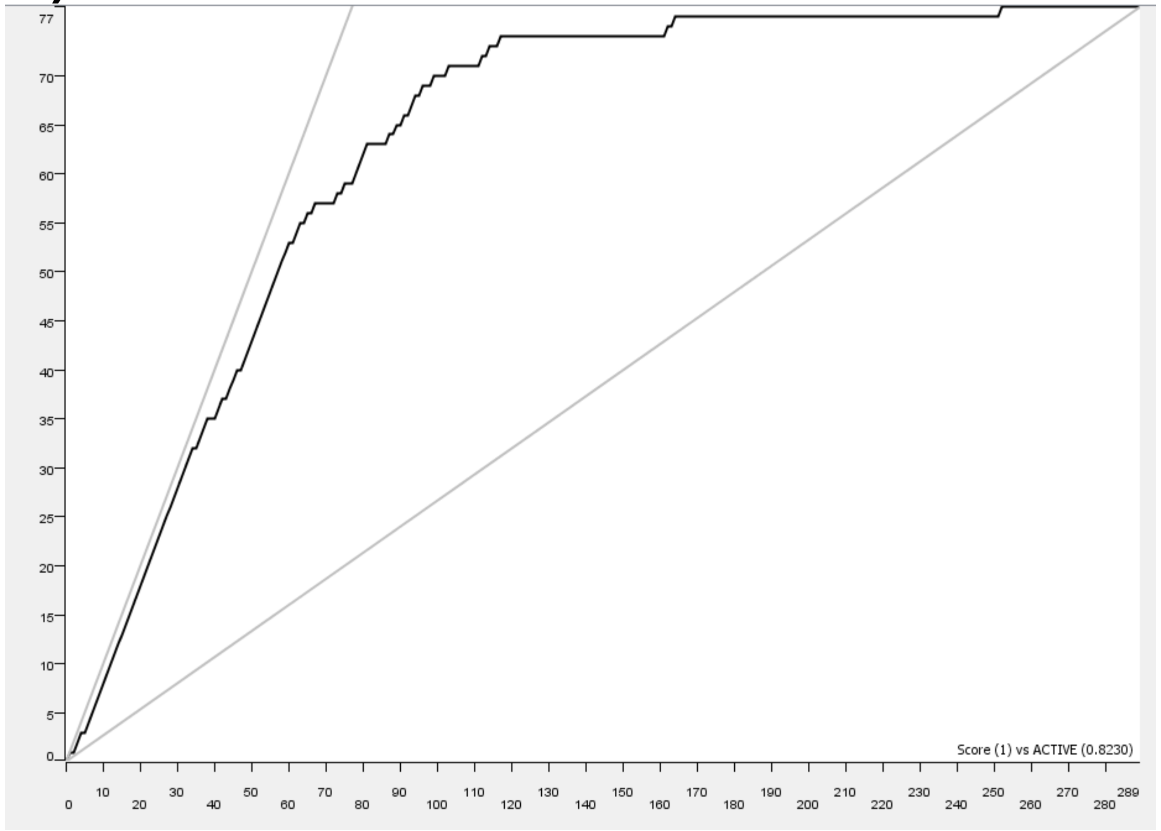

N=289

**Top 10% Mean IC50**	**35.5 nM (one 800 nM compound)**

	Predicted Active	Predicted Inactive
Active	70	7
Inactive	31	181

<15 nM DefGood in c-Abl, 15% error

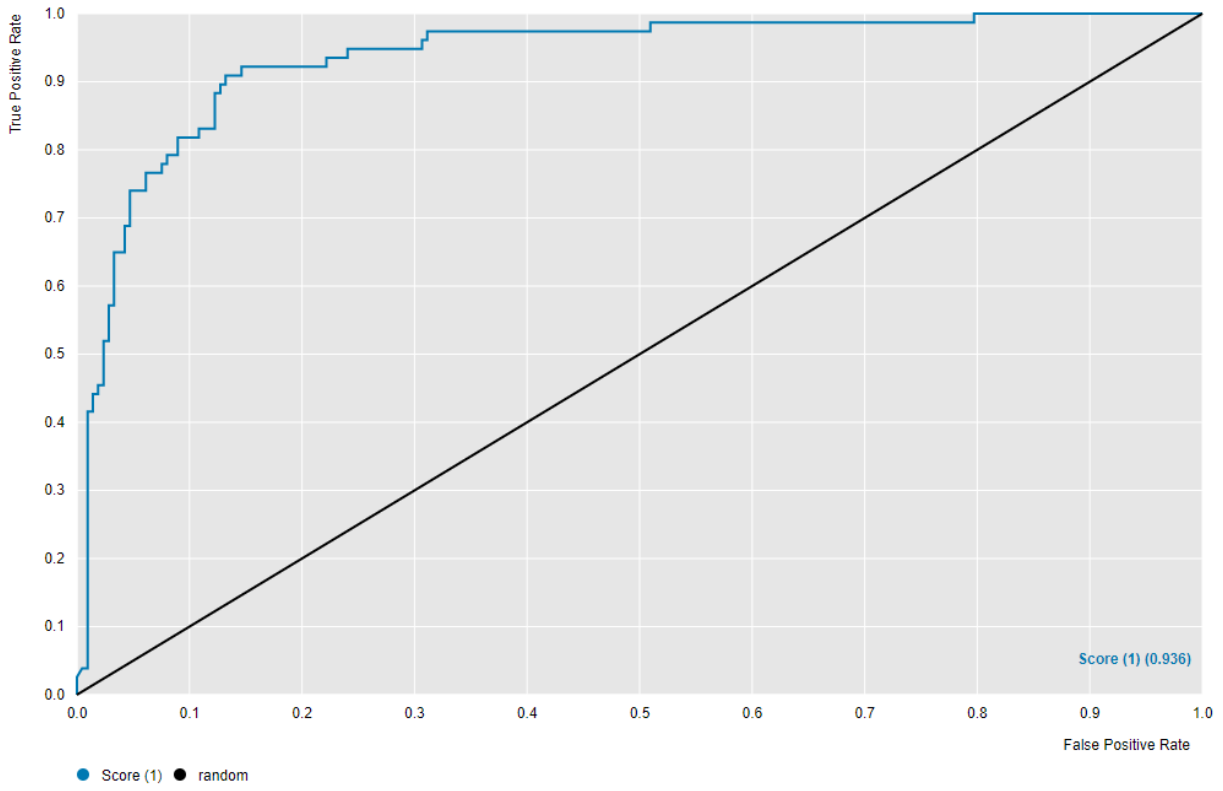

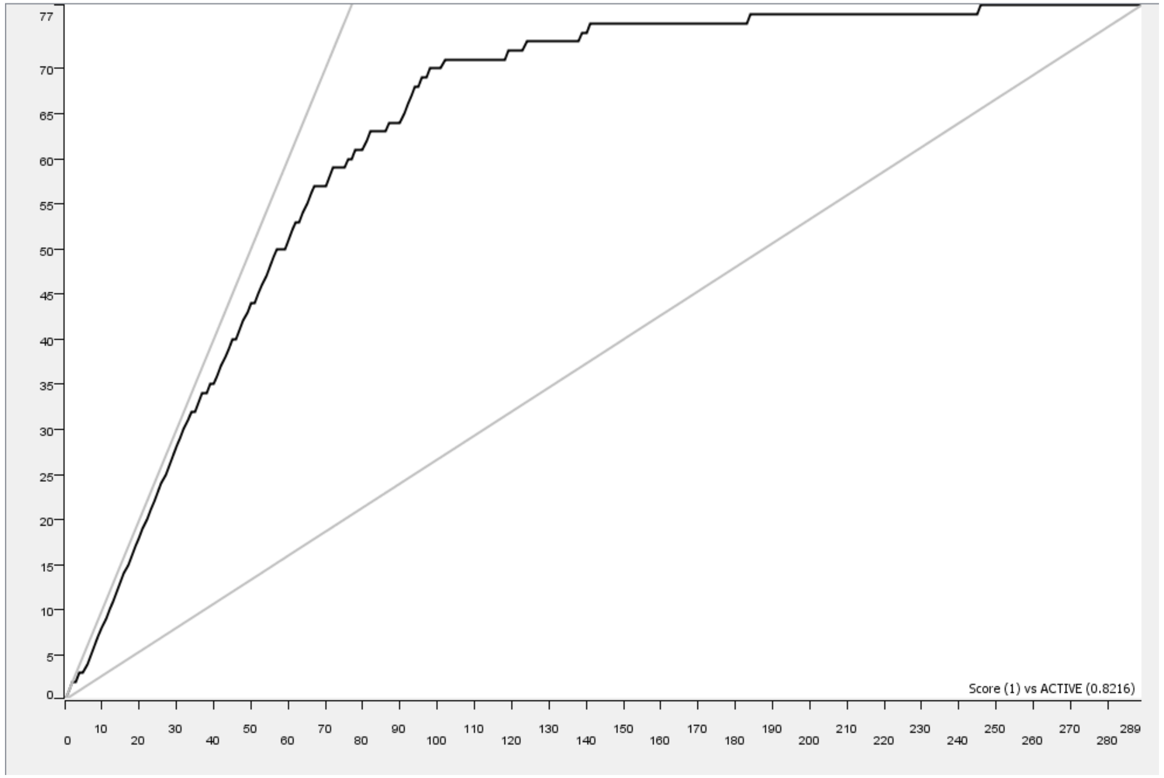

N=289

**Top 10% Mean IC50**	**35.4 nM (one 800 nM compound)**

	Predicted Active	Predicted Inactive
Active	71	6
Inactive	33	179

<15 nM DefGood in c-Abl, 20% error

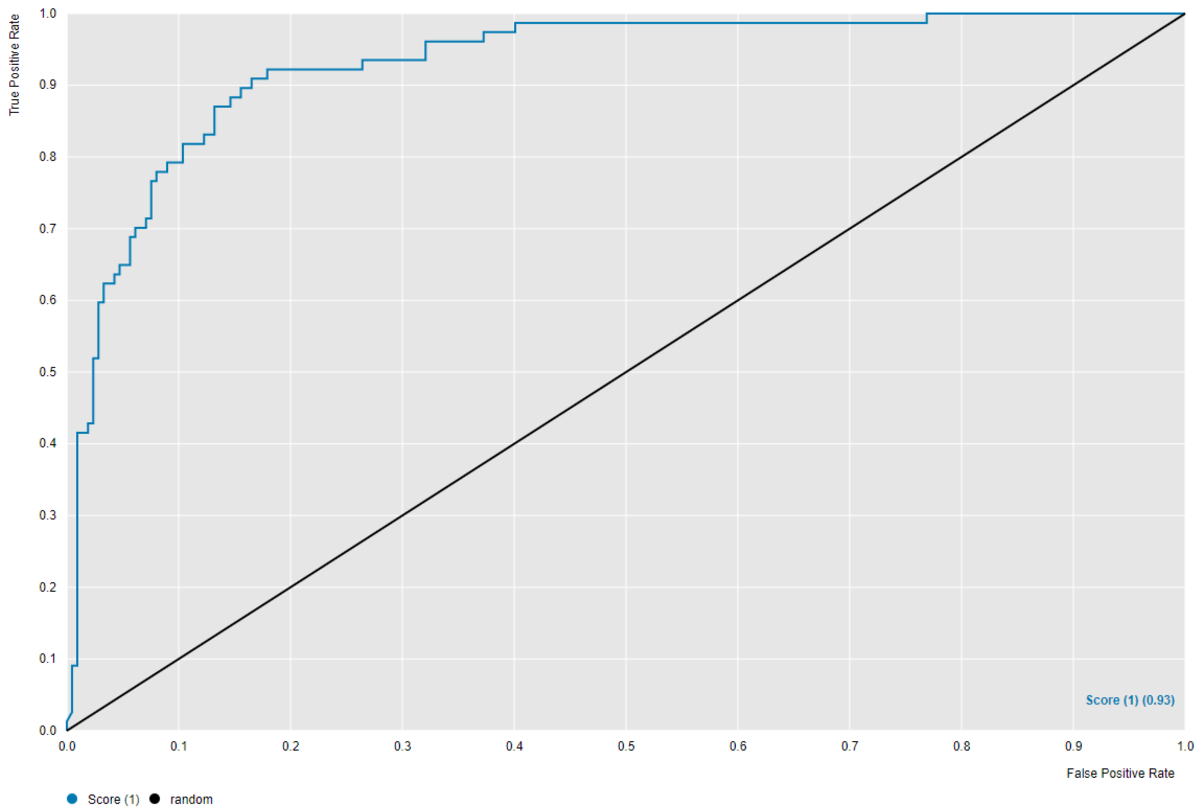

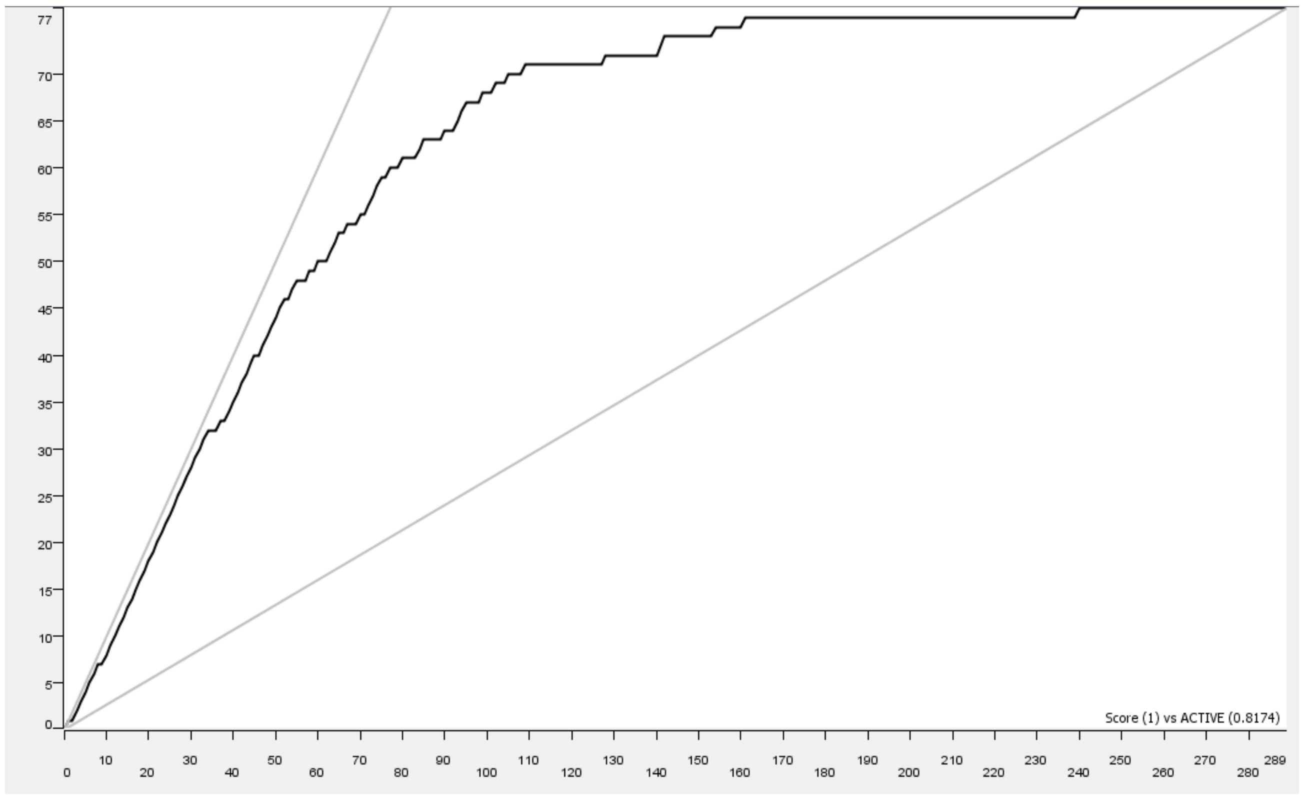

N=289

Top 10% Mean IC50	35.4 nM (one 800 nM compound)

	Predicted Active	Predicted Inactive
Active	74	3
Inactive	73	139

<15 nM DefGood in c-Abl, 30% error

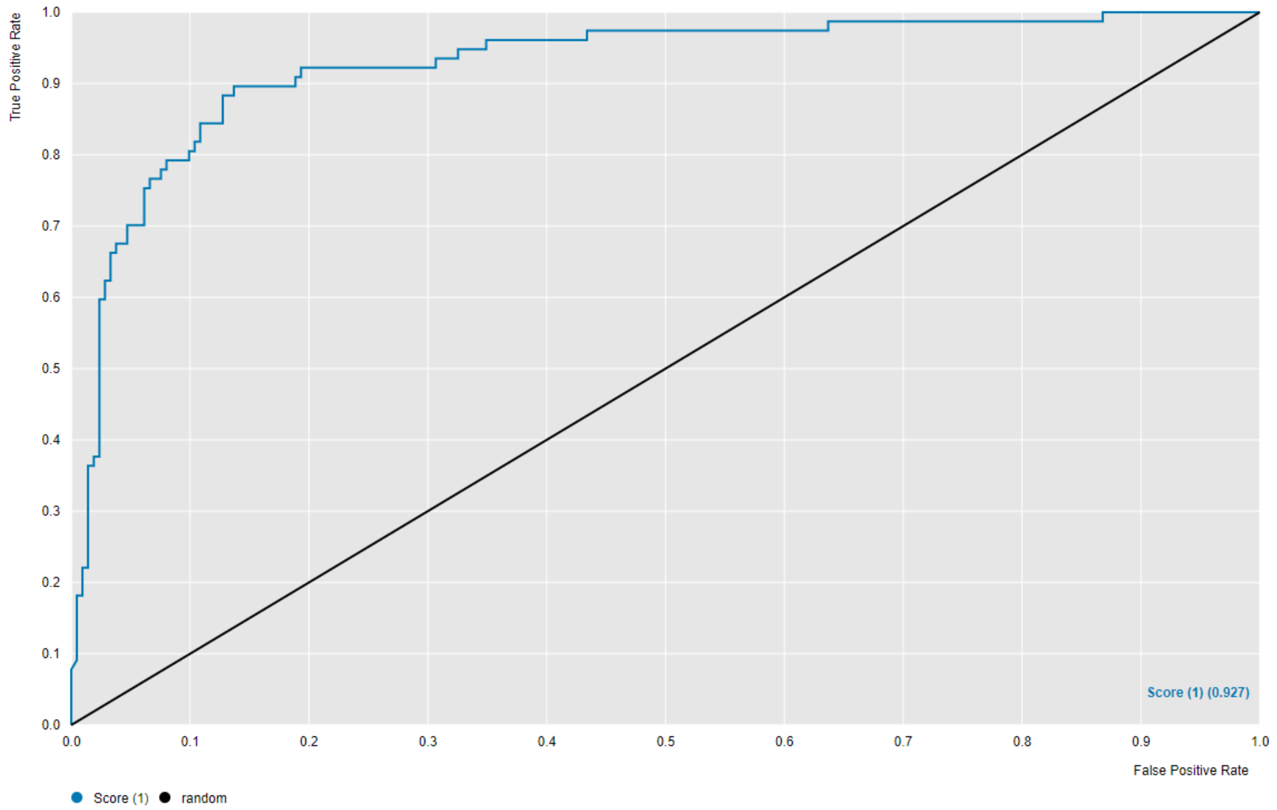

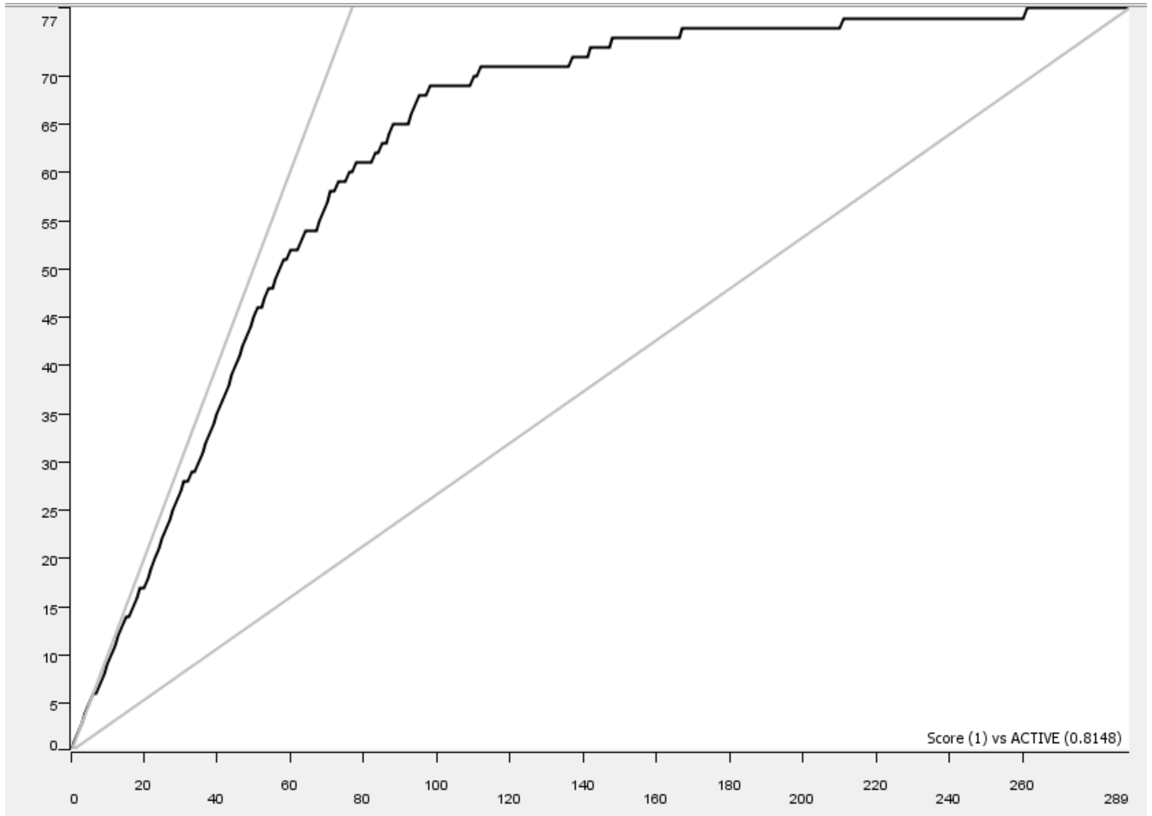

N=289

Top 10% Mean IC50	36.0 nM (one 800 nM compound)

	Predicted Active	Predicted Inactive
Active	67	10
Inactive	27	185

<15 nM DefGood in c-Abl, 40% error

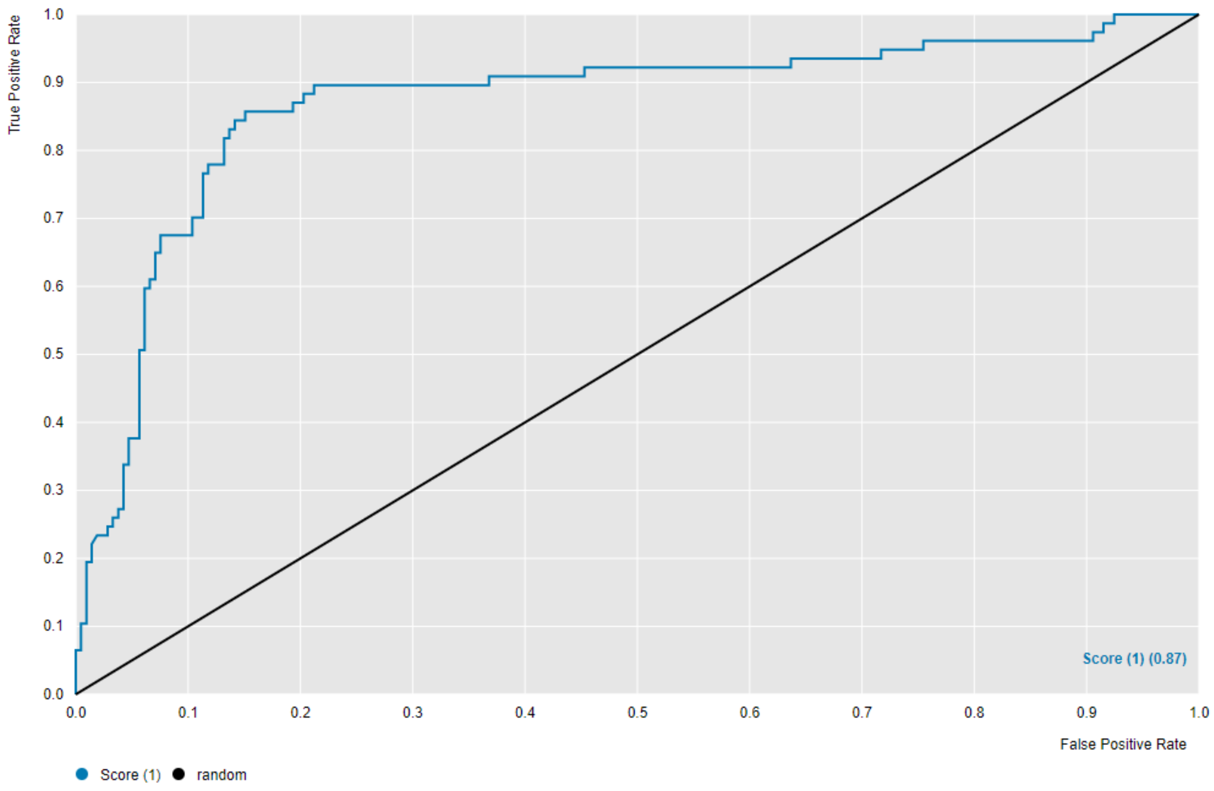

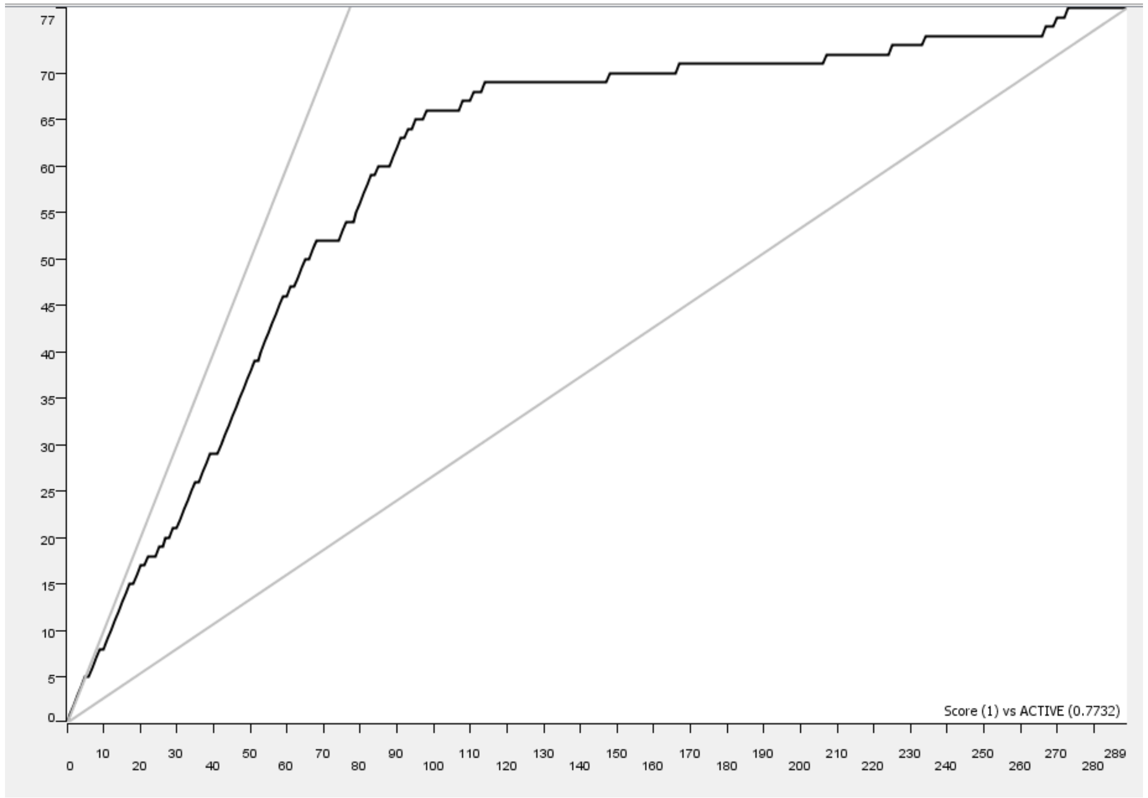

N=289

Top 10% Mean IC50	41.6 nM (one 800 nM compound)

	Predicted Active	Predicted Inactive
Active	64	13
Inactive	29	183

<15 nM DefGood in c-Abl, 45% error

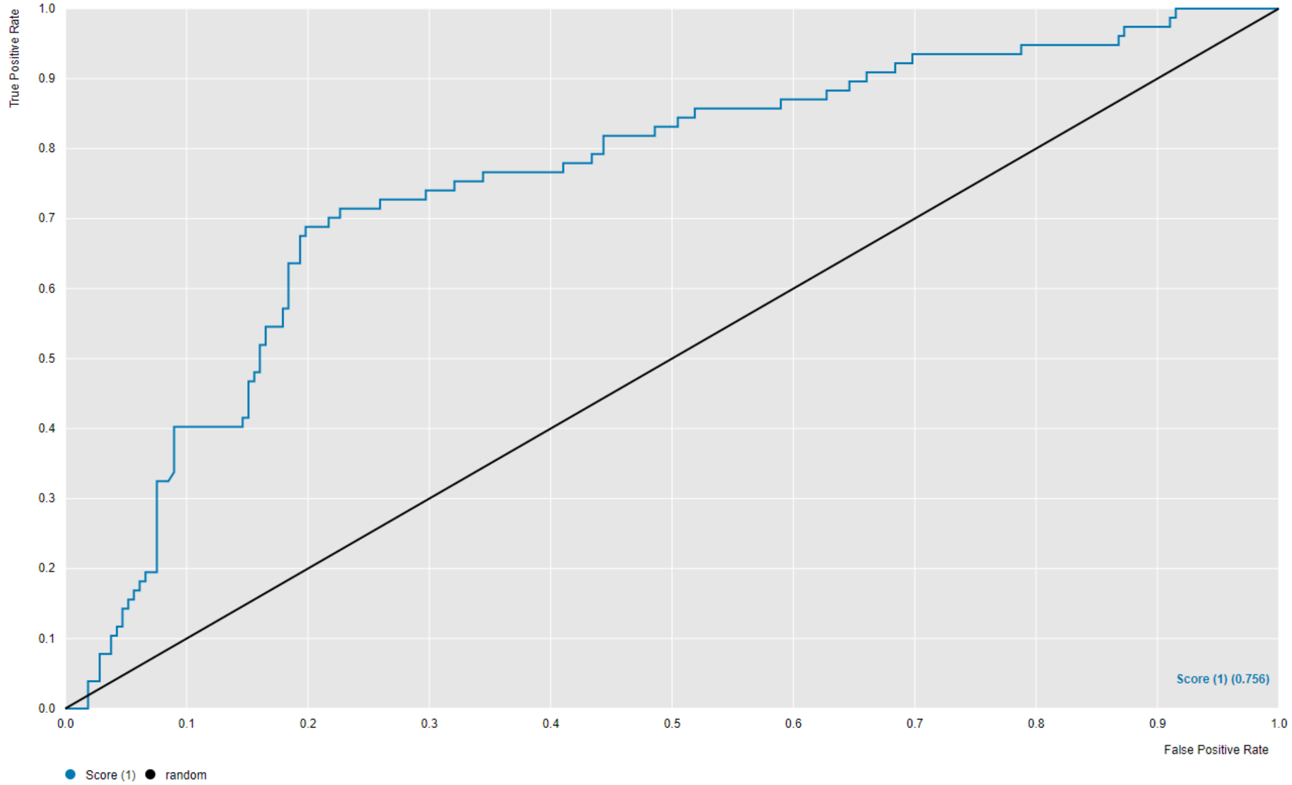

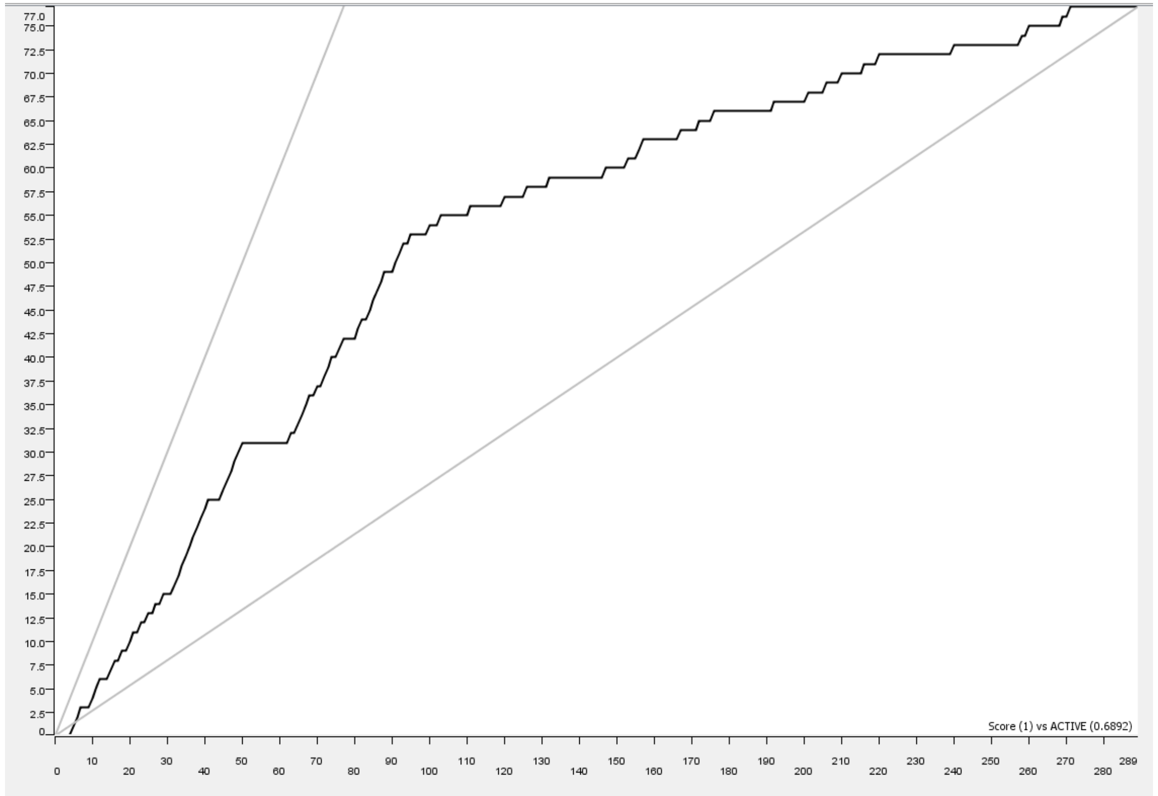

N=289

**Top 10% Mean IC50**	**2,000 nM**

	Predicted Active	Predicted Inactive
Active	53	24
Inactive	46	166

<15 nM DefGood in c-Abl, 50% error

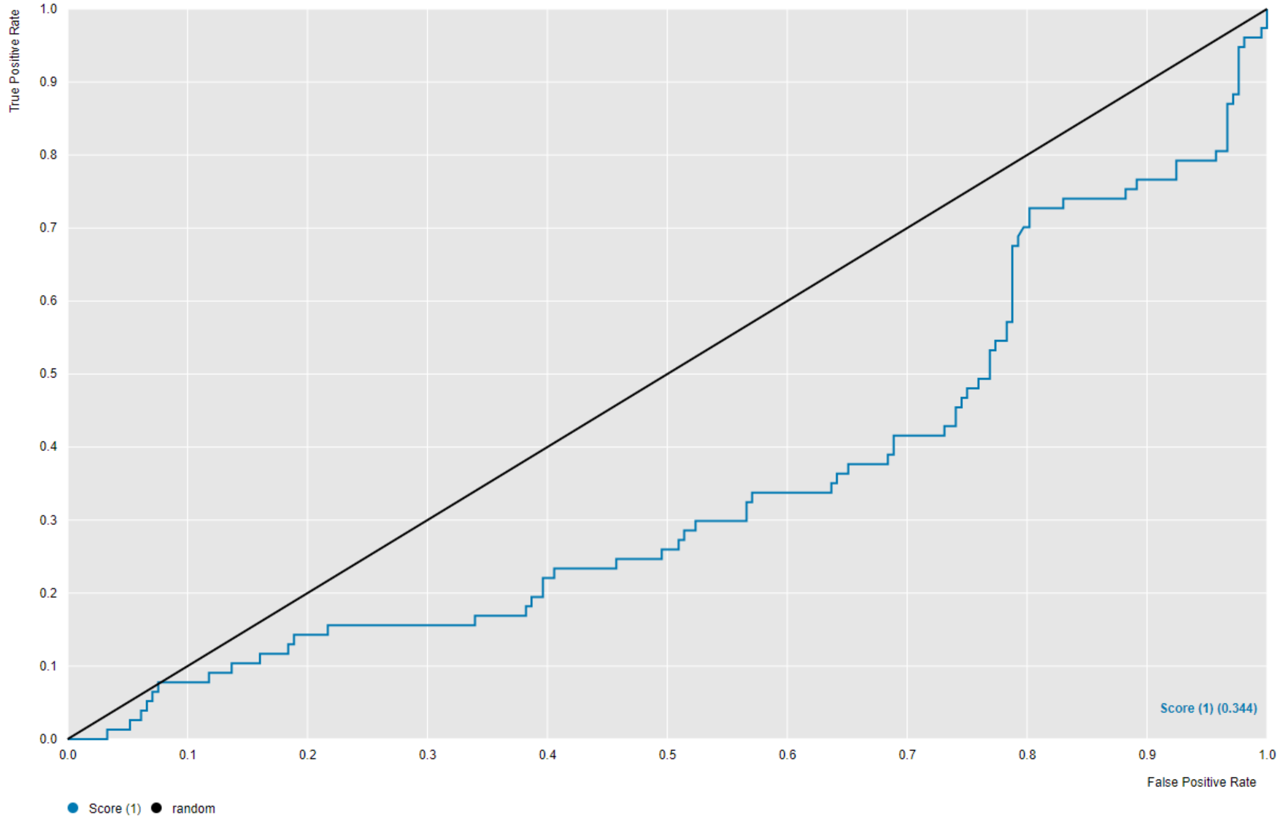

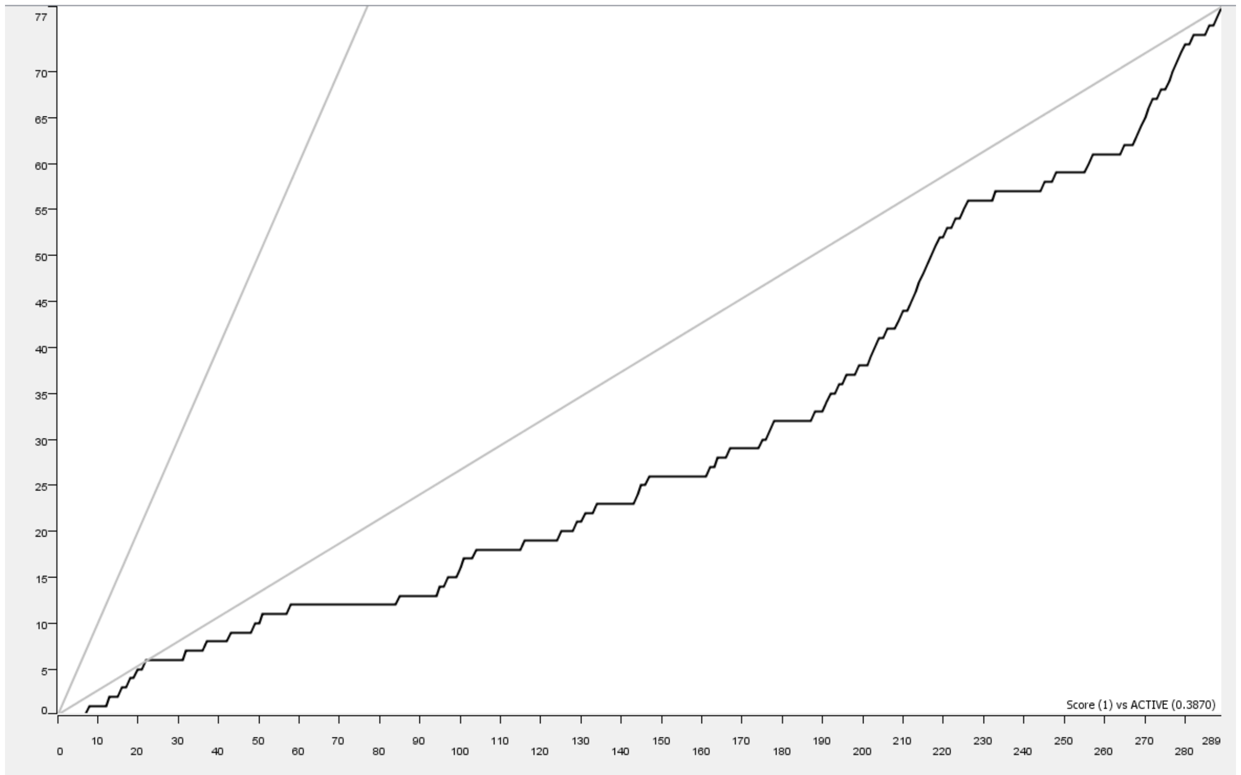

N=289

**Top 10% Mean IC50**	**6,100 nM**

	Predicted Active	Predicted Inactive
Active	0	77
Inactive	6	206

<15 nM DefGood in c-Abl, 10% error; Random seed = 429

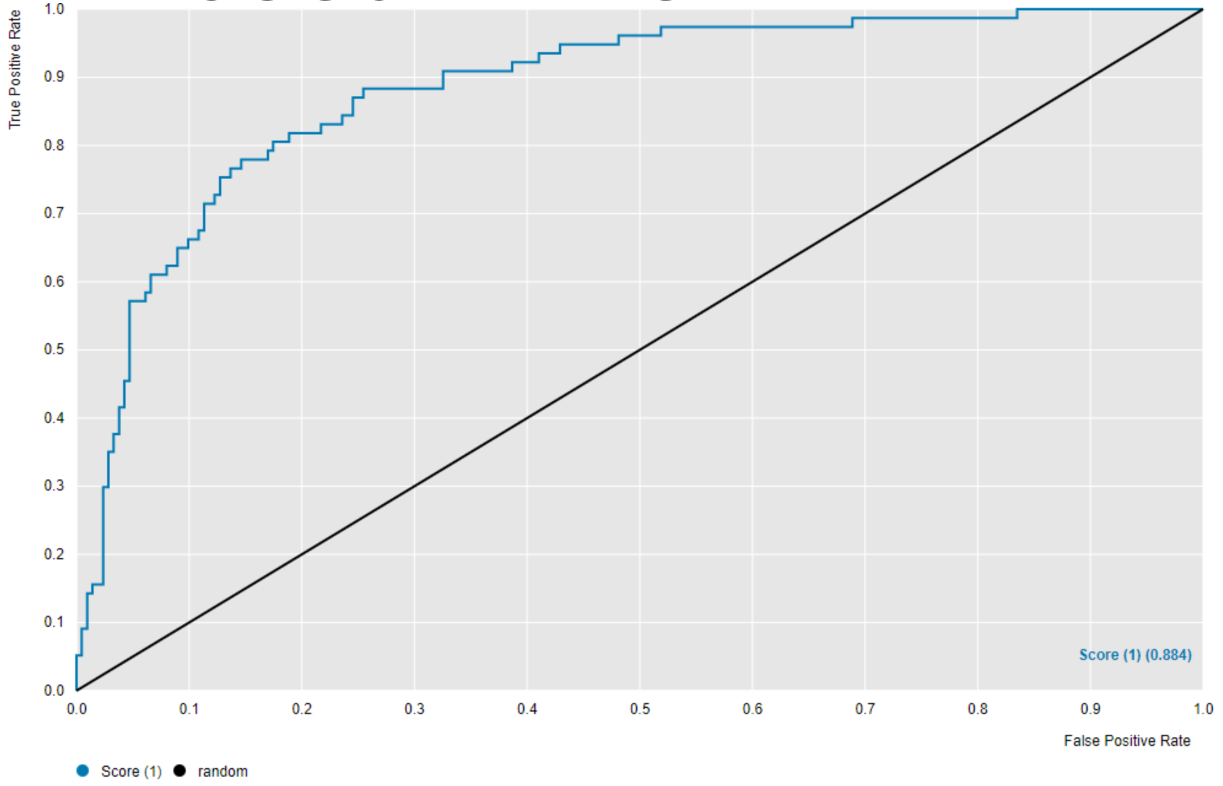

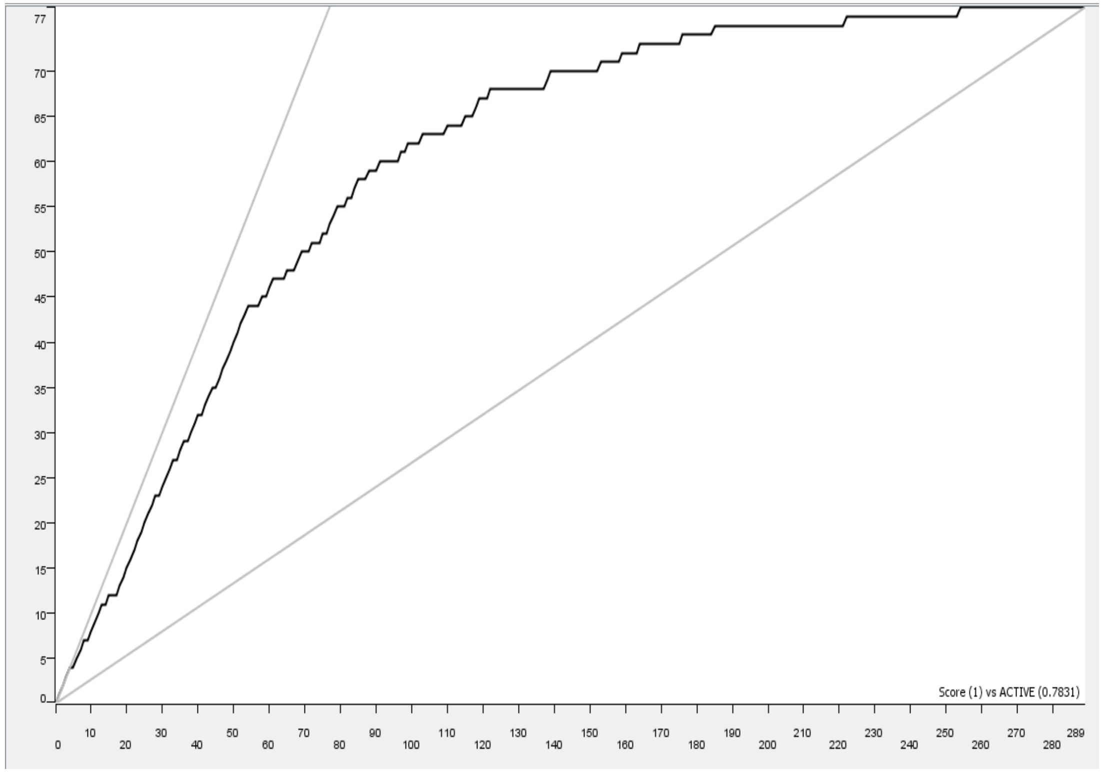

N=289

Top 10% Mean IC50	39.5 nM (one 800 nM compound)

	Predicted Active	Predicted Inactive
Active	60	17
Inactive	32	180

<15 nM DefGood in c-Abl, 20% error; Random seed = 429

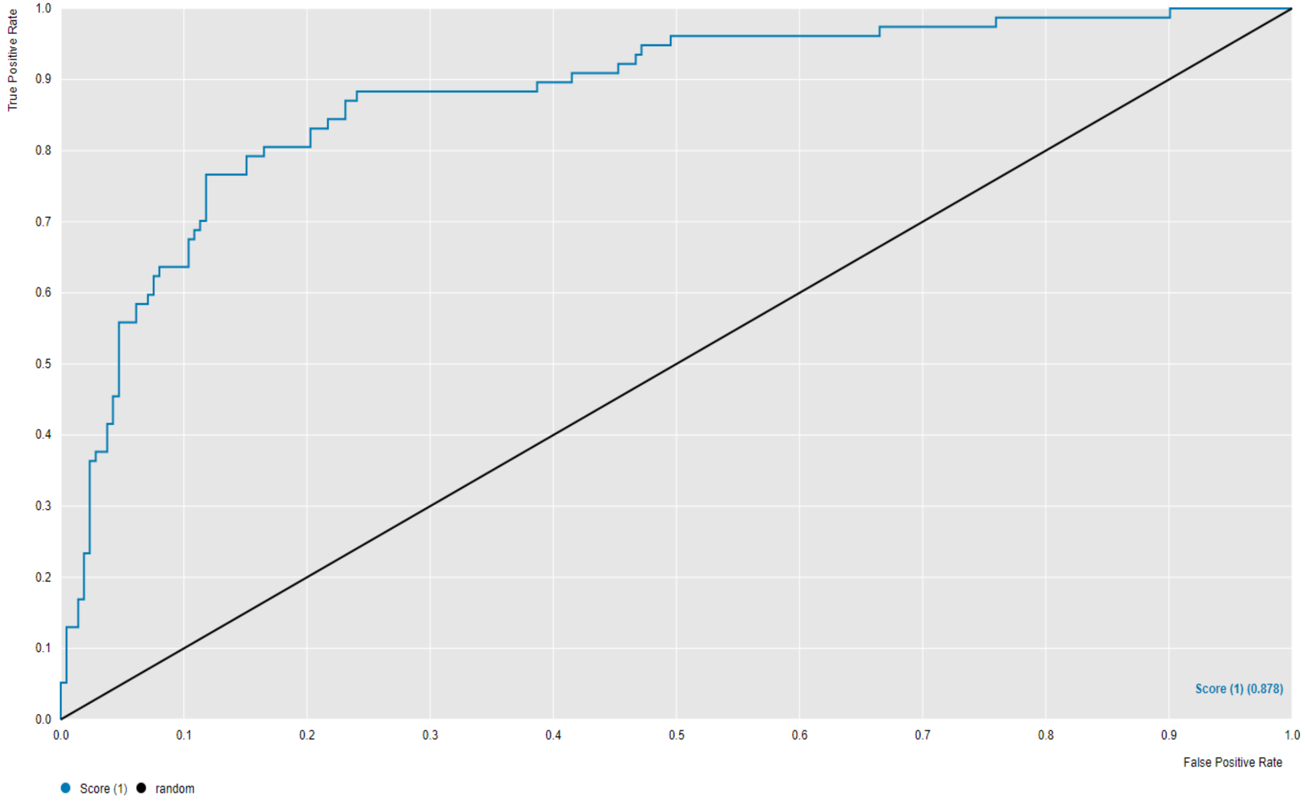

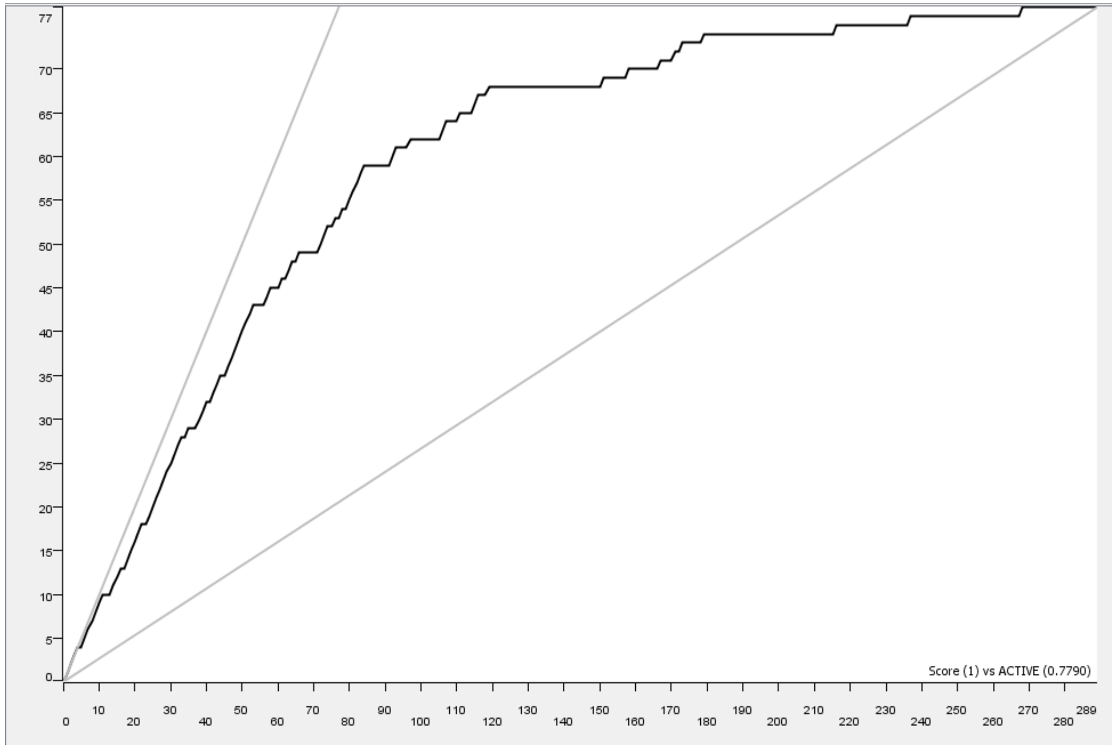

N=289

Top 10% Mean IC50	39.2 nM (one 800 nM compound)

	Predicted Active	Predicted Inactive
Active	52	25
Inactive	23	189

<15 nM DefGood in c-Abl, 40% error; Random seed = 429

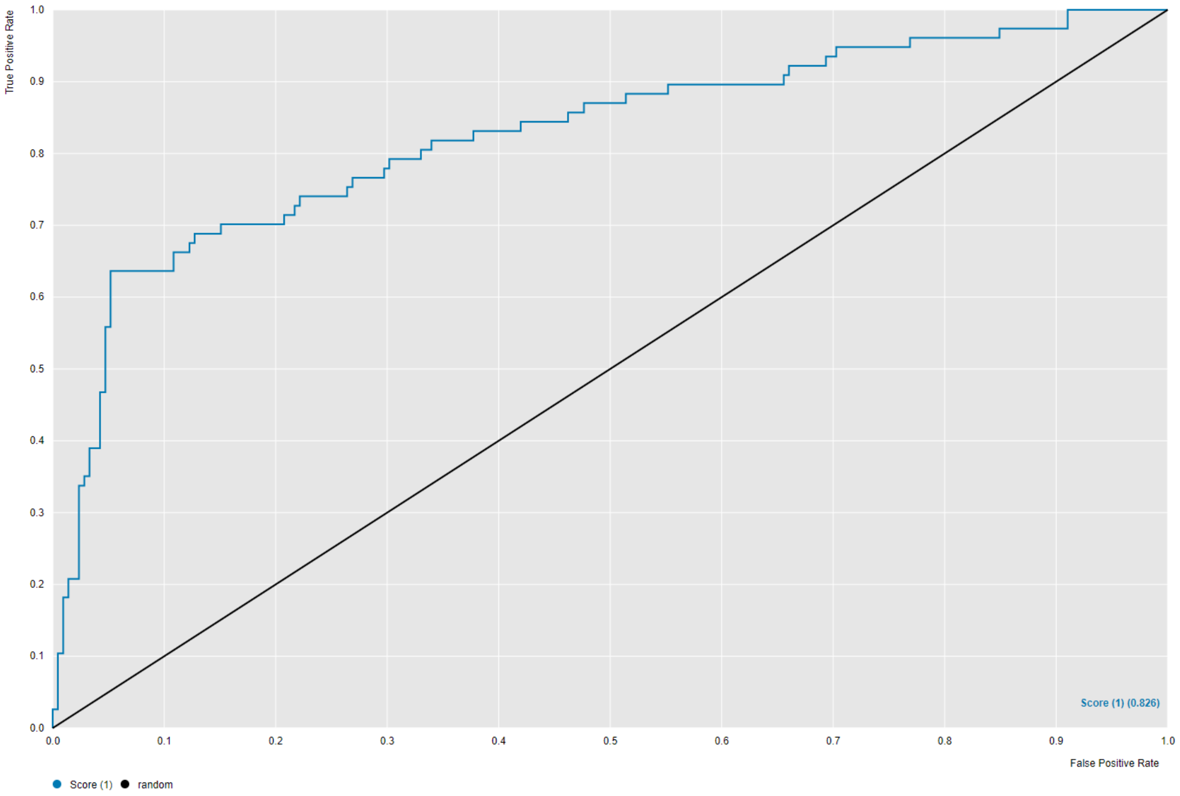

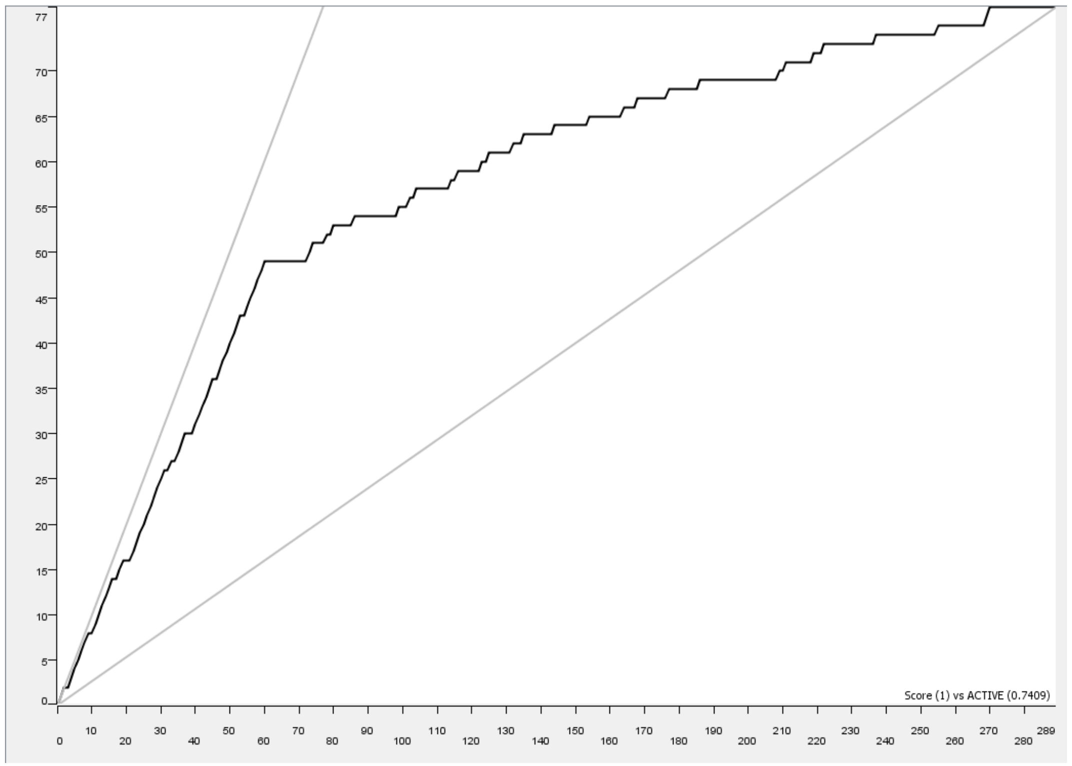

N=289

Top 10% Mean IC50	39.9 nM (one 800 nM compound)

	Predicted Active	Predicted Inactive
Active	39	38
Inactive	10	202

<15 nM DefGood in c-Abl, 45% error; Random seed = 429

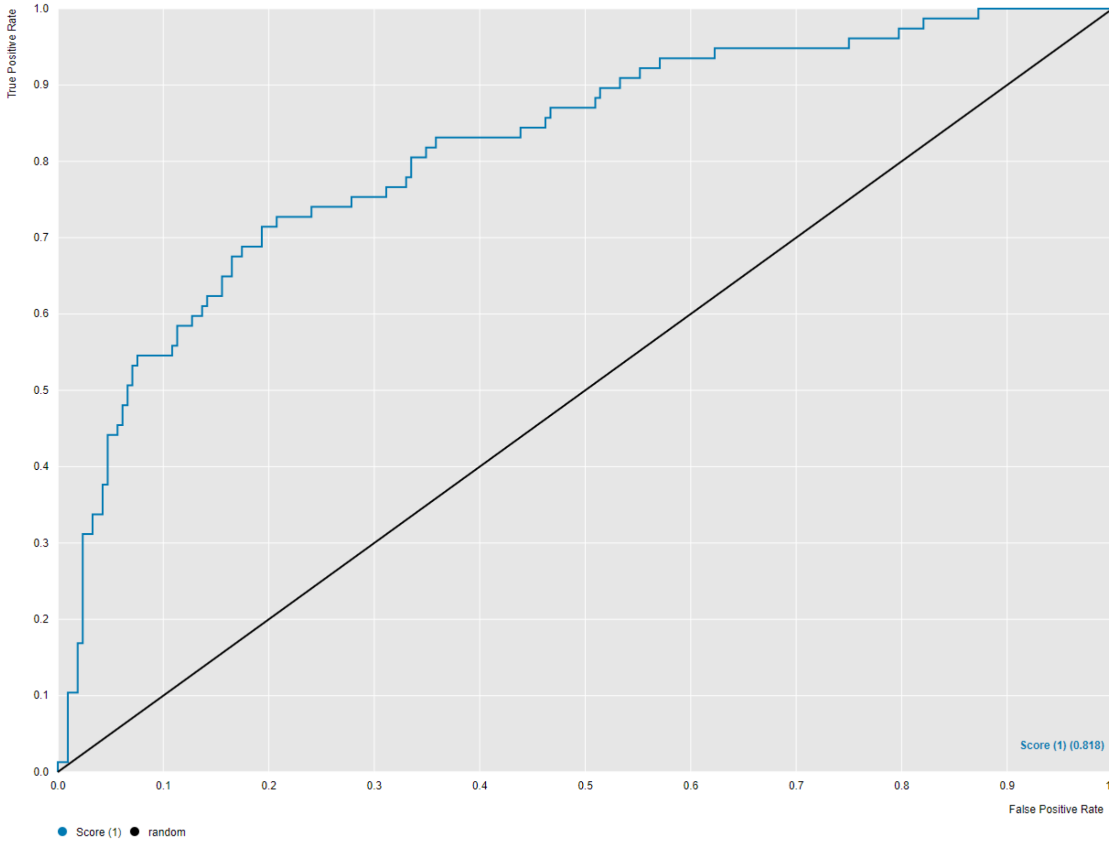

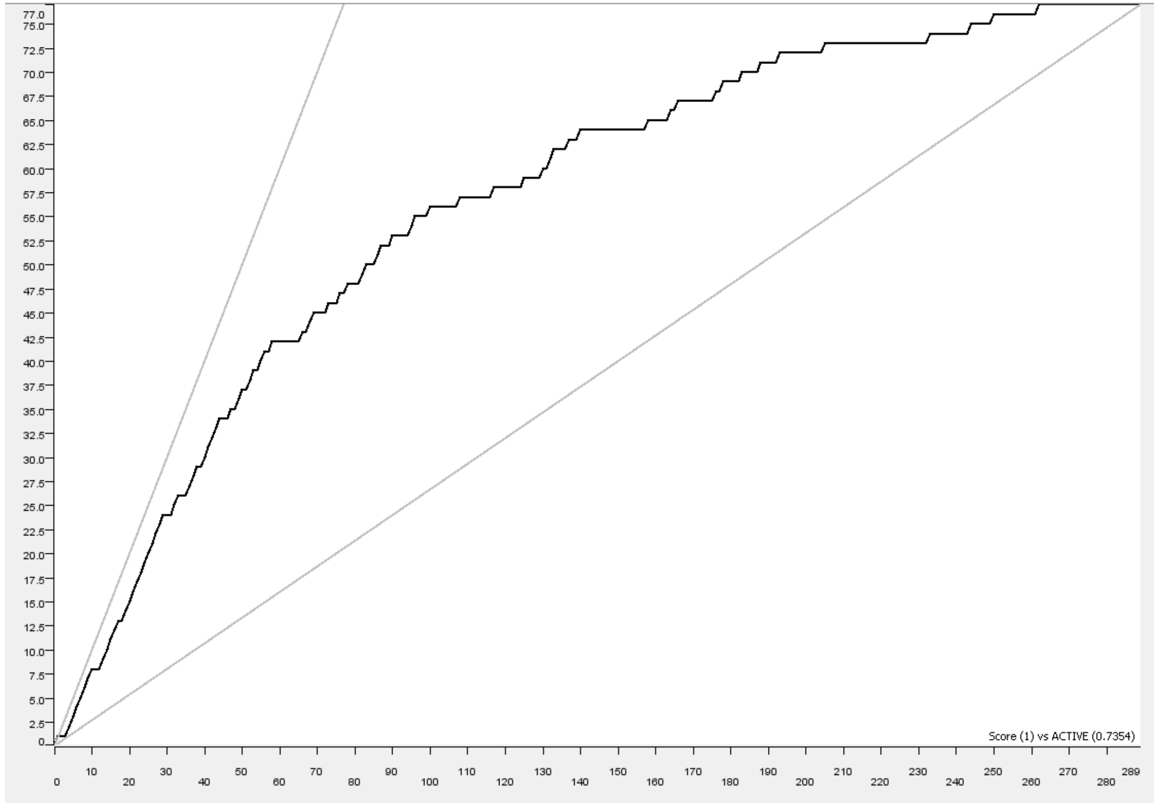

N=289

**Top 10% Mean IC50**	**13.5 nM (one 800 nM compound)**

	Predicted Active	Predicted Inactive
Active	20	57
Inactive	5	207

<15 nM DefGood in c-Abl, 50% error; Random seed = 429

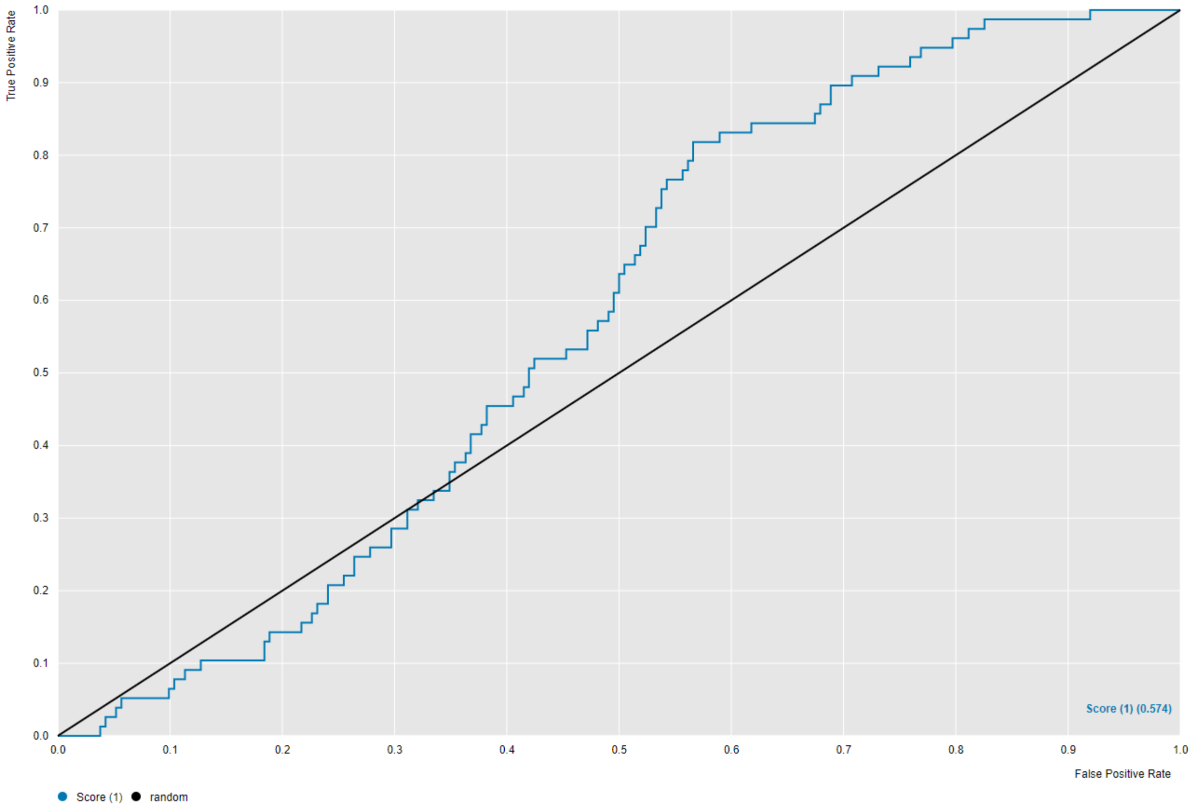

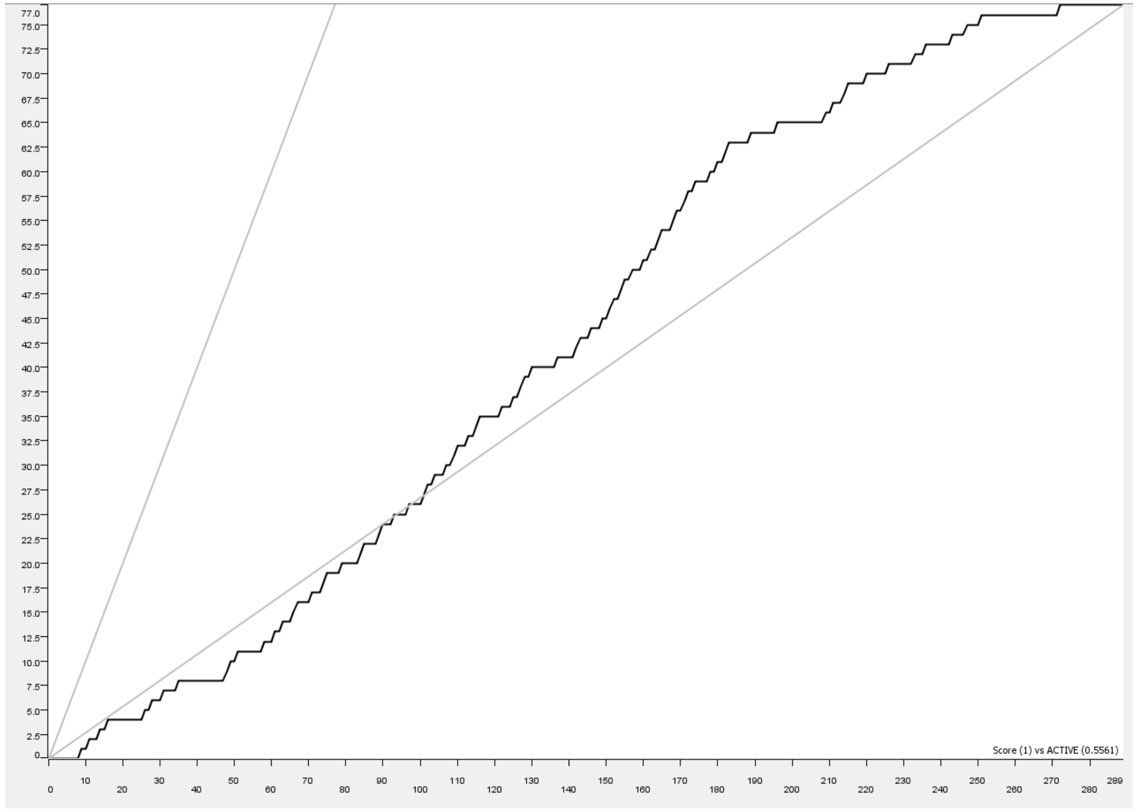

N=289

Top 10% Mean IC50	4,200 nM

	Predicted Active	Predicted Inactive
Active	77	0
Inactive	204	8

<15 nM DefGood in c-Abl, 10% error; Random seed = 121783

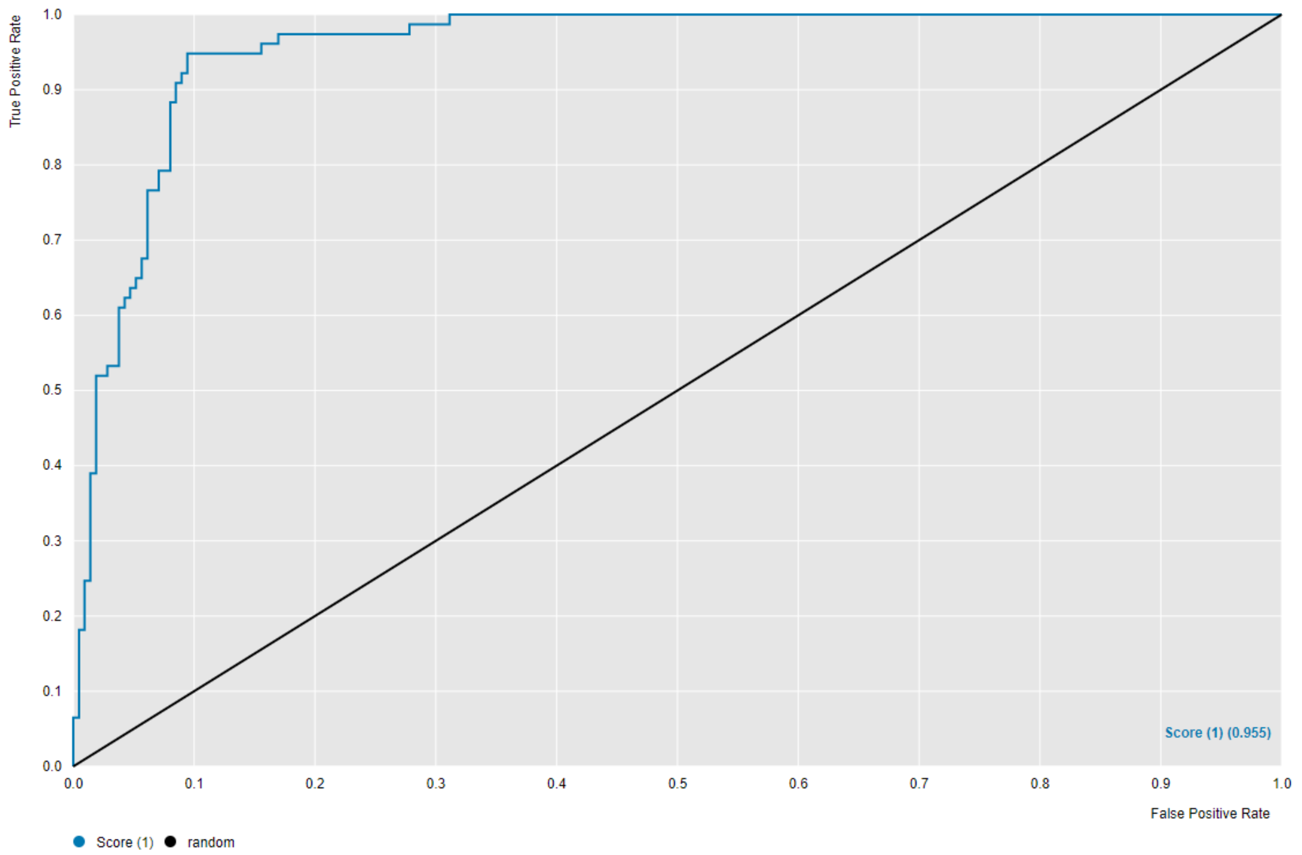

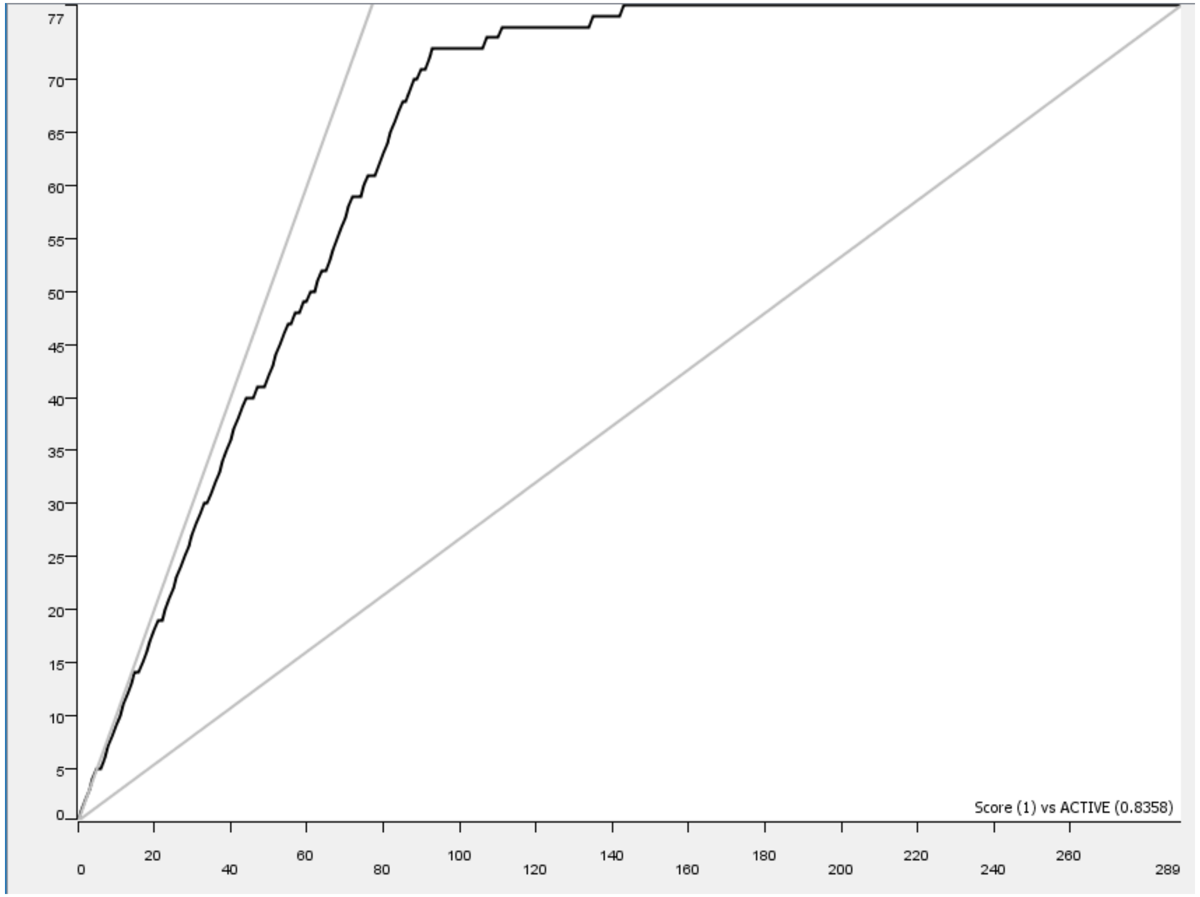

N=289

Top 10% Mean IC50	101.7 nM (3 > 800 nM compounds)

	Predicted Active	Predicted Inactive
Active	73	4
Inactive	27	185

<15 nM DefGood in c-Abl, 20% error; Random seed = 121783

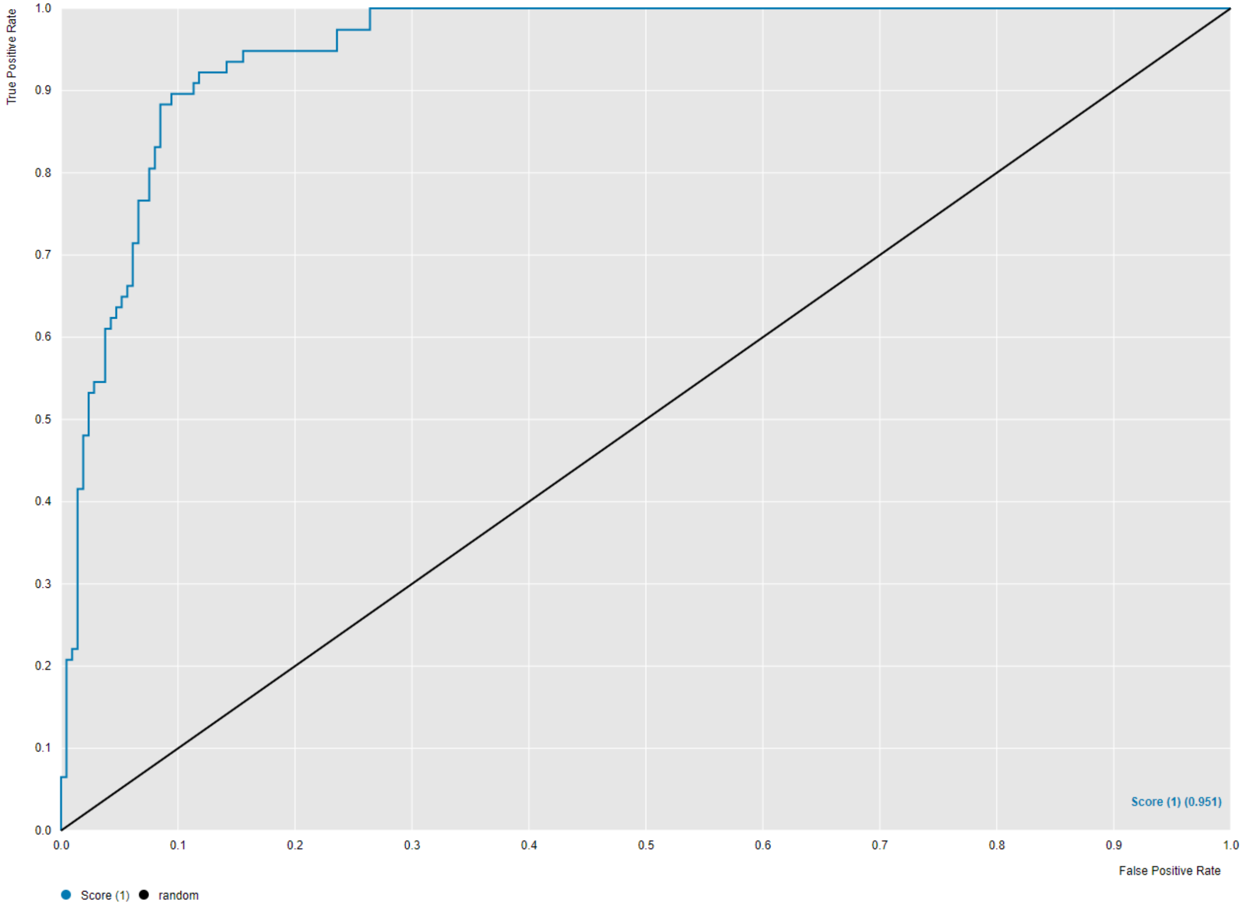

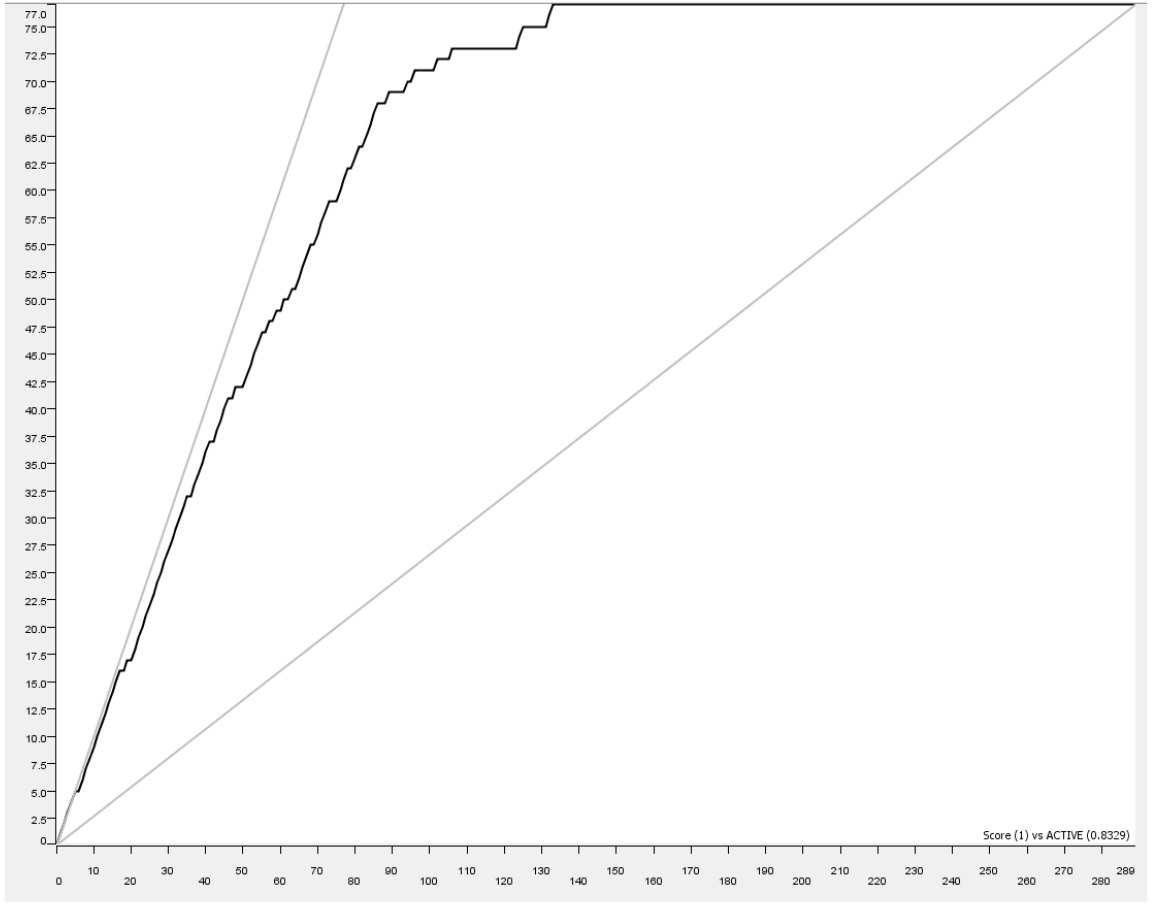

N=289

**Top 10% Mean IC50**	**101.8 nM (3 > 800 nM compounds)**

	Predicted Active	Predicted Inactive
Active	64	13
Inactive	17	195

<15 nM DefGood in c-Abl, 40% error; Random seed = 121783

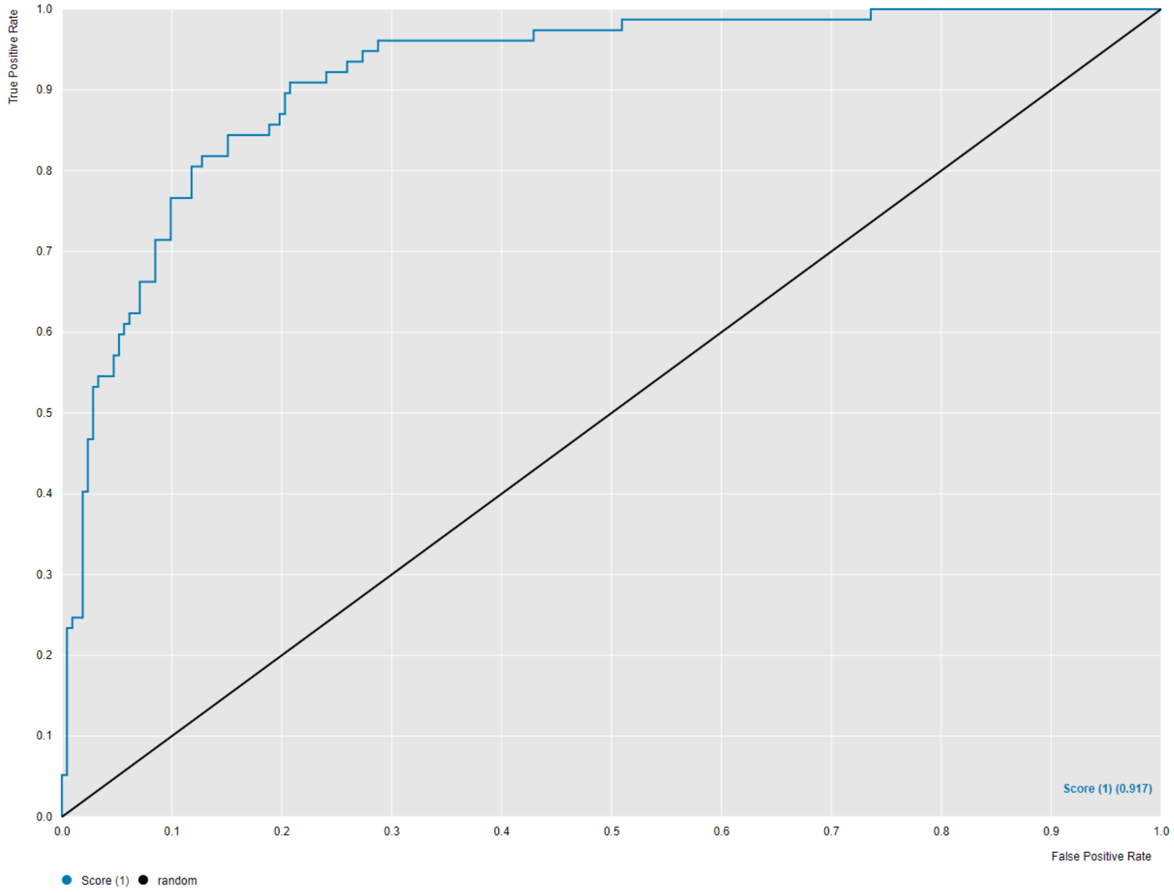

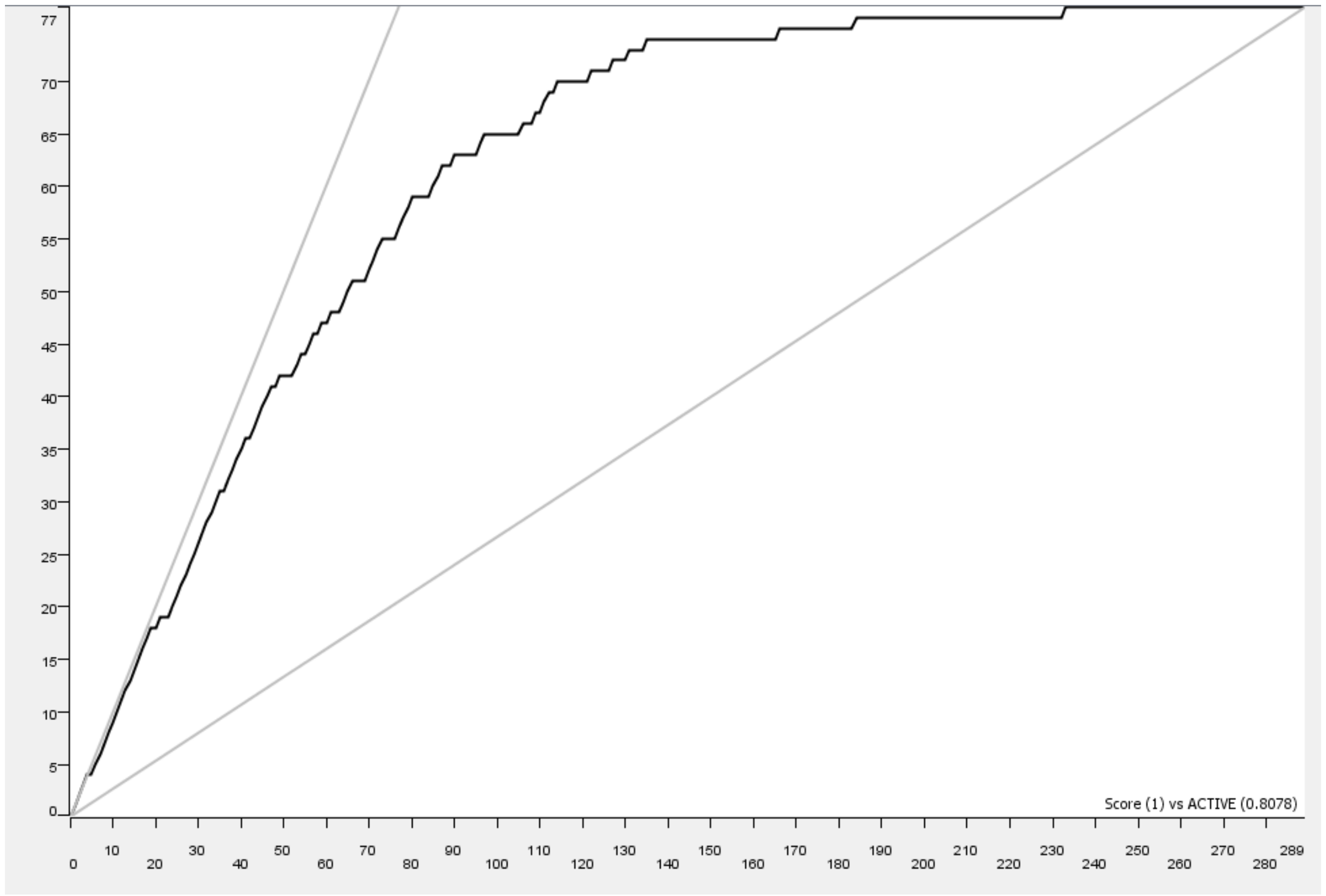

N=289

**Top 10% Mean IC50**	**112.3 nM (3 > 800 nM compounds)**

	Predicted Active	Predicted Inactive
Active	59	18
Inactive	23	189

<15 nM DefGood in c-Abl, 45% error; Random seed = 121783

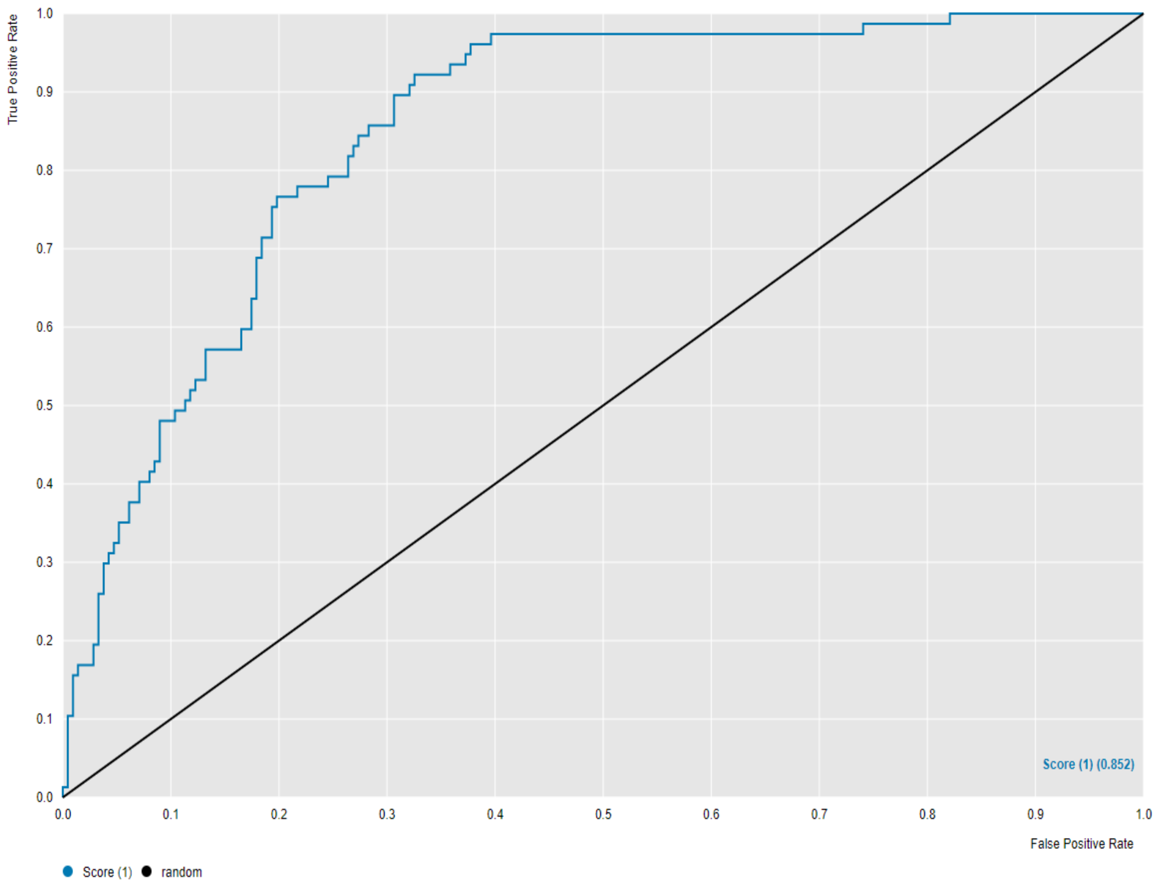

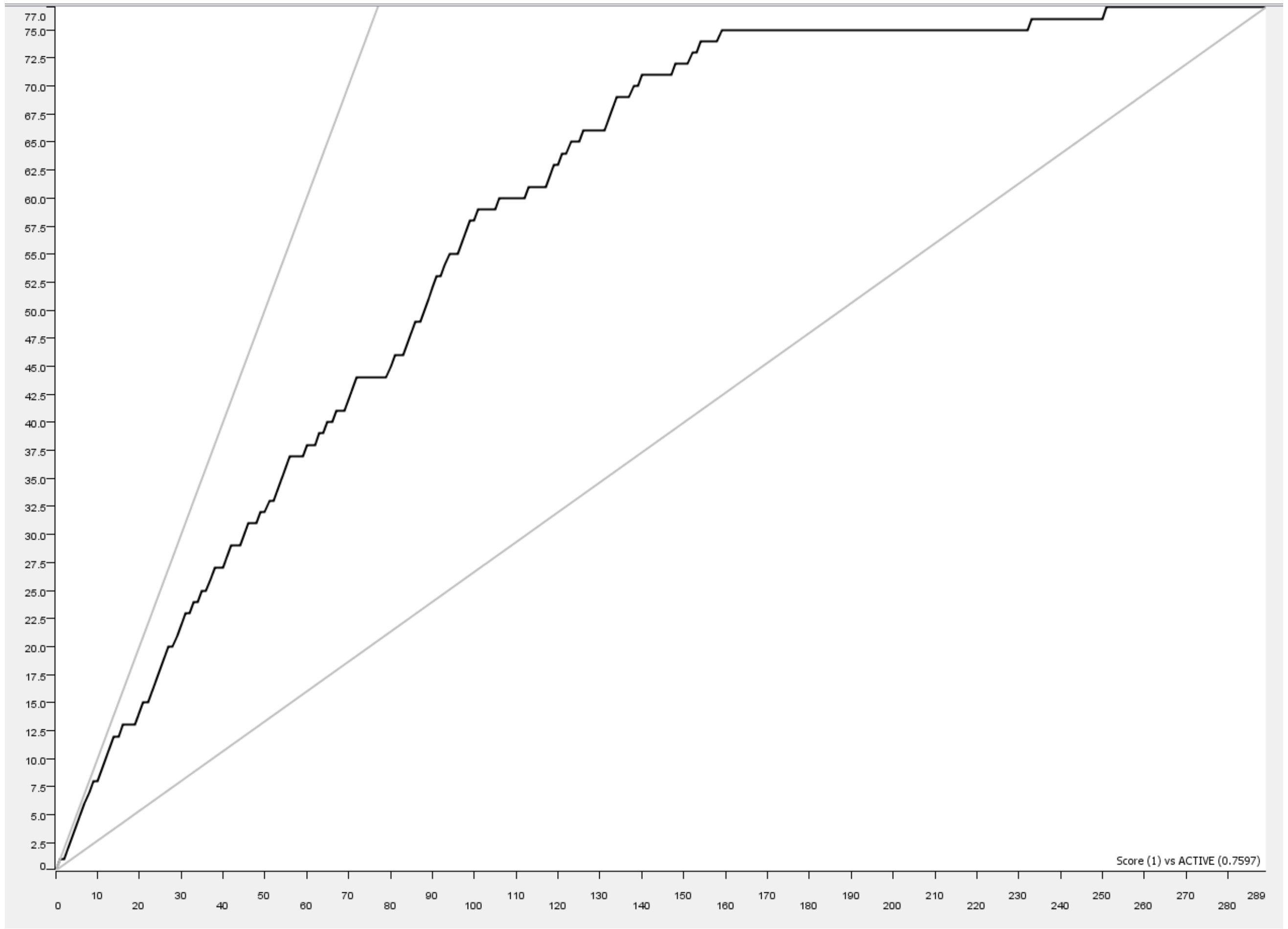

N=289

Top 10% Mean IC50	4,900 nM

	Predicted Active	Predicted Inactive
Active	66	11
Inactive	61	151

<15 nM DefGood in c-Abl, 50% error; Random seed = 121783

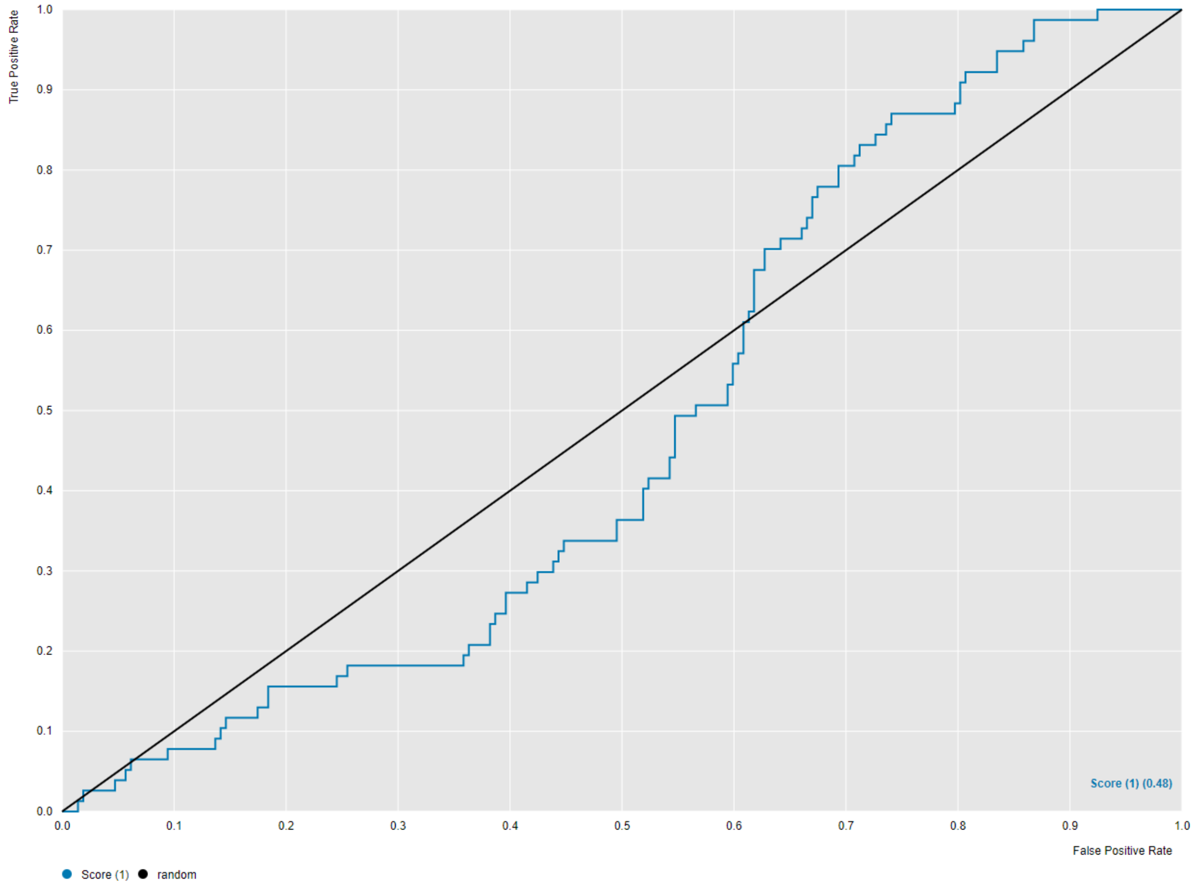

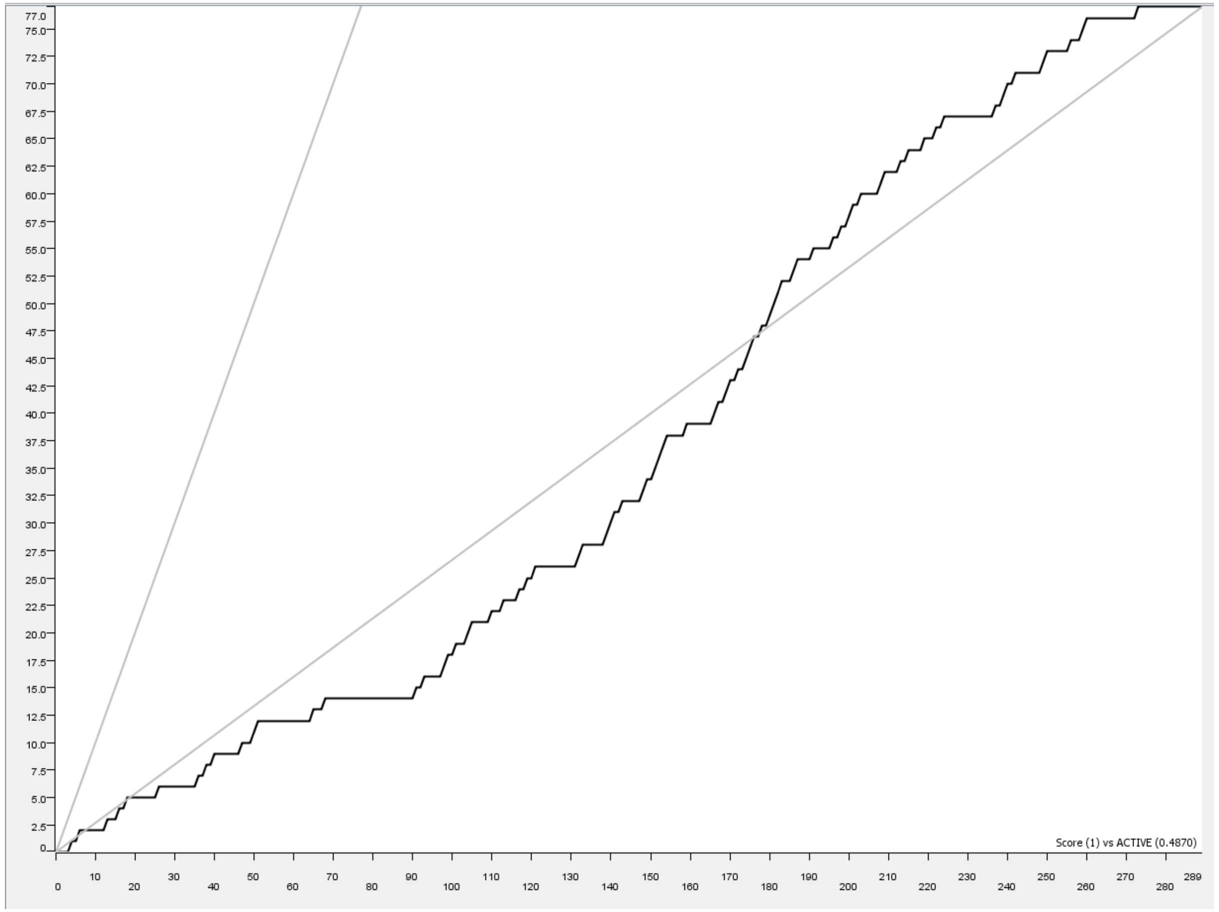

N=289

Top 10% Mean IC50	15,000 nM

	Predicted Active	Predicted Inactive
Active	77	0
Inactive	207	5

Conclusion - NBN

- Across three random seeds, the % error that lead to a significant enrichment failure and top 10% IC50 increase in the retrospective test was 45%, 50% and 45% for each unique random seed.
- An NBN can be very tolerant of data error in the c-Abl space.

RF - <15 nM DefGood in c-Abl, 5% error ; Random seed = 1515533876005

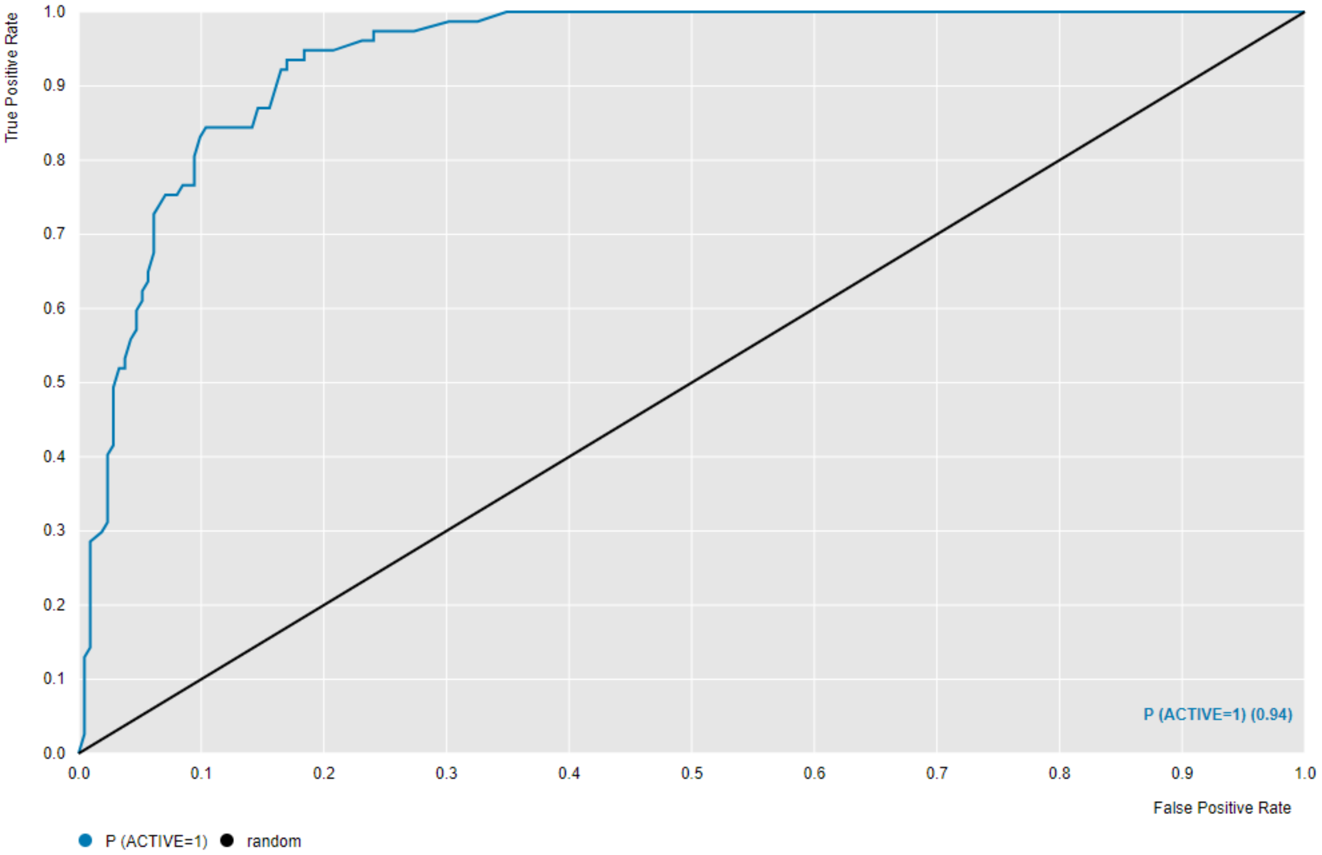

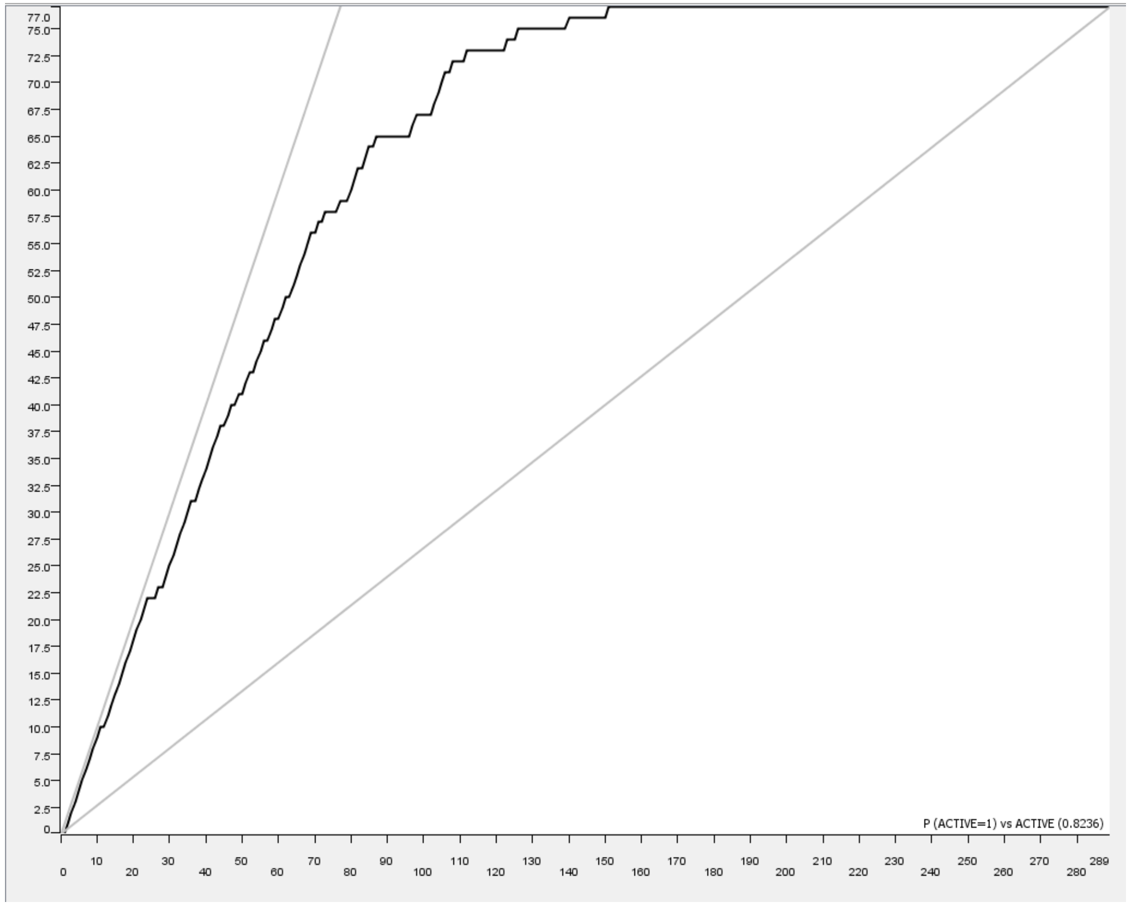

N=289

Top 10% Mean IC50	45.7 nM (one 800 nM compound)

	Predicted Active	Predicted Inactive
Active	59	18
Inactive	19	193

RF - <15 nM DefGood in c-Abl, 10% error

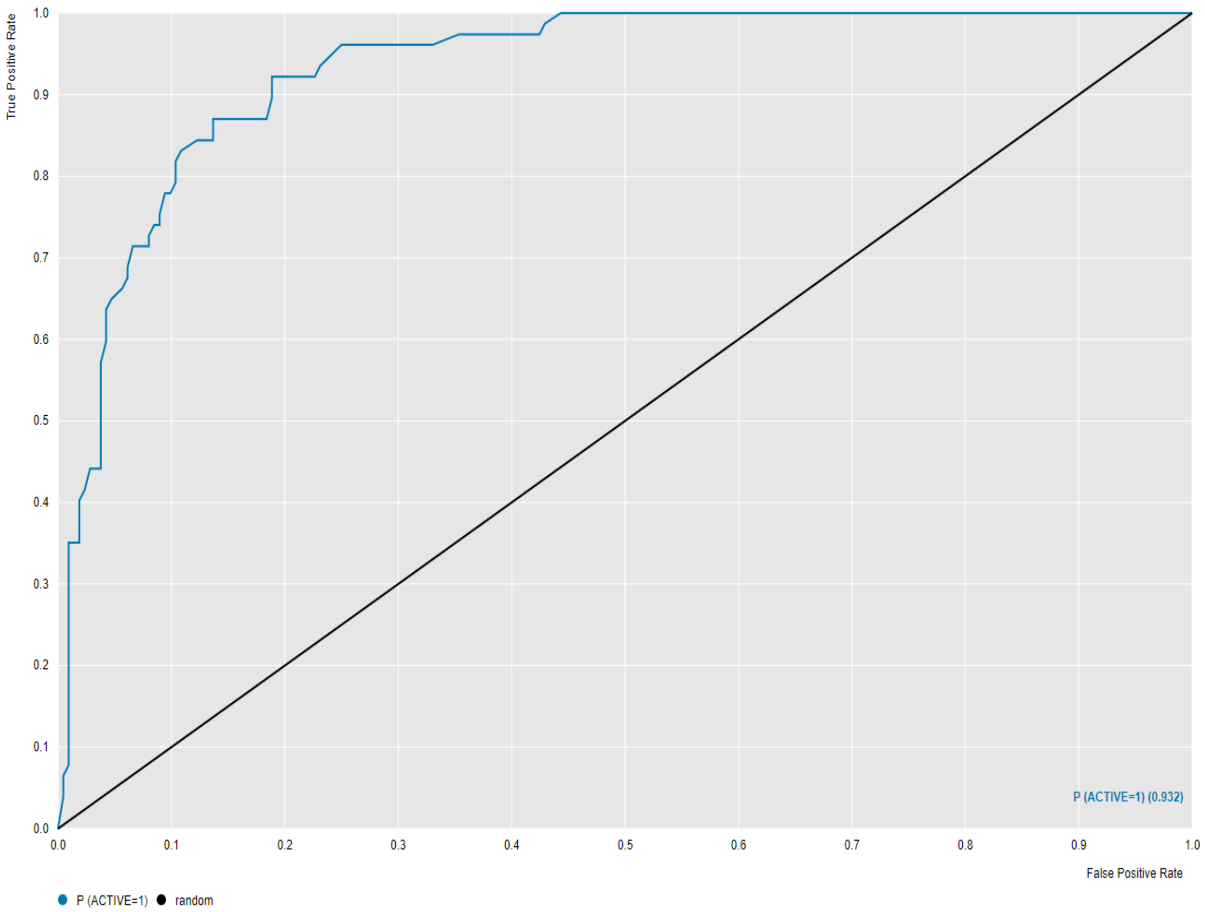

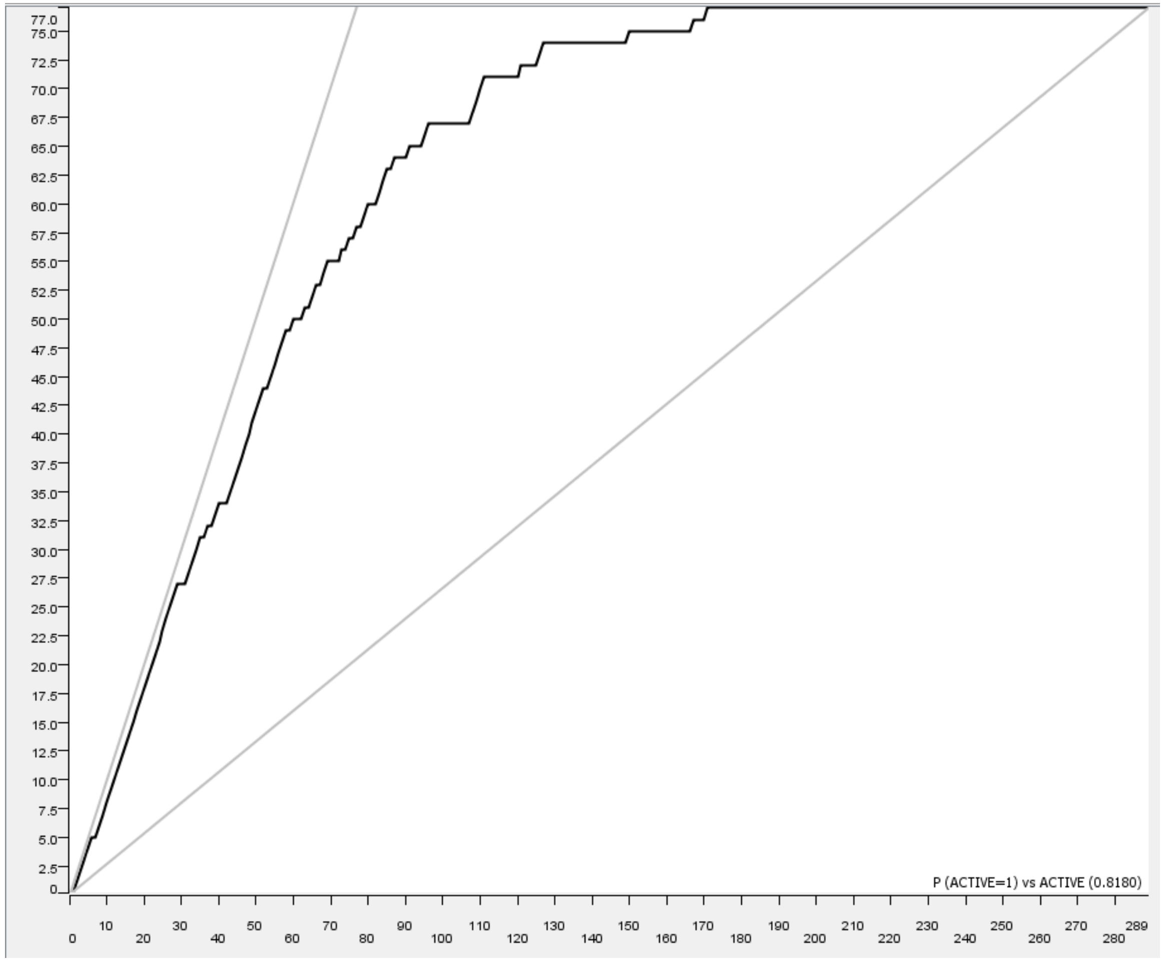

N=289

Top 10% Mean IC50	34.9 nM (one 800 nM compound)

	Predicted Active	Predicted Inactive
Active	60	17
Inactive	20	192

RF - <15 nM DefGood in c-Abl, 15% error

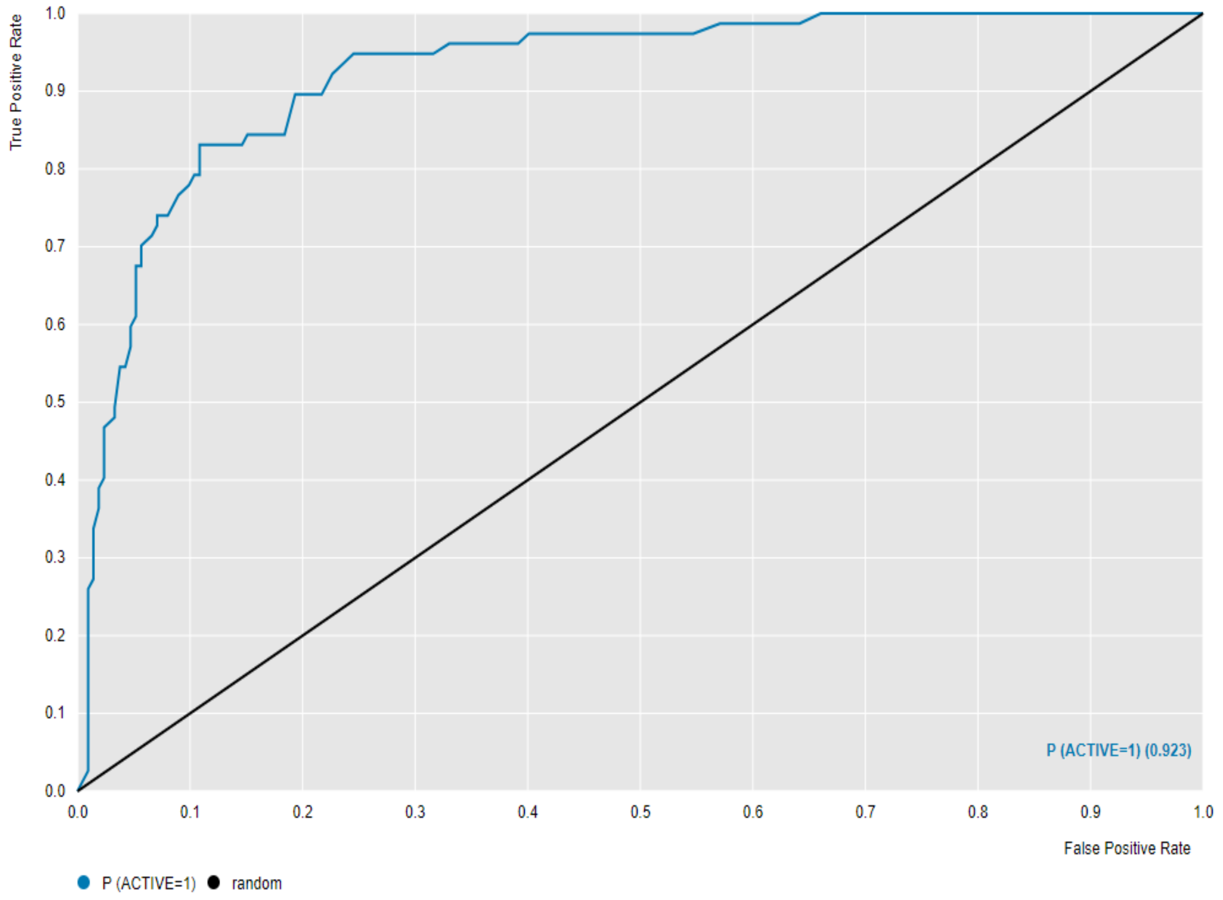

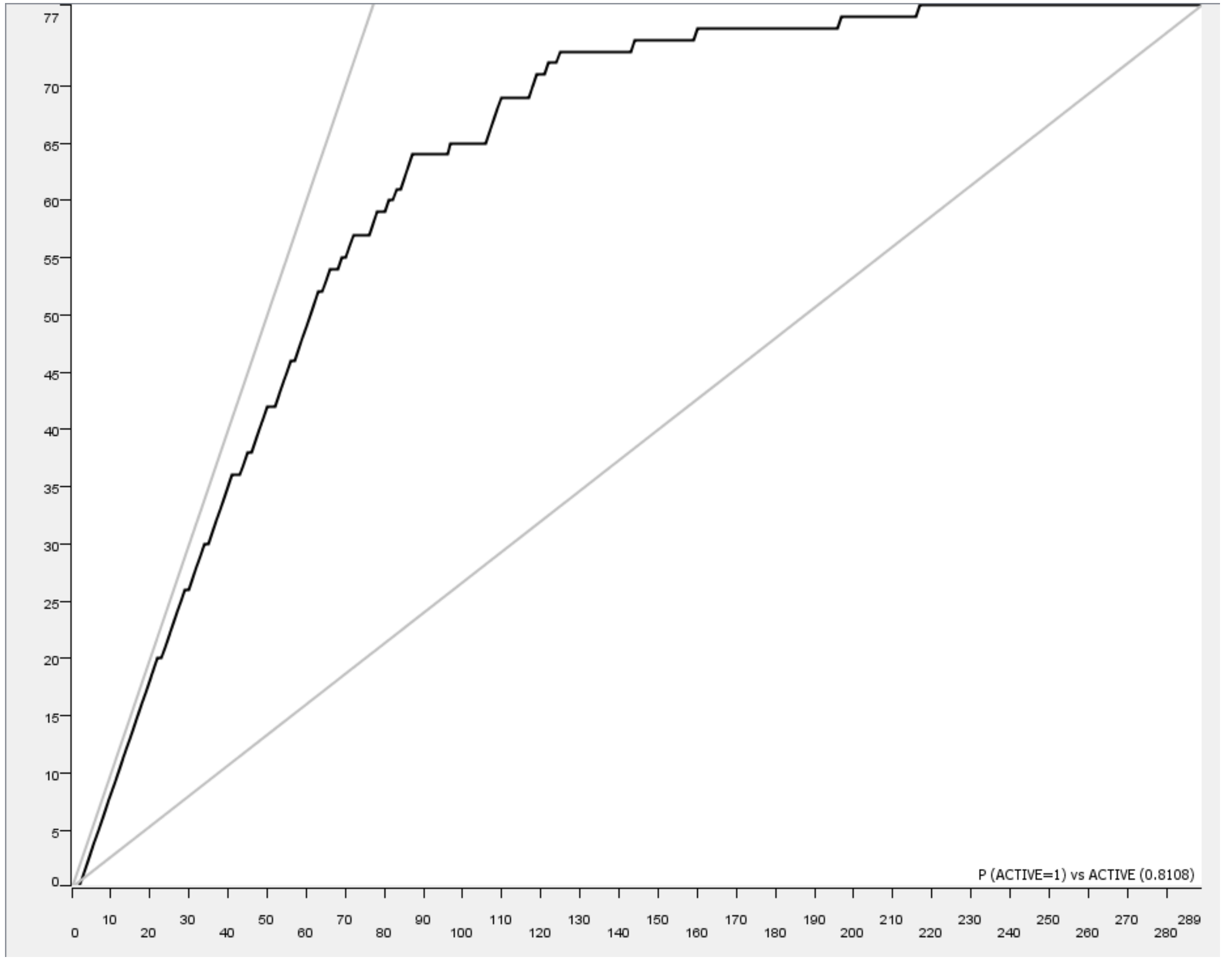

N=289

**Top 10% Mean IC50**	**35.9 nM (one 800 nM compound)**

	Predicted Active	Predicted Inactive
Active	61	16
Inactive	22	190

RF - <15 nM DefGood in c-Abl, 20% error

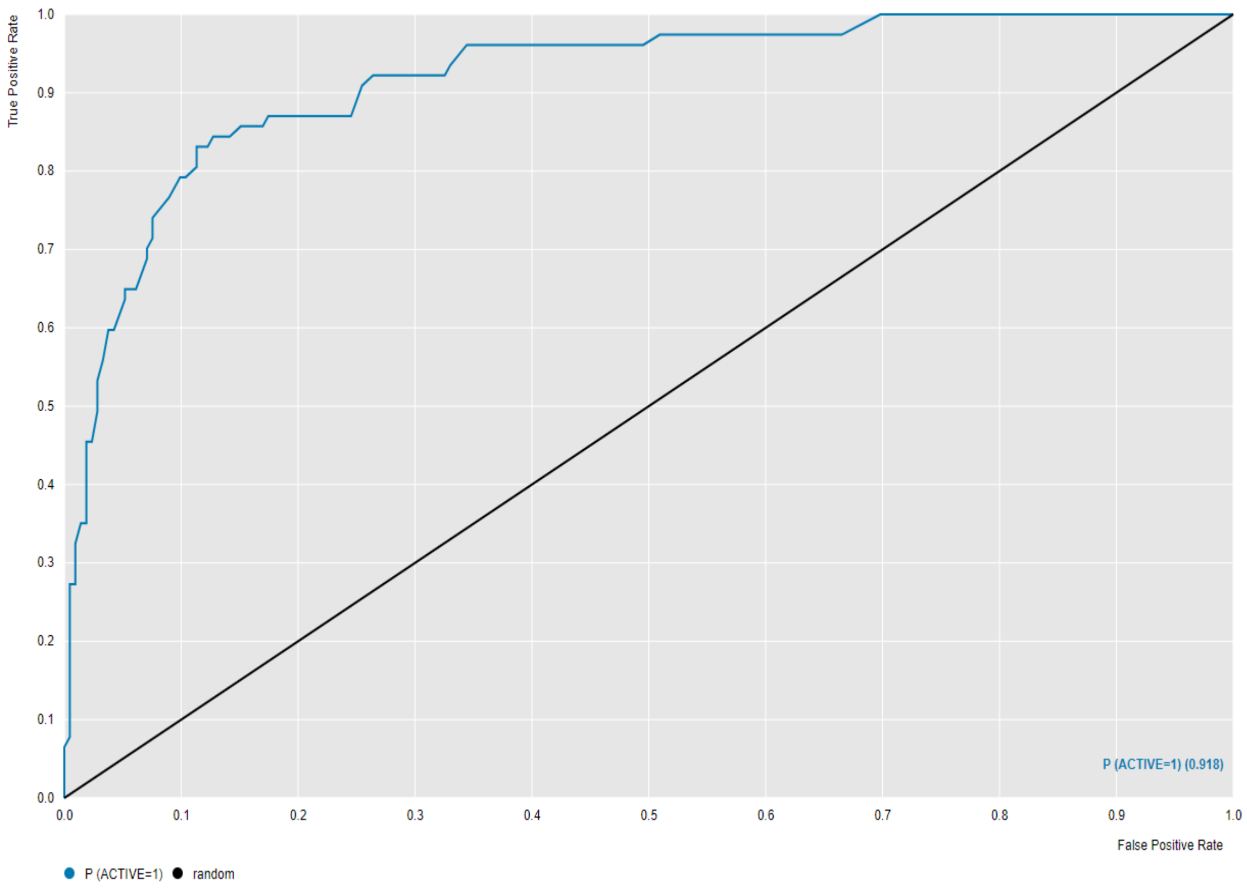

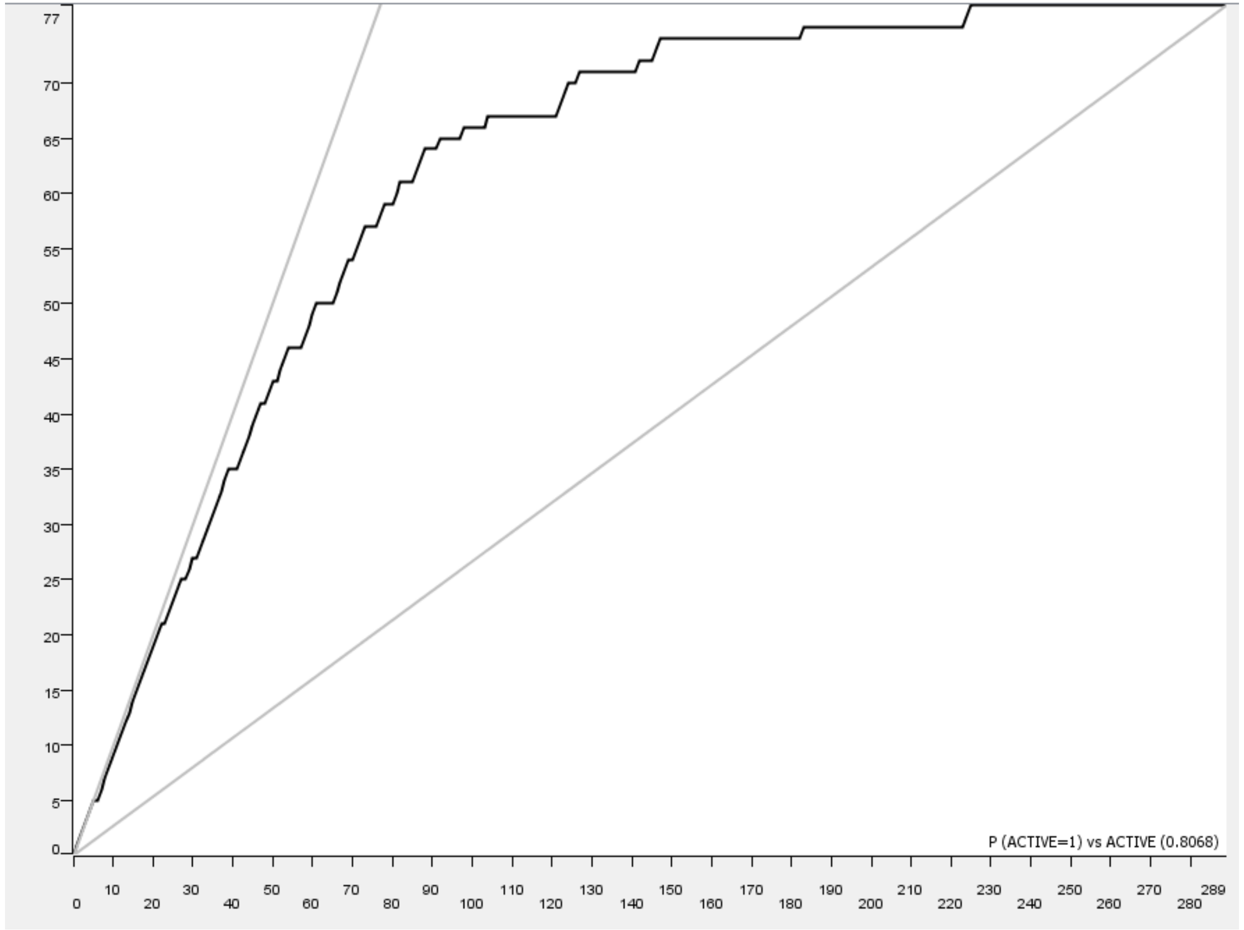

N=289

Top 10% Mean IC50	140 nM (one 800 nM compound, and one 3 uM compound)

	Predicted Active	Predicted Inactive
Active	65	12
Inactive	27	185

RF - <15 nM DefGood in c-Abl, 30% error

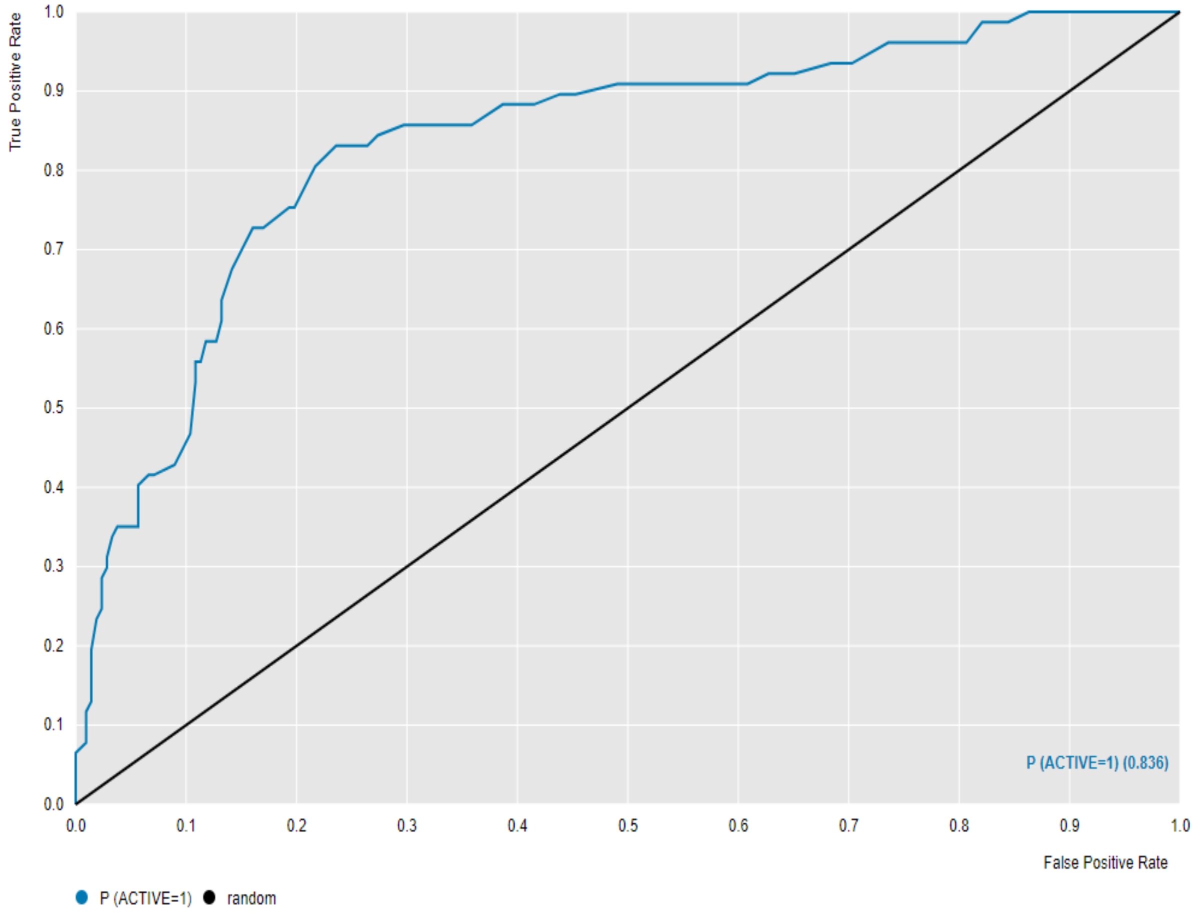

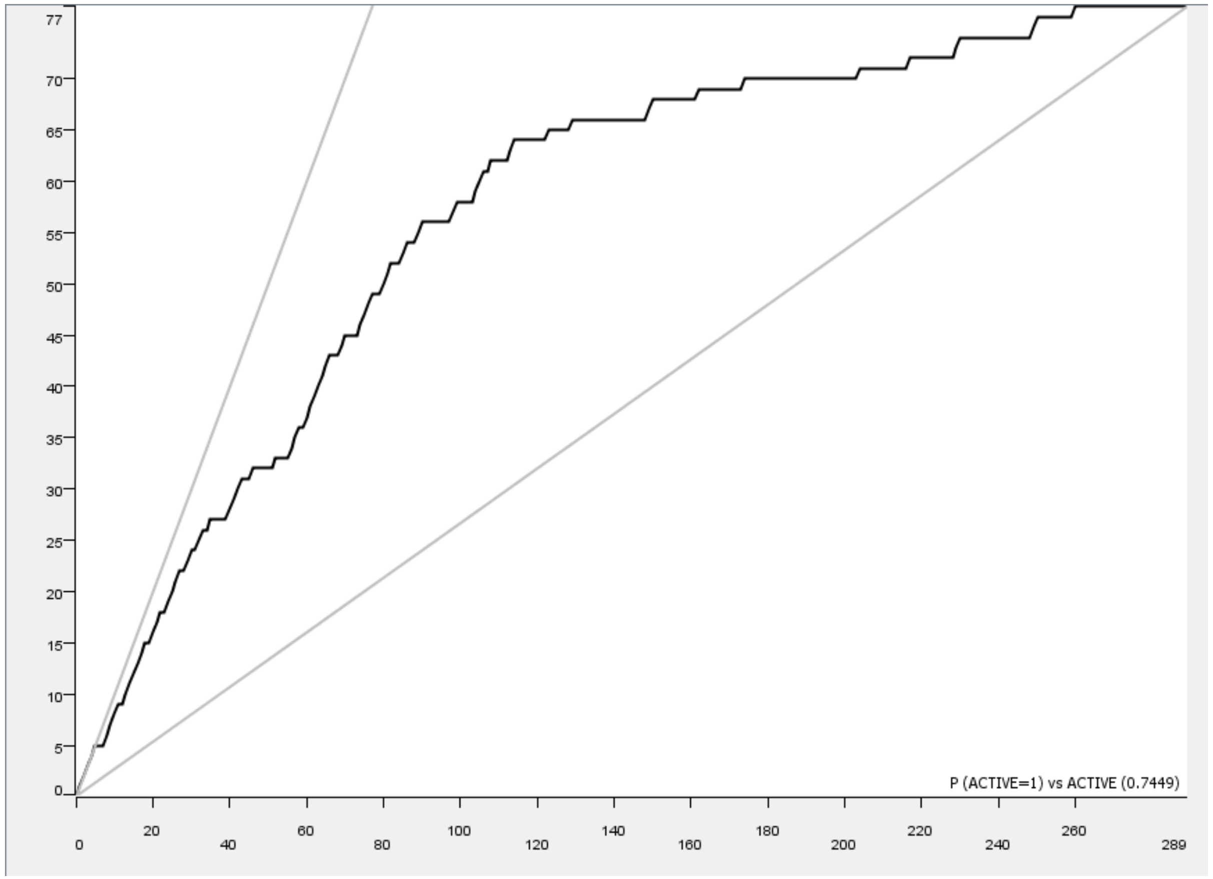

N=289

Top 10% Mean IC50	140 nM<br>(one 800 nM compound, and one 3 uM compound)

	Predicted Active	Predicted Inactive
Active	56	21
Inactive	34	178

RF - <15 nM DefGood in c-Abl, 35% error

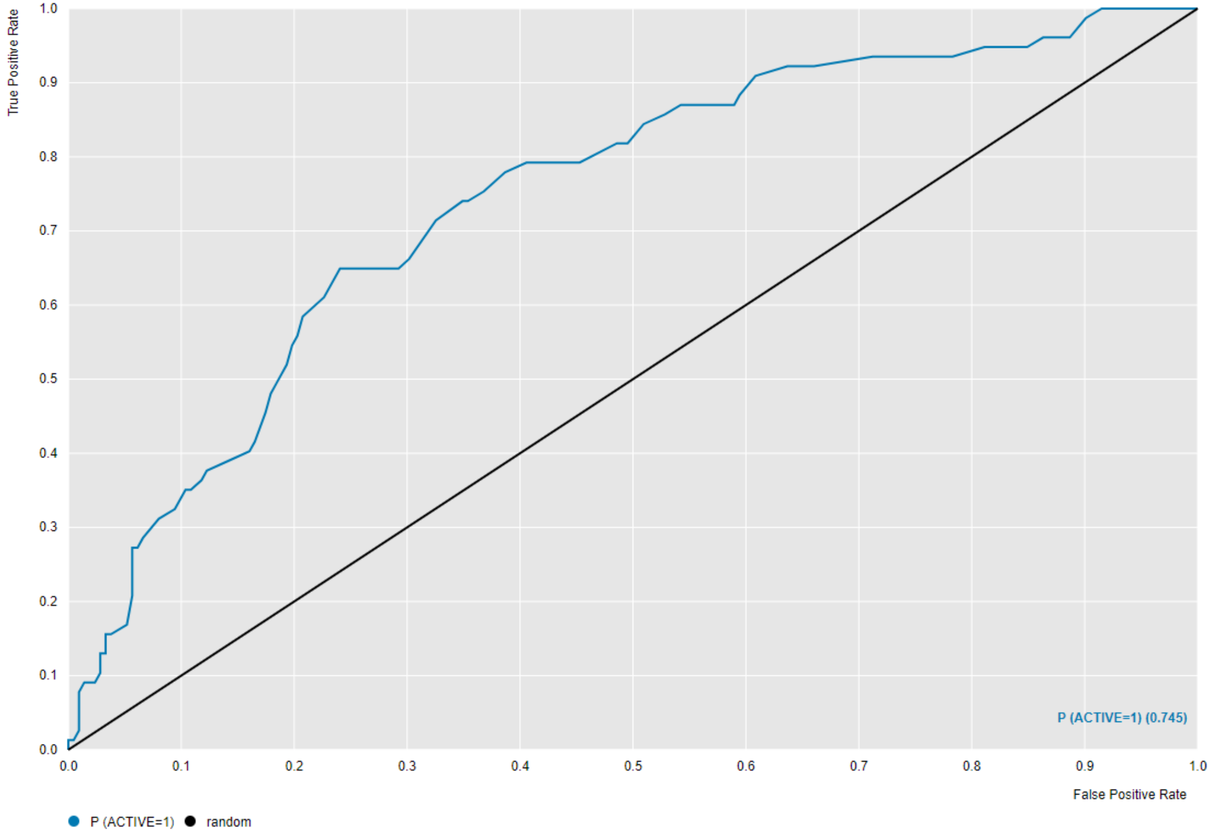

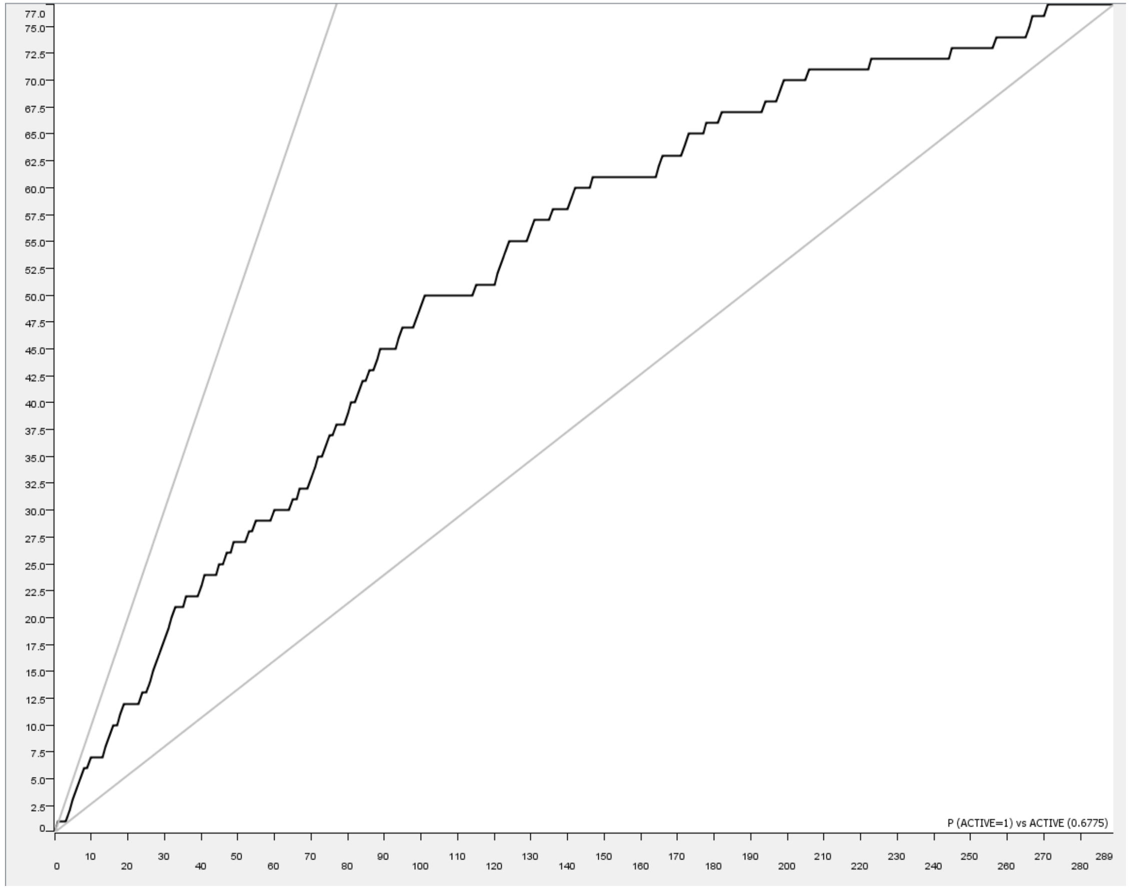

N=289

Top 10% Mean IC50	390 nM<br>(many micromolar errors)

	Predicted Active	Predicted Inactive
Active	45	32
Inactive	44	168

RF - <15 nM DefGood in c-Abl, 40% error

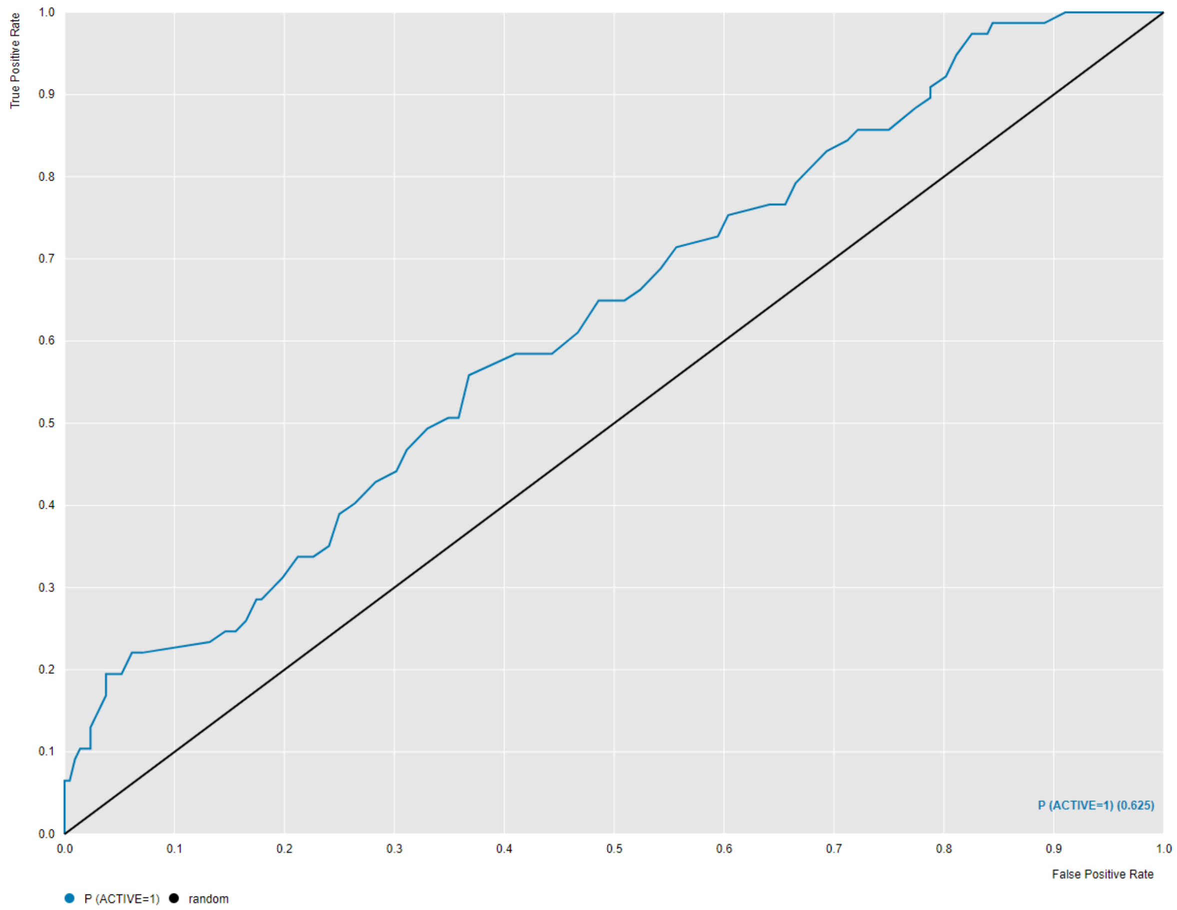

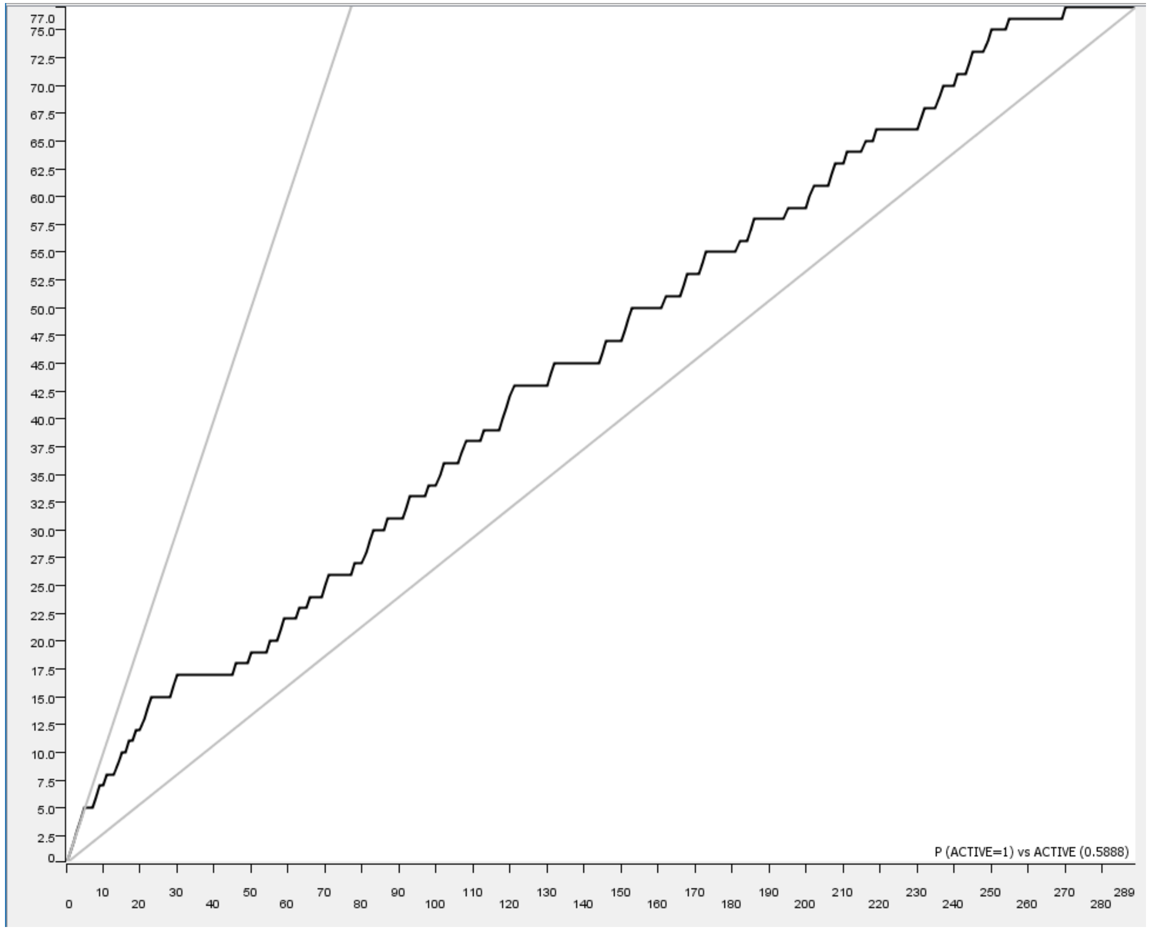

N=289

Top 10% Mean IC50	3,800 nM

	Predicted Active	Predicted Inactive
Active	38	39
Inactive	70	142

RF - <15 nM DefGood in c-Abl, 10% error; Random Seed = 429

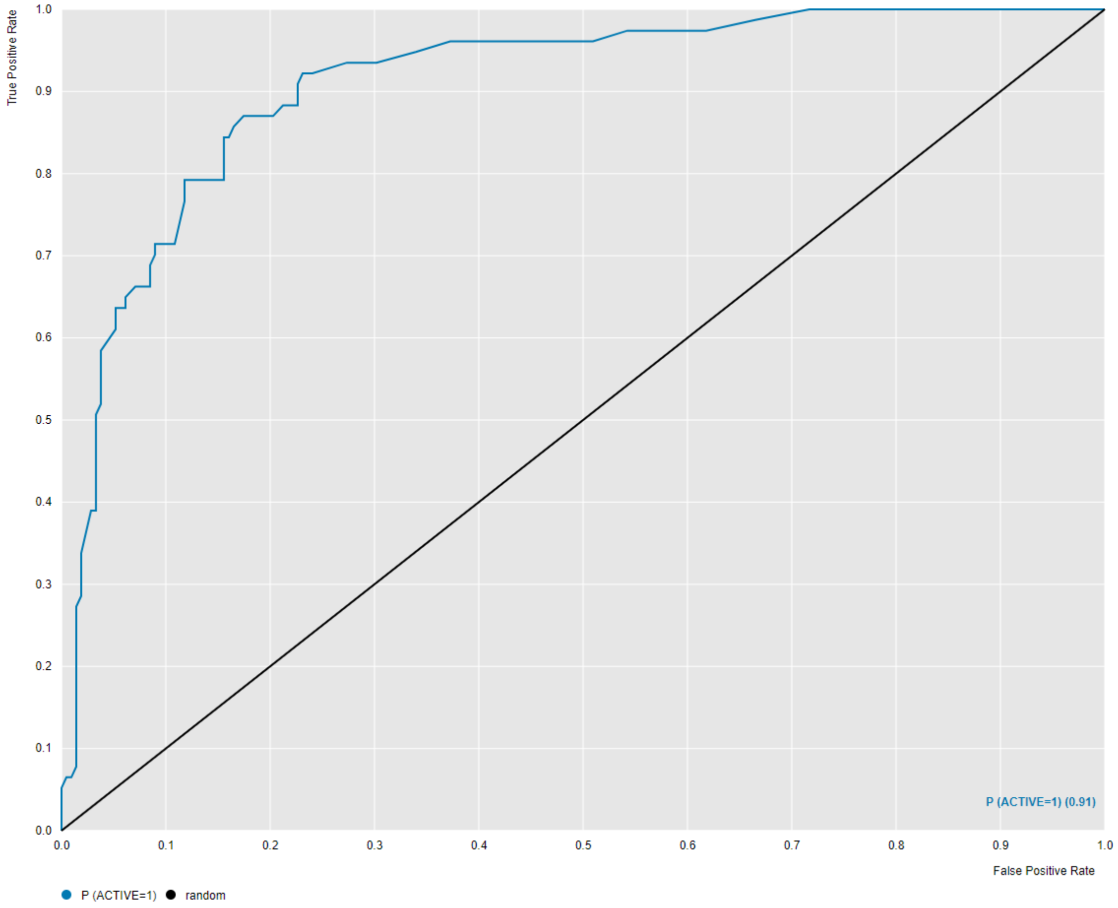

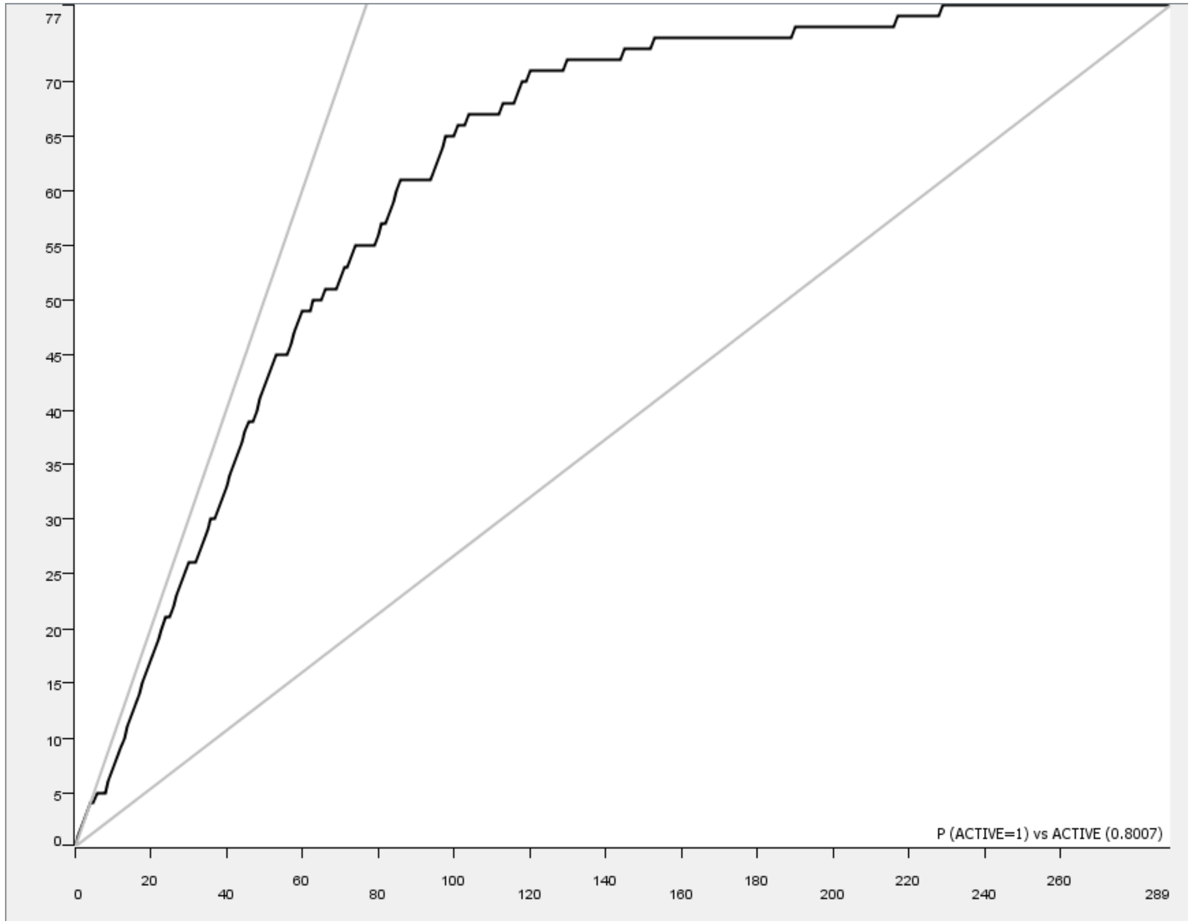

N=289

**Top 10% Mean IC50**	**39.6 nM (one 800 nM compound)**

	**Predicted Active**	**Predicted Inactive**
Active	52	25
Inactive	18	194

RF - <15 nM DefGood in c-Abl, 20% error; Random Seed = 429

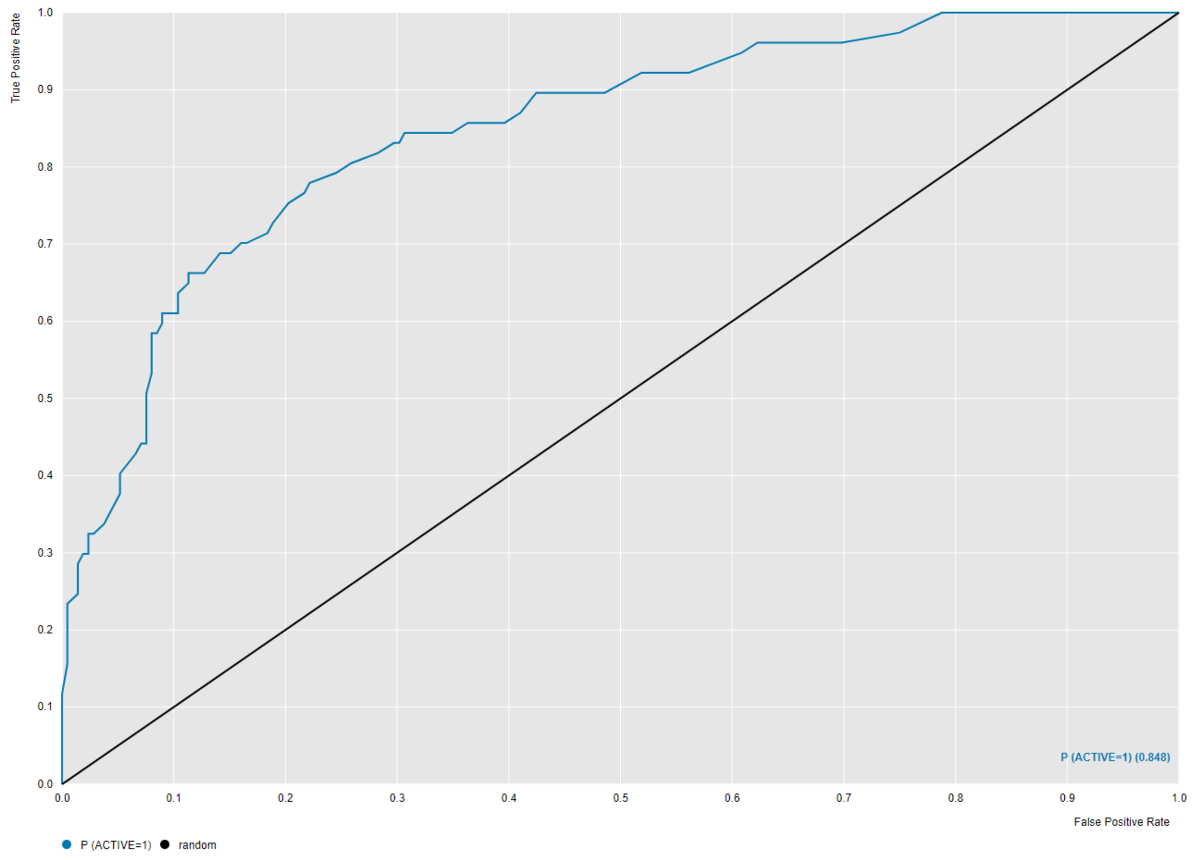

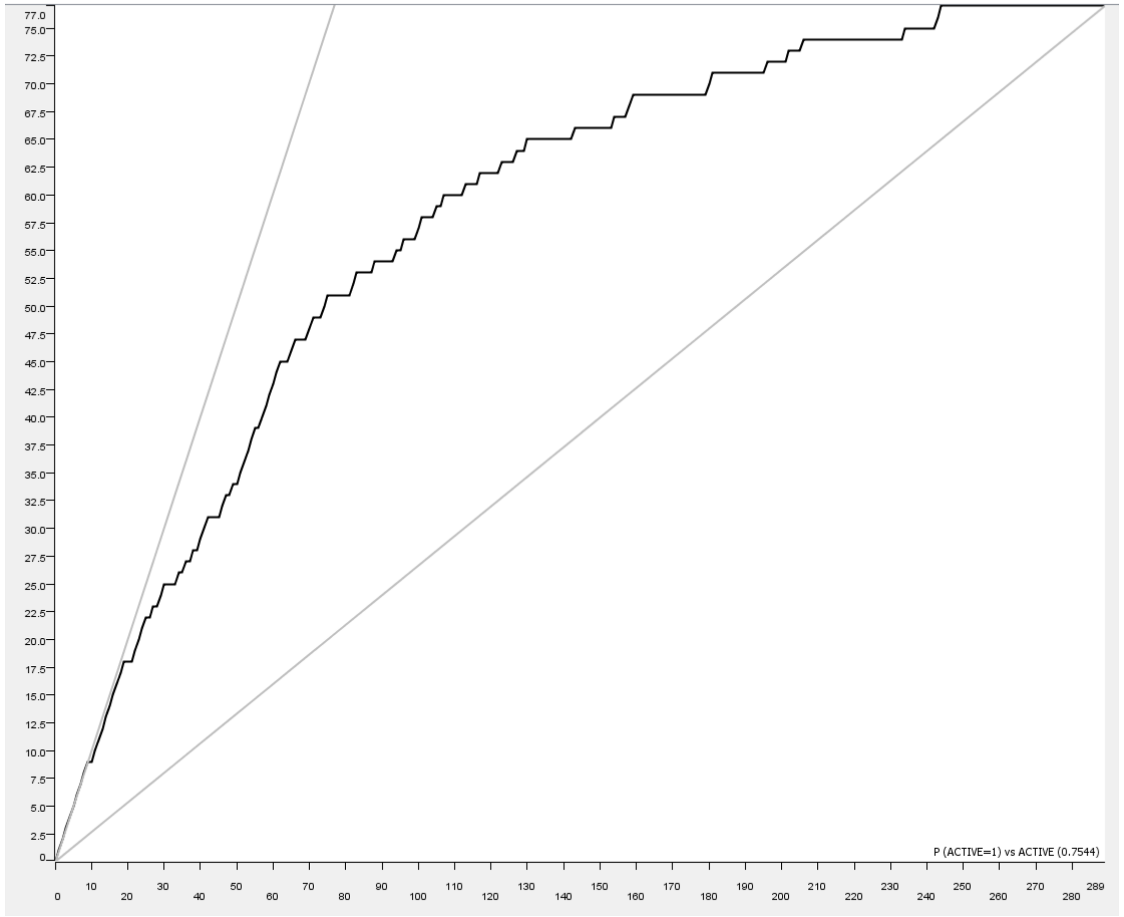

N=289

Top 10% Mean IC50	11.5 nM

	Predicted Active	Predicted Inactive
Active	51	26
Inactive	24	188

RF - <15 nM DefGood in c-Abl, 25% error; Random Seed = 429

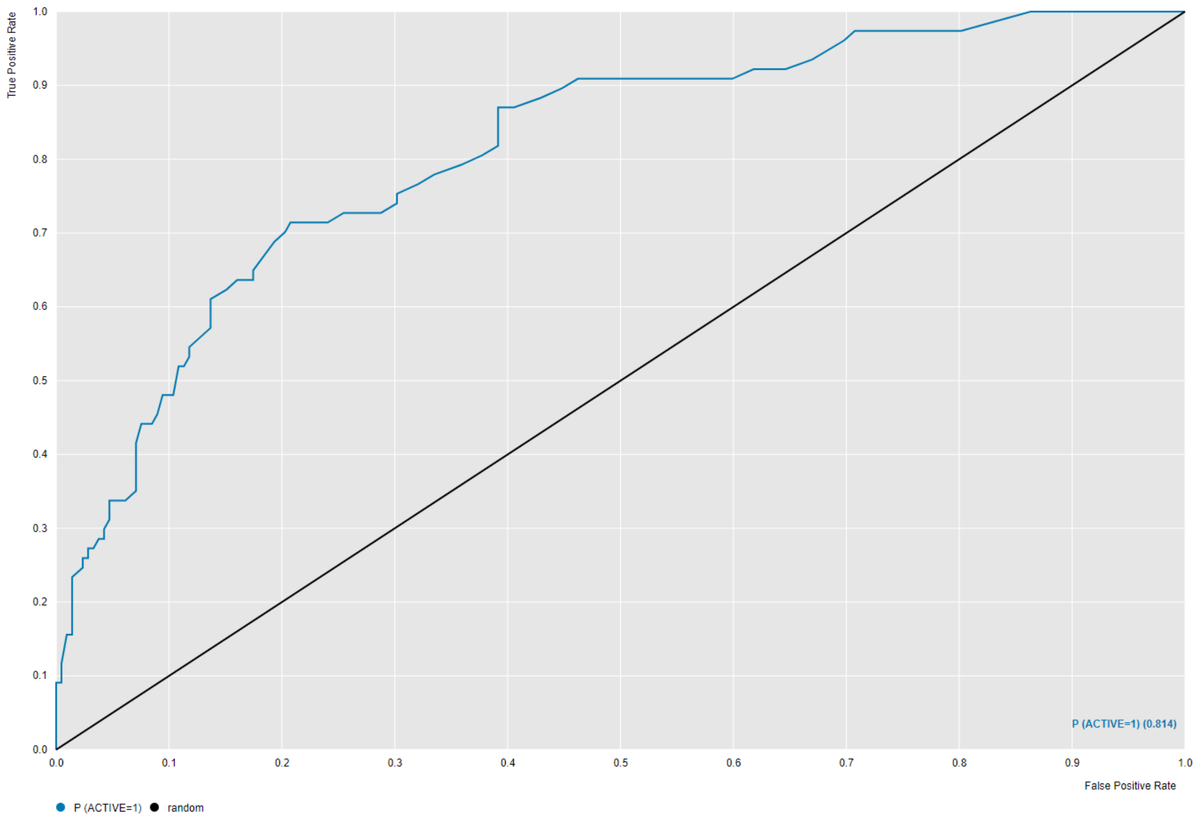

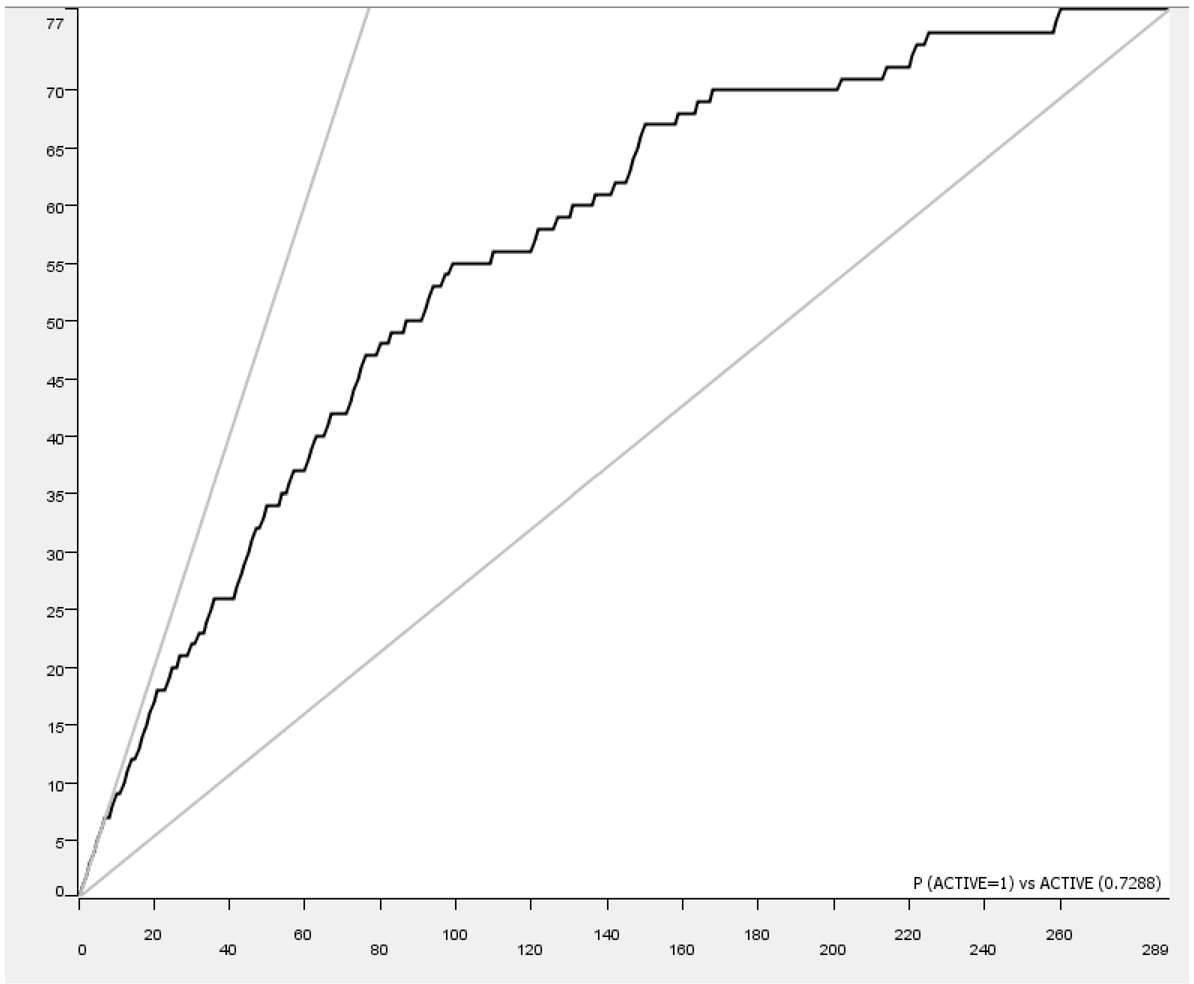

N=289

**Top 10% Mean IC50**	**2,400 nM (one 65 uM mistake)**

	Predicted Active	Predicted Inactive
Active	47	30
Inactive	29	183

RF - <15 nM DefGood in c-Abl, 30% error; Random Seed = 429

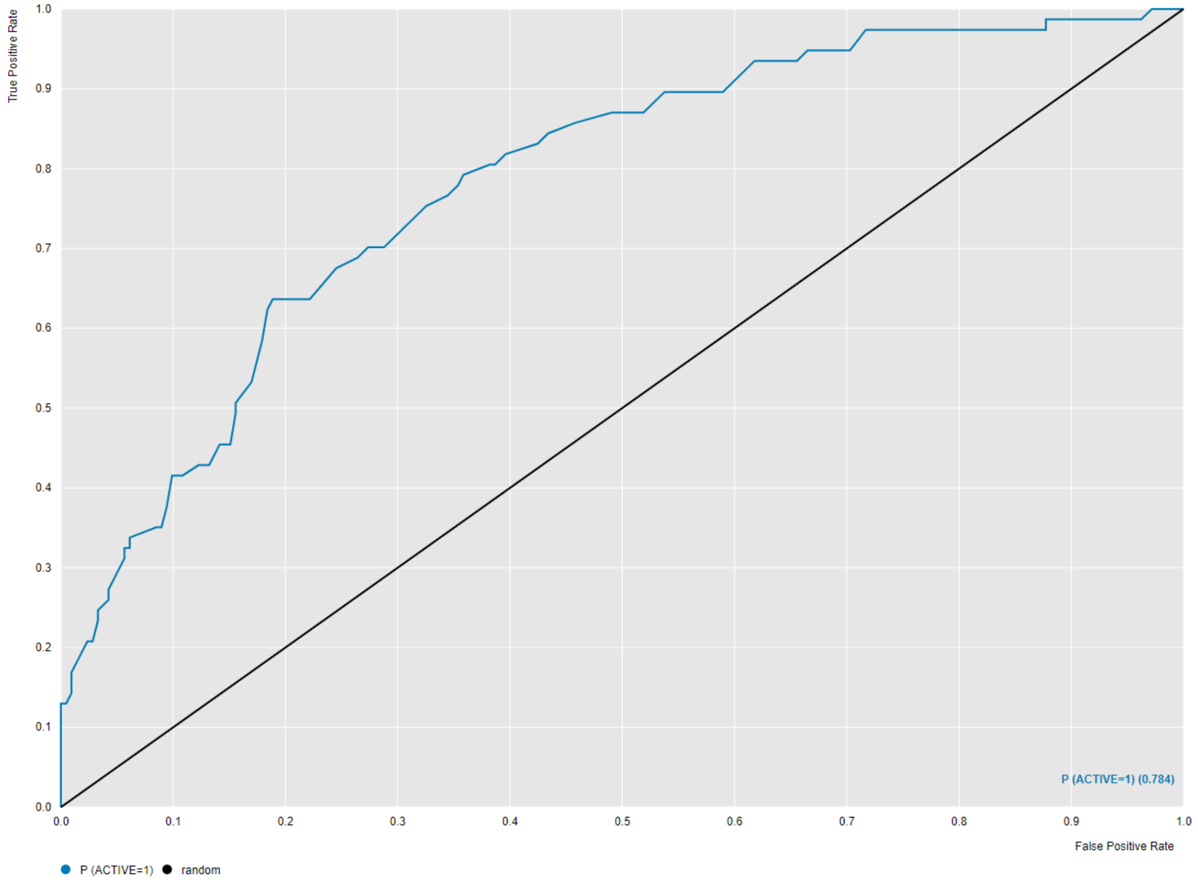

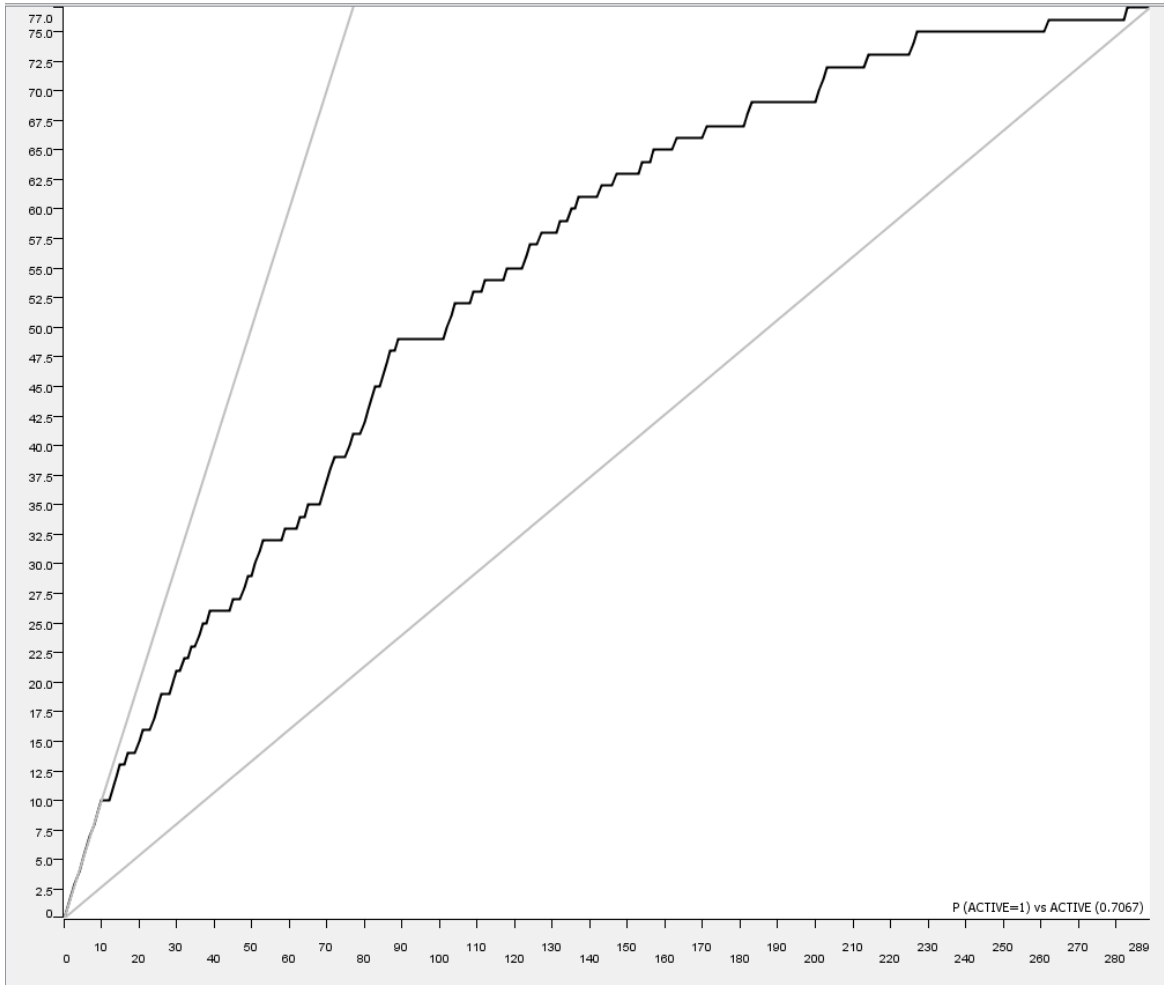

N=289

**Top 10% Mean IC50**	**2,500 nM**

	Predicted Active	Predicted Inactive
Active	48	29
Inactive	39	173

RF - <15 nM DefGood in c-Abl, 10% error; Random Seed = 121783

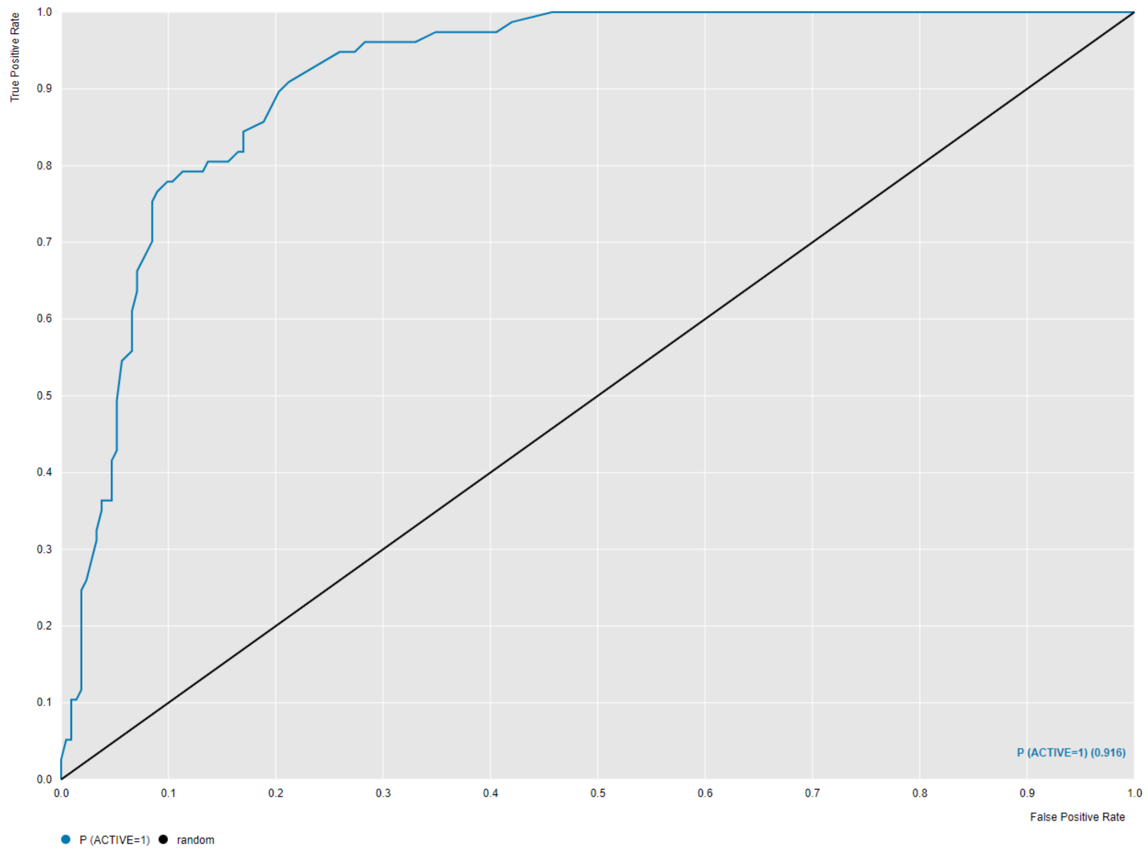

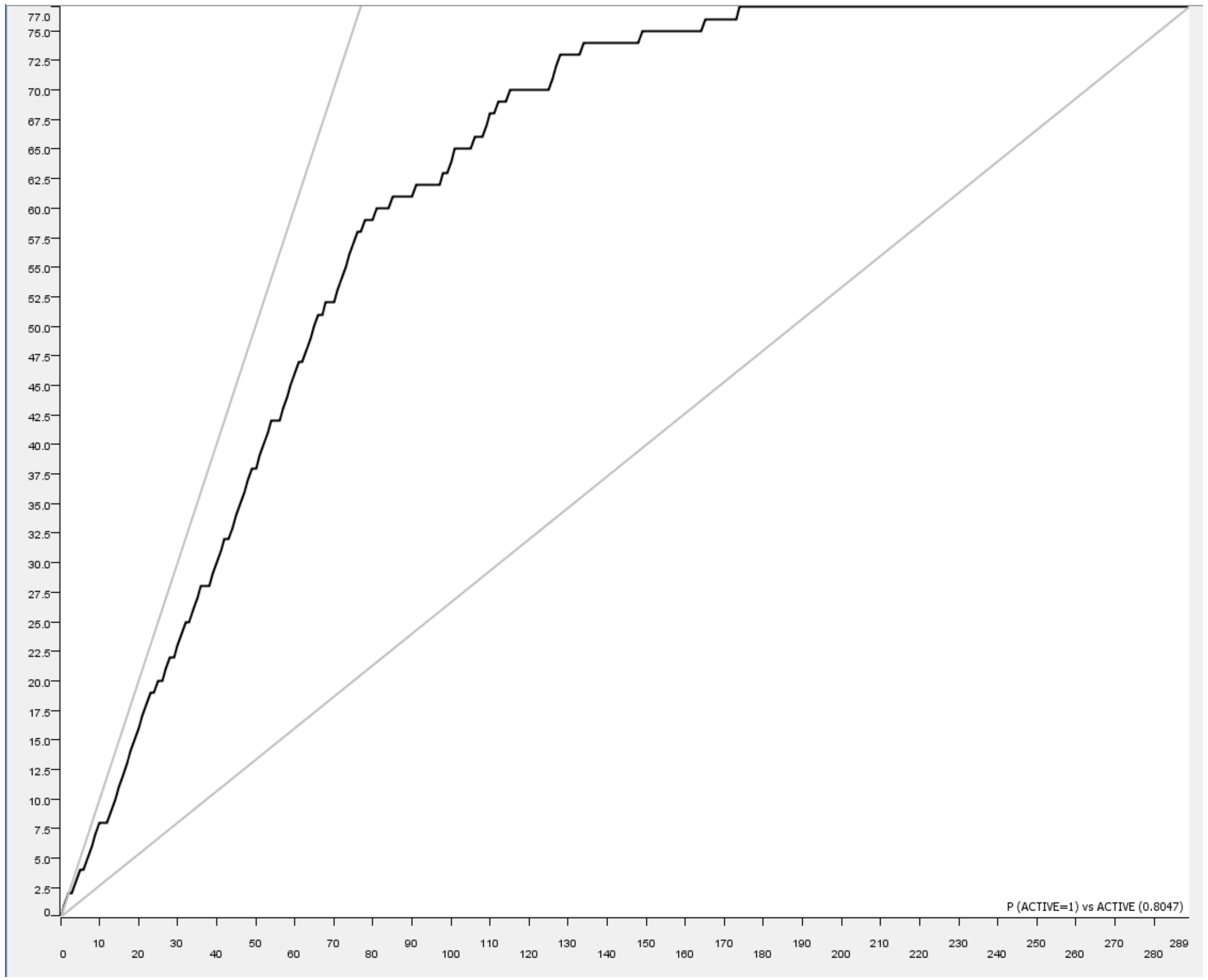

N=289

**Top 10% Mean IC50**	**117 nM (3 > 800 nM compounds)**

	Predicted Active	Predicted Inactive
Active	52	25
Inactive	16	196

RF - <15 nM DefGood in c-Abl, 20% error; Random Seed = 121783

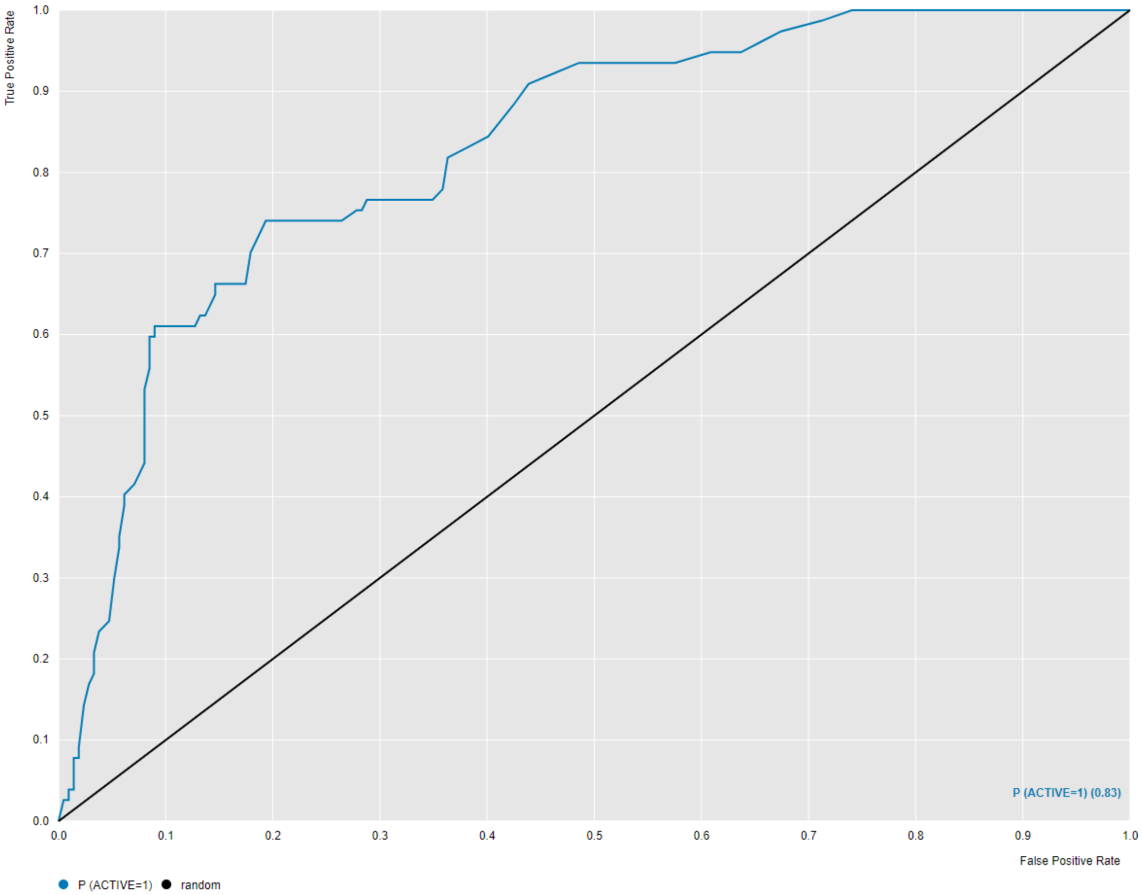

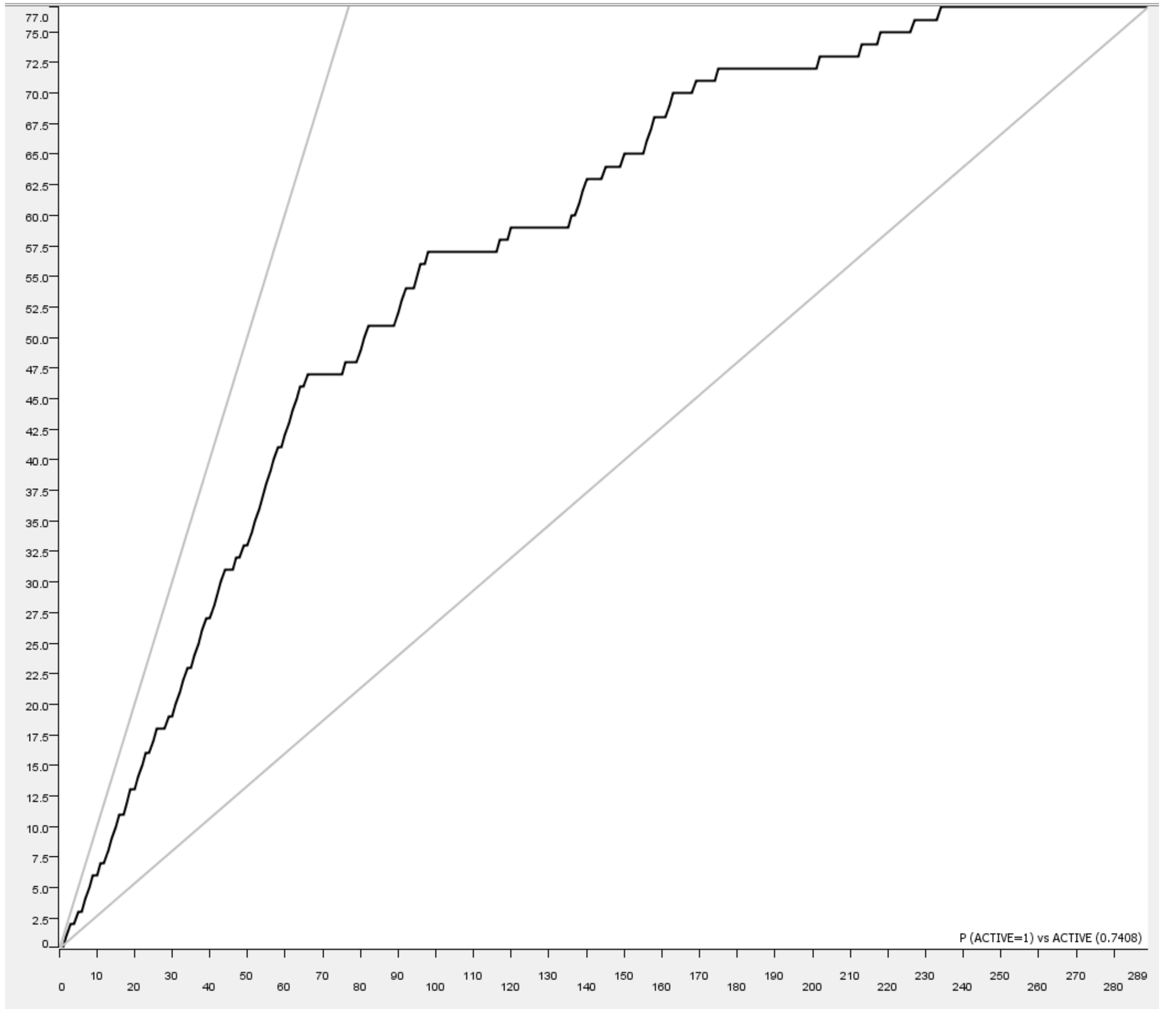

N=289

Top 10% Mean IC50	137 nM<br>(3 > 800 nM compounds)

	Predicted Active	Predicted Inactive
Active	47	30
Inactive	27	185

RF - <15 nM DefGood in c-Abl, 30% error; Random Seed = 121783

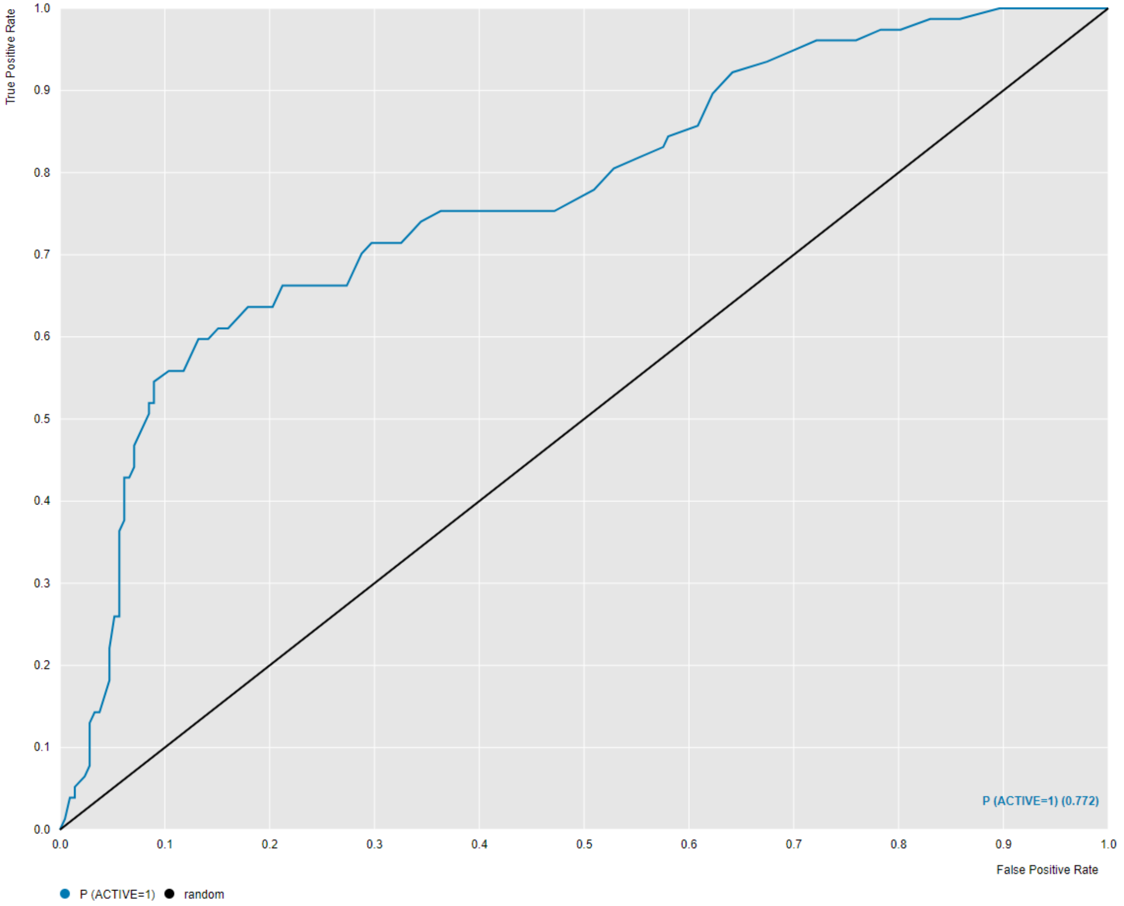

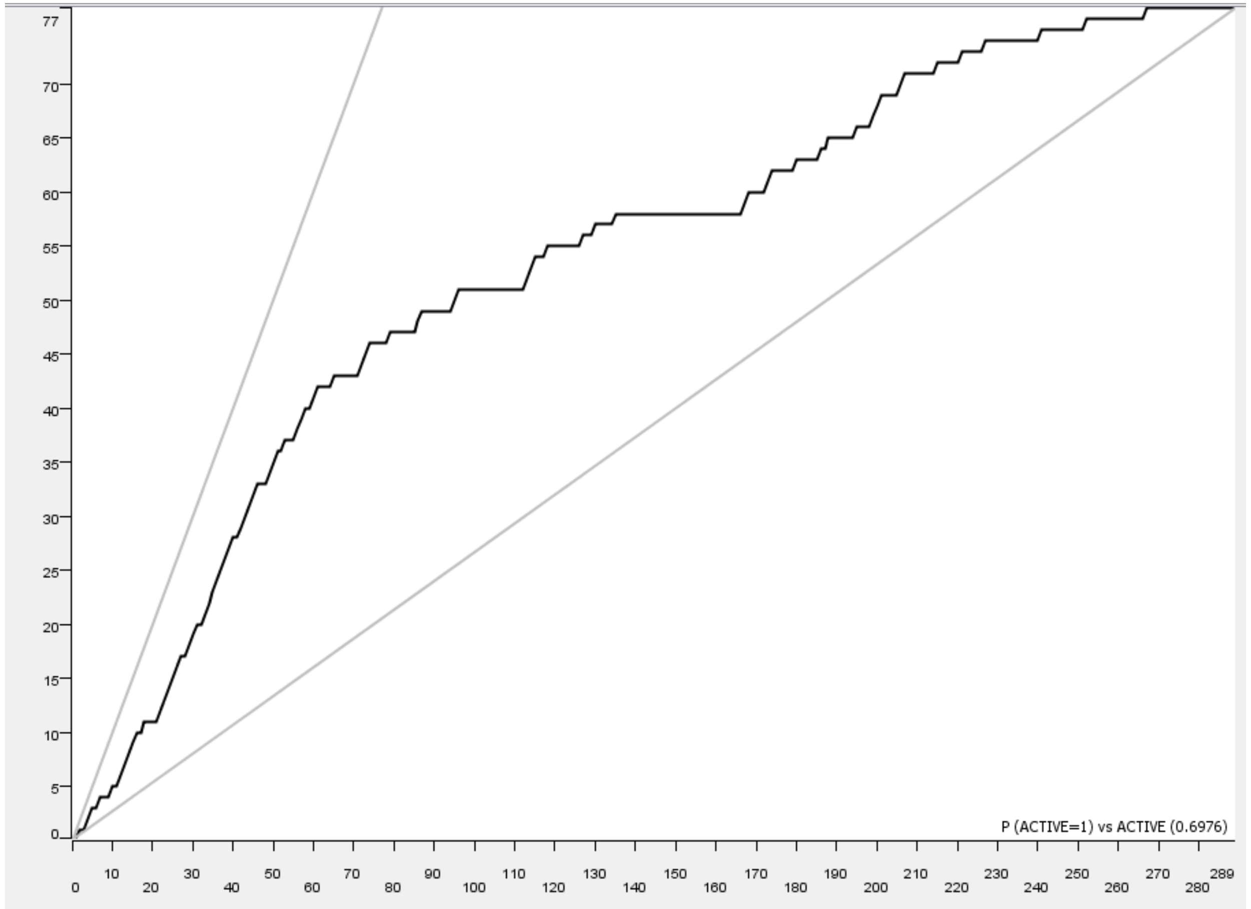

N=289

Top 10% Mean IC50	138 nM (3 > 800 nM compounds)

	Predicted Active	Predicted Inactive
Active	46	31
Inactive	30	182

RF - <15 nM DefGood in c-Abl, 35% error; Random Seed = 121783

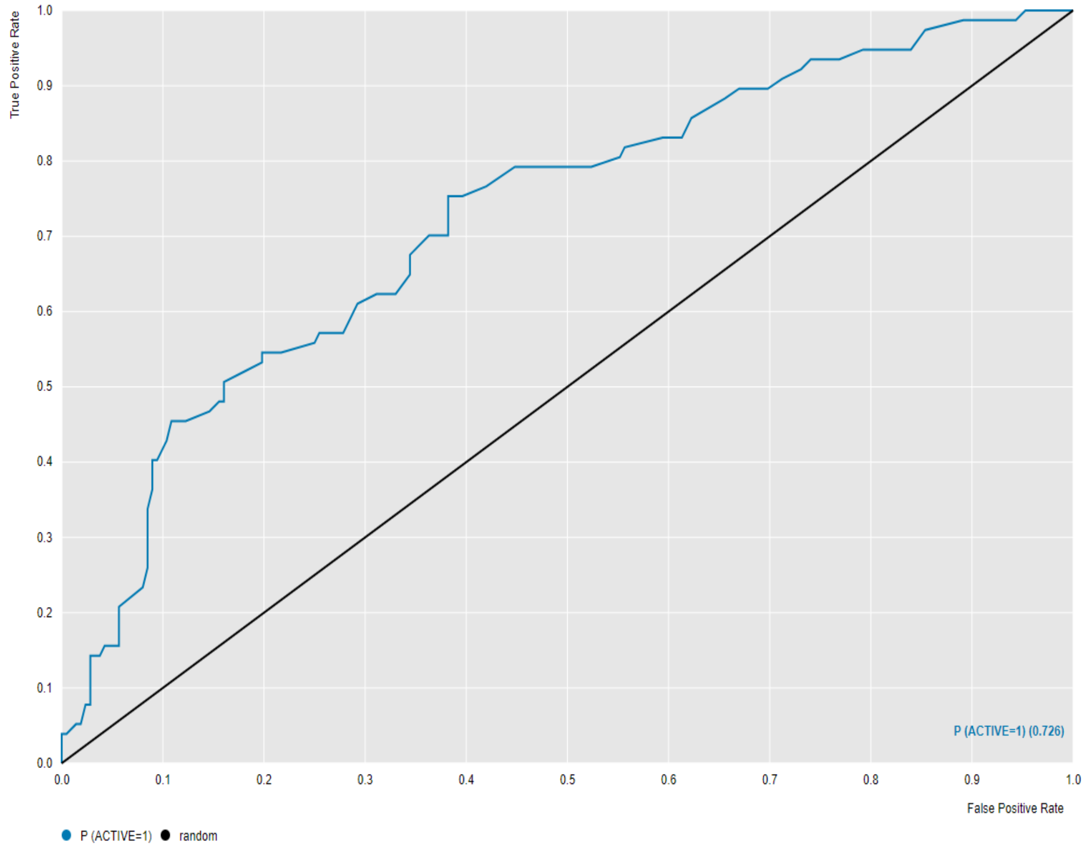

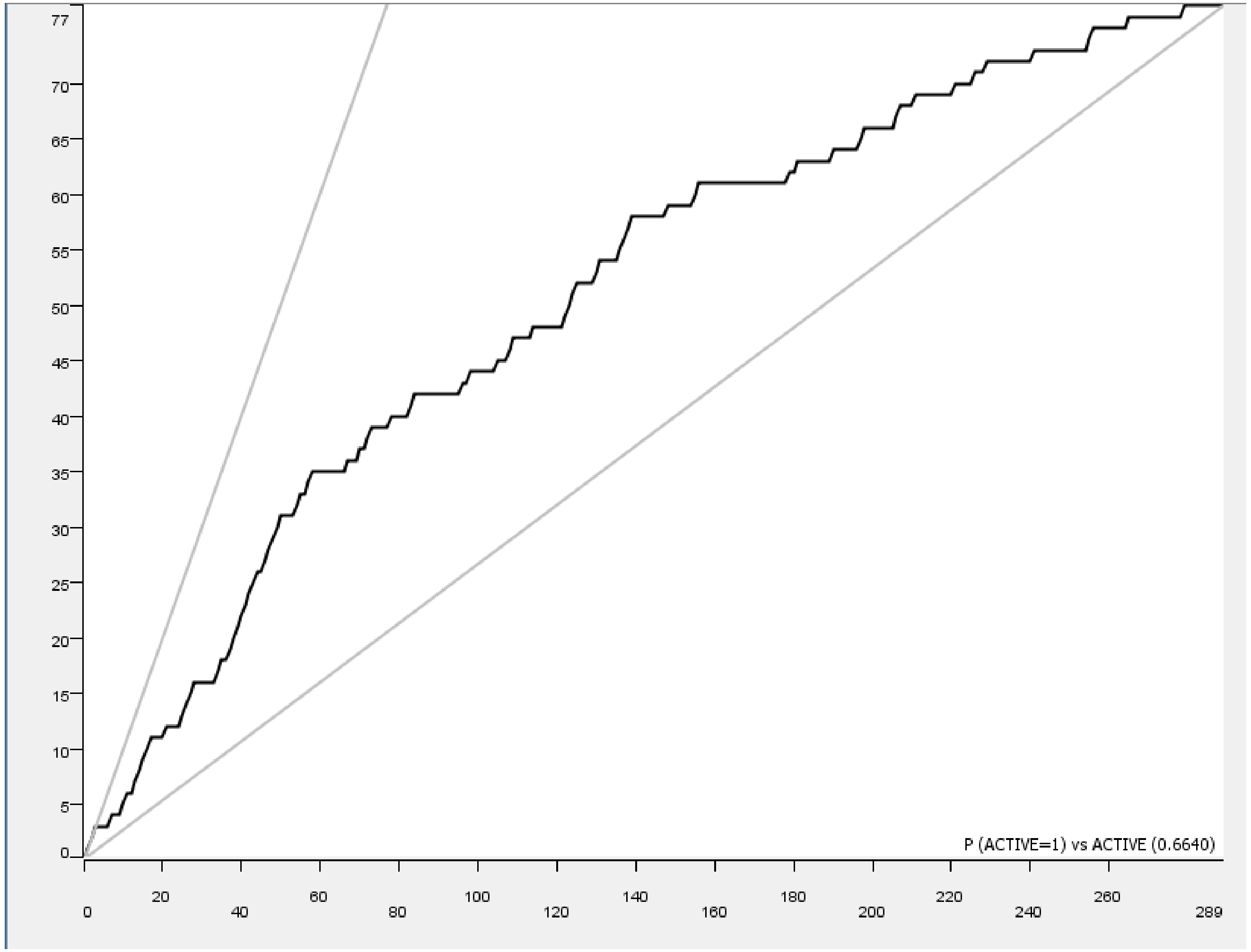

N=289

Top 10% Mean IC50	1,100 nM

	Predicted Active	Predicted Inactive
Active	42	35
Inactive	46	166

Conclusion - RF

- Across three random seeds, the % error that lead to a significant enrichment failure and top 10% IC50 increase in the retrospective test was 35%, 25% and 35% for each unique random seed.
- An RF can be very tolerant of data error in the c-Abl space, but it is less tolerant than an NBN.

PNN - <15 nM DefGood in c-Abl, 5% error ; Random seed = 1515533876005

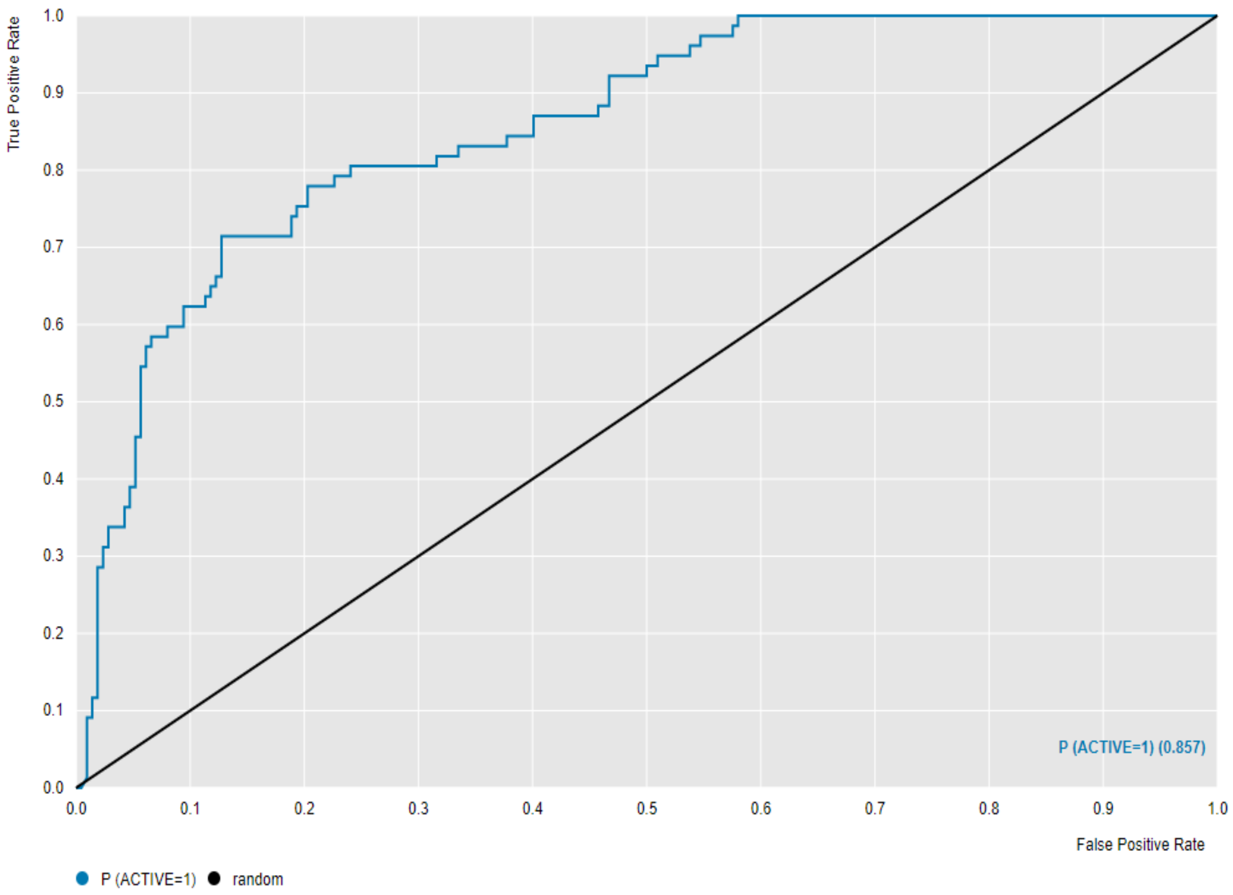

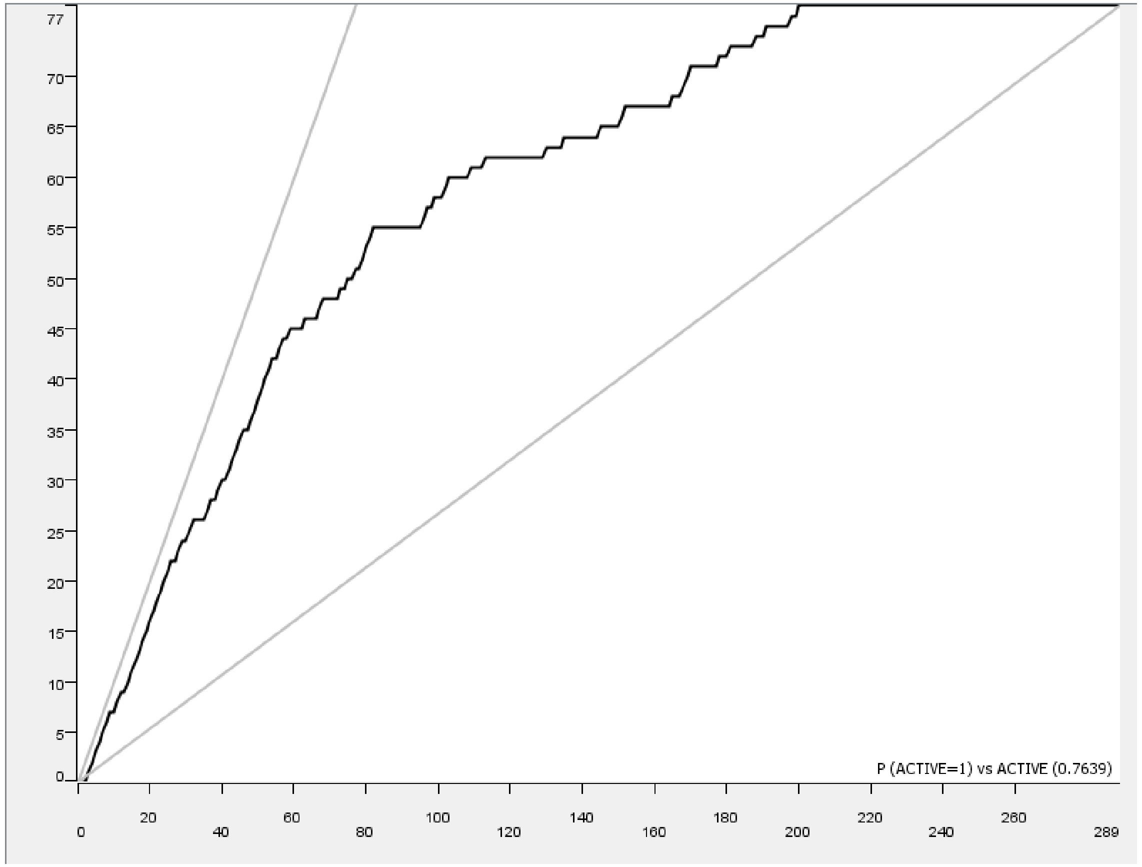

N=289

Top 10% Mean IC50	390 nM (one >10,000 nM compound, ranked first)

	Predicted Active	Predicted Inactive
Active	40	37
Inactive	12	200

PNN - <15 nM DefGood in c-Abl, 10% error

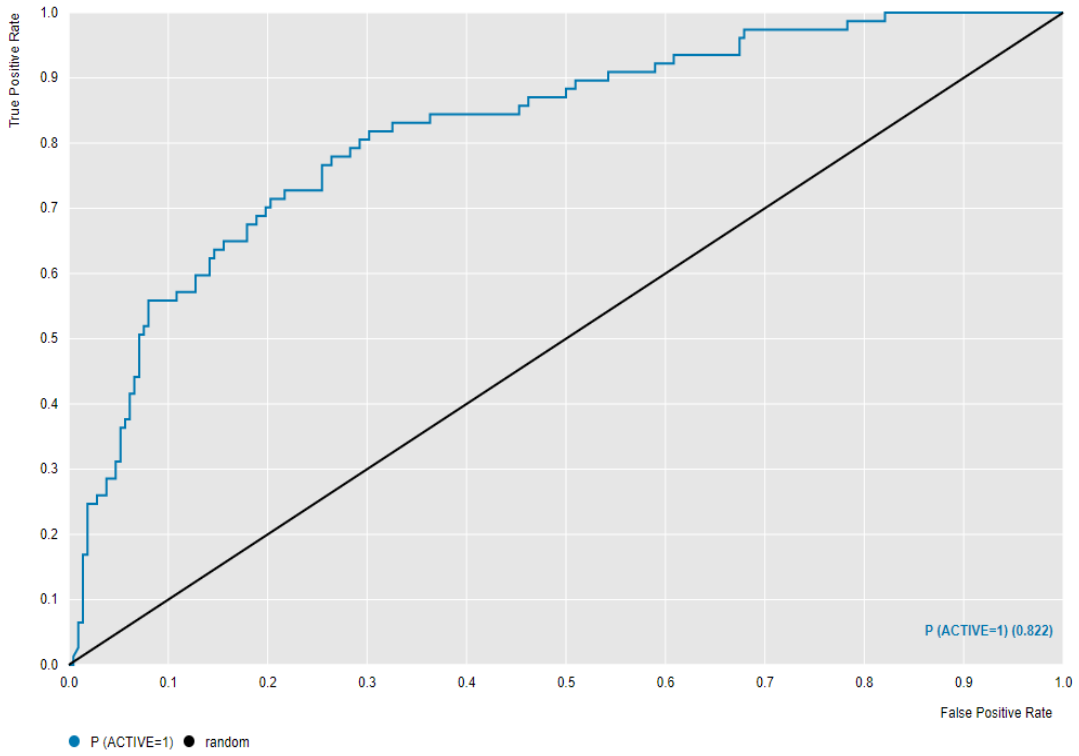

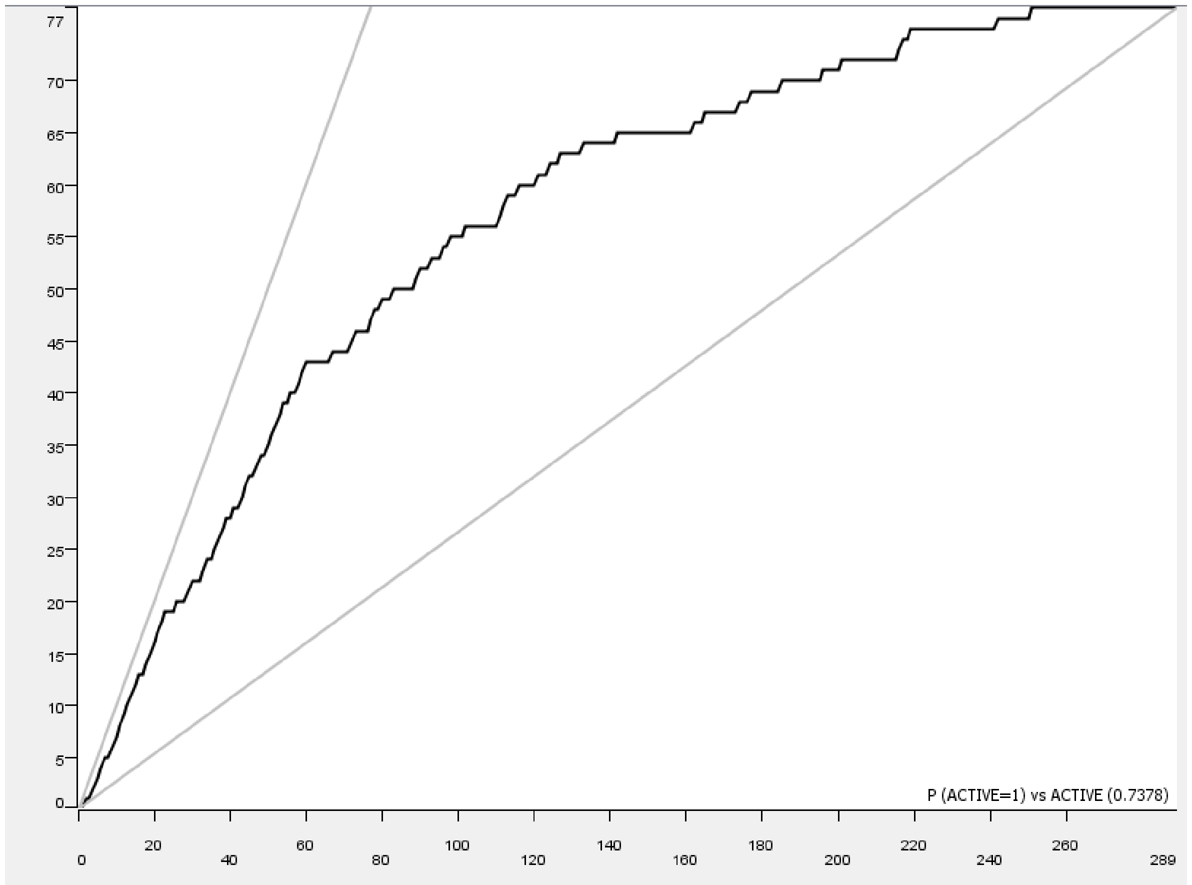

N=289

Top 10% Mean IC50	470 nM (one >10,000 nM compound, ranked first)

	Predicted Active	Predicted Inactive
Active	33	44
Inactive	14	198

PNN - <15 nM DefGood in c-Abl, 15% error

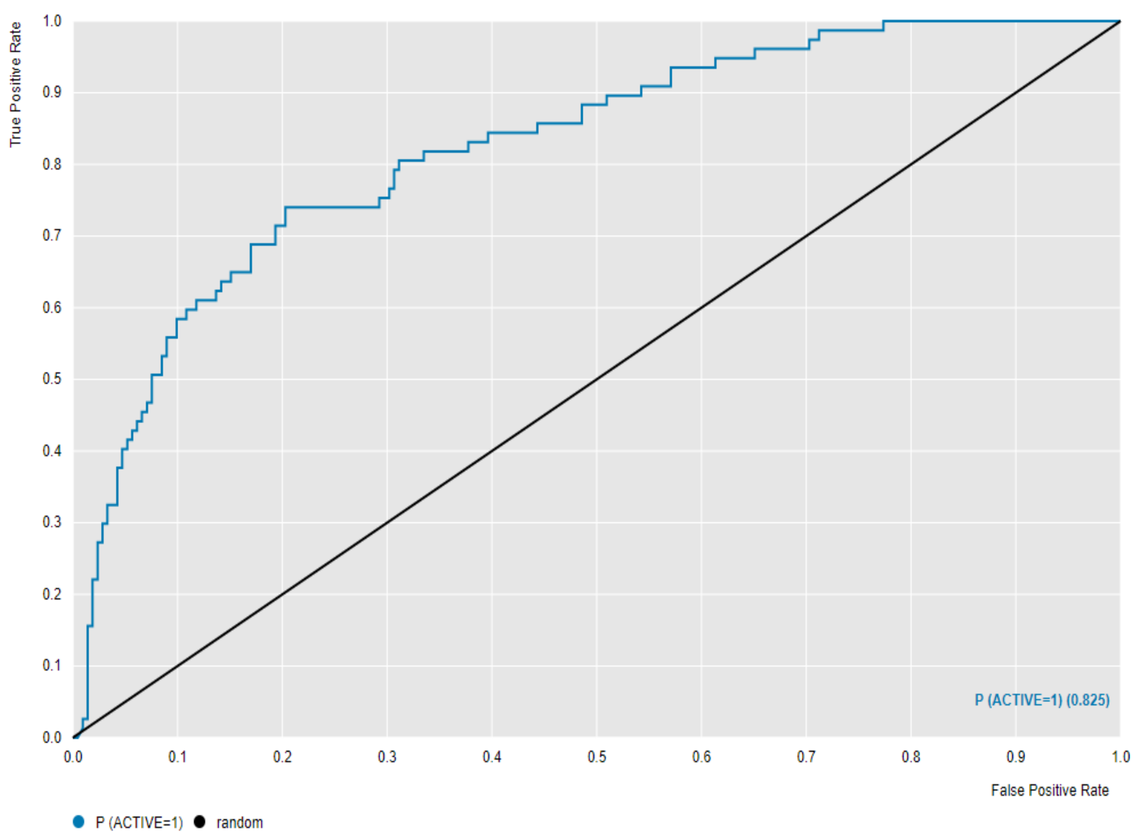

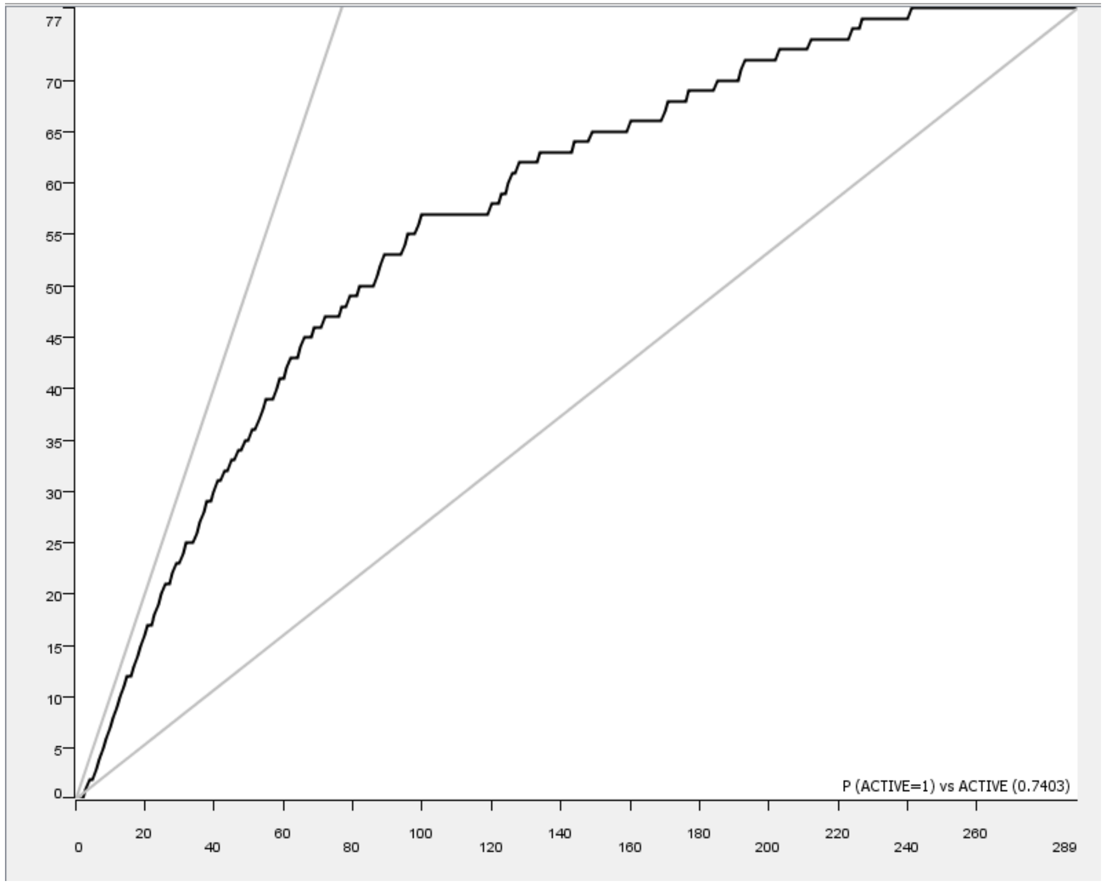

N=289

Top 10% Mean IC50	400 nM (one >10,000 nM compound, ranked first)

	Predicted Active	Predicted Inactive
Active	45	32
Inactive	23	189

PNN - <15 nM DefGood in c-Abl, 20% error

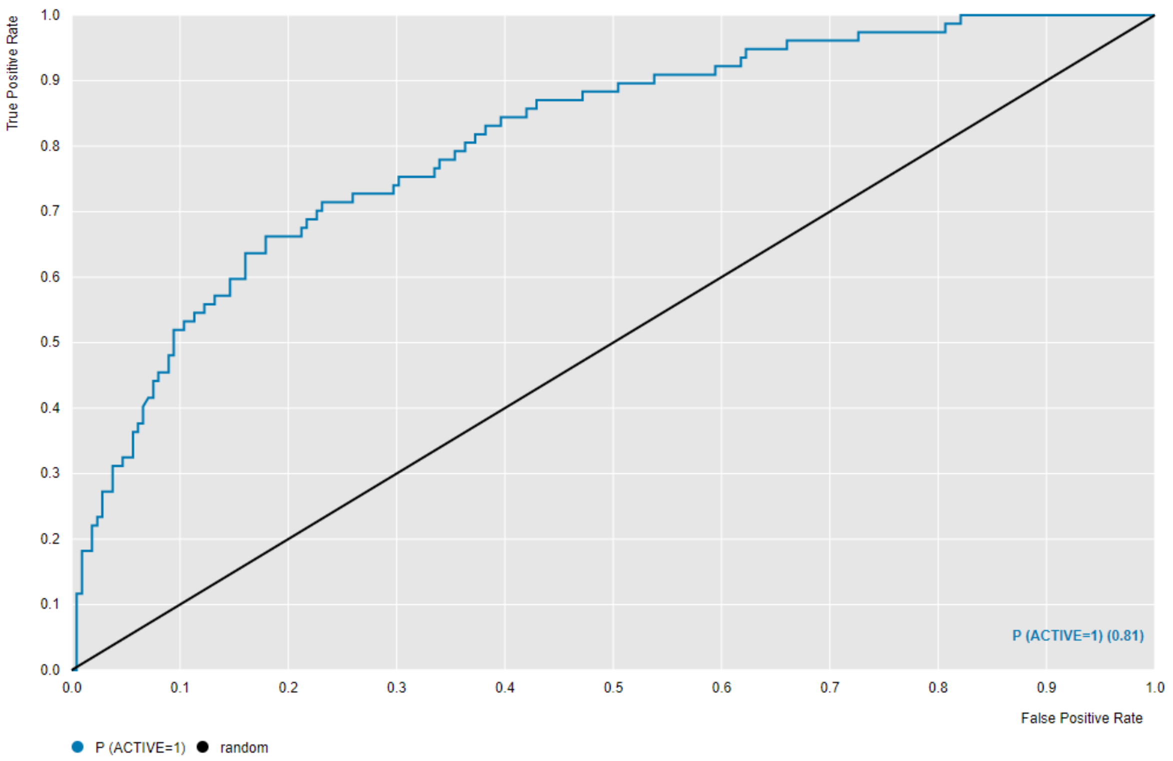

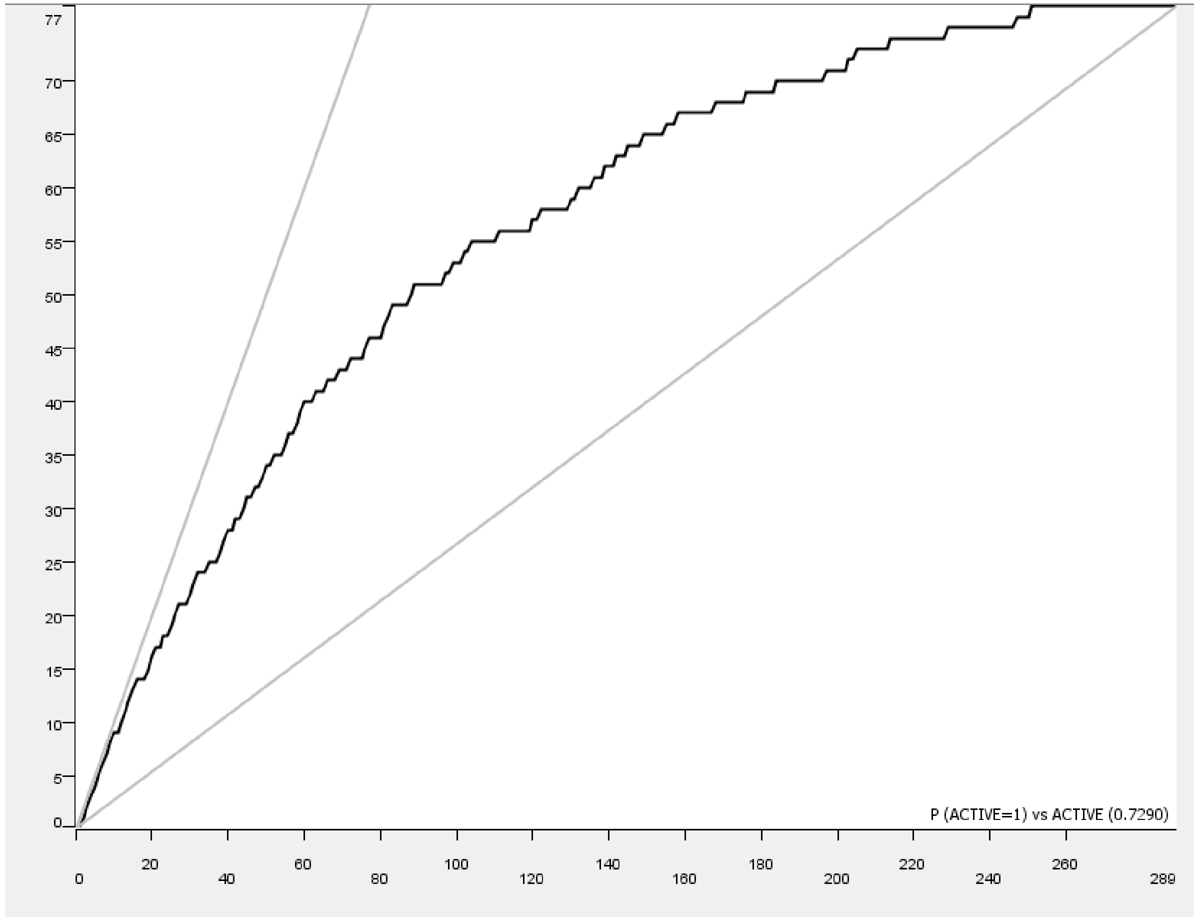

N=289

	Predicted Active	Predicted Inactive
Active	42	35
Inactive	26	186

Top 10% Mean IC50	565 nM(one >10,000 nM compound, ranked first)

PNN - <15 nM DefGood in c-Abl, 25% error

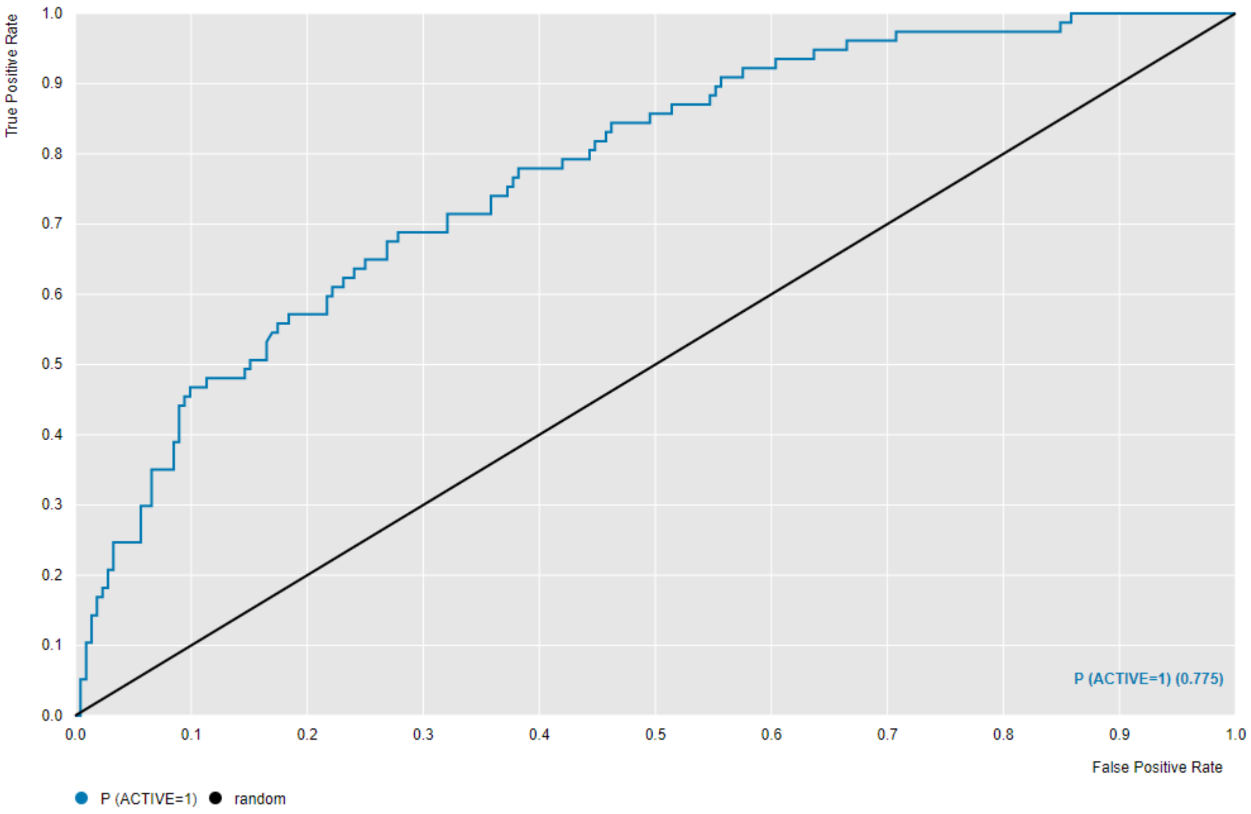

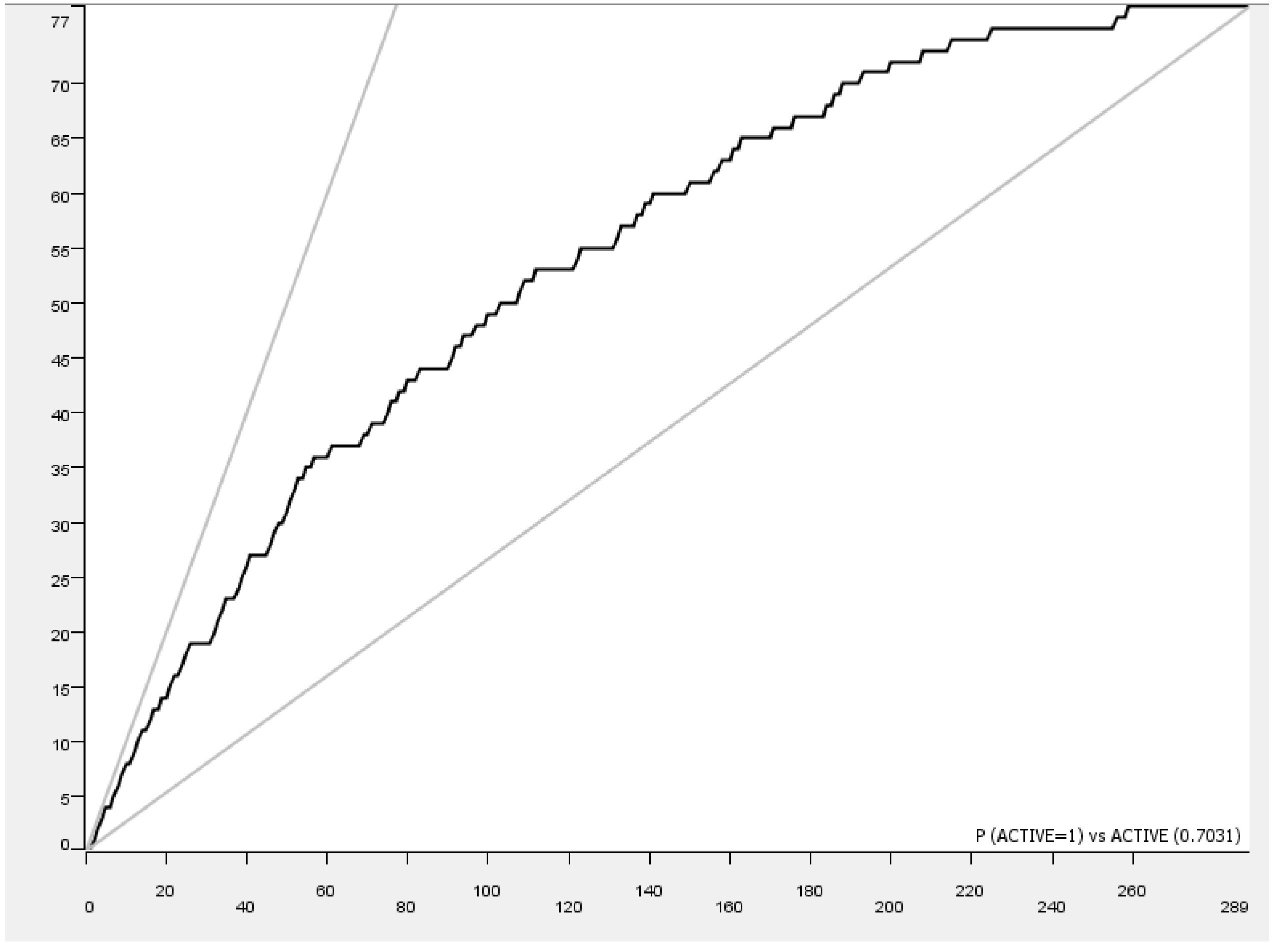

N=289

**Top 10% Mean IC50**	**560 nM(one >10,000 nM compound, ranked first)**

	Predicted Active	Predicted Inactive
Active	37	40
Inactive	26	186

PNN - <15 nM DefGood in c-Abl, 30% error

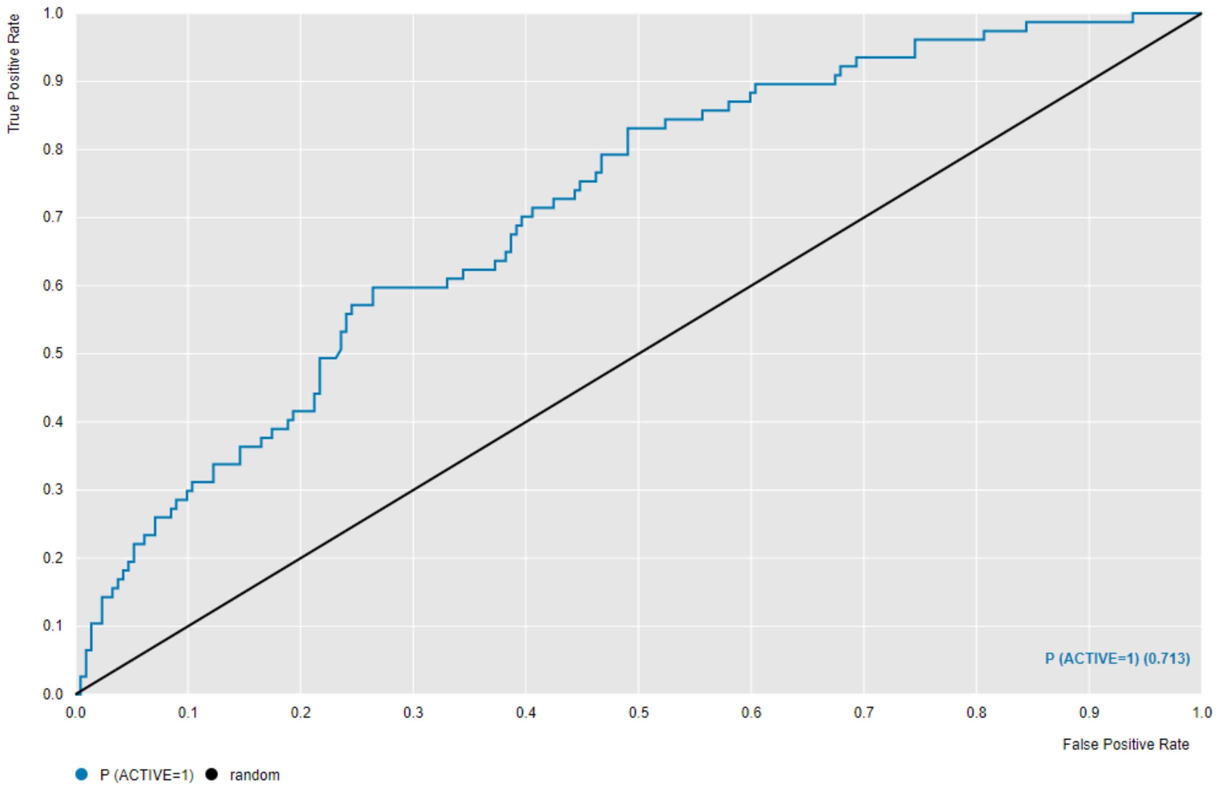

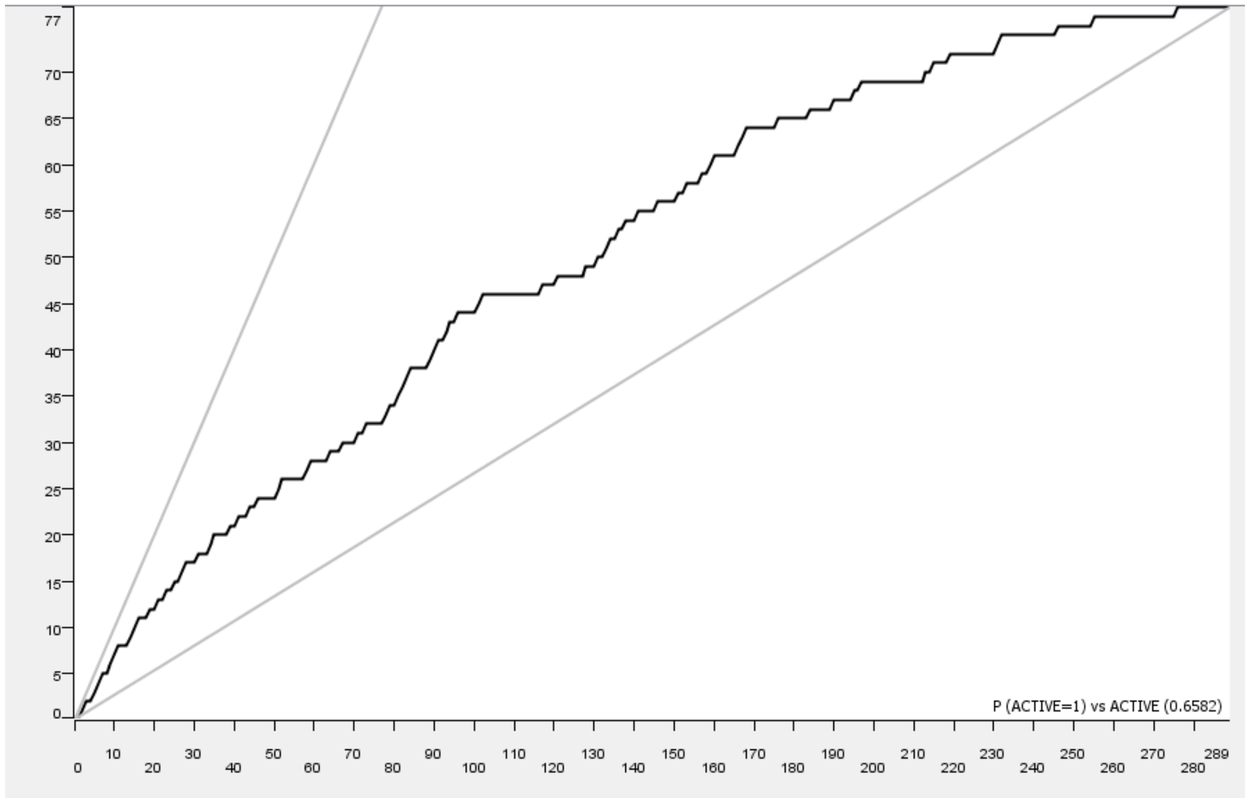

N=289

Top 10% Mean IC50	503 nM (one >10,000 nM compound, ranked first)

	Predicted Active	Predicted Inactive
Active	30	47
Inactive	39	173

PNN - <15 nM DefGood in c-Abl, 35% error

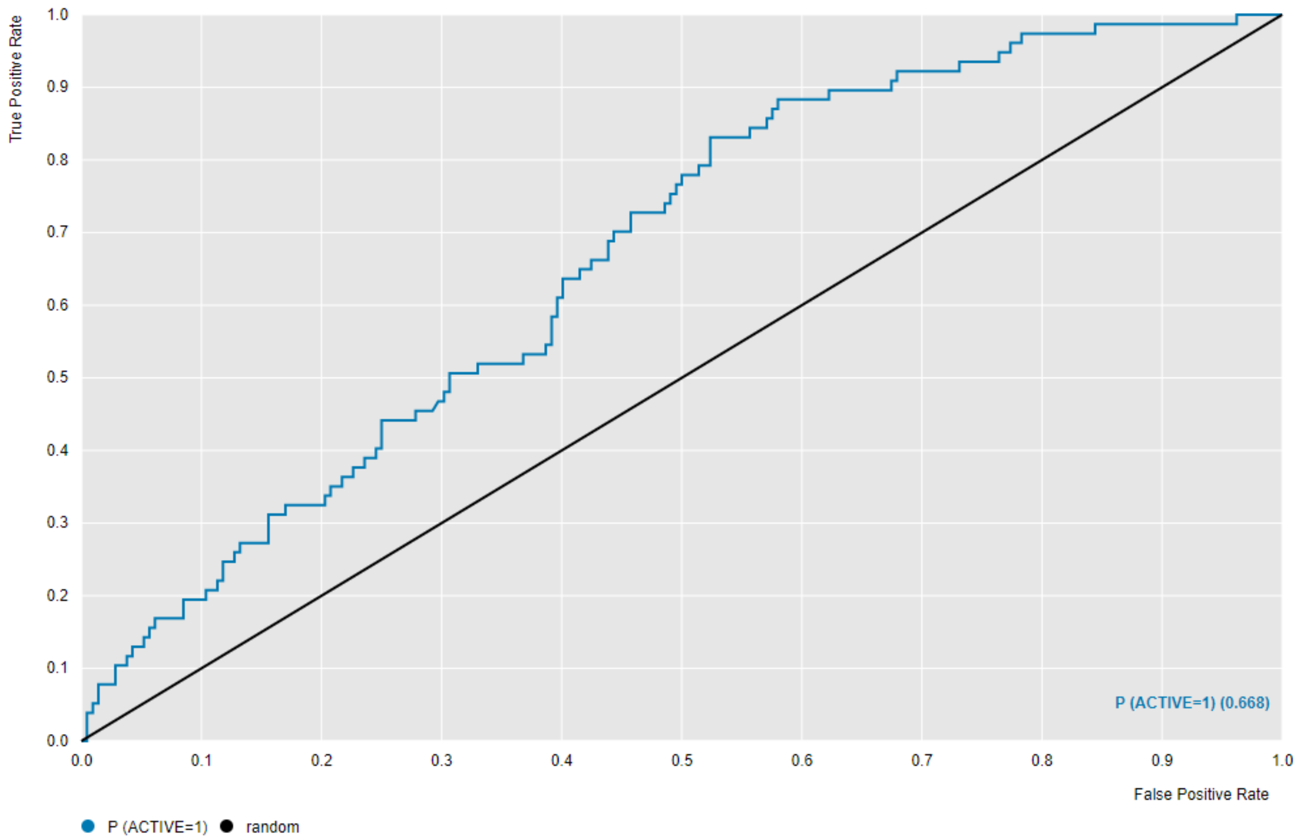

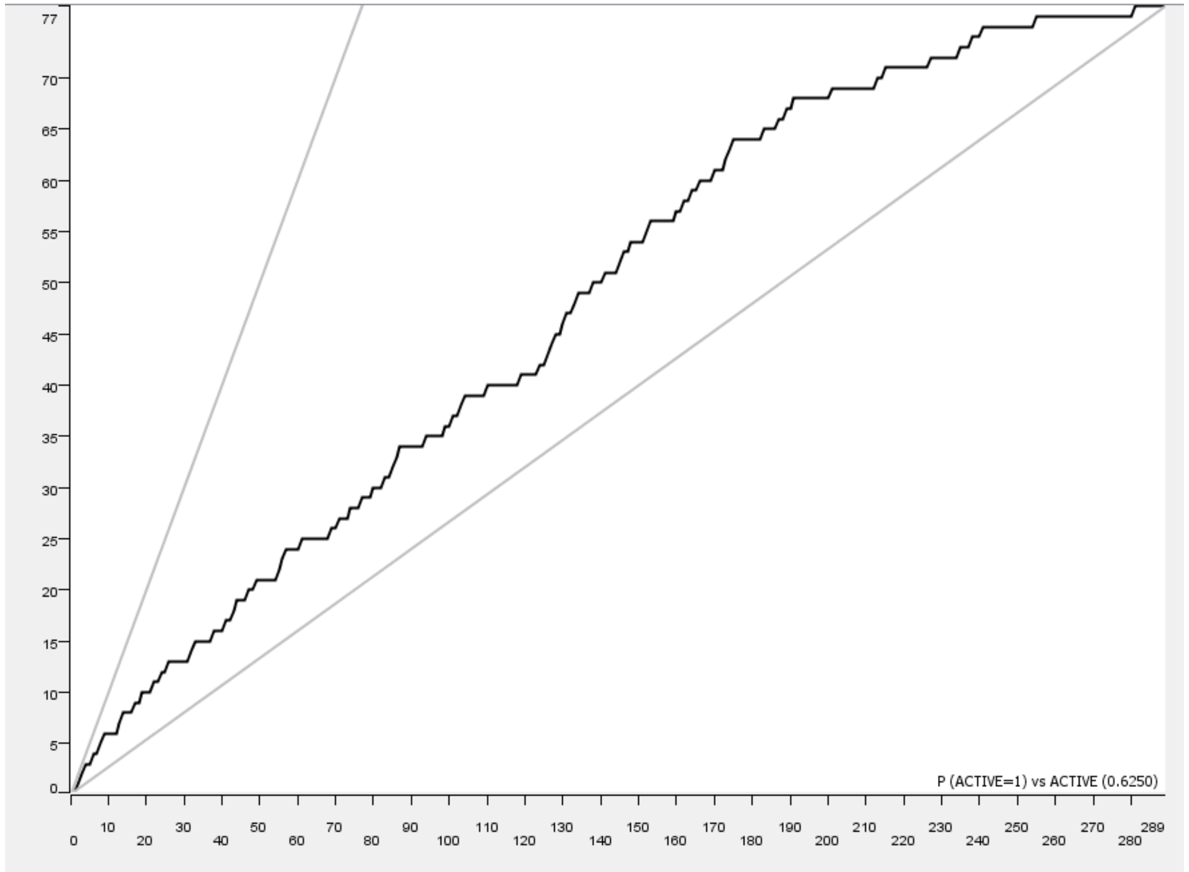

N=289

Top 10% Mean IC50	4,300

	Predicted Active	Predicted Inactive
Active	29	48
Inactive	48	164

PNN - <15 nM DefGood in c-Abl, 5% error; Random seed = 429

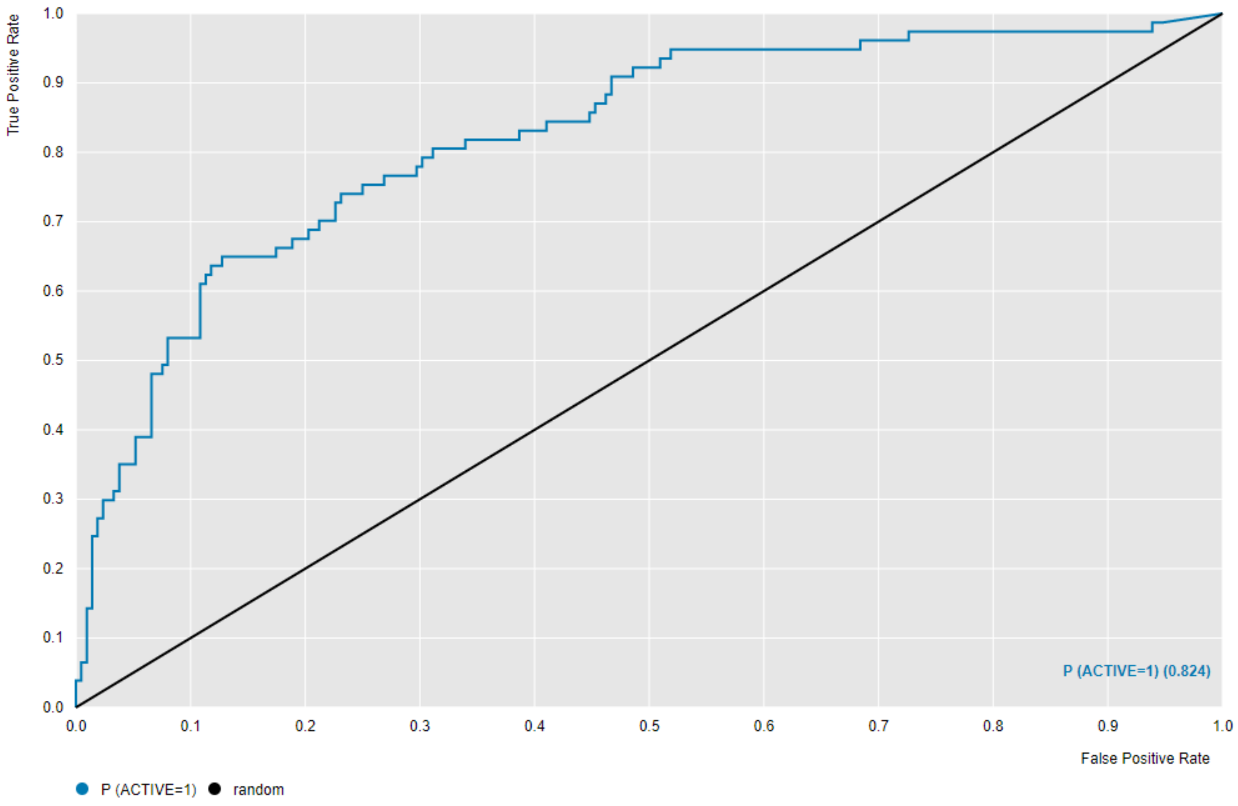

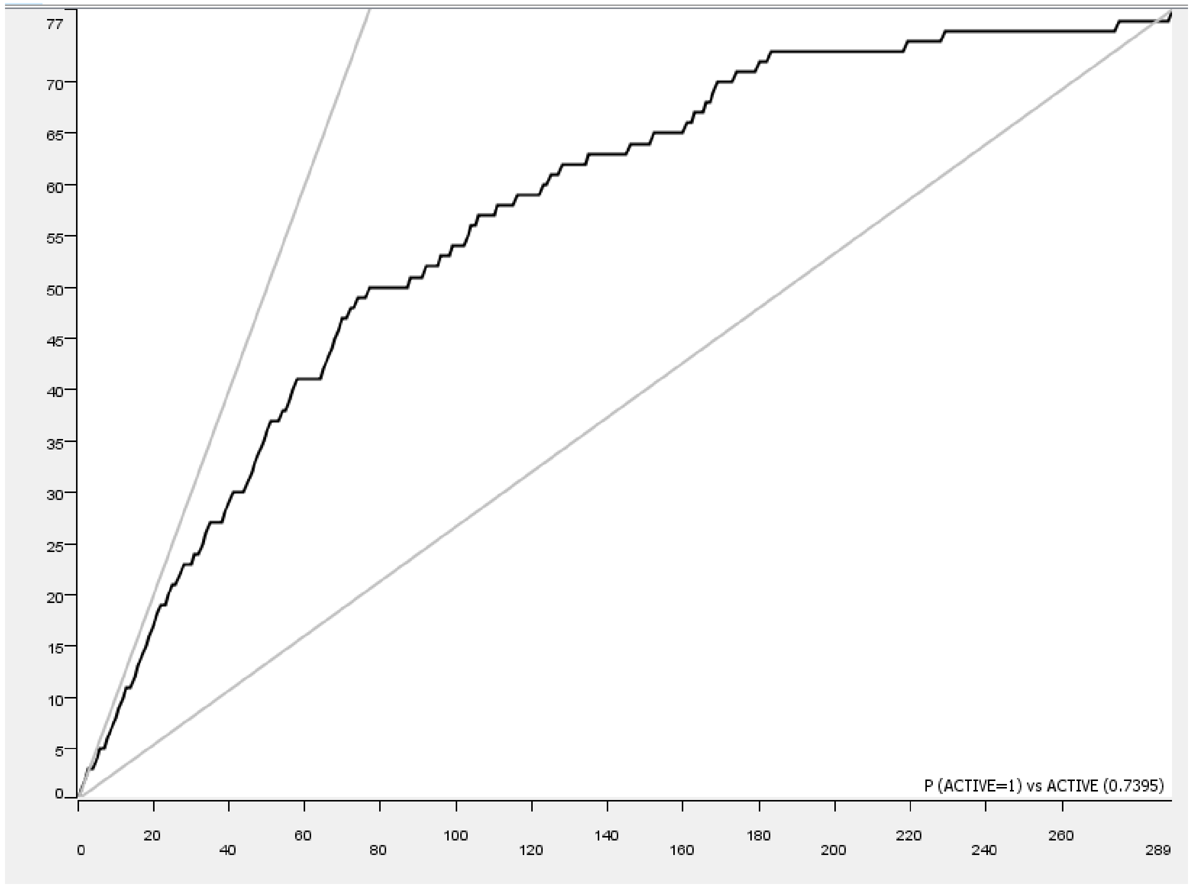

N=289

Top 10% Mean IC50	61.5 nM (one >800 nM Compound)

	Predicted Active	Predicted Inactive
Active	23	54
Inactive	5	207

PNN - <15 nM DefGood in c-Abl, 10% error; Random seed = 429

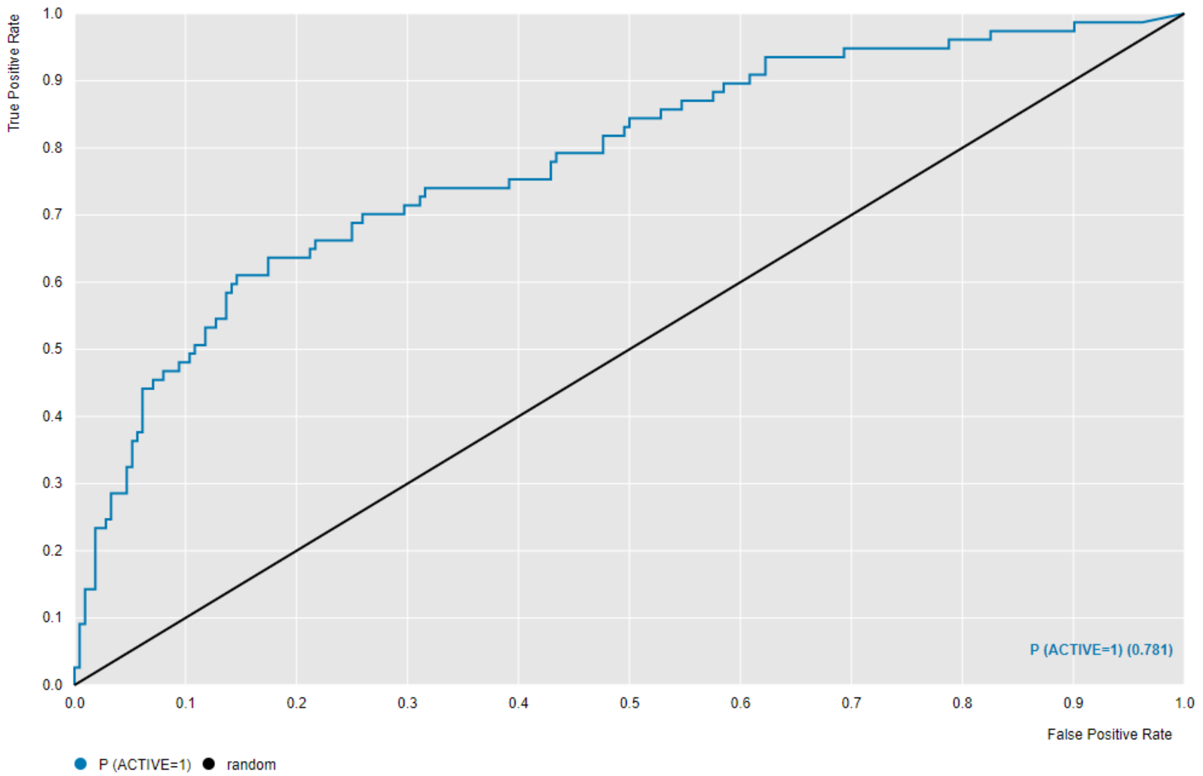

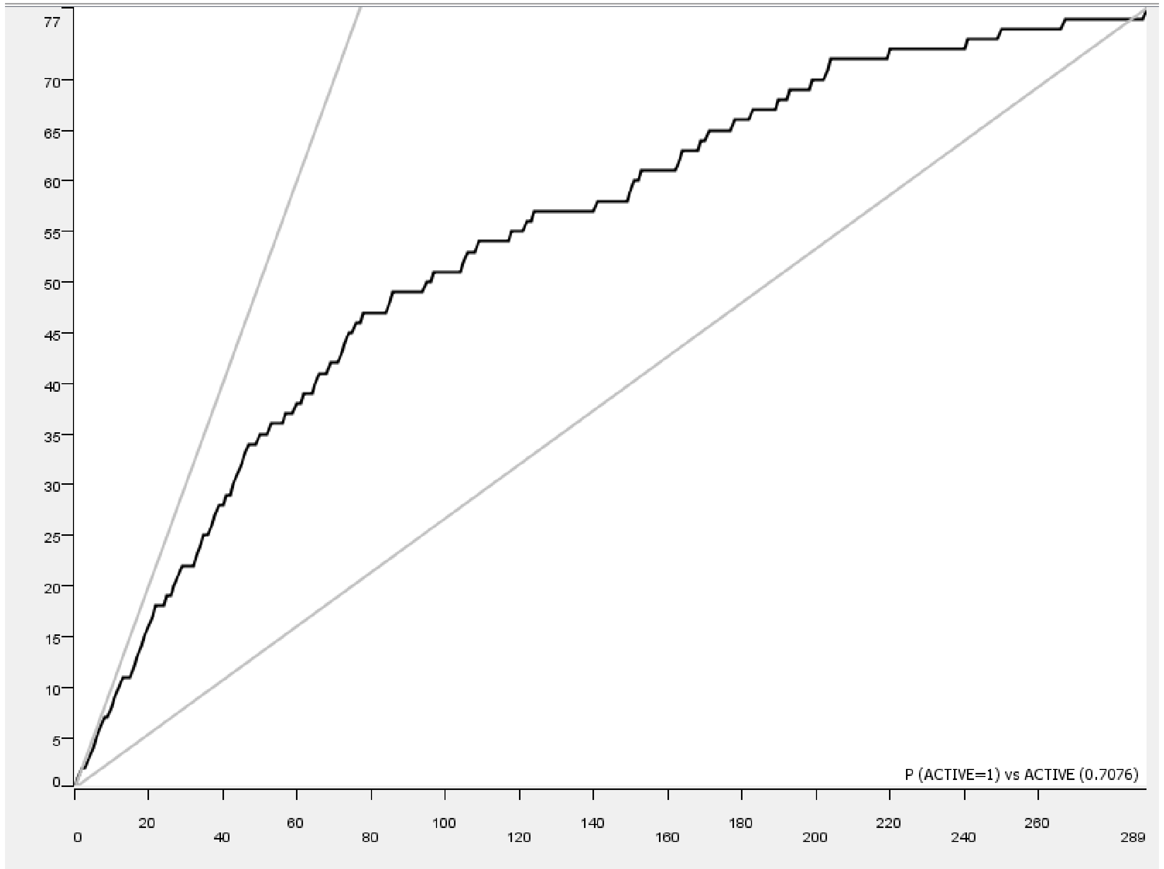

N=289

**Top 10% Mean IC50**	**420 nM (one >800 nM compound, and one >10,000 nM)**

	**Predicted Active**	**Predicted Inactive**
Active	32	45
Inactive	13	199

PNN - <15 nM DefGood in c-Abl, 15% error; Random seed = 429

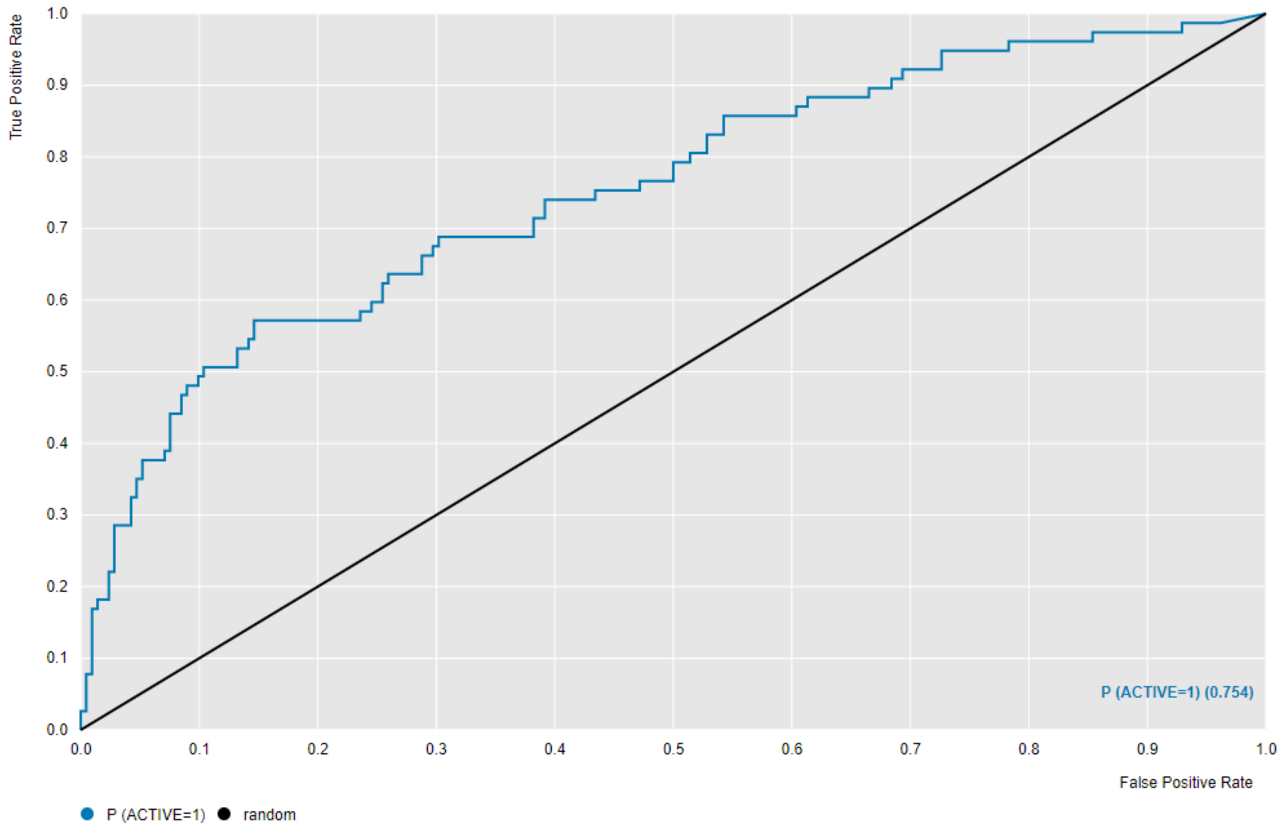

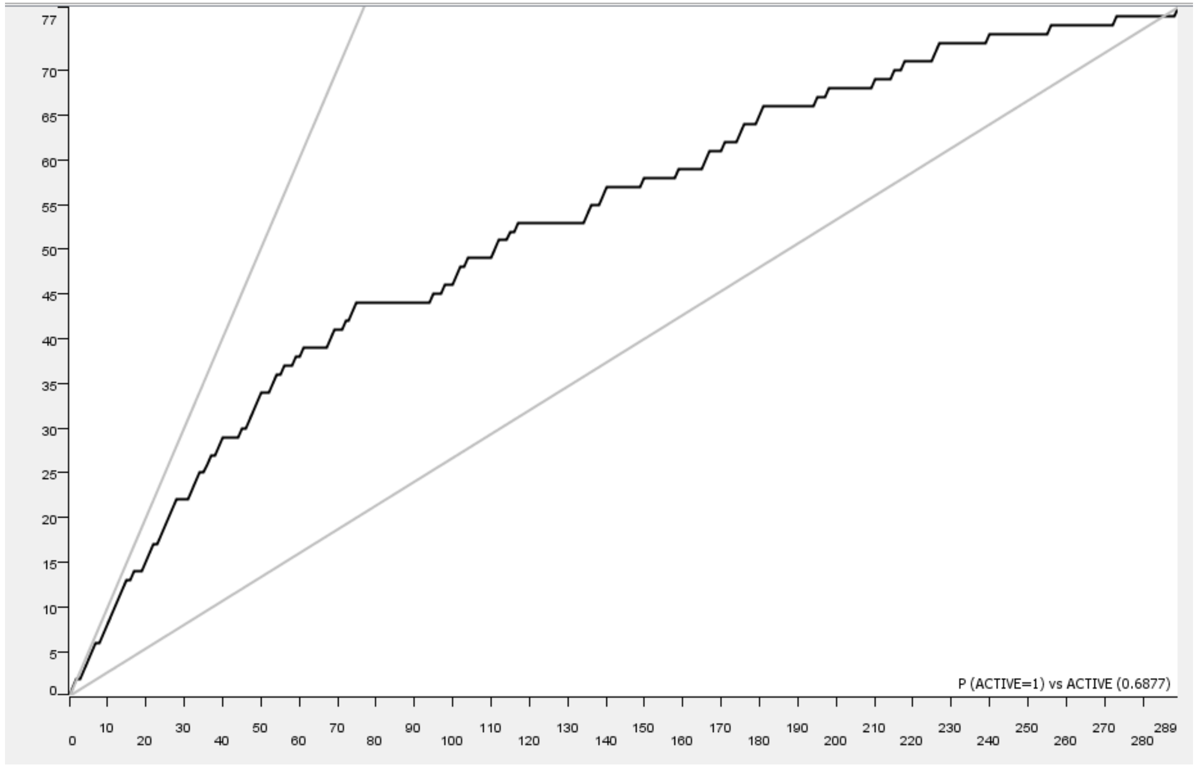

N=289

**Top 10% Mean IC50**	**390 nM (one >800 nM compound, and one >10,000 nM)**

	Predicted Active	Predicted Inactive
Active	34	43
Inactive	17	195

PNN - <15 nM DefGood in c-Abl, 20% error ; Random seed = 429

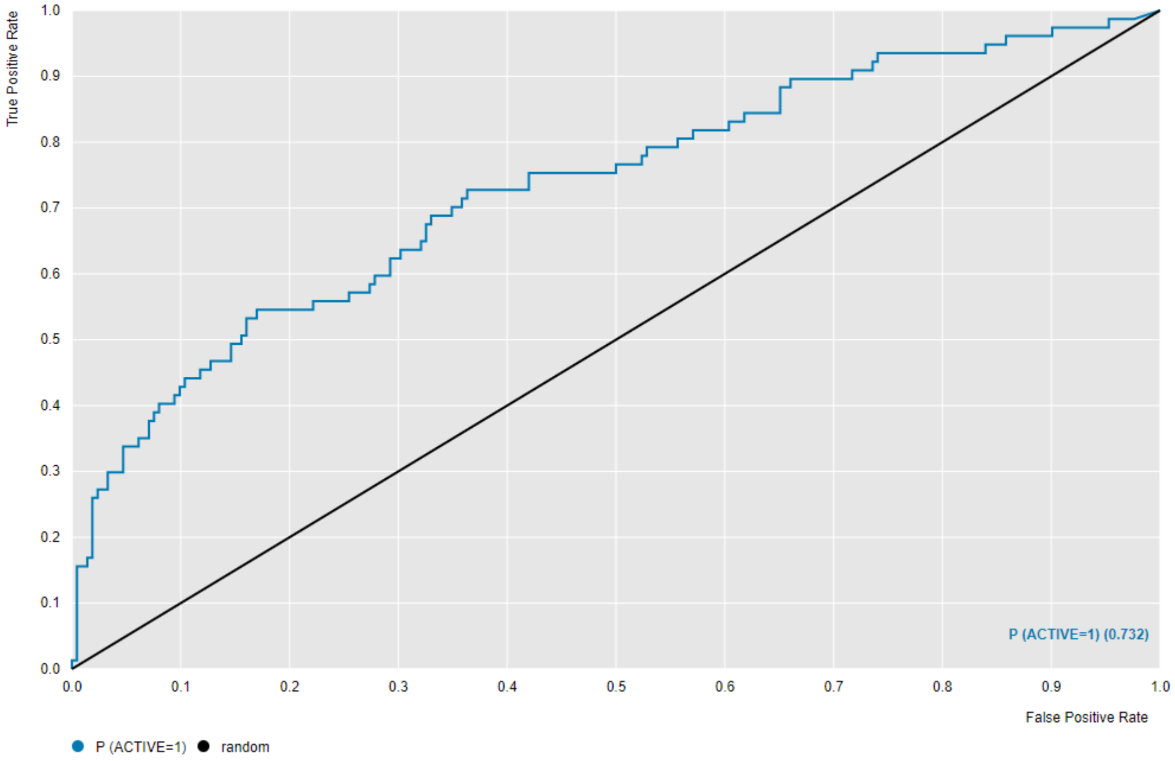

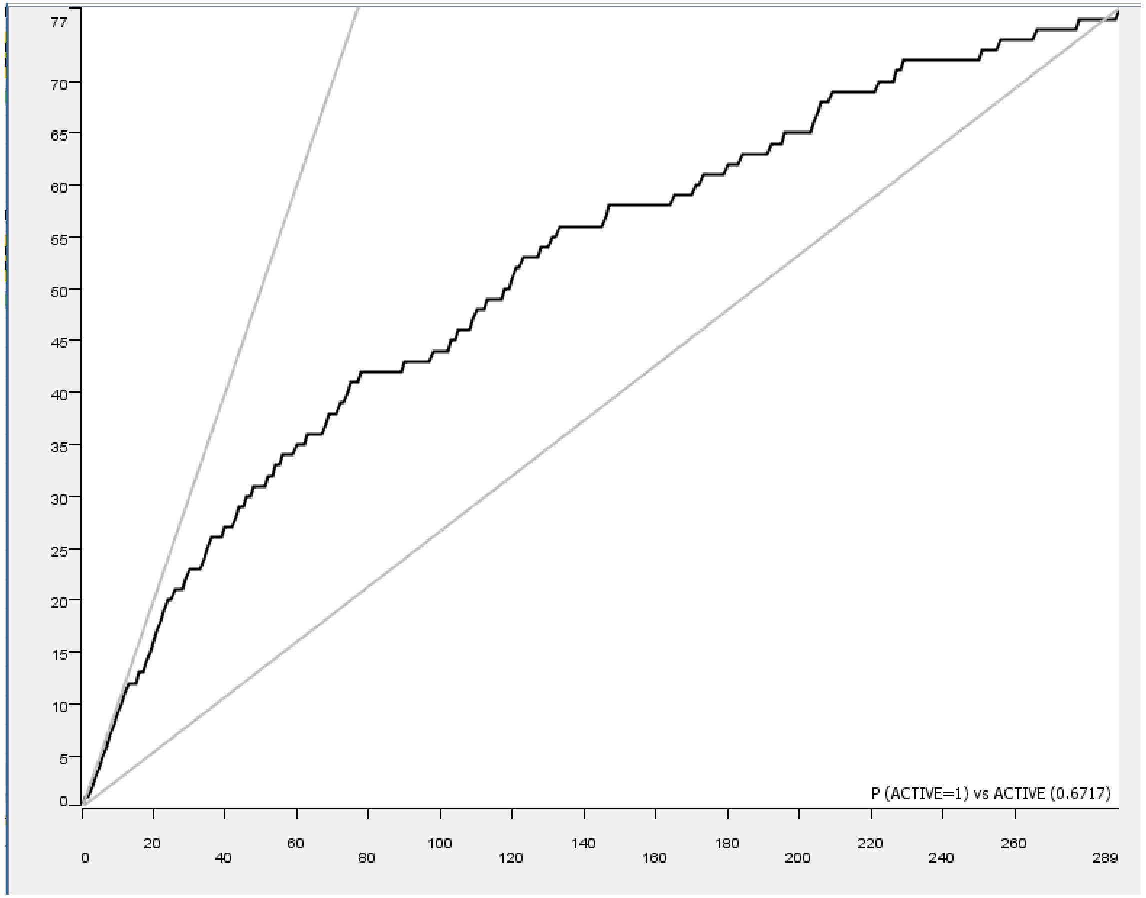

N=289

	Predicted Active	Predicted Inactive
Active	42	35
Inactive	40	172

**Top 10% Mean IC50**	**480 nM**<br>**Several uM mistakes**

PNN - <15 nM DefGood in c-Abl, 25% error ; Random seed = 429

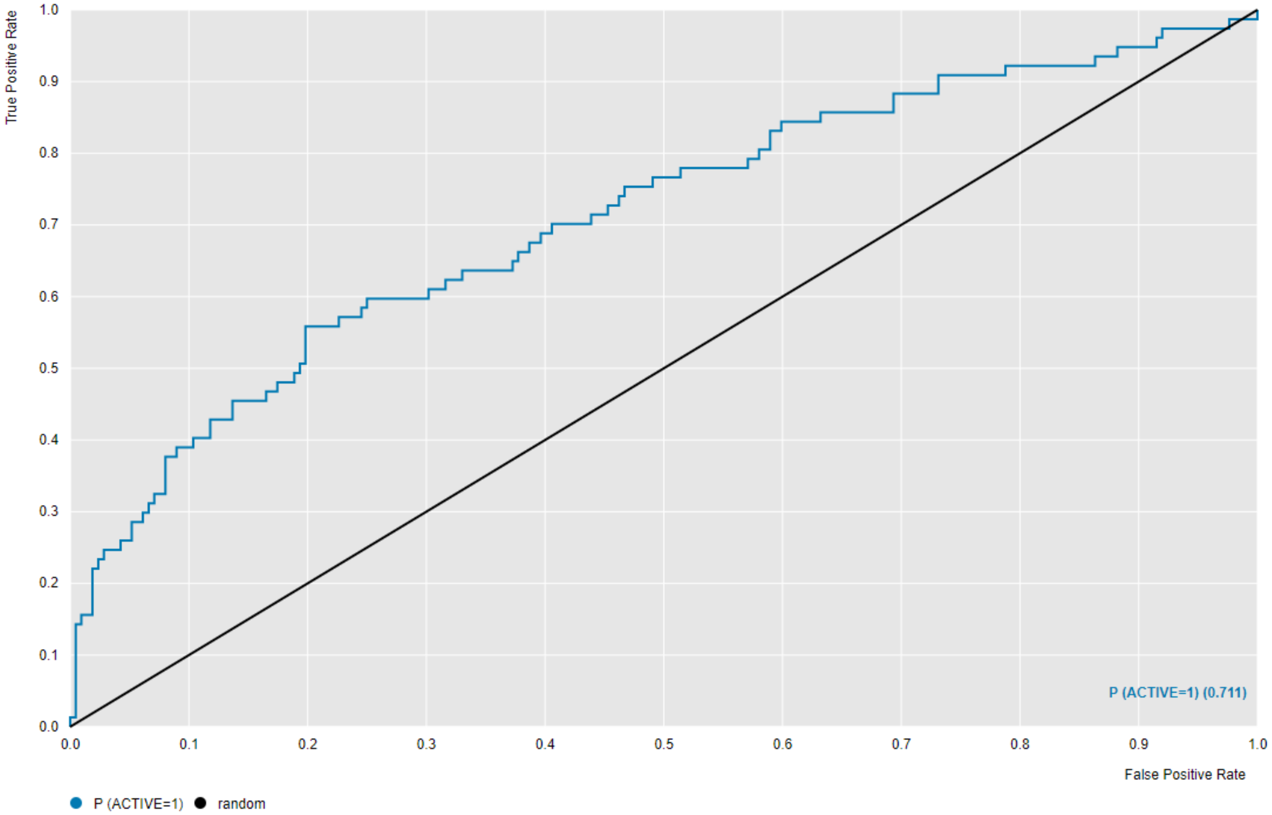

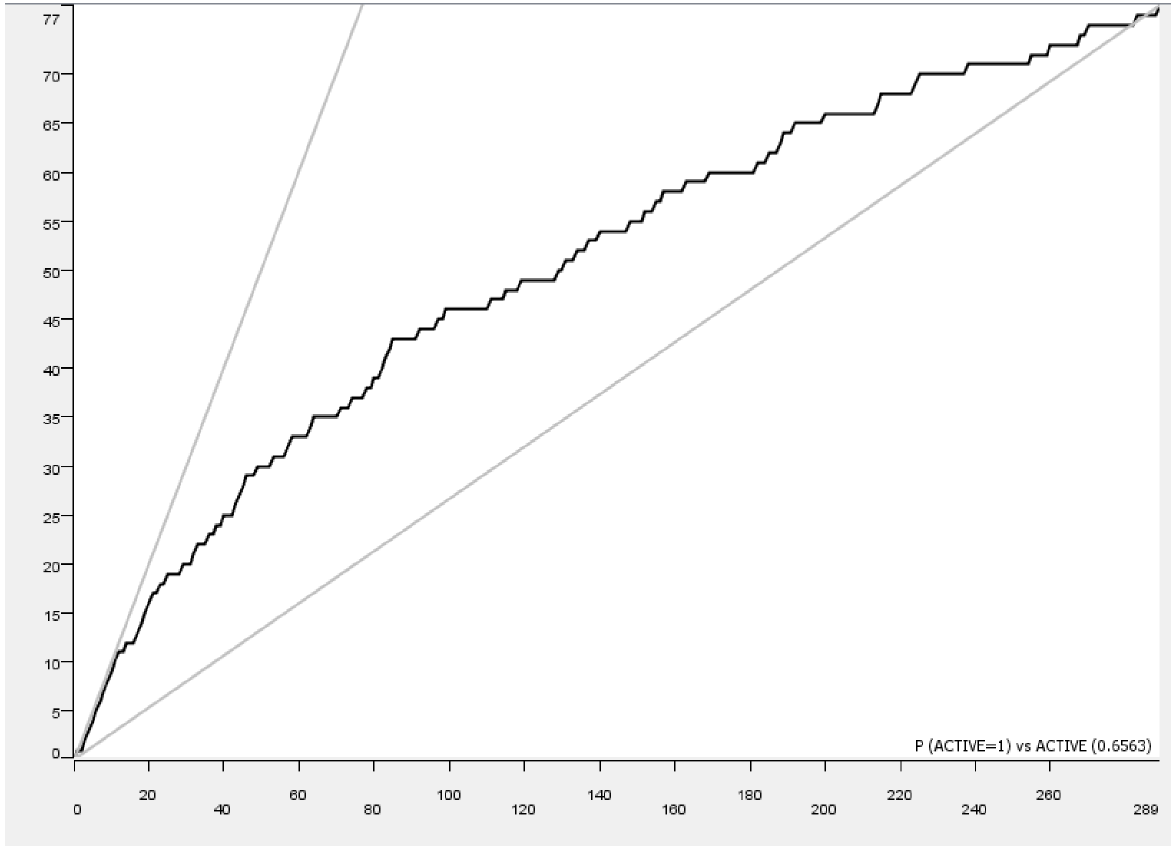

N=289

**Top 10% Mean IC50**	**1,200 nM**

	Predicted Active	Predicted Inactive
Active	45	32
Inactive	52	160

PNN - <15 nM DefGood in c-Abl, 30% error ; Random seed = 429

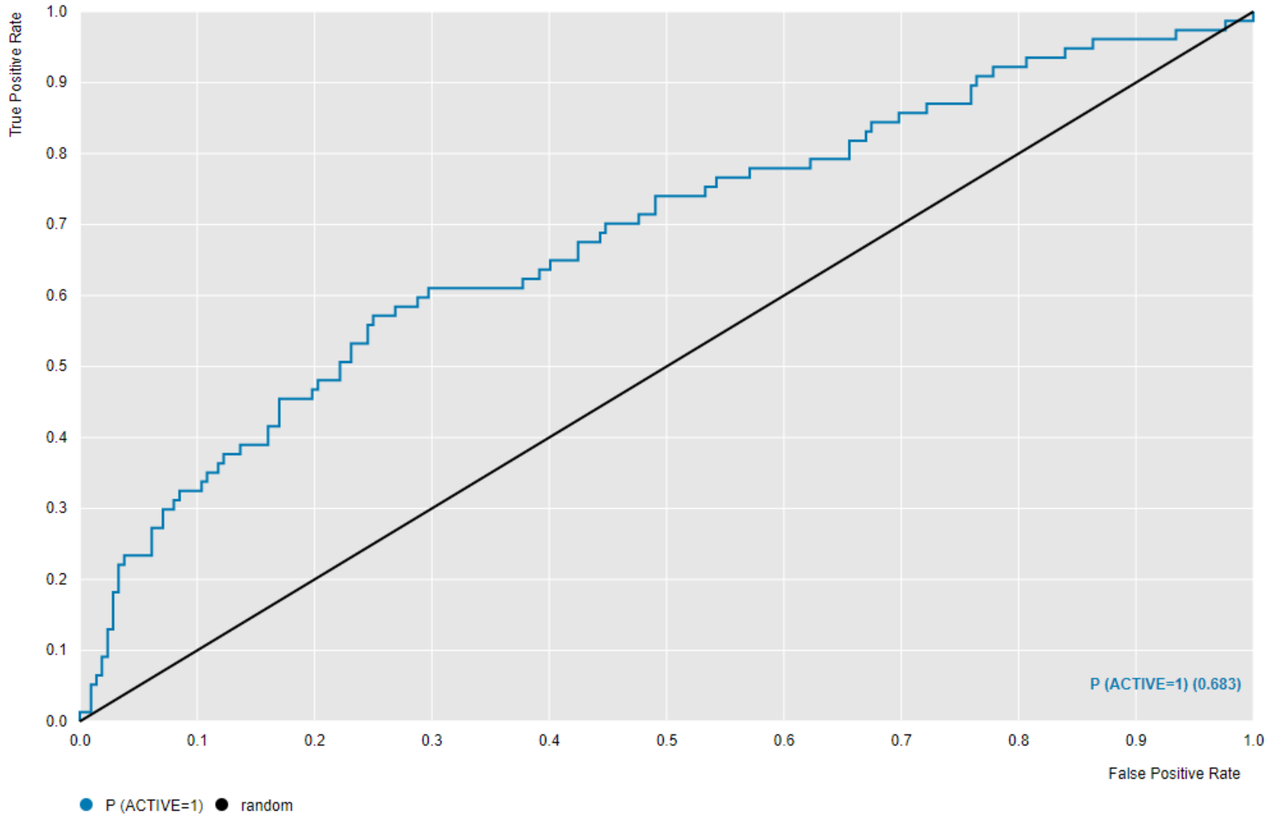

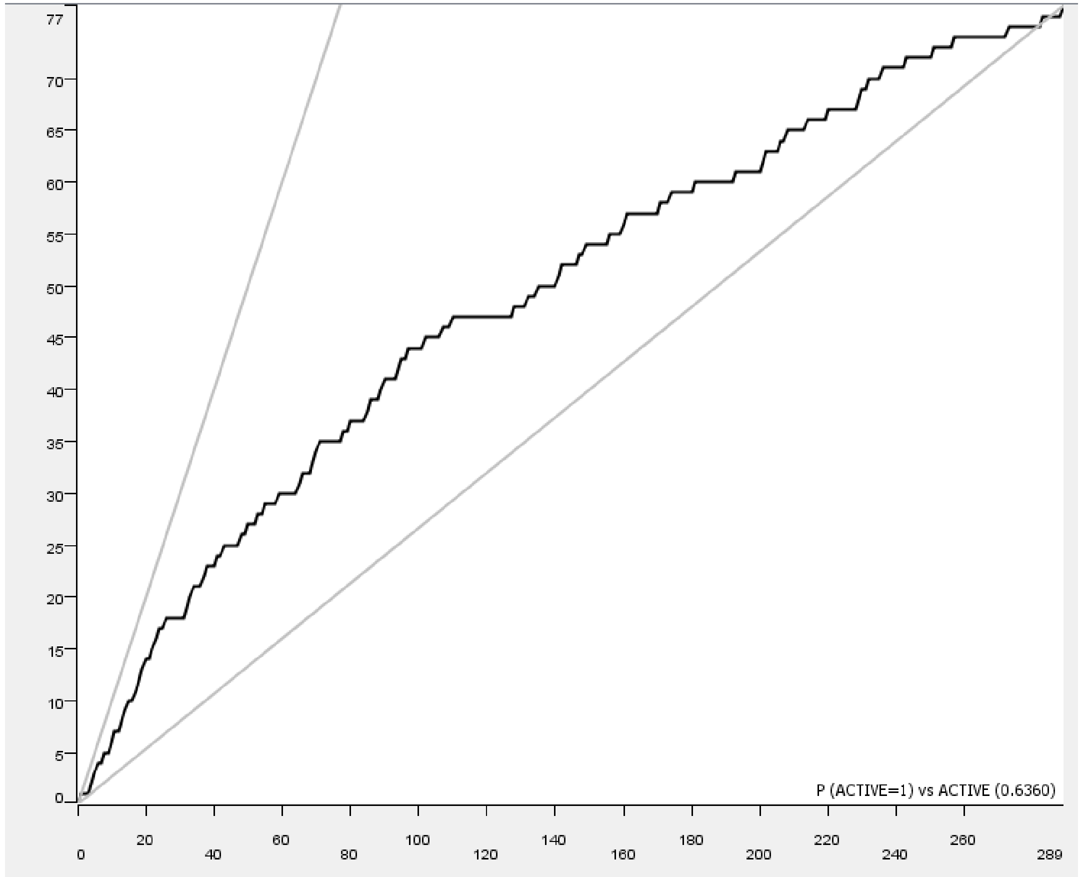

N=289

	Predicted Active	Predicted Inactive
Active	46	31
Inactive	61	151

**Top 10% Mean IC50**	**840 nM<br>Several uM mistakes**

PNN - <15 nM DefGood in c-Abl, 5% error; Random seed = 121783

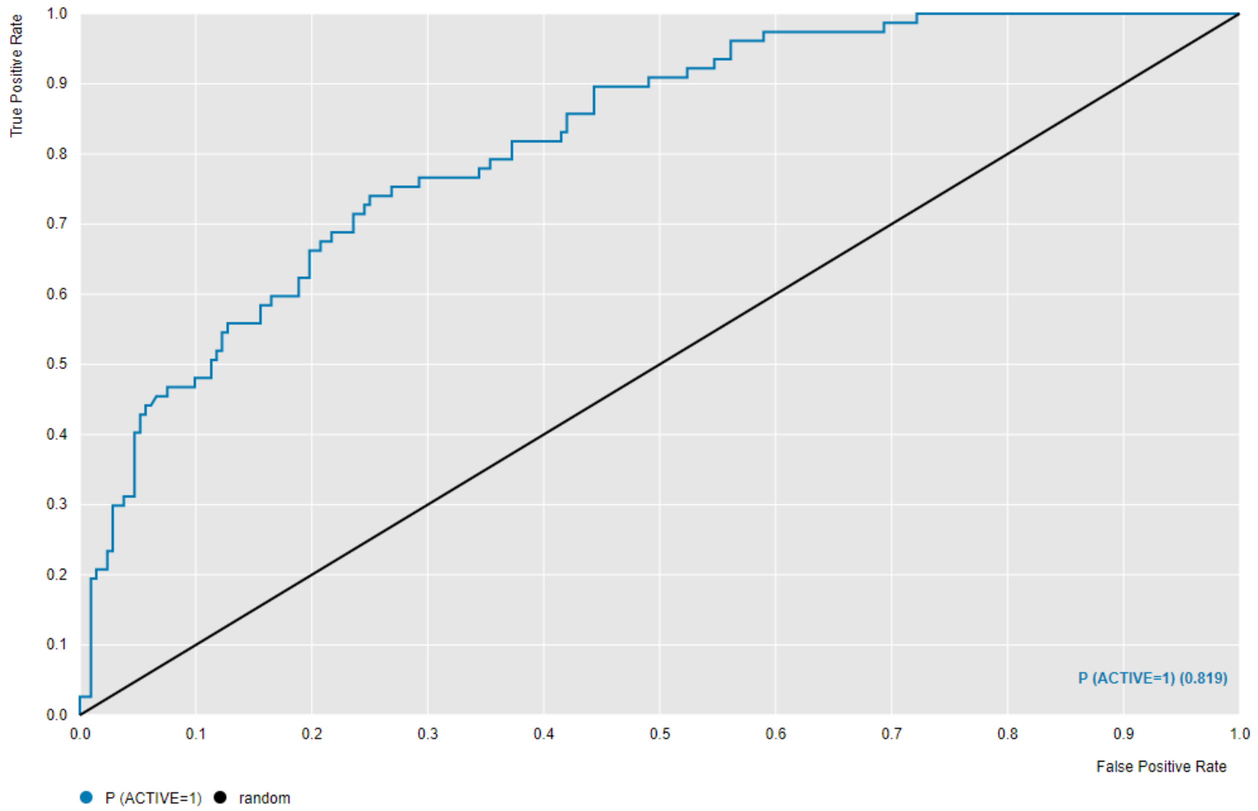

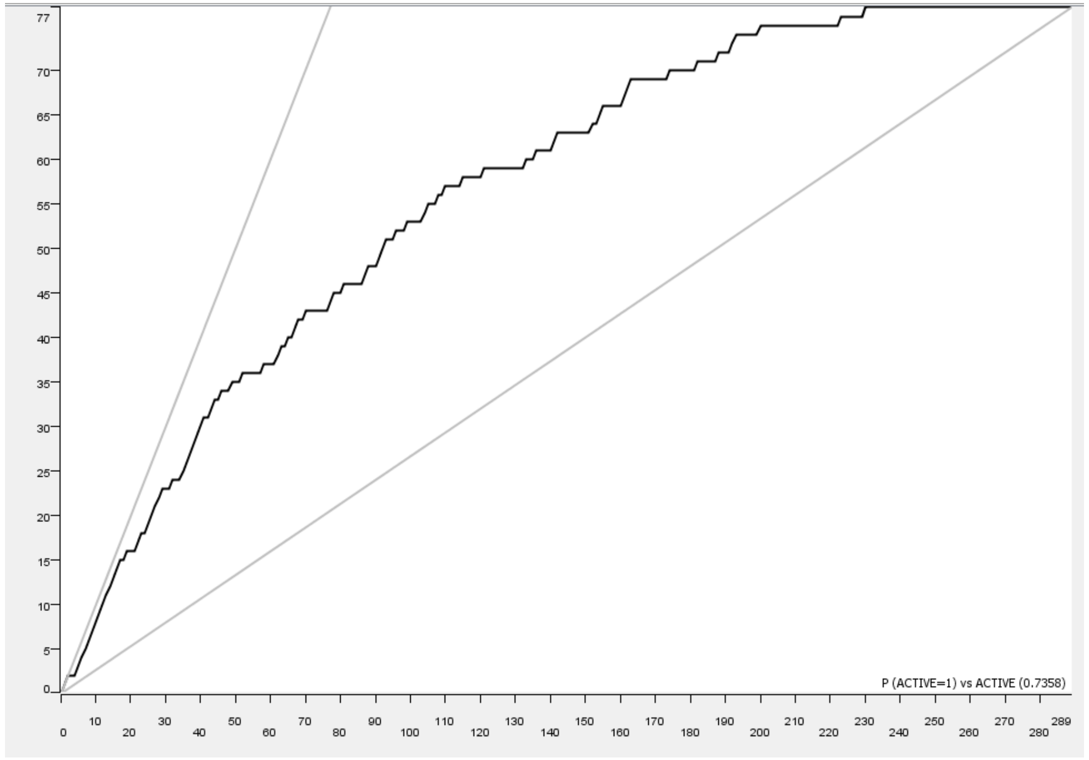

N=289

**Top 10% Mean IC50**	**110 nM (two >800 nM compounds)**

	Predicted Active	Predicted Inactive
Active	11	66
Inactive	2	210

PNN - <15 nM DefGood in c-Abl, 10% error; Random seed = 121783

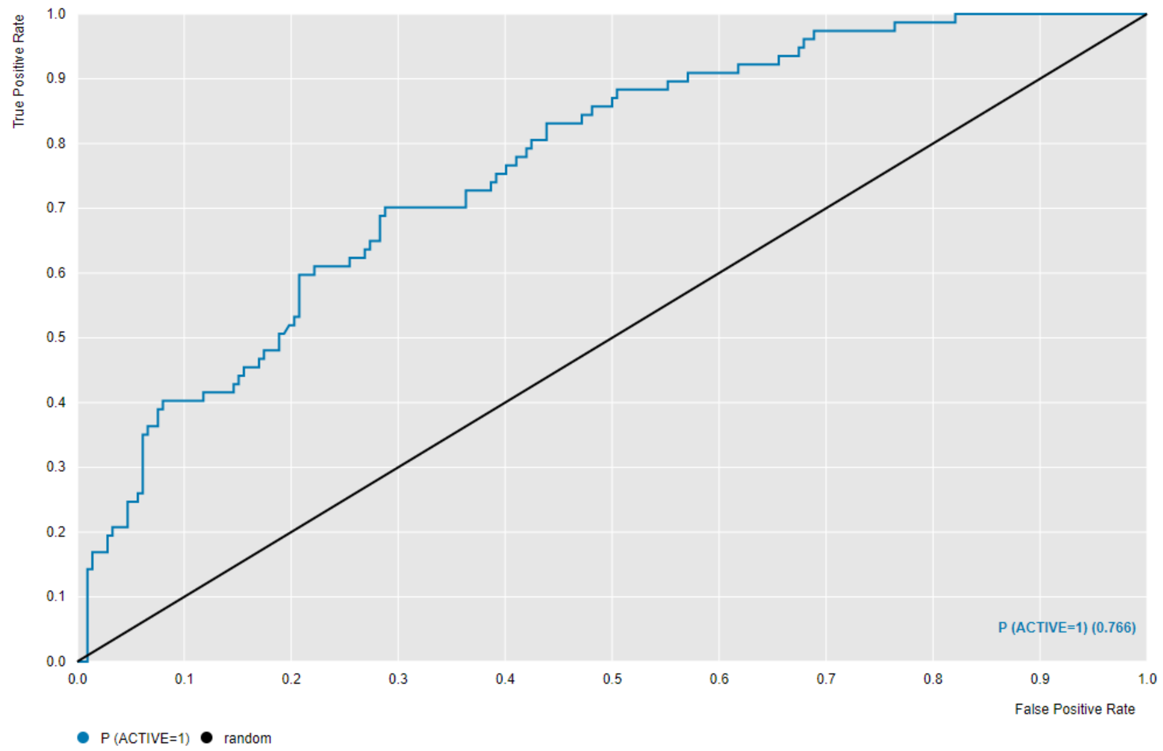

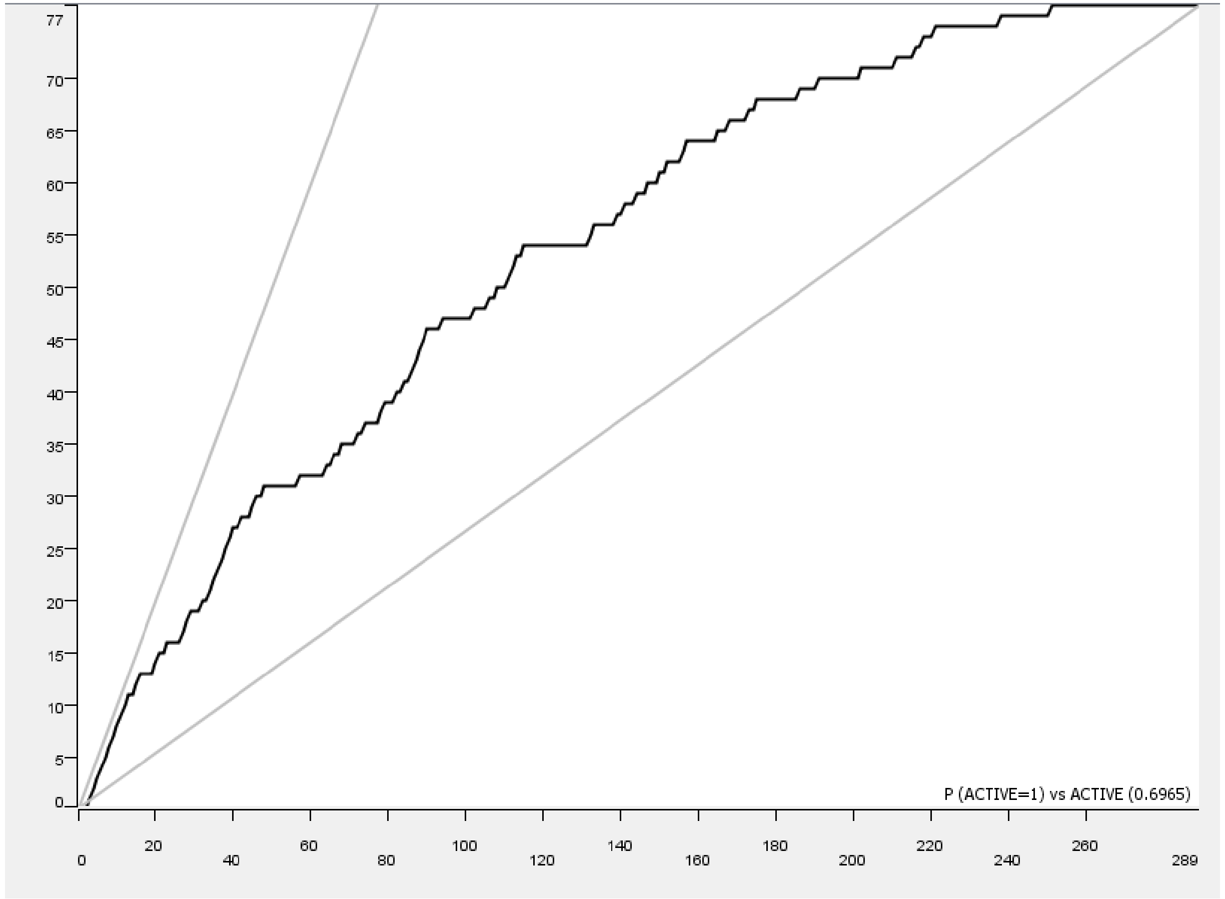

N=289

Top 10% Mean IC50	370 nM (several severe mistakes)

	Predicted Active	Predicted Inactive
Active	8	69
Inactive	2	210

PNN - <15 nM DefGood in c-Abl, 15% error; Random seed = 121783

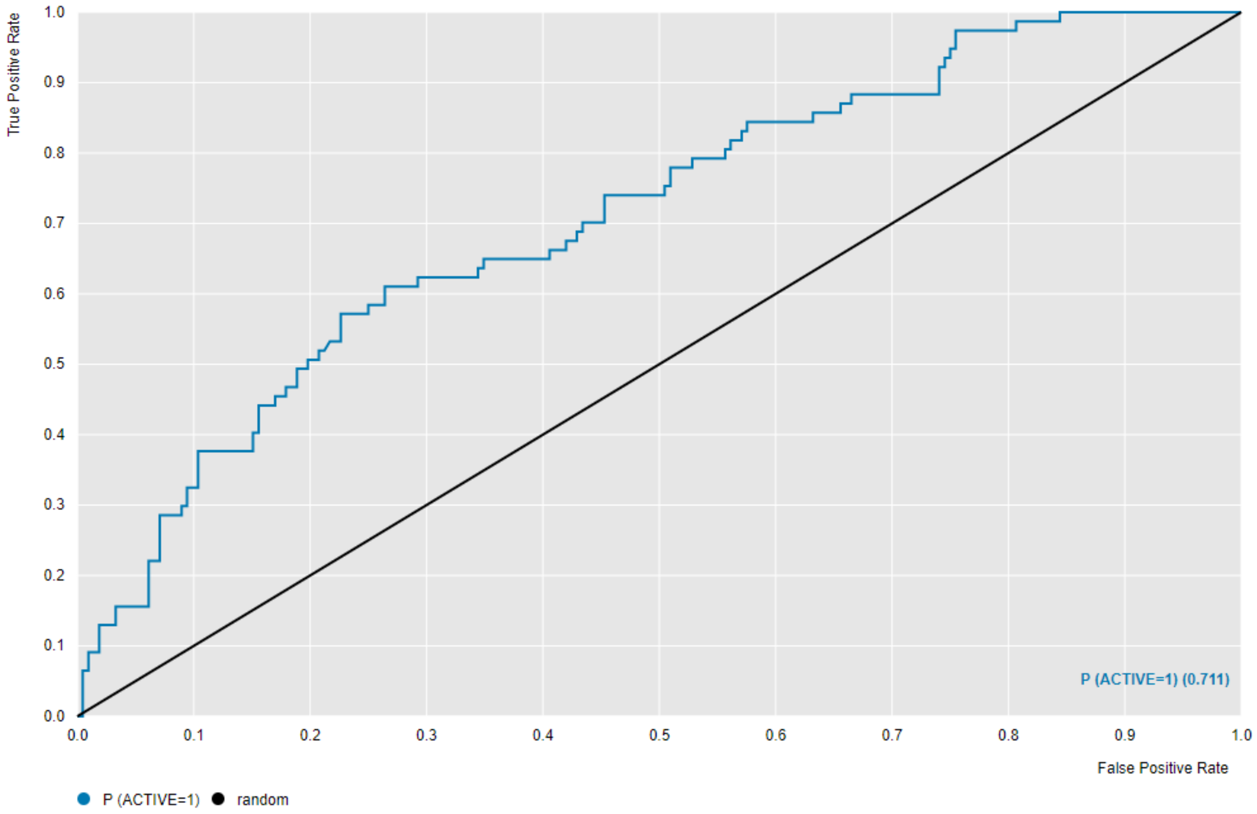

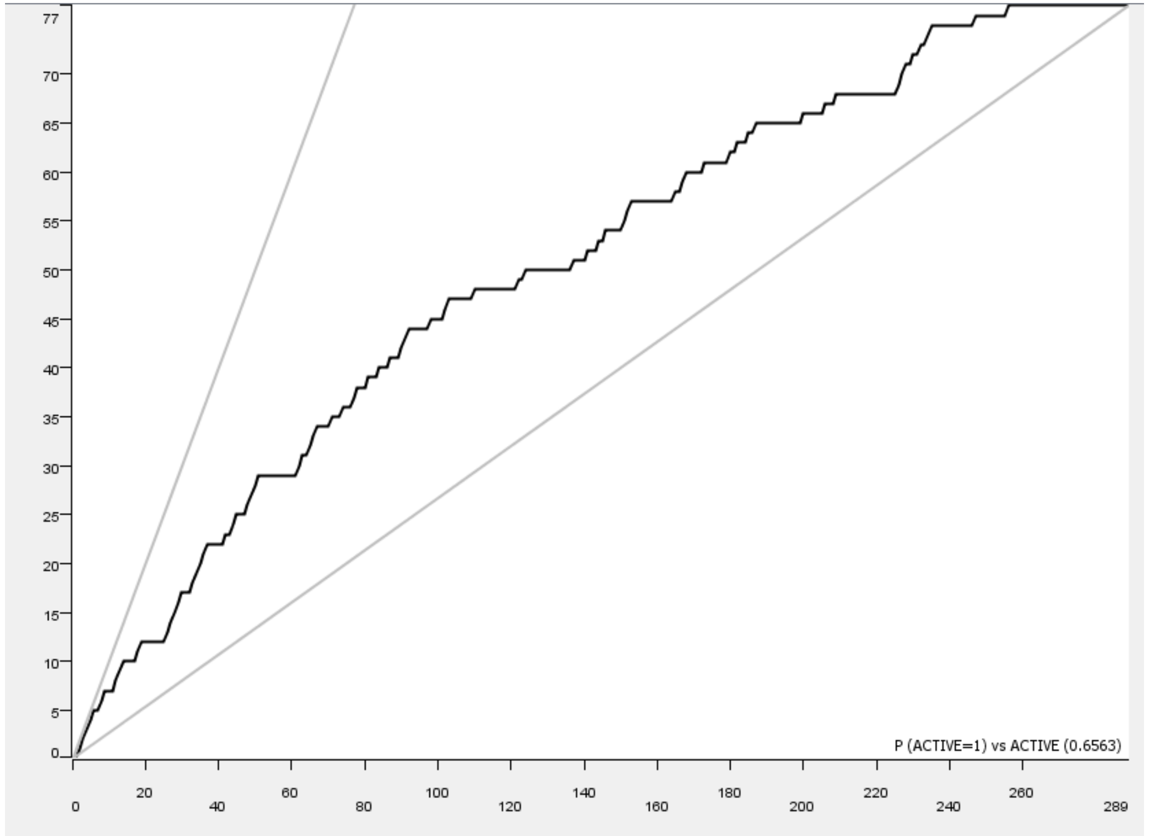

N=289

**Top 10% Mean IC50**	**2,500 nM**

	**Predicted Active**	**Predicted Inactive**
Active	5	72
Inactive	2	210

Reparameterization Control - 15% error; Random seed = 121783

- Theta minus – 0.05
- Theta Plus – 0.85
- Reparameterization failed to recover predictivity Mean 10% IC50 – 2,500 nM

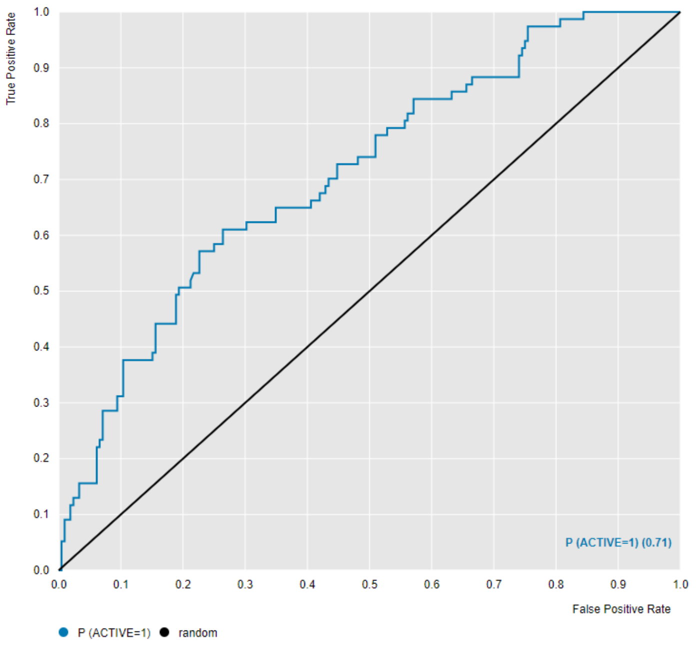

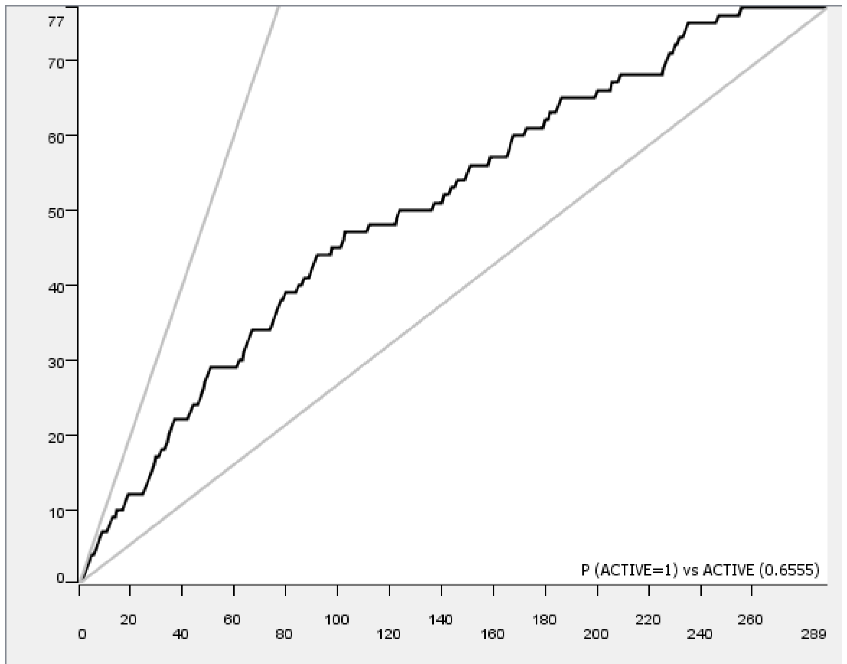

Conclusion - PNN

- Across three random seeds, the % error that lead to a significant specificity failure, enrichment failure and/or top 10% IC50 increase in the retrospective test was 30%, 25% and 15% for each unique random seed.
- Reparameterization failed to rescue the 15% learner.

Activated Xa

Factor Xa NBN Design

- Rules - $RELATION$ = "=" OR $IC50$ > 999 OR $IC50$ < 10 => TRUE
- Sort ascending and remove duplicates
- DefGood scanned between 2.5 and 25 nM
- 80-20 split for both actives and inactives
- NBN – ECFP4 used Classifier where Prediction was Active = True
- Resulted in 1657 compounds after cleaning the data

<2.5 nM DefGood in Xa

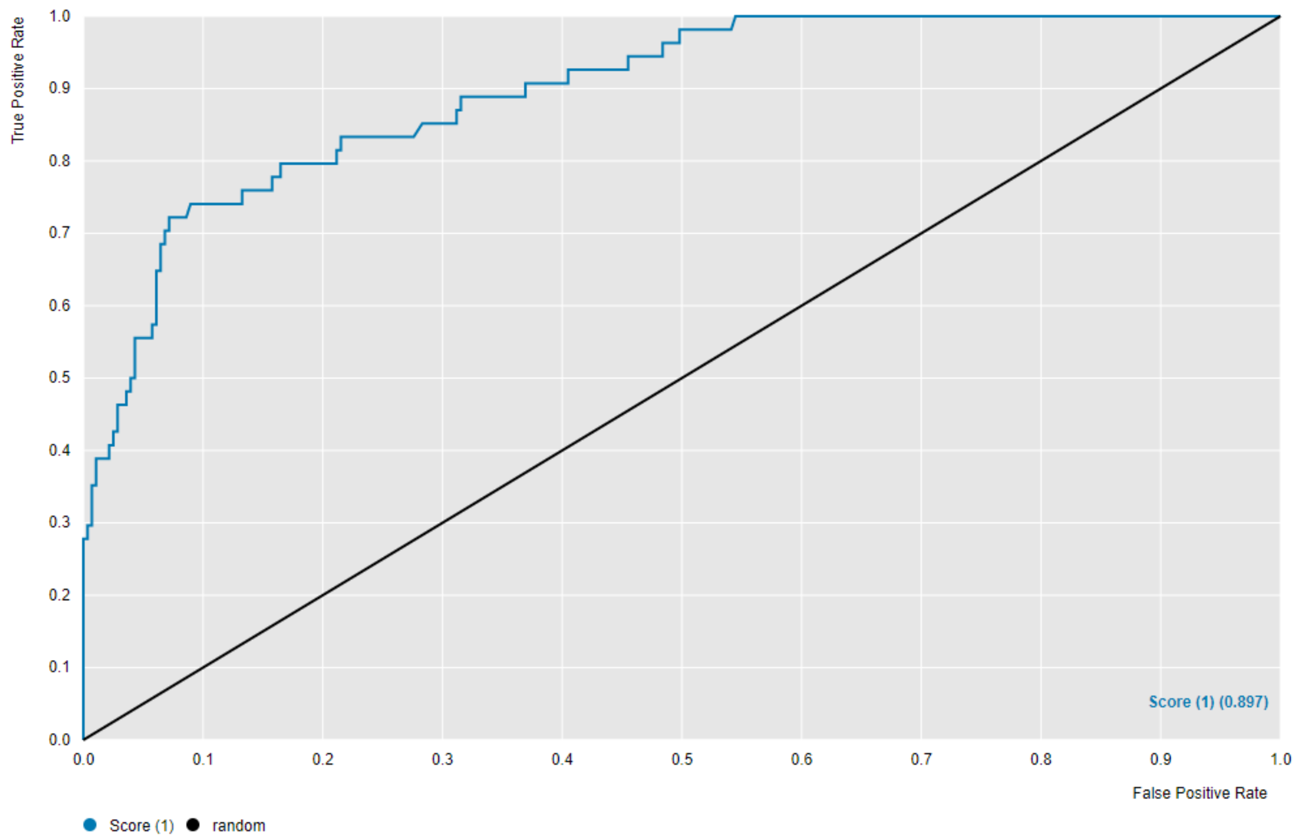

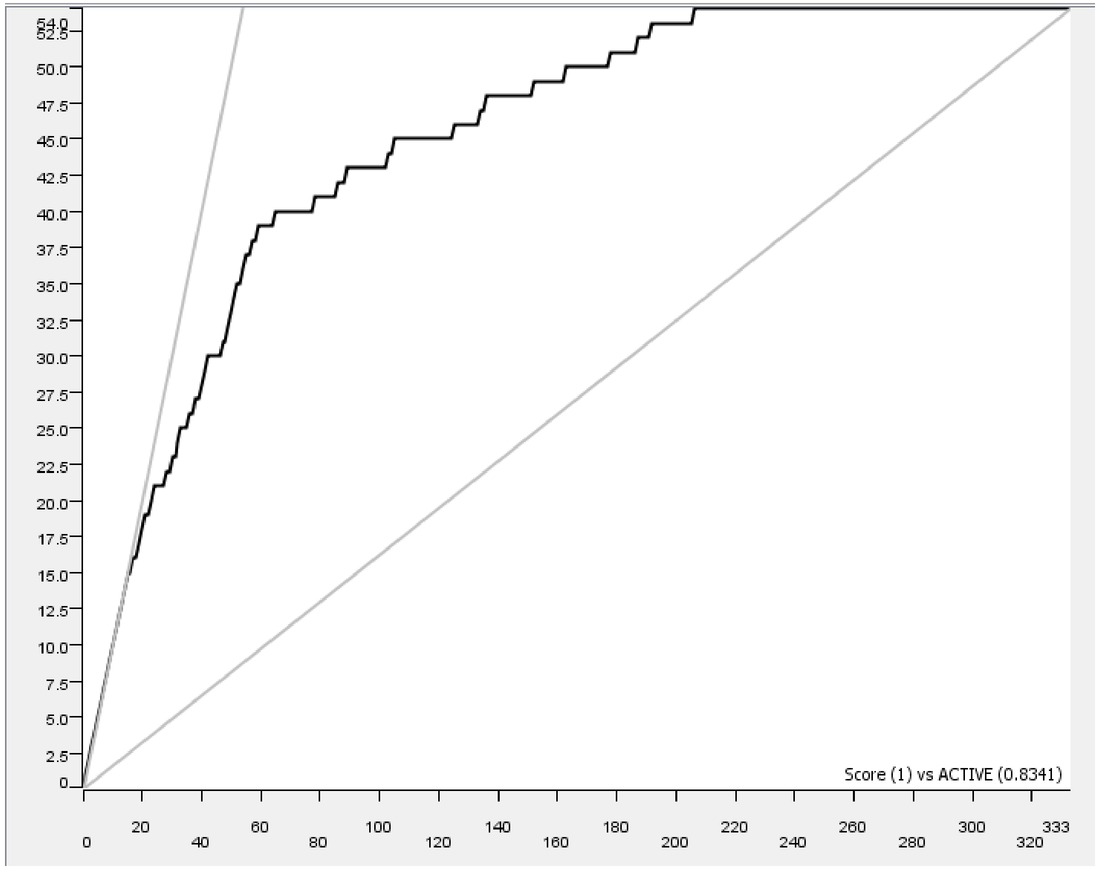

N=332

Top 10% Mean IC50	3.2 nM

	Predicted Active	Predicted Inactive
Active	43	11
Inactive	52	227

<5 nM DefGood in Xa

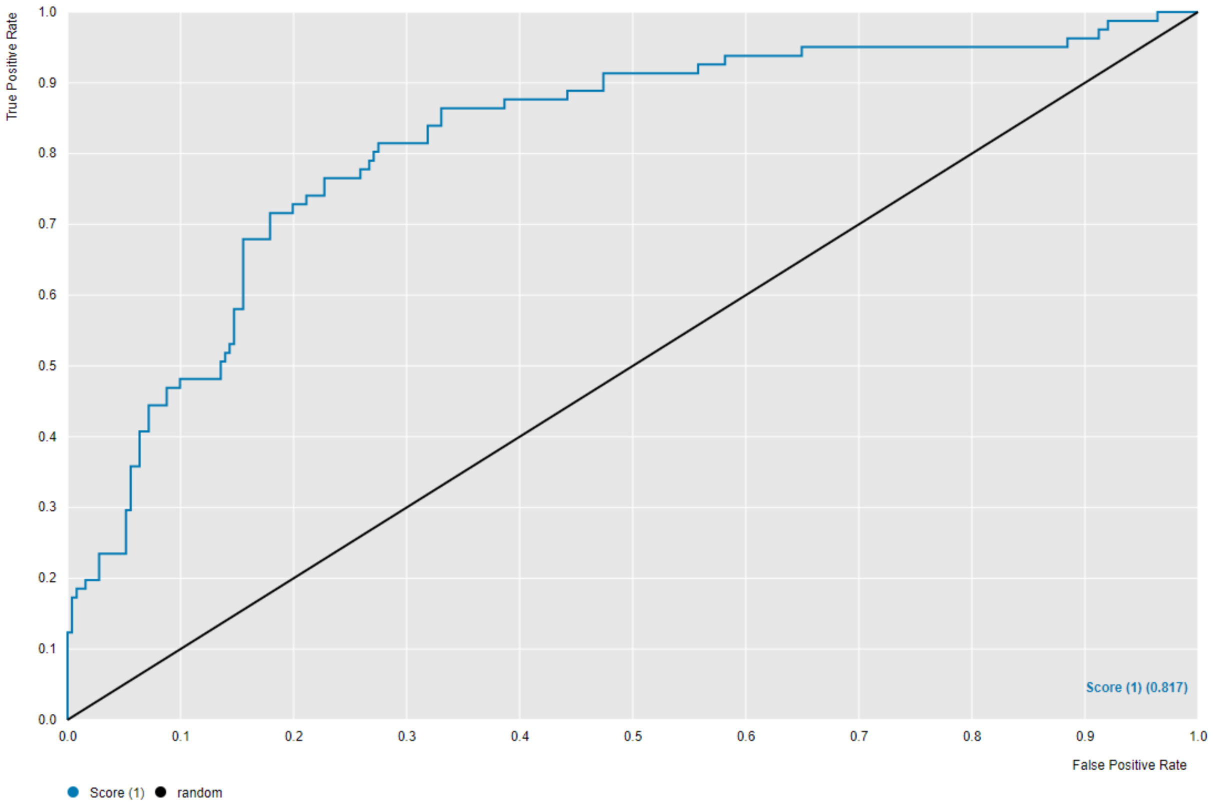

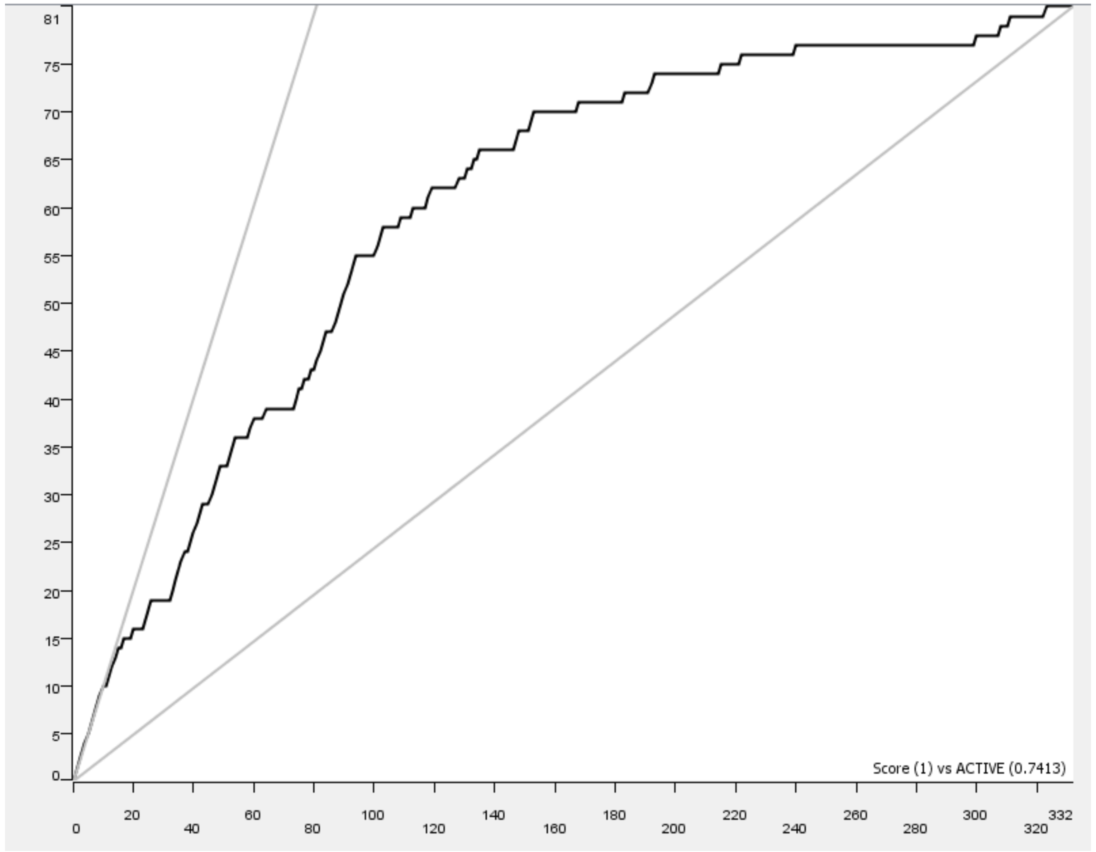

N=332

Top 10% Mean IC50	9.9 nM

	Predicted Active	Predicted Inactive
Active	66	15
Inactive	69	182

<10 nM DefGood in Xa

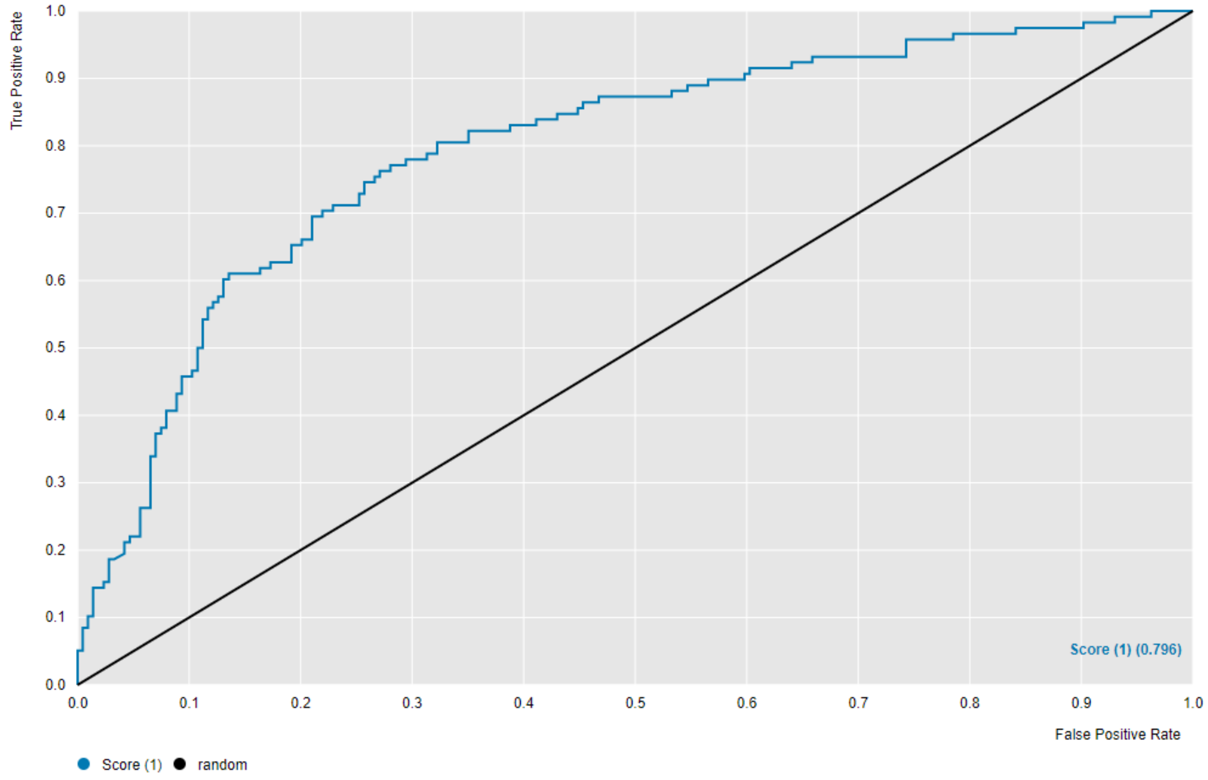

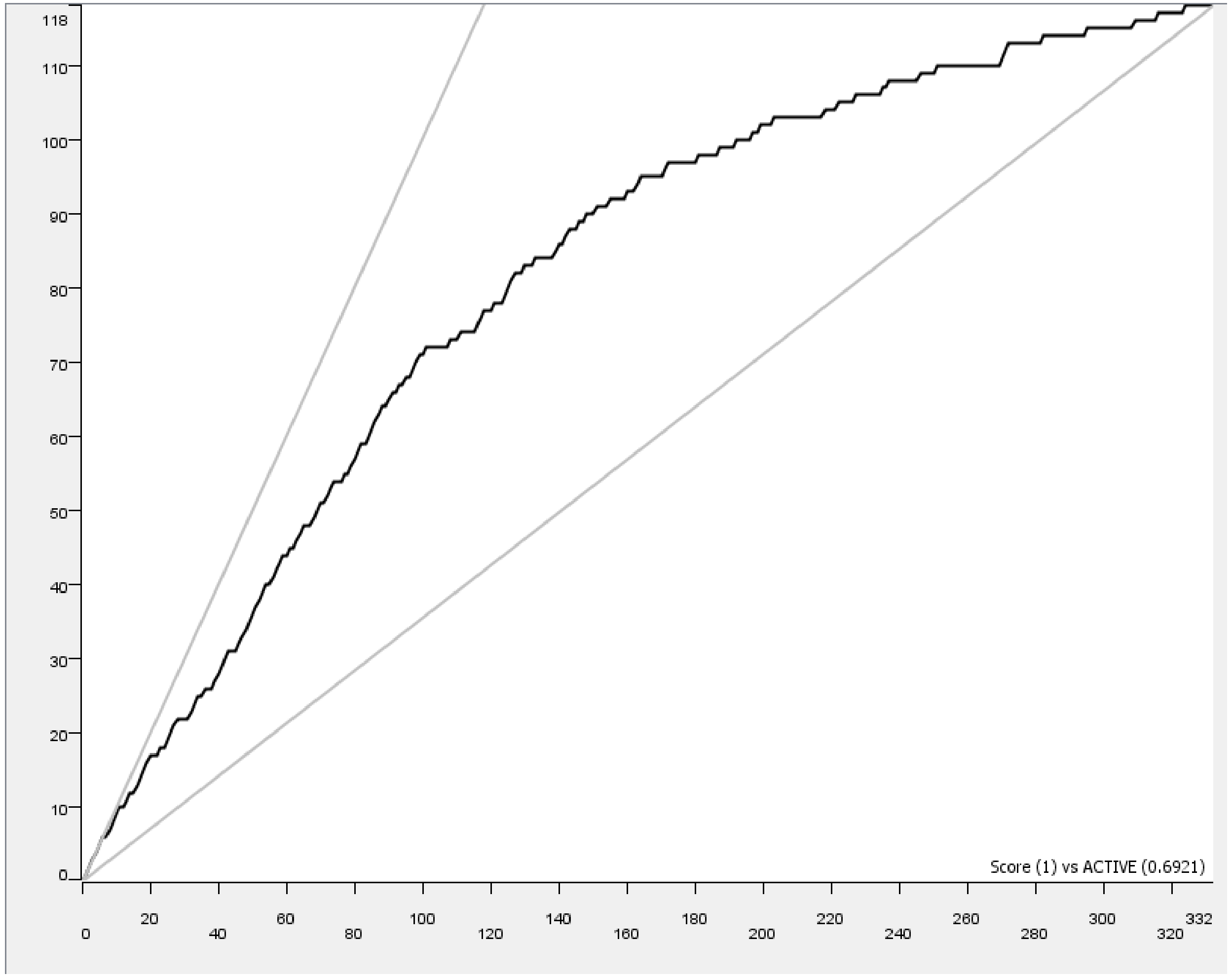

N=332

Top 10% Mean IC50	11 nM

	Predicted Active	Predicted Inactive
Active	83	35
Inactive	47	167

<15 nM DefGood in Xa

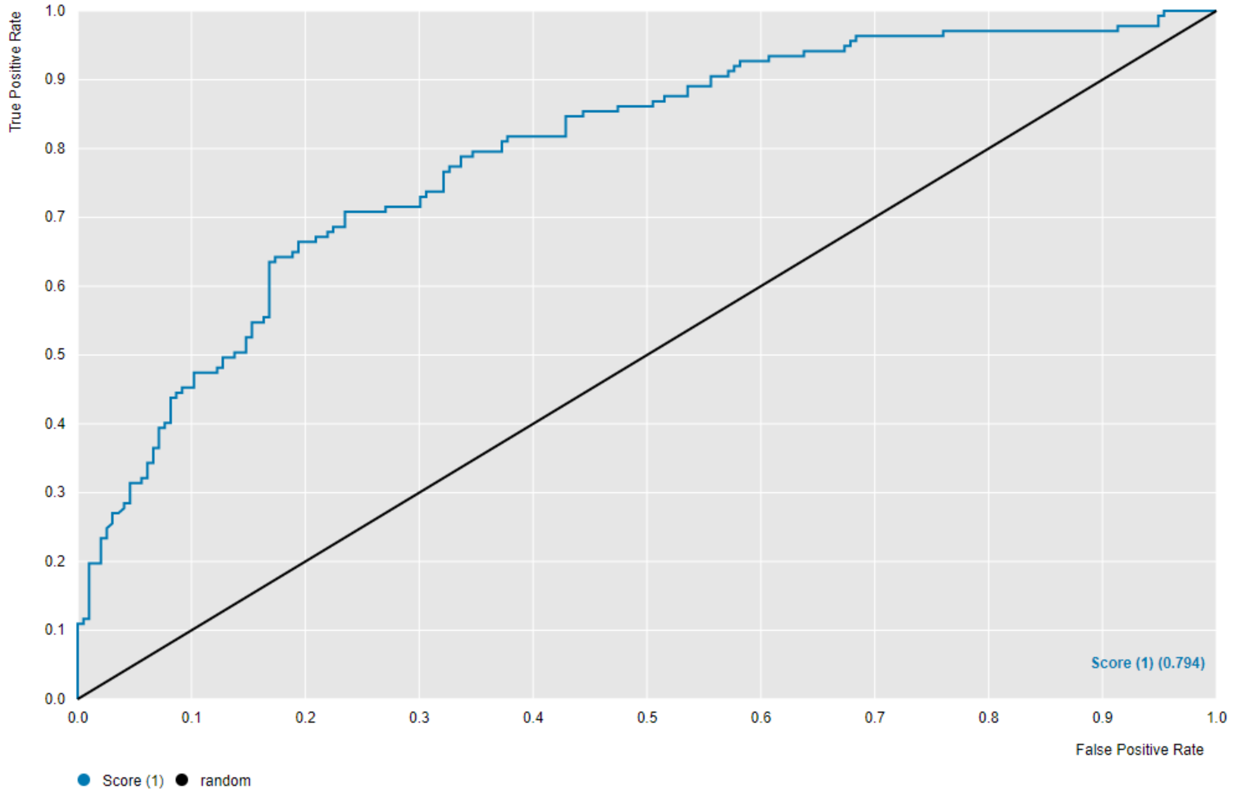

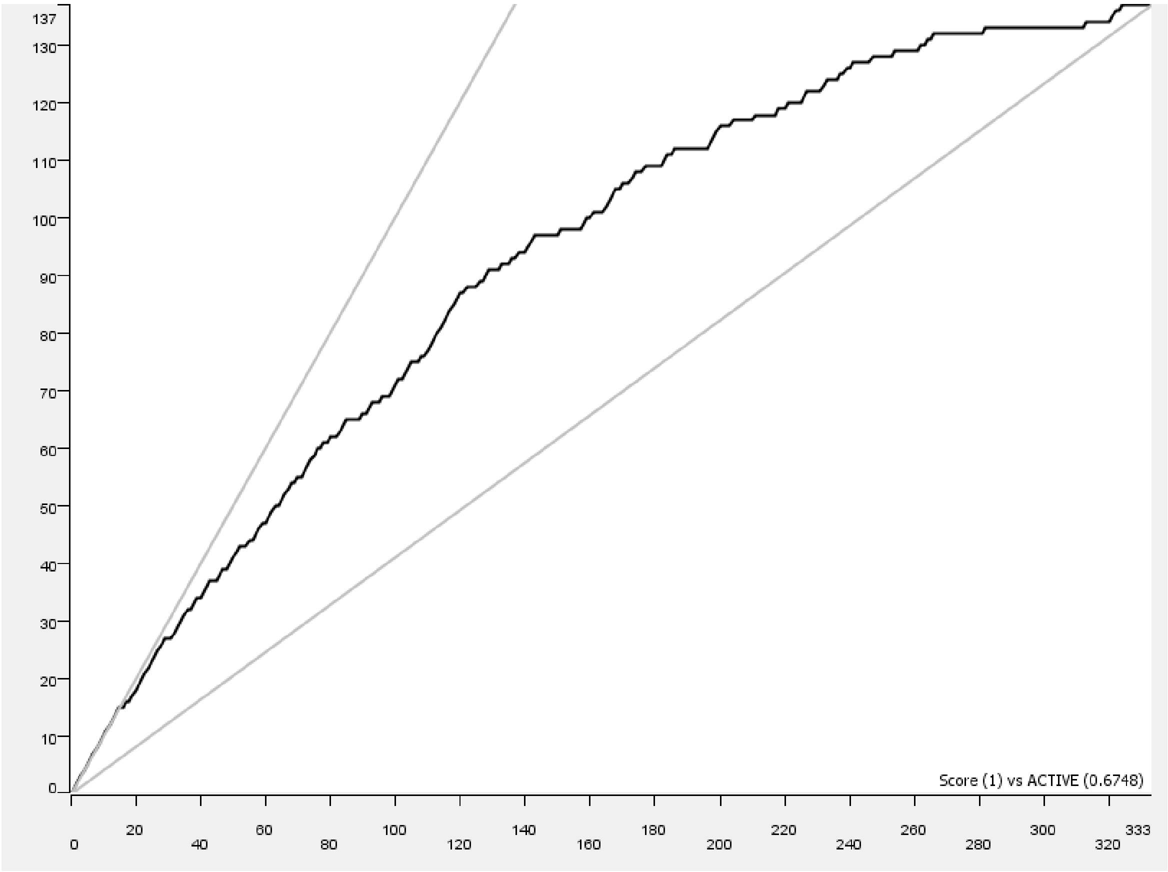

N=333

**Top 10% Mean IC50**	**5.5 nM**

	**Predicted Active**	**Predicted Inactive**
Active	101	36
Inactive	62	134

<20 nM DefGood in Xa

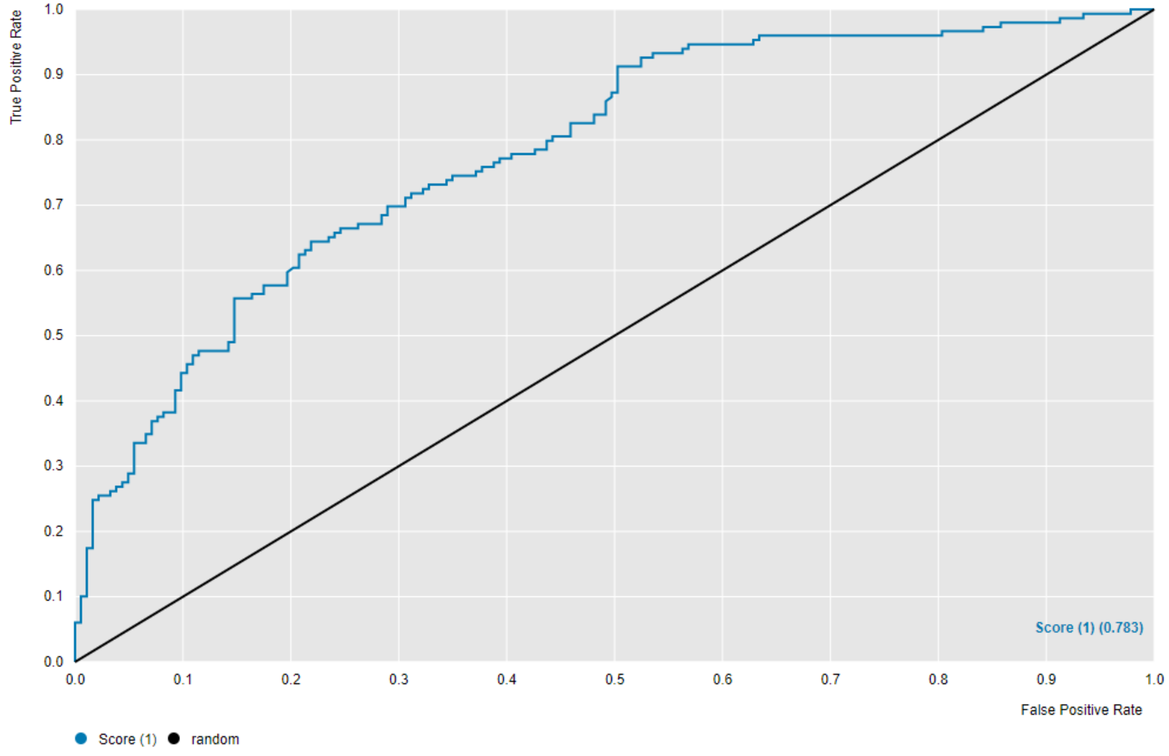

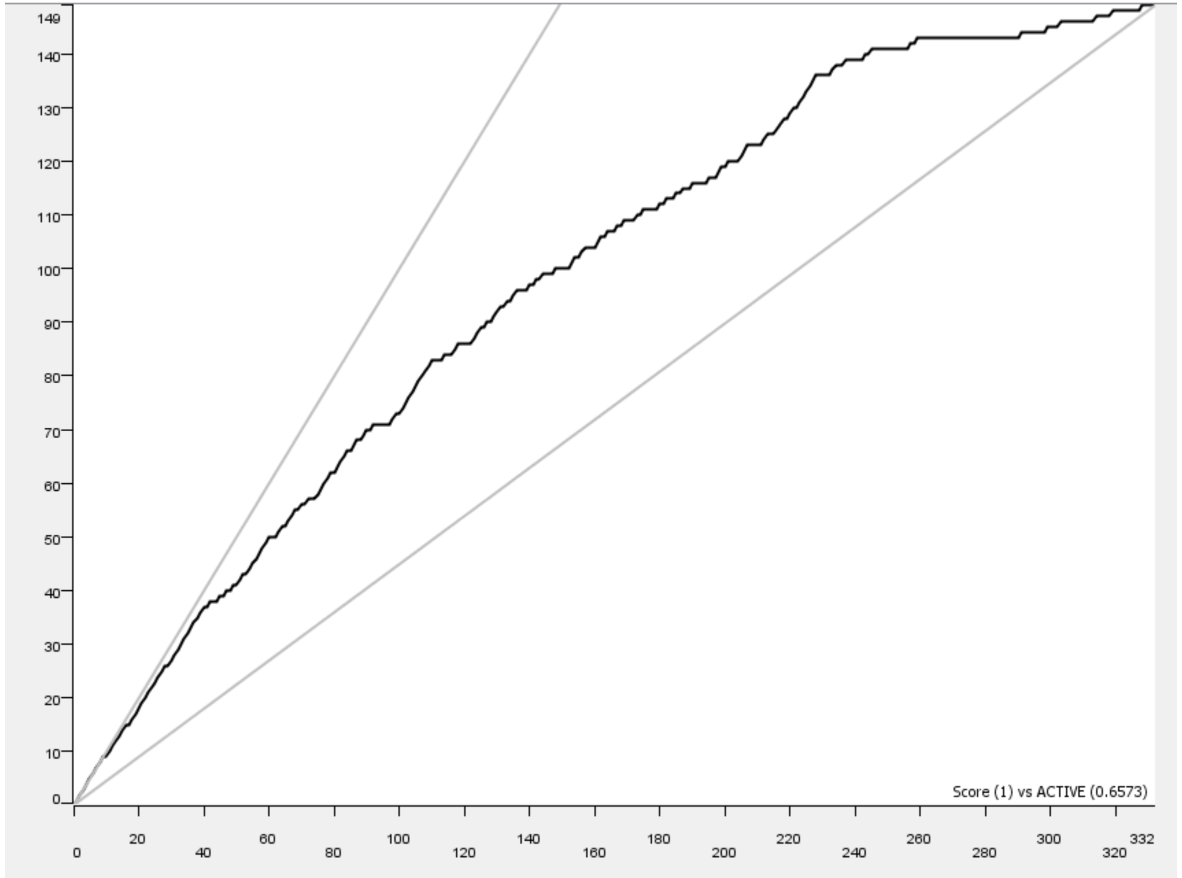

N=332

Top 10% Mean IC50	5.5 nM

	Predicted Active	Predicted Inactive
Active	113	36
Inactive	70	113

Decision on DefGood

- <2.5 nM has the best IC50 mean at top 10% and enrichment. The specificity is the worst compared to the others

RF - <2.5 nM DefGood in Xa

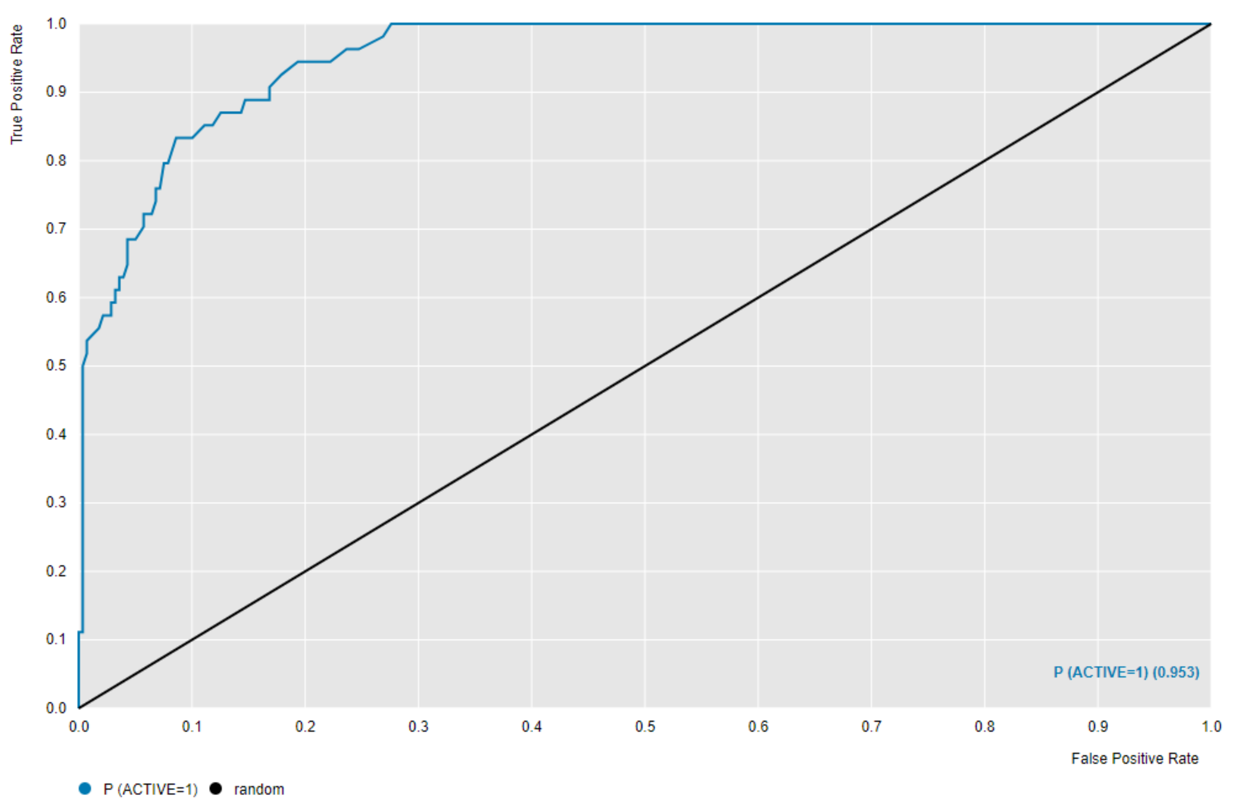

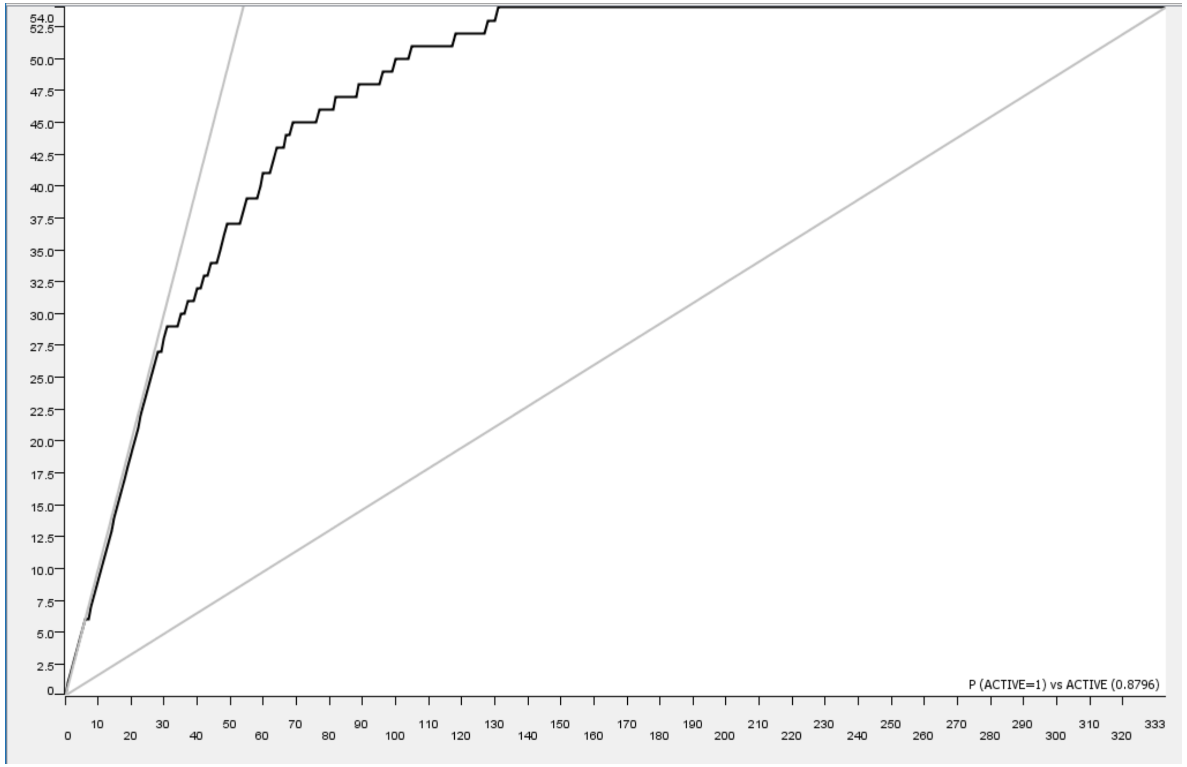

N=289

Top 10% Mean IC50	10 nM

	Predicted Active	Predicted Inactive
Active	38	16
Inactive	16	263

PNN - <2.5 nM DefGood in Xa

- Properties List (46 calculatable properties were used)
- Parameterization (Global Accuracy for Activity at a 2.5 nM decision value was used, and a 5-fold stratified sampling cross validation was used to parameterize theta minus and theta plus) Theta Minus = 0.05 and Theta Plus = 0.75

PNN - <2.5 nM DefGood in Xa

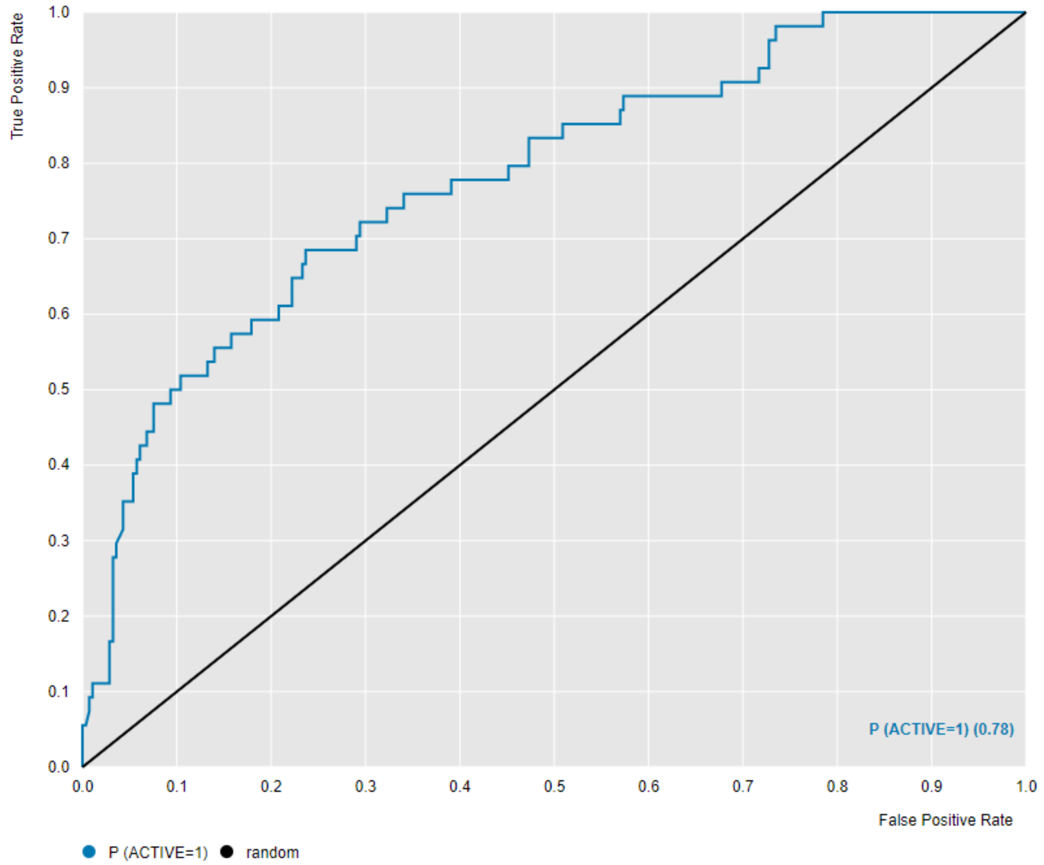

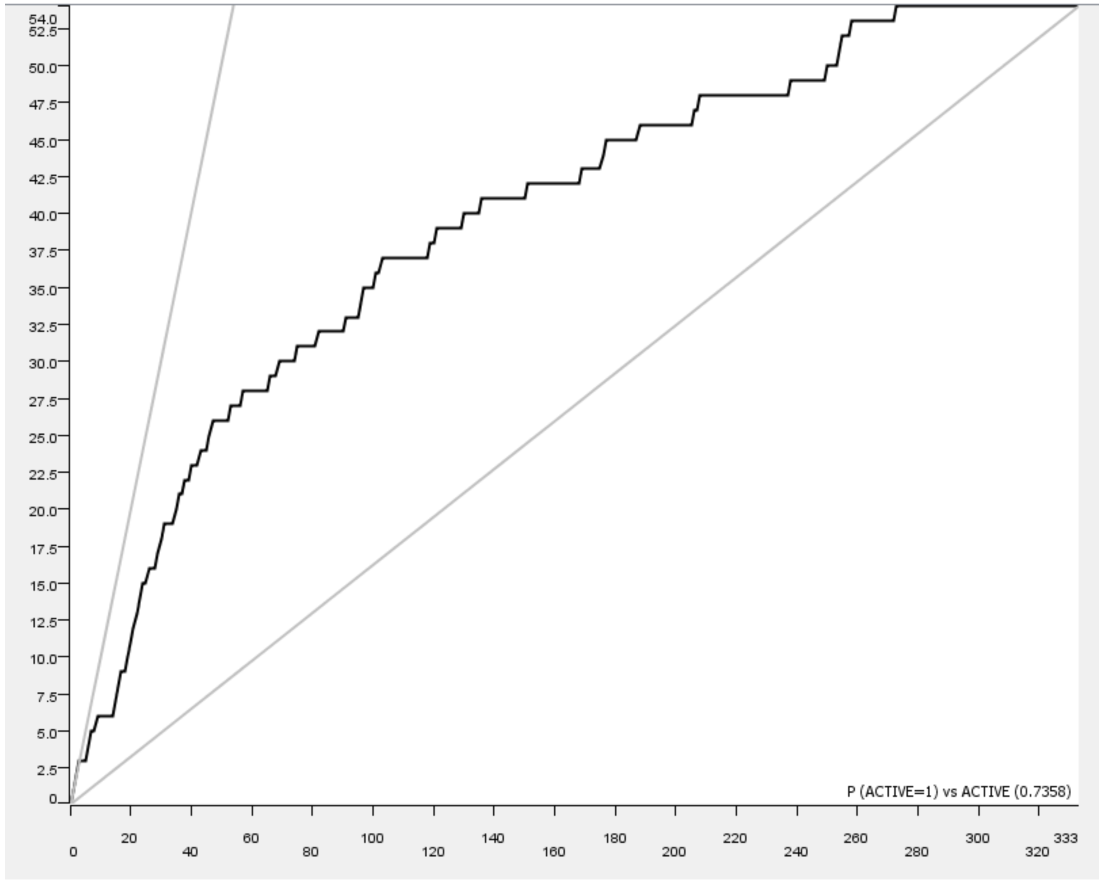

N=333

Top 10% Mean IC50	23 nM

	Predicted Active	Predicted Inactive
Active	18	36
Inactive	12	267

NBN Error Tolerance- <2.5 nM DefGood in Xa

- 0-50% absolute error

<2.5 nM DefGood in Xa, 5% error; Random seed = 1515533876005

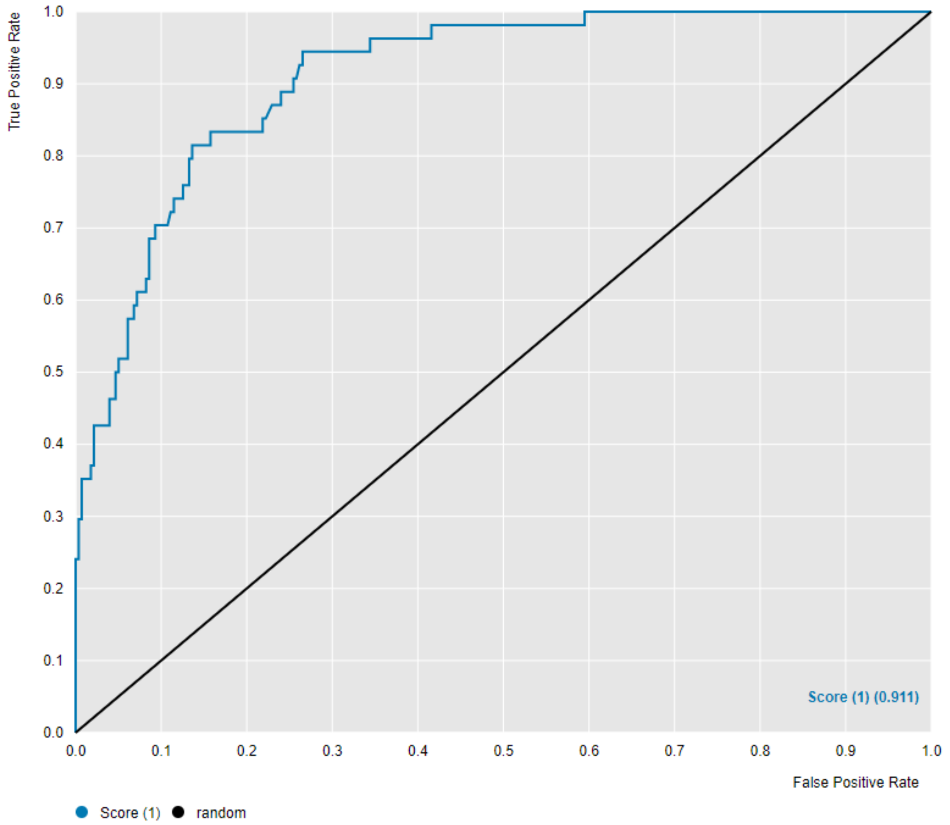

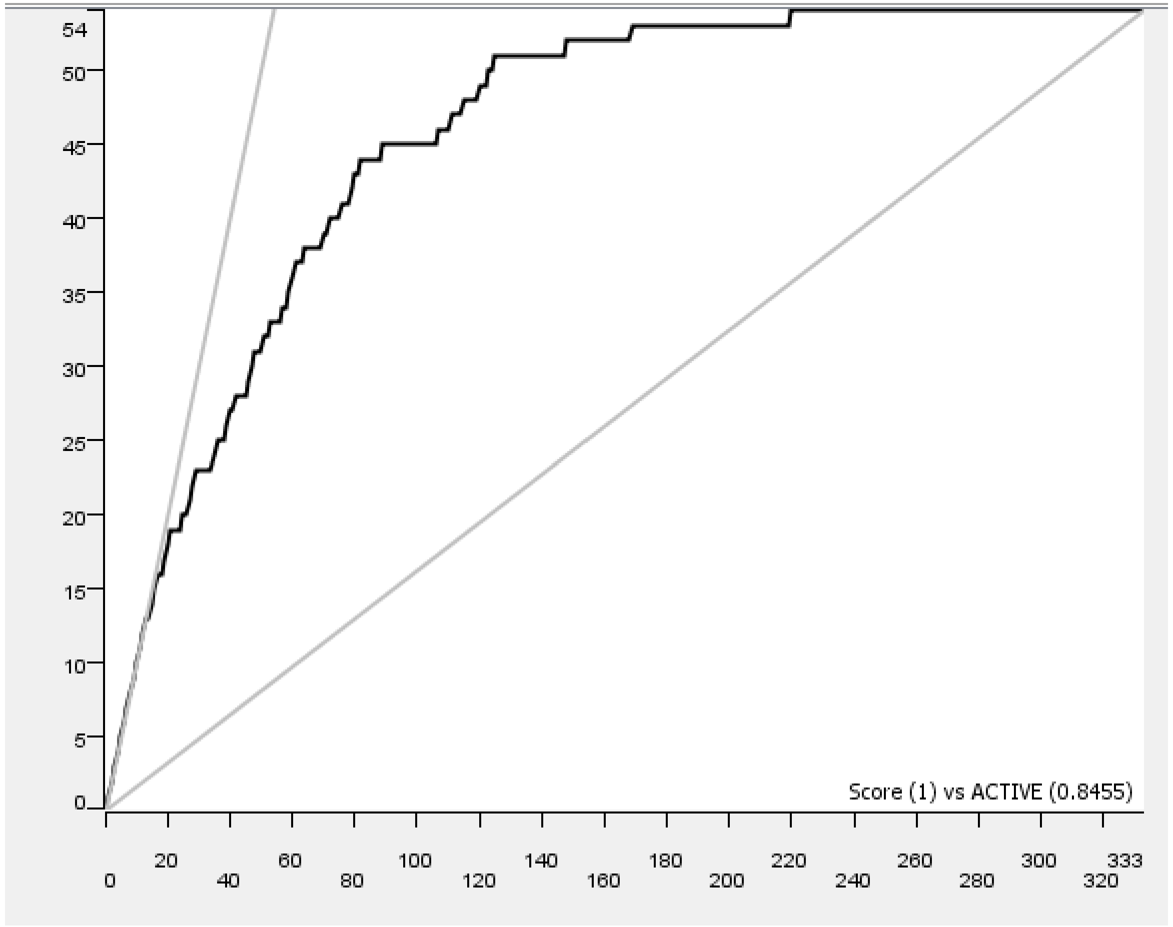

N=333

Top 10% Mean IC50	6.3

	Predicted Active	Predicted Inactive
Active	48	6
Inactive	70	209

<2.5 nM DefGood in Xa, 10% error

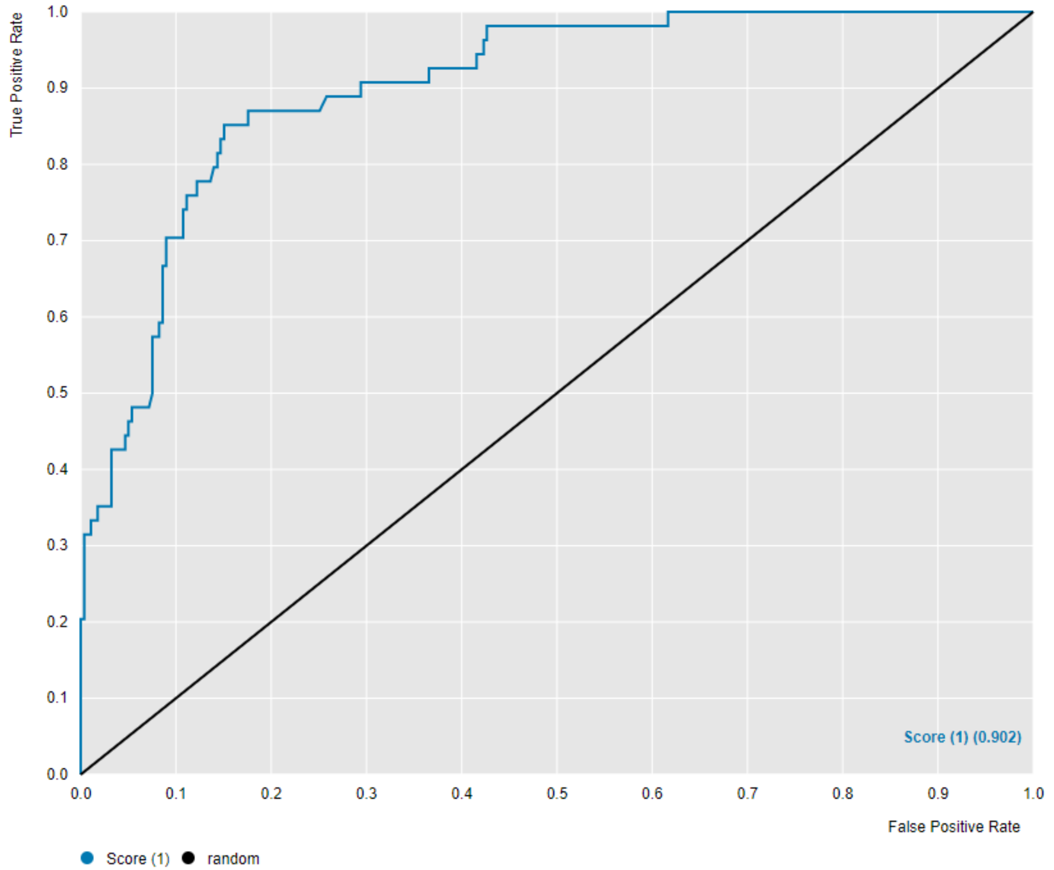

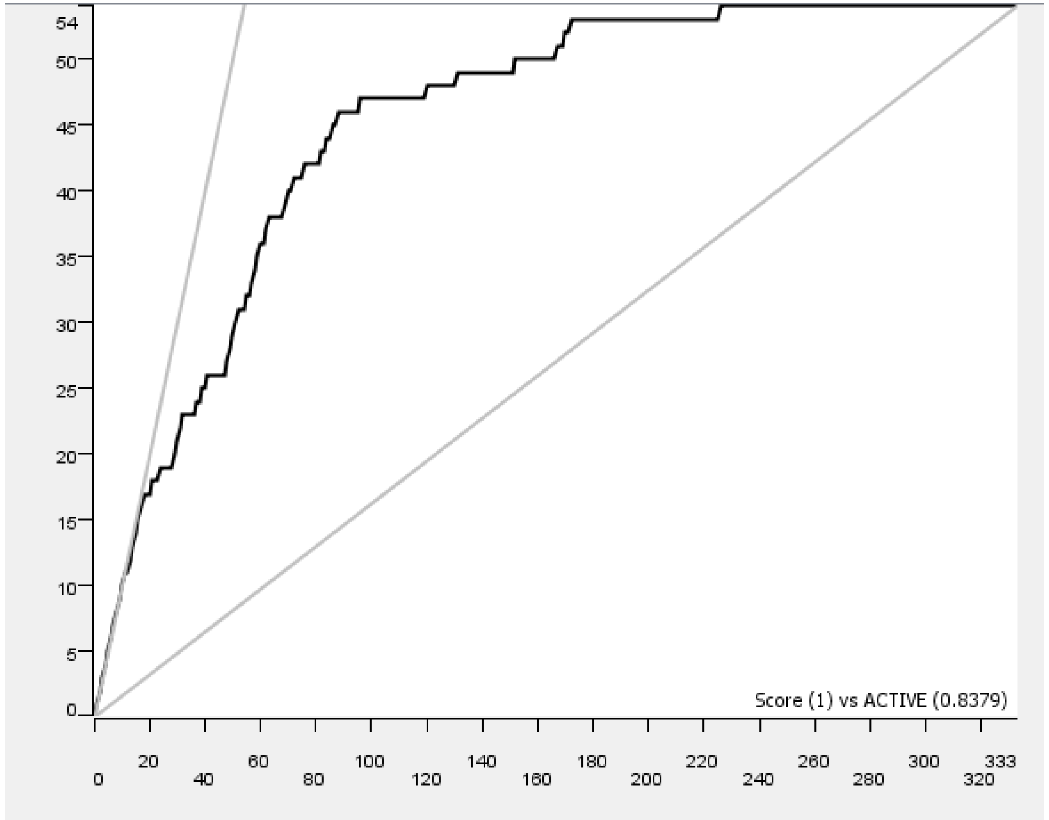

N=333

Top 10% Mean IC50	6.2 nM

	Predicted Active	Predicted Inactive
Active	49	5
Inactive	87	192

<2.5 nM DefGood in Xa, 15% error

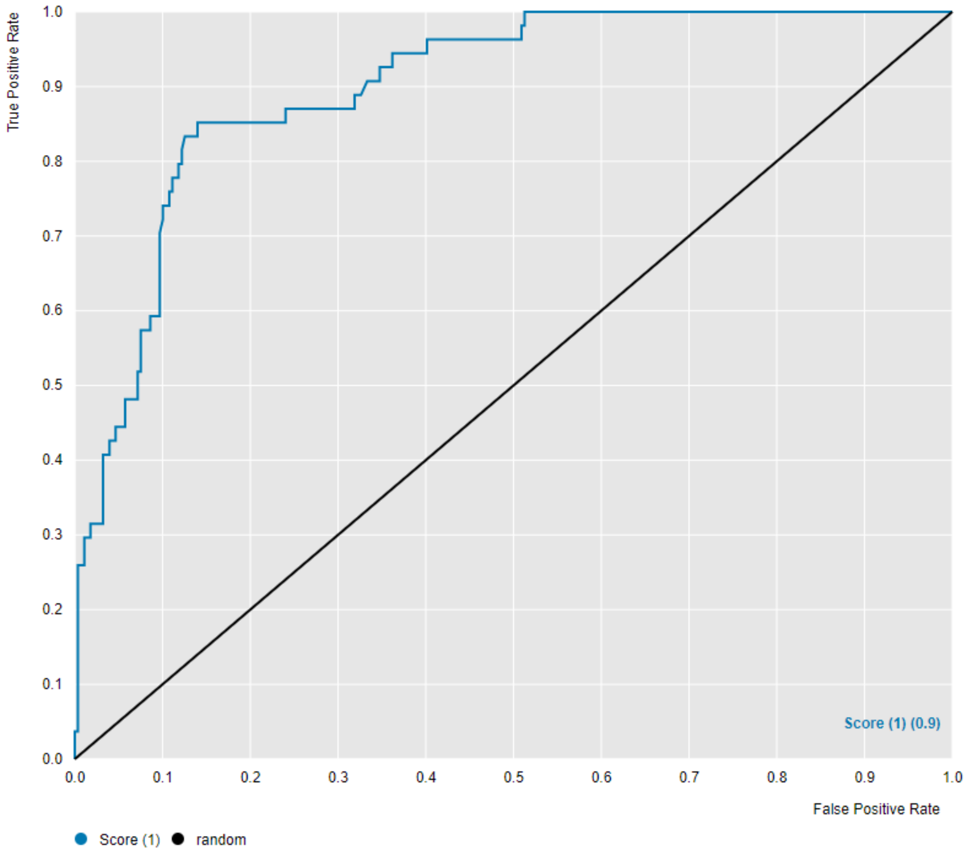

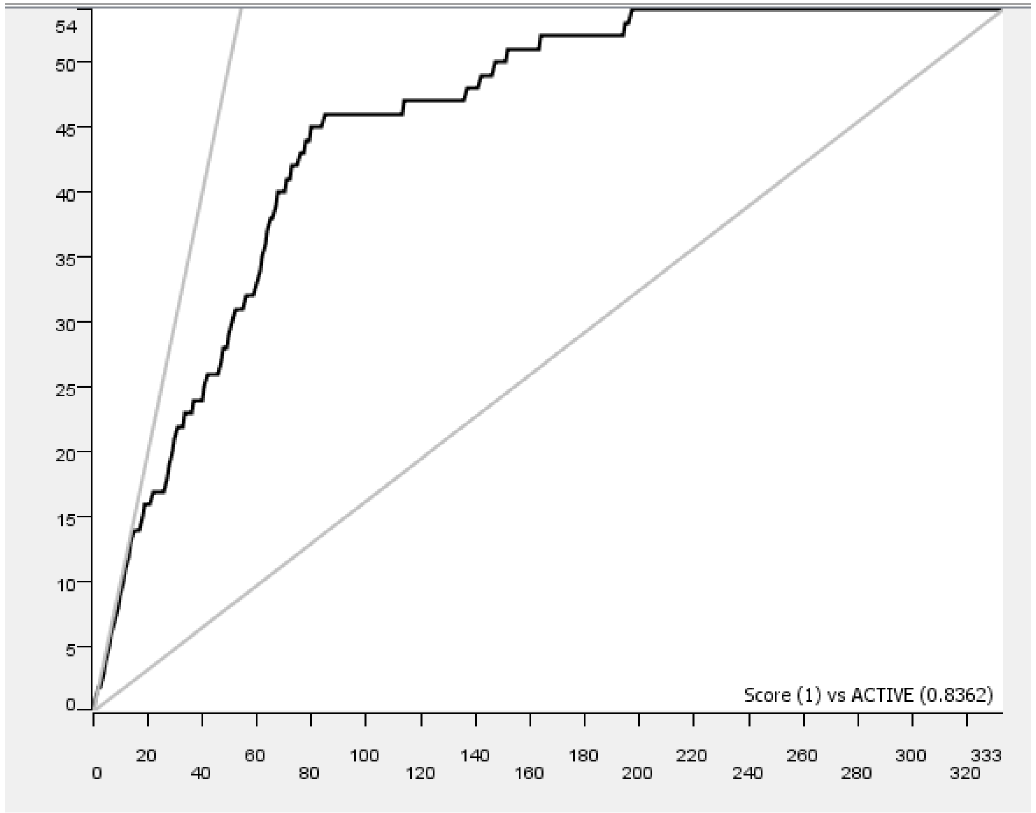

N=333

Top 10% Mean IC50	5.5 nM

	Predicted Active	Predicted Inactive
Active	46	8
Inactive	42	237

<2.5 nM DefGood in Xa, 20% error

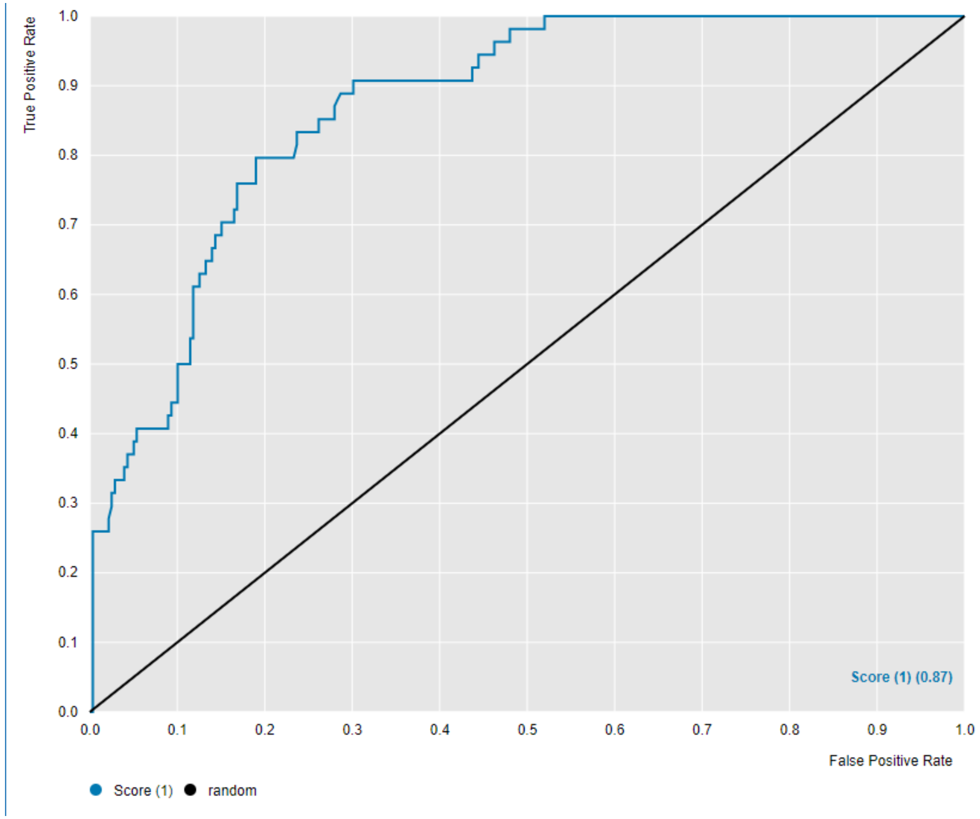

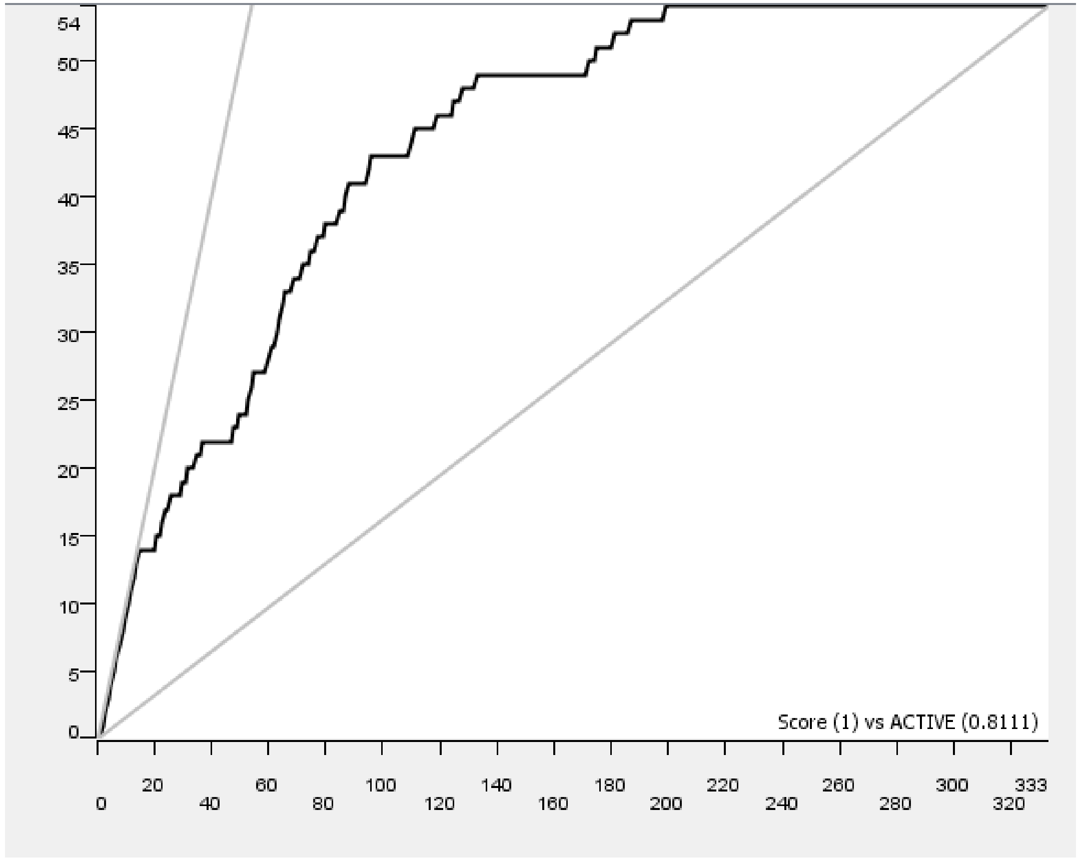

N=333

**Top 10% Mean IC50**	**5.5 nM**

	Predicted Active	Predicted Inactive
Active	37	17
Inactive	40	239

<2.5 nM DefGood in Xa, 30% error

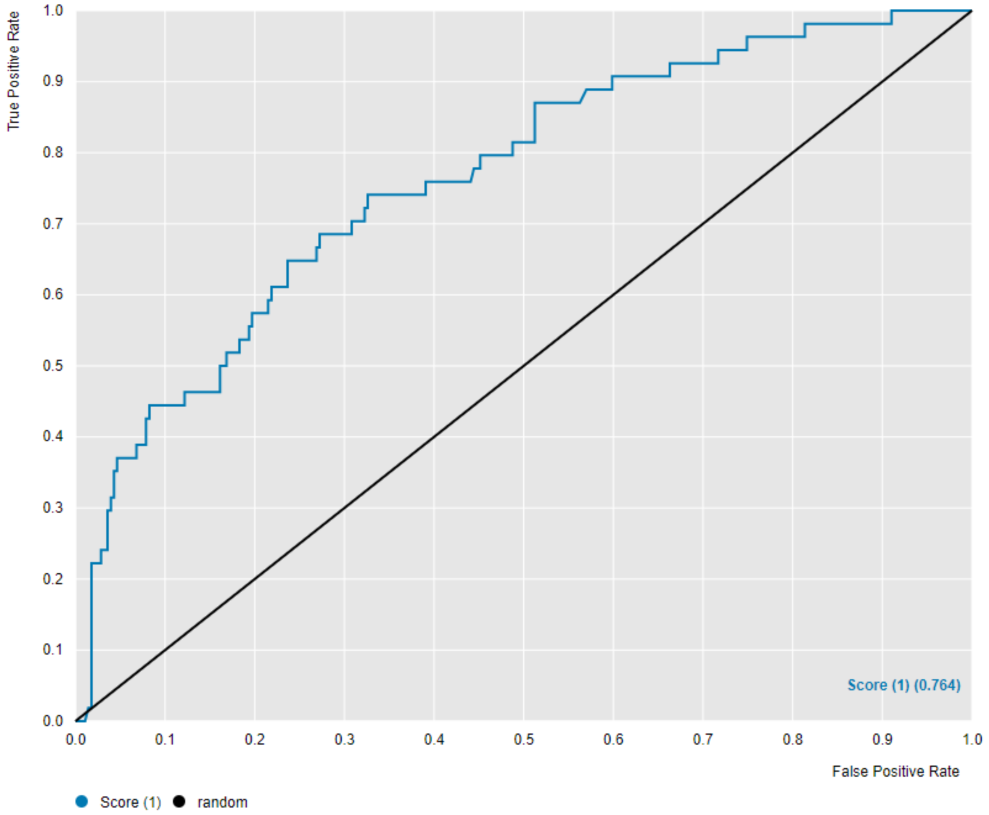

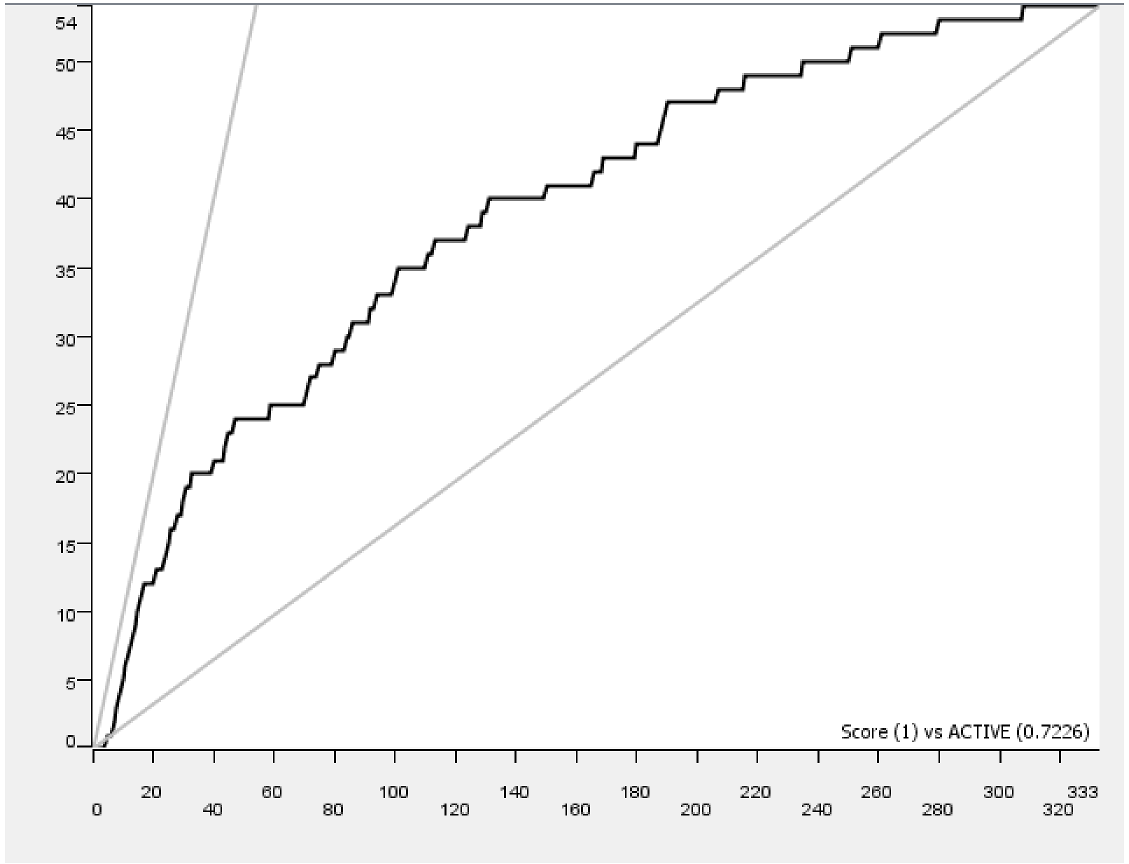

N=333

Top 10% Mean IC50	9.2 nM

	Predicted Active	Predicted Inactive
Active	35	19
Inactive	74	205

<2.5 nM DefGood in Xa, 35% error

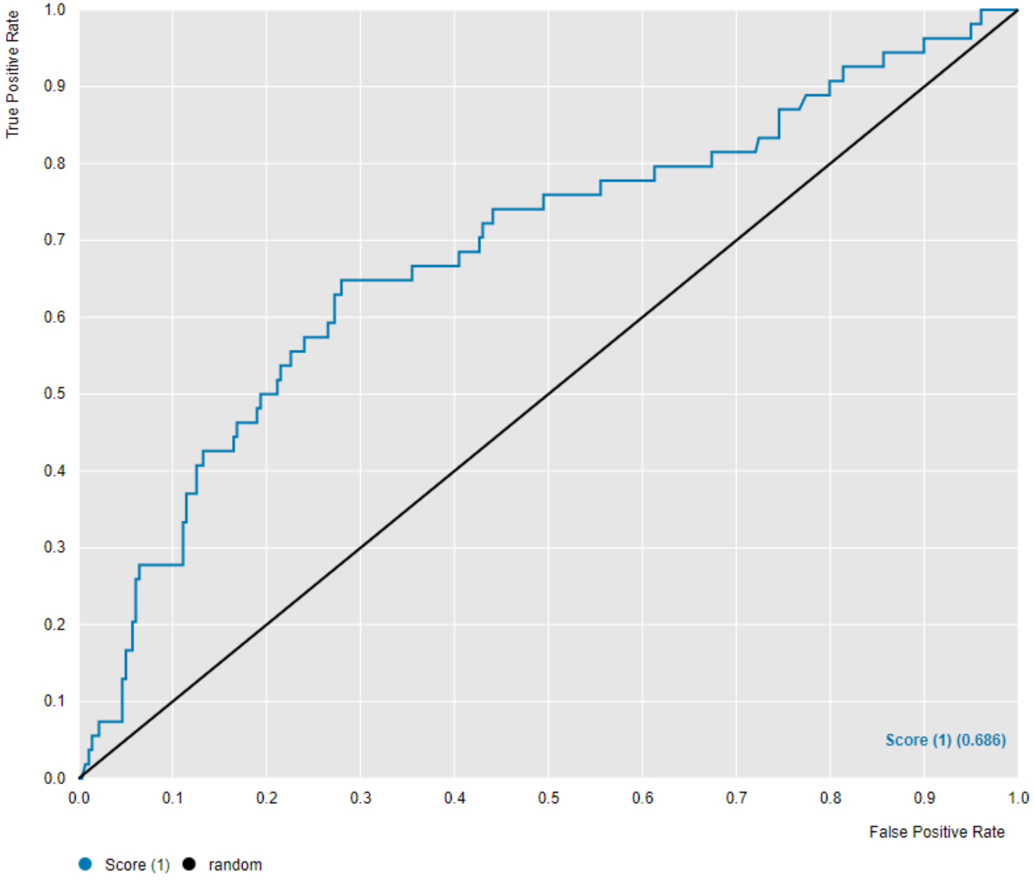

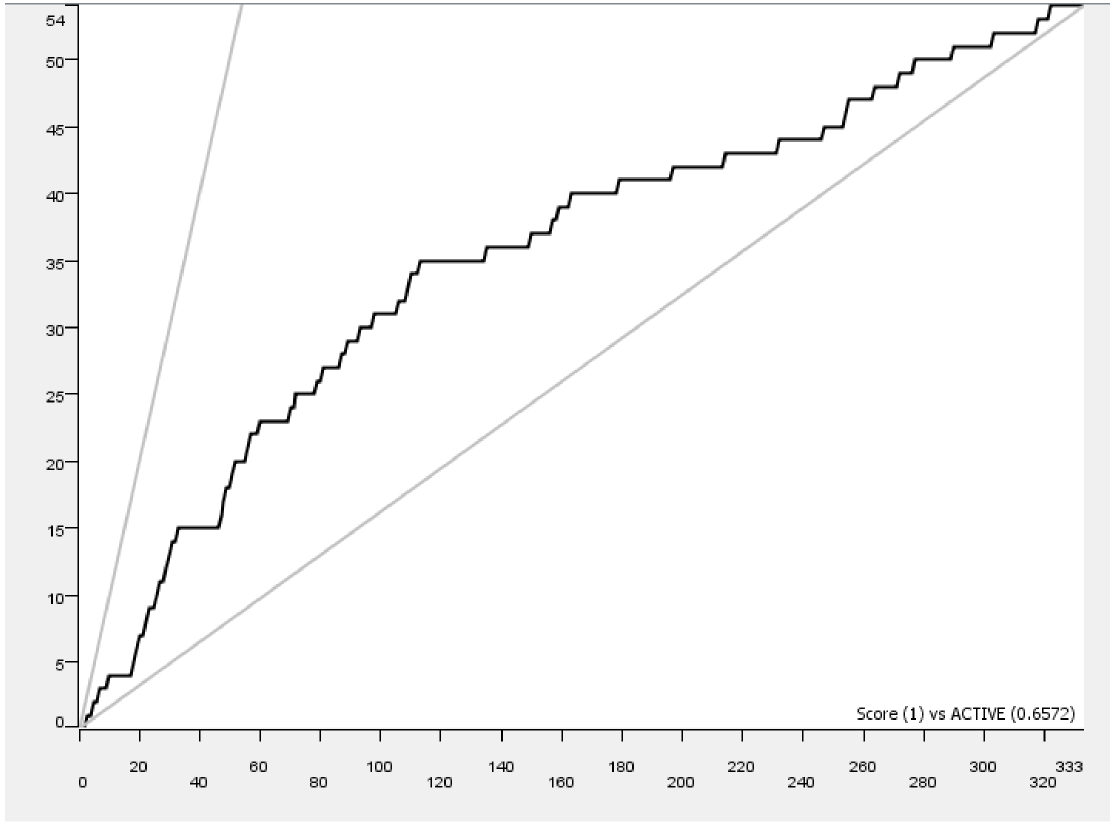

N=333

Top 10% Mean IC50	9,500 nM

	Predicted Active	Predicted Inactive
Active	35	19
Inactive	87	192

<2.5 nM DefGood in Xa, 40% error

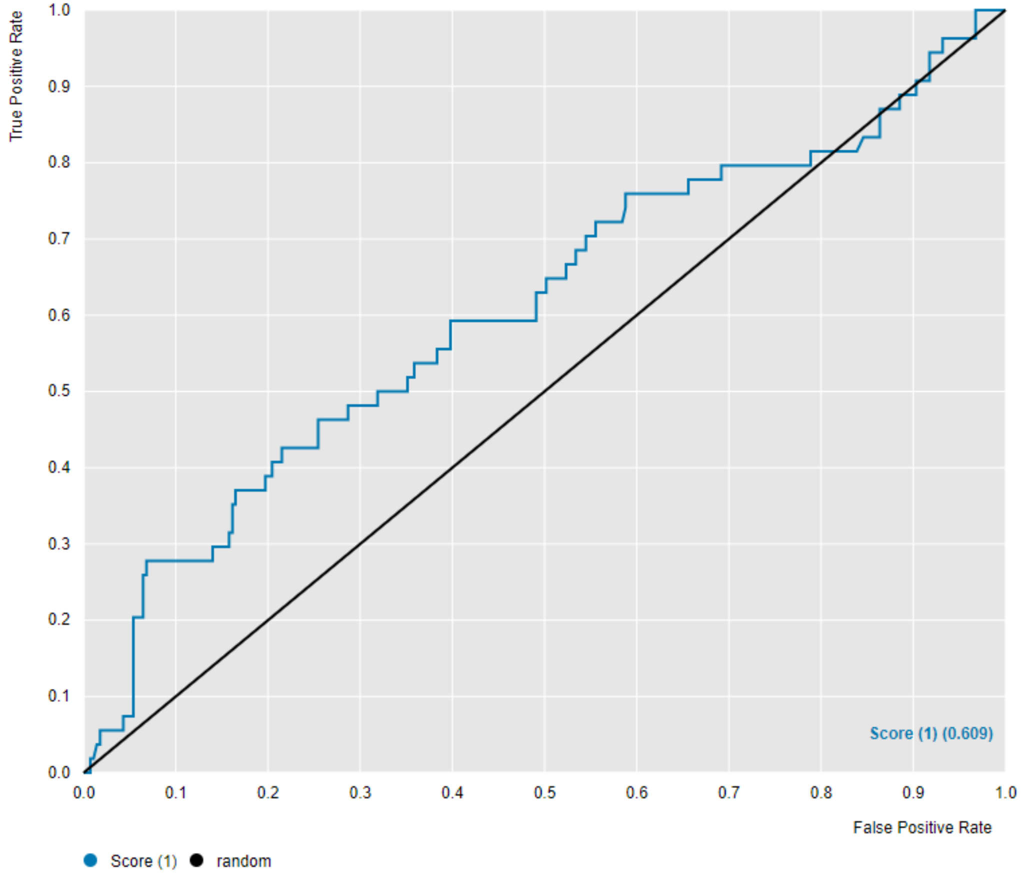

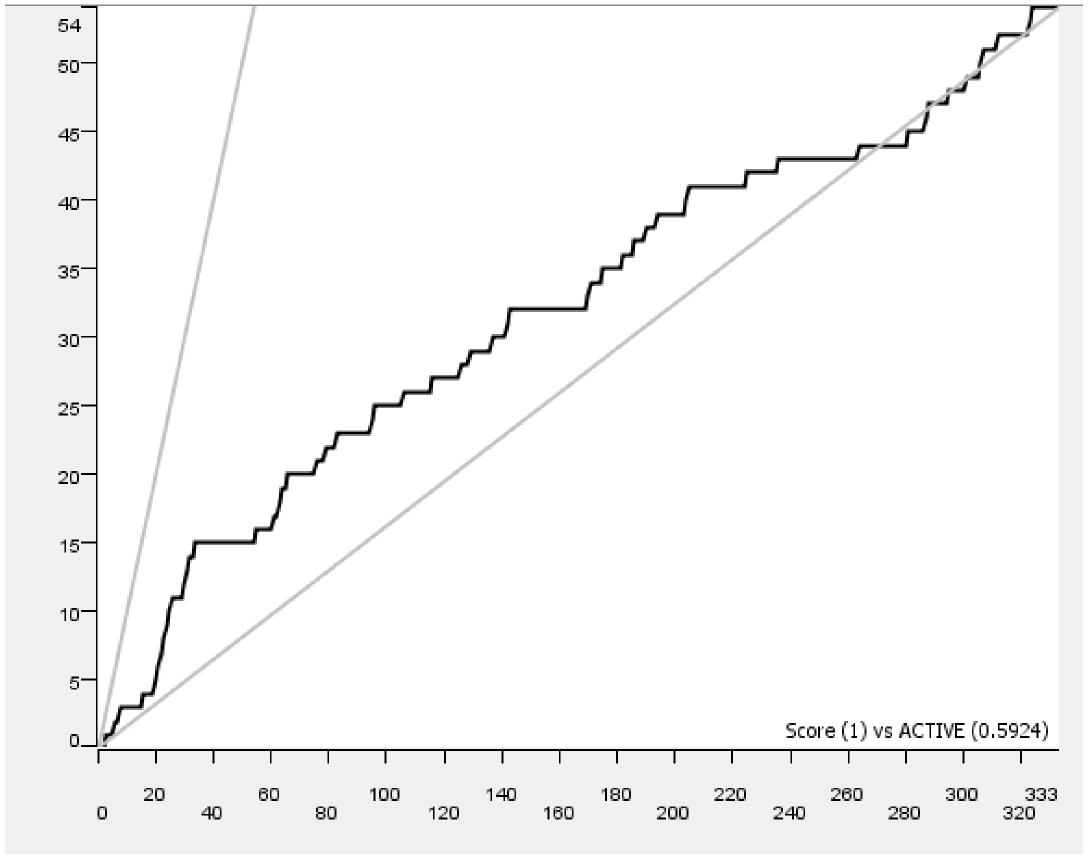

N=333

**Top 10% Mean IC50**	**6,500 Nm**

	**Predicted Active**	**Predicted Inactive**
Active	32	22
Inactive	137	142

<2.5 nM DefGood in Xa, 10% error; Random seed = 429

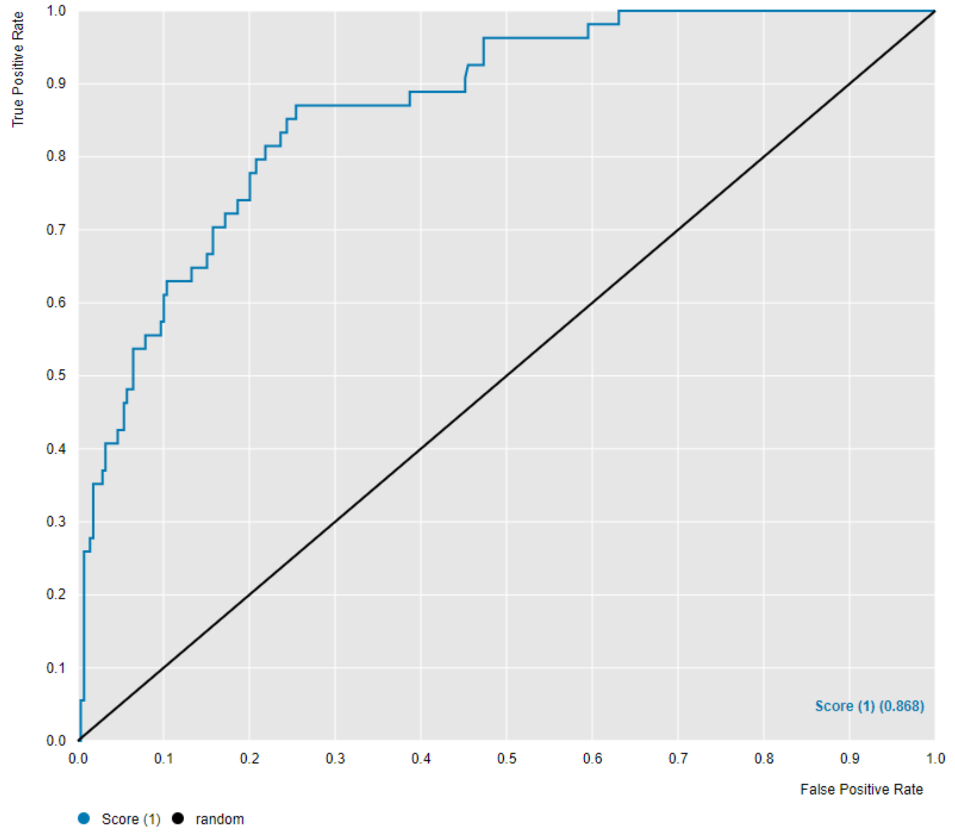

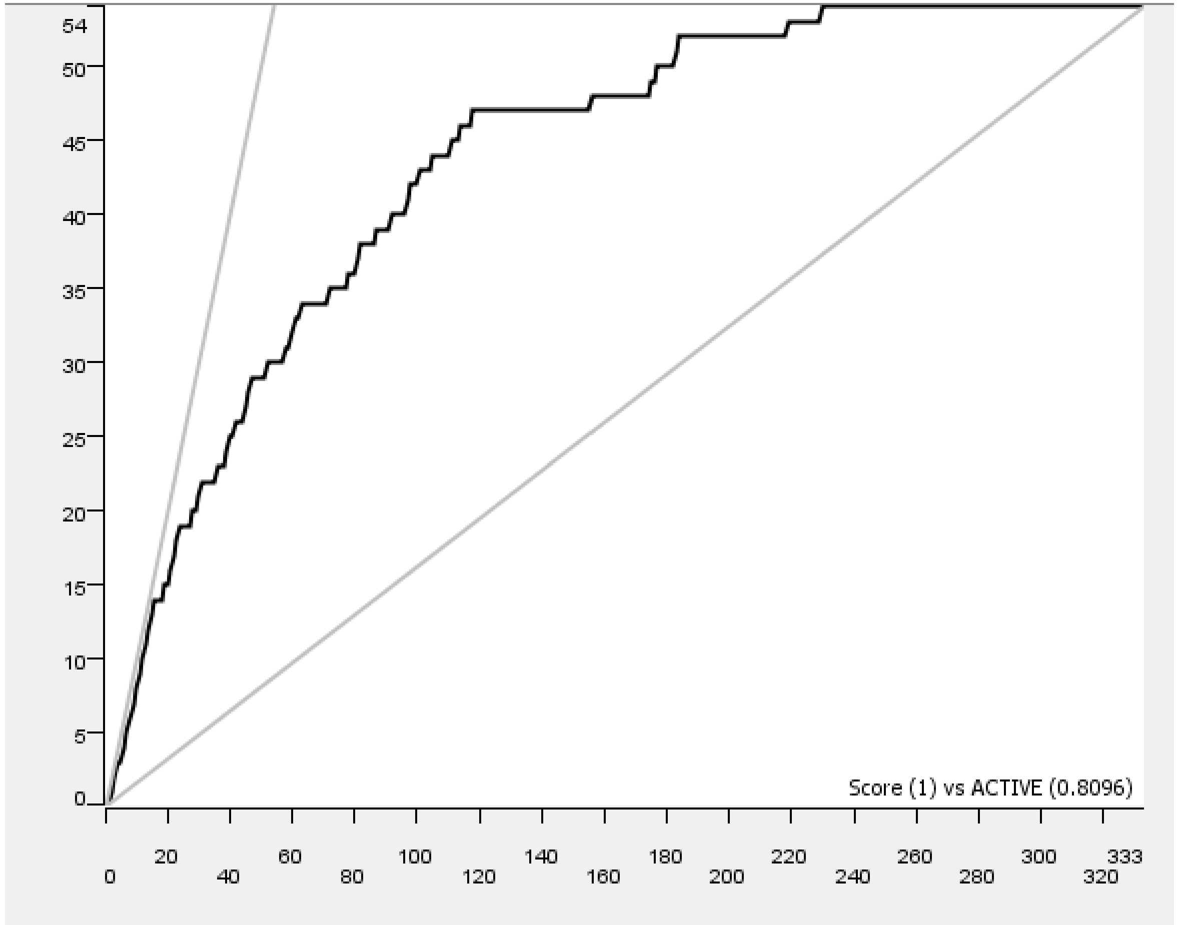

N=333

Top 10% Mean IC50	8.2 nM

	Predicted Active	Predicted Inactive
Active	45	9
Inactive	67	212

<2.5 nM DefGood in Xa, 20% error; Random seed = 429

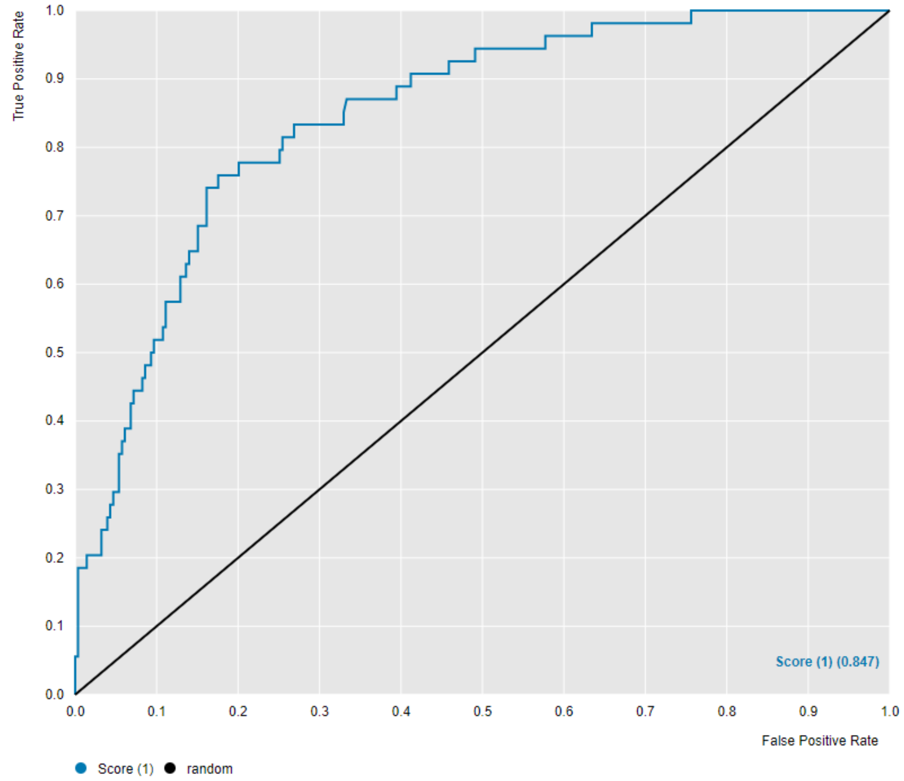

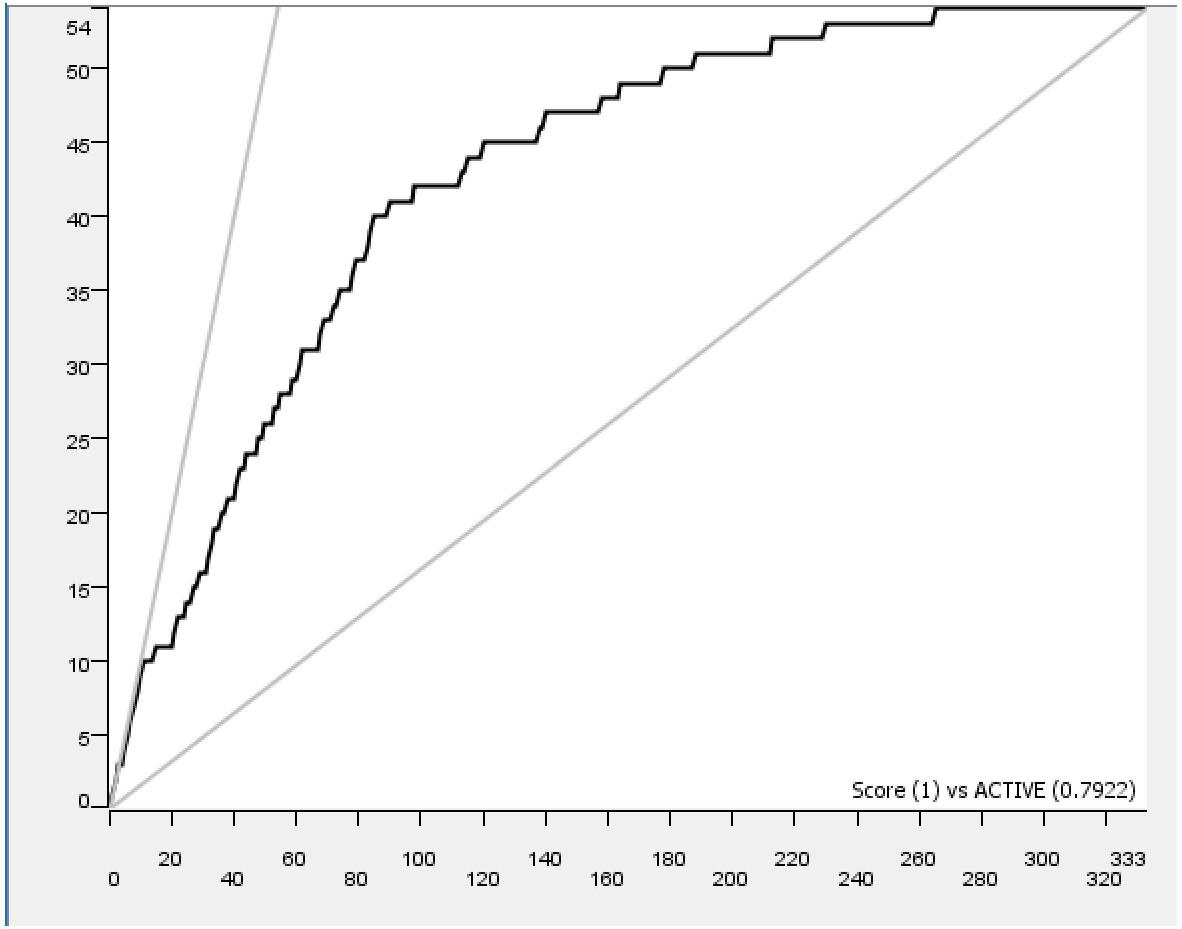

N=333

**Top 10% Mean IC50**	**9.1**

	Predicted Active	Predicted Inactive
Active	45	9
Inactive	78	201

<2.5 nM DefGood in Xa, 30% error; Random seed = 429

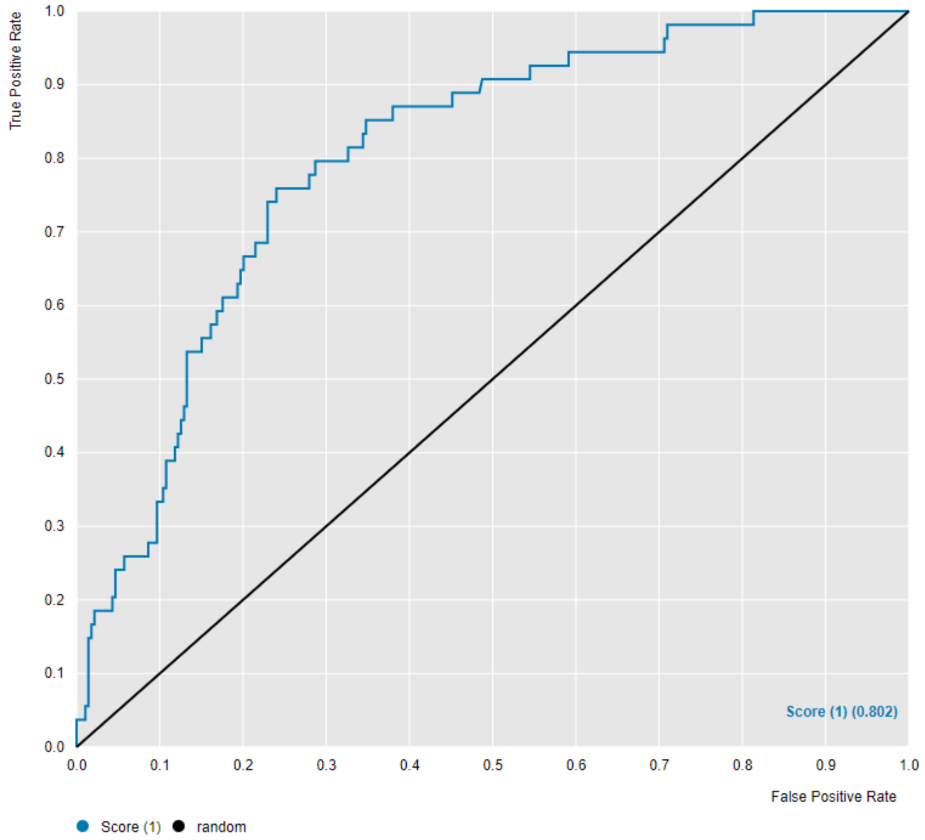

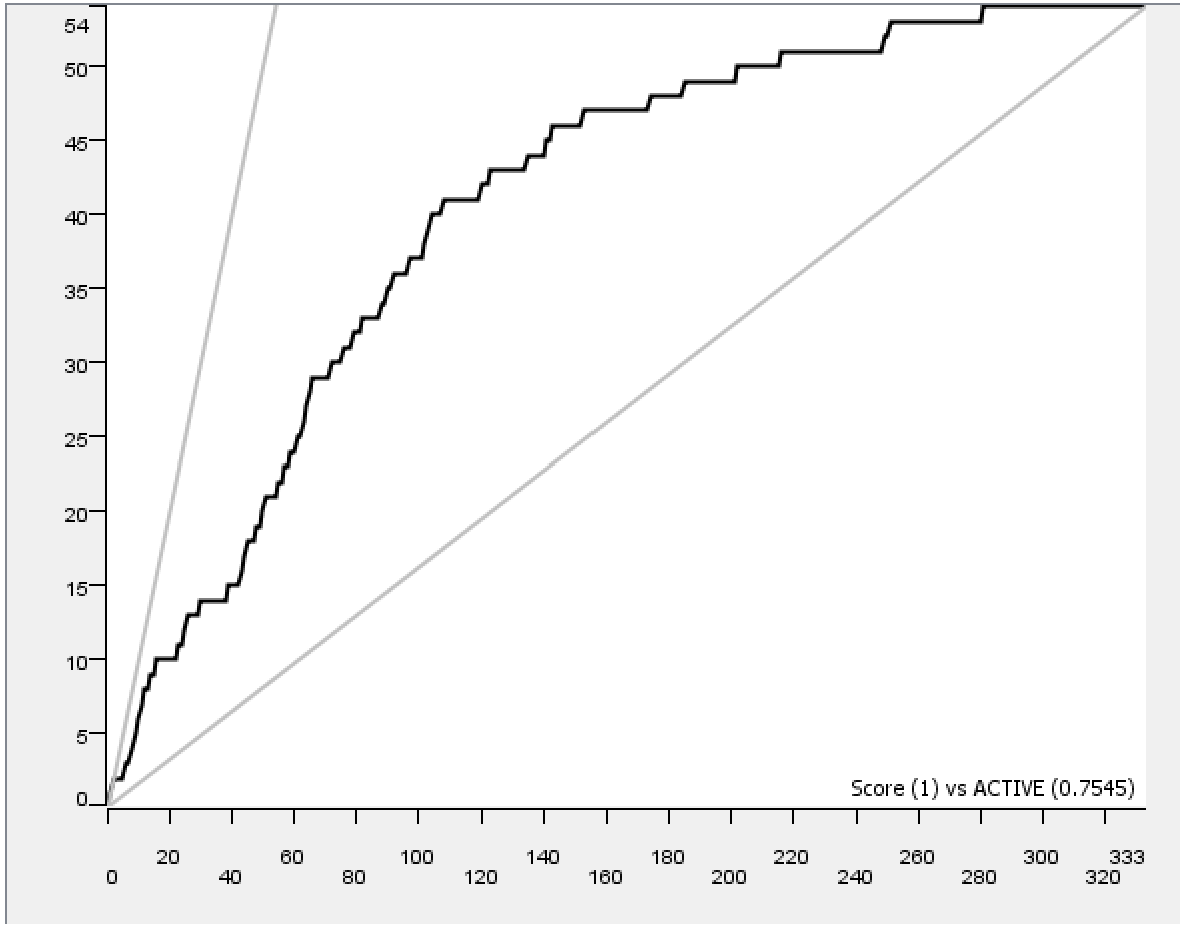

N=333

Top 10% Mean IC50	9.3 nM

	Predicted Active	Predicted Inactive
Active	51	3
Inactive	175	104

<2.5 nM DefGood in Xa, 35% error; Random seed = 429

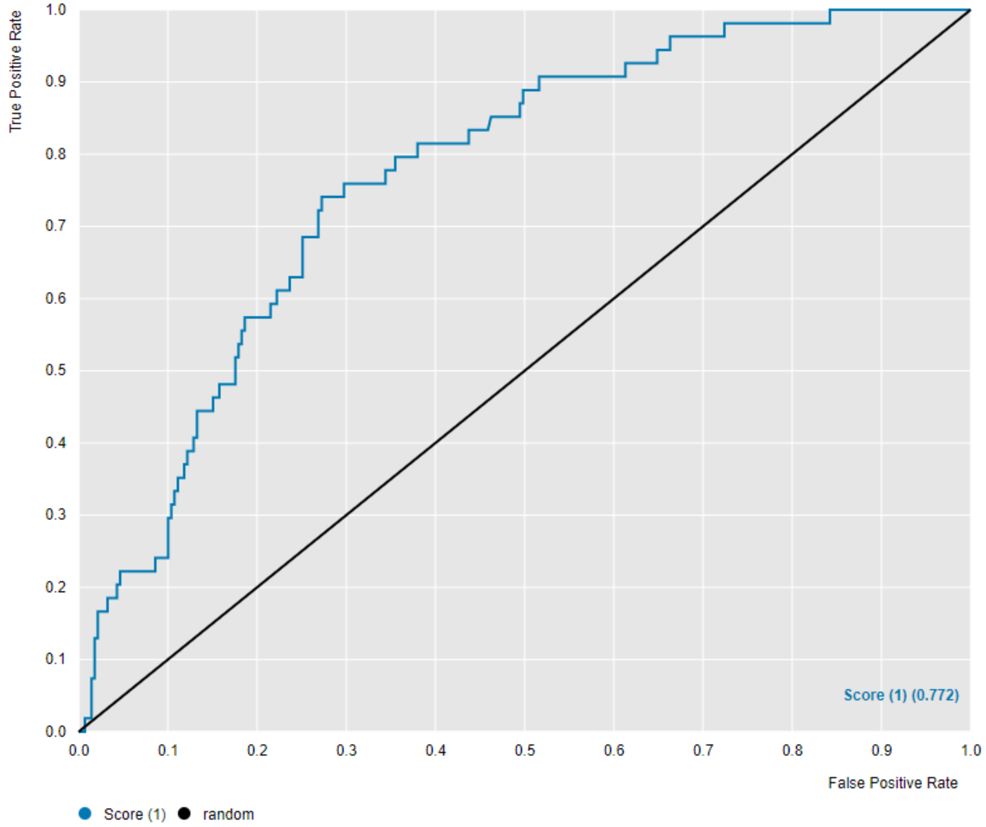

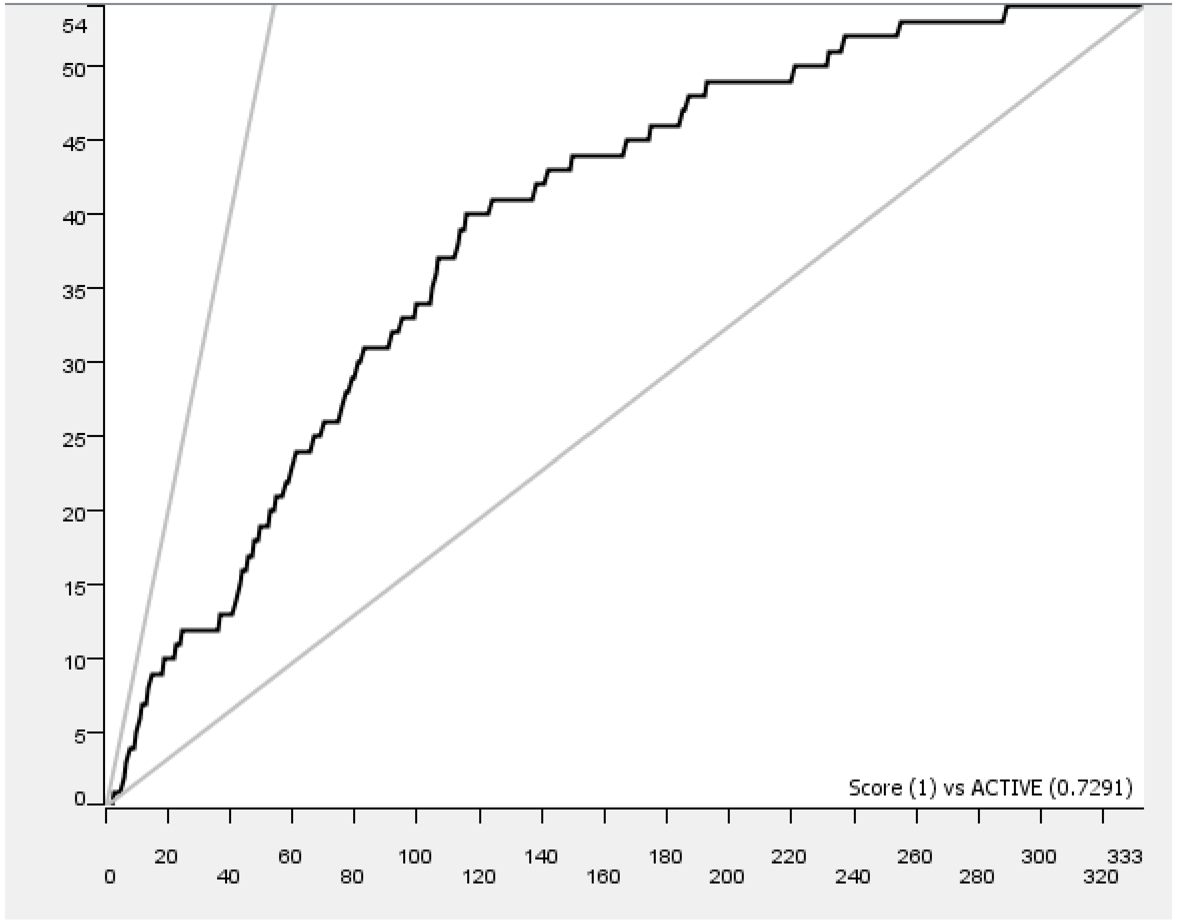

N=333

Top 10% Mean IC50	20.7 nM<br>No mistakes greater than 200 nM

	Predicted Active	Predicted Inactive
Active	33	21
Inactive	65	214

<2.5 nM DefGood in Xa, 40% error; Random seed = 429

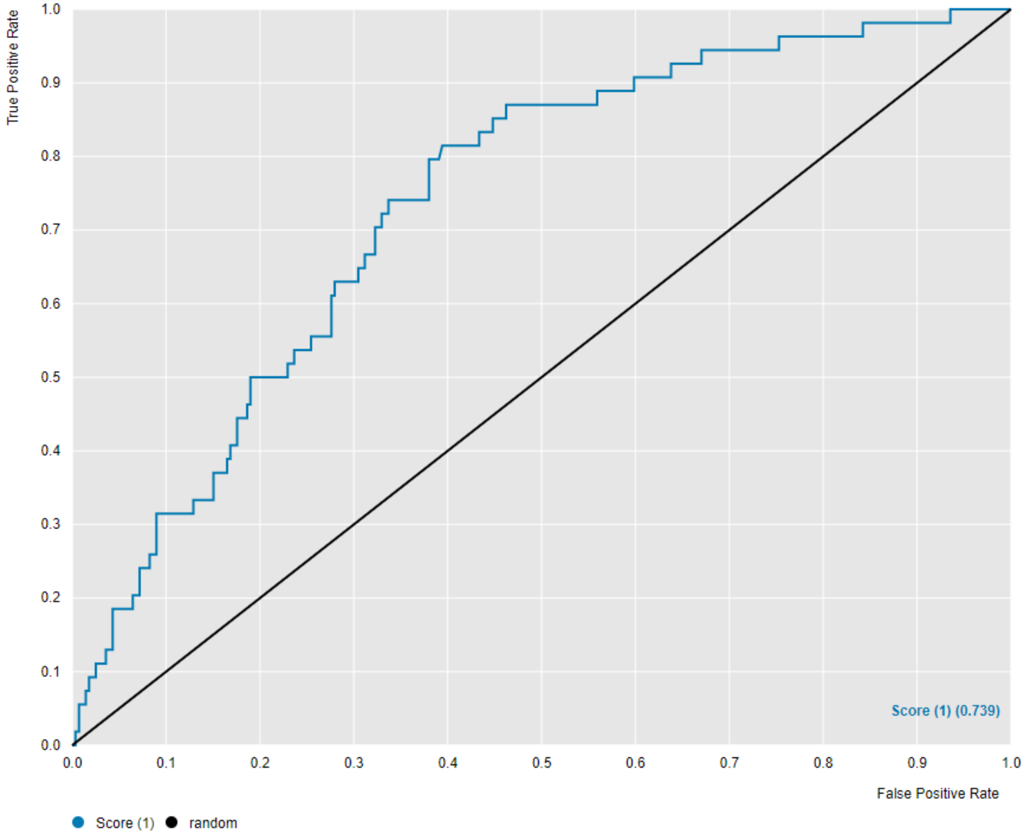

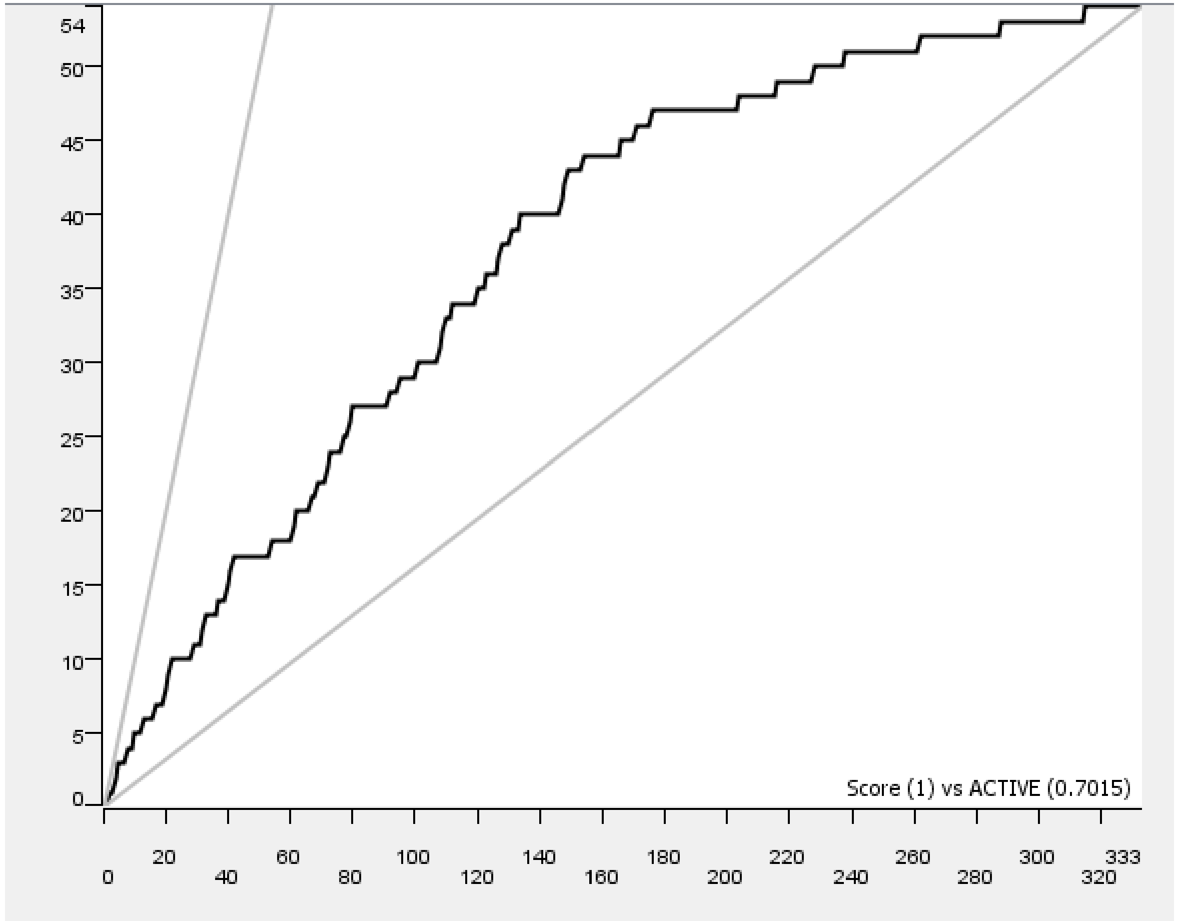

N=333

Top 10% Mean IC50	12.1 nM

	Predicted Active	Predicted Inactive
Active	32	22
Inactive	77	202

<2.5 nM DefGood in Xa, 45% error; Random seed = 429

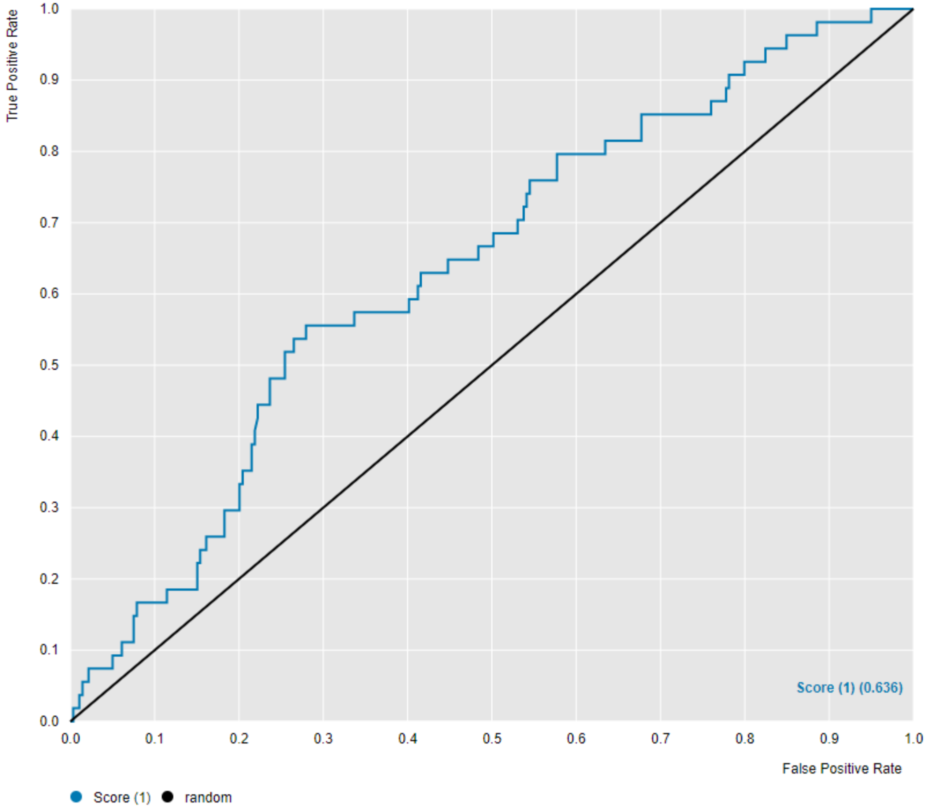

N=333

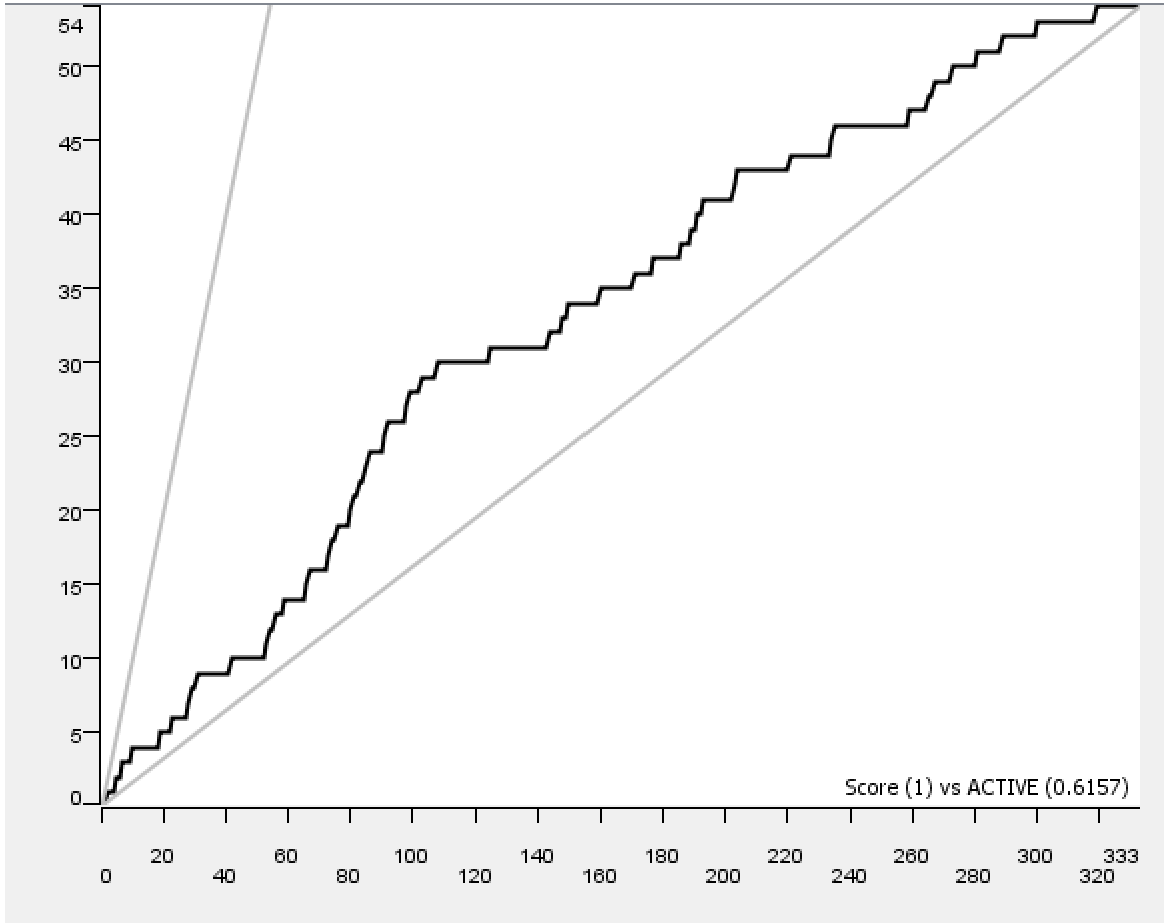

Top 10% Mean IC50	110 nM Several severe mistakes

	Predicted Active	Predicted Inactive
Active	32	22
Inactive	115	164

<2.5 nM DefGood in Xa, 10% error; Random seed = 121783

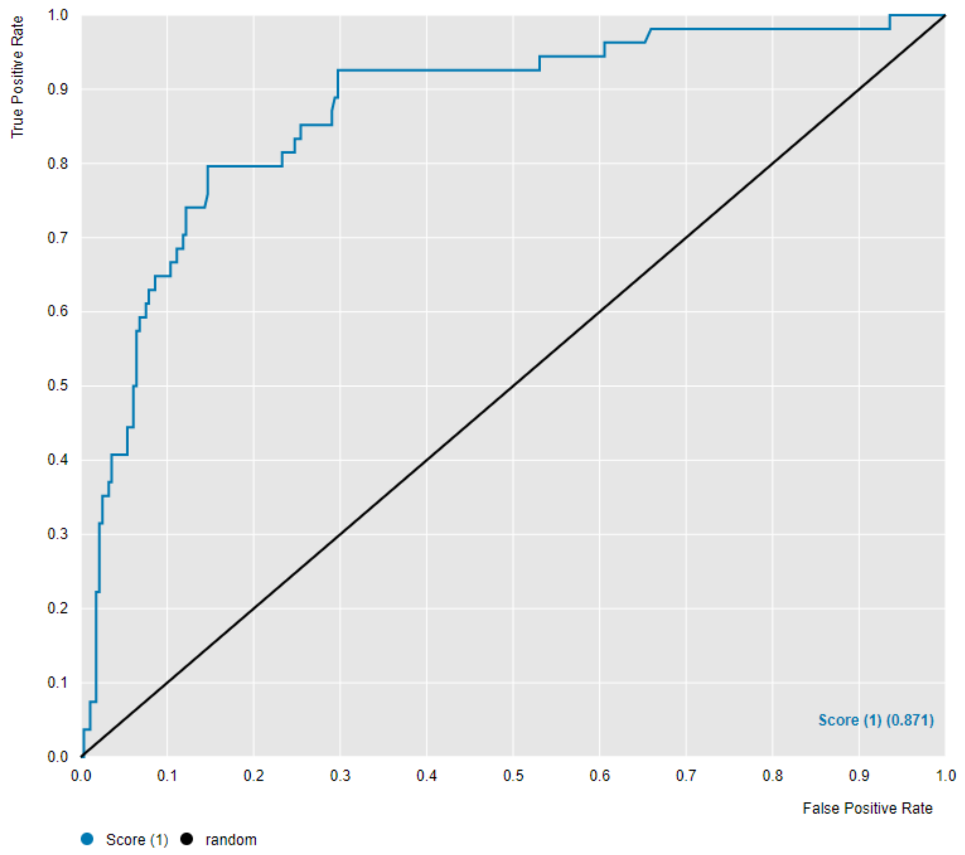

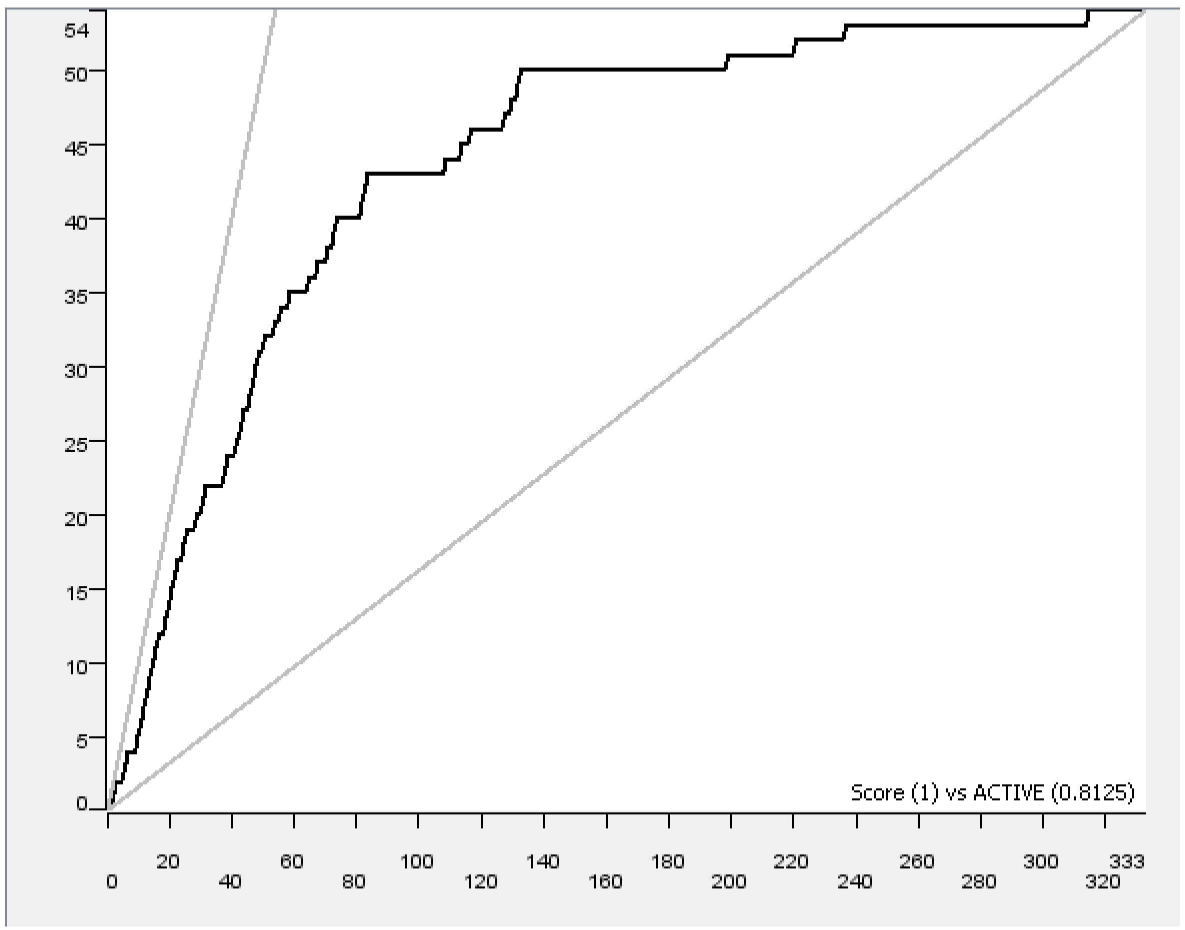

N=333

**Top 10% Mean IC50**	**5.9 nM**

	Predicted Active	Predicted Inactive
Active	40	14
Inactive	35	244

<2.5 nM DefGood in Xa, 20% error; Random seed = 121783

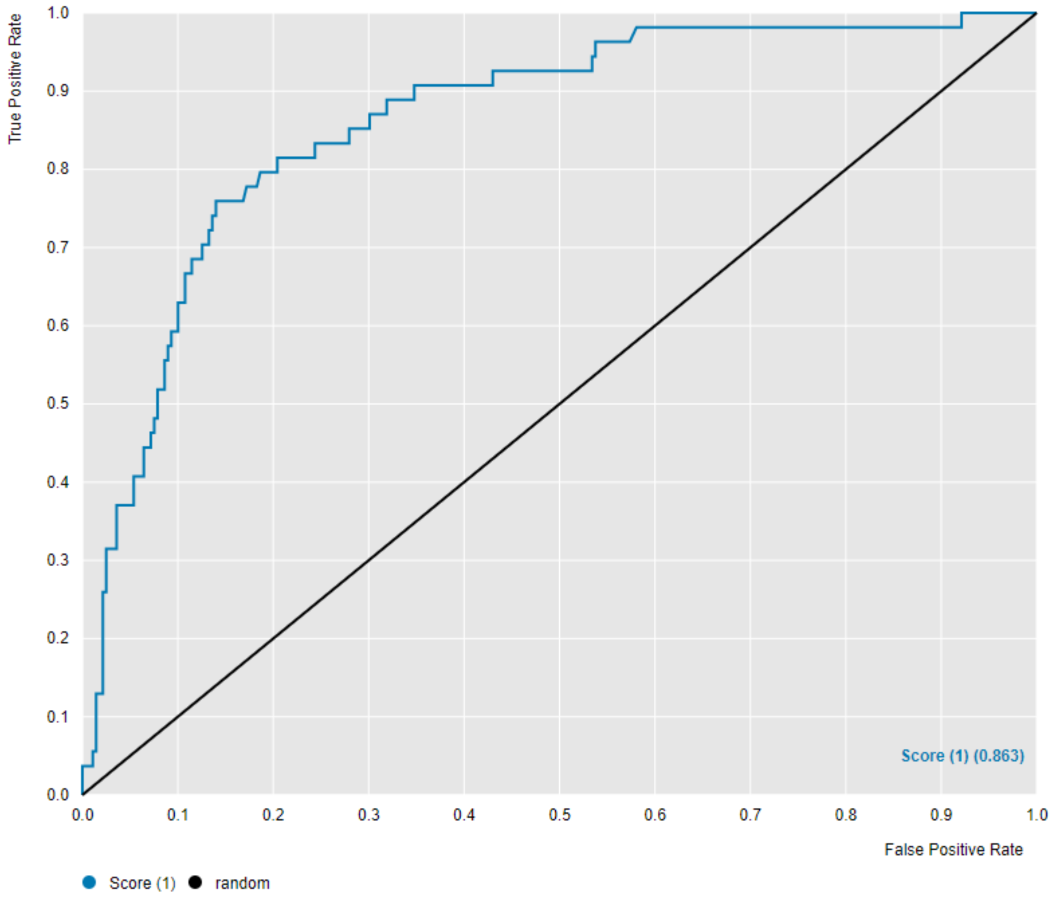

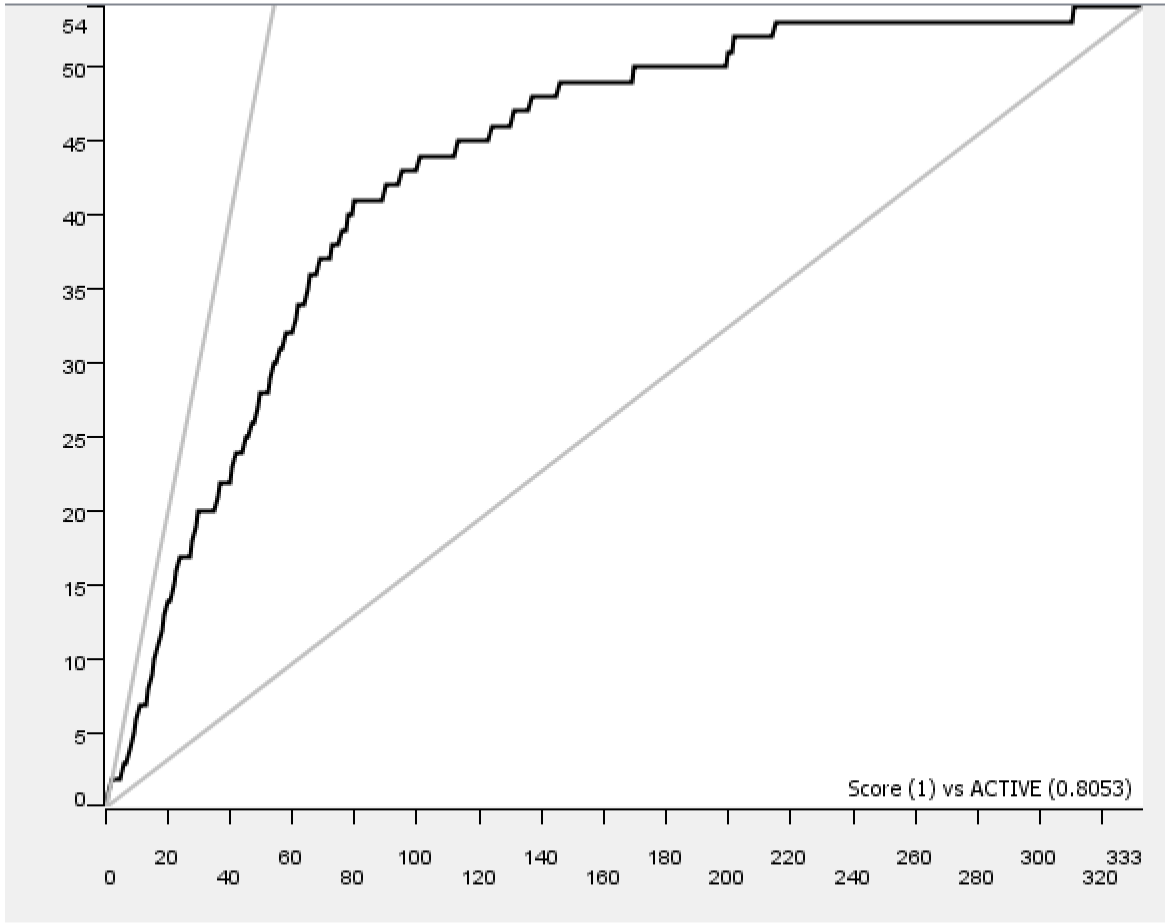

N=333

Top 10% Mean IC50	10.0 nM

	Predicted Active	Predicted Inactive
Active	39	15
Inactive	37	242

<2.5 nM DefGood in Xa, 35% error; Random seed = 121783

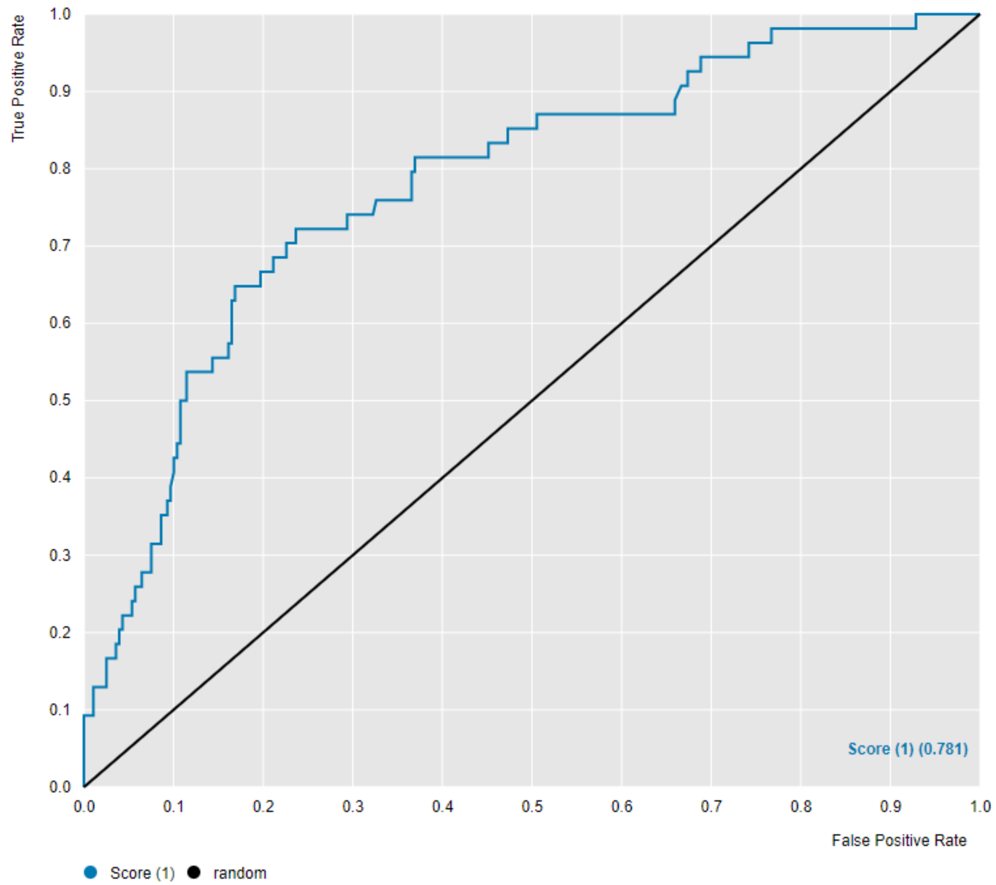

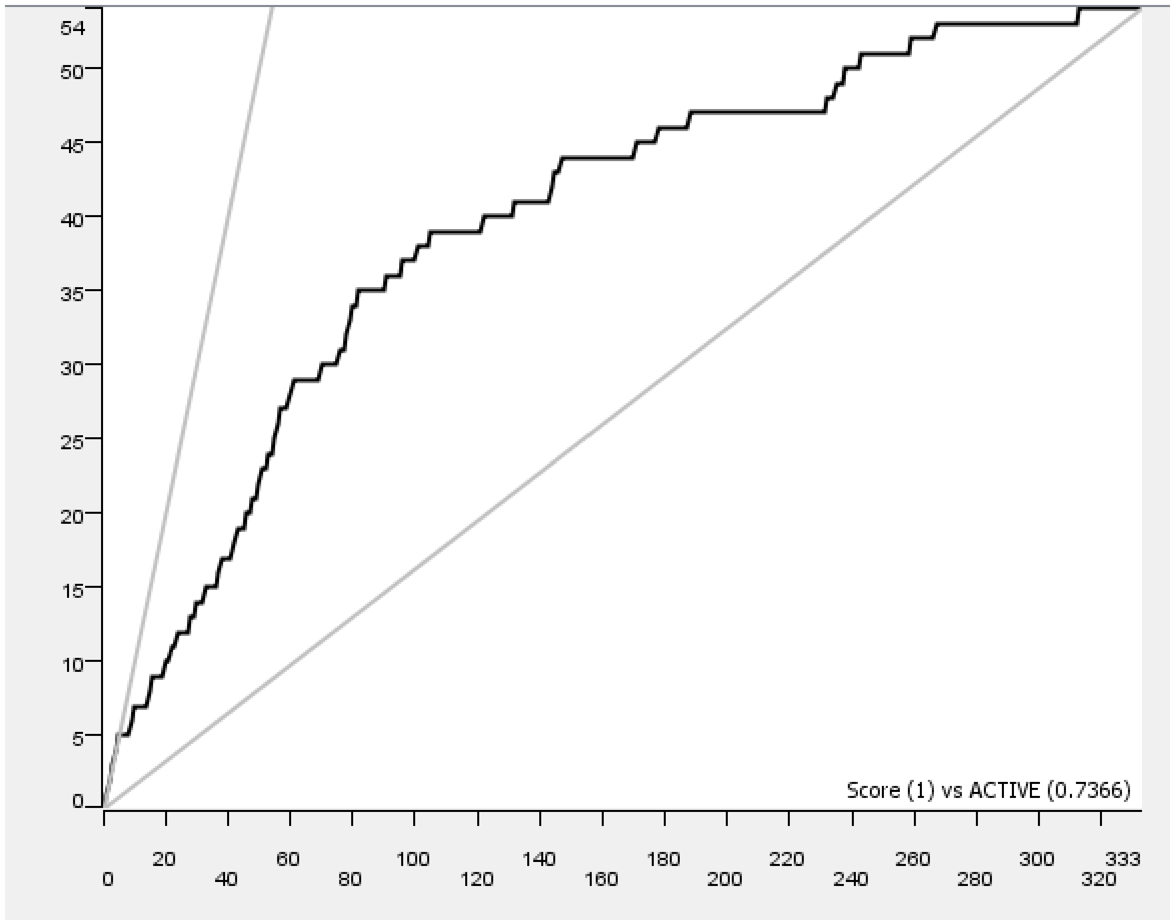

N=333

Top 10% Mean IC50	40.0 nM (One mistake - 930 nM)

	Predicted Active	Predicted Inactive
Active	47	7
Inactive	152	127

<2.5 nM DefGood in Xa, 40% error; Random seed = 121783

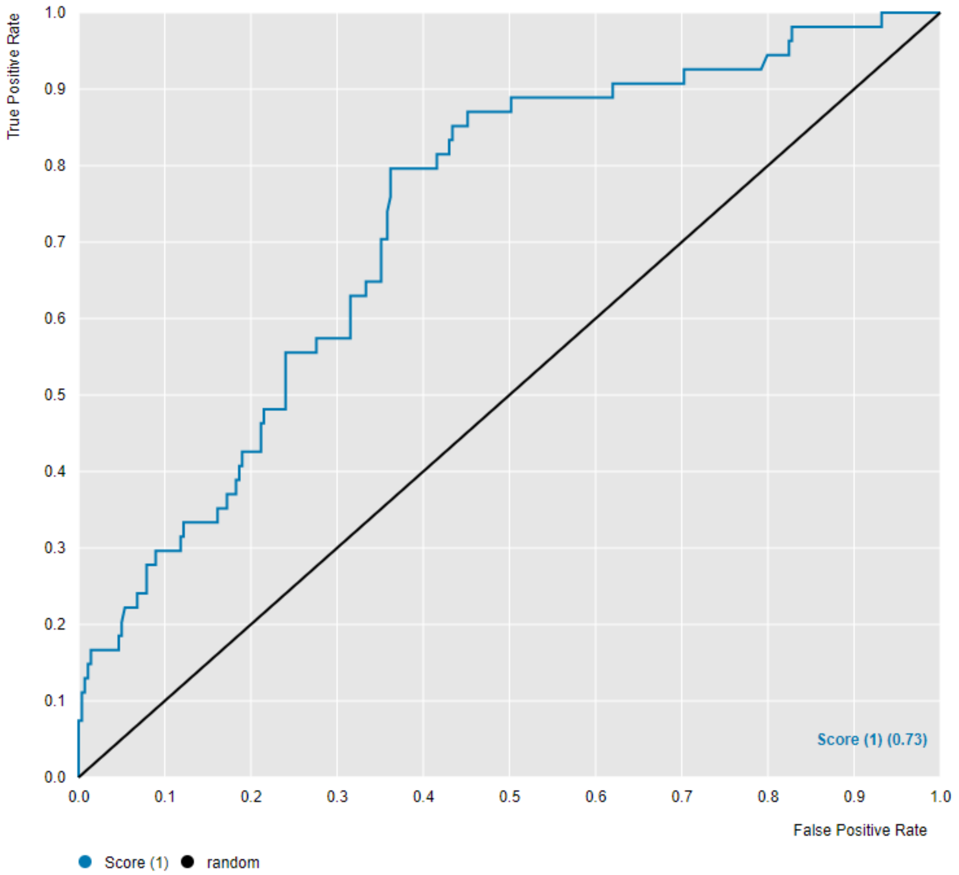

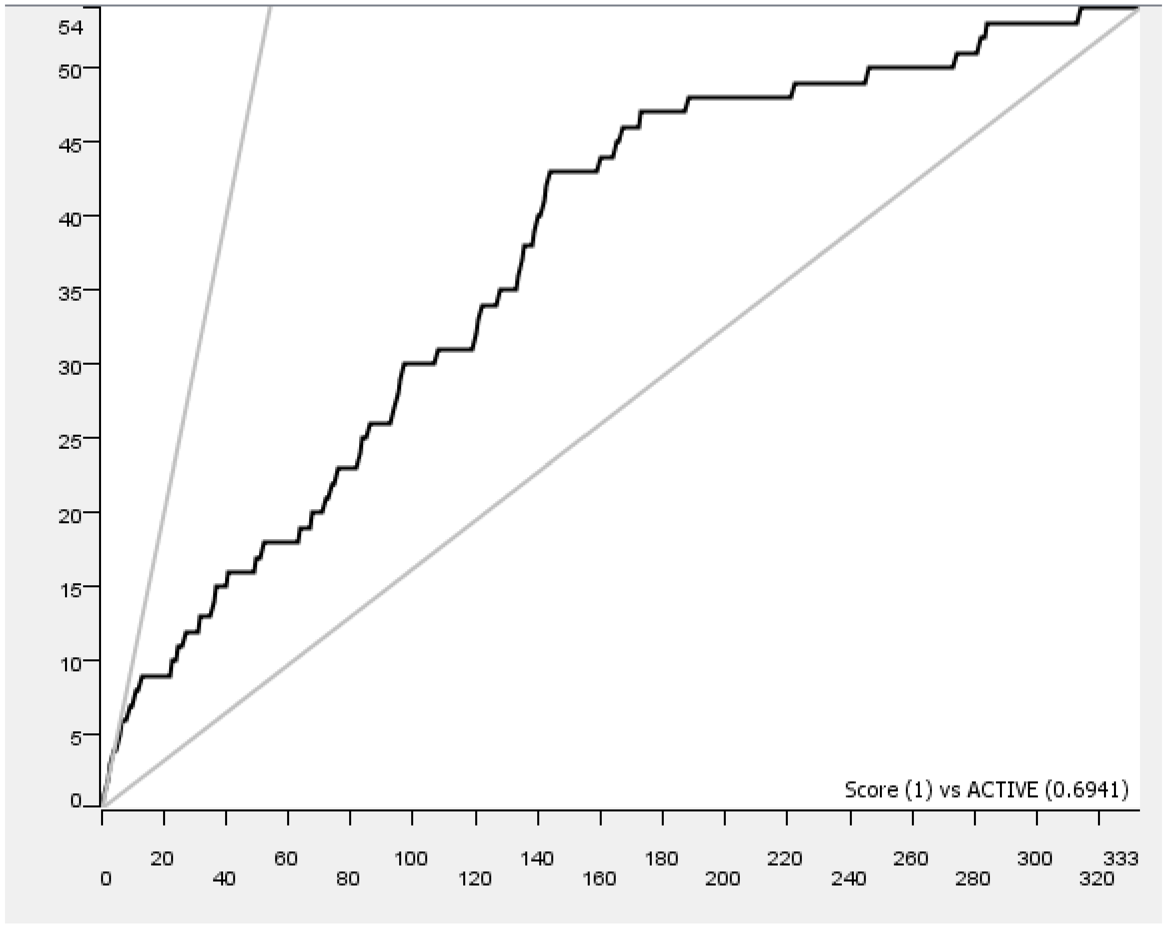

N=333

**Top 10% Mean IC50**	**43.5 nM (One mistake - 930 nM)**

	Predicted Active	Predicted Inactive
Active	34	20
Inactive	89	190

<2.5 nM DefGood in Xa, 45% error; Random seed = 121783

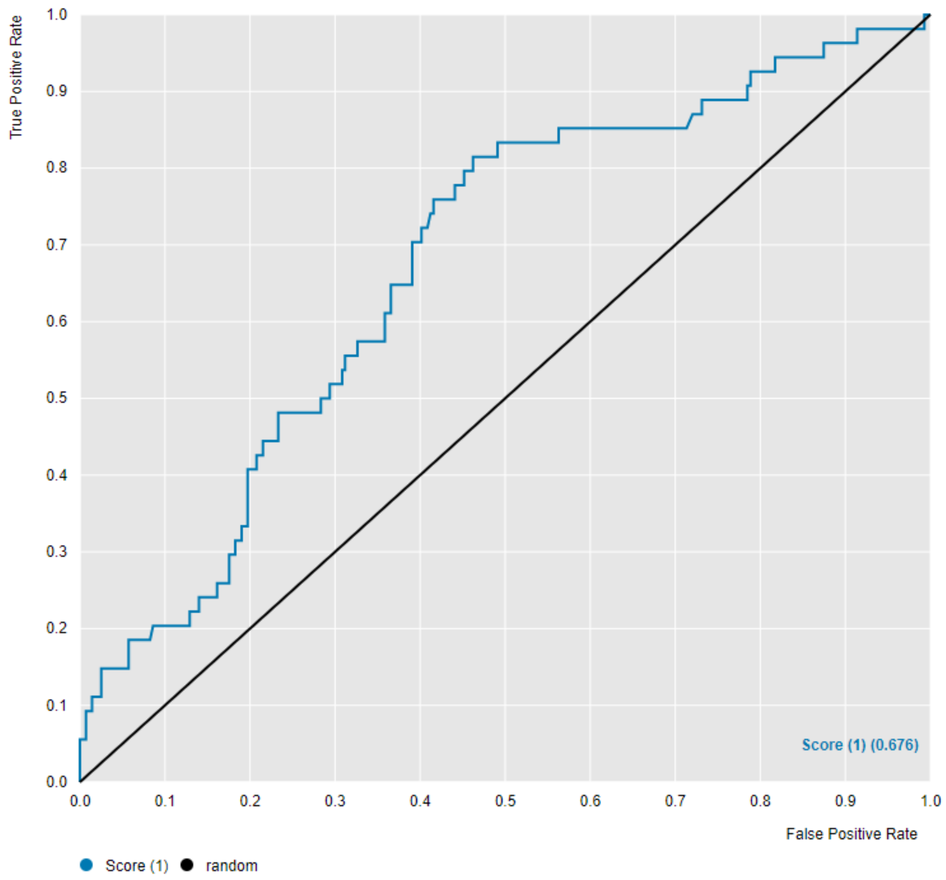

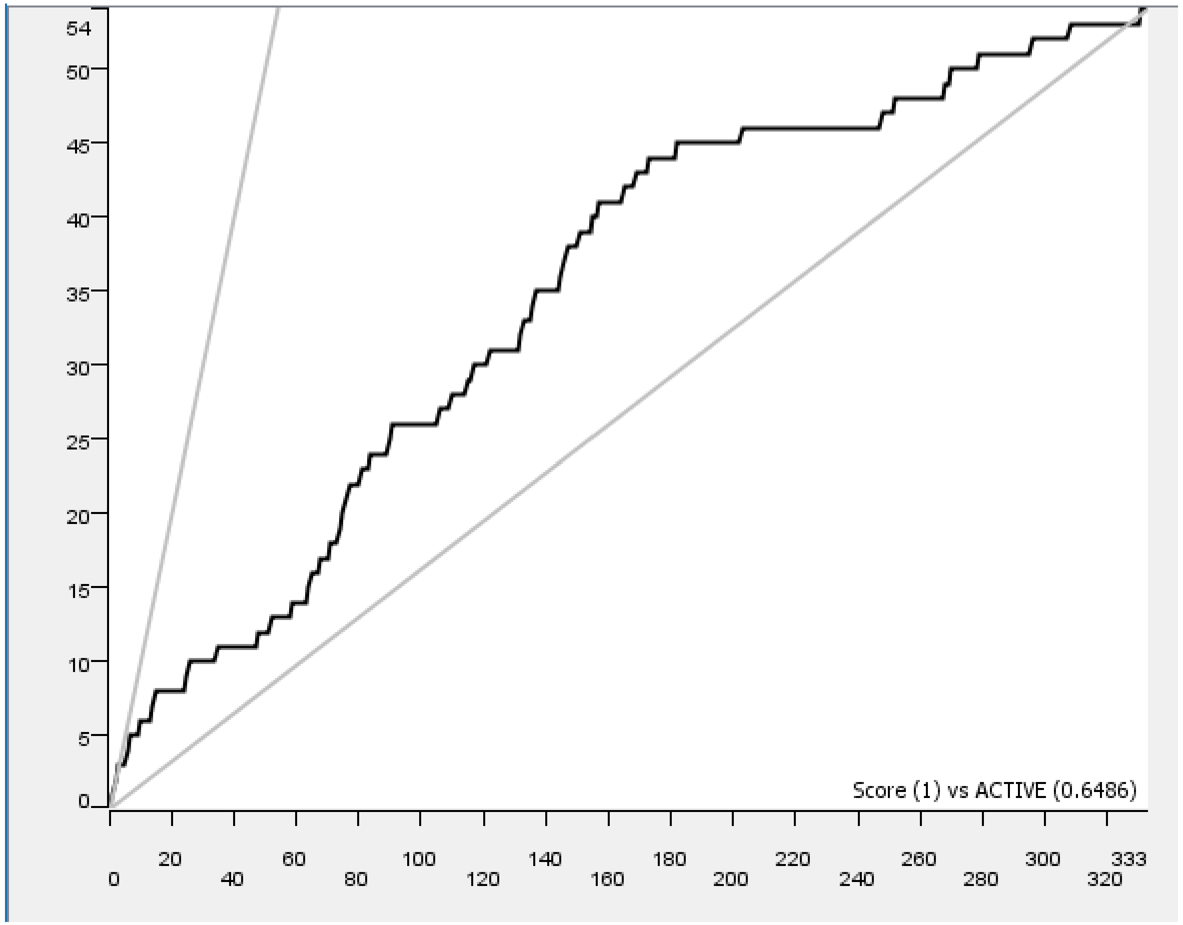

N=333

Top 10% Mean IC50	350 nM (several severe micromolar mistakes

	Predicted Active	Predicted Inactive
Active	23	31
Inactive	60	219

Conclusion - NBN

- An NBN could be generated for Factor Xa with a decision value of <2.5 nM as defined as active. The predictivity of the algorithm could be preserved in three random train/test cycles. The point of failure was 35%, 45% and 45% error.

RF Error Tolerance- <2.5 nM DefGood in Xa

- 0-50% absolute error

RF- <2.5 nM DefGood in Xa, 5% error; Random seed = 1515533876005

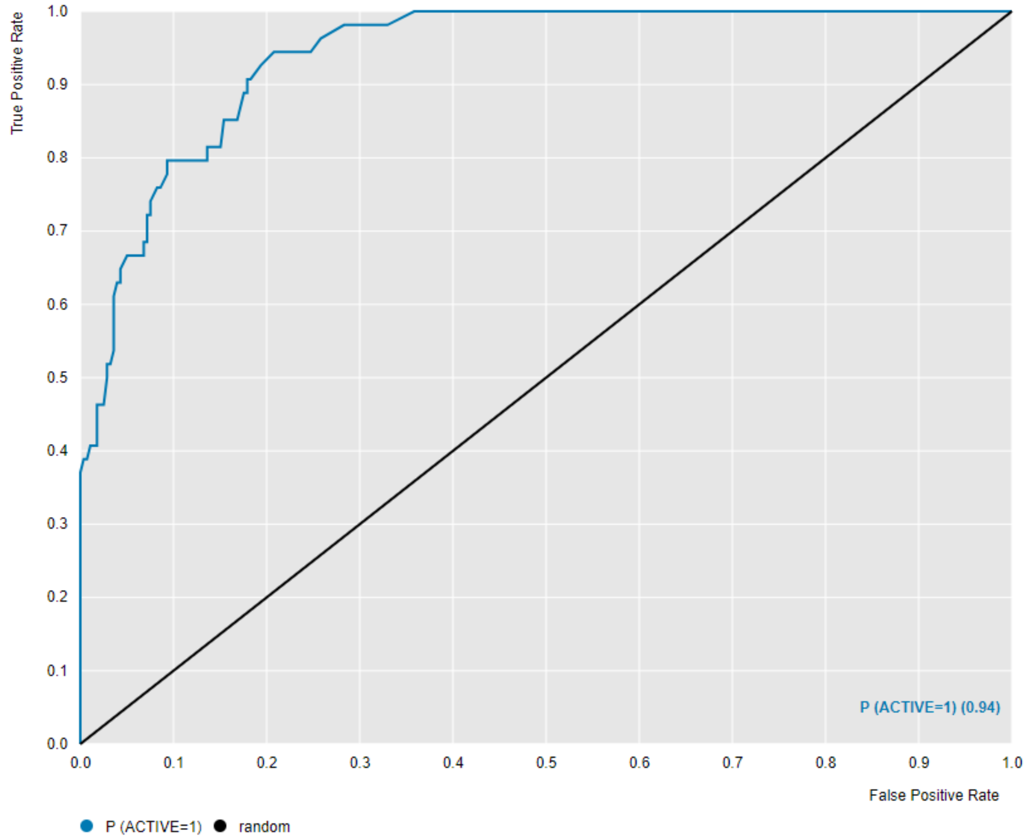

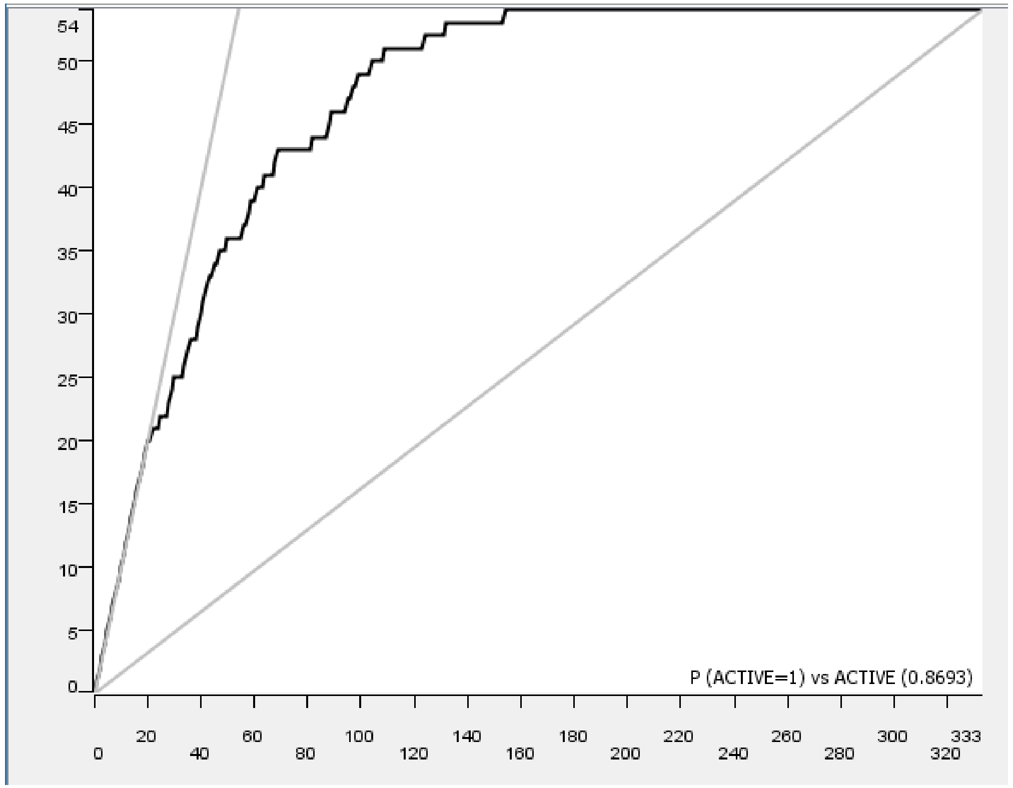

N=333

Top 10% Mean IC50	11.5

	Predicted Active	Predicted Inactive
Active	36	18
Inactive	19	260

RF-<2.5 nM DefGood in Xa, 10% error

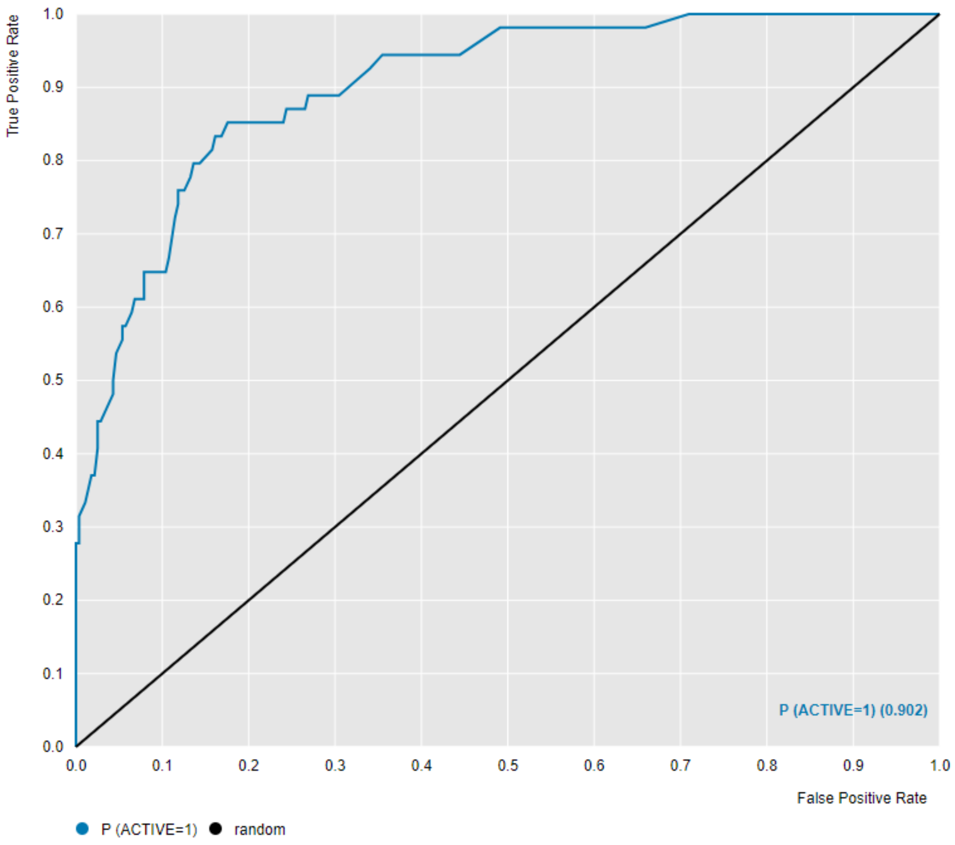

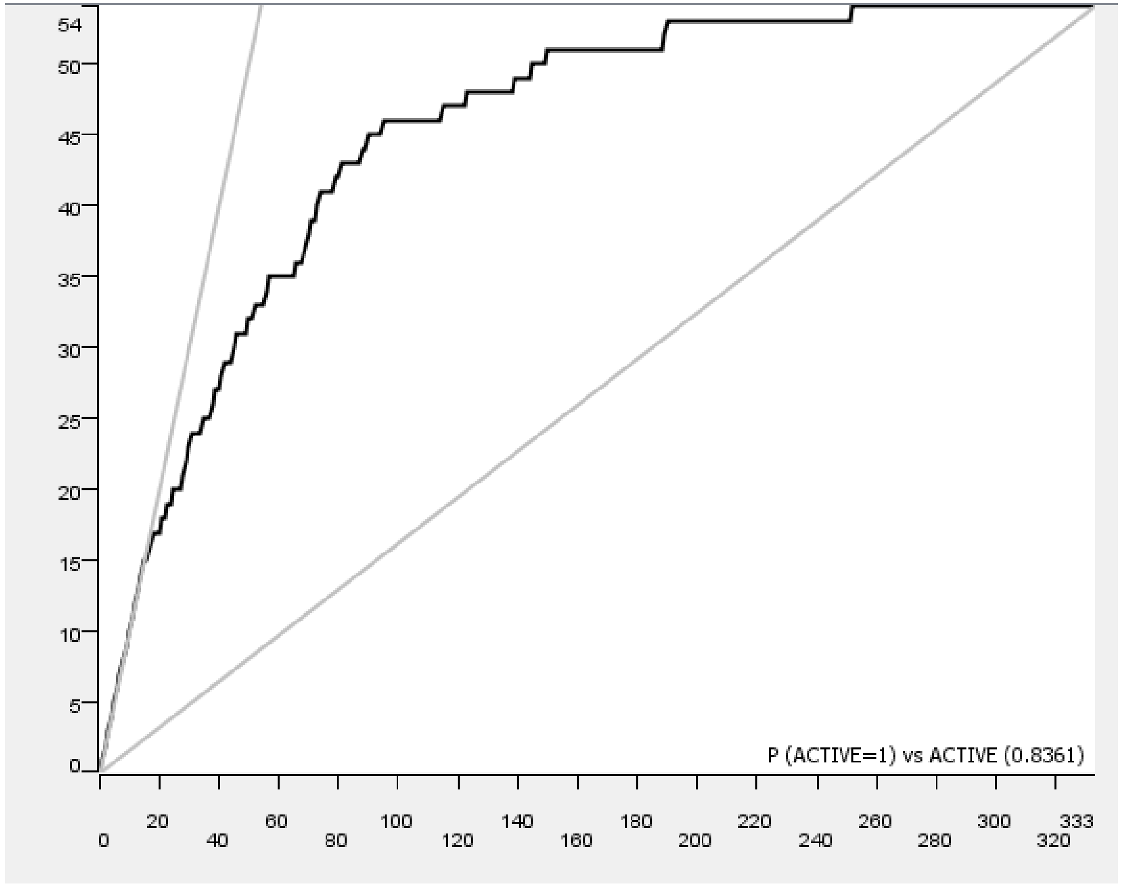

N=333

Top 10% Mean IC50	11.9 nM

	Predicted Active	Predicted Inactive
Active	32	22
Inactive	18	261

RF-<2.5 nM DefGood in Xa, 20% error

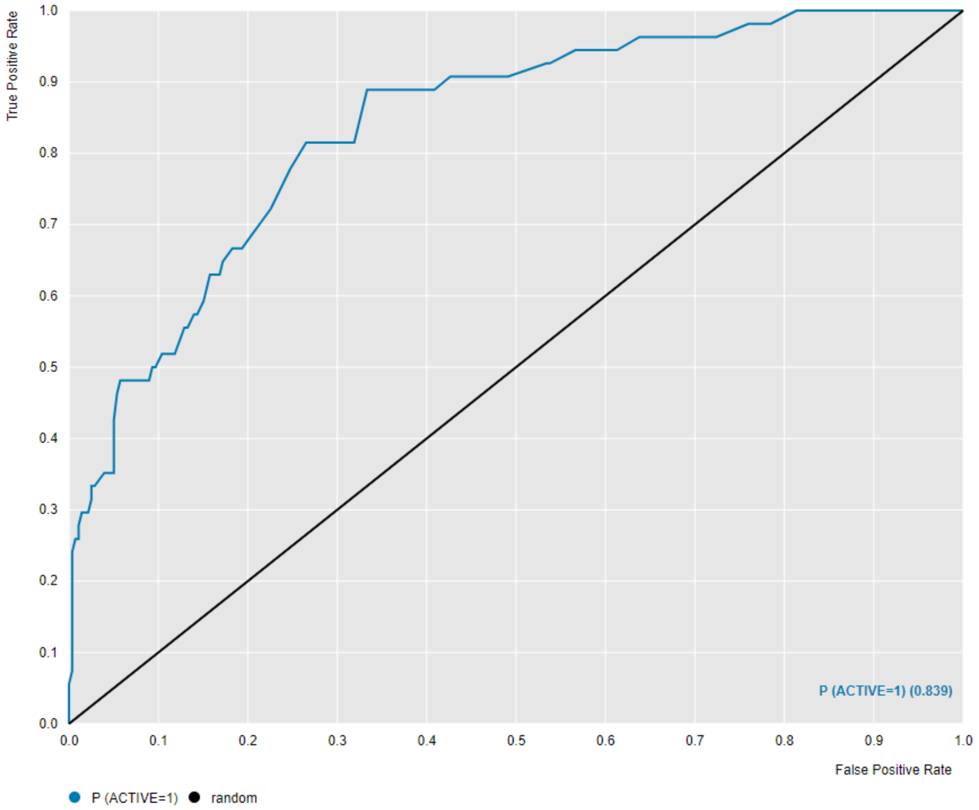

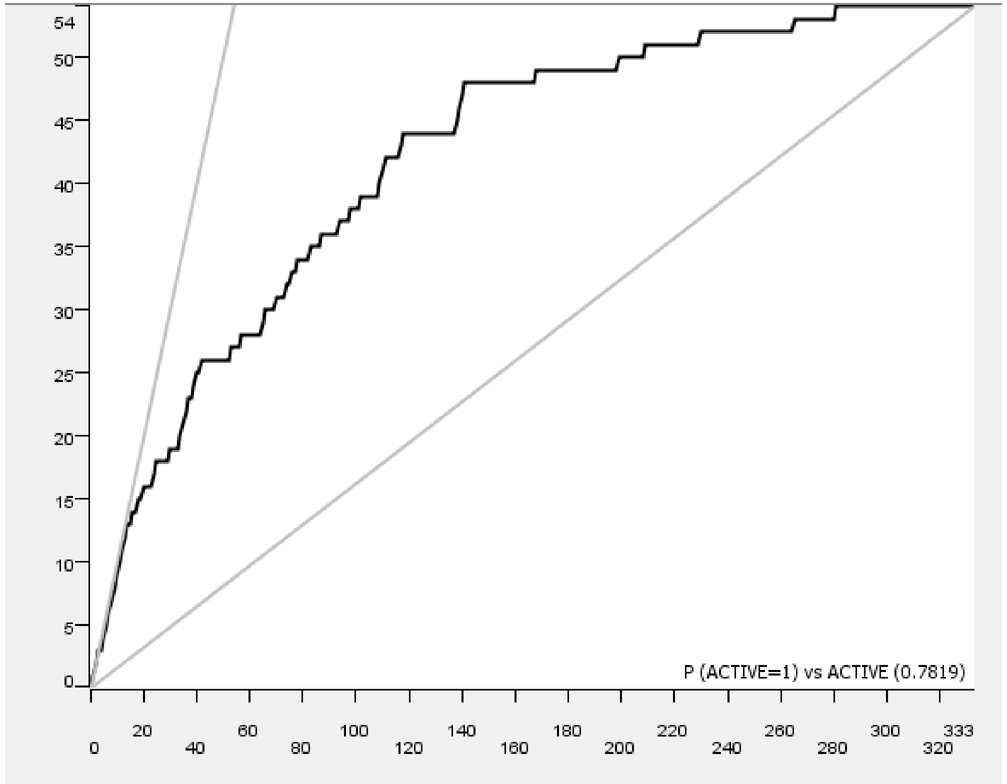

N=333

Top 10% Mean IC50	36.8 nM

	Predicted Active	Predicted Inactive
Active	28	26
Inactive	32	247

RF-<2.5 nM DefGood in Xa, 25% error

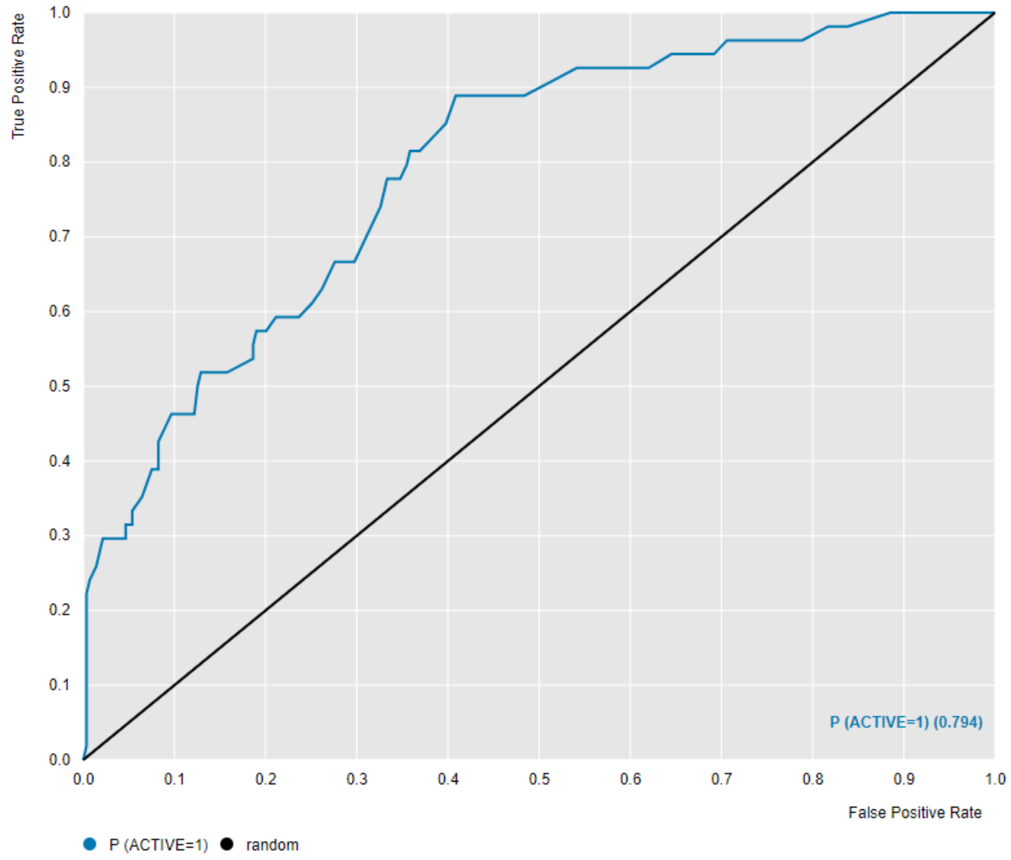

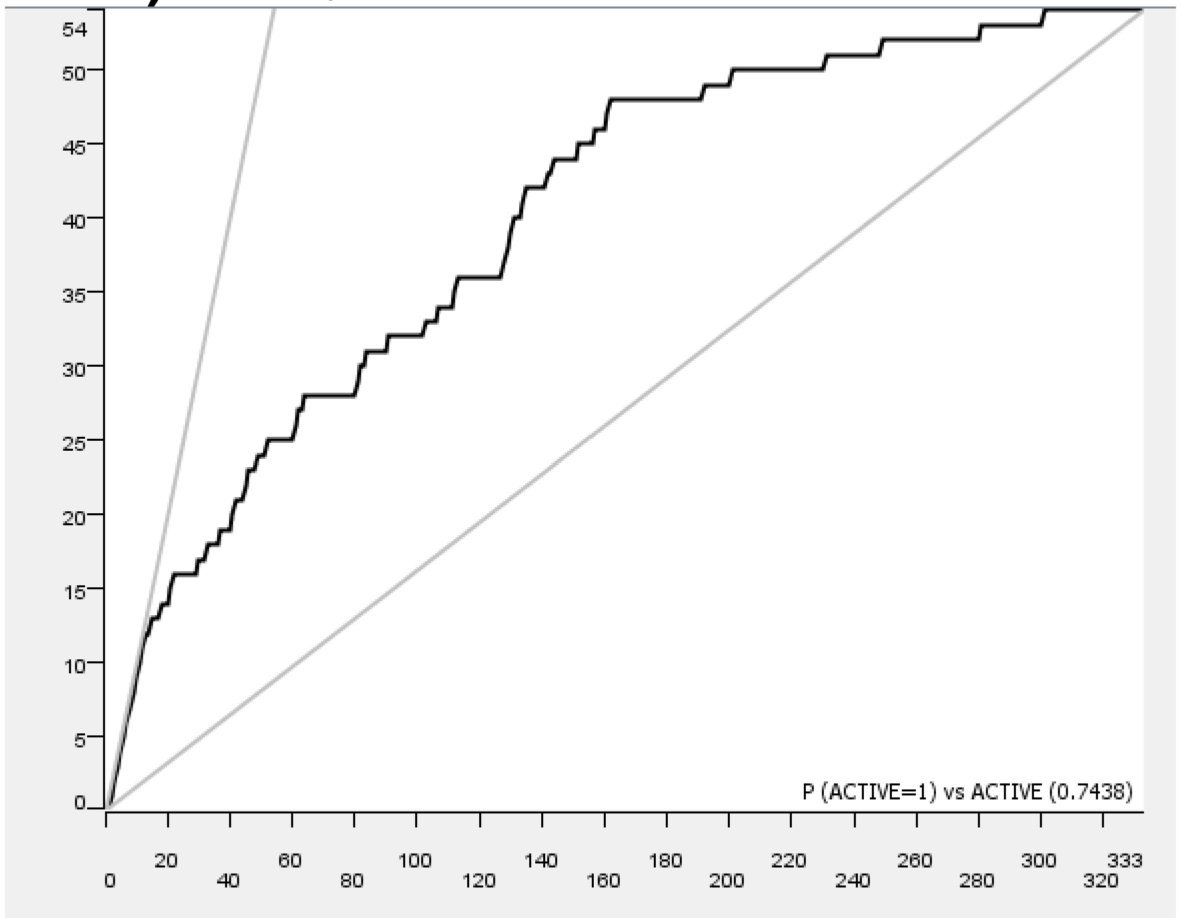

N=333

Top 10% Mean IC50	30.2 nM

	Predicted Active	Predicted Inactive
Active	25	29
Inactive	34	245

<2.5 nM DefGood in Xa, 30% error

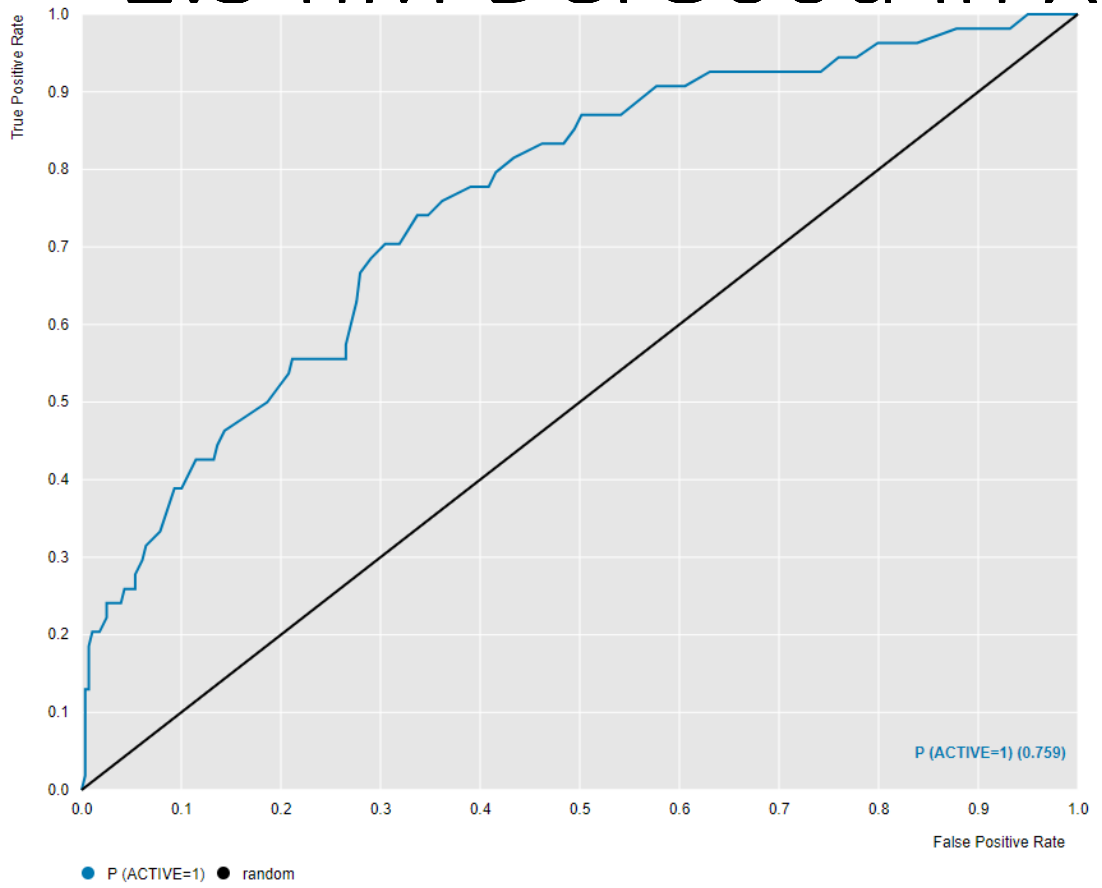

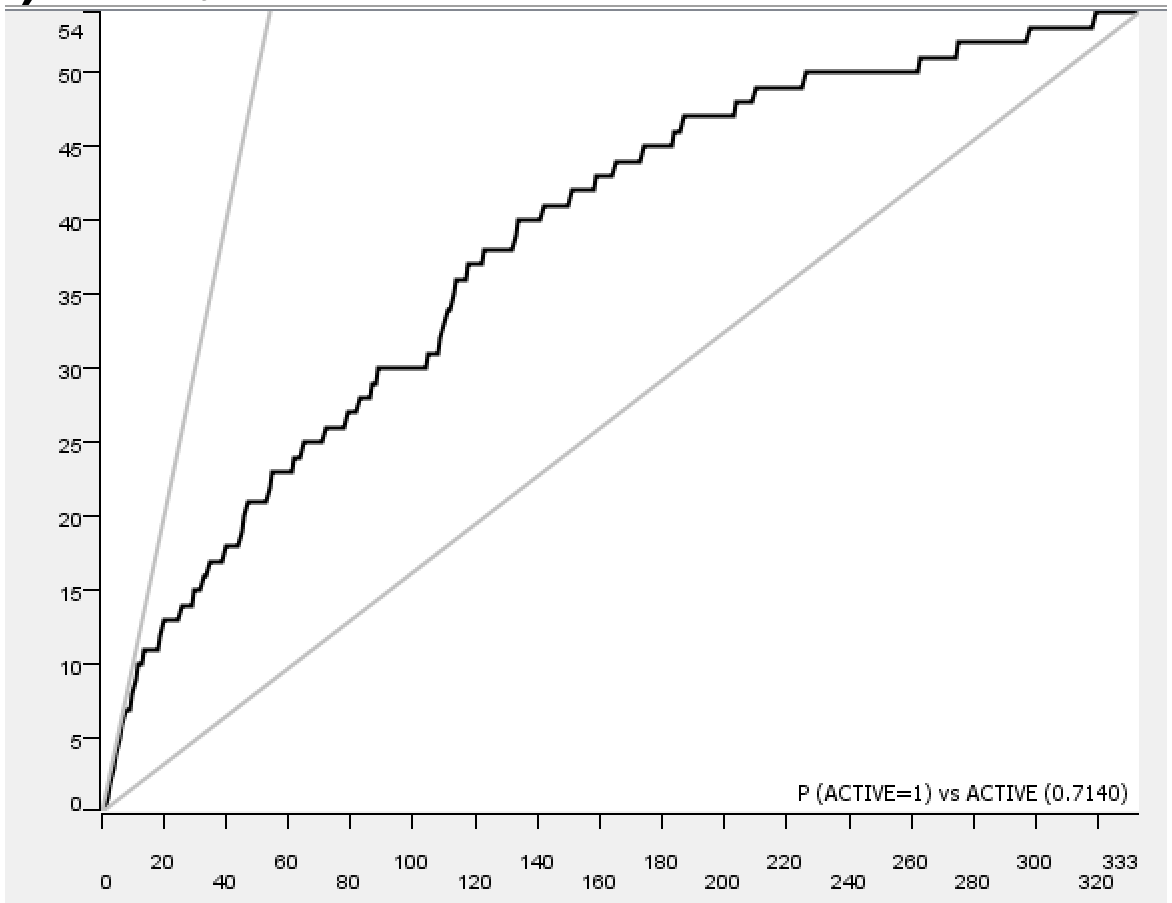

N=333

**Top 10% Mean IC50**	**1,000 nM**

	Predicted Active	Predicted Inactive
Active	27	27
Inactive	52	227

<2.5 nM DefGood in Xa, 10% error; Random seed = 429

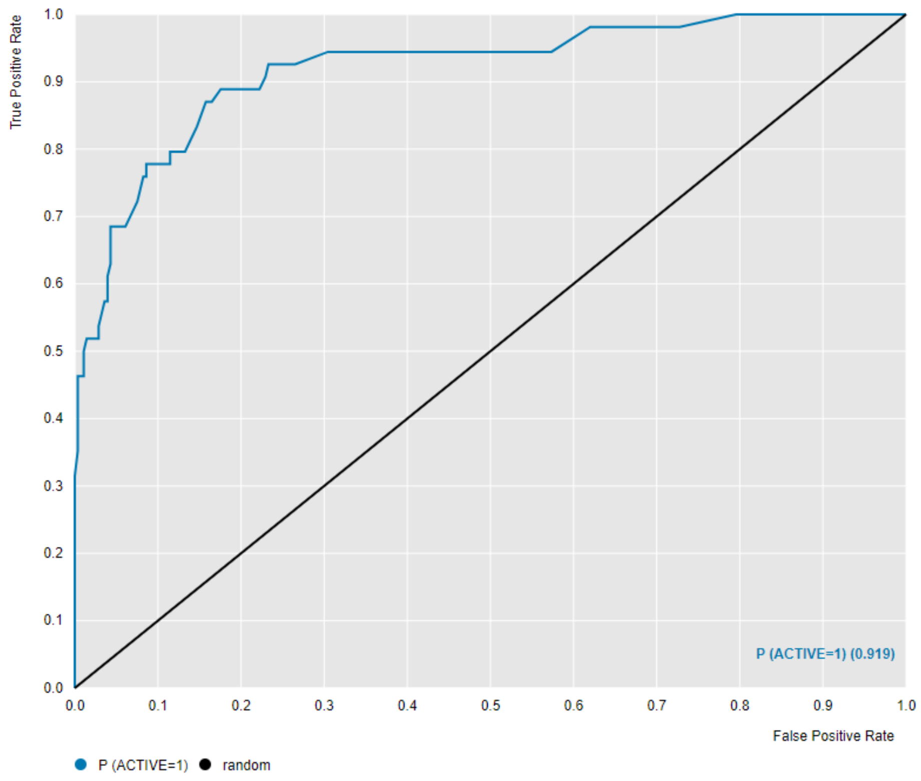

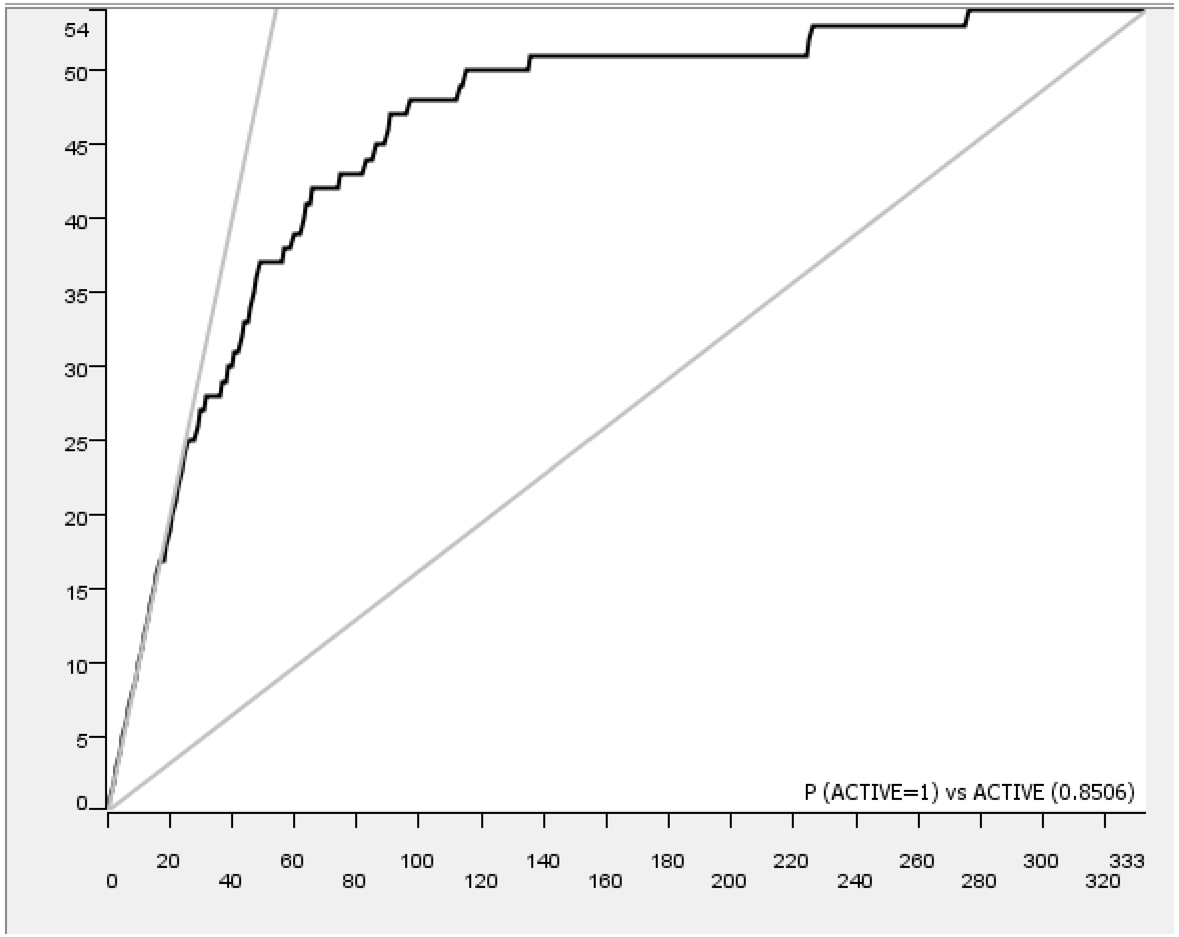

N=333

Top 10% Mean IC50	3.3 nM

	Predicted Active	Predicted Inactive
Active	37	17
Inactive	13	266

<2.5 nM DefGood in Xa, 20% error; Random seed = 429

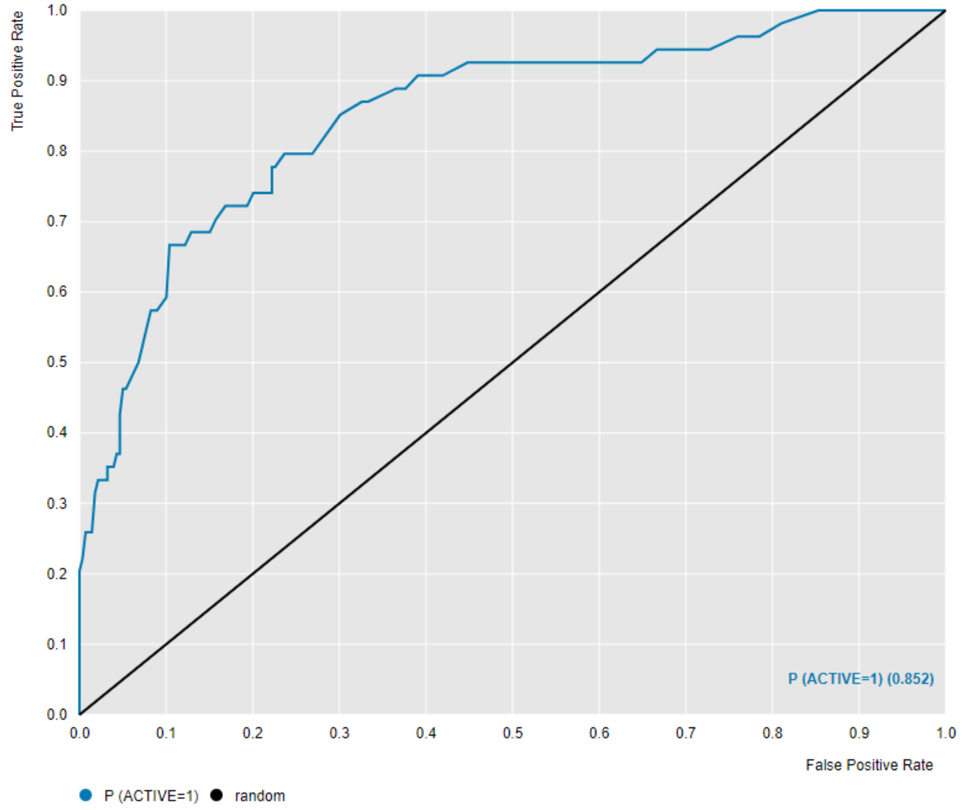

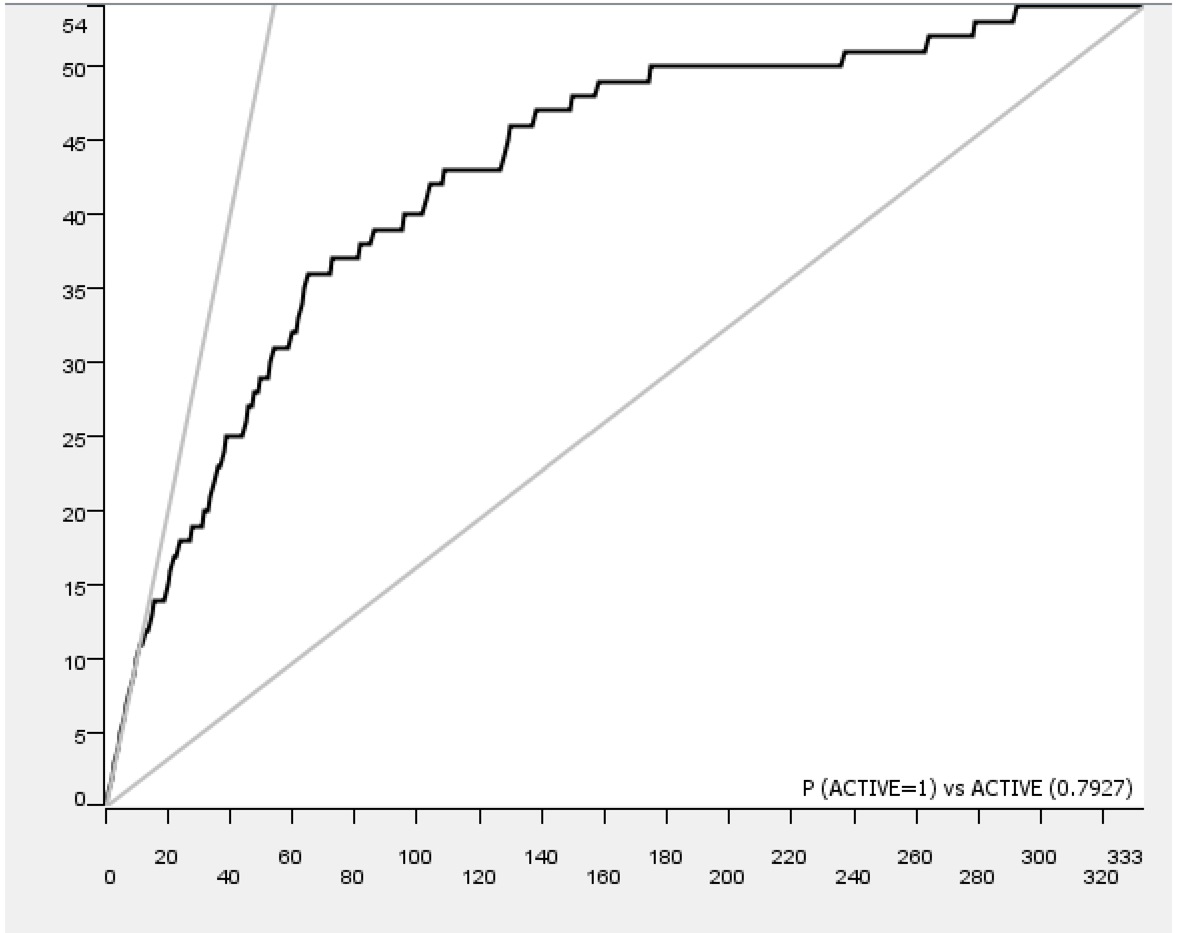

N=333

Top 10% Mean IC50	48.7 nM<br>One (1,100 nM) mistake

	Predicted Active	Predicted Inactive
Active	31	23
Inactive	23	256

<2.5 nM DefGood in Xa, 25% error; Random seed = 429

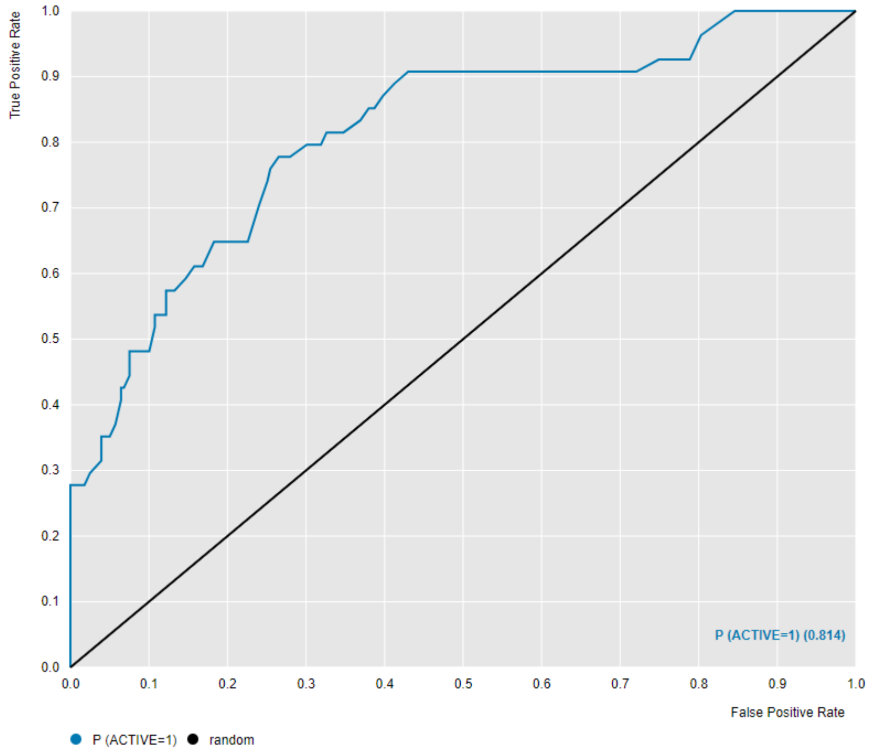

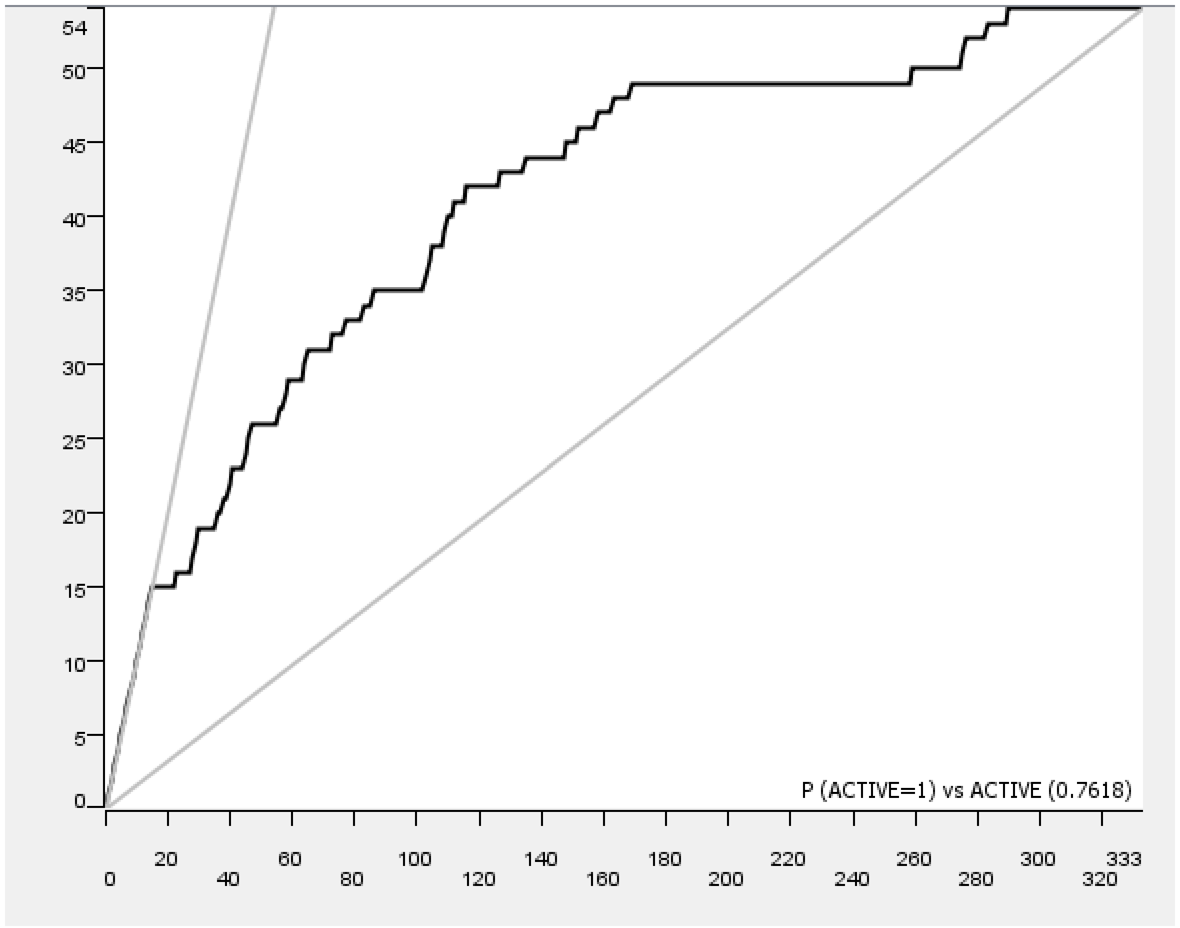

N=333

**Top 10% Mean IC50**	**60.5 nM One (1,100 nM) mistake**

	**Predicted Active**	**Predicted Inactive**
Active	29	25
Inactive	34	245

<2.5 nM DefGood in Xa, 30% error; Random seed = 429

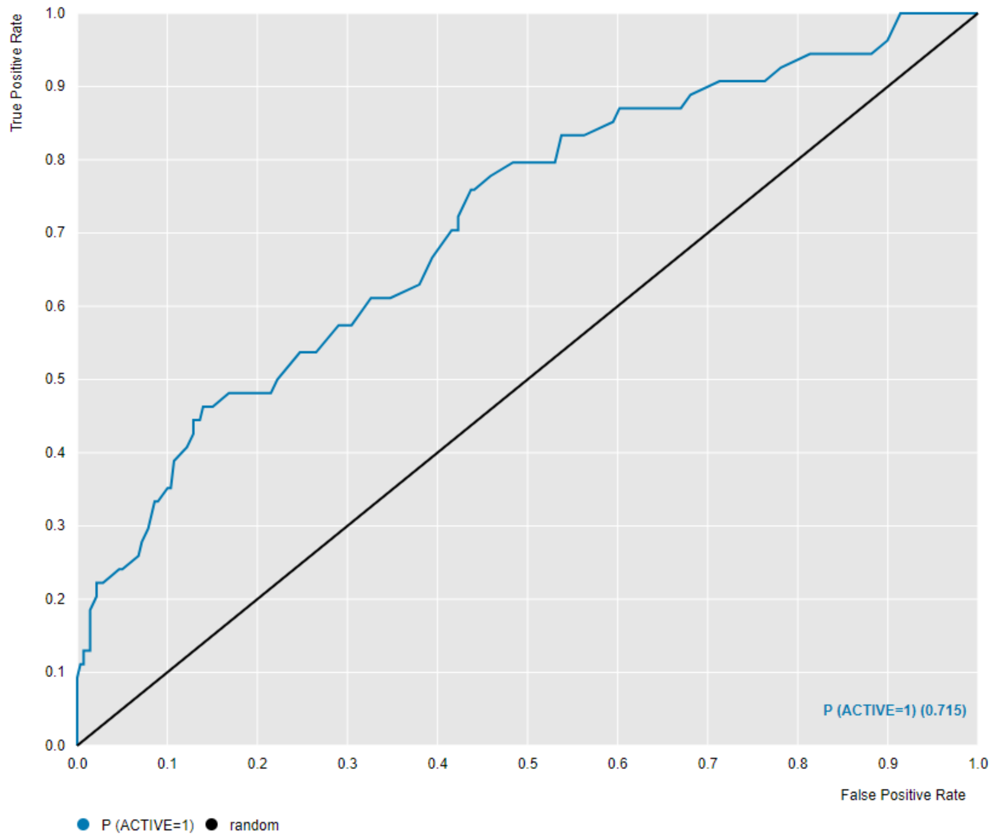

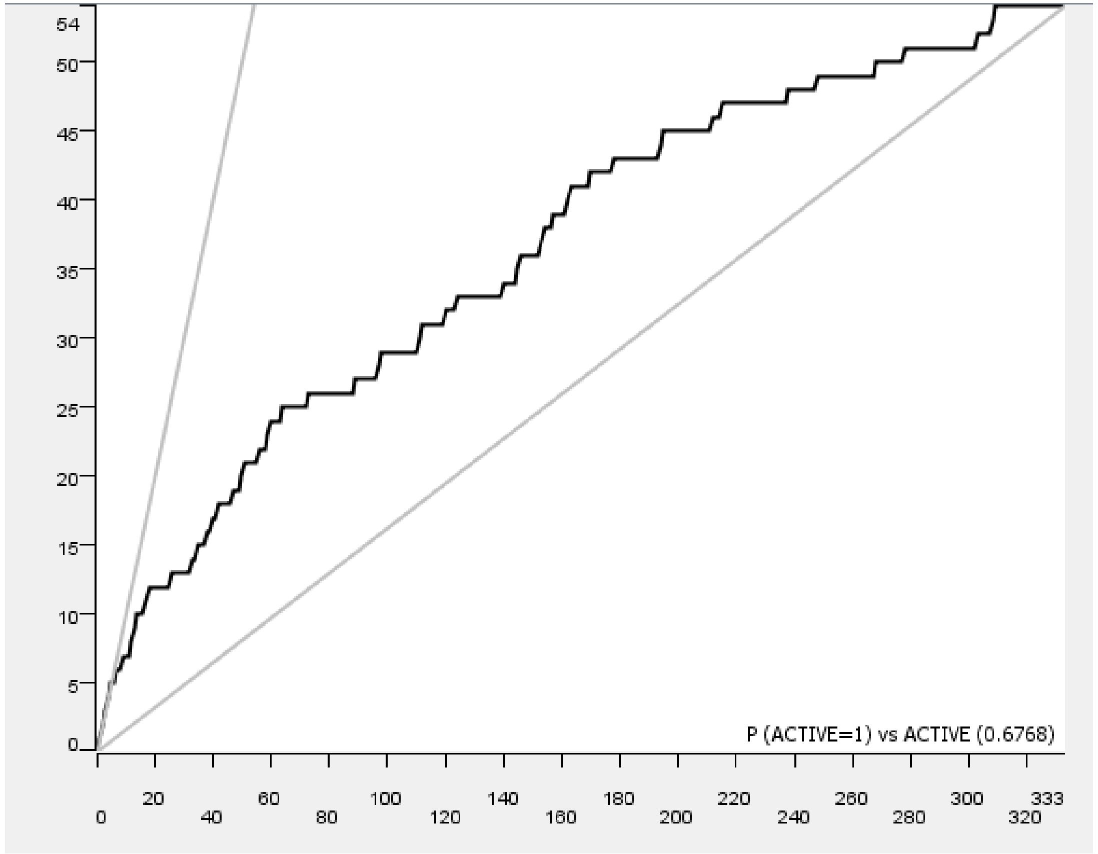

N=333

Top 10% Mean IC50	530 nM

	Predicted Active	Predicted Inactive
Active	26	28
Inactive	49	230

<2.5 nM DefGood in Xa, 10% error; Random seed = 121783

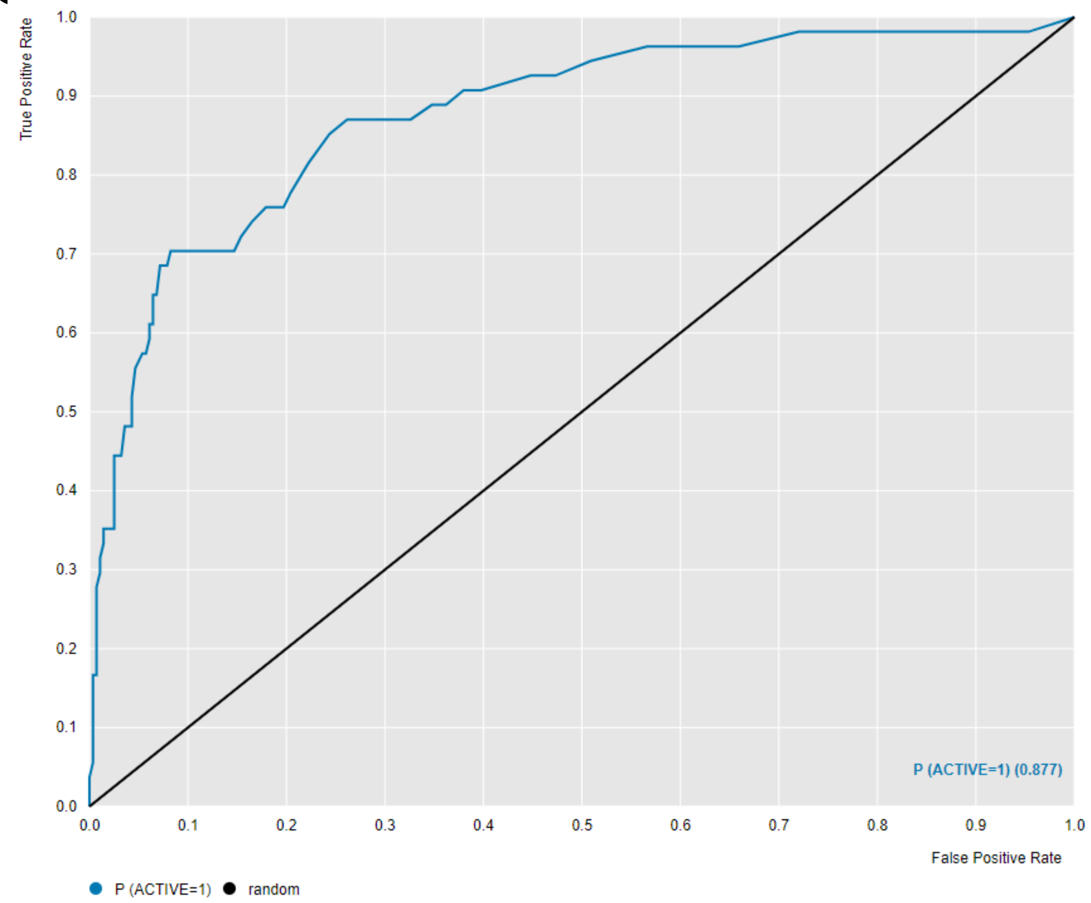

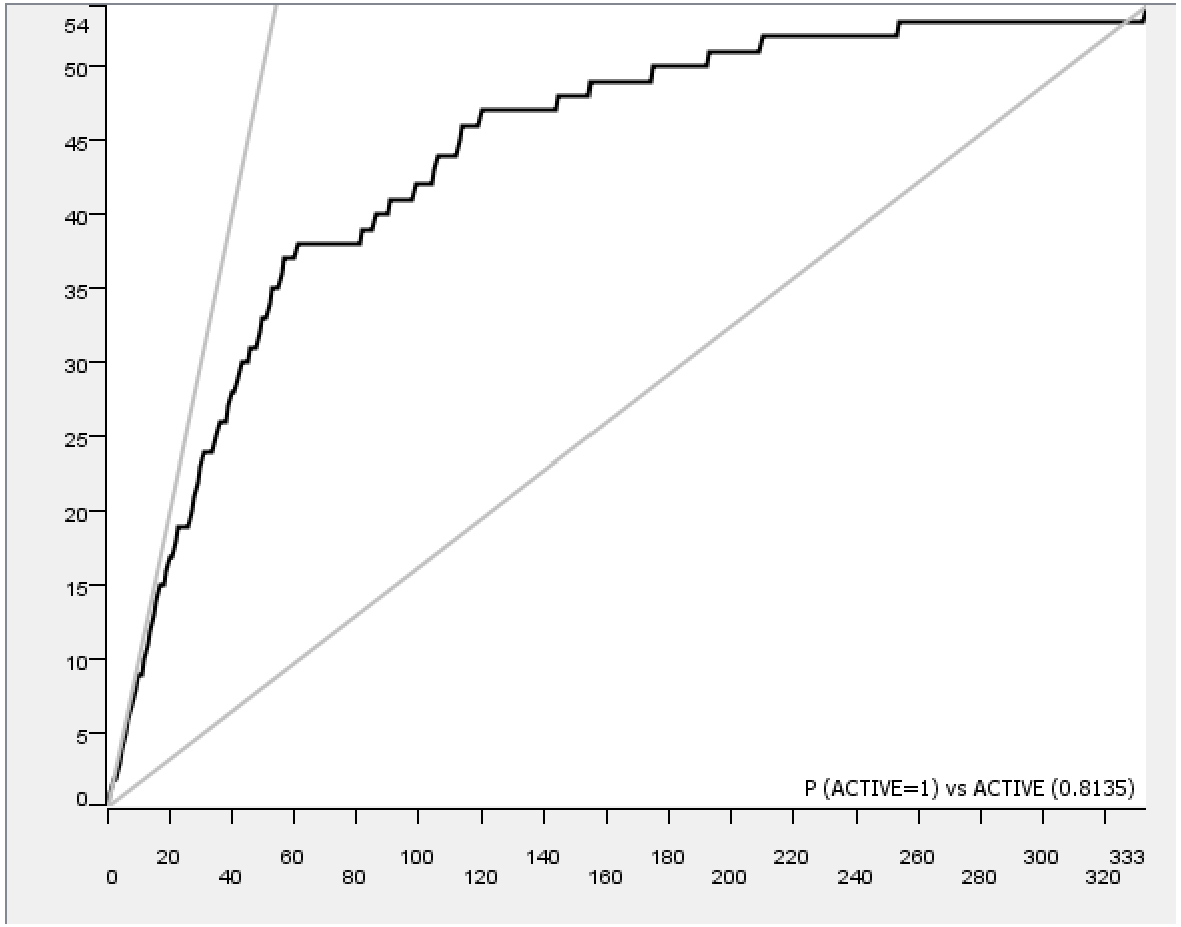

N=333

**Top 10% Mean IC50**	**12.5 nM**

	**Predicted Active**	**Predicted Inactive**
Active	30	24
Inactive	13	266

<2.5 nM DefGood in Xa, 20% error; Random seed = 121783

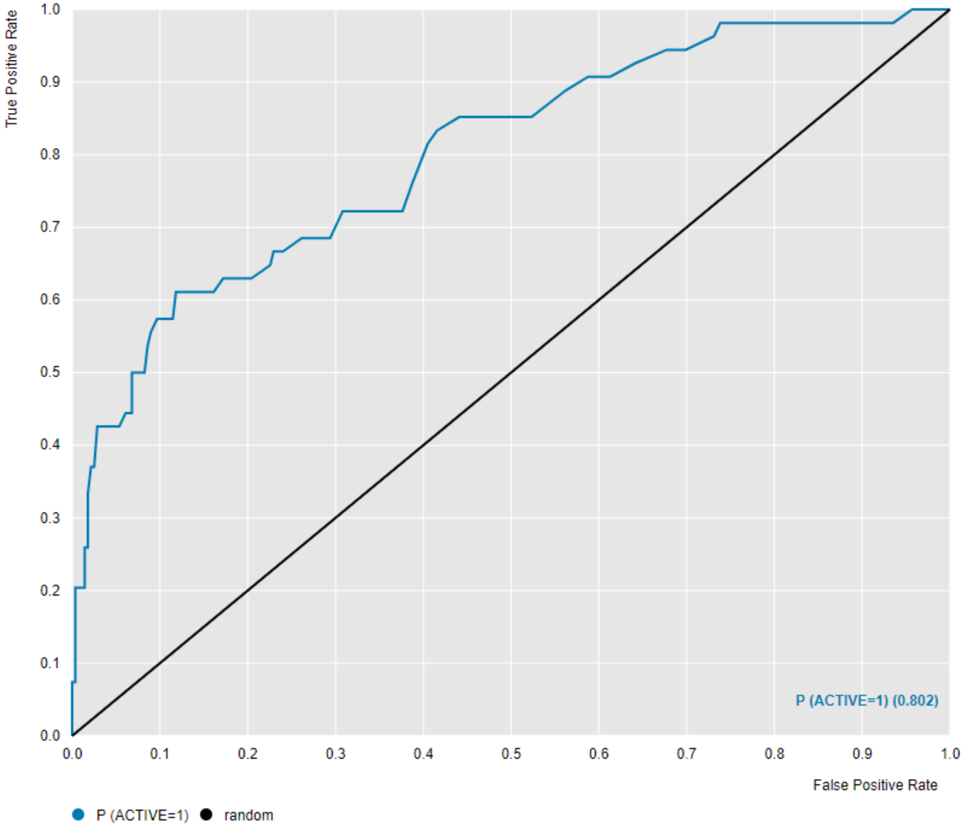

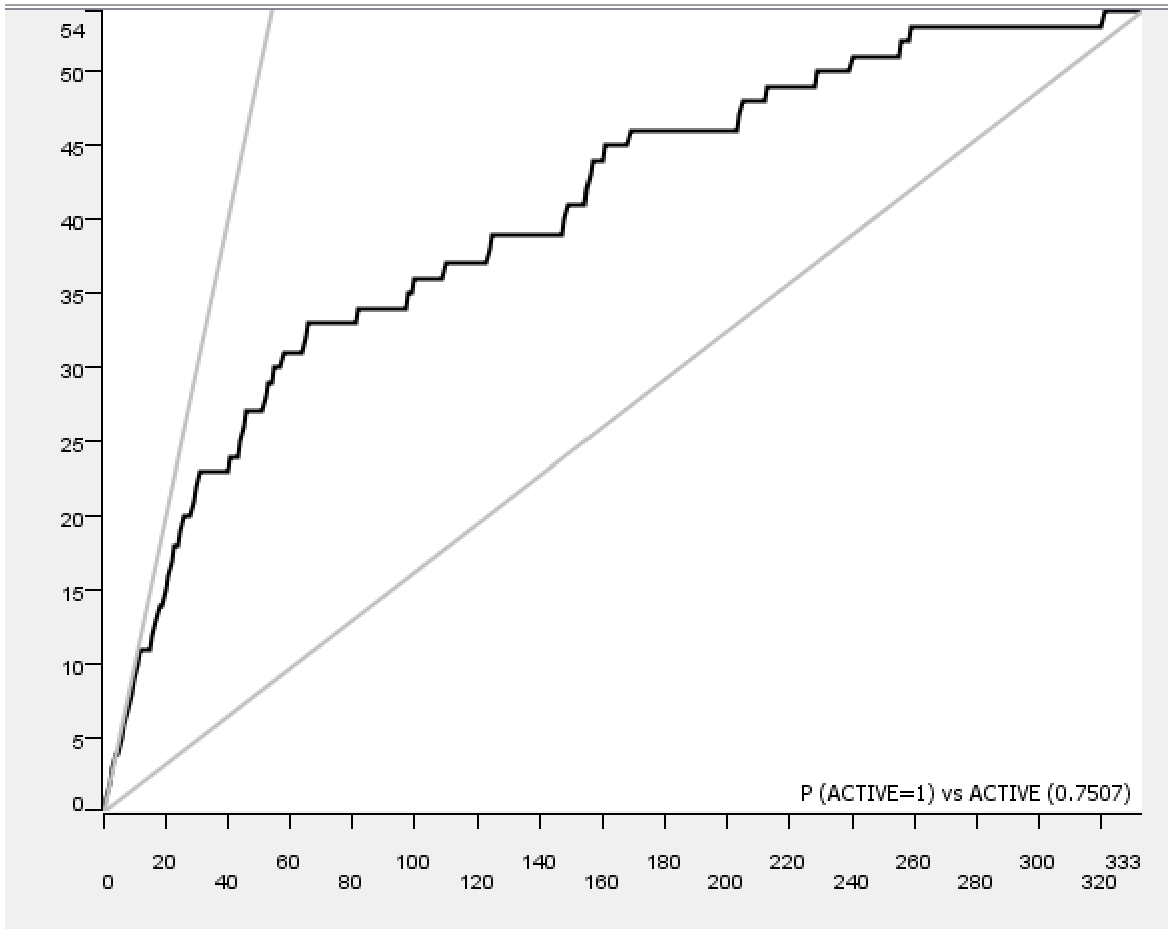

N=333

Top 10% Mean IC50	200 nM (2 micromolar mistakes)

	Predicted Active	Predicted Inactive
Active	27	27
Inactive	22	257

<2.5 nM DefGood in Xa, 25% error; Random seed = 121783

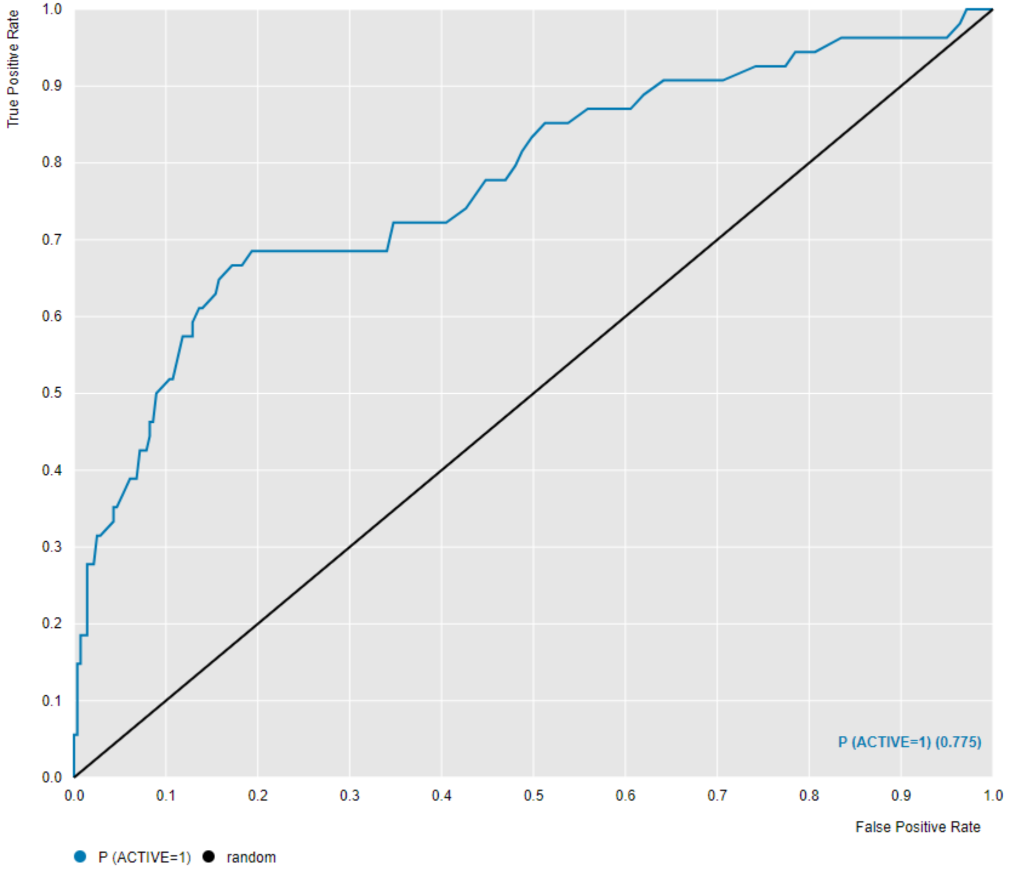

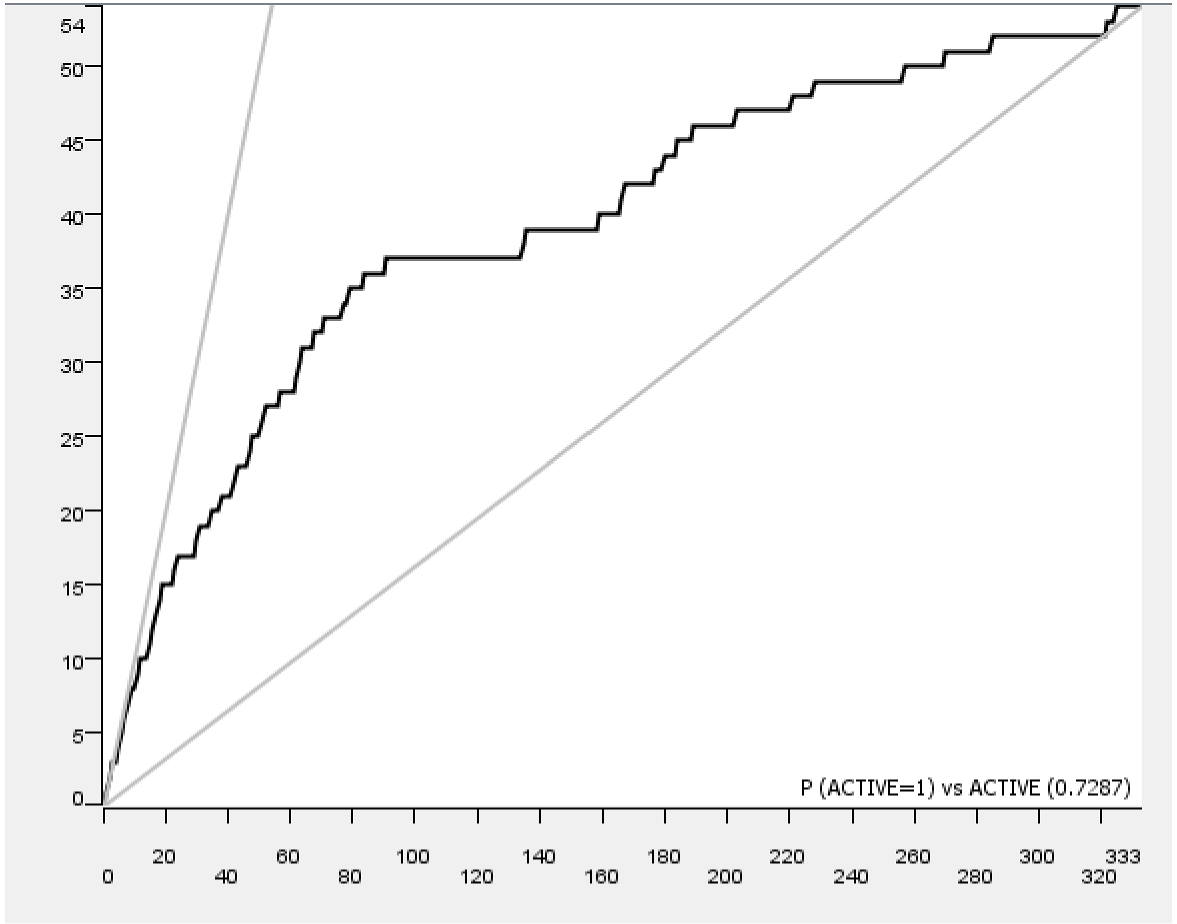

N=333

**Top 10% Mean IC50**	**130 nM (One mistake - 930 nM)**

	Predicted Active	Predicted Inactive
Active	31	23
Inactive	33	246

<2.5 nM DefGood in Xa, 30% error; Random seed = 121783

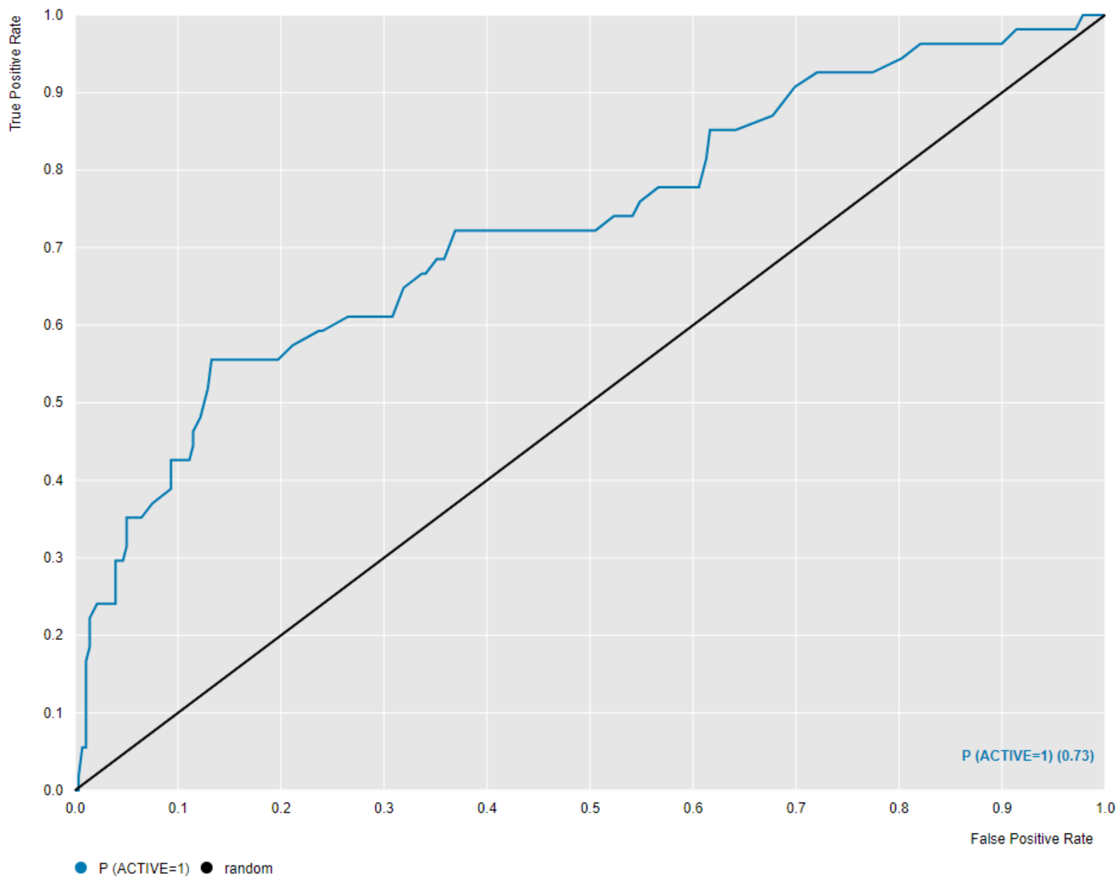

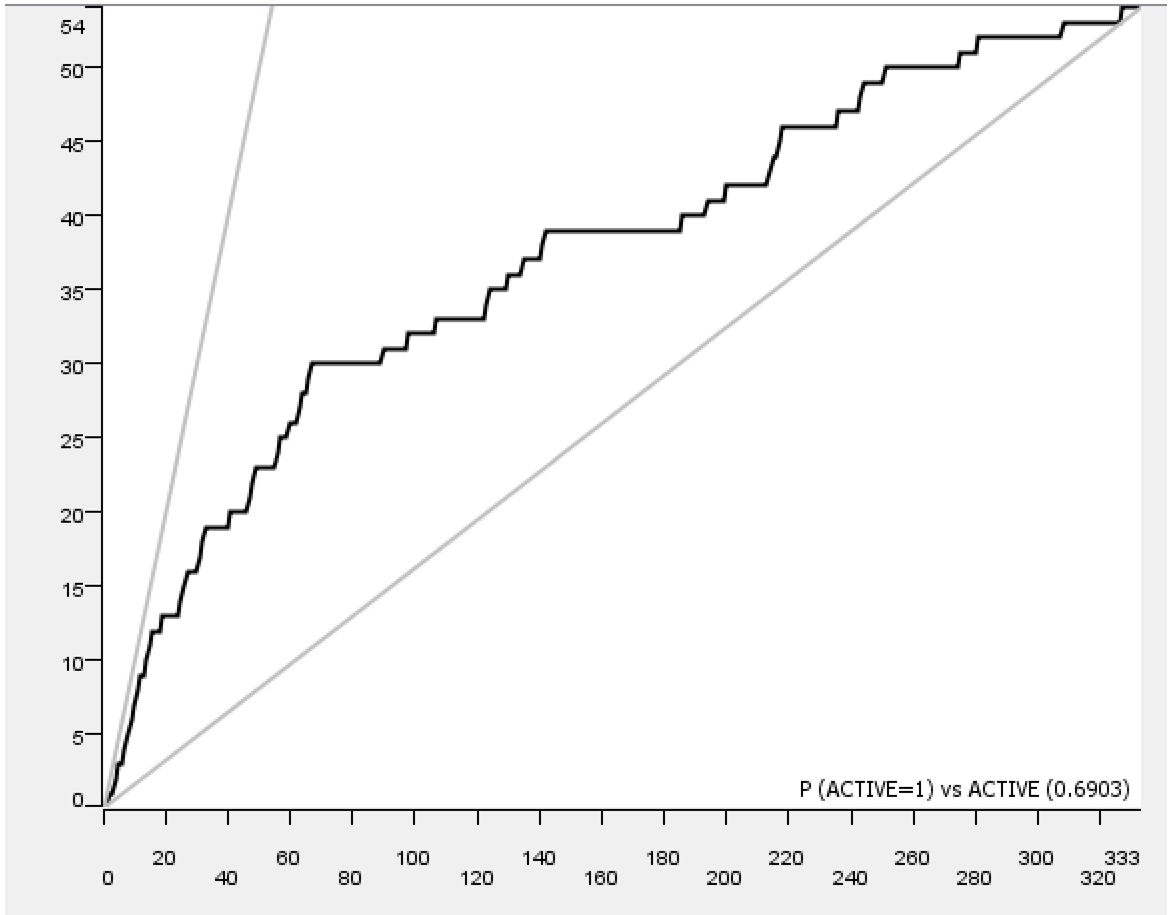

N=333

**Top 10% Mean IC50**	**680 nM**

	Predicted Active	Predicted Inactive
Active	30	24
Inactive	51	228

Conclusion - RF

- A Random Forrest could be generated for Factor Xa with a decision value of <2.5 nM as defined as active. The predictivity of the algorithm could be preserved in three random train/test cycles. The point of failure was 30%, 30% and 30% error.

PNN - <2.5 nM DefGood in Xa, 5% error ; Random seed = 1515533876005

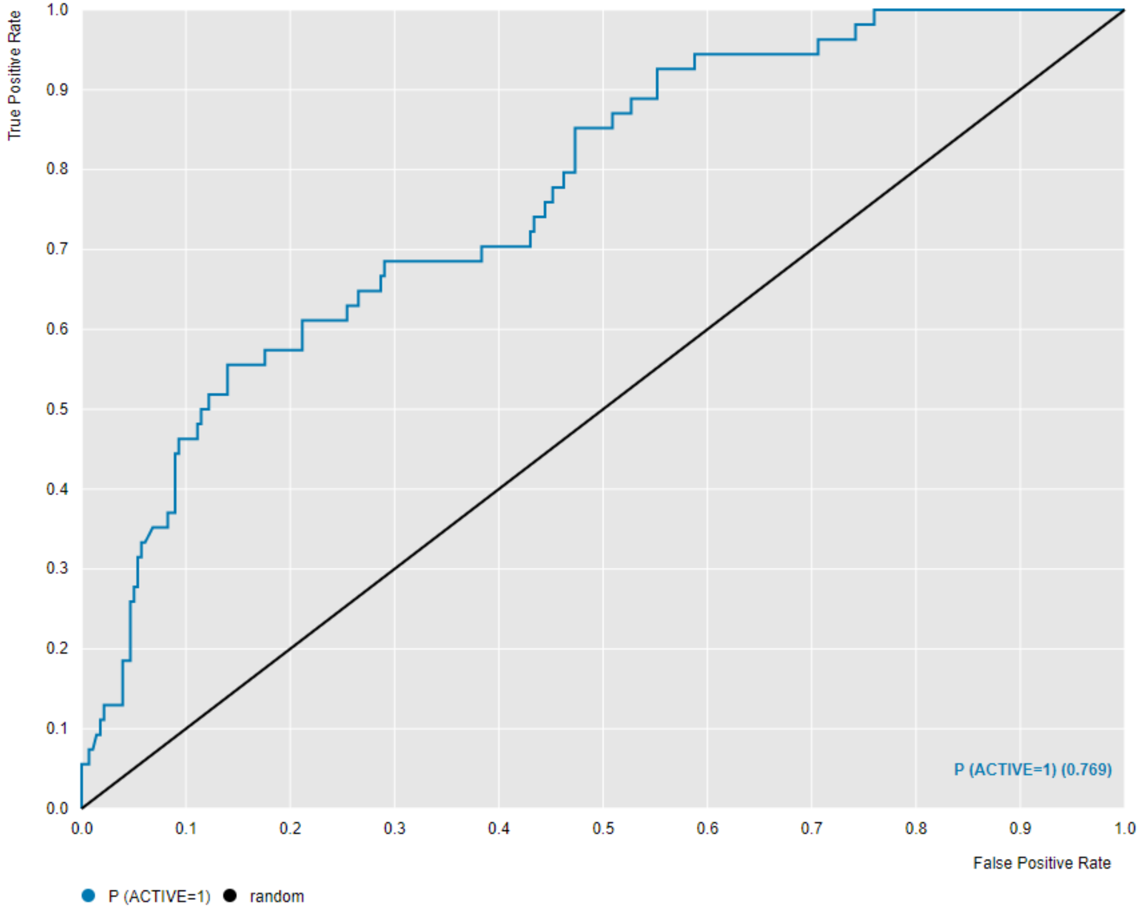

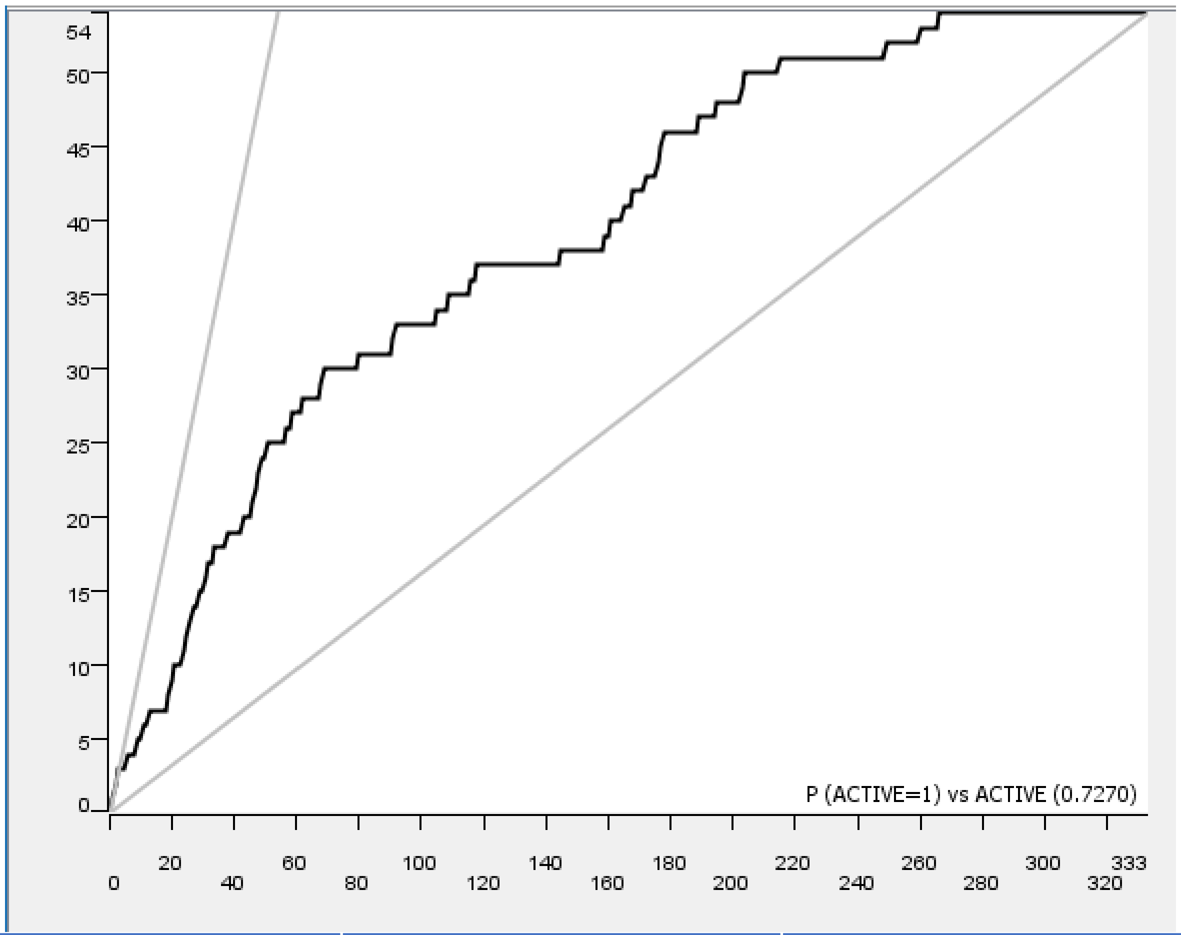

N=333

**Top 10% Mean IC50**	**83.5 nM (one 2,000 nM compound)**

	**Predicted Active**	**Predicted Inactive**
Active	19	35
Inactive	20	259

PNN - <2.5 nM DefGood in Xa, 10% error

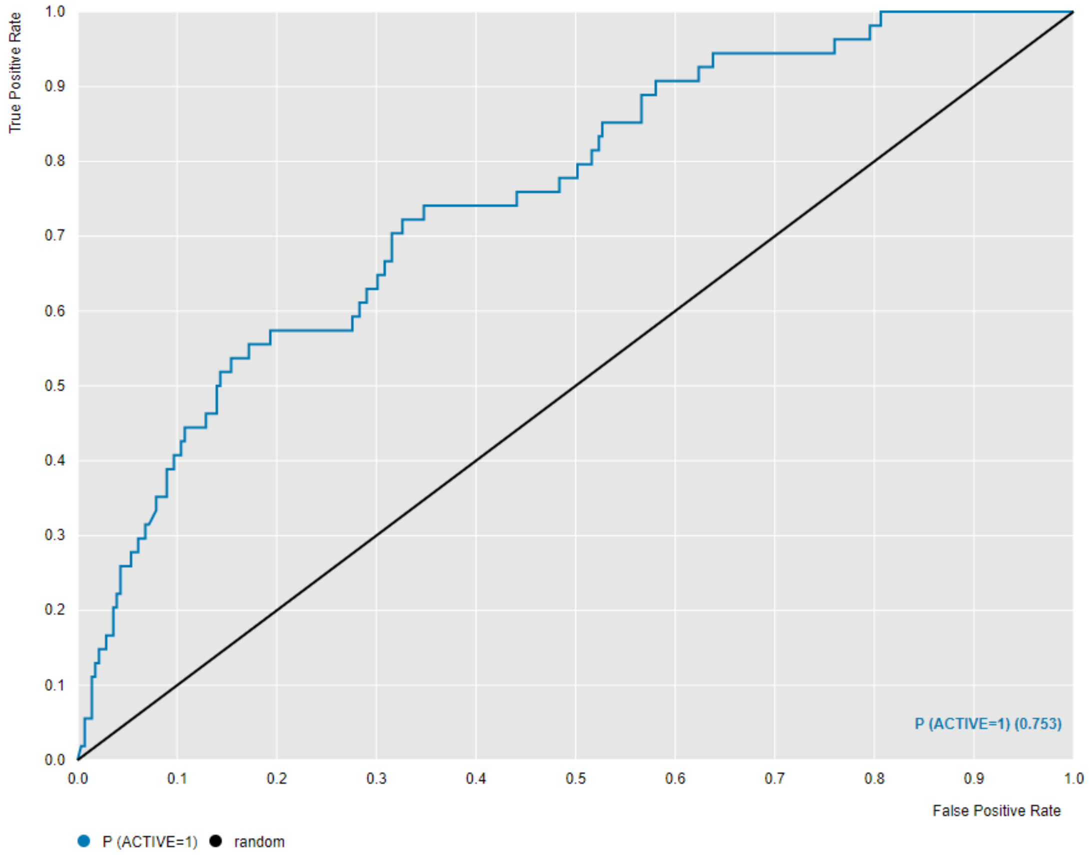

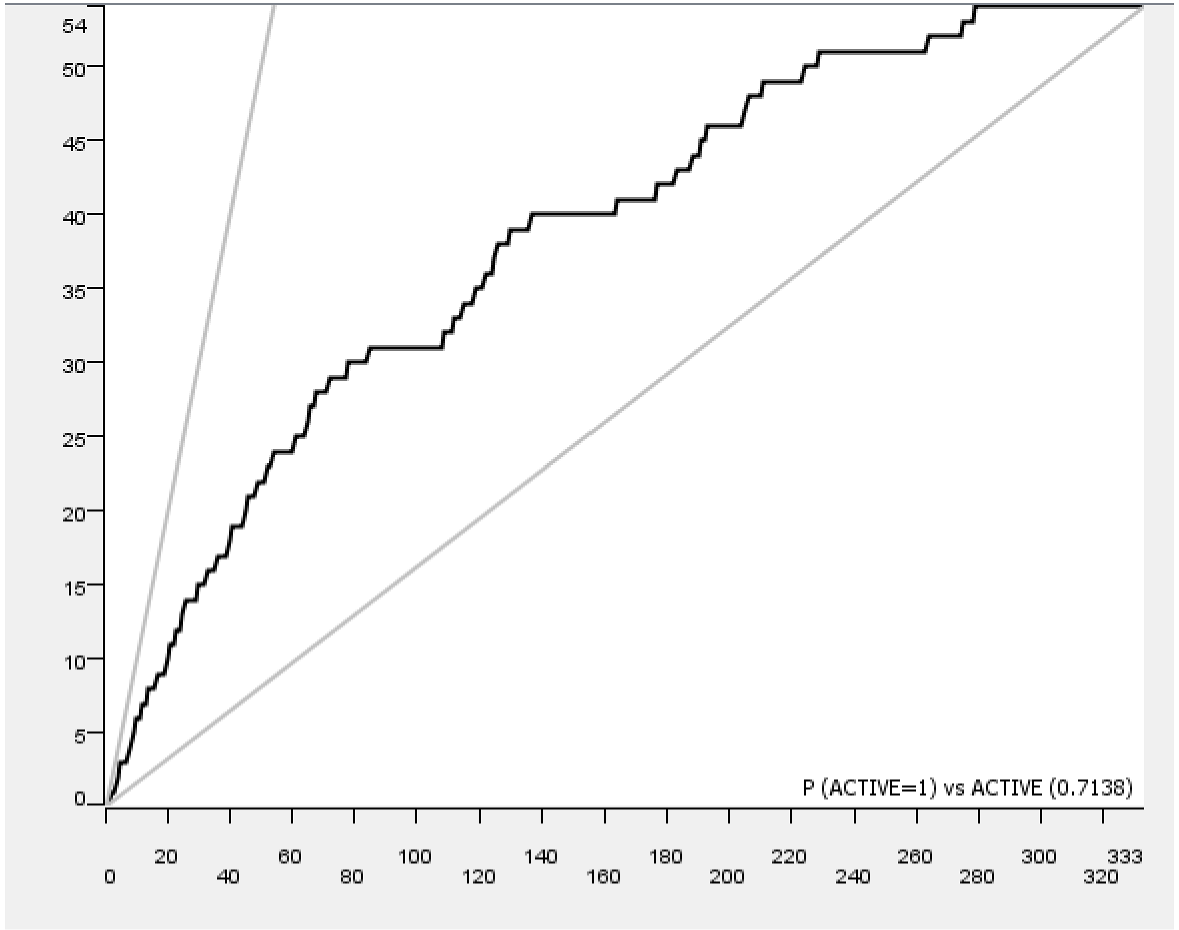

N=333

**Top 10% Mean IC50**	**120 nM (two micromolar mistakes)**

	Predicted Active	Predicted Inactive
Active	19	35
Inactive	23	256

PNN - <2.5 nM DefGood in Xa, 15% error

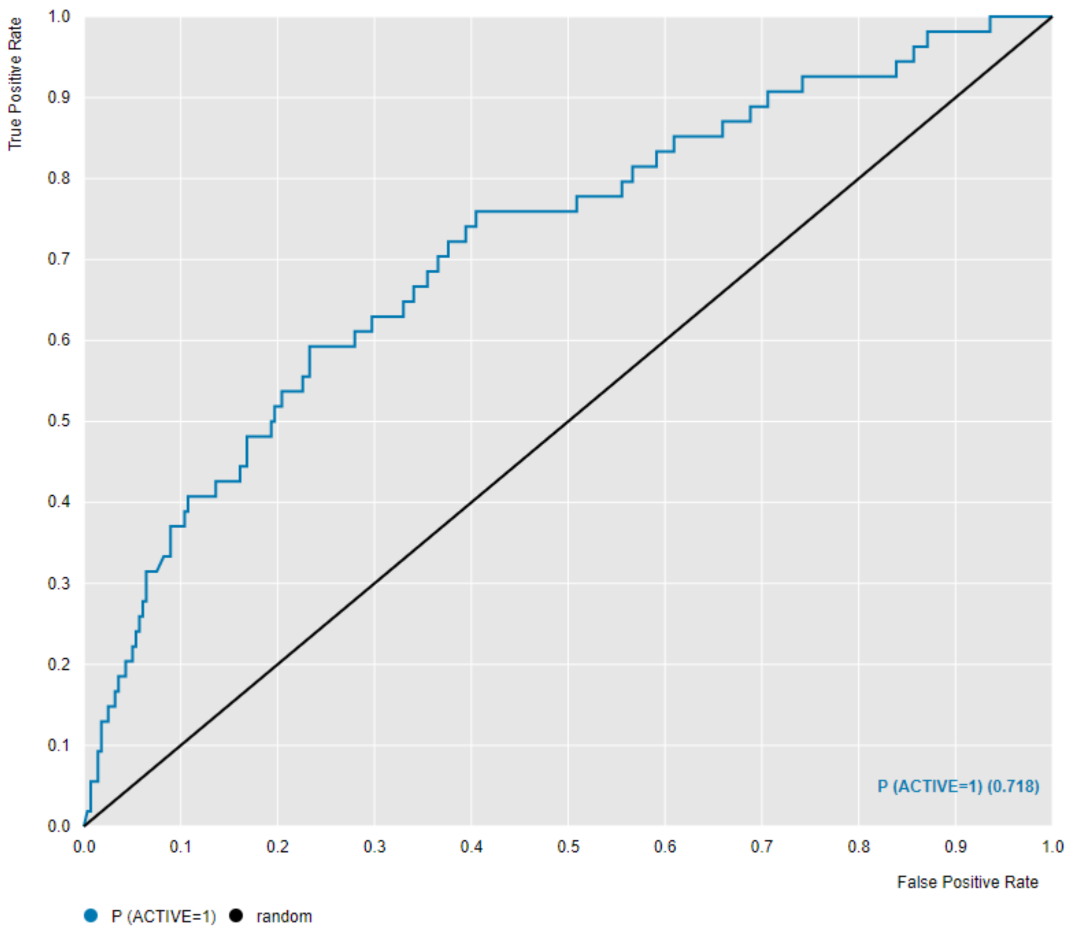

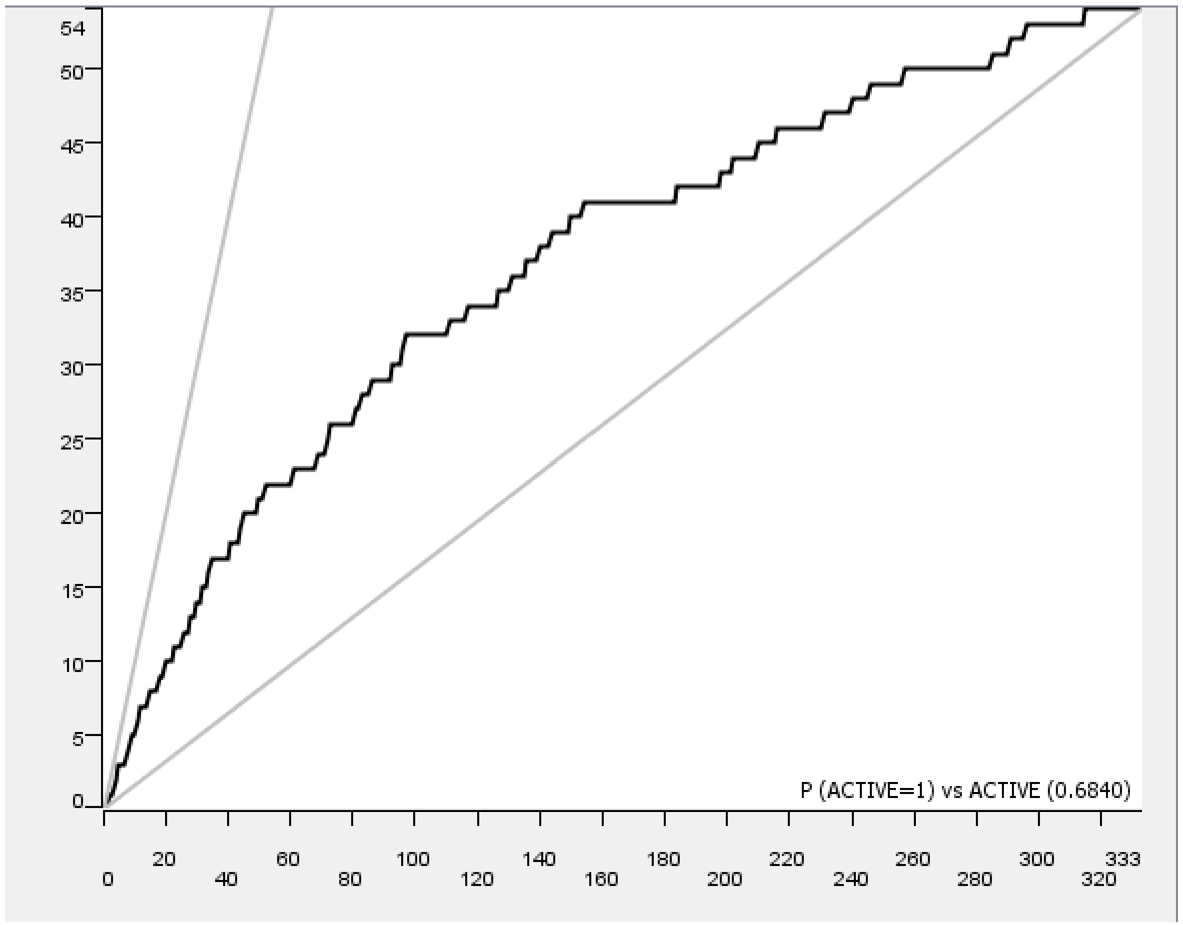

N=333

Top 10% Mean IC50	120 nM (two micromolar mistakes)

	Predicted Active	Predicted Inactive
Active	20	34
Inactive	28	251

PNN - <2.5 nM DefGood in Xa, 20% error

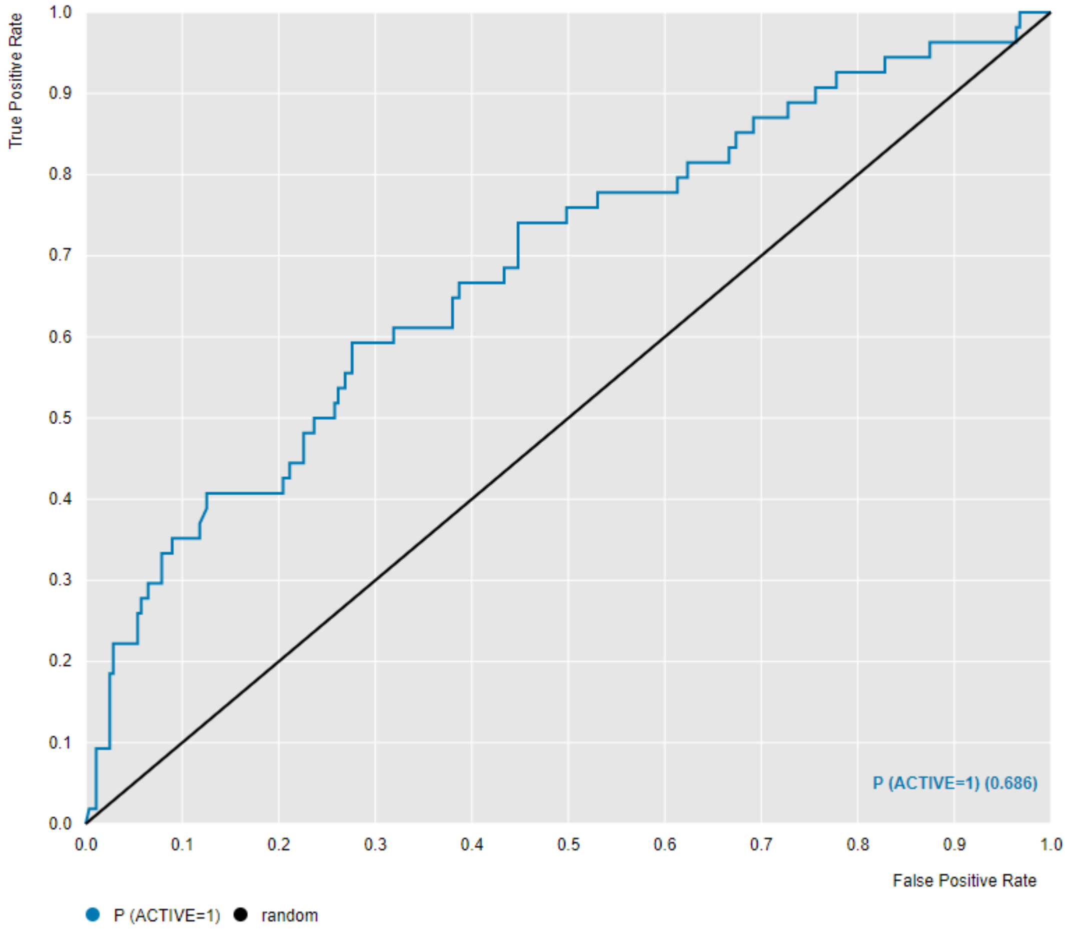

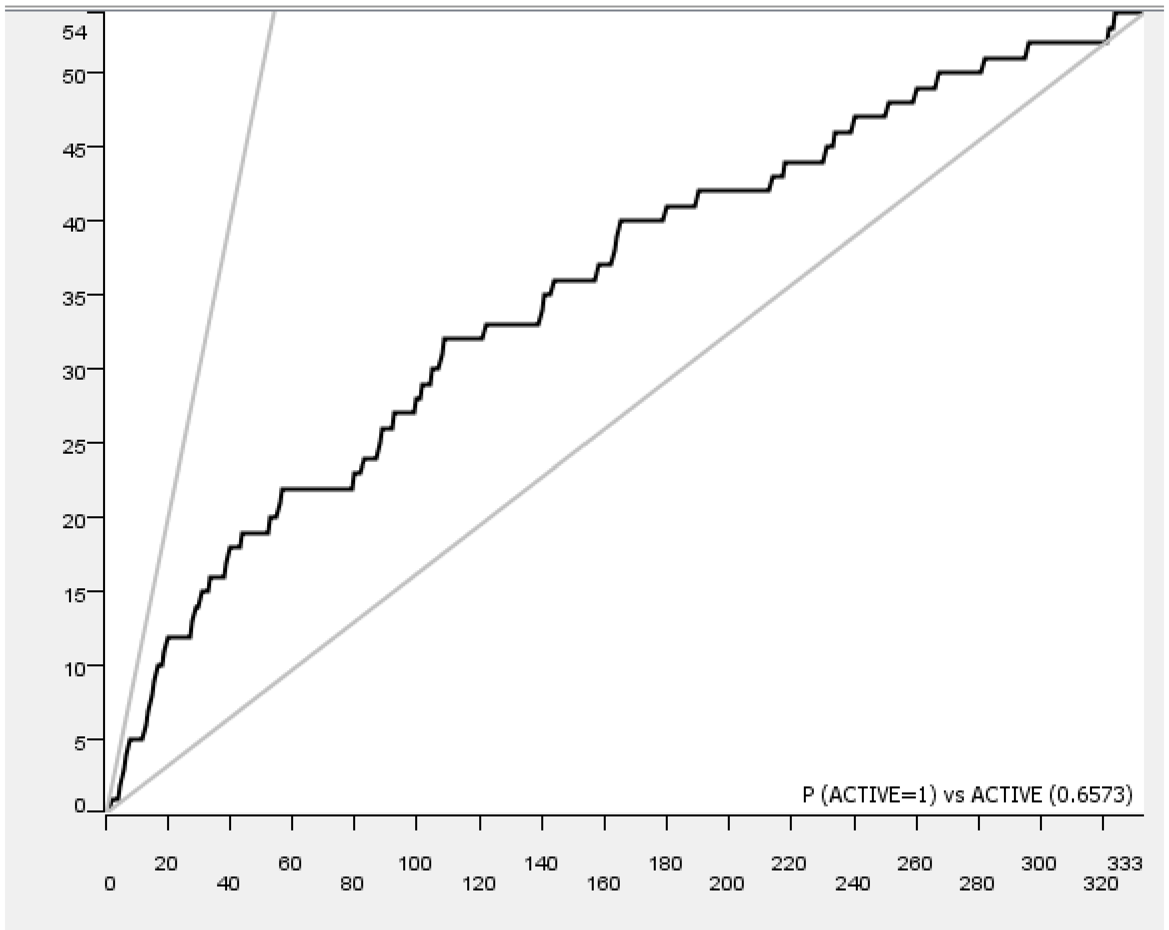

N=333

Top 10% Mean IC50	89 nM

	Predicted Active	Predicted Inactive
Active	22	32
Inactive	39	240

PNN - <2.5 nM DefGood in Xa, 25% error

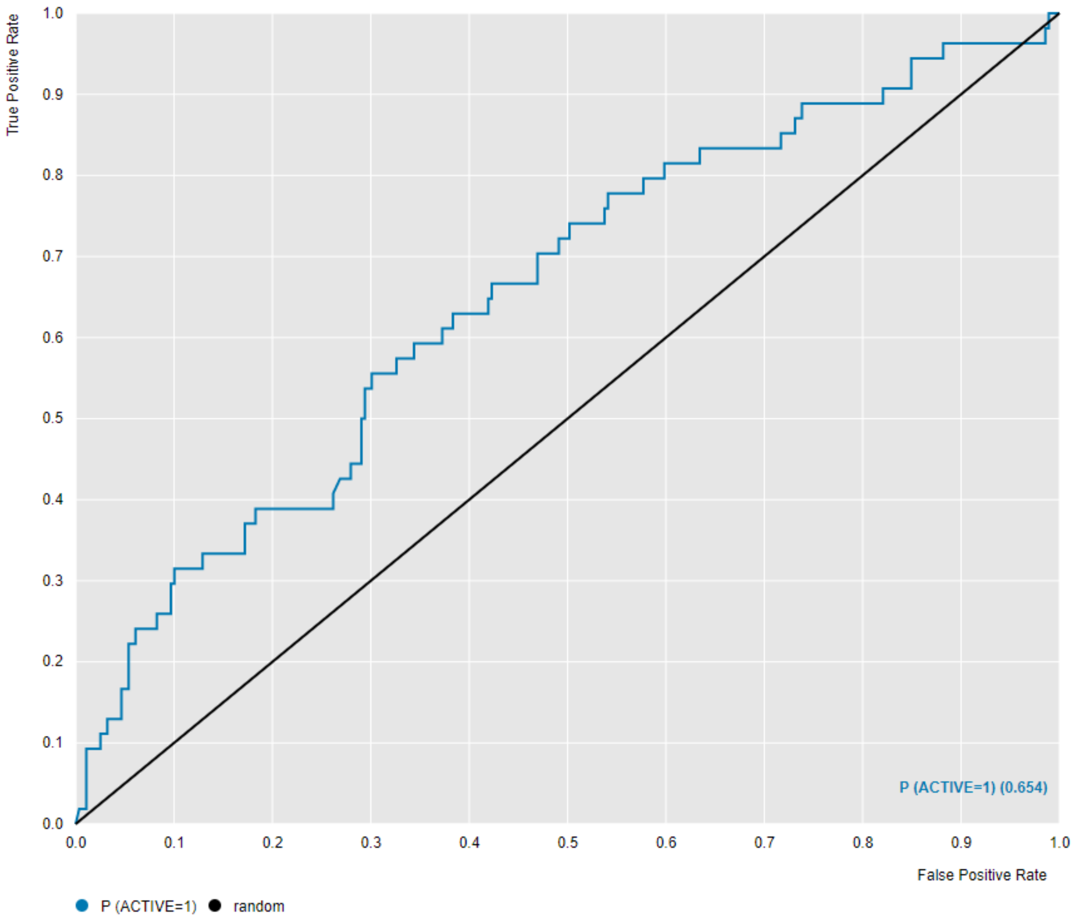

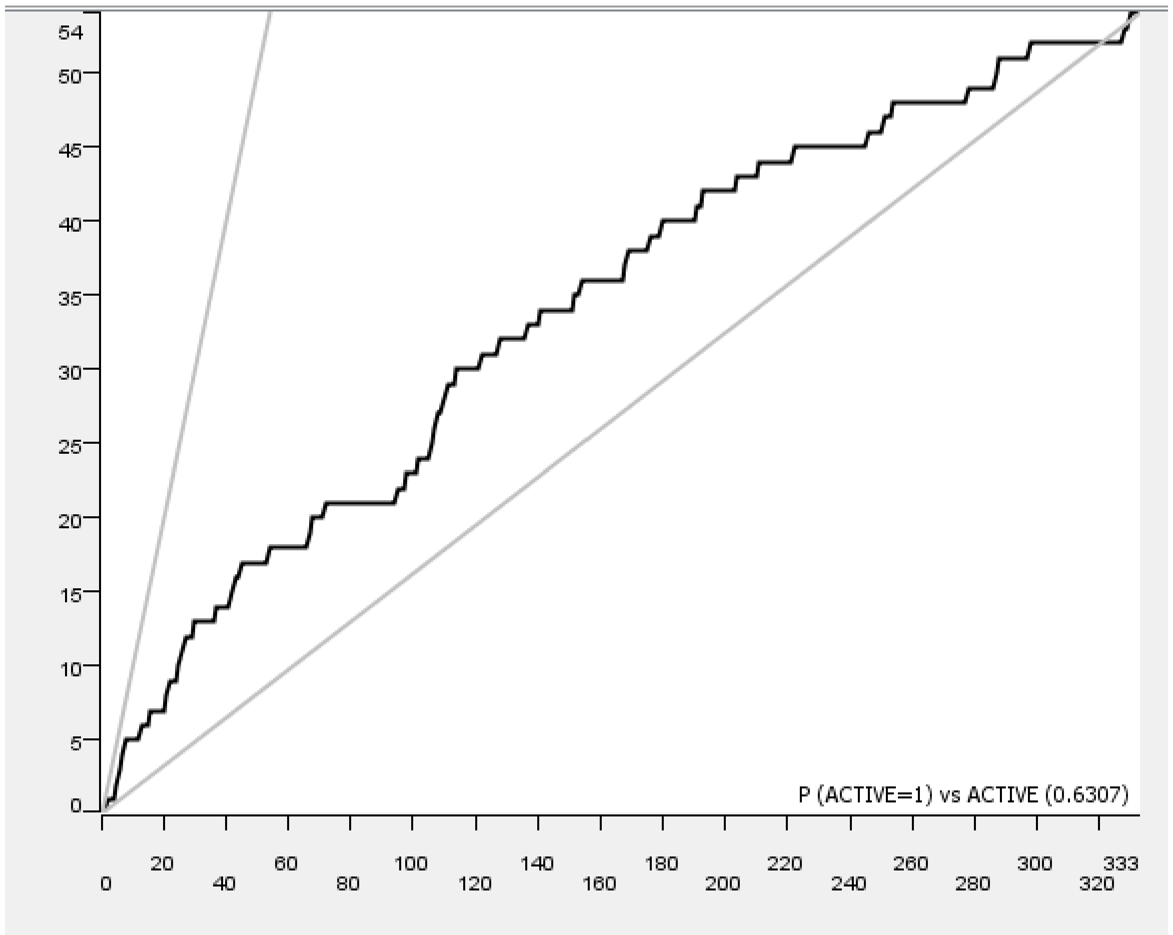

N=333

Top 10% Mean IC50	915 nM

	Predicted Active	Predicted Inactive
Active	21	33
Inactive	56	223

PNN - <2.5 nM DefGood in Xa, 5% error; Random seed = 429

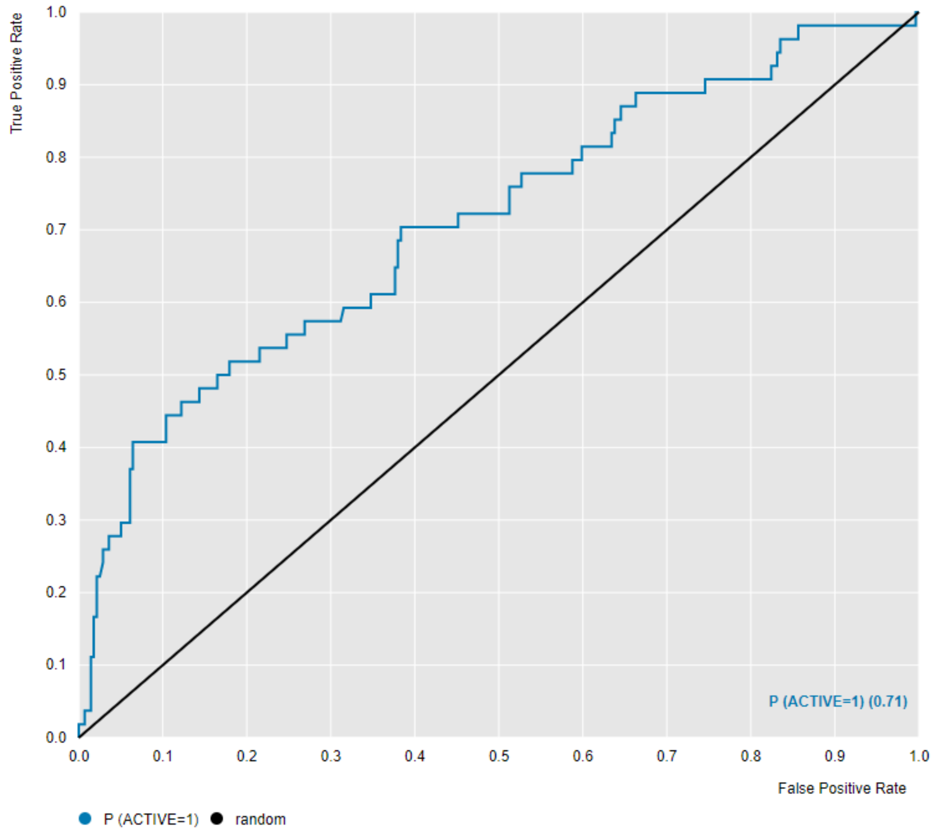

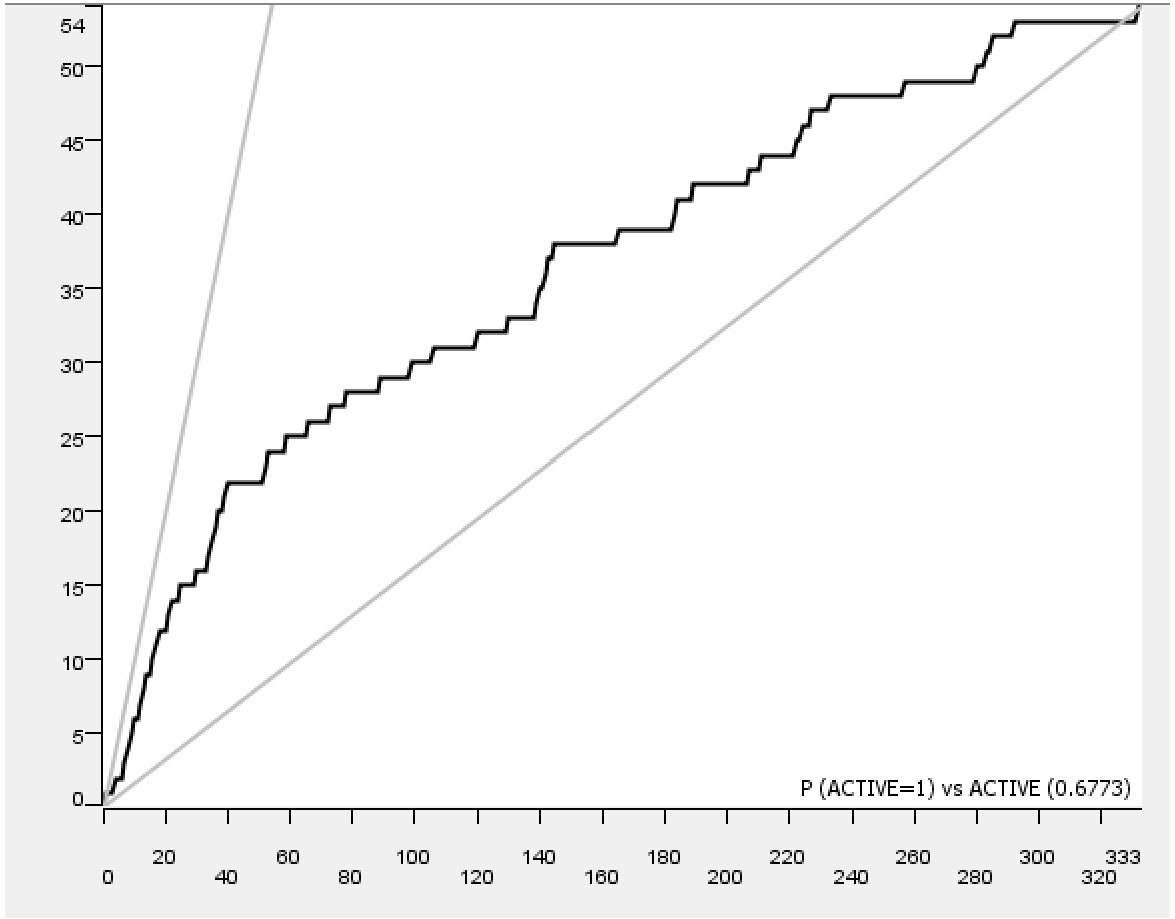

N=333

**Top 10% Mean IC50**	**66.3 nM (one >800 nM Compound)**

	**Predicted Active**	**Predicted Inactive**
Active	15	39
Inactive	10	269

PNN - <2.5 nM DefGood in Xa, 10% error; Random seed = 429

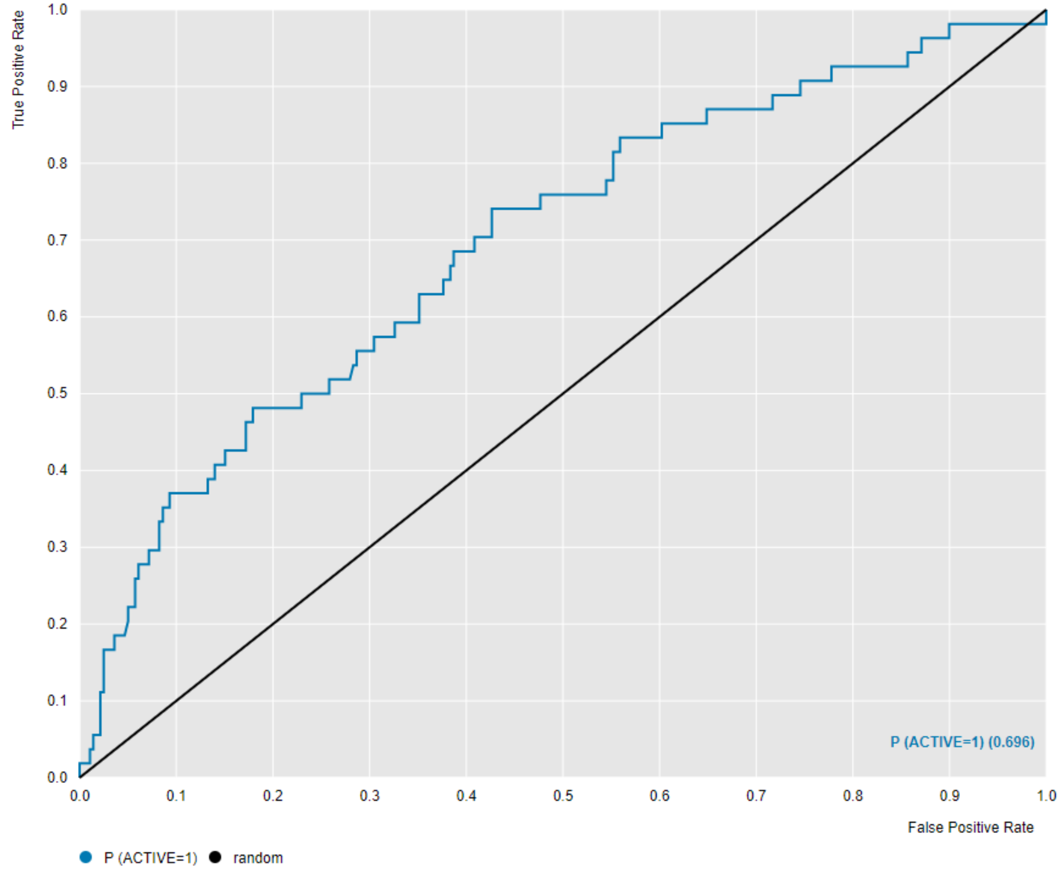

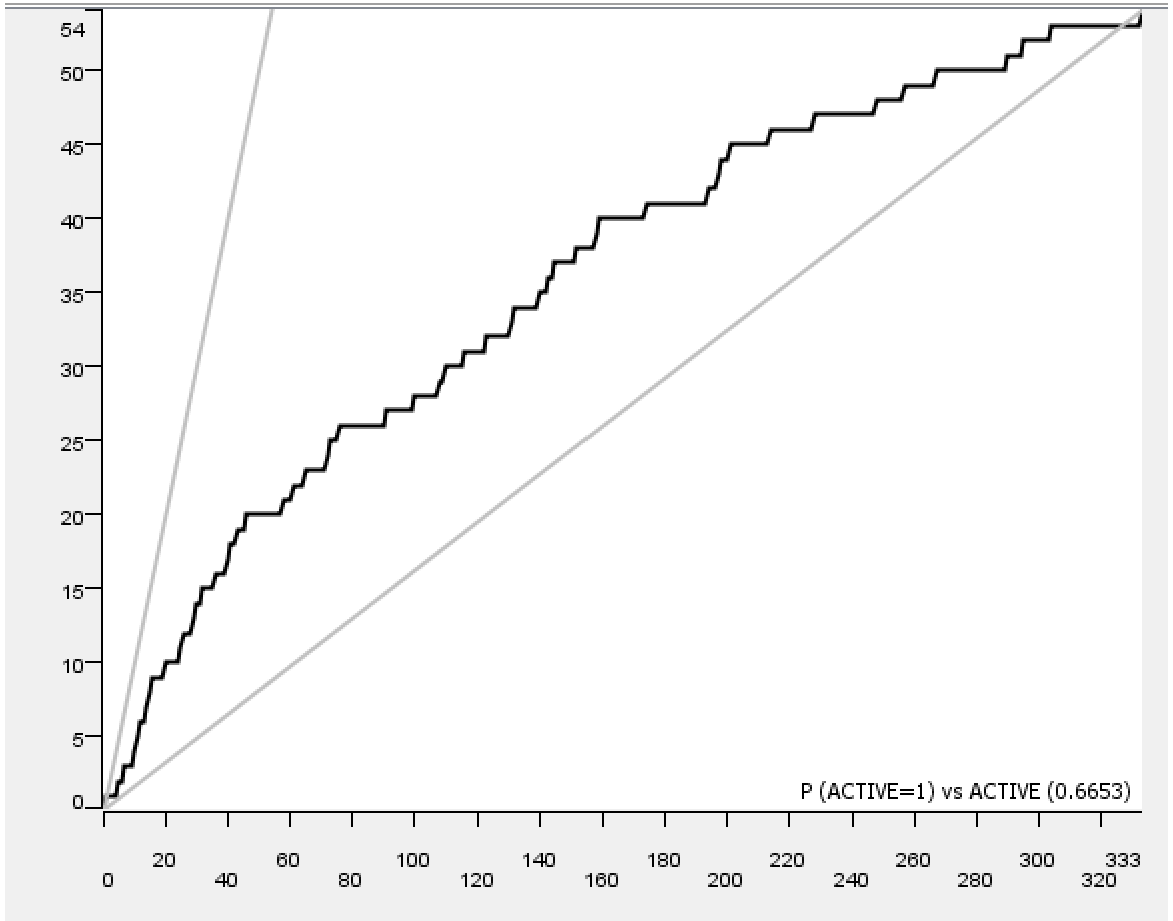

N=333

Top 10% Mean IC50	145 nM (one >800 nM compound, and one >1,000 nM)

	Predicted Active	Predicted Inactive
Active	12	42
Inactive	15	264

PNN - <2.5 nM DefGood in Xa, 15% error; Random seed = 429

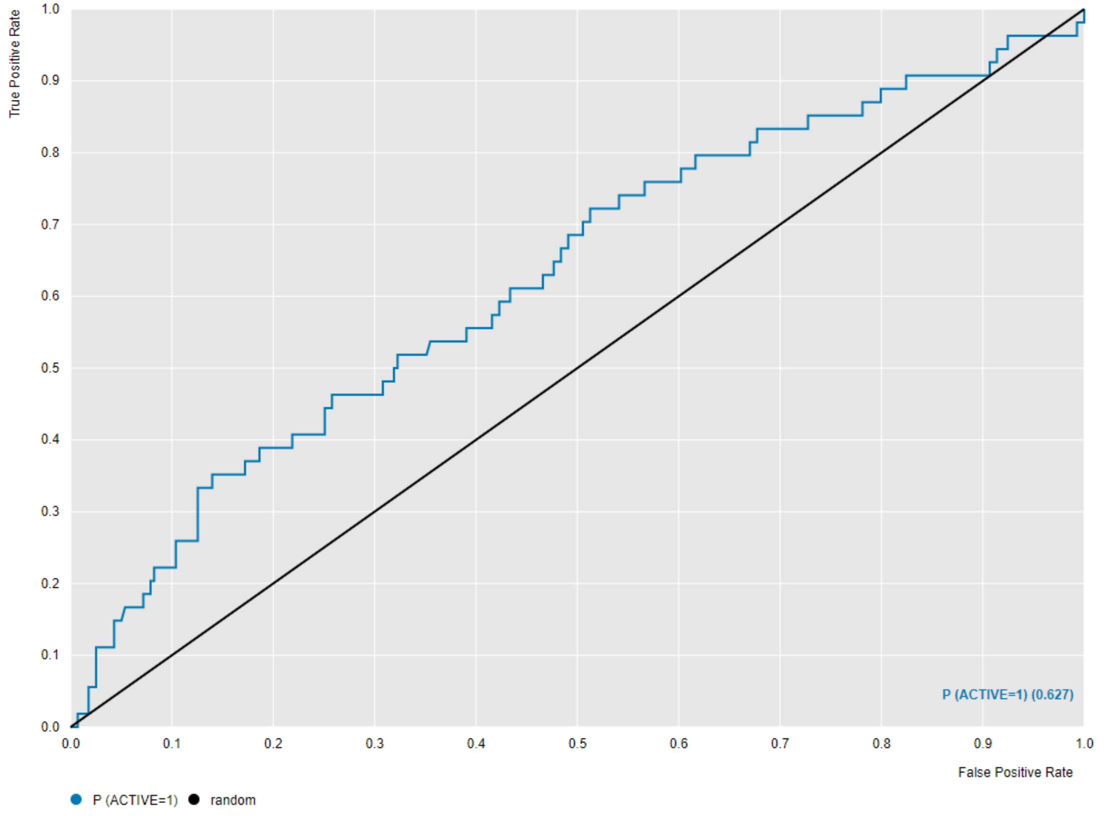

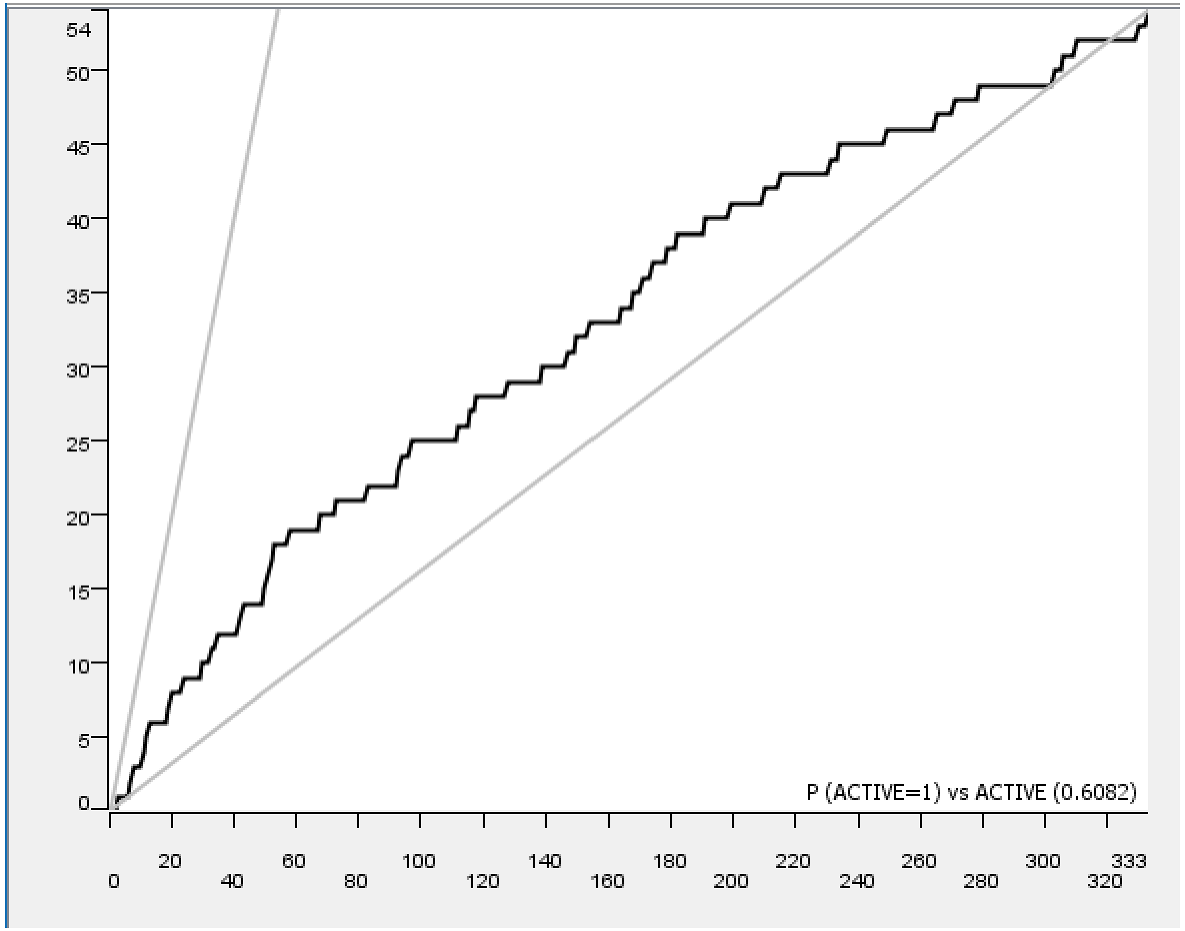

N=333

**Top 10% Mean IC50**	**190 nM (two micromolar mistakes)**

	Predicted Active	Predicted Inactive
Active	10	44
Inactive	22	257

PNN - <2.5 nM DefGood in Xa, 20% error ; Random seed = 429

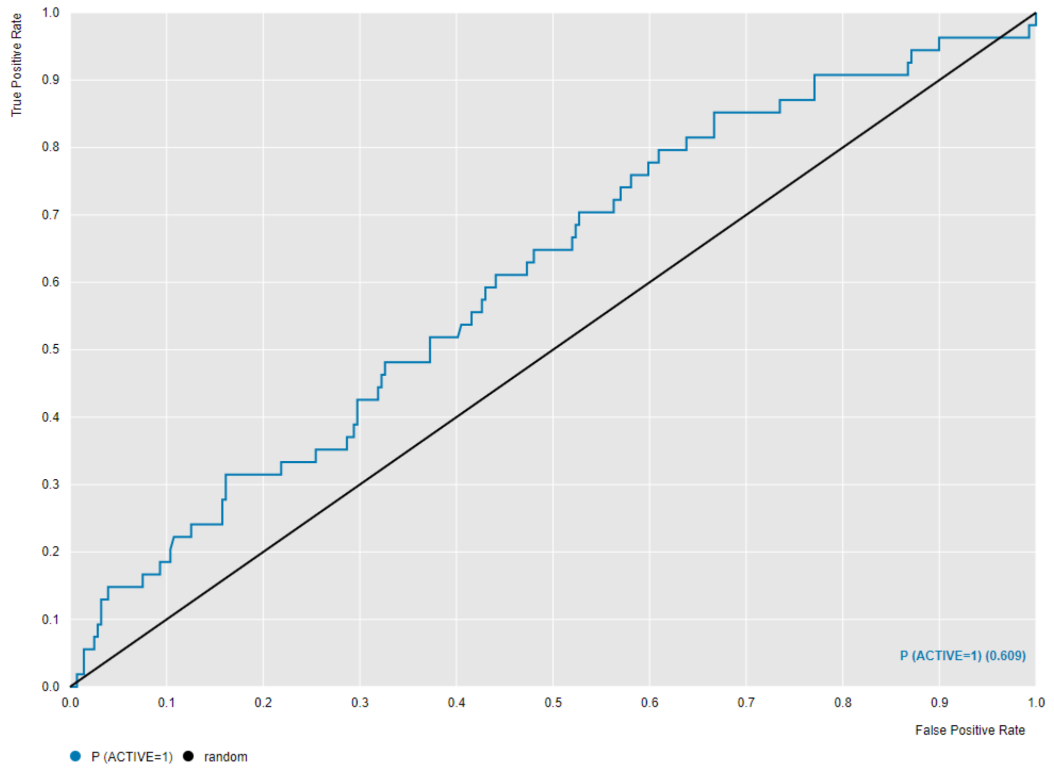

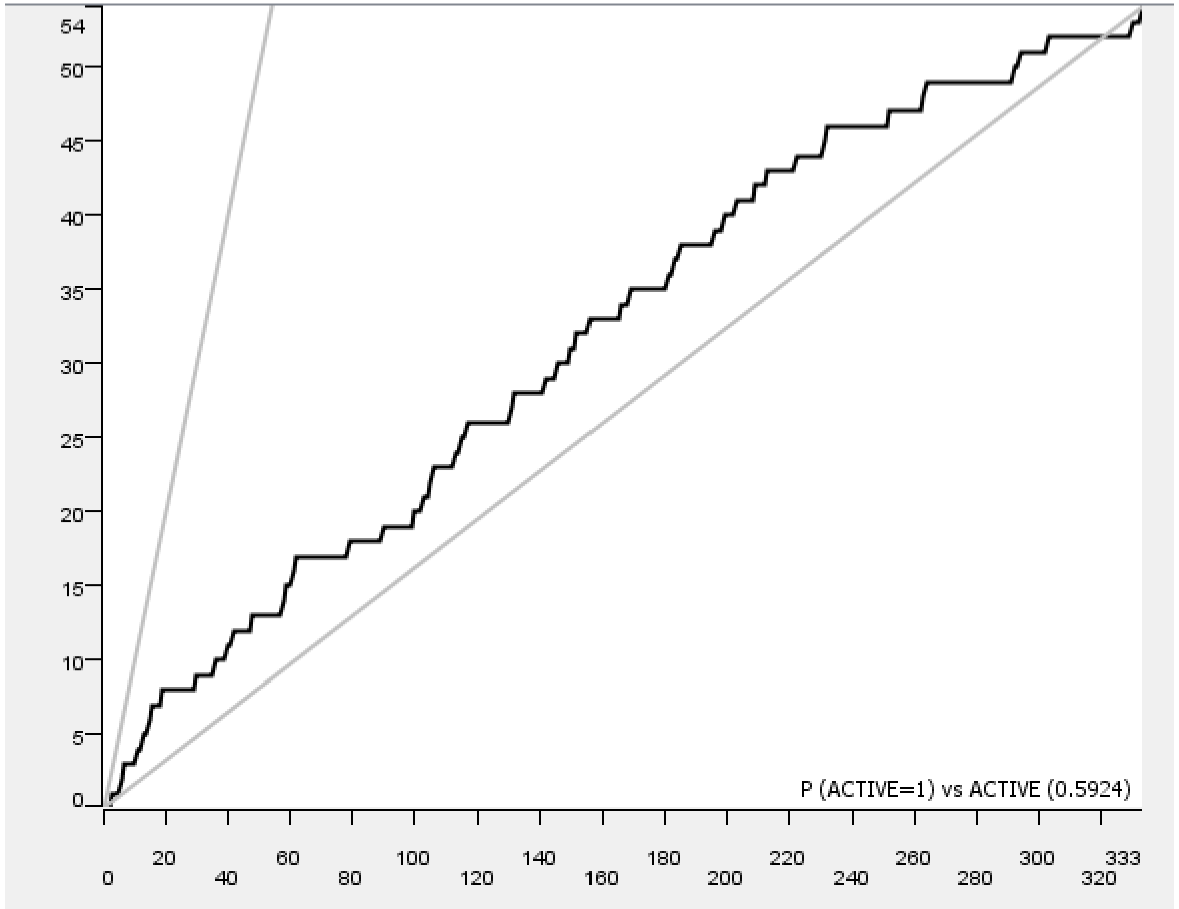

N=333

Top 10% Mean IC50	1,000 nM

	Predicted Active	Predicted Inactive
Active	10	44
Inactive	27	252

PNN - <2.5 nM DefGood in Xa, 5% error; Random seed = 121783

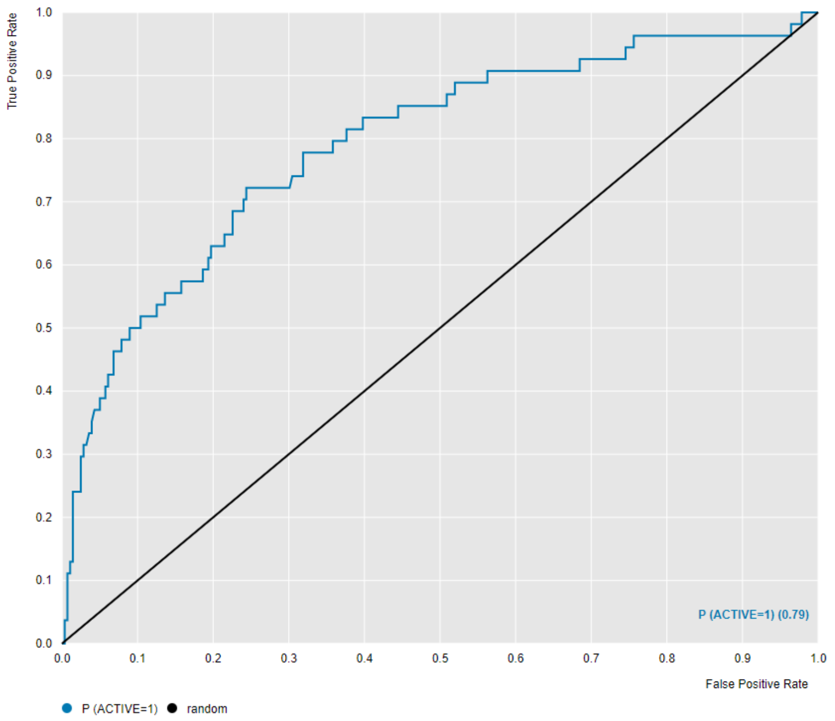

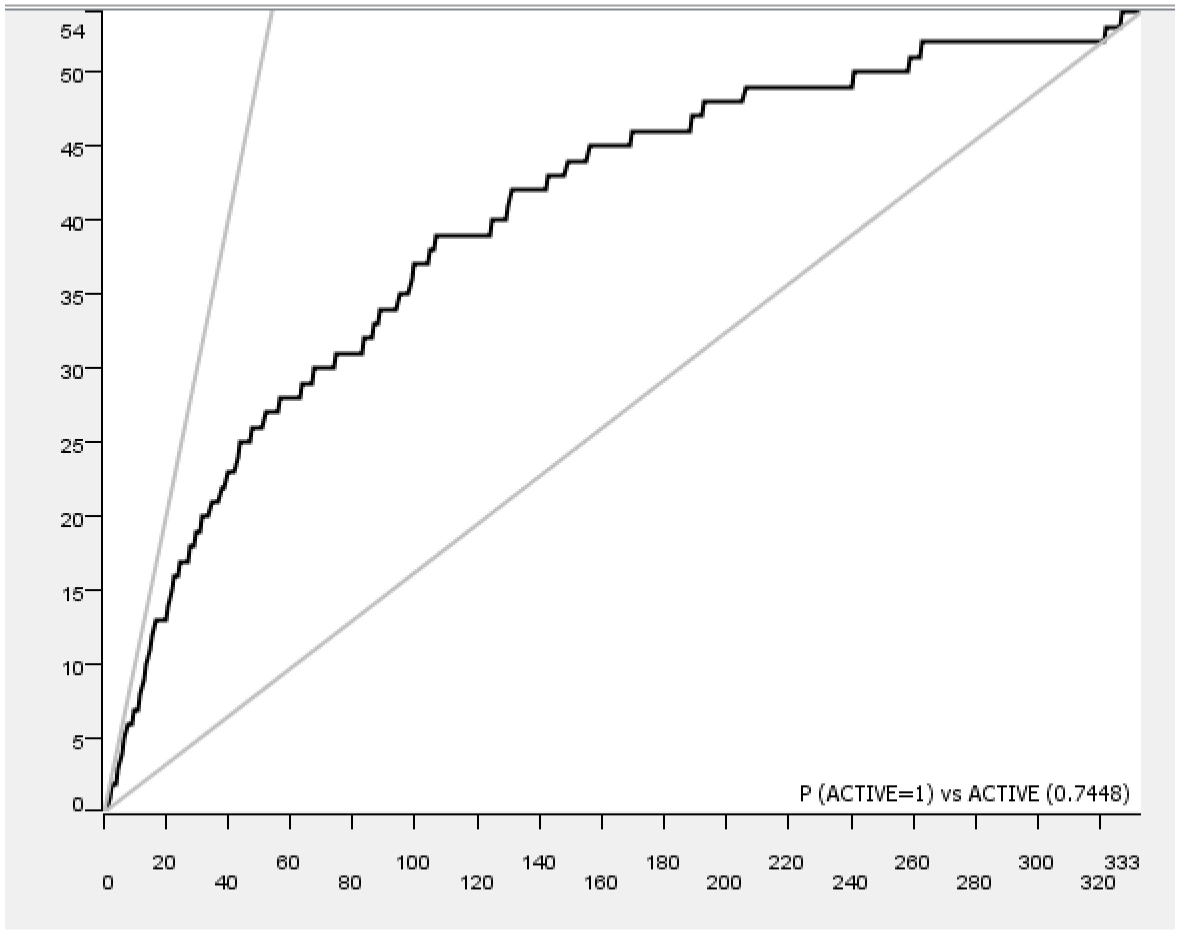

N=333

Top 10% Mean IC50	190 nM (2,500 nM compound is ranked first)

	Predicted Active	Predicted Inactive
Active	17	37
Inactive	8	271

PNN - <2.5 nM DefGood in Xa, 10% error; Random seed = 121783

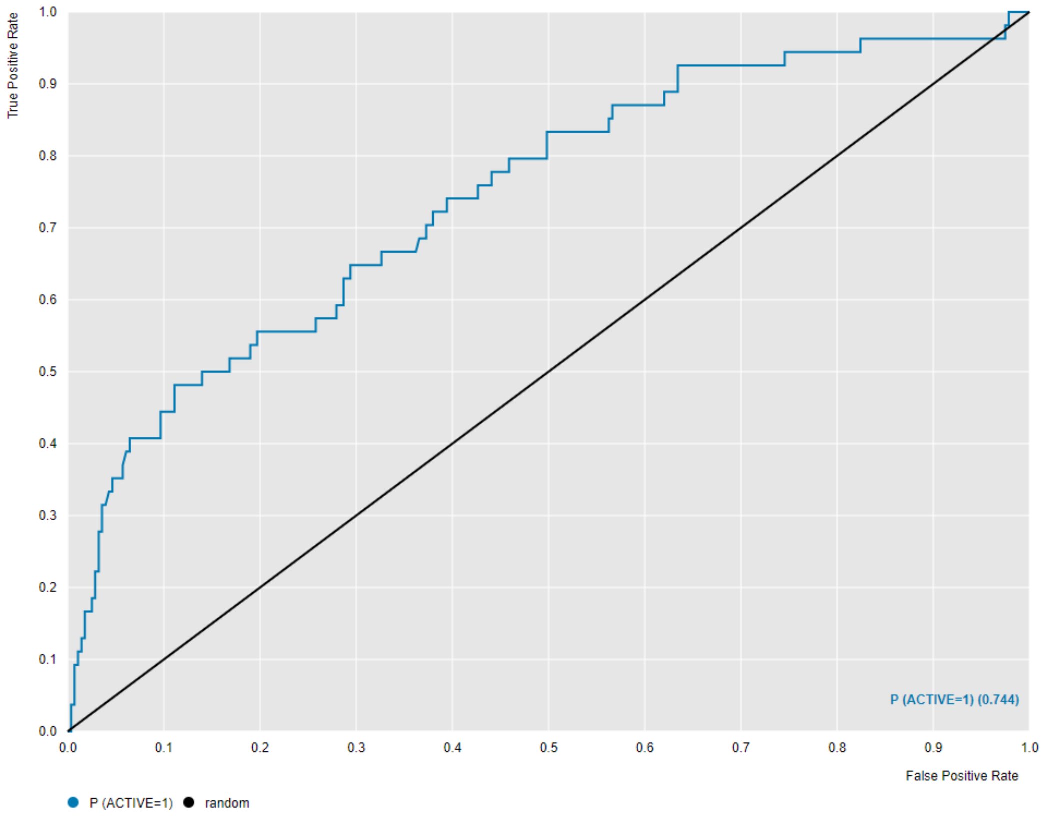

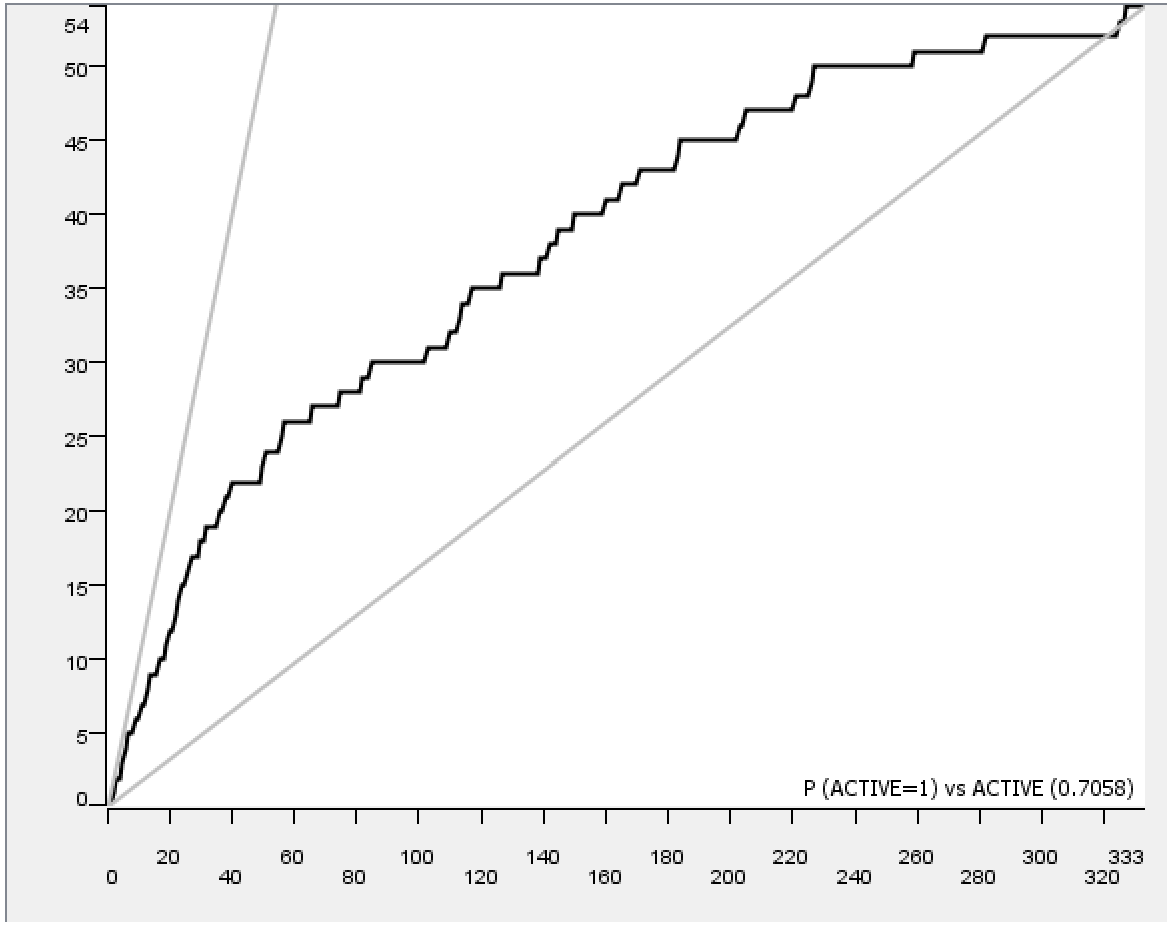

N=333

**Top 10% Mean IC50**	**120 nM (2,500 nM compound is ranked first)**

	Predicted Active	Predicted Inactive
Active	16	38
Inactive	10	269

PNN - <2.5 nM DefGood in Xa, 15% error; Random seed = 121783

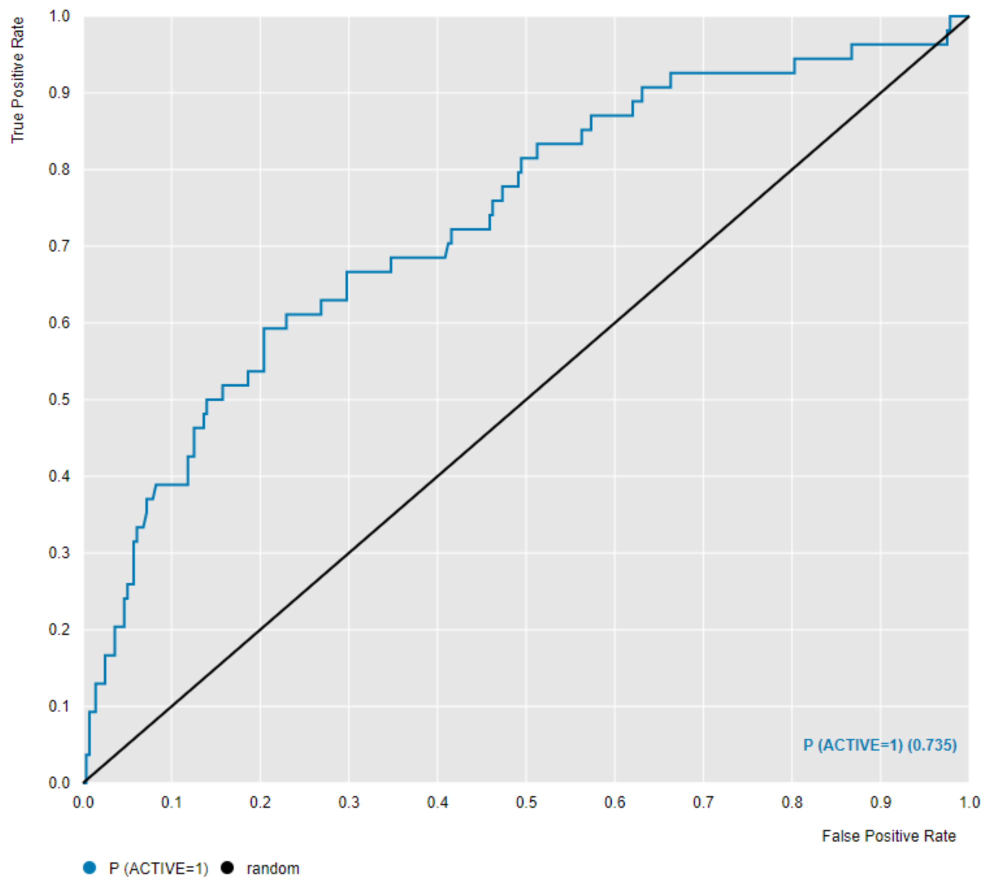

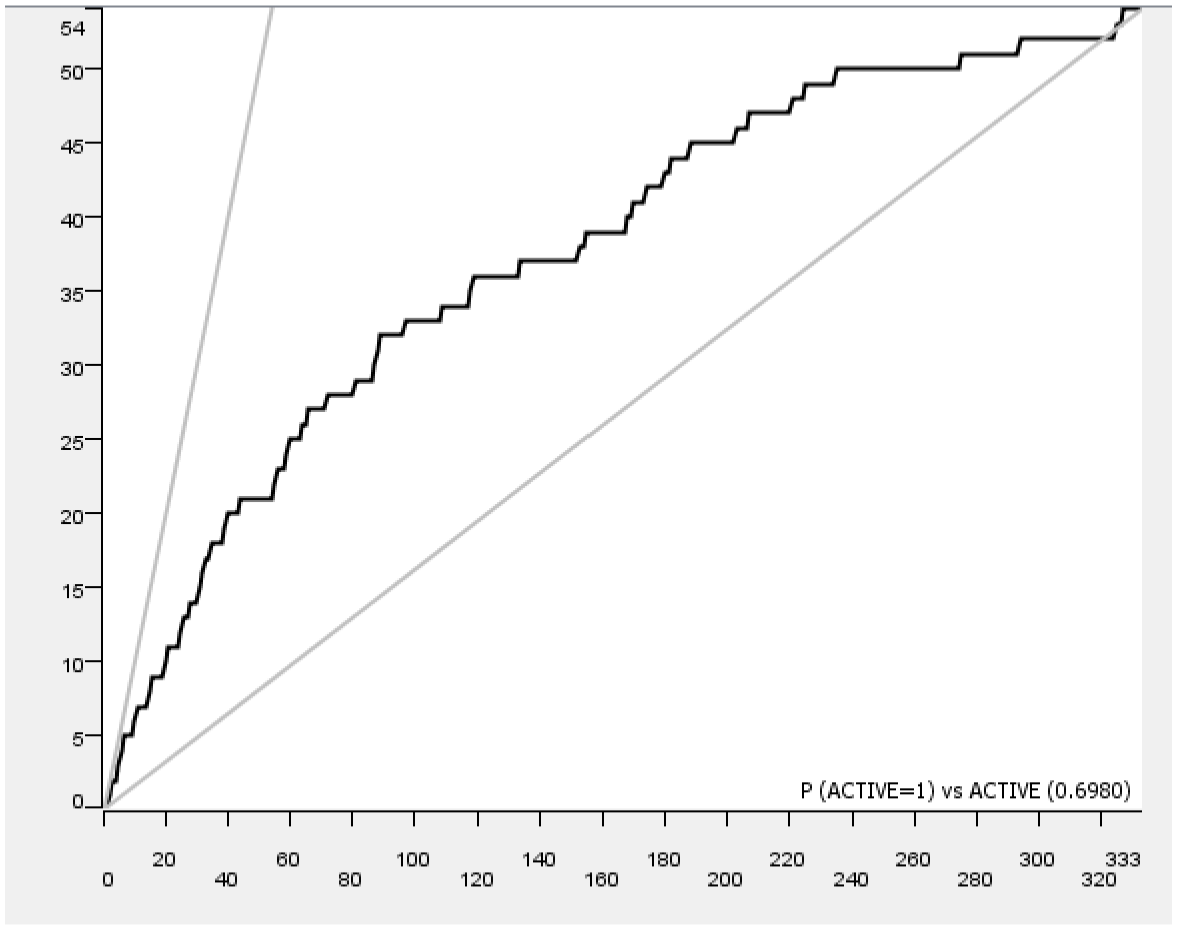

N=333

Top 10% Mean IC50	120 nM (2,500 nM compound is ranked first)

	Predicted Active	Predicted Inactive
Active	18	36
Inactive	18	261

PNN - <2.5 nM DefGood in Xa, 20% error; Random seed = 121783

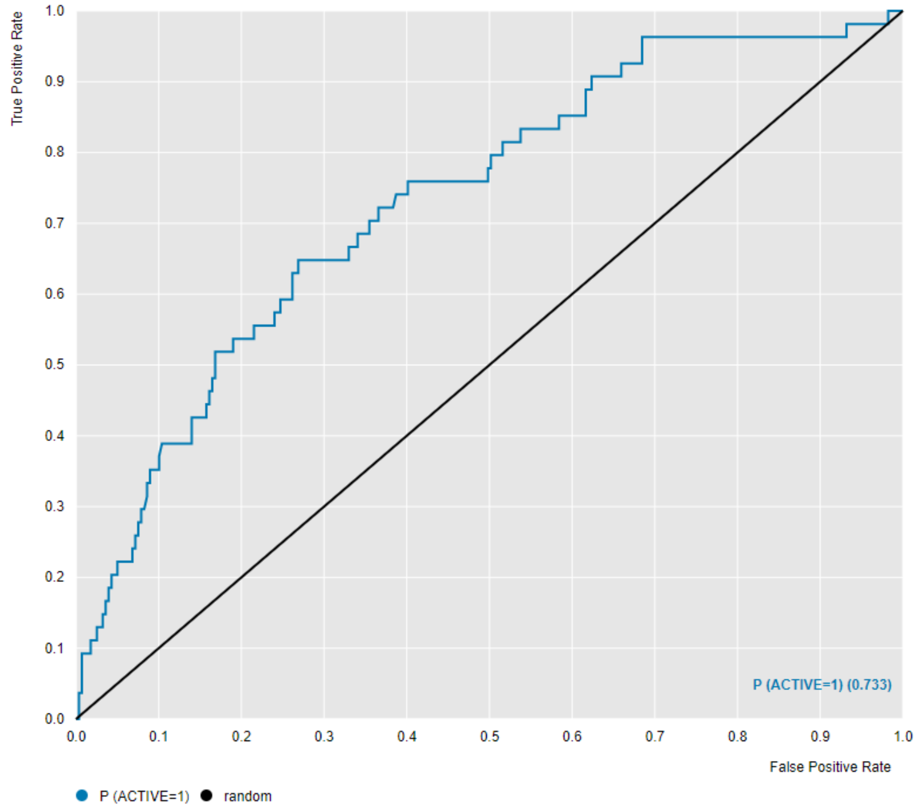

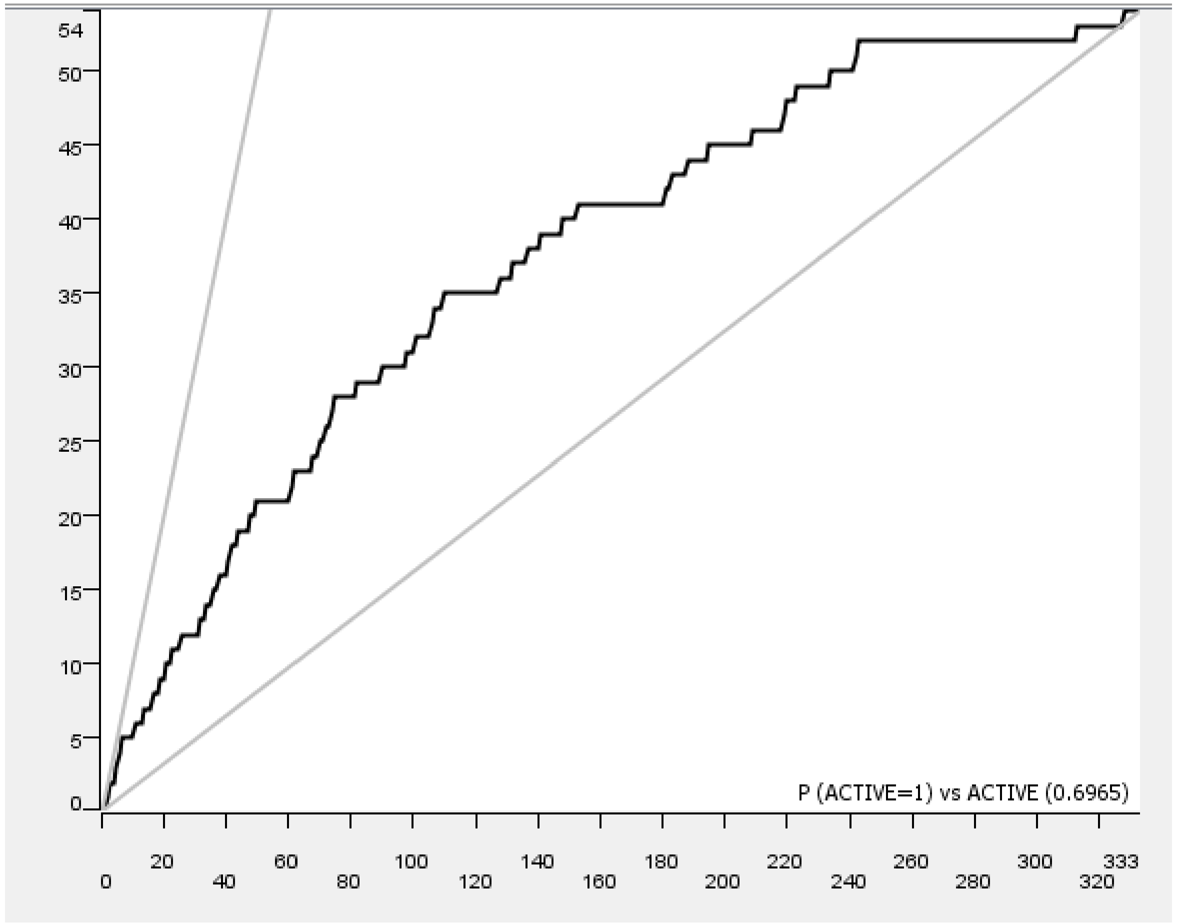

N=333

Top 10% Mean IC50	210 nM (2,500 nM compound is ranked first)

	Predicted Active	Predicted Inactive
Active	19	35
Inactive	26	253

PNN - <2.5 nM DefGood in Xa, 30% error; Random seed = 121783

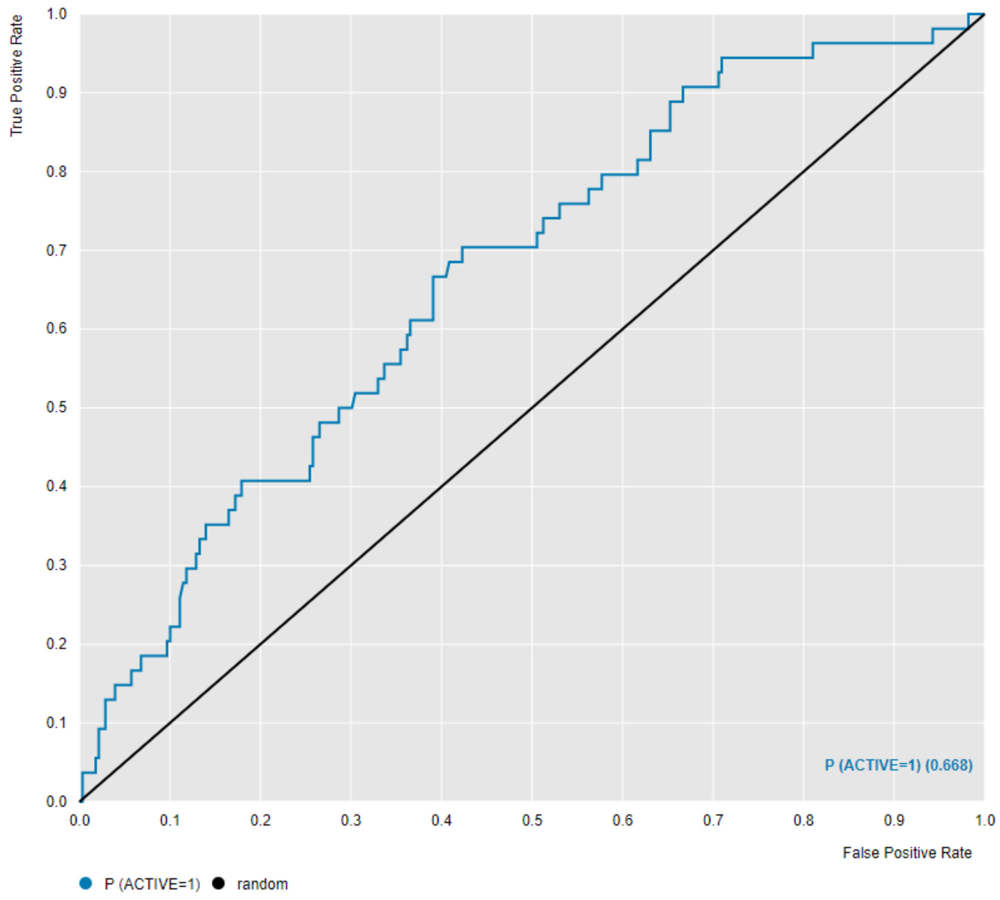

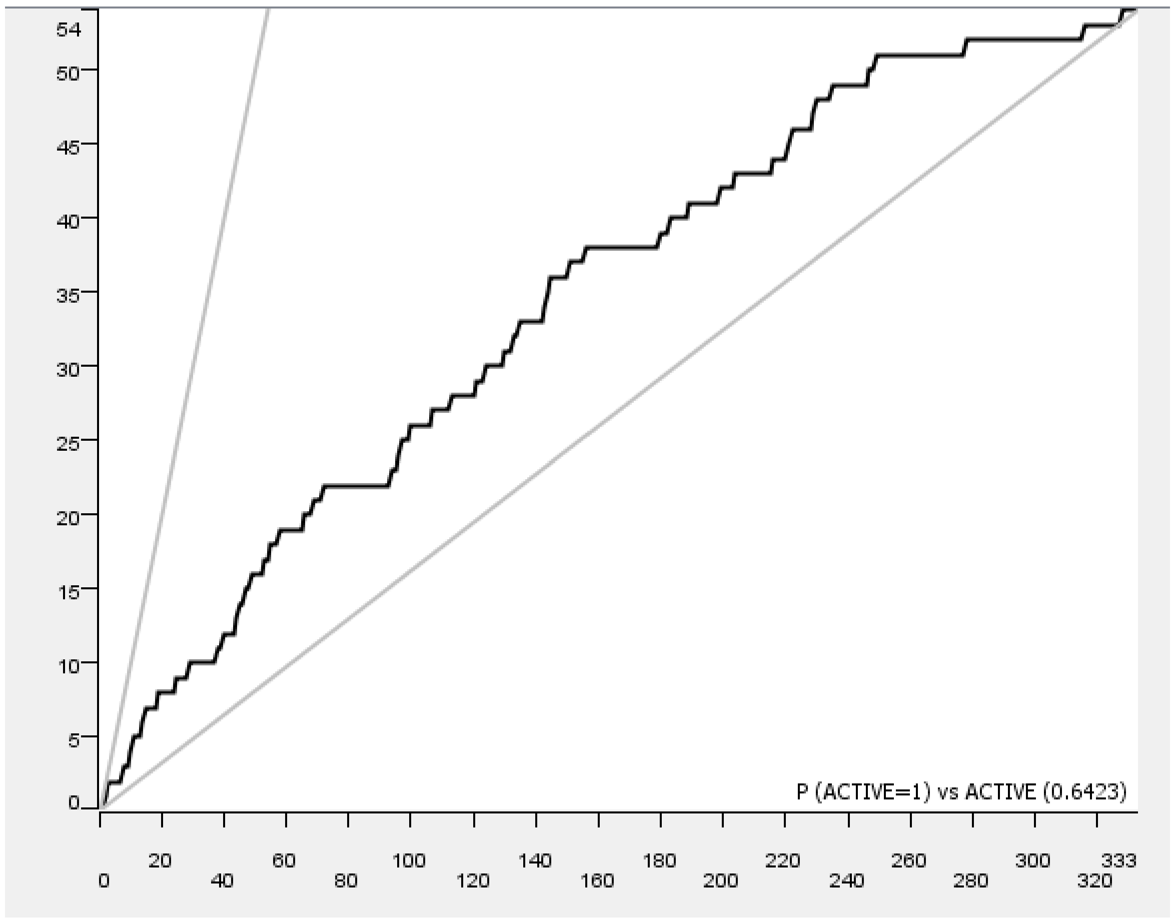

N=333

Top 10% Mean IC50	290 nM (2,500 nM compound is ranked first)

	Predicted Active	Predicted Inactive
Active	21	33
Inactive	49	230

PNN - <2.5 nM DefGood in Xa, 40% error; Random seed = 121783

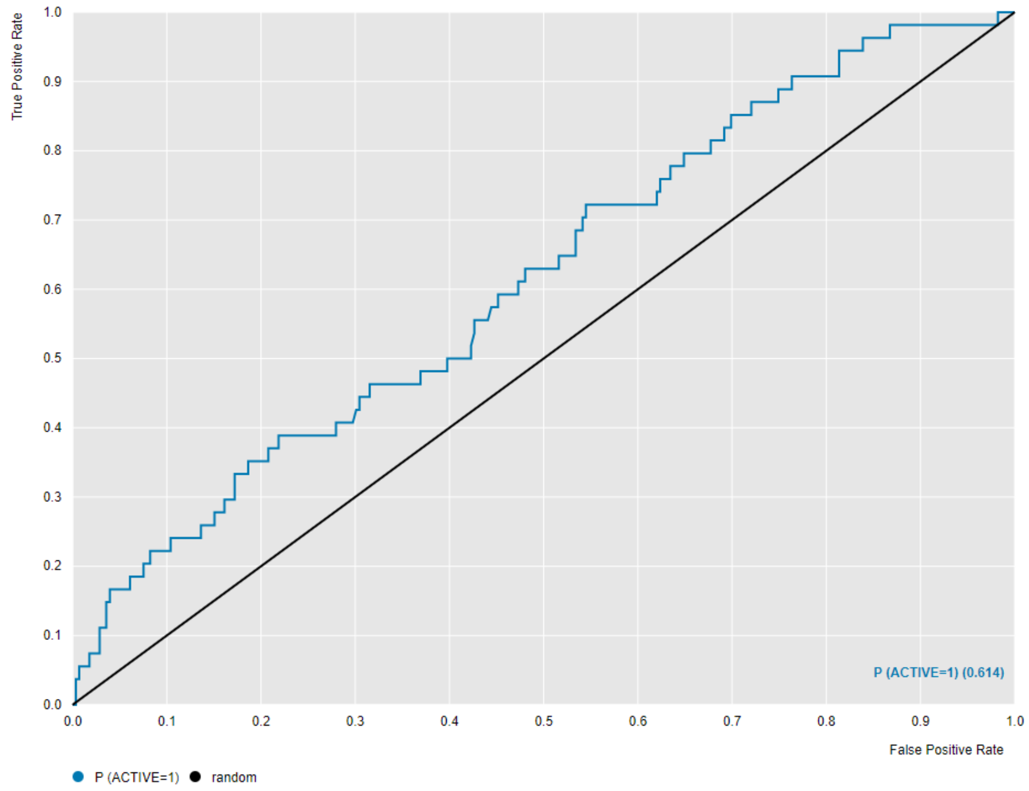

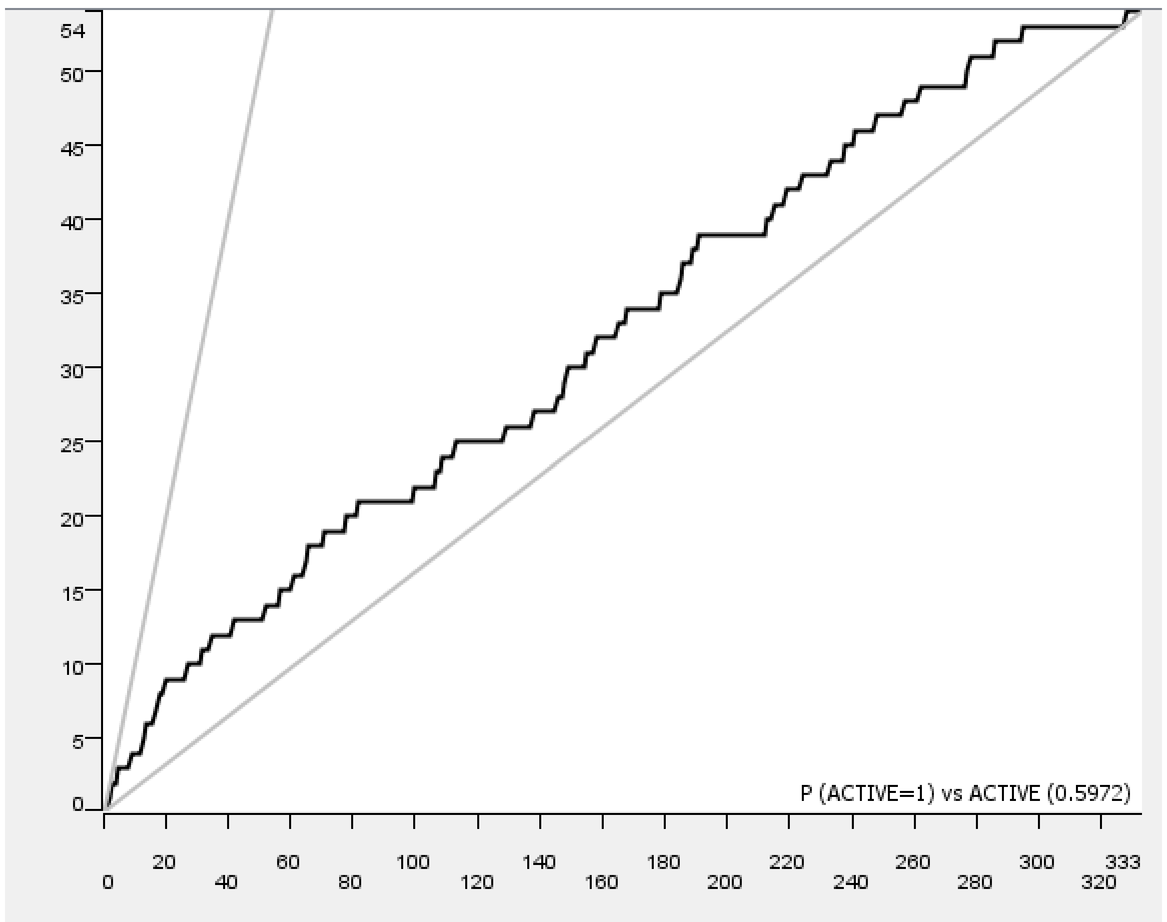

N=333

Top 10% Mean IC50	200 nM (2,500 nM compound is ranked first)

	Predicted Active	Predicted Inactive
Active	22	32
Inactive	82	197

PNN - <2.5 nM DefGood in Xa, 45% error; Random seed = 121783

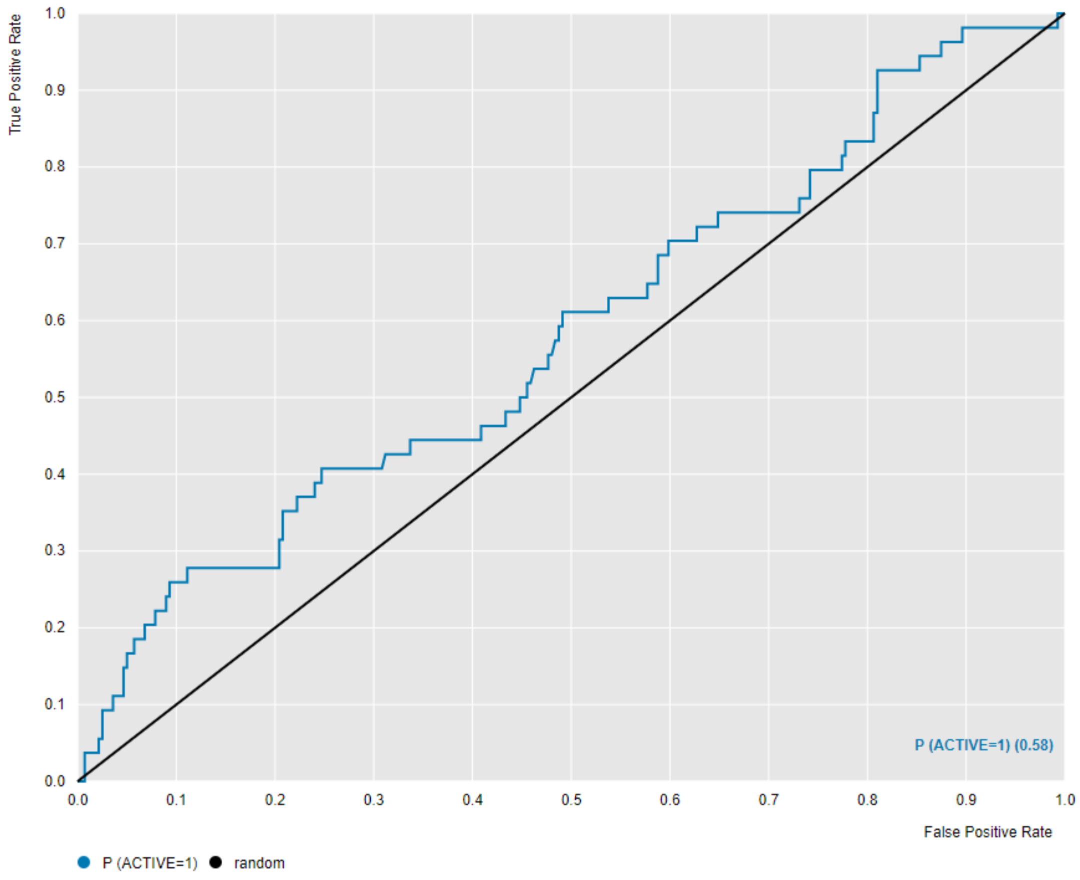

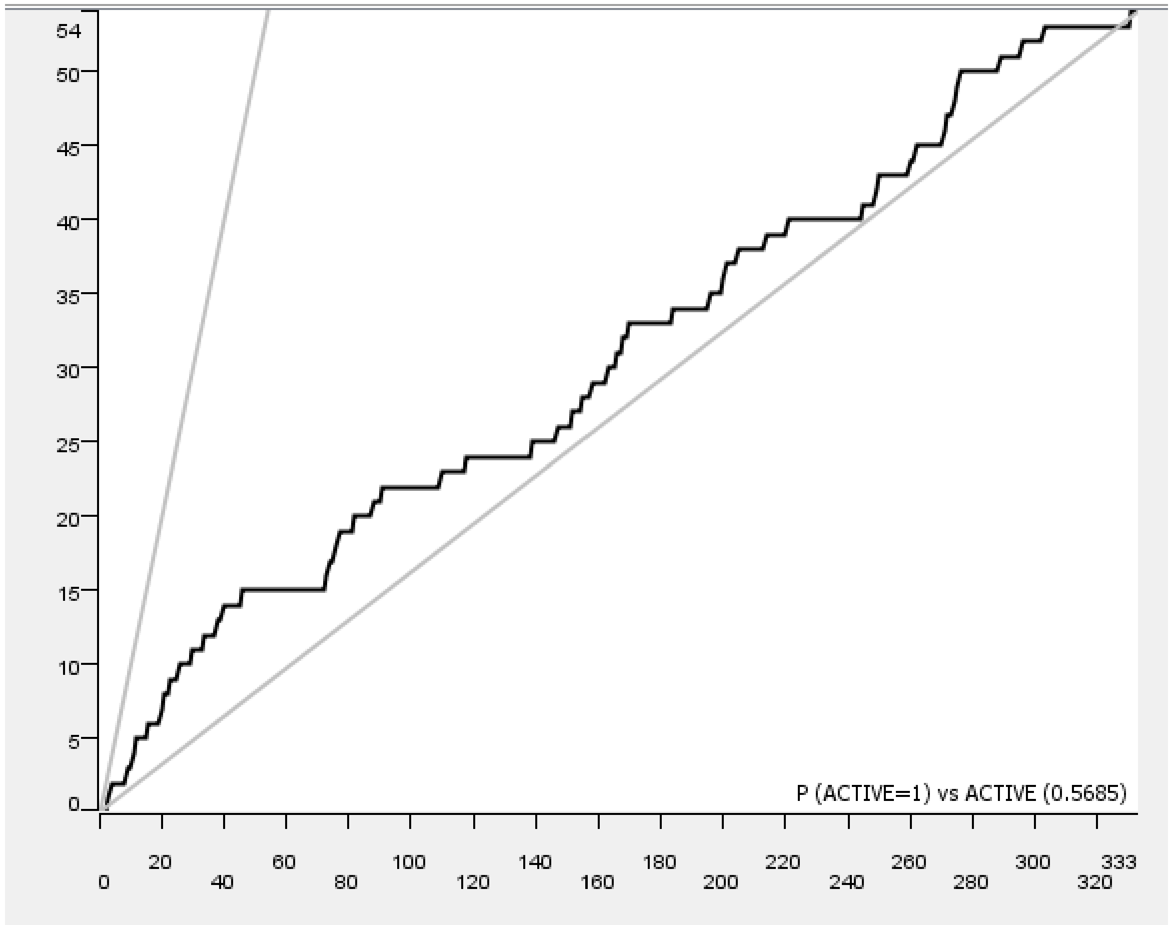

N=333

Top 10% Mean IC50	1,200 nM

	Predicted Active	Predicted Inactive
Active	22	32
Inactive	85	194

Conclusion - PNN

- Across three random seeds, the % error that lead to a significant enrichment failure and/or top 10% IC50 increase in the retrospective test was 20%, 10% and 30% for each unique random seed.

HIV Protease

HIV Protease NBN Design

- Rules - $RELATION$ = "=" OR $IC50$ > 999 OR $IC50$ < 10 => TRUE
- Sort ascending and remove duplicates
- $DESCRIPTION$ MATCHES ".*strain.*" OR $DESCRIPTION$ MATCHES ".*V18.*" OR $DESCRIPTION$ MATCHES ".*NL4-3.*" OR $DESCRIPTION$ MATCHES ".*mutant.*“ was removed
- DefGood scanned between 2.5 and 25 nM
- 80-20 split for both actives and inactives
- NBN – ECFP4 used Classifier where Prediction was Active = True
- Resulted in 2544 compounds after cleaning the data

<2.5 nM DefGood in HIV Protease

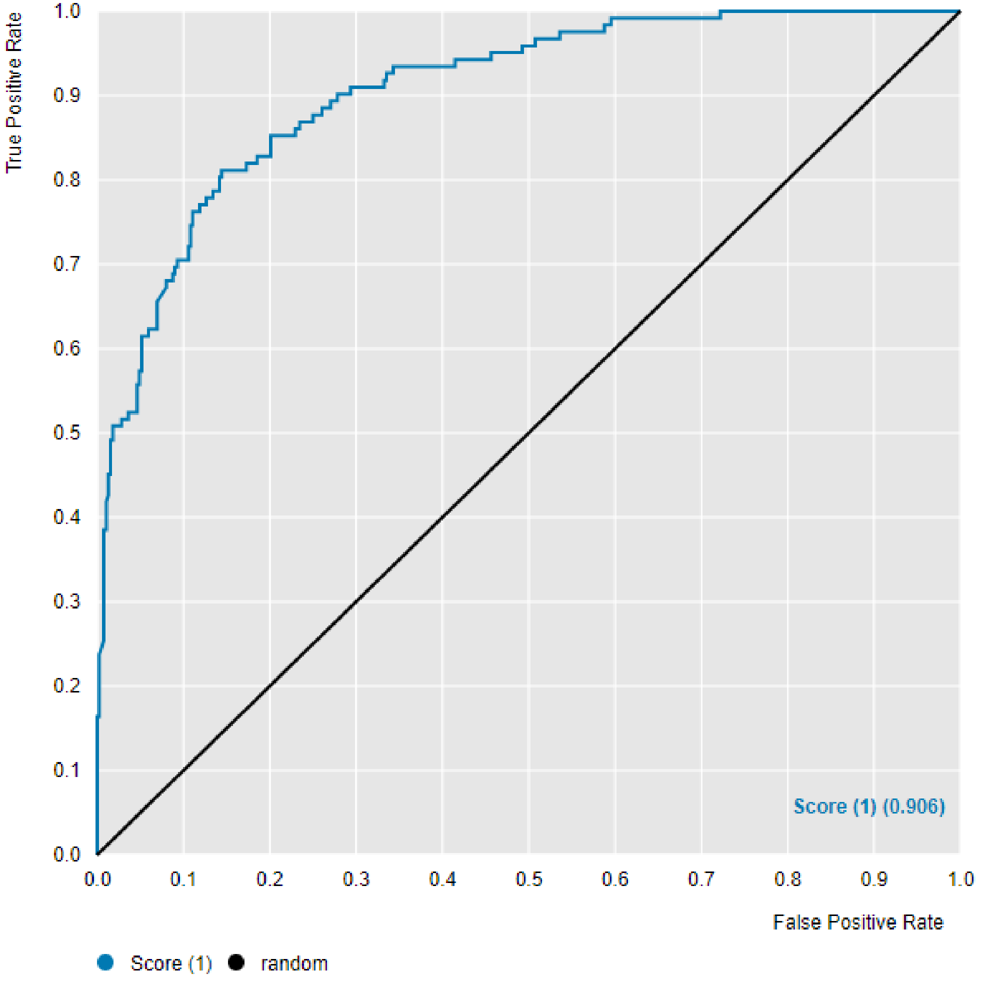

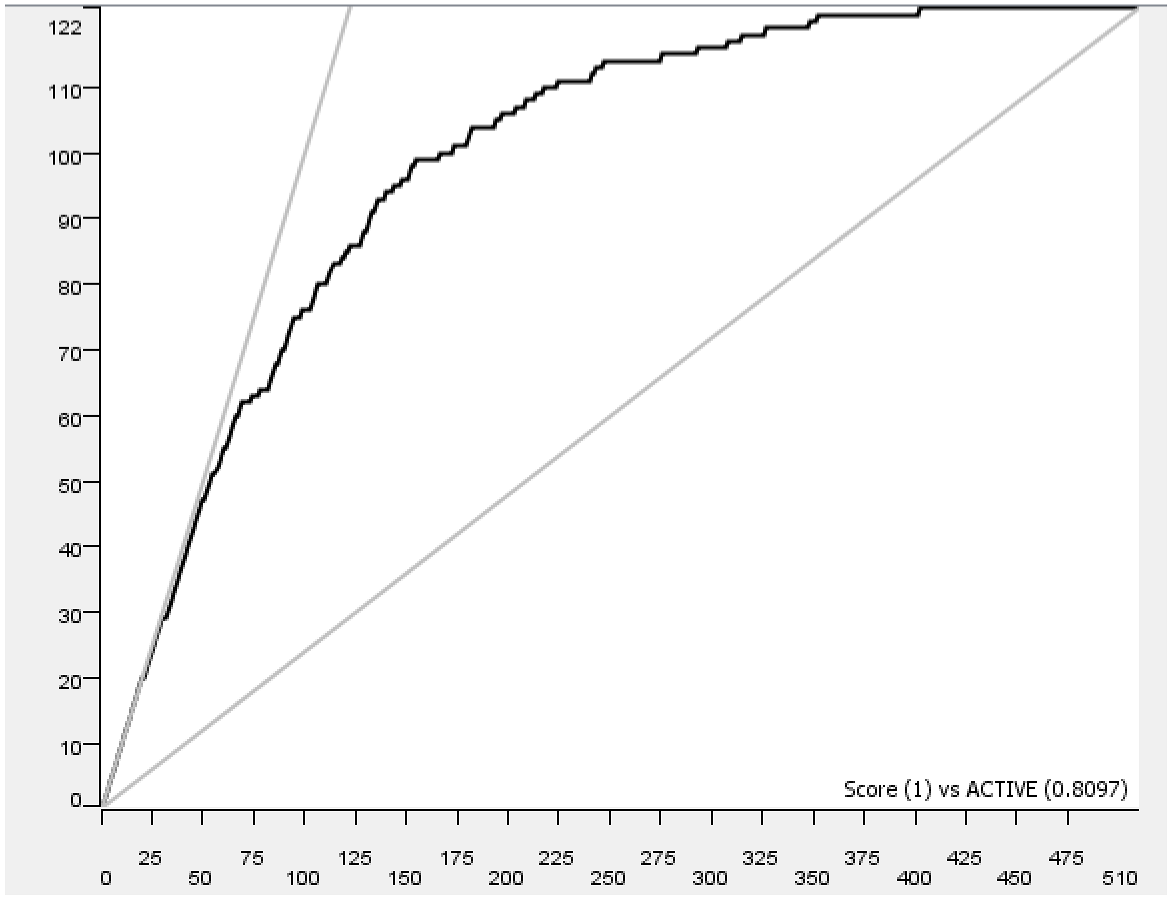

N=510

Top 10% Mean IC50	8.31 nM

	Predicted Active	Predicted Inactive
Active	111	11
Inactive	114	274

<5 nM DefGood in HIV Protease

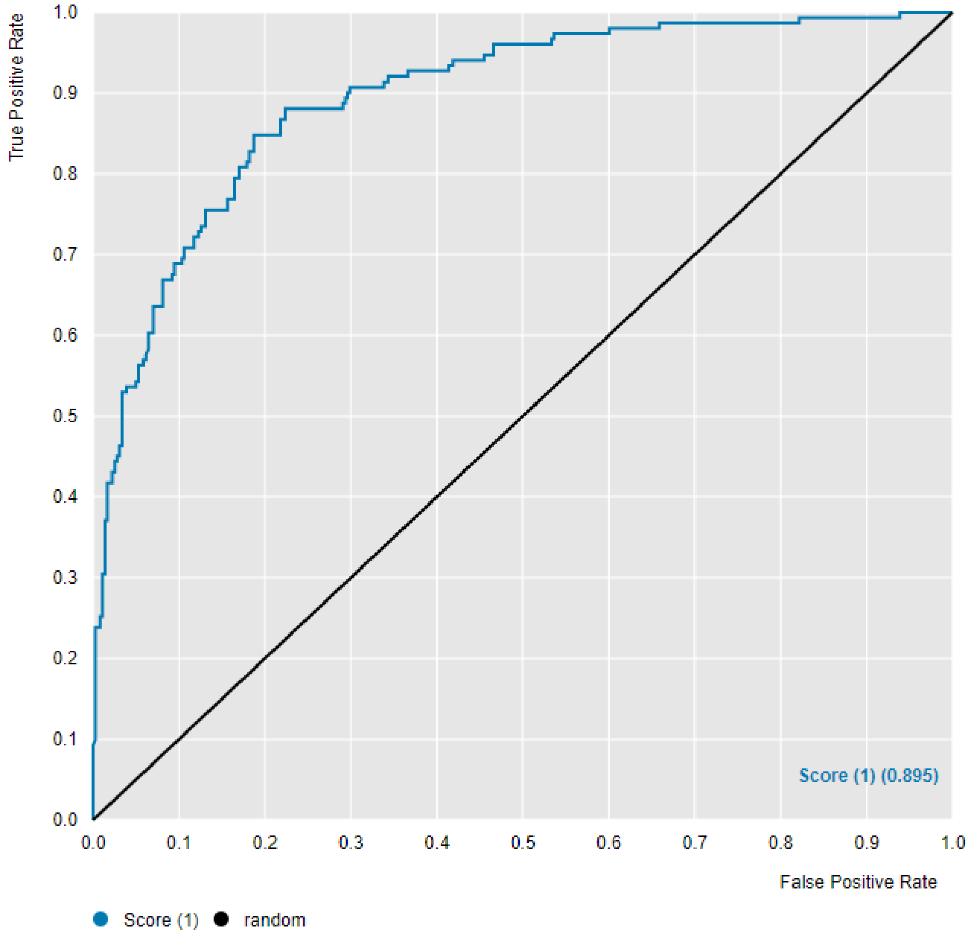

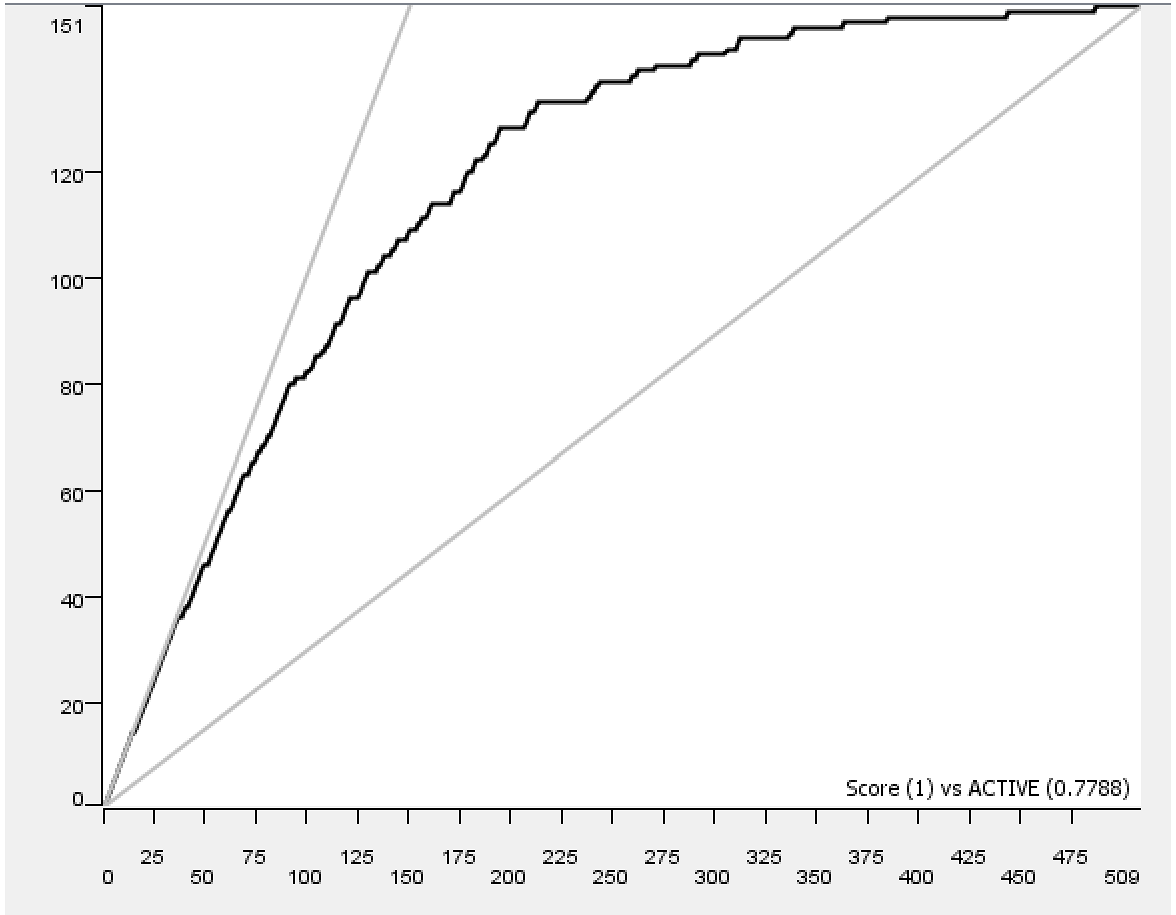

N=509

Top 10% Mean IC50	15.0 nM

	Predicted Active	Predicted Inactive
Active	123	28
Inactive	65	293

<10 nM DefGood in HIV Protease

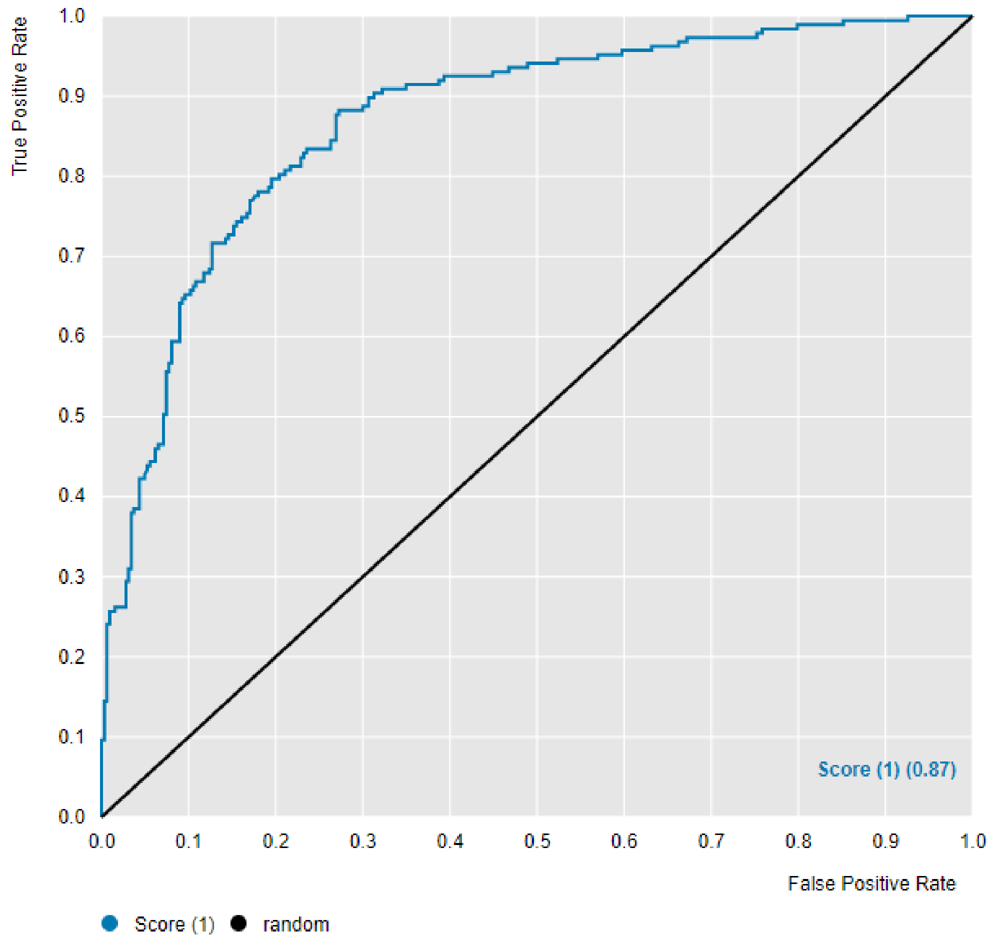

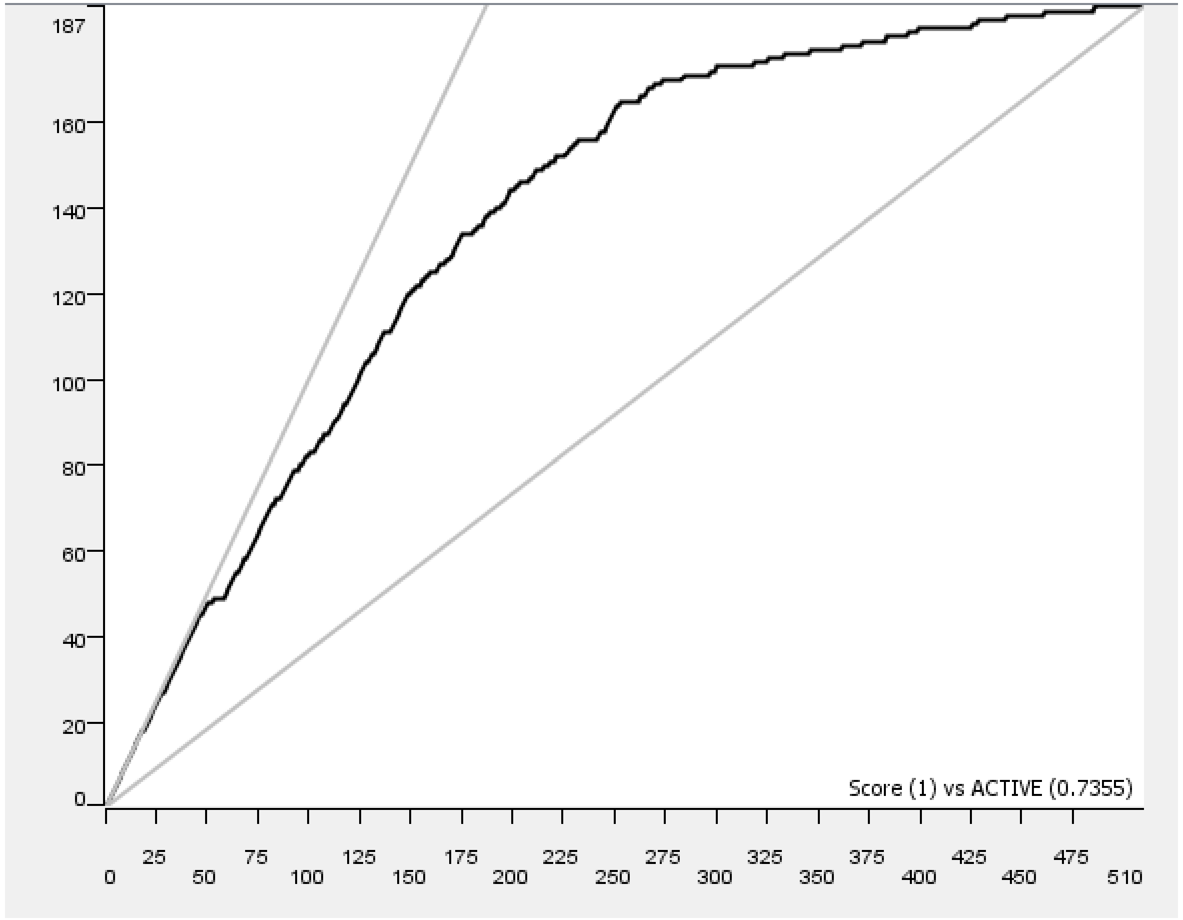

N=510

Top 10% Mean IC50	2.9 nM

	Predicted Active	Predicted Inactive
Active	166	21
Inactive	98	255

<15 nM DefGood in HIV Protease

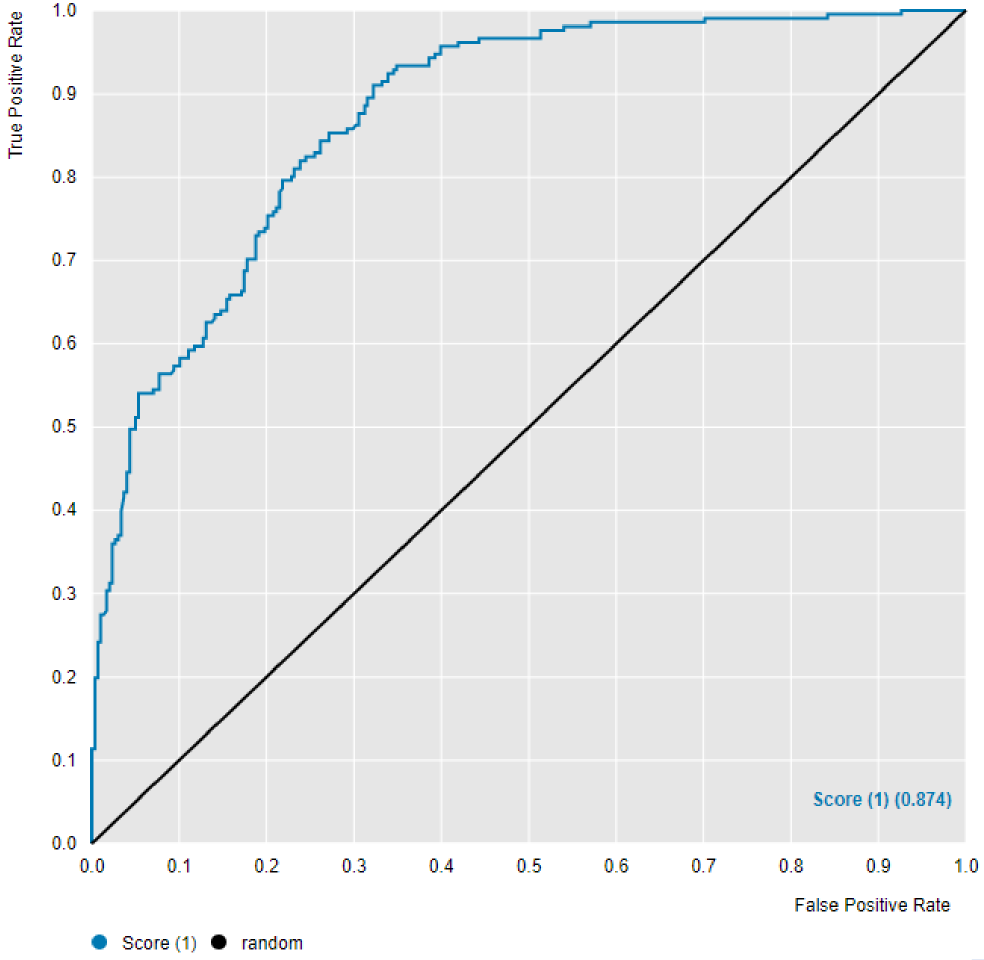

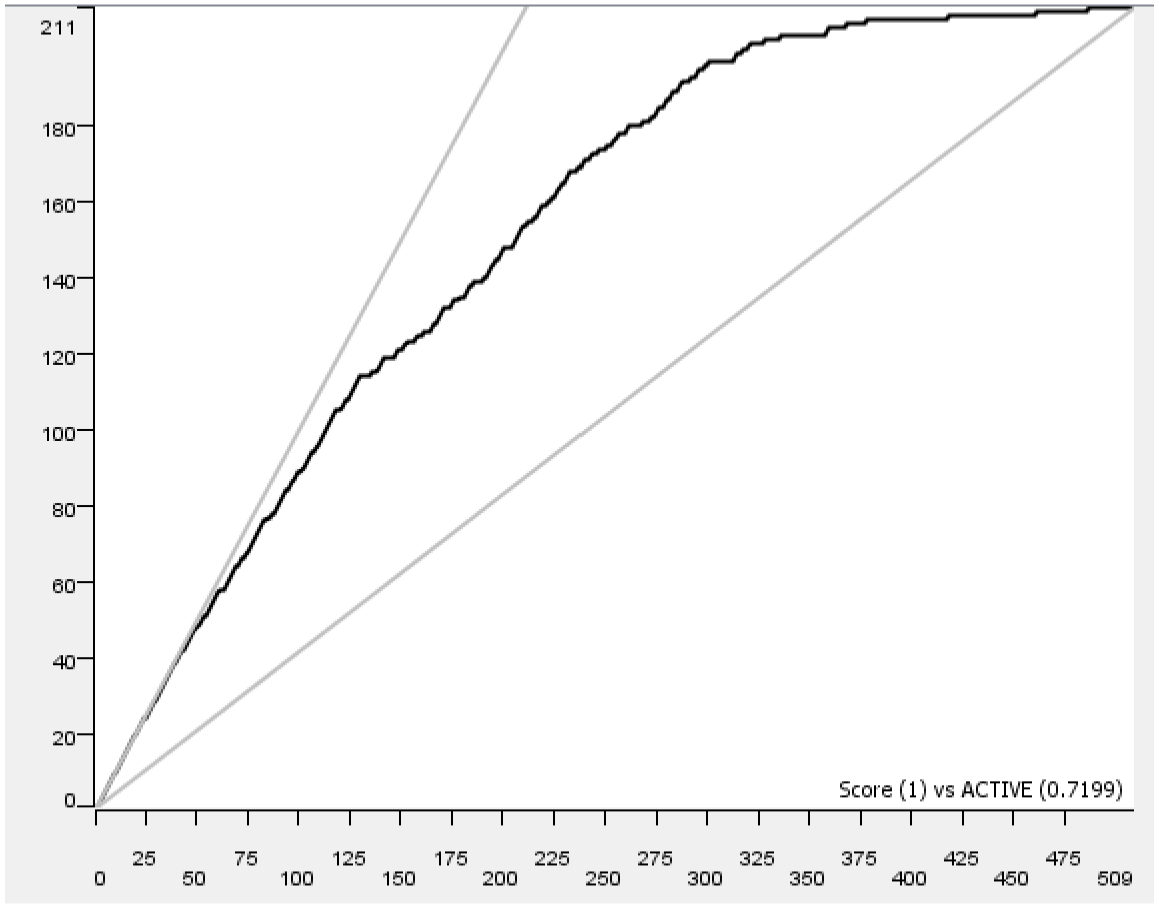

N=509

Top 10% Mean IC50	1.66 nM

	Predicted Active	Predicted Inactive
Active	187	24
Inactive	93	205

<20 nM DefGood in HIV Protease

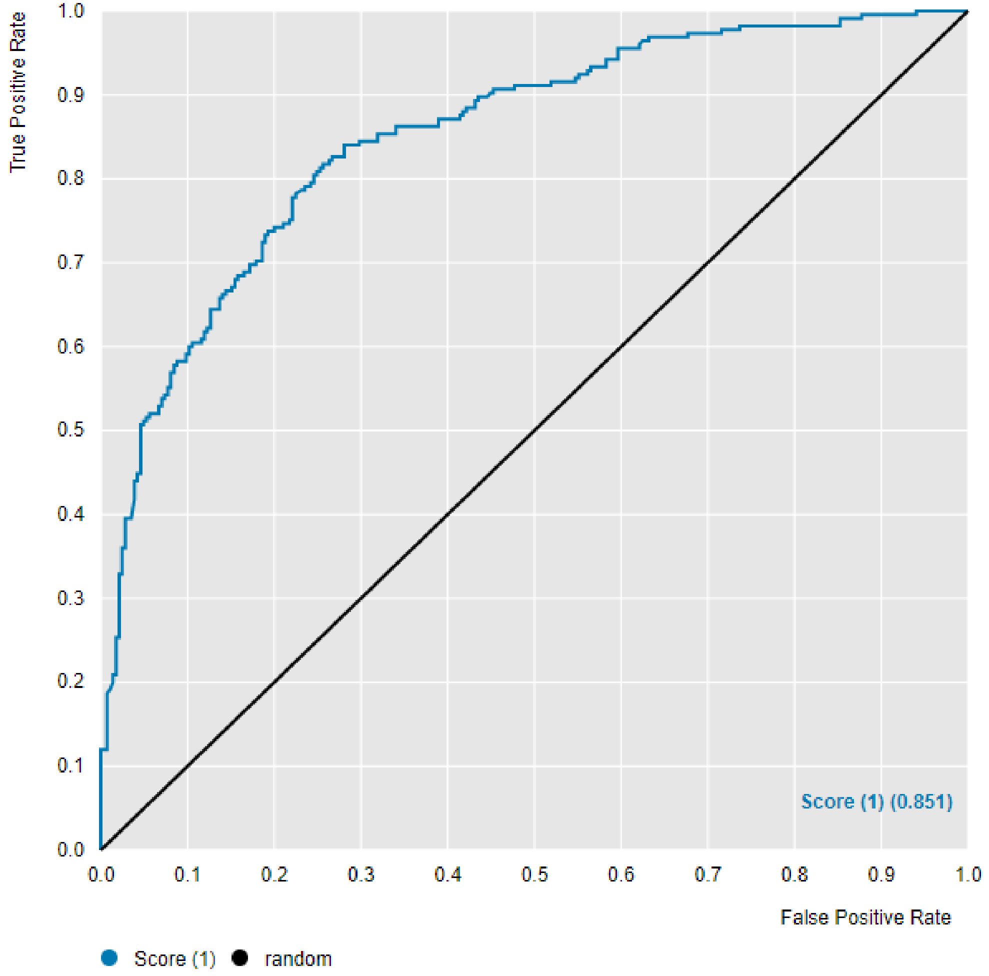

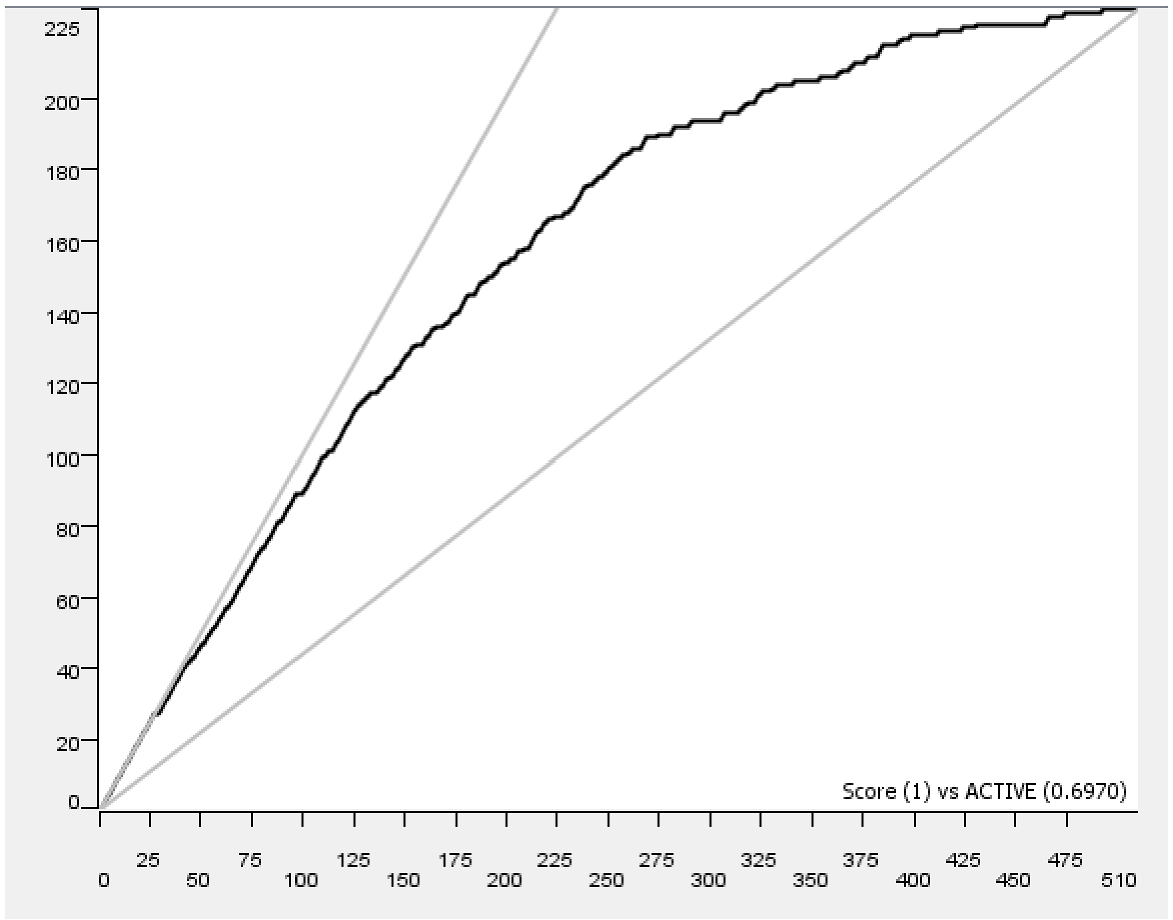

N=510

**Top 10% Mean IC50**	**15.0 nM**

	**Predicted Active**	**Predicted Inactive**
Active	181	44
Inactive	70	215

<25 nM DefGood in HIV Protease

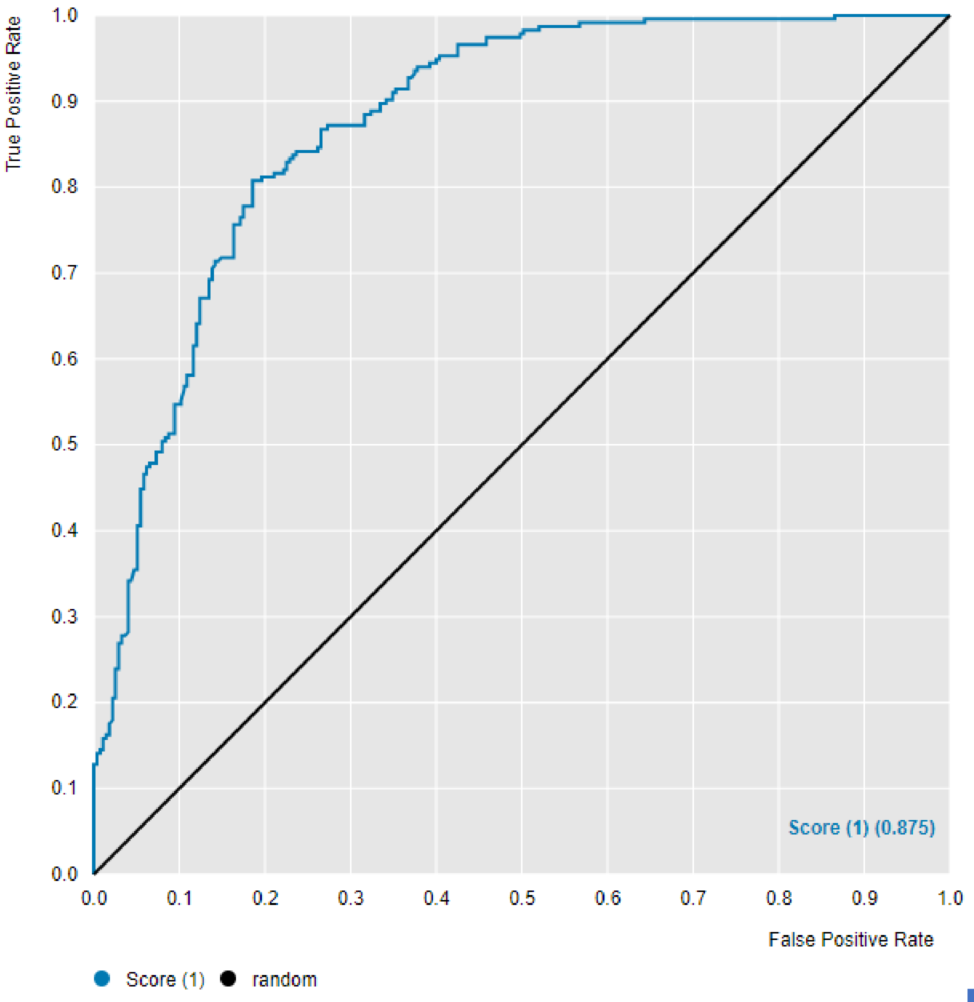

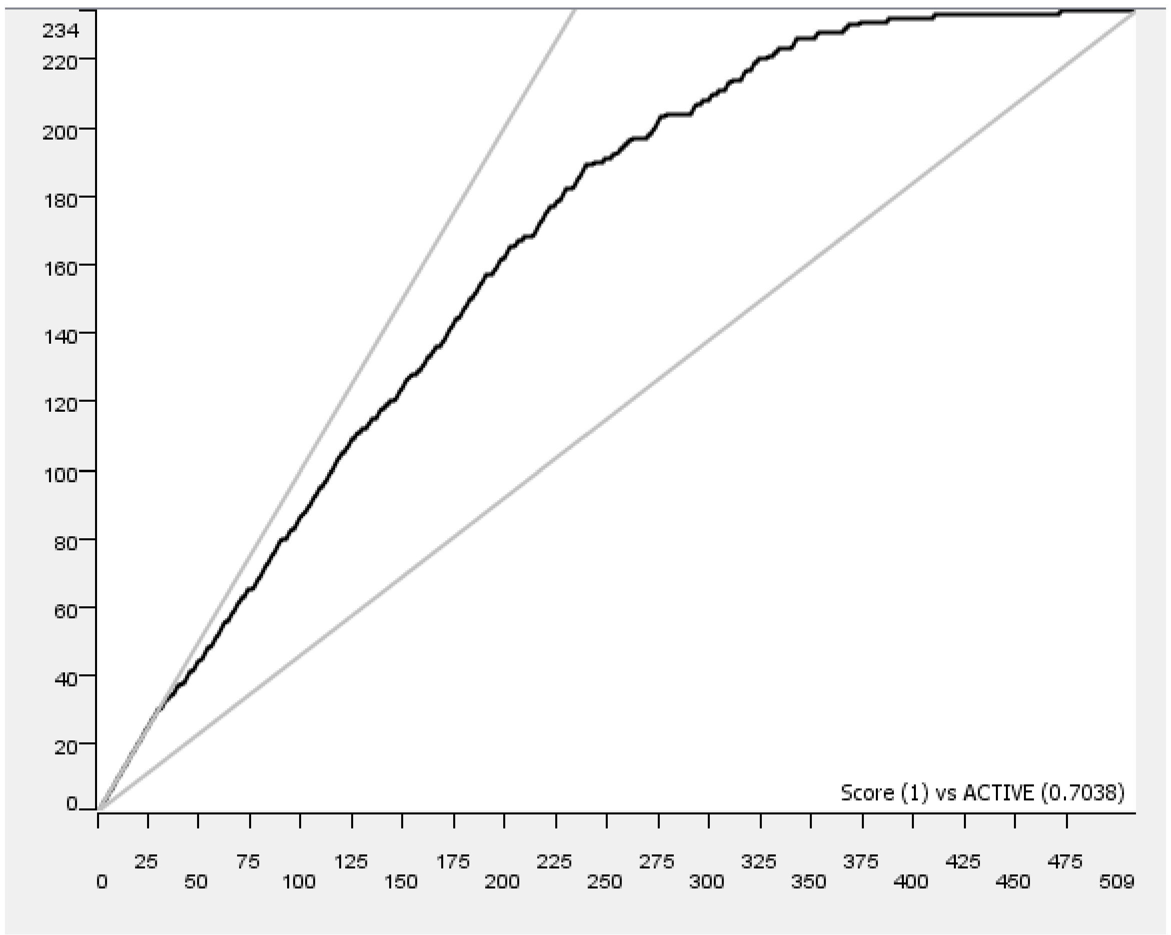

N=509

Top 10% Mean IC50	11.8 nM

	Predicted Active	Predicted Inactive
Active	204	30
Inactive	75	200

Decision on DefGood

- <15 nM has the best performance with regard to enrichment and mean top 10% IC50.

RF - <15 nM DefGood in HIV Protease

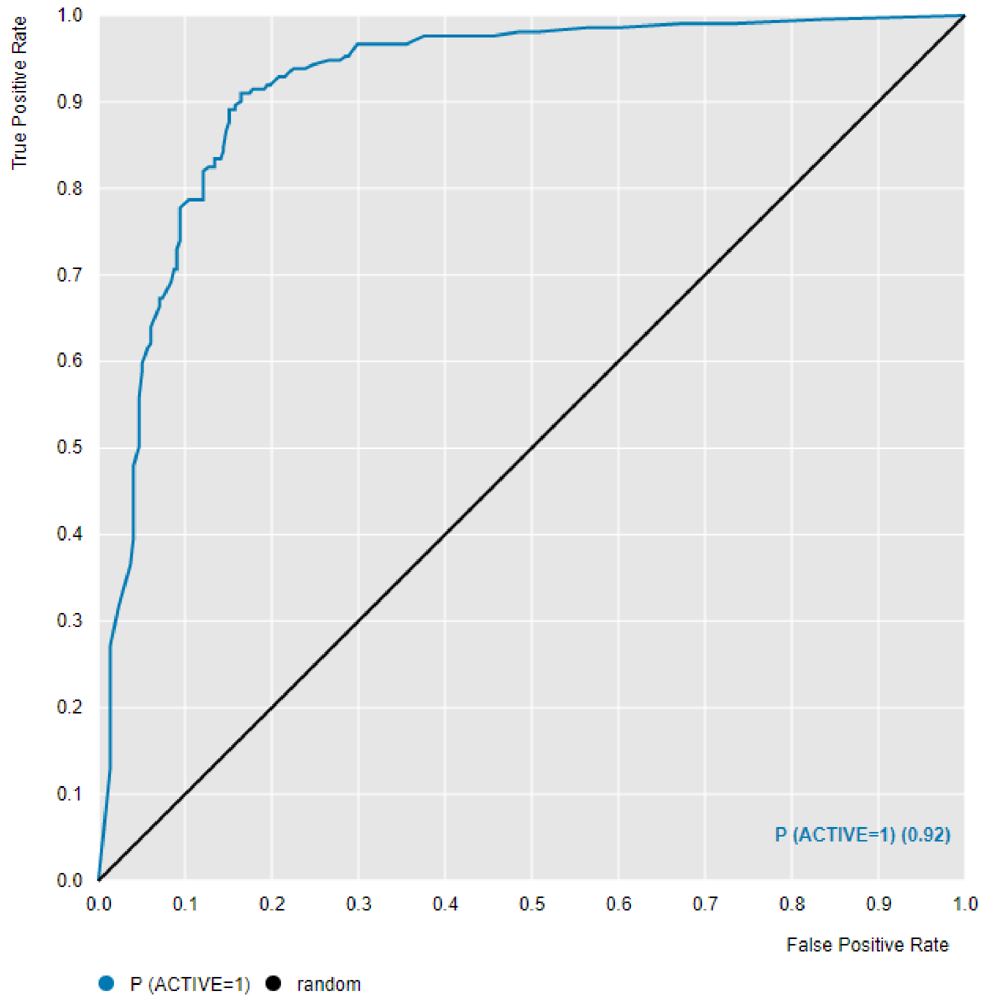

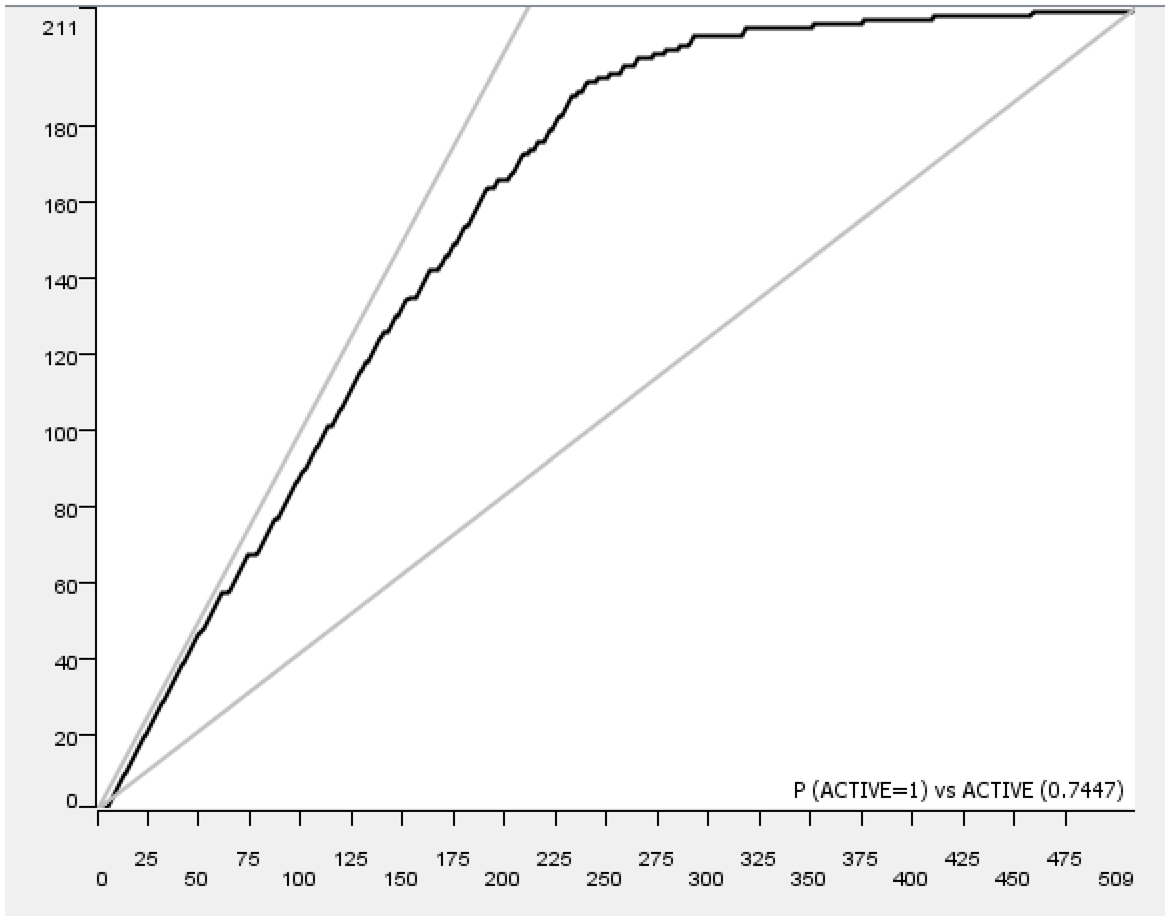

N=509

Top 10% Mean IC50	18.7 nM

	Predicted Active	Predicted Inactive
Active	179	32
Inactive	43	255

Decision on DefGood - RF

- Work in ALK showed DefGood in the NBN was similar to DefGood in an RF. Therefore, this value for DefGood from the NBN design was used as DefGood.

PNN - <15 nM DefGood in HIV Protease

- Properties List (46 calculatable properties were used)
- Parameterization (Global Accuracy for Activity at a 15 nM decision value was used, and a 5-fold stratified sampling cross validation was used to parameterize theta minus and theta plus) Theta Minus = 0.05 and Theta Plus = 0.35

PNN - <15 nM DefGood in HIV Protease

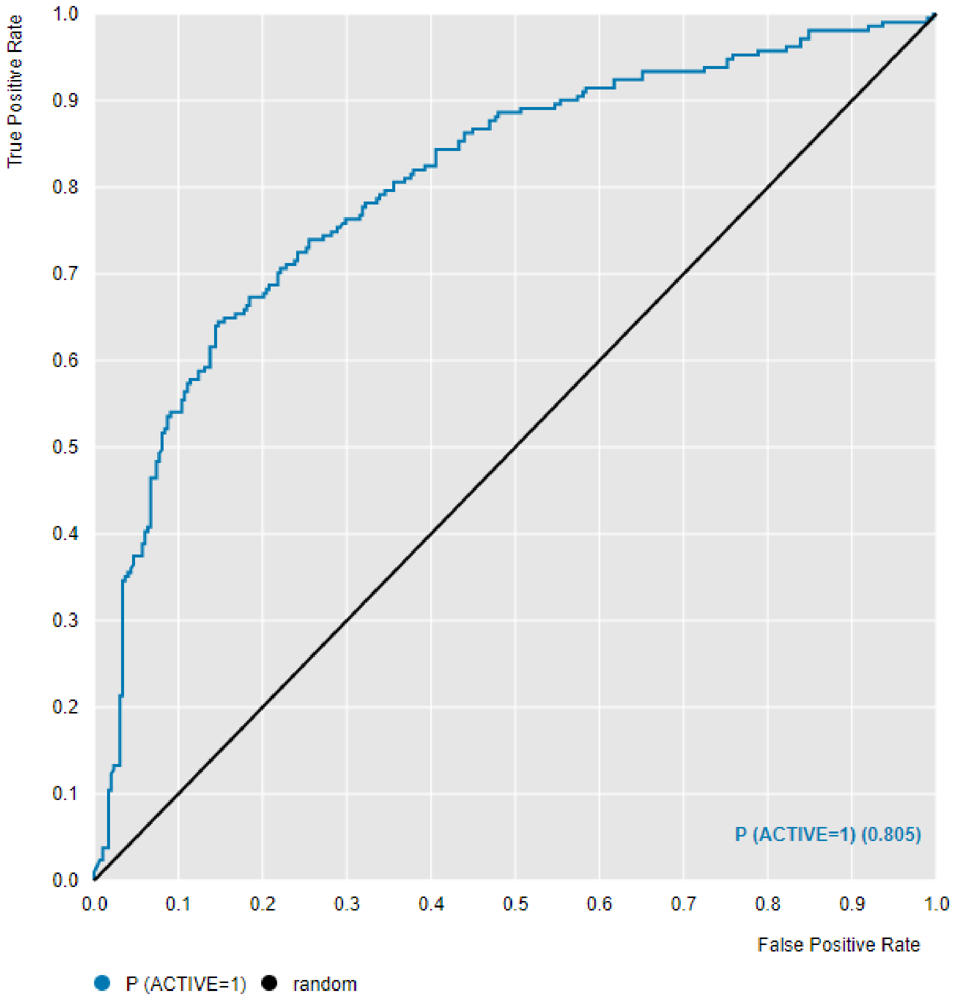

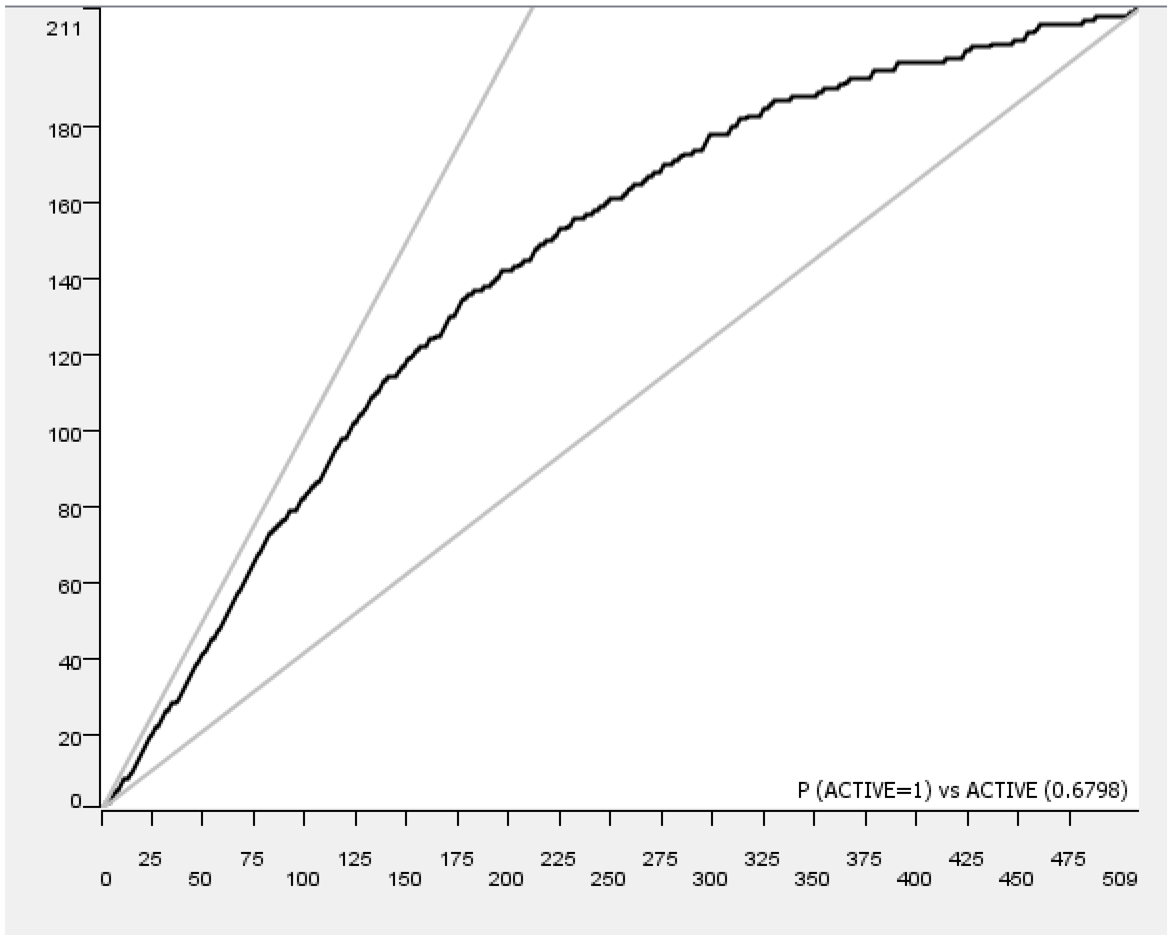

N=509

**Top 10% Mean IC50**	**117 nM Several significant mistakes**

	Predicted Active	Predicted Inactive
Active	126	85
Inactive	41	257

NBN Error Tolerance- <15 nM DefGood in HIV Protease

- 0-50% absolute error

<15 nM DefGood in HIV Protease, 10% error; Random seed = 1515533876005

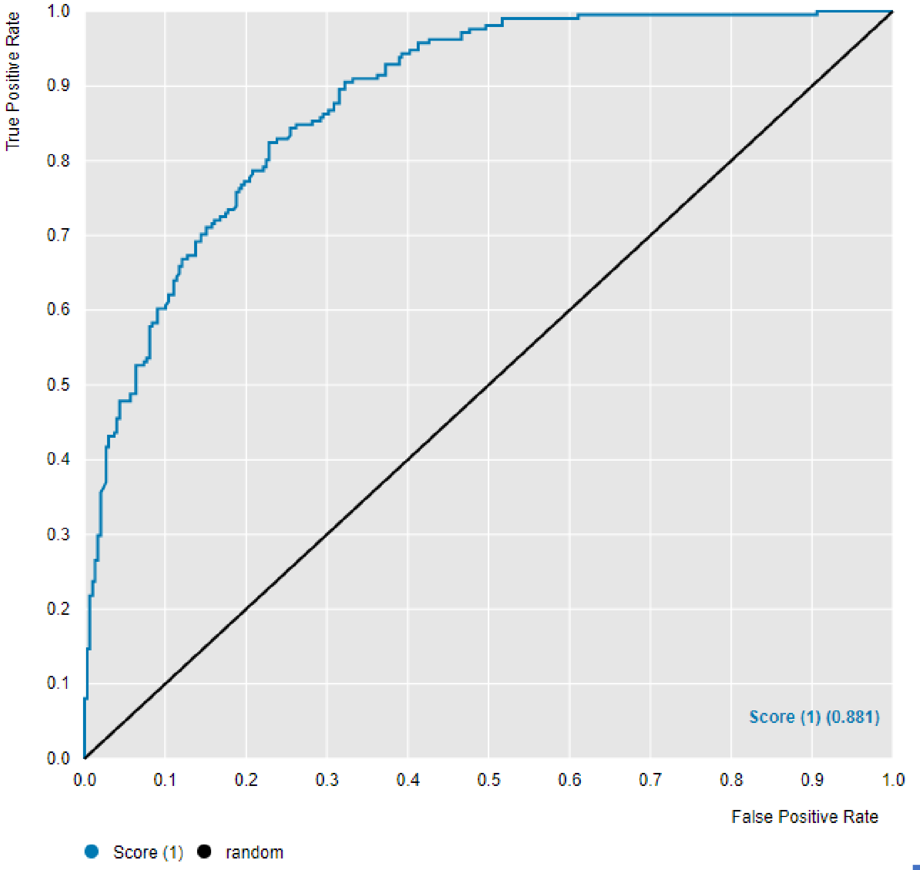

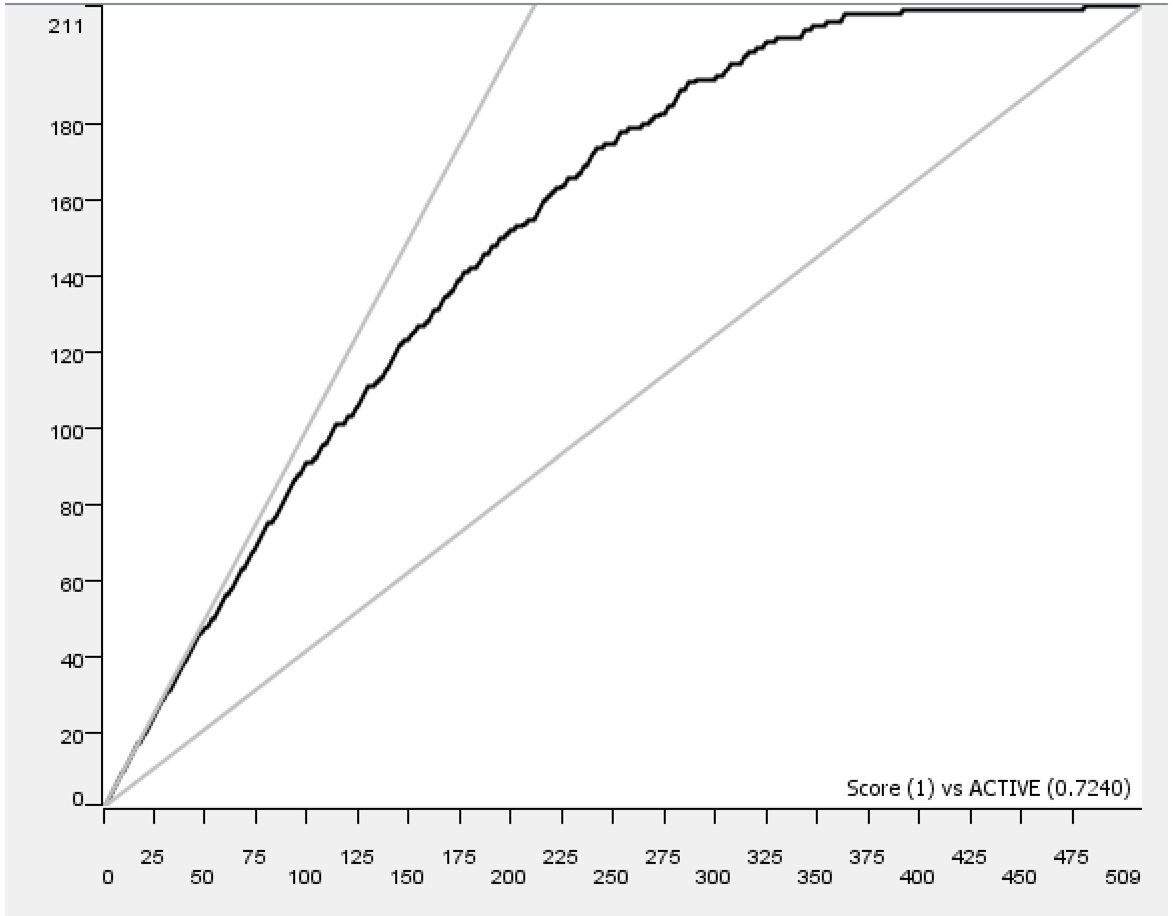

N=509

**Top 10% Mean IC50**	**1.83 nM**

	**Predicted Active**	**Predicted Inactive**
Active	174	37
Inactive	69	229

<15 nM DefGood in HIV Protease, 20% error;

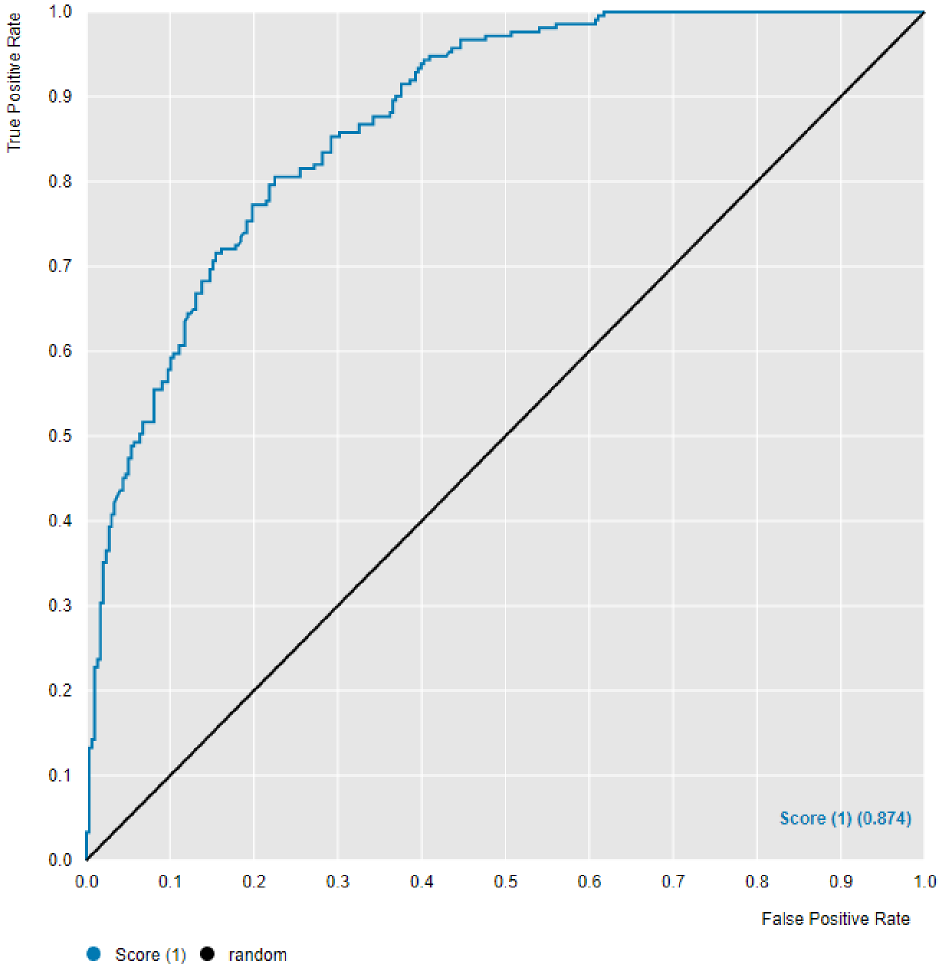

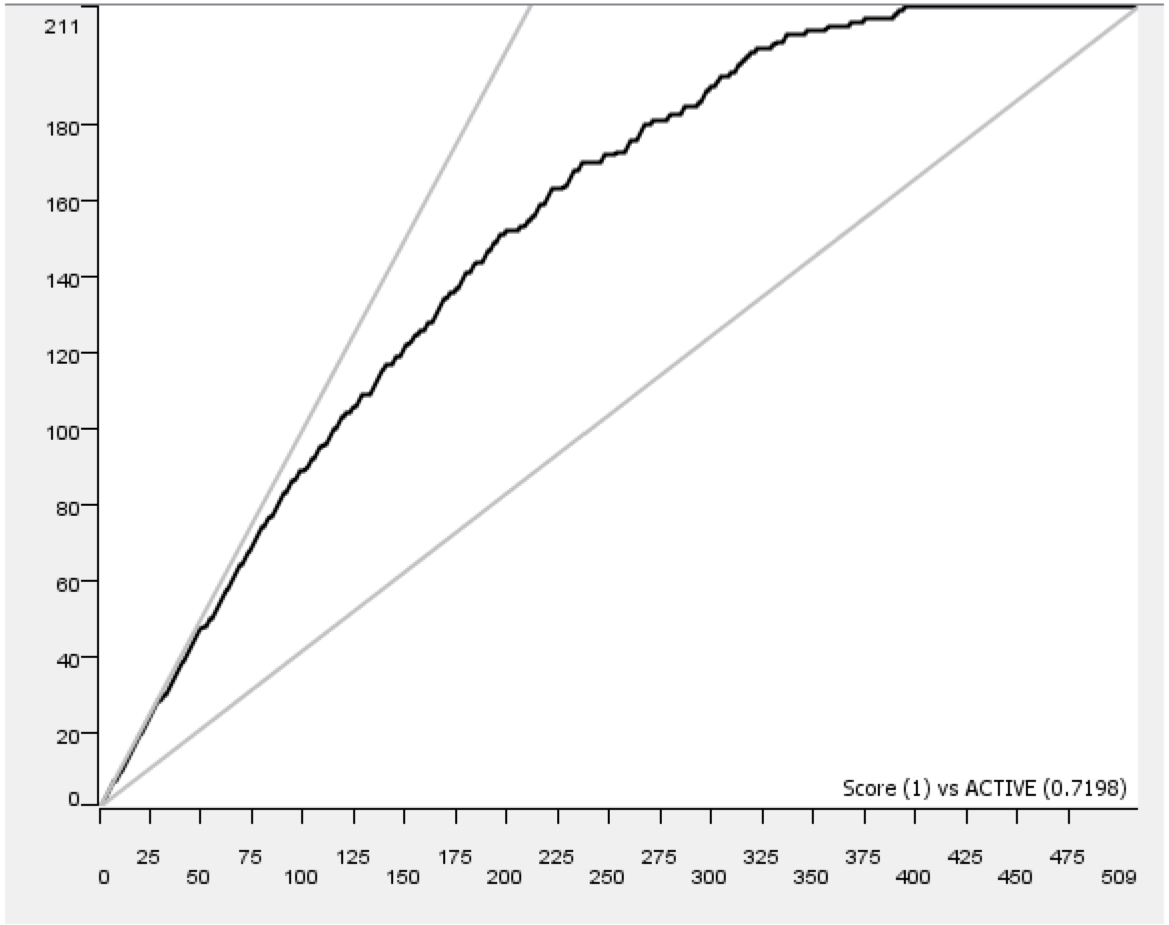

N=509

Top 10% Mean IC50	2.26 nM

	Predicted Active	Predicted Inactive
Active	177	34
Inactive	87	211

<15 nM DefGood in HIV Protease, 30% error;

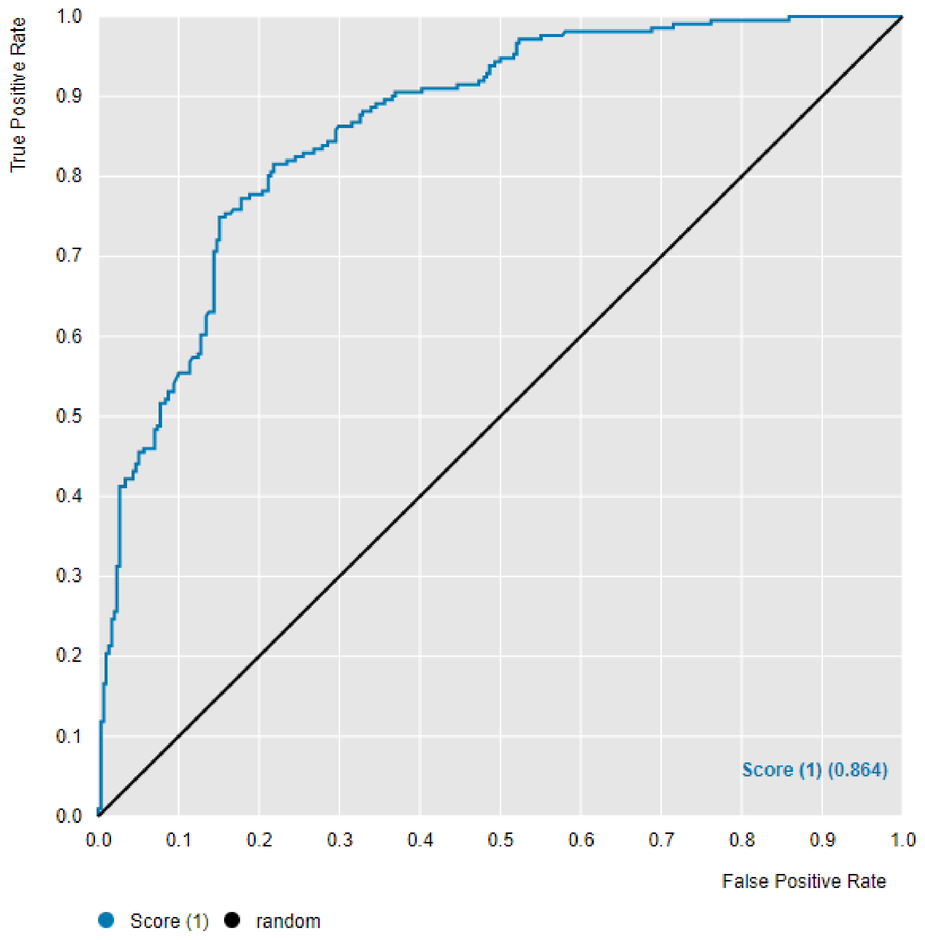

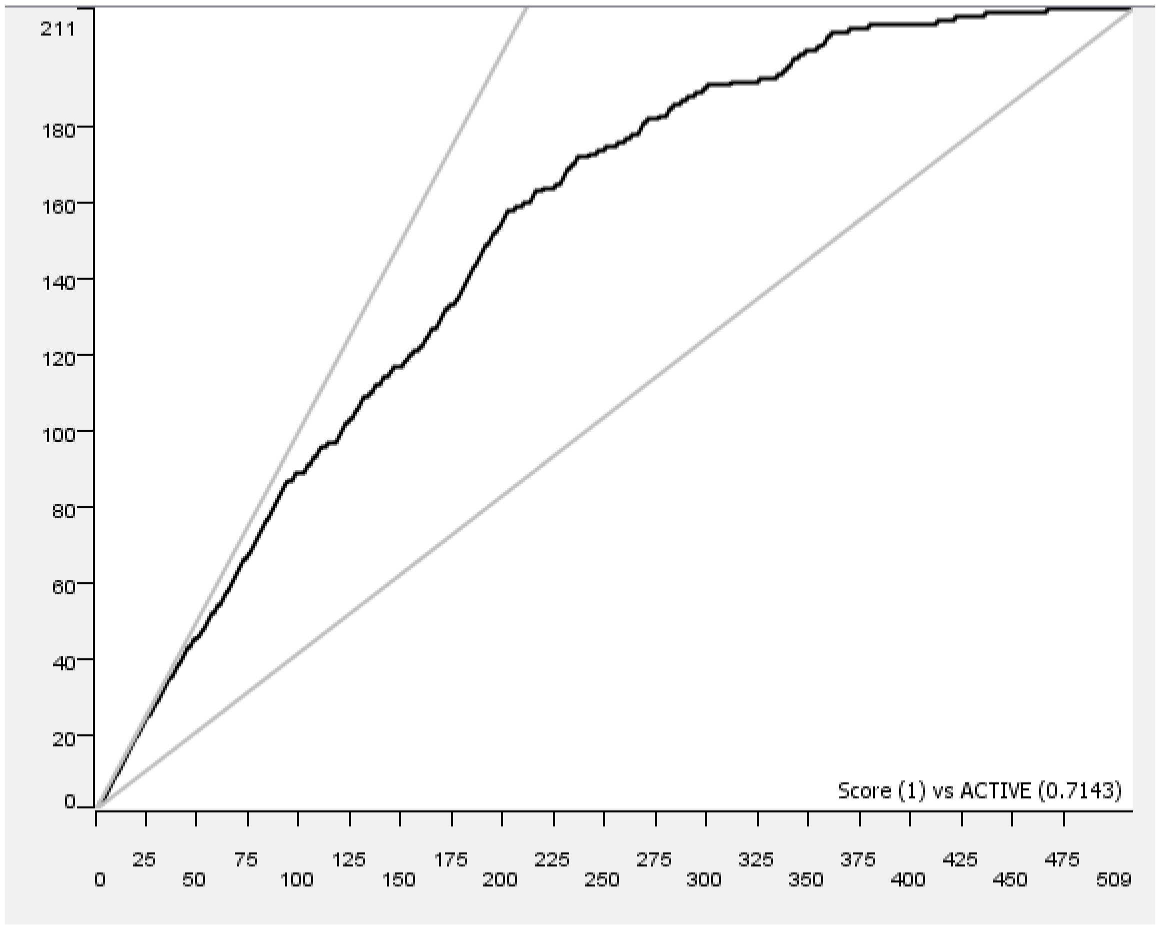

N=509

Top 10% Mean IC50	7.90 nM

	Predicted Active	Predicted Inactive
Active	175	36
Inactive	78	220

<15 nM DefGood in HIV Protease, 40% error;

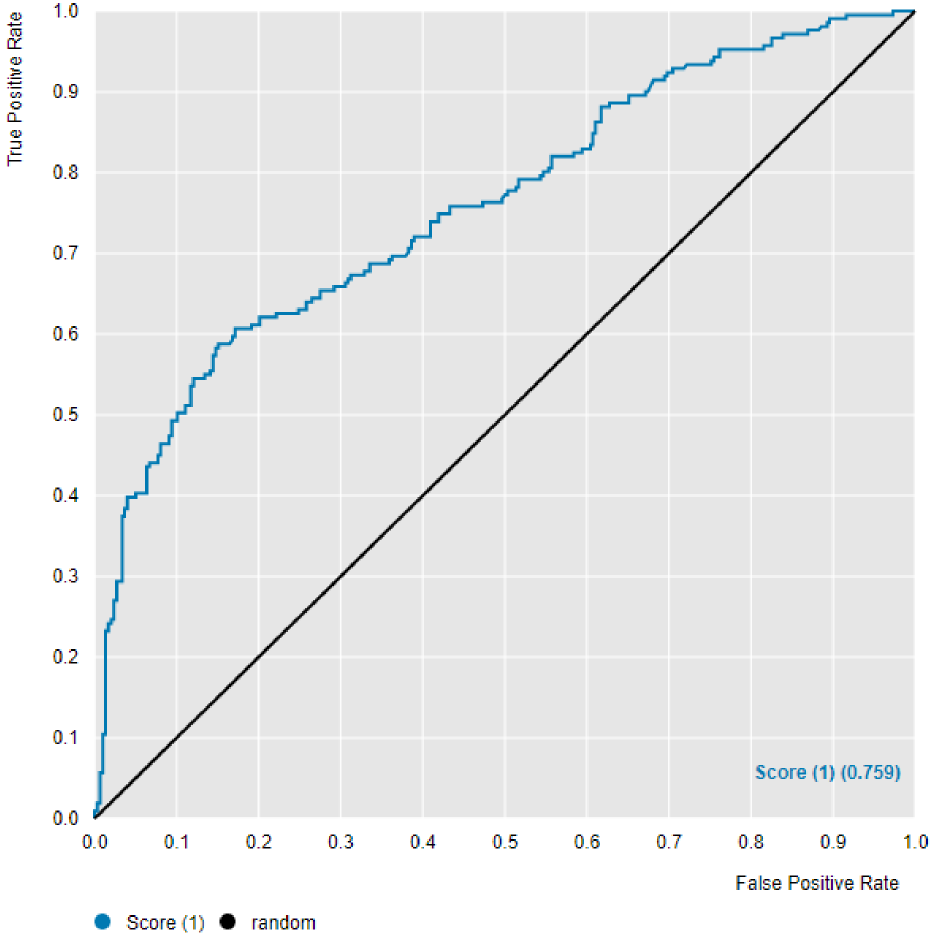

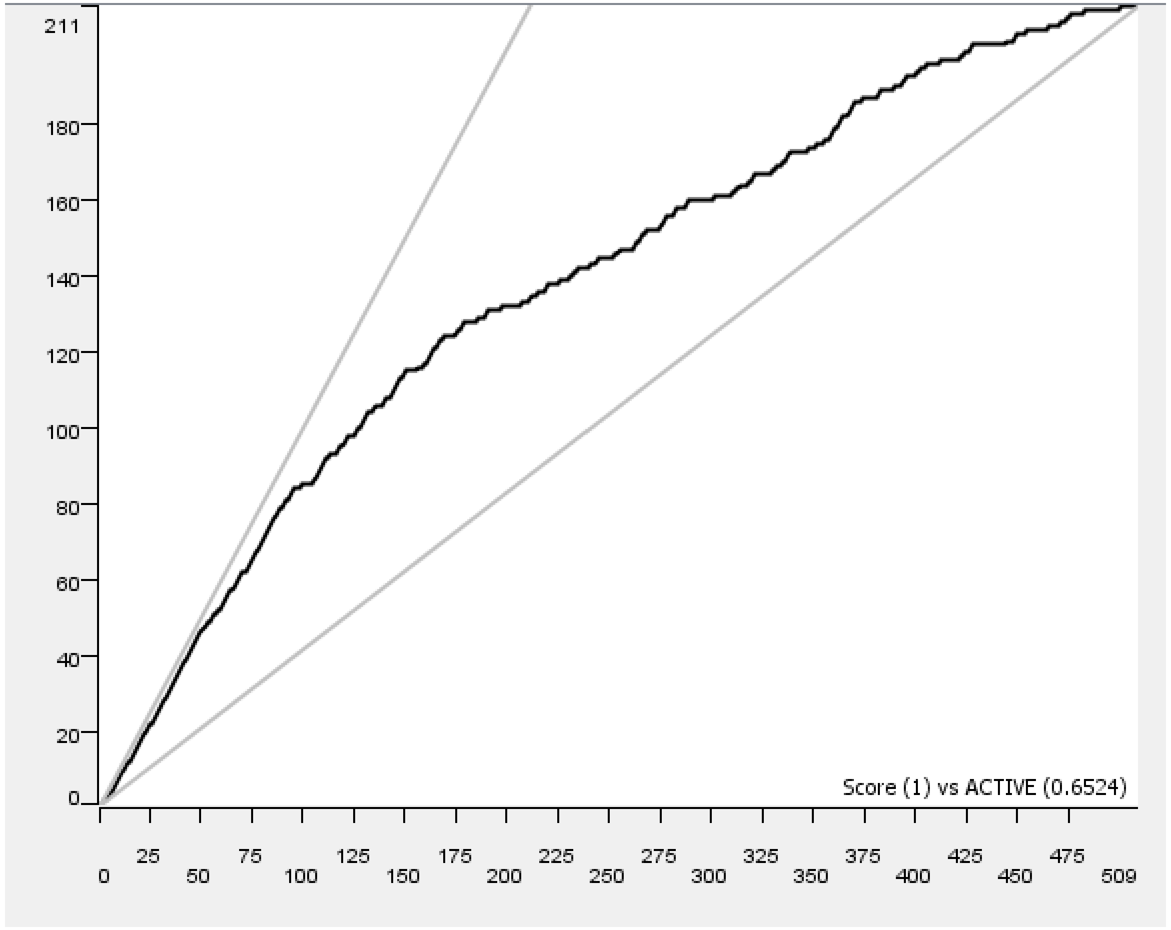

N=509

**Top 10% Mean IC50**	**4.6 nM**

	Predicted Active	Predicted Inactive
Active	116	95
Inactive	42	256

<15 nM DefGood in HIV Protease, 45% error;

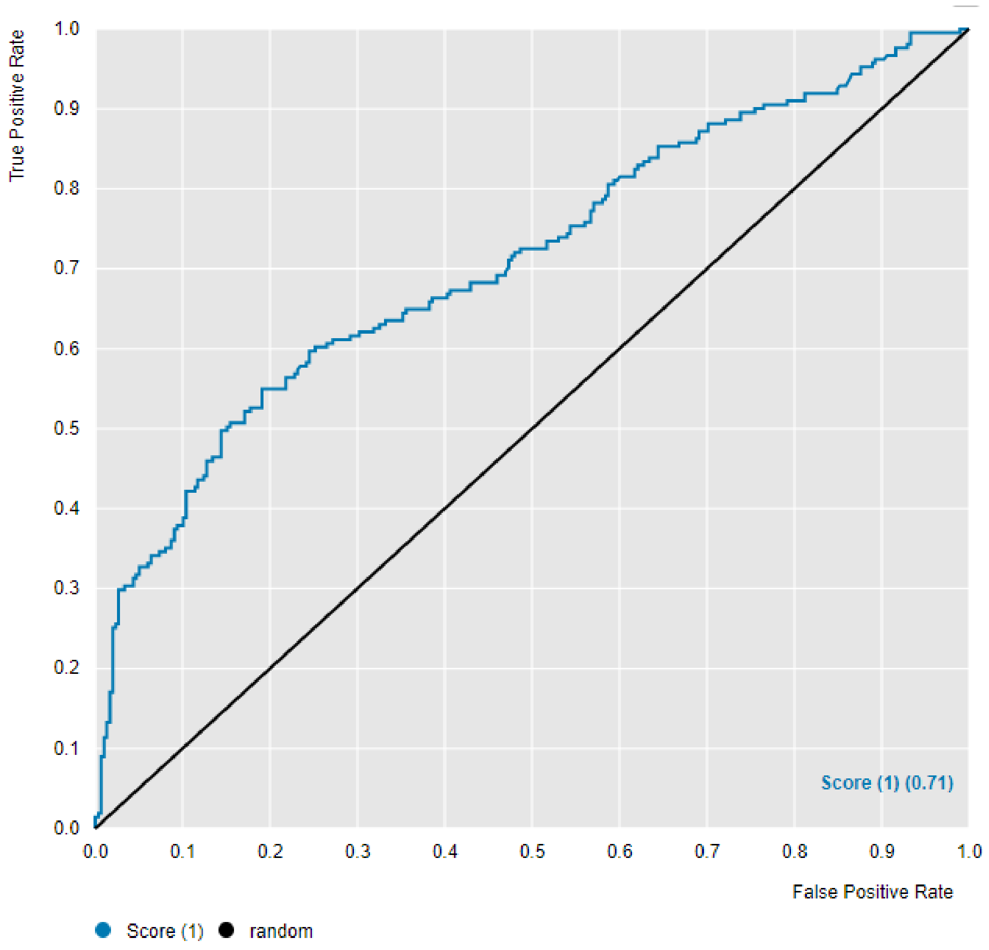

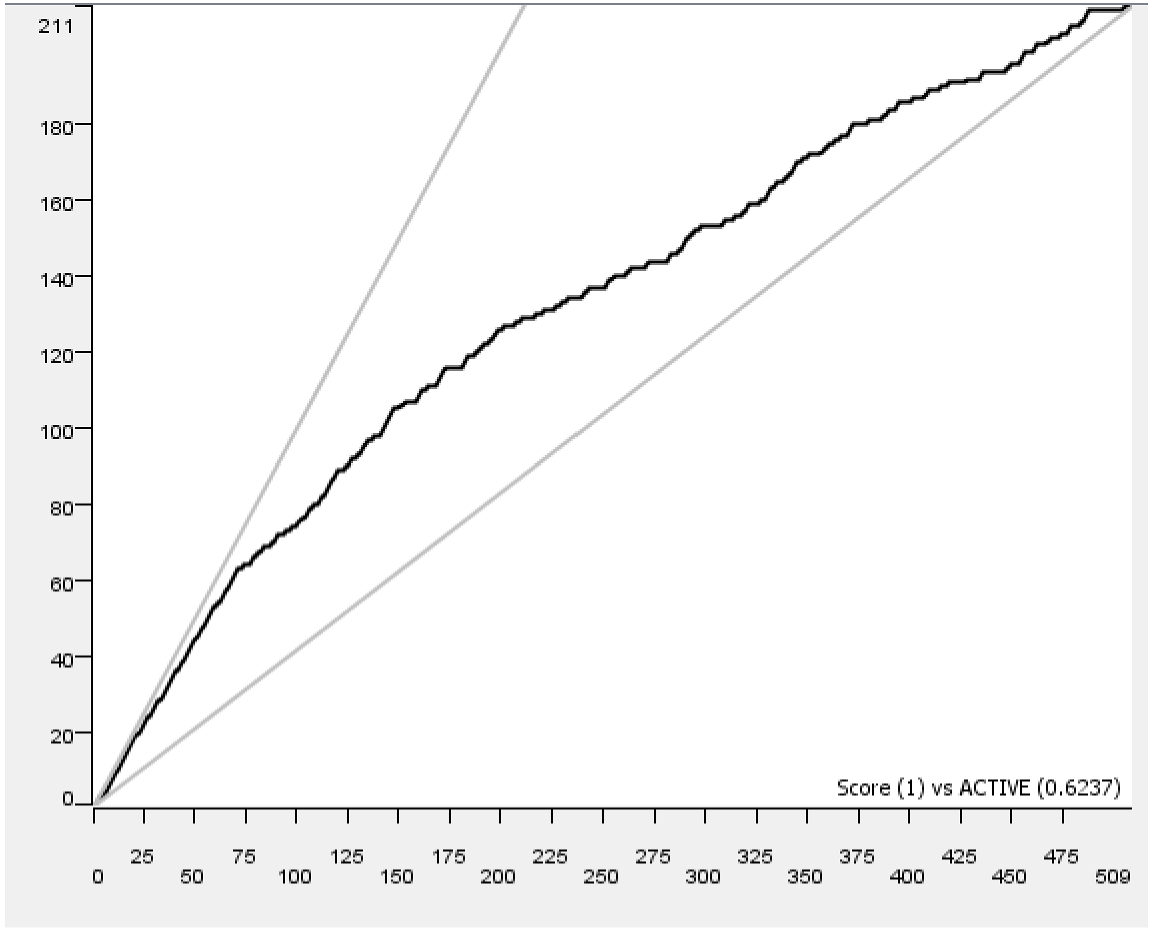

N=509

**Top 10% Mean IC50**	**5.8 nM**

	Predicted Active	Predicted Inactive
Active	123	88
Inactive	72	226

<15 nM DefGood in HIV Protease, 50% error;

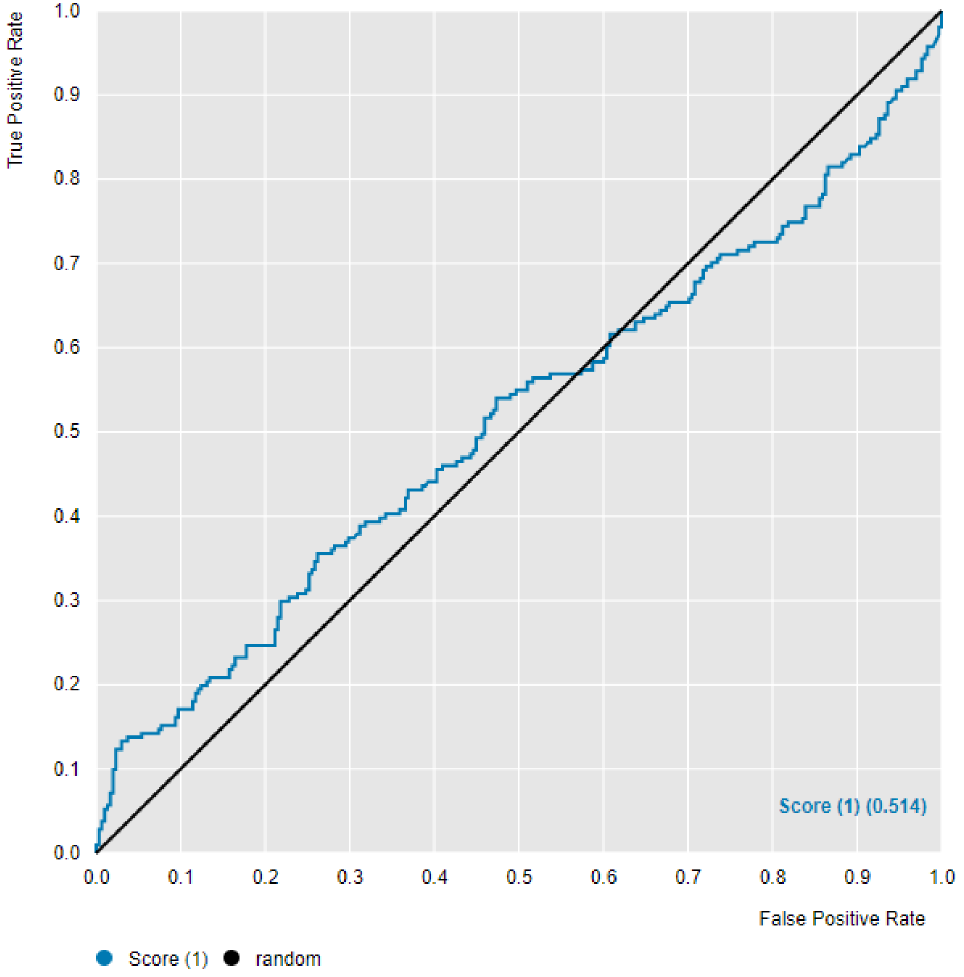

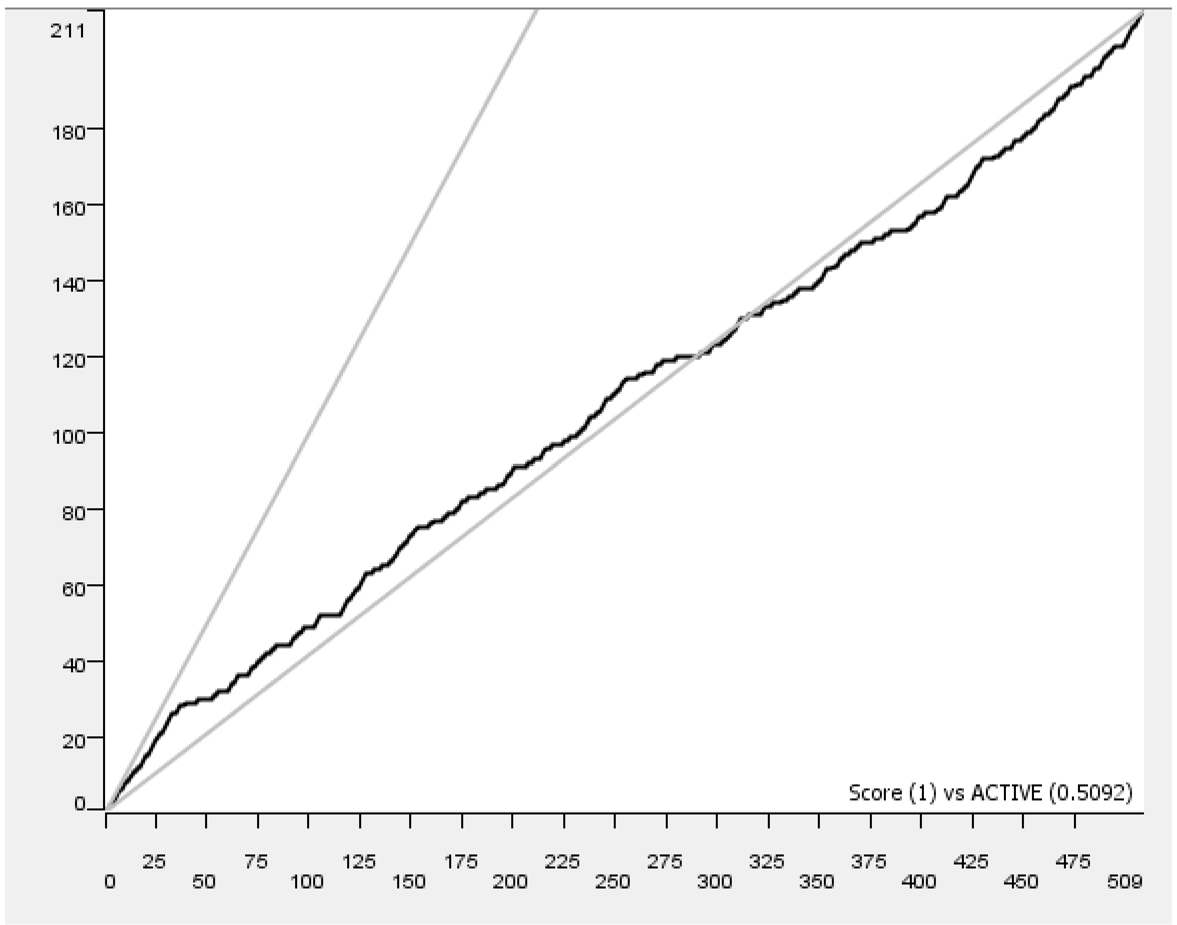

N=509

**Top 10% Mean IC50**	**8,500 nM**

	Predicted Active	Predicted Inactive
Active	39	172
Inactive	35	263

<15nM DefGood in HIV Protease, 10% error; Random seed = 429

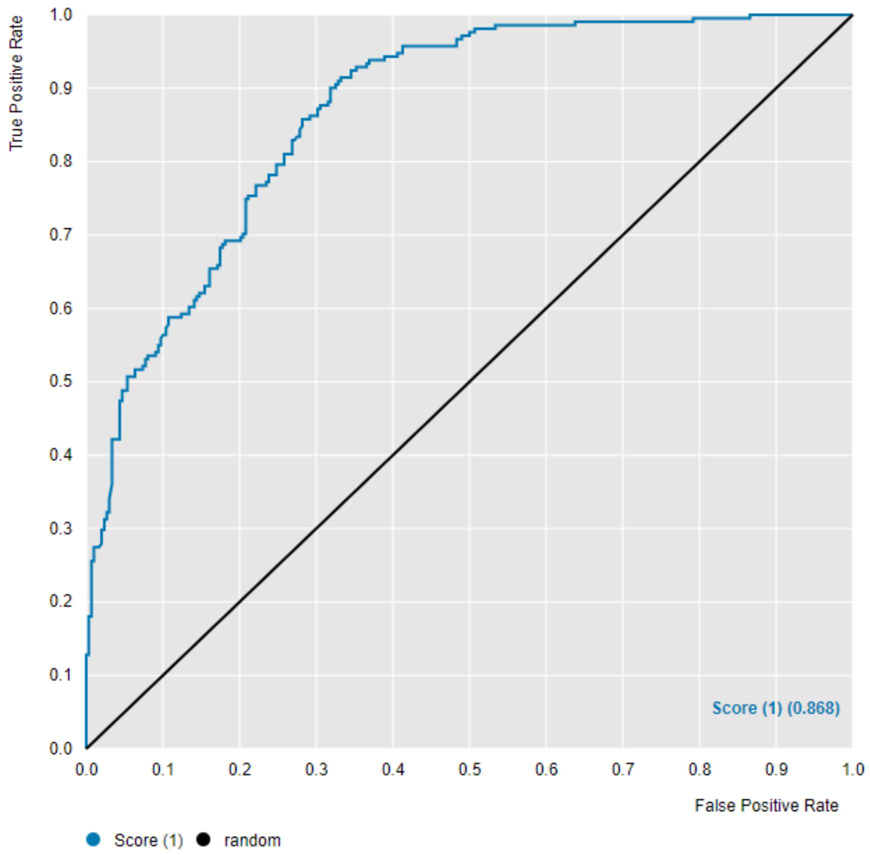

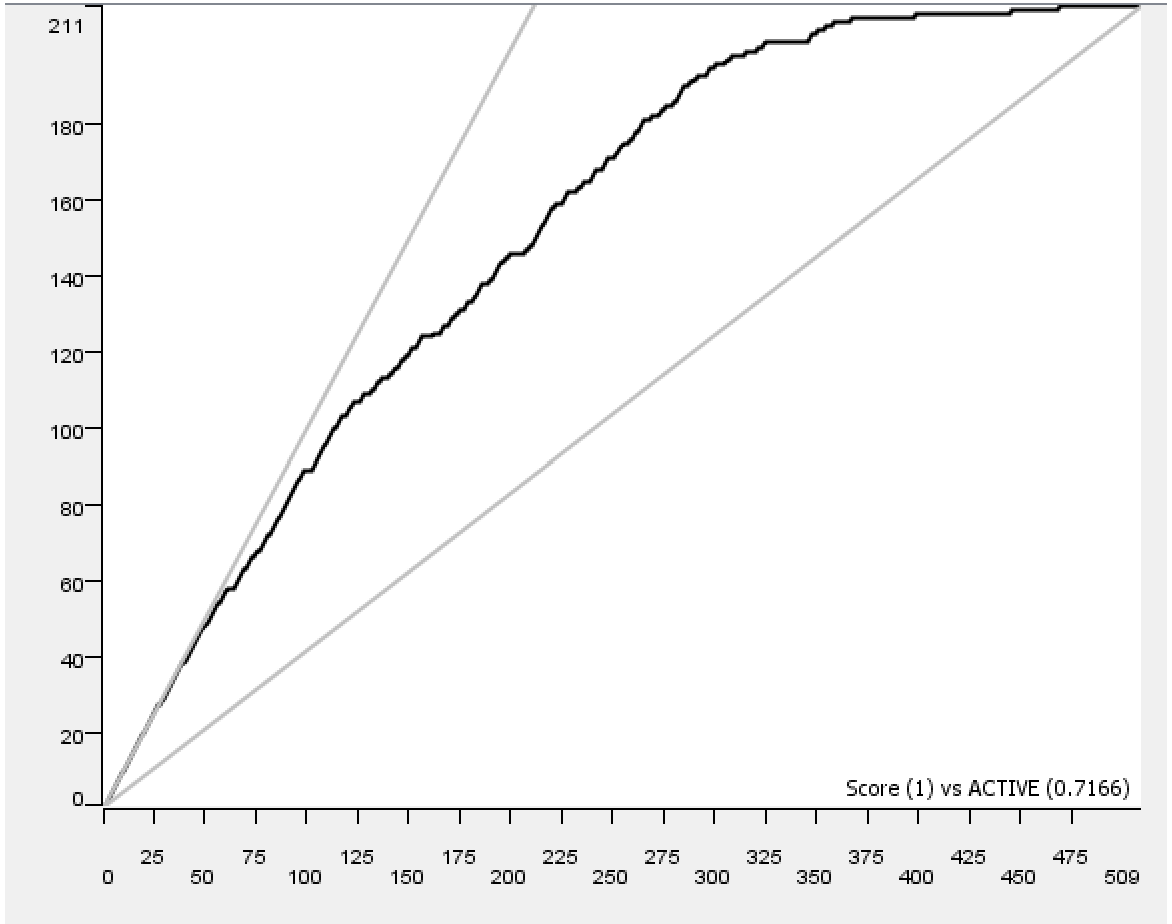

N=509

**Top 10% Mean IC50**	**1.58 nM**

	Predicted Active	Predicted Inactive
Active	171	40
Inactive	80	218

<15nM DefGood in HIV Protease, 20% error; Random seed = 429

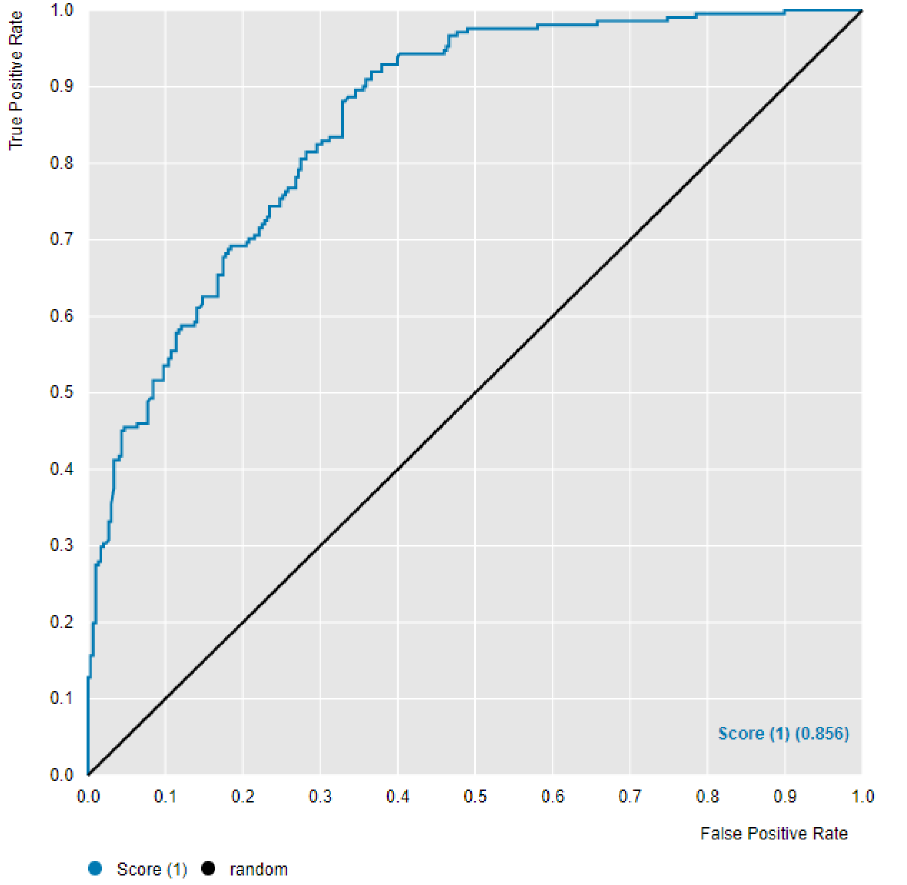

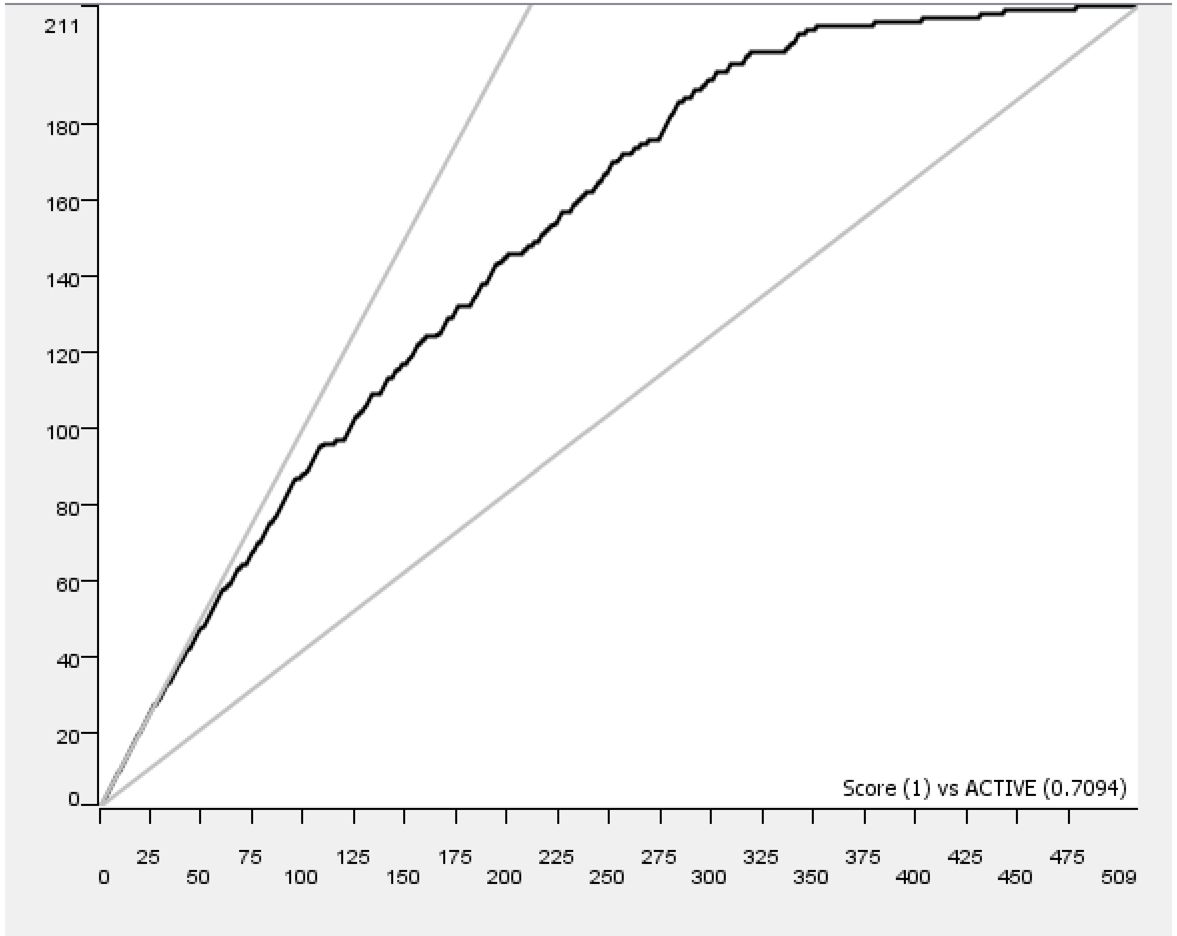

N=509

Top 10% Mean IC50	2.65 nM

	Predicted Active	Predicted Inactive
Active	175	36
Inactive	92	206

<15nM DefGood in HIV Protease, 30% error; Random seed = 429

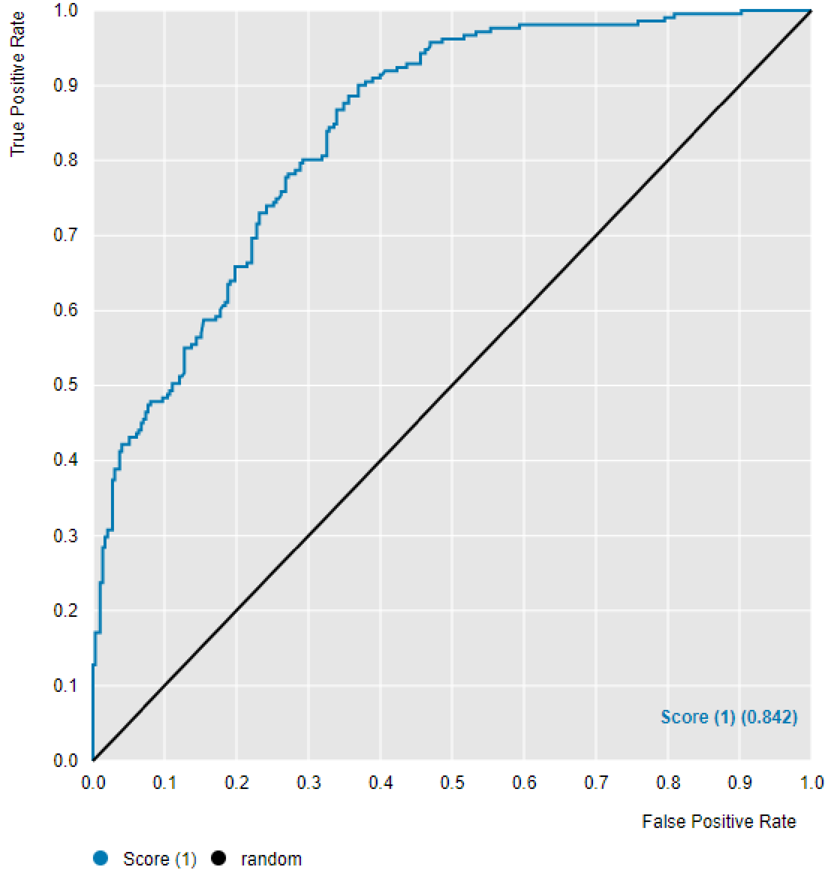

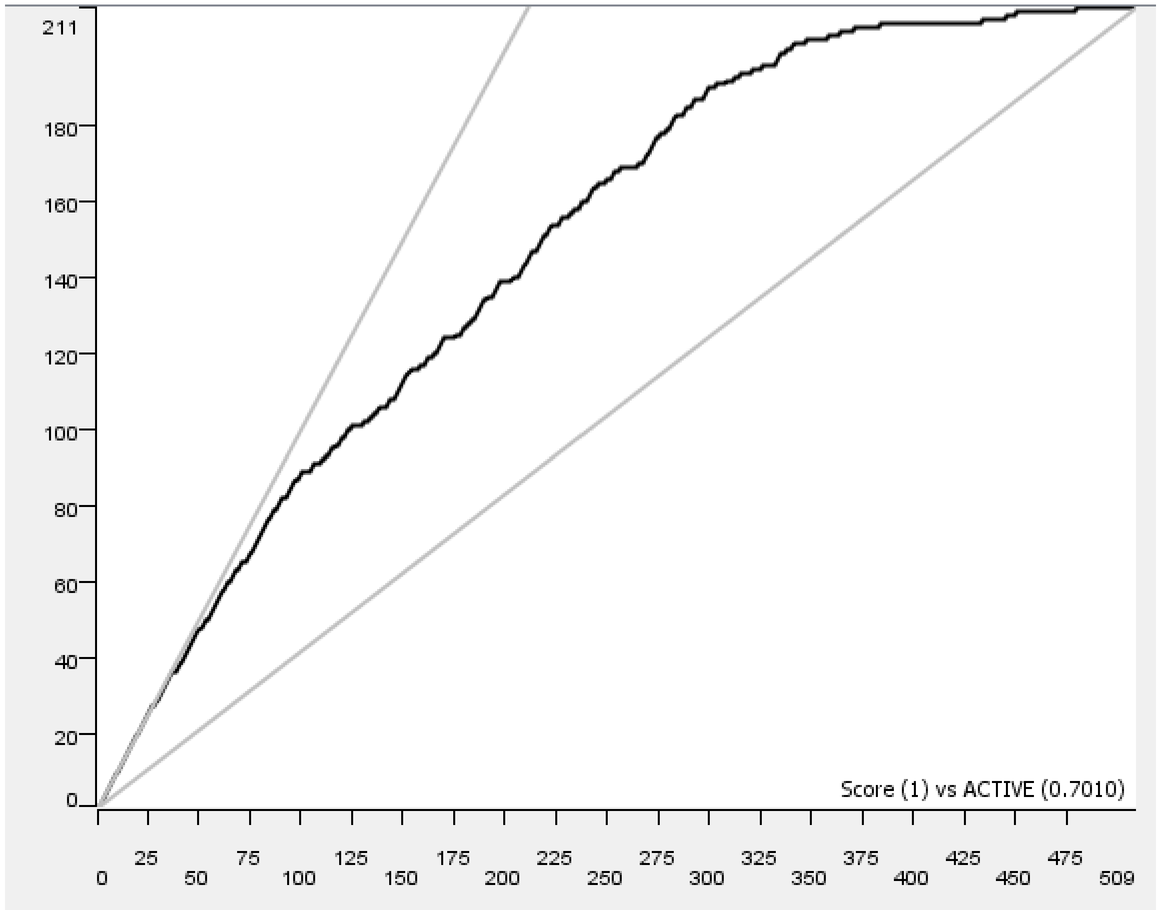

N=509

**Top 10% Mean IC50**	**3.13 nM**

	Predicted Active	Predicted Inactive
Active	187	24
Inactive	110	188

<15nM DefGood in HIV Protease, 40% error; Random seed = 429

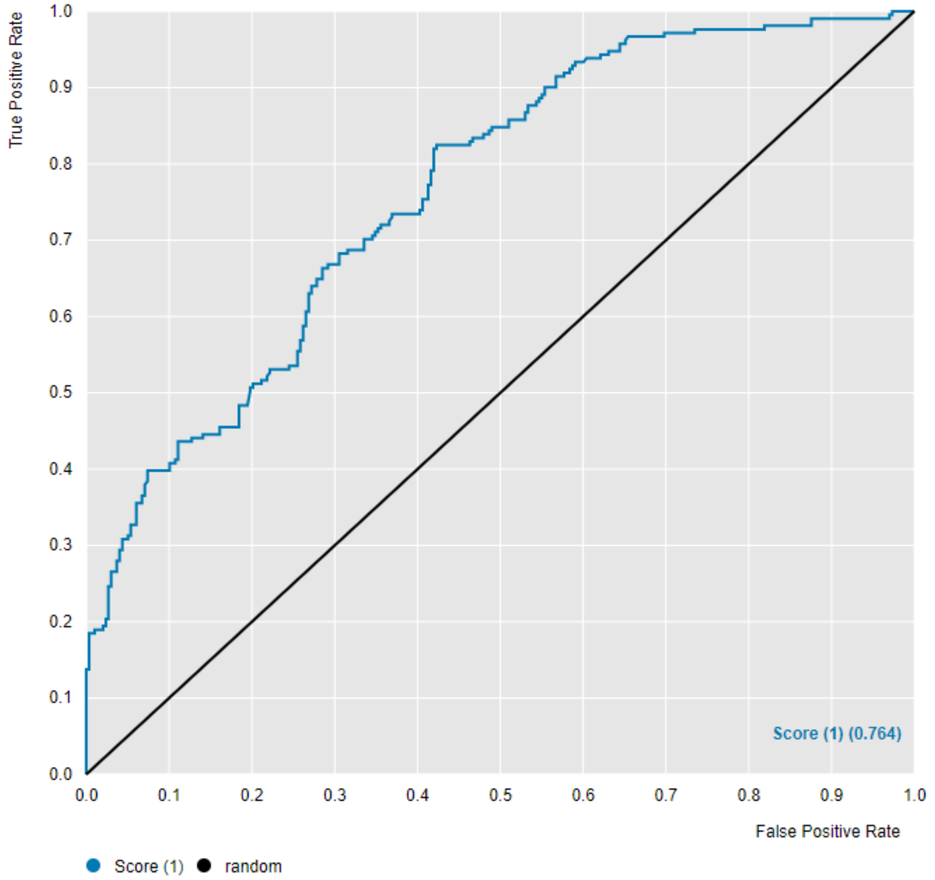

N=509

Top 10% Mean IC50	10.6 nM

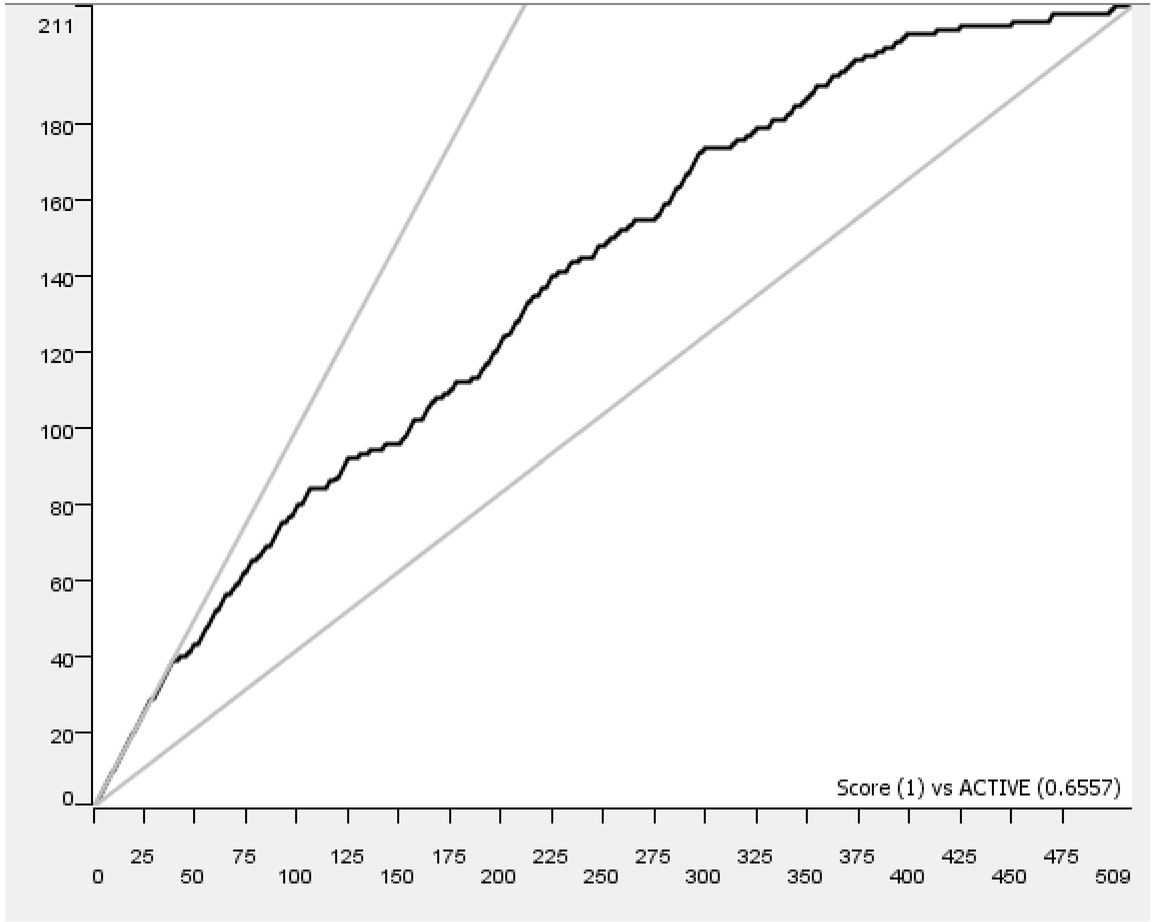

	Predicted Active	Predicted Inactive
Active	196	15
Inactive	176	22

<15nM DefGood in HIV Protease, 45% error; Random seed = 429

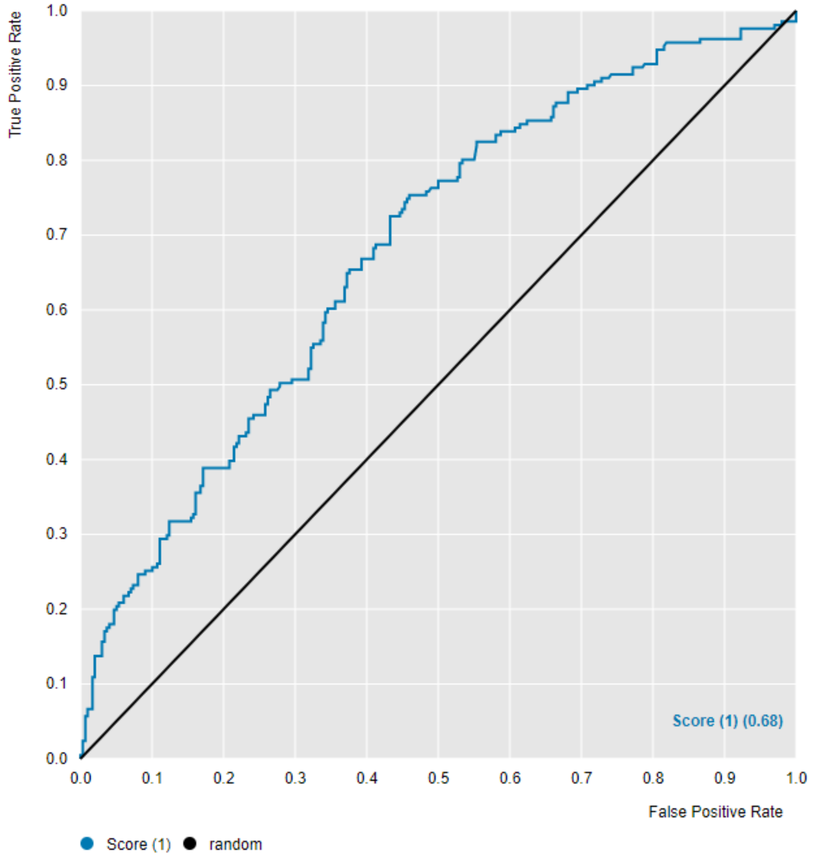

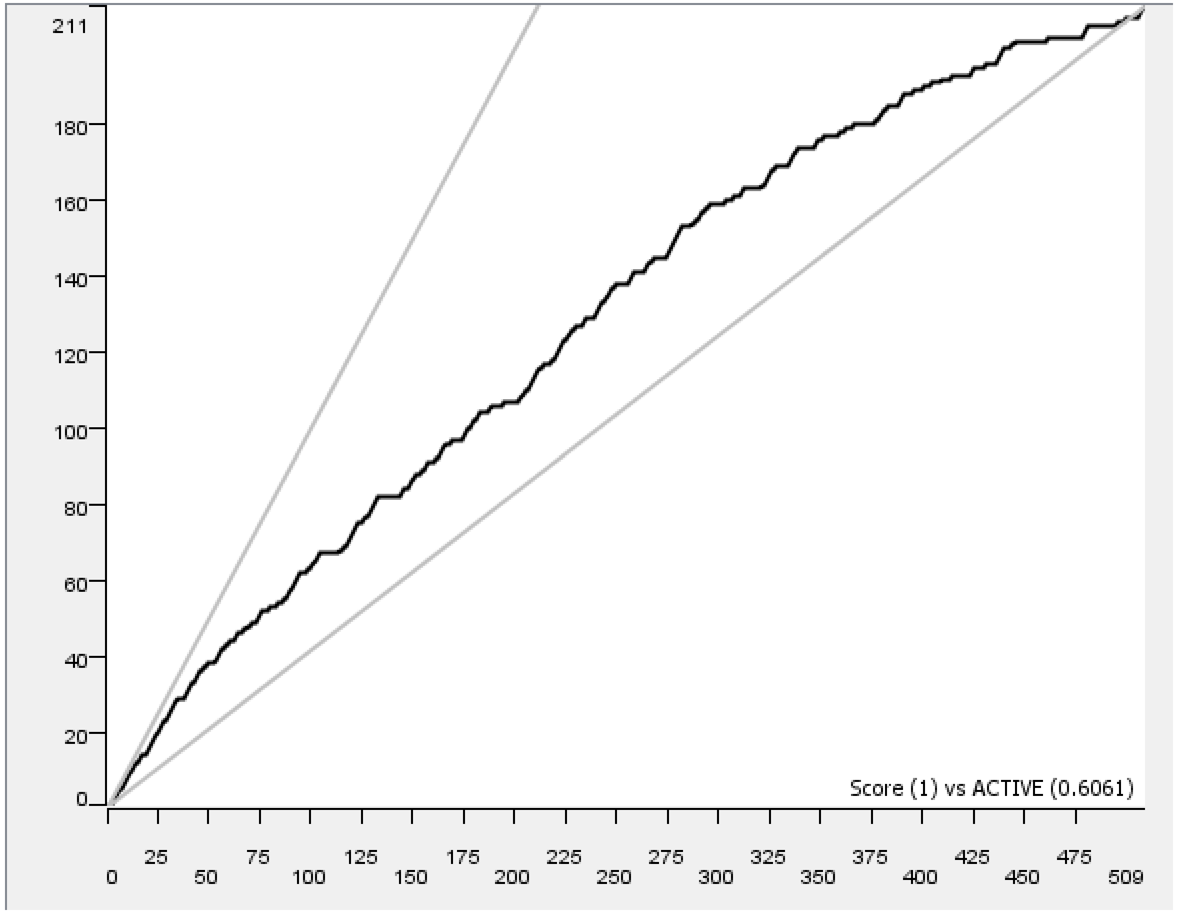

N=509

Top 10% Mean IC50	893 nM

	Predicted Active	Predicted Inactive
Active	189	22
Inactive	210	88

<15nM DefGood in HIV Protease, 10% error; Random seed = 121783

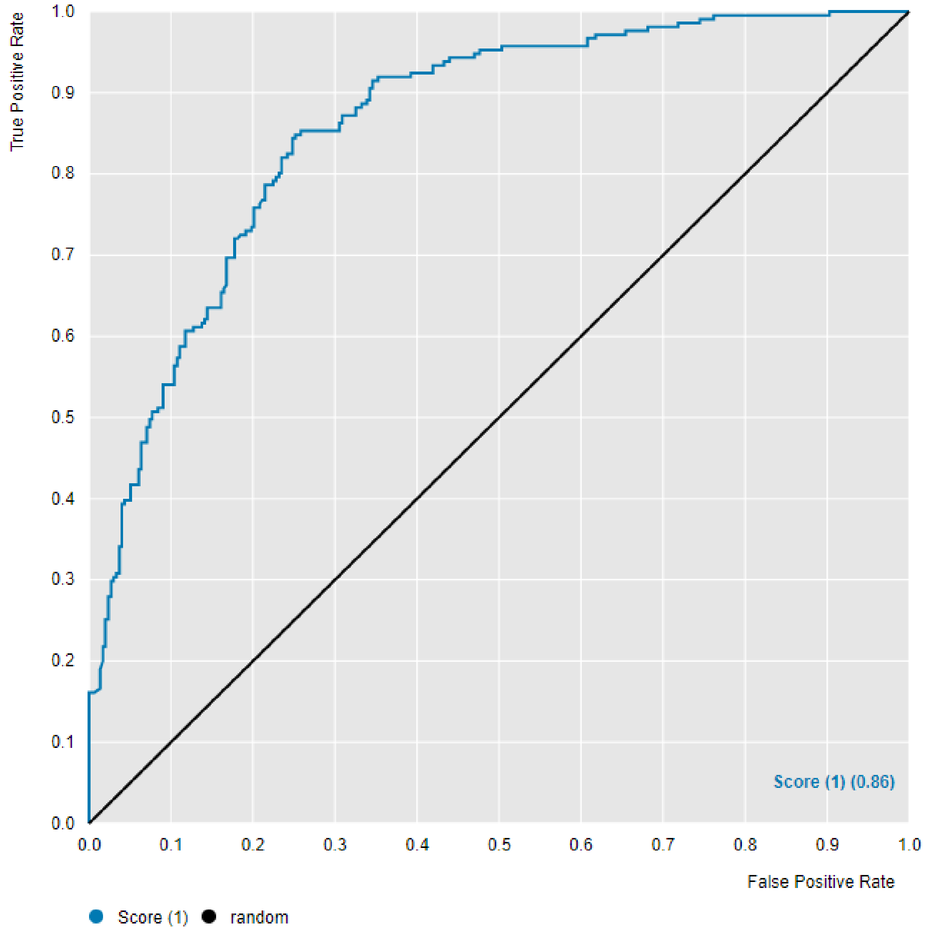

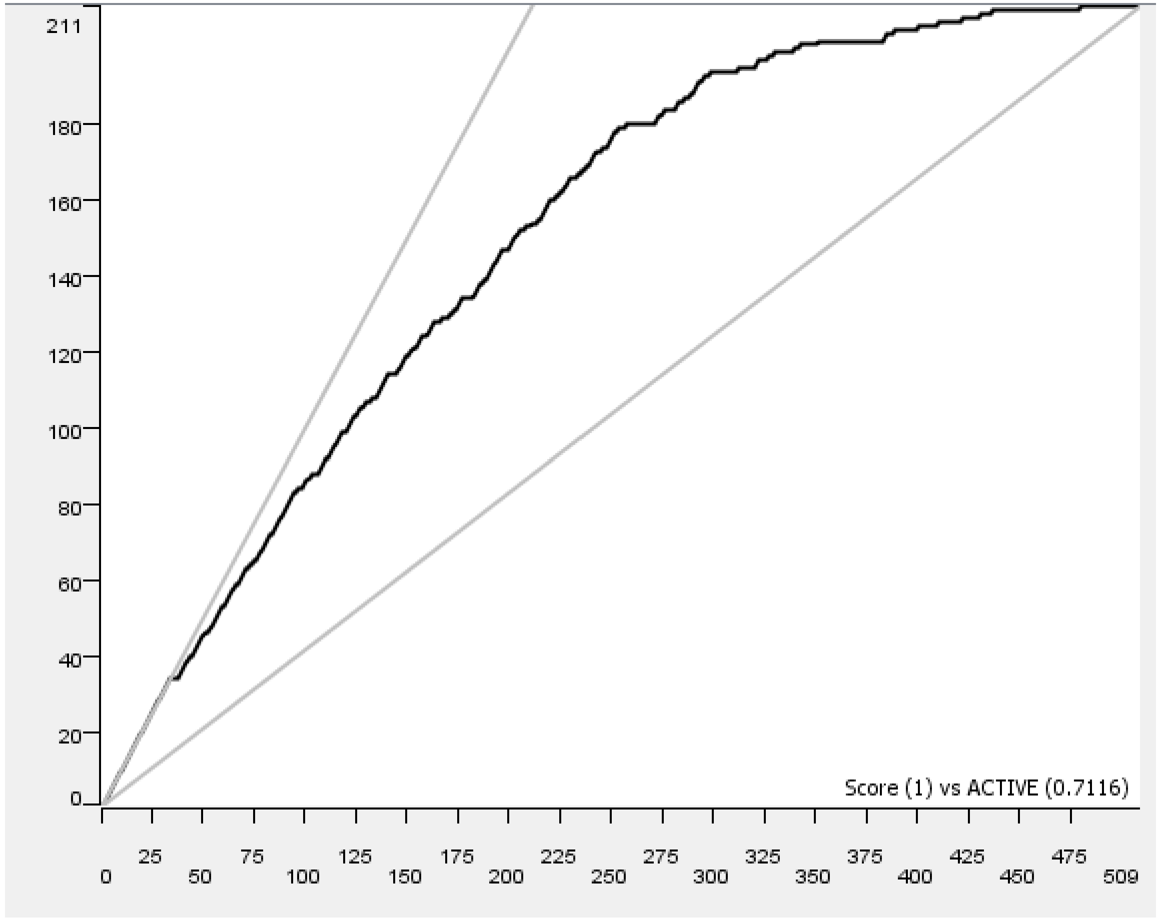

N=509

Top 10% Mean IC50	10.6 nM

	Predicted Active	Predicted Inactive
Active	177	34
Inactive	74	224

<15nM DefGood in HIV Protease, 20% error; Random seed = 121783

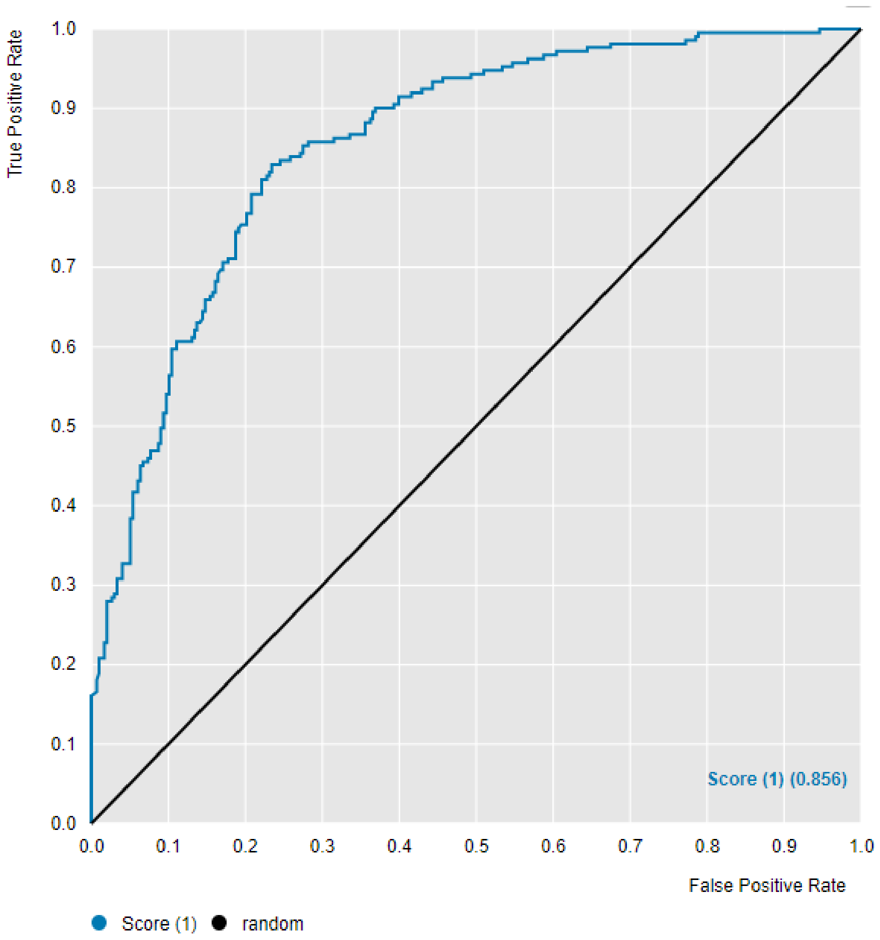

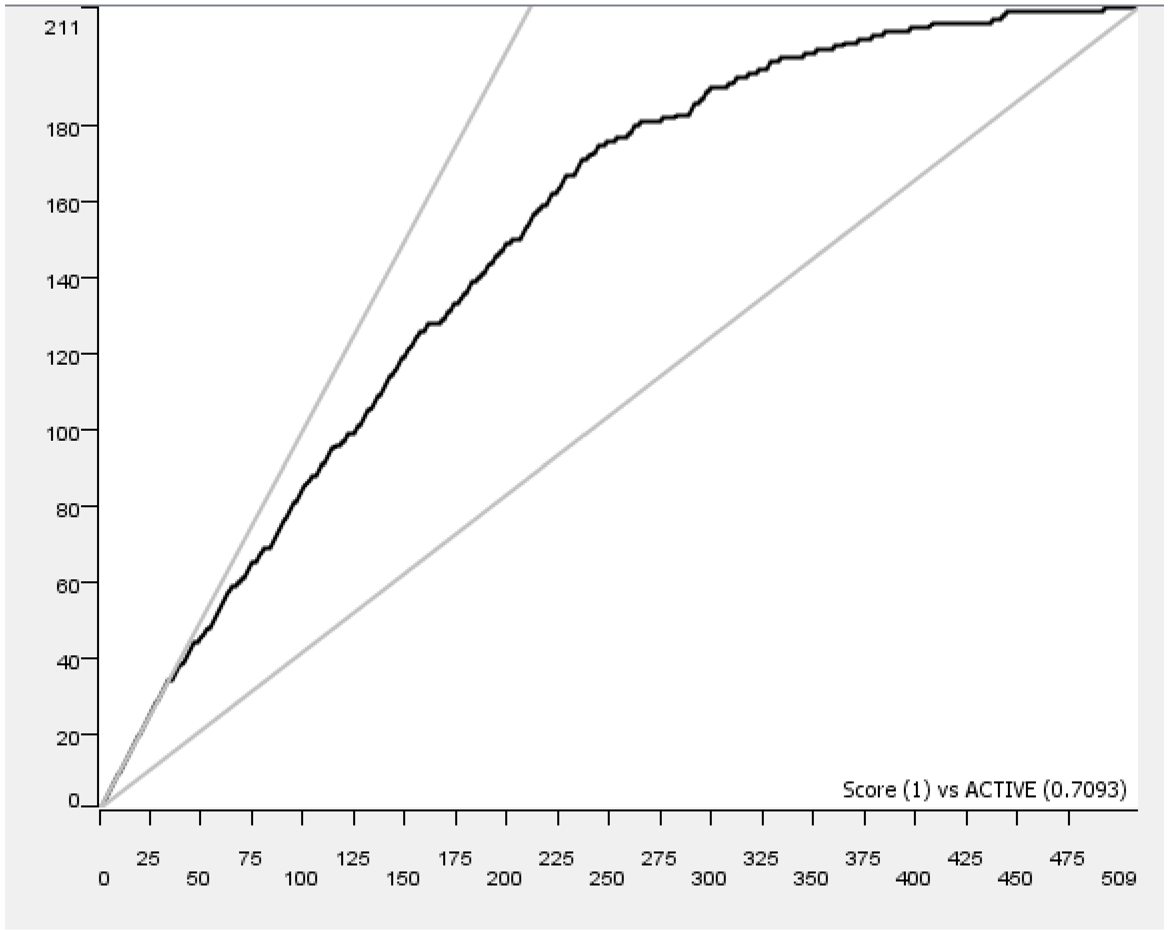

N=509

**Top 10% Mean IC50**	**10.6 nM**

	Predicted Active	Predicted Inactive
Active	175	36
Inactive	70	228

<15nM DefGood in HIV Protease, 30% error; Random seed = 121783

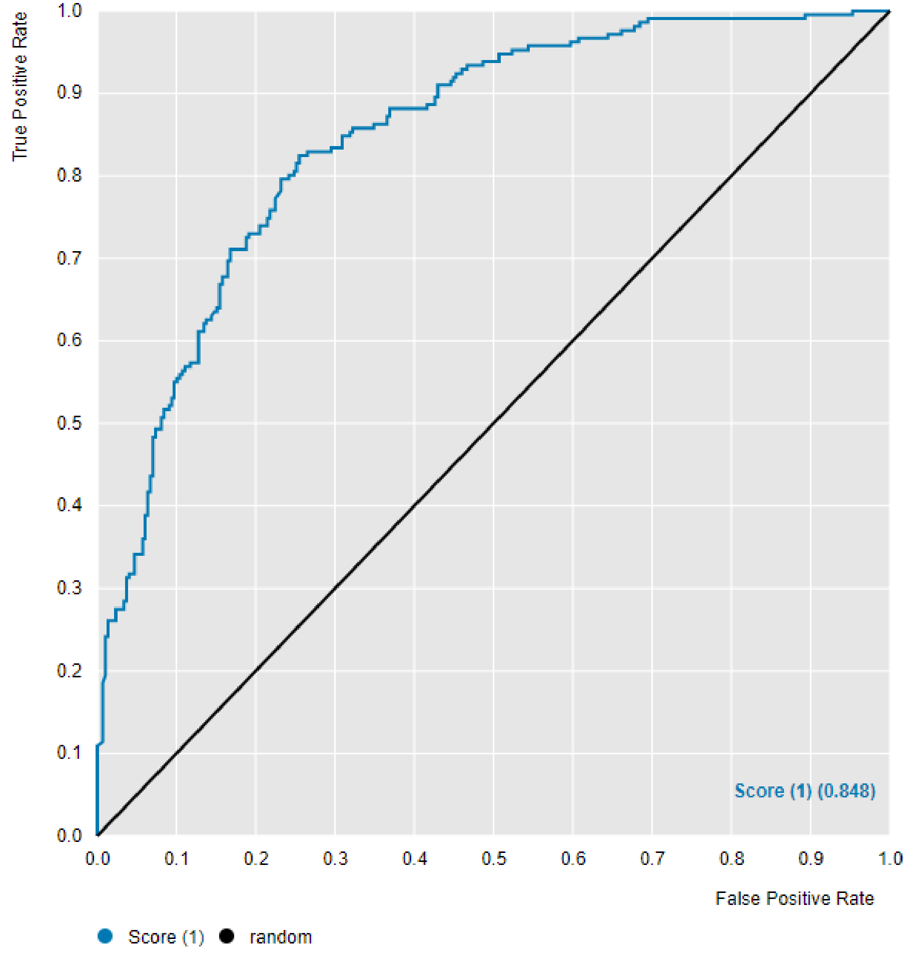

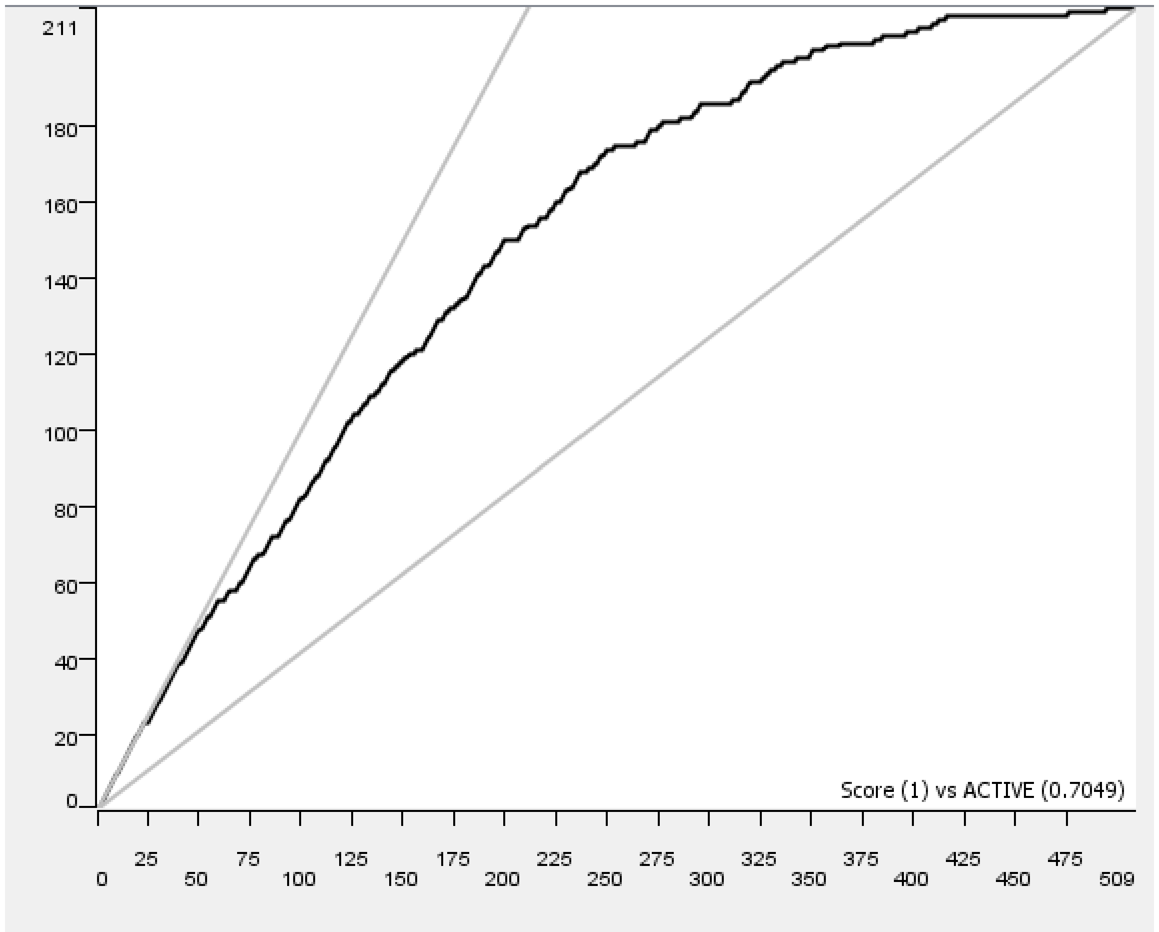

N=509

Top 10% Mean IC50	7.52 nM

	Predicted Active	Predicted Inactive
Active	168	43
Inactive	71	227

<15nM DefGood in HIV Protease, 40% error; Random seed = 121783

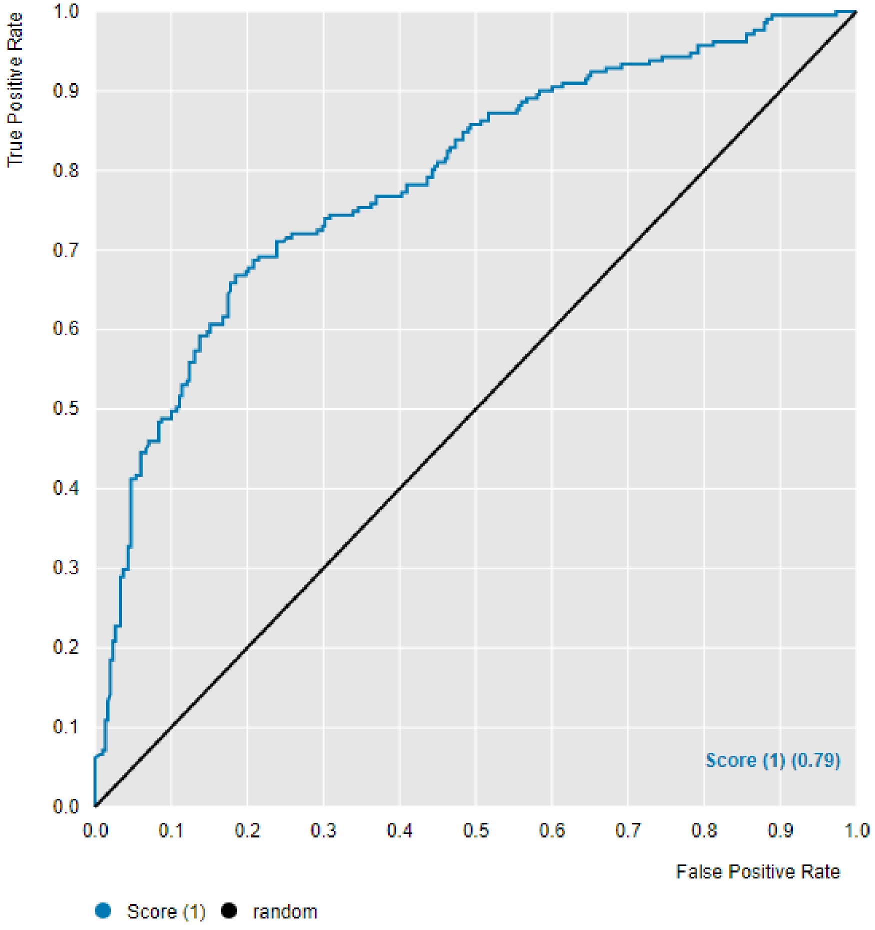

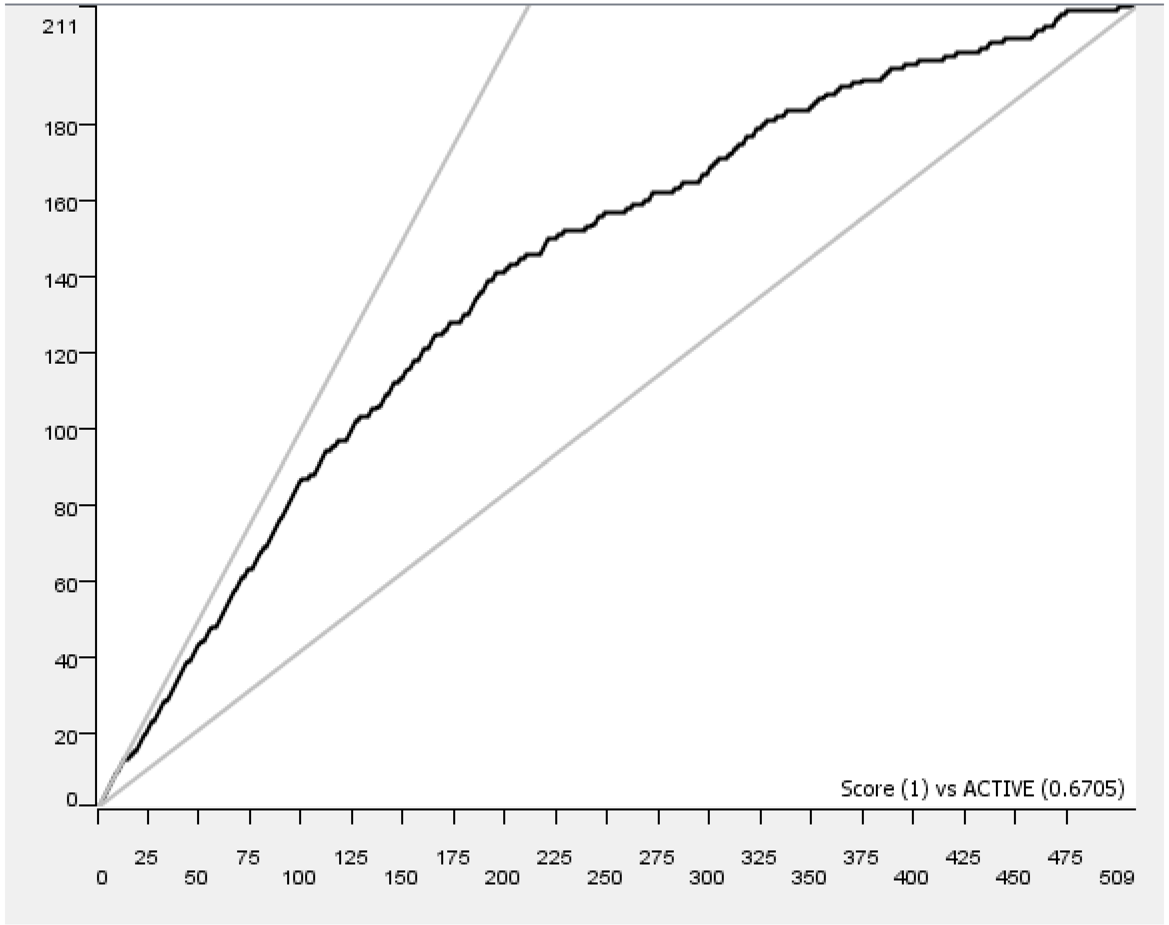

N=509

**Top 10% Mean IC50**	**15.2 nM**

	**Predicted Active**	**Predicted Inactive**
Active	135	76
Inactive	52	246

<15nM DefGood in HIV Protease, 45% error; Random seed = 121783

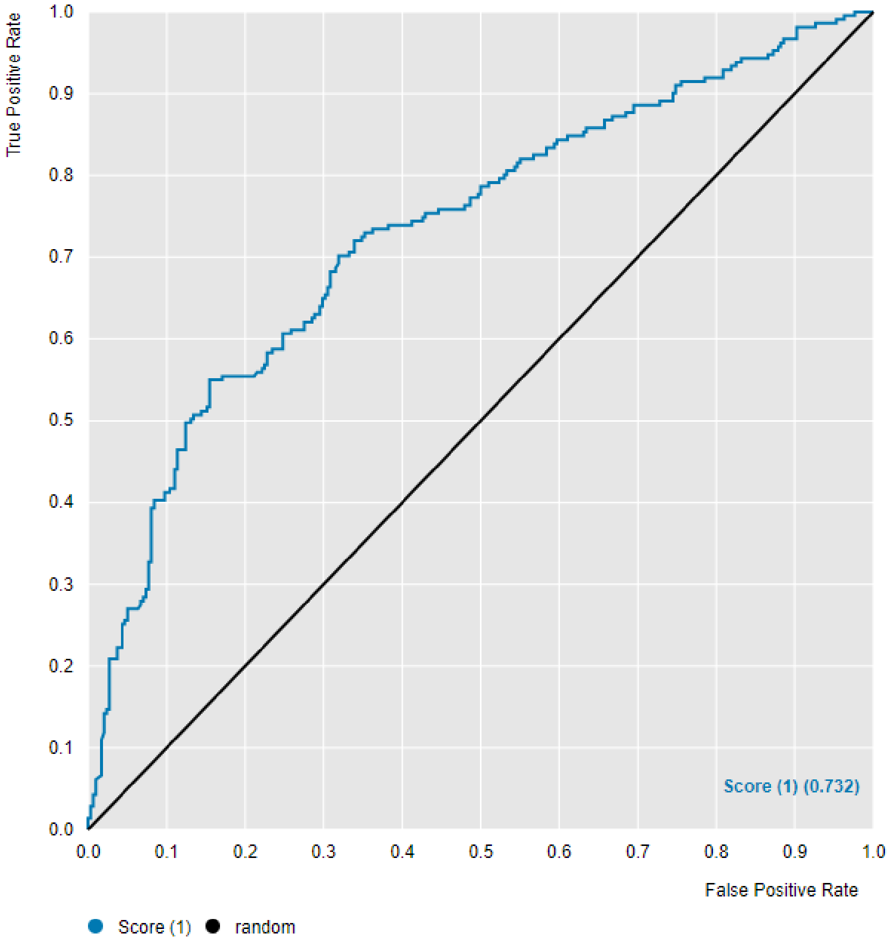

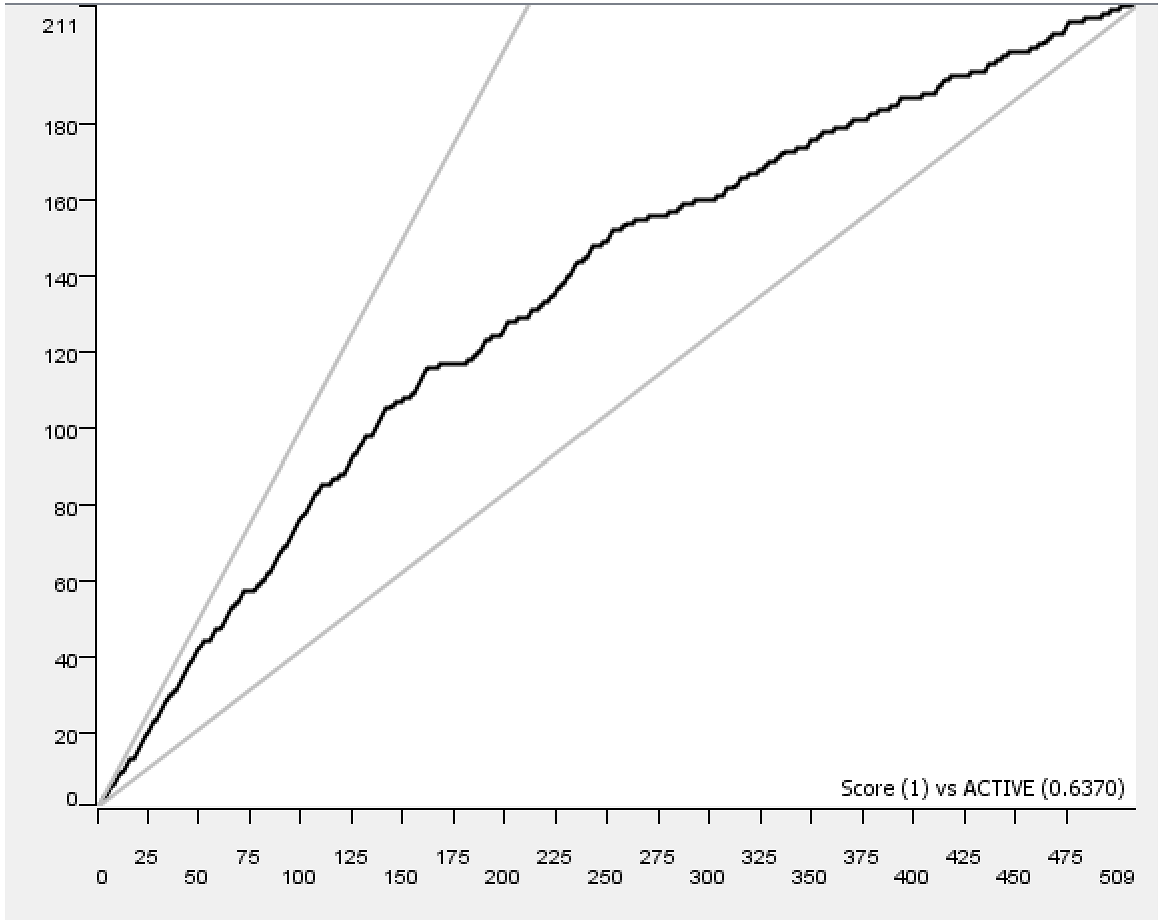

N=509

**Top 10% Mean IC50**	**16.5 nM**

	**Predicted Active**	**Predicted Inactive**
Active	152	59
Inactive	104	194

<15nM DefGood in HIV Protease, 50% error; Random seed = 121783

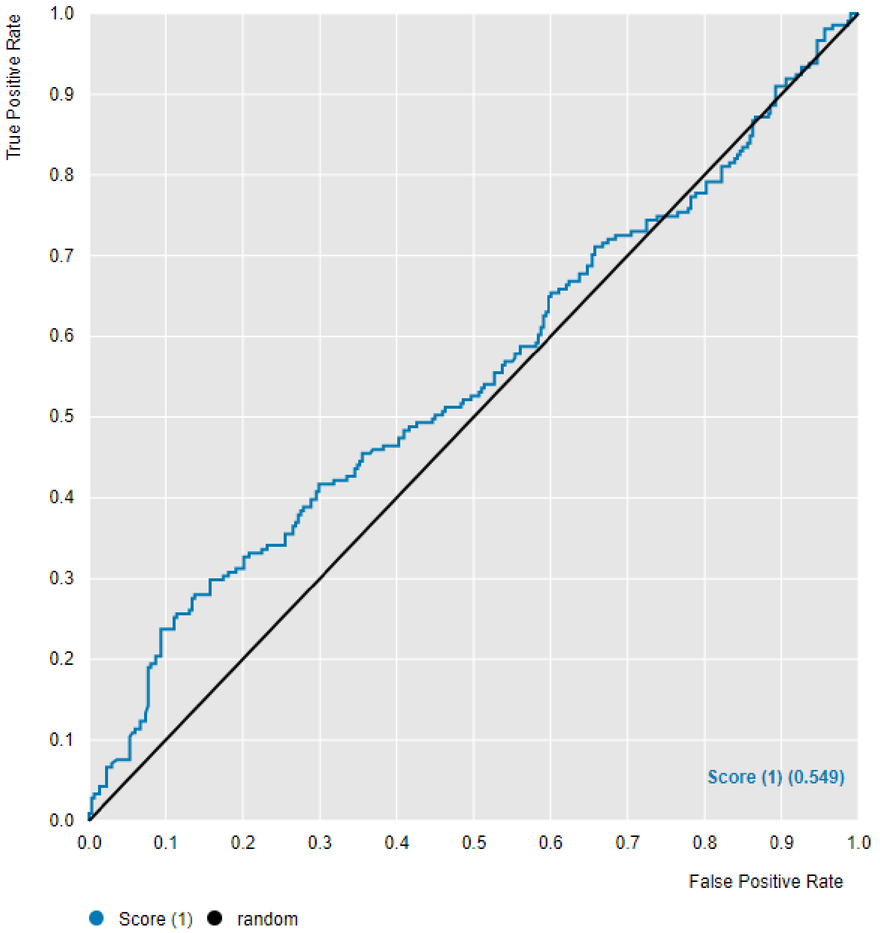

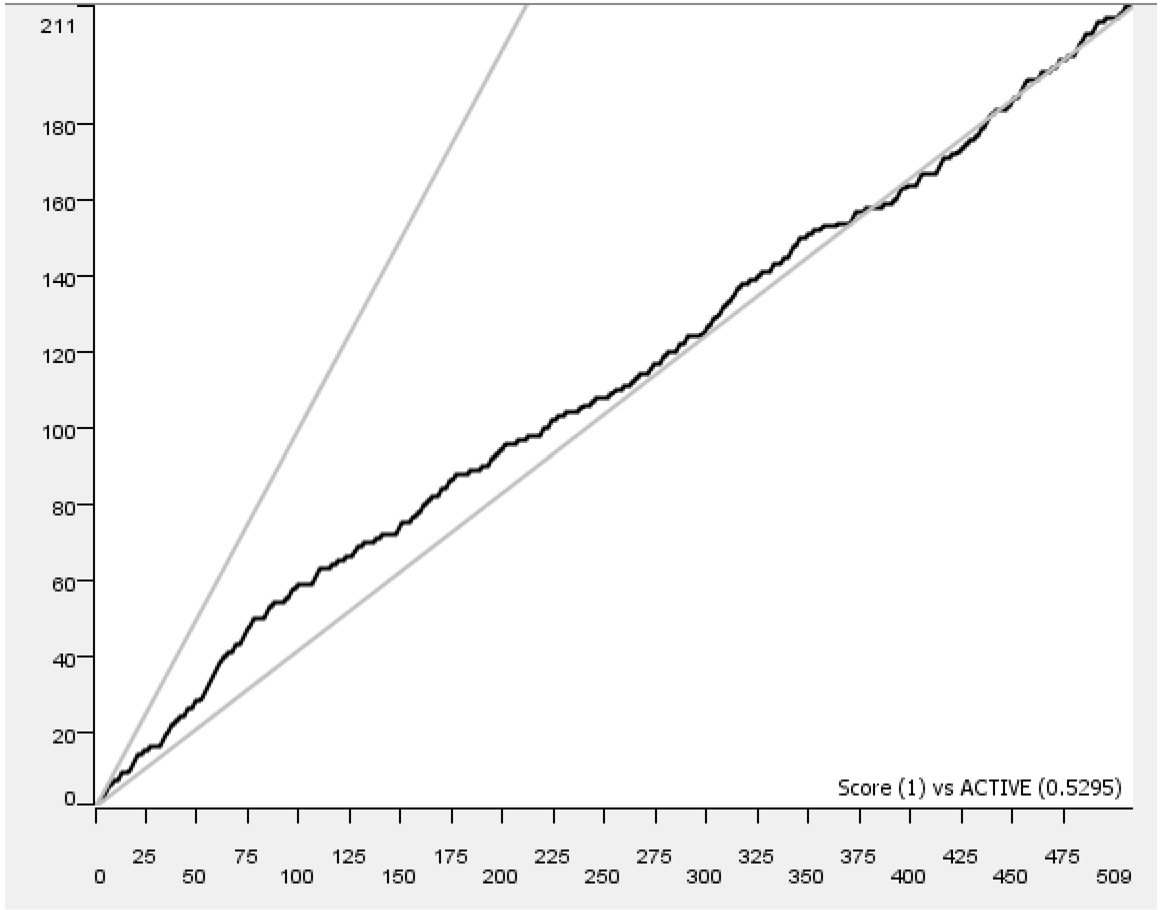

N=509

**Top 10% Mean IC50**	**12,000 nM**

	**Predicted Active**	**Predicted Inactive**
Active	194	17
Inactive	274	24

Conclusion - NBN

- An NBN could be generated for HIV Protease with a decision value of <15 nM as defined as active. The predictivity of the algorithm could be preserved in three random train/test cycles. The point of failure was 50%, 45% and 50% error.

RF Error Tolerance- <15 nM DefGood in HIV Protease

- 0-50% absolute error

RF- <15nM DefGood in HIV Protease, 10% error

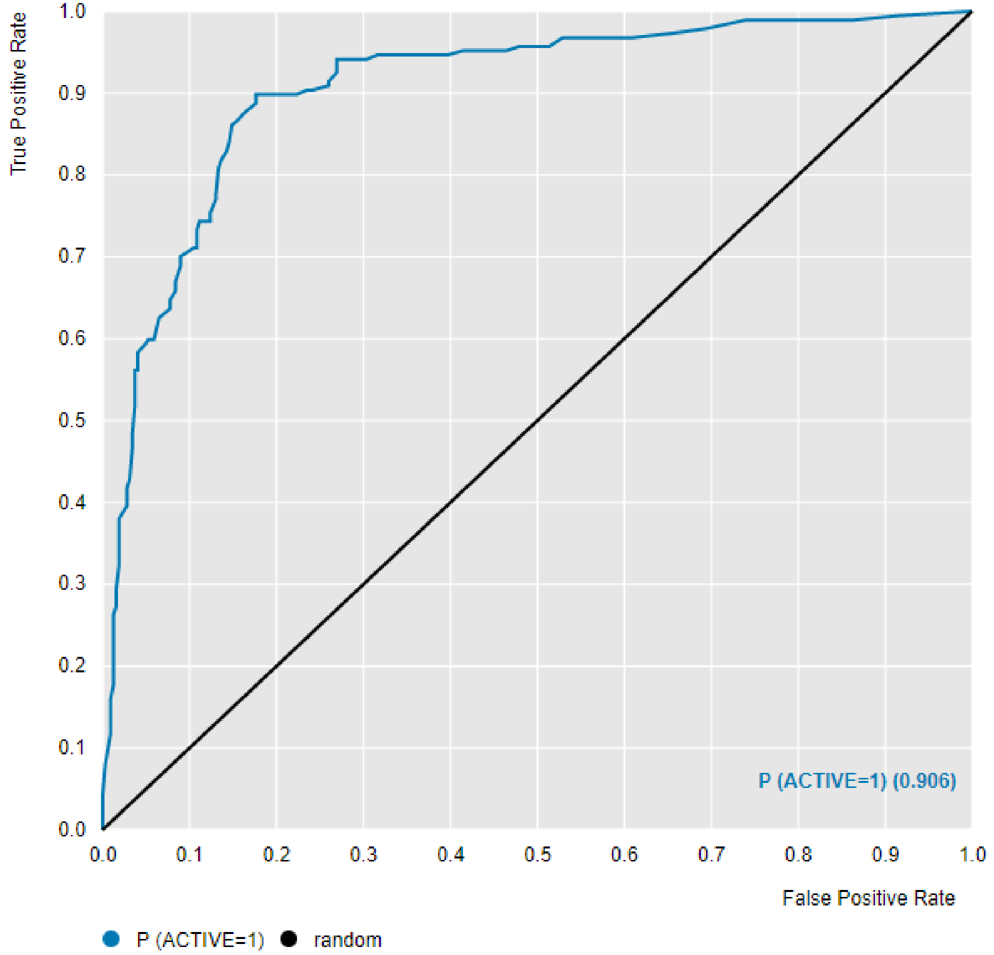

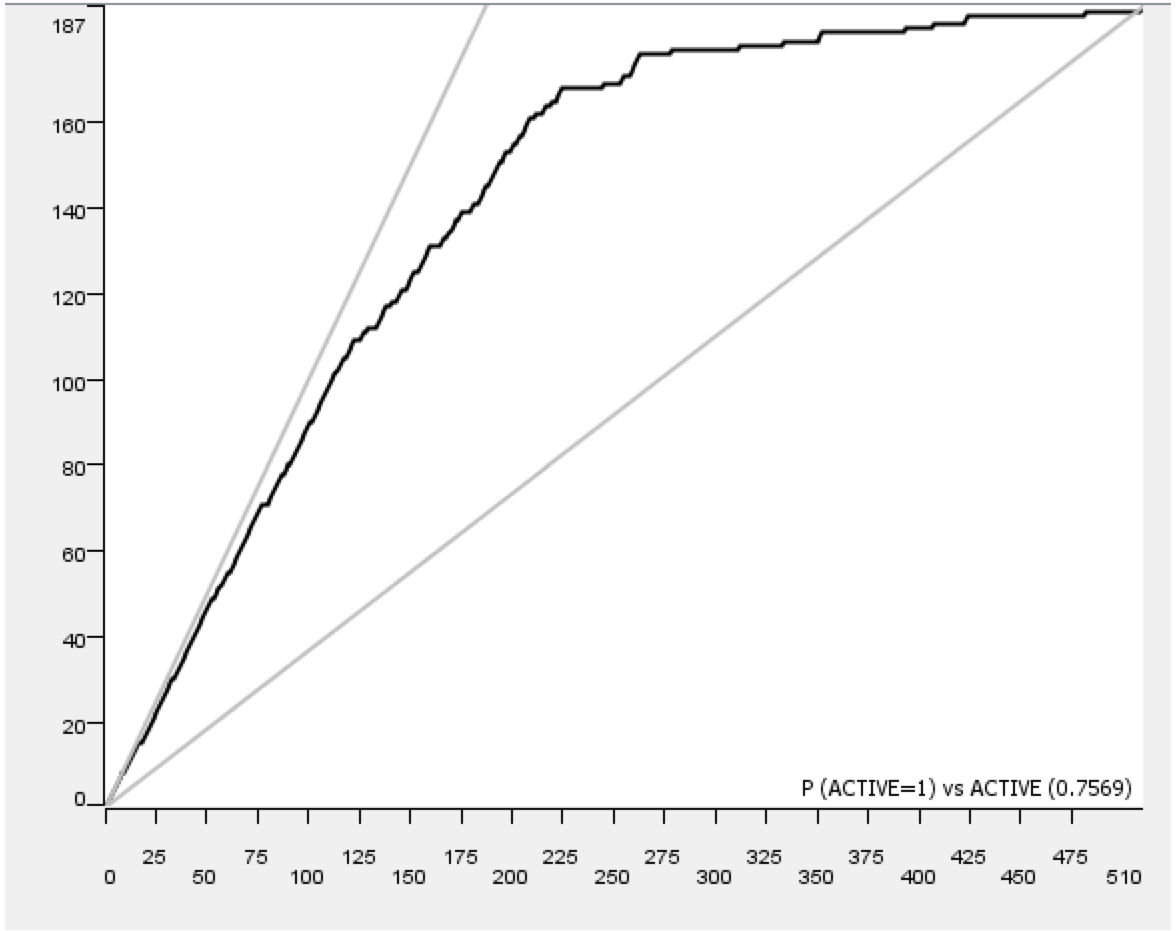

N=510

Top 10% Mean IC50	12.7 nM

	Predicted Active	Predicted Inactive
Active	139	48
Inactive	39	284

RF- <15nM DefGood in HIV Protease, 20% error

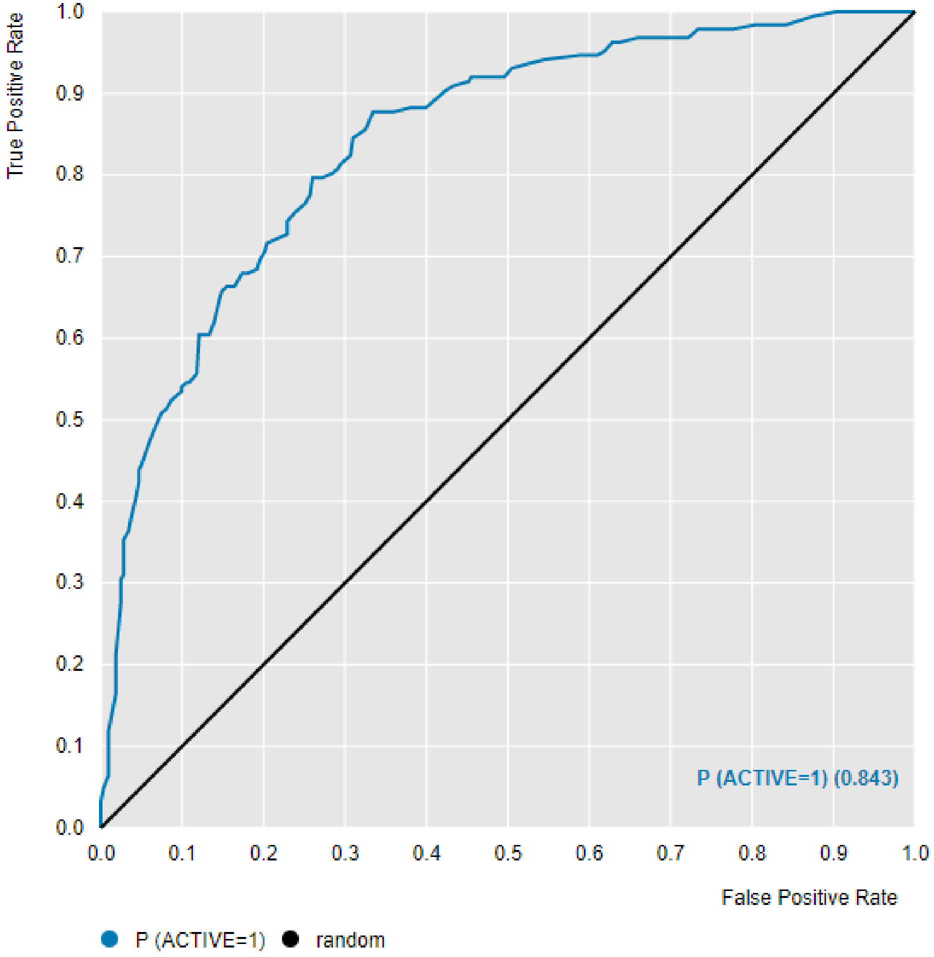

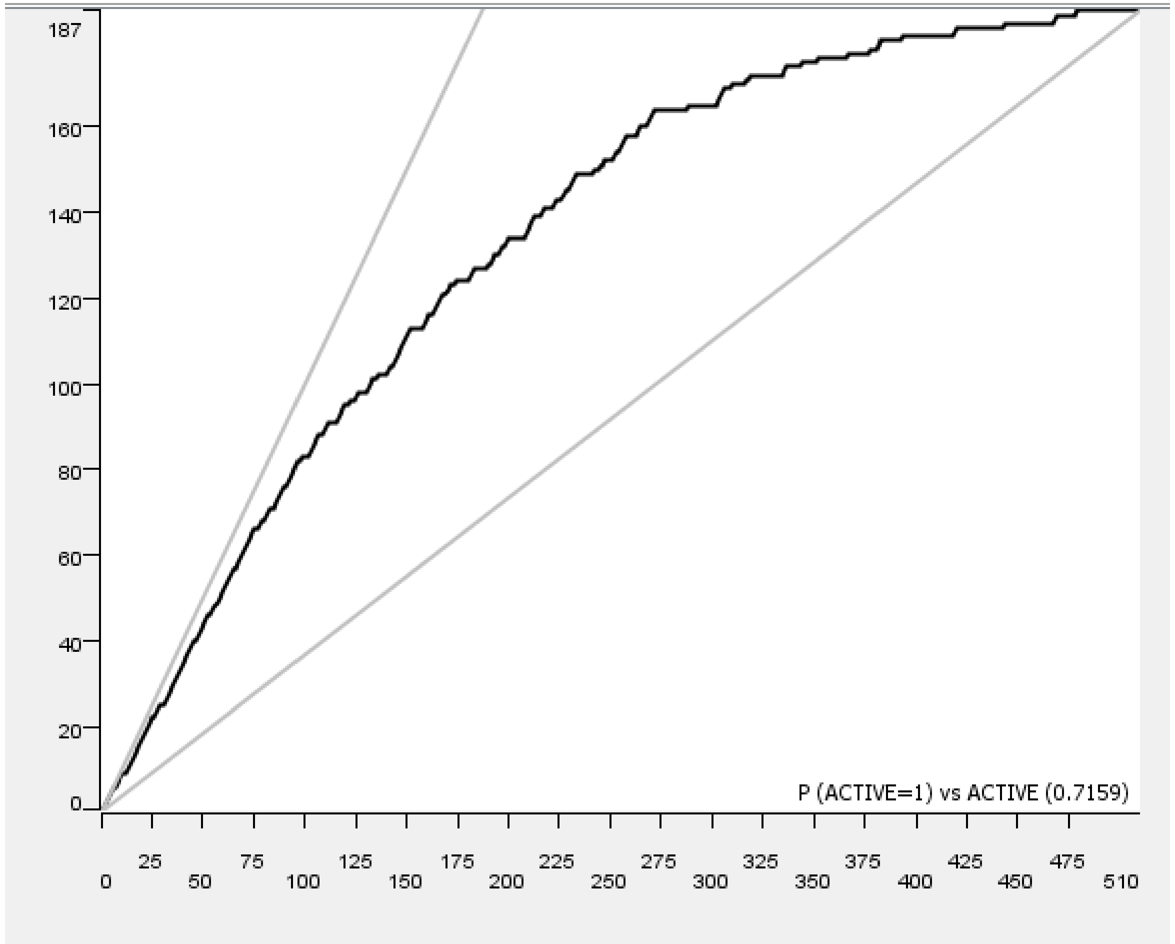

N=510

**Top 10% Mean IC50**	**14.7 nM**

	**Predicted Active**	**Predicted Inactive**
Active	130	57
Inactive	63	260

RF- <15nM DefGood in HIV Protease, 30% error

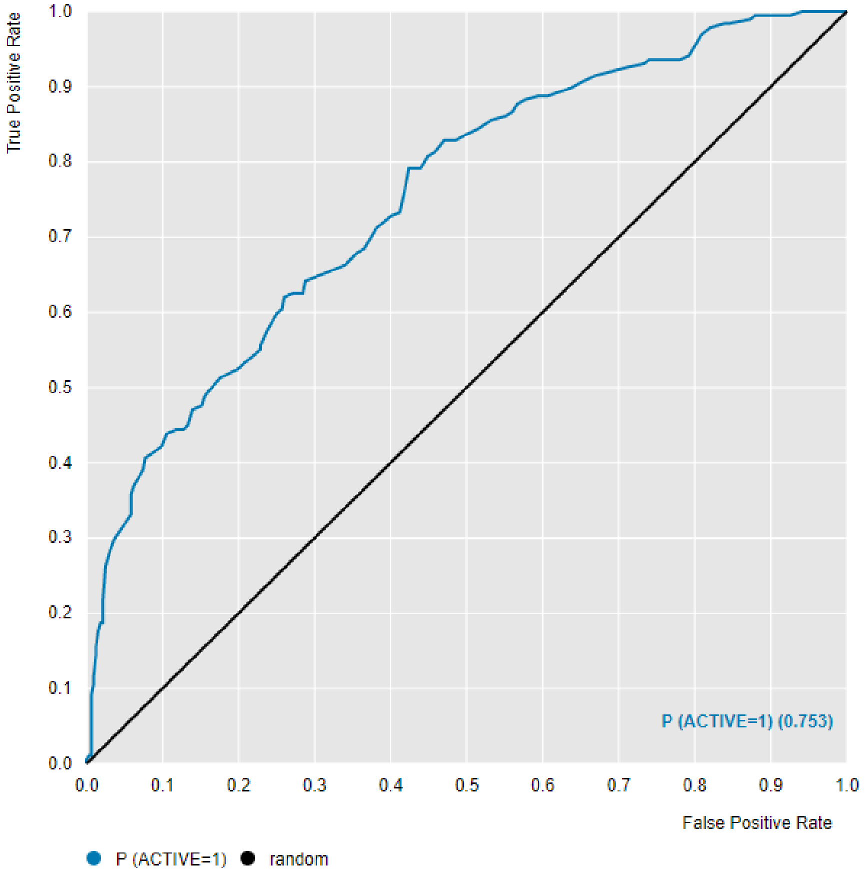

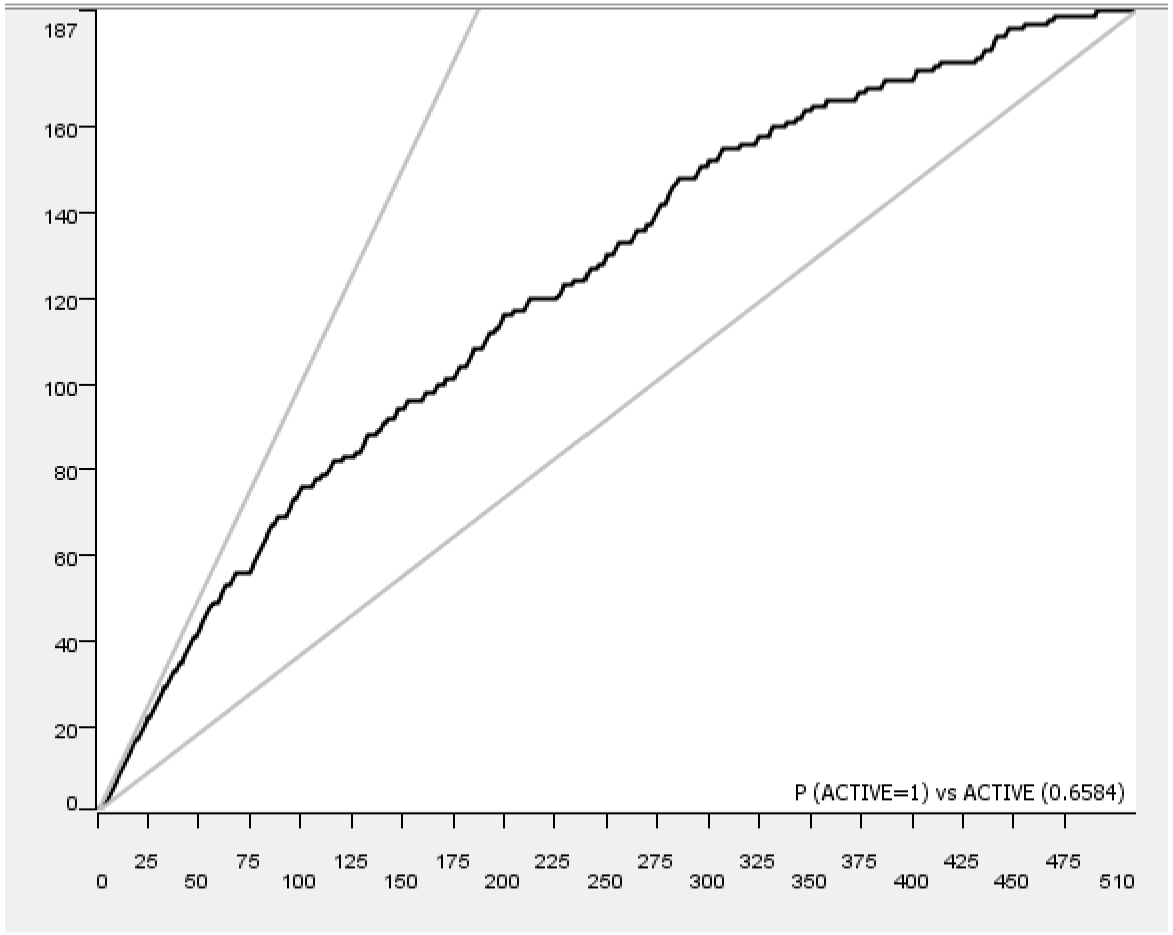

N=510

**Top 10% Mean IC50**	**10.2 nM**

	**Predicted Active**	**Predicted Inactive**
Active	116	71
Inactive	84	239

RF- <15nM DefGood in HIV Protease, 35% error

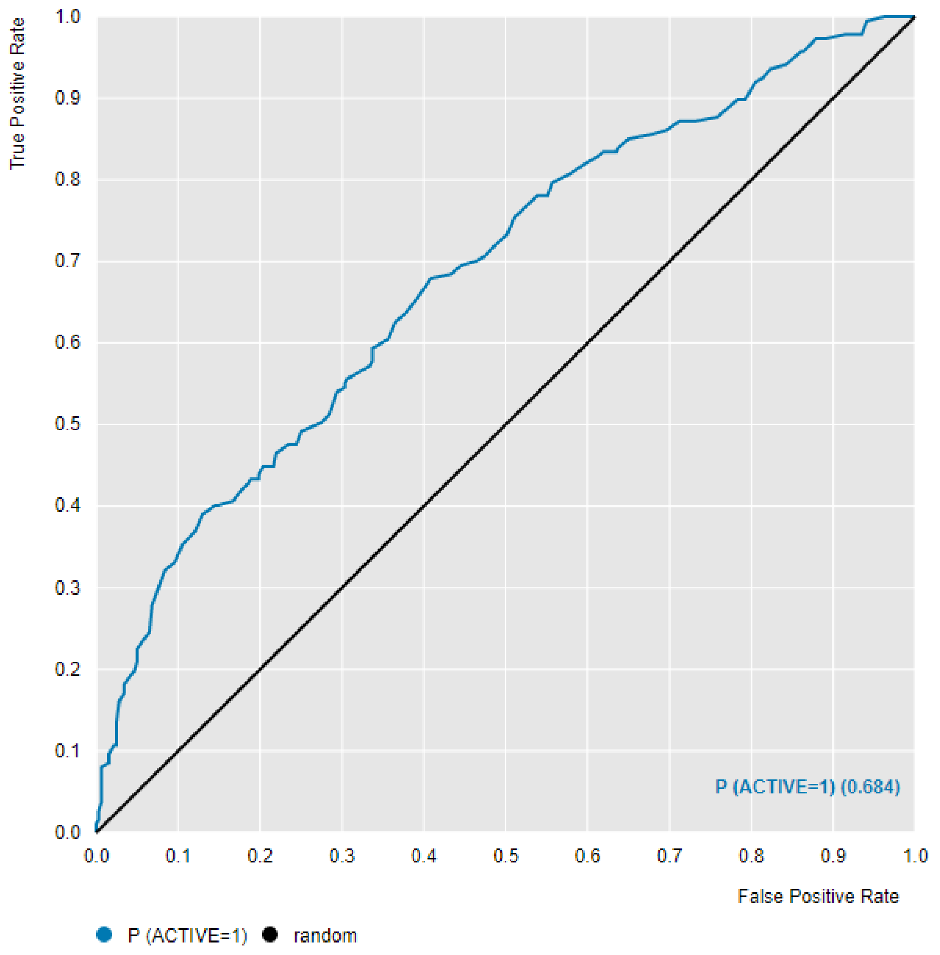

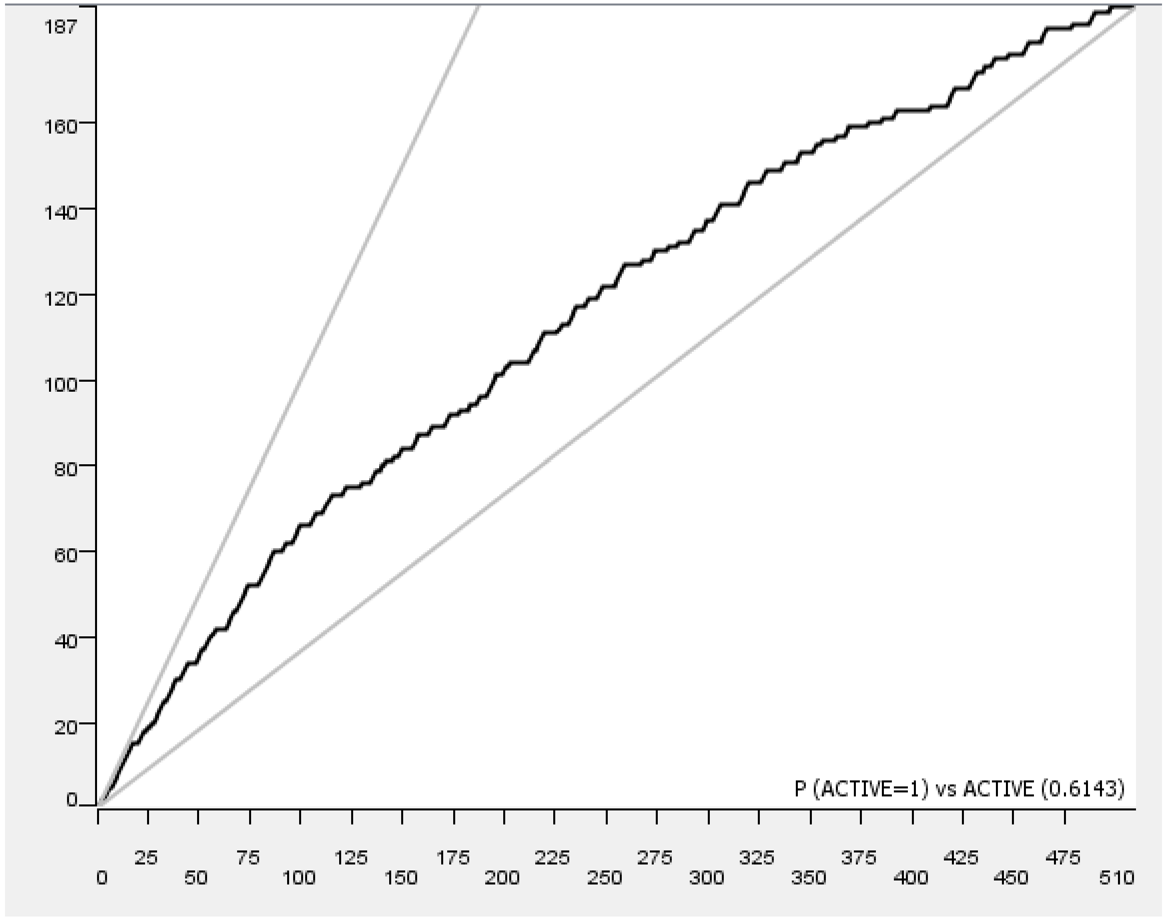

N=510

Top 10% Mean IC50	3,700 nM

	Predicted Active	Predicted Inactive
Active	103	84
Inactive	98	225

<15nM DefGood in HIV Protease, 10% error; Random seed = 429

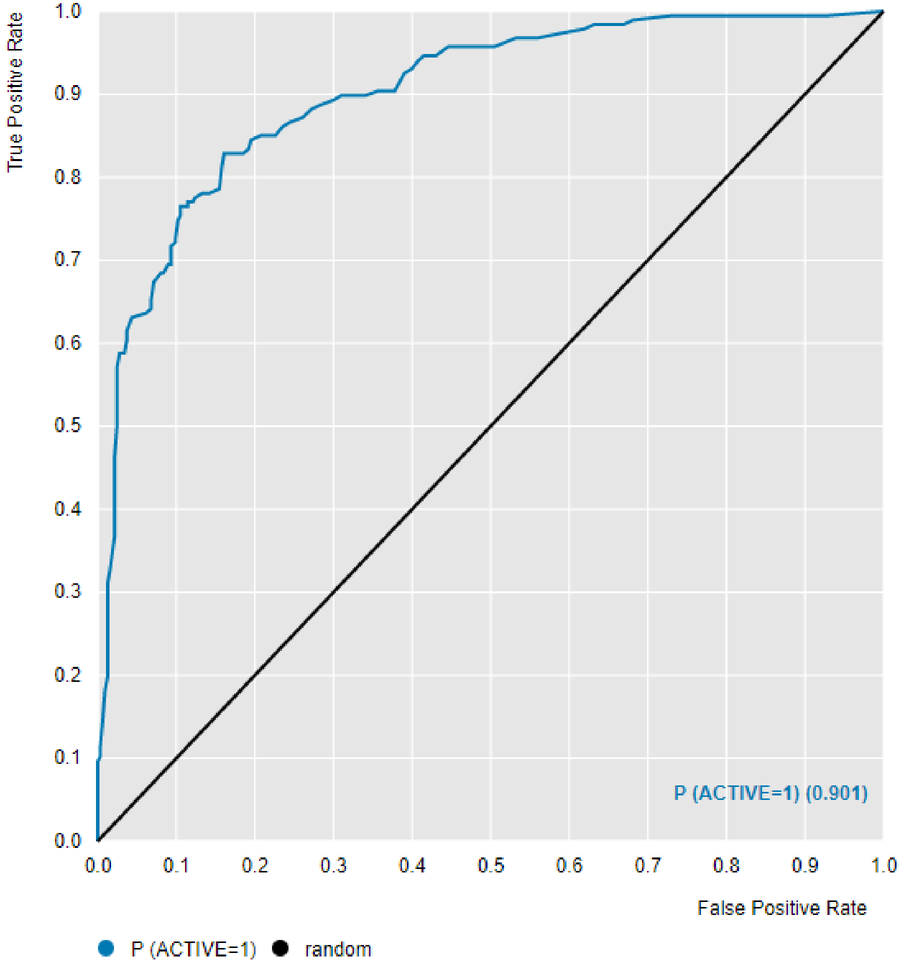

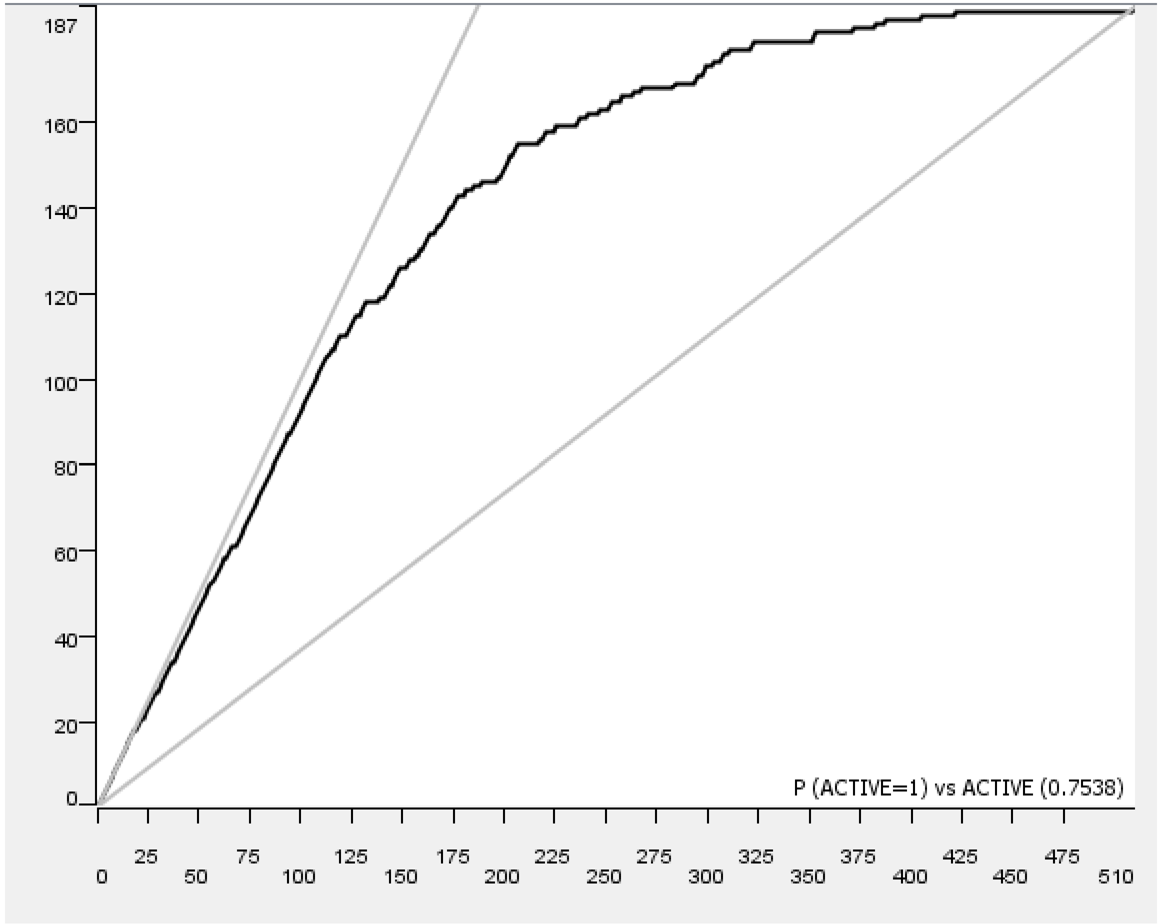

N=510

**Top 10% Mean IC50**	**10.2 nM**

	Predicted Active	Predicted Inactive
Active	143	44
Inactive	37	286

<15nM DefGood in HIV Protease, 15% error; Random seed = 429

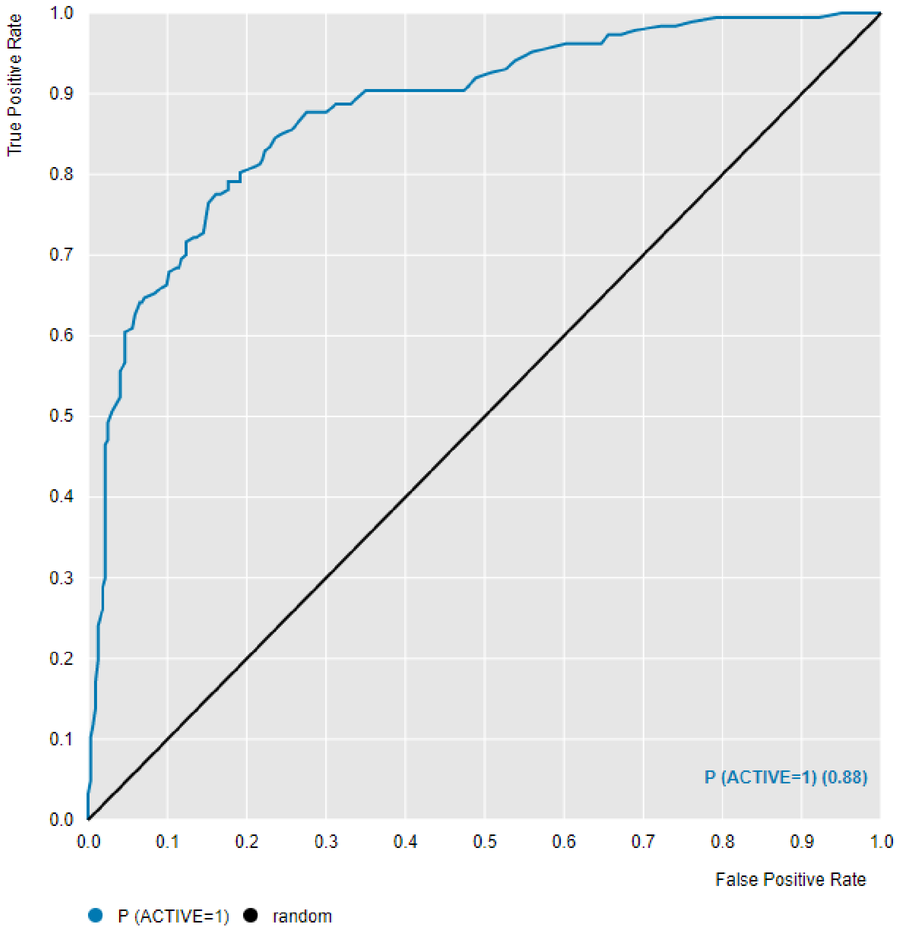

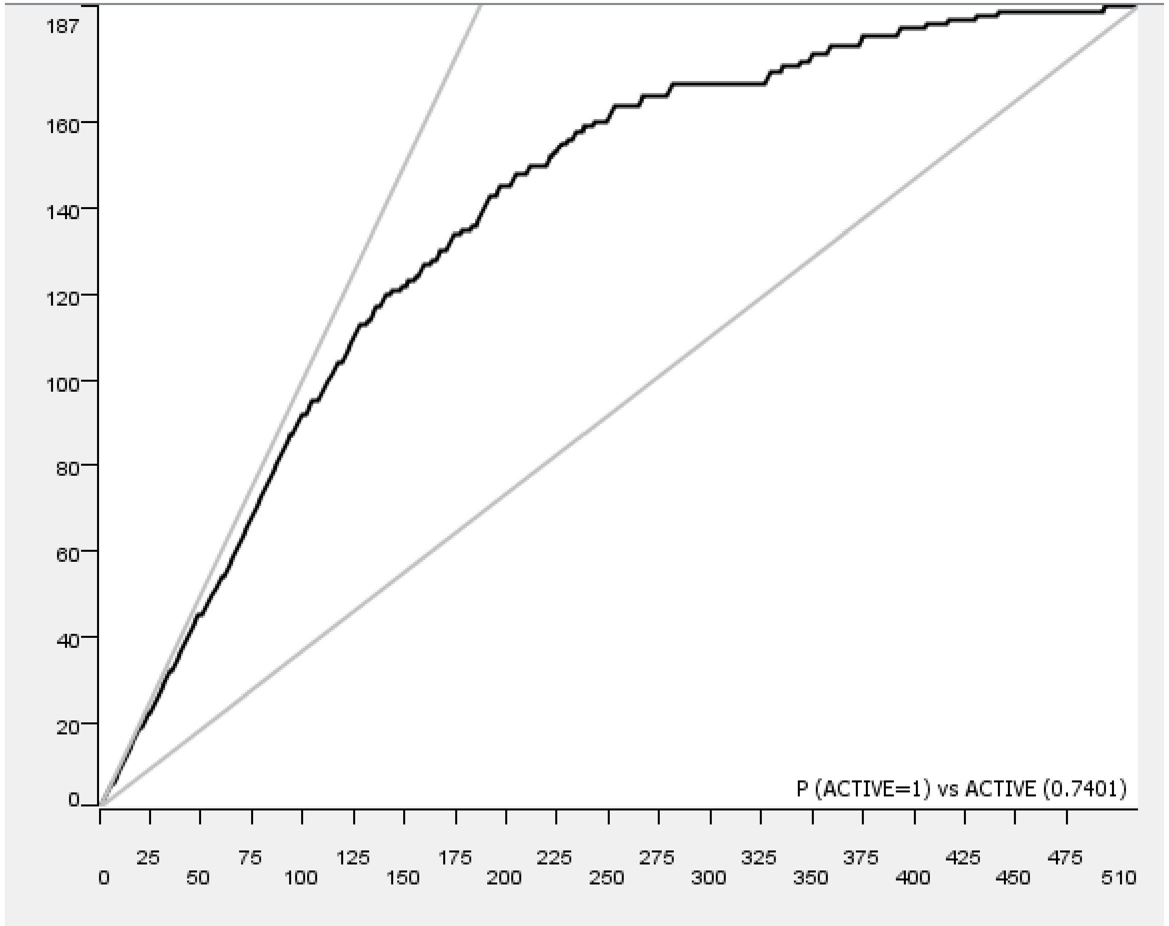

N=510

Top 10% Mean IC50	10.3 nM

	Predicted Active	Predicted Inactive
Active	135	52
Inactive	44	279

<15nM DefGood in HIV Protease, 20% error; Random seed = 429

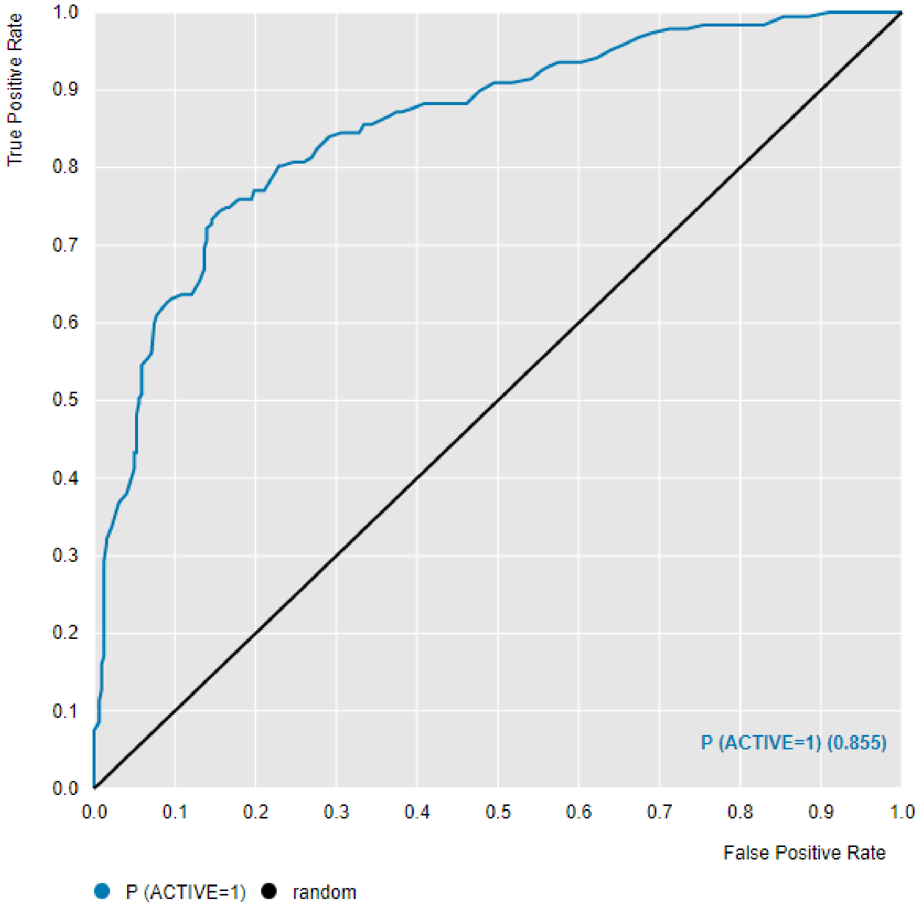

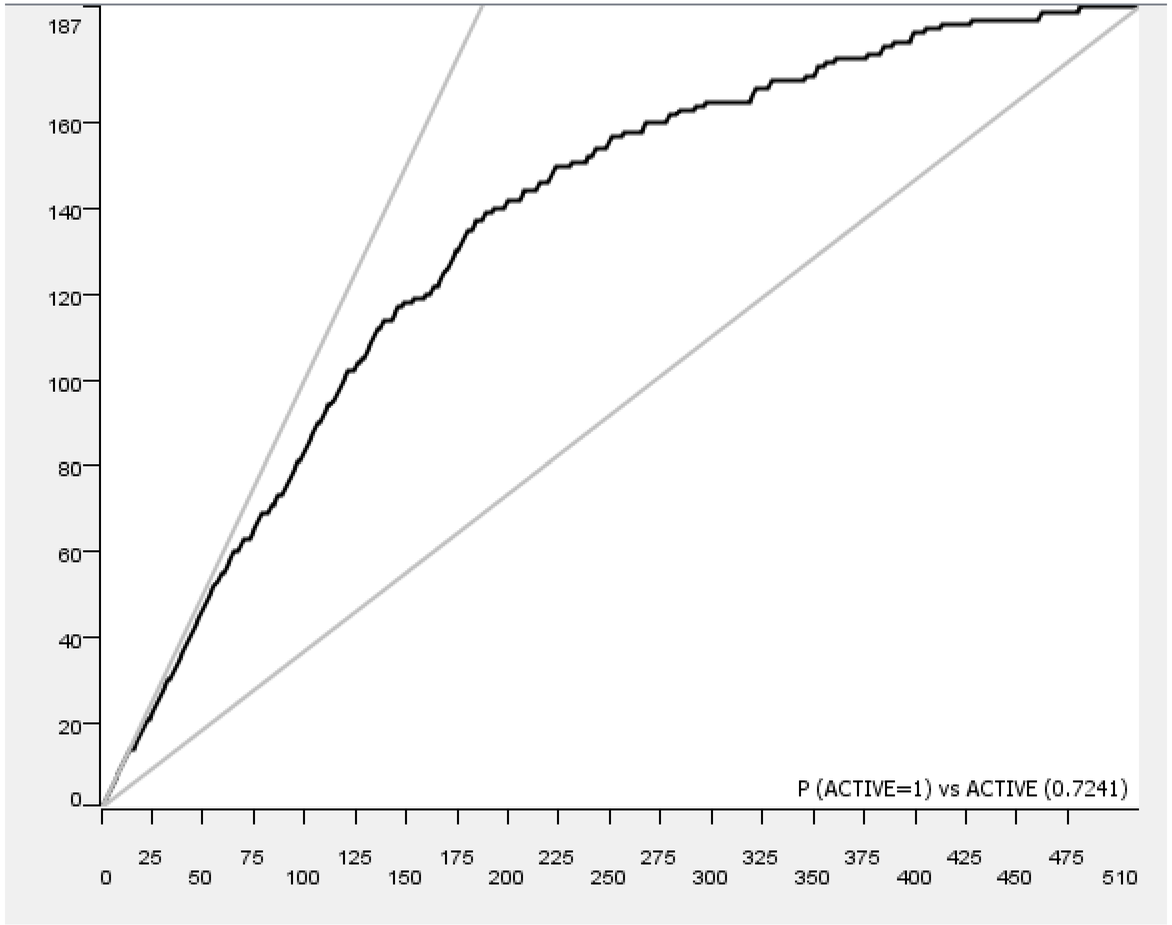

N=510

**Top 10% Mean IC50**	**941 nM<br>One 47,000 nM mistake**

	Predicted Active	Predicted Inactive
Active	139	48
Inactive	50	273

<15nM DefGood in HIV Protease, 10% error; Random seed = 121783

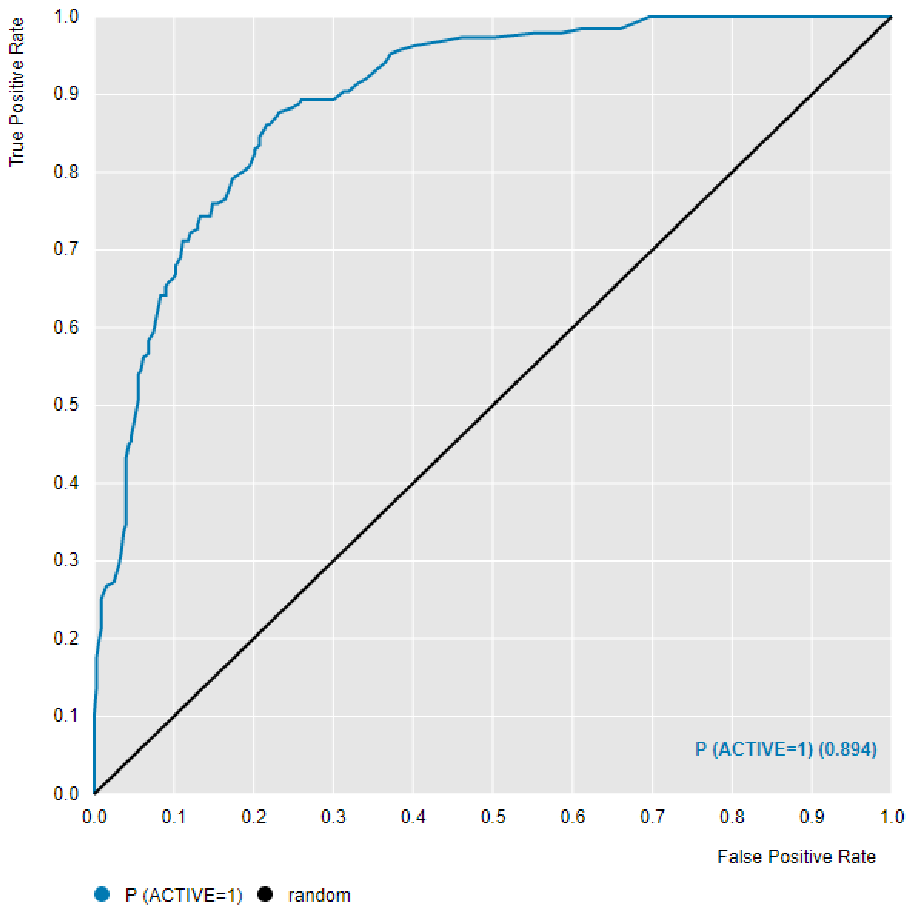

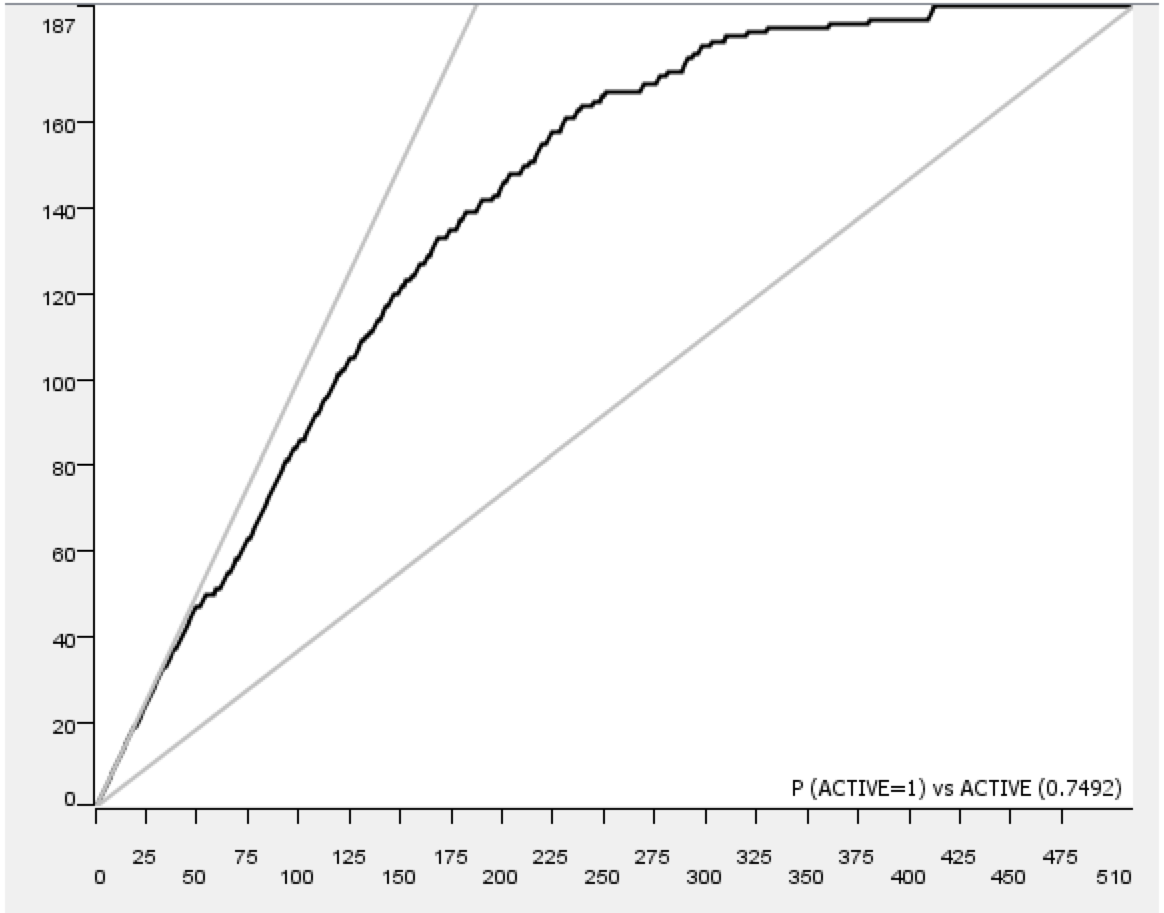

N=510

Top 10% Mean IC50	17.9 nM

	Predicted Active	Predicted Inactive
Active	133	54
Inactive	36	287

<15nM DefGood in HIV Protease, 15% error; Random seed = 121783

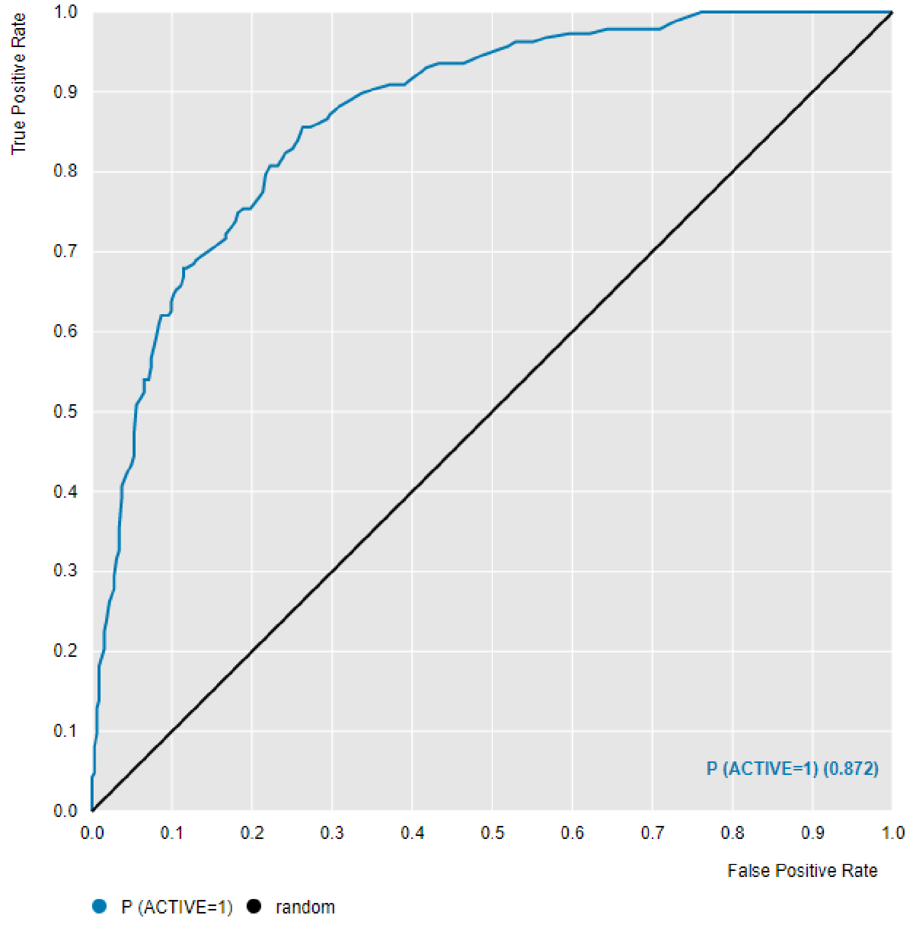

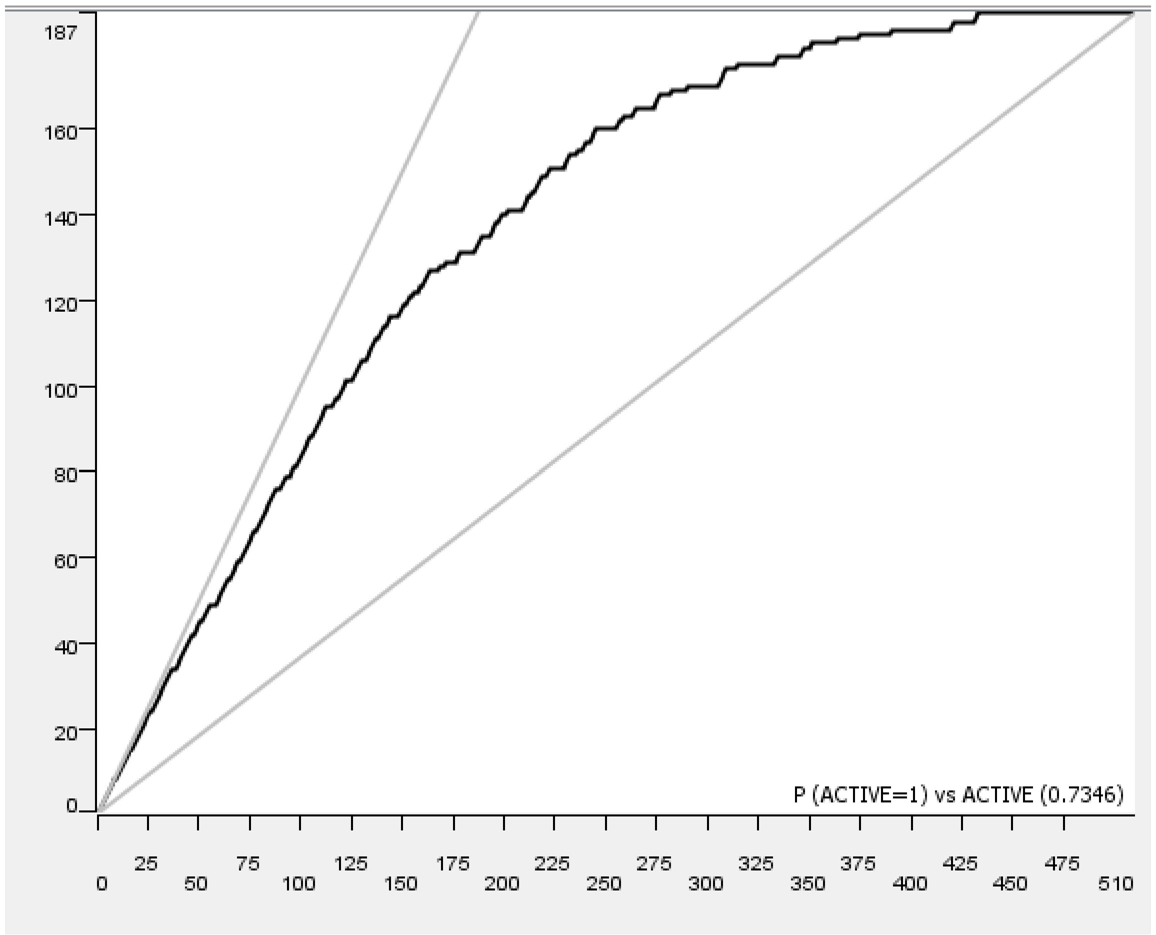

N=510

Top 10% Mean IC50	1,100 nM

	Predicted Active	Predicted Inactive
Active	127	60
Inactive	37	286

Conclusion - RF

- A Random Forrest could be generated for HIV Protease with a decision value of <15 nM as defined as active. The point of failure was 35%, 20% and 15% error.

PNN Error Tolerance- <15 nM DefGood in HIV Protease

- 0-50% absolute error

PNN- <15nM DefGood in HIV Protease, 10% error

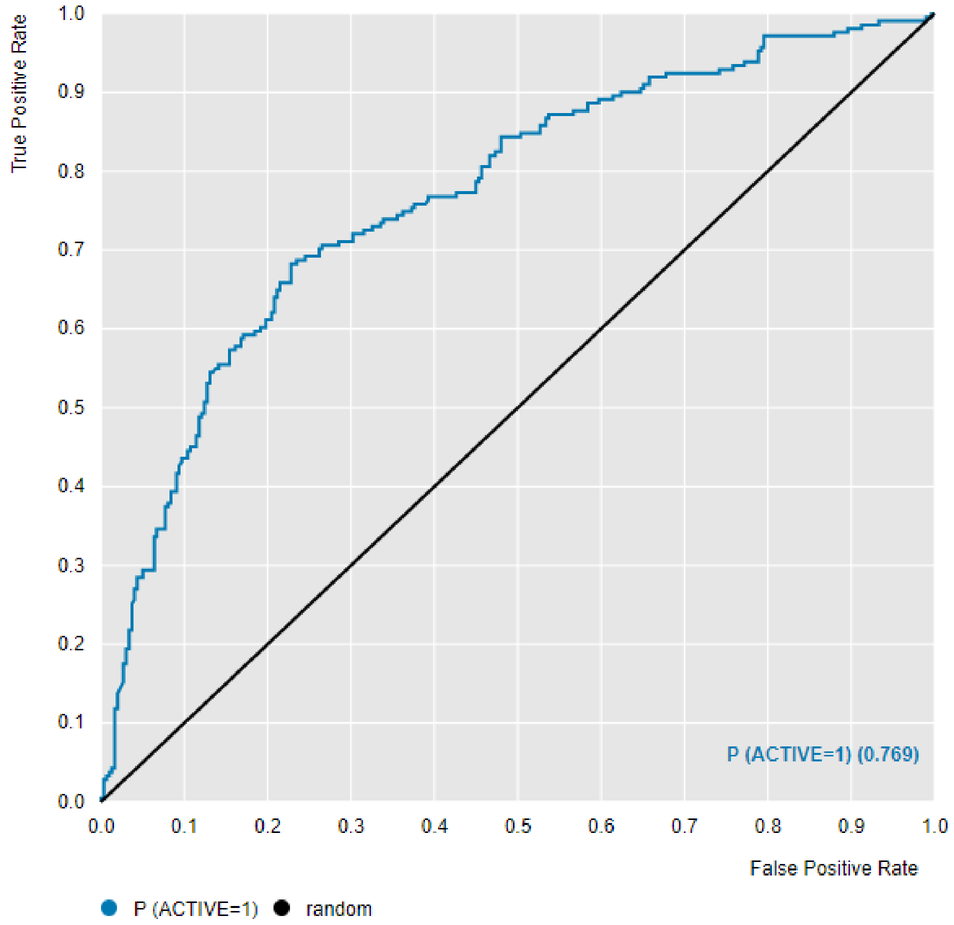

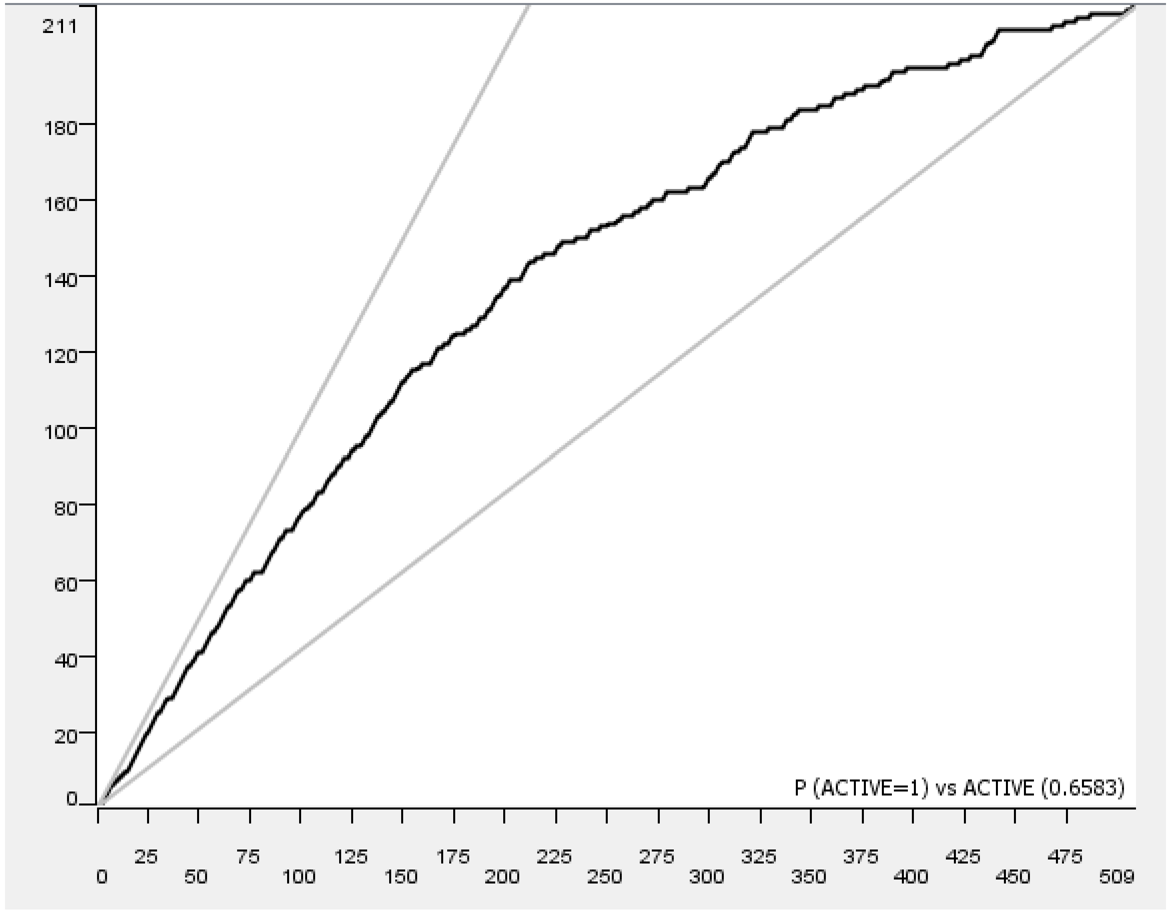

N=509

**Top 10% Mean IC50**	**111 nM**

	Predicted Active	Predicted Inactive
Active	128	83
Inactive	59	239

PNN- <15nM DefGood in HIV Protease, 20% error

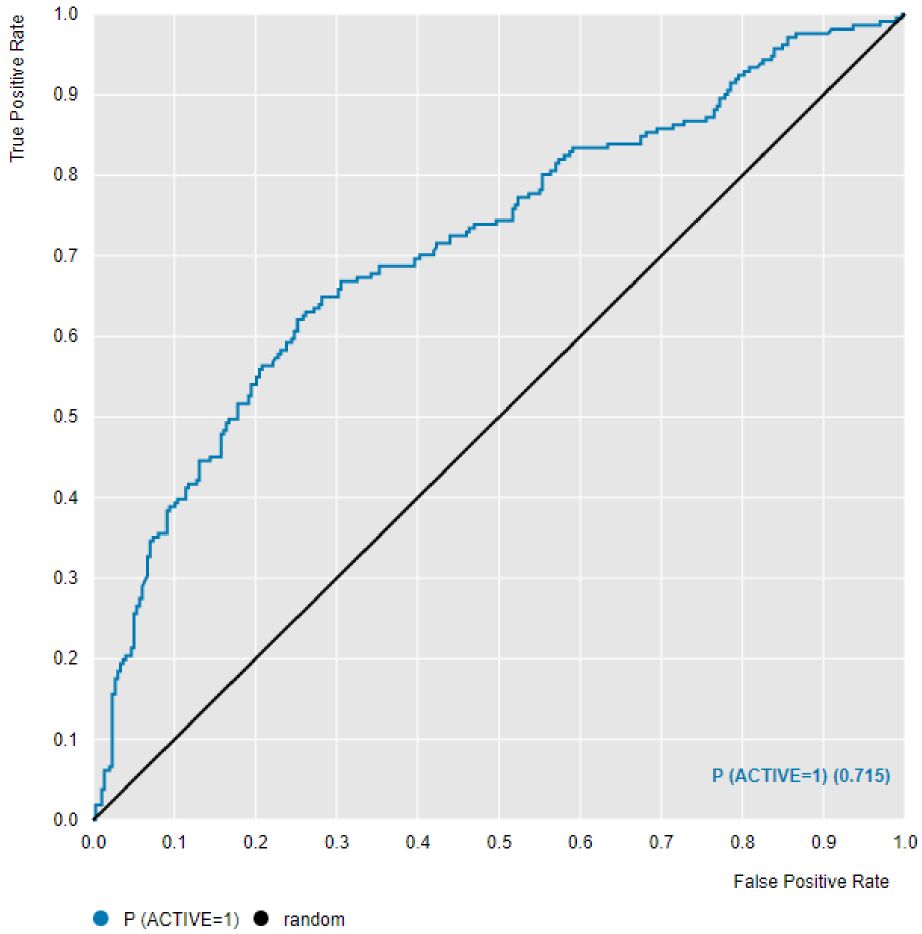

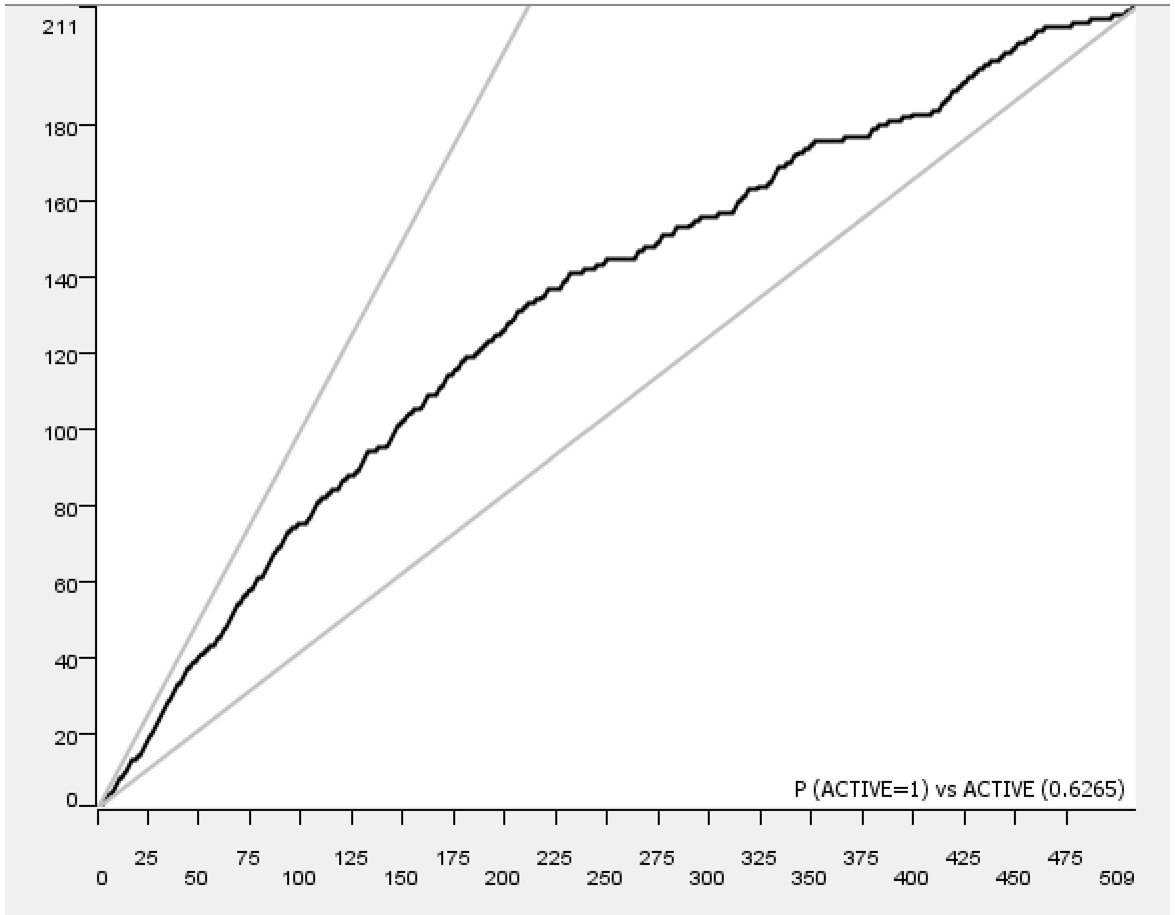

N=509

Top 10% Mean IC50	114 nM

	Predicted Active	Predicted Inactive
Active	139	72
Inactive	91	207

PNN- <15nM DefGood in HIV Protease, 25% error

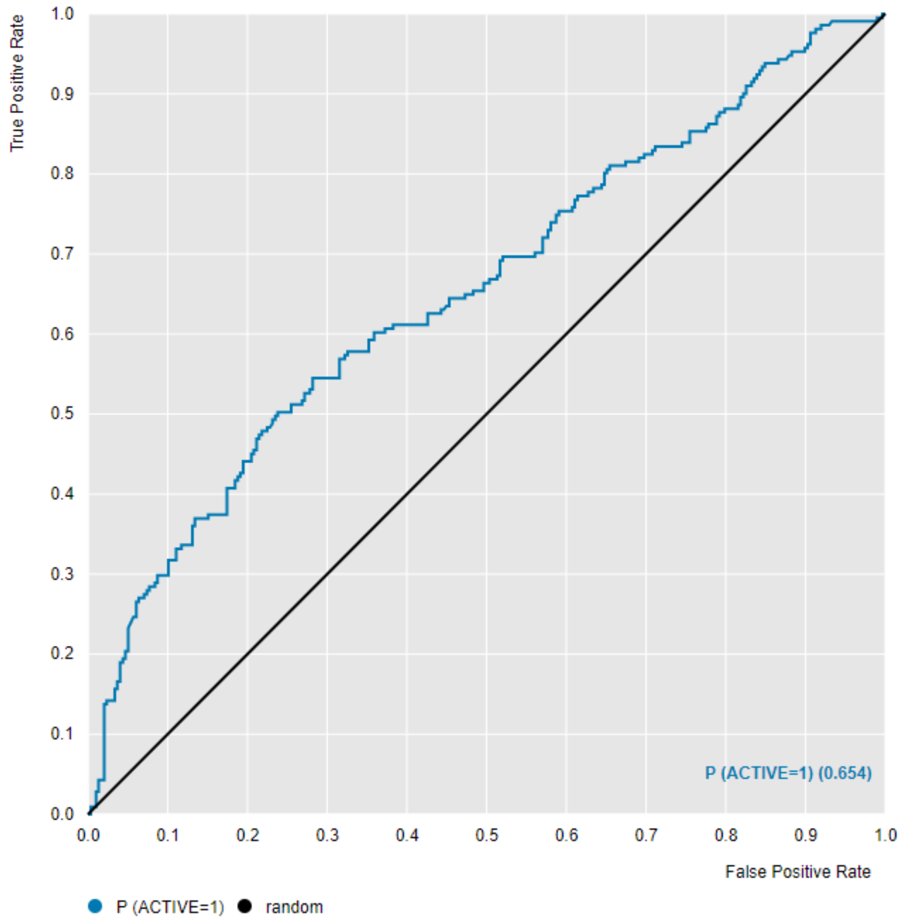

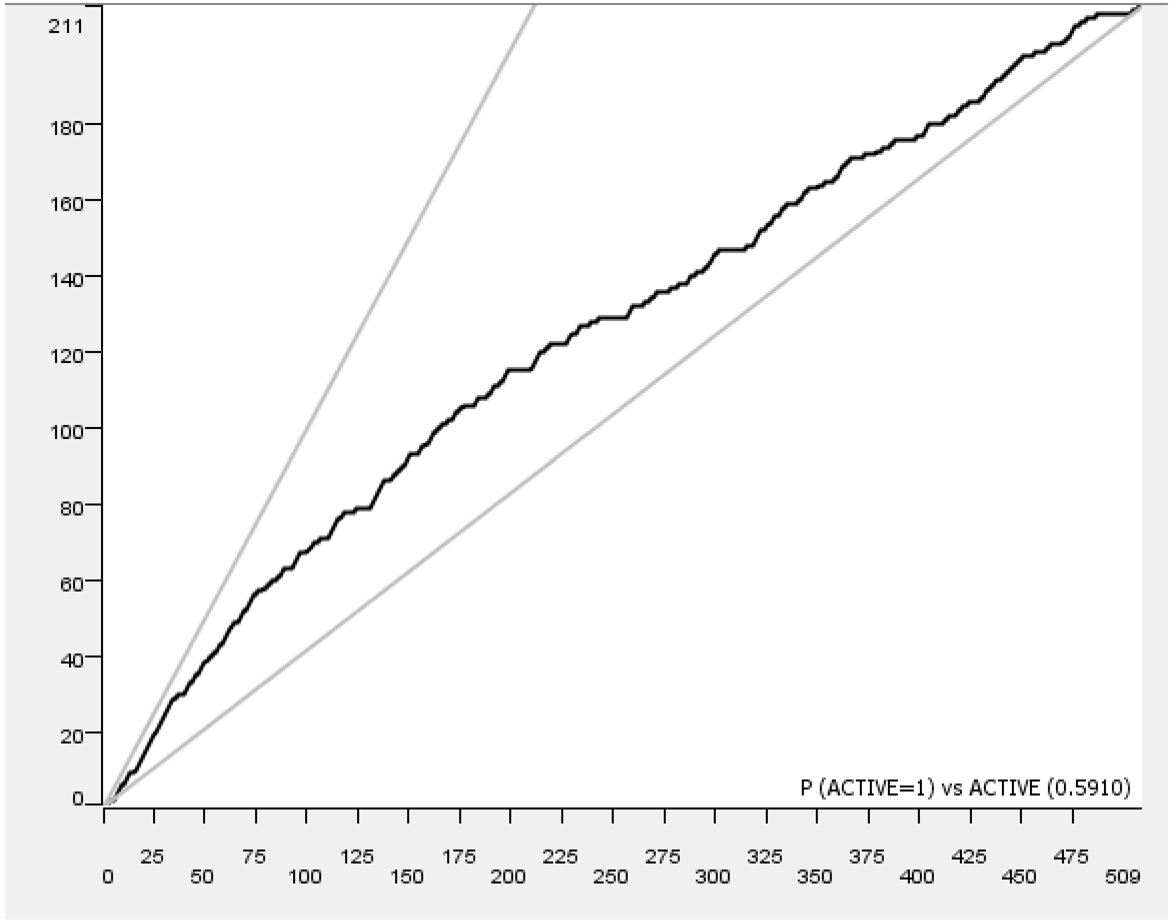

N=509

**Top 10% Mean IC50**	**217 nM**

	Predicted Active	Predicted Inactive
Active	120	91
Inactive	96	202

PNN - <15nM DefGood in HIV Protease, 25% error; Random seed = 1515533876005 (reparameterized Theta minus = 0.55; theta plus = 0.75)

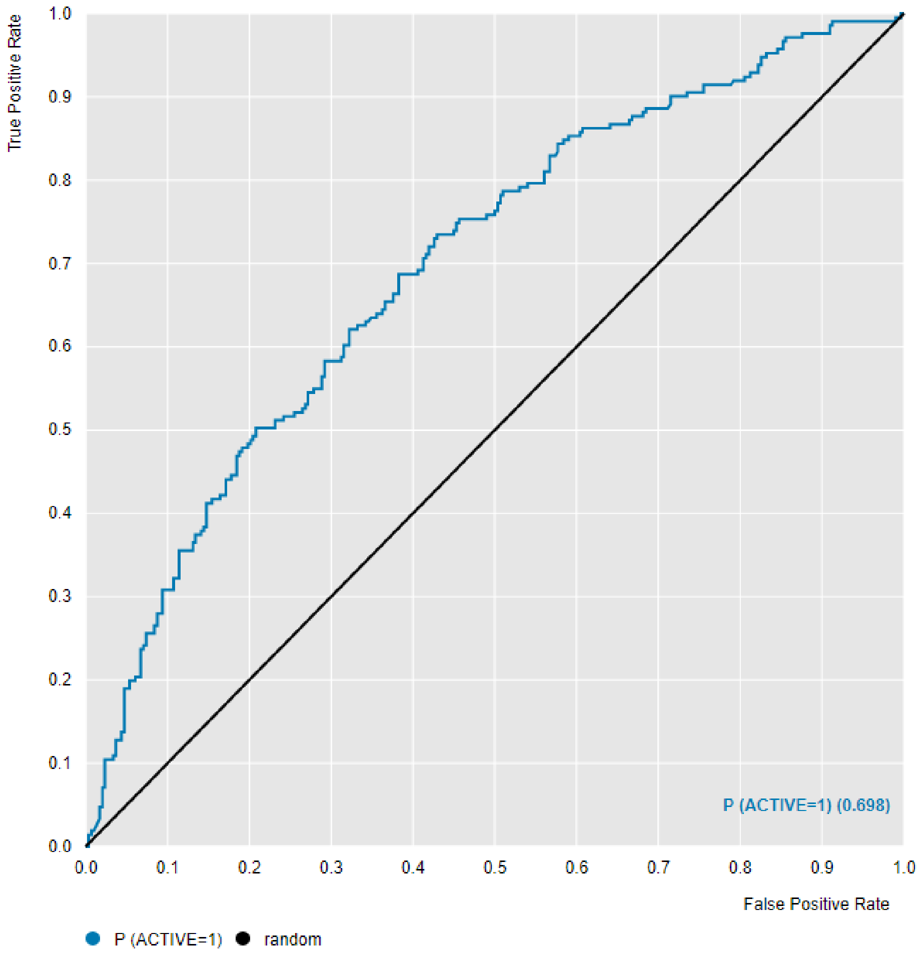

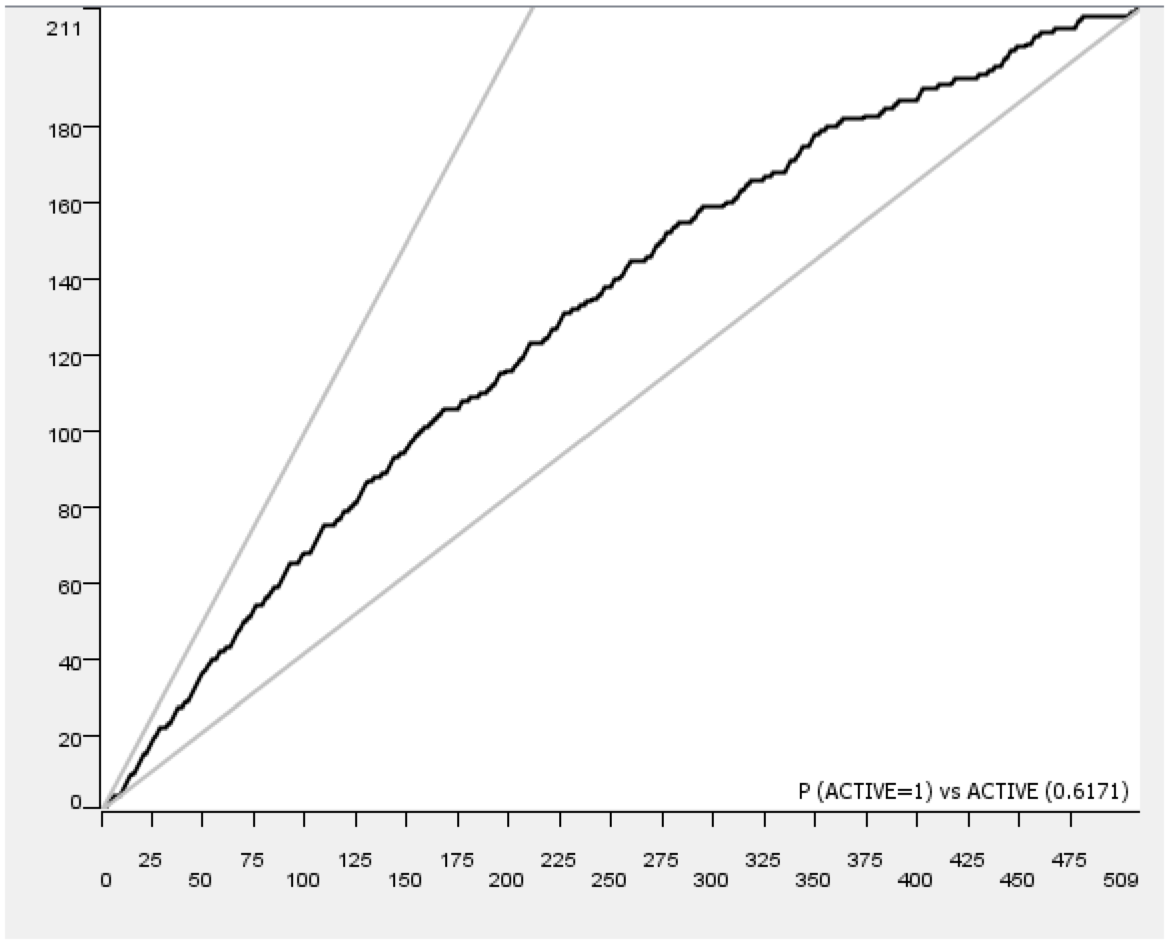

N=509

Top 10% Mean IC50	150 nM

	Predicted Active	Predicted Inactive
Active	84	127
Inactive	44	254

PNN- <15nM DefGood in HIV Protease, 10% error; Random seed = 429

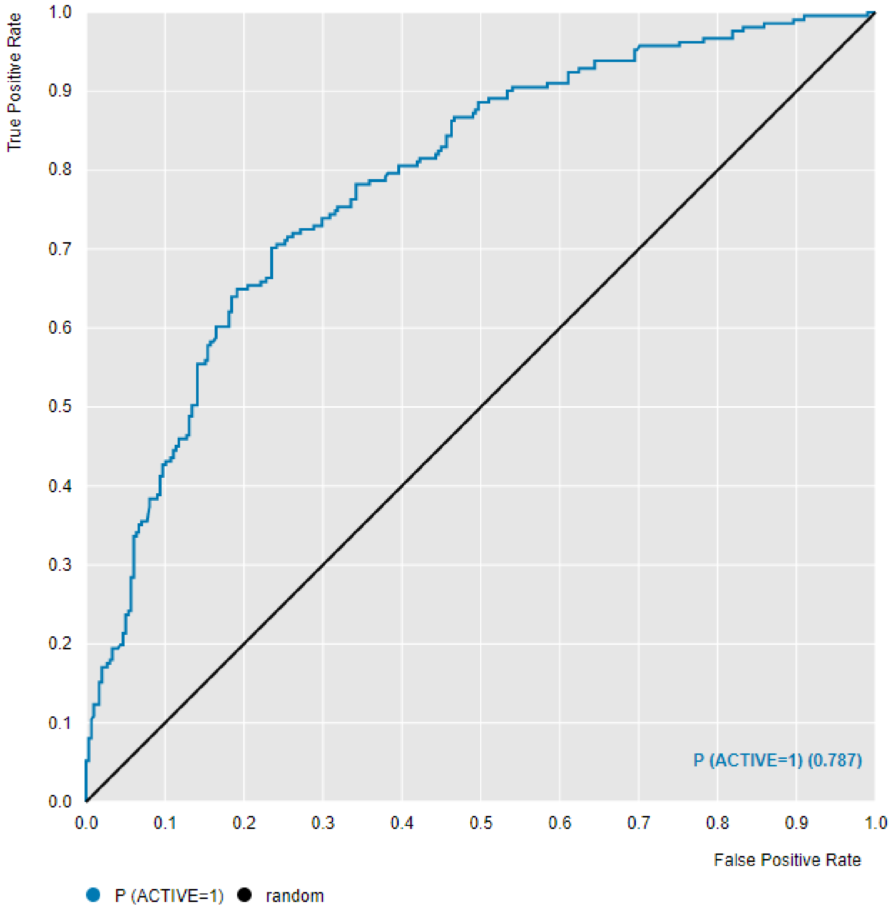

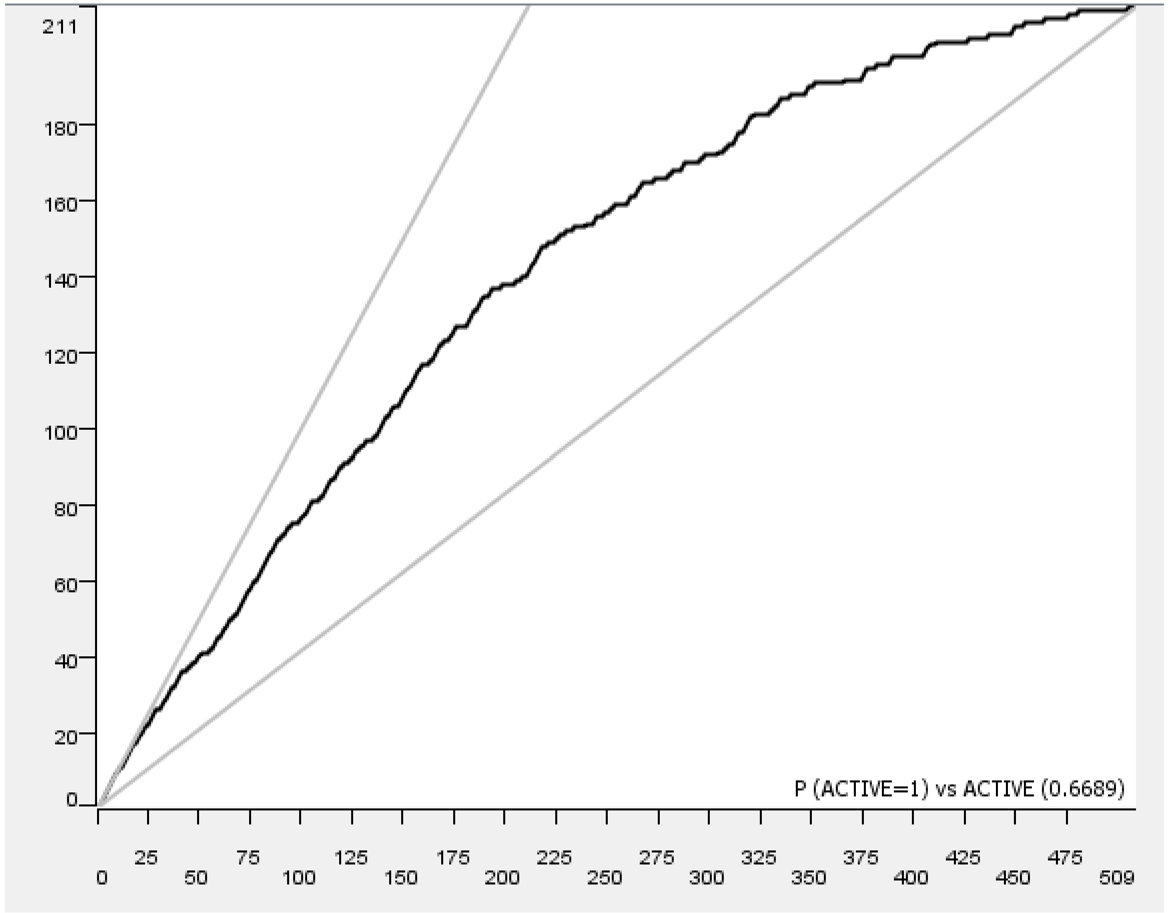

N=509

Top 10% Mean IC50	251 nM

	Predicted Active	Predicted Inactive
Active	123	88
Inactive	48	250

PNN- <15nM DefGood in HIV Protease, 15% error; Random seed = 429

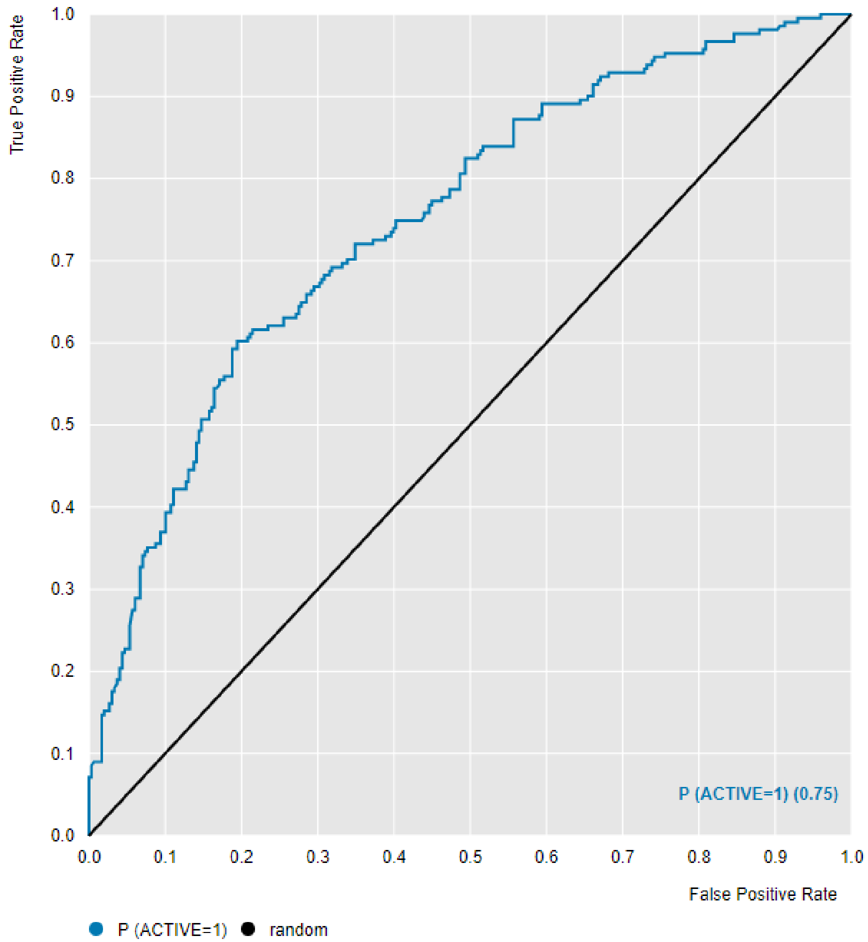

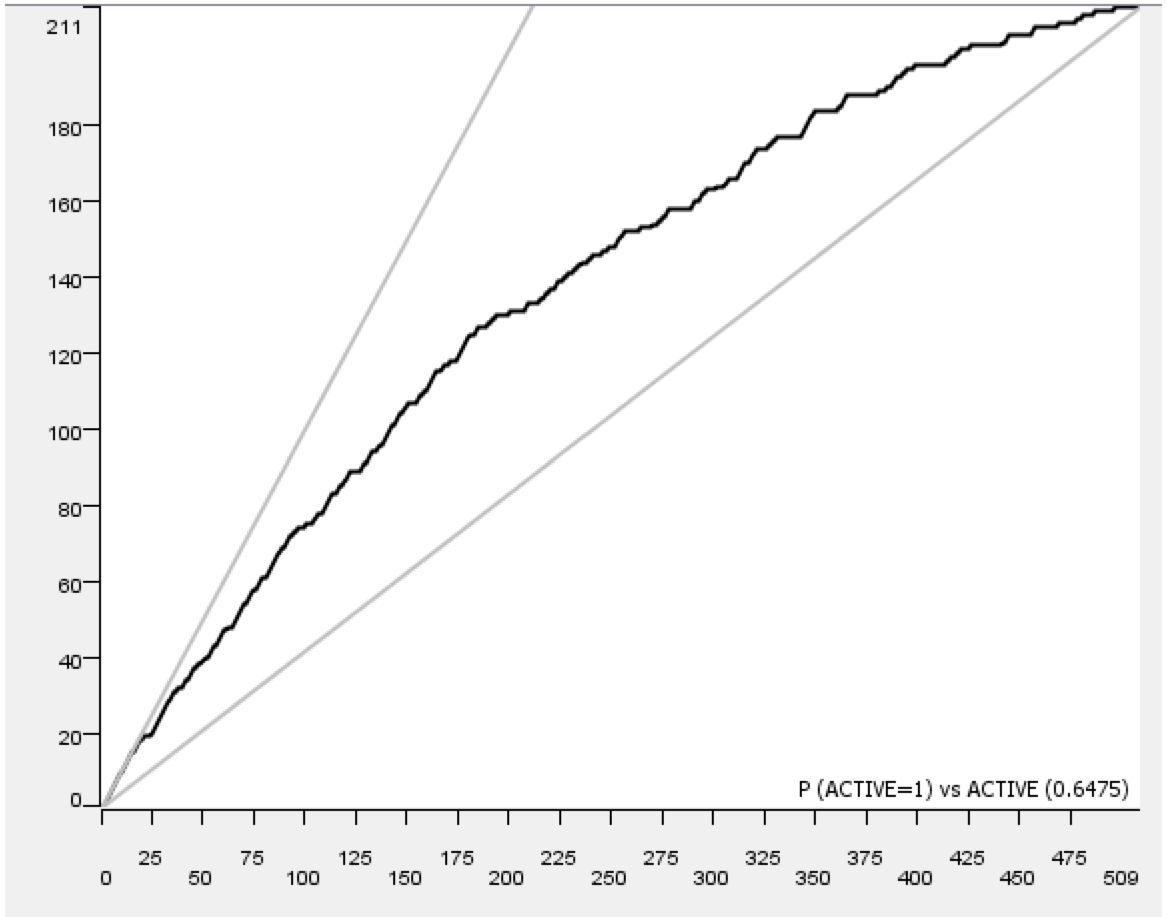

N=509

**Top 10% Mean IC50**	**756 nM (2 >10,000 nM mistakes)**

	Predicted Active	Predicted Inactive
Active	109	102
Inactive	48	250

PNN- <15nM DefGood in HIV Protease, 10% error; Random seed = 12178

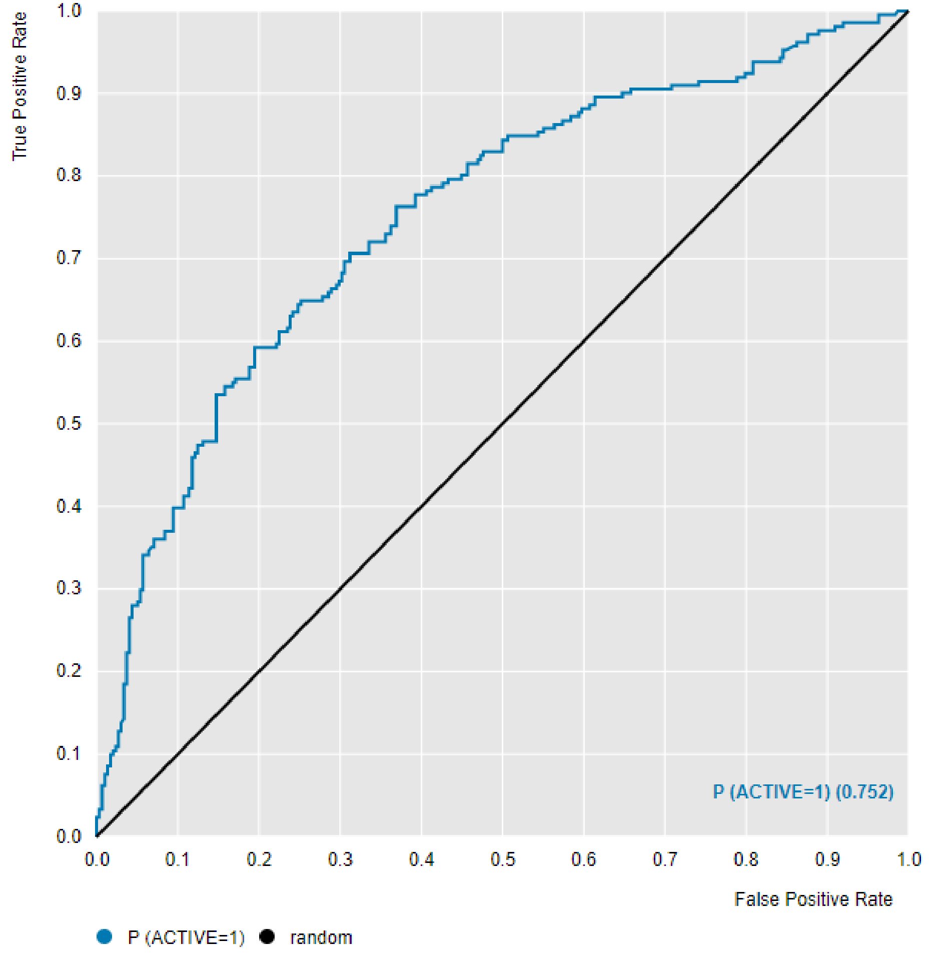

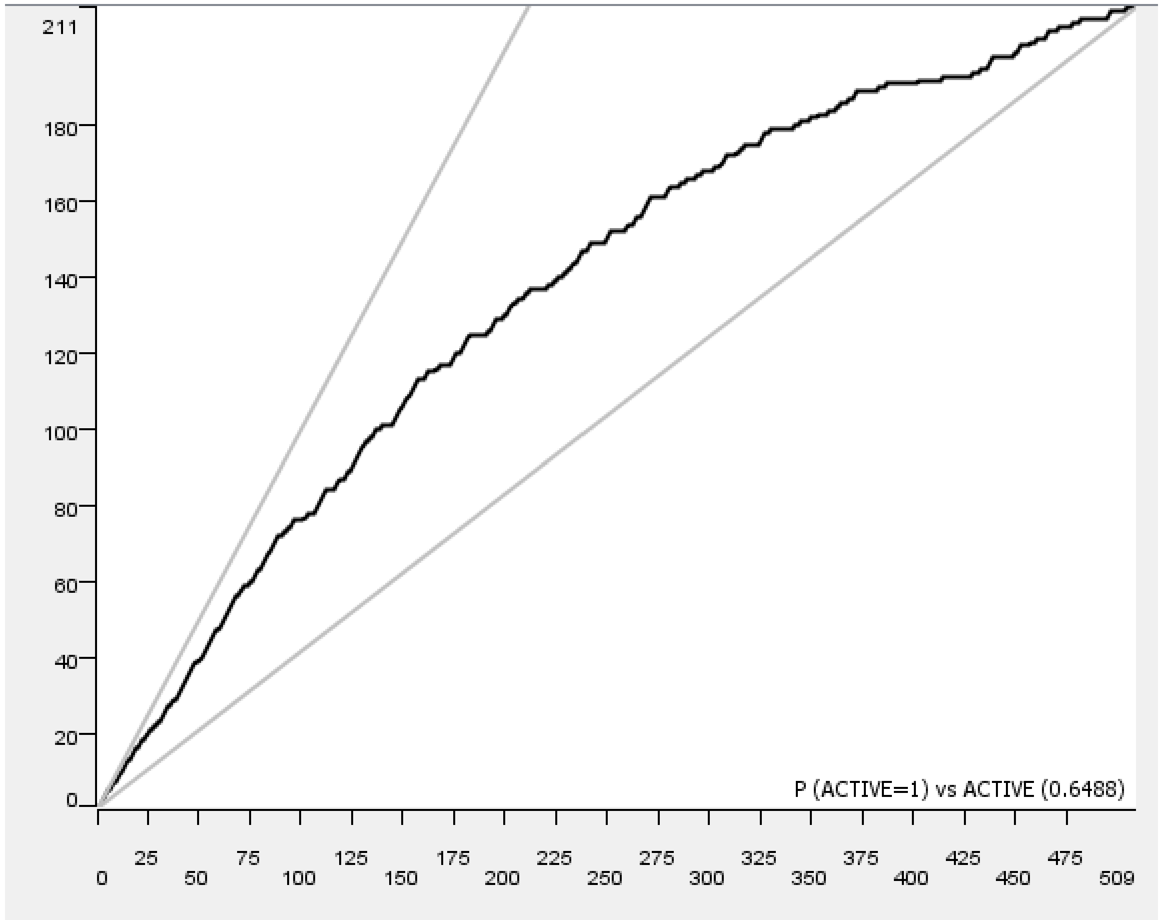

N=509

**Top 10% Mean IC50**	**269 nM**

	**Predicted Active**	**Predicted Inactive**
Active	104	107
Inactive	44	254

PNN- <15nM DefGood in HIV Protease, 20% error; Random seed = 12178

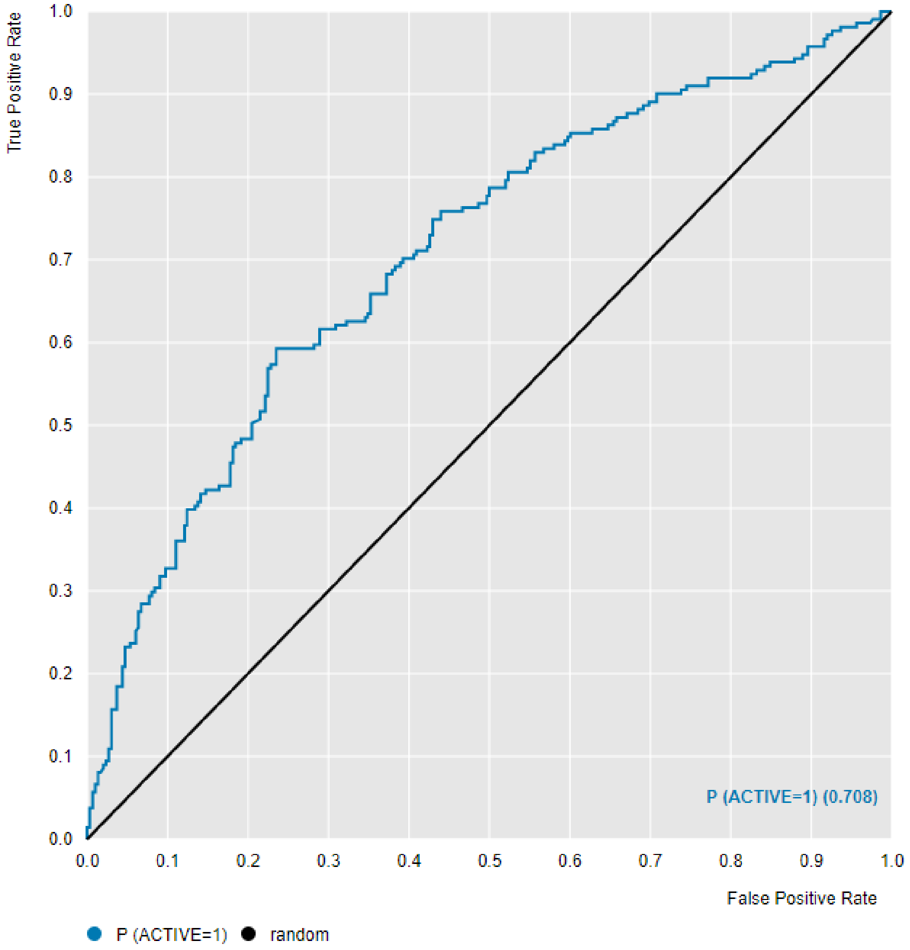

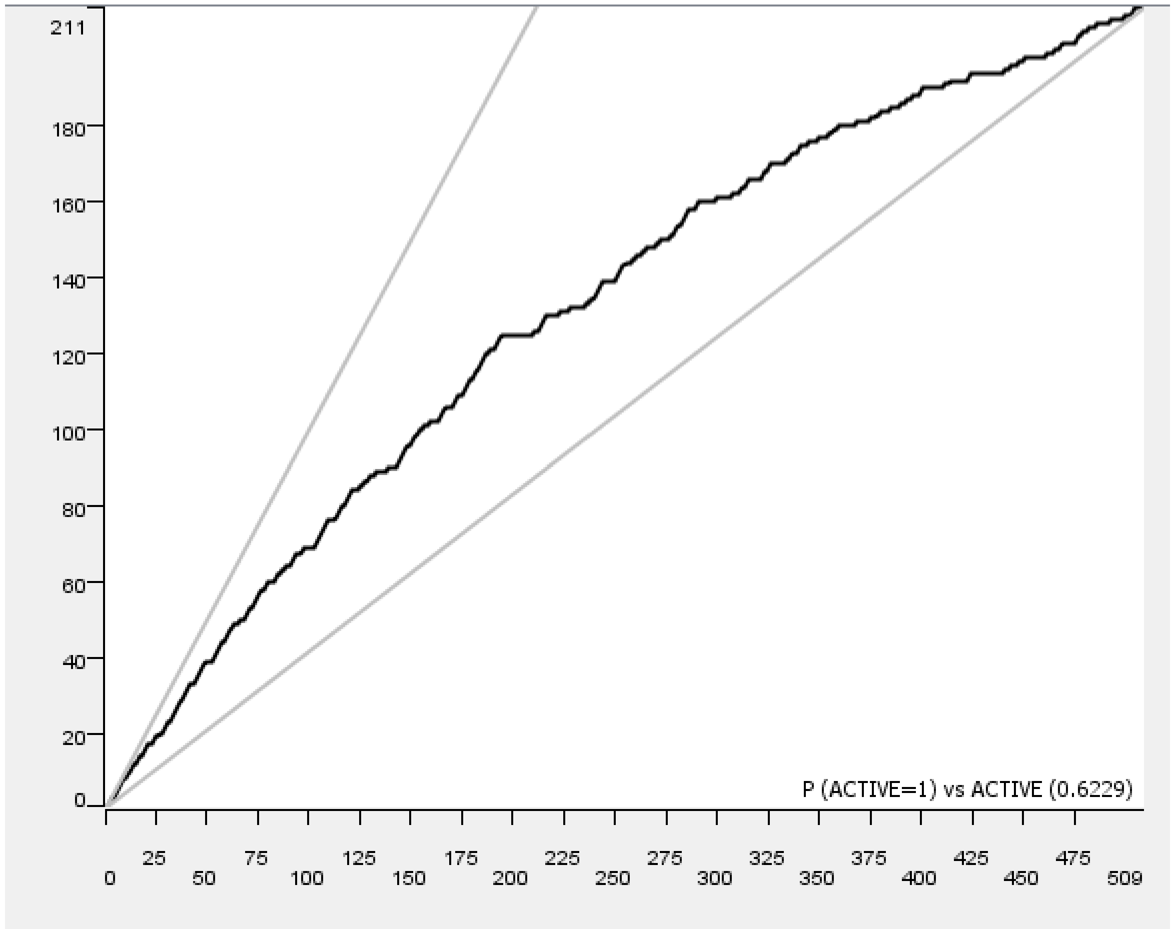

N=509

Top 10% Mean IC50	267 nM

	Predicted Active	Predicted Inactive
Active	93	118
Inactive	53	245

PNN- <15nM DefGood in HIV Protease, 25% error; Random seed = 12178

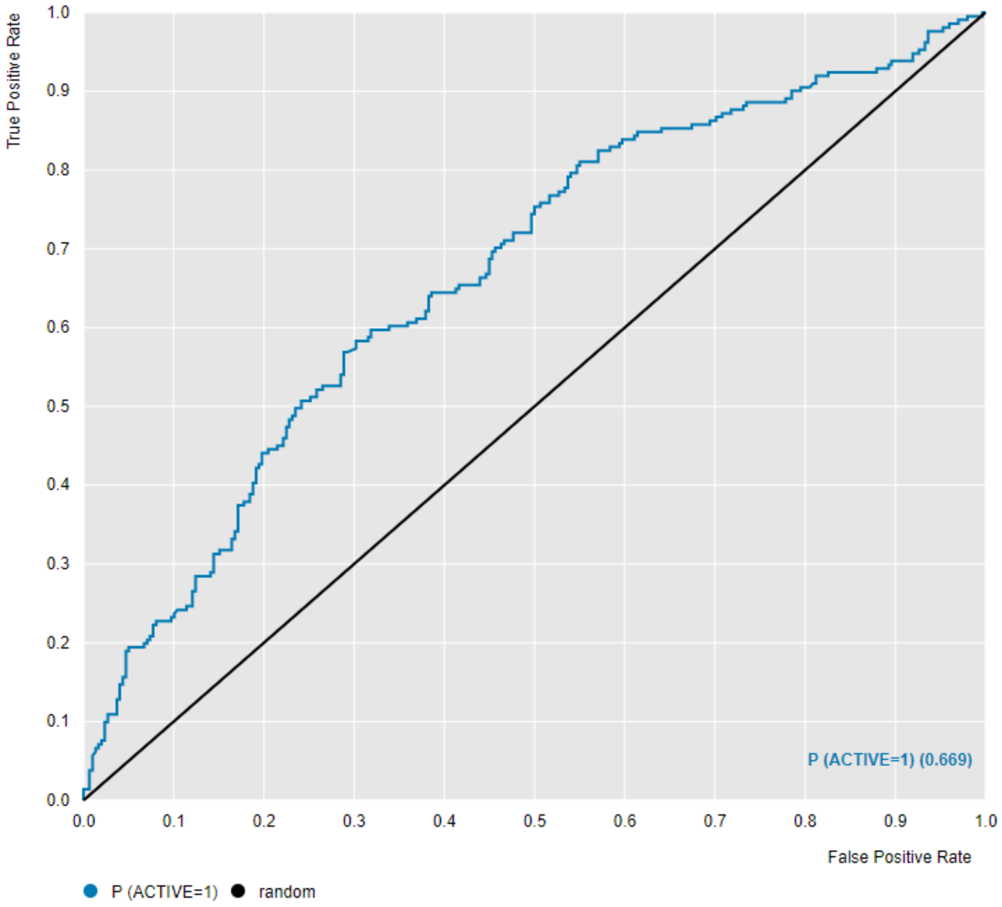

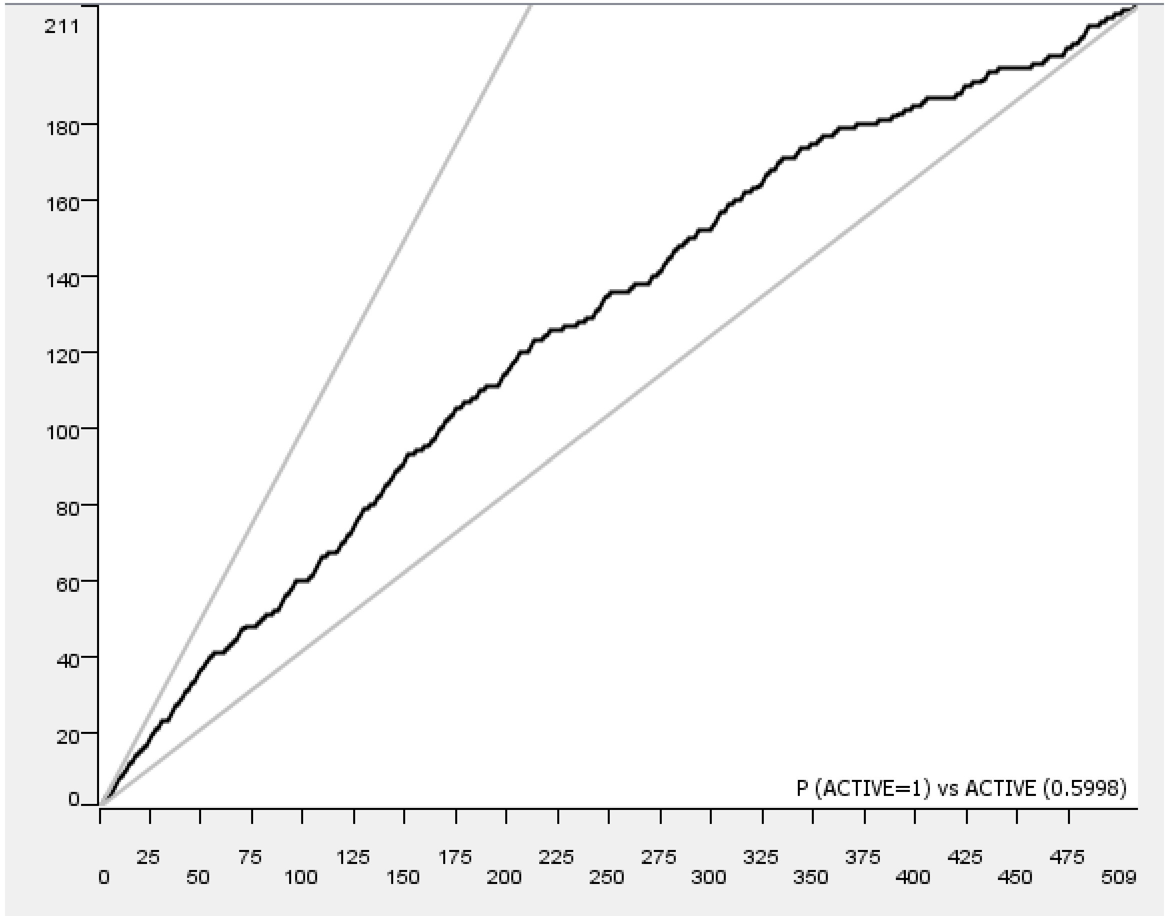

N=509

**Top 10% Mean IC50**	**498 nM**

	Predicted Active	Predicted Inactive
Active	109	102
Inactive	77	221

PNN - <15nM DefGood in HIV Protease, 25% error; Random seed = 12178 (reparameterized Theta minus = 0.05; theta plus = 0.25)

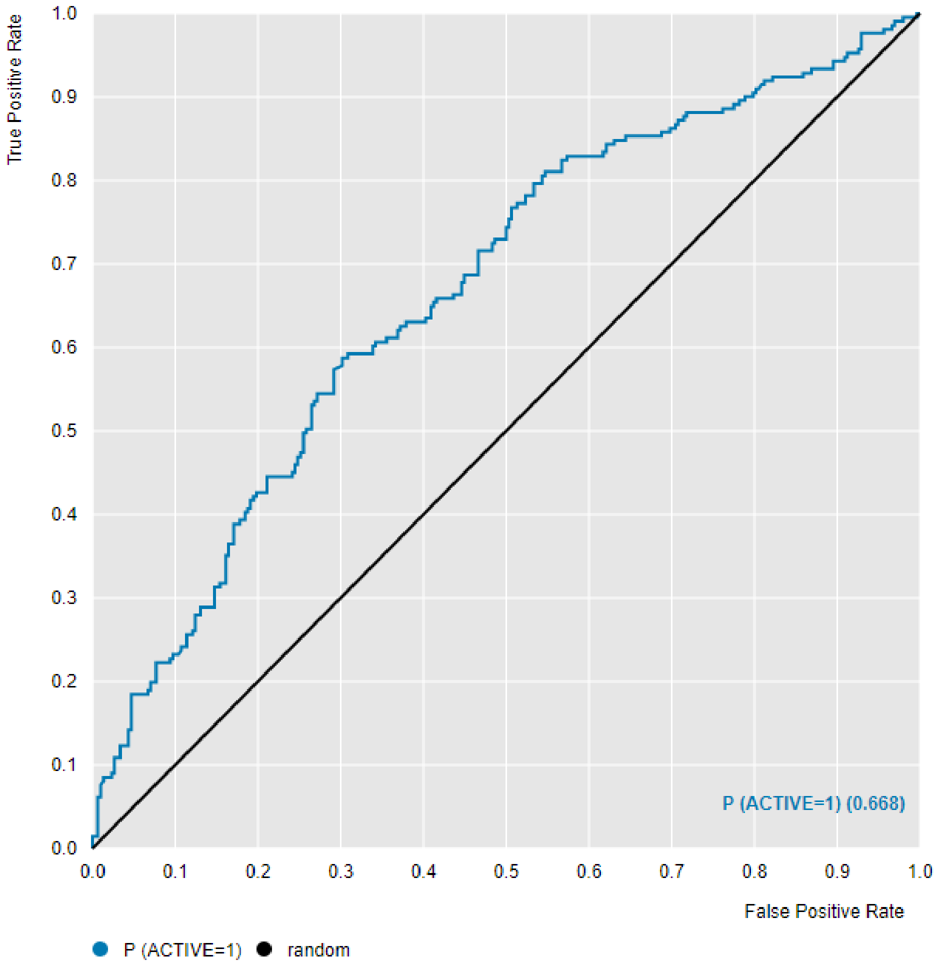

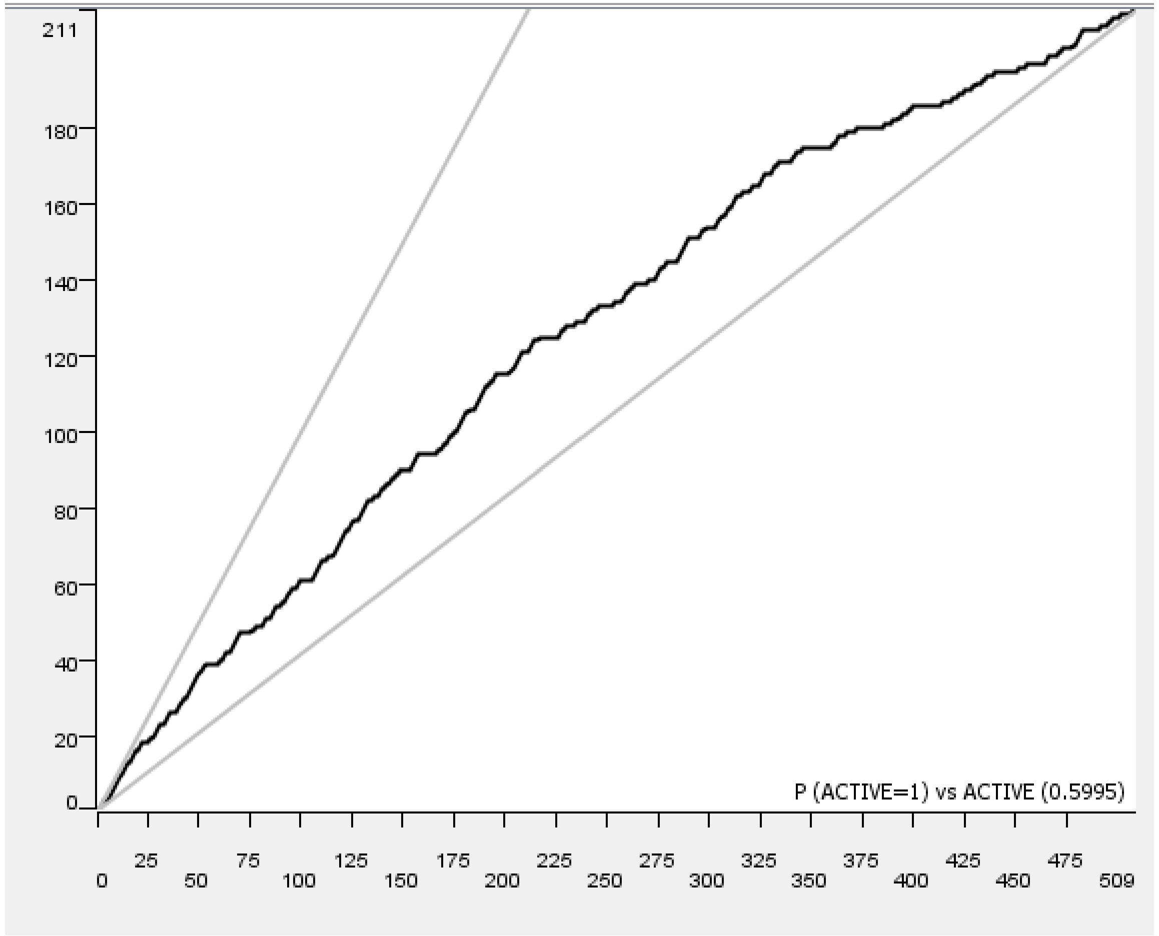

N=509

Top 10% Mean IC50	498 nM

	Predicted Active	Predicted Inactive
Active	110	101
Inactive	79	210

Conclusion - PNN

- Across three random seeds, the % error that lead to a significant enrichment failure and/or top 10% IC50 increase in the retrospective test was 25%, 15% and 25% for each unique random seed.
- Parameterization did not alter the failure point for each random split evaluated
- Split 121783 had a significant mistake (>100,000) even in the 0% error set. Used 12178 as the random seed

JAK2

JAK2 NBN Design

- Rules - $RELATION$ = "=" OR $IC50$ > 999 OR $IC50$ < 10 => TRUE
- Sort ascending and remove duplicates
- DefGood scanned between 5 and 25 nM
- 80-20 split for both actives and inactives
- NBN – ECFP4 used Classifier where Prediction was Active = True
- V617F mutant data was removed from the dataset as we were only interested in WT JAK2
- Resulted in 3624 compounds after cleaning the data

<5 nM DefGood in JAK2

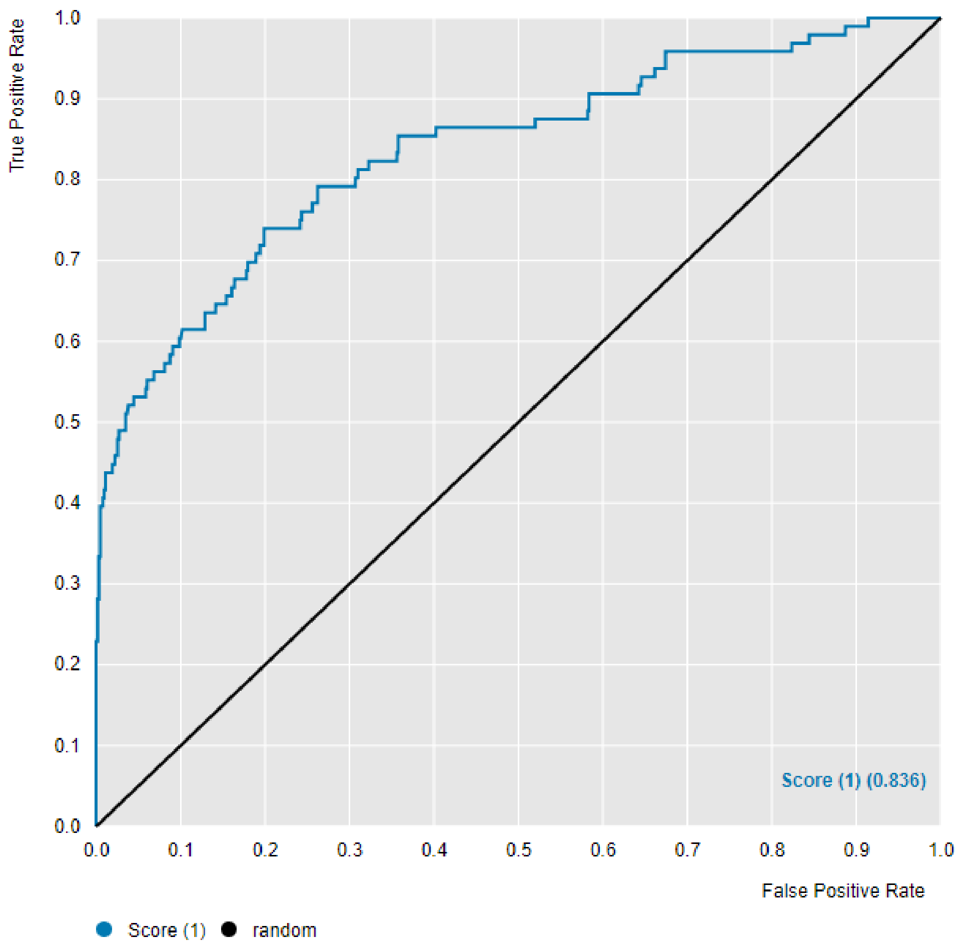

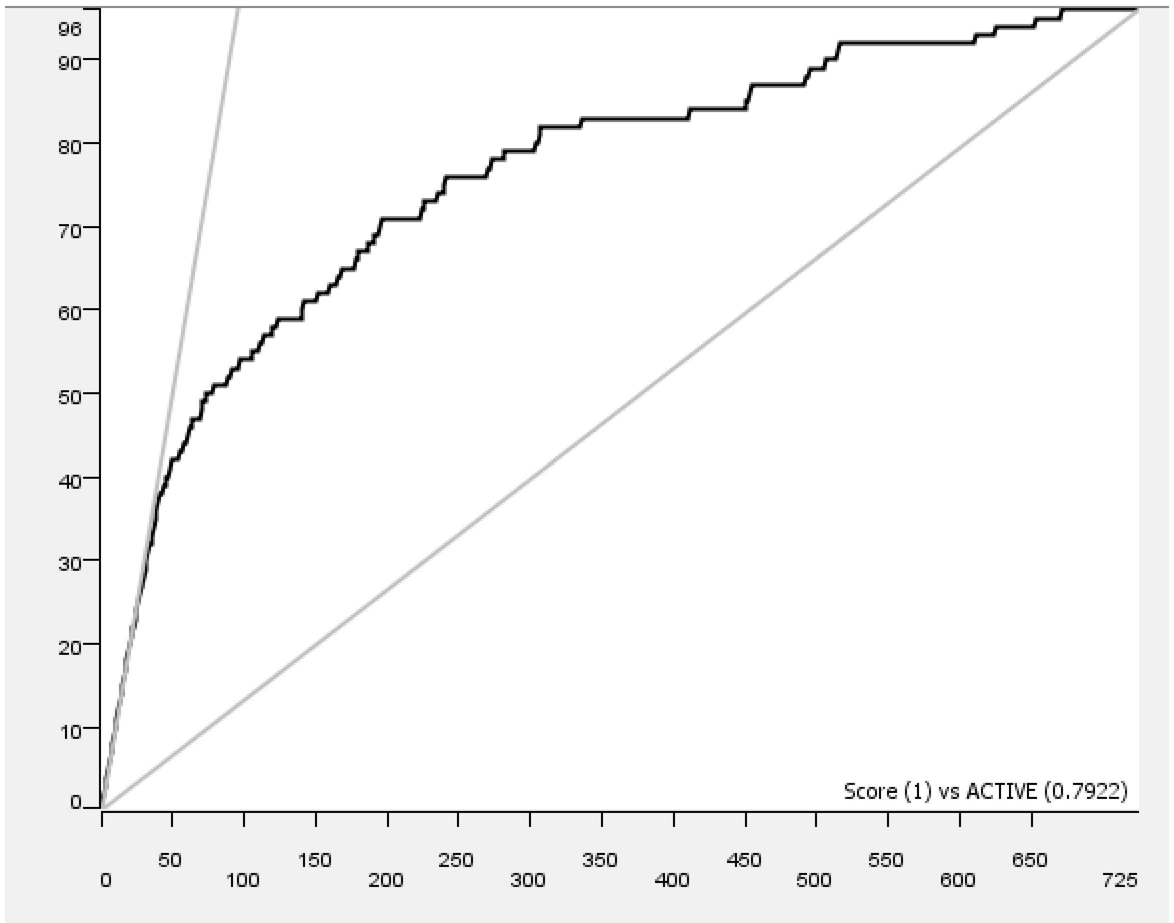

N=725

Top 10% Mean IC50	175 nM

	Predicted Active	Predicted Inactive
Active	67	29
Inactive	119	510

<10 nM DefGood in JAK2

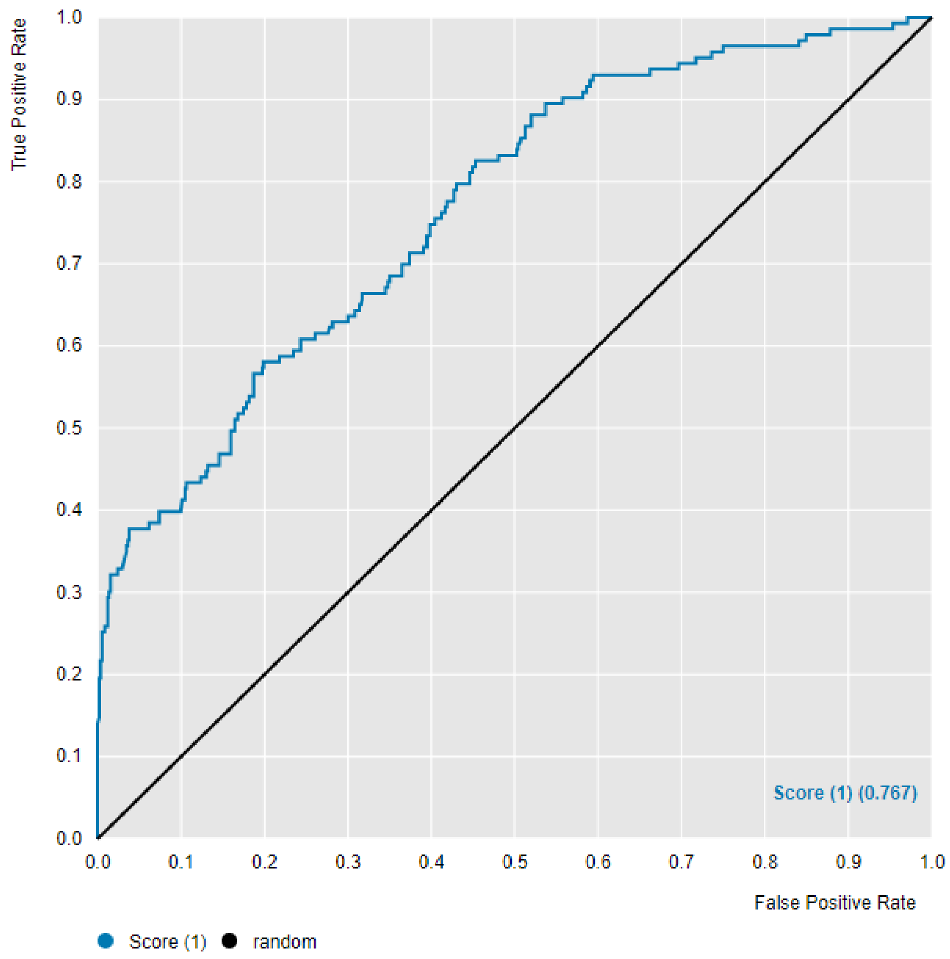

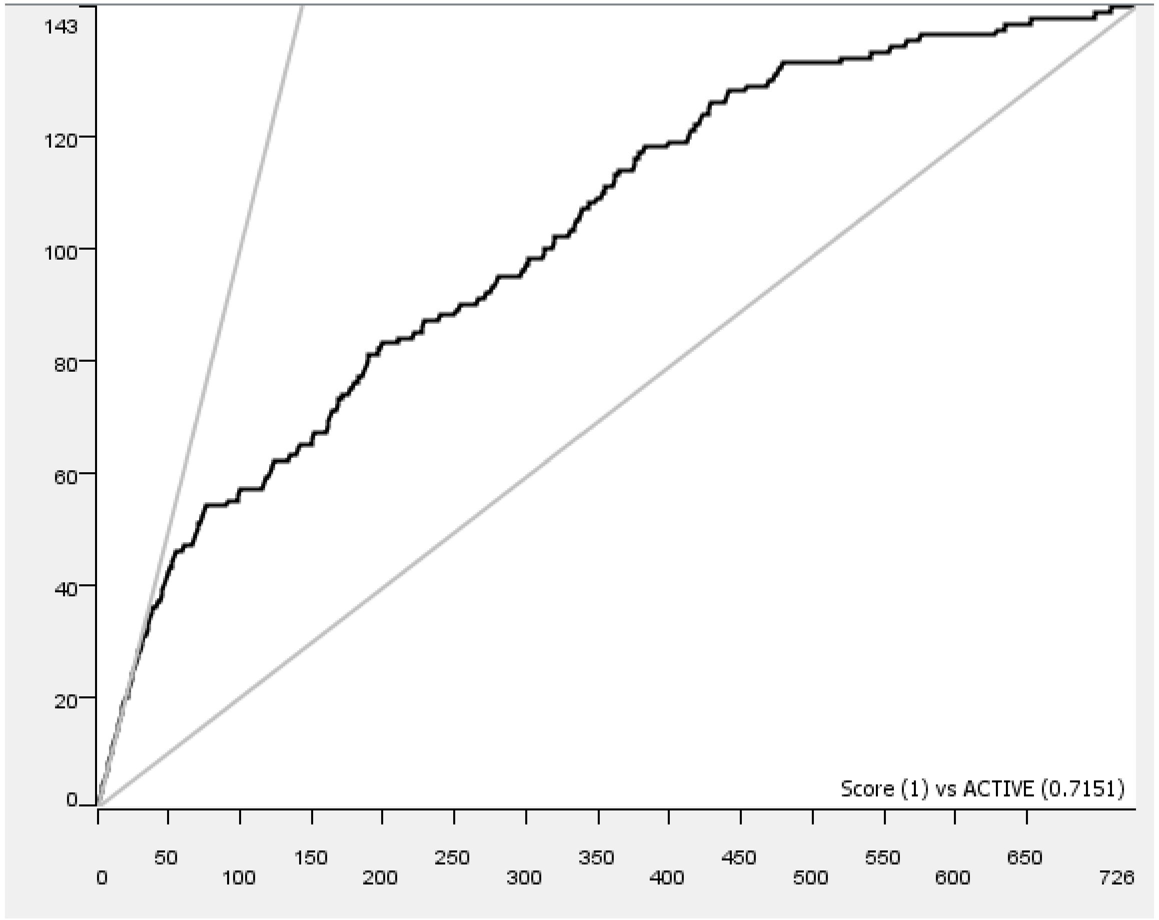

N=726

Top 10% Mean IC50	309 nM

	Predicted Active	Predicted Inactive
Active	97	46
Inactive	203	380

<15 nM DefGood in JAK2

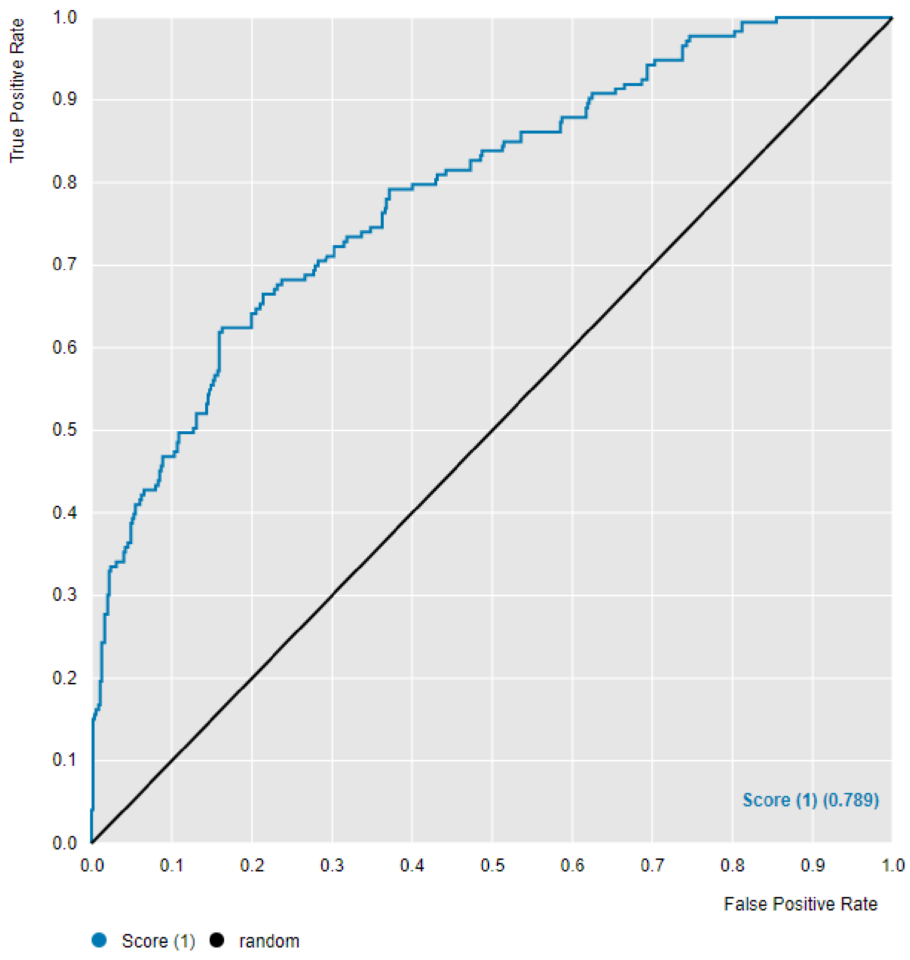

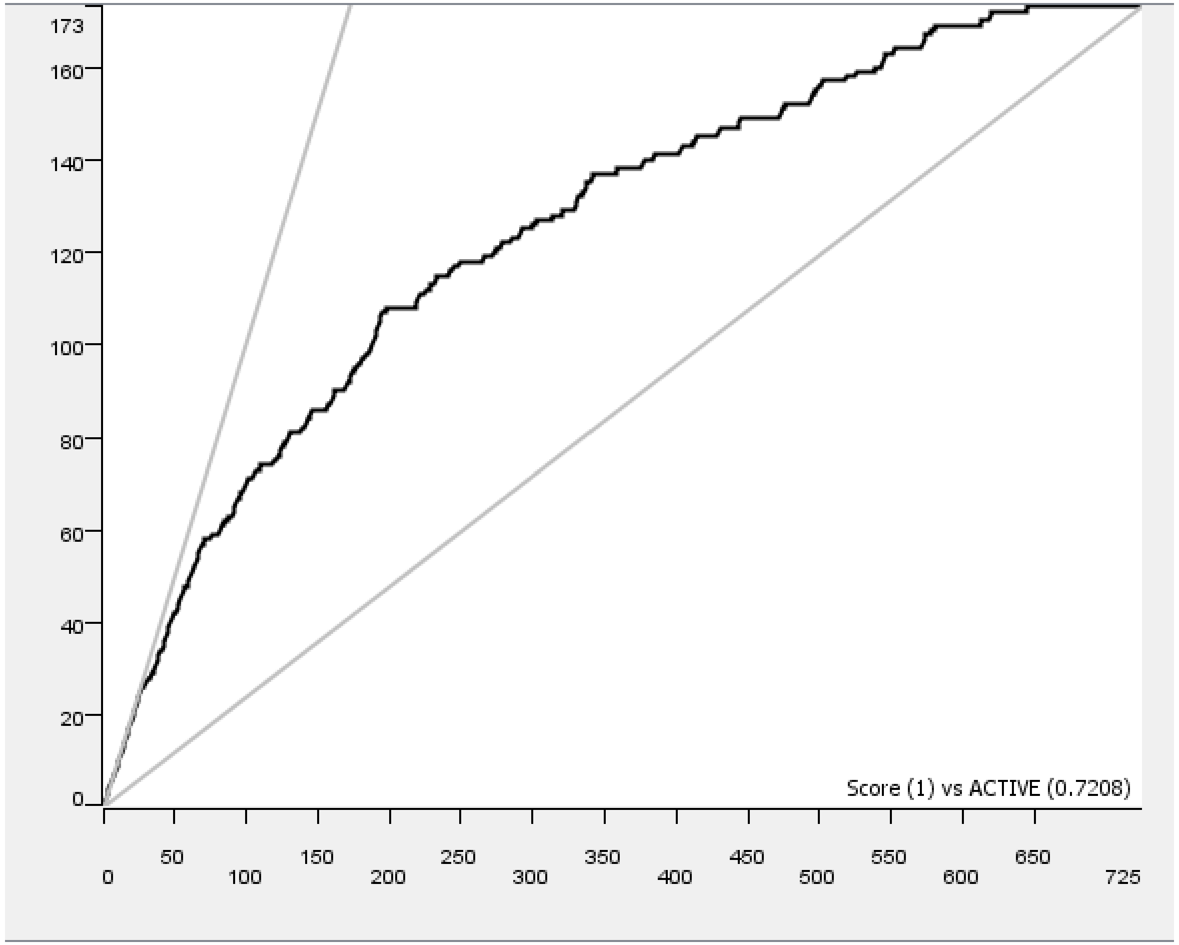

N=725

Top 10% Mean IC50	38.3 nM

	Predicted Active	Predicted Inactive
Active	110	63
Inactive	110	442

<20 nM DefGood in JAK2

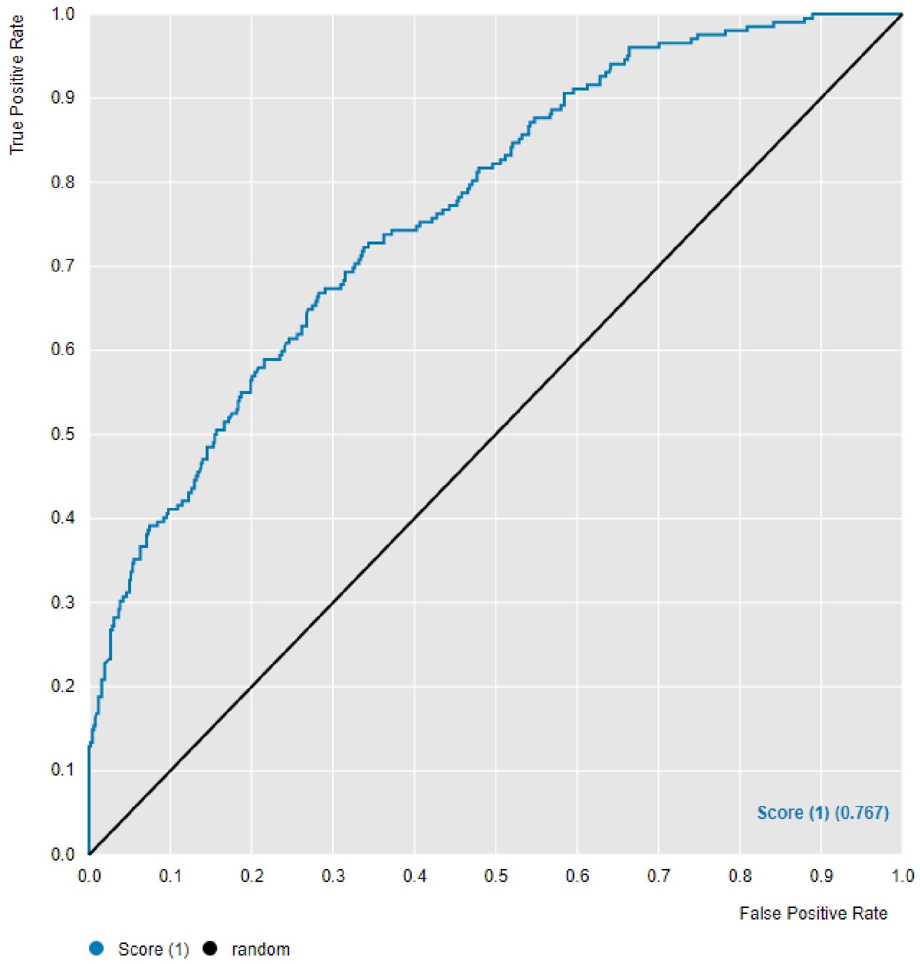

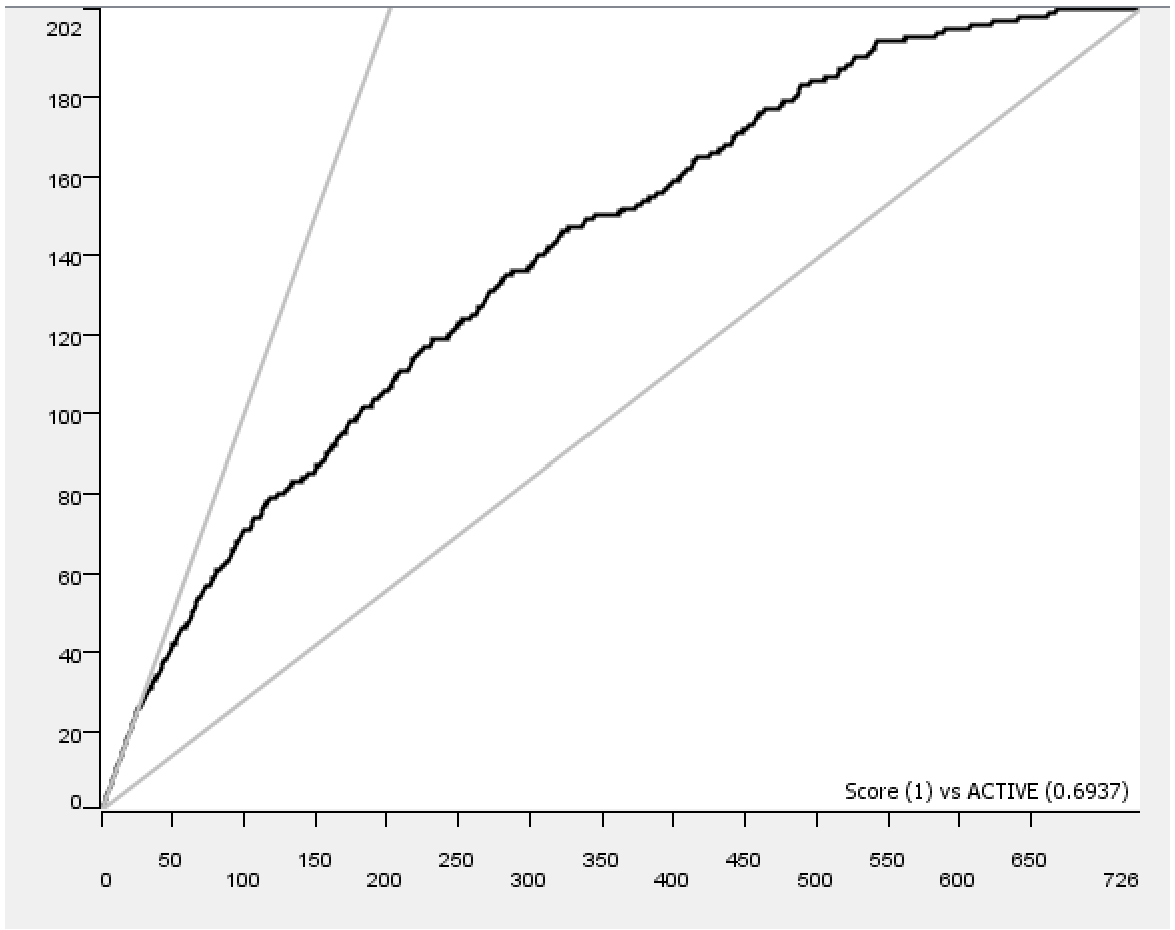

N=726

Top 10% Mean IC50	32.6 nM

	Predicted Active	Predicted Inactive
Active	117	85
Inactive	110	414

<25 nM DefGood in JAK2

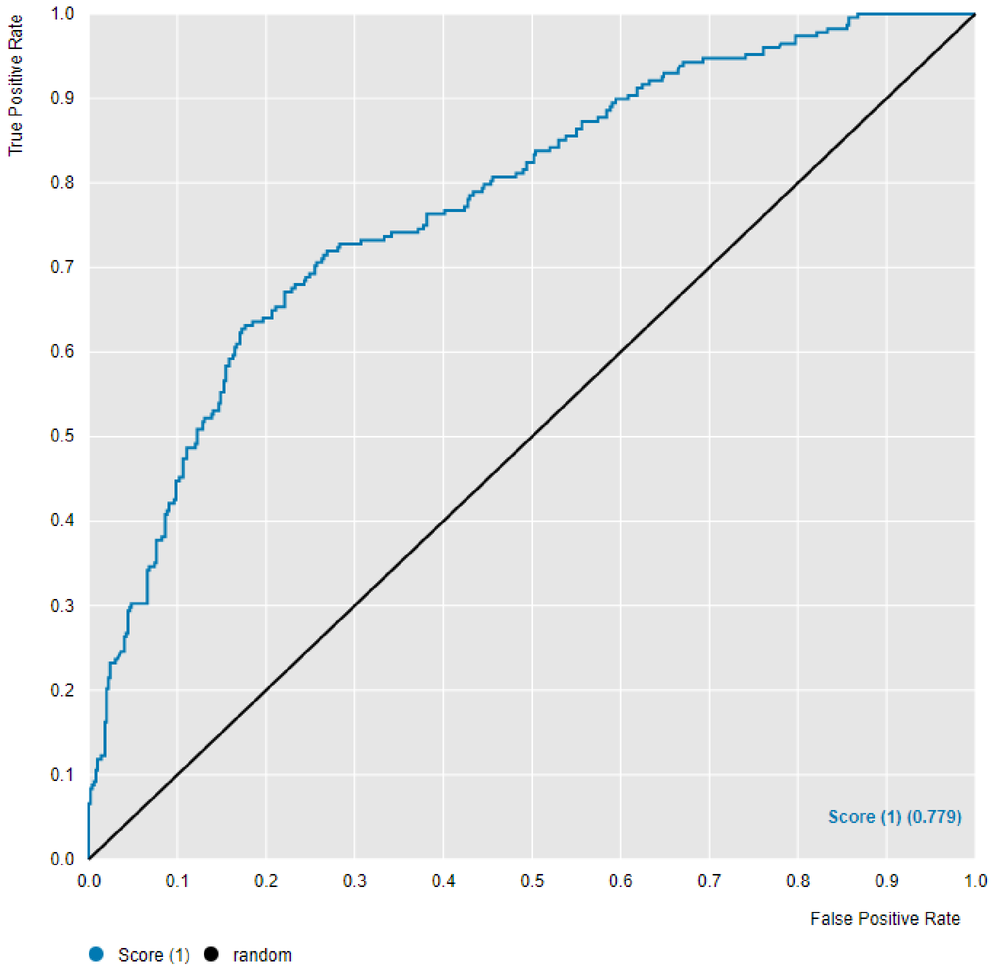

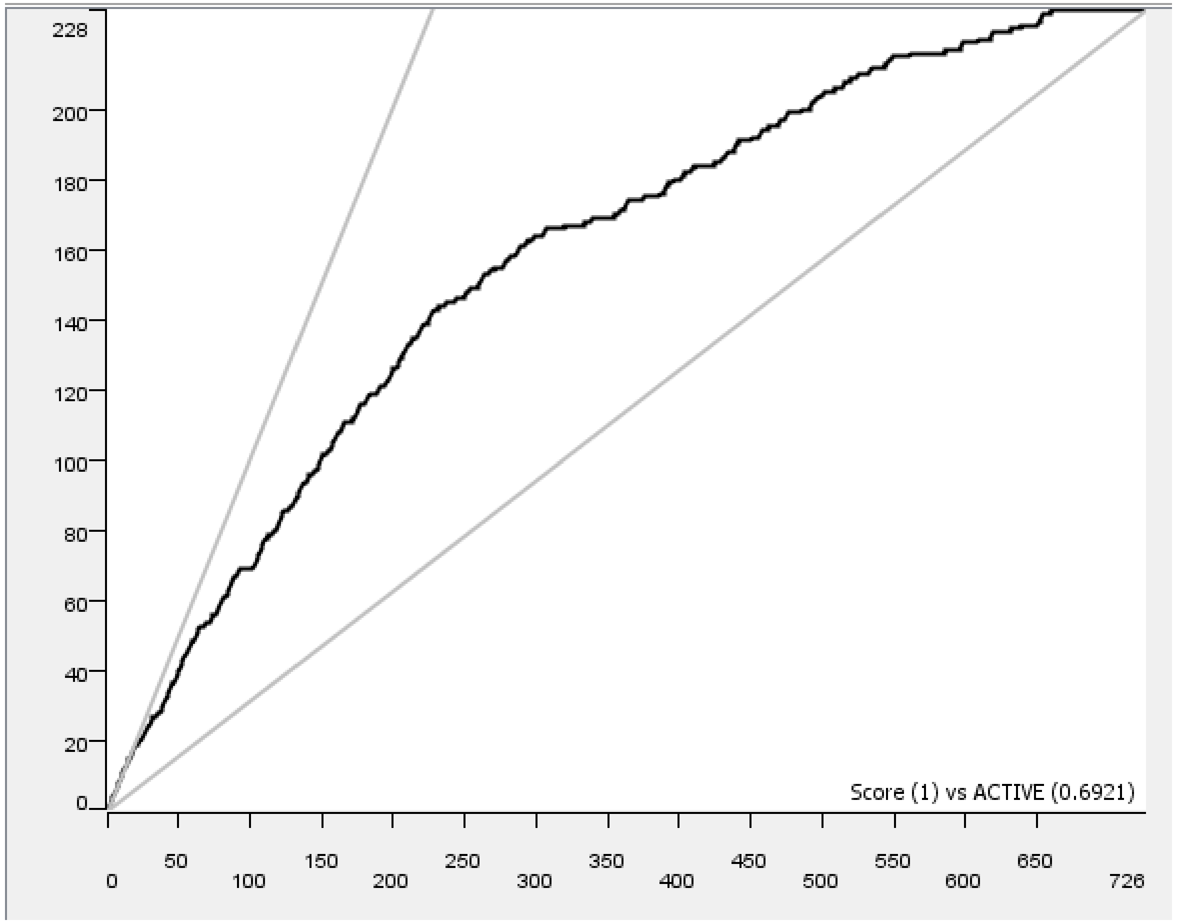

N=726

Top 10% Mean IC50	37.4 nM

	Predicted Active	Predicted Inactive
Active	161	67
Inactive	129	369

Decision on DefGood

- <15 nM has the best performance with regard to enrichment, ROC mean top 10% IC50. The <5 definition of good had excellent enrichment and a better ROC, but when mistakes were made by the algorithm, they were severe.

RF - <15 nM DefGood in JAK2

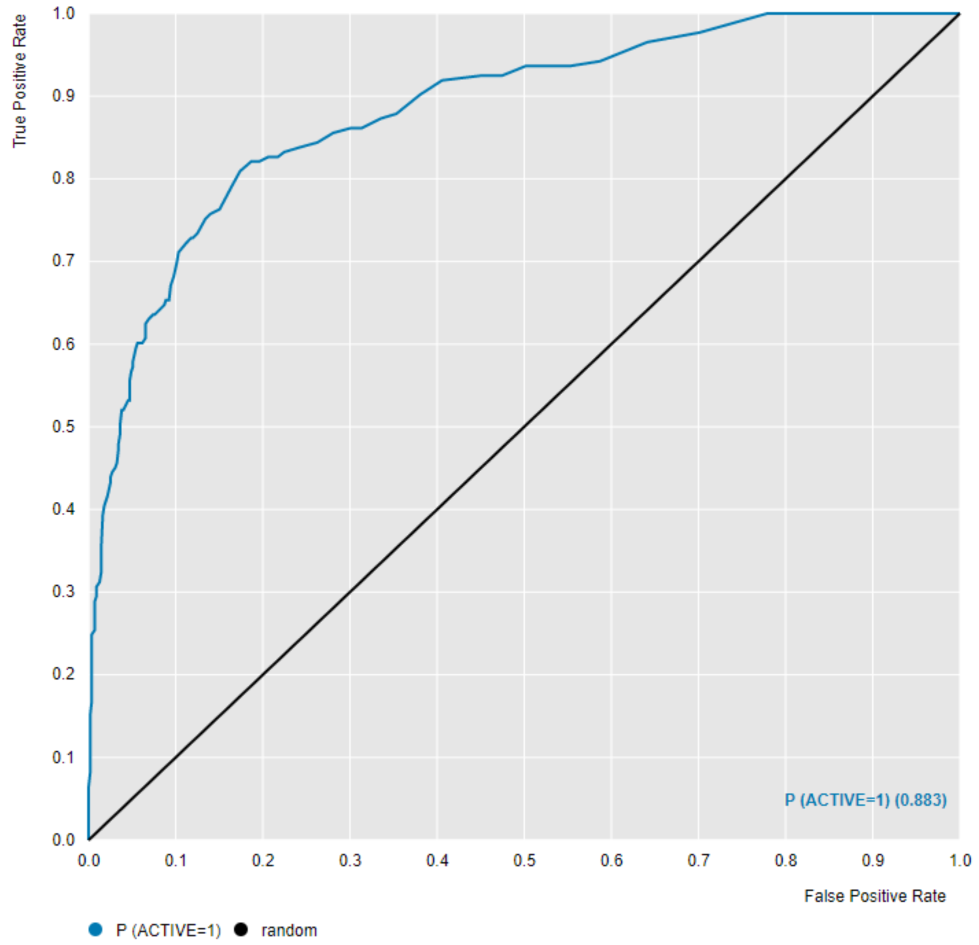

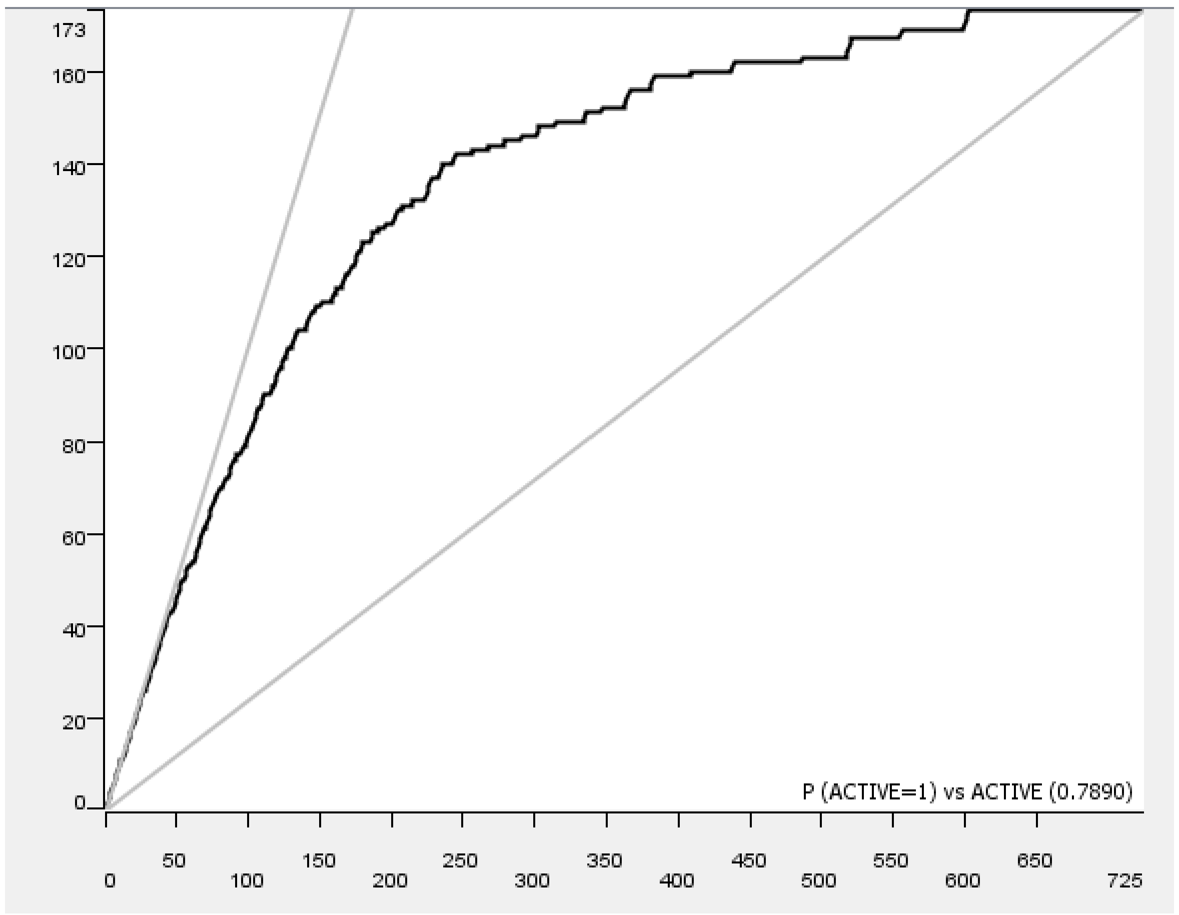

N=725

**Top 10% Mean IC50**	**26.4 nM**

	Predicted Active	Predicted Inactive
Active	99	74
Inactive	28	524

Decision on DefGood - RF

- Work in ALK showed DefGood in the NBN was similar to DefGood in an RF. Therefore, this value for DefGood from the NBN design was used as DefGood.

PNN - <5 nM DefGood in JAK2

- Properties List (46 calculatable properties were used)
- Parameterization (Global Accuracy for Activity at a 5 nM decision value was used, and a 5-fold stratified sampling cross validation was used to parameterize theta minus and theta plus) Theta Minus = 0.95 and Theta Plus = 0.95

PNN - <5 nM DefGood in JAK2 (0.95, 0.95)

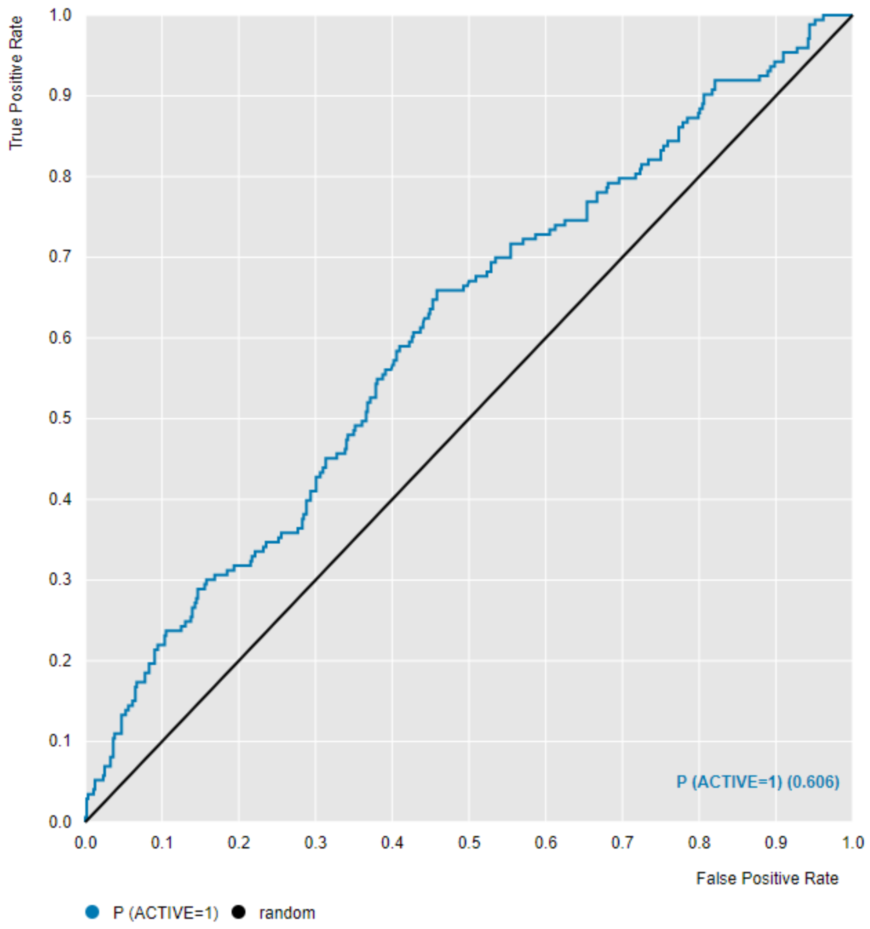

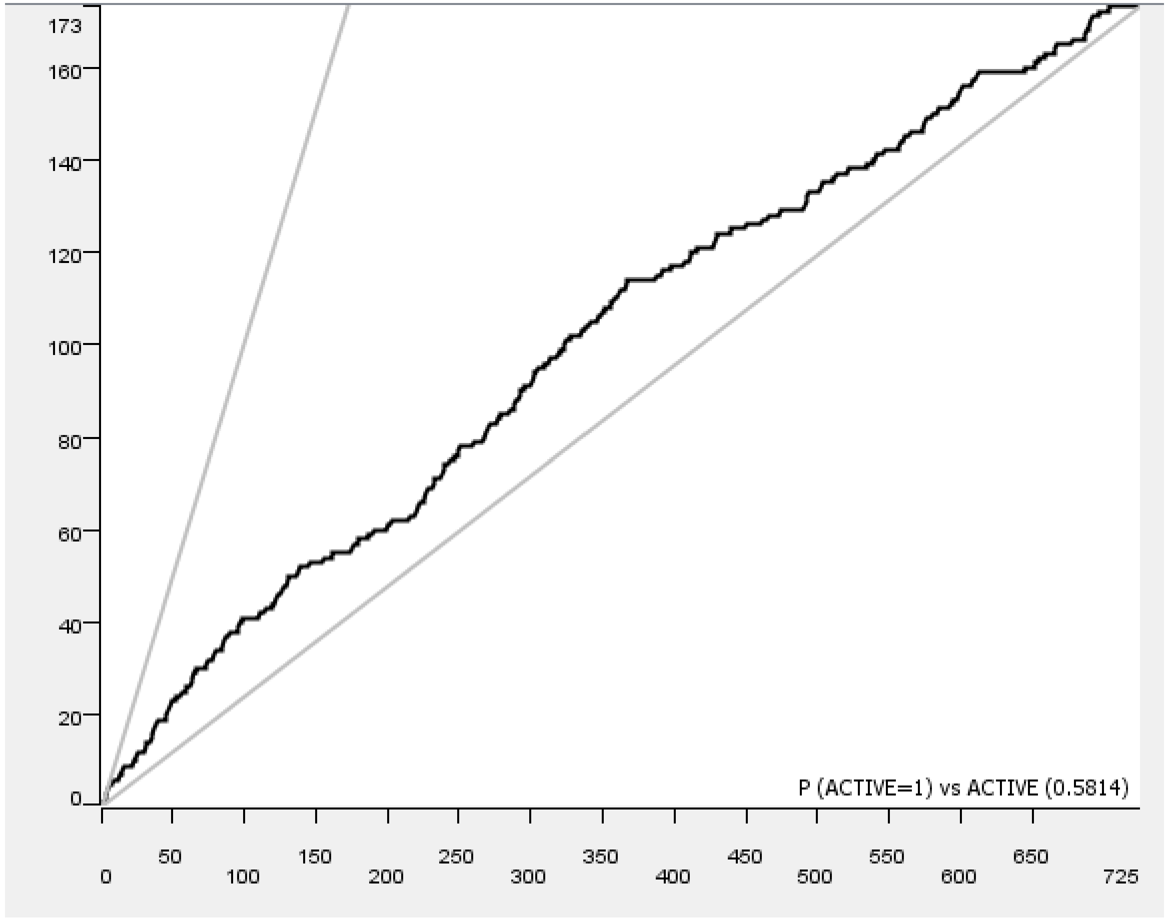

N=725

**Top 10% Mean IC50**	**3,600 nM**

	Predicted Active	Predicted Inactive
Active	0	173
Inactive	0	552

Decision on DefGood - PNN

- Parameterization failed to generate theta values that were useful in parameterizing the PNN.

NBN Error Tolerance- <15 nM DefGood in JAK2

- 0-50% absolute error

<15 nM DefGood in JAK2, 5% error; Random seed = 1515533876005

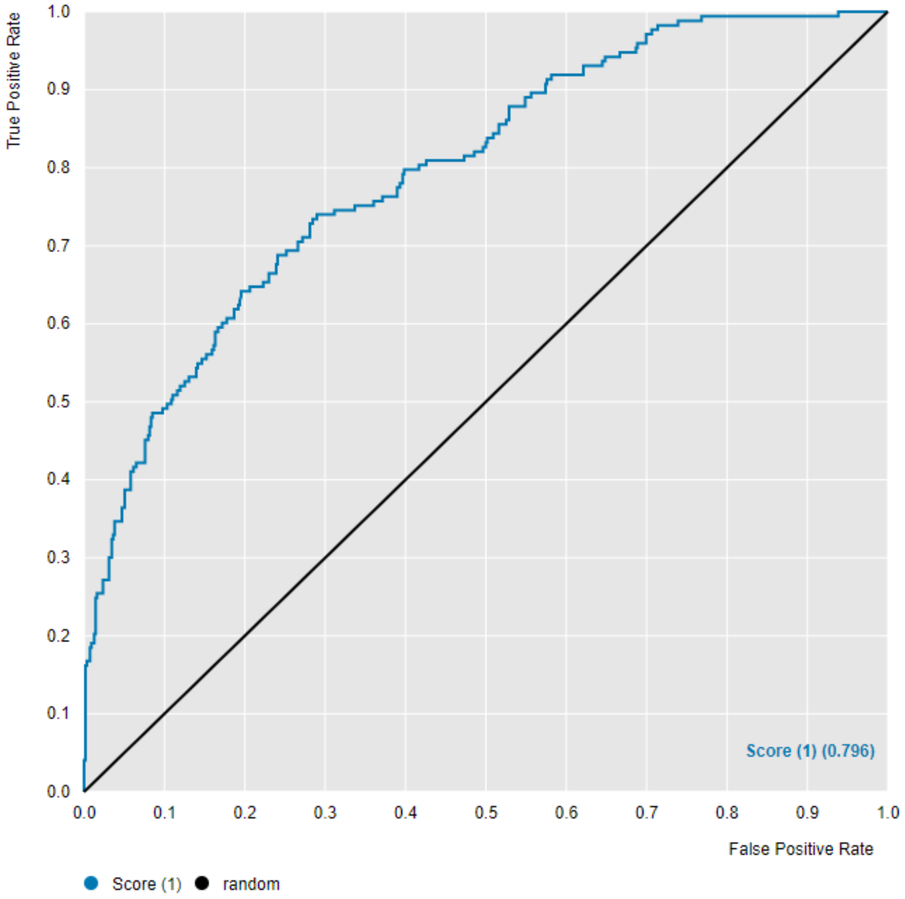

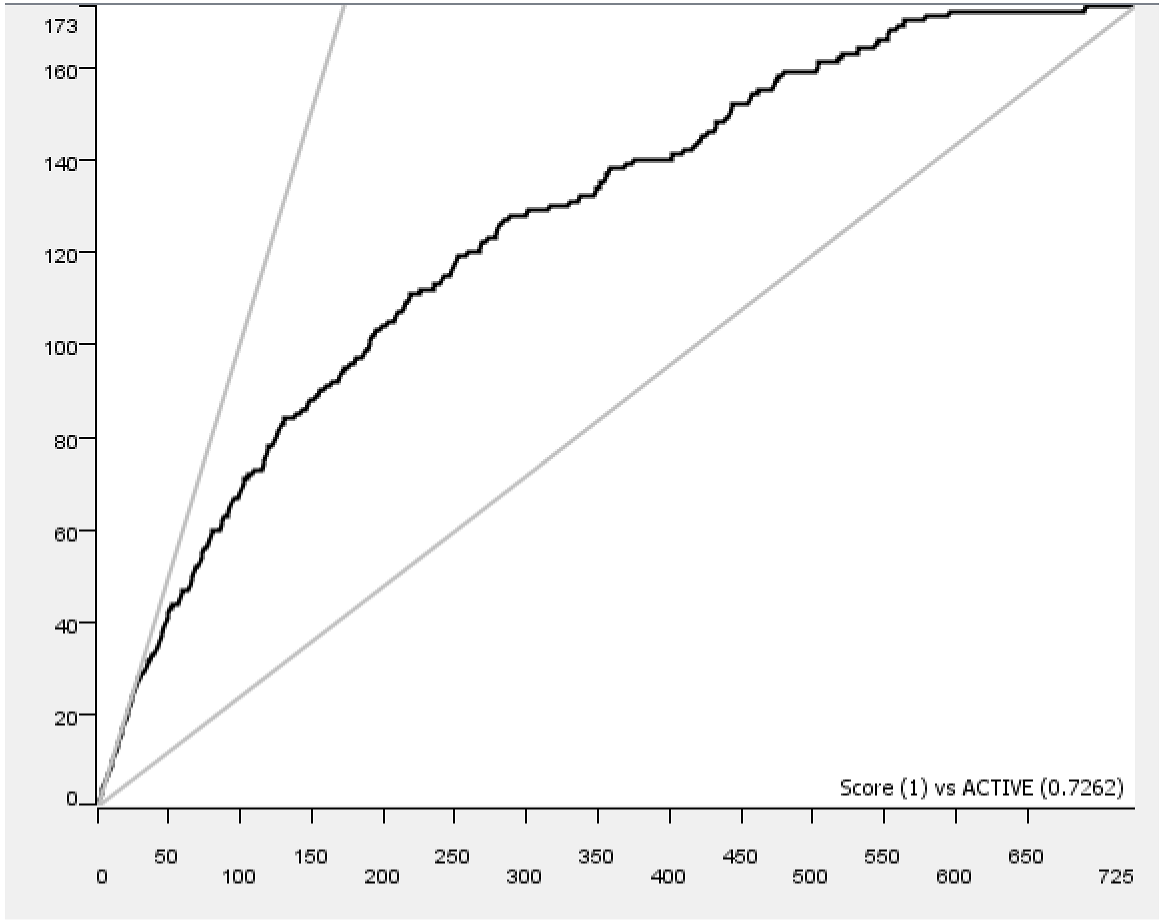

N=725

**Top 10% Mean IC50**	**304 nM (one 14000 nM)**

	Predicted Active	Predicted Inactive
Active	127	46
Inactive	160	392

<15nM DefGood in JAK2, 10% error

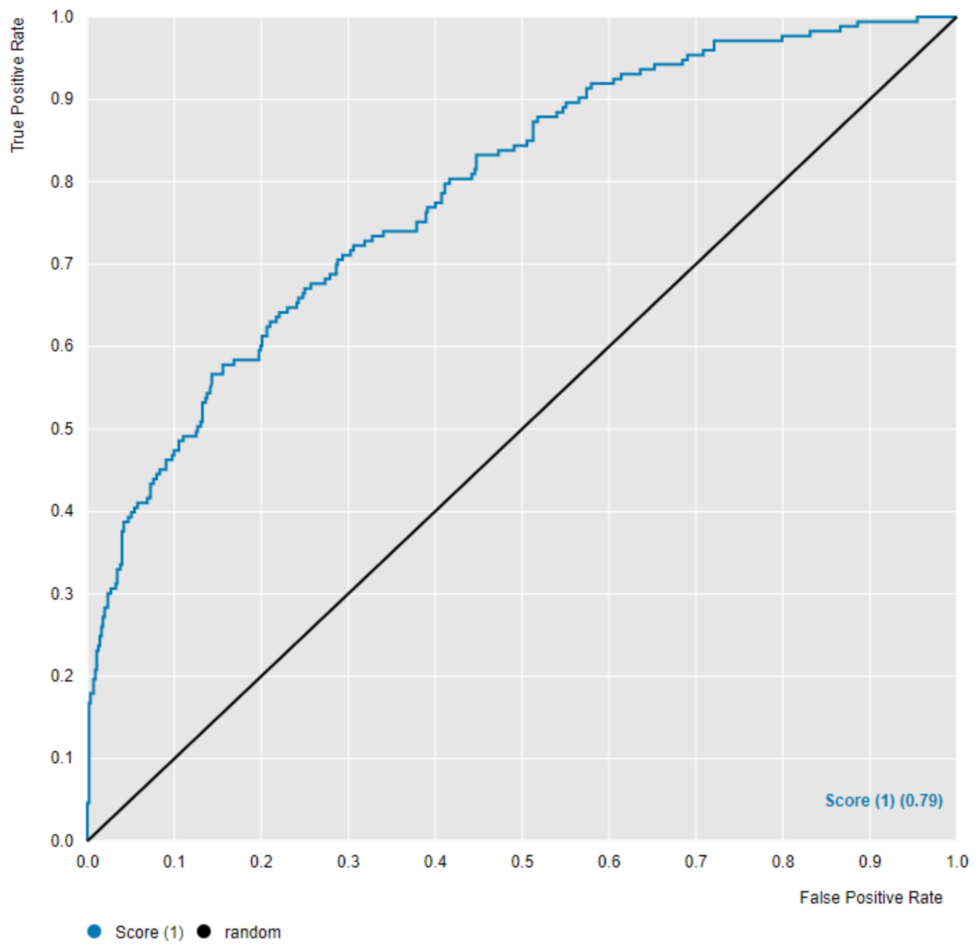

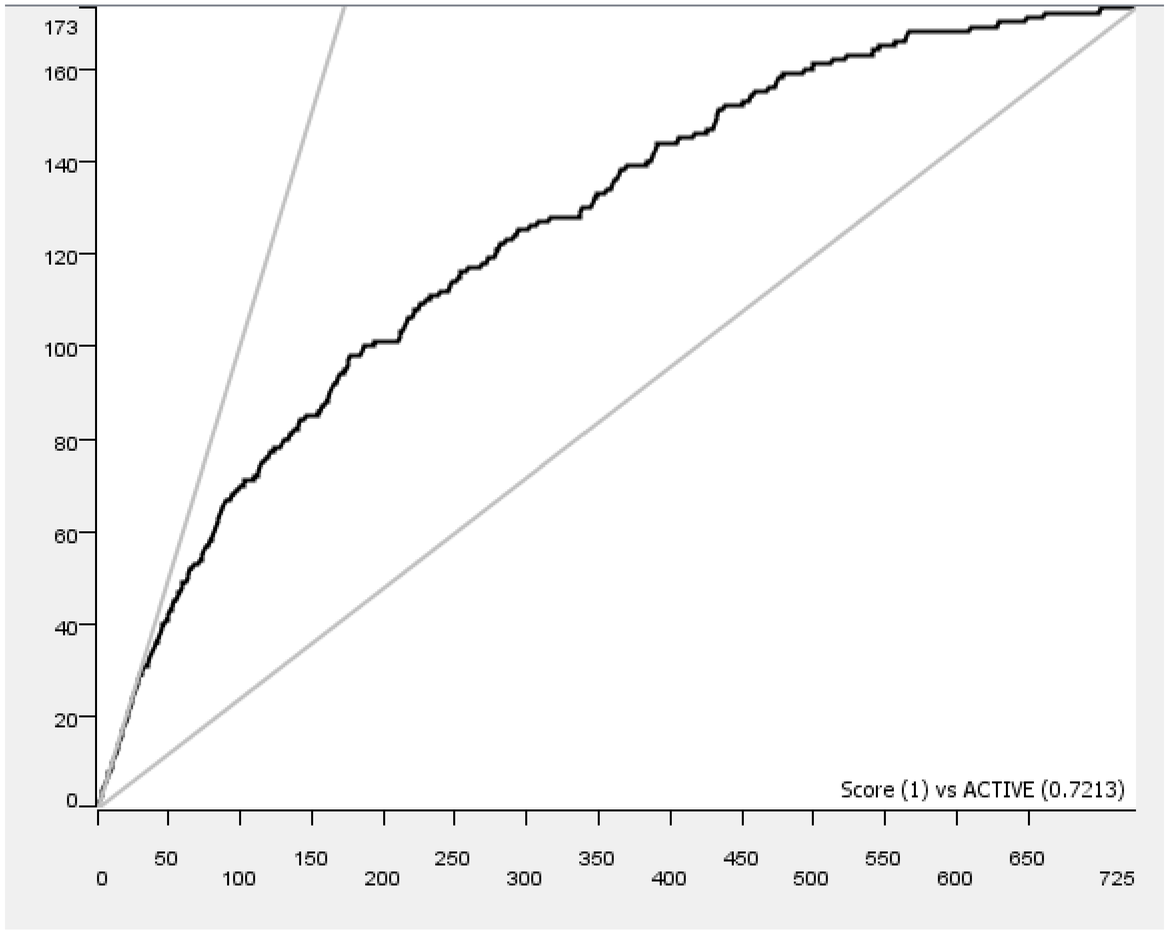

N=725

**Top 10% Mean IC50**	**258 nM (one 14000 nM)**

	**Predicted Active**	**Predicted Inactive**
Active	125	48
Inactive	170	382

<15nM DefGood in JAK2, 15% error

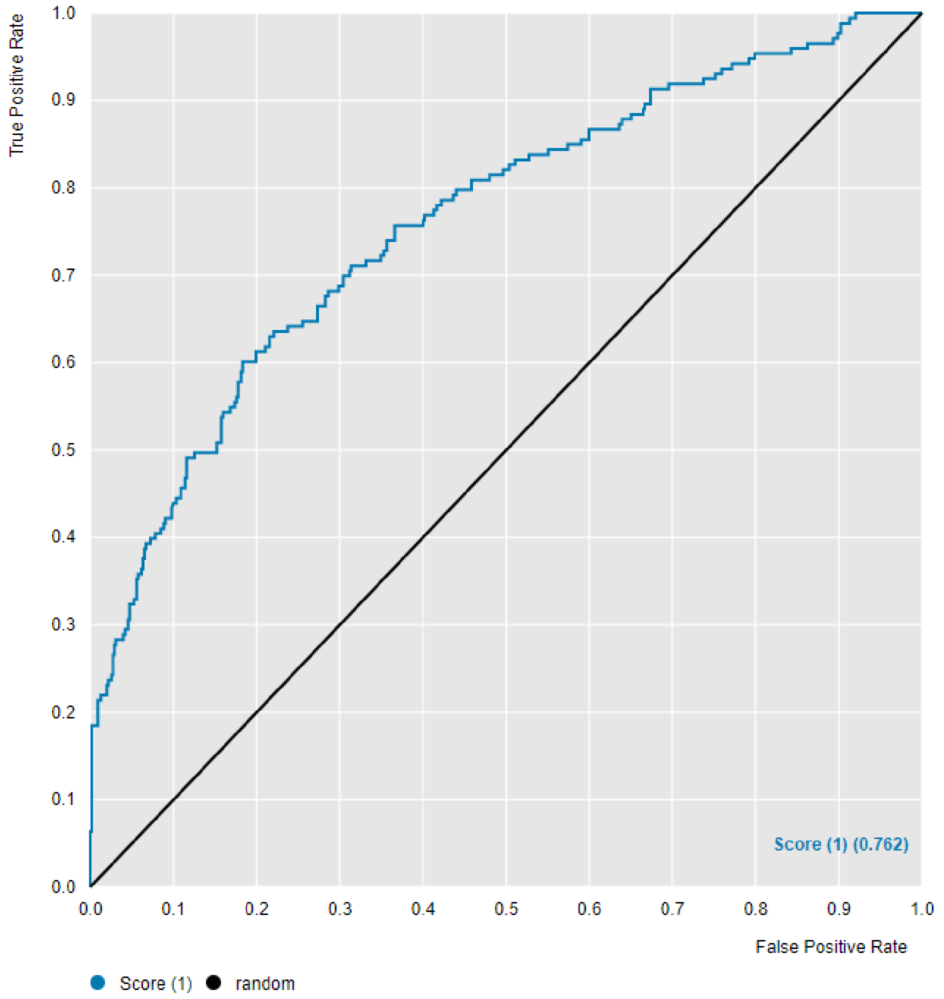

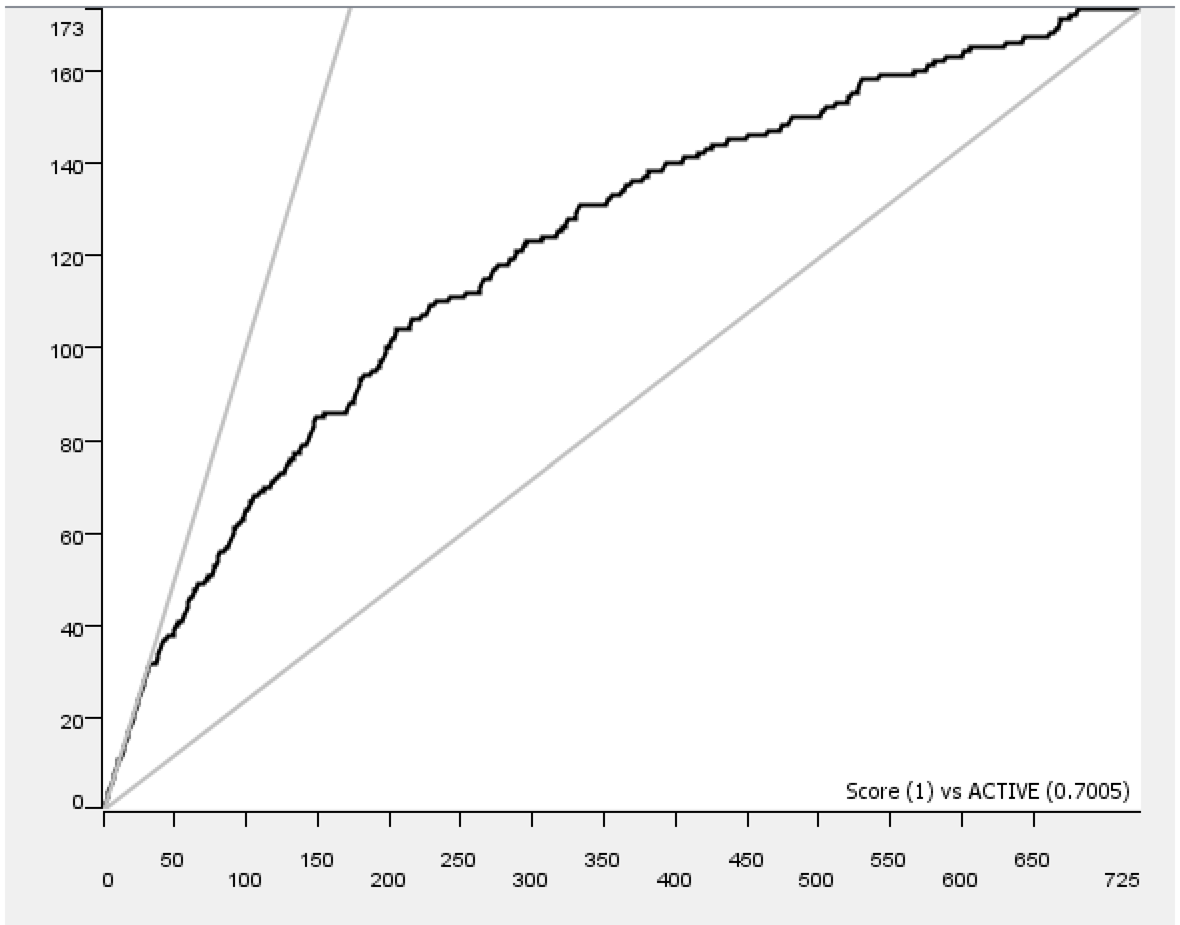

N=725

Top 10% Mean IC50	82.3 nM

	Predicted Active	Predicted Inactive
Active	104	69
Inactive	105	447

<15nM DefGood in JAK2, 20% error

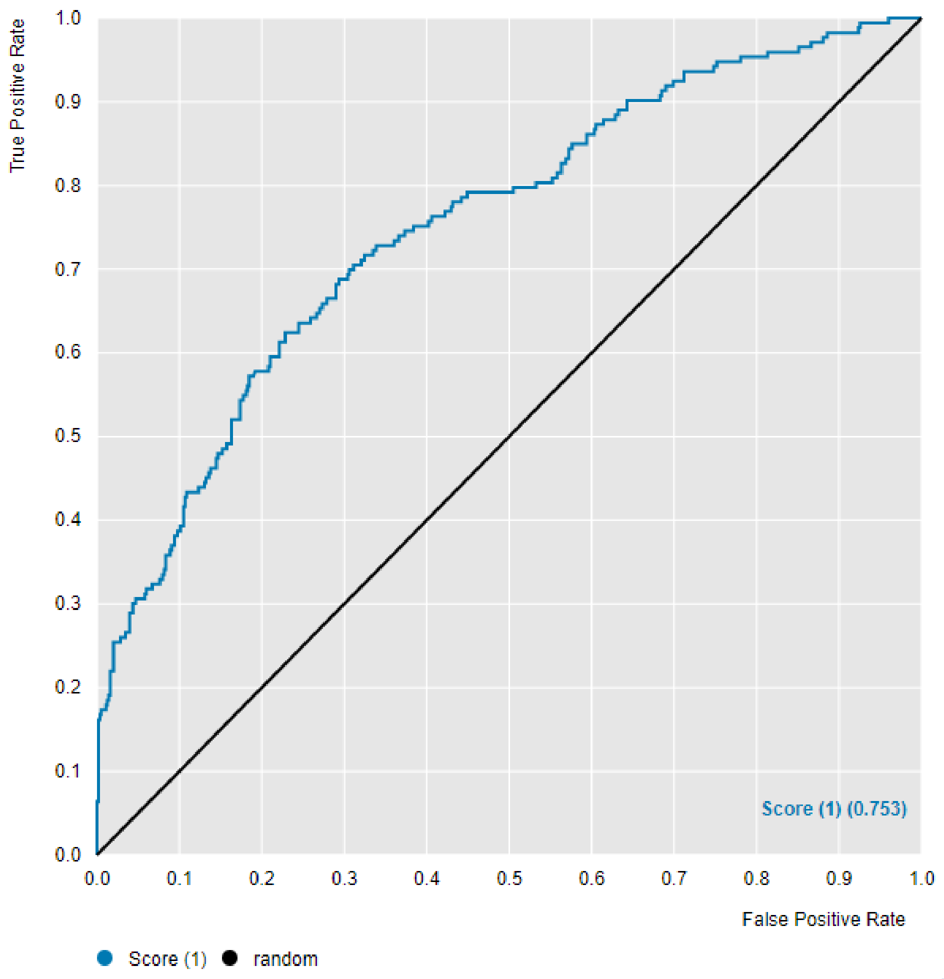

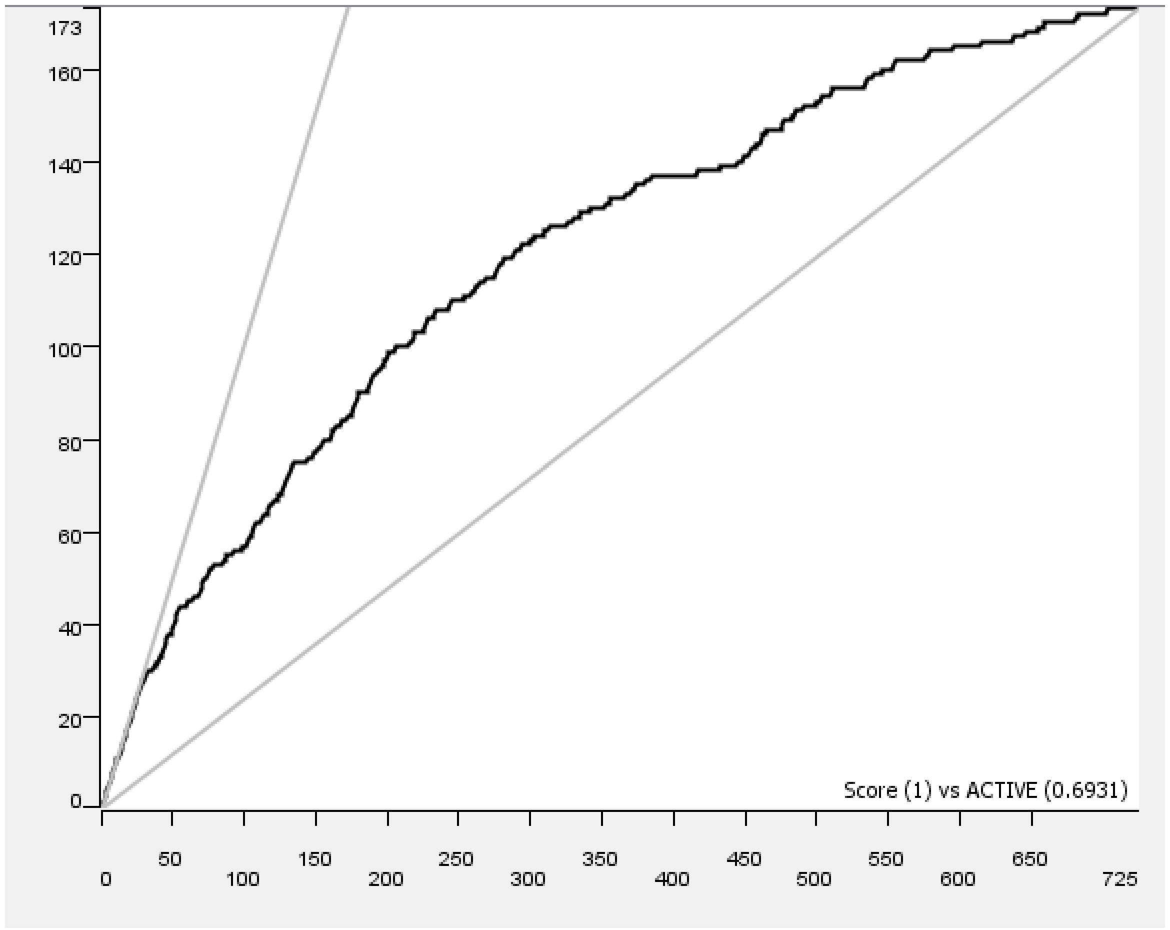

N=725

Top 10% Mean IC50	55.7 nM

	Predicted Active	Predicted Inactive
Active	128	45
Inactive	203	349

<15nM DefGood in JAK2, 30% error

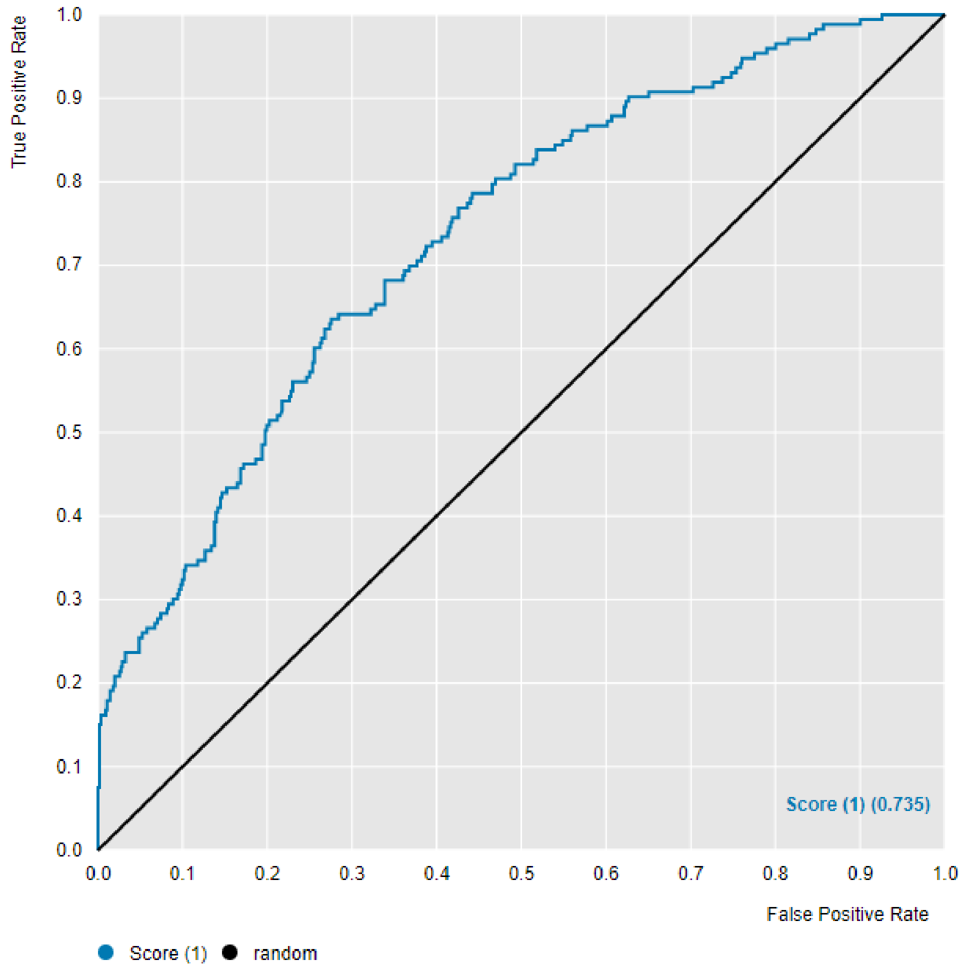

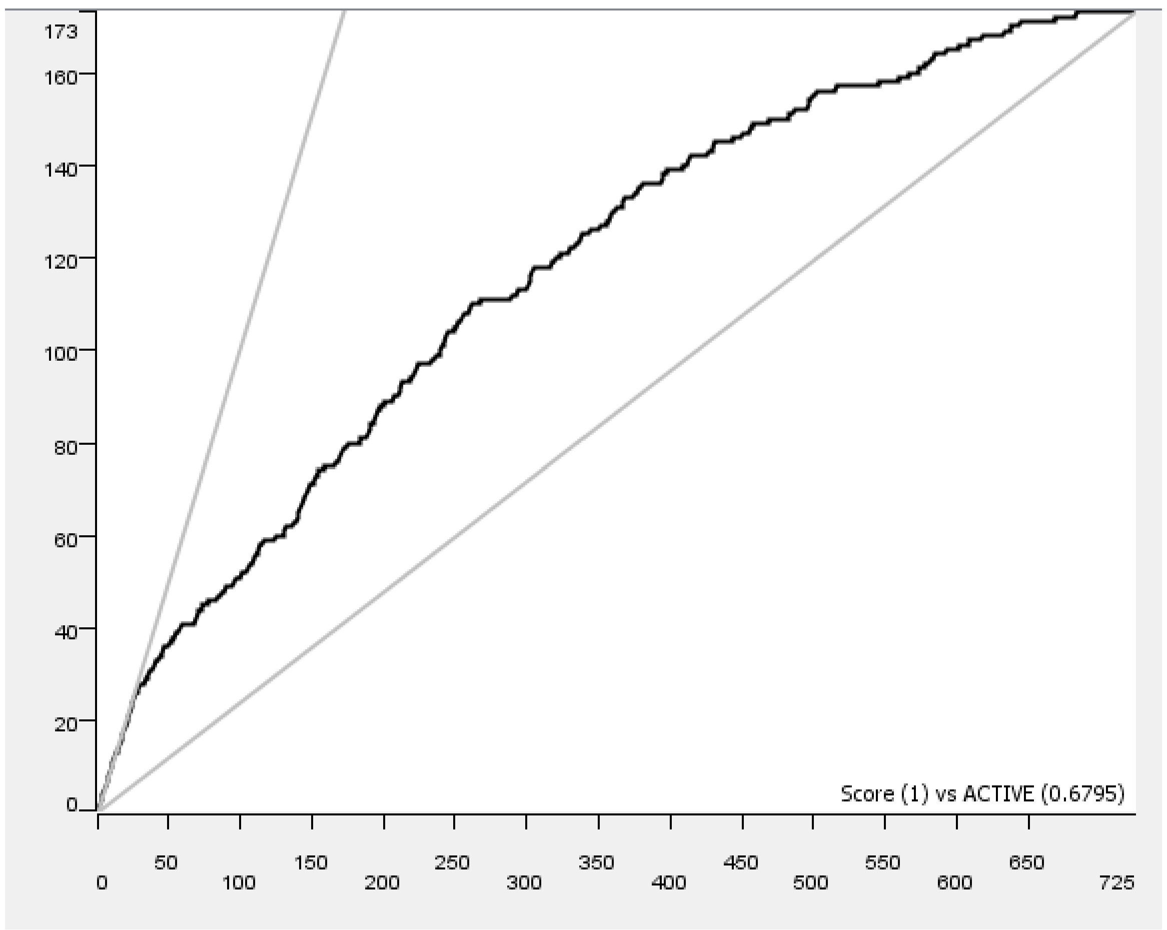

N=725

Top 10% Mean IC50	53.4 nM

	Predicted Active	Predicted Inactive
Active	109	64
Inactive	151	401

<15nM DefGood in JAK2, 35% error

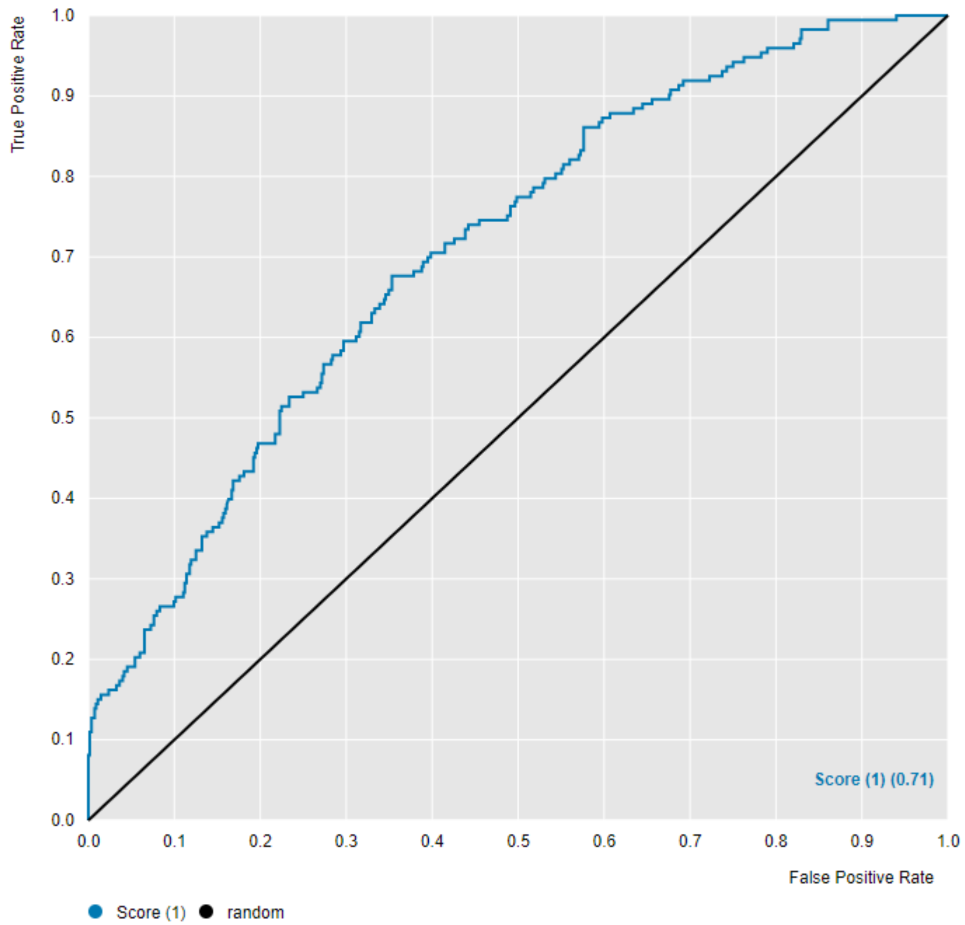

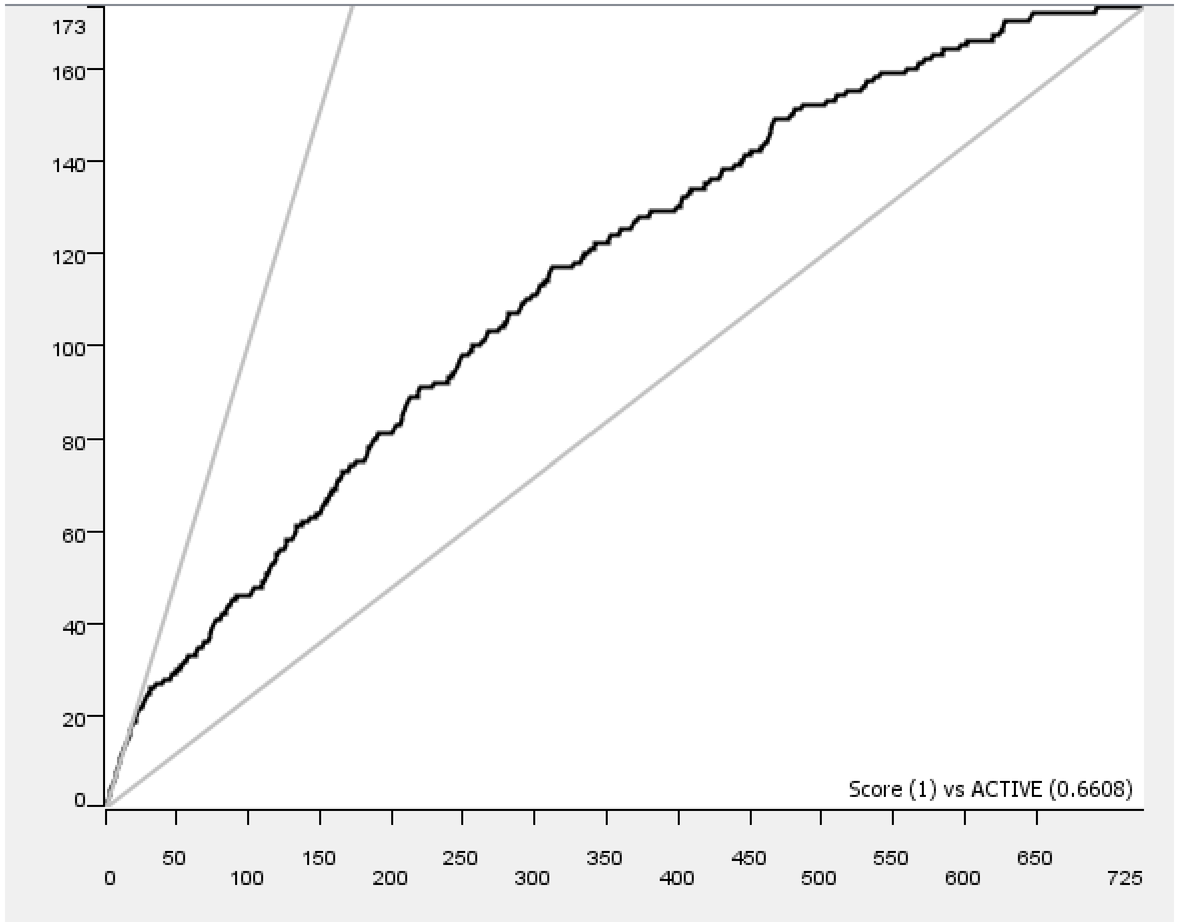

N=725

Top 10% Mean IC50	77.7 nM

	Predicted Active	Predicted Inactive
Active	136	37
Inactive	288	264

<15nM DefGood in JAK2, 40% error

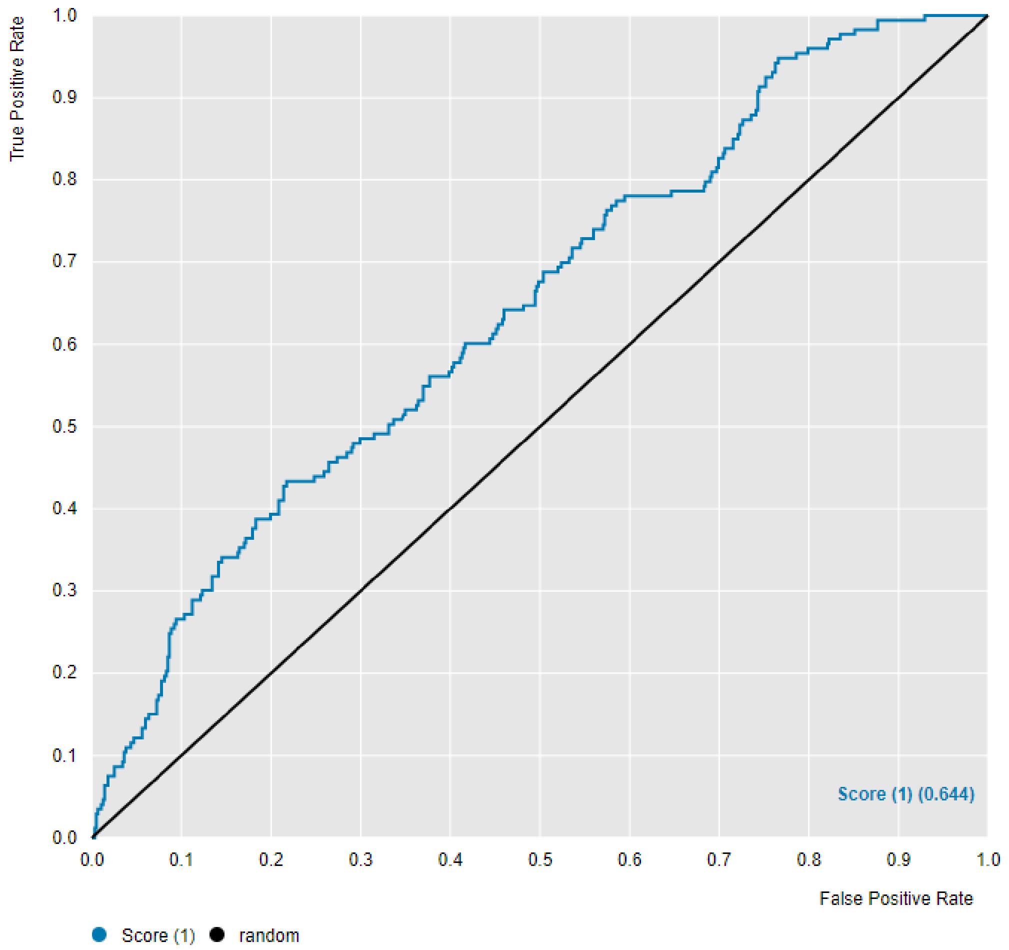

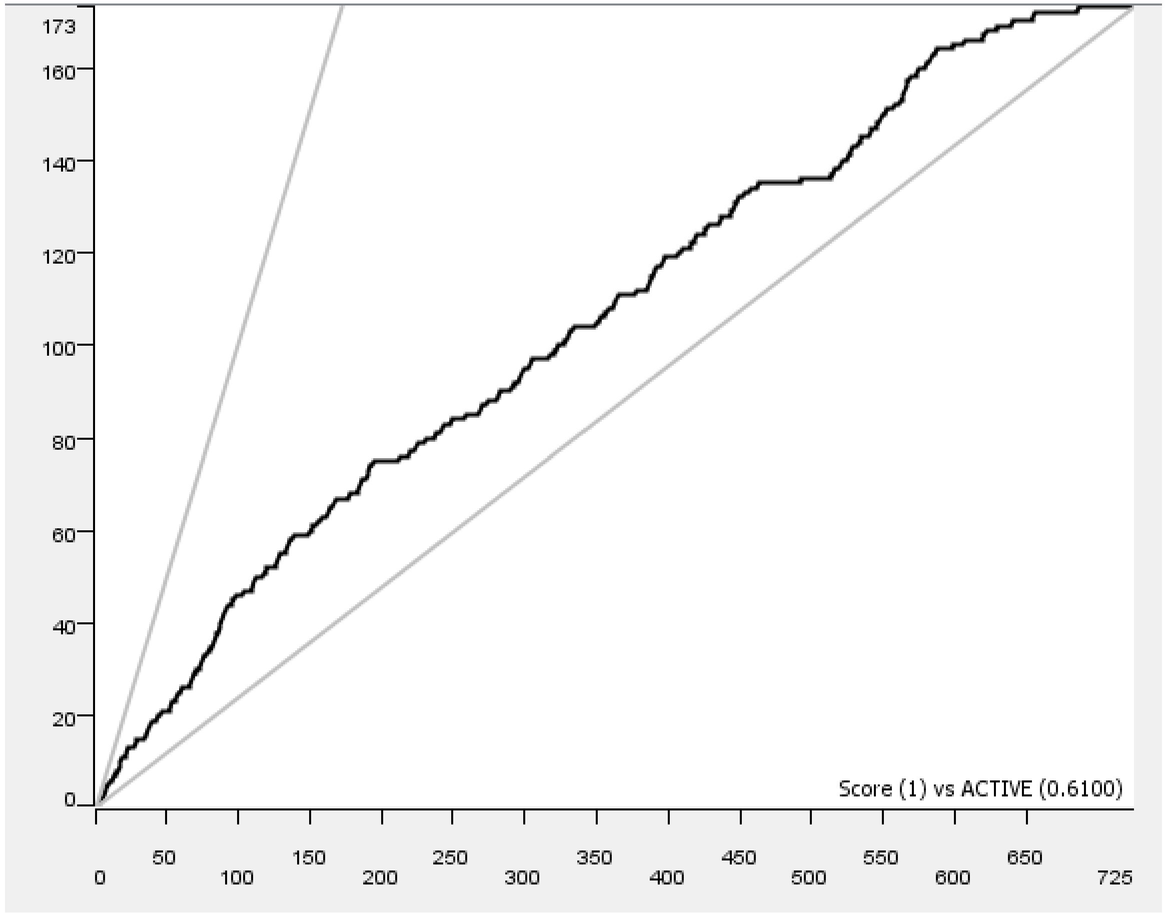

N=725

**Top 10% Mean IC50**	**131 nM**

	Predicted Active	Predicted Inactive
Active	75	98
Inactive	134	418

<15nM DefGood in JAK2, 10% error; Random seed = 429

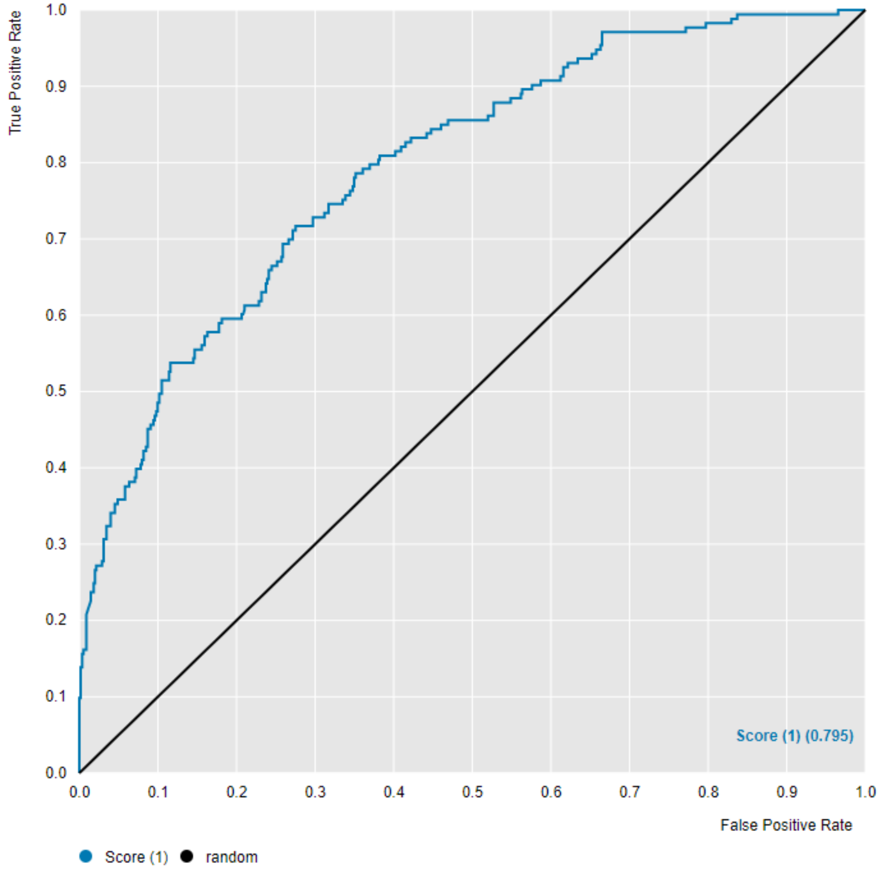

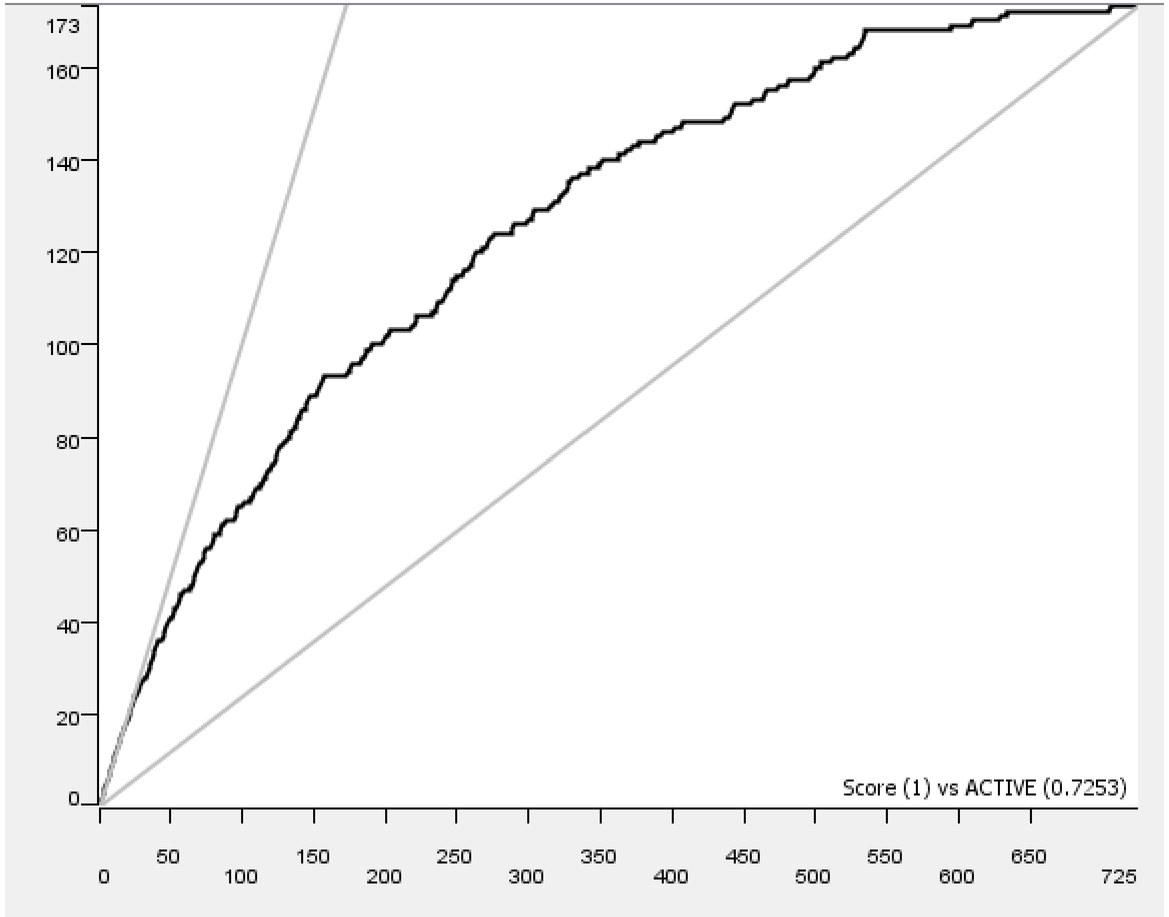

N=725

**Top 10% Mean IC50**	**71.9 nM**

	Predicted Active	Predicted Inactive
Active	109	64
Inactive	128	424

<15nM DefGood in JAK2, 20% error; Random seed = 429

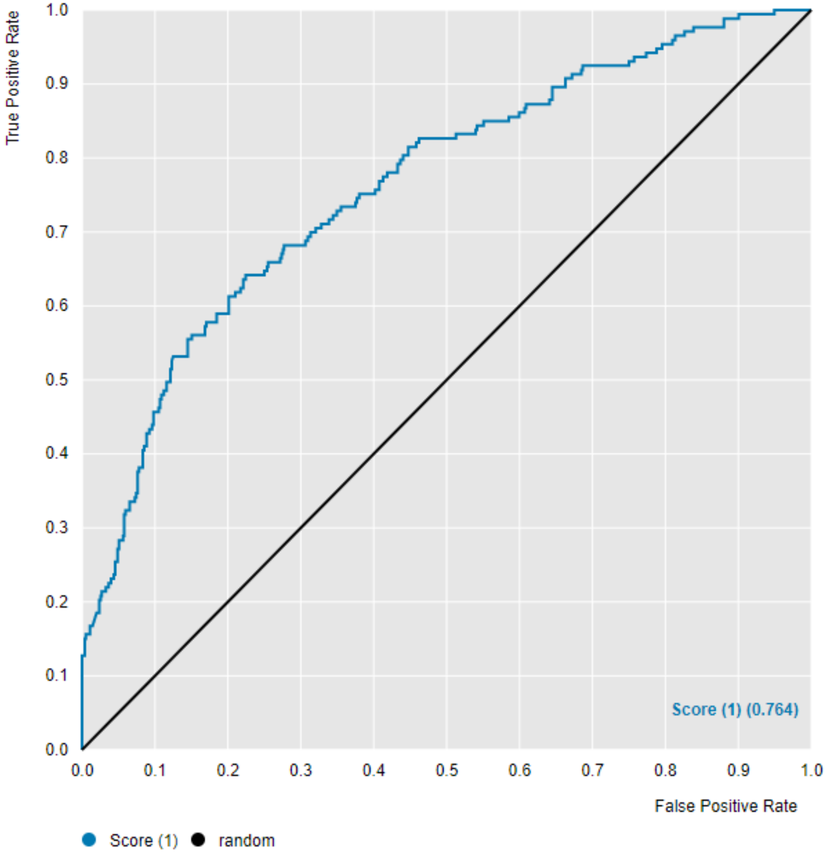

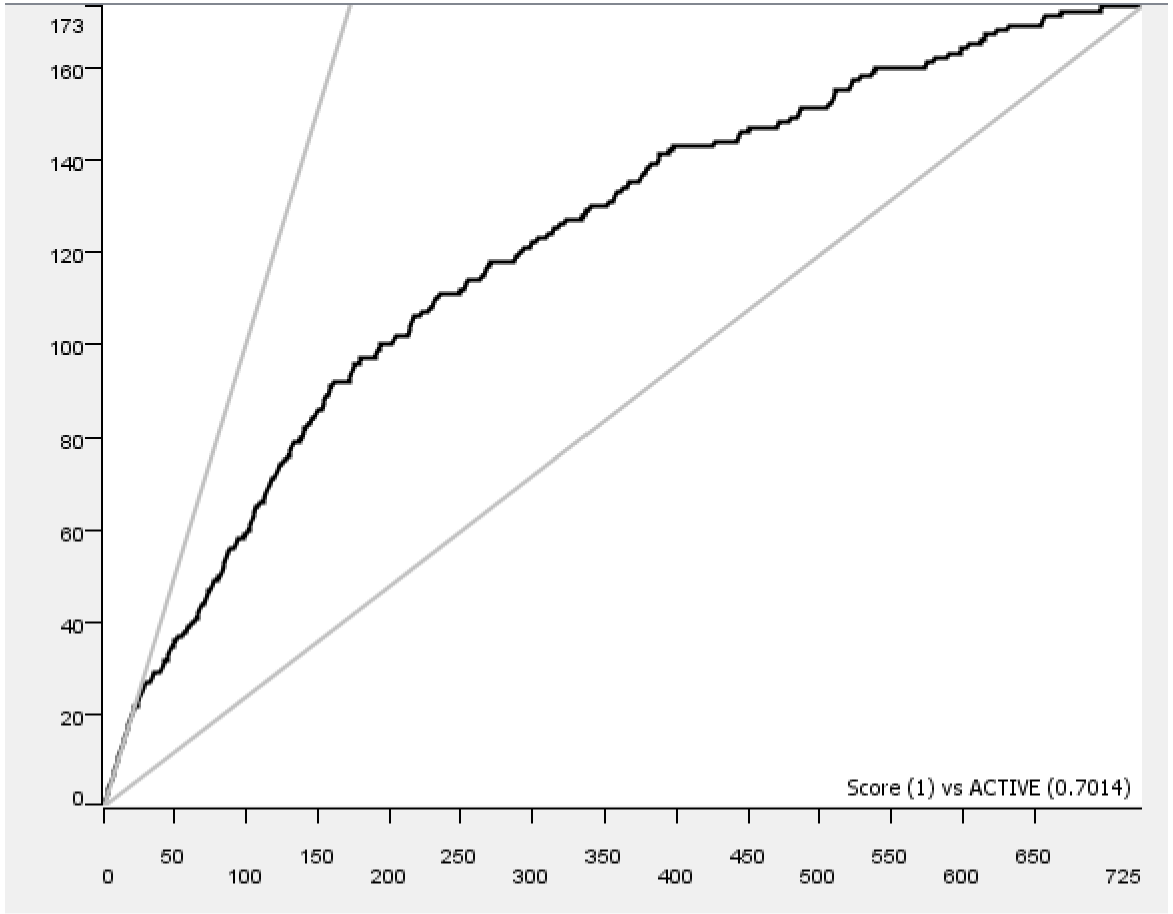

N=725

**Top 10% Mean IC50**	**419 nM**

	Predicted Active	Predicted Inactive
Active	118	55
Inactive	155	397

<15nM DefGood in JAK2, 25% error; Random seed = 429

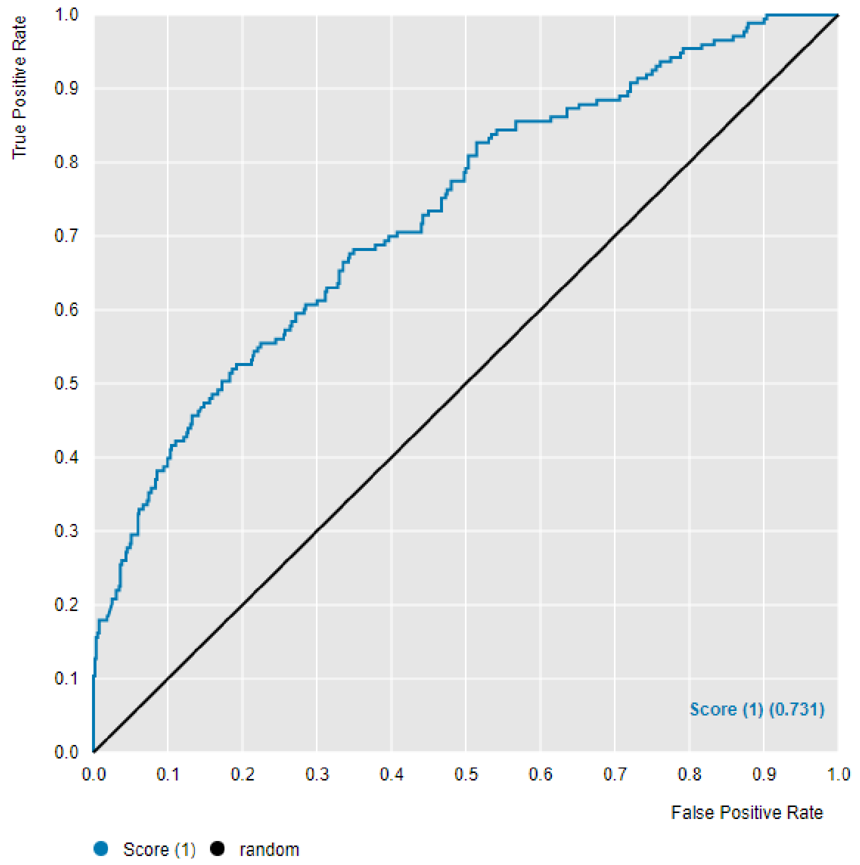

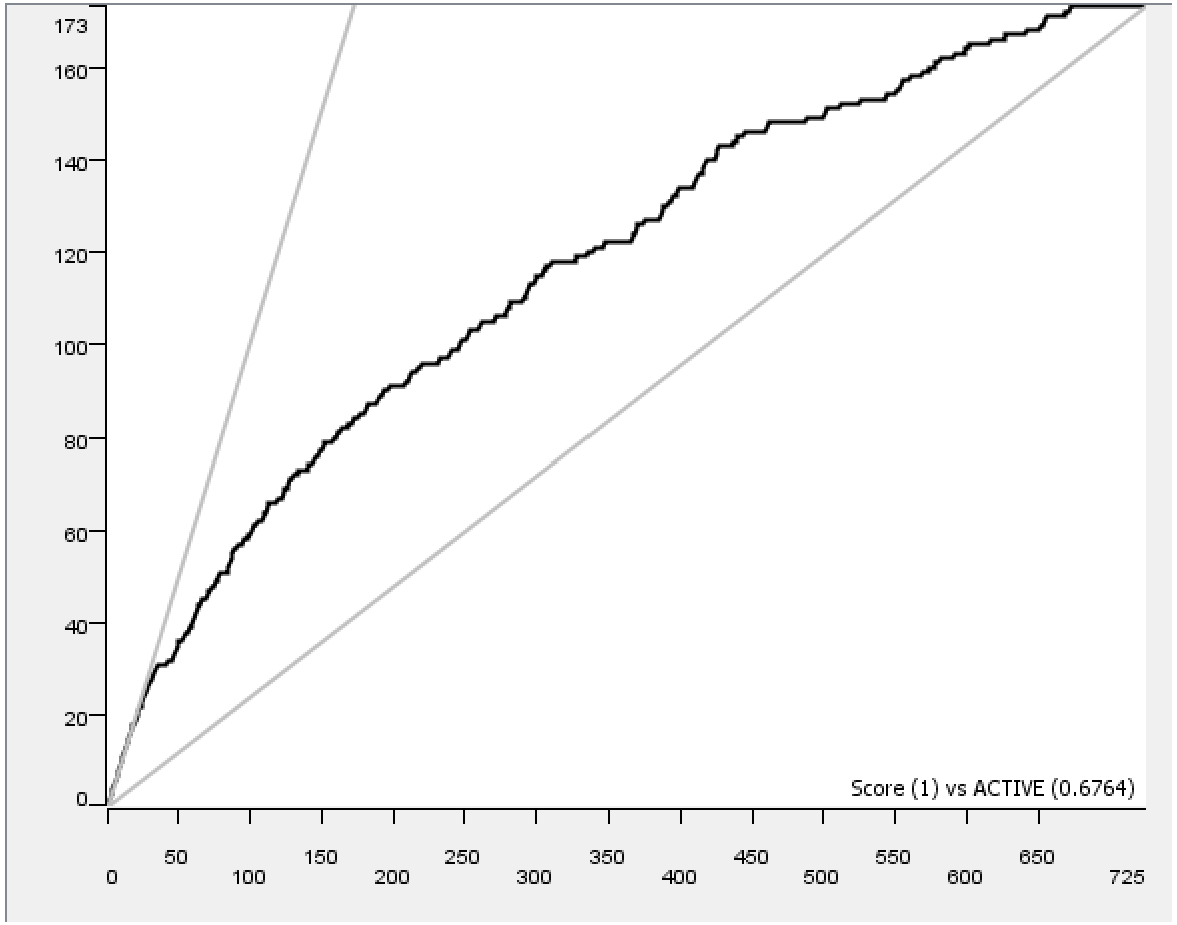

N=725

Top 10% Mean IC50	431 nM

	Predicted Active	Predicted Inactive
Active	77	96
Inactive	73	479

<5nM DefGood in JAK2, 30% error; Random seed = 429

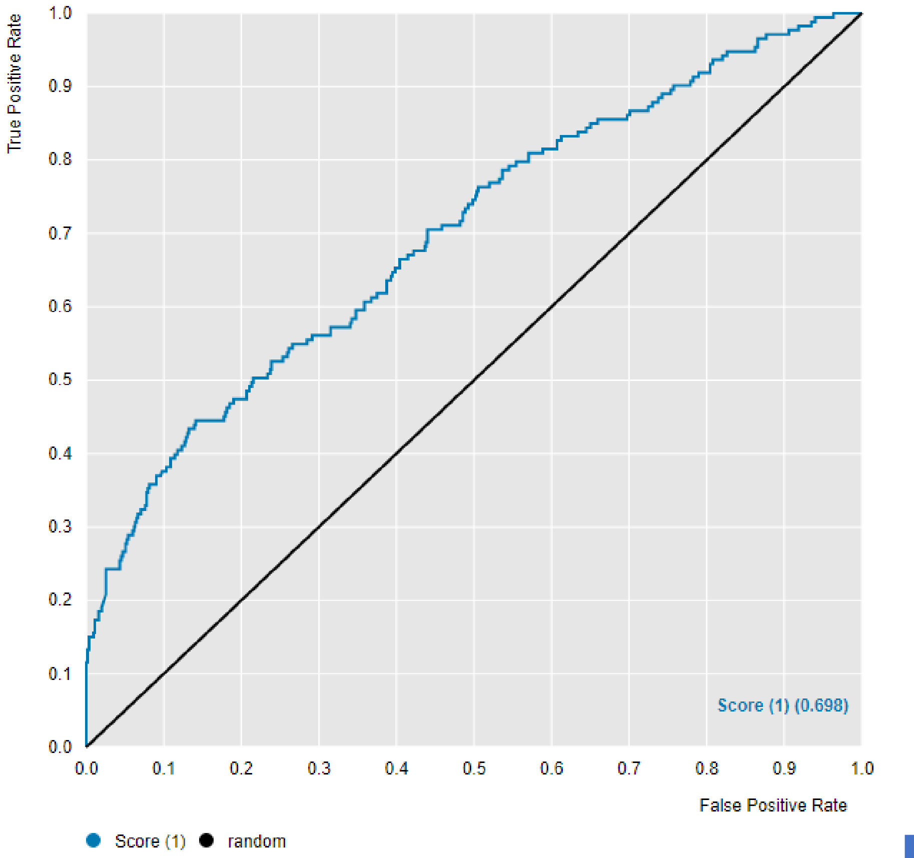

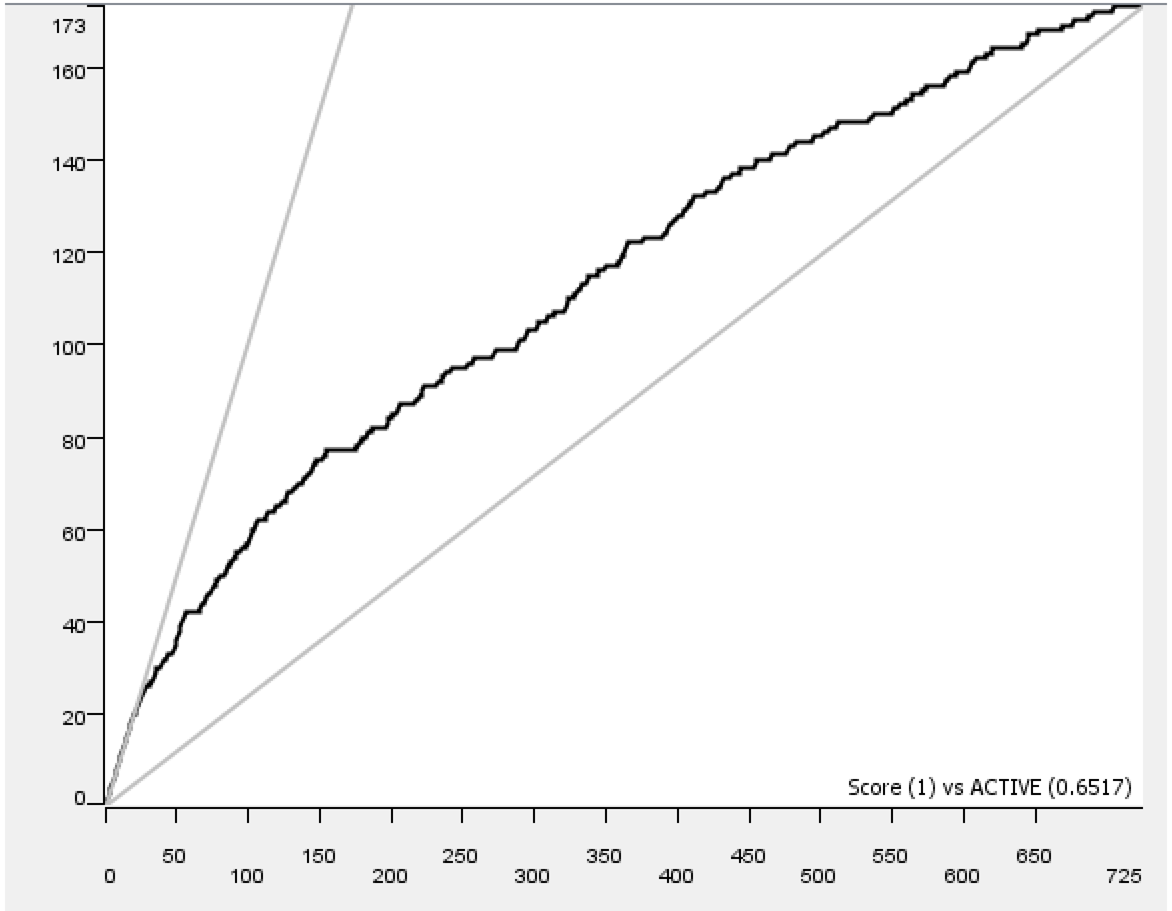

N=725

**Top 10% Mean IC50**	**418 nM**

	Predicted Active	Predicted Inactive
Active	97	76
Inactive	166	386

<15nM DefGood in JAK2, 10% error; Random seed = 121783

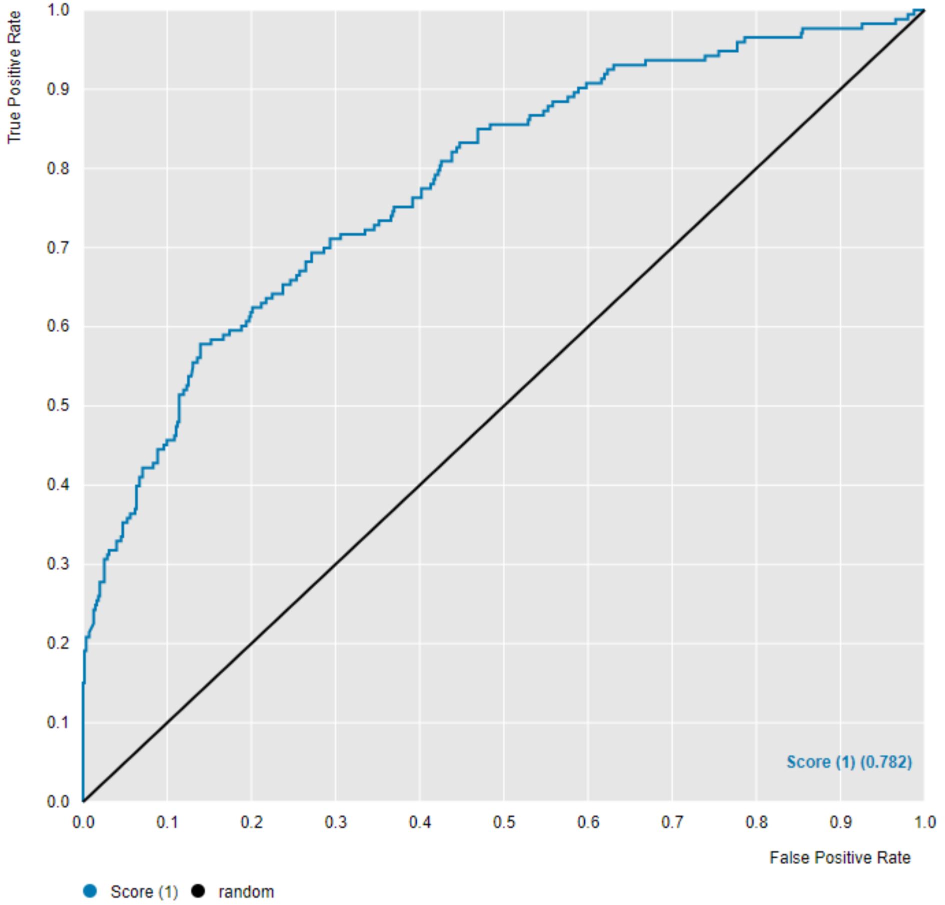

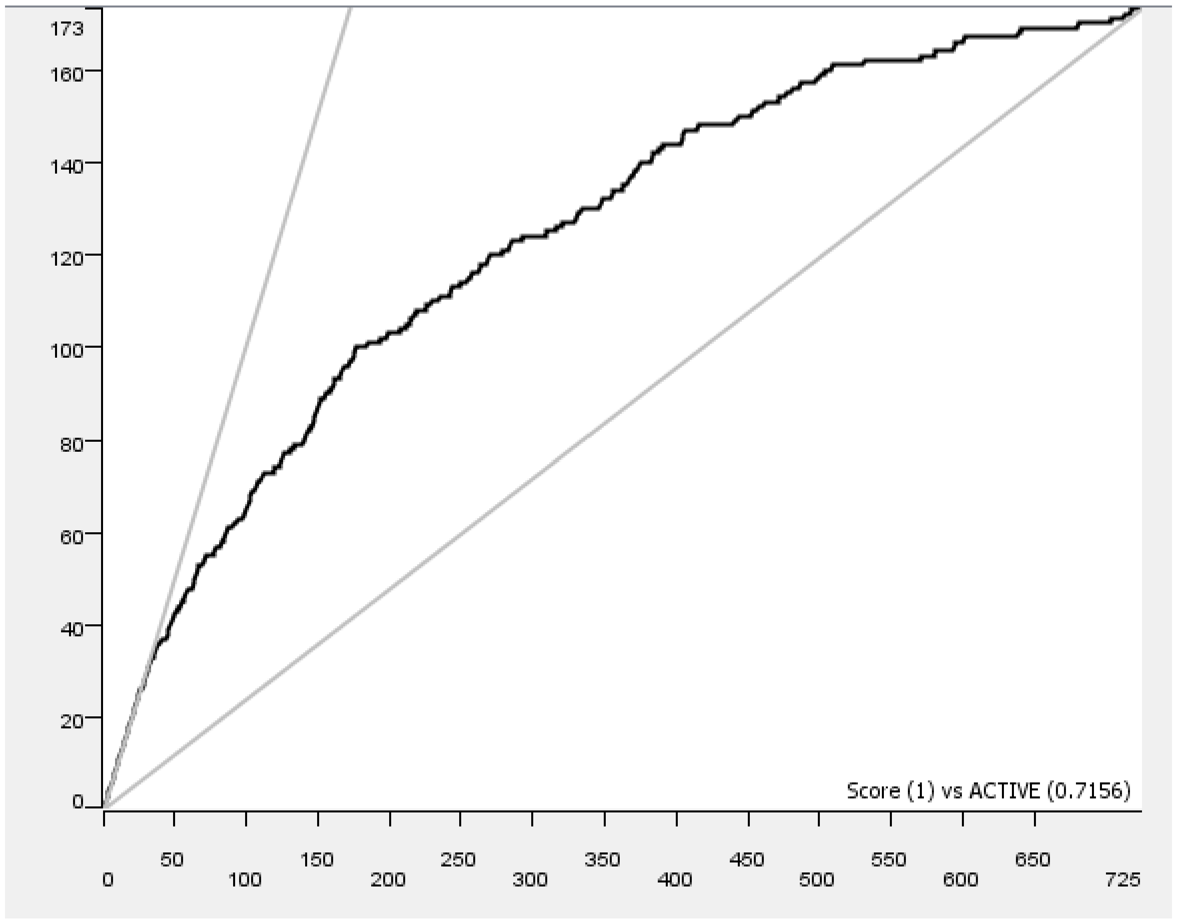

N=725

Top 10% Mean IC50	1,427 nM (4 micromolar mistakes)

	Predicted Active	Predicted Inactive
Active	134	39
Inactive	227	325

<15nM DefGood in JAK2, 0% error; Random seed = 121783

**Top 10% Mean IC50**	**1,427 nM<br>(4 micromolar mistakes)<br><br>This sample was discarded**

N=725

	Predicted Active	Predicted Inactive
Active	134	39
Inactive	227	325

<15nM DefGood in JAK2, 10% error; Random seed = 12178

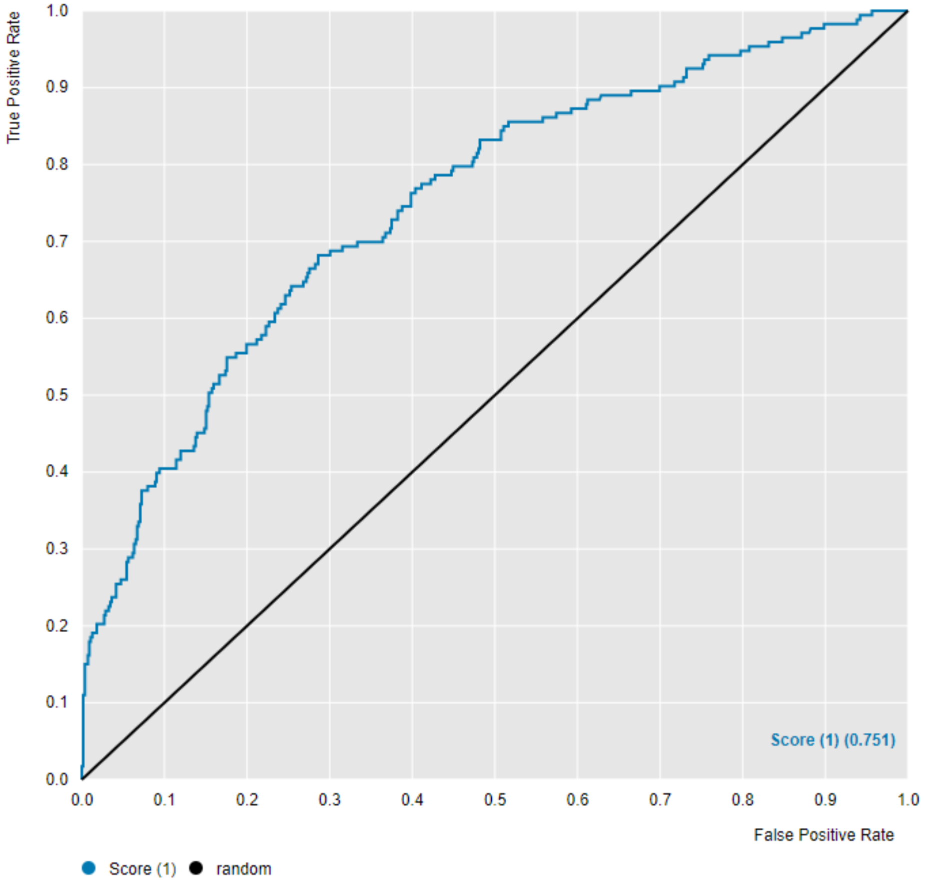

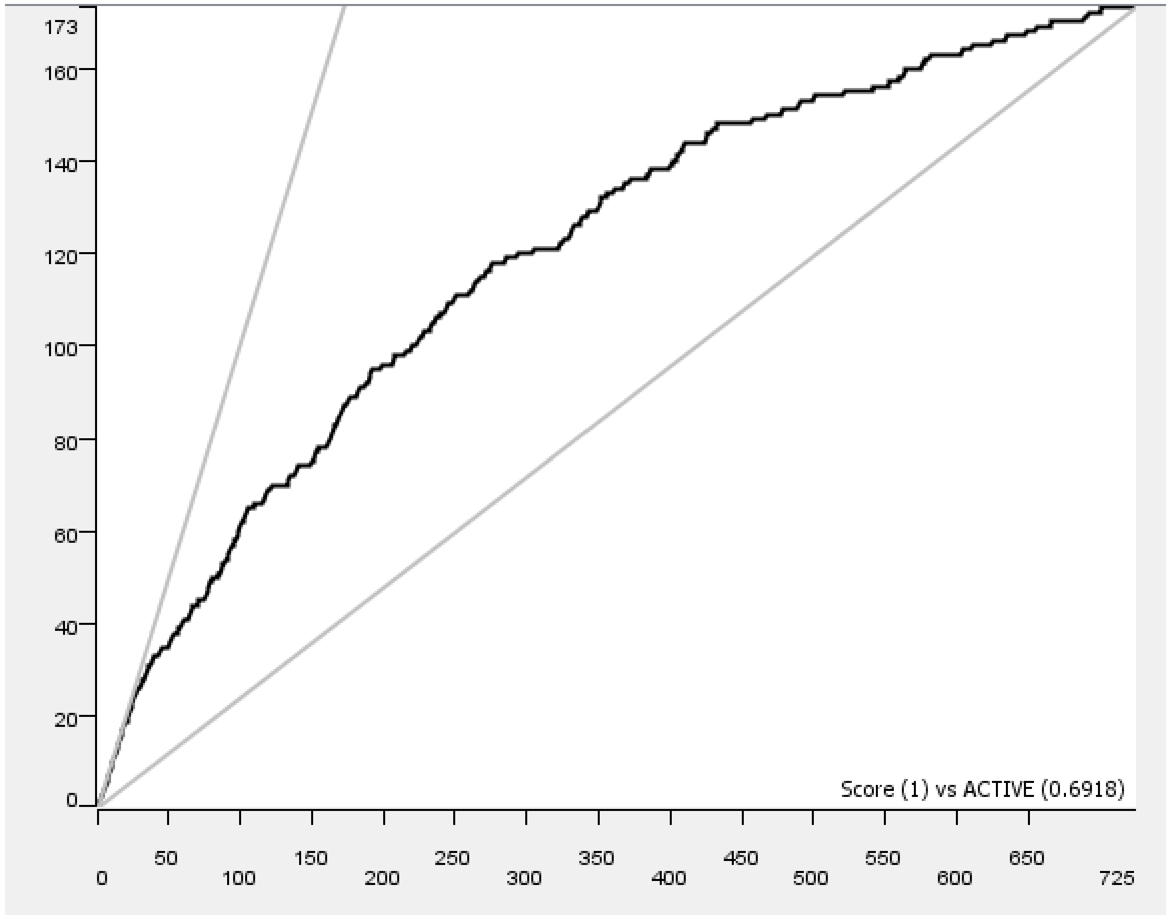

N=725

**Top 10% Mean IC50**	**65.7 nM**

	**Predicted Active**	**Predicted Inactive**
Active	112	61
Inactive	148	404

<15nM DefGood in JAK2, 20% error; Random seed = 12178

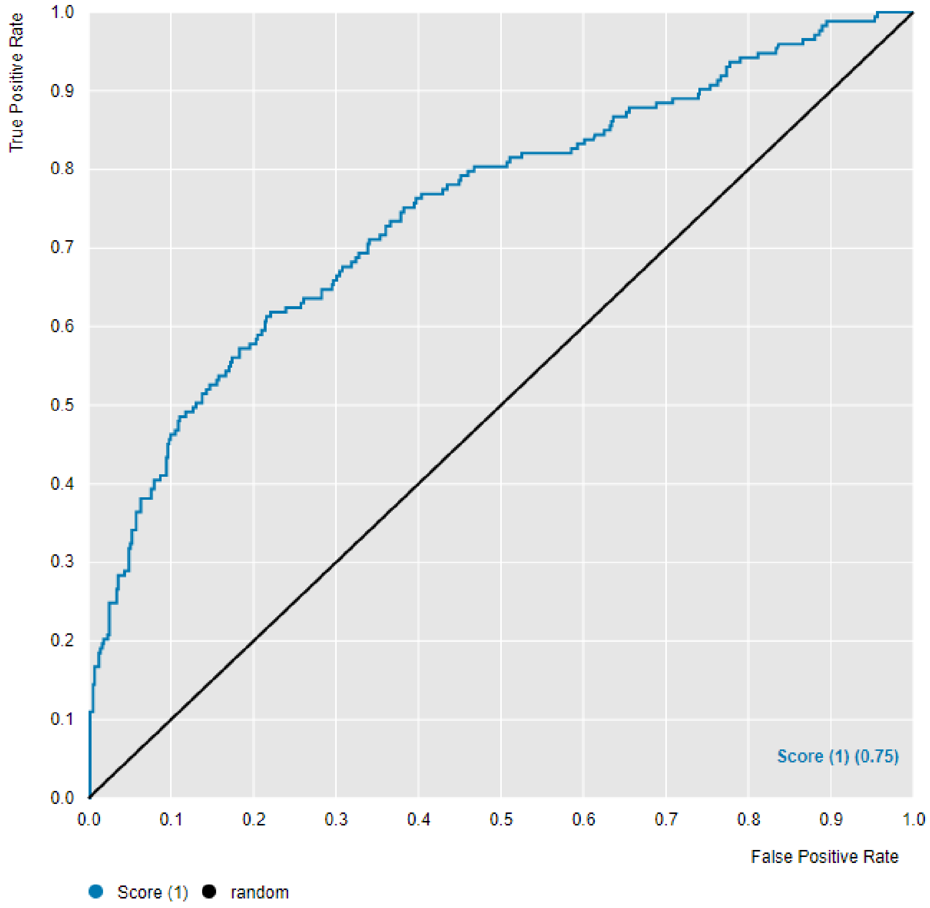

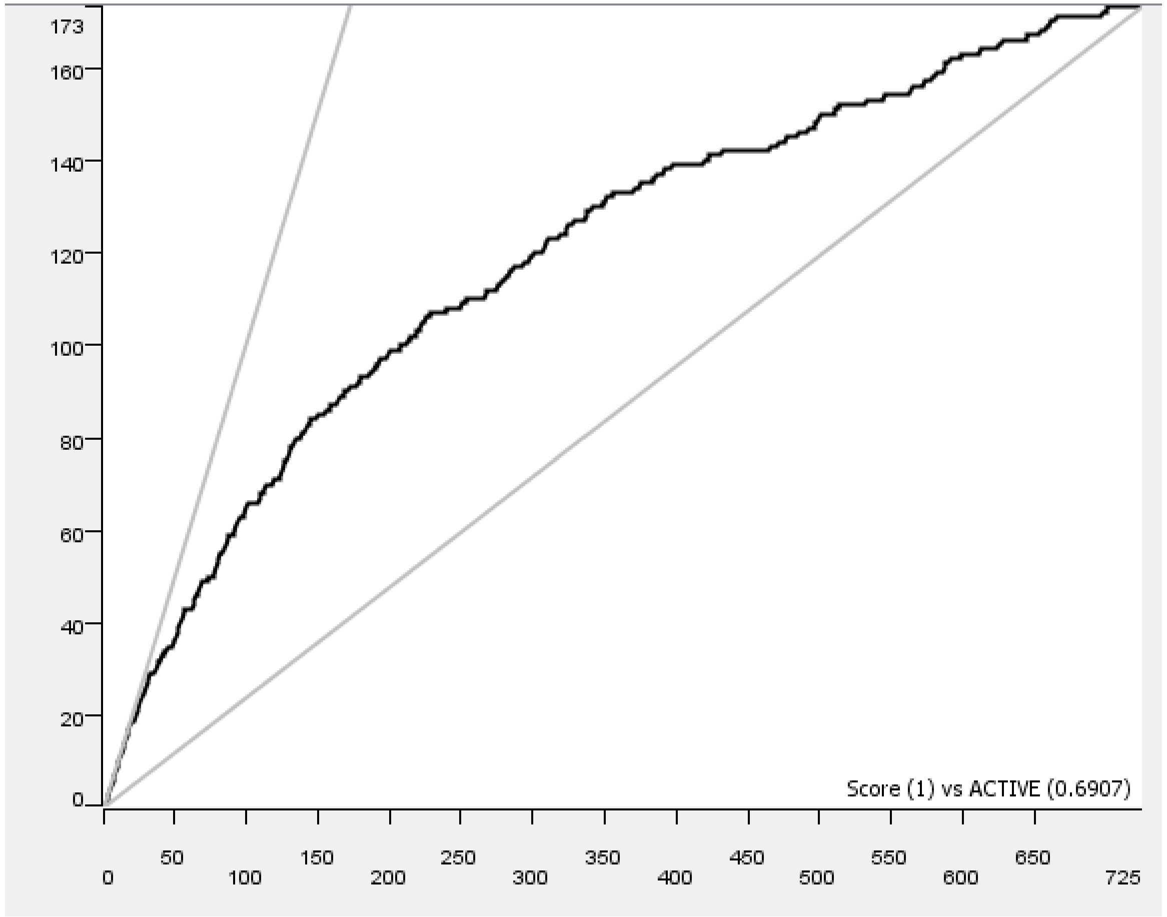

N=725

Top 10% Mean IC50	50.6 nM

	Predicted Active	Predicted Inactive
Active	100	73
Inactive	109	443

<15nM DefGood in JAK2, 30% error; Random seed = 12178

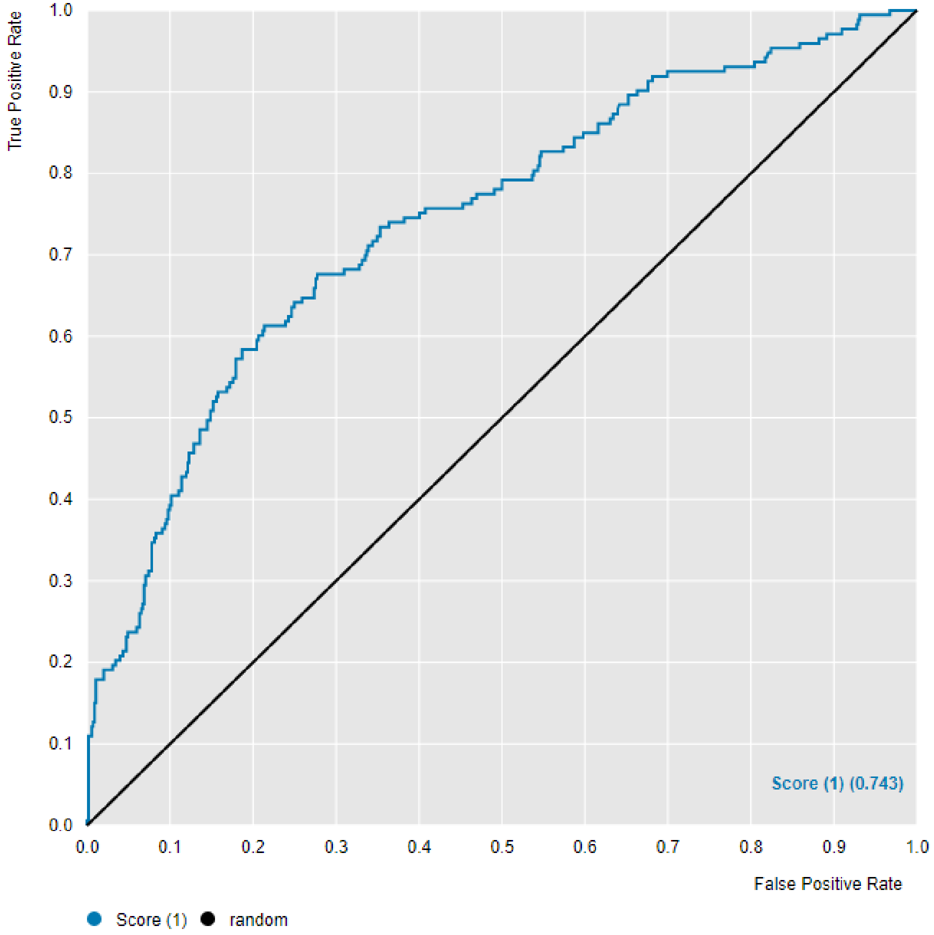

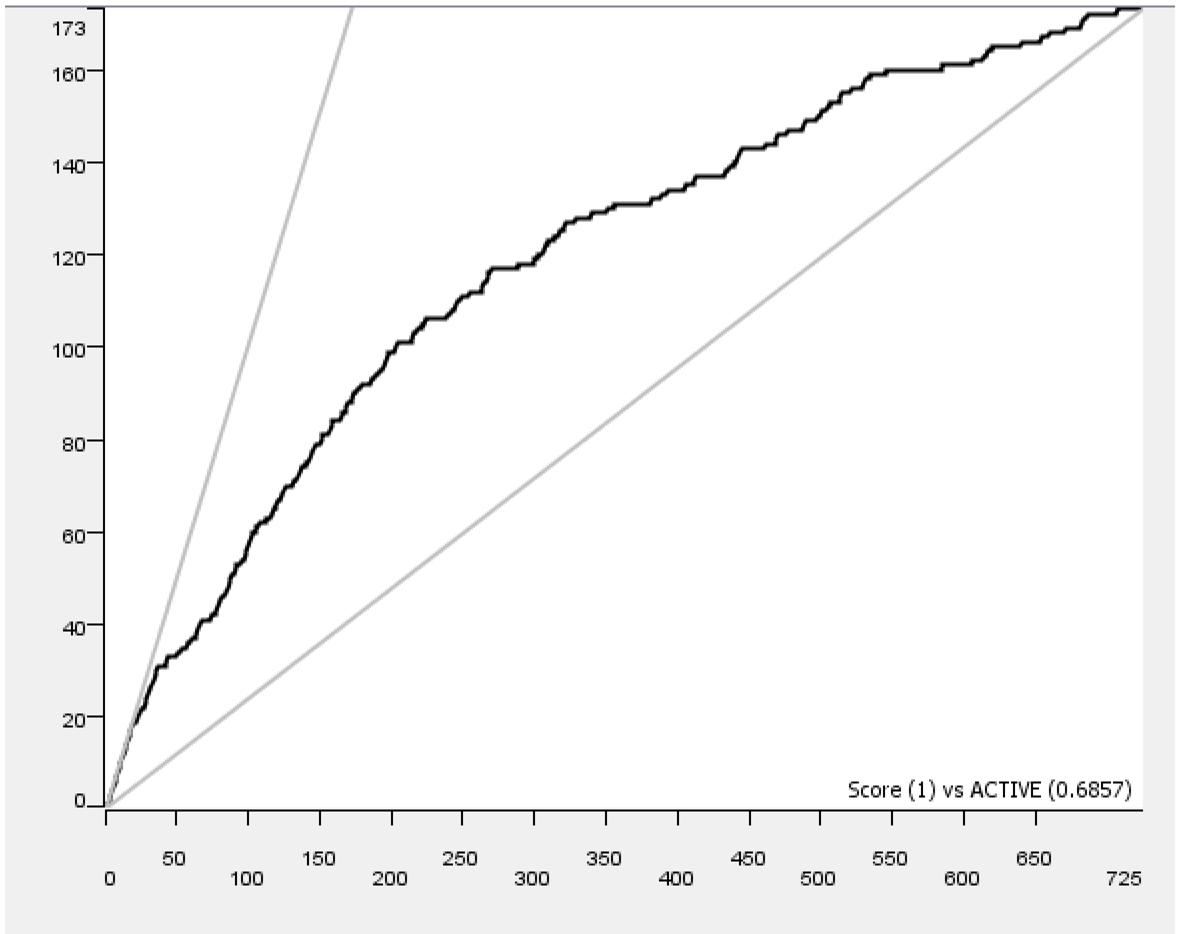

N=725

Top 10% Mean IC50	136 nM

	Predicted Active	Predicted Inactive
Active	122	51
Inactive	187	365

<15nM DefGood in JAK2, 35% error; Random seed = 12178

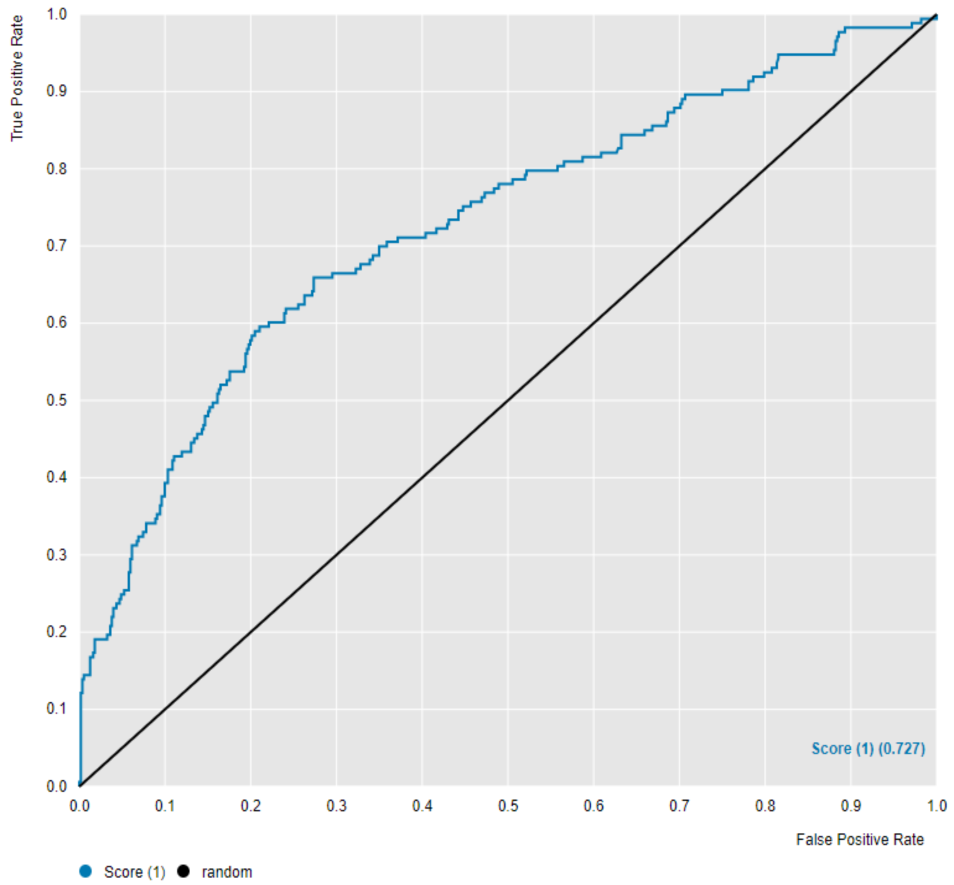

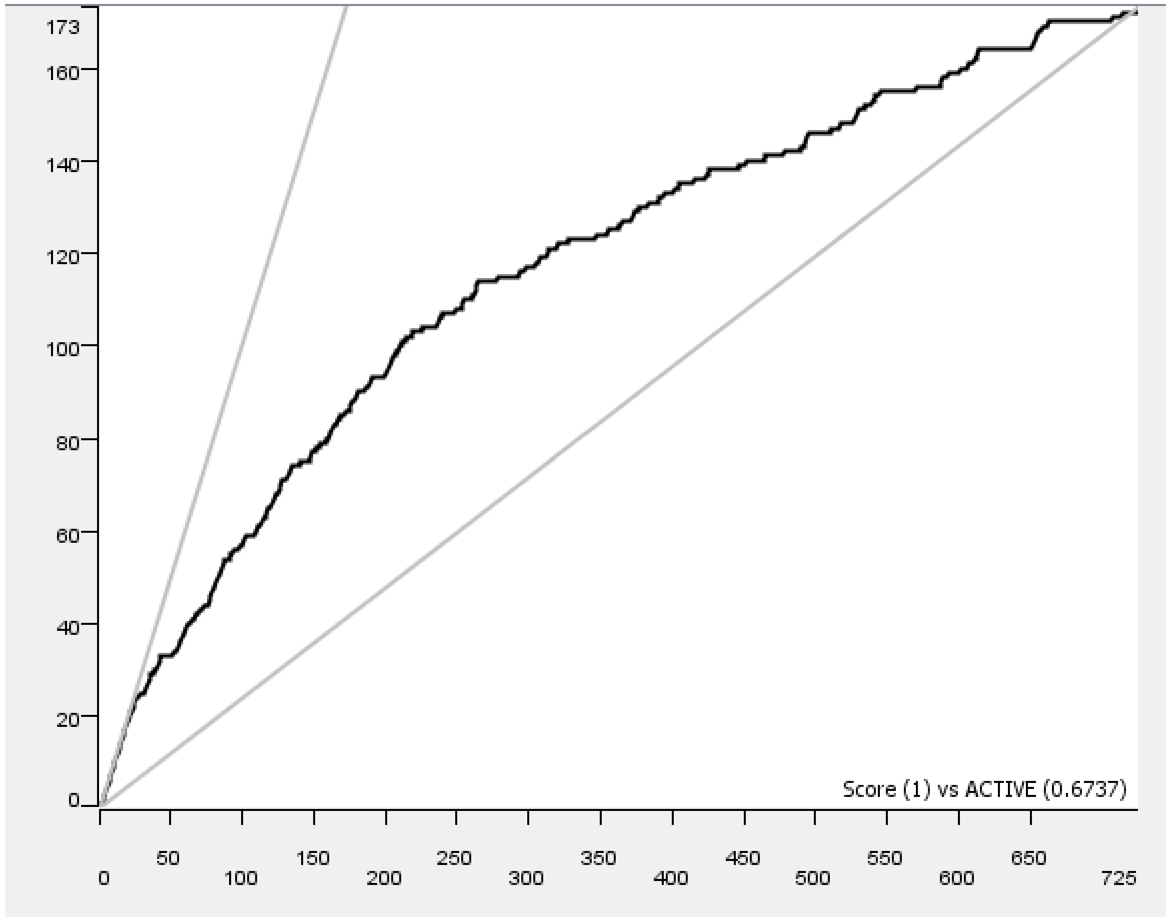

N=725

Top 10% Mean IC50	117 nM

	Predicted Active	Predicted Inactive
Active	106	67
Inactive	132	420

<15nM DefGood in JAK2, 40% error; Random seed = 121783

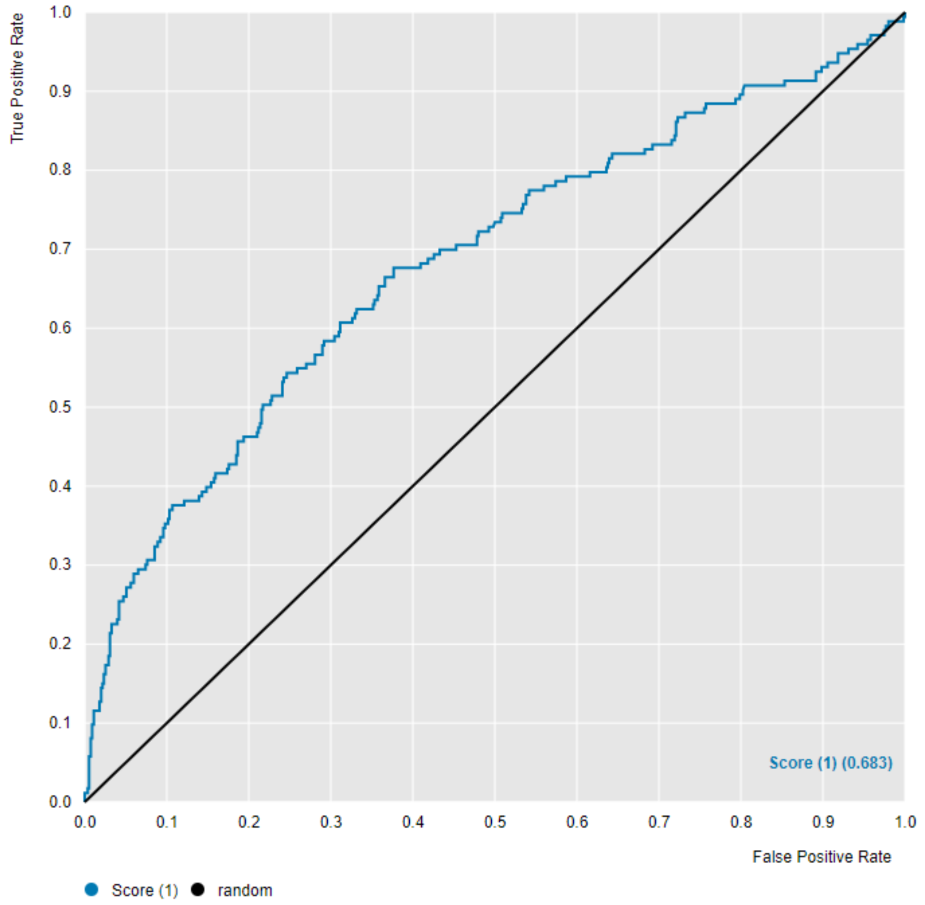

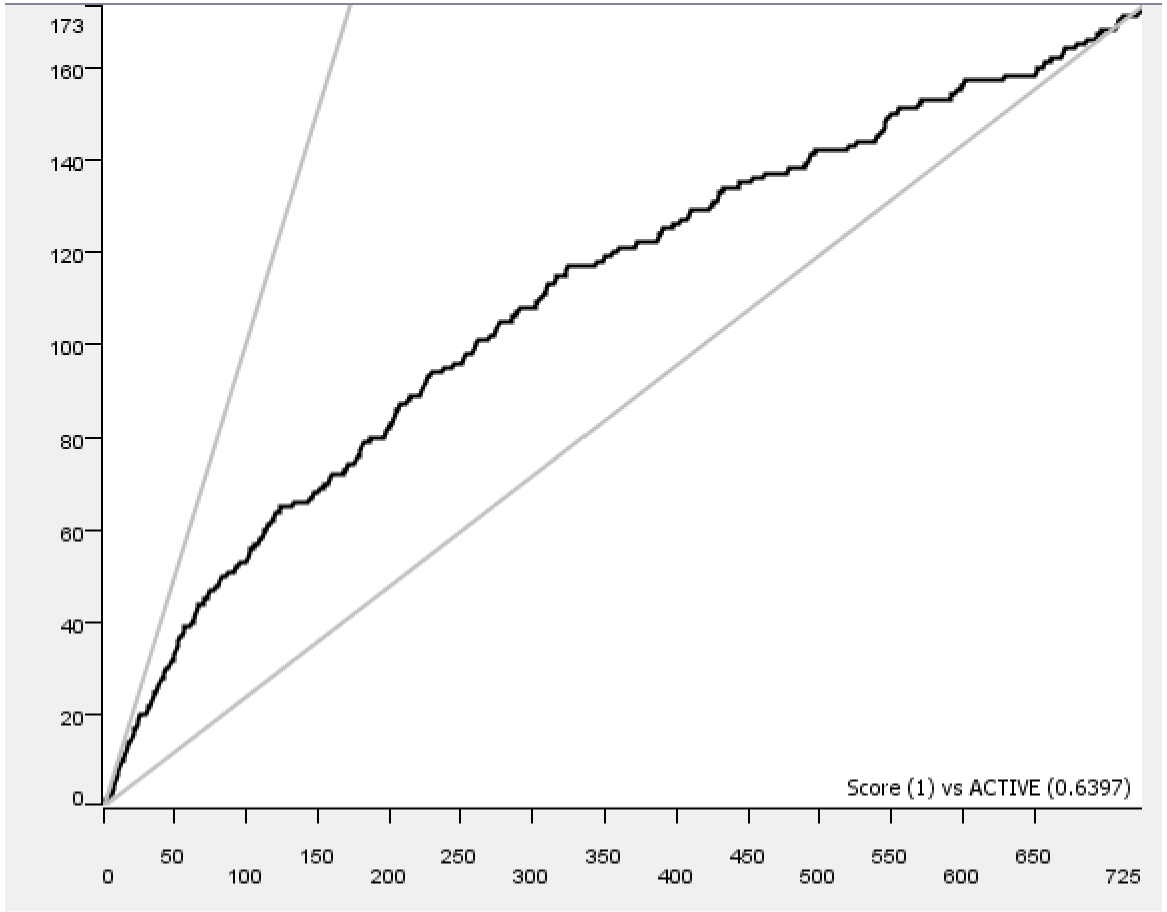

N=725

Top 10% Mean IC50	141 nM

	Predicted Active	Predicted Inactive
Active	95	78
Inactive	143	408

Conclusion - NBN

- An NBN could be generated for JAK2 kinase with a decision value of <5 nM as defined as active. The predictivity of the algorithm could be preserved in three random train/test cycles. The point of failure was 40%, 30% and 40% error.
- Importantly, the split with a random seed of 121783 had a catastrophic mistake from the very beginning without error. Another random seed was used instead

RF Error Tolerance- <5 nM DefGood in JAK2

- 0-50% absolute error

RF- <15nM DefGood in JAK2, 10% error

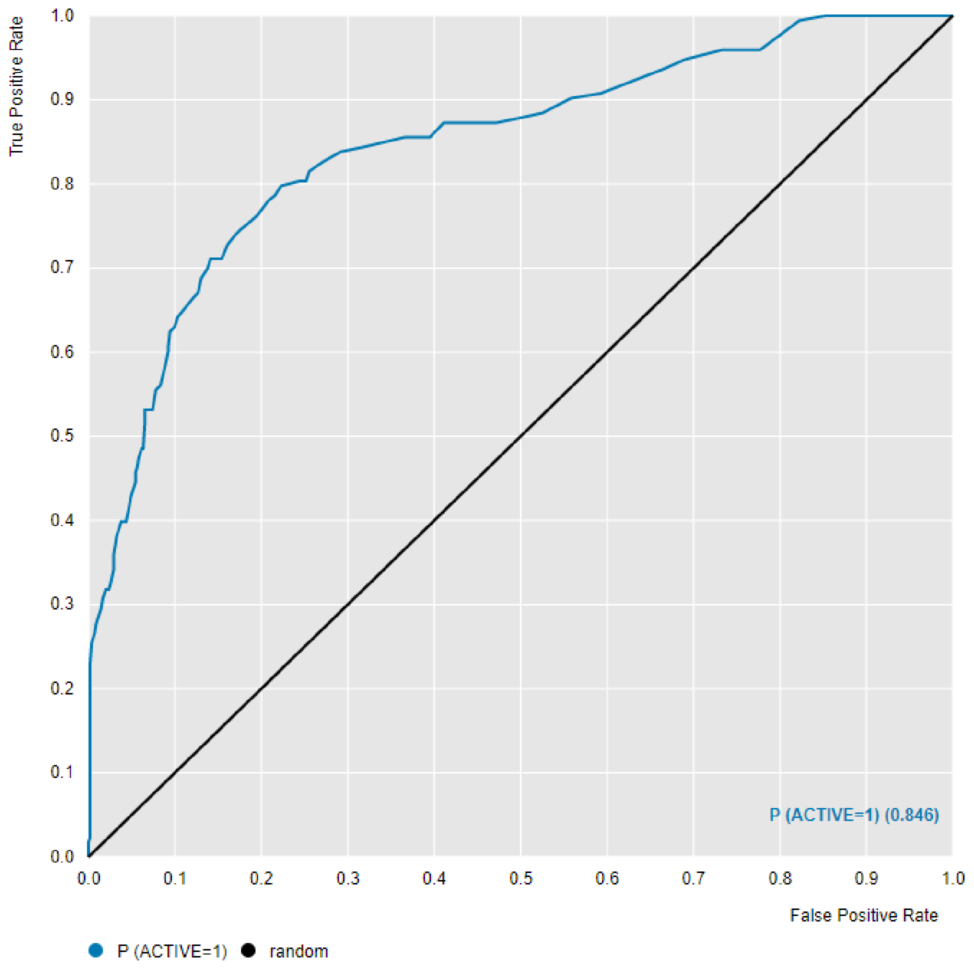

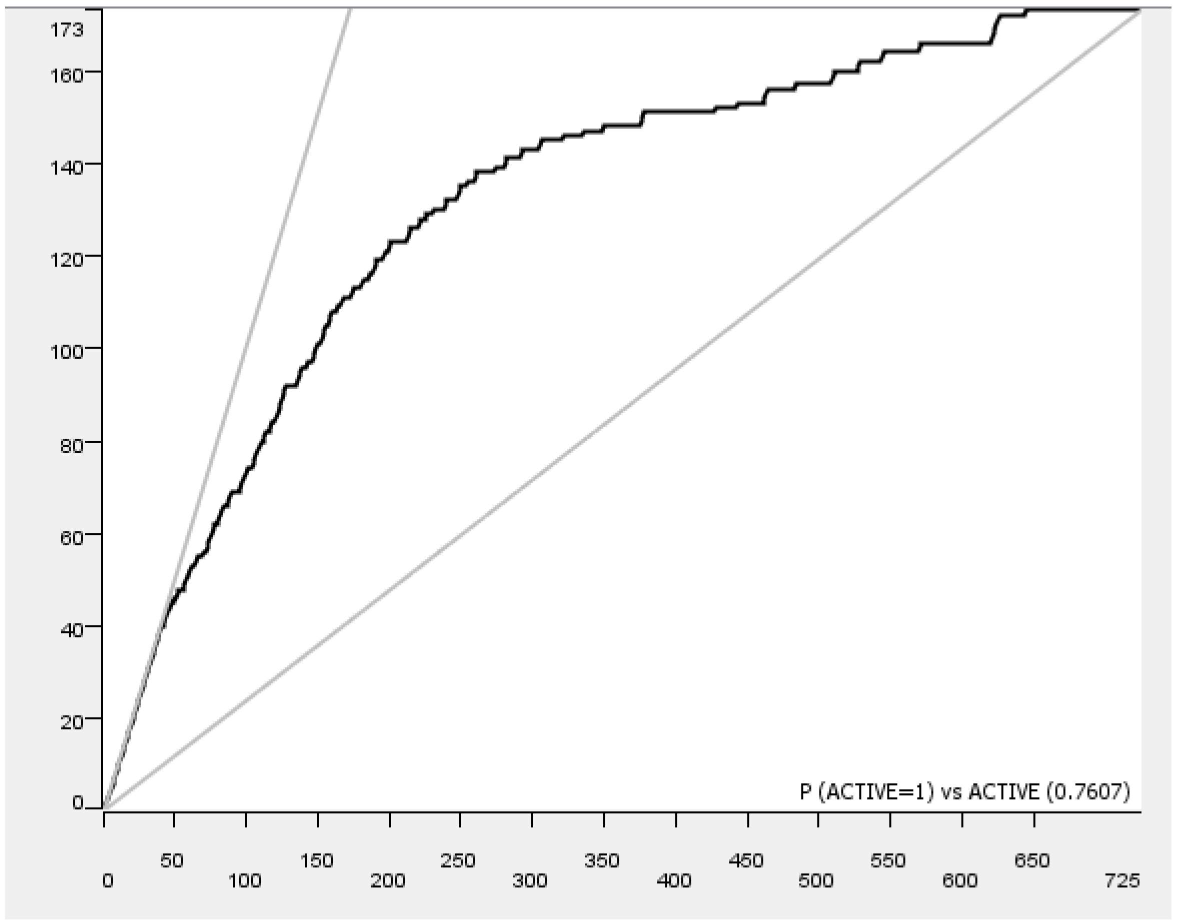

N=725

Top 10% Mean IC50	50.3 nM

	Predicted Active	Predicted Inactive
Active	96	77
Inactive	43	509

RF- <15nM DefGood in JAK2, 20% error

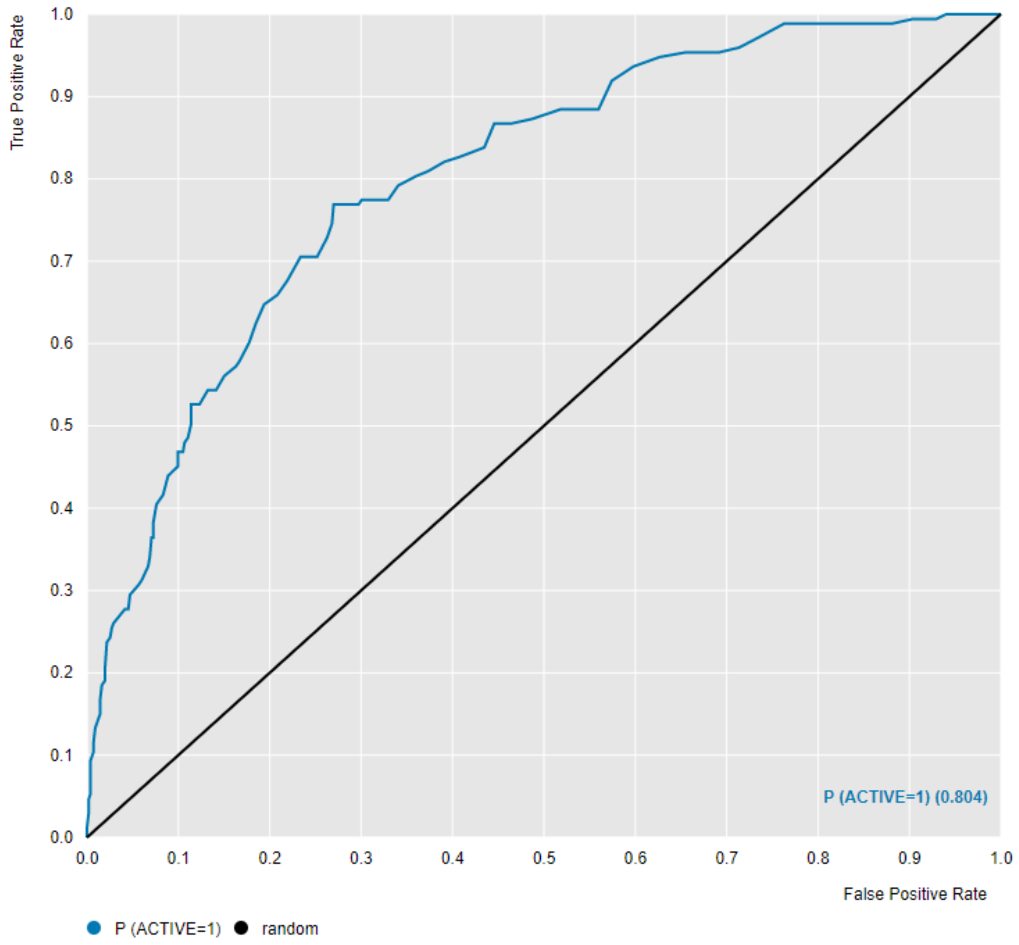

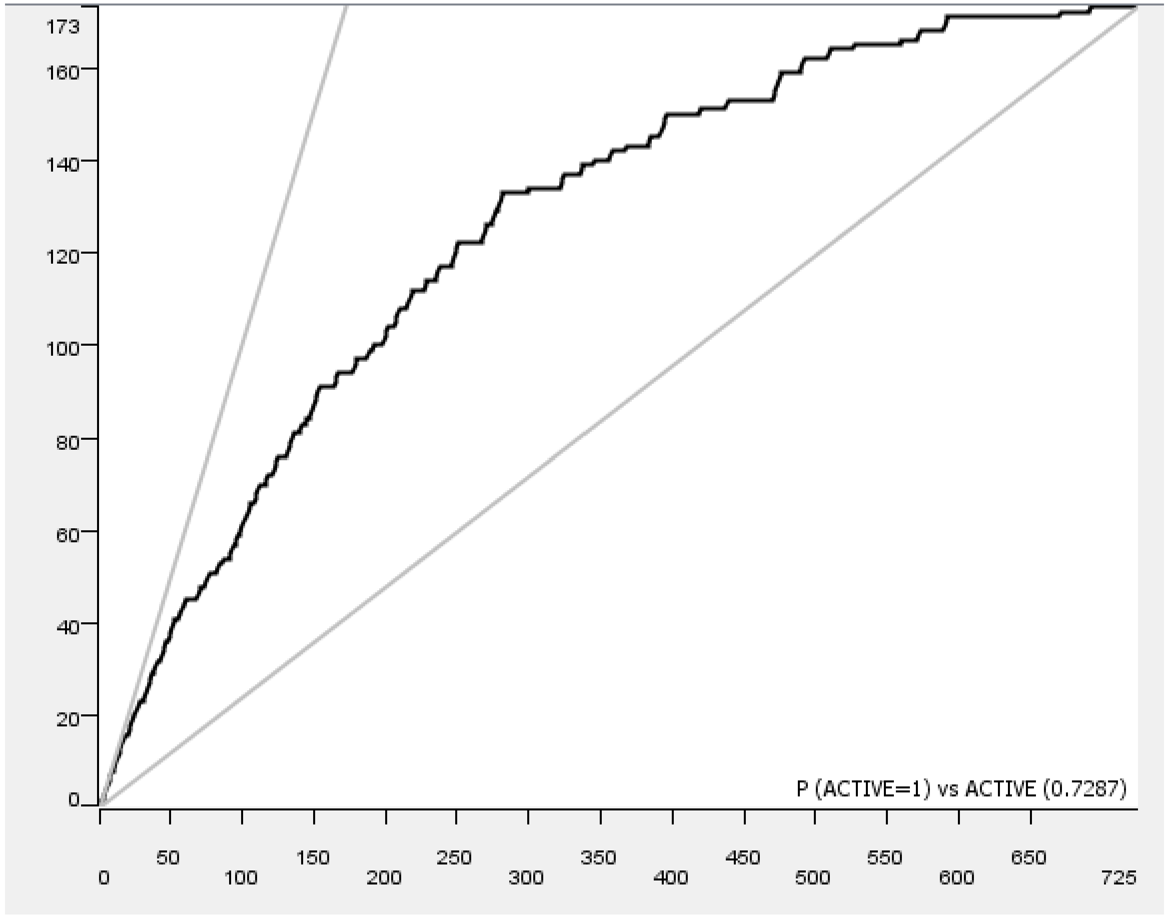

N=725

**Top 10% Mean IC50**	**139 nM**

	Predicted Active	Predicted Inactive
Active	91	82
Inactive	63	489

RF- <15nM DefGood in JAK2, 25% error

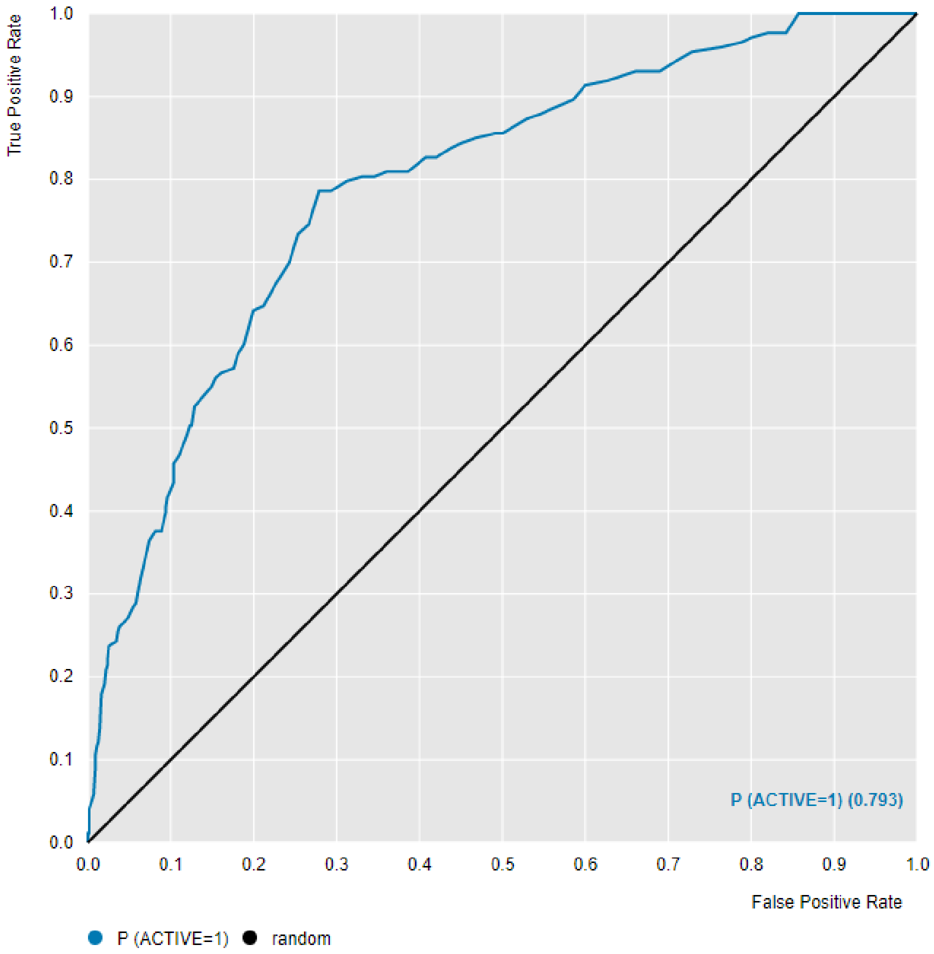

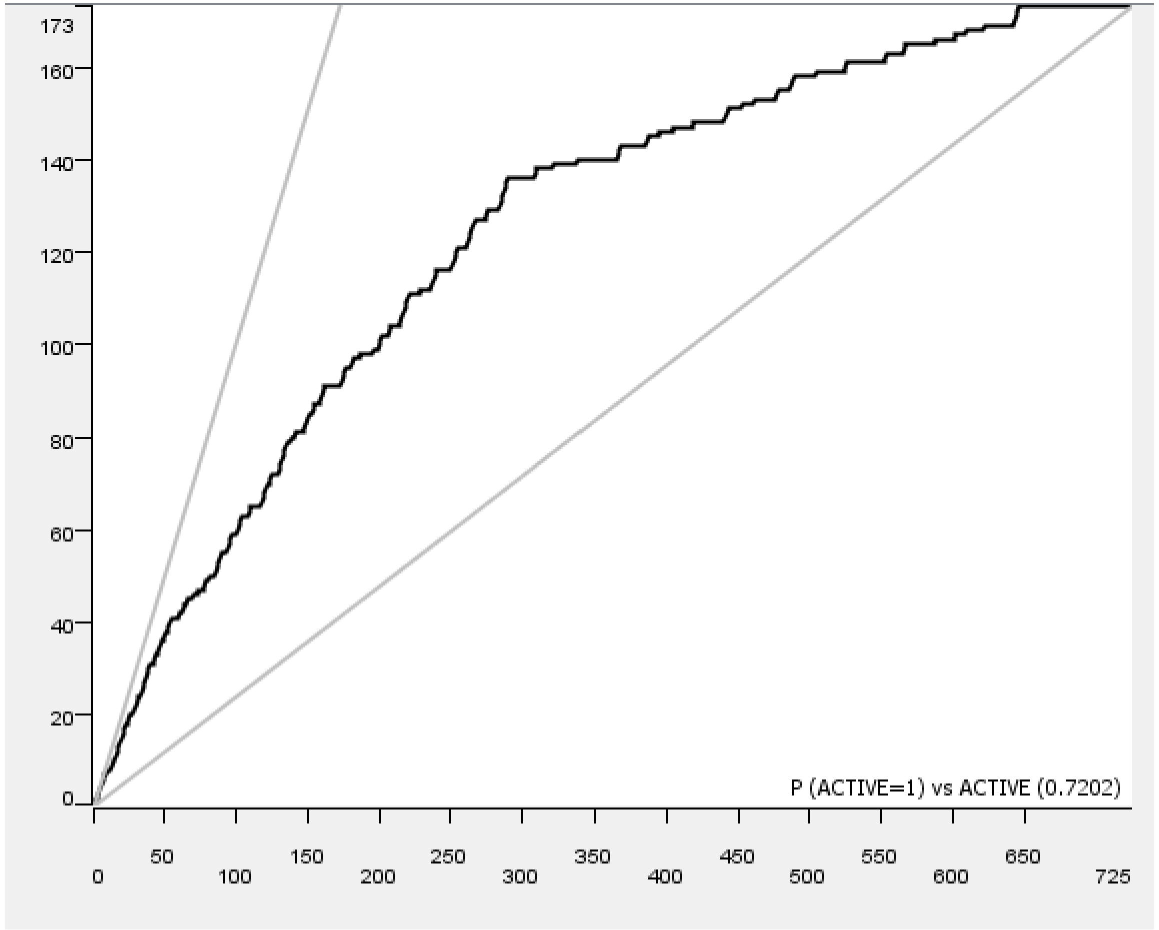

N=725

Top 10% Mean IC50	94.8 nM

	Predicted Active	Predicted Inactive
Active	95	78
Inactive	82	470

RF- <15nM DefGood in JAK2, 30% error

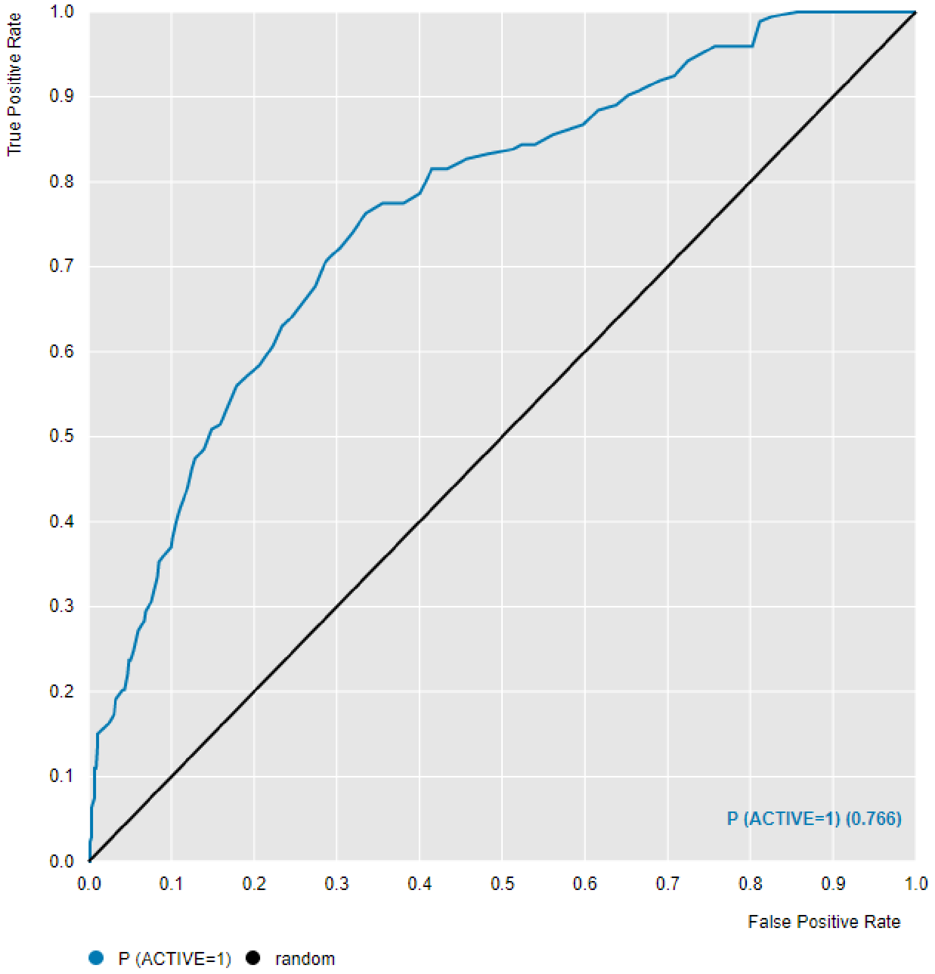

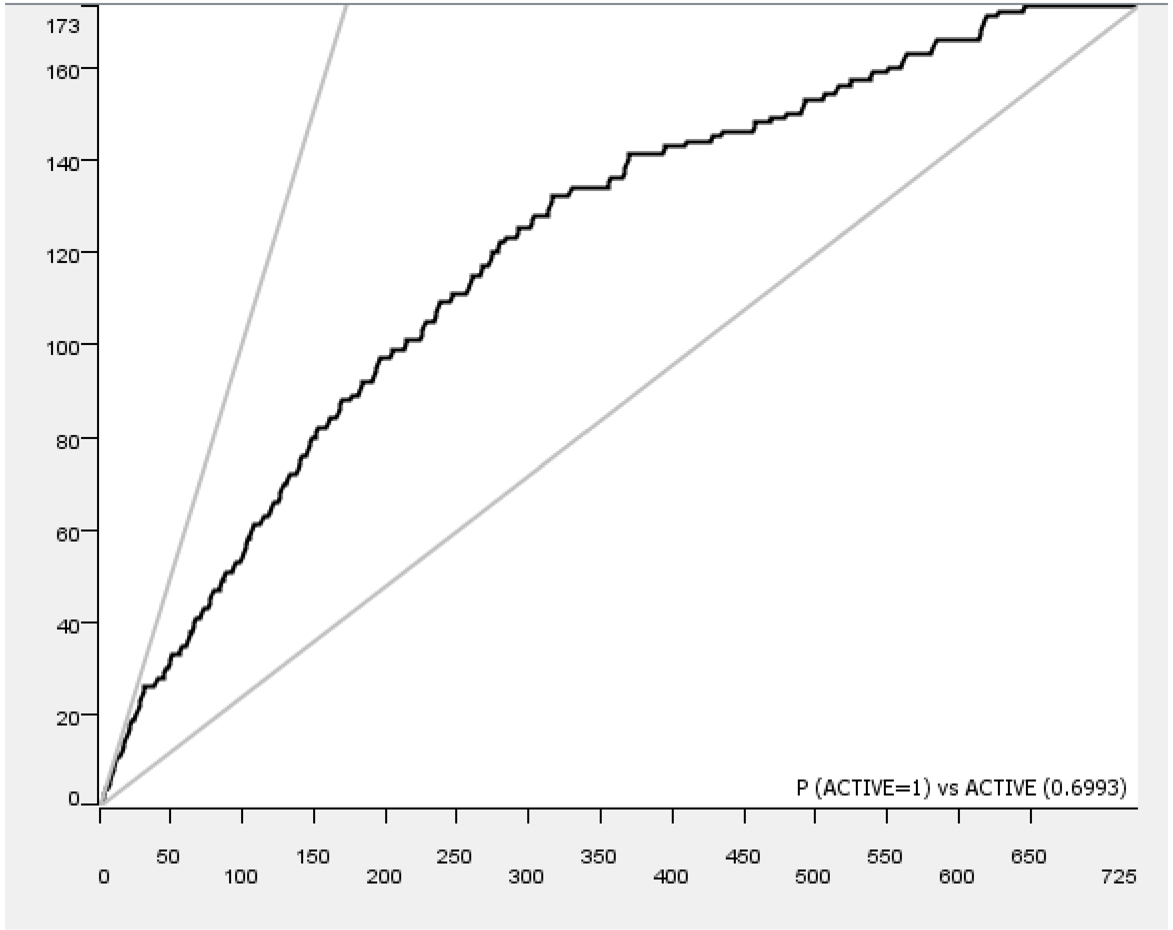

N=725

**Top 10% Mean IC50**	**231 nM**

	**Predicted Active**	**Predicted Inactive**
Active	97	76
Inactive	99	453

RF- <15nM DefGood in JAK2, 35% error

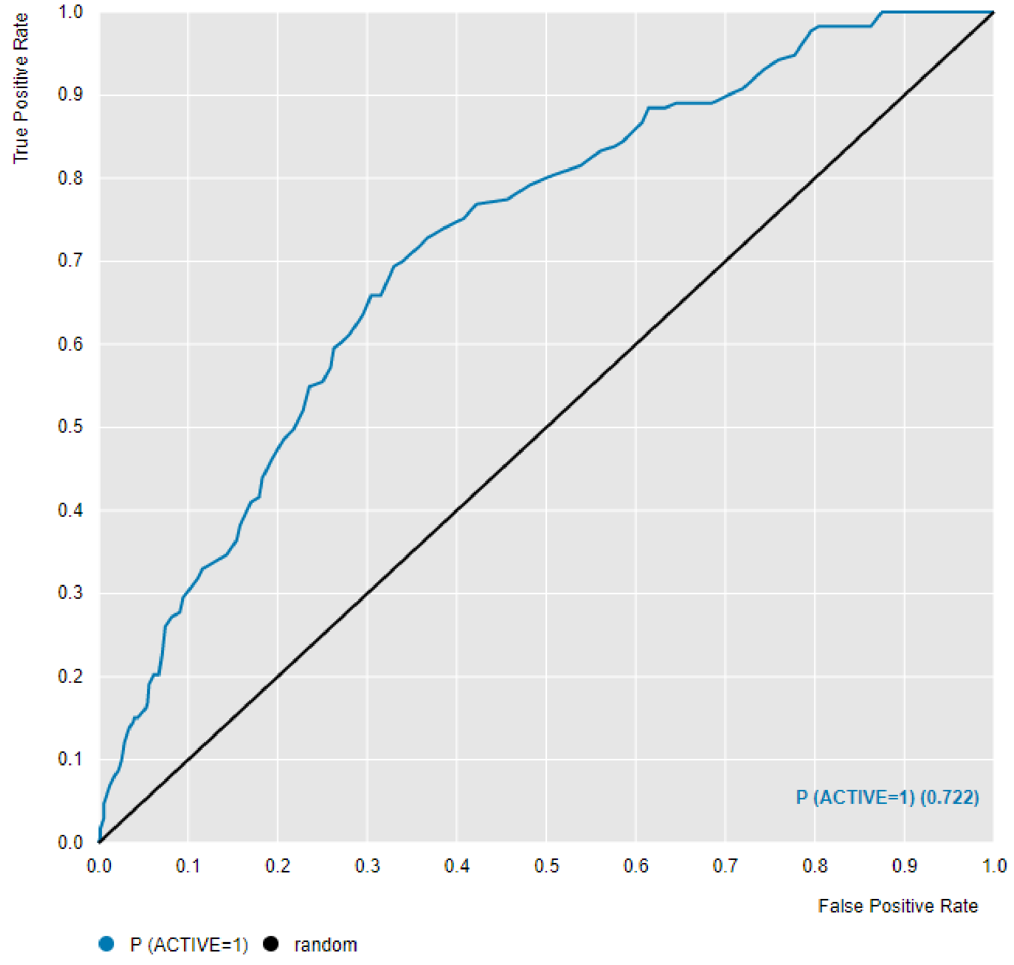

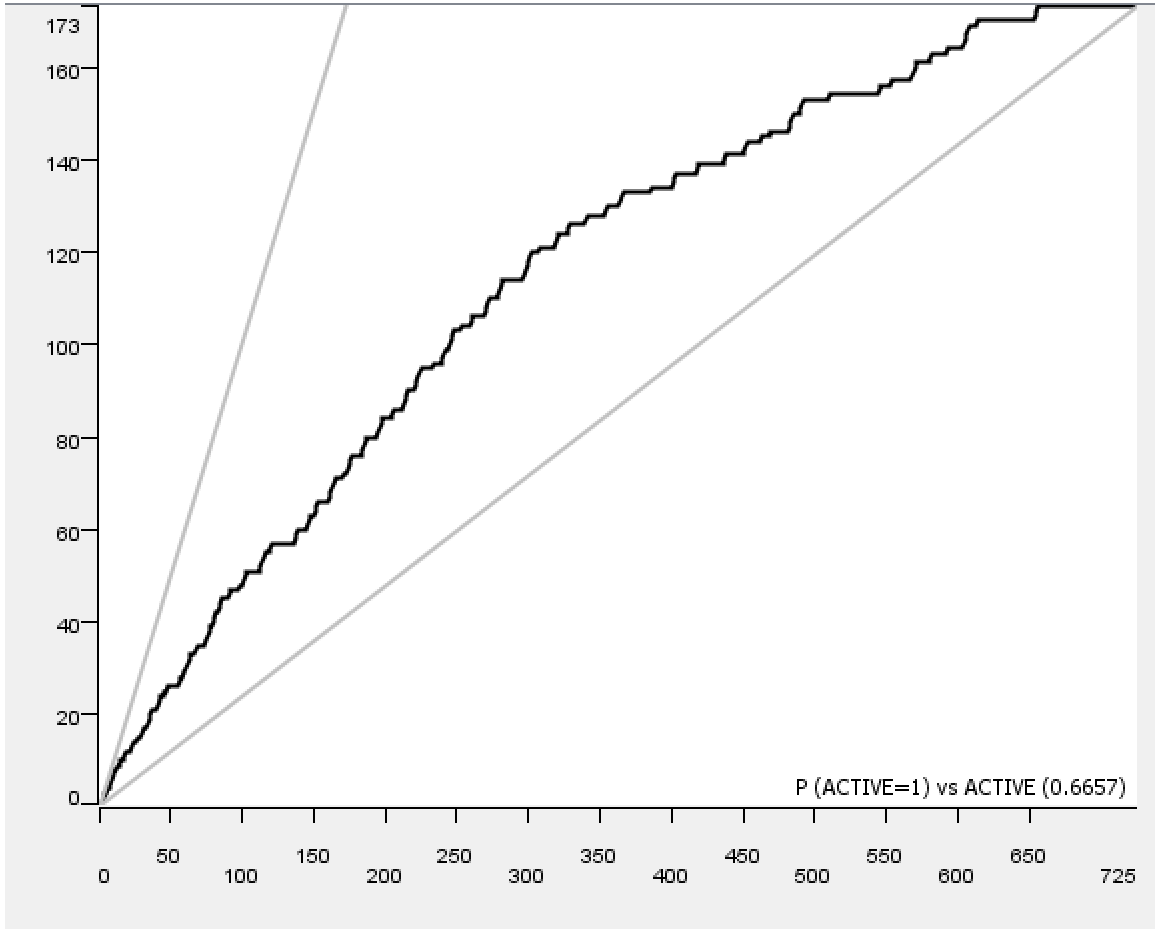

N=725

Top 10% Mean IC50	264 nM

	Predicted Active	Predicted Inactive
Active	99	74
Inactive	143	409

RF- <15nM DefGood in JAK2, 40% error

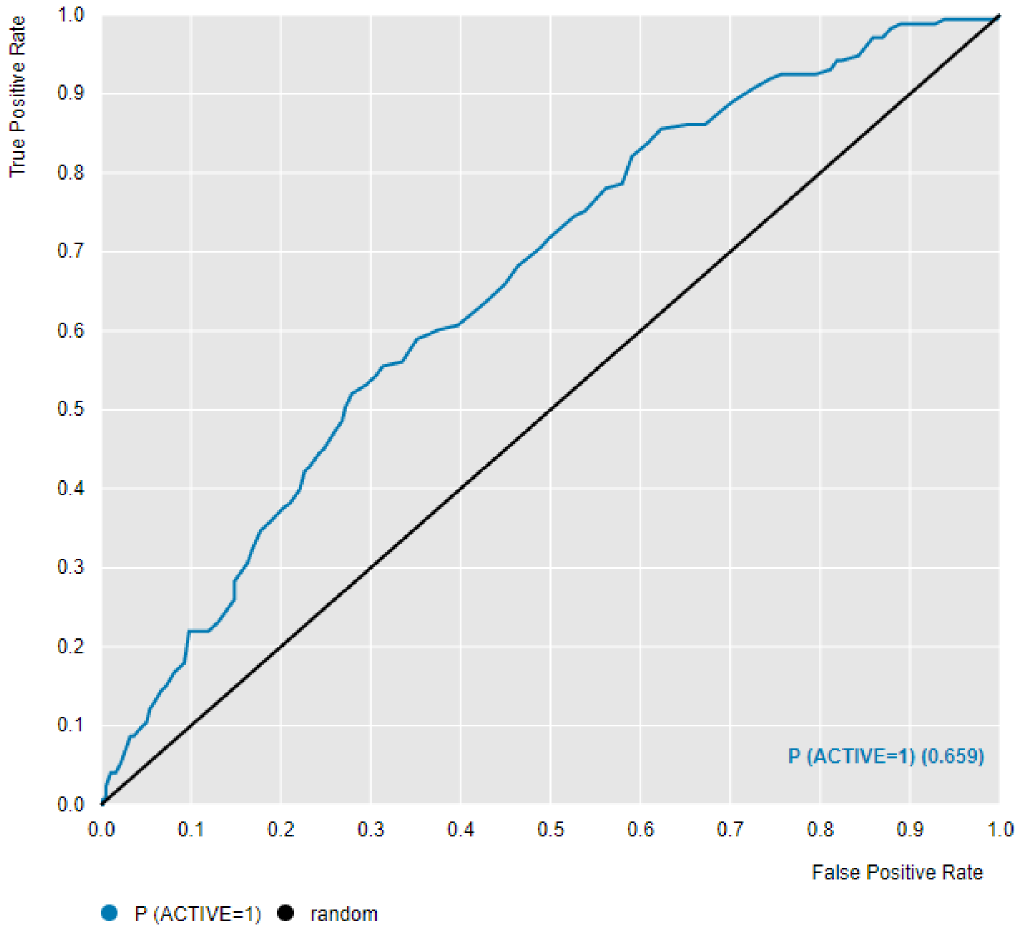

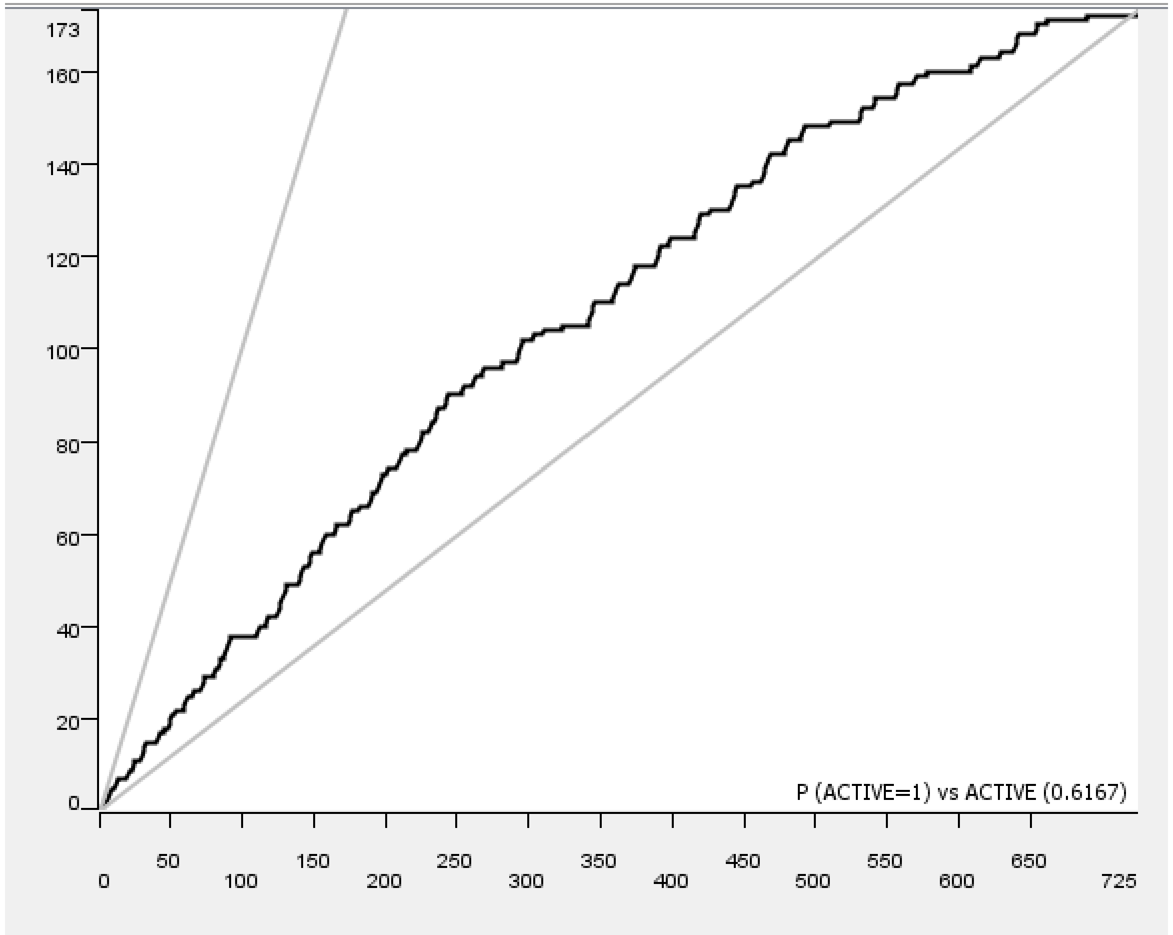

N=725

**Top 10% Mean IC50**	**266.8 nM**

	Predicted Active	Predicted Inactive
Active	90	83
Inactive	154	398

<15nM DefGood in JAK2, 10% error; Random seed = 429

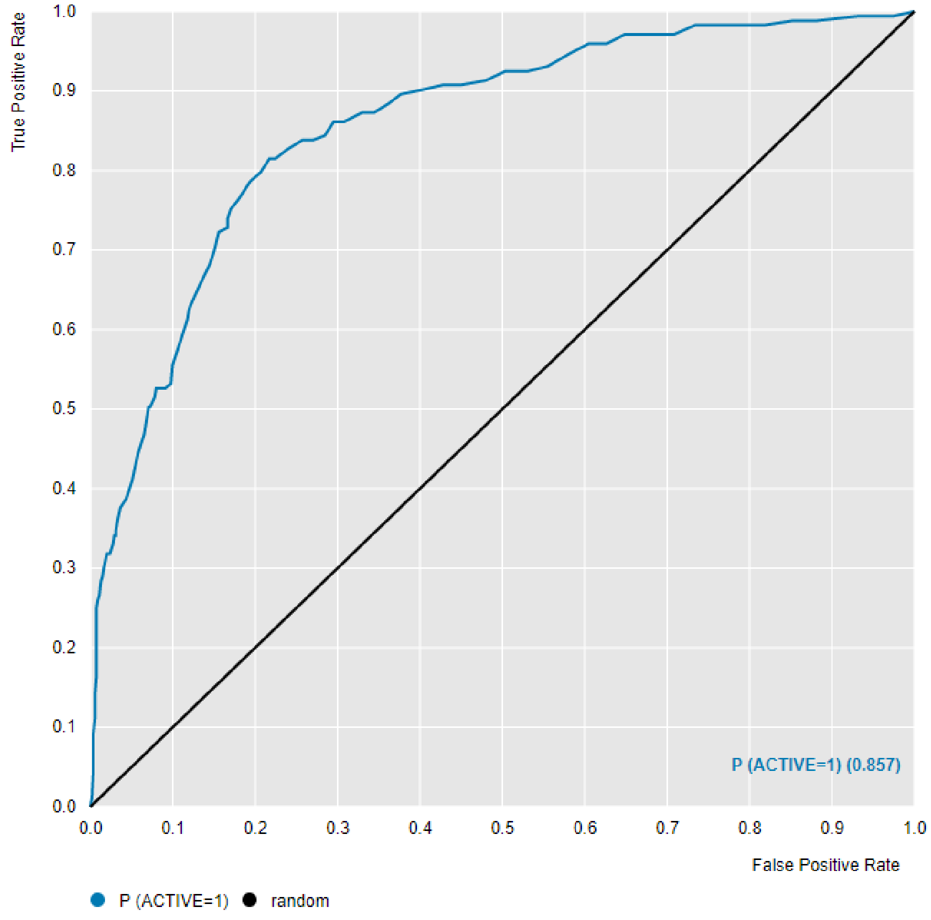

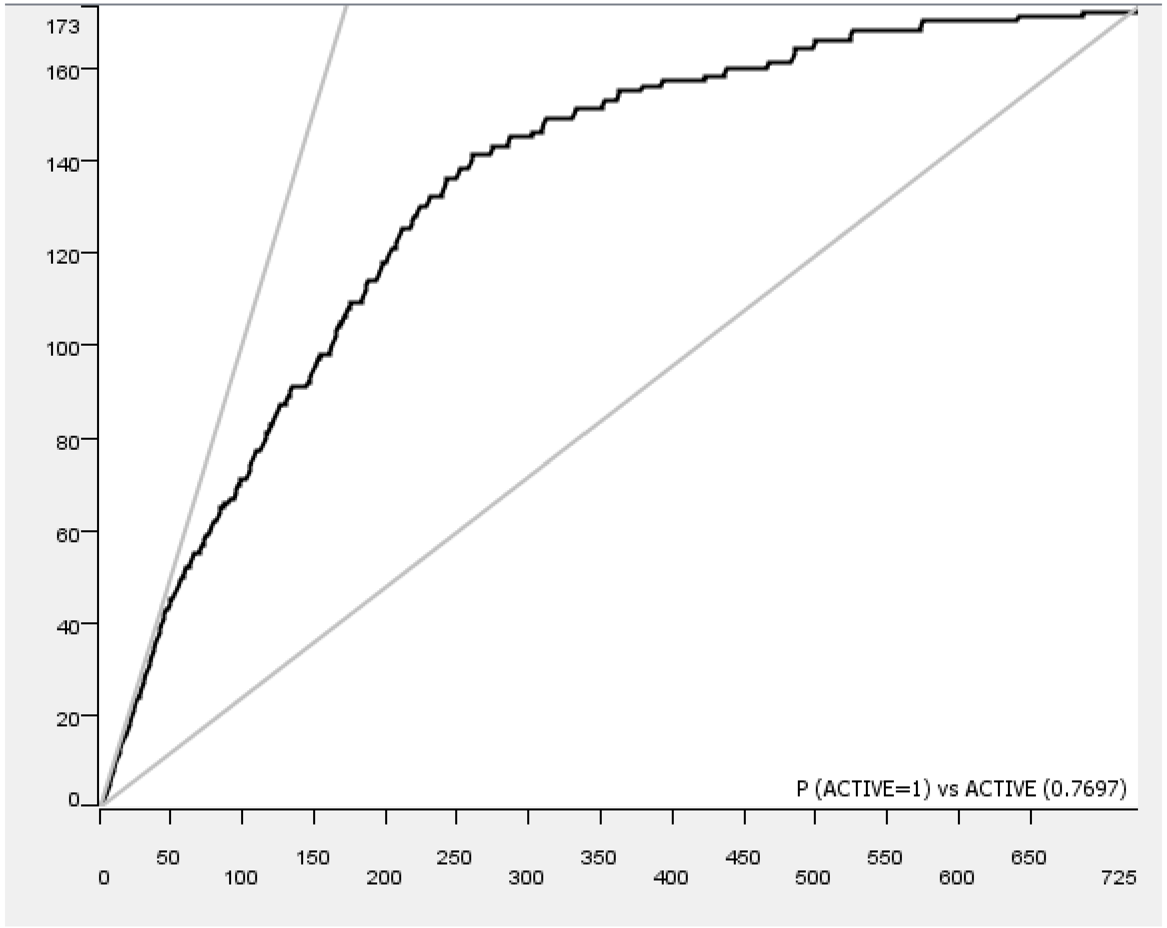

N=725

**Top 10% Mean IC50**	**52 nM**

	Predicted Active	Predicted Inactive
Active	91	82
Inactive	50	502

<15nM DefGood in JAK2, 15% error; Random seed = 429

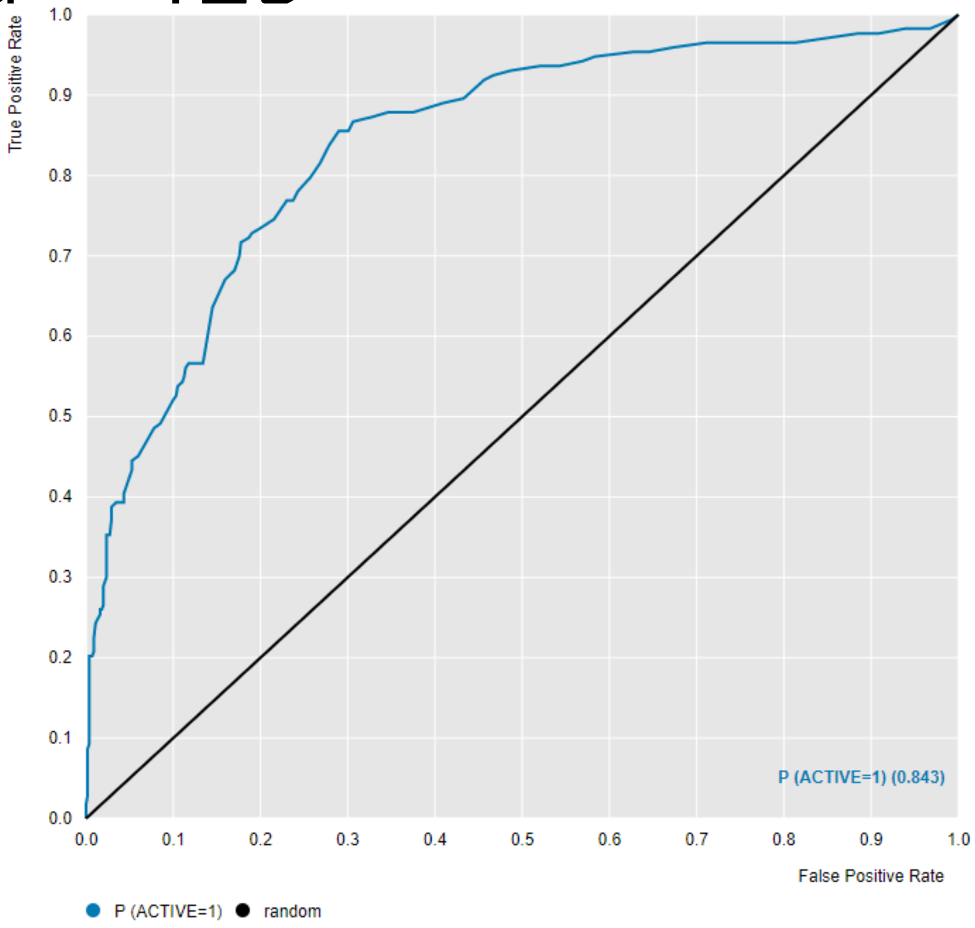

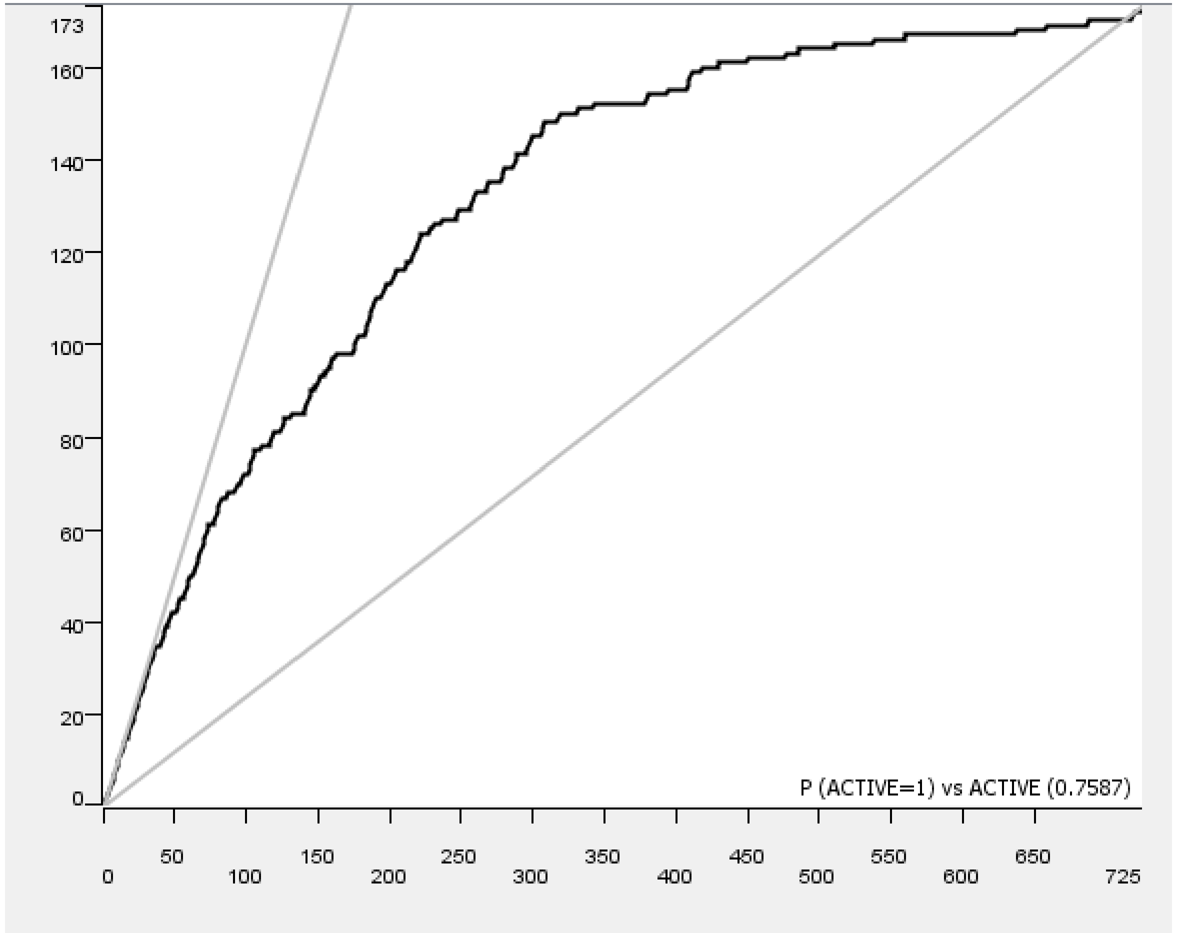

N=725

**Top 10% Mean IC50**	**34.0 nM**

	Predicted Active	Predicted Inactive
Active	94	79
Inactive	61	491

<15nM DefGood in JAK2, 20% error; Random seed = 429

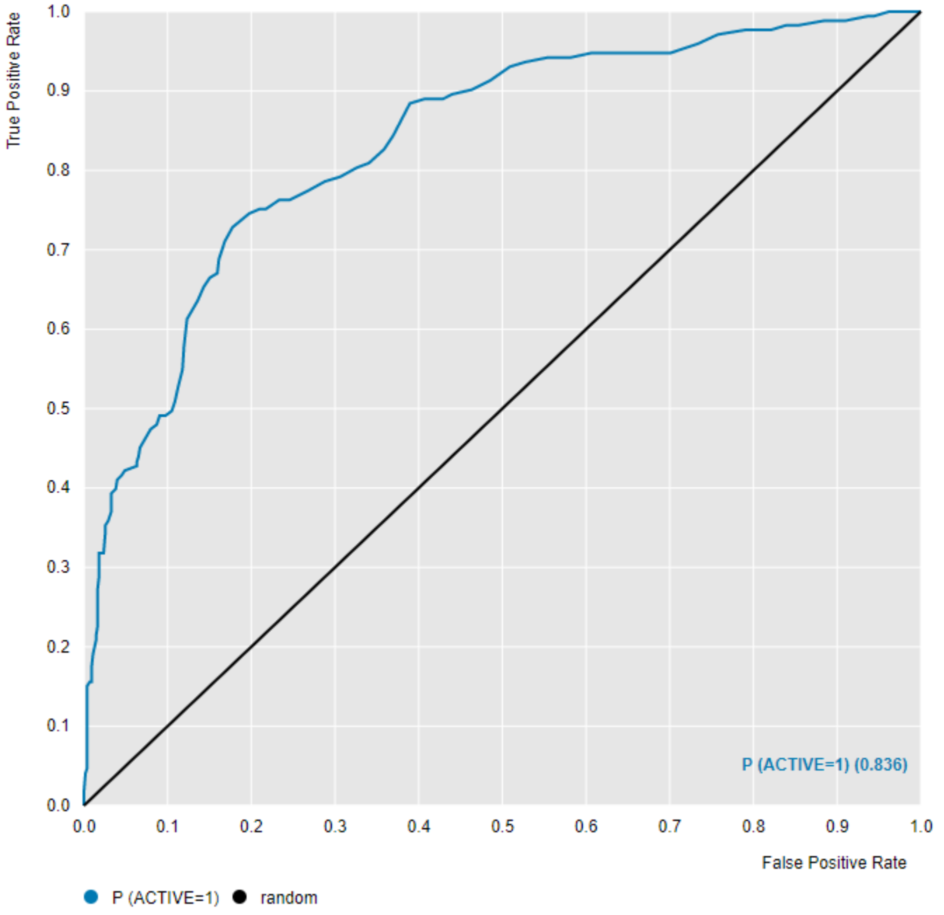

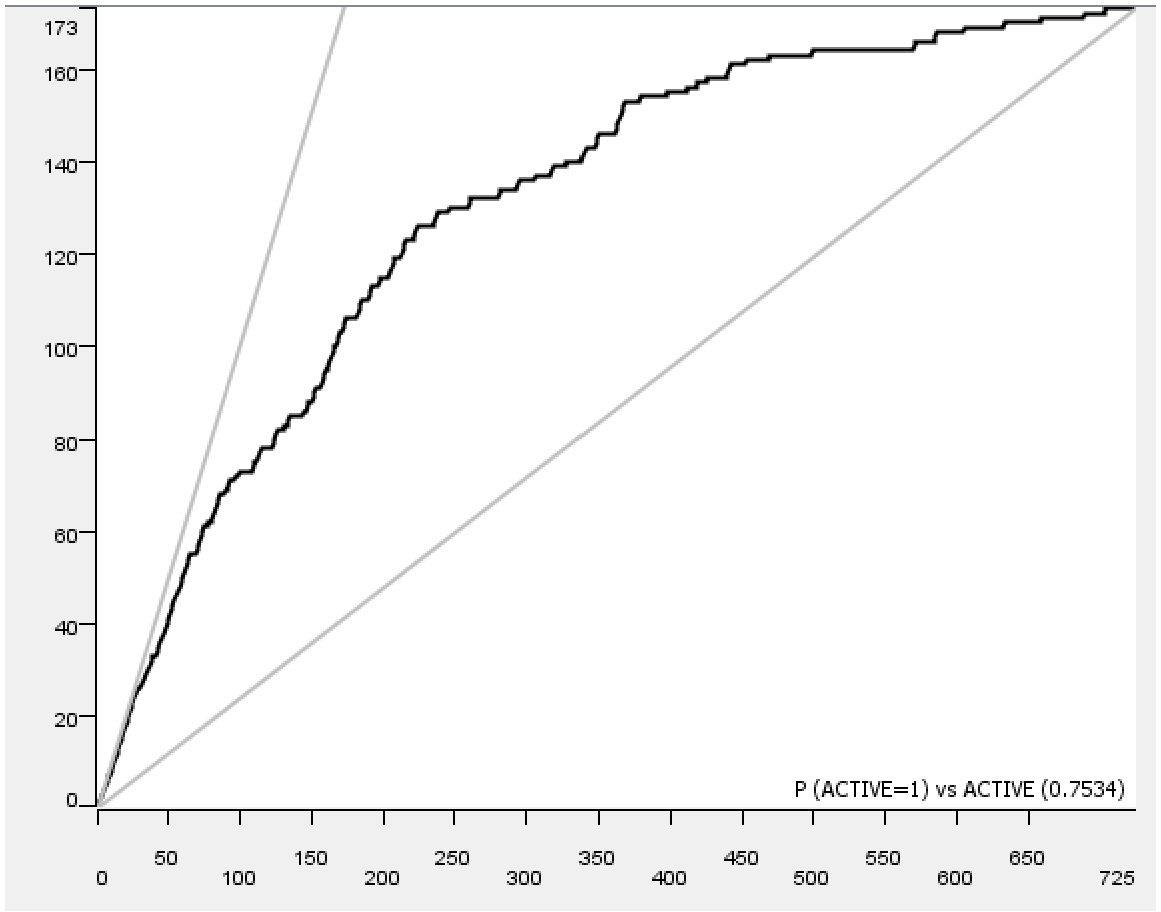

N=725

**Top 10% Mean IC50**	**46.8 nM**

	Predicted Active	Predicted Inactive
Active	95	78
Inactive	65	487

<15nM DefGood in JAK2, 30% error; Random seed = 429

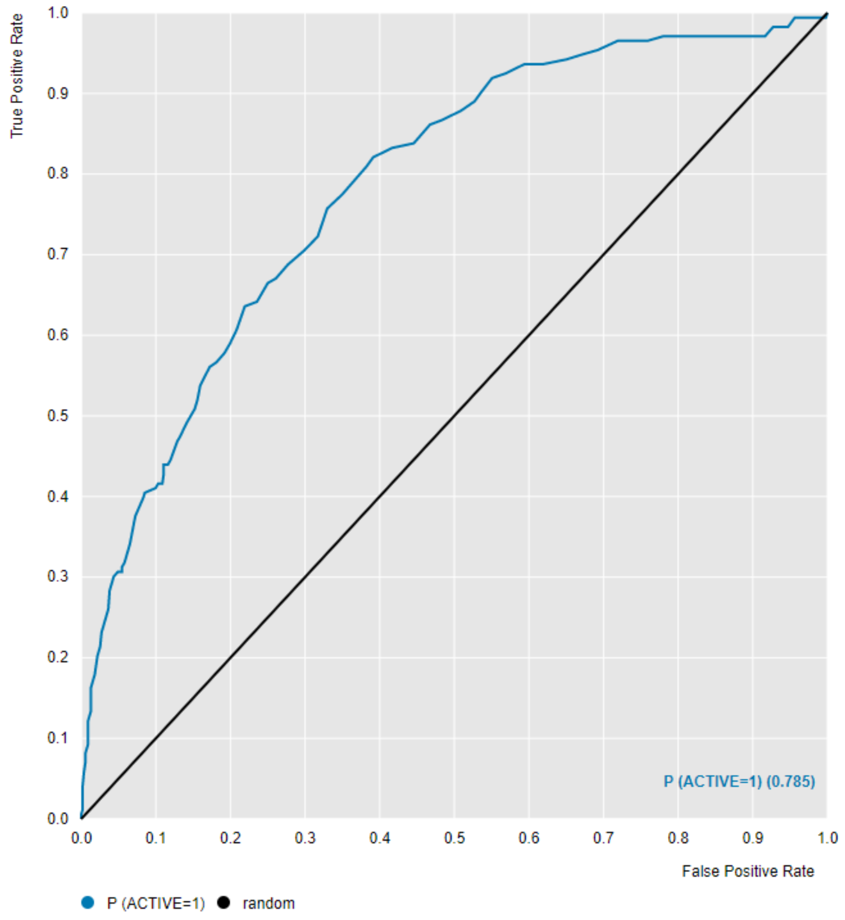

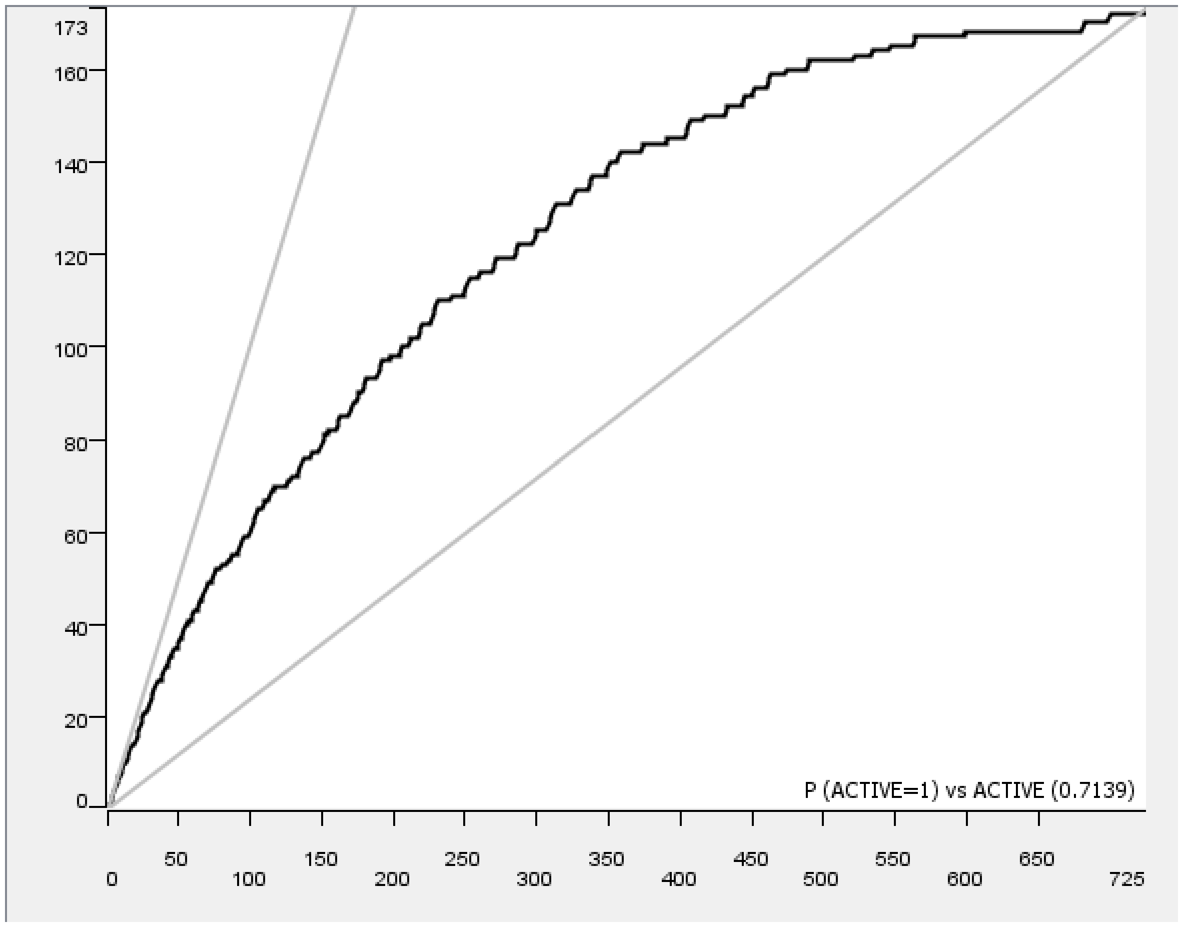

N=725

Top 10% Mean IC50	2,300 nM

	Predicted Active	Predicted Inactive
Active	97	76
Inactive	95	457

<15nM DefGood in JAK2, 10% error; Random seed = 121783

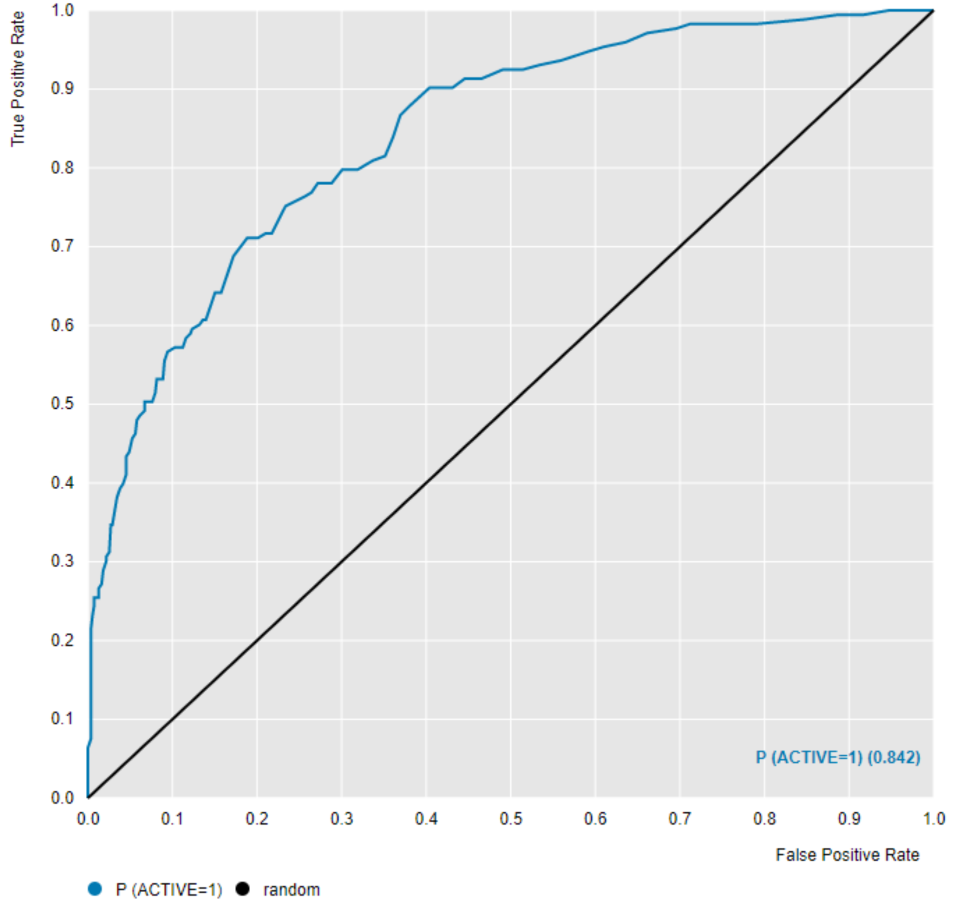

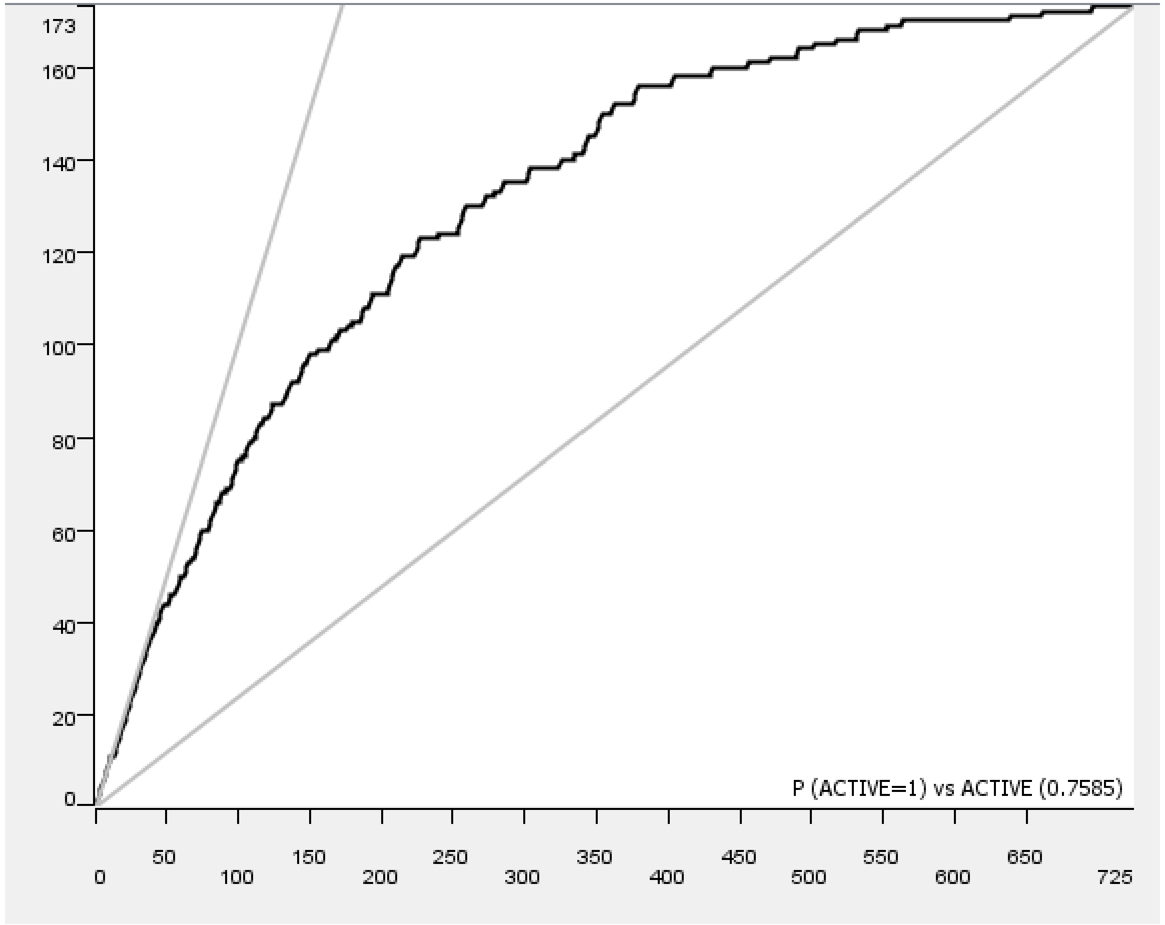

N=725

**Top 10% Mean IC50**	**454 nM**

	**Predicted Active**	**Predicted Inactive**
Active	98	75
Inactive	52	500

<15nM DefGood in JAK2, 20% error; Random seed = 121783

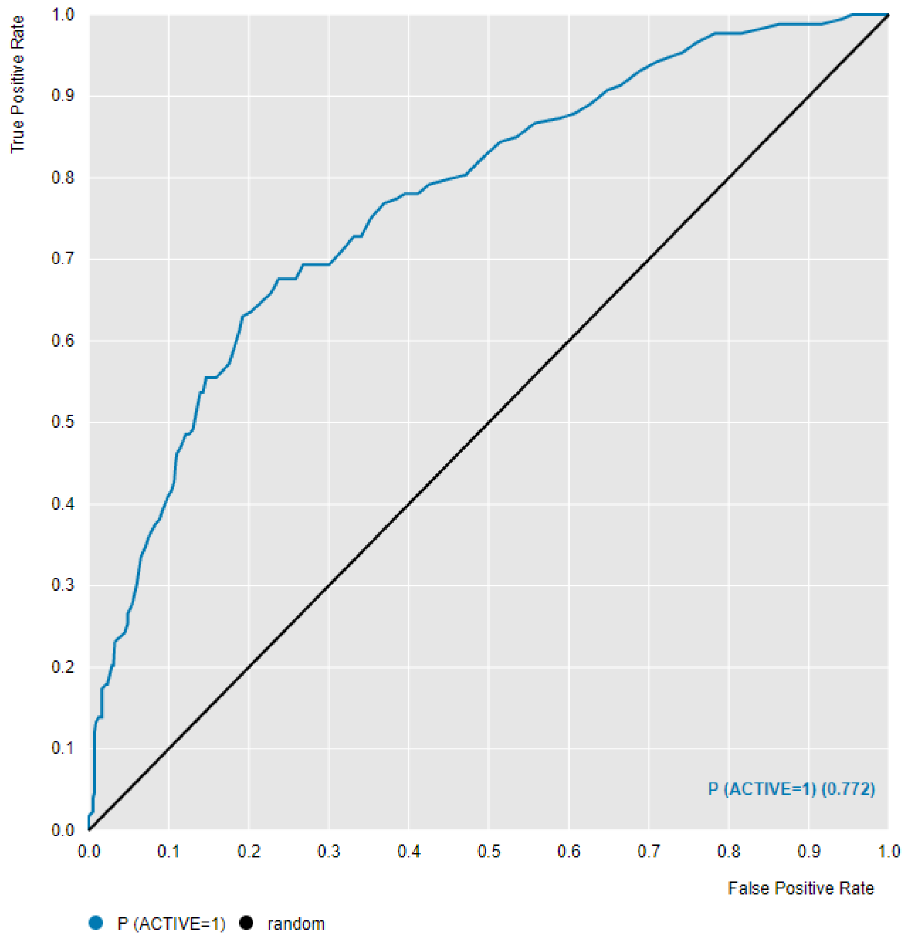

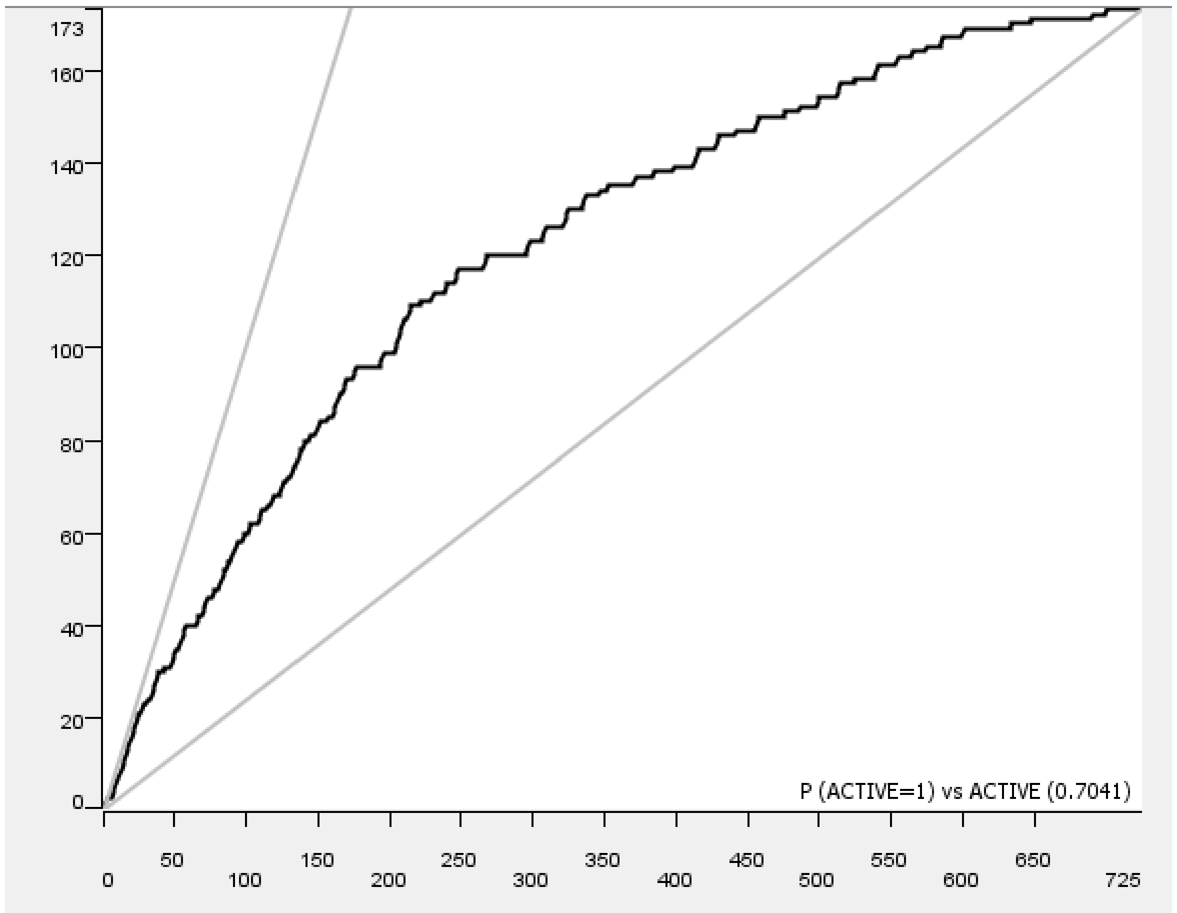

N=725

Top 10% Mean IC50	653 nM

	Predicted Active	Predicted Inactive
Active	90	83
Inactive	75	477

<15nM DefGood in JAK2, 30% error; Random seed = 121783

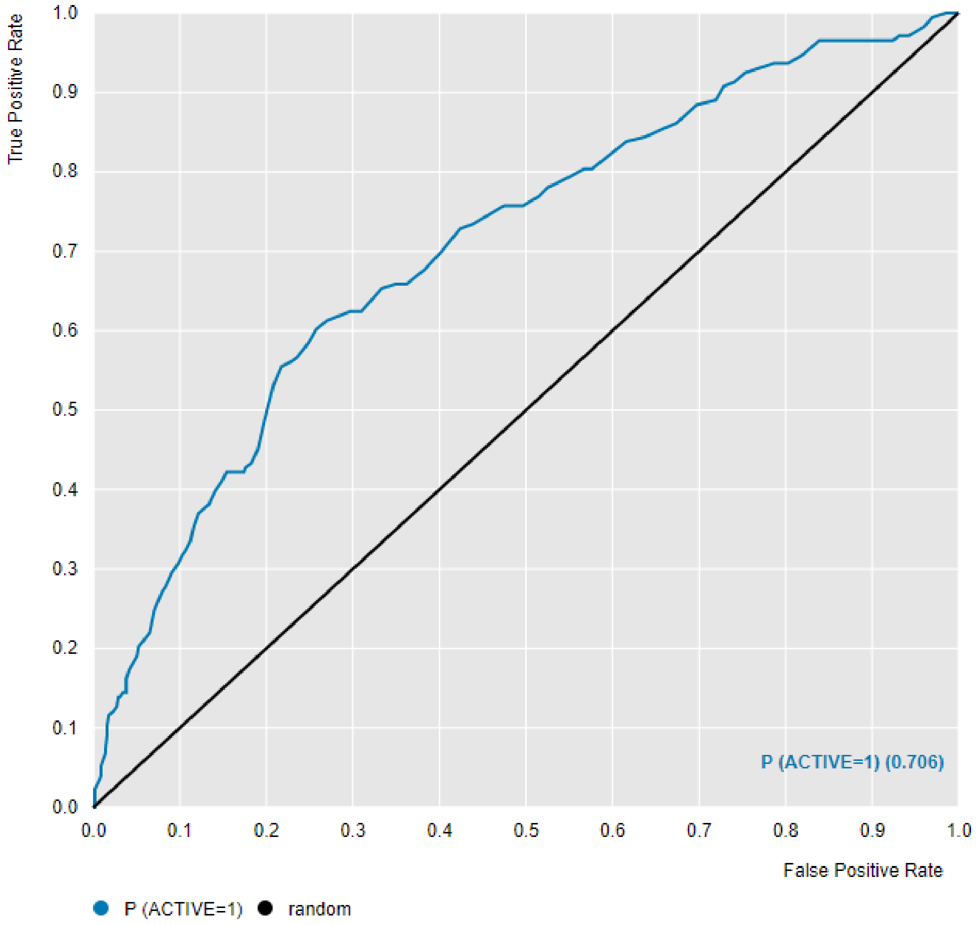

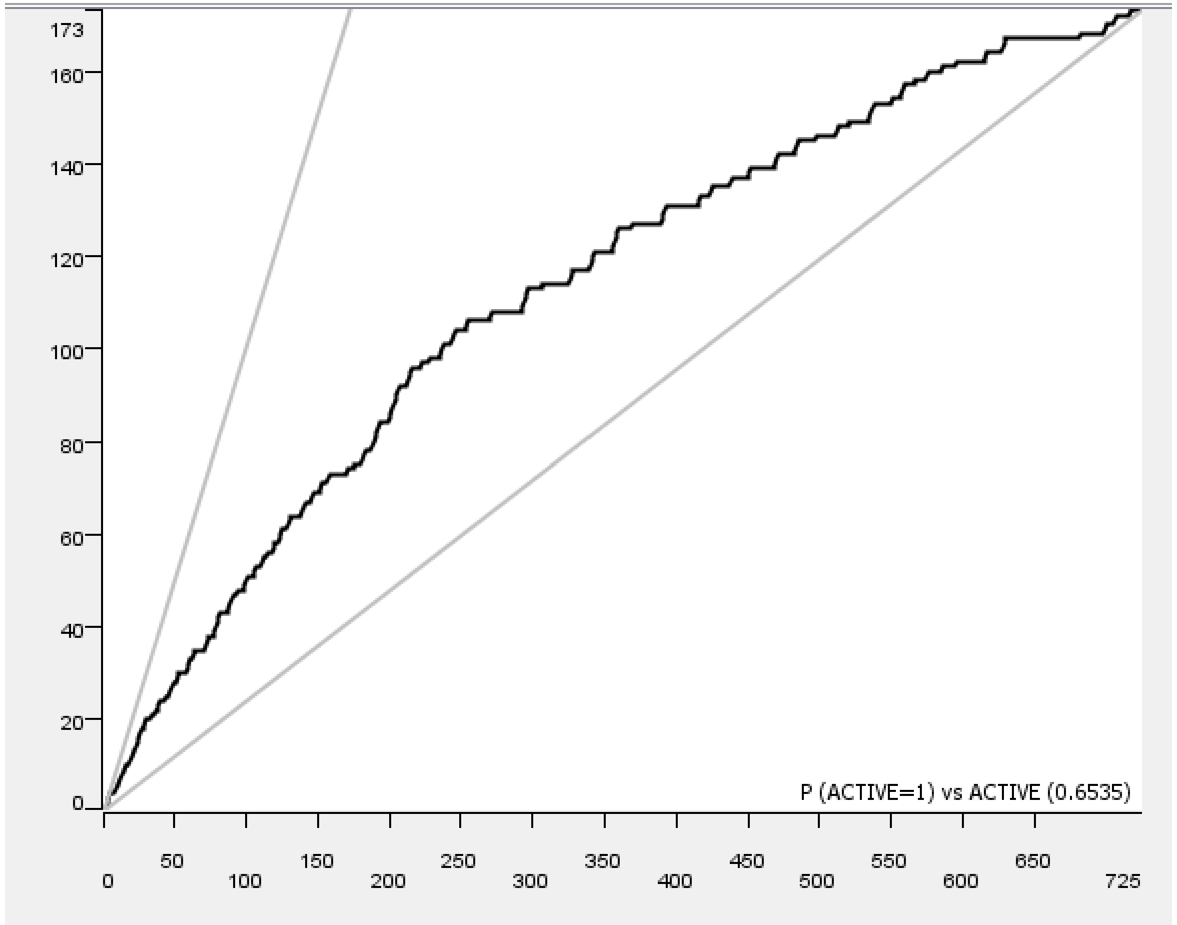

N=725

**Top 10% Mean IC50**	**563 nM**

	**Predicted Active**	**Predicted Inactive**
Active	84	89
Inactive	109	443

<15nM DefGood in JAK2, 35% error; Random seed = 121783

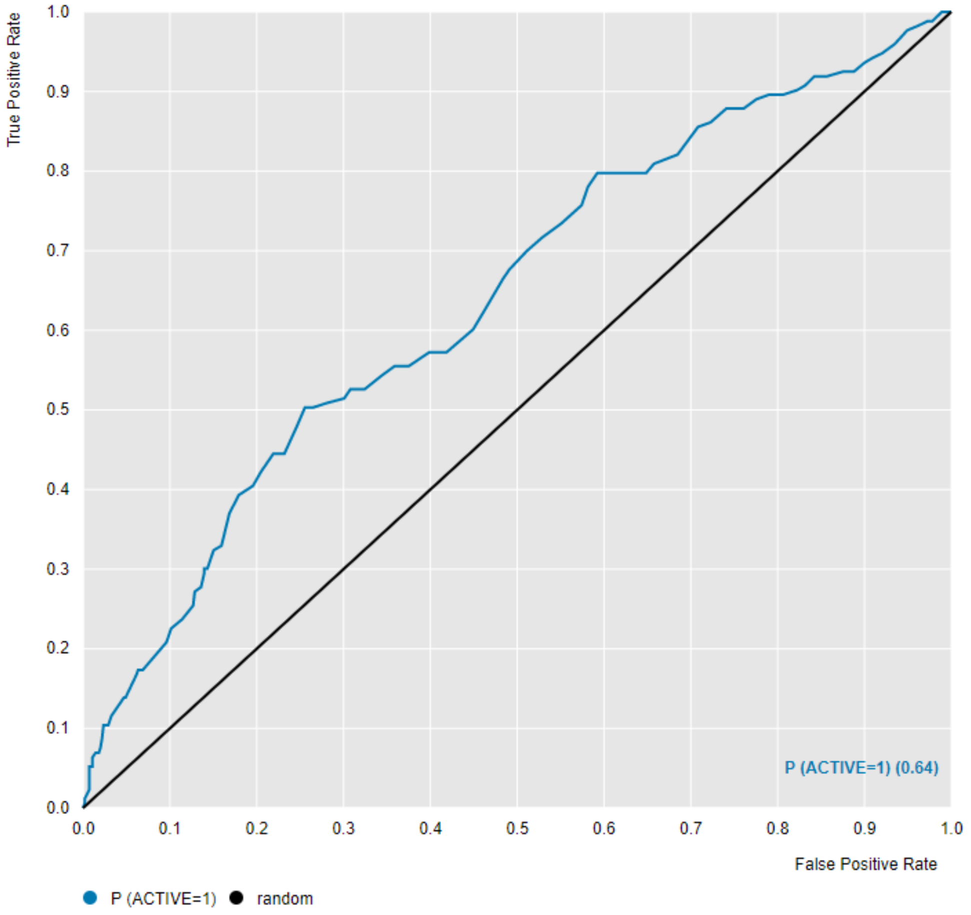

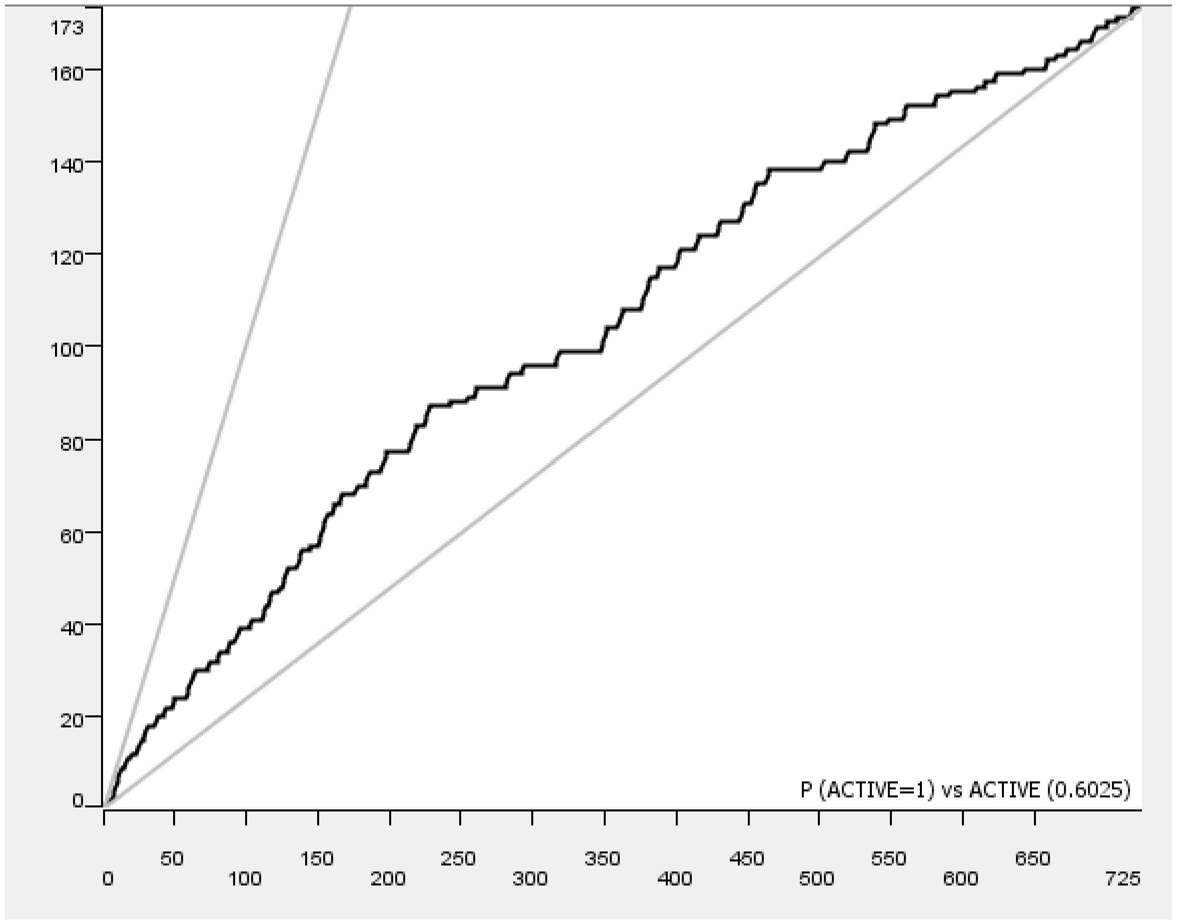

N=725

**Top 10% Mean IC50**	**743 nM**

	Predicted Active	Predicted Inactive
Active	87	86
Inactive	141	411

Conclusion - RF

- A Random Forrest could be generated for JAK2 kinase with a decision value of <15 nM as defined as active. The predictivity of the algorithm could be preserved in three random train/test cycles as error was introduced in the training set. The point of failure was 40%, 30% and 35% error.

Conclusion - PNN

- PNN failed in the control phase

MEK1

MEK1 NBN Design

- Rules - $RELATION$ = "=" OR $IC50$ > 999 OR $IC50$ < 10 => TRUE
- Sort ascending and remove duplicates
- DefGood scanned between 5 and 50 nM
- 80-20 split for both actives and inactives
- NBN – ECFP4 used Classifier where Prediction was Active = True
- Resulted in 823 compounds after cleaning the data

<5 nM DefGood in MEK1

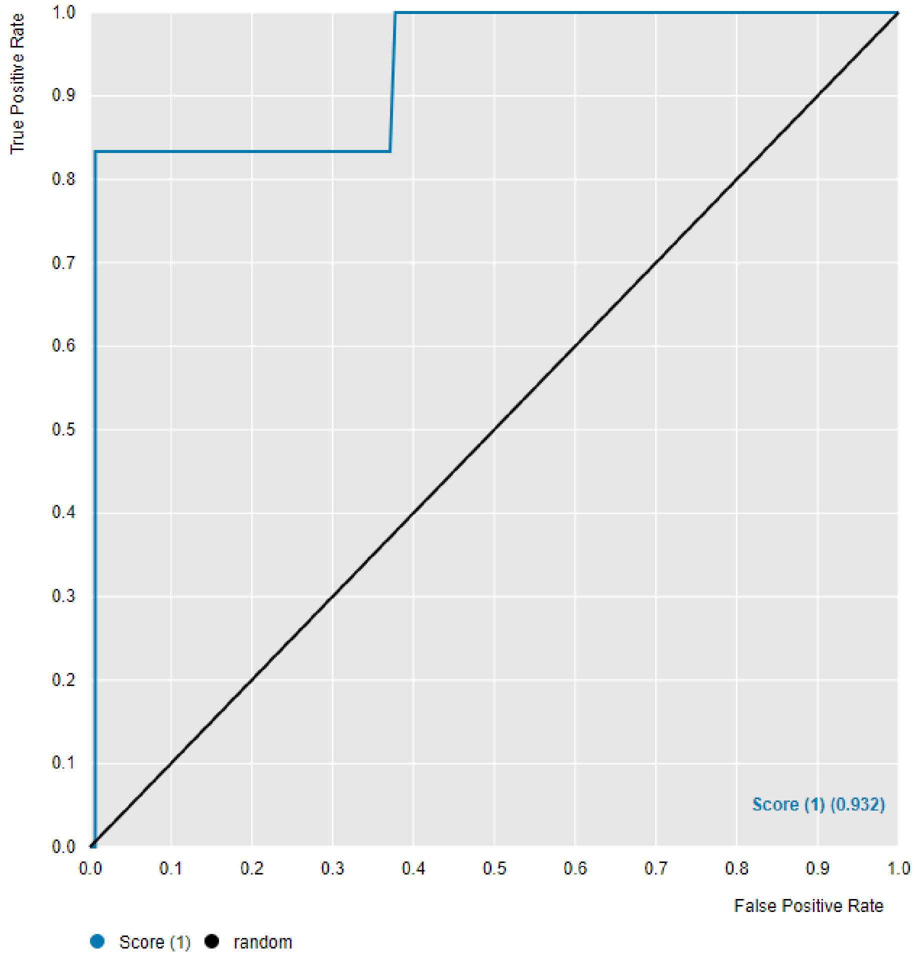

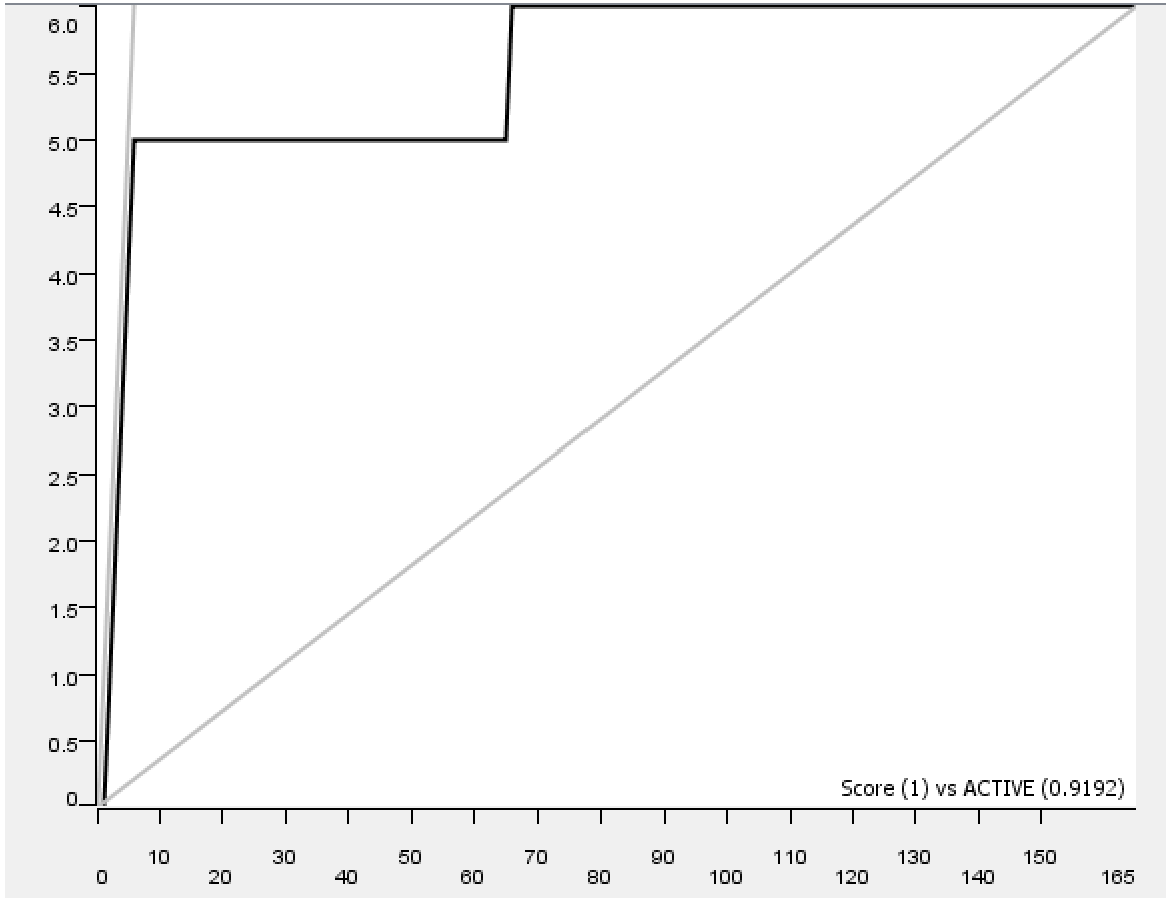

N=165

Top 10% Mean IC50	271 nM

	Predicted Active	Predicted Inactive
Active	5	1
Inactive	51	108

<10 nM DefGood in MEK1

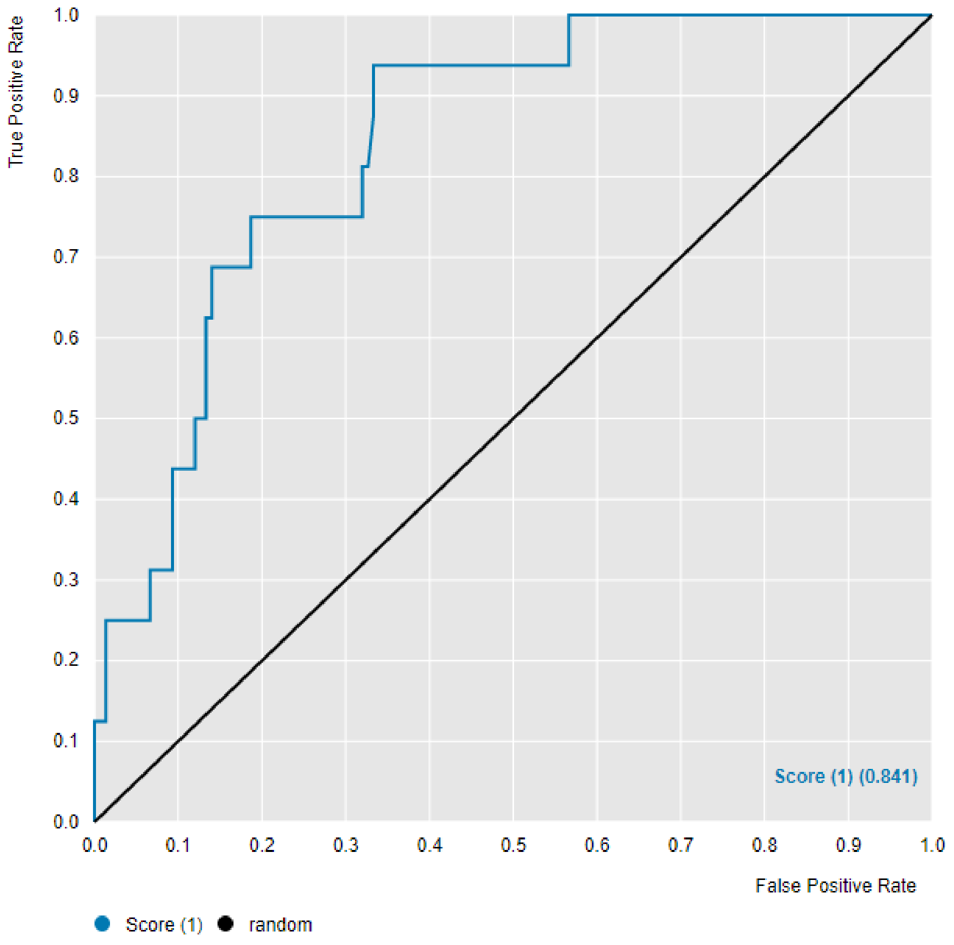

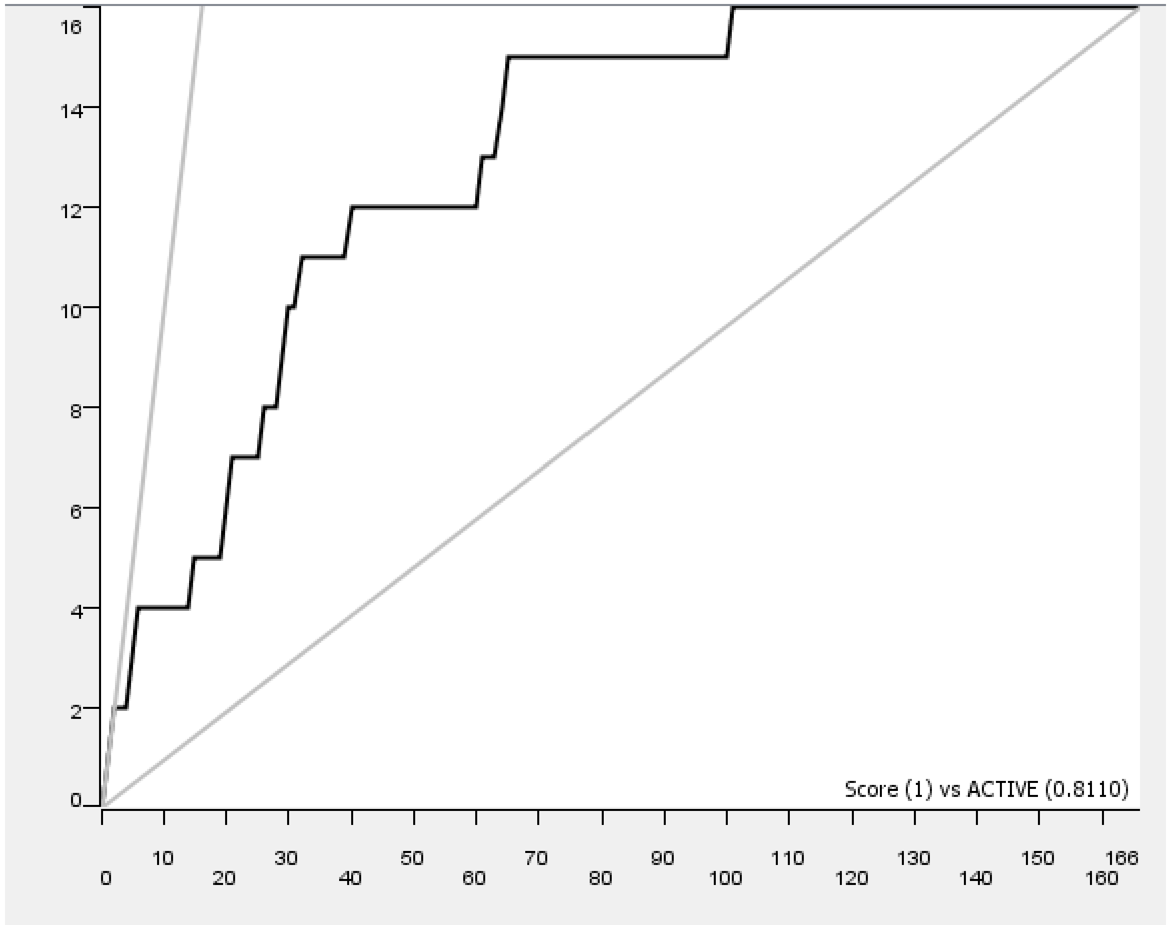

N=165

Top 10% Mean IC50	93.5 nM

	Predicted Active	Predicted Inactive
Active	11	5
Inactive	28	122

<15 nM DefGood in MEK1

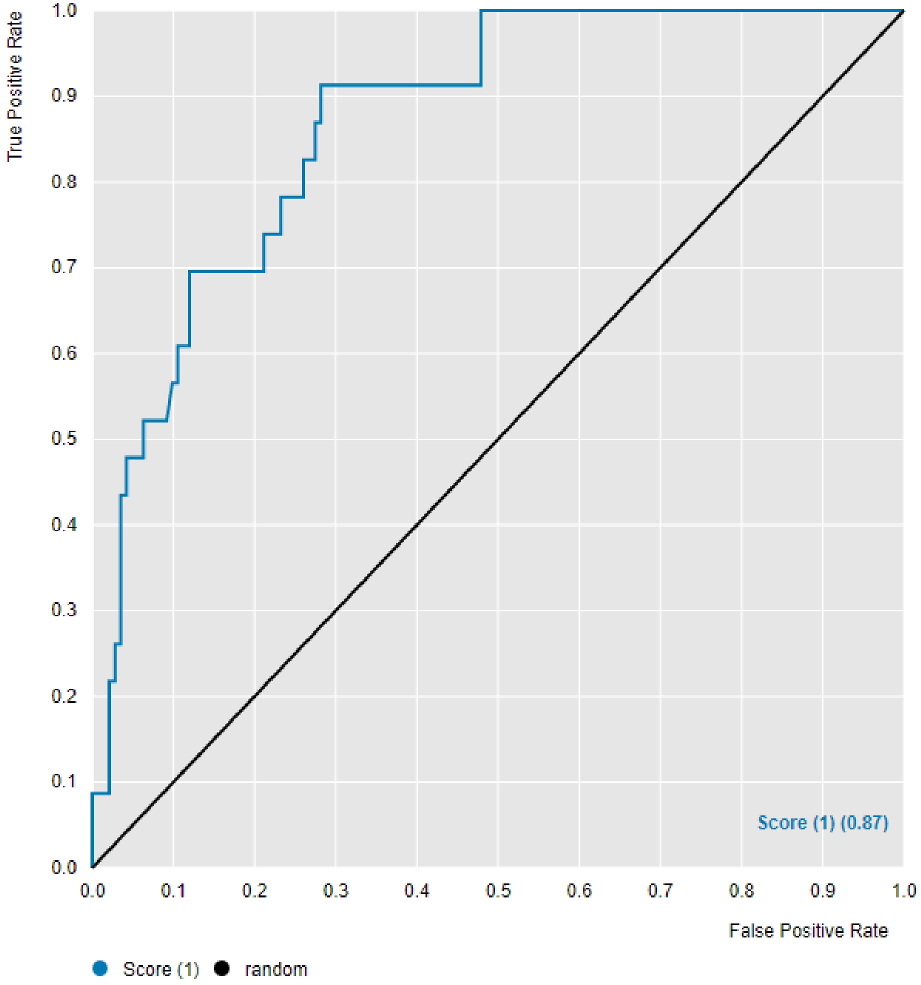

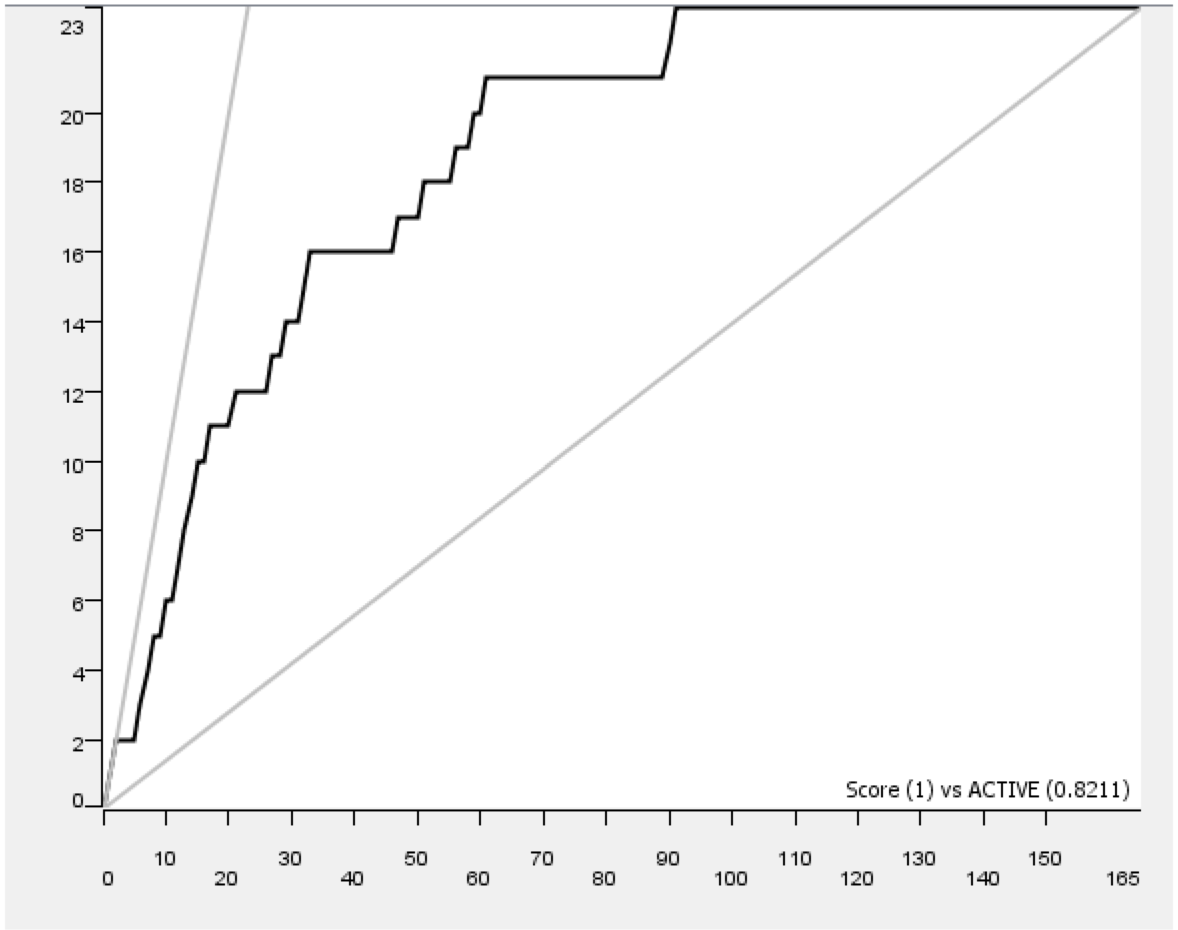

N=165

**Top 10% Mean IC50**	**129 nM**

	Predicted Active	Predicted Inactive
Active	21	2
Inactive	43	99

<20 nM DefGood in MEK1

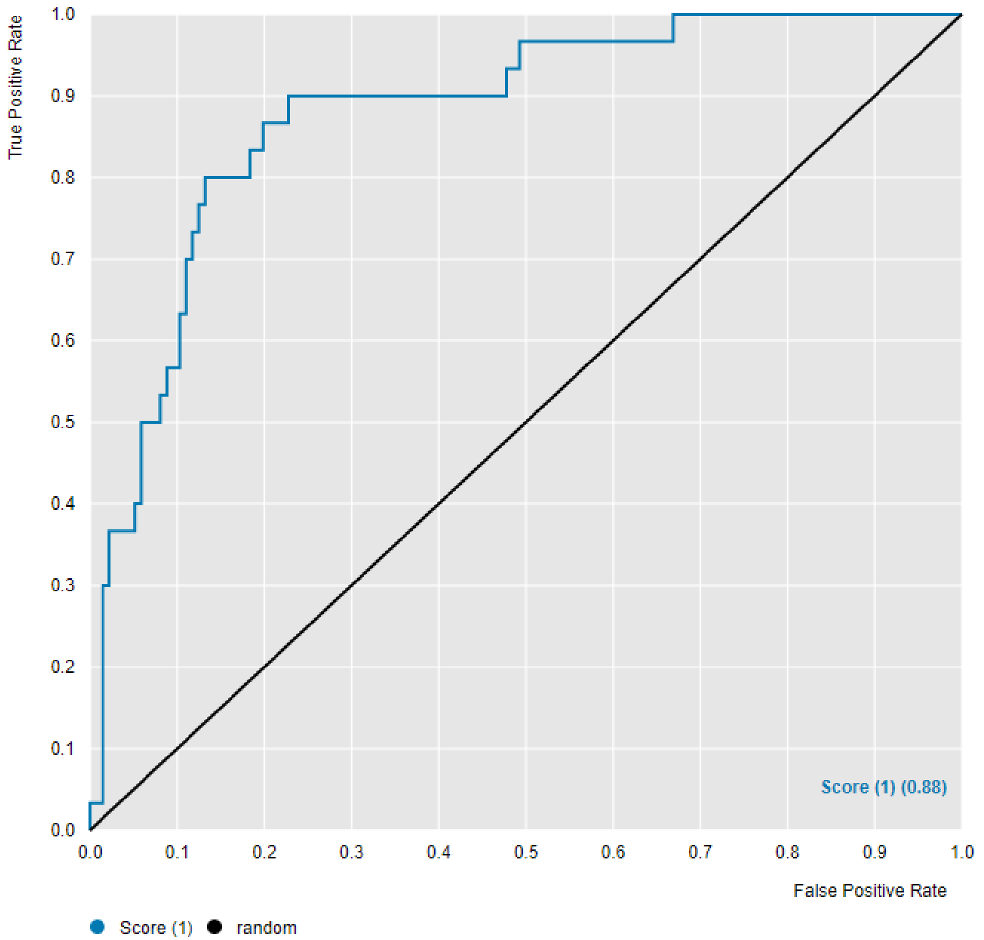

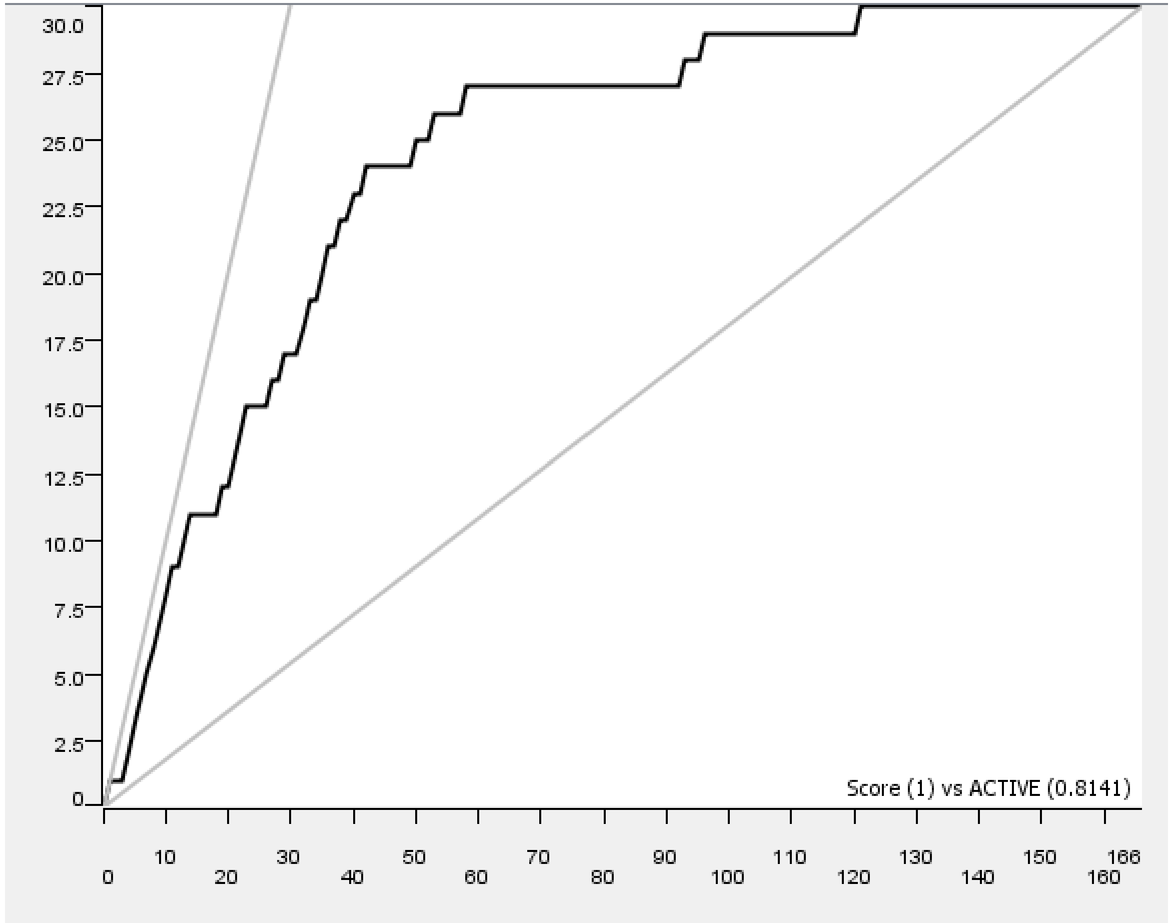

N=166

Top 10% Mean IC50	121 nM

	Predicted Active	Predicted Inactive
Active	27	3
Inactive	38	98

<25 nM DefGood in MEK1

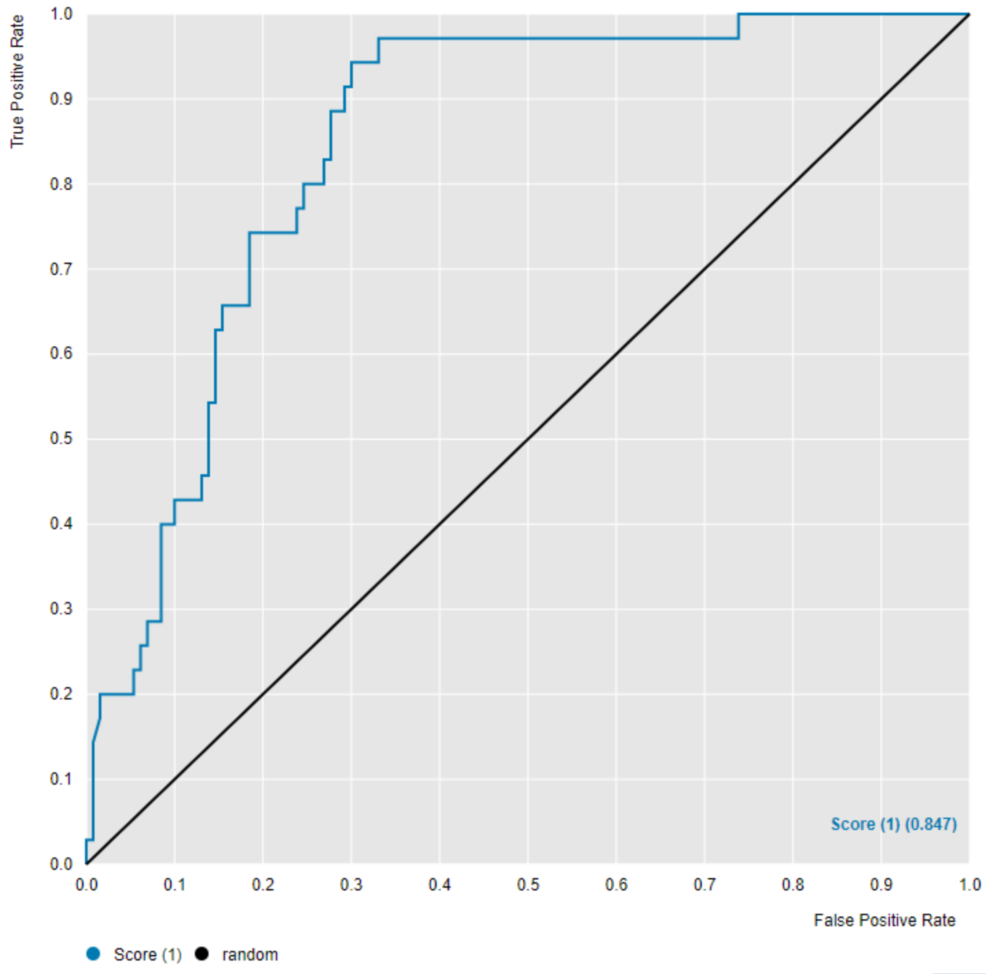

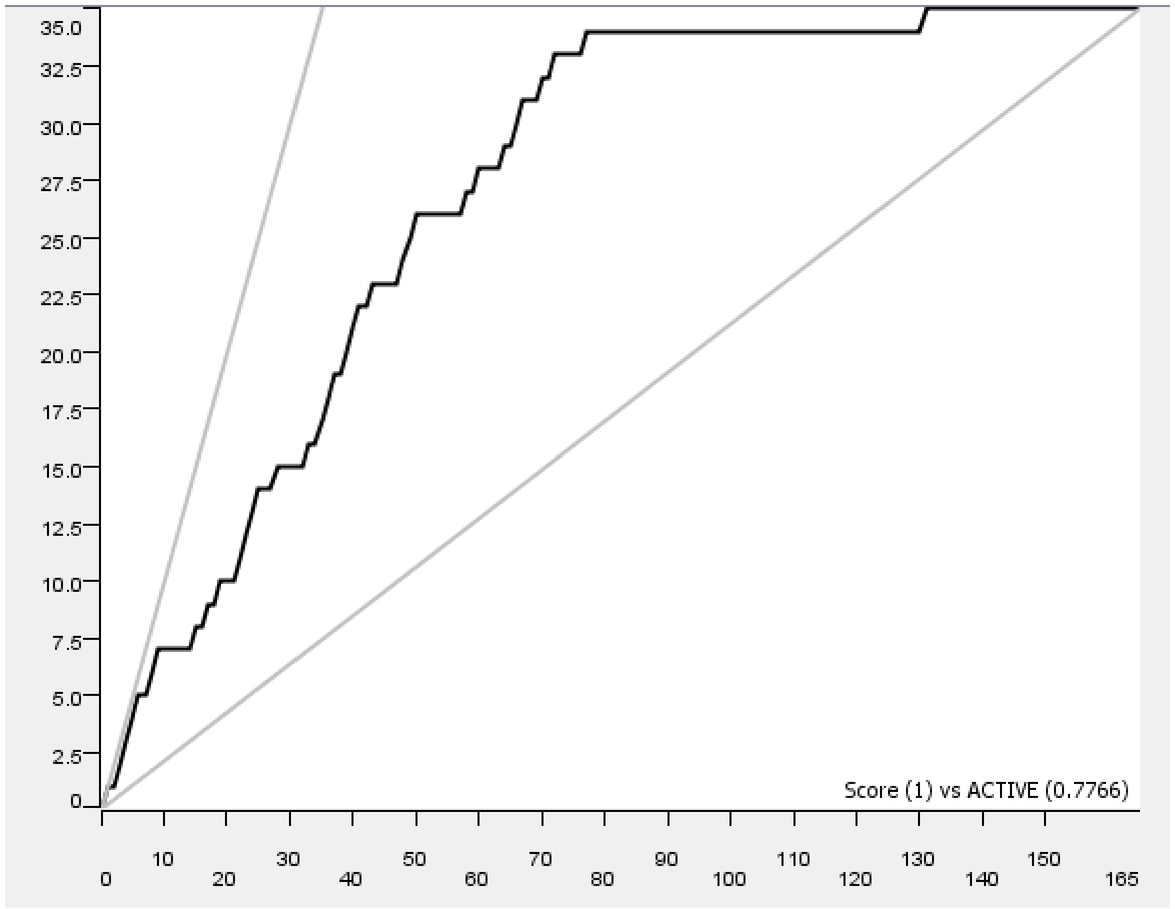

N=166

**Top 10% Mean IC50**	**54 nM**

	**Predicted Active**	**Predicted Inactive**
Active	25	10
Inactive	24	106

<35 nM DefGood in MEK1

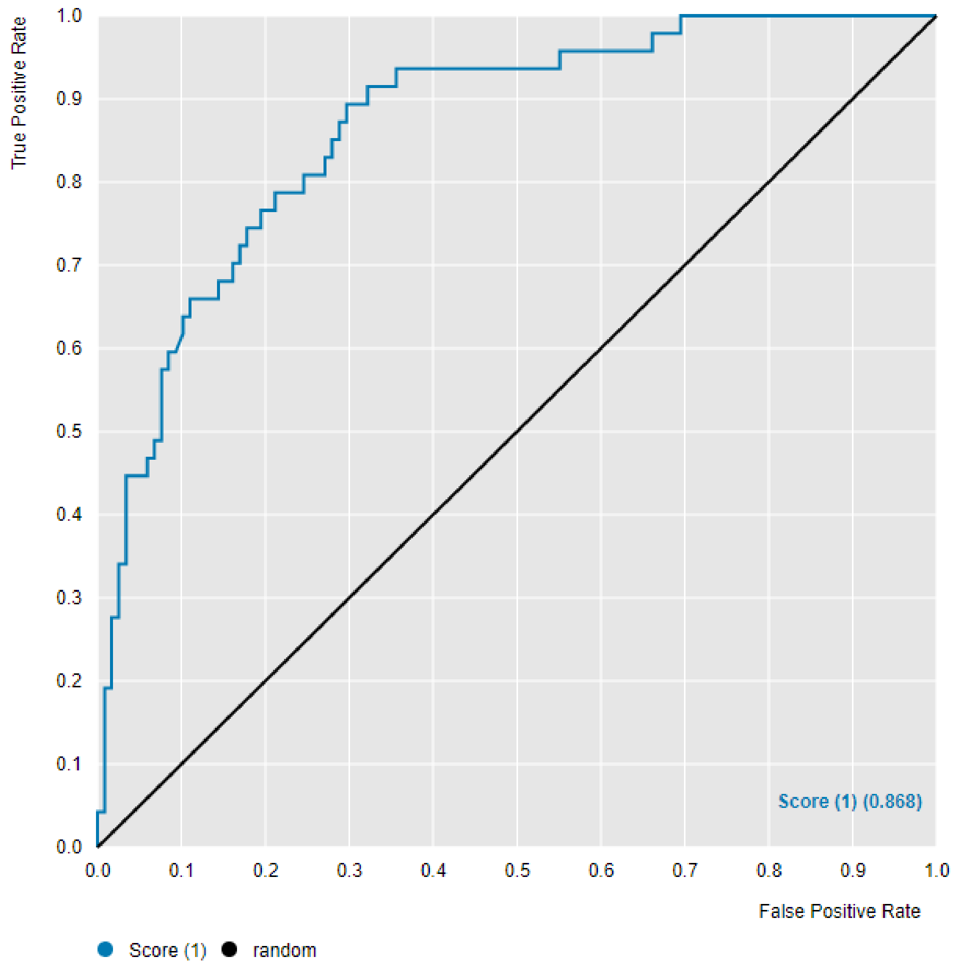

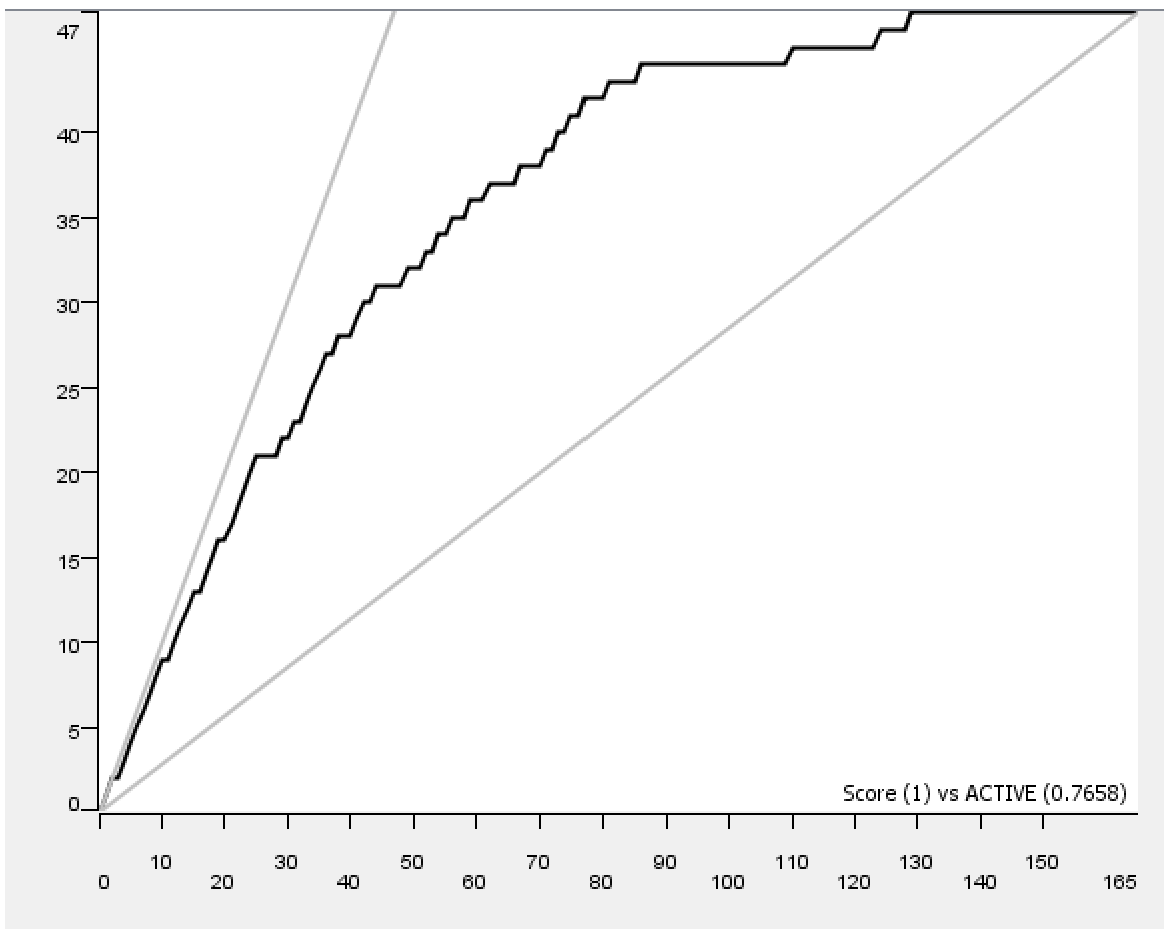

N=165

**Top 10% Mean IC50**	**23 nM**

	**Predicted Active**	**Predicted Inactive**
Active	34	13
Inactive	20	98

<50 nM DefGood in MEK1

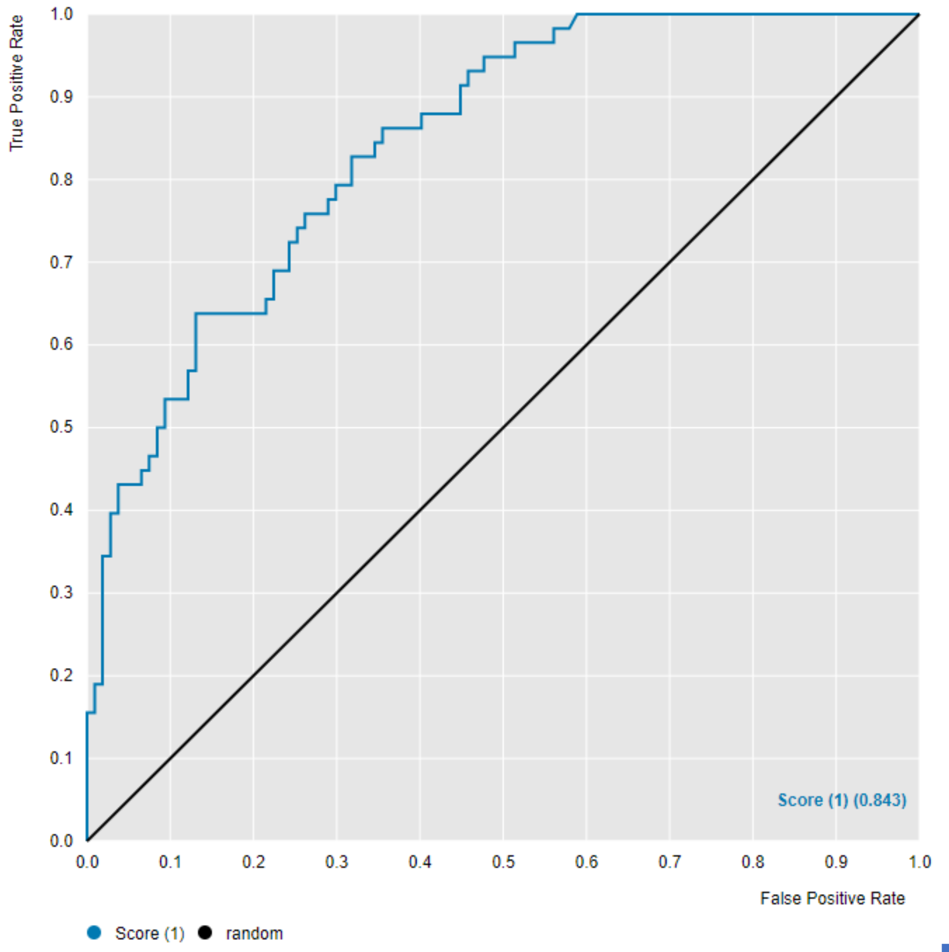

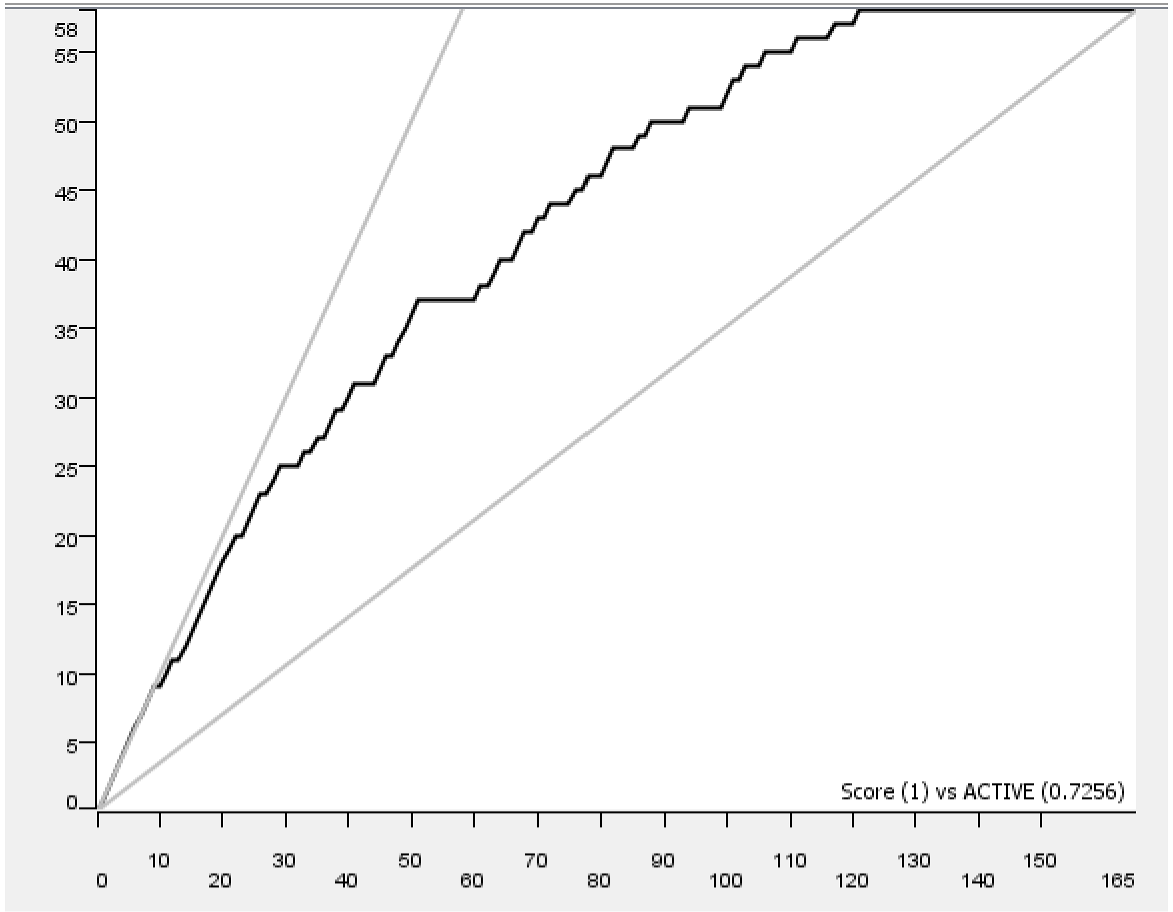

N=165

Top 10% Mean IC50	23.4 Nm

	Predicted Active	Predicted Inactive
Active	37	21
Inactive	19	88

Decision on DefGood

- <35 nM has the best performance with regard to enrichment and mean top 10% IC50, although <50 nM has a similar performance. The precision of the <35 nM NBN was 0.629 whereas the precision of the <50 nM NBN was 0.66. Therefore, <50 nM was used as DefGood

RF - <50 nM DefGood in MEK1

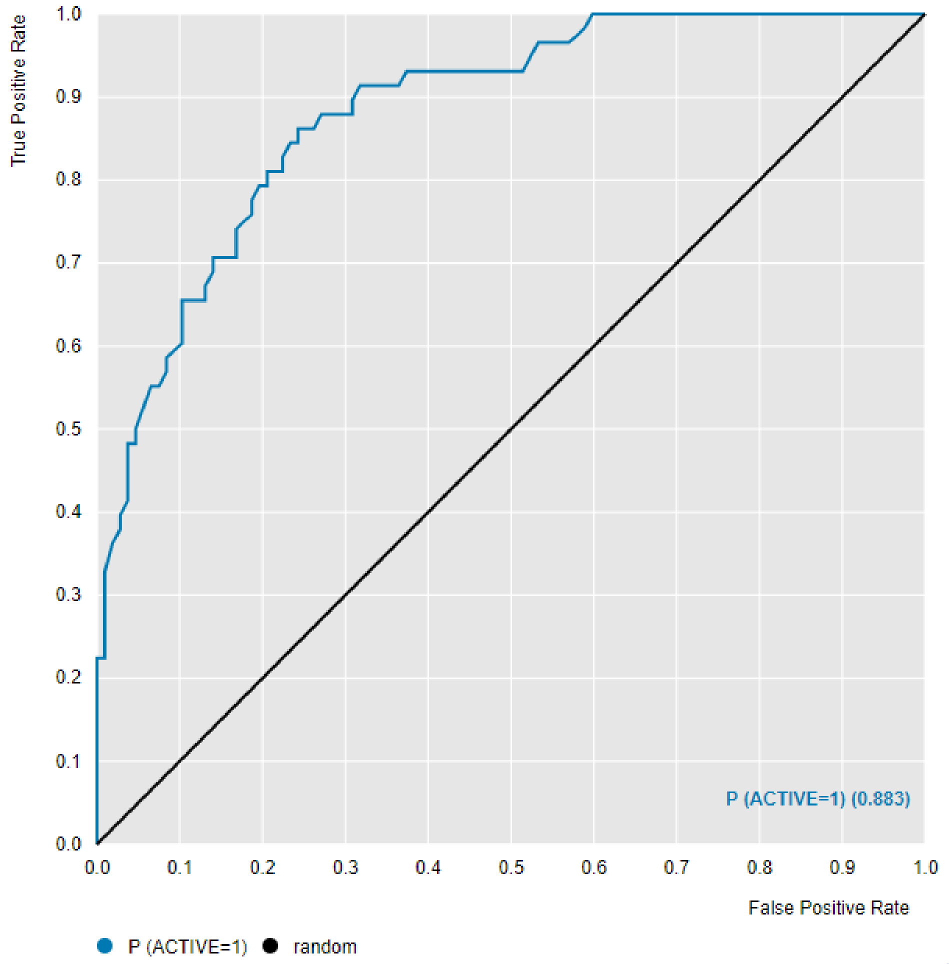

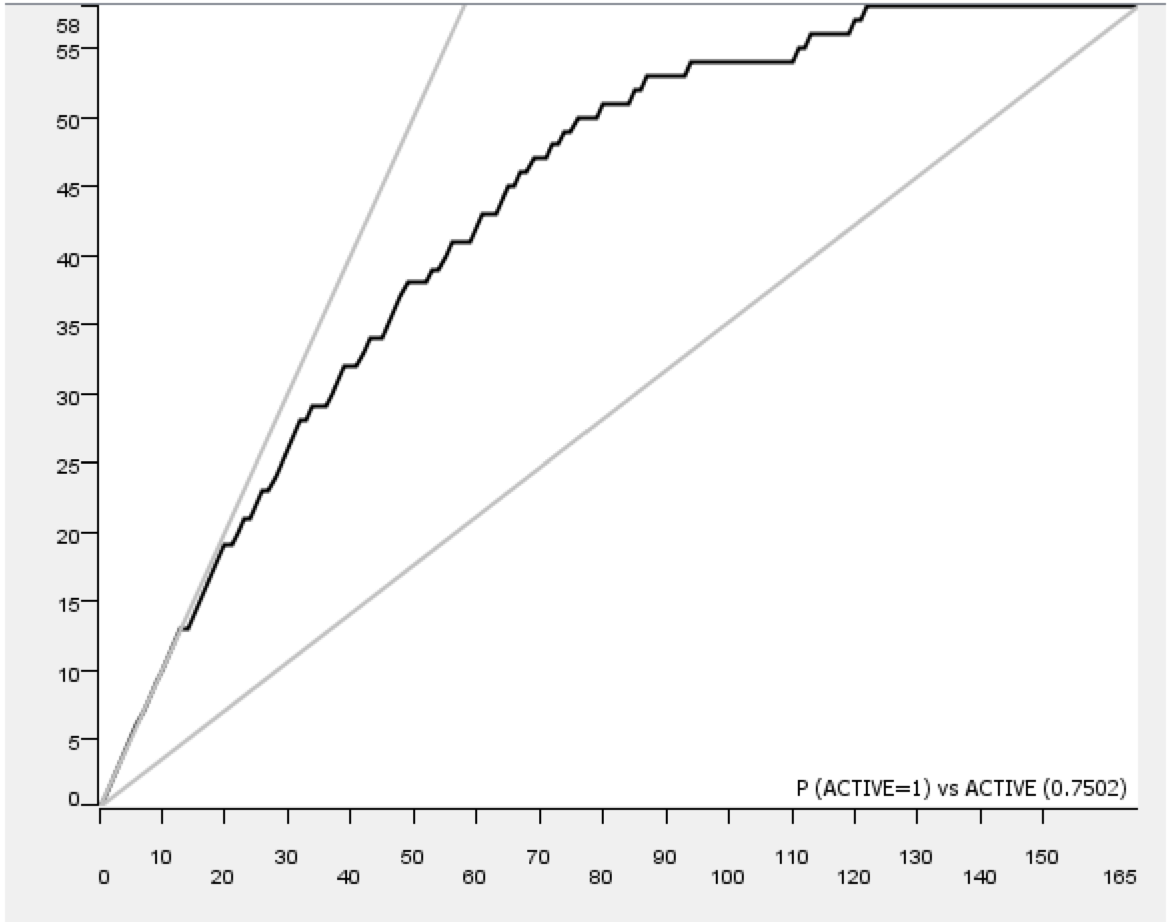

N=165

Top 10% Mean IC50	120 nM (one 1,700 nM mistake)

	Predicted Active	Predicted Inactive
Active	43	15
Inactive	18	89

Decision on DefGood - RF

- Work in ALK showed DefGood in the NBN was similar to DefGood in an RF. Therefore, this value for DefGood from the NBN design was used as DefGood.

PNN - <50 nM DefGood in MEK1

- Properties List (46 calculatable properties were used)
- Parameterization (Global Accuracy for Activity at a 5 nM decision value was used, and a 5-fold stratified sampling cross validation was used to parameterize theta minus and theta plus) Theta Minus = 0.05 and Theta Plus = 0.75

PNN - <50 nM DefGood in MEK1

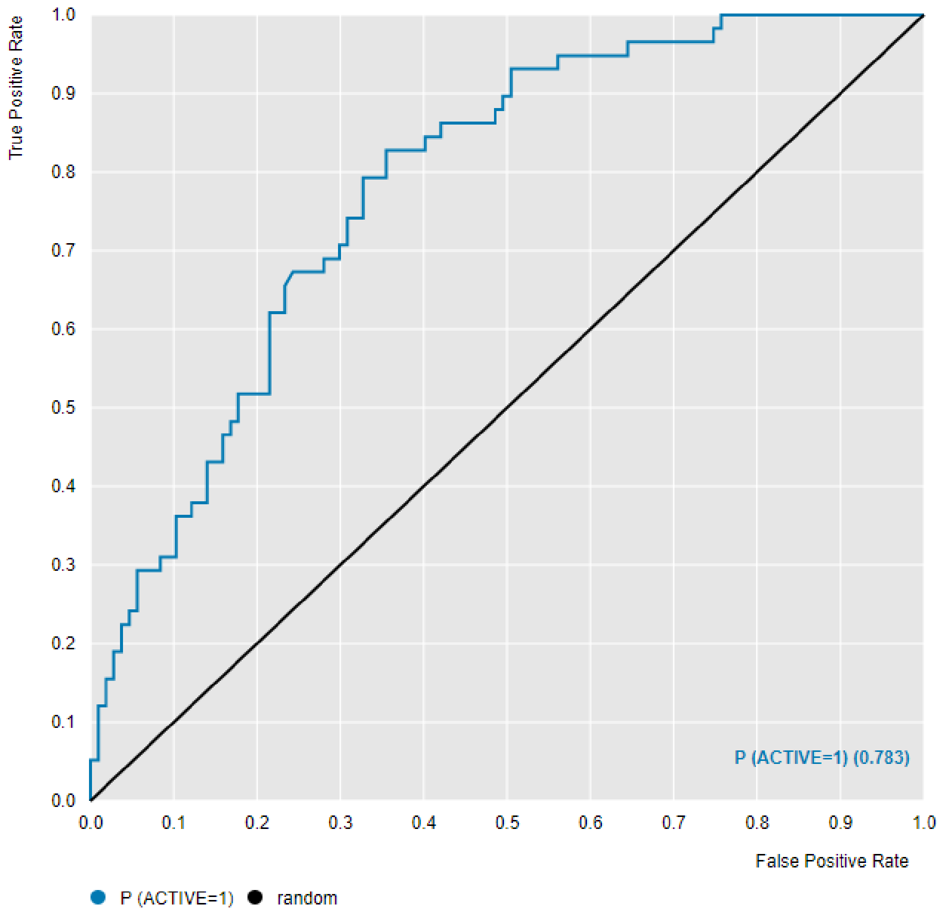

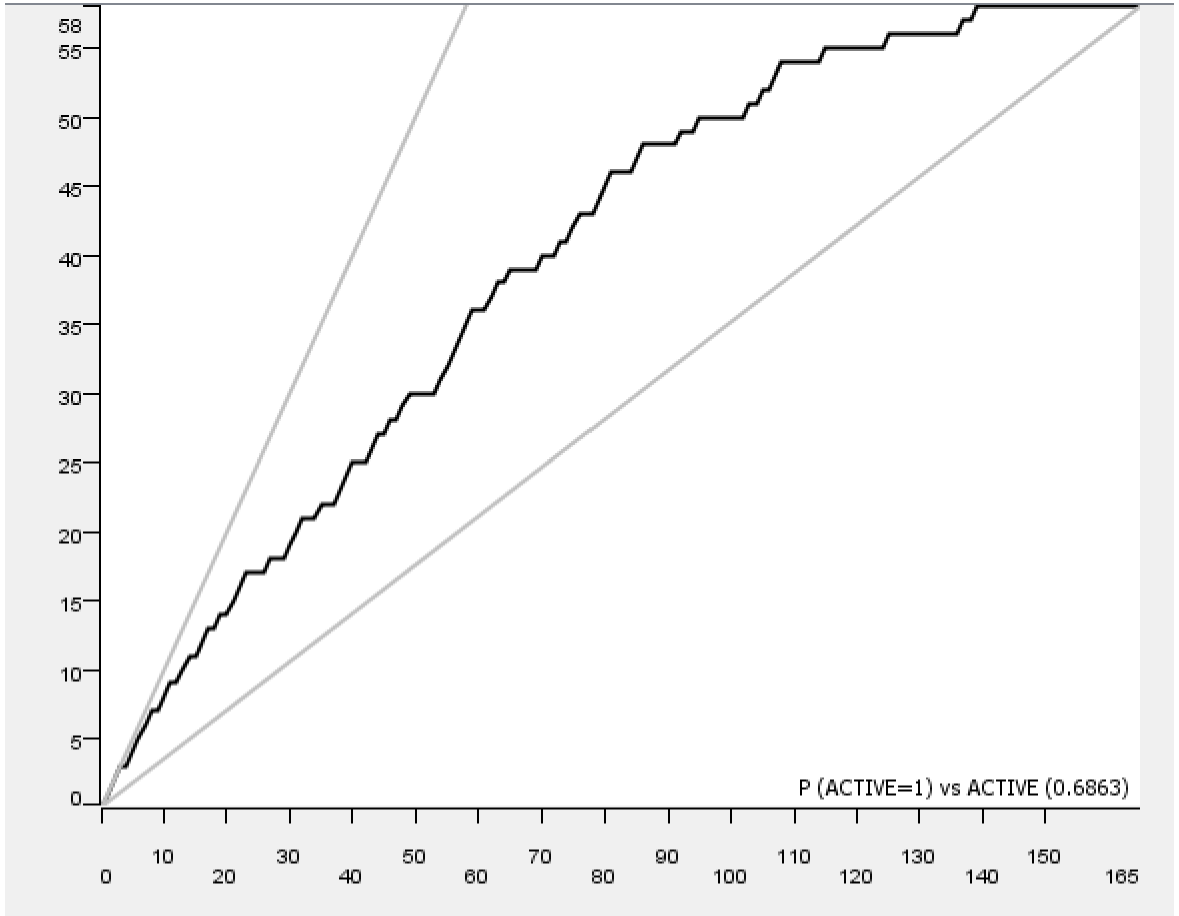

N=165

**Top 10% Mean IC50**	**47.9 nM**

	Predicted Active	Predicted Inactive
Active	30	28
Inactive	19	88

Decision on DefGood - PNN

- Parameterization resulted in a useful PNN with the <50 nM DefGood. Parameterization for each model adds significant time to the process. A rough benchmark can be applied where point of failure is discovered and then the model is parameterized to ensure that the situation is optimal for the PNN. This will be applied to the discovery of the error tolerance threshold for the PNN as was done in the beta 2 adrenergic receptor space.

NBN Error Tolerance- <50 nM DefGood in MEK1

- 0-50% absolute error

<50 nM DefGood in MEK1, 5% error; Random seed = 1515533876005

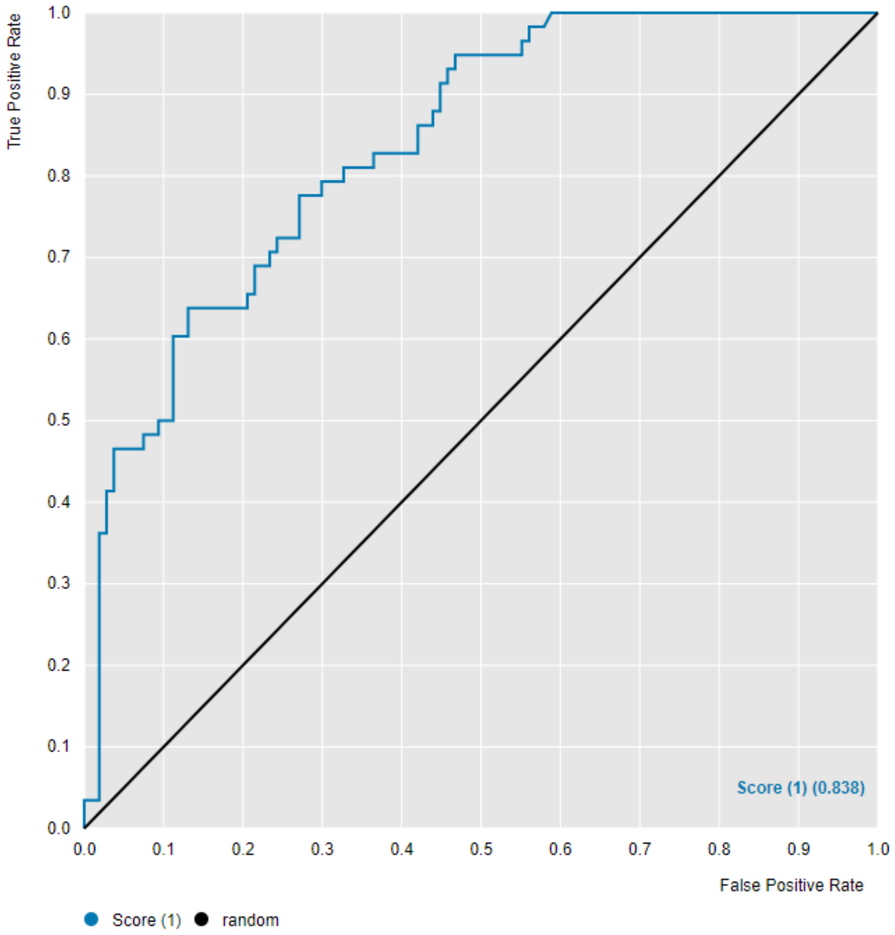

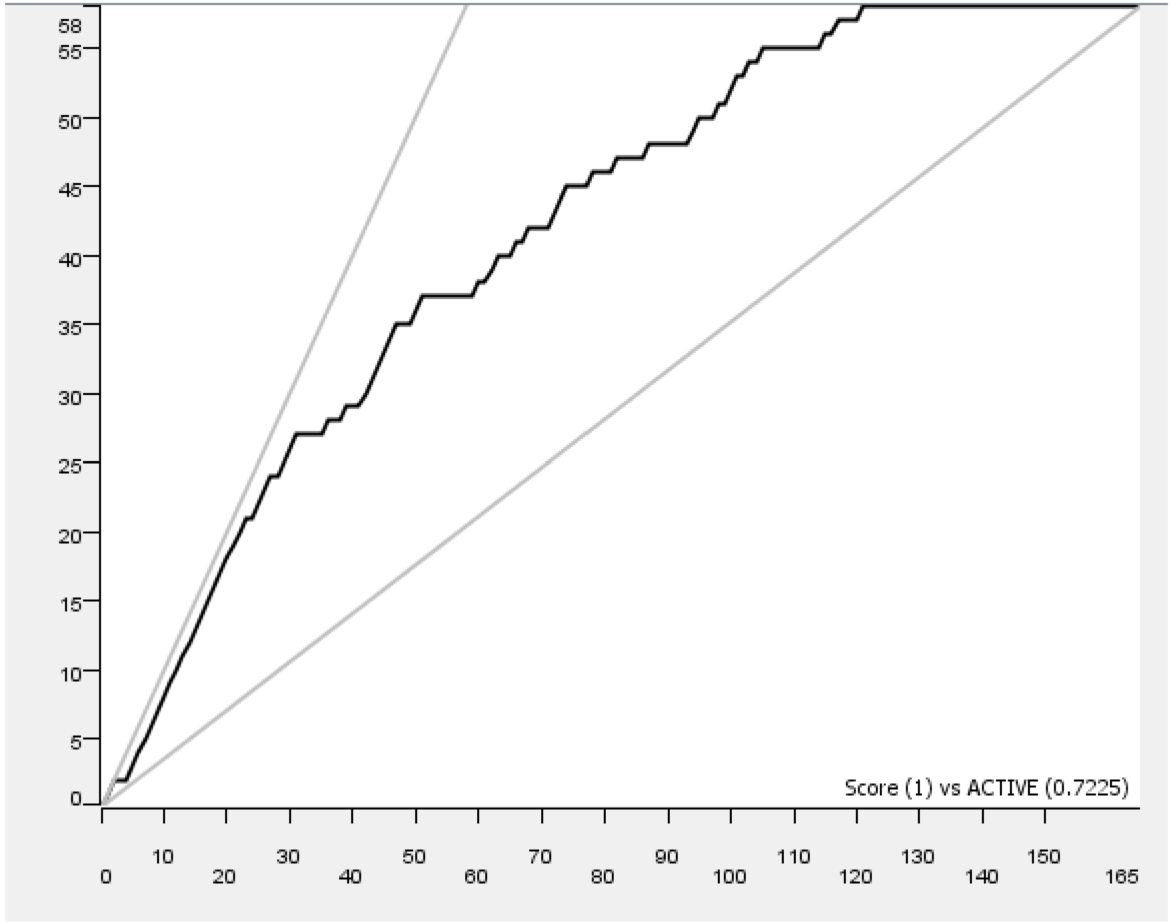

N=165

Top 10% Mean IC50	20.4 nM

	Predicted Active	Predicted Inactive
Active	45	13
Inactive	31	76

<50nM DefGood in MEK1, 10% error

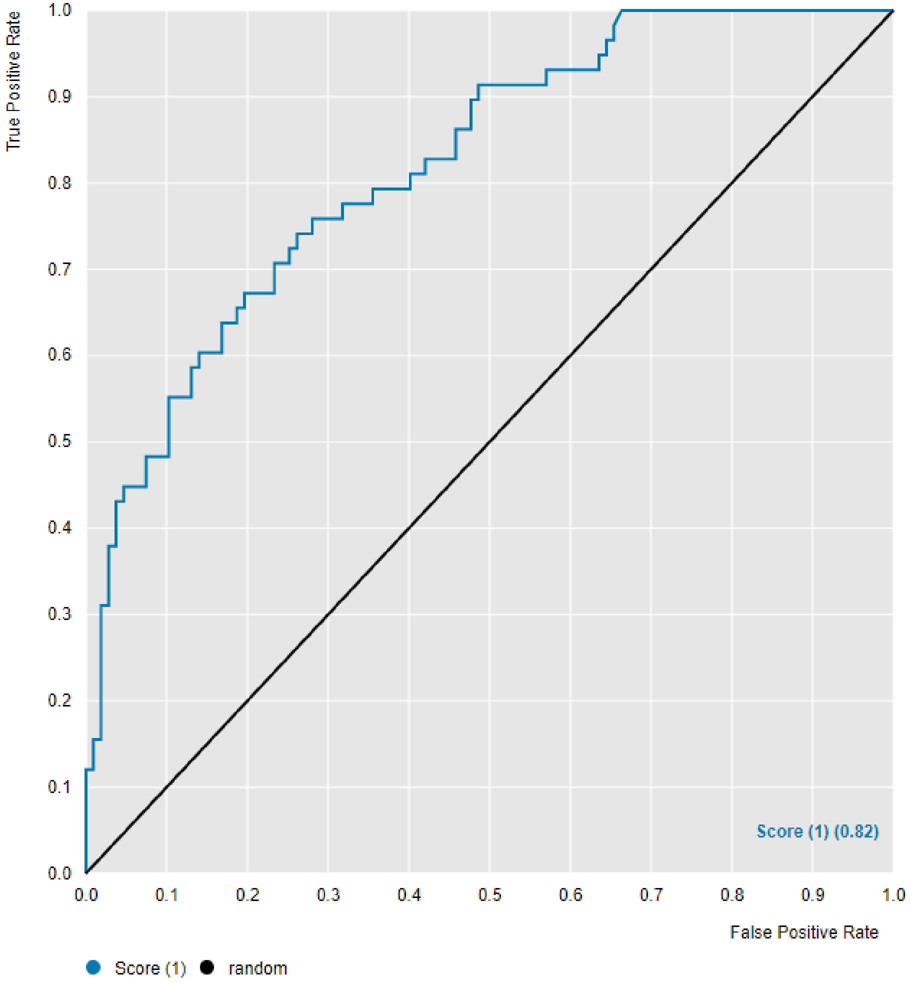

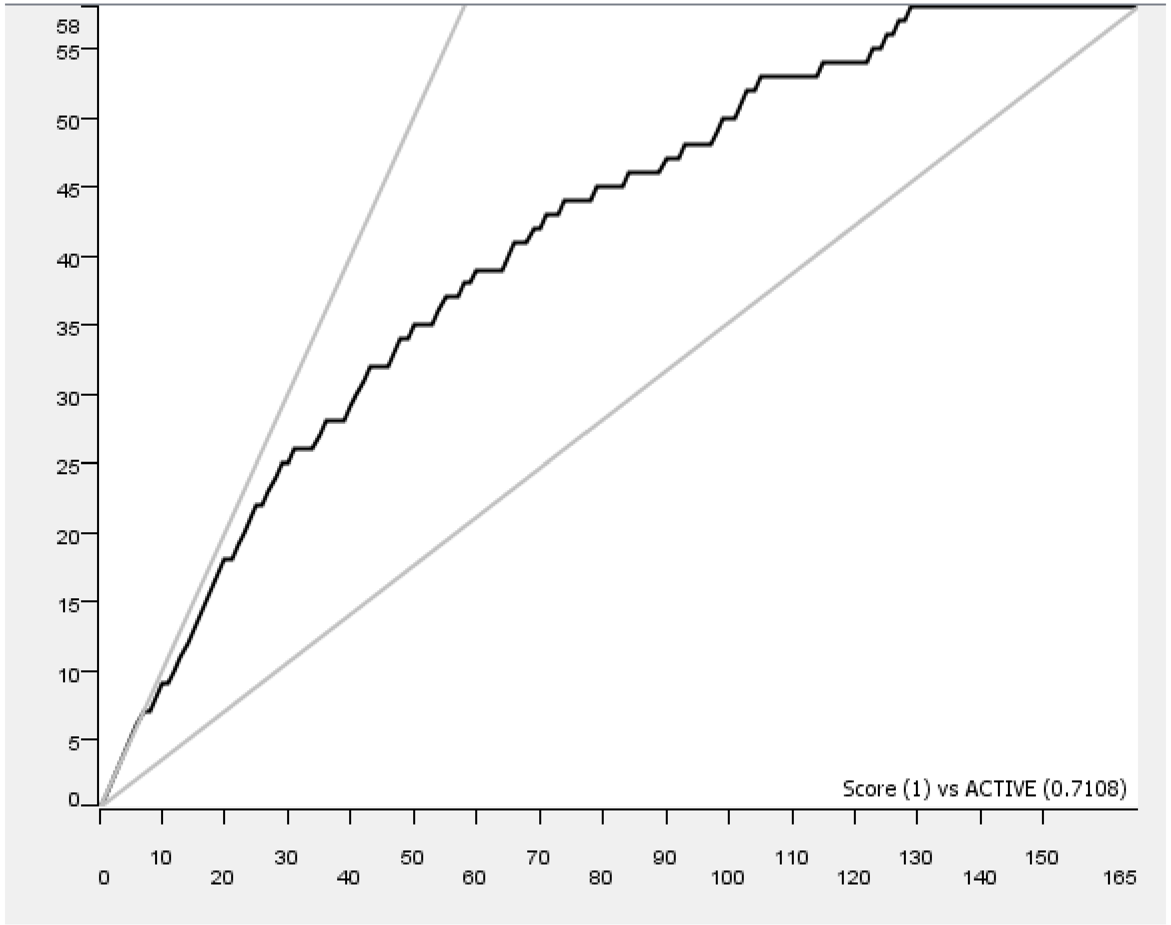

N=165

Top 10% Mean IC50	23.4 nM

	Predicted Active	Predicted Inactive
Active	41	17
Inactive	26	81

<50nM DefGood in MEK1, 20% error

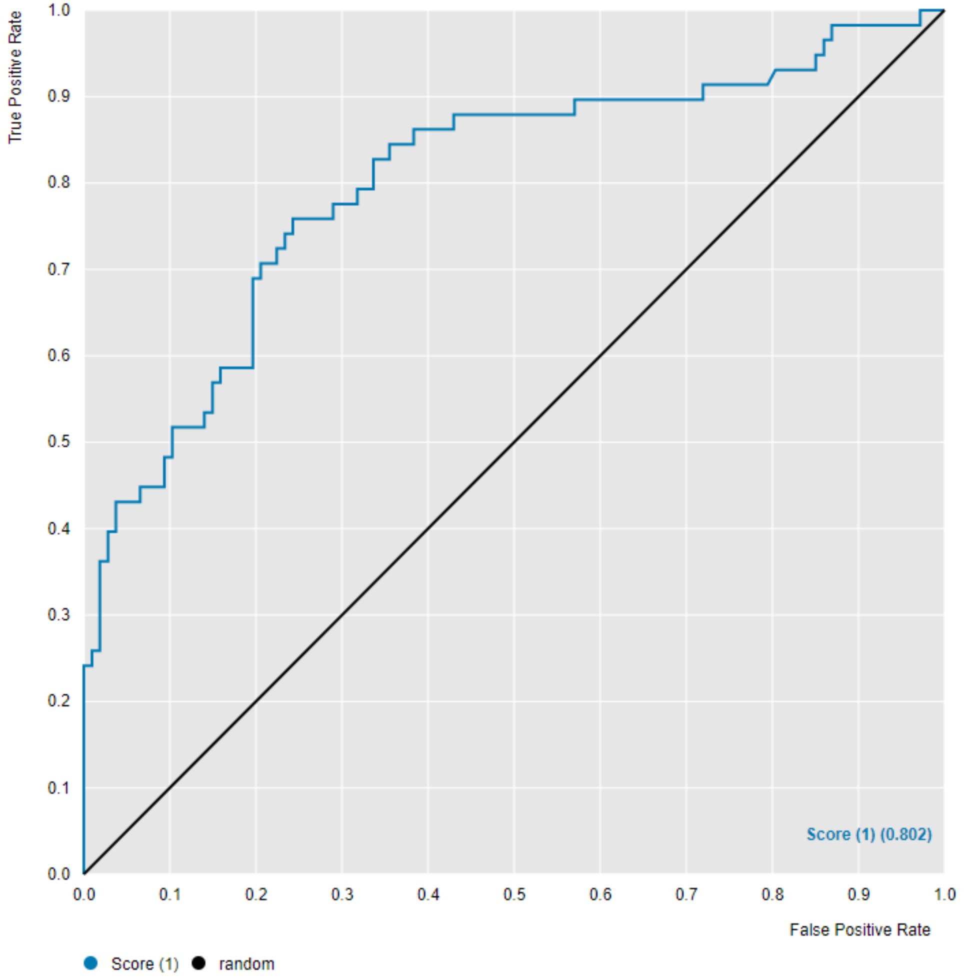

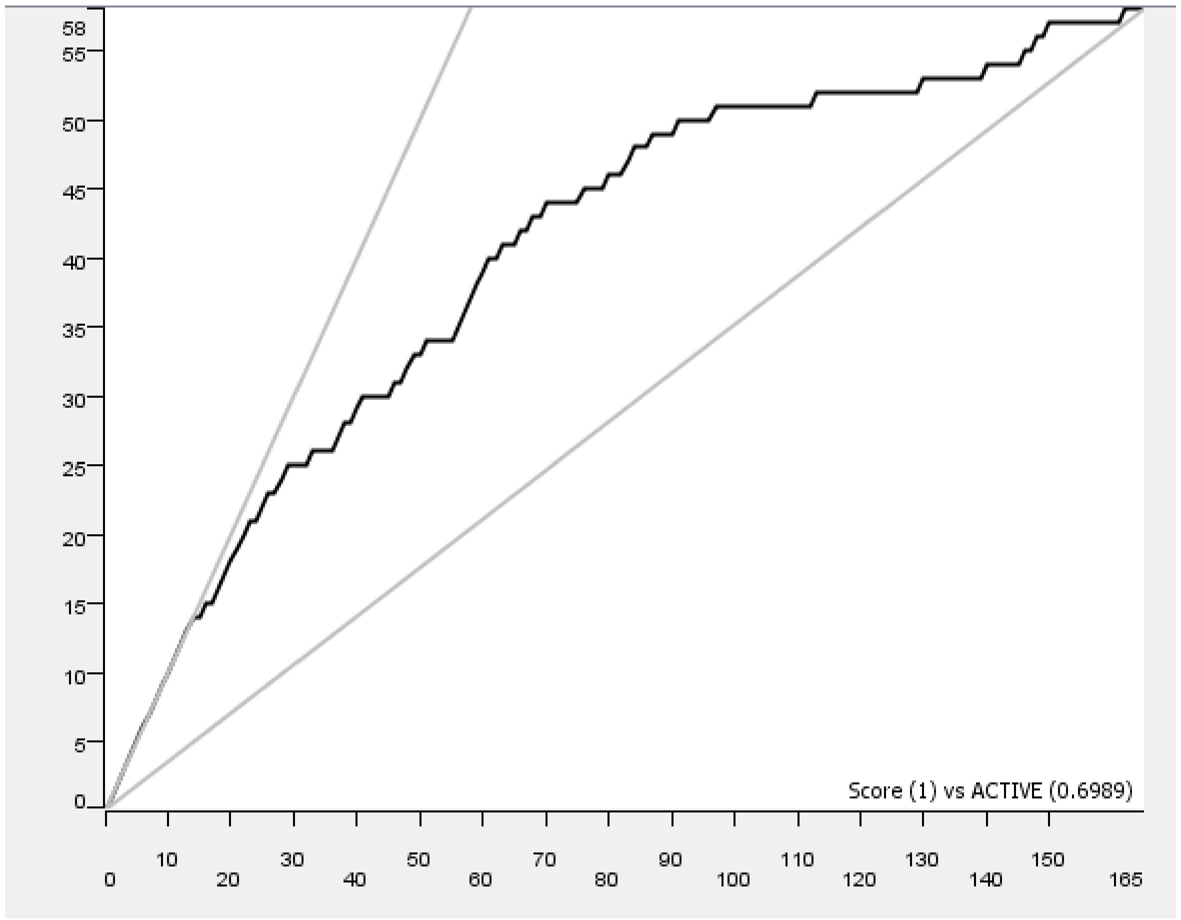

N=750

Top 10% Mean IC50	23.1 nM

	Predicted Active	Predicted Inactive
Active	51	7
Inactive	49	58

<50nM DefGood in MEK1, 30% error

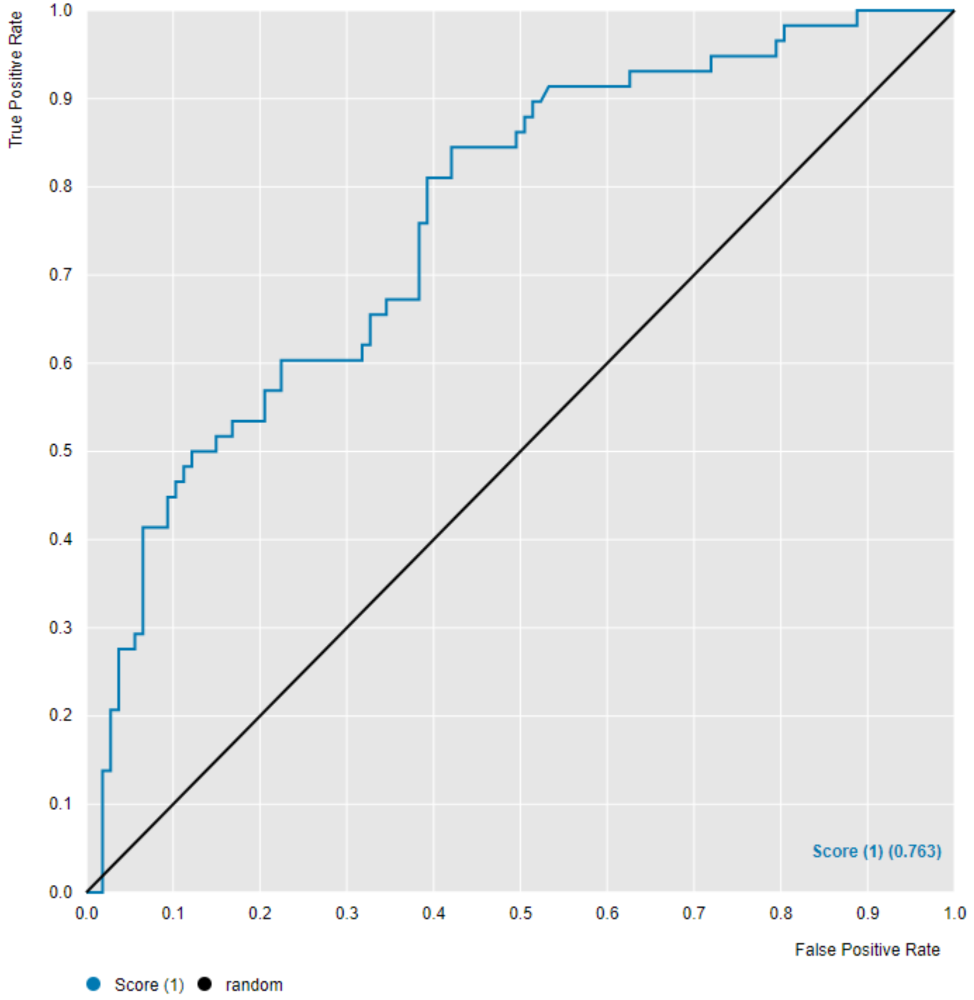

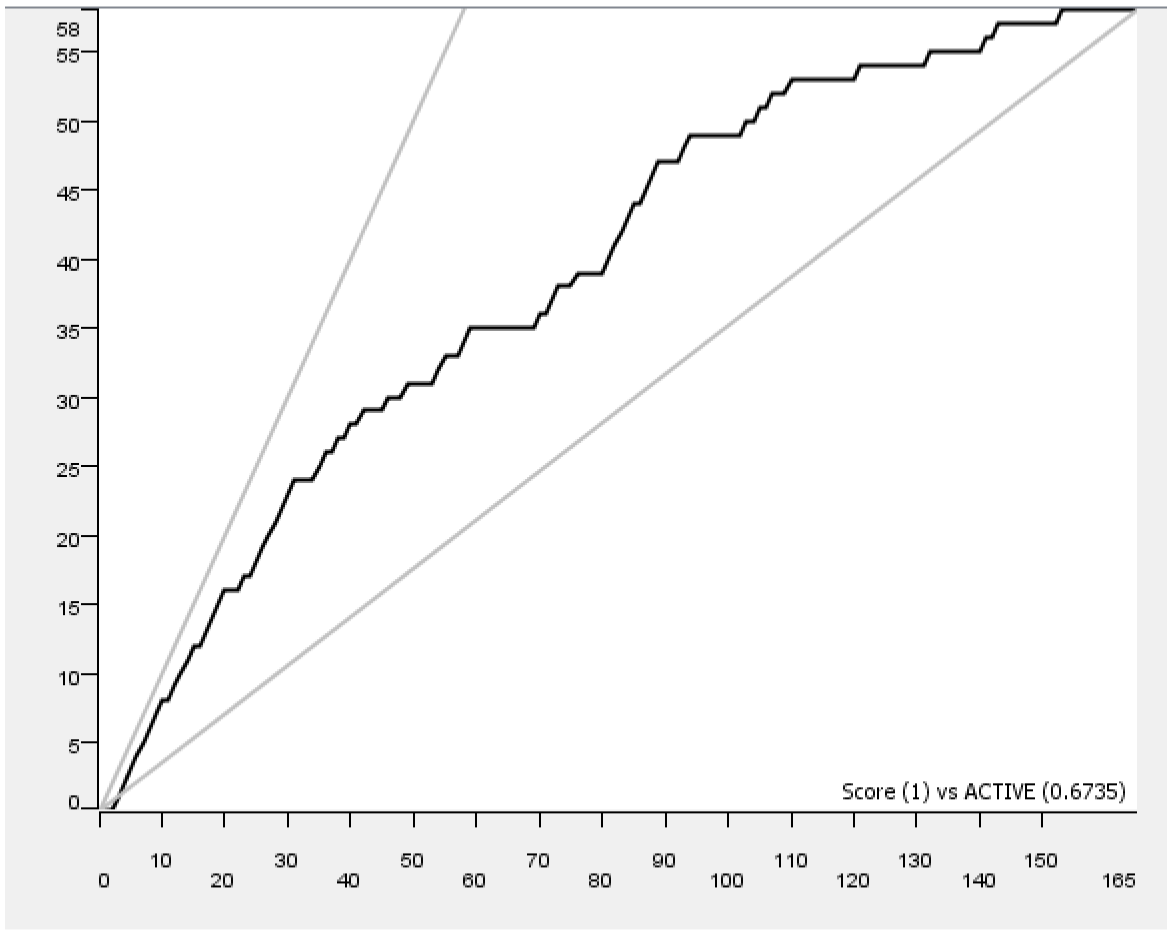

N=165

Top 10% Mean IC50	419.7 nM

	Predicted Active	Predicted Inactive
Active	53	5
Inactive	64	43

<50nM DefGood in MEK1, 35% error

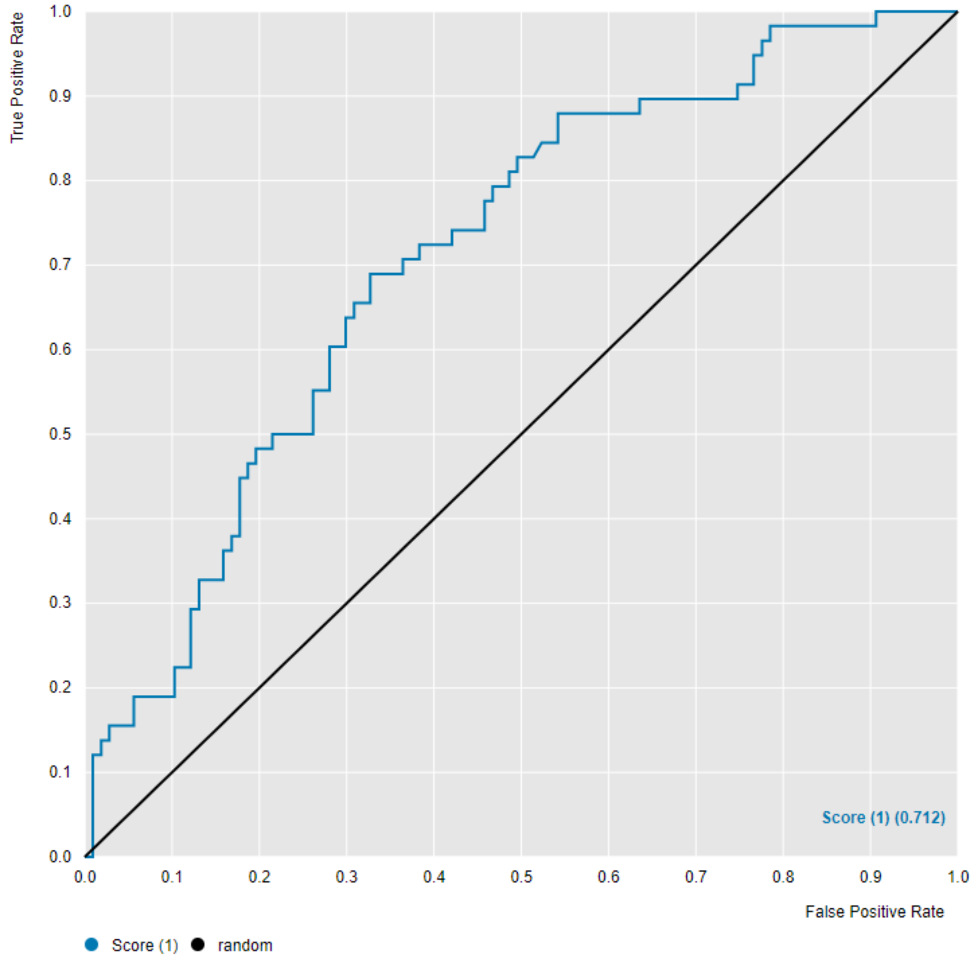

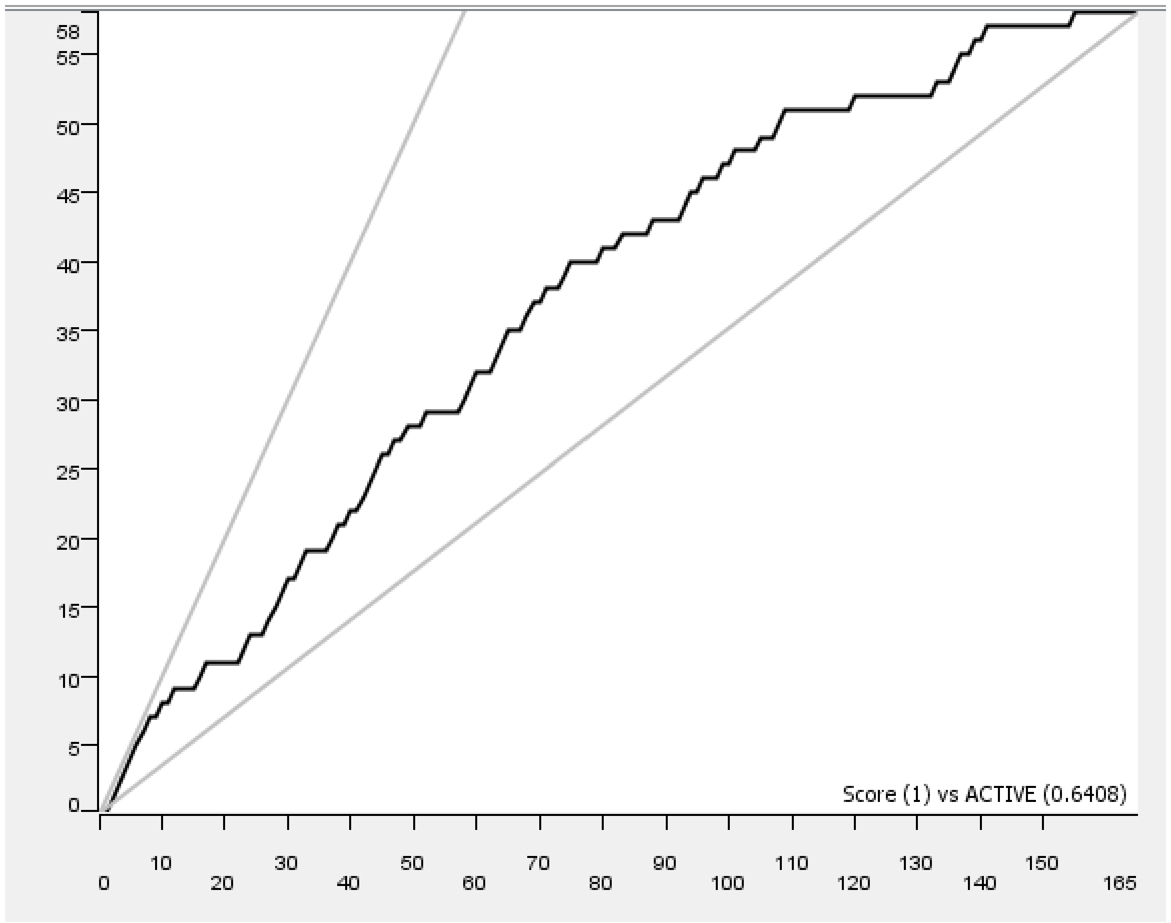

N=165

Top 10% Mean IC50	491 nM

	Predicted Active	Predicted Inactive
Active	51	7
Inactive	63	44

<50nM DefGood in MEK1, 40% error

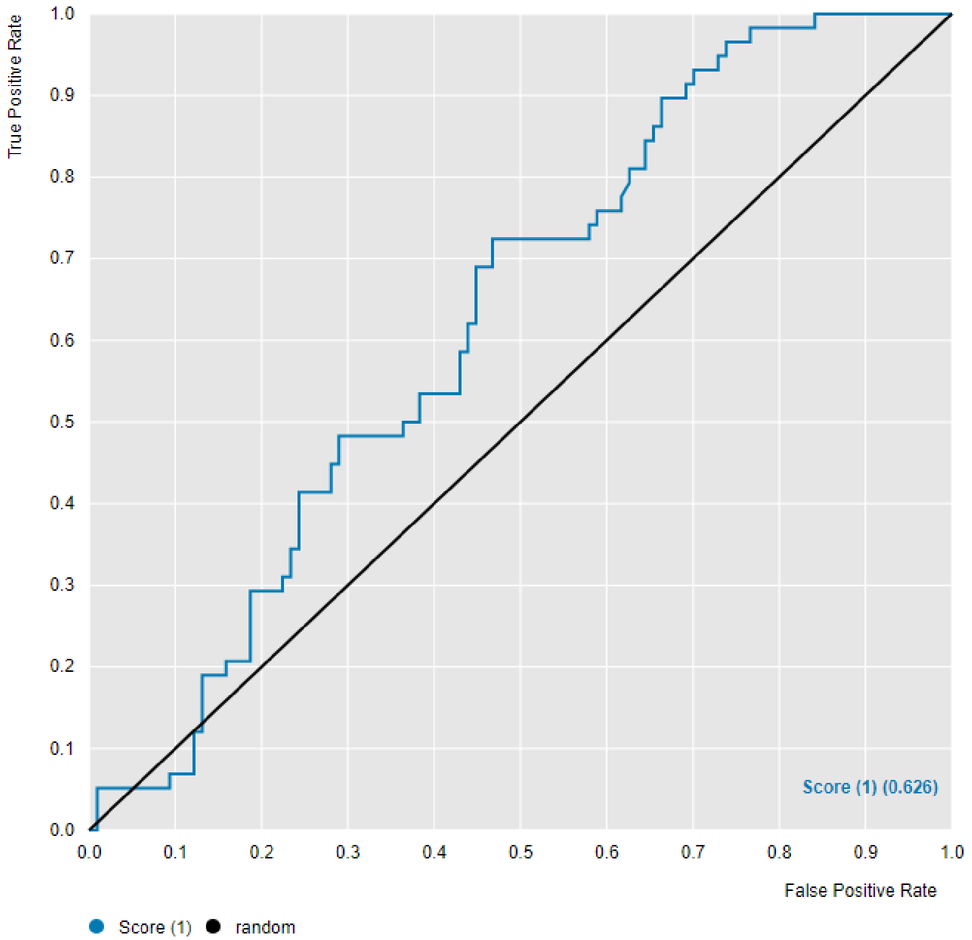

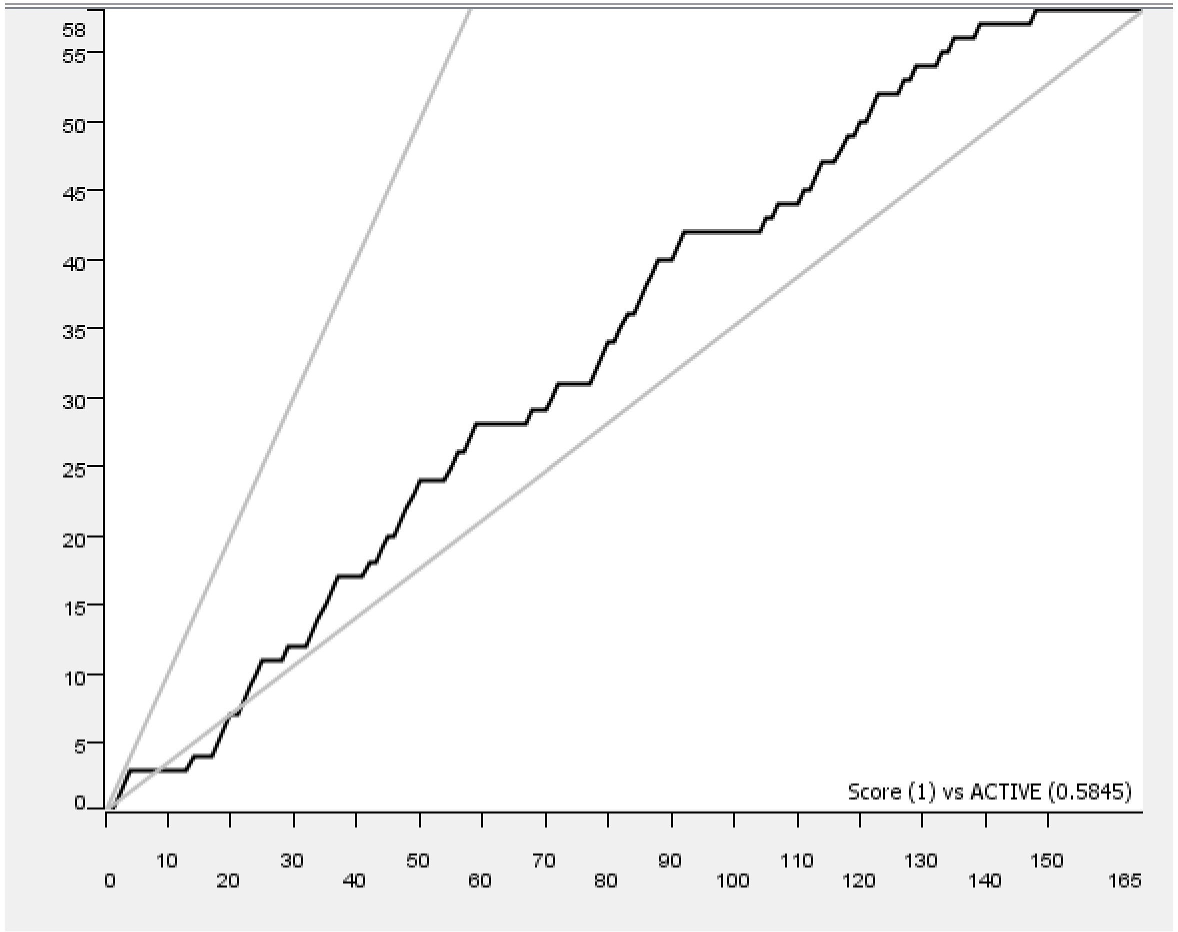

N=165

Top 10% Mean IC50	3,700 nM

	Predicted Active	Predicted Inactive
Active	42	16
Inactive	54	53

<50nM DefGood in MEK1, 20% error; Random seed = 429

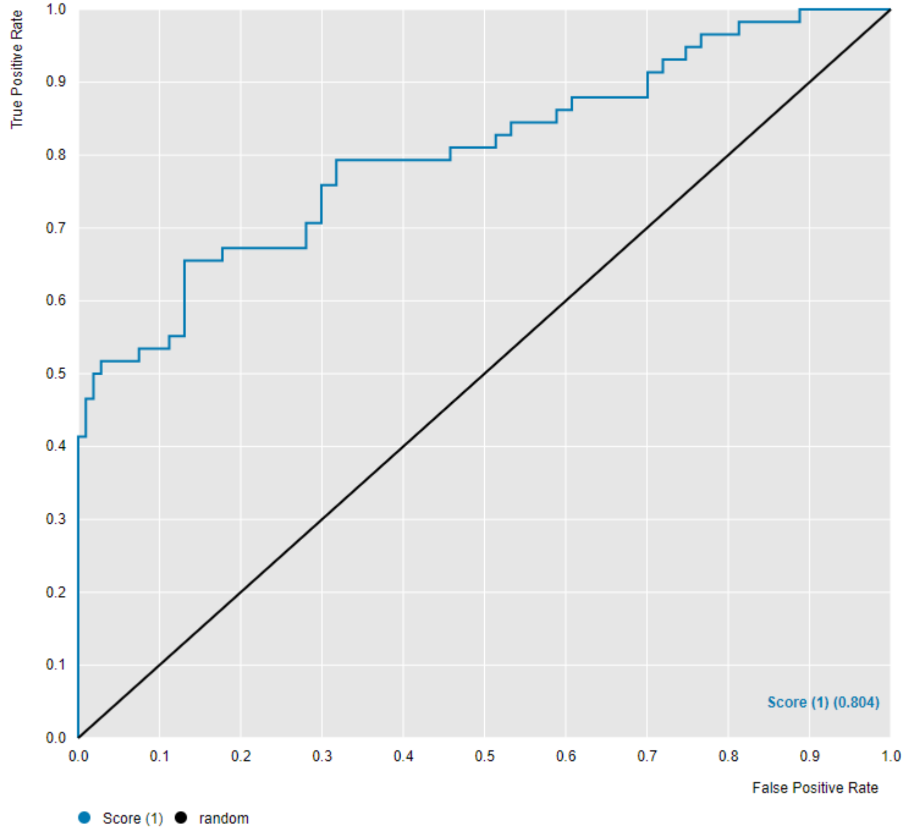

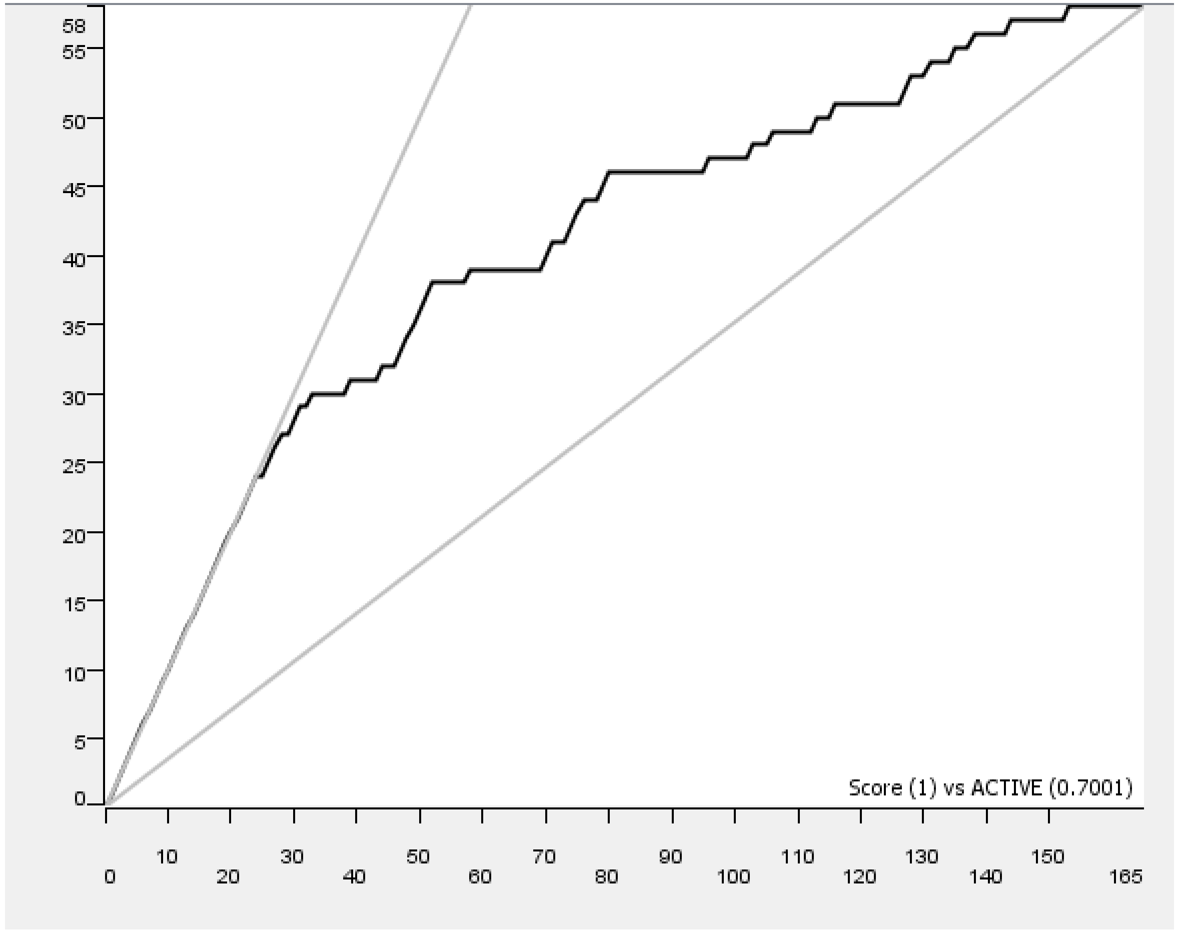

N=165

Top 10% Mean IC50	22.6 nM

	Predicted Active	Predicted Inactive
Active	40	18
Inactive	30	77

<50nM DefGood in MEK1, 30% error; Random seed = 429

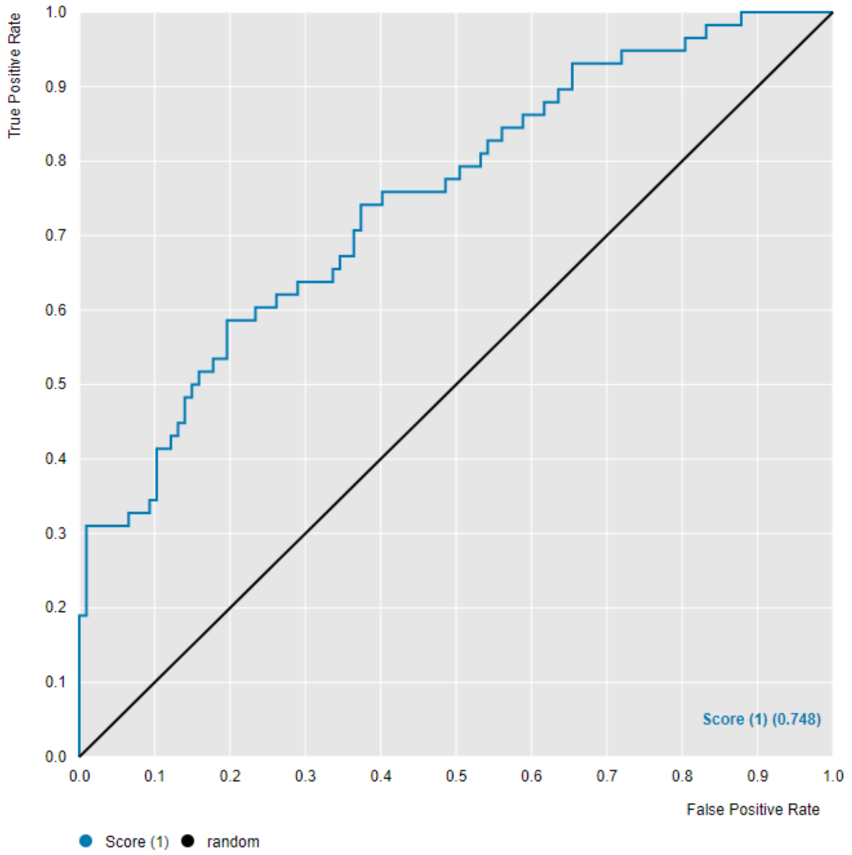

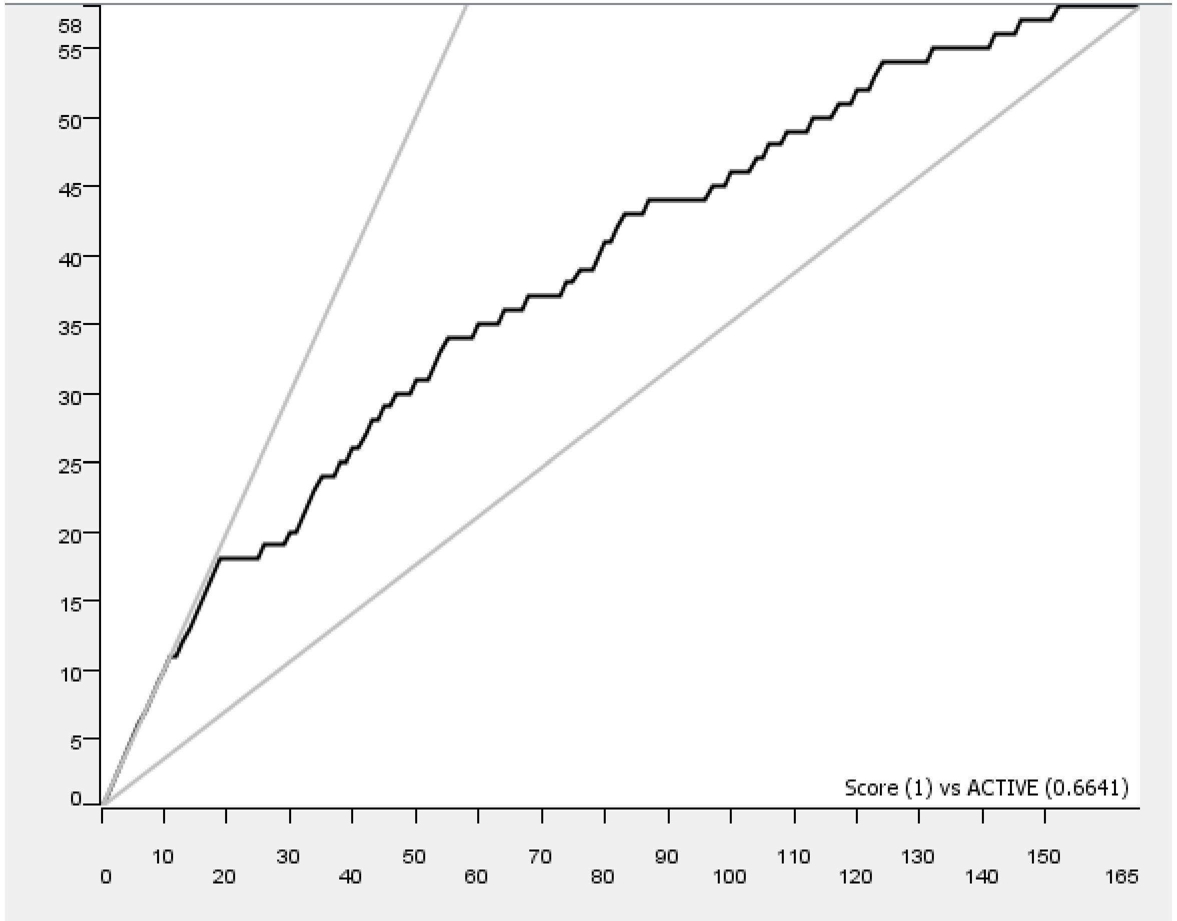

N=165

Top 10% Mean IC50	59.8 nM

	Predicted Active	Predicted Inactive
Active	40	18
Inactive	39	68

<50nM DefGood in MEK1, 35% error; Random seed = 429

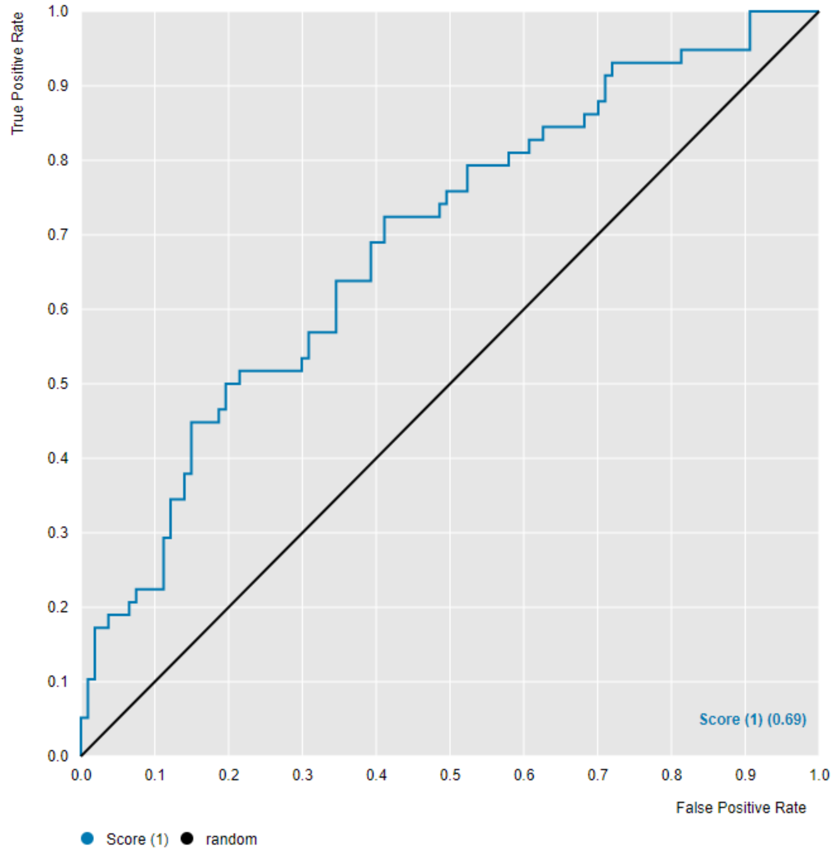

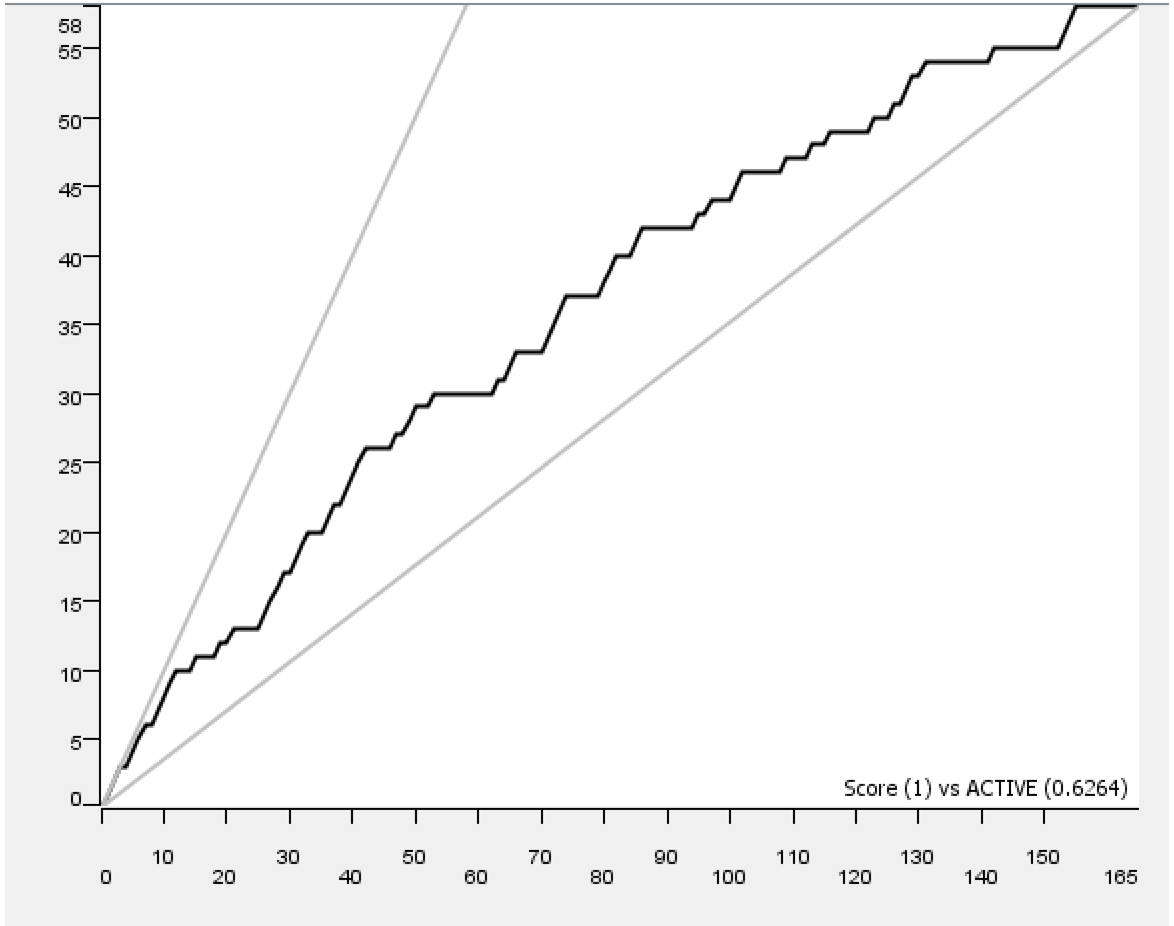

N=165

**Top 10% Mean IC50**	**96.0 nM**

	Predicted Active	Predicted Inactive
Active	41	17
Inactive	44	63

<50nM DefGood in MEK1, 40% error; Random seed = 429

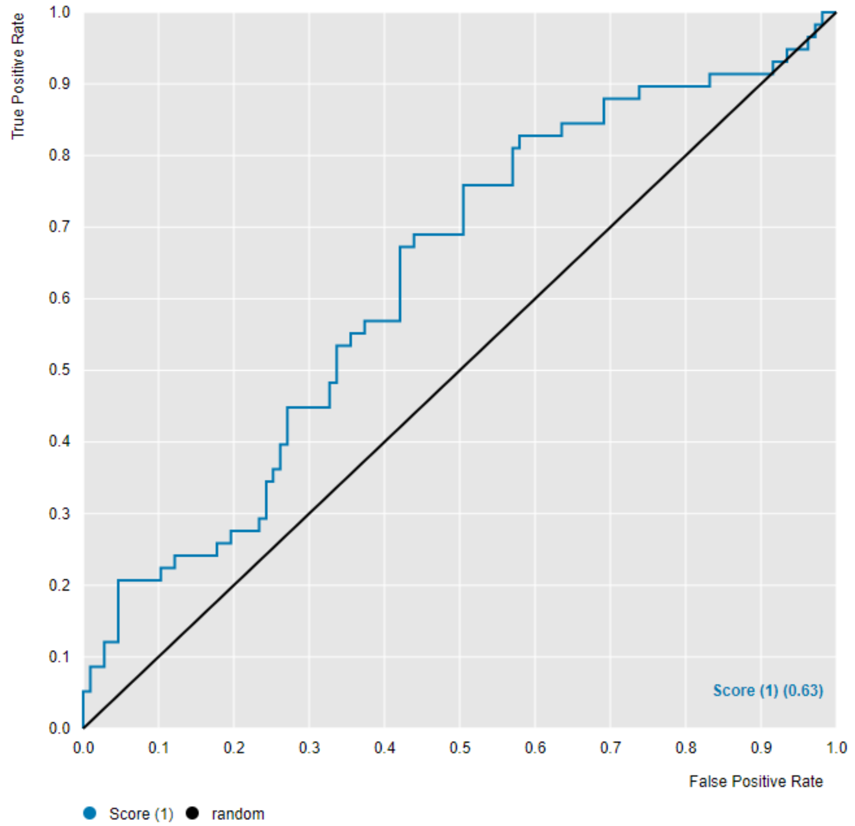

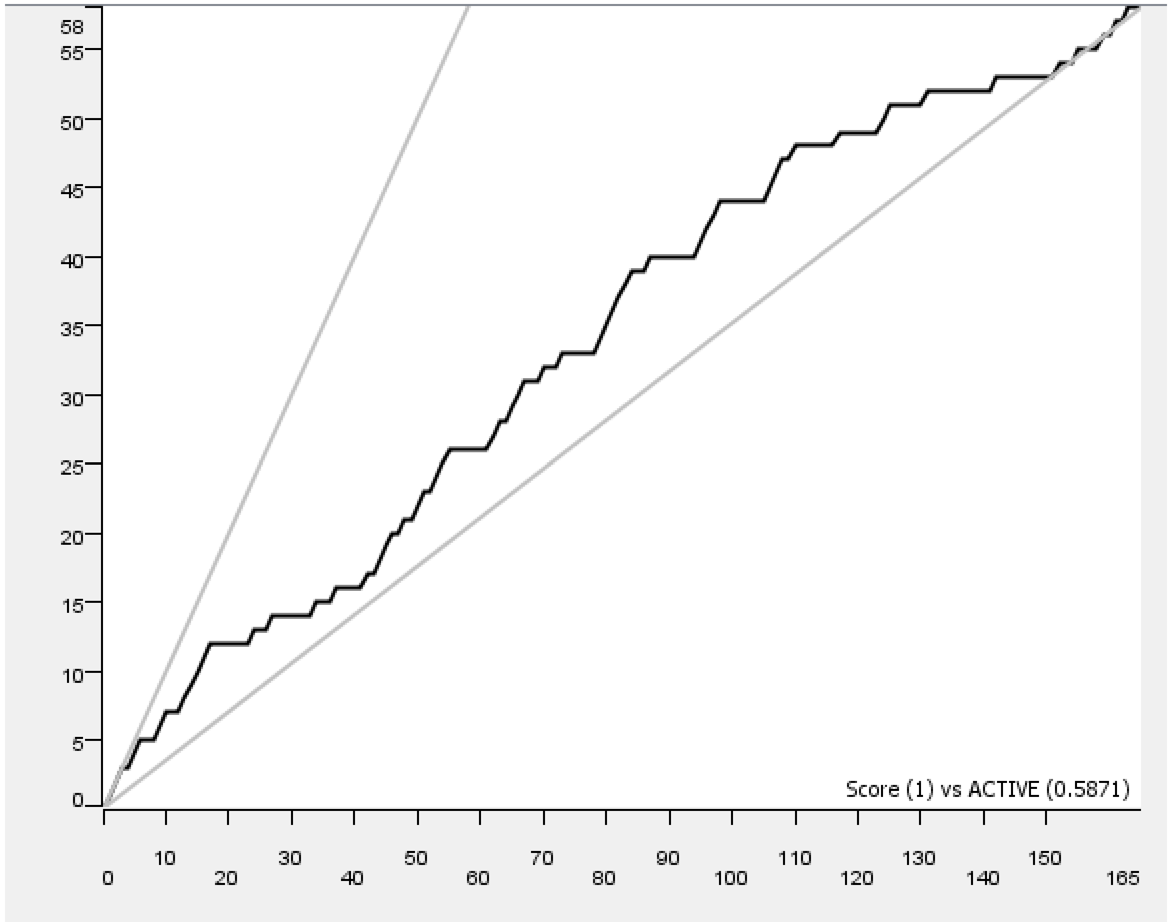

N=165

**Top 10% Mean IC50**	**69.0 nM**

	Predicted Active	Predicted Inactive
Active	55	3
Inactive	100	7

<50nM DefGood in MEK1, 20% error; Random seed = 121783

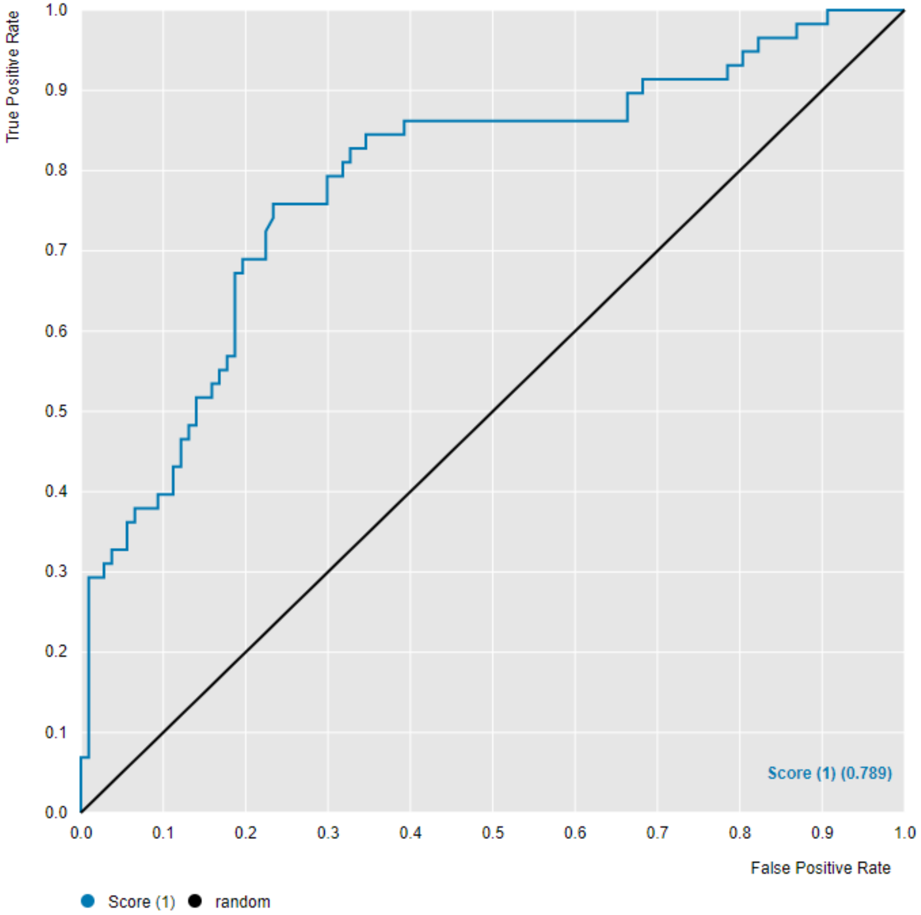

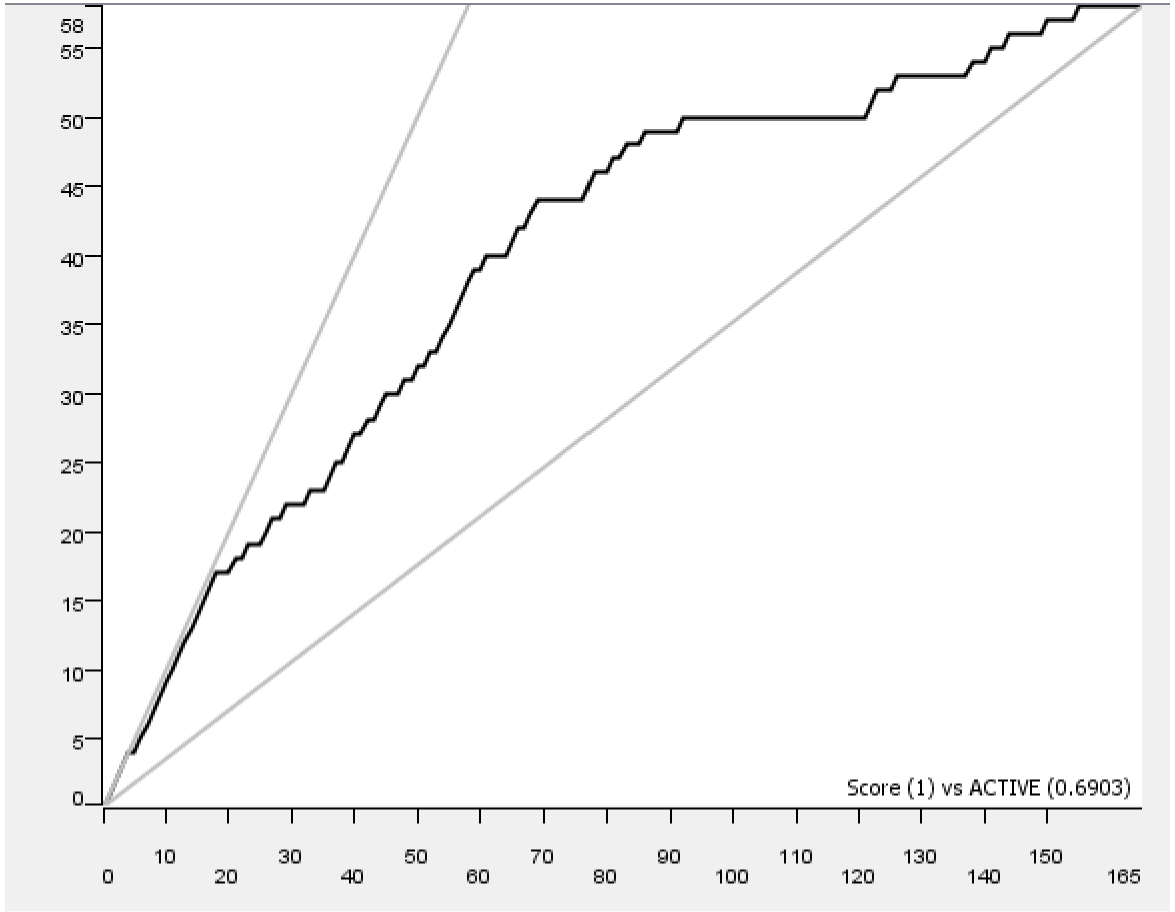

N=165

**Top 10% Mean IC50**	**20.8 nM**

	Predicted Active	Predicted Inactive
Active	39	19
Inactive	20	87

<50nM DefGood in MEK1, 30% error; Random seed = 121783

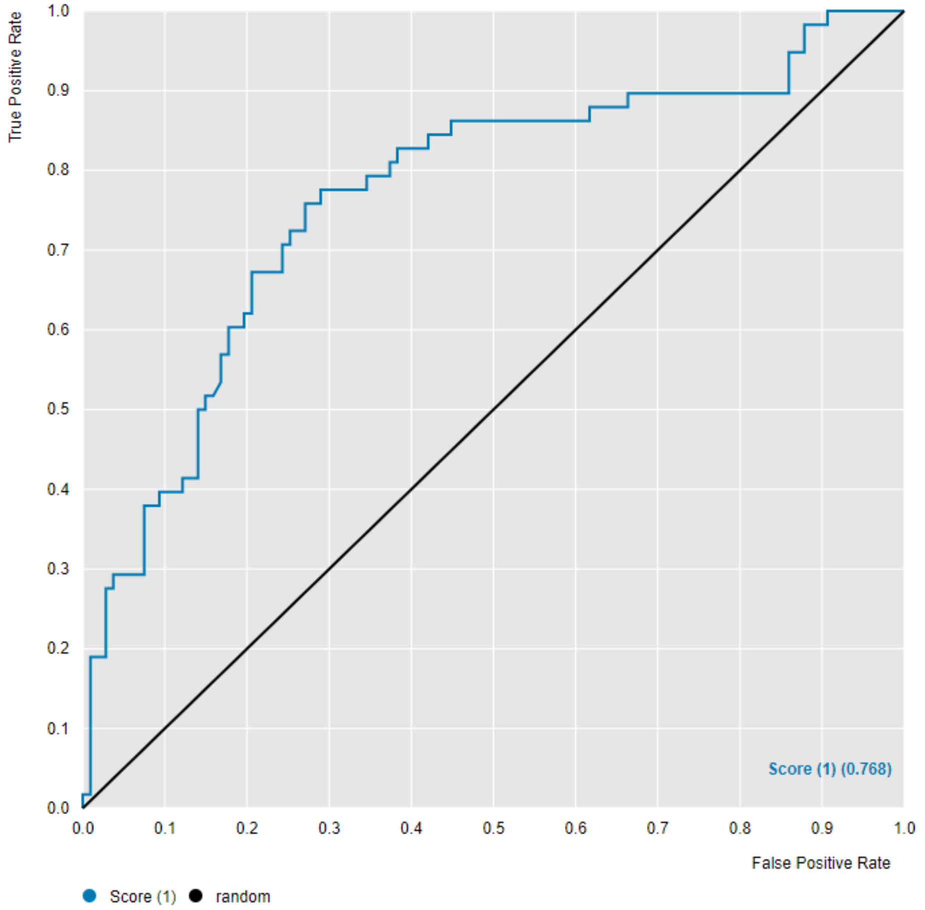

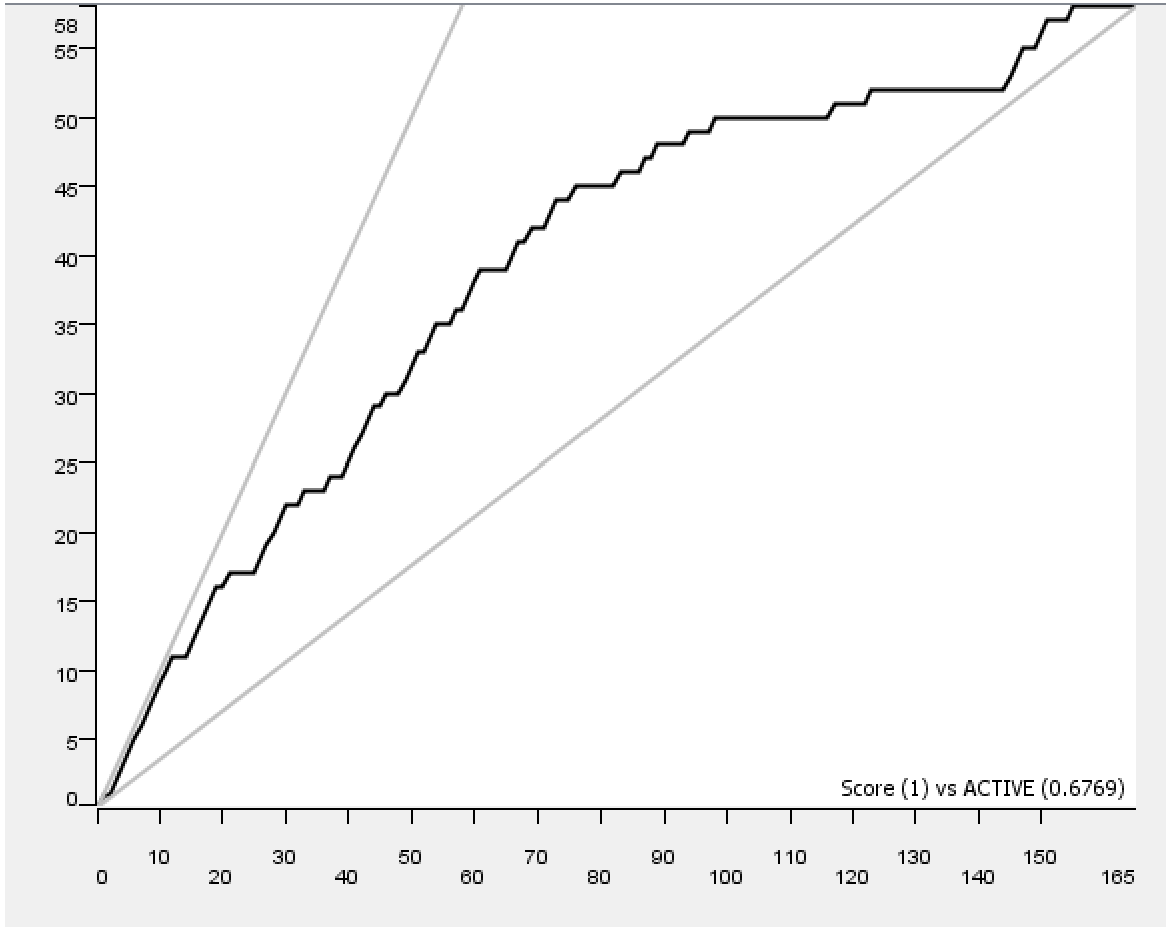

N=165

Top 10% Mean IC50	40.6 nM

	Predicted Active	Predicted Inactive
Active	44	14
Inactive	29	78

<50nM DefGood in MEK1, 35% error; Random seed = 121783

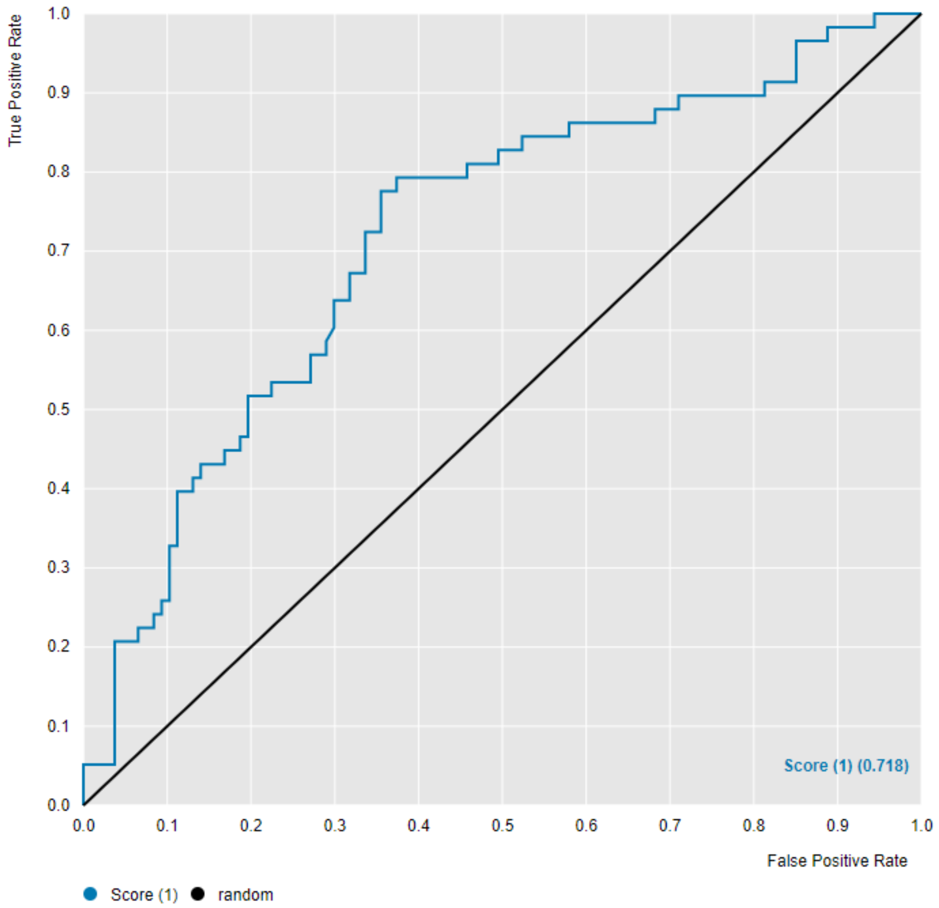

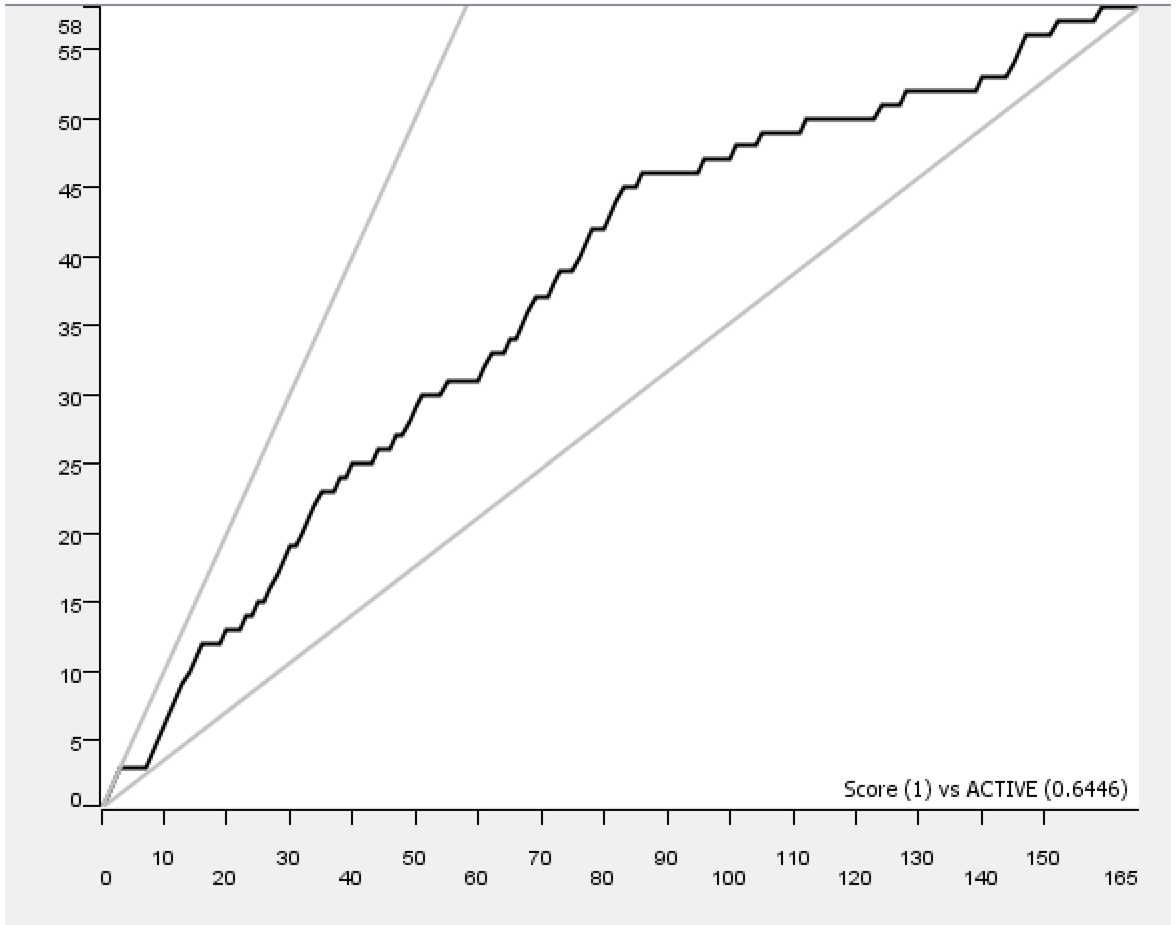

N=165

**Top 10% Mean IC50**	**45.5 nM**

	Predicted Active	Predicted Inactive
Active	49	9
Inactive	56	51

<50nM DefGood in MEK1, 40% error; Random seed = 121783

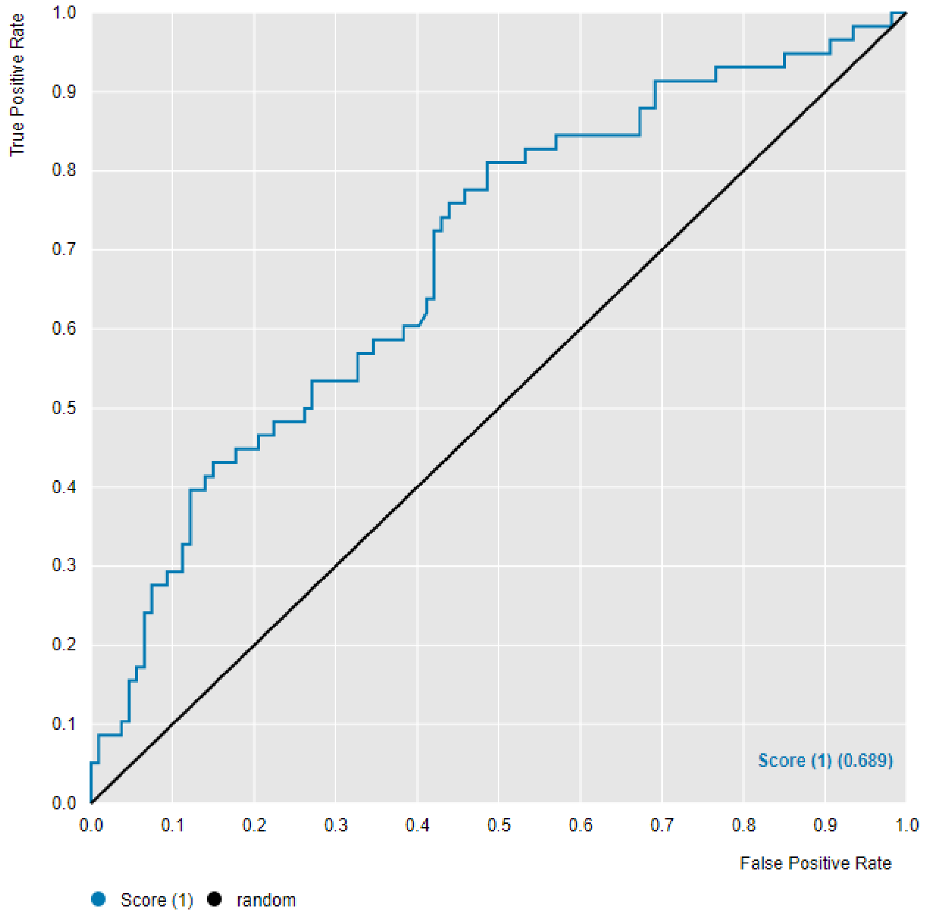

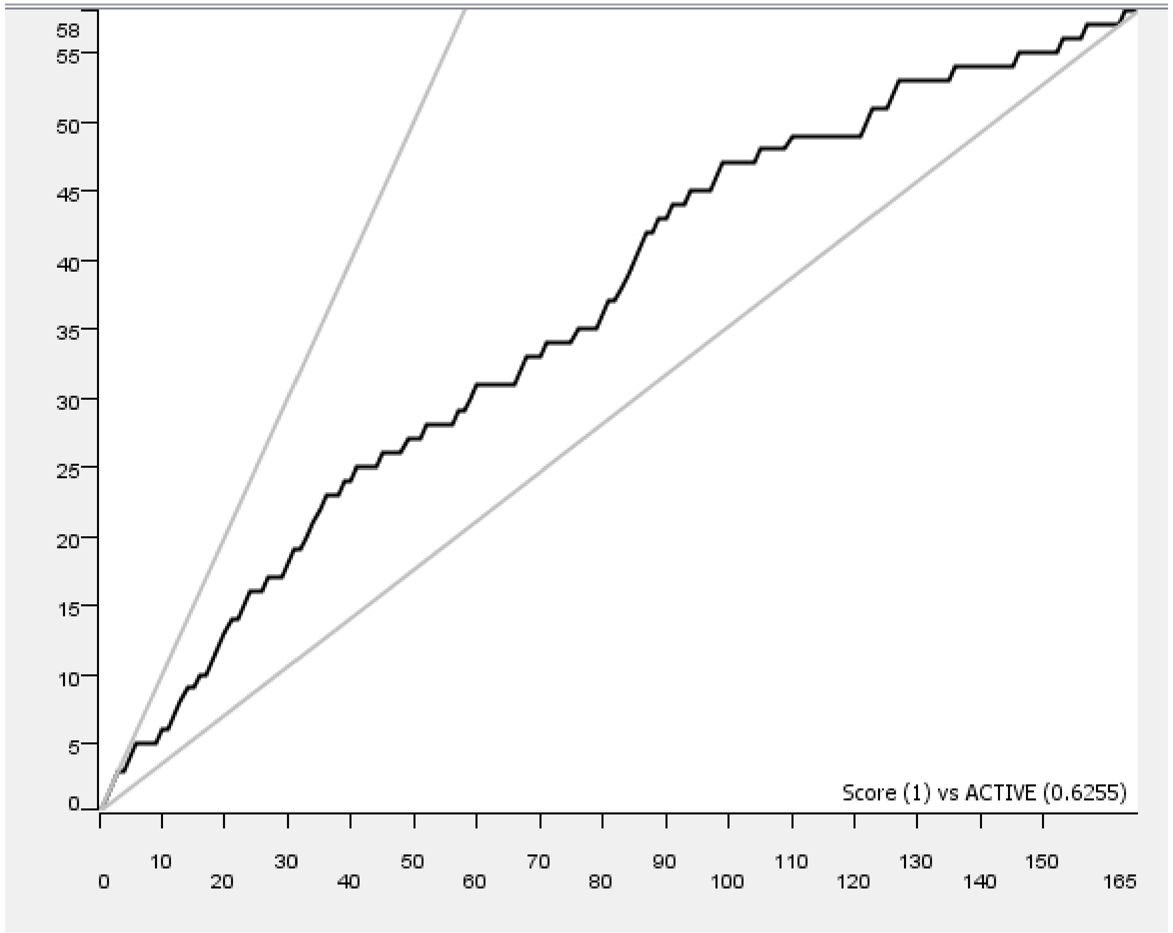

N=165

Top 10% Mean IC50	104 nM

	Predicted Active	Predicted Inactive
Active	49	9
Inactive	70	37

Conclusion - NBN

- An NBN could be generated for MEK1 kinase with a decision value of <50 nM as defined as active. The predictivity of the algorithm could be preserved in three random train/test cycles. The point of failure was 40%, 35% and 40% error.

RF Error Tolerance- <50 nM DefGood in MEK1

- 0-50% absolute error

RF- <50nM DefGood in MEK1, 10% error

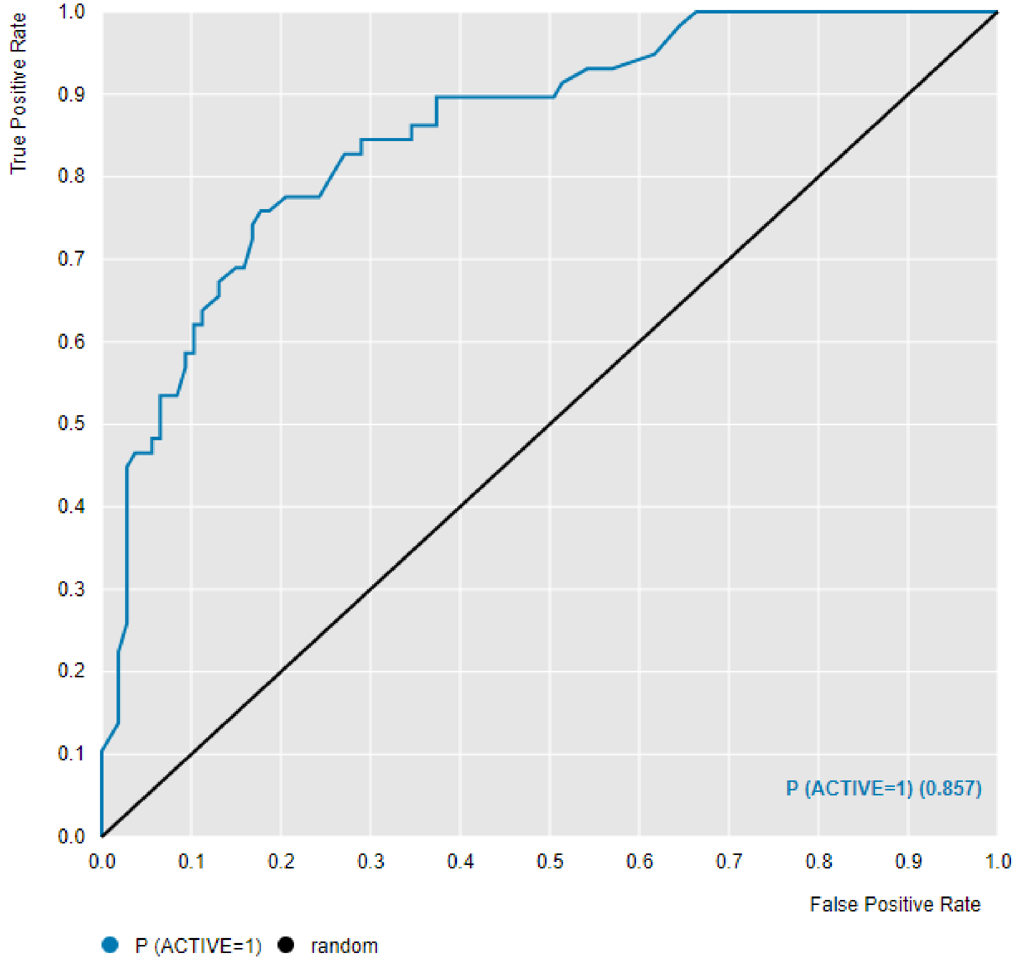

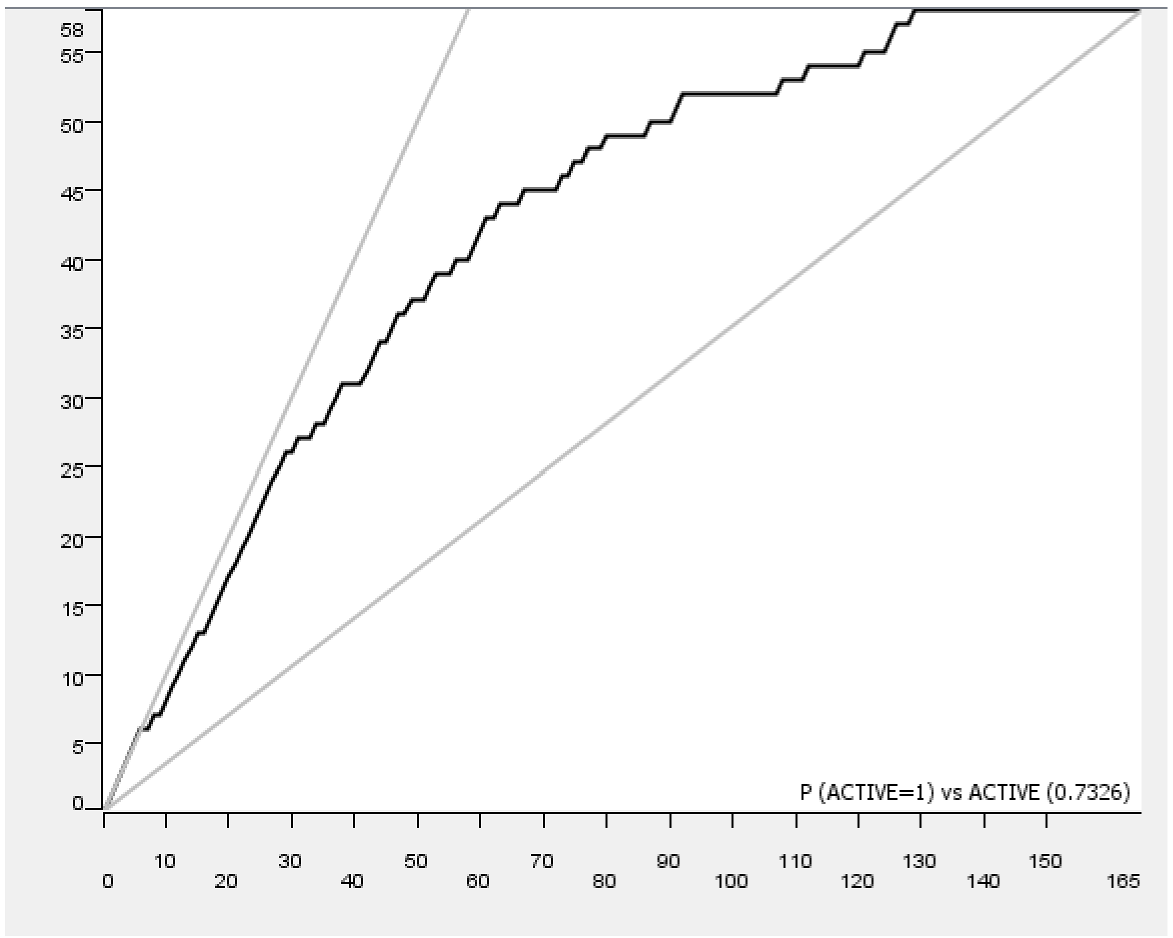

N=165

**Top 10% Mean IC50**	**138nM One micromolar mistake**

	**Predicted Active**	**Predicted Inactive**
Active	40	18
Inactive	16	91

RF- <50nM DefGood in MEK1, 20% error

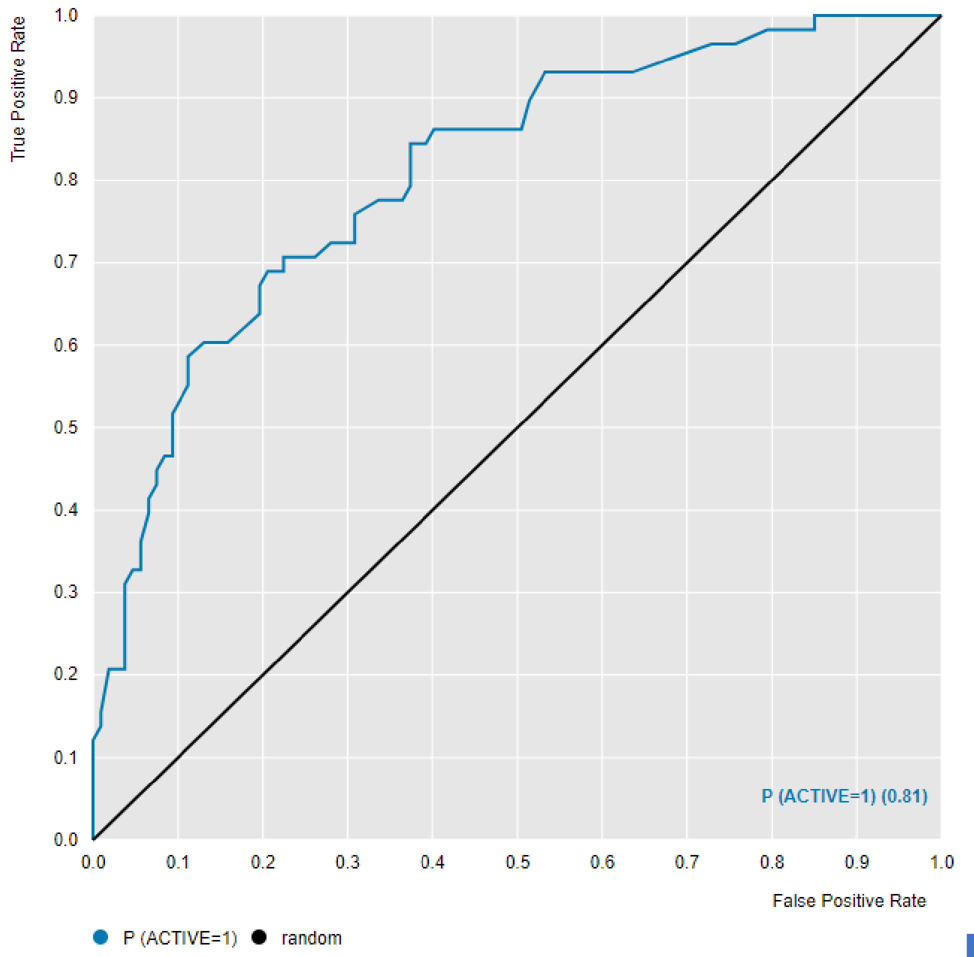

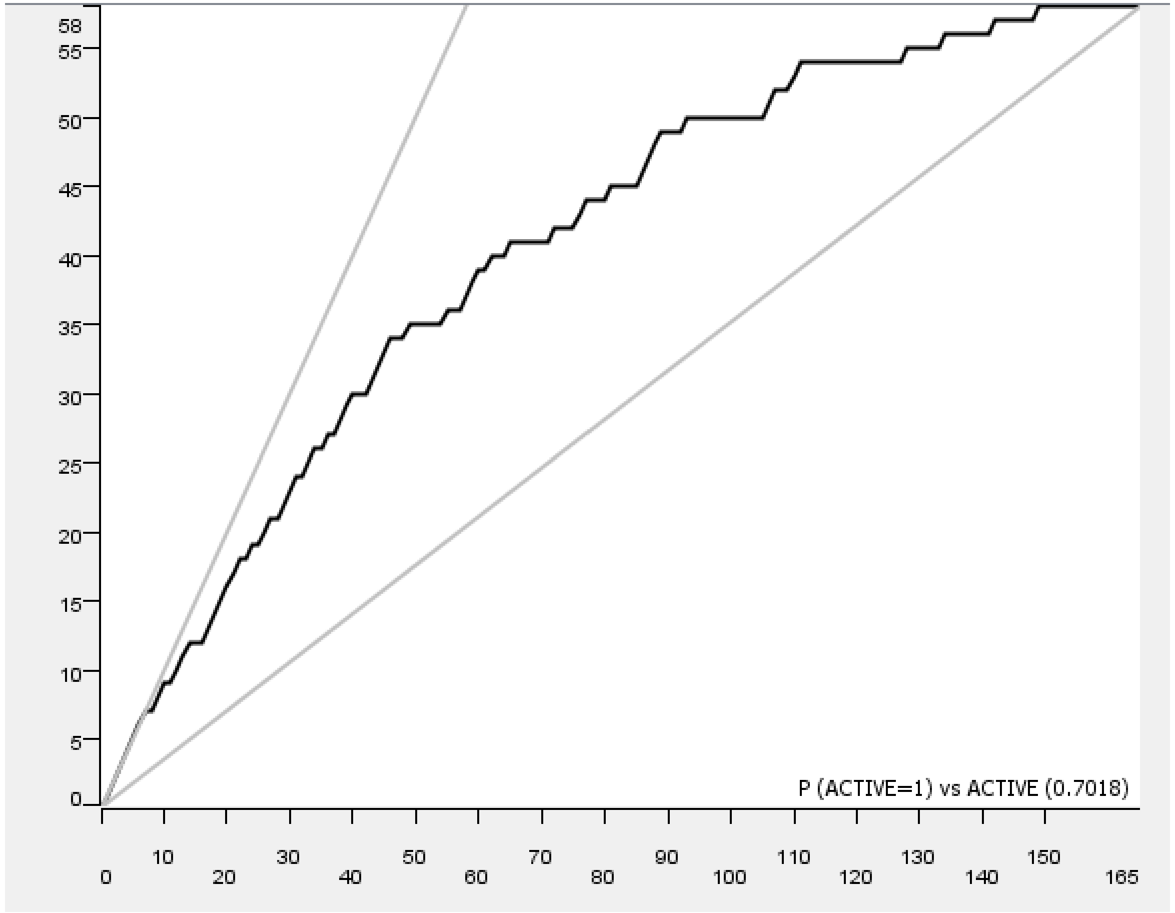

N=165

Top 10% Mean IC50	148 nM

	Predicted Active	Predicted Inactive
Active	39	19
Inactive	21	86

RF- <50nM DefGood in MEK1, 25% error

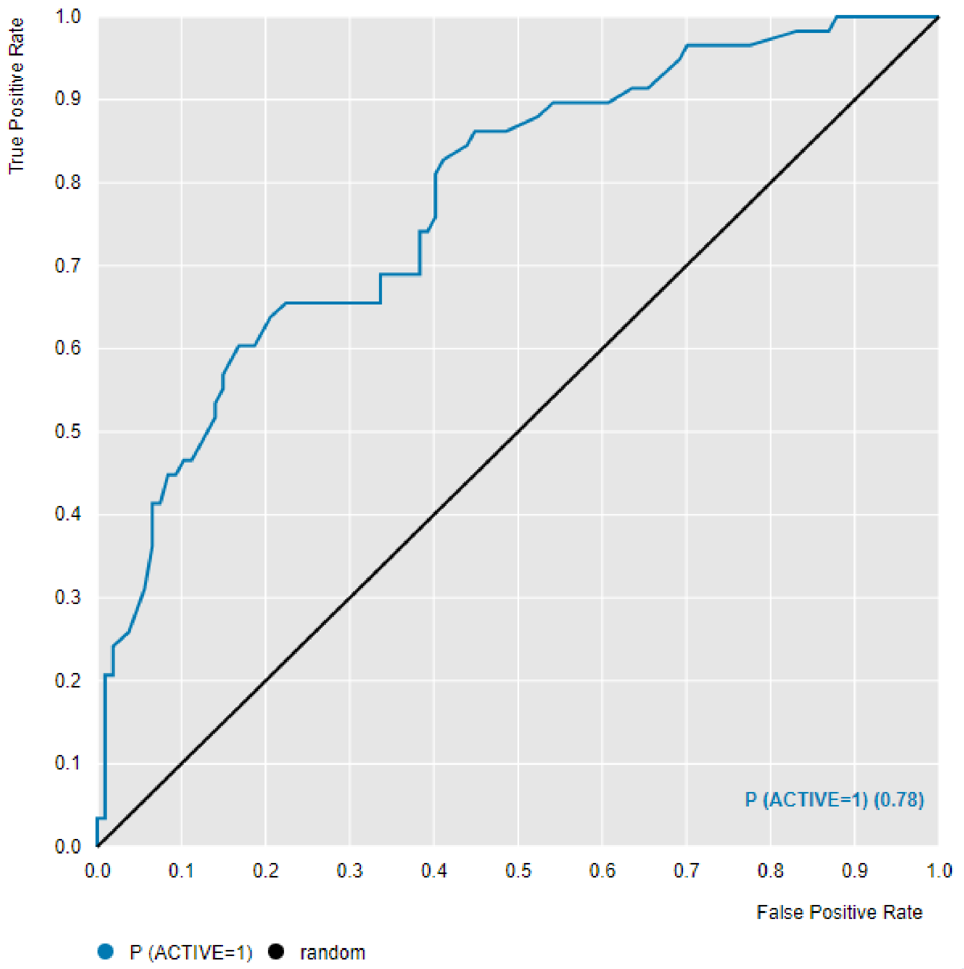

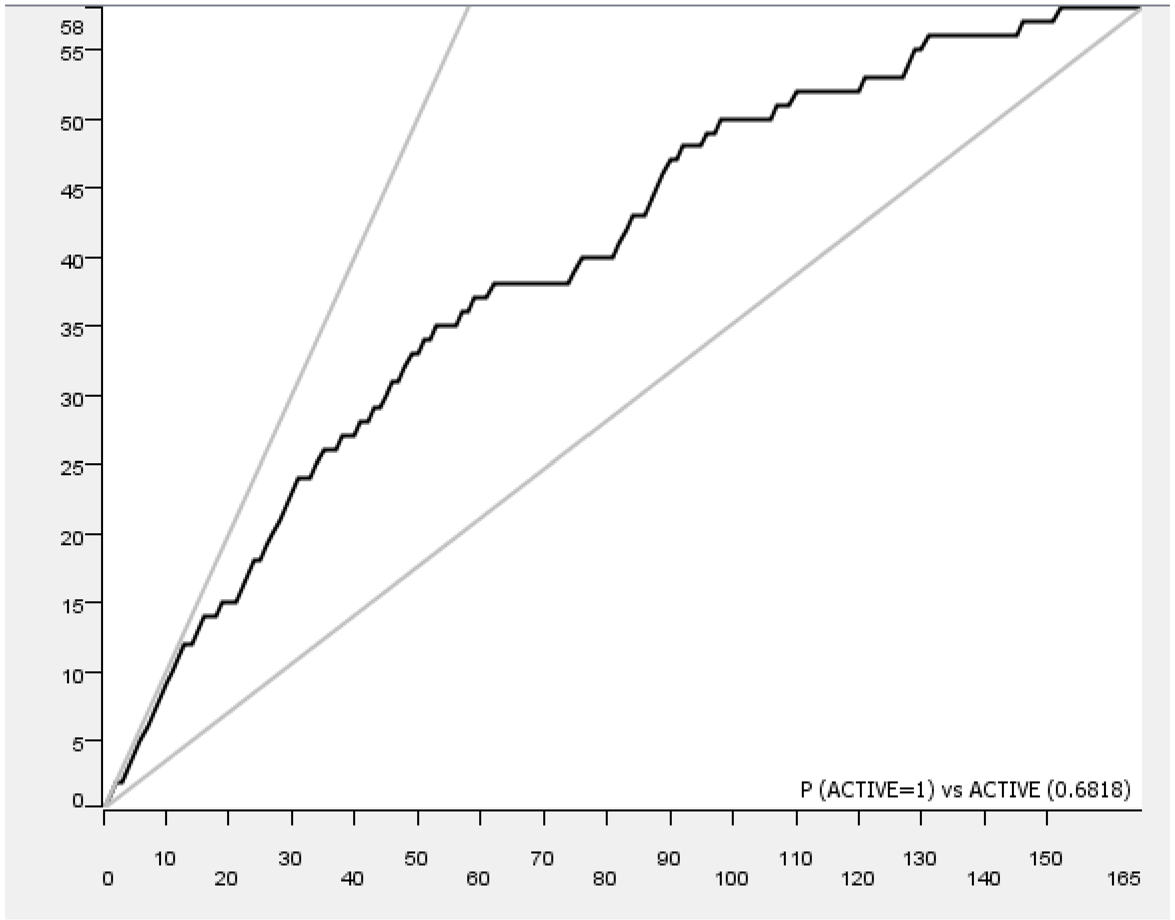

N=165

**Top 10% Mean IC50**	**76.9 nM**

	**Predicted Active**	**Predicted Inactive**
Active	36	22
Inactive	21	86

RF- <50nM DefGood in MEK1, 30% error

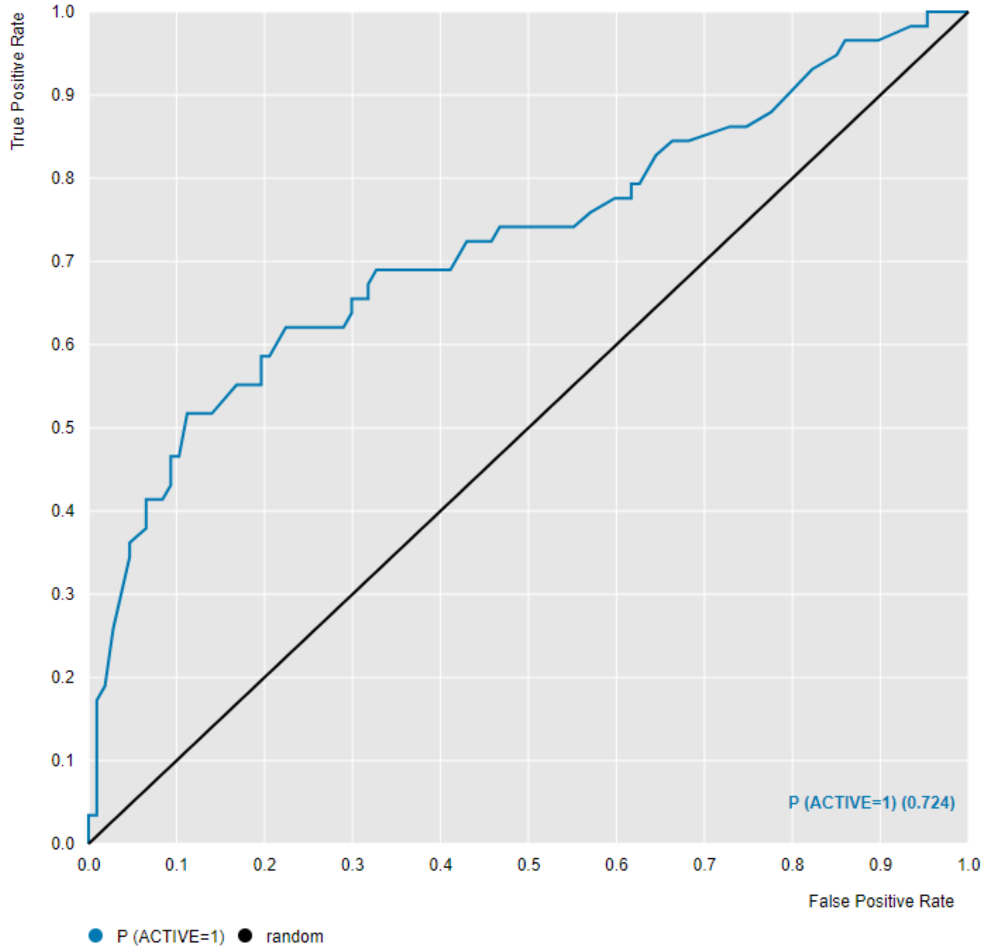

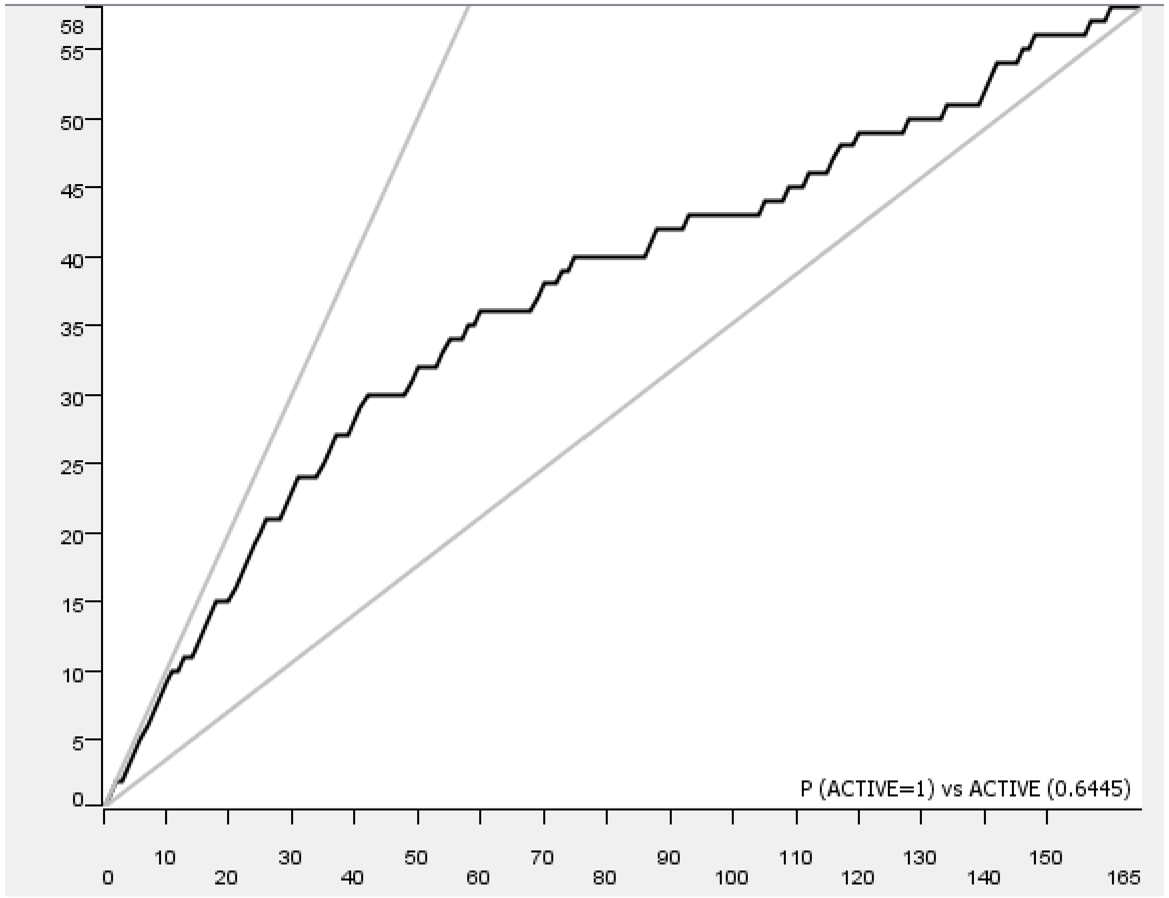

N=165

**Top 10% Mean IC50**	**1,200 nM**

	**Predicted Active**	**Predicted Inactive**
Active	36	22
Inactive	26	81

<50nM DefGood in MEK1, 10% error; Random seed = 429

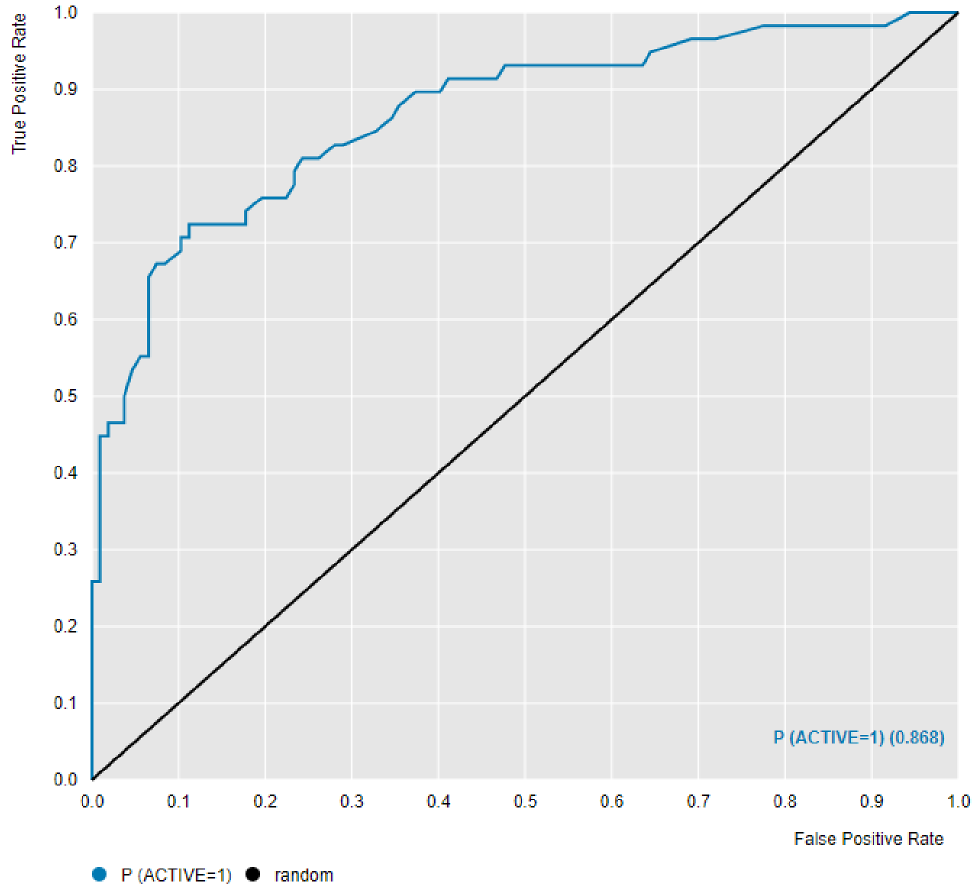

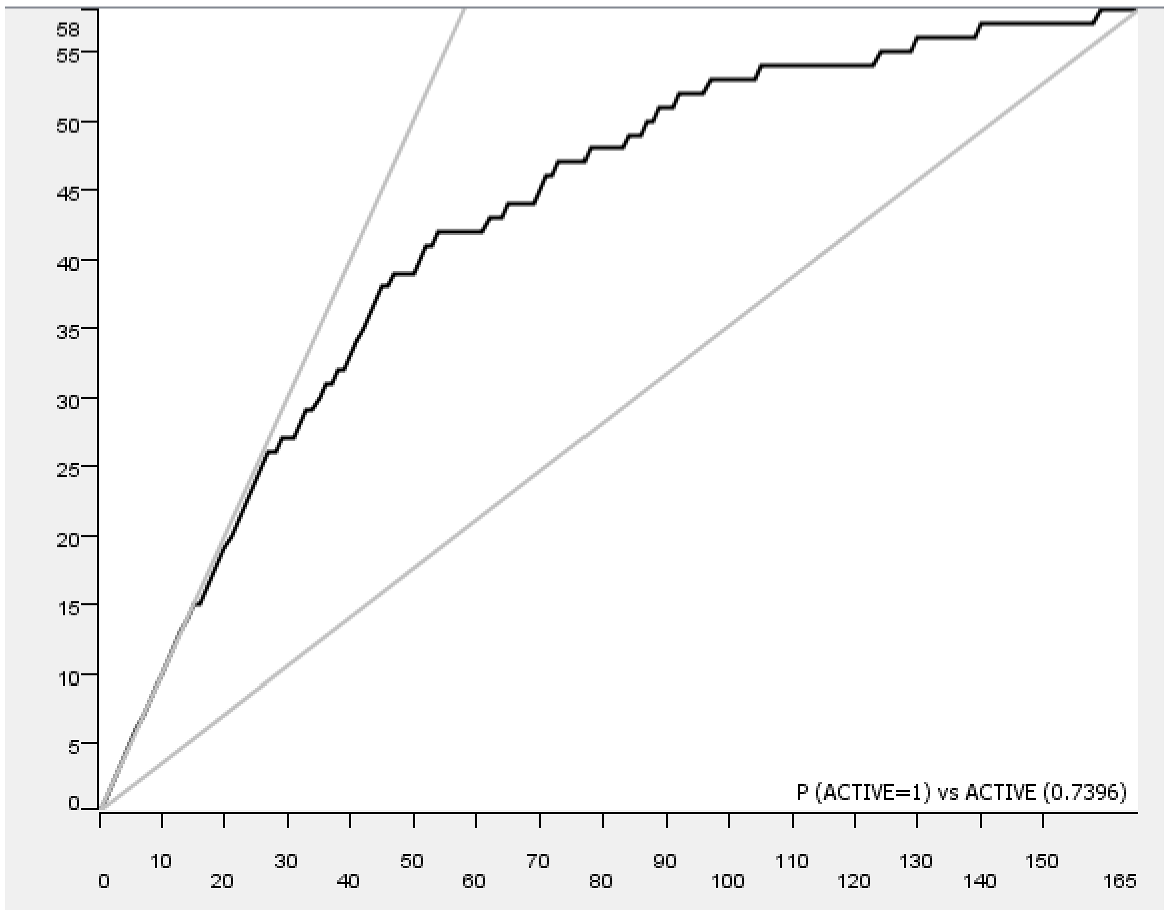

N=165

Top 10% Mean IC50	38.5 nM

	Predicted Active	Predicted Inactive
Active	40	18
Inactive	11	96

<50nM DefGood in MEK1, 20% error; Random seed = 429

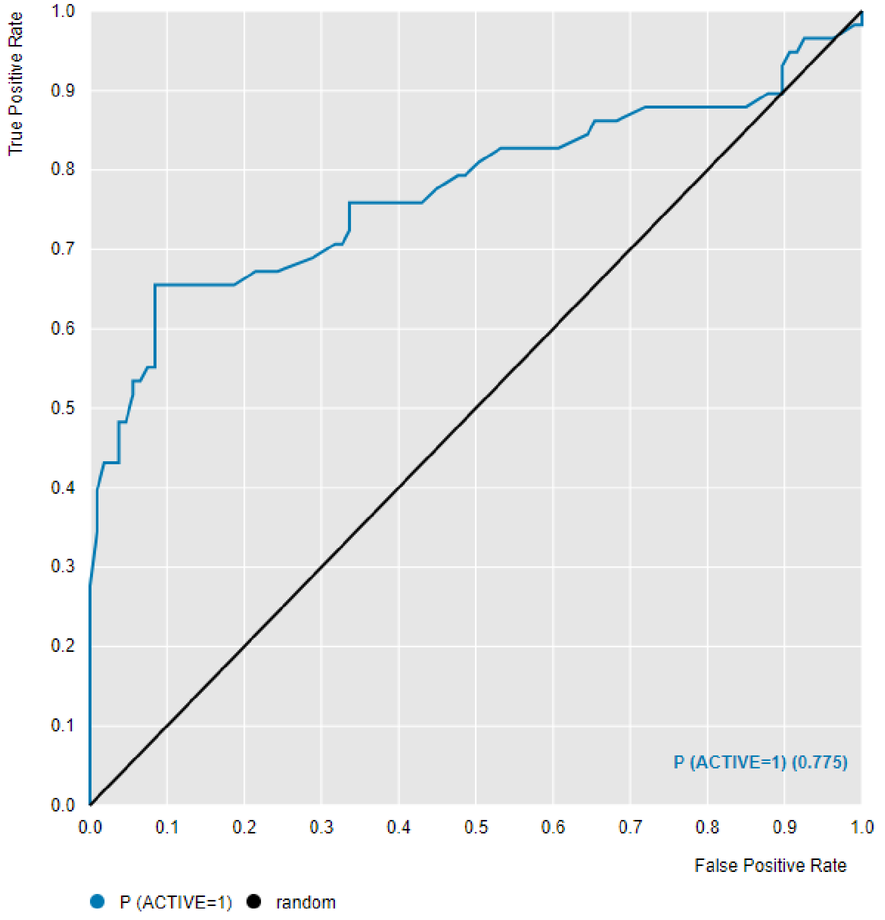

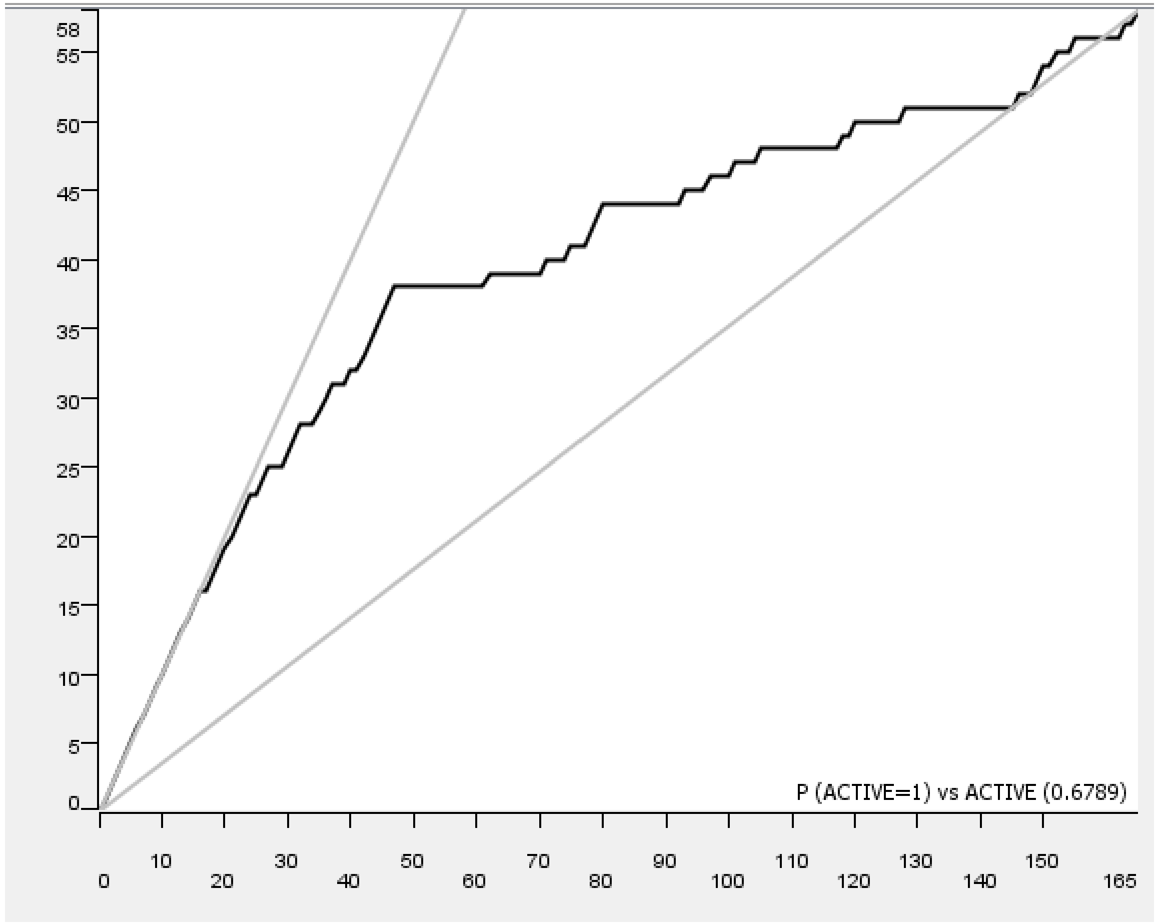

N=165

Top 10% Mean IC50	20.3 nM

	Predicted Active	Predicted Inactive
Active	38	20
Inactive	14	93

<50nM DefGood in MEK1, 25% error; Random seed = 429

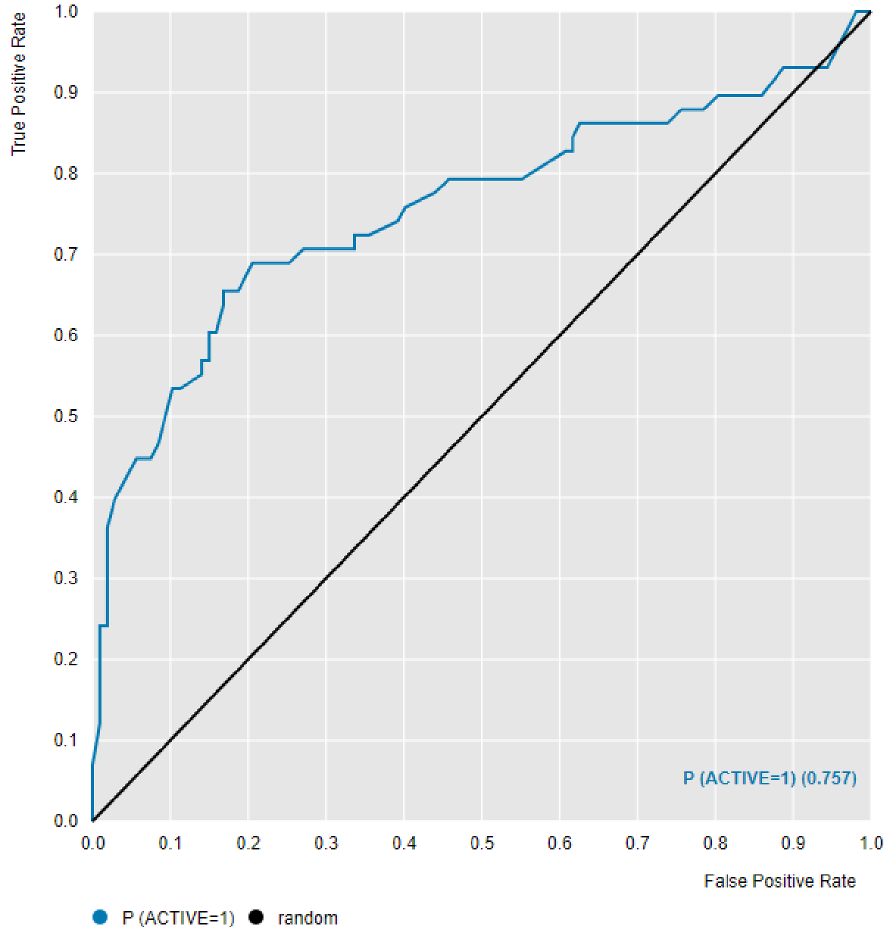

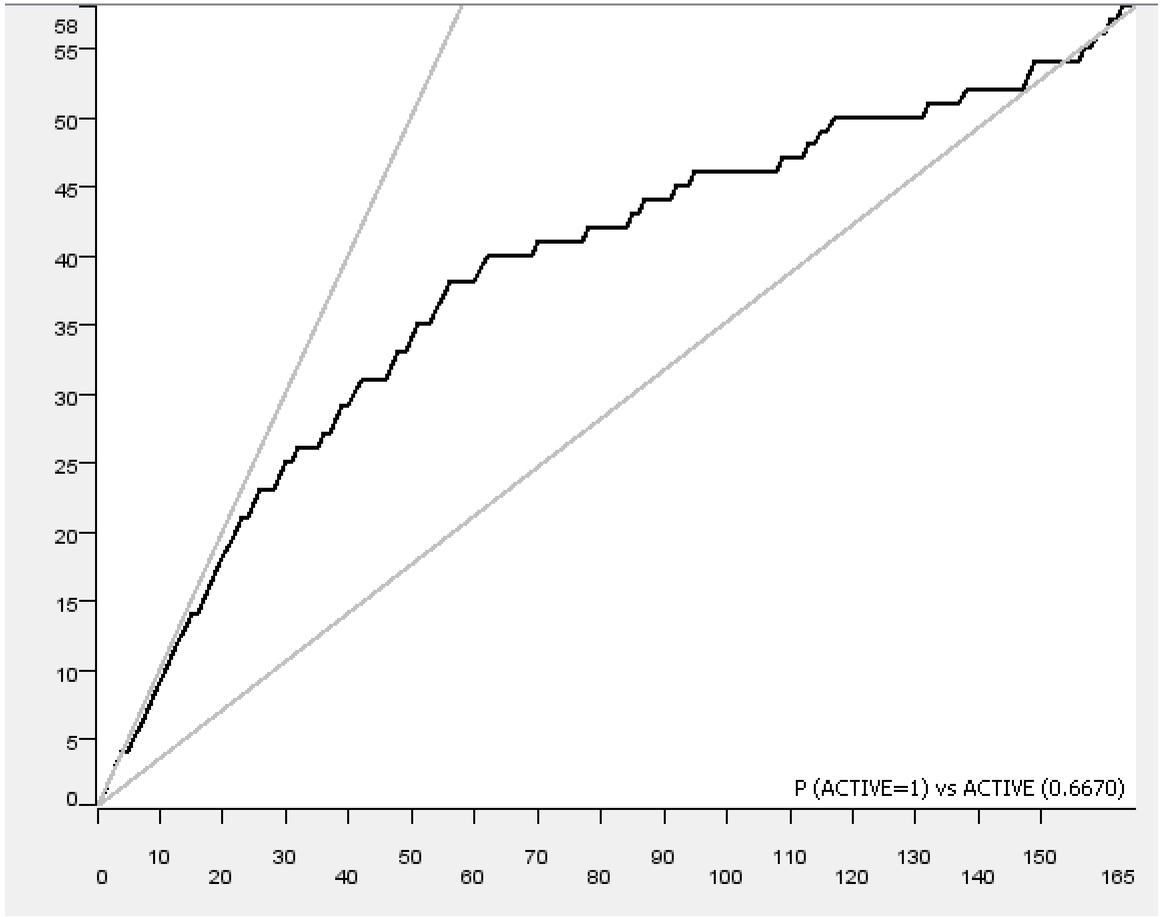

N=165

**Top 10% Mean IC50**	**20.3 nM**

	Predicted Active	Predicted Inactive
Active	38	20
Inactive	20	87

<50nM DefGood in MEK1, 30% error; Random seed = 429

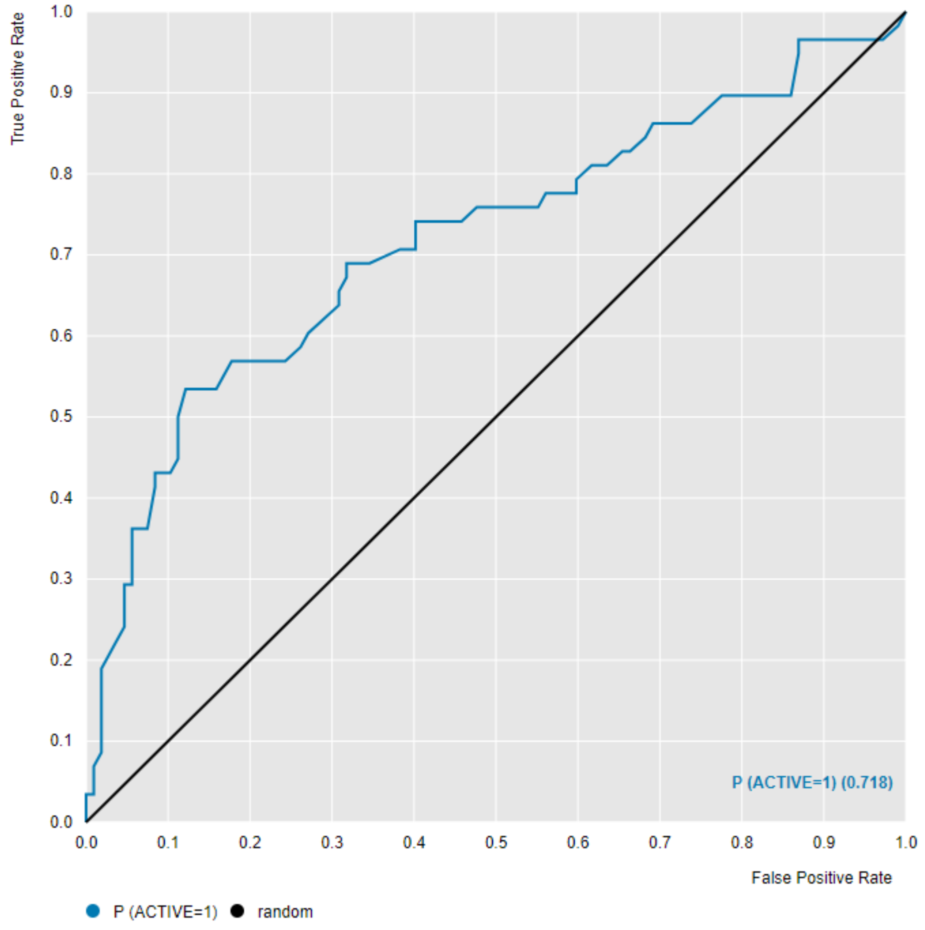

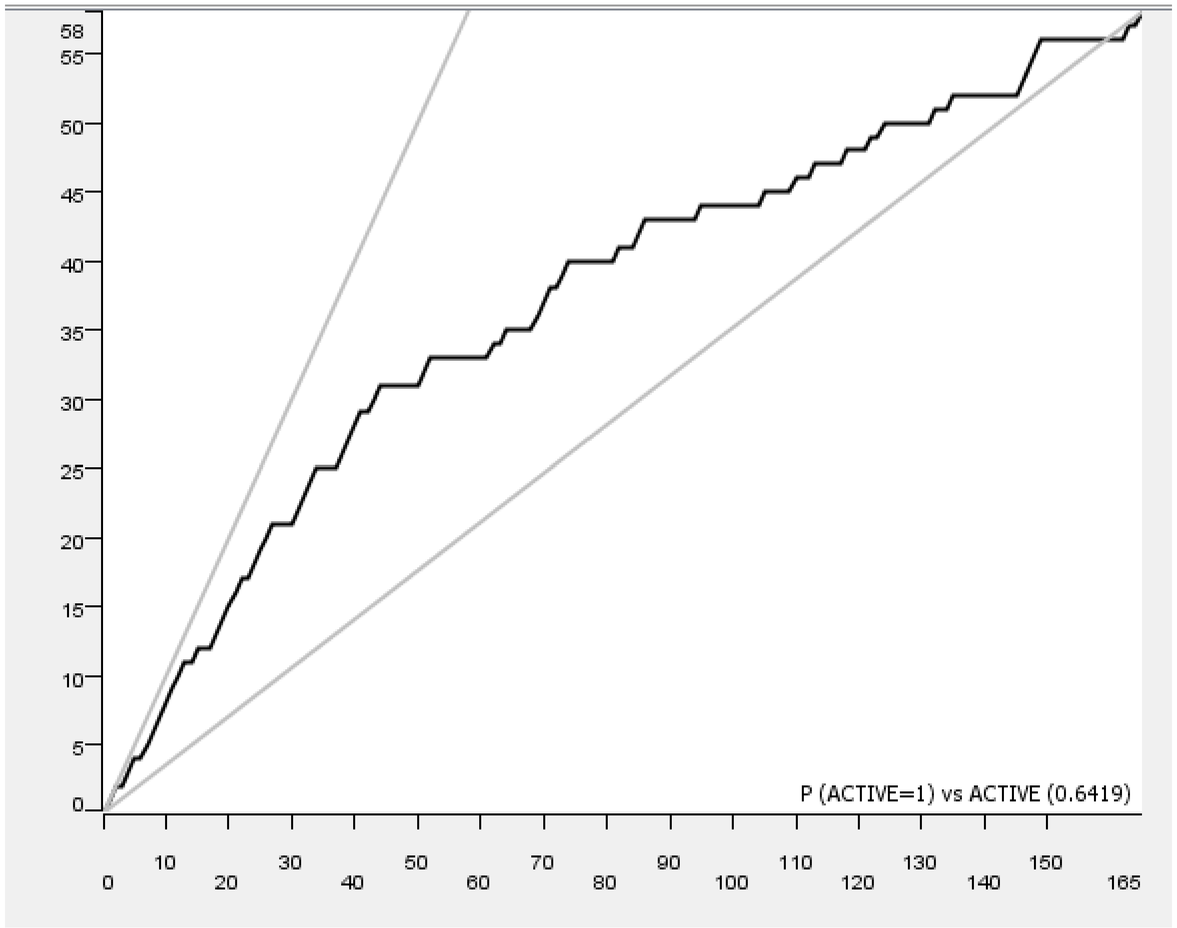

N=165

Top 10% Mean IC50	689 nM

	Predicted Active	Predicted Inactive
Active	39	19
Inactive	34	73

<50nM DefGood in MEK1, 35% error; Random seed = 429

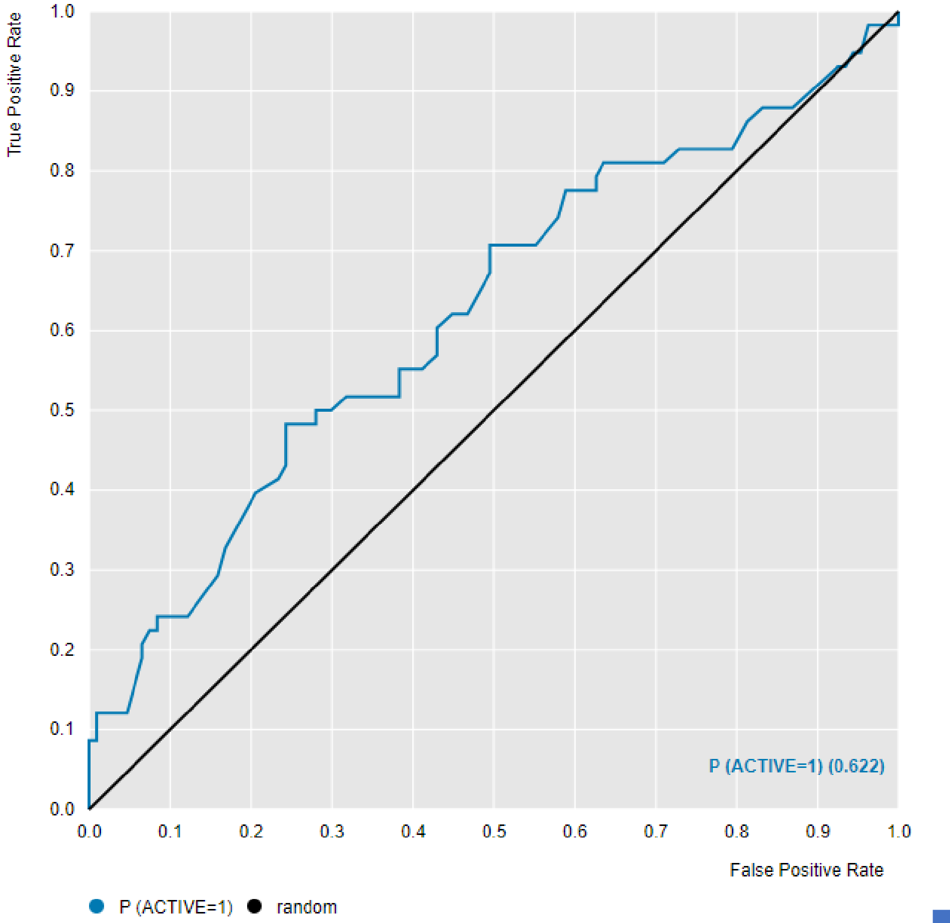

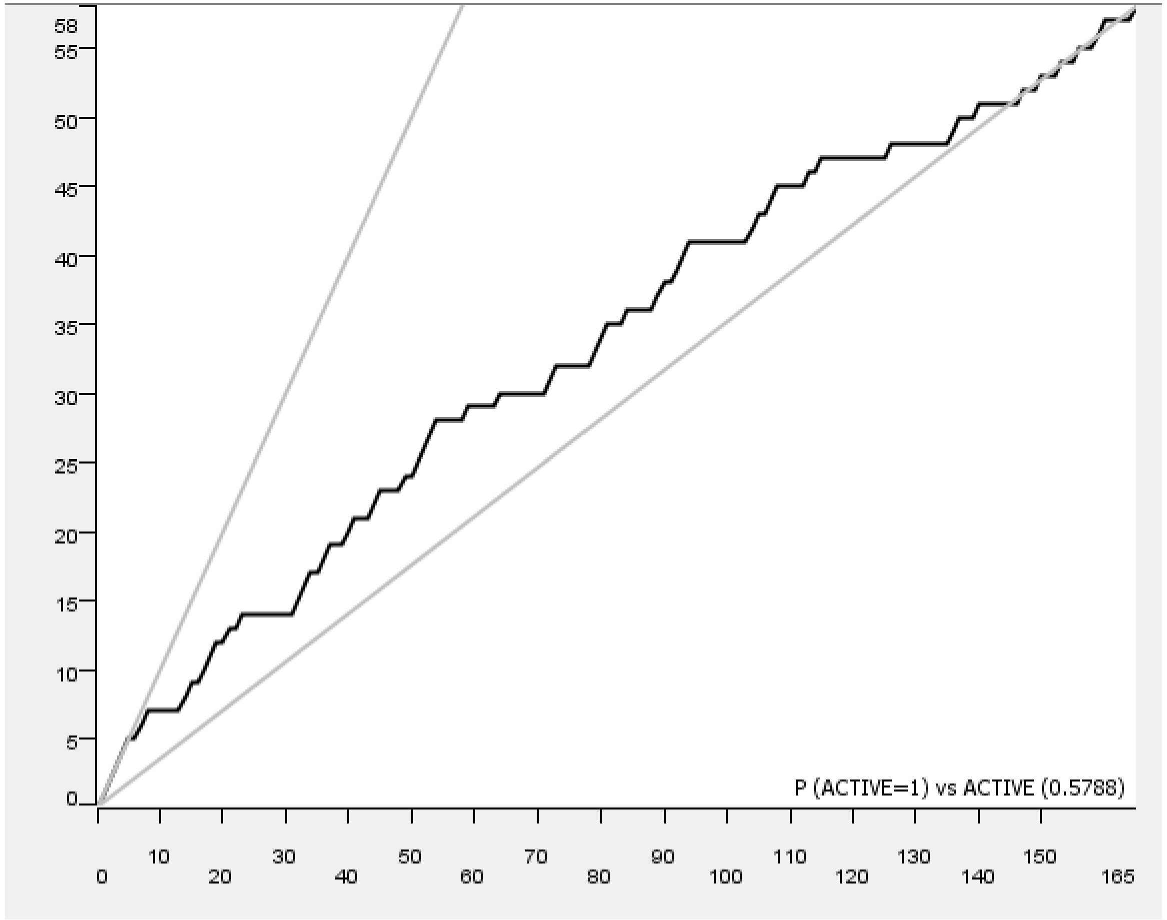

N=165

**Top 10% Mean IC50**	**832 nM**

	Predicted Active	Predicted Inactive
Active	32	26
Inactive	42	65

<50nM DefGood in MEK1, 10% error; Random seed = 121783

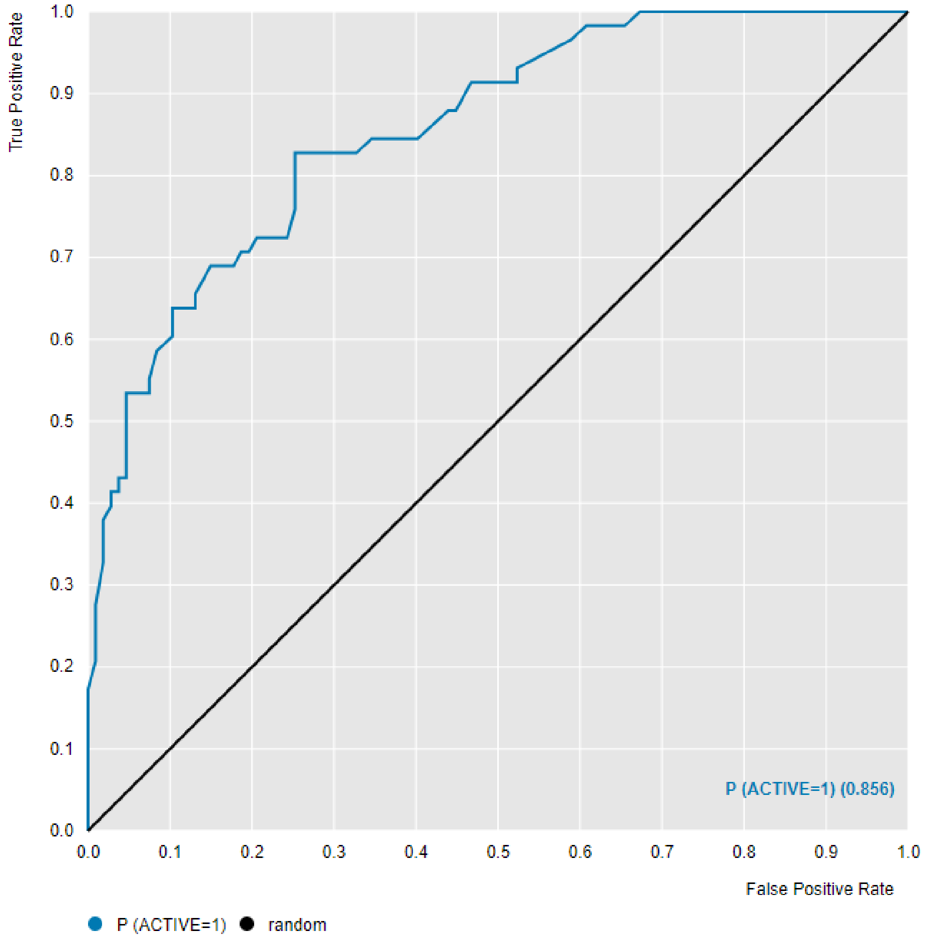

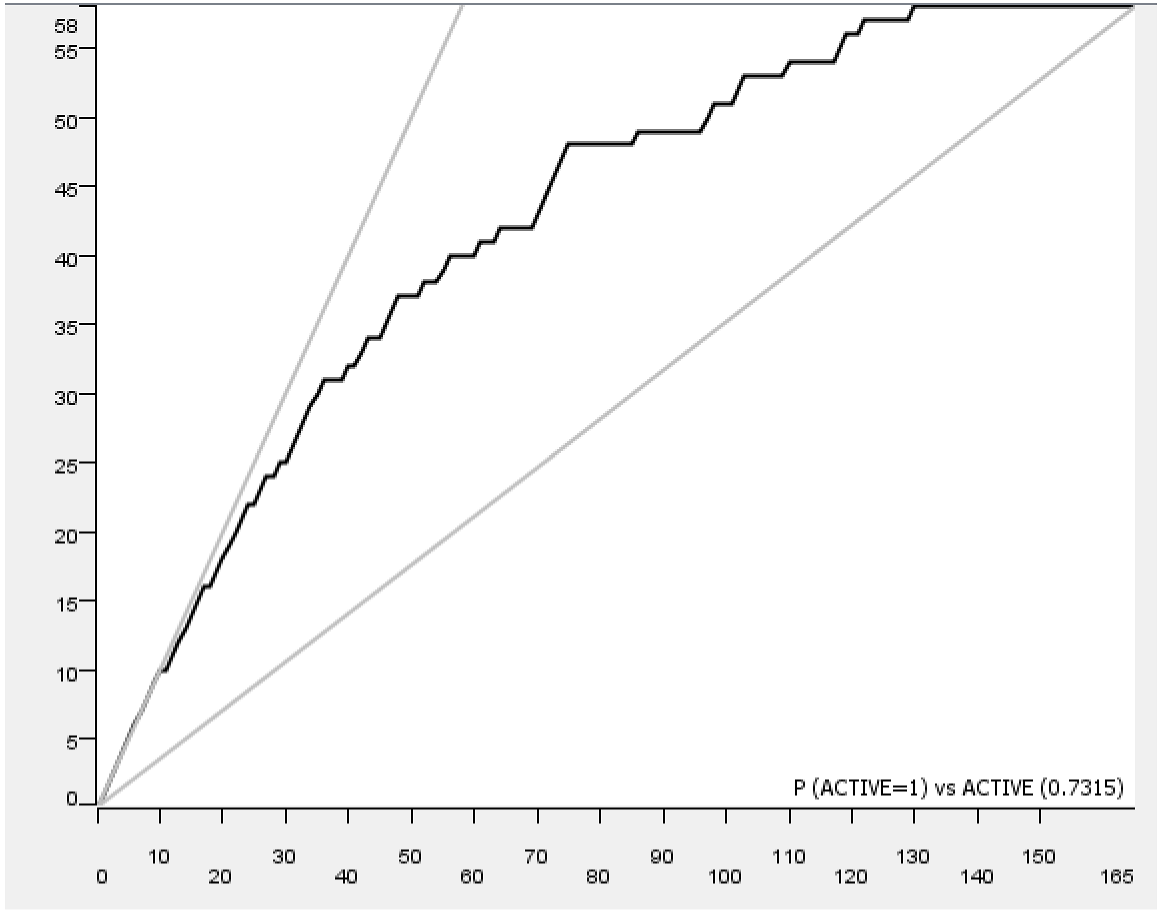

N=165

Top 10% Mean IC50	19.5 nM

	Predicted Active	Predicted Inactive
Active	37	21
Inactive	14	93

<50nM DefGood in MEK1, 20% error; Random seed = 121783

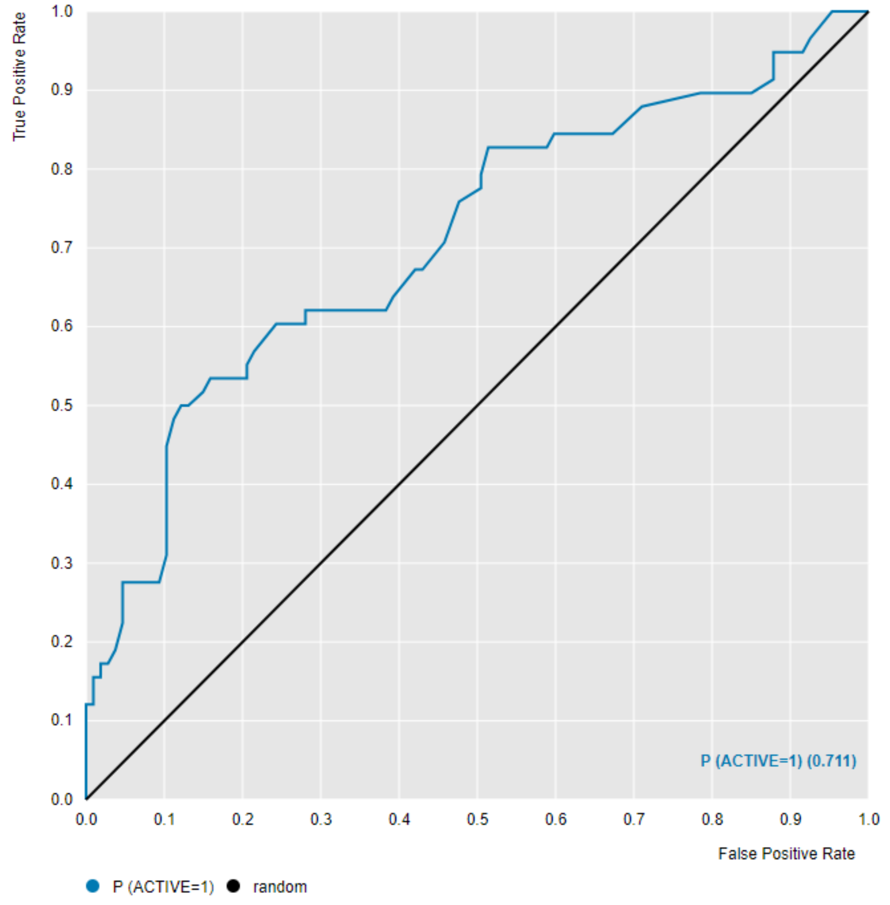

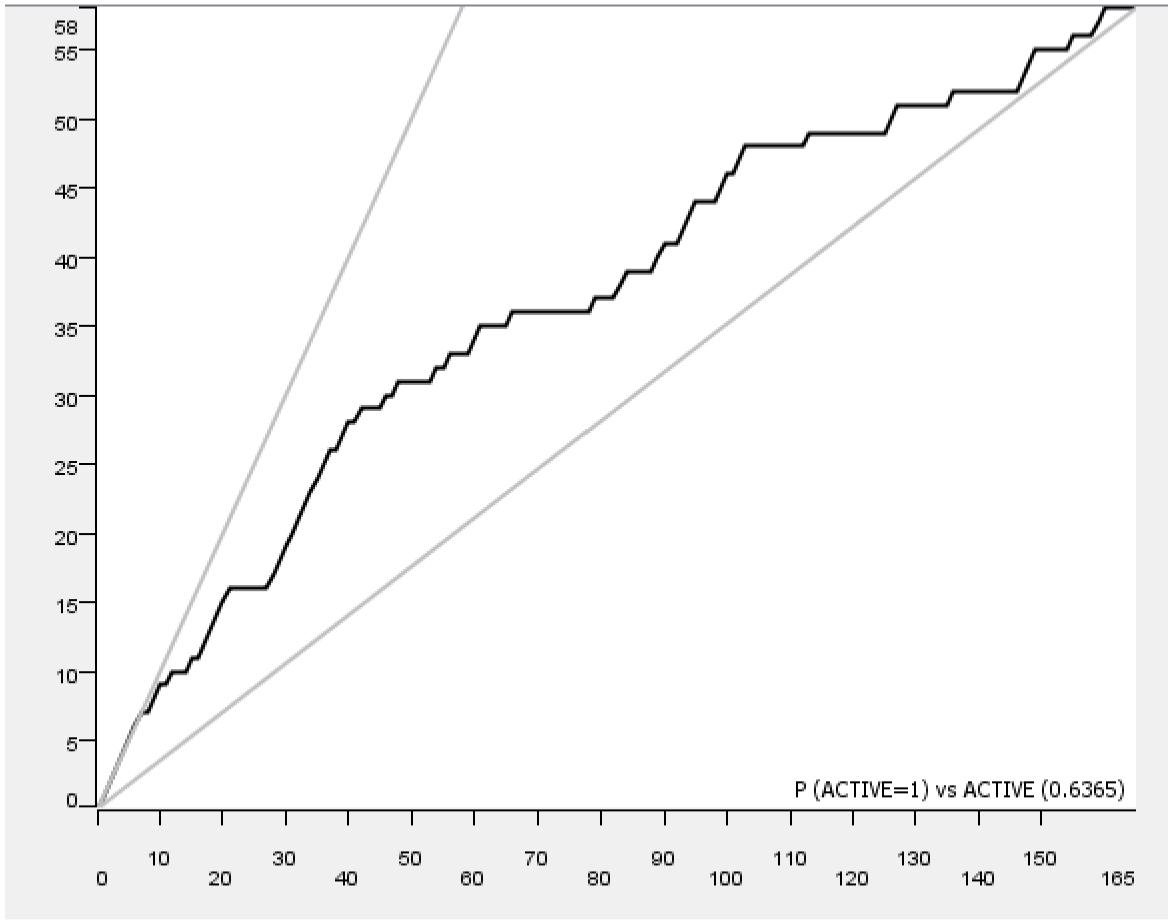

N=165

Top 10% Mean IC50	56.3 nM

	Predicted Active	Predicted Inactive
Active	31	27
Inactive	20	87

<50nM DefGood in MEK1, 25% error; Random seed = 121783

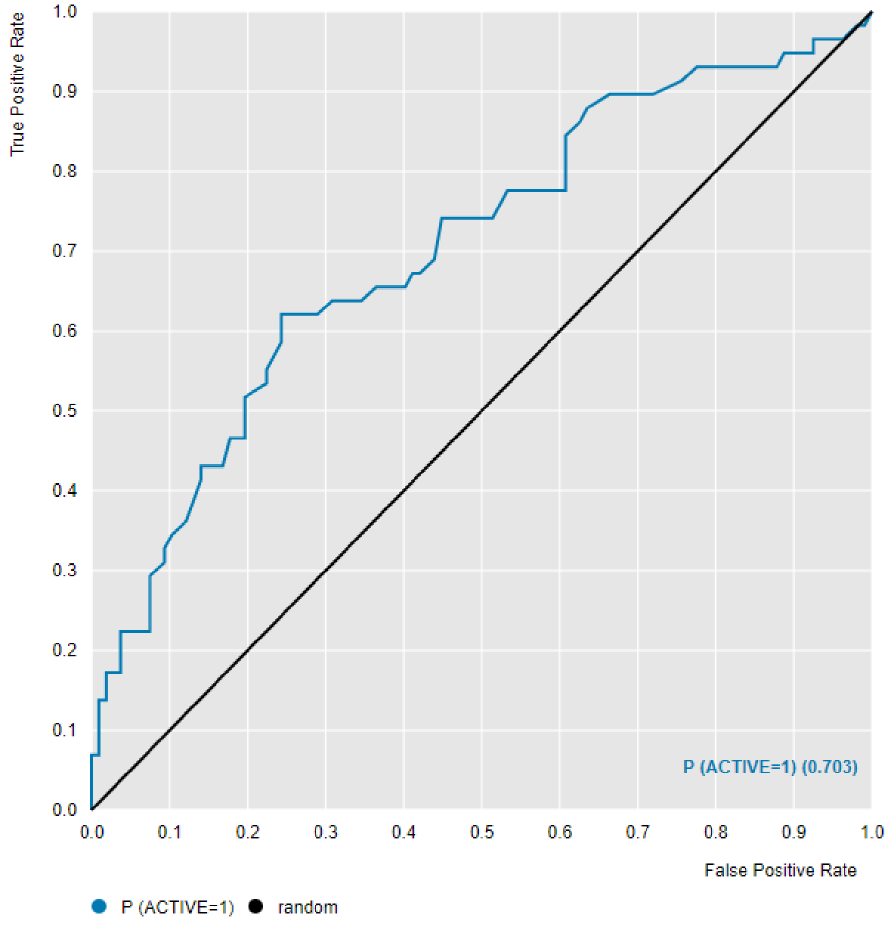

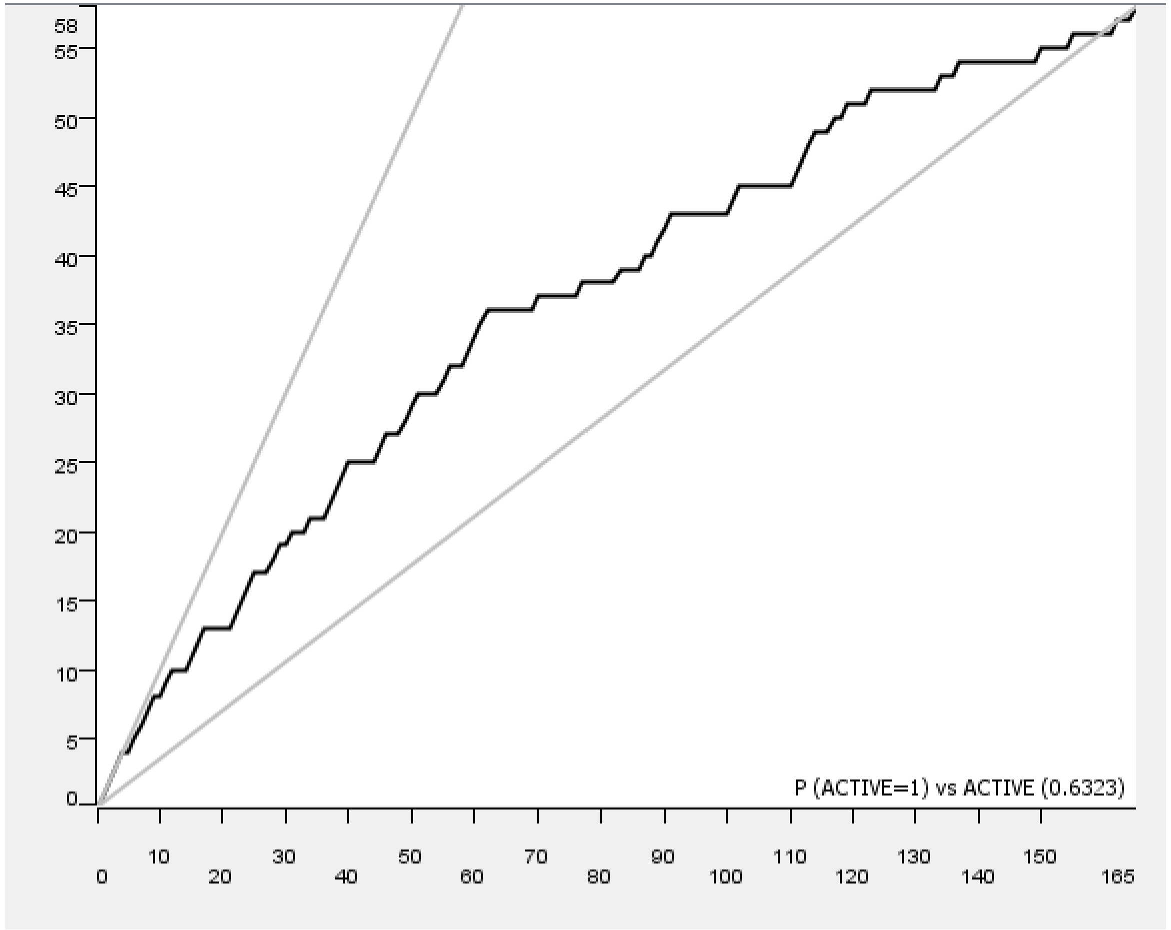

N=165

**Top 10% Mean IC50**	**6,900 nM**

	**Predicted Active**	**Predicted Inactive**
Active	34	24
Inactive	26	81

Conclusion - RF

- A Random Forrest could be generated for MEK1 kinase with a decision value of <50 nM as defined as active. The predictivity of the algorithm could be preserved in three random train/test cycles as error was introduced in the training set. The point of failure was 30%, 35% and 25% error.

PNN- <50nM DefGood in MEK1, 5% error ; Random seed = 1515533876005

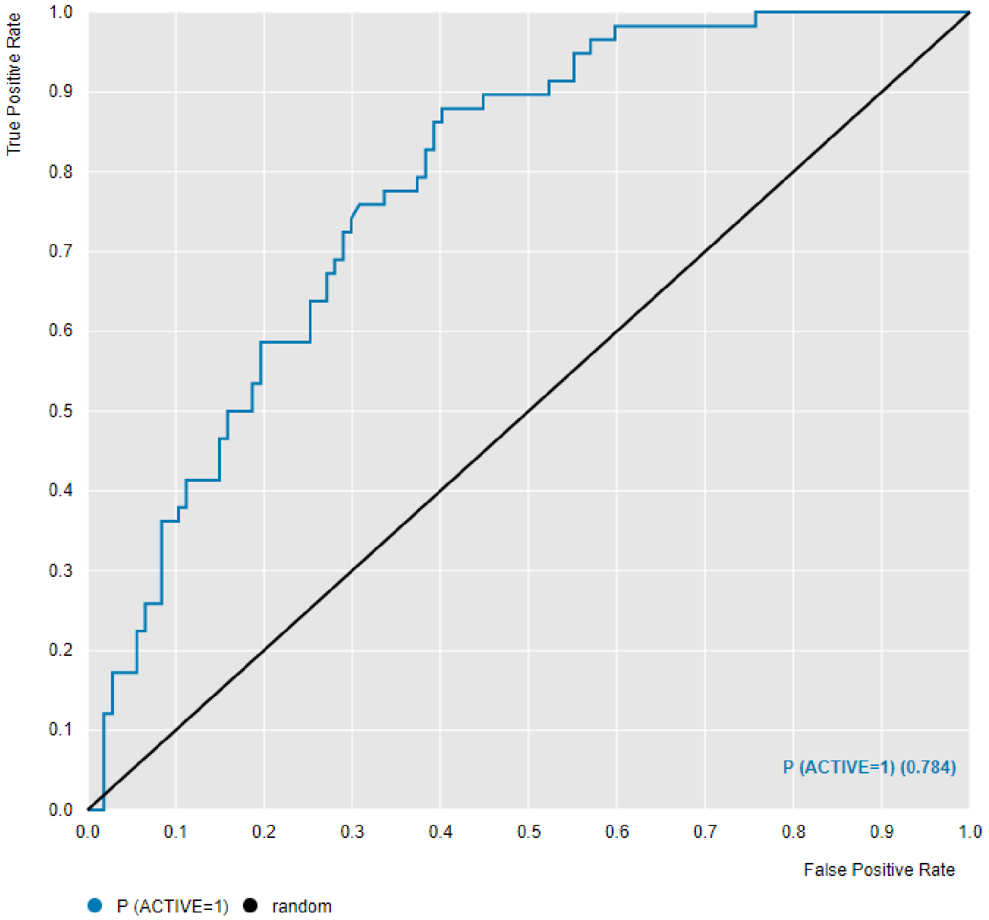

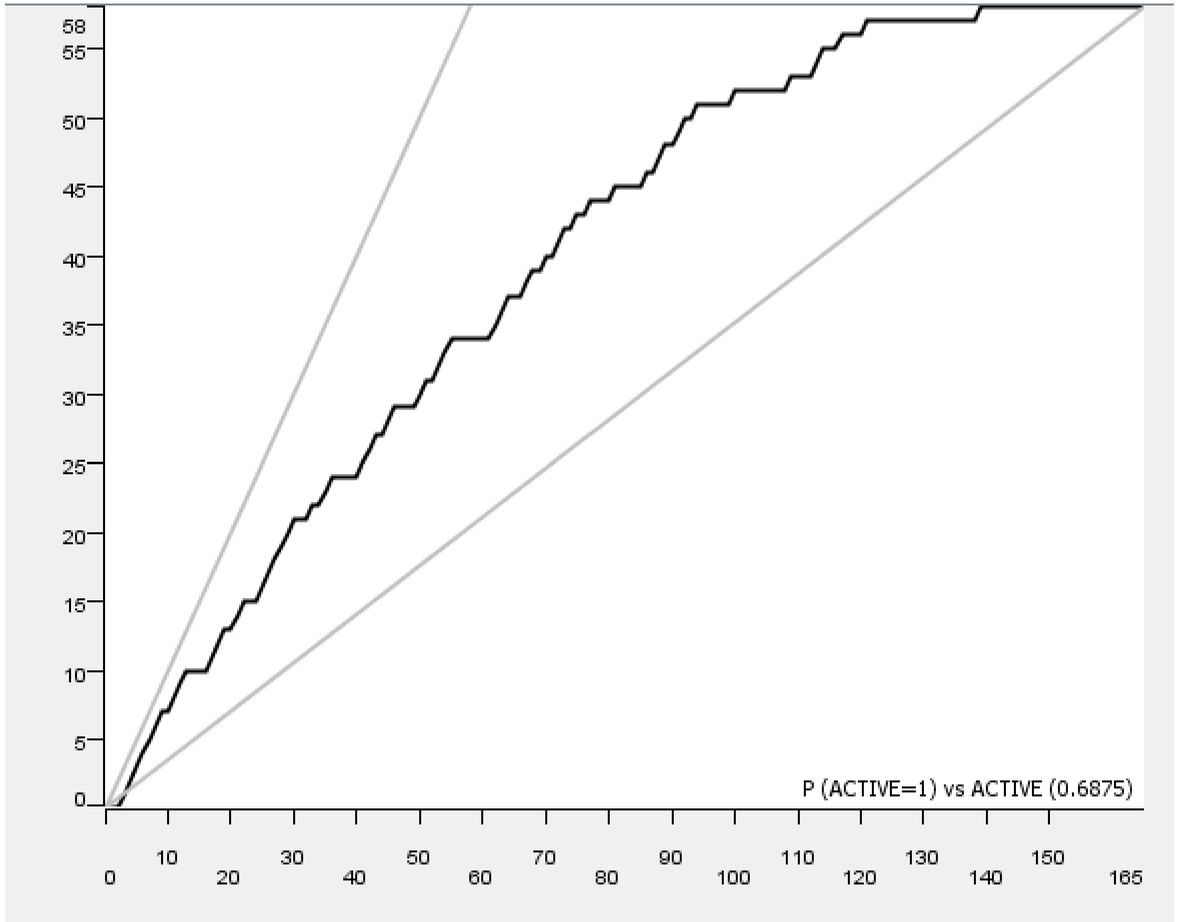

N=165

Top 10% Mean IC50	778 nM<br>Several Micromolar mistakes

	Predicted Active	Predicted Inactive
Active	34	24
Inactive	22	85

PNN- <50nM DefGood in MEK1, 5% error ; Random seed = 429

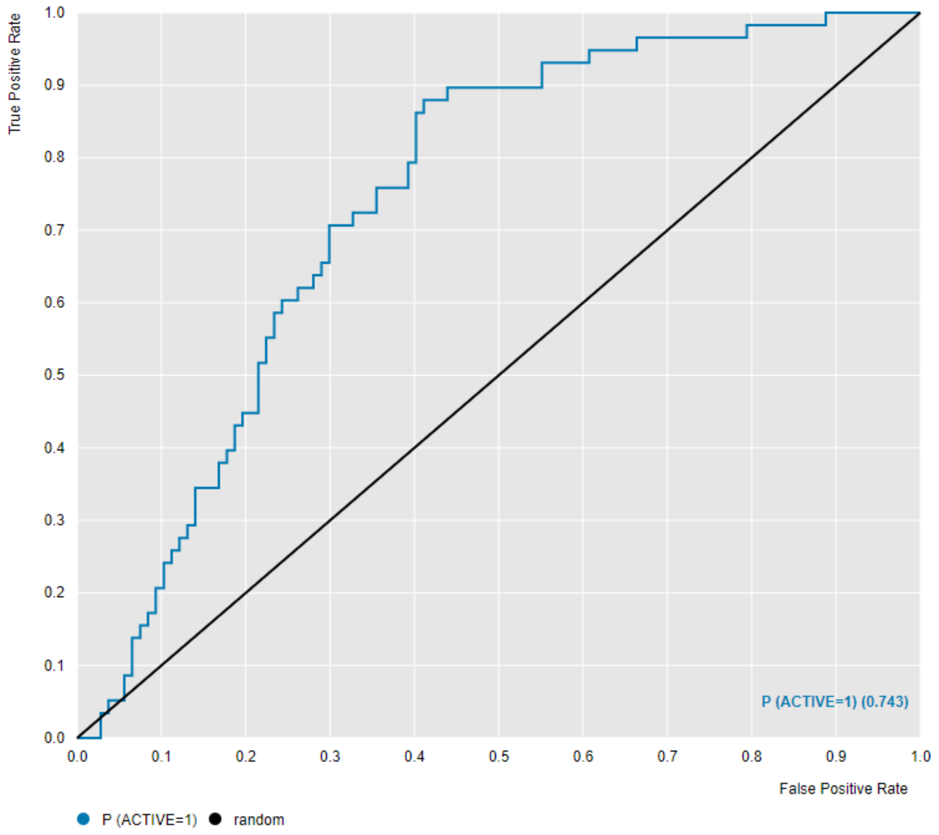

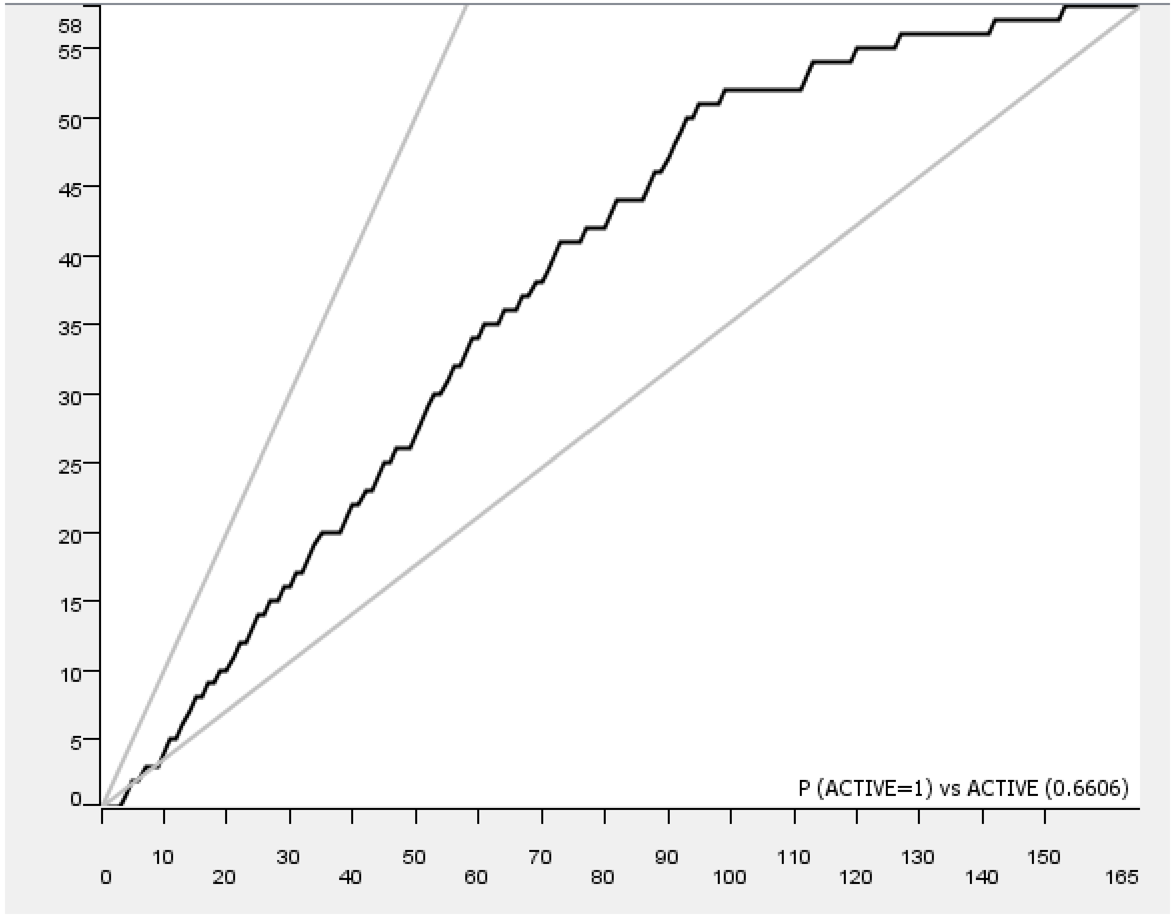

N=165

Top 10% Mean IC50	1,700 nM

	Predicted Active	Predicted Inactive
Active	29	29
Inactive	23	84

PNN - <50nM DefGood in MEK1, 5% error; Random seed = 429 (reparameterized Theta minus = 0.15; theta plus = 0.85)

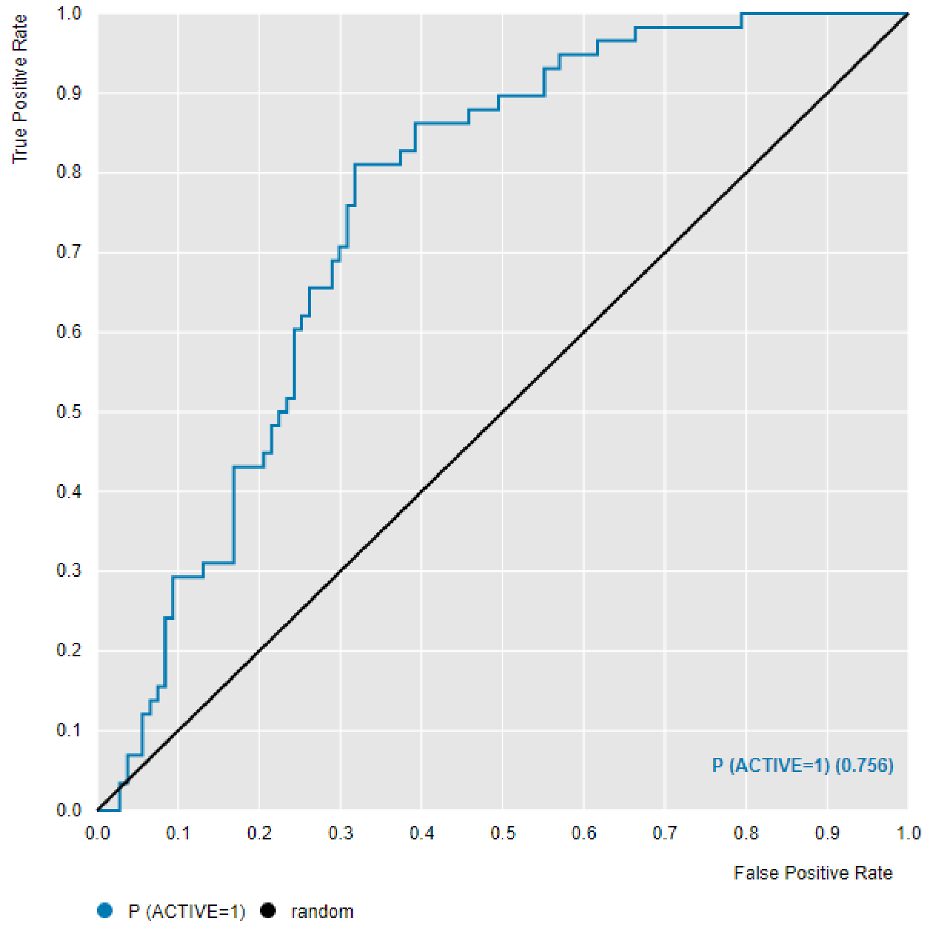

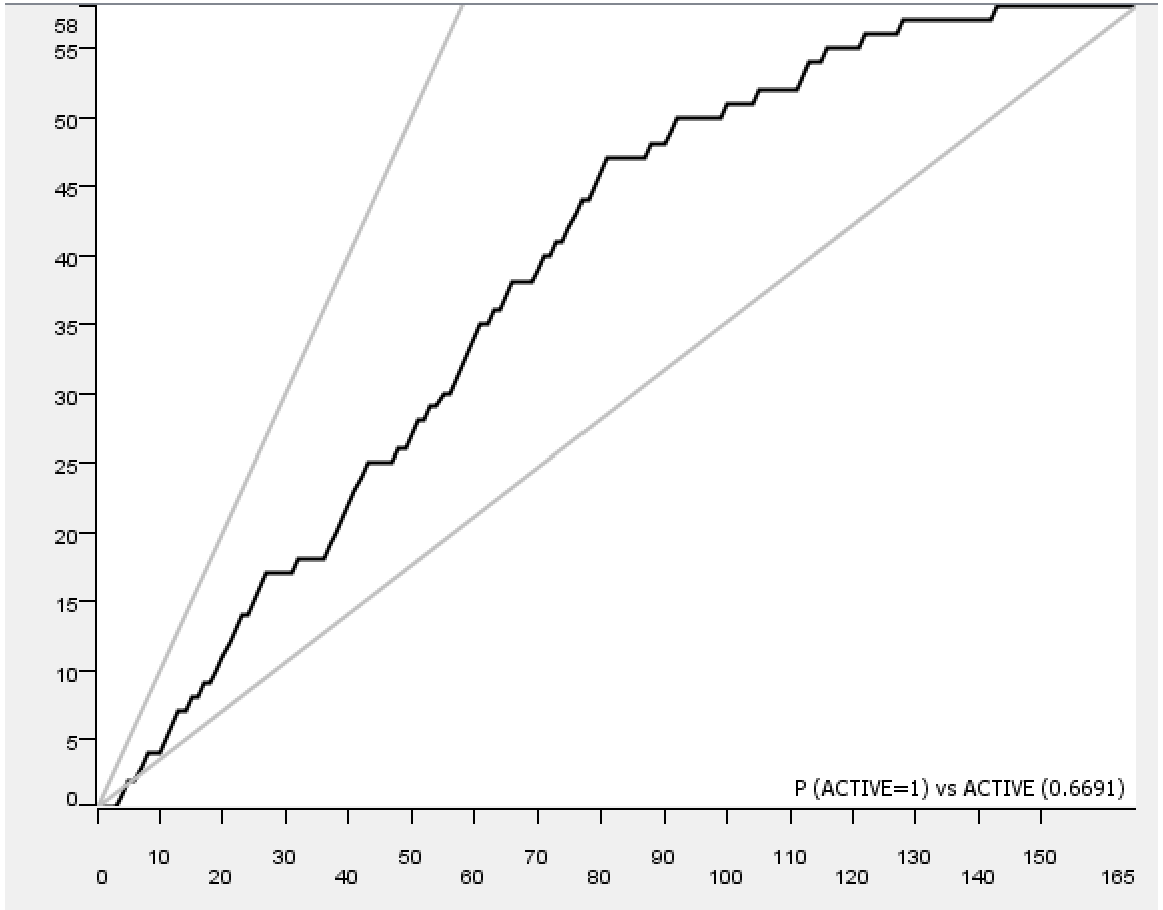

N=165

Top 10% Mean IC50	1,700 nM

	Predicted Active	Predicted Inactive
Active	26	32
Inactive	22	85

PNN - <50nM DefGood in MEK1, 5% error; Random seed = 121783

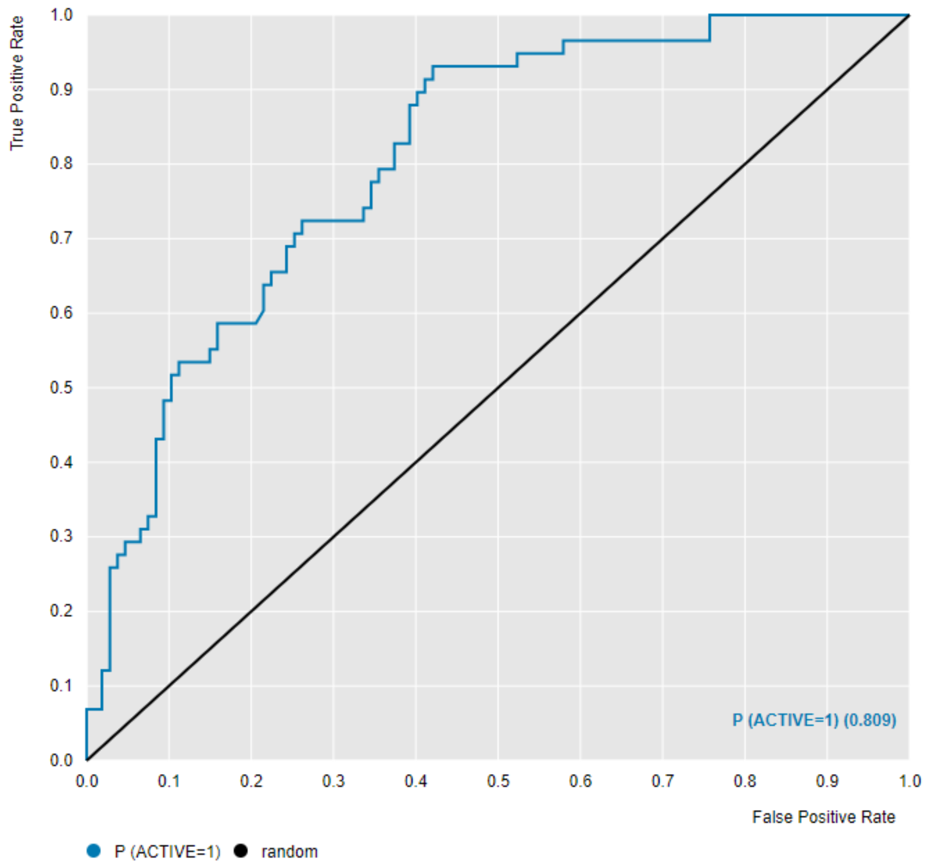

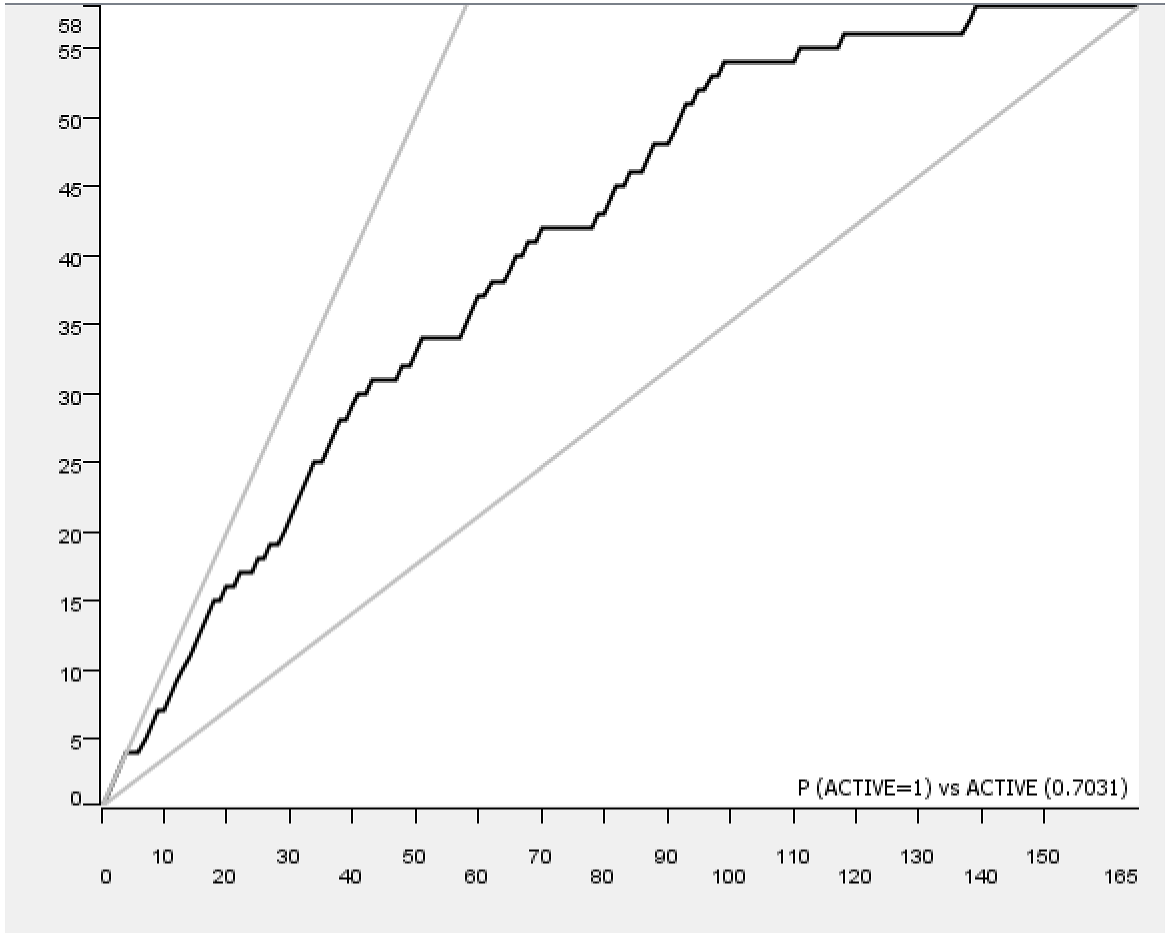

N=165

Top 10% Mean IC50	72.1 nM

	Predicted Active	Predicted Inactive
Active	29	29
Inactive	11	96

PNN - <50nM DefGood in MEK1, 10% error; Random seed = 121783

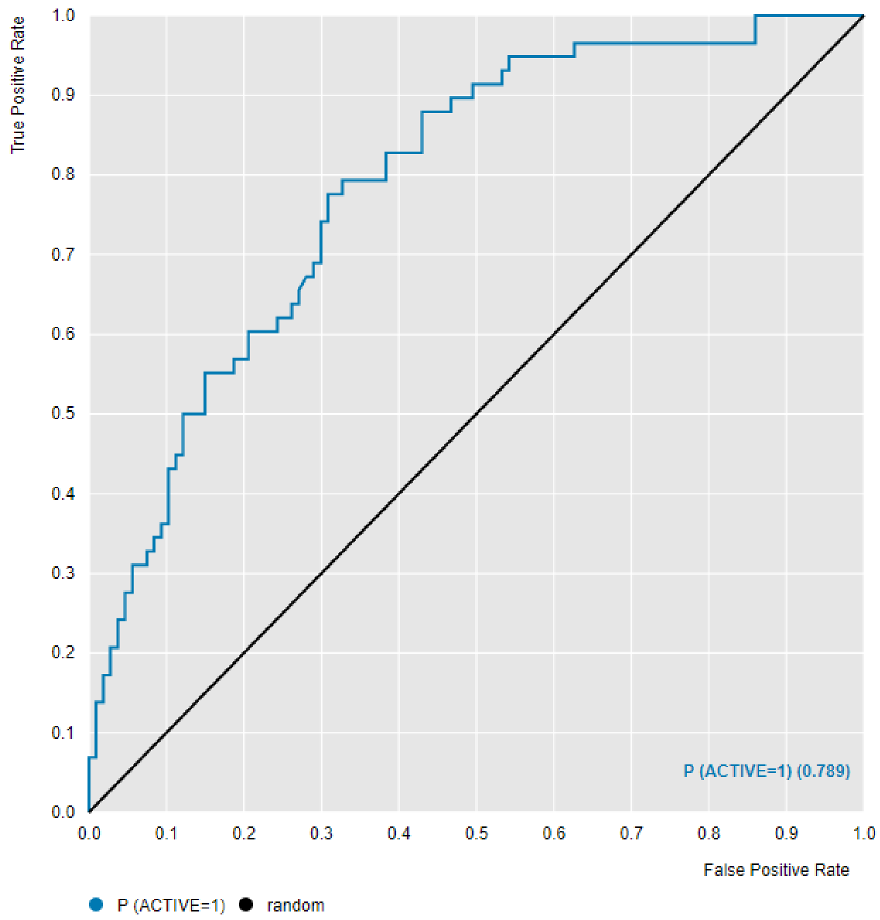

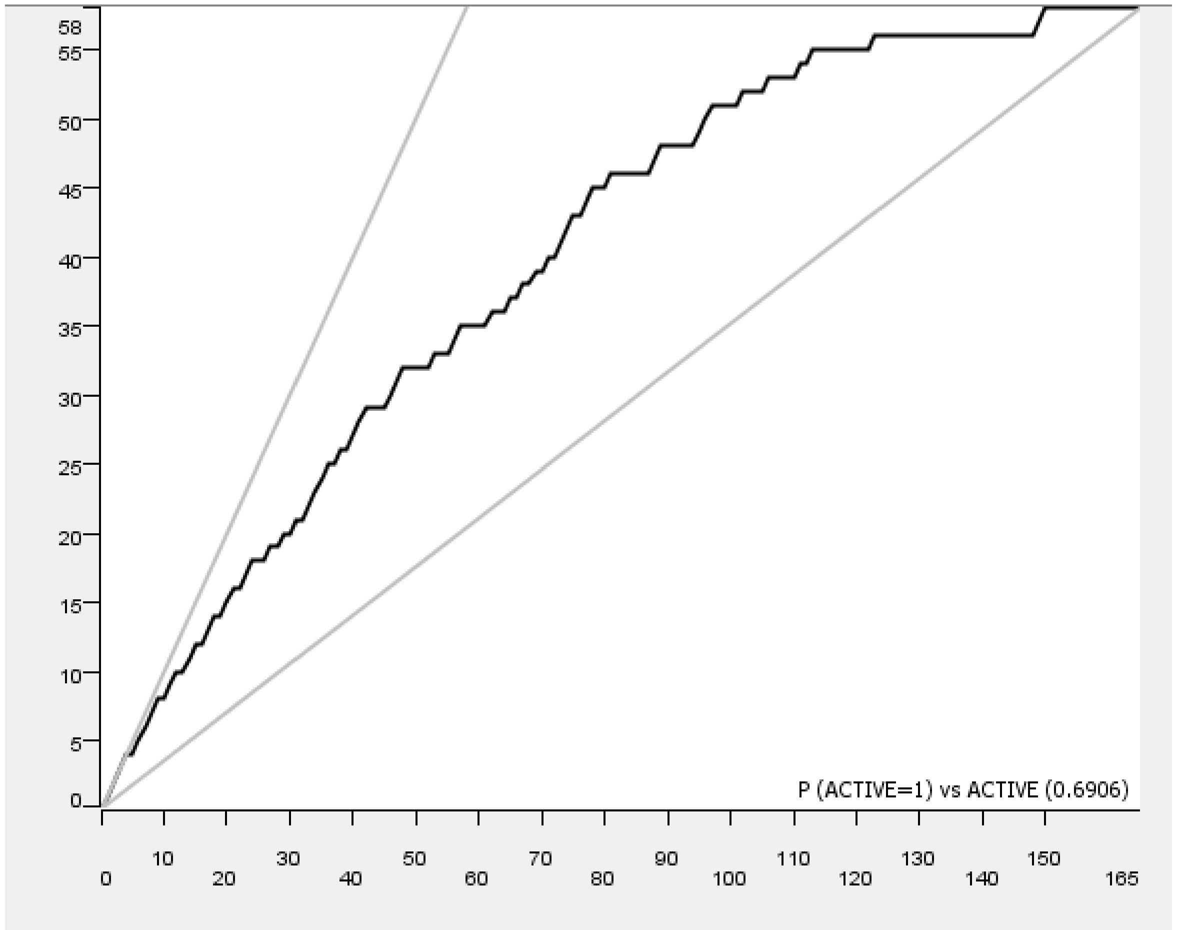

N=165

Top 10% Mean IC50	91.8 nM

	Predicted Active	Predicted Inactive
Active	26	32
Inactive	13	94

PNN - <50nM DefGood in MEK1, 15% error; Random seed = 121783

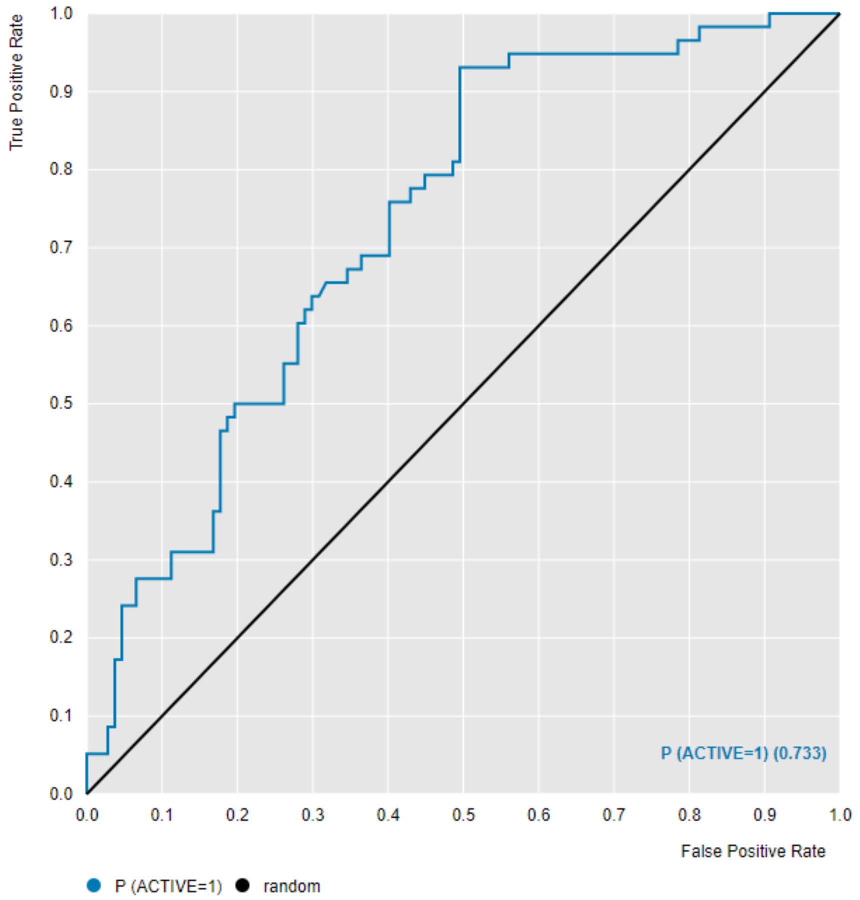

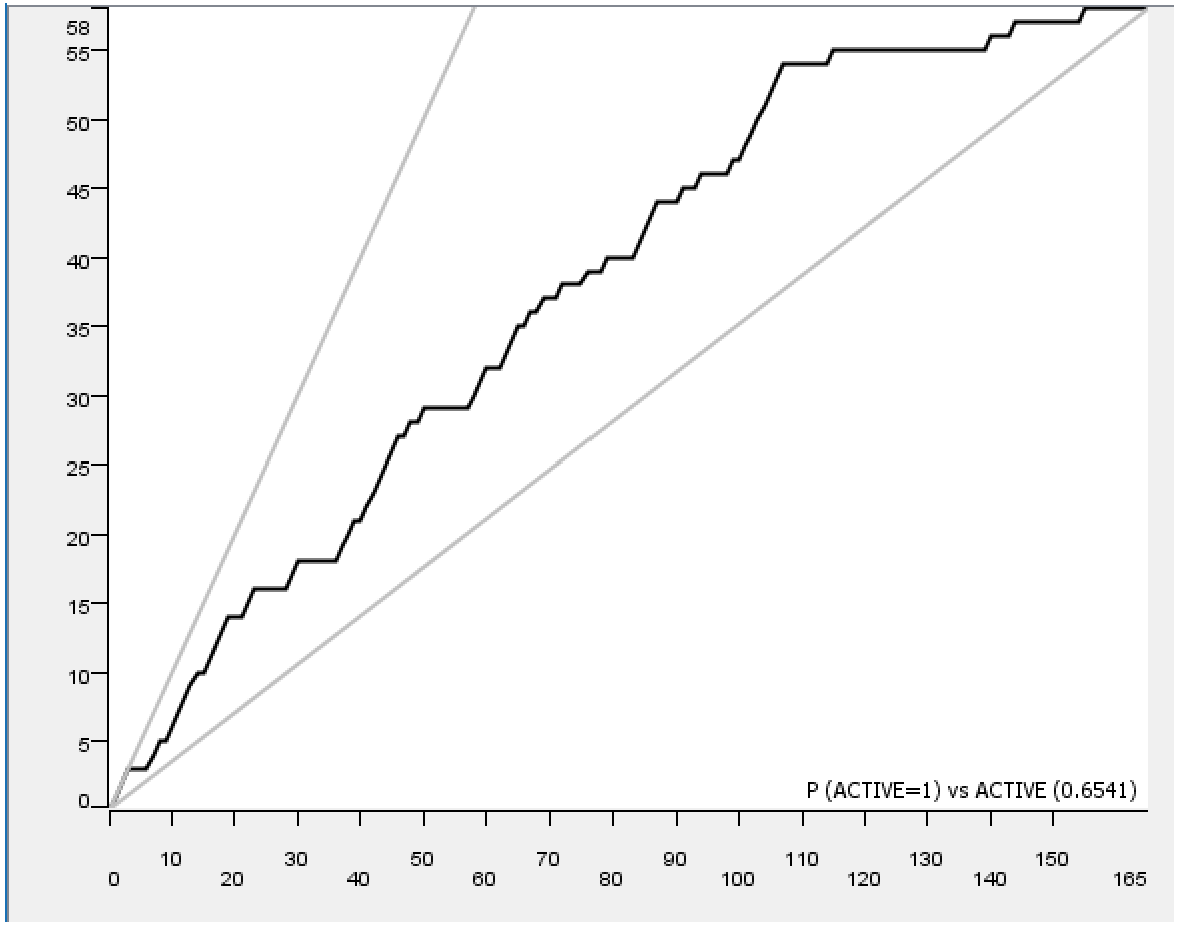

N=165

Top 10% Mean IC50	102 nM

	Predicted Active	Predicted Inactive
Active	29	29
Inactive	28	79

PNN - <50nM DefGood in MEK1, 20% error; Random seed = 121783

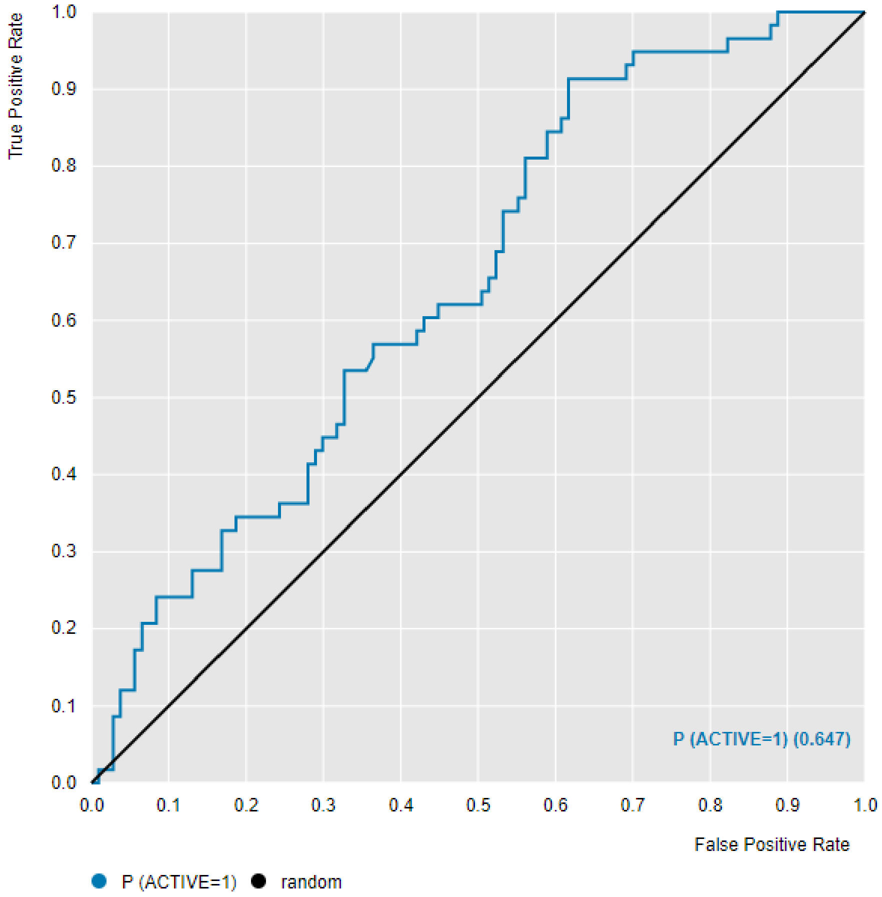

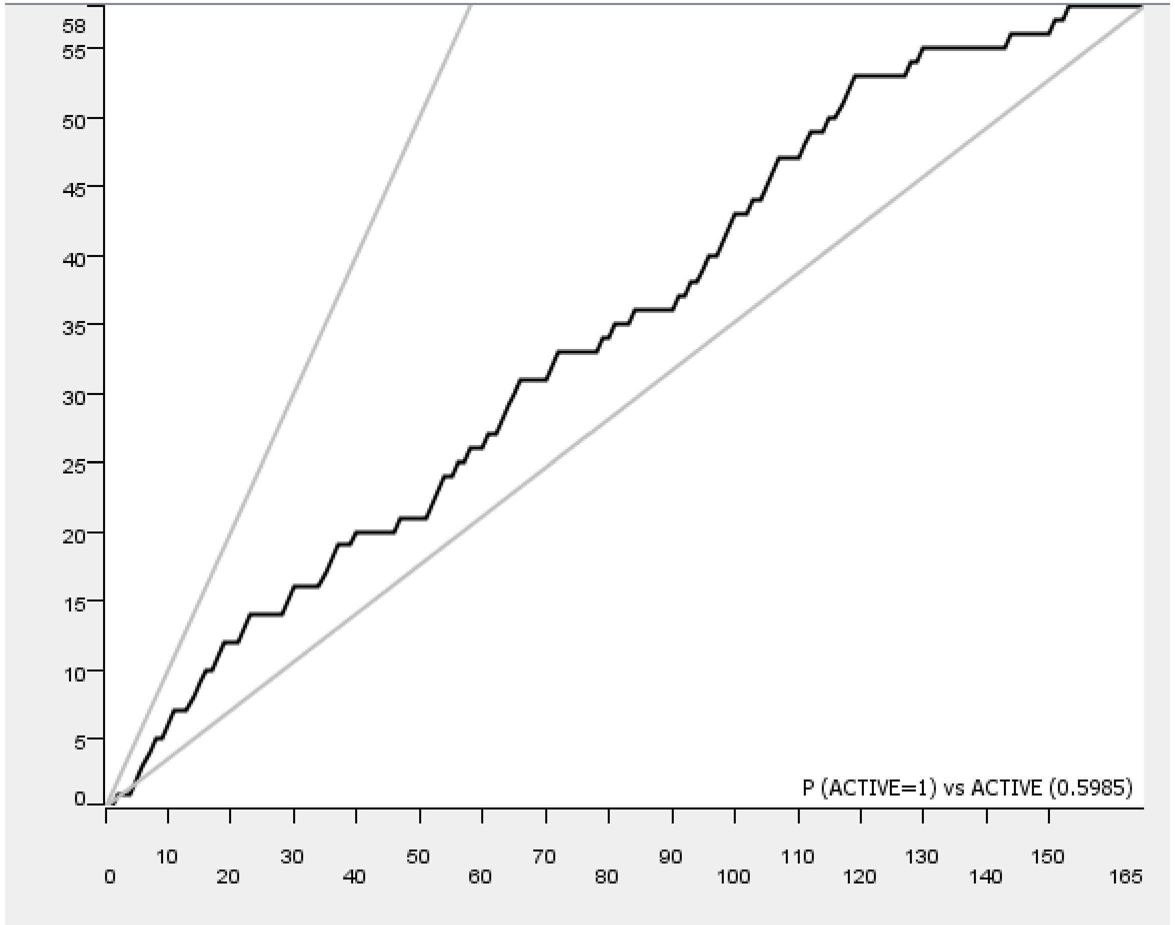

N=165

**Top 10% Mean IC50**	**12,600 nM**

	Predicted Active	Predicted Inactive
Active	22	36
Inactive	30	77

PNN - <50nM DefGood in MEK1, 20% error; Random seed = 121783 (reparameterized Theta minus = 0.35; theta plus = 0.35)

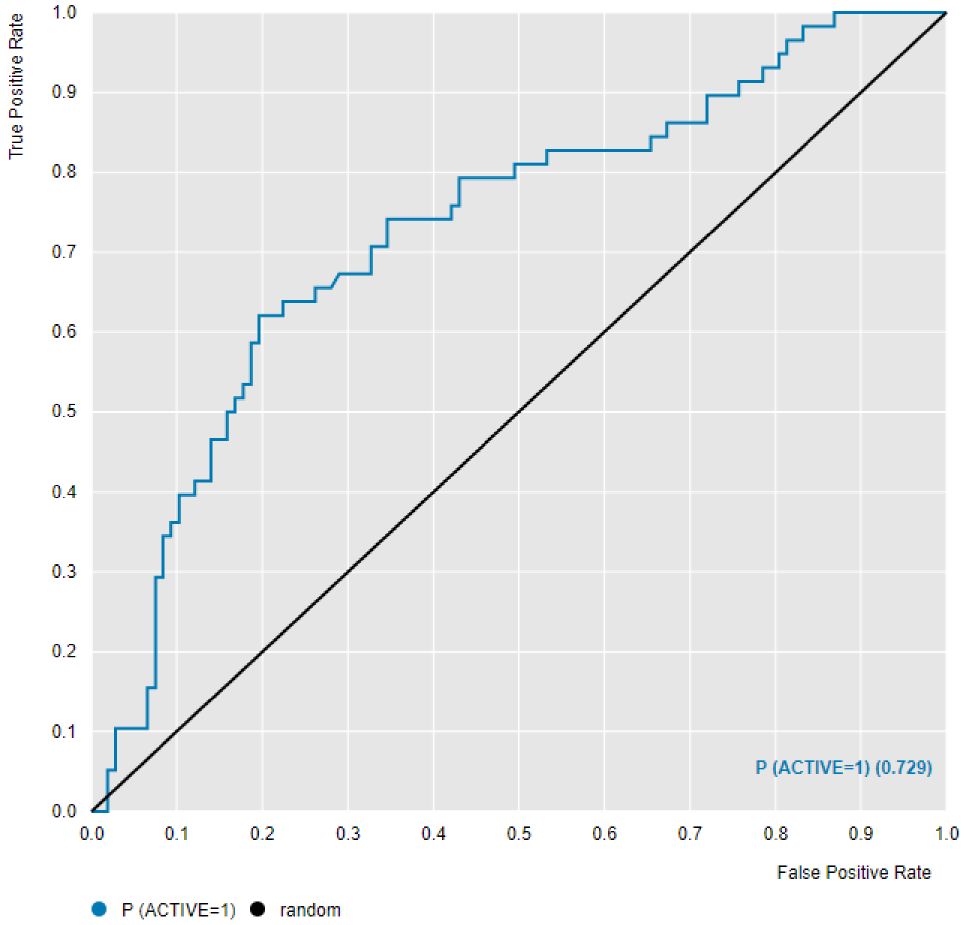

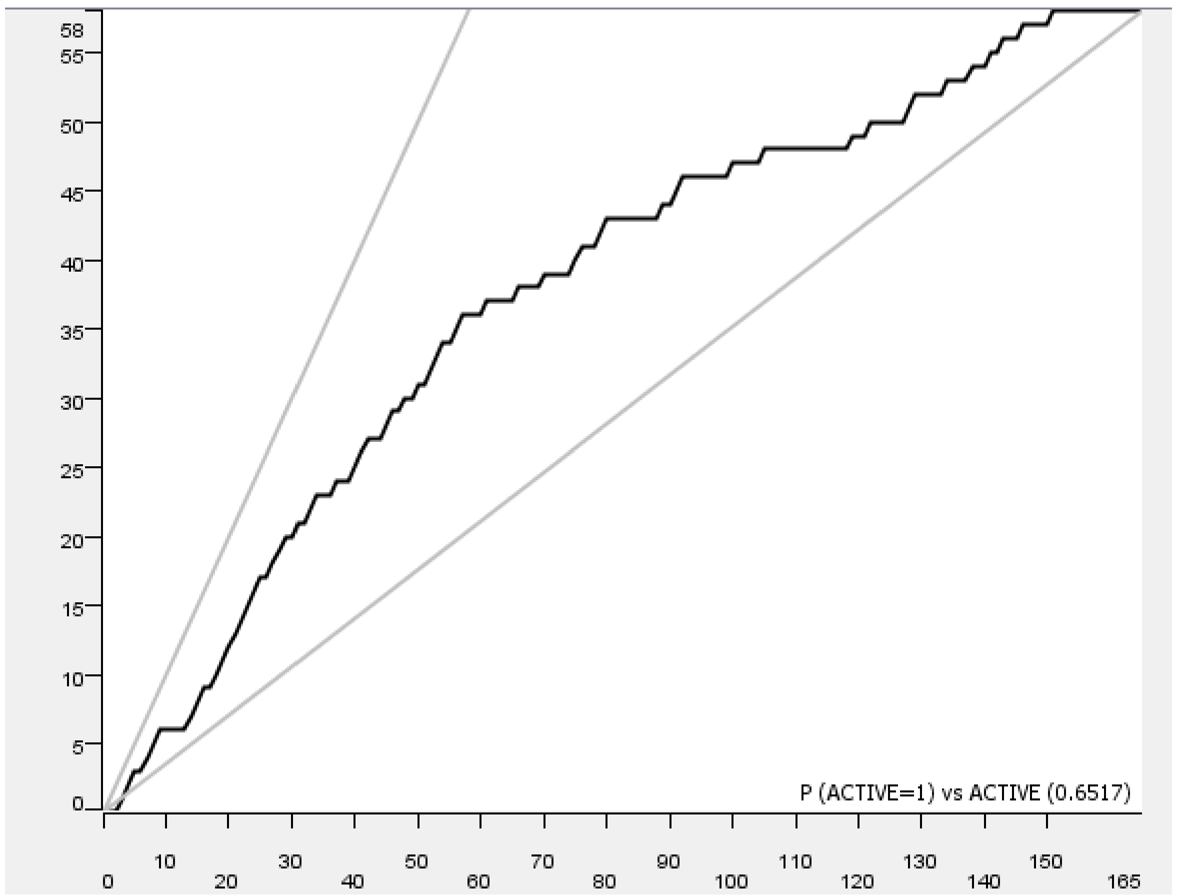

N=165

Top 10% Mean IC50	115 nM

	Predicted Active	Predicted Inactive
Active	17	41
Inactive	9	98

PNN - <50nM DefGood in MEK1, 25% error; Random seed = 121783 (reparameterized Theta minus = 0.35; theta plus = 0.35)

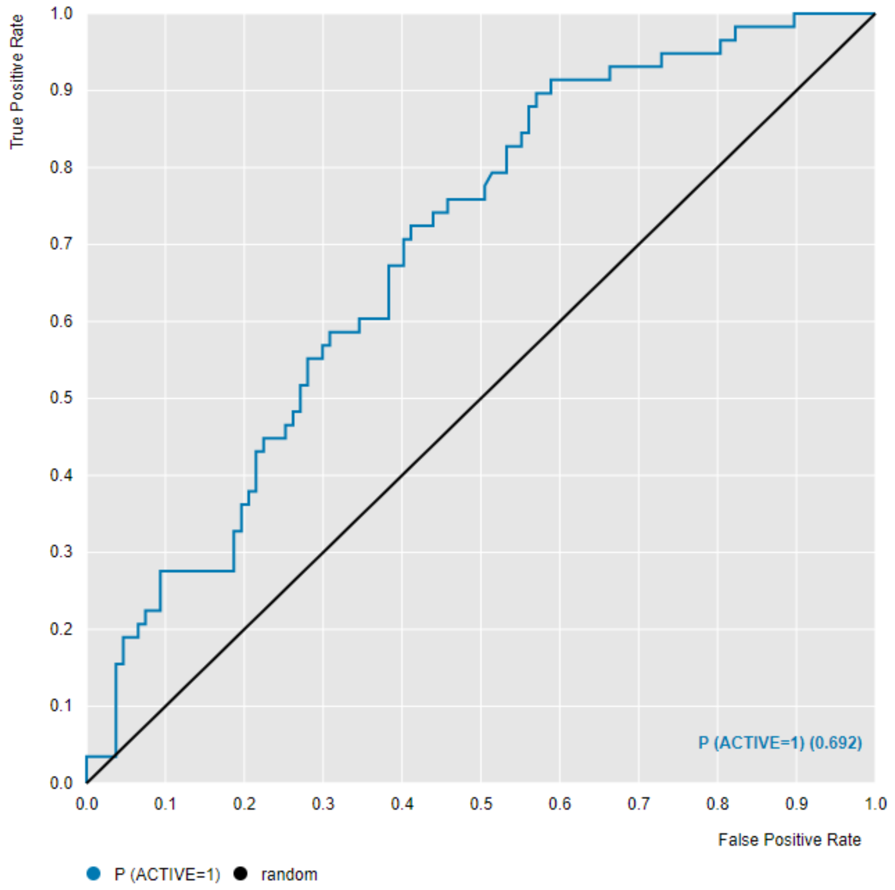

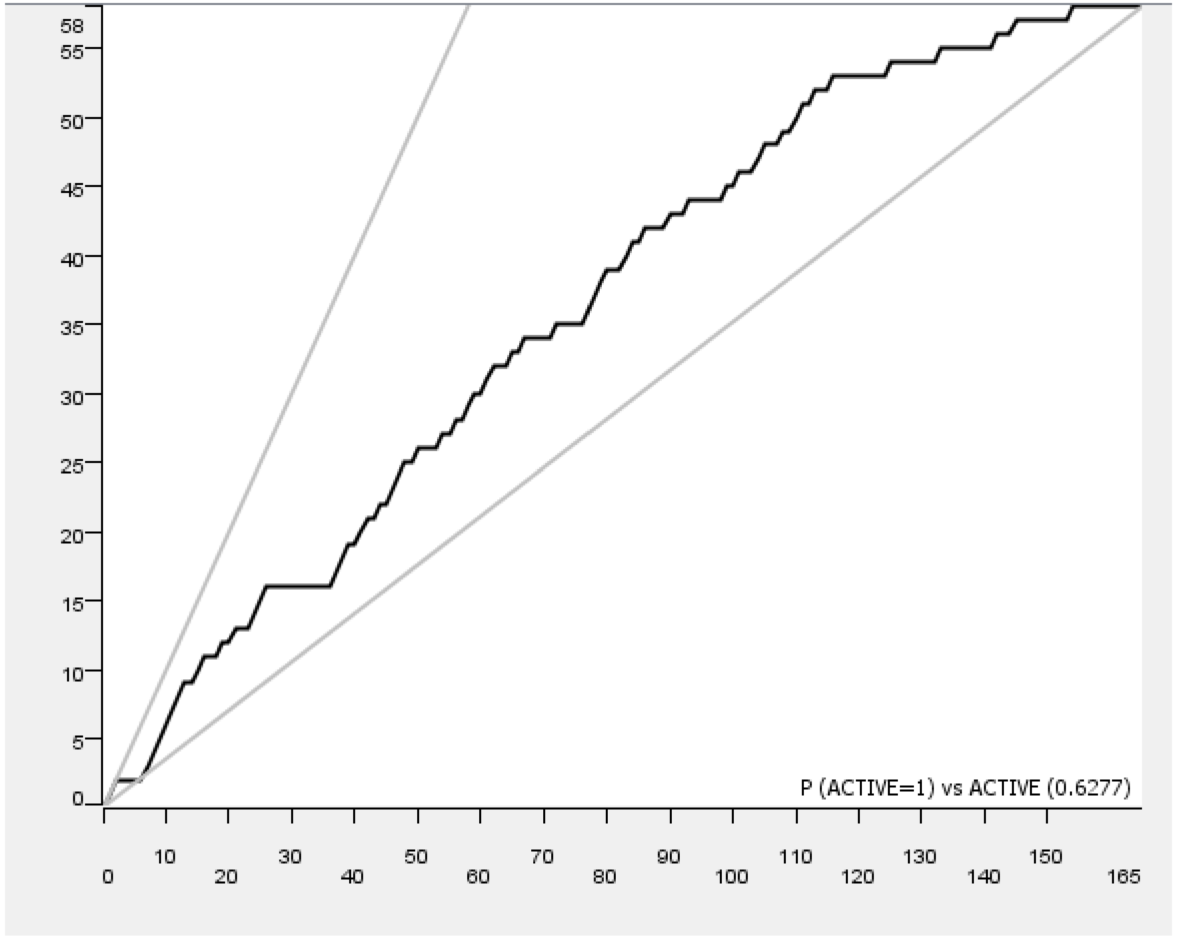

N=165

Top 10% Mean IC50	79.0 nM

	Predicted Active	Predicted Inactive
Active	24	34
Inactive	23	84

Conclusion - PNN

- Across three random seeds, the % error that lead to a significant enrichment failure and/or top 10% IC50 increase in the retrospective test was 5%, 5% and 25% for each unique random seed.
- Parameterization did not alter one of the failure points, and moved the point of failure from 20% to 25% in the last random split.

PARP1

PARP1 NBN Design

- Rules - $RELATION$ = "=" OR $IC50$ > 999 OR $IC50$ < 10 => TRUE
- Sort ascending and remove duplicates
- DefGood scanned between 5 and 25 nM
- 80-20 split for both actives and inactives
- NBN – ECFP4 used Classifier where Prediction was Active = True
- Resulted in 1933 compounds after cleaning the data

<5 nM DefGood in PARP1

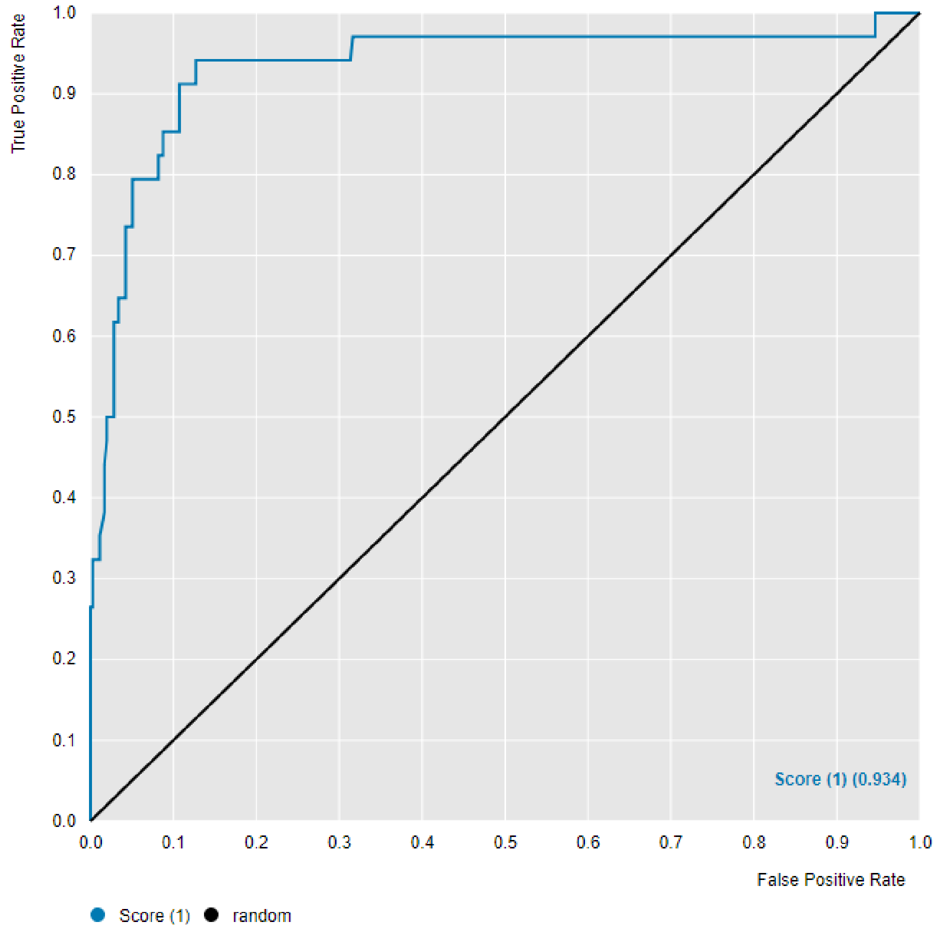

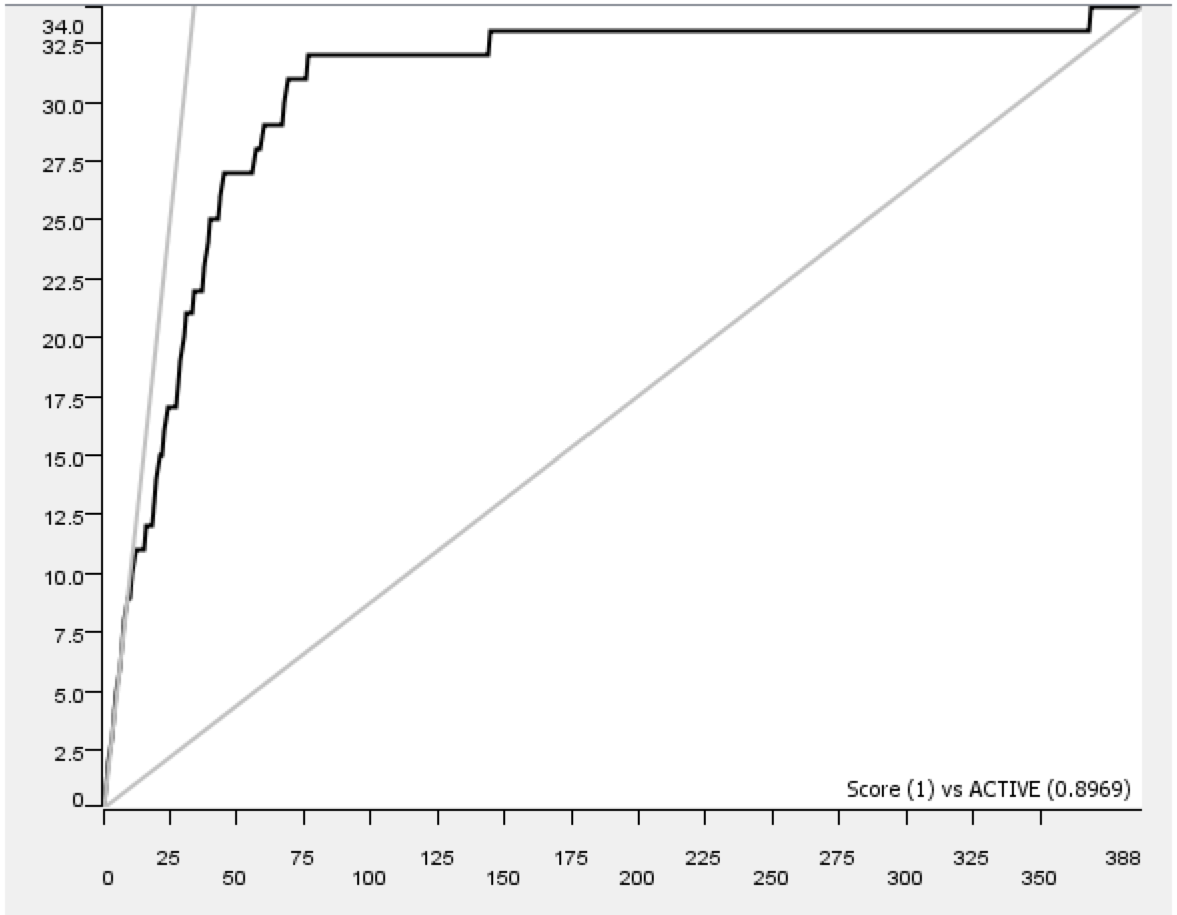

N=388

Top 10% Mean IC50	106 nM

	Predicted Active	Predicted Inactive
Active	31	3
Inactive	42	312

<10 nM DefGood in PARP1

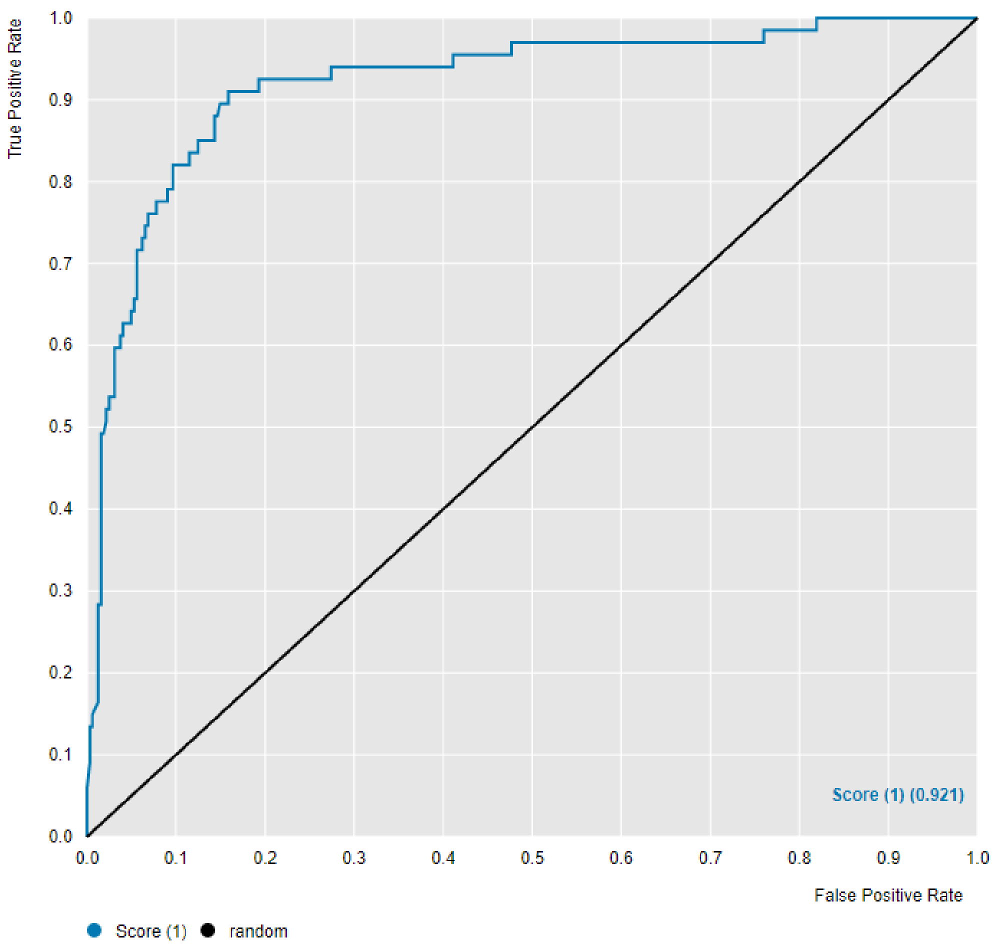

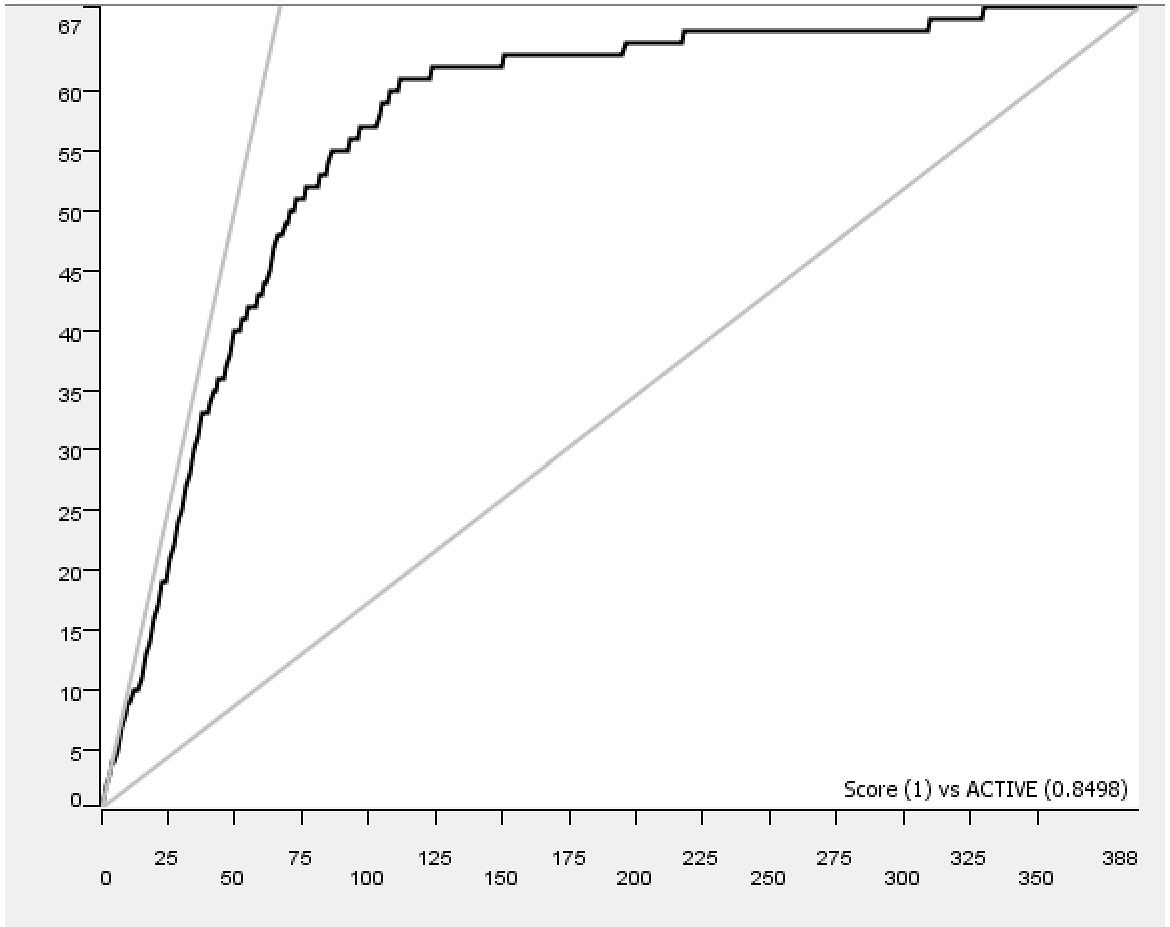

N=388

Top 10% Mean IC50	10.5 nM

	Predicted Active	Predicted Inactive
Active	60	7
Inactive	48	273

<15 nM DefGood in PARP1

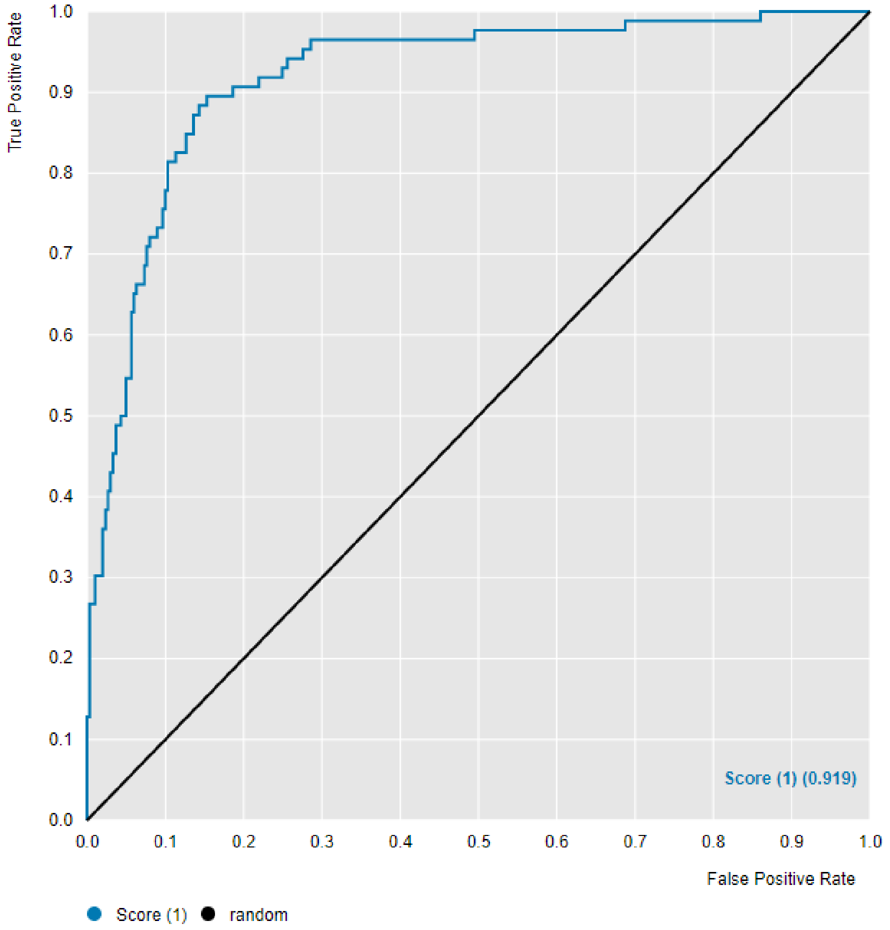

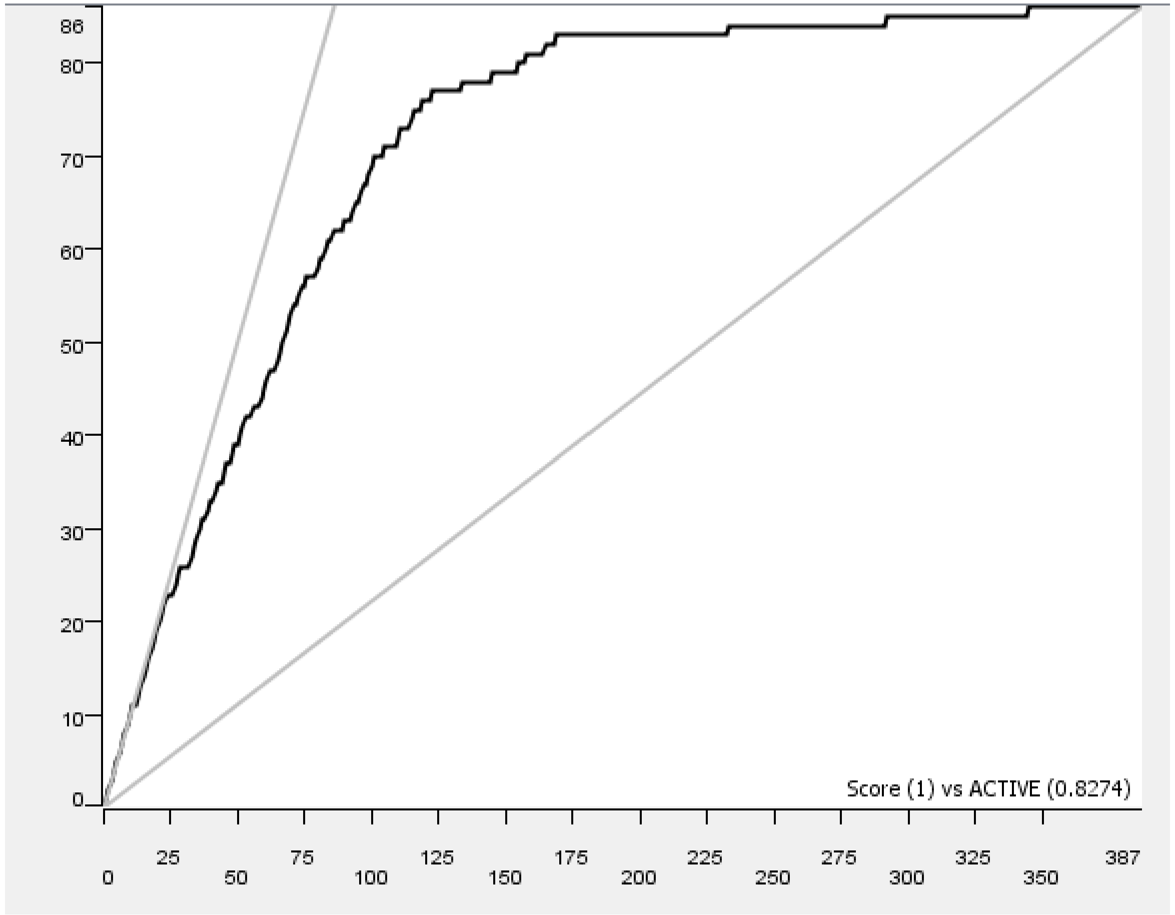

N=388

Top 10% Mean IC50	13.4 nM

	Predicted Active	Predicted Inactive
Active	76	10
Inactive	45	256

<20 nM DefGood in PARP1

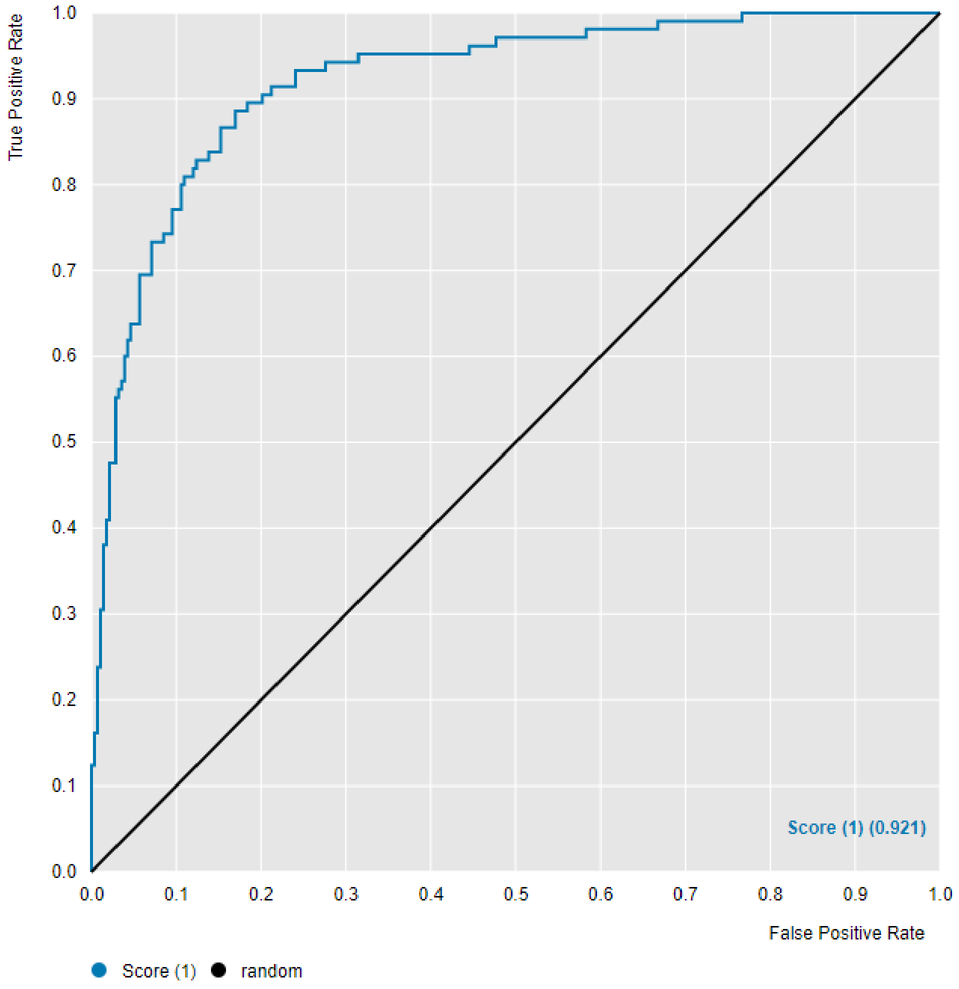

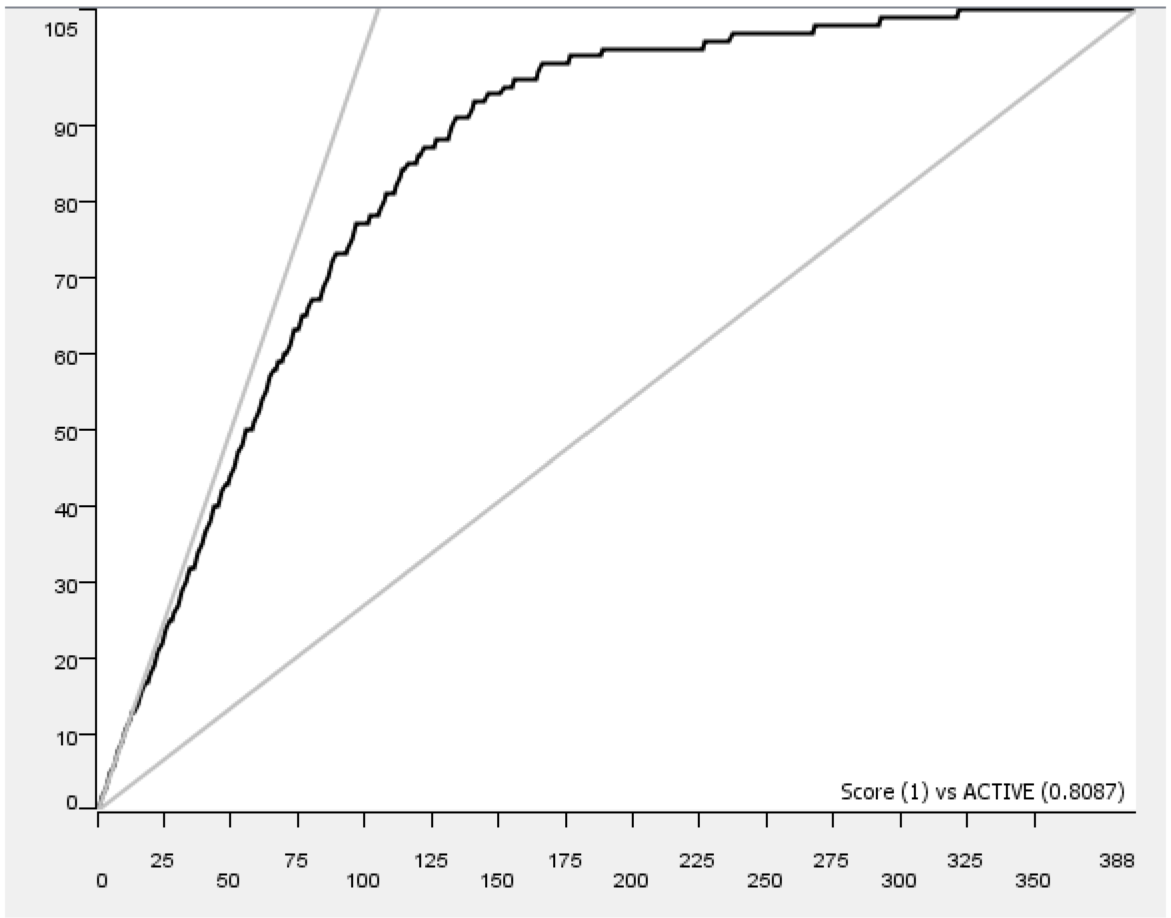

N=388

**Top 10% Mean IC50**	**7.5 nM**

	Predicted Active	Predicted Inactive
Active	88	17
Inactive	42	241

<25 nM DefGood in PARP1

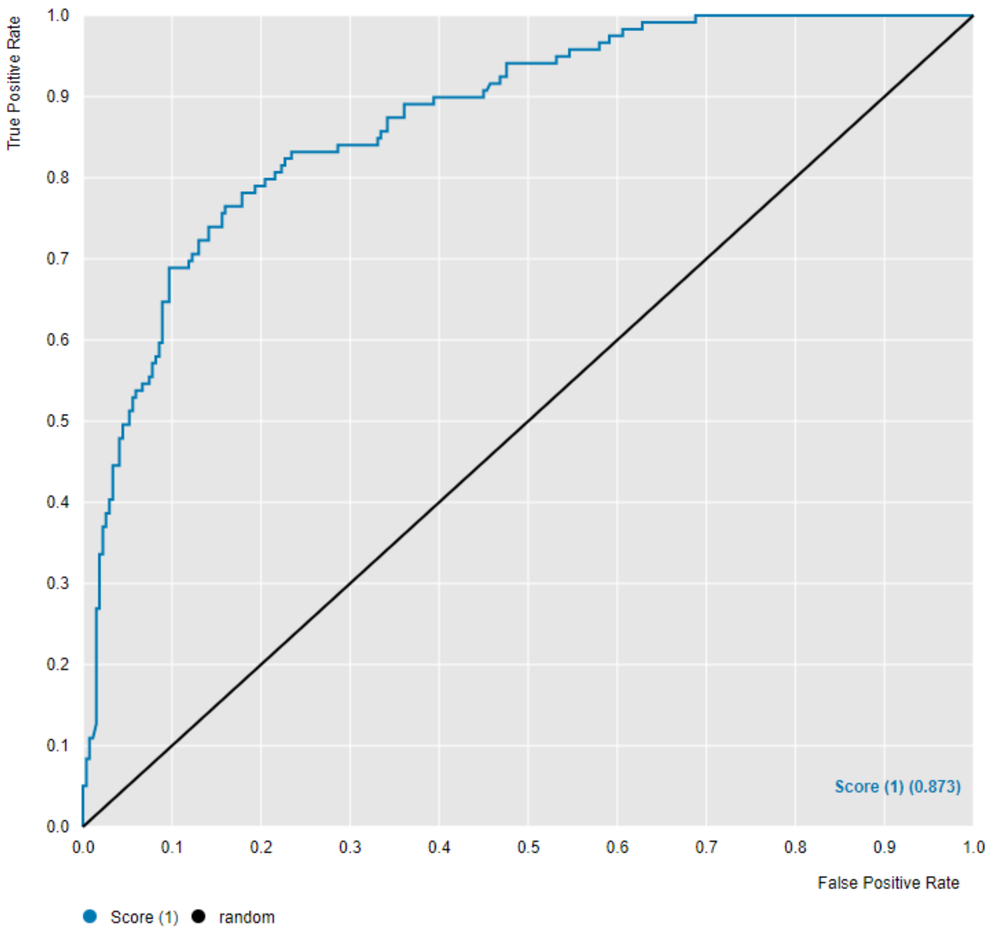

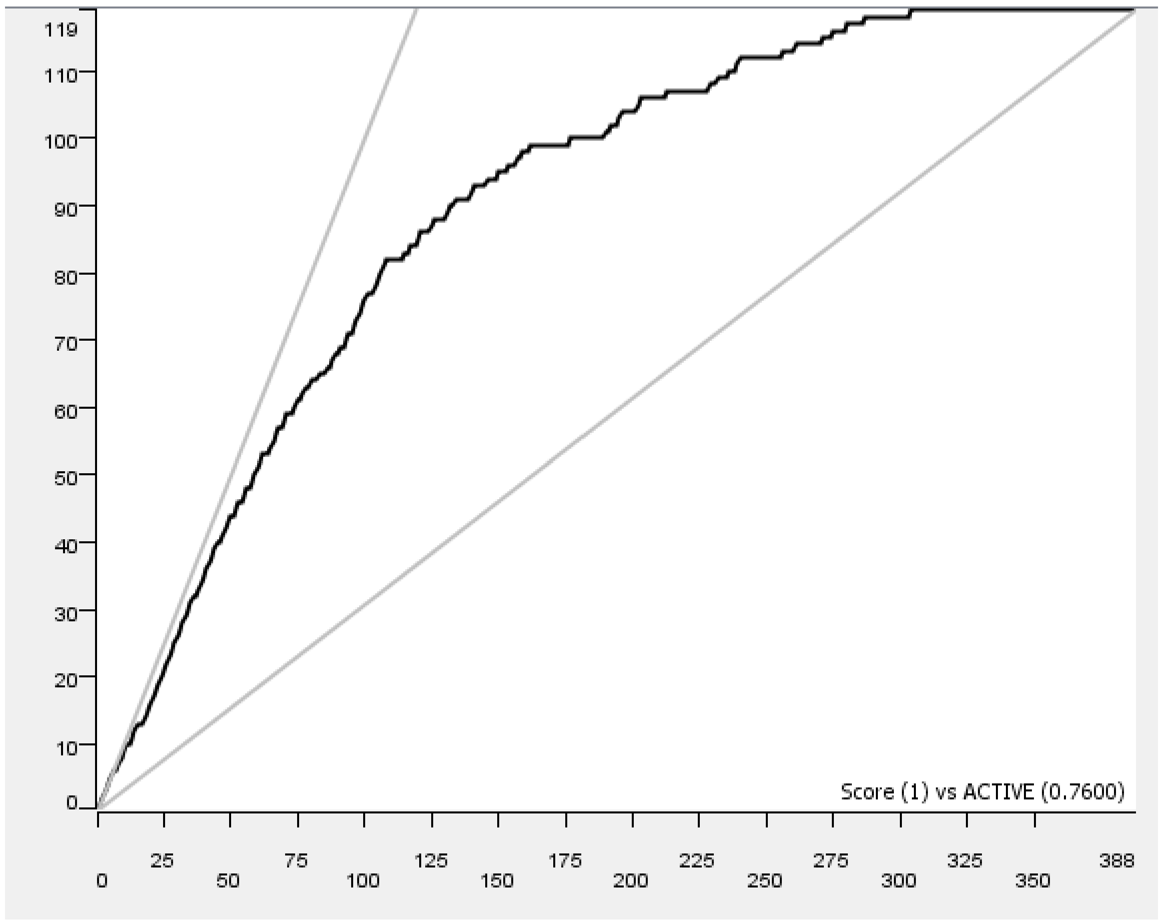

N=388

Top 10% Mean IC50	15.7 nM

	Predicted Active	Predicted Inactive
Active	89	30
Inactive	42	227

Decision on DefGood

- <20 nM has the best performance with regard to enrichment and mean top 10% IC50.

RF - <20 nM DefGood in PARP1

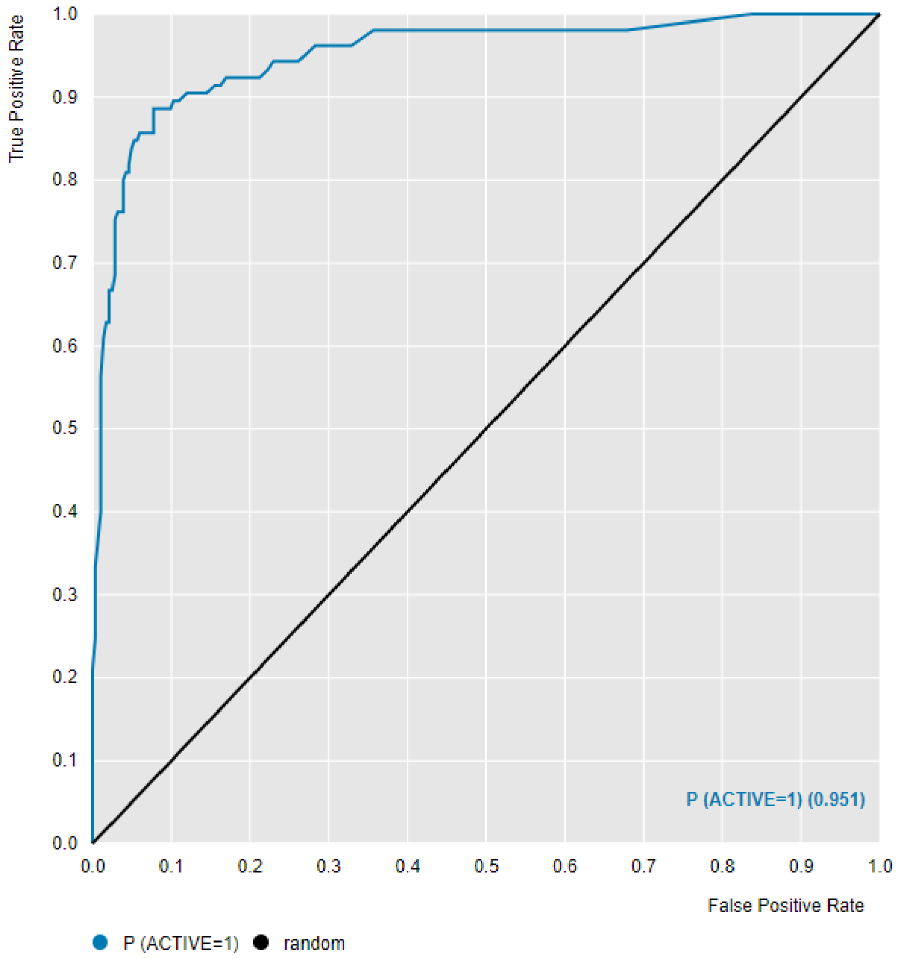

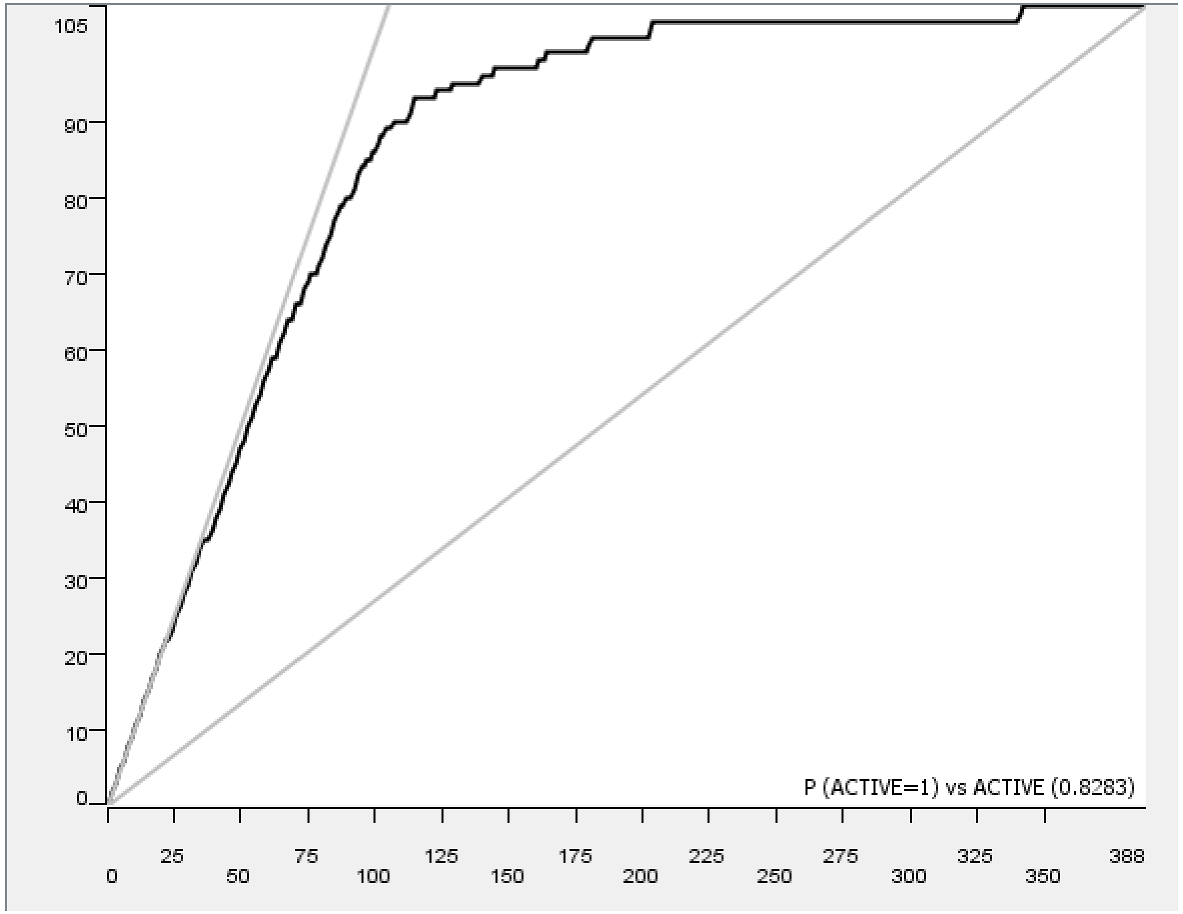

N=388

**Top 10% Mean IC50**	**6.1 nM**

	**Predicted Active**	**Predicted Inactive**
Active	90	15
Inactive	17	266

Decision on DefGood - RF

- Work in ALK showed DefGood in the NBN was similar to DefGood in an RF. Therefore, this value for DefGood from the NBN design was used as DefGood.

PNN - <20 nM DefGood in PARP1

- Properties List (46 calculatable properties were used)
- Parameterization (Global Accuracy for Activity at a 20 nM decision value was used, and a 5-fold stratified sampling cross validation was used to parameterize theta minus and theta plus) Theta Minus = 0.05 and Theta Plus = 0.55

PNN - <20 nM DefGood in PARP1

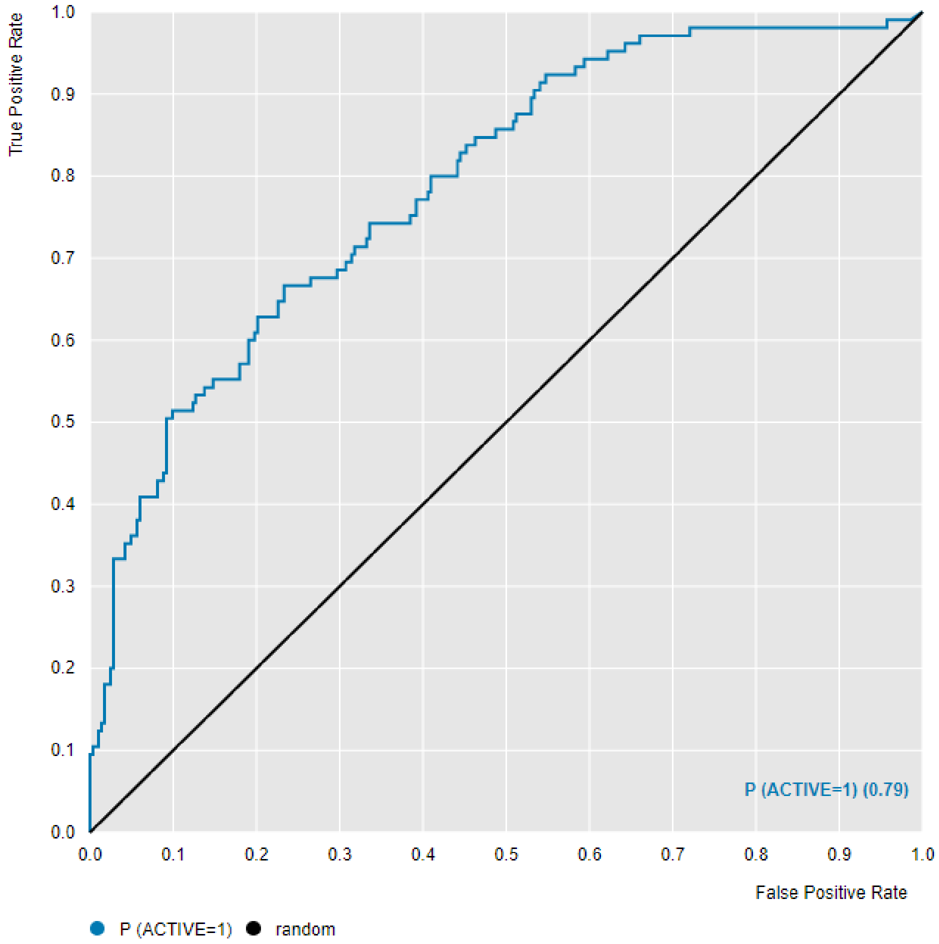

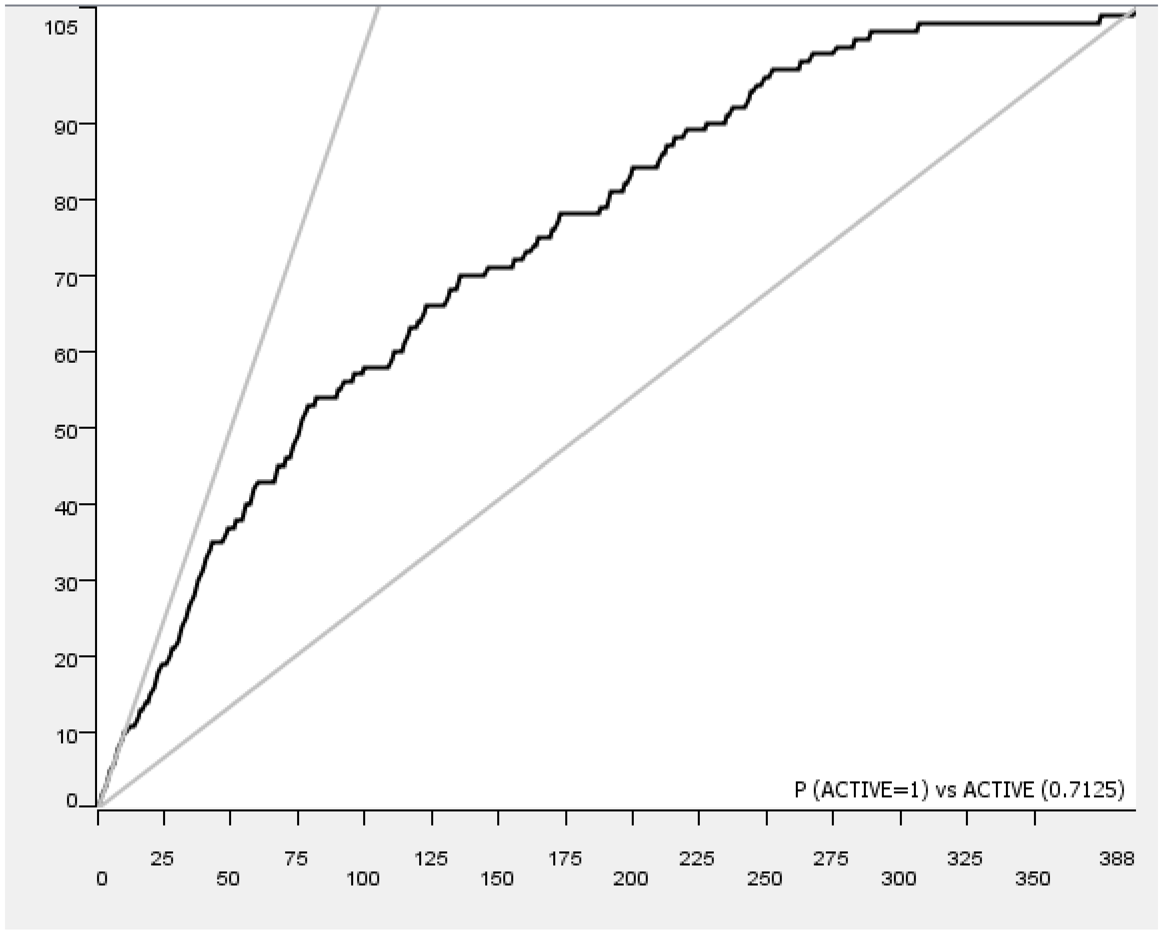

N=388

**Top 10% Mean IC50**	**47.7 nM**

	**Predicted Active**	**Predicted Inactive**
Active	19	86
Inactive	5	278

Decision on DefGood - PNN

- Parameterization resulted in a useful PNN with the <20 nM DefGood. Parameterization for each model adds significant time to the process. A rough benchmark can be applied where point of failure is discovered and then the model is parameterized to ensure that the situation is optimal for the PNN. This will be applied to the discovery of the error tolerance threshold for the PNN as was done in the beta 2 adrenergic receptor space.

NBN Error Tolerance- <20 nM DefGood in PARP1

- 0-50% absolute error

<20 nM DefGood in PARP1, 5% error; Random seed = 1515533876005

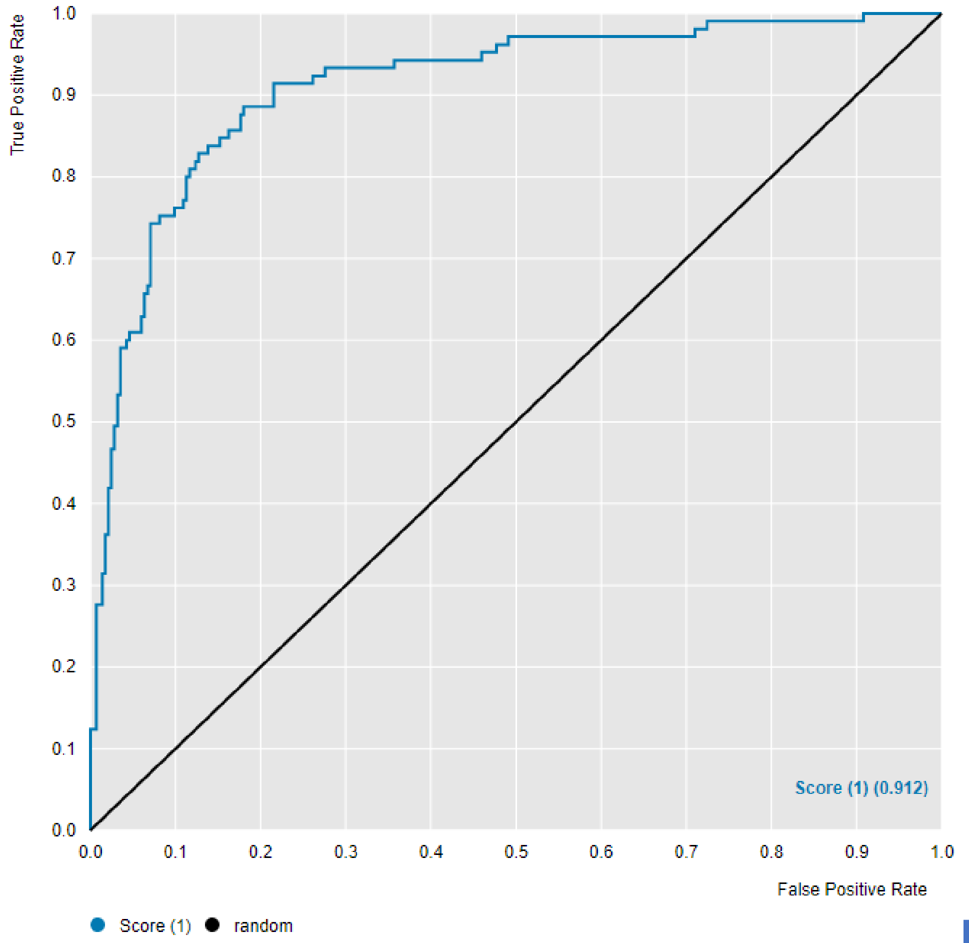

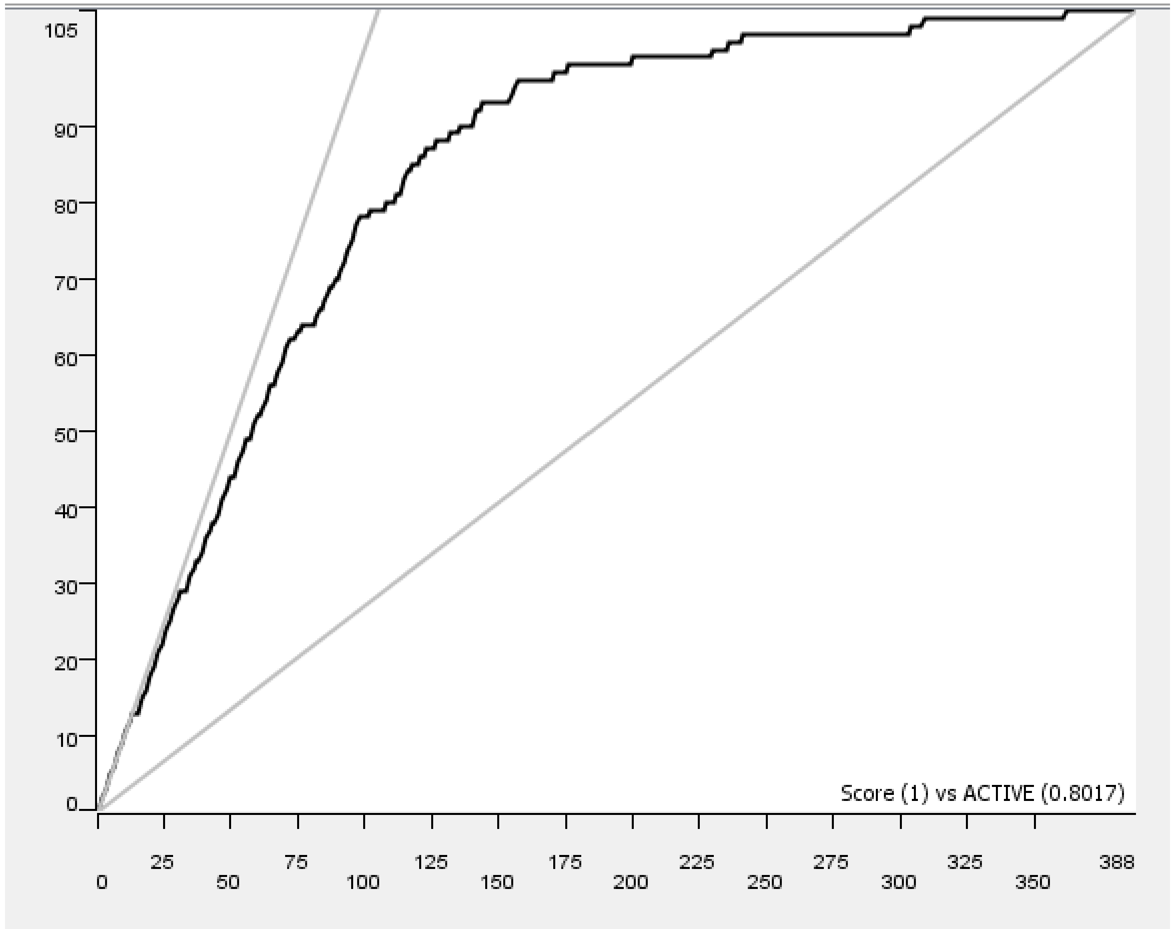

N=388

**Top 10% Mean IC50**	**10,200 nM One >380,000 mistake at the bottom**

	Predicted Active	Predicted Inactive
Active	90	15
Inactive	50	233

<20nM DefGood in PARP1, 5% error; Random seed = 429

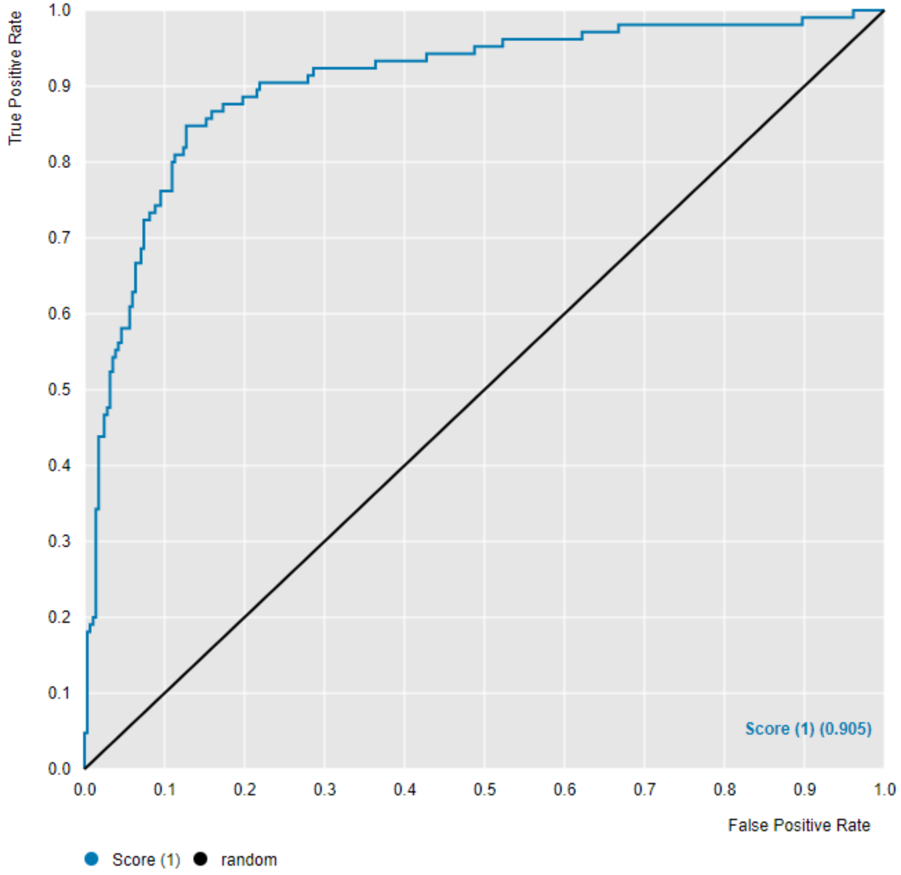

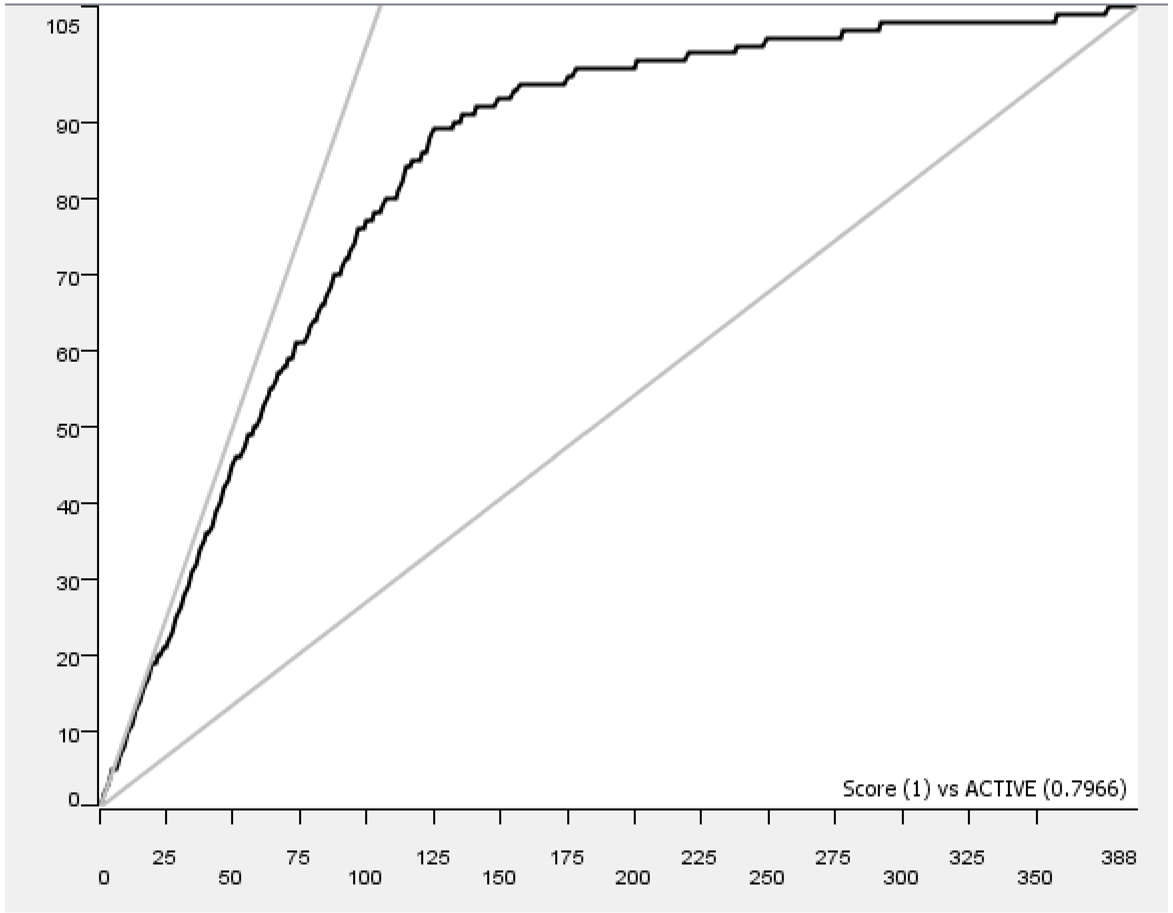

N=388

Top 10% Mean IC50	9.6 nM

	Predicted Active	Predicted Inactive
Active	89	16
Inactive	41	242

<20nM DefGood in PARP1, 10% error; Random seed = 429

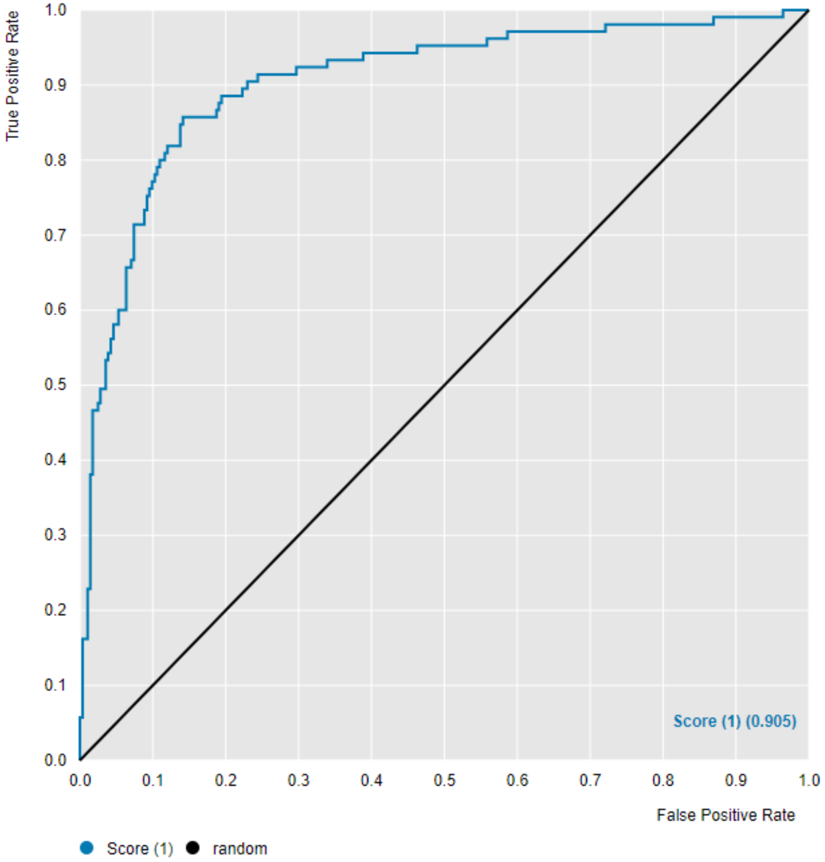

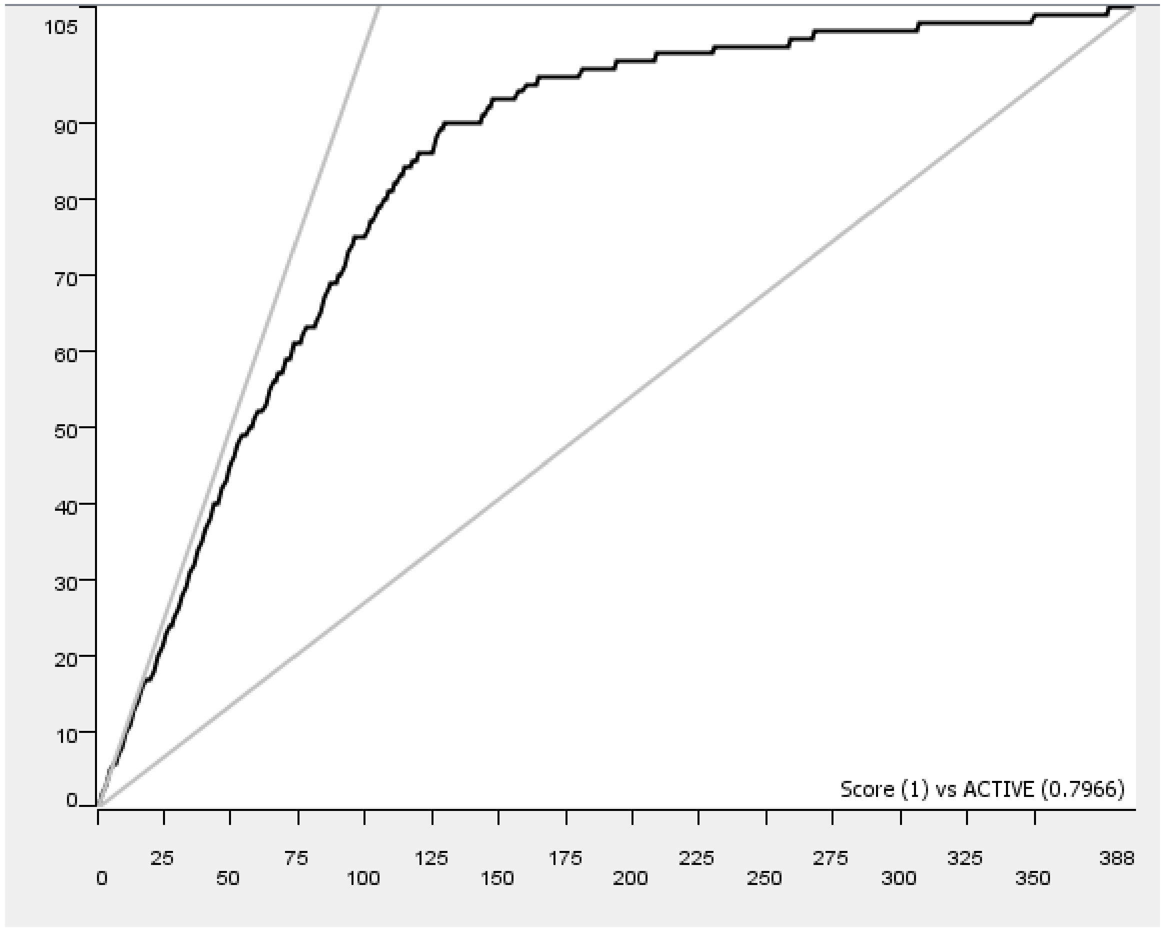

N=388

Top 10% Mean IC50	9.5 nM

	Predicted Active	Predicted Inactive
Active	90	15
Inactive	42	241

<20nM DefGood in PARP1, 20% error; Random seed = 429

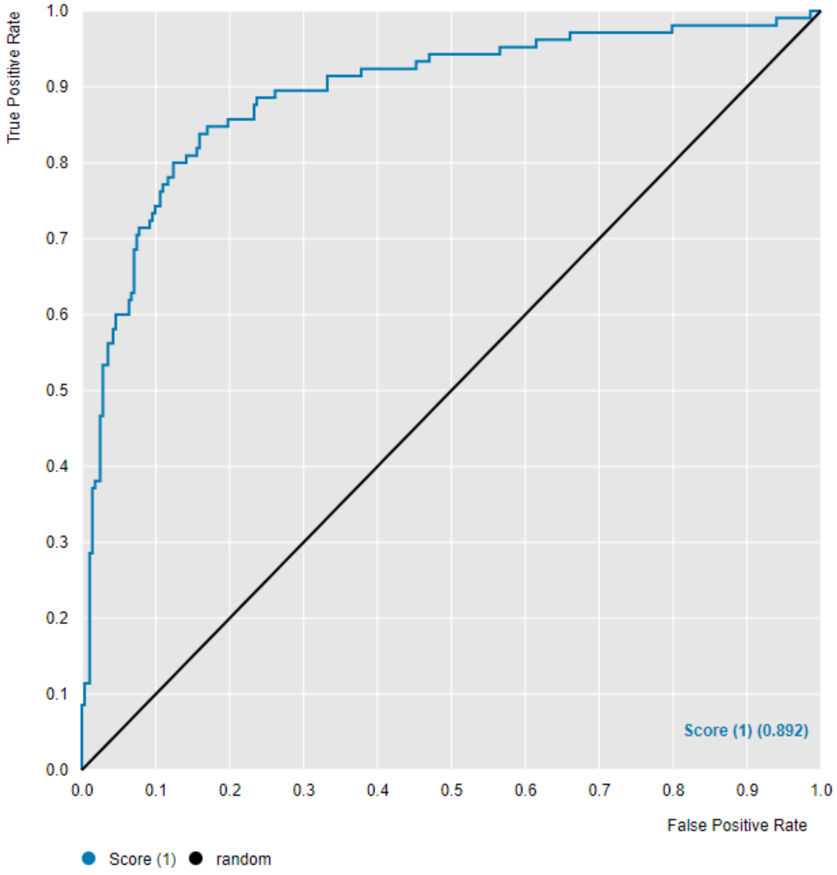

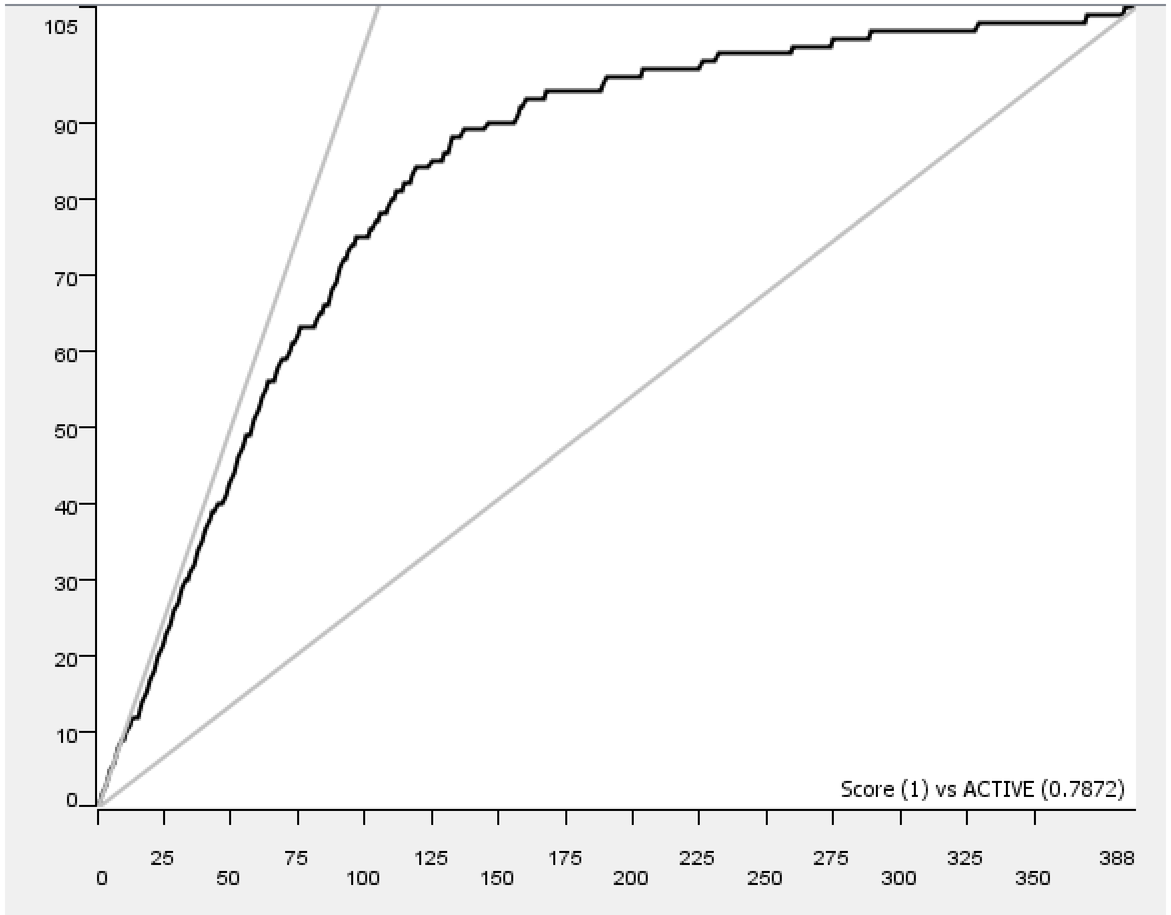

N=388

Top 10% Mean IC50	9.6 nM

	Predicted Active	Predicted Inactive
Active	78	27
Inactive	30	253

<20nM DefGood in PARP1, 30% error; Random seed = 429

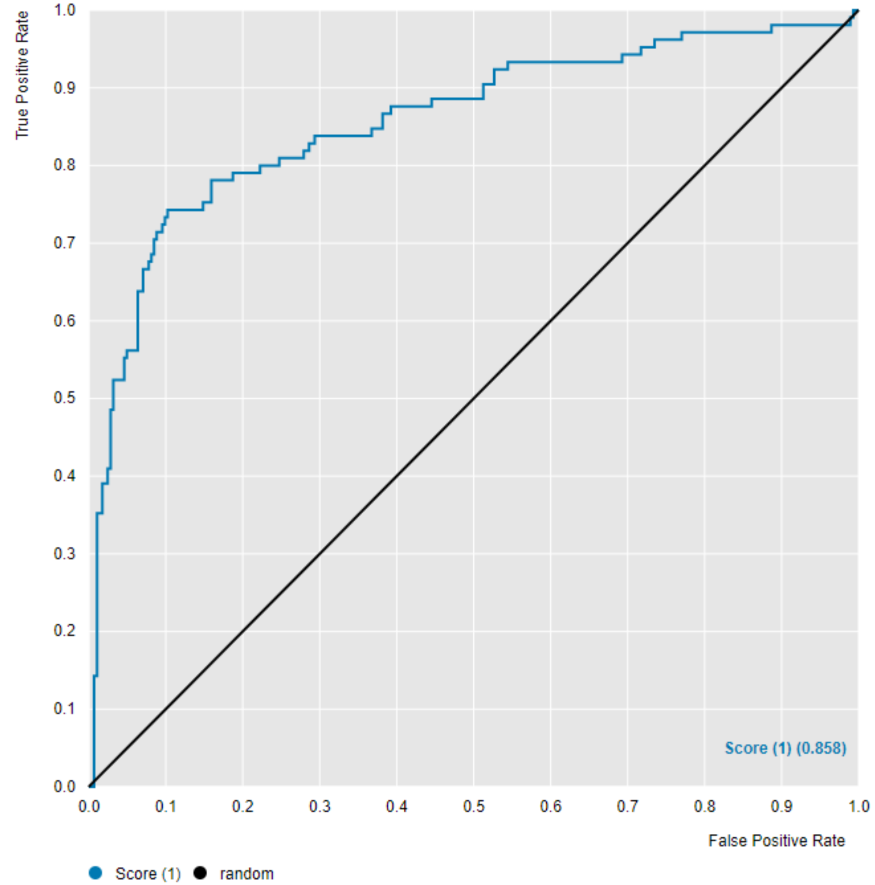

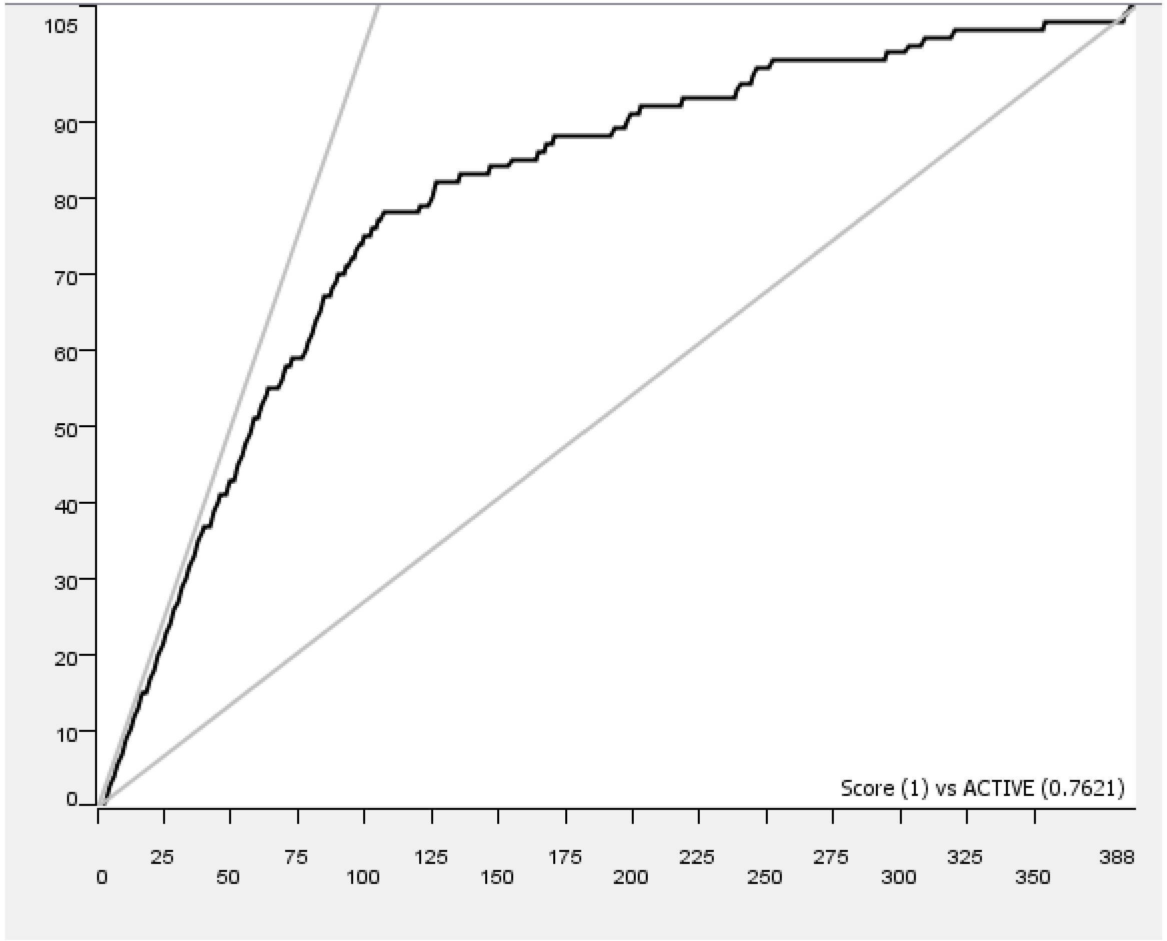

N=388

**Top 10% Mean IC50**	**9.2 nM**

	Predicted Active	Predicted Inactive
Active	87	18
Inactive	82	201

<20nM DefGood in PARP1, 40% error; Random seed = 429

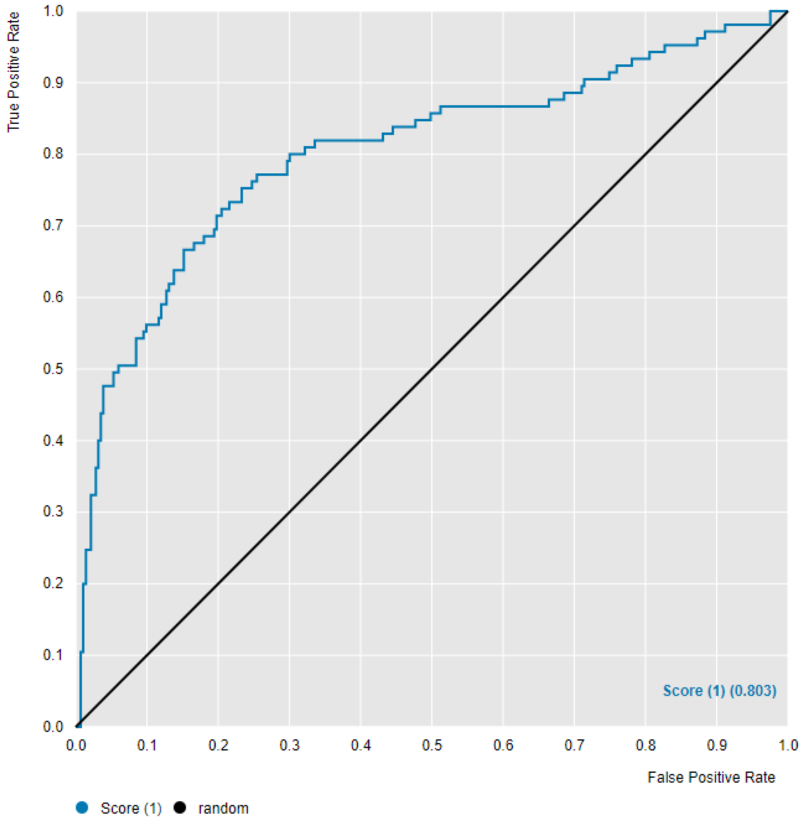

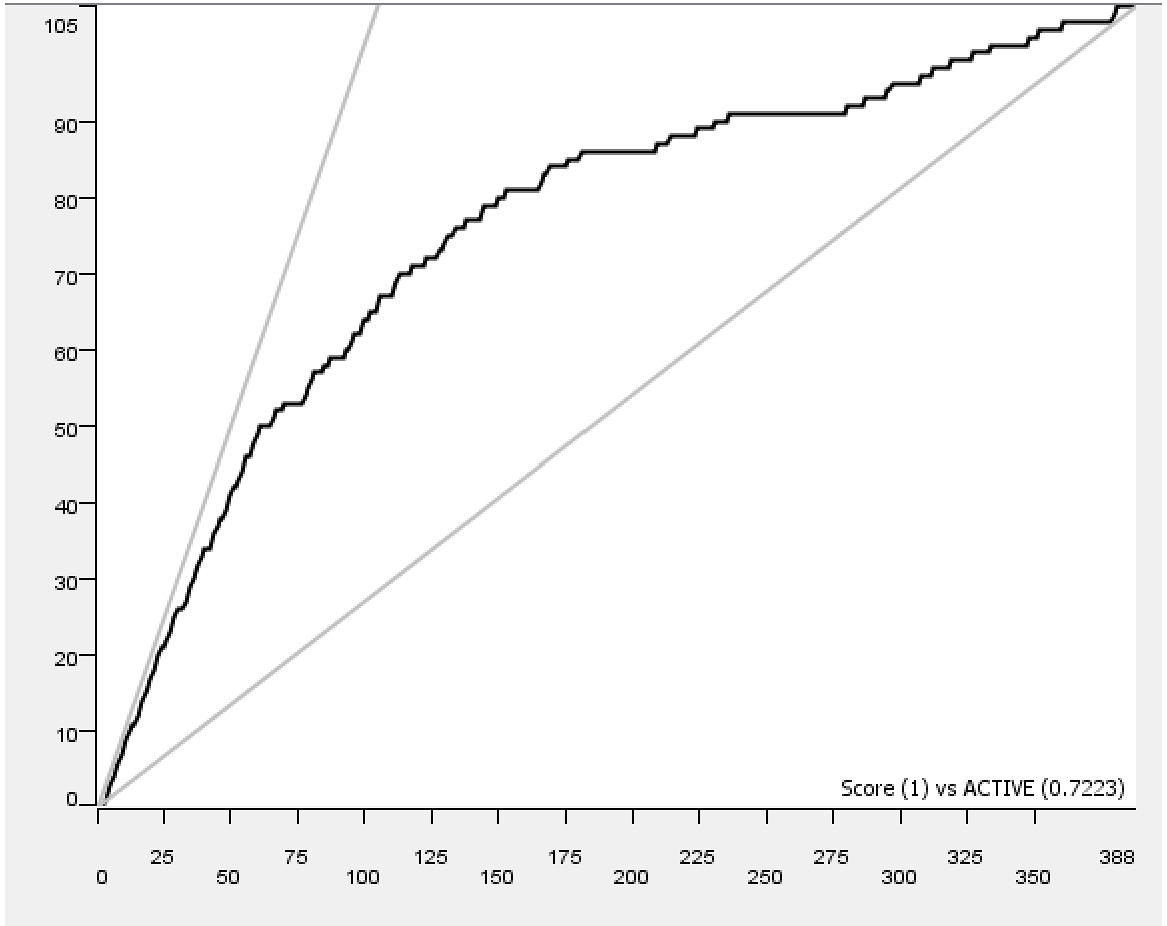

N=388

Top 10% Mean IC50	510 nM

	Predicted Active	Predicted Inactive
Active	77	28
Inactive	61	222

<20nM DefGood in PARP1, 45% error; Random seed = 429

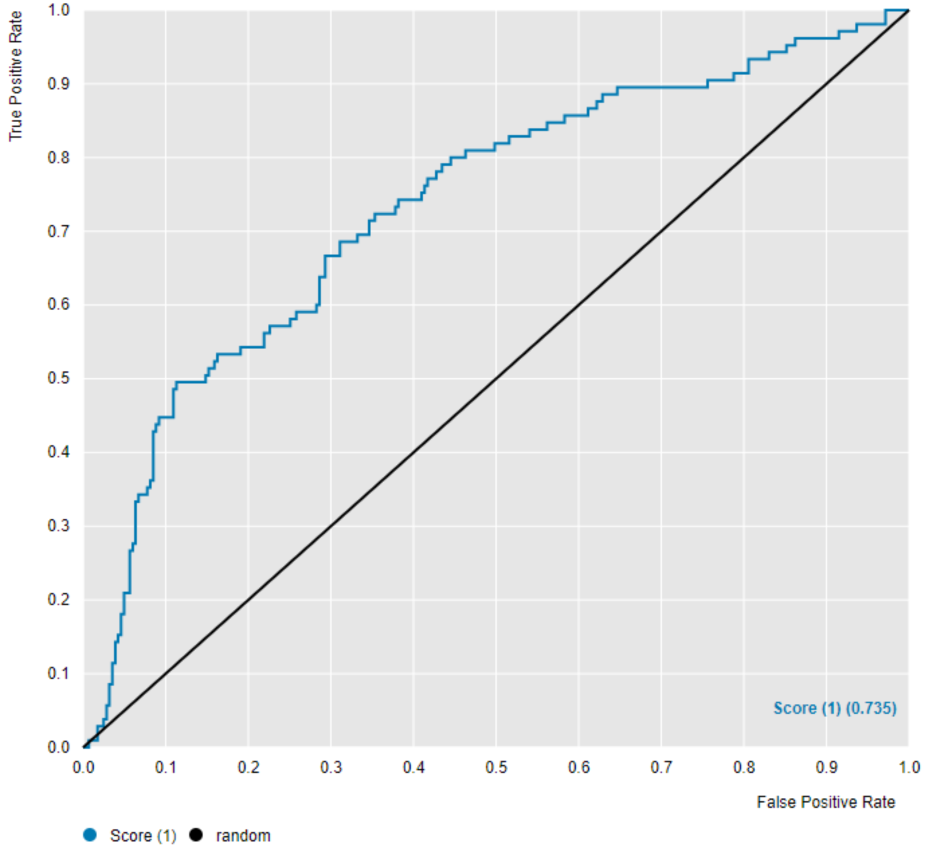

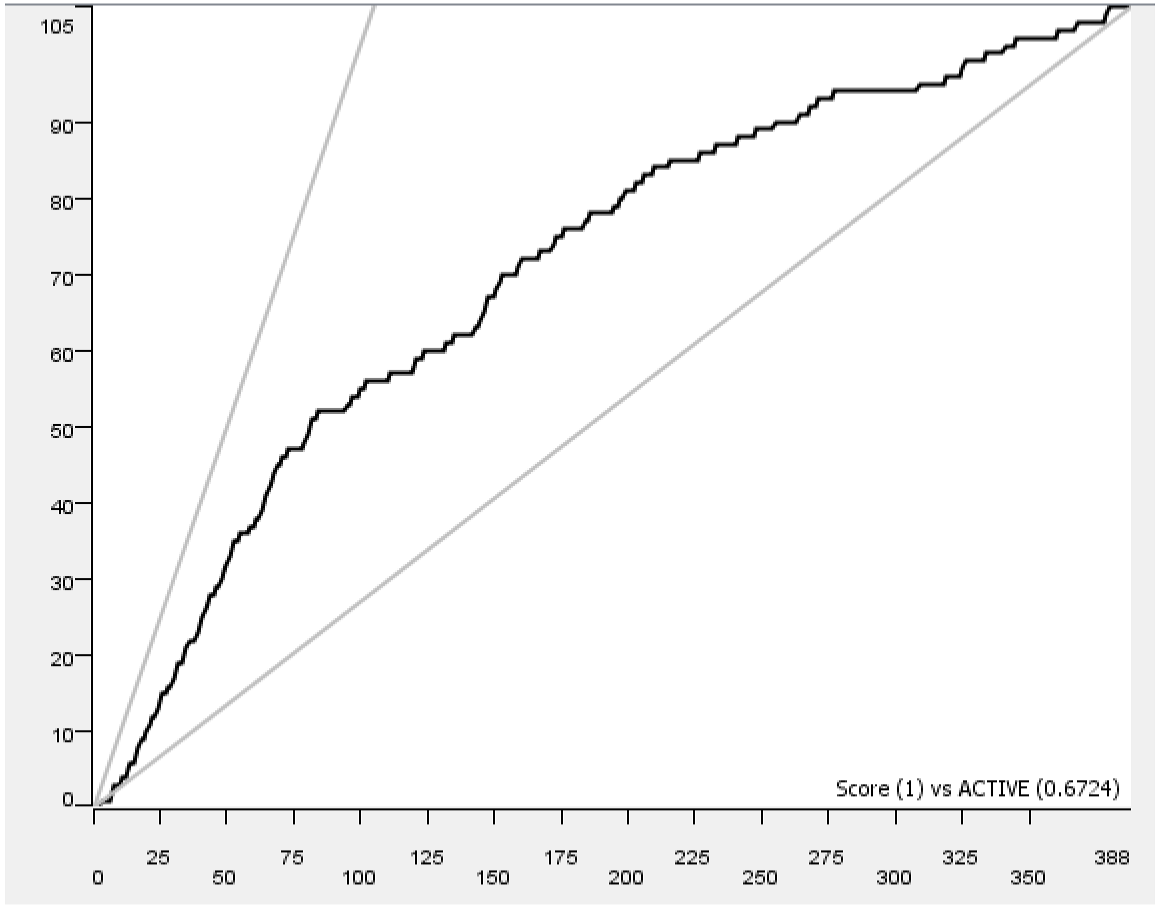

N=388

Top 10% Mean IC50	7,400 nM

	Predicted Active	Predicted Inactive
Active	85	20
Inactive	134	145

<20nM DefGood in PARP1, 20% error; Random seed = 121783

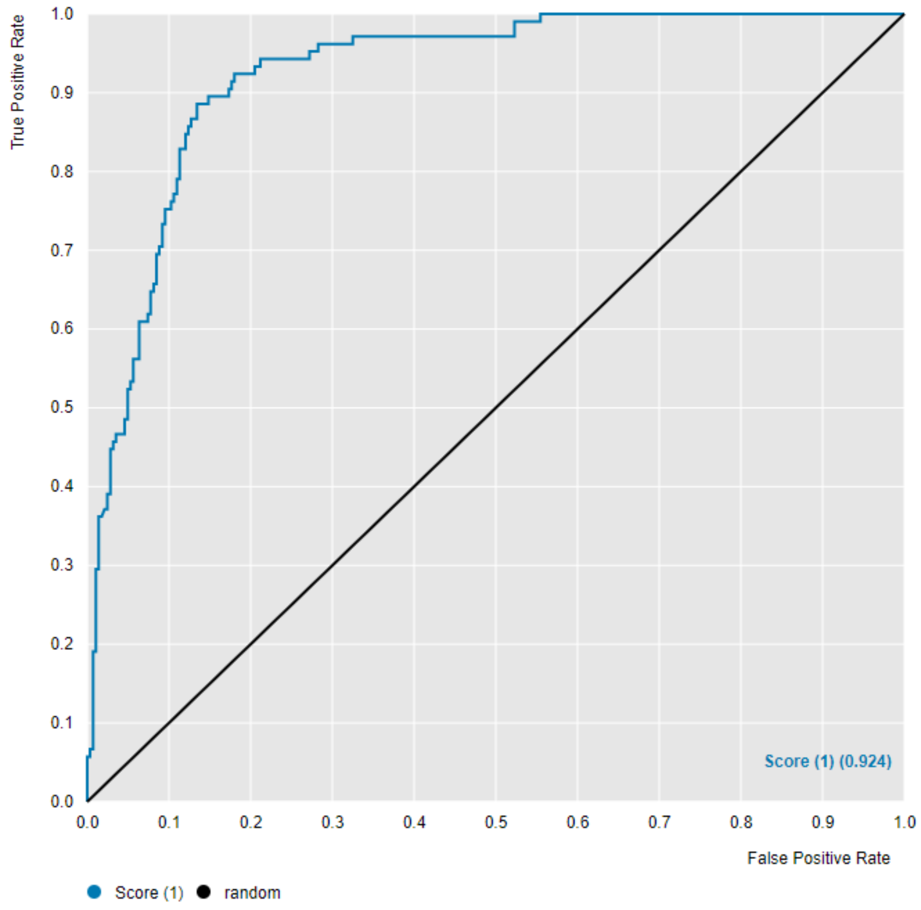

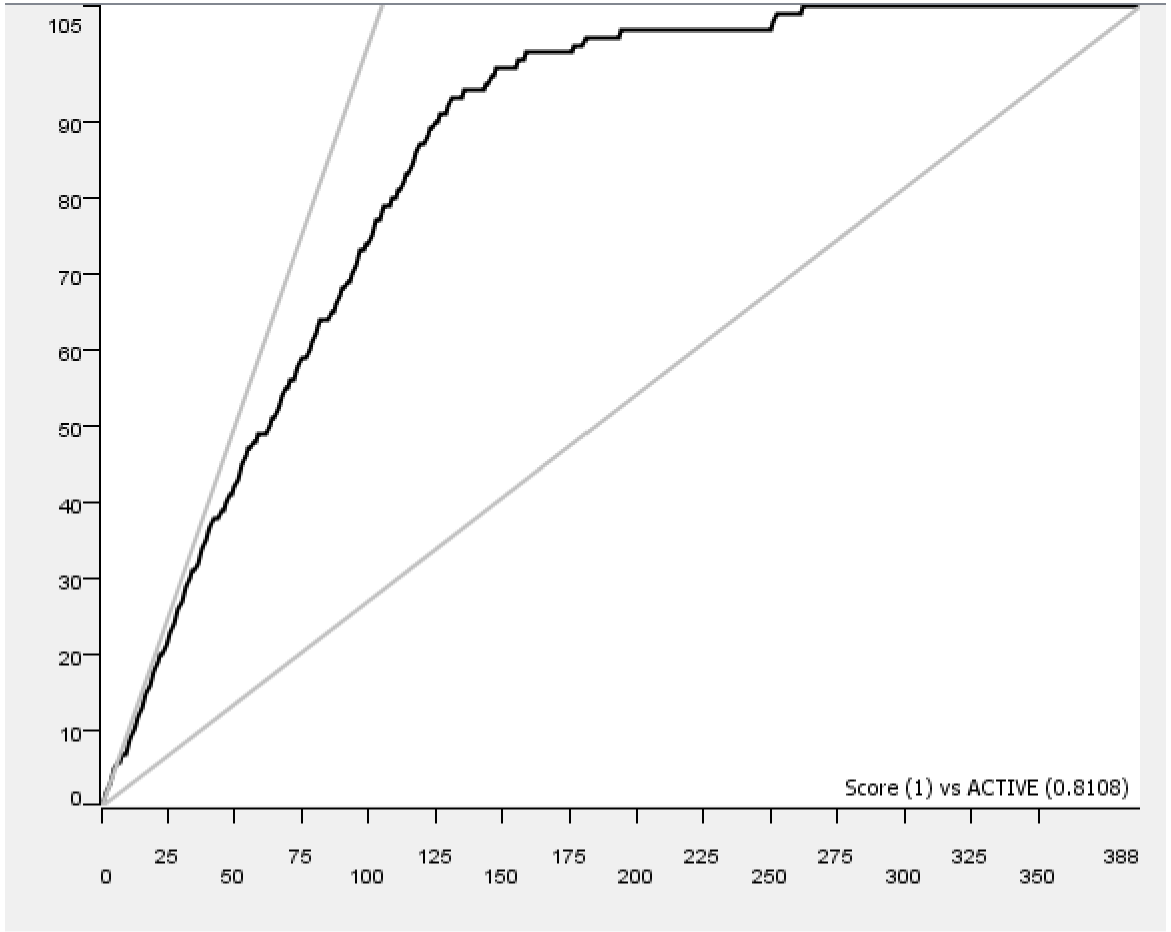

N=388

Top 10% Mean IC50	13.2 nM

	Predicted Active	Predicted Inactive
Active	94	11
Inactive	44	239

<20nM DefGood in PARP1, 30% error; Random seed = 121783

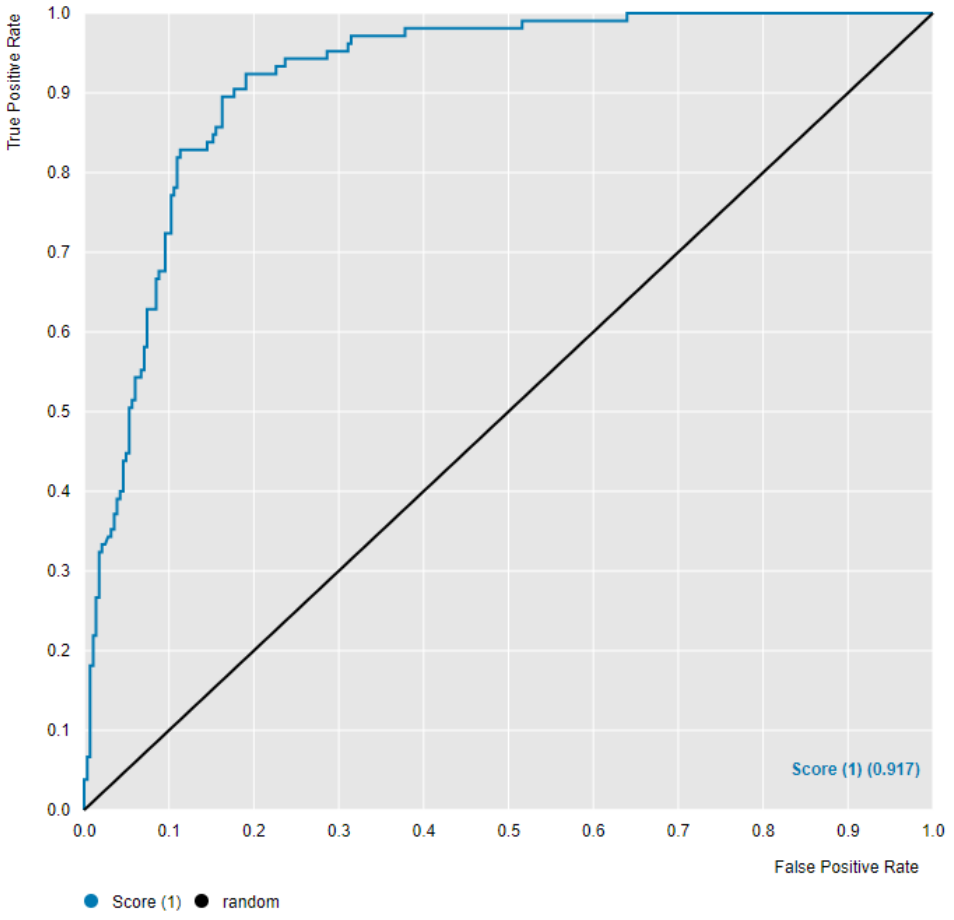

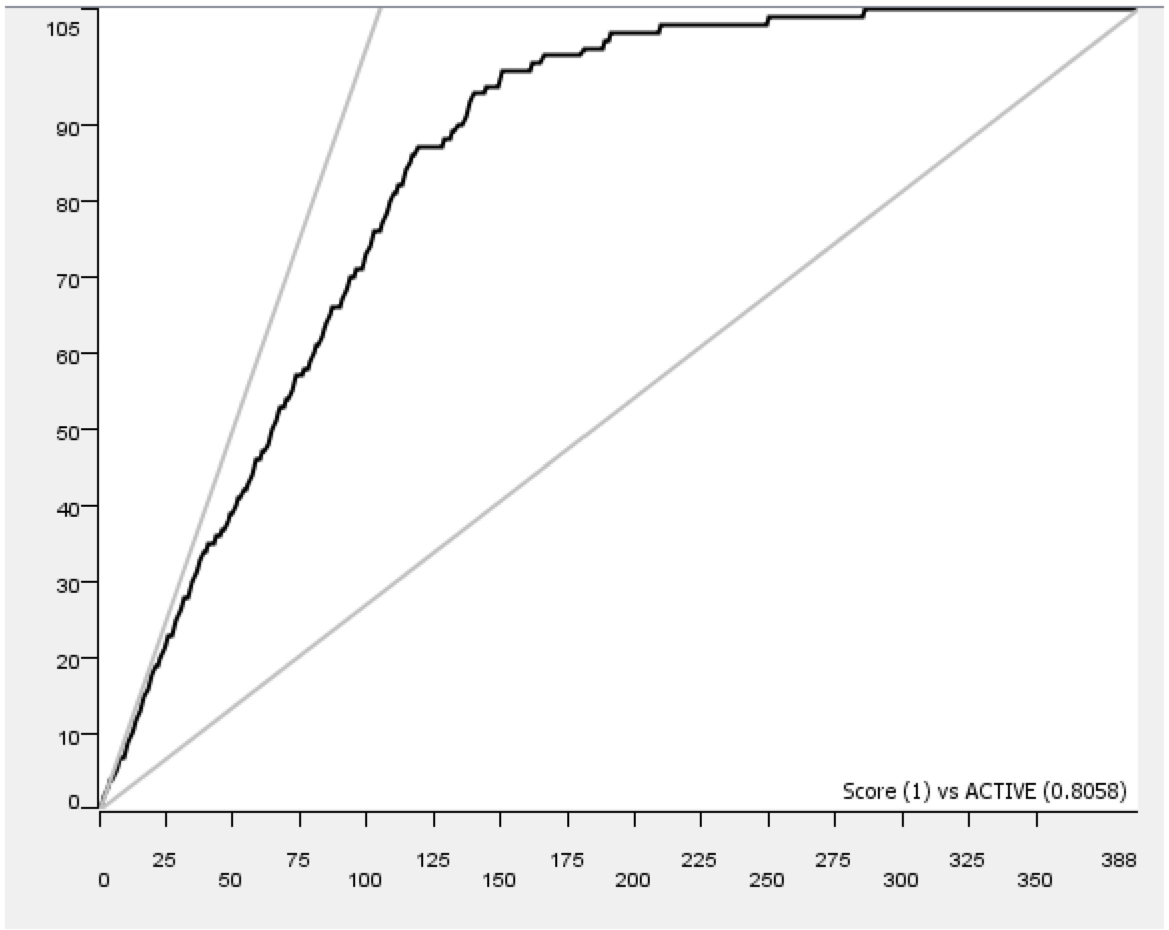

N=388

Top 10% Mean IC50	9.4 nM

	Predicted Active	Predicted Inactive
Active	87	18
Inactive	36	247

<20nM DefGood in PARP1, 40% error; Random seed = 121783

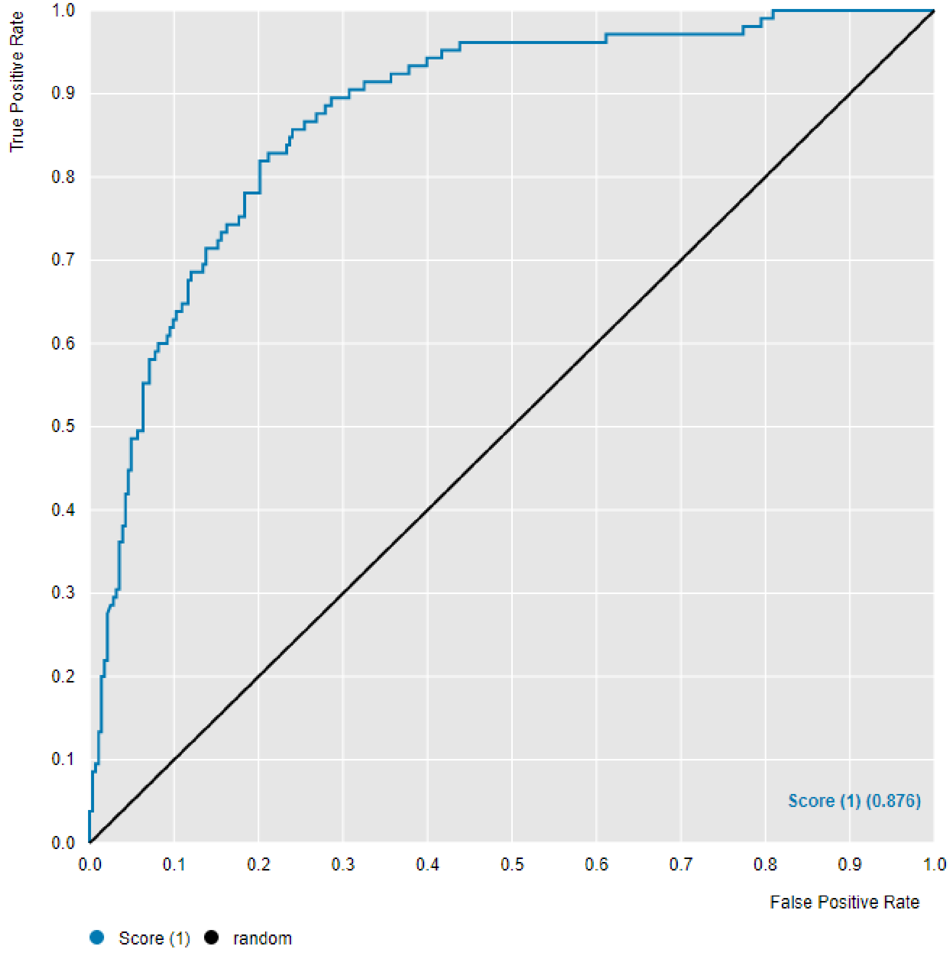

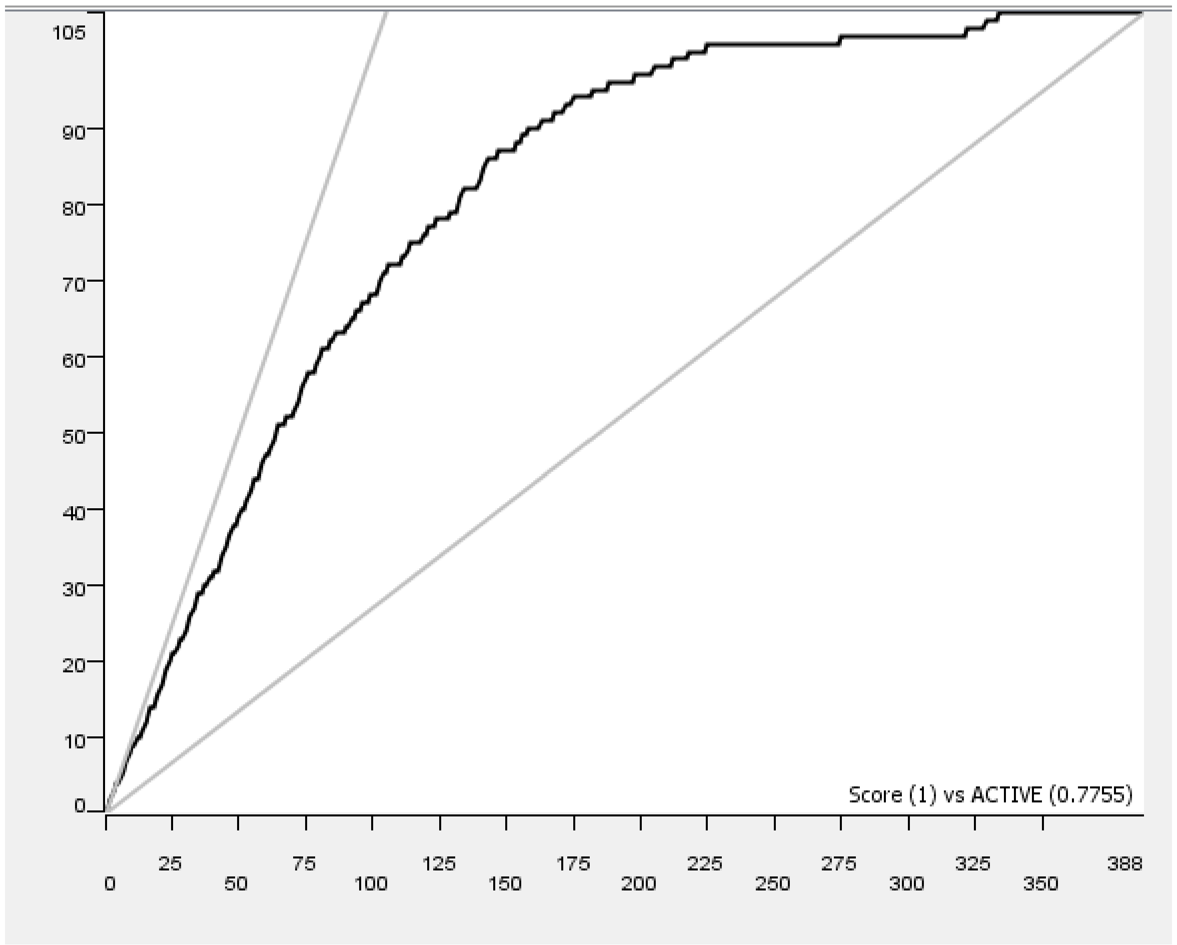

N=388

**Top 10% Mean IC50**	**280 nM**

	Predicted Active	Predicted Inactive
Active	93	12
Inactive	79	204

<20nM DefGood in PARP1, 45% error; Random seed = 121783

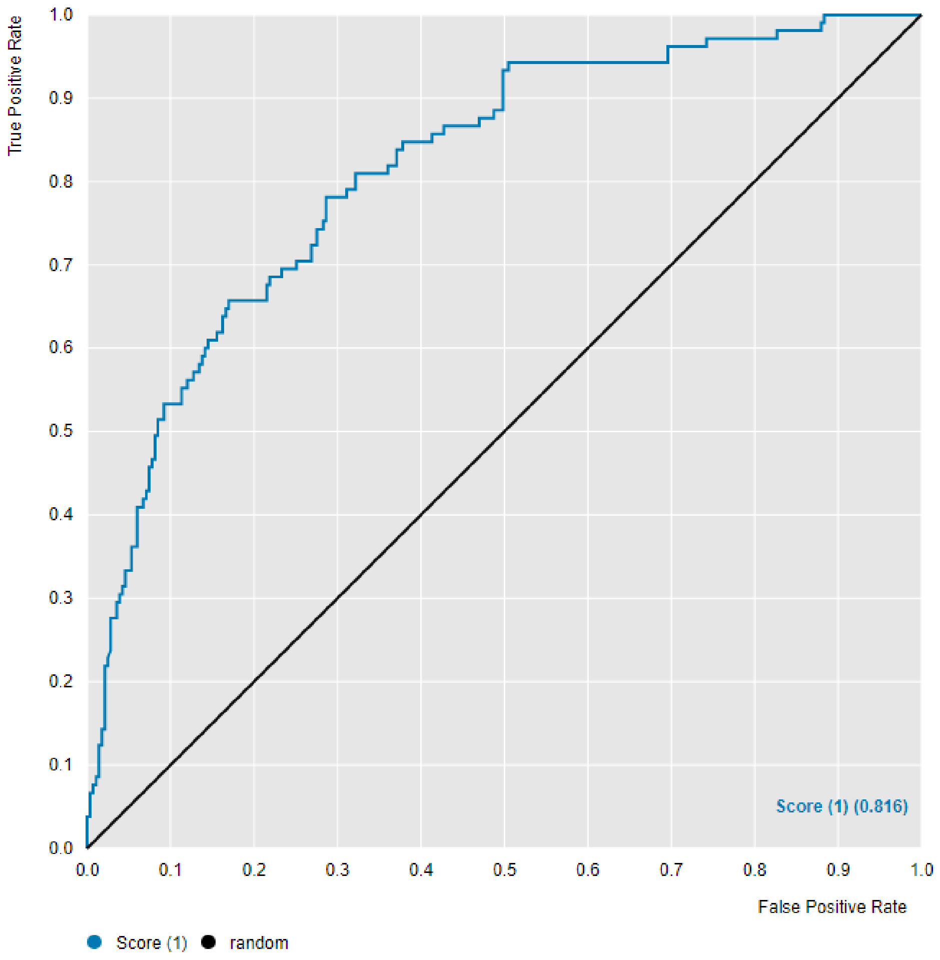

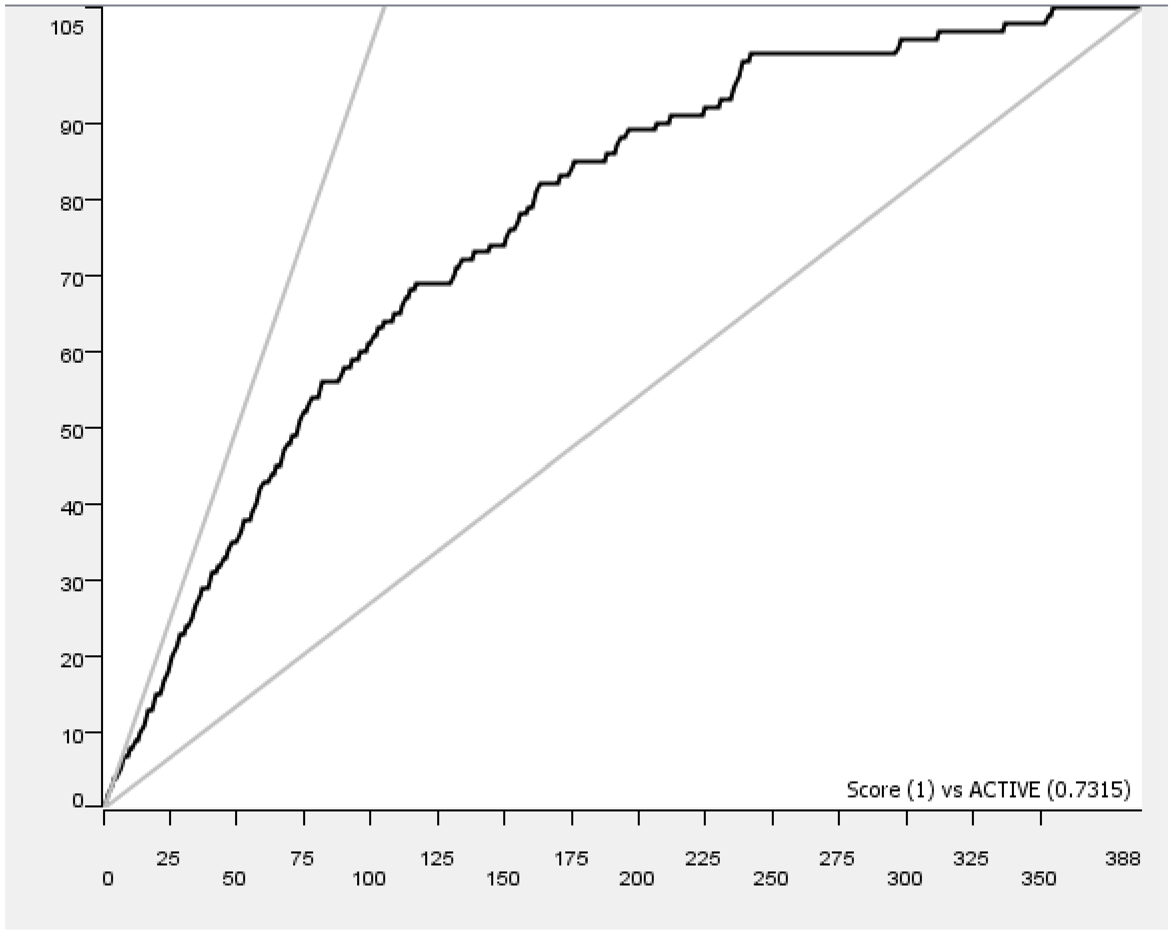

N=388

Top 10% Mean IC50	21.4 nM

	Predicted Active	Predicted Inactive
Active	99	6
Inactive	174	109

<20nM DefGood in PARP1, 50% error; Random seed = 121783

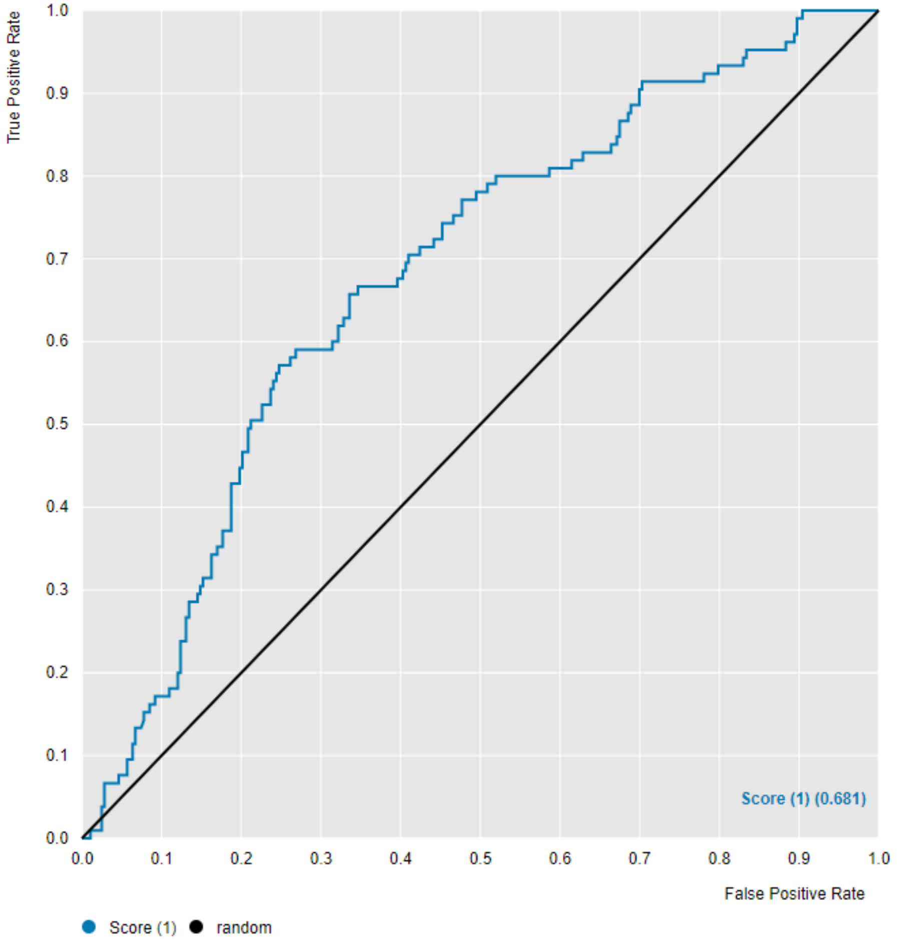

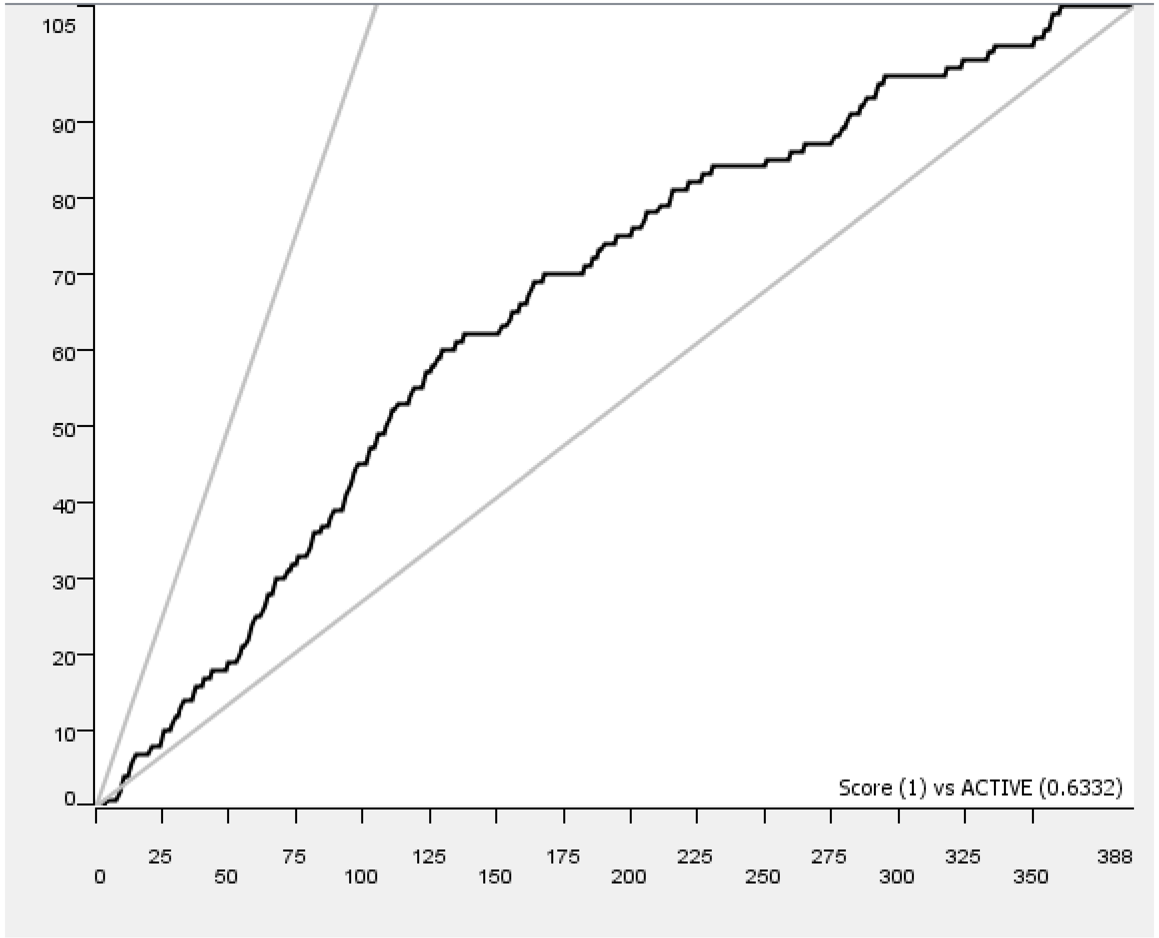

N=388

**Top 10% Mean IC50**	**4,700 nM**

	Predicted Active	Predicted Inactive
Active	105	0
Inactive	281	2

Conclusion - NBN

- An NBN could be generated for PARP1 kinase with a decision value of <20 nM as defined as active. The predictivity of the algorithm could be preserved in three random train/test cycles. The point of failure was 5%, 45% and 50% error.
- The 5% failure point had one mistake ranked at the bottom of the top 10% which was >300,000 nM.

RF Error Tolerance- <20 nM DefGood in PARP1

- 0-50% absolute error

RF- <20nM DefGood in PARP1, 10% error

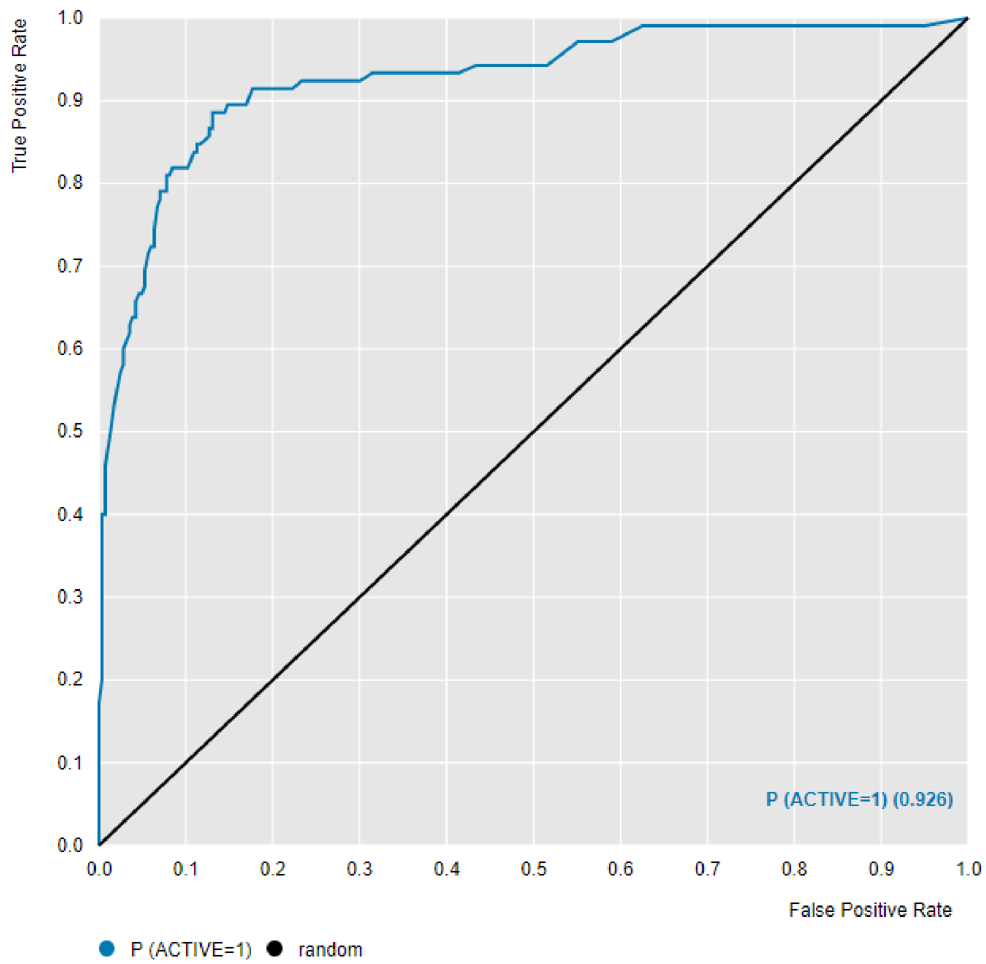

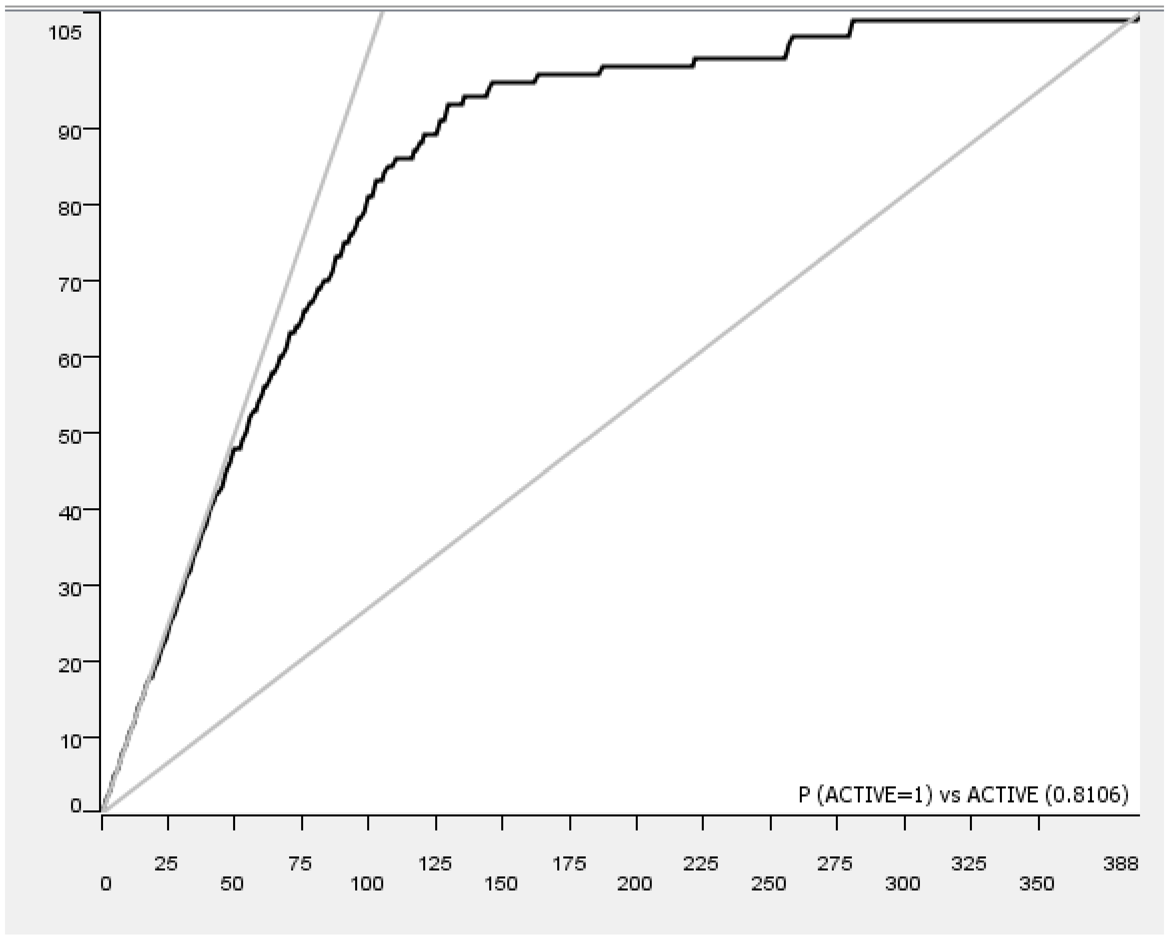

N=388

**Top 10% Mean IC50**	**7.3 nM**

	Predicted Active	Predicted Inactive
Active	86	19
Inactive	26	257

RF- <20nM DefGood in PARP1, 20% error

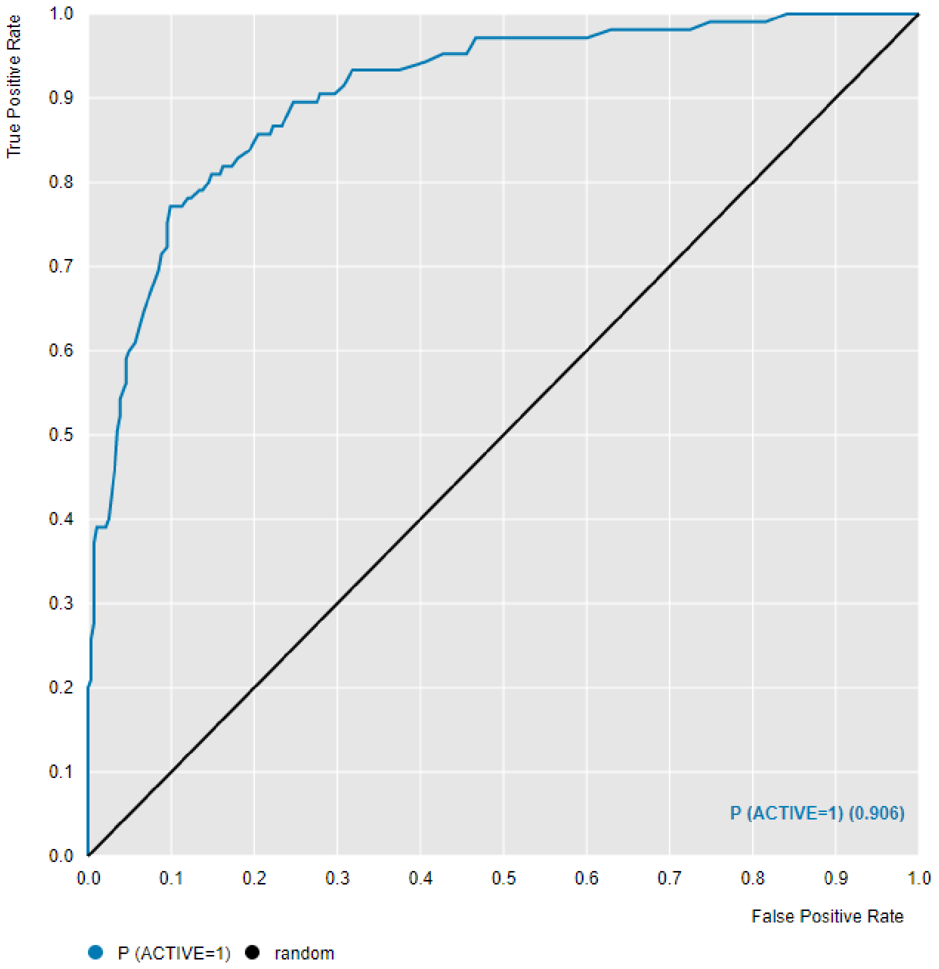

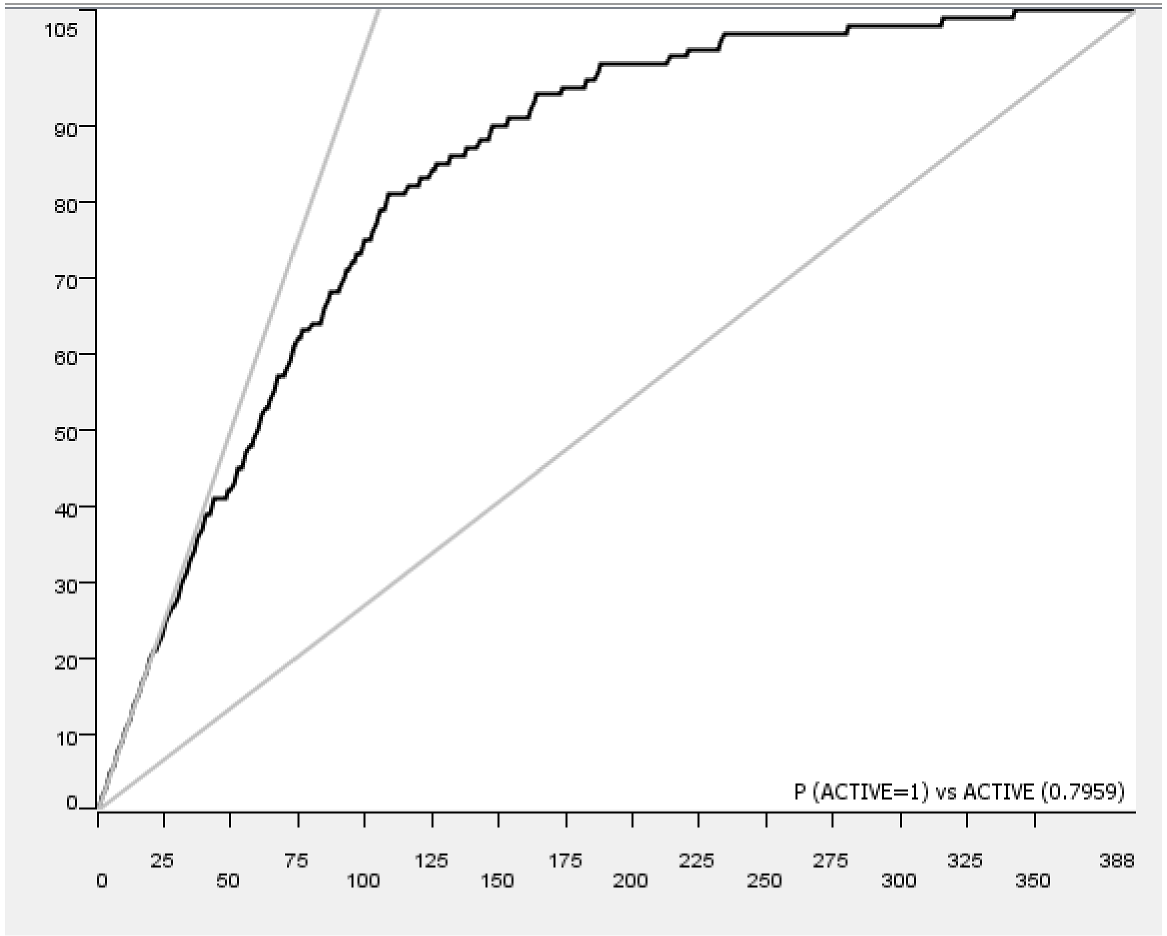

N=388

**Top 10% Mean IC50**	**18.1 nM**

	Predicted Active	Predicted Inactive
Active	83	22
Inactive	38	245

RF- <20nM DefGood in PARP1, 25% error

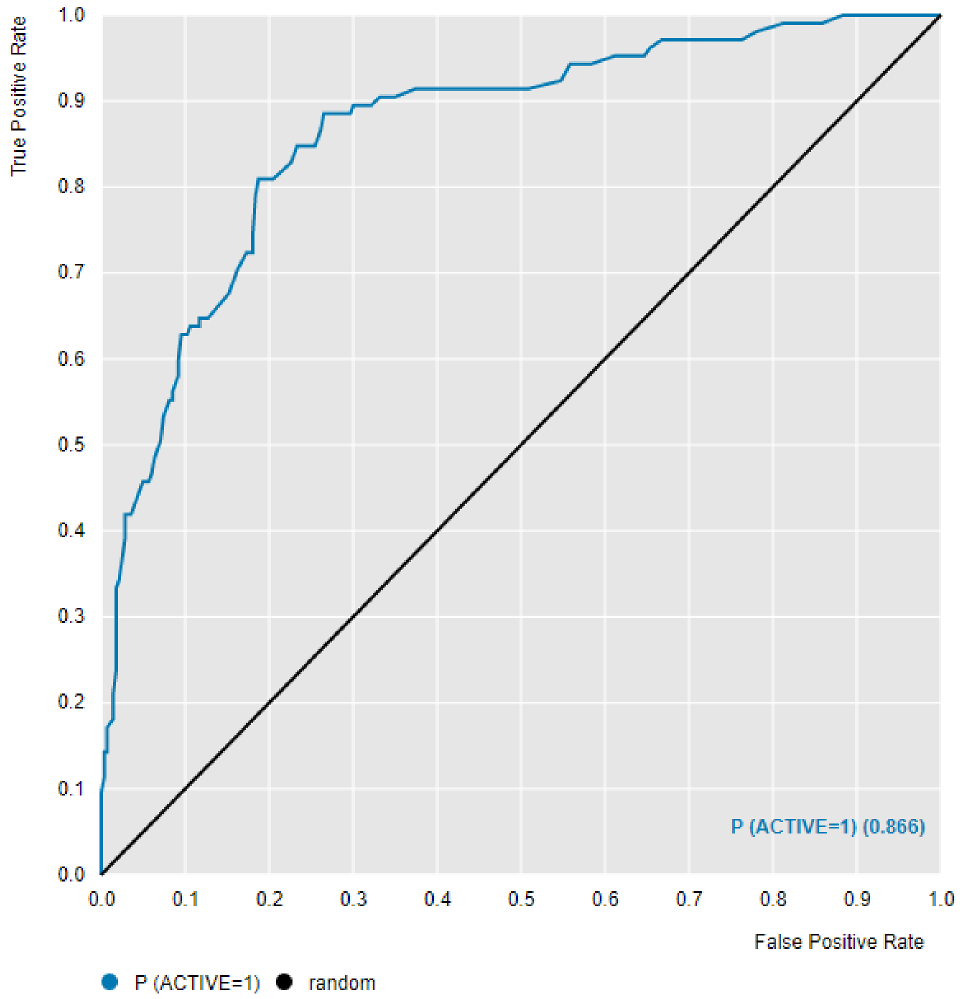

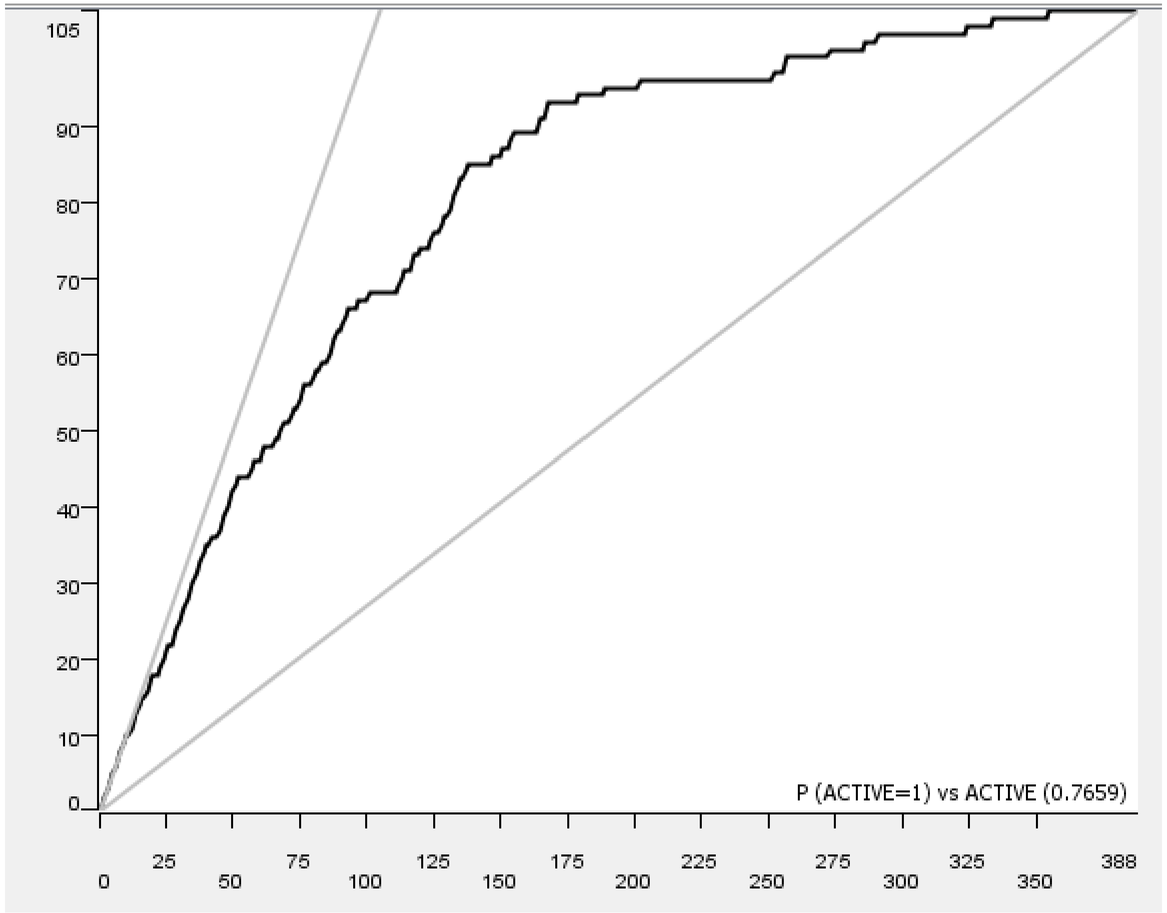

N=388

Top 10% Mean IC50	282 nM

	Predicted Active	Predicted Inactive
Active	74	31
Inactive	46	237

RF- <20nM DefGood in PARP1, 30% error

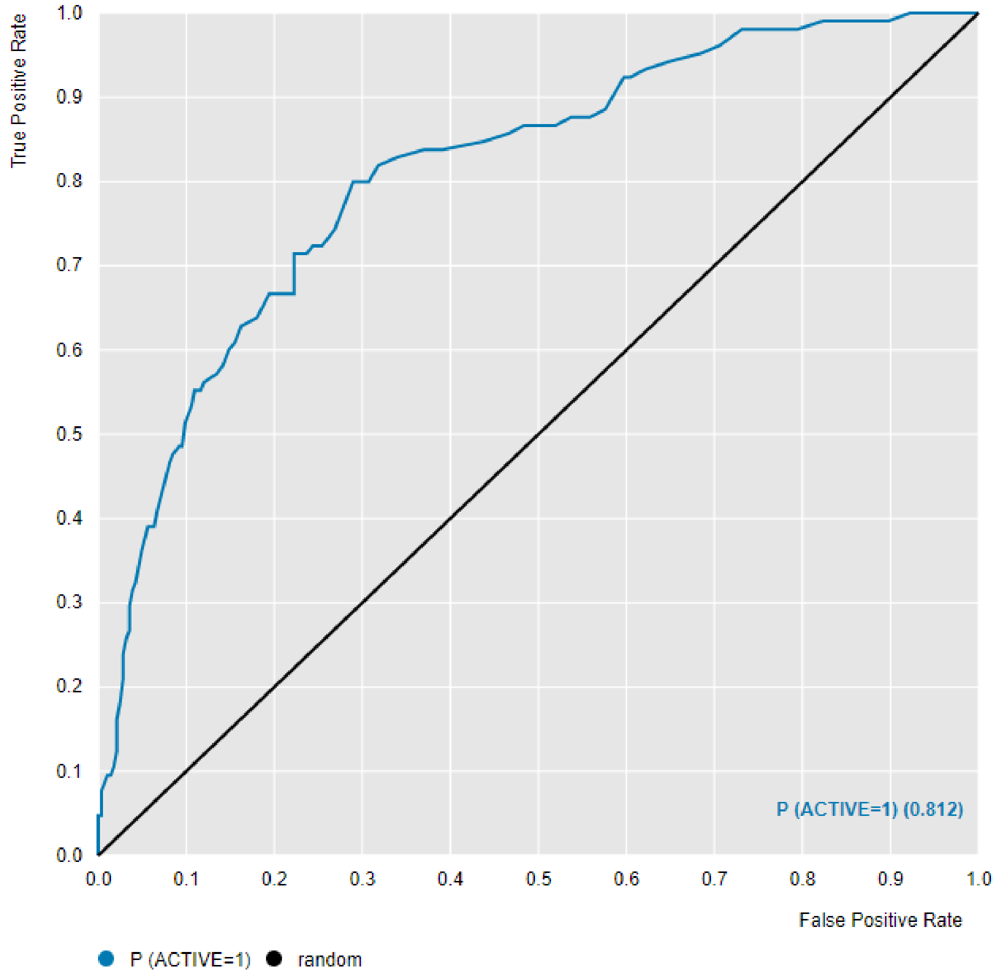

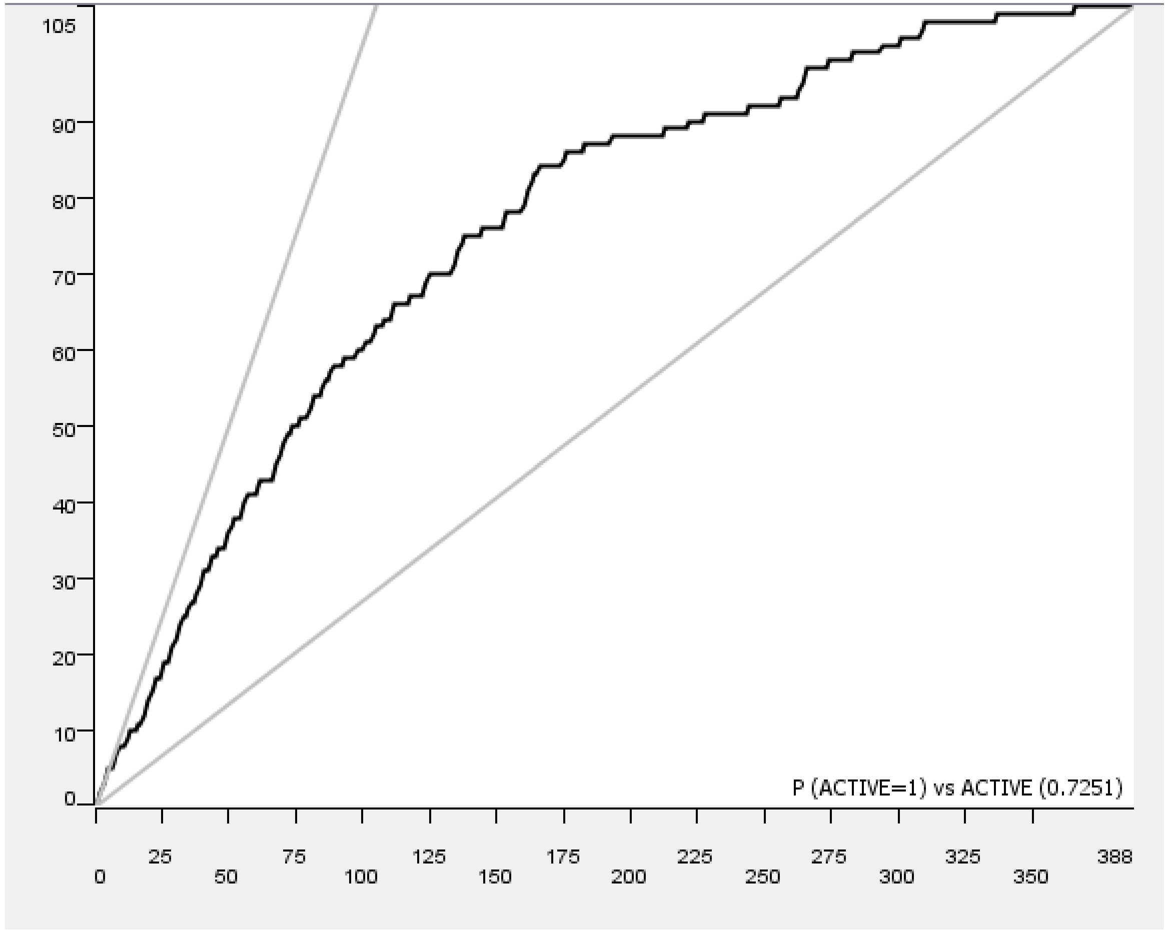

N=388

**Top 10% Mean IC50**	**546 nM**

	Predicted Active	Predicted Inactive
Active	70	35
Inactive	55	228

RF- <20nM DefGood in PARP1, 35% error

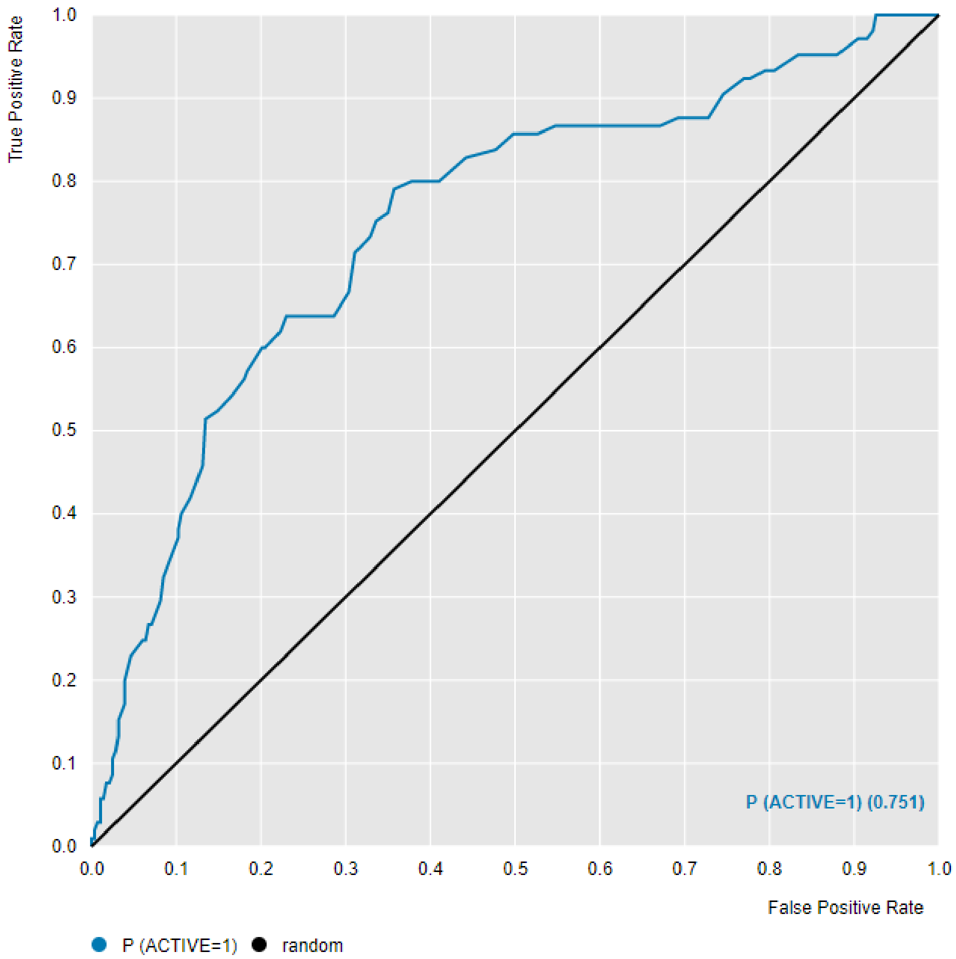

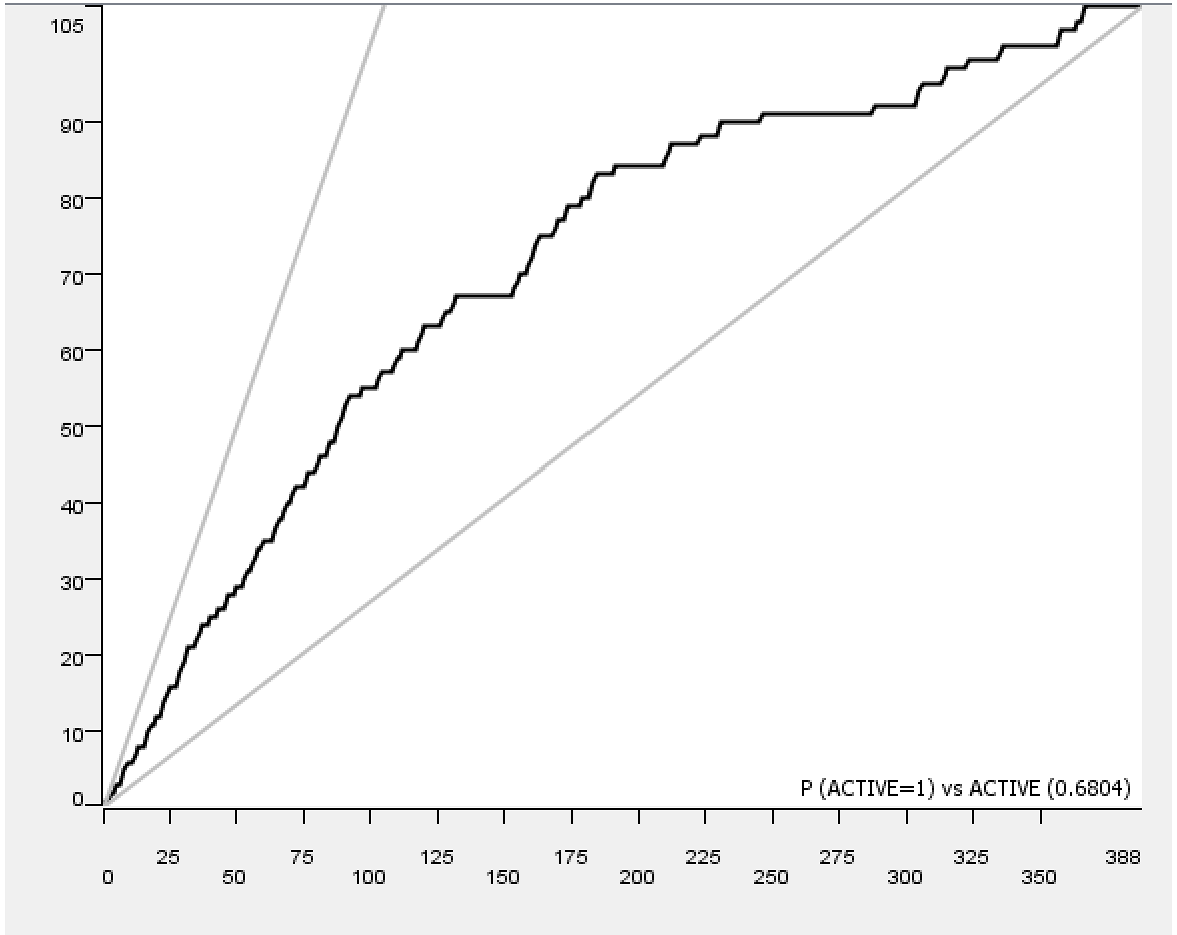

N=388

**Top 10% Mean IC50**	**631 nM**

	Predicted Active	Predicted Inactive
Active	63	42
Inactive	58	225

RF- <20nM DefGood in PARP1, 40% error

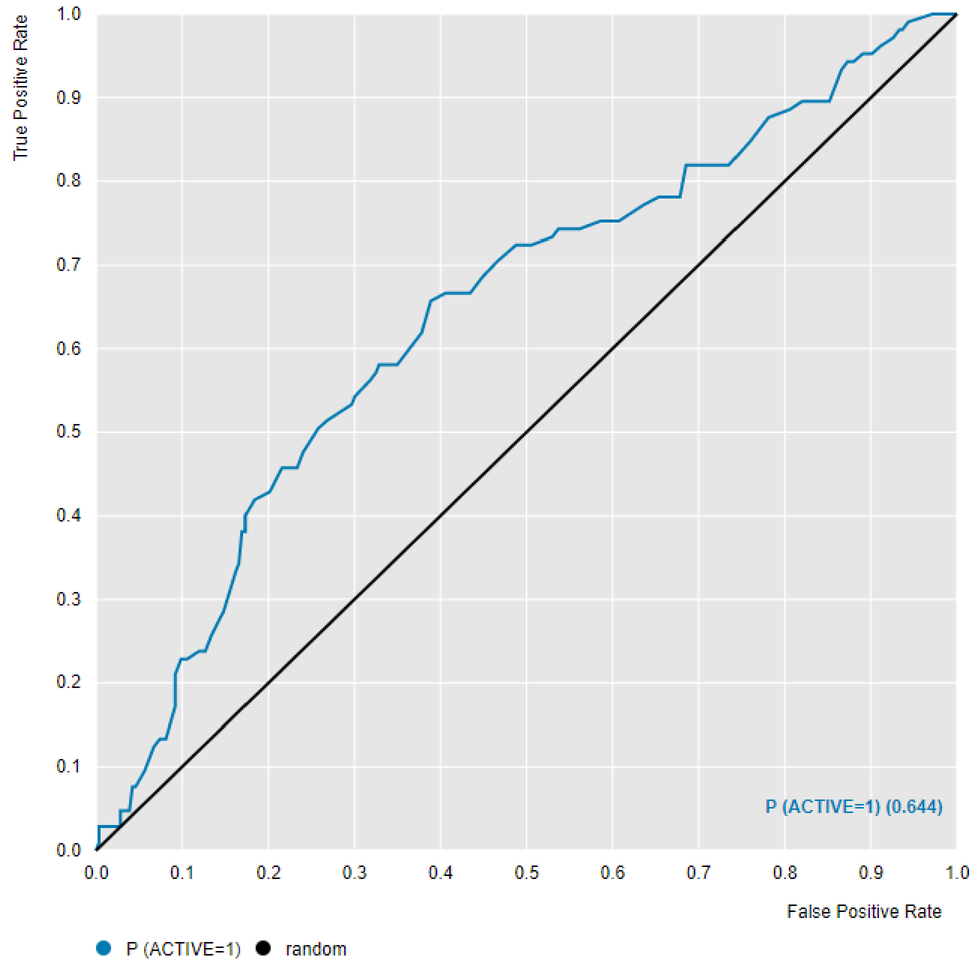

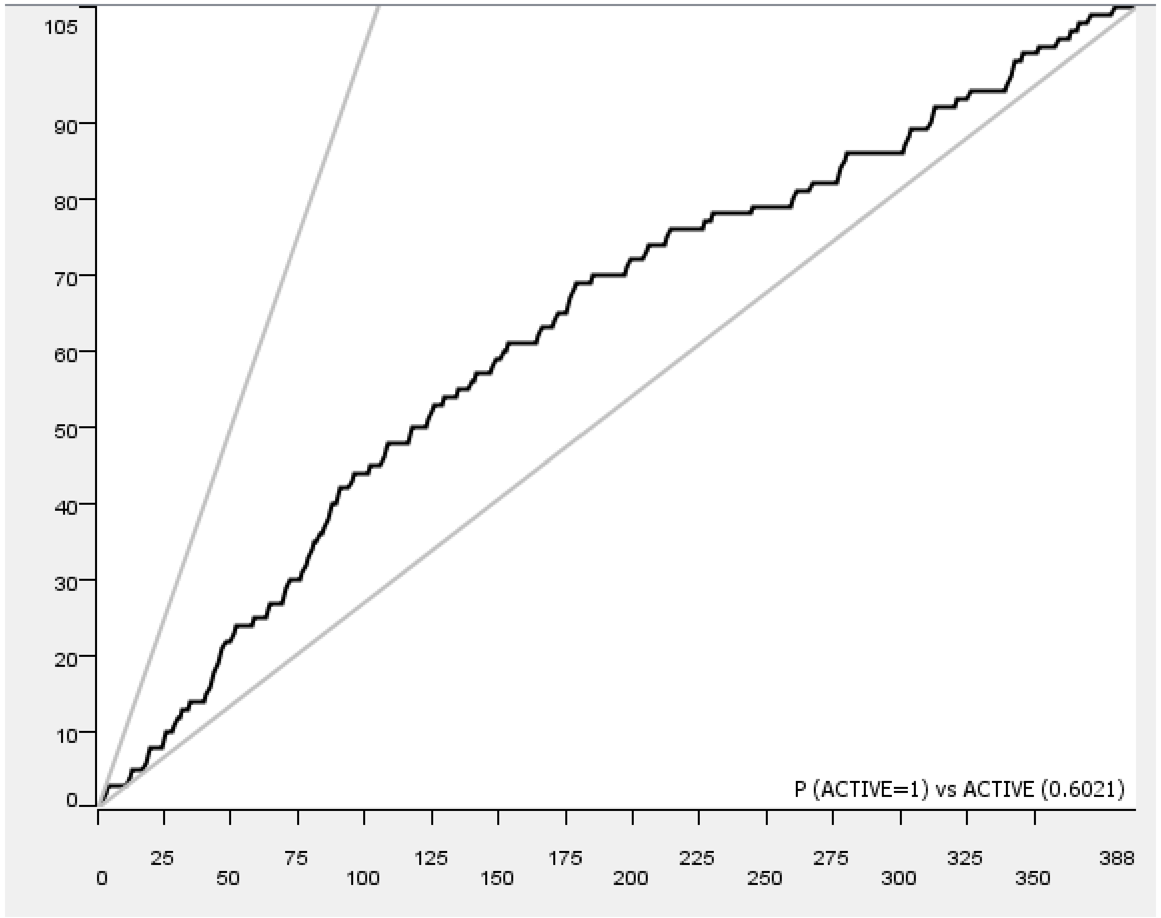

N=388

Top 10% Mean IC50	3,600 nM

	Predicted Active	Predicted Inactive
Active	56	49
Inactive	84	199

<20nM DefGood in PARP1, 10% error; Random seed = 429

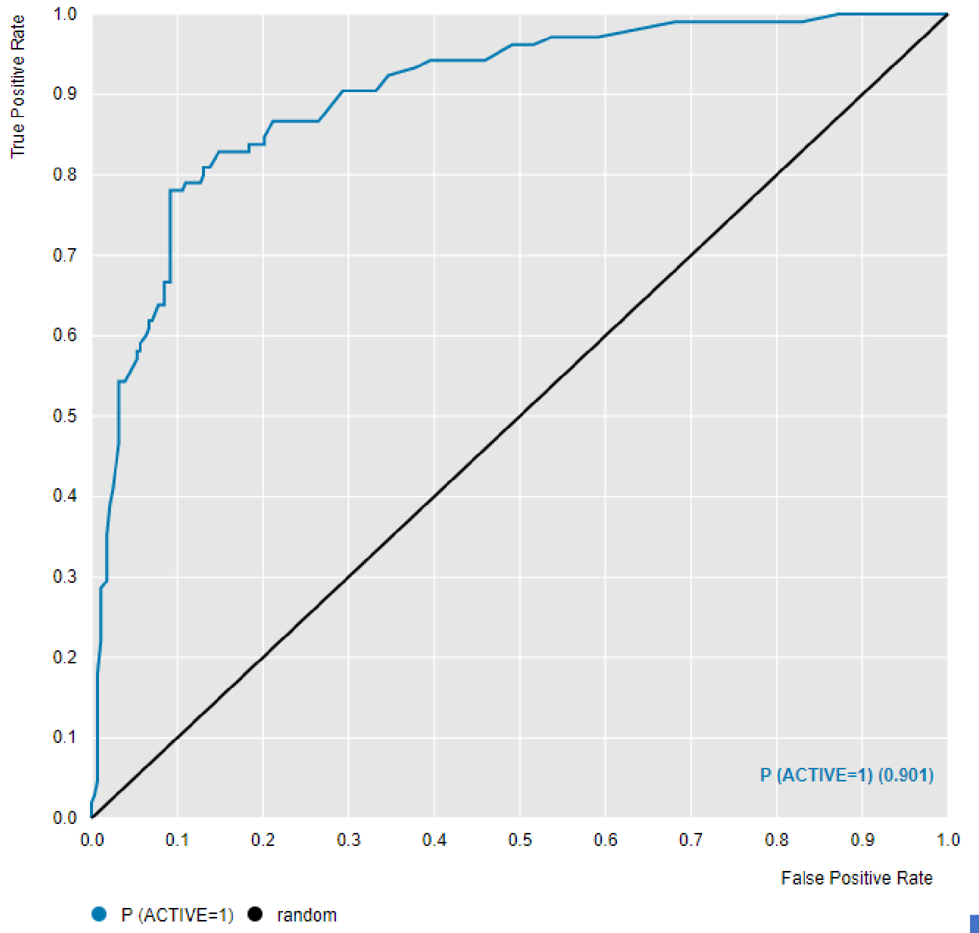

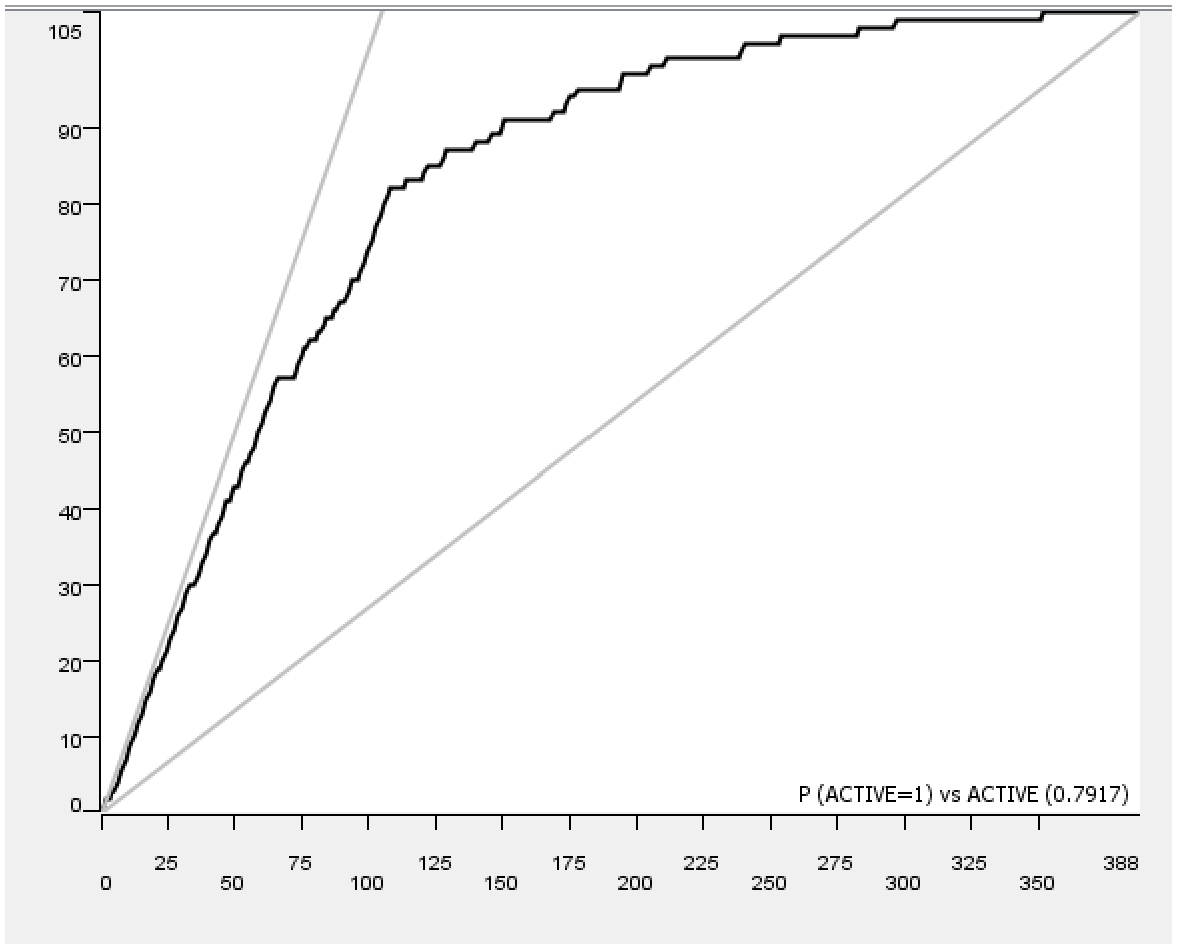

N=388

Top 10% Mean IC50	12.1 nM

	Predicted Active	Predicted Inactive
Active	75	30
Inactive	26	257

<20nM DefGood in PARP1, 20% error; Random seed = 429

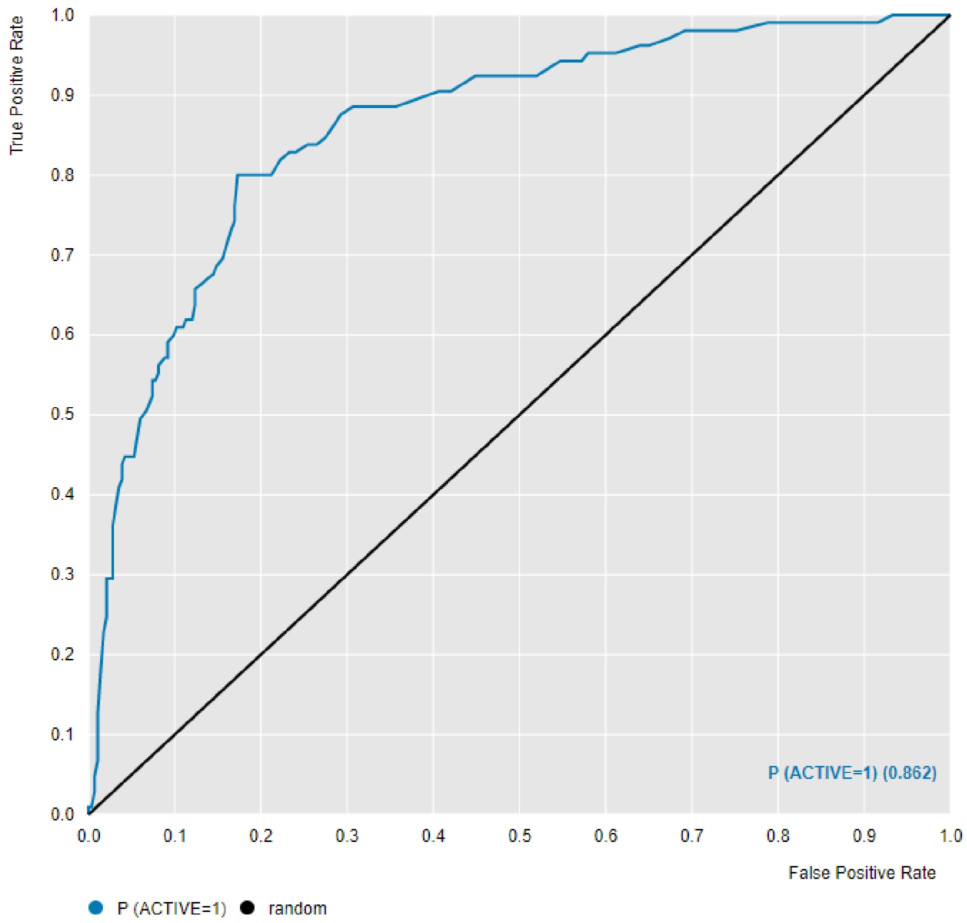

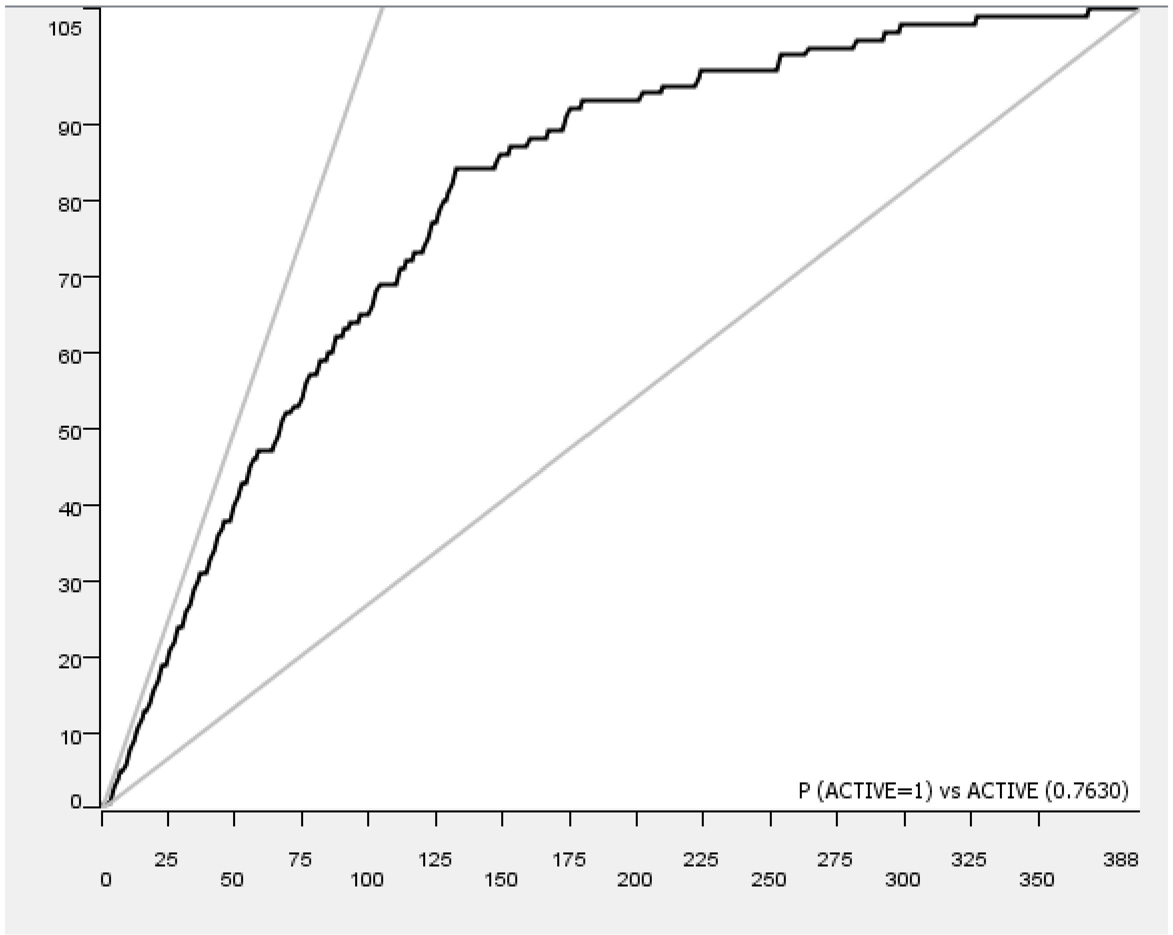

N=388

Top 10% Mean IC50	109 nM

	Predicted Active	Predicted Inactive
Active	69	36
Inactive	35	248

<20nM DefGood in PARP1, 25% error; Random seed = 429

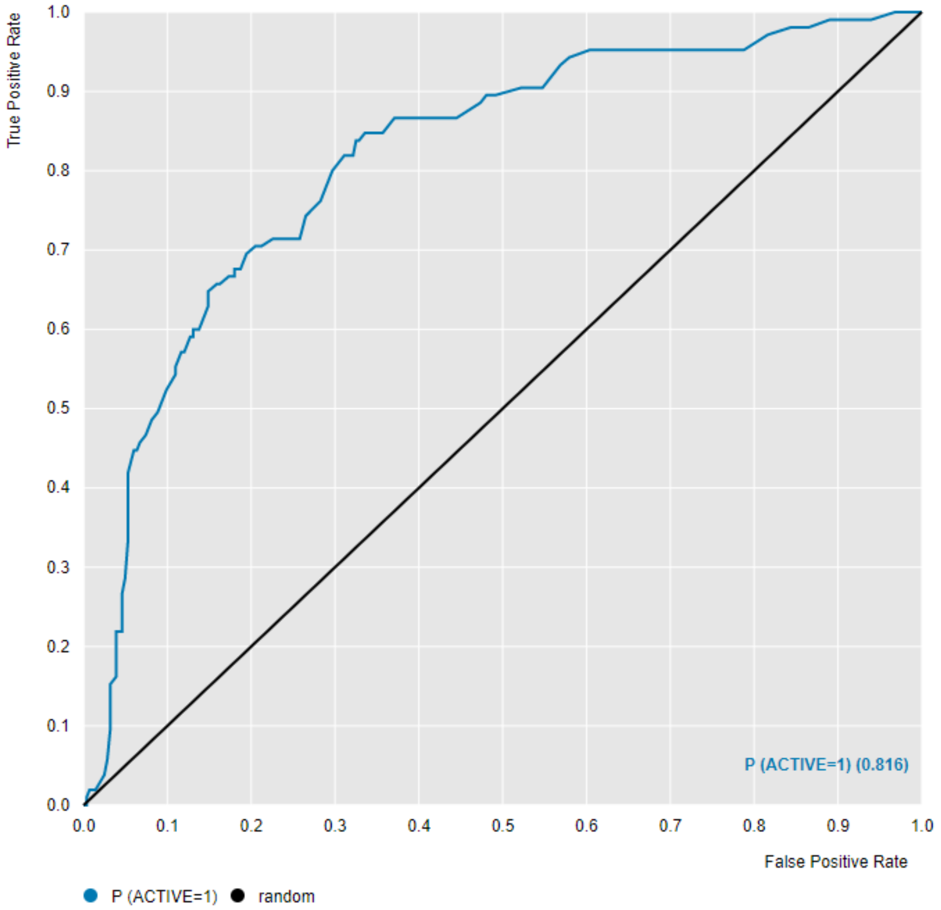

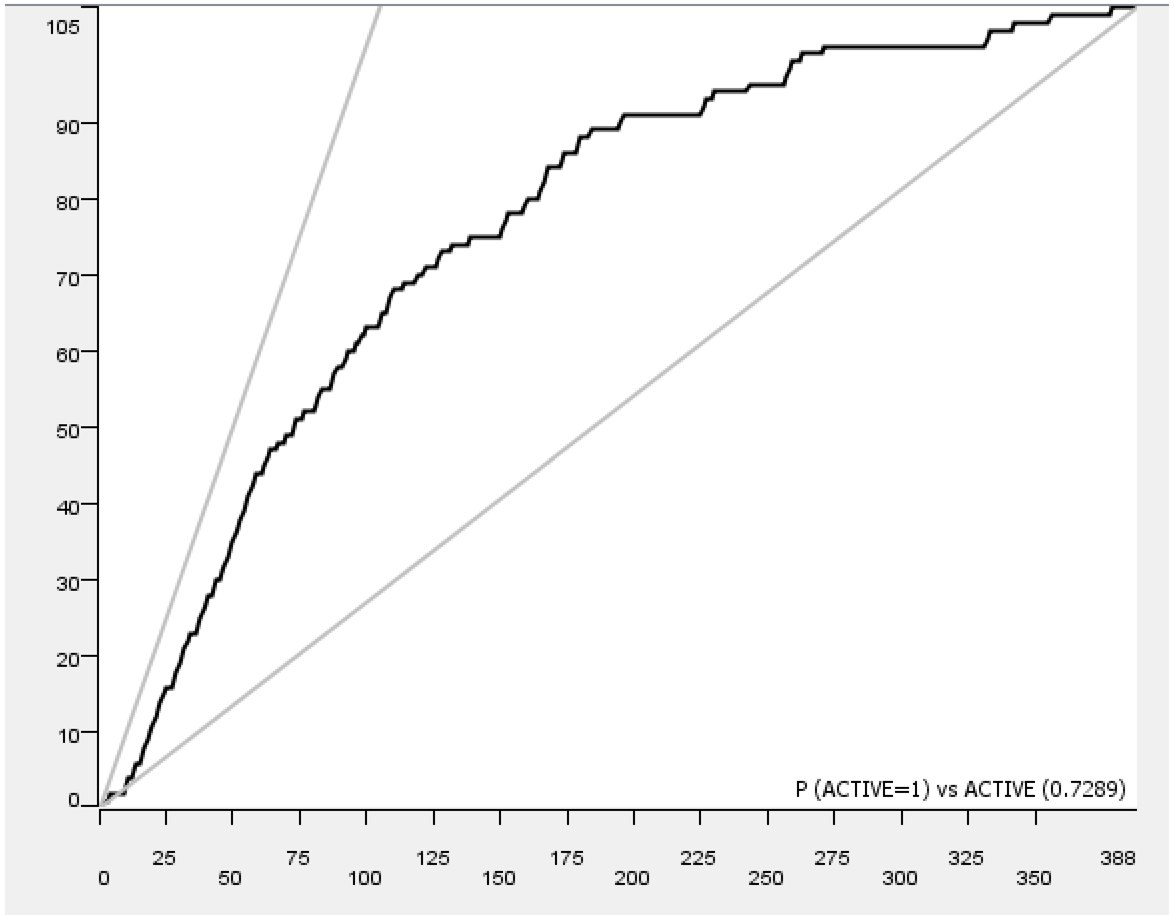

N=388

Top 10% Mean IC50	2,700 nM

	Predicted Active	Predicted Inactive
Active	70	35
Inactive	51	232

<20nM DefGood in PARP1, 30% error; Random seed = 429

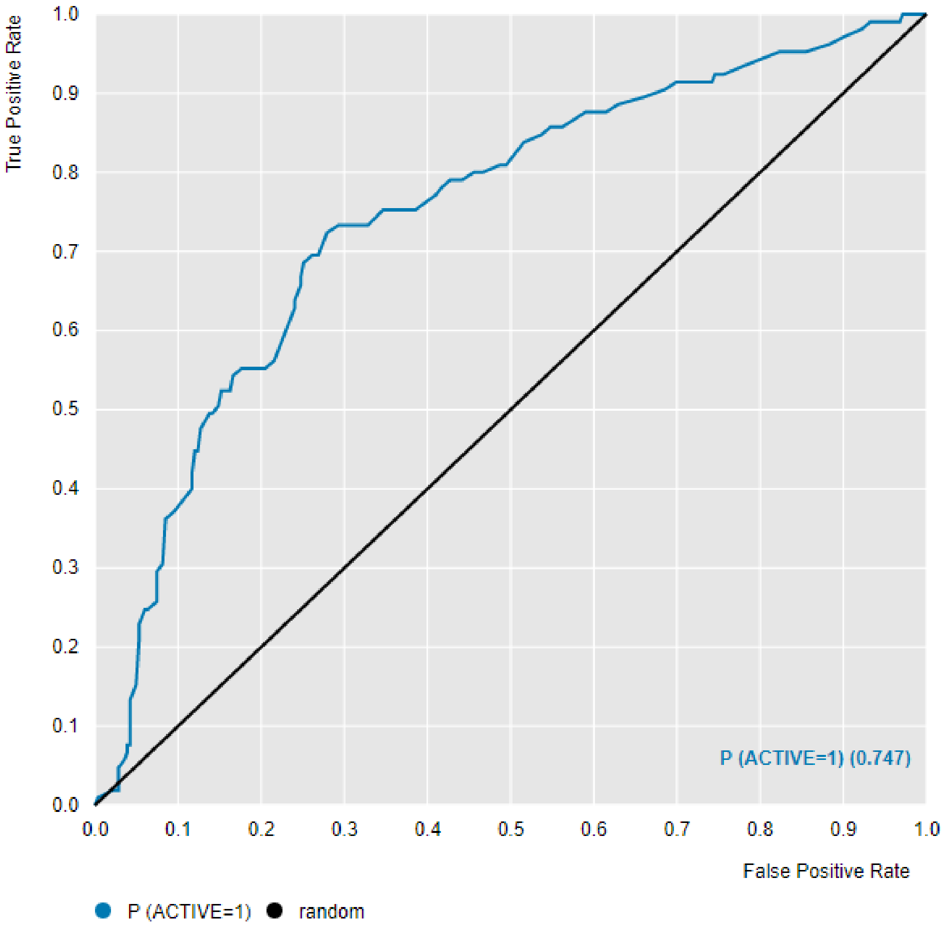

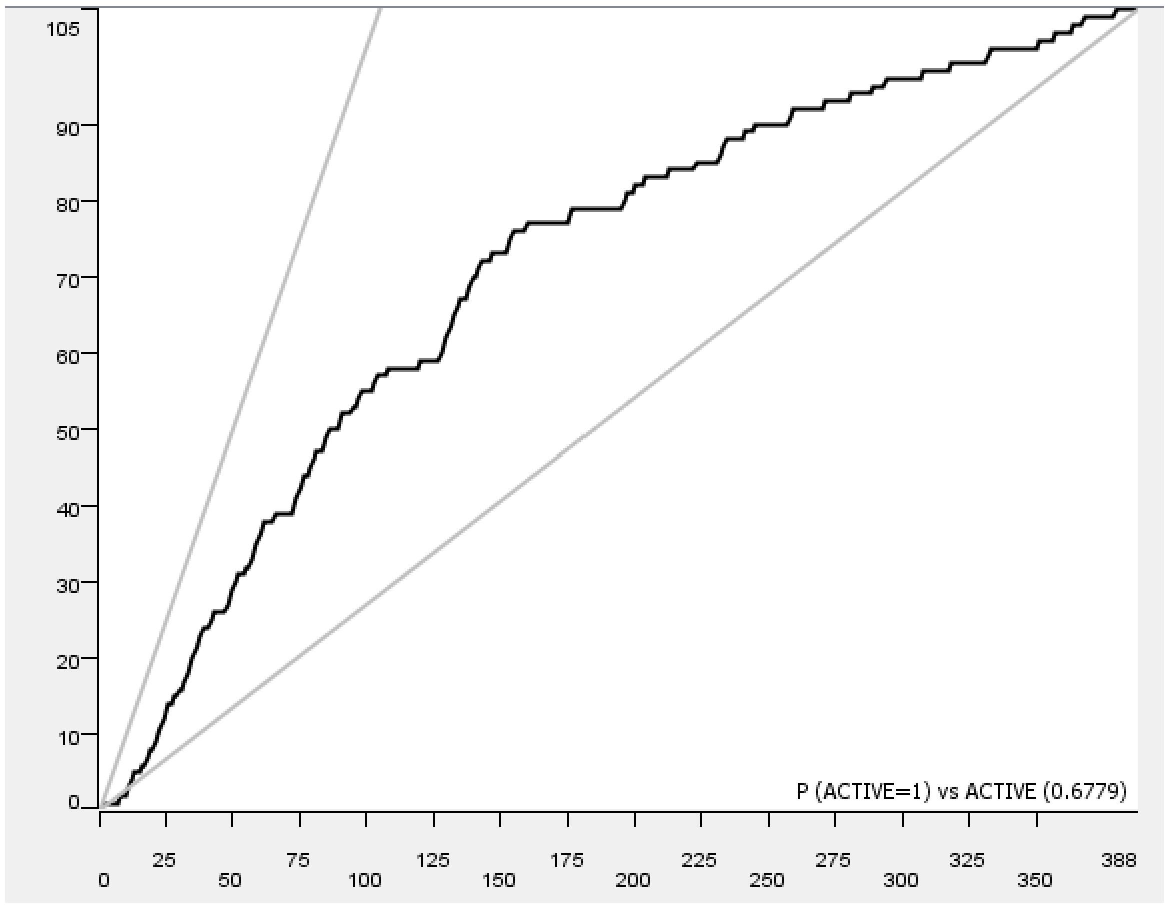

N=388

Top 10% Mean IC50	3,200 nM

	Predicted Active	Predicted Inactive
Active	66	39
Inactive	68	215

<20nM DefGood in PARP1, 10% error; Random seed = 121783

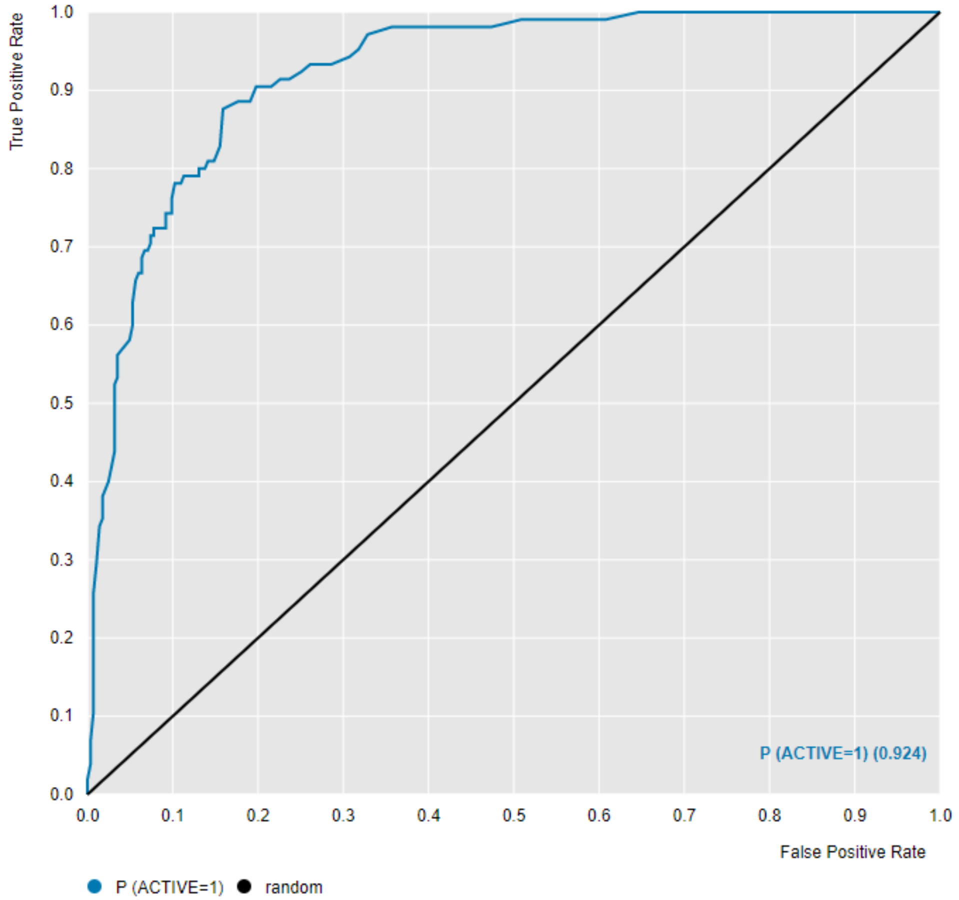

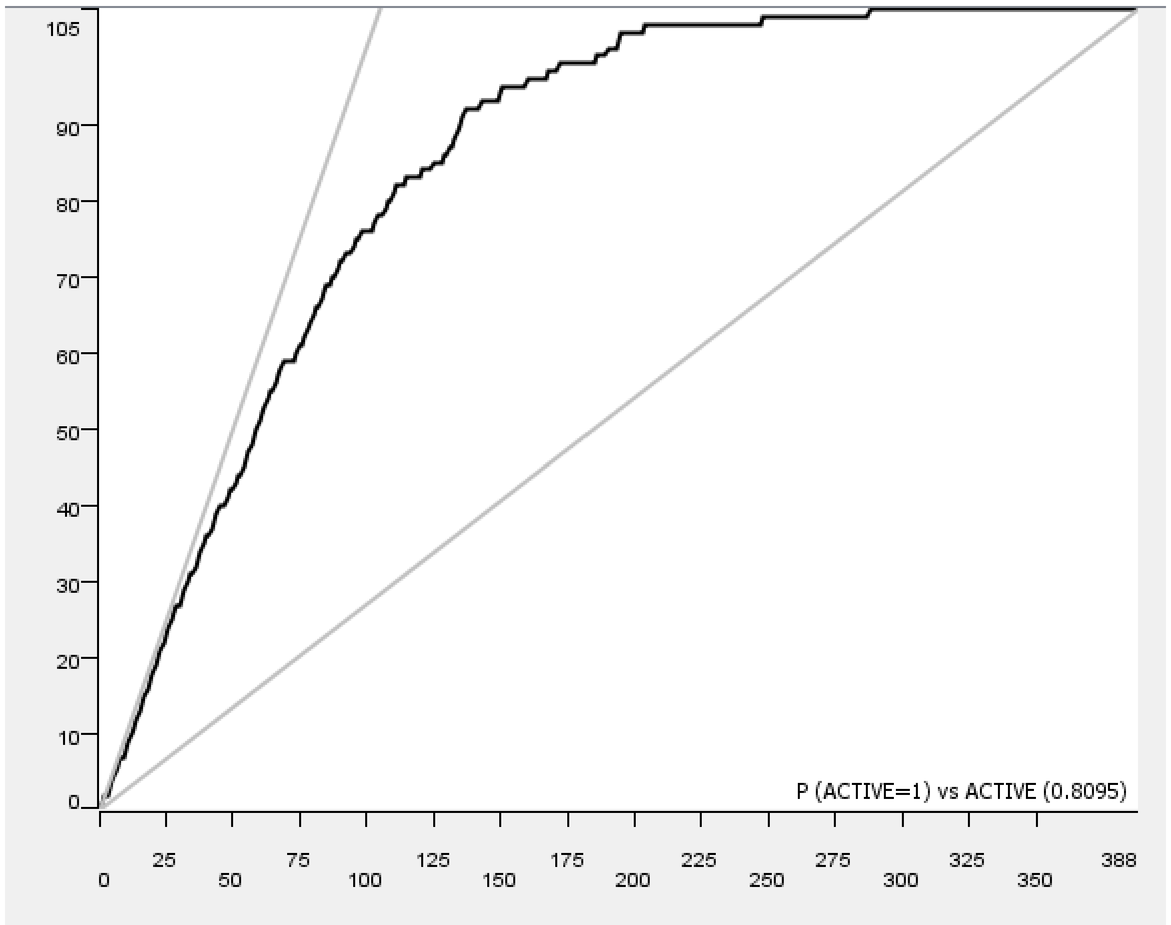

N=388

Top 10% Mean IC50	11.9 nM

	Predicted Active	Predicted Inactive
Active	78	27
Inactive	27	256

<20nM DefGood in PARP1, 20% error; Random seed = 121783

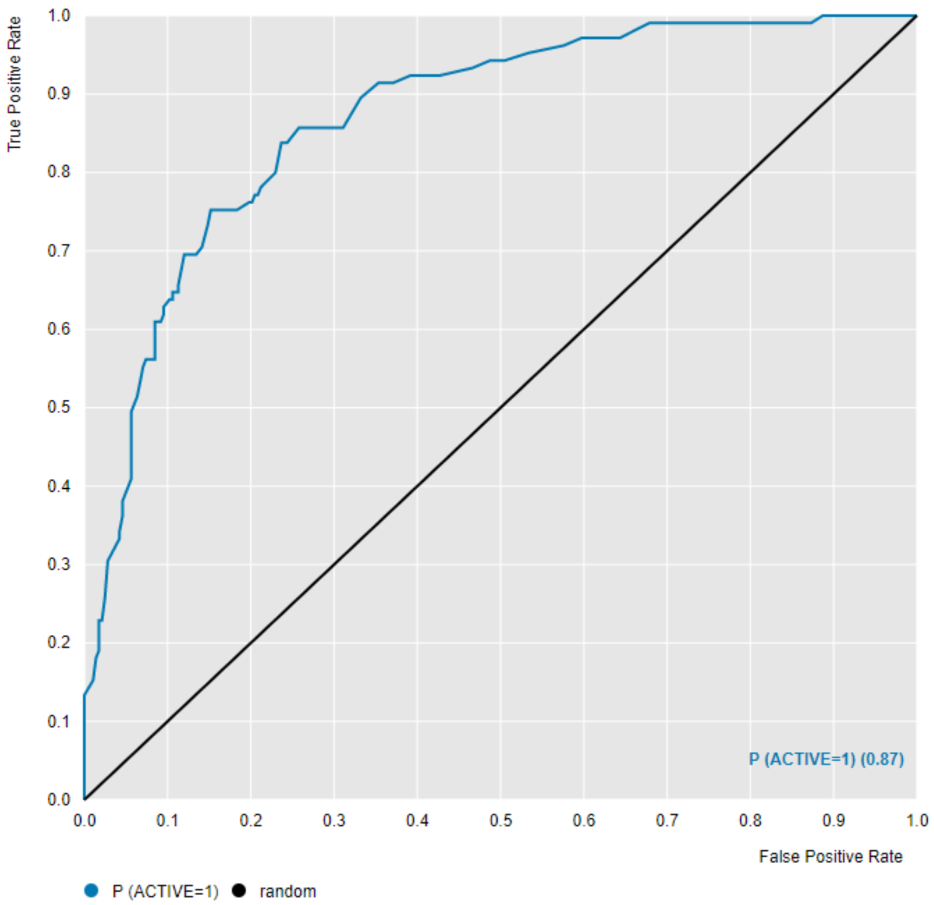

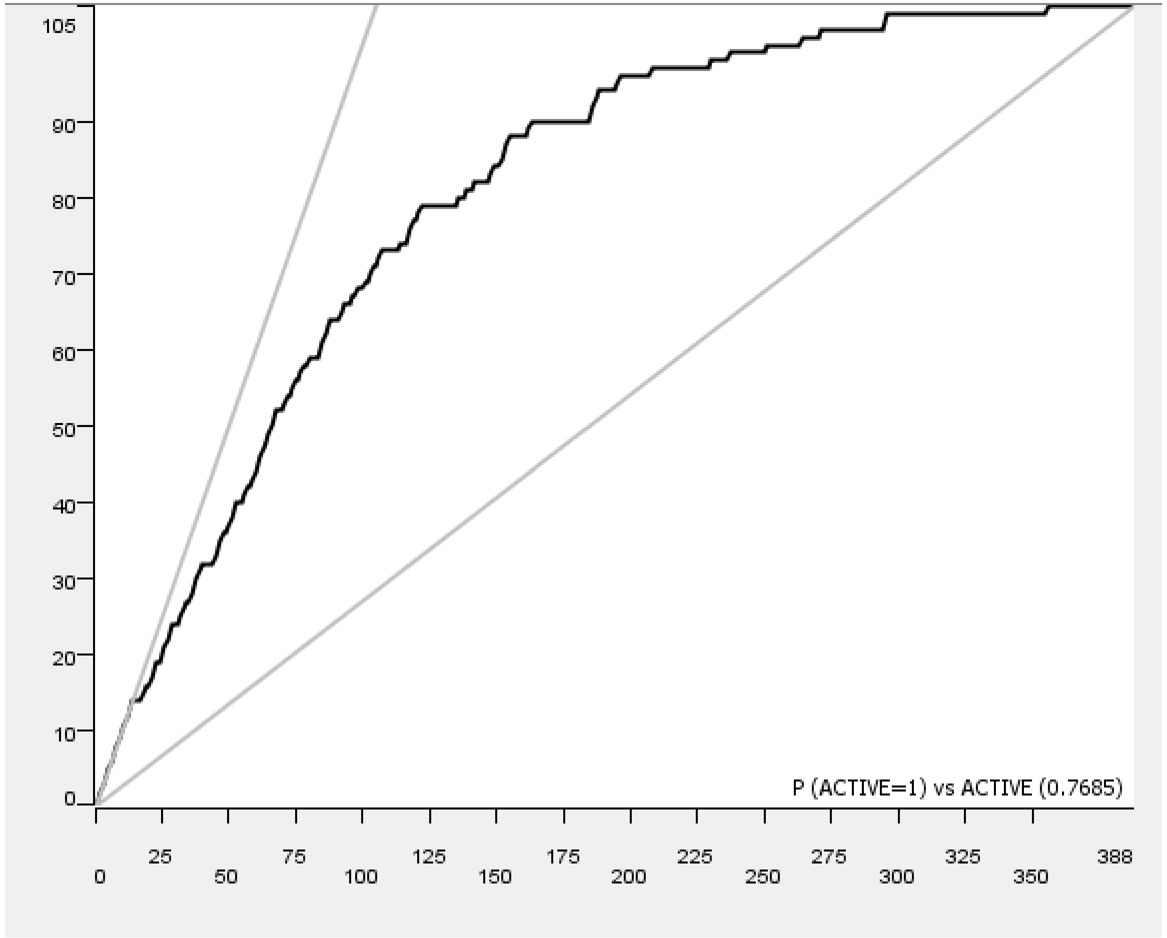

N=388

Top 10% Mean IC50	16.9 nM

	Predicted Active	Predicted Inactive
Active	73	32
Inactive	35	248

<20nM DefGood in PARP1, 25% error; Random seed = 121783

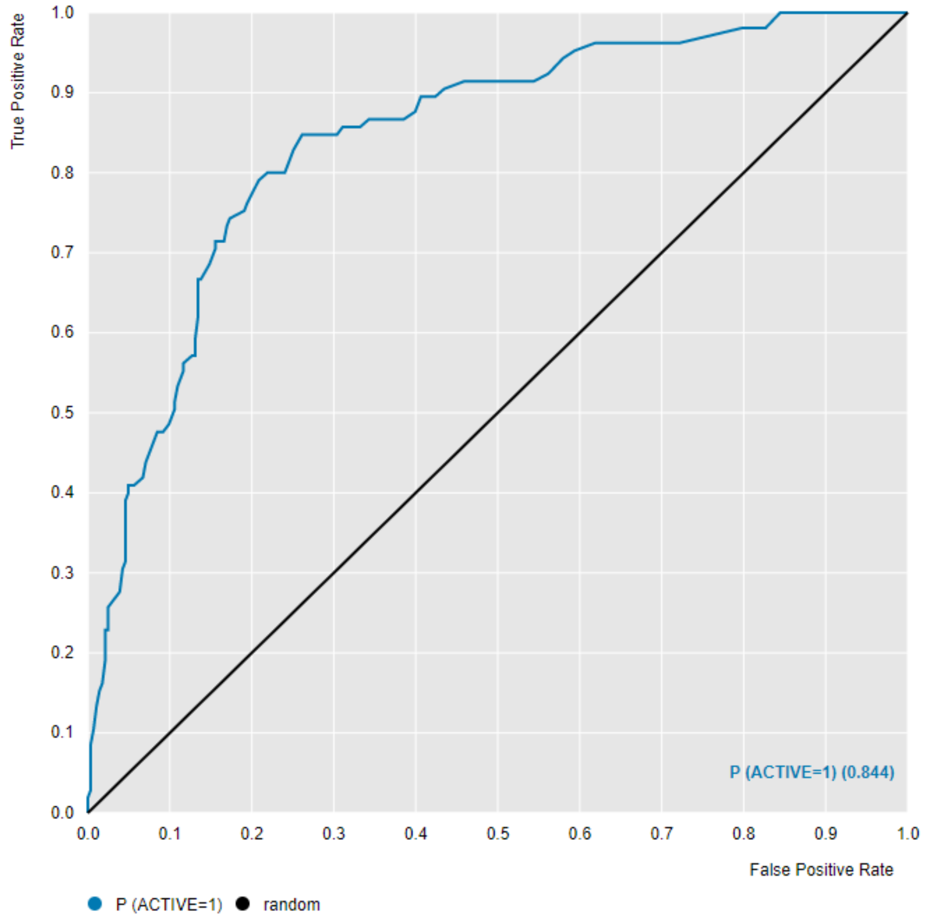

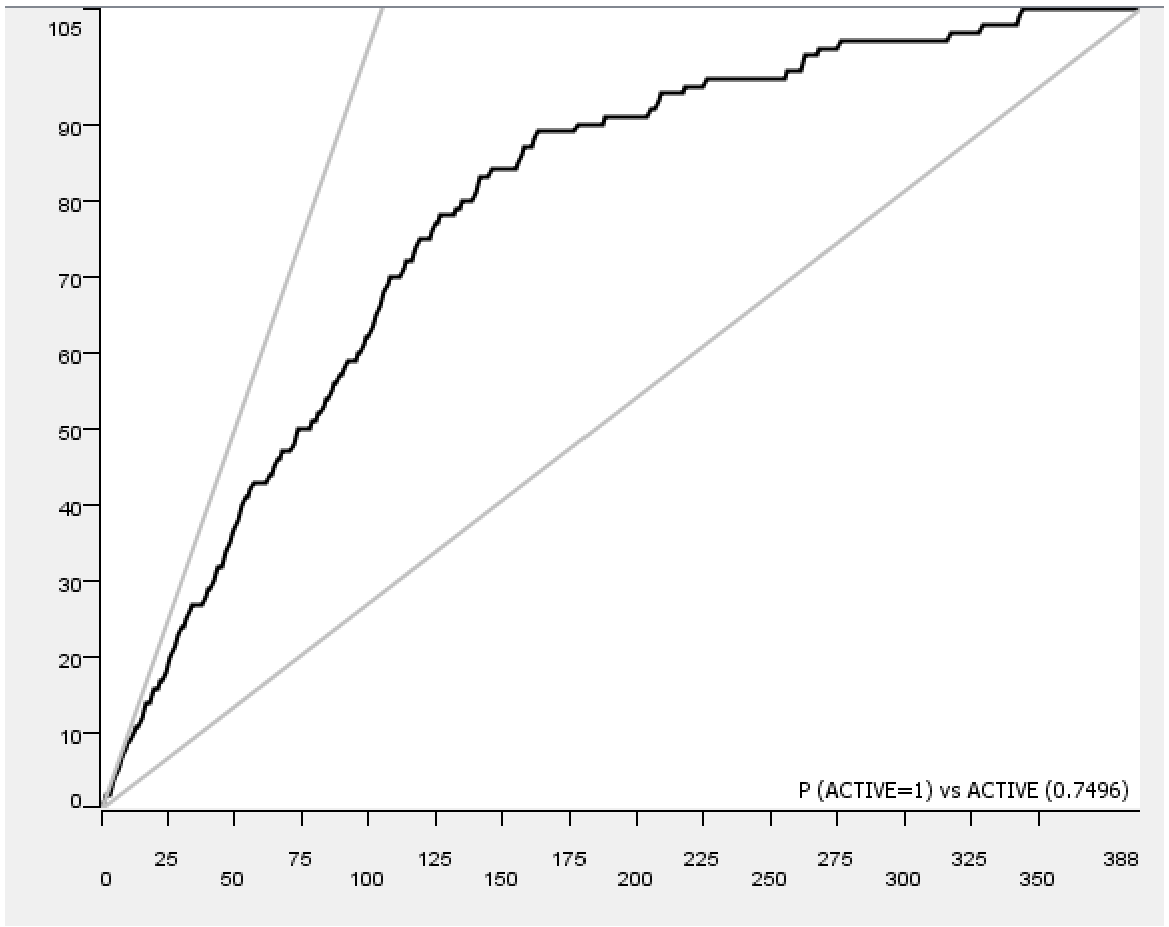

N=388

Top 10% Mean IC50	24.3 nM

	Predicted Active	Predicted Inactive
Active	72	33
Inactive	42	241

<20nM DefGood in PARP1, 30% error; Random seed = 121783

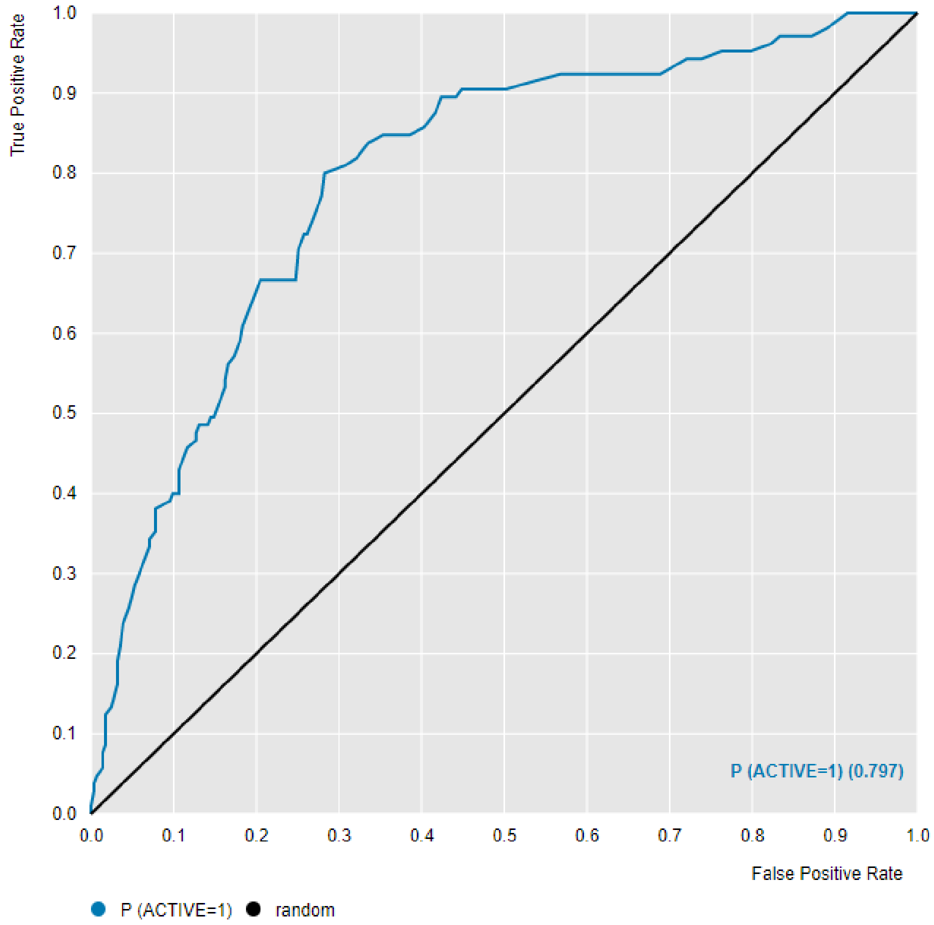

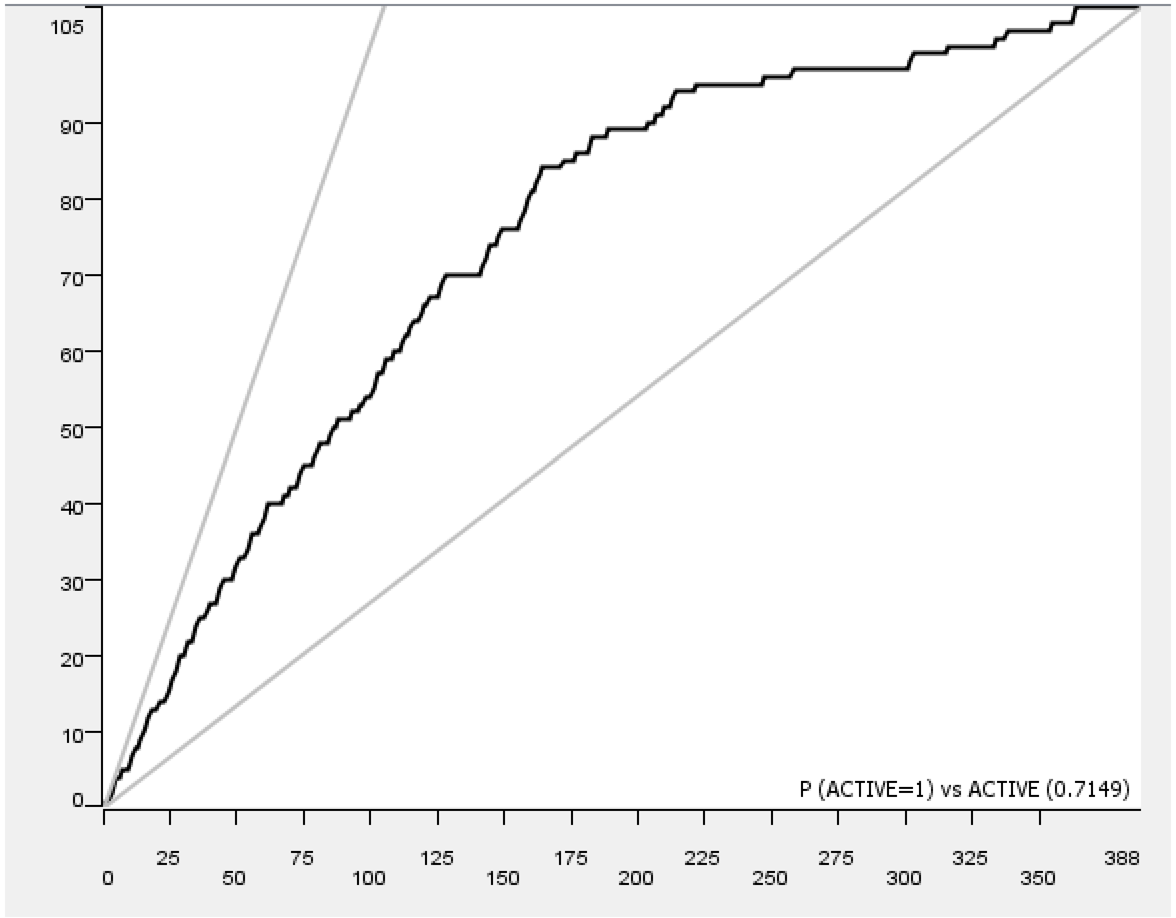

N=388

**Top 10% Mean IC50**	**32.5 nM**

	Predicted Active	Predicted Inactive
Active	70	35
Inactive	58	225

<20nM DefGood in PARP1, 35% error; Random seed = 121783

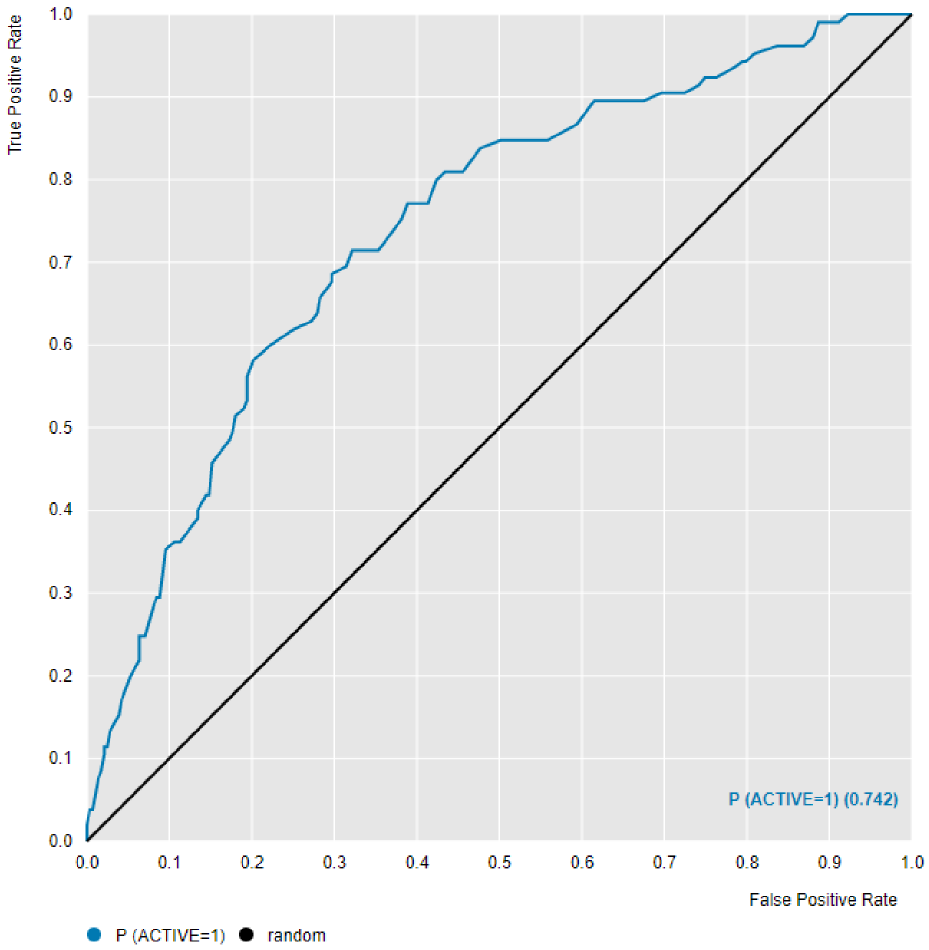

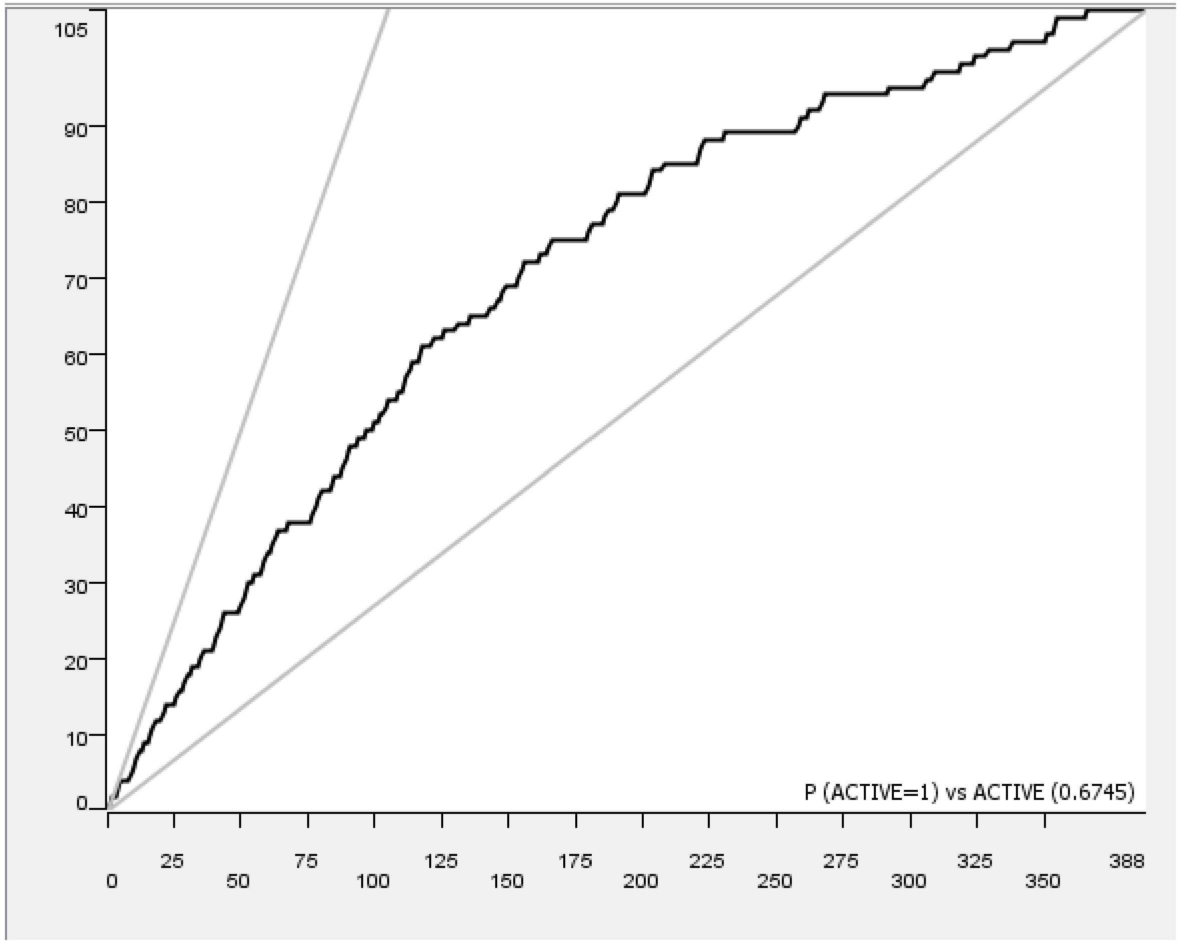

N=388

**Top 10% Mean IC50**	**64.8 nM**

	Predicted Active	Predicted Inactive
Active	64	41
Inactive	67	216

<20nM DefGood in PARP1, 40% error; Random seed = 121783

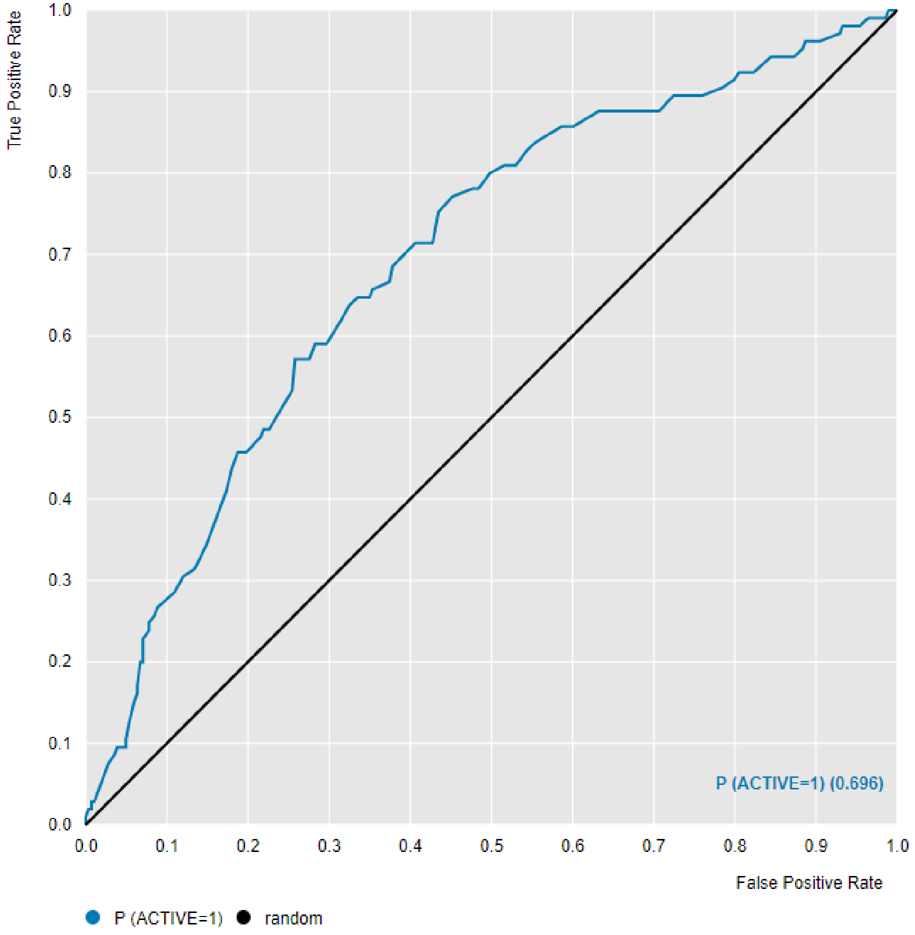

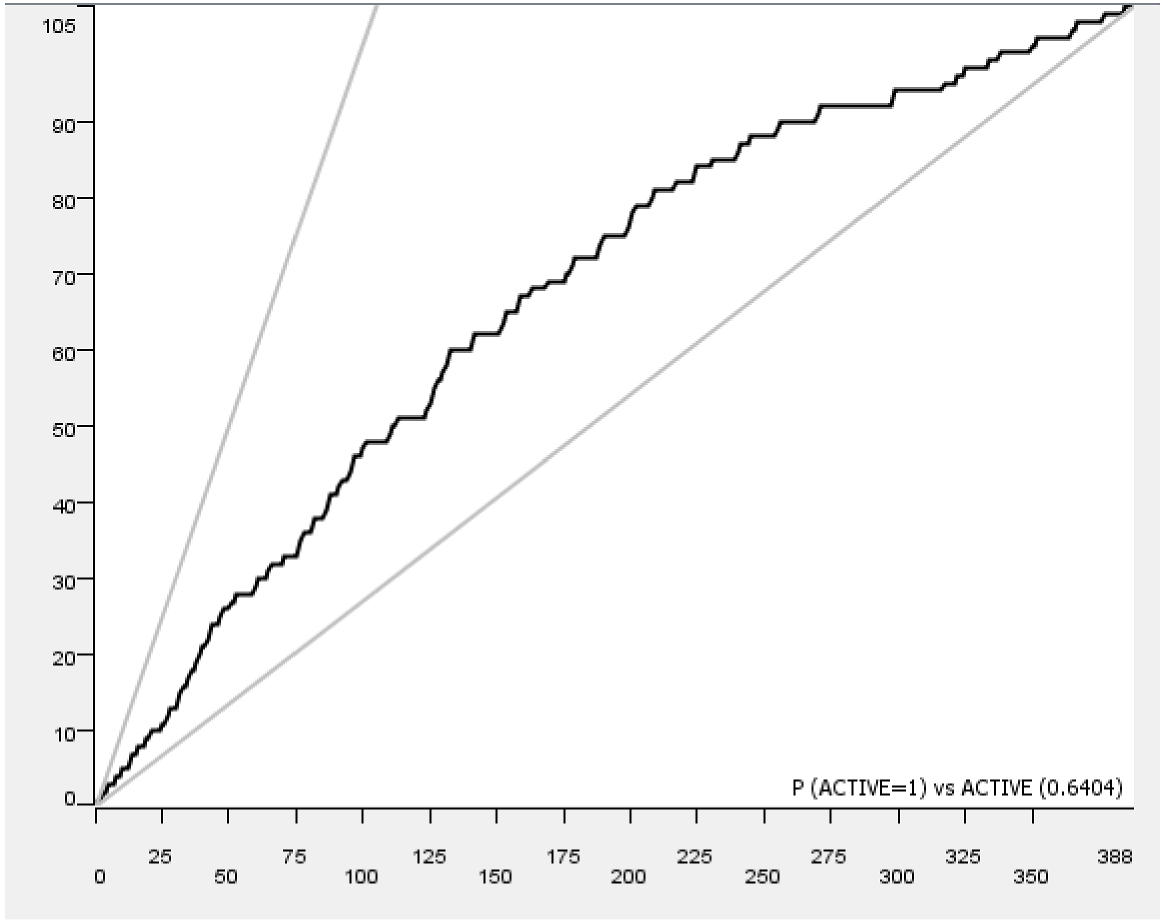

N=388

**Top 10% Mean IC50**	**194 nM**

	**Predicted Active**	**Predicted Inactive**
Active	62	43
Inactive	82	201

Conclusion - RF

- A Random Forrest could be generated for PARP1 kinase with a decision value of <20 nM as defined as active. The predictivity of the algorithm could be preserved in three random train/test cycles as error was introduced in the training set. The point of failure was 40%, 25% and 40% error.

PNN- <20nM DefGood in PARP1, 5% error ; Random seed = 1515533876005

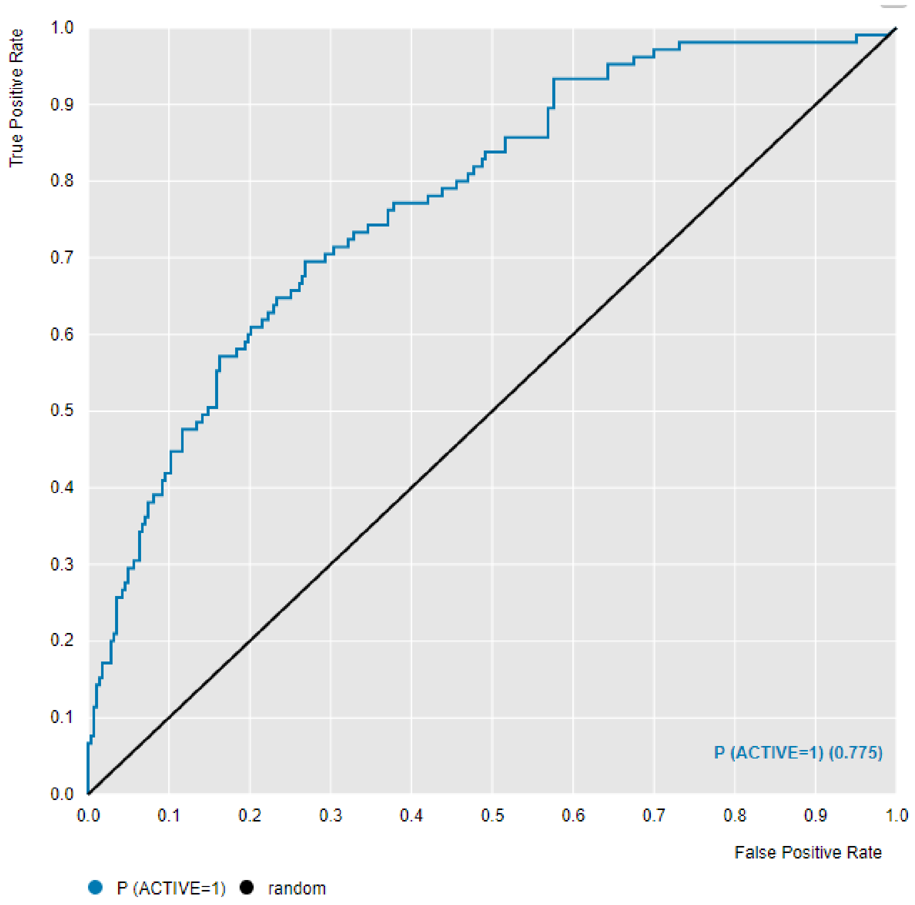

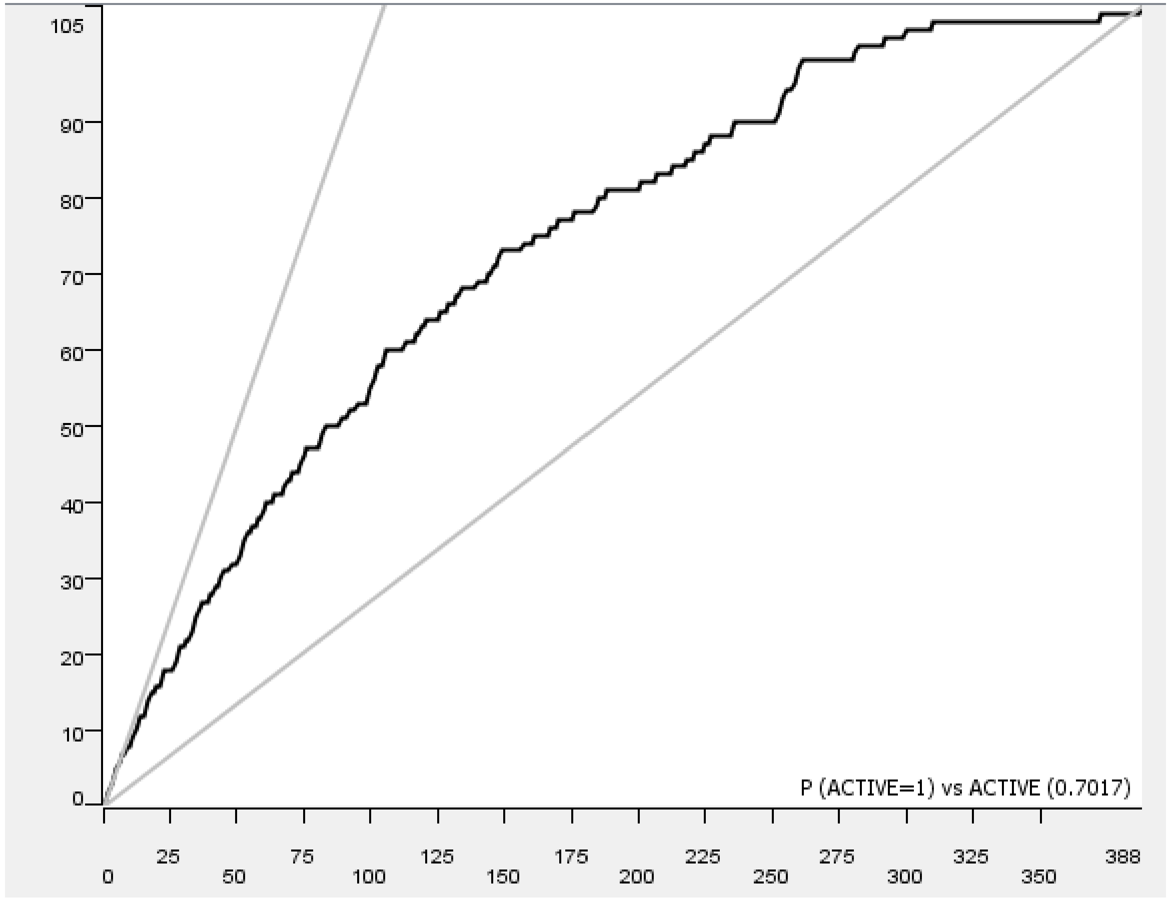

N=388

Top 10% Mean IC50	2,600 nM<br>Several Micromolar mistakes

	Predicted Active	Predicted Inactive
Active	7	98
Inactive	0	283

PNN - <20nM DefGood in PARP1, 5% error; Random seed = 1515533876005 (reparameterized Theta minus = 0.05; theta plus = 0.95)

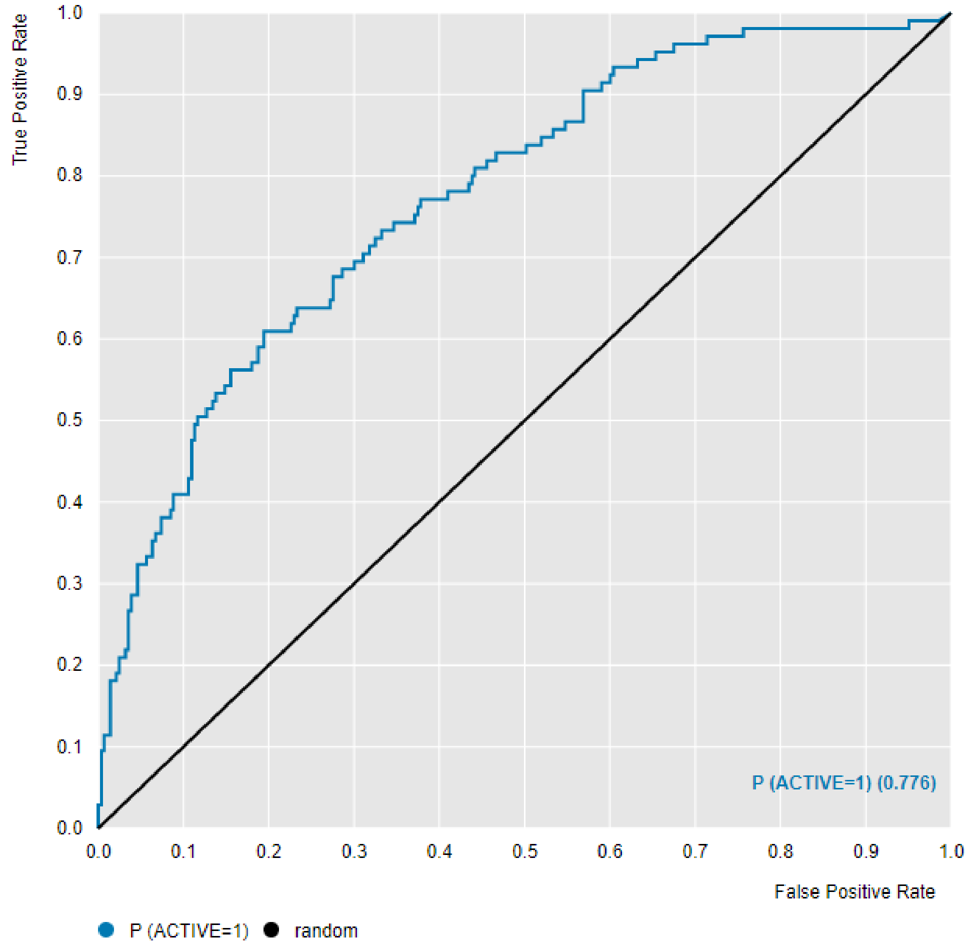

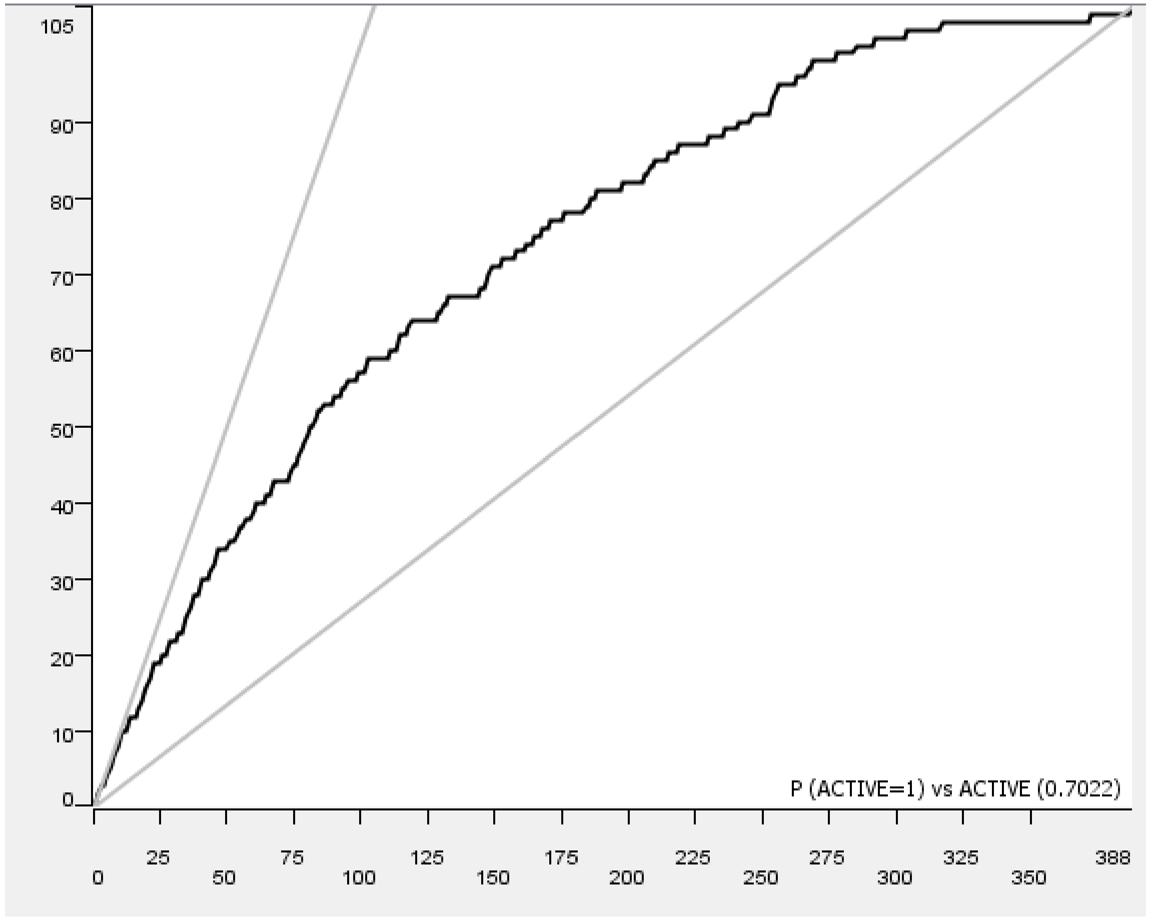

N=388

Top 10% Mean IC50	49.9 nM

	Predicted Active	Predicted Inactive
Active	24	81
Inactive	10	273

PNN- <20nM DefGood in PARP1, 10% error ; Random seed = 1515533876005

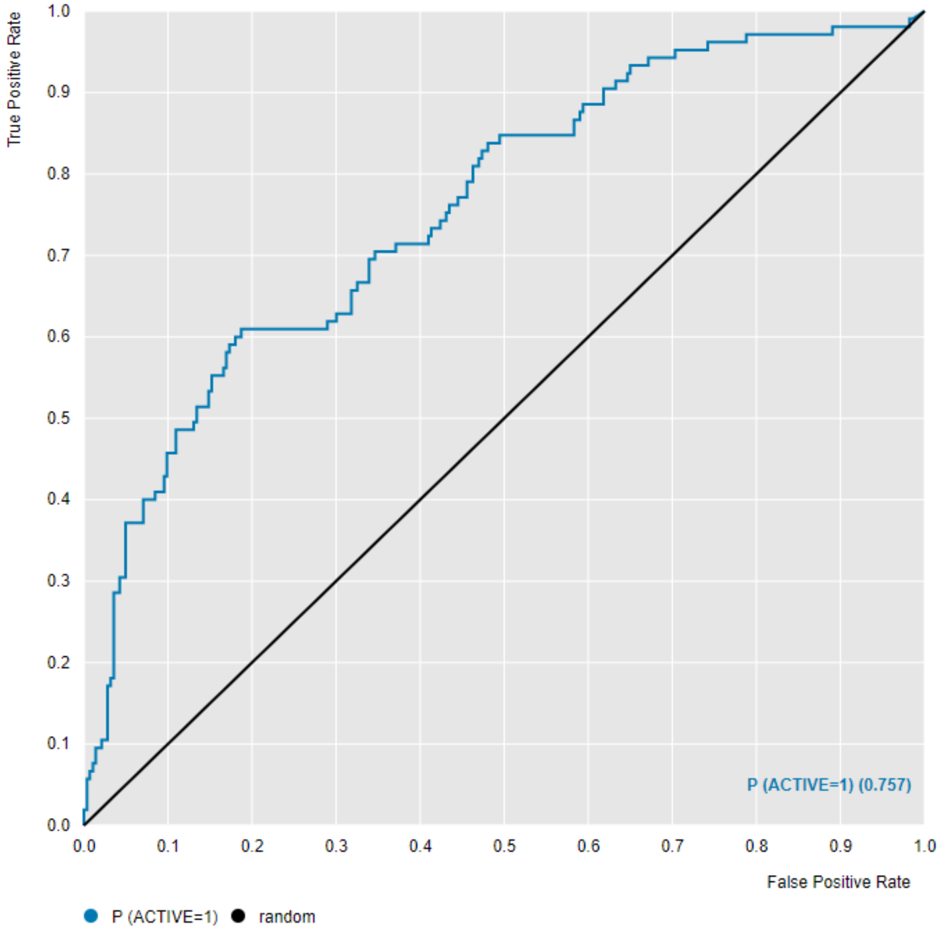

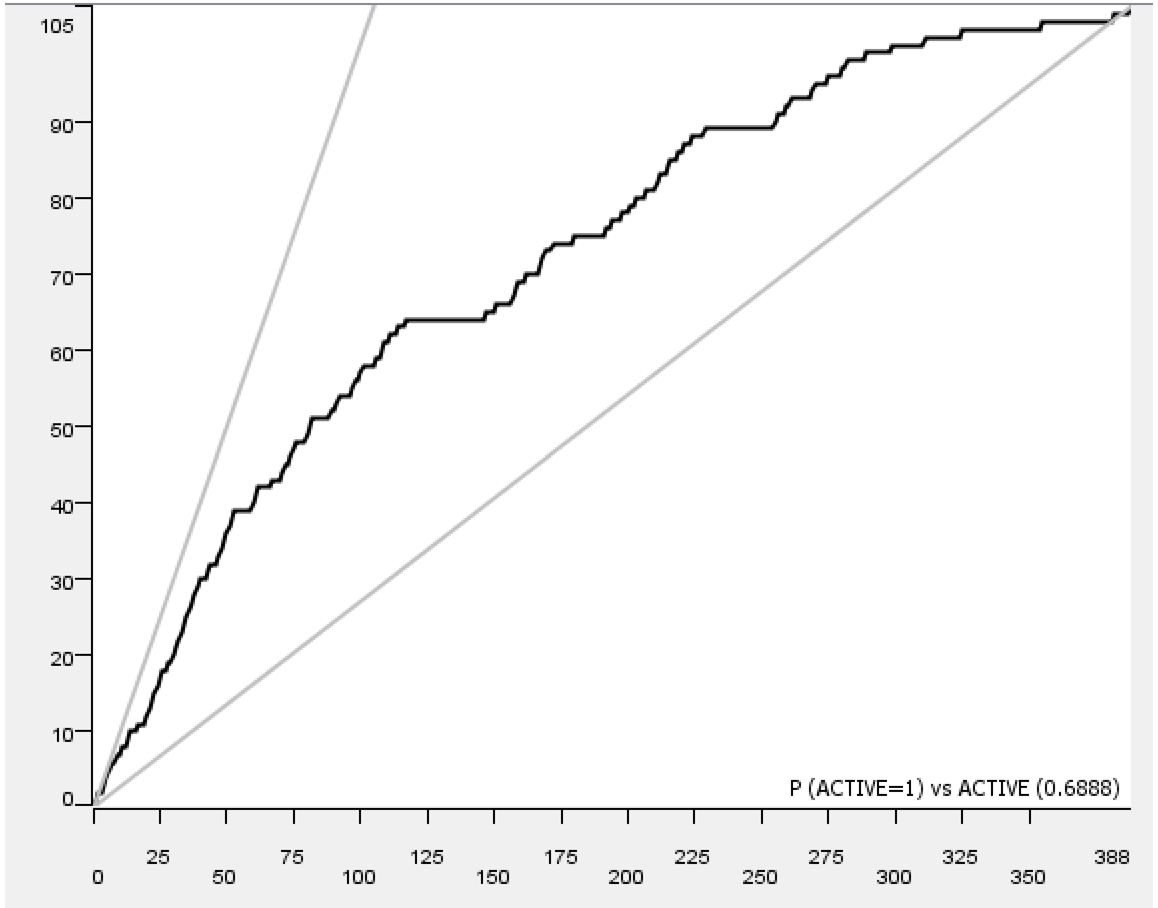

N=388

Top 10% Mean IC50	27.4 nM

	Predicted Active	Predicted Inactive
Active	42	63
Inactive	23	260

PNN- <20nM DefGood in PARP1, 20% error ; Random seed = 1515533876005

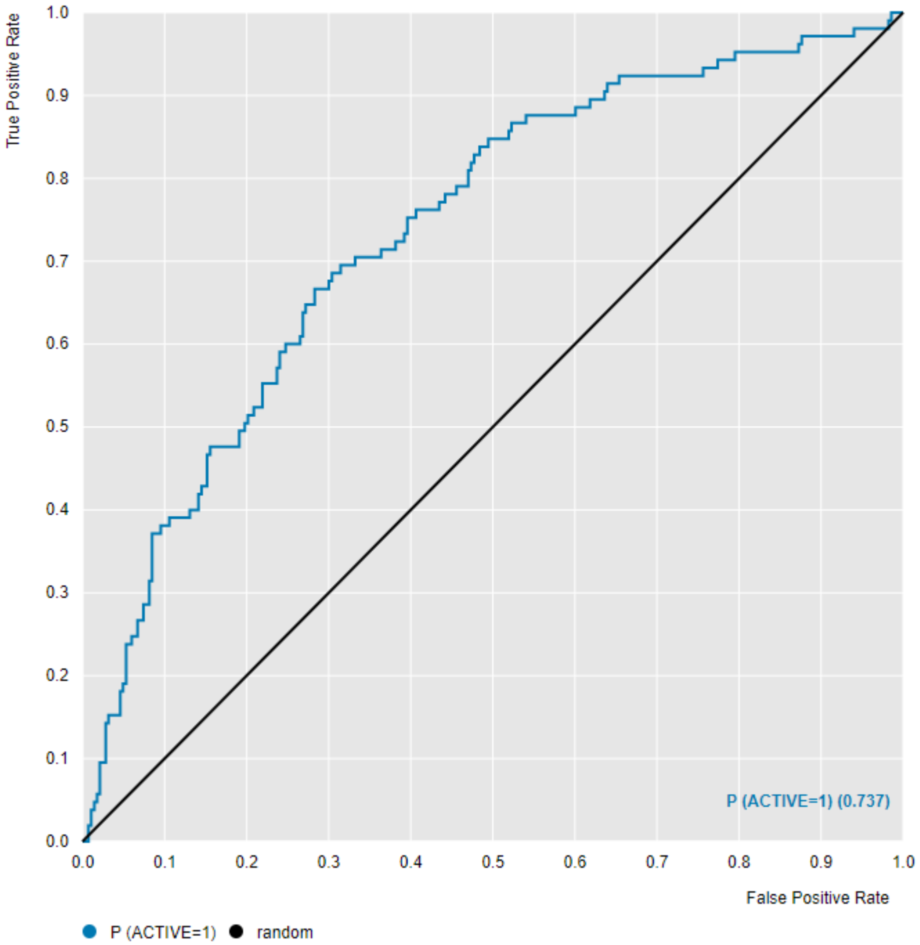

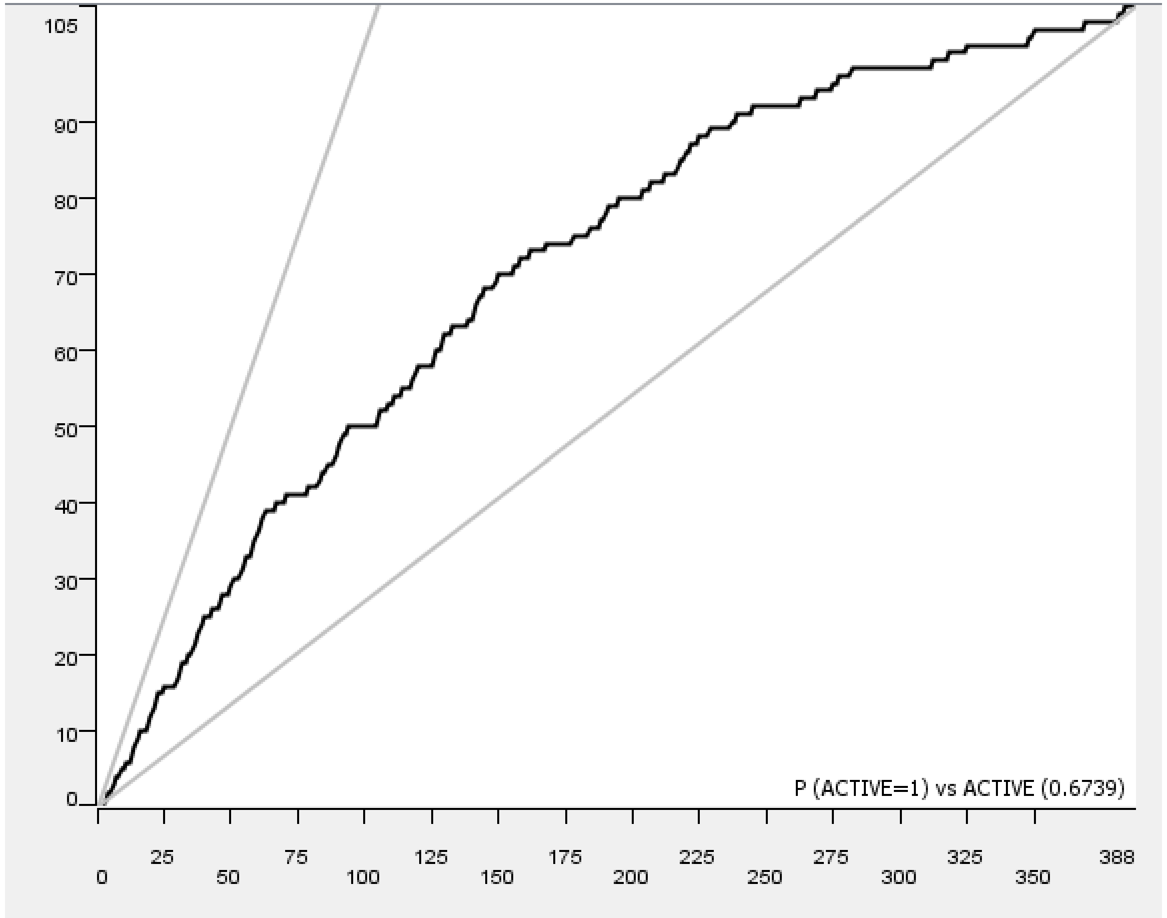

N=388

Top 10% Mean IC50	146 nM

	Predicted Active	Predicted Inactive
Active	41	64
Inactive	36	247

PNN- <20nM DefGood in PARP1, 25% error ; Random seed = 1515533876005

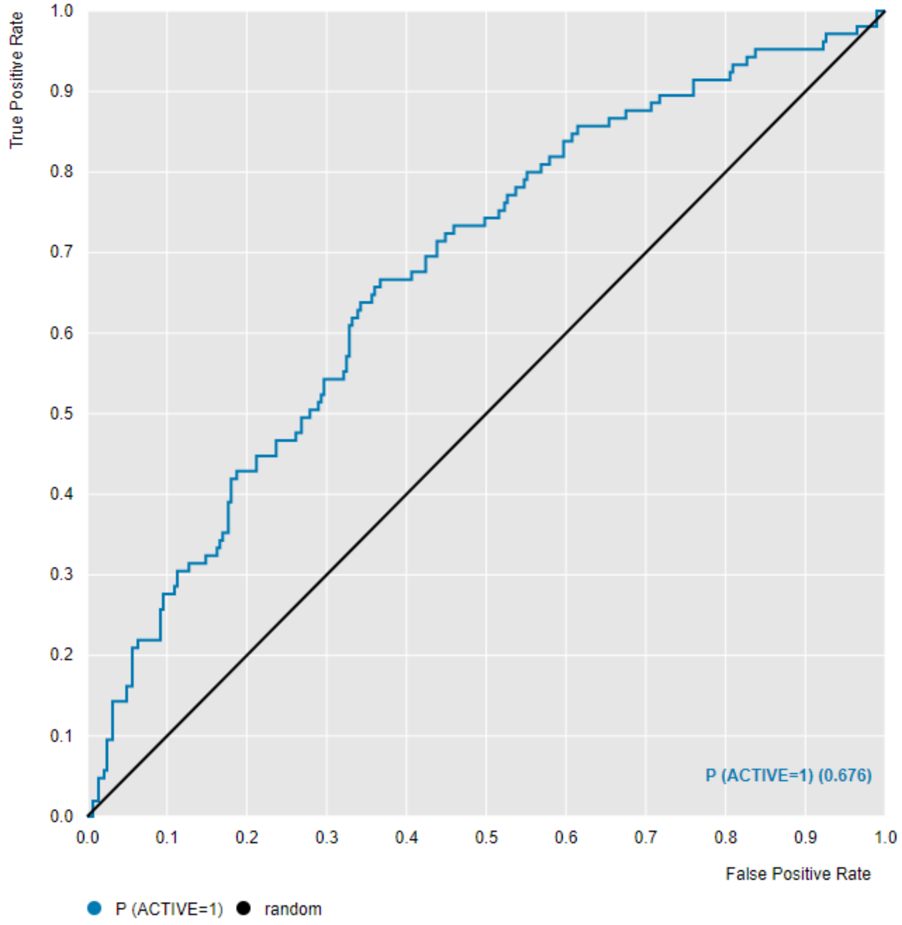

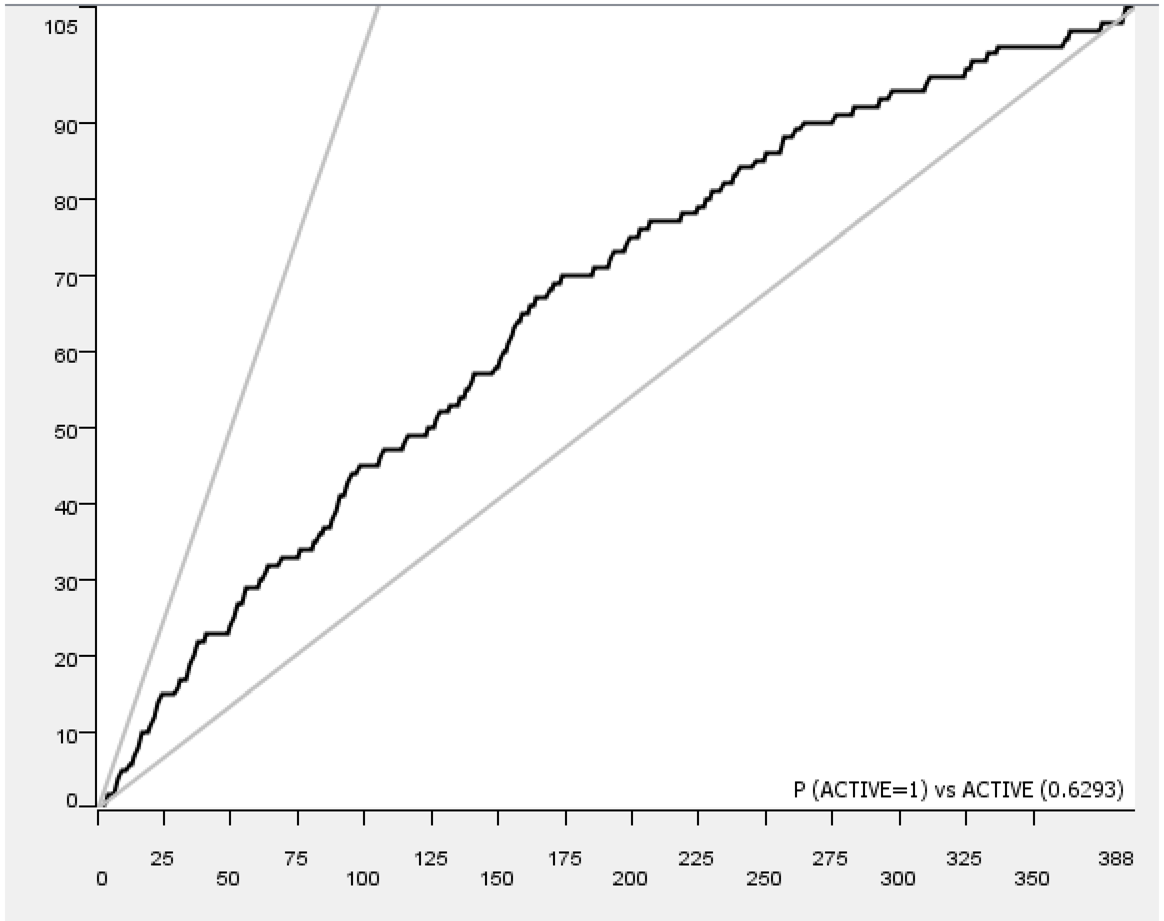

N=388

Top 10% Mean IC50	318 nM

	Predicted Active	Predicted Inactive
Active	34	71
Inactive	42	241

PNN - <20nM DefGood in PARP1, 25% error; Random seed = 1515533876005 (reparameterized Theta minus = 0.55; theta plus = 0.65)

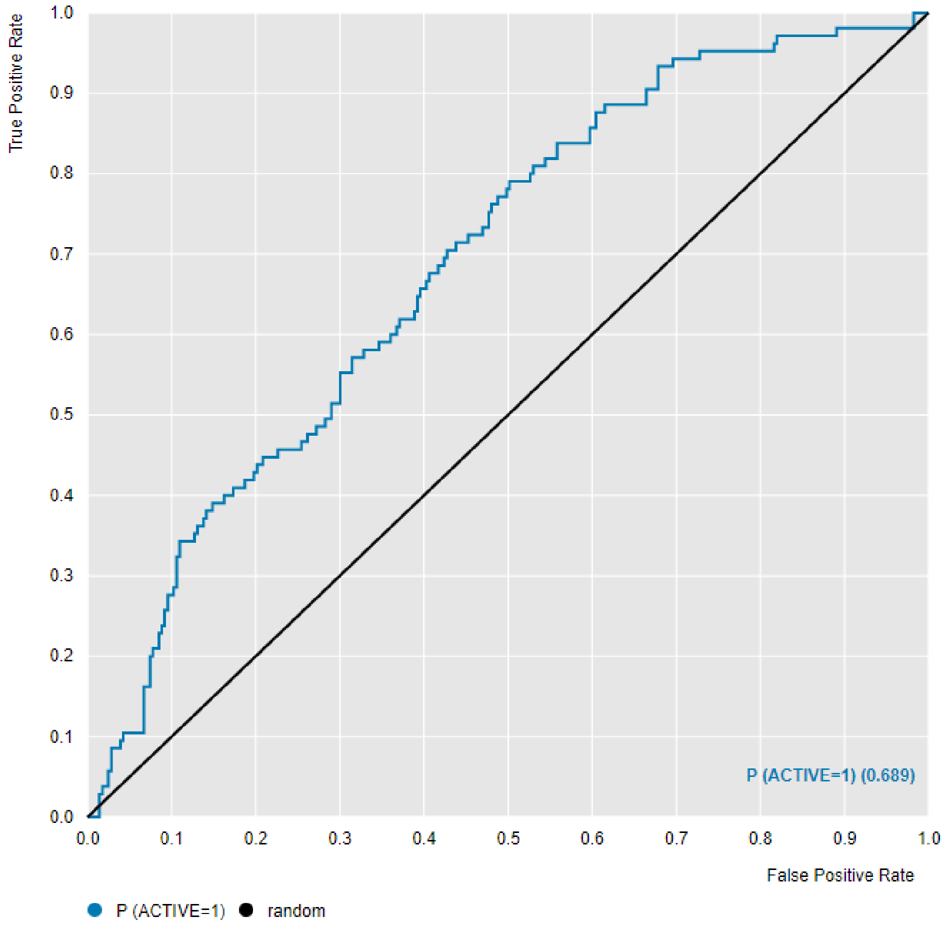

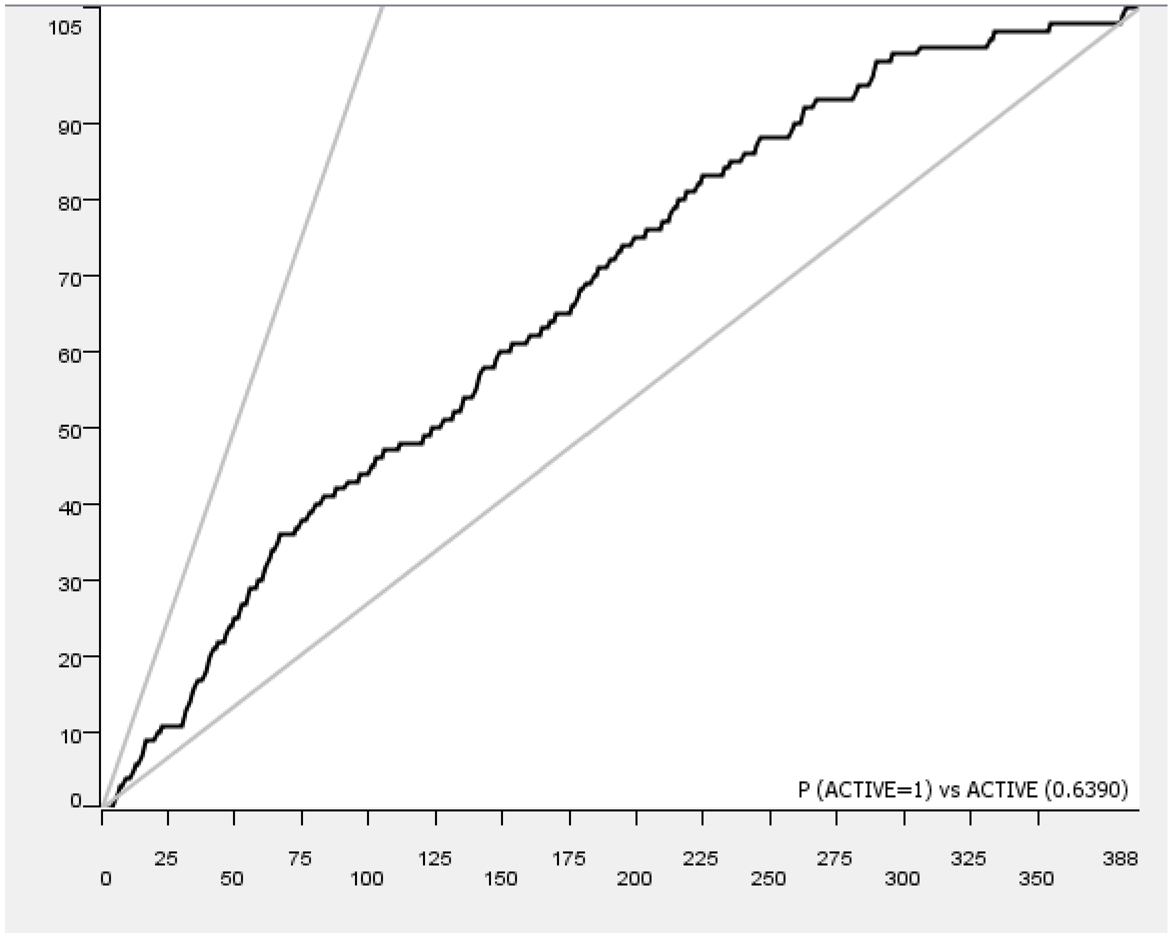

N=388

Top 10% Mean IC50	718 nM

	Predicted Active	Predicted Inactive
Active	0	105
Inactive	4	279

PNN- <20nM DefGood in PARP1, 5% error ; Random seed = 429

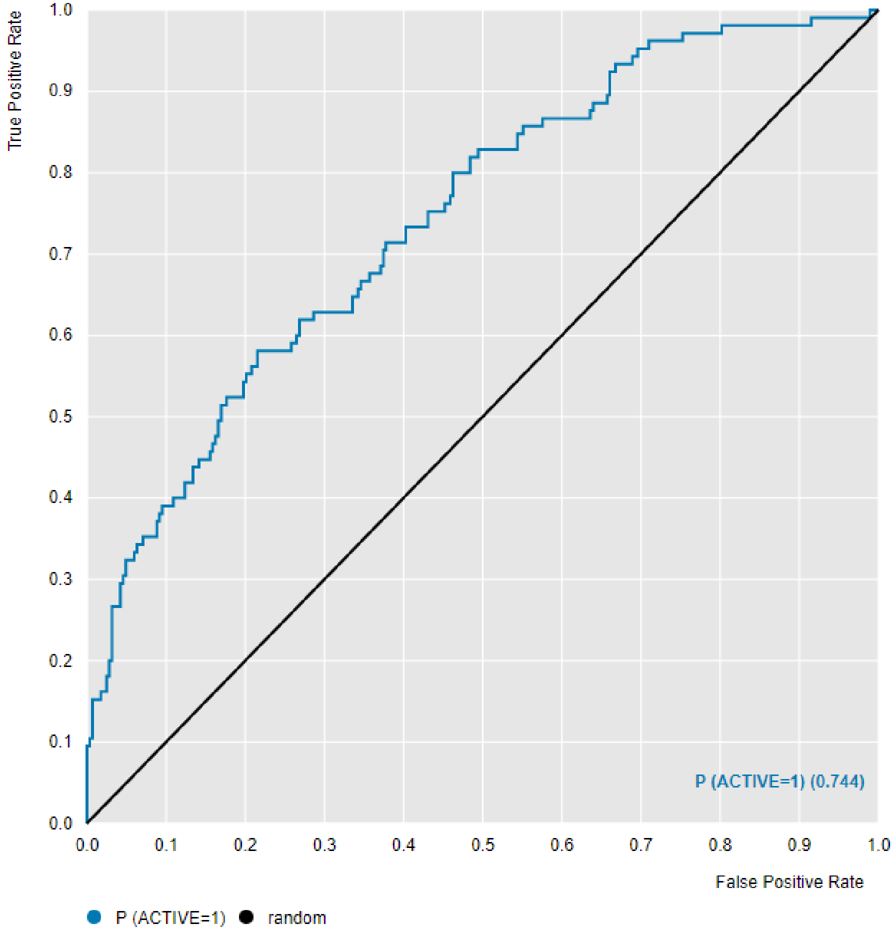

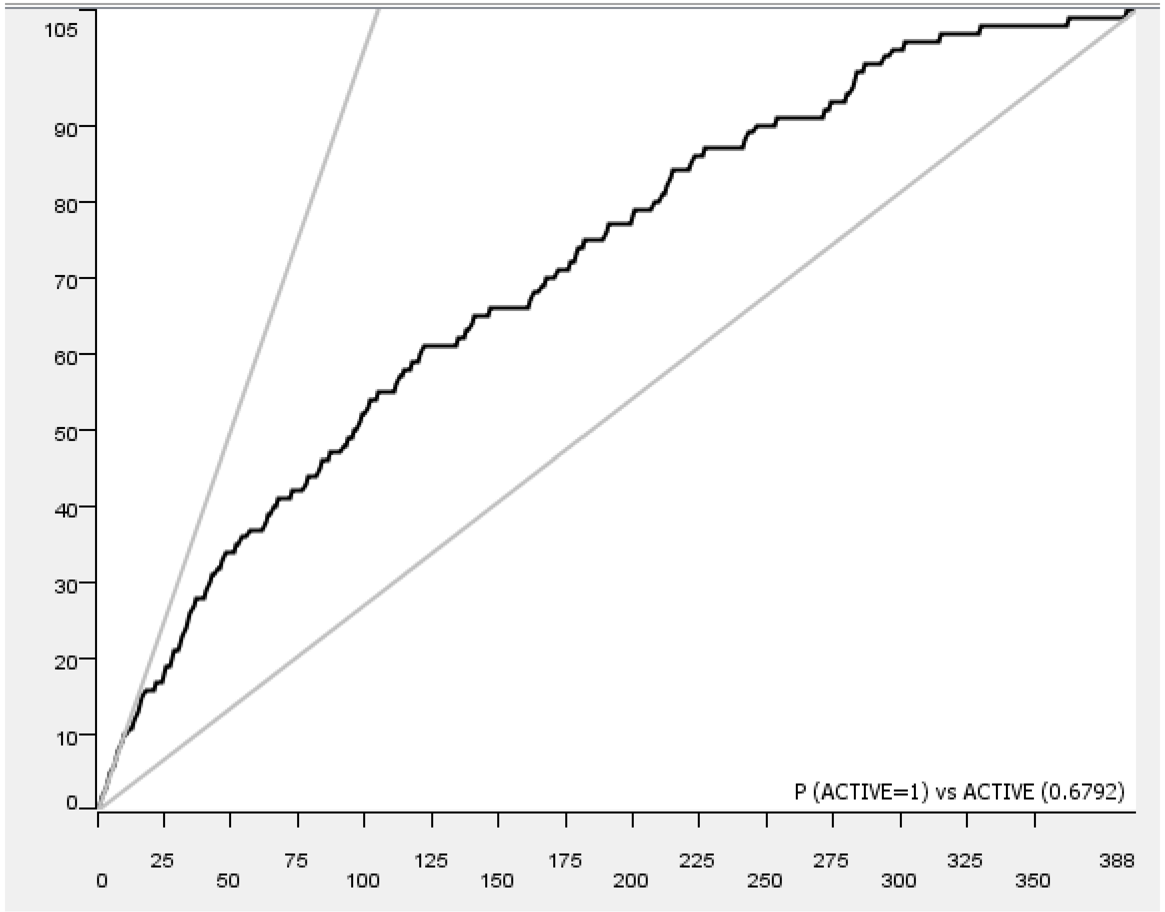

N=388

**Top 10% Mean IC50**	**2,800 nM**

	Predicted Active	Predicted Inactive
Active	18	87
Inactive	7	276

PNN - <20nM DefGood in PARP1, 5% error; Random seed = 429 (reparameterized Theta minus = 0.05; theta plus = 0.75)

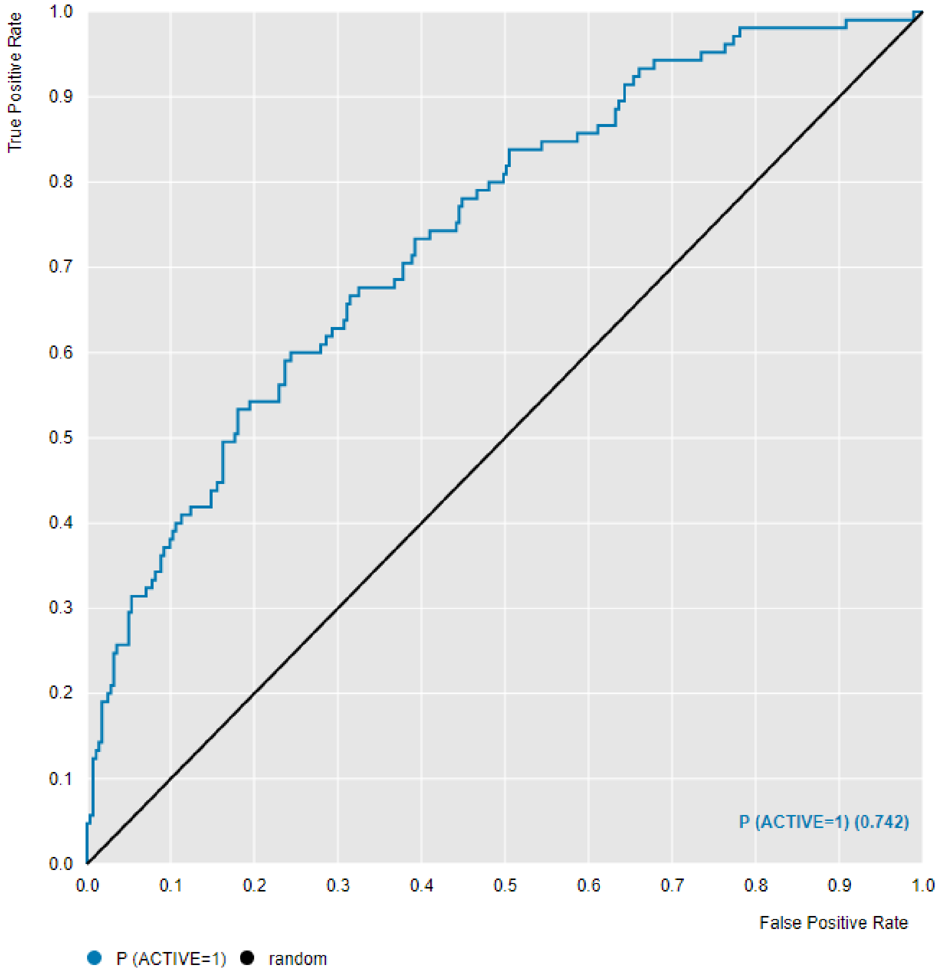

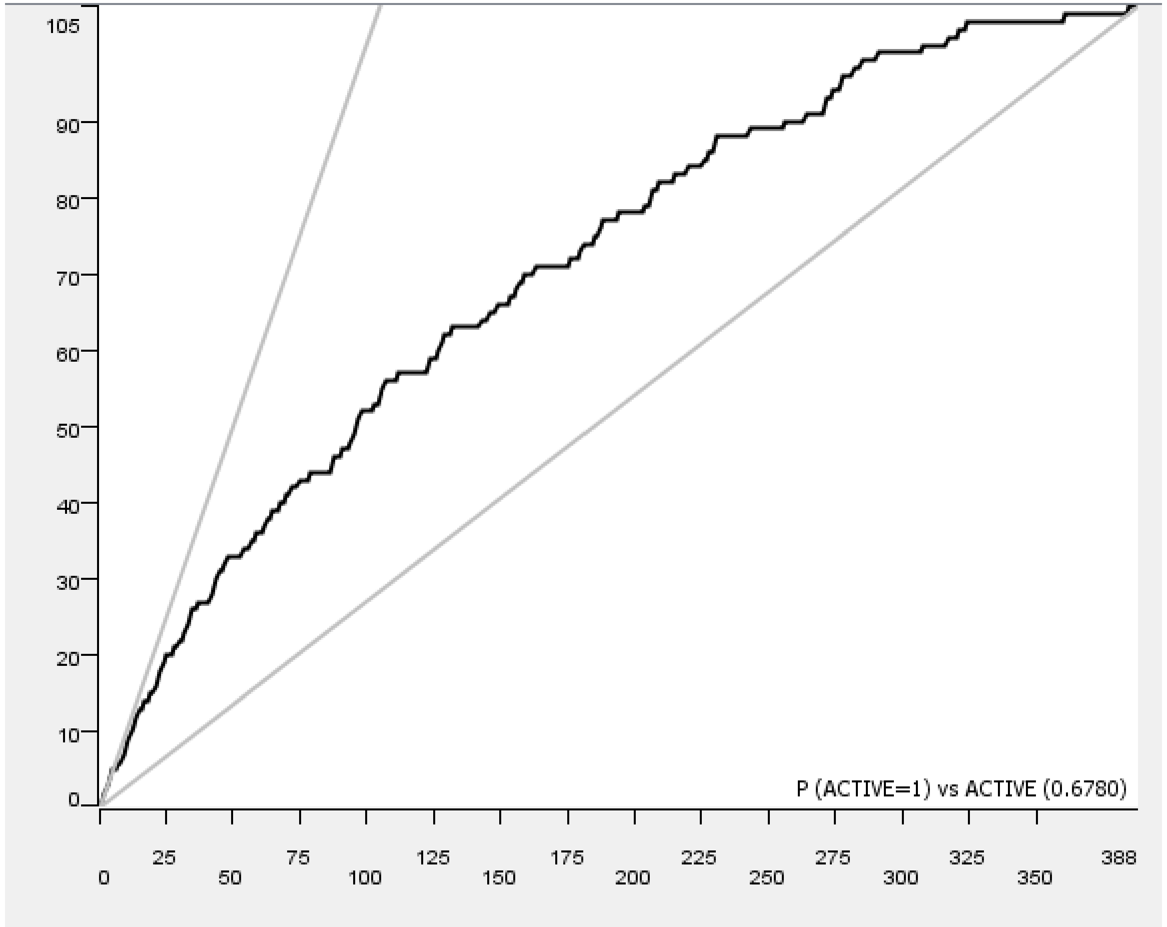

N=388

Top 10% Mean IC50	2,800 nM

	Predicted Active	Predicted Inactive
Active	35	70
Inactive	22	261

PNN - <20nM DefGood in PARP1, 5% error; Random seed = 121783

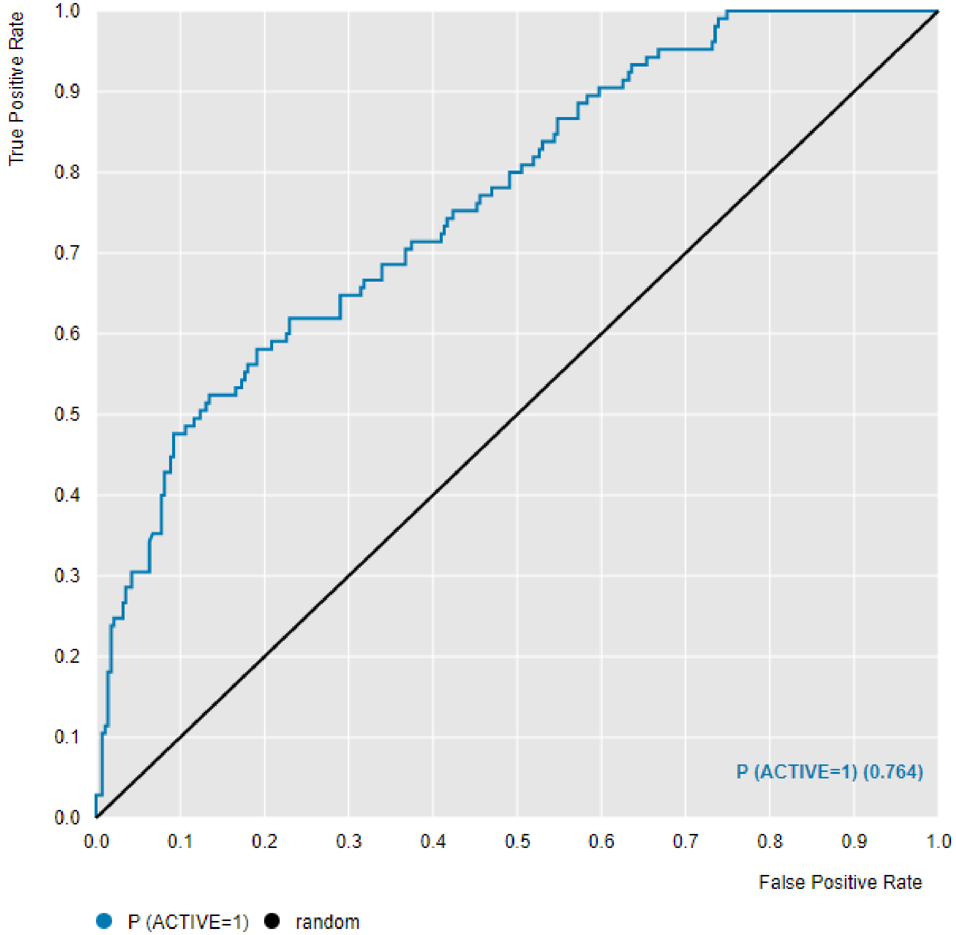

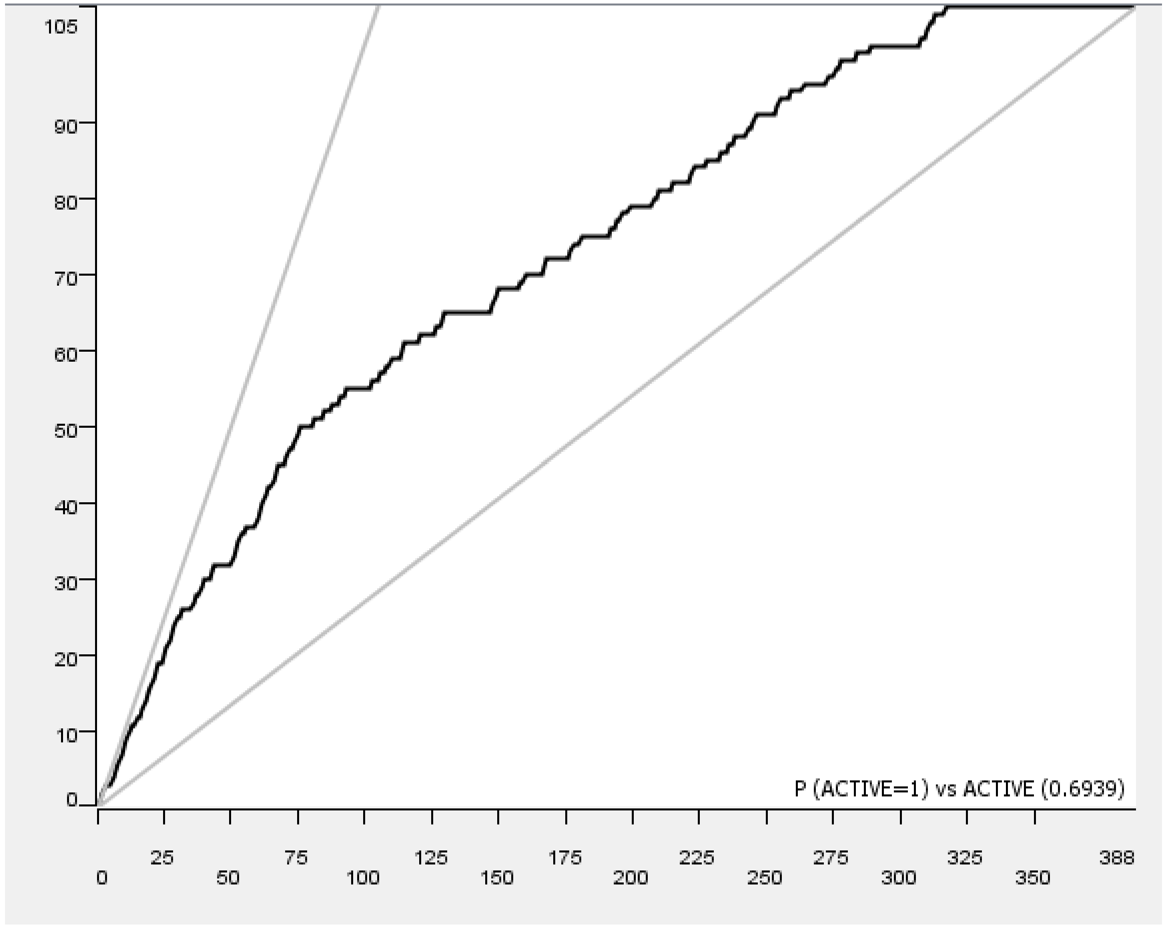

N=388

Top 10% Mean IC50	73.0 nM

	Predicted Active	Predicted Inactive
Active	16	89
Inactive	4	279

PNN - <20nM DefGood in PARP1, 10% error; Random seed = 121783

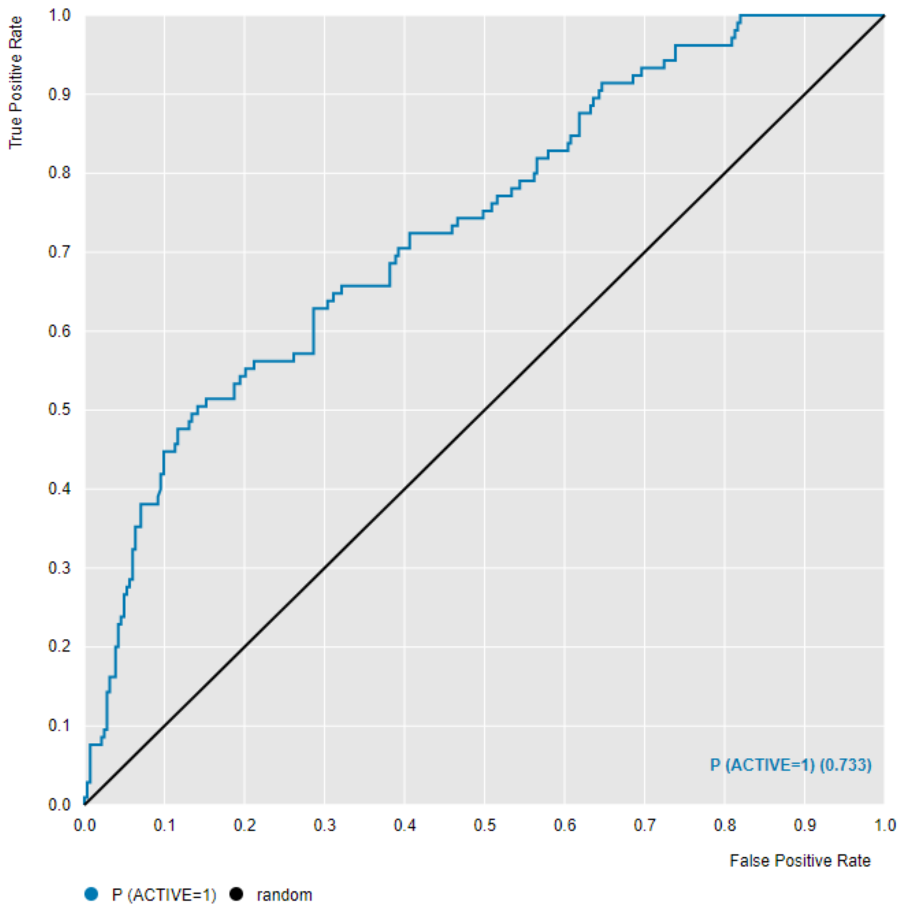

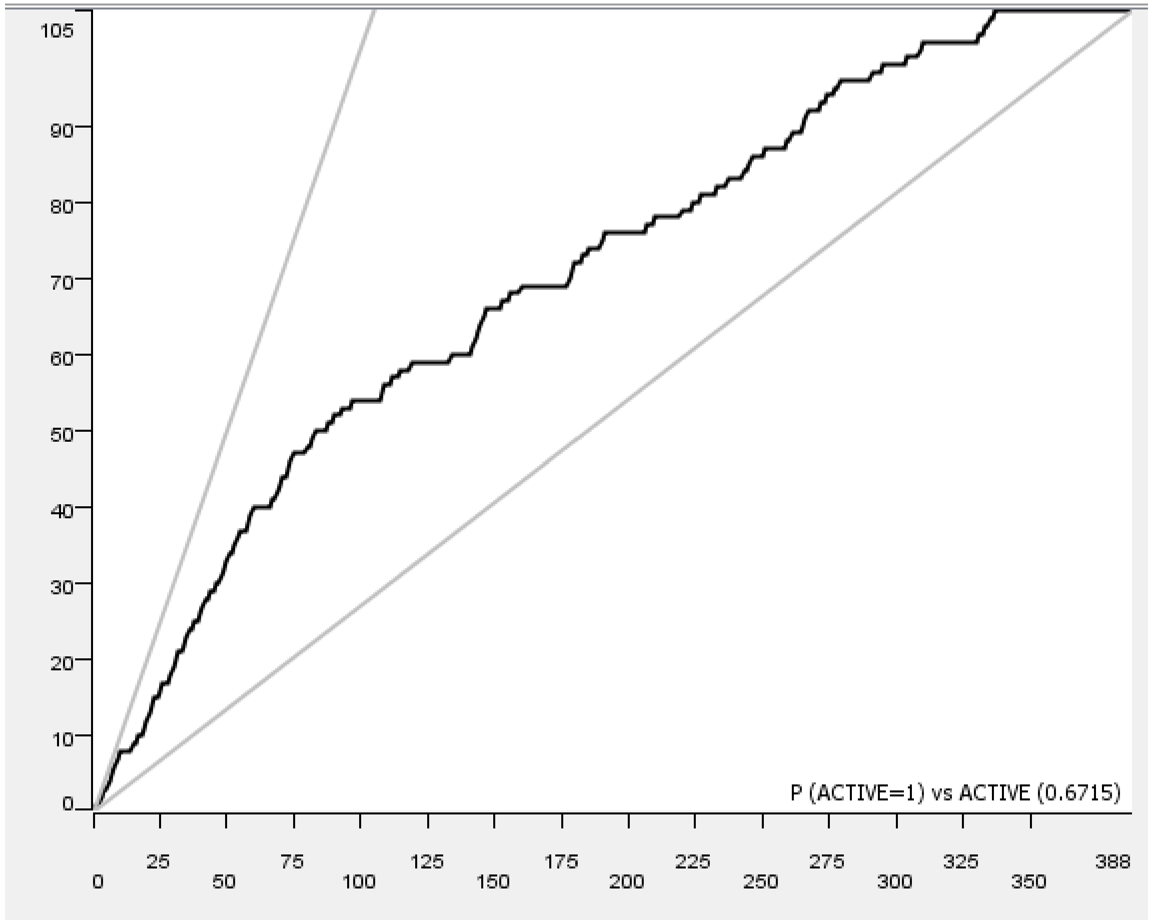

N=388

Top 10% Mean IC50	274 nM

	Predicted Active	Predicted Inactive
Active	18	87
Inactive	11	272

PNN - <20nM DefGood in PARP1, 15% error; Random seed = 121783

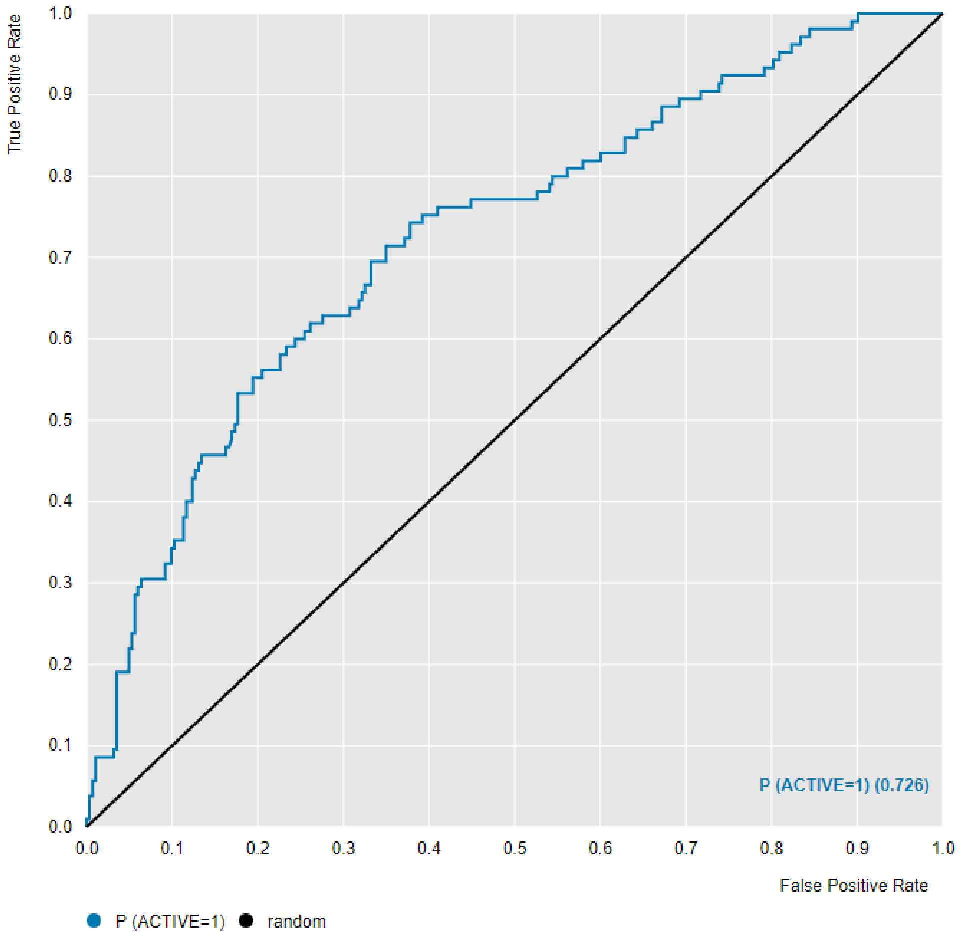

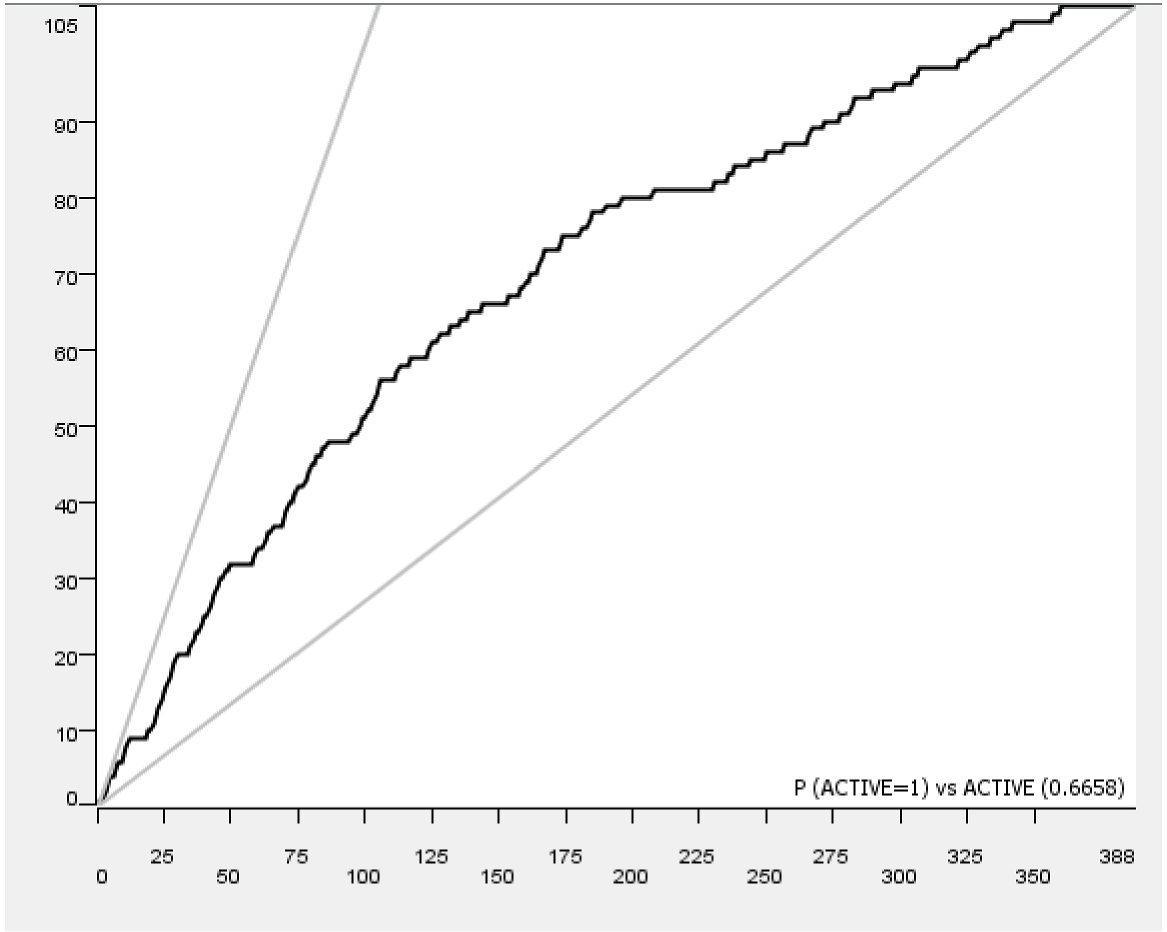

N=388

**Top 10% Mean IC50**	**232 nM**

	**Predicted Active**	**Predicted Inactive**
Active	20	85
Inactive	14	269

PNN - <20nM DefGood in PARP1, 20% error; Random seed = 121783

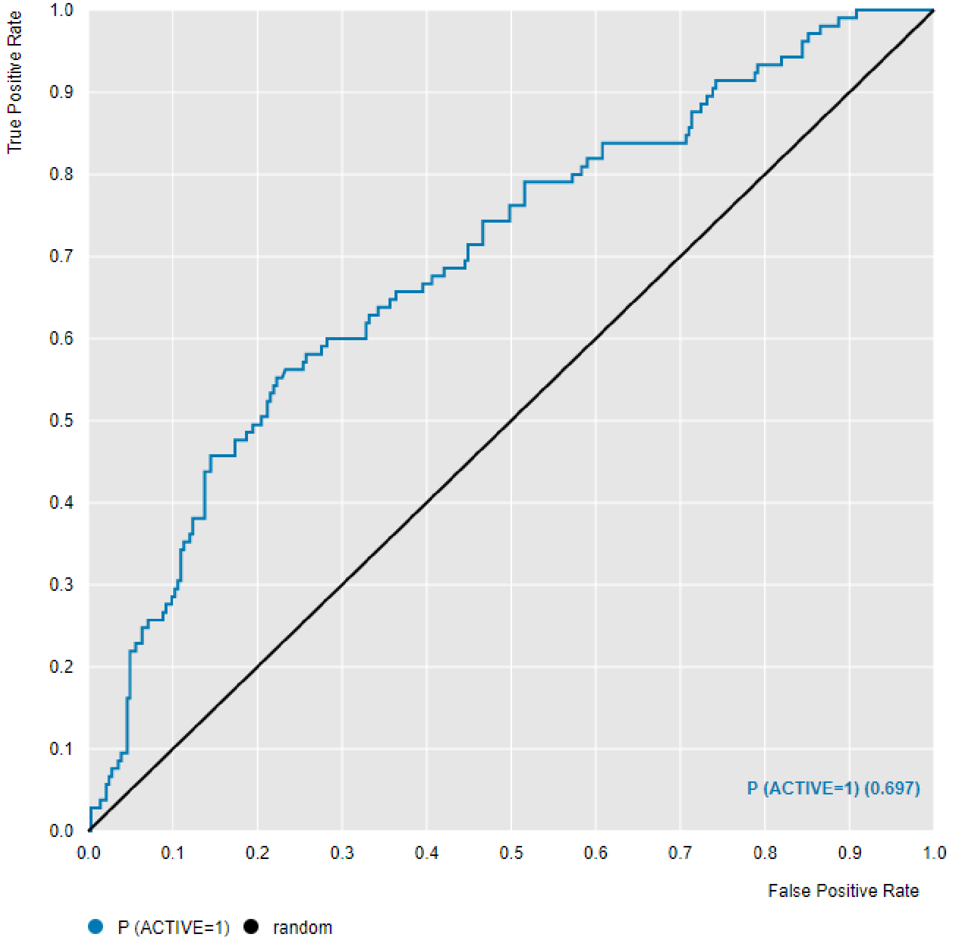

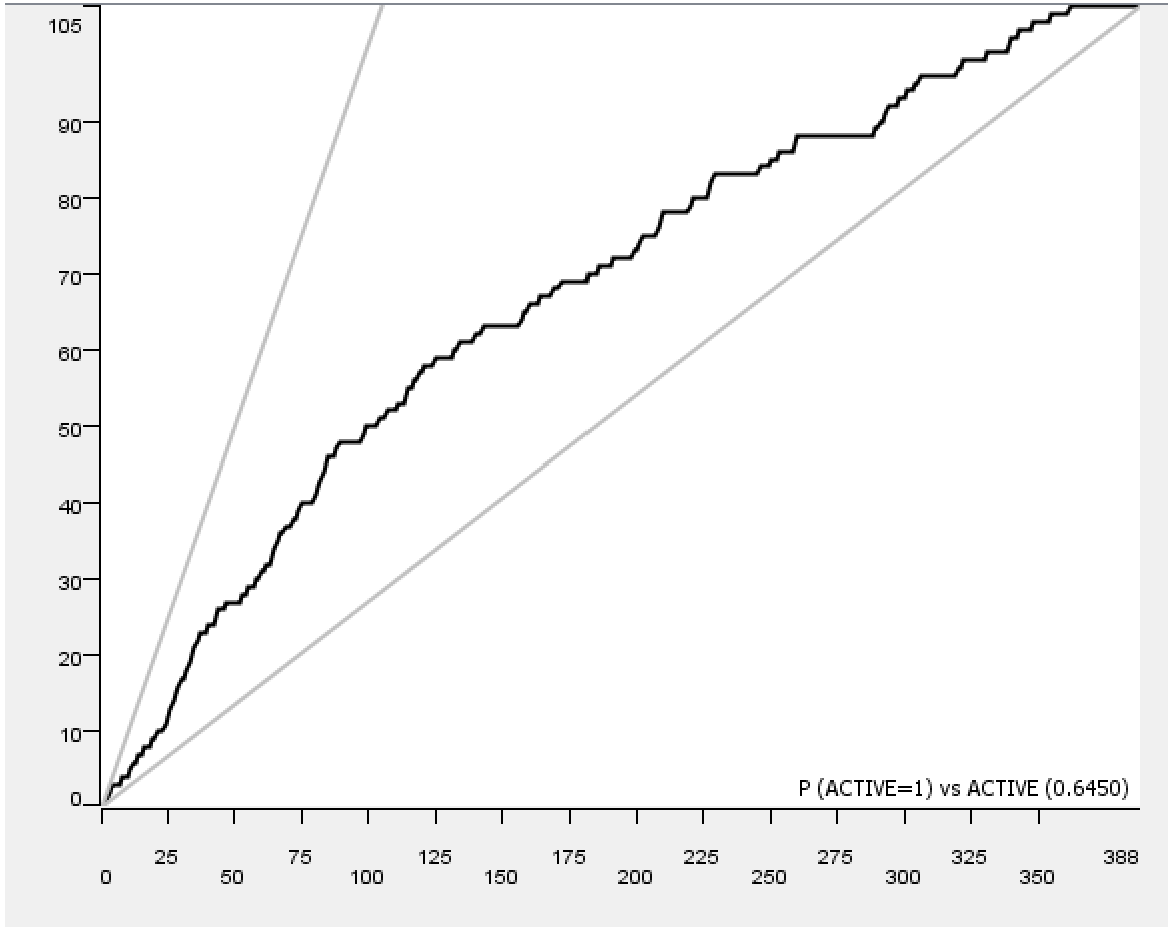

N=388

**Top 10% Mean IC50**	**336 nM**

	Predicted Active	Predicted Inactive
Active	42	63
Inactive	39	244

PNN - <20nM DefGood in PARP1, 20% error; Random seed = 121783 (reparameterized Theta minus = 0.35; theta plus = 0.75)

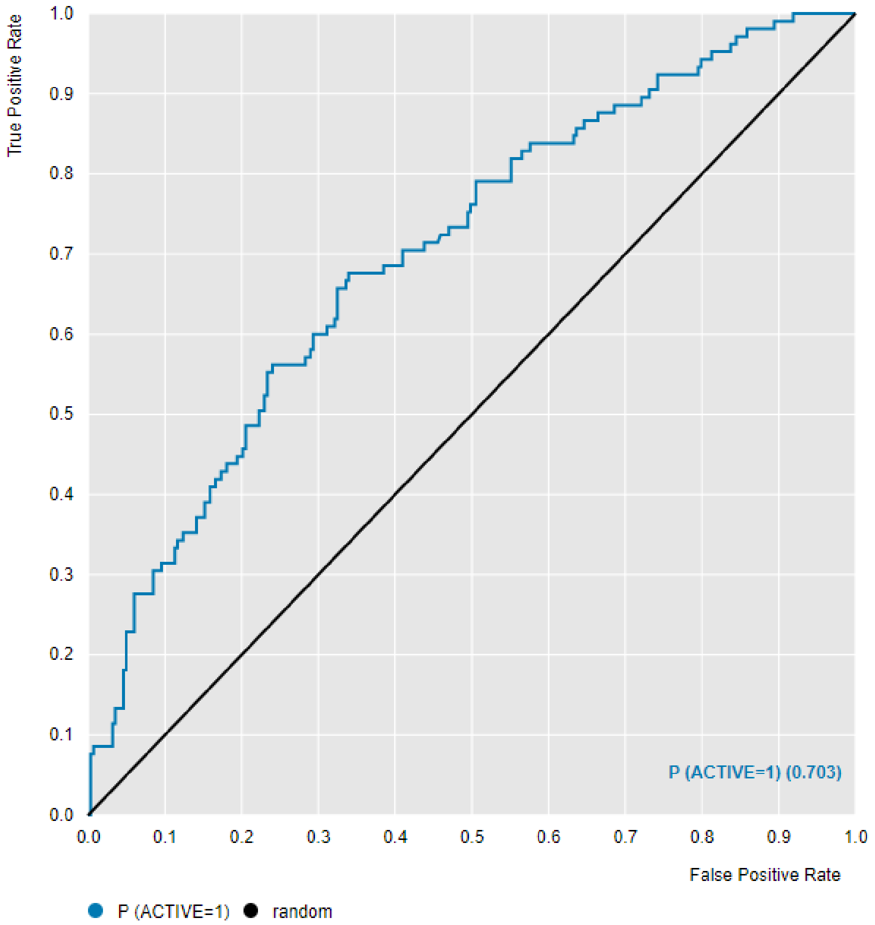

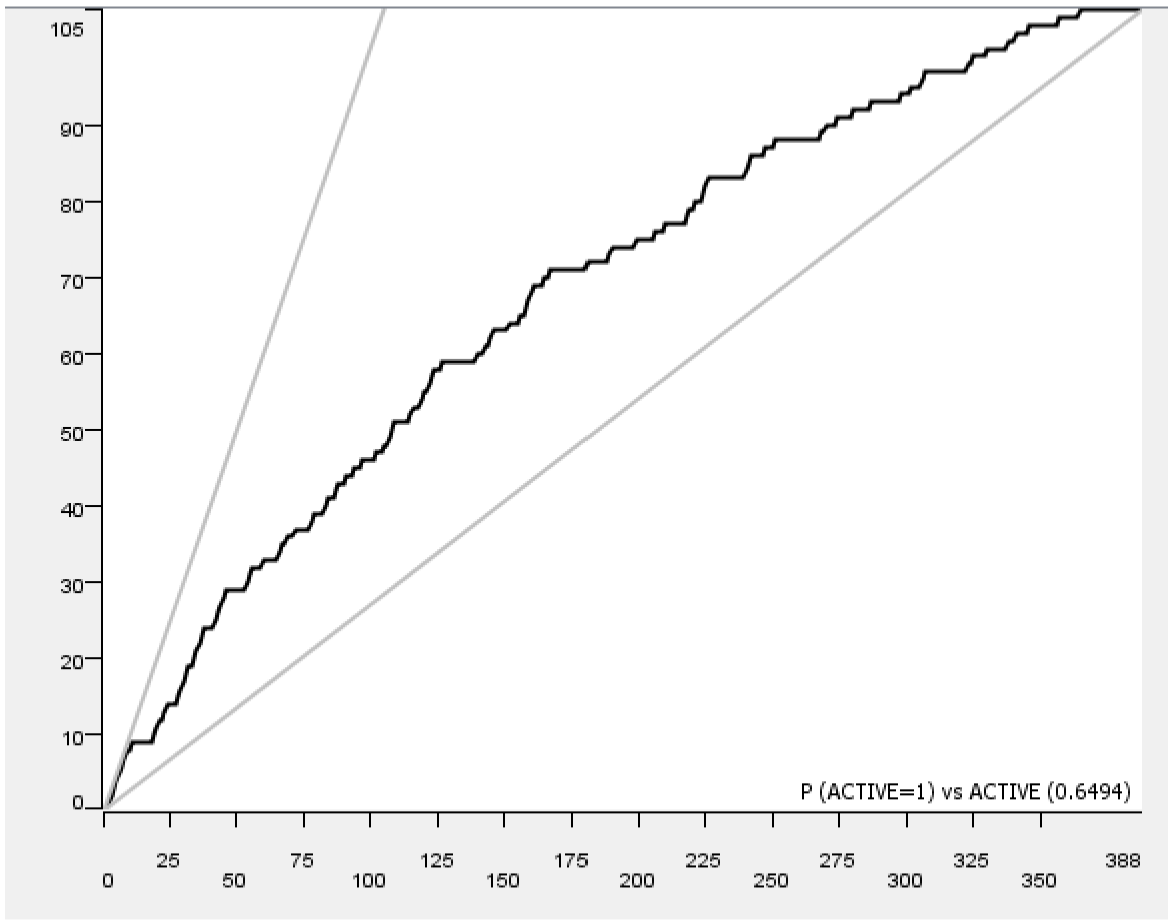

N=388

Top 10% Mean IC50	150 nM

	Predicted Active	Predicted Inactive
Active	14	91
Inactive	12	271

PNN - <20nM DefGood in PARP1, 25% error; Random seed = 121783 (reparameterized Theta minus = 0.35; theta plus = 0.65)

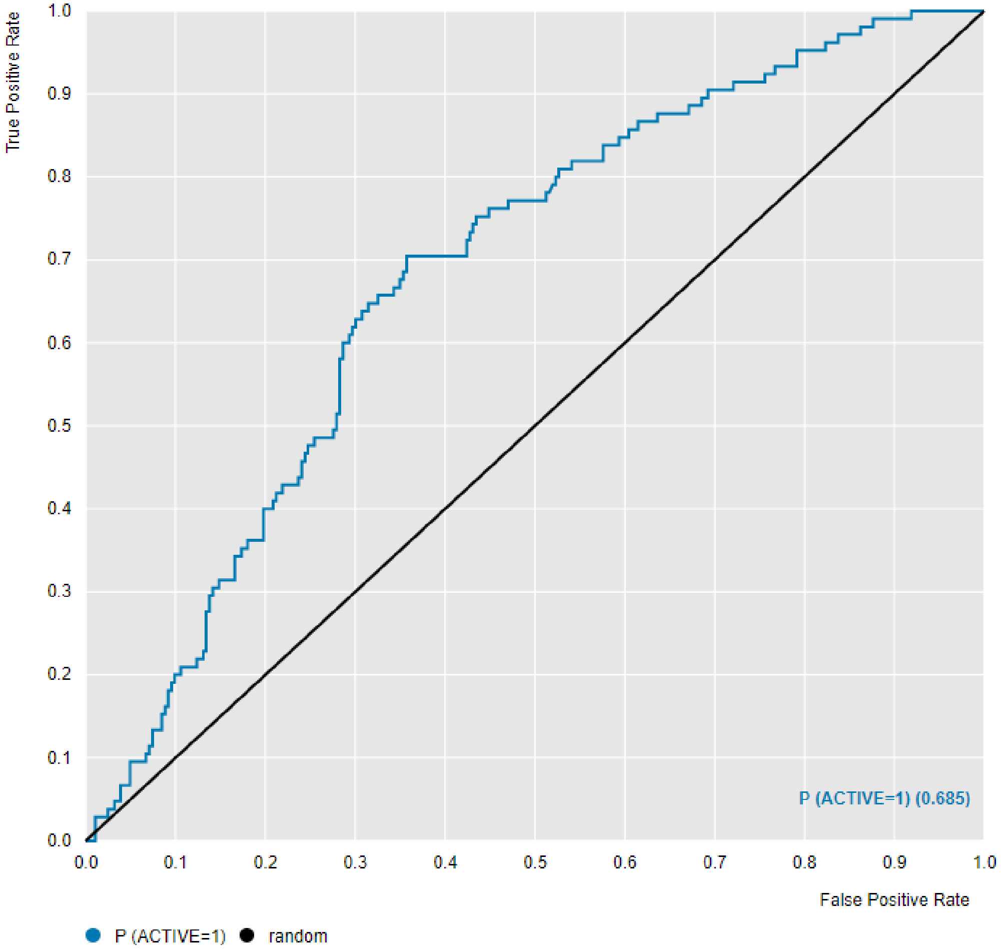

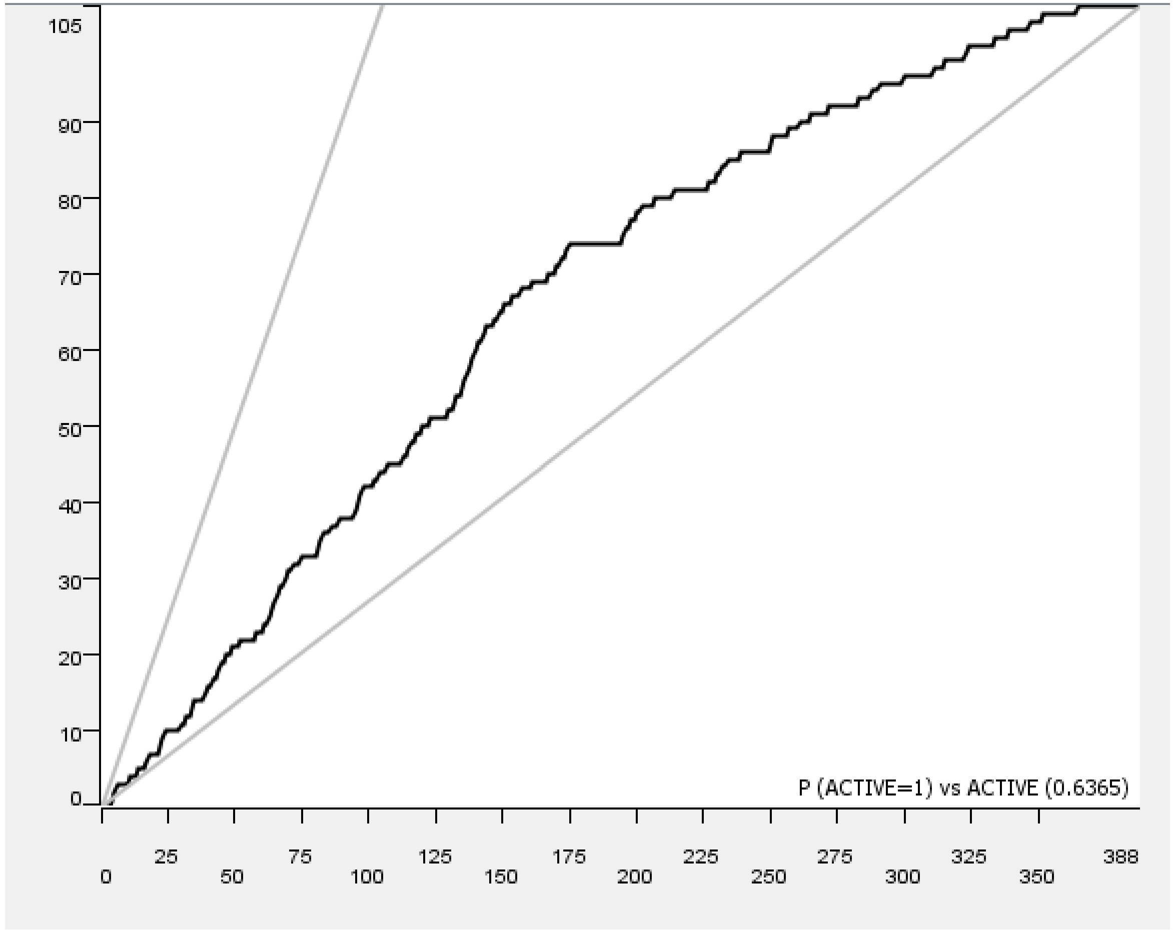

N=388

Top 10% Mean IC50	1,900 nM

	Predicted Active	Predicted Inactive
Active	32	73
Inactive	40	243

Conclusion - PNN

- Across three random seeds, the % error that lead to a significant enrichment failure and/or top 10% IC50 increase in the retrospective test was 25%, 5% and 25% for each unique random seed.
- Parameterization did not alter one of the failure points, and moved the point of failure from 20% to 25% in the last random split. However, reparameterization did rescue a significant failure in this instance as the first random split went from 5% to 25%.

TYRO3

TYRO3 NBN Design

- Rules - $RELATION$ = "=" OR $IC50$ > 999 OR $IC50$ < 10 => TRUE
- Sort ascending and remove duplicates
- DefGood scanned between 5 and 35 nM
- 80-20 split for both actives and inactives
- NBN – ECFP4 used Classifier where Prediction was Active = True
- Resulted in 277 compounds after cleaning

<5 nM DefGood in TYRO3

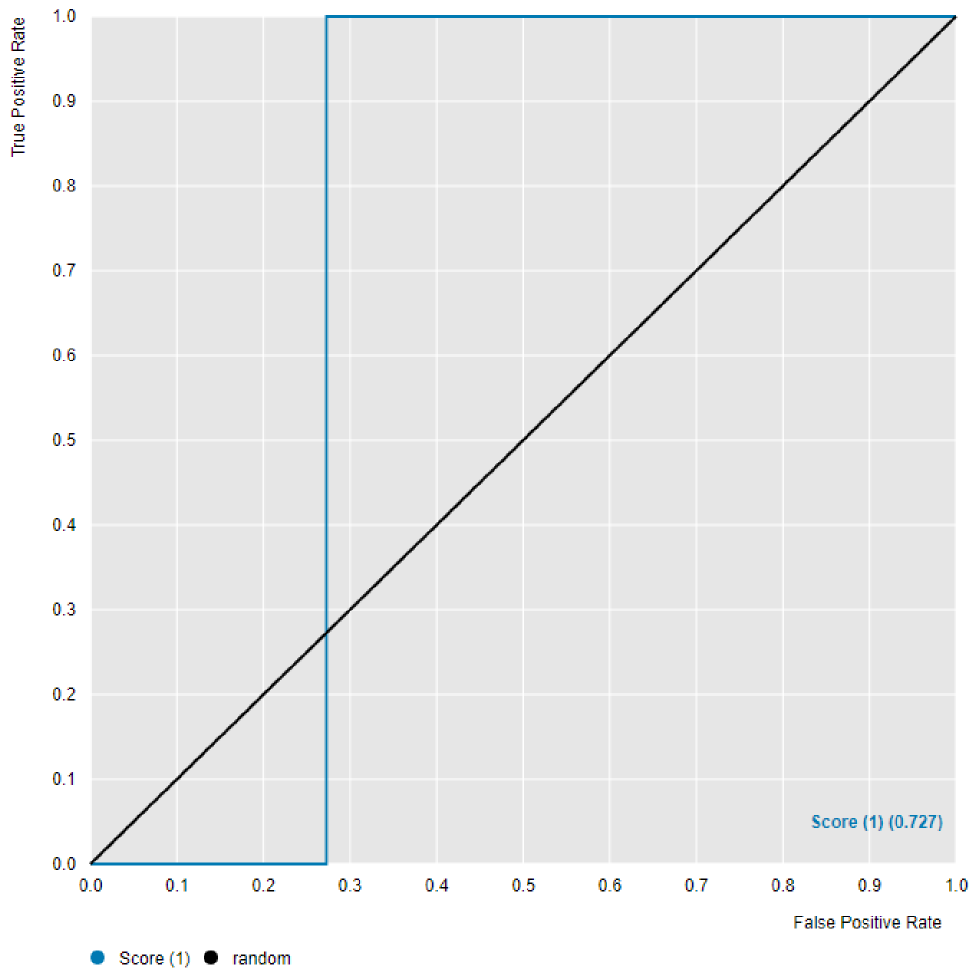

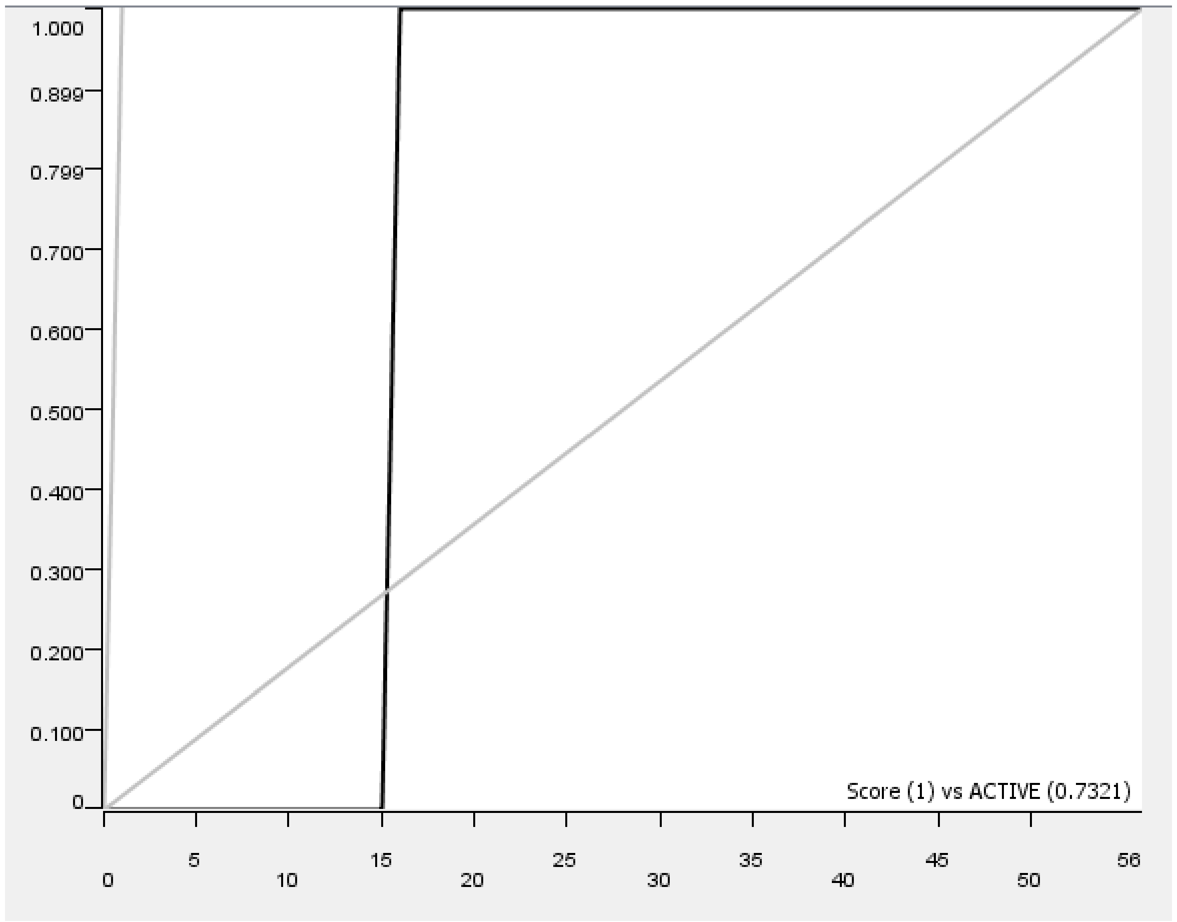

N=56

**Top 10% Mean IC50**	**61.0 nM**

	Predicted Active	Predicted Inactive
Active	0	1
Inactive	1	54

<10 nM DefGood in TYRO3

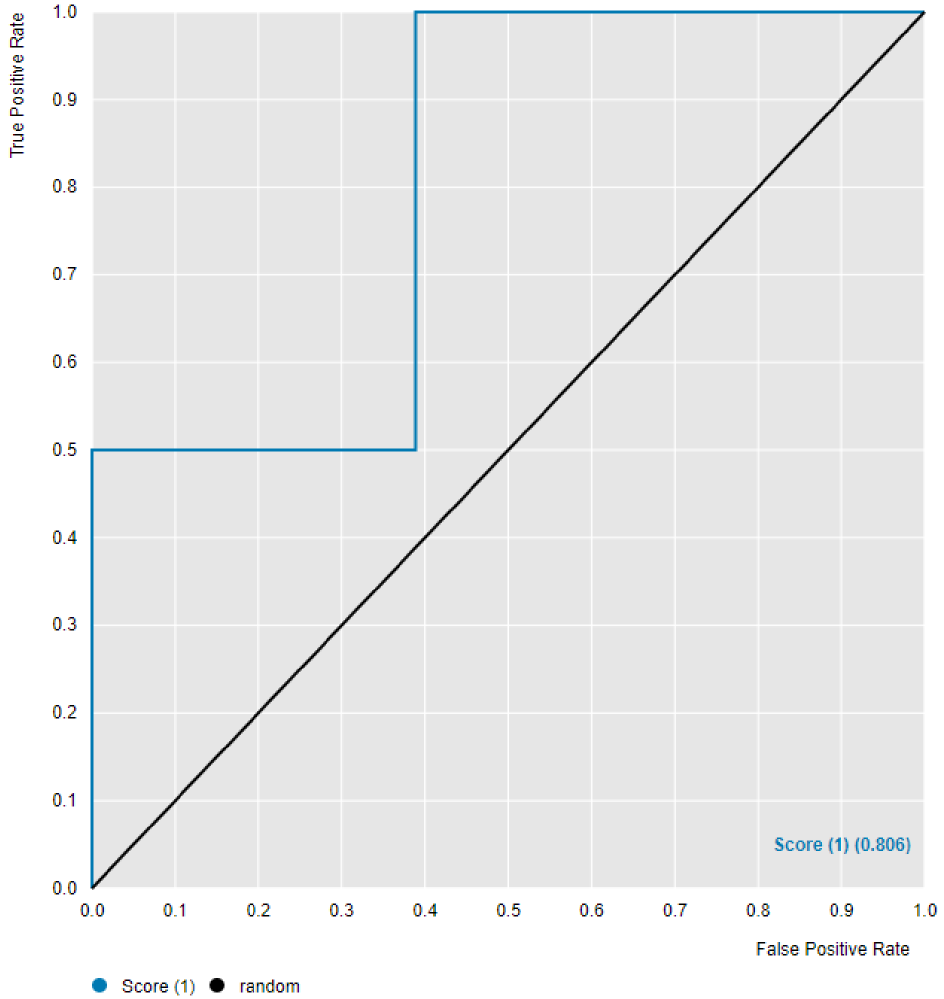

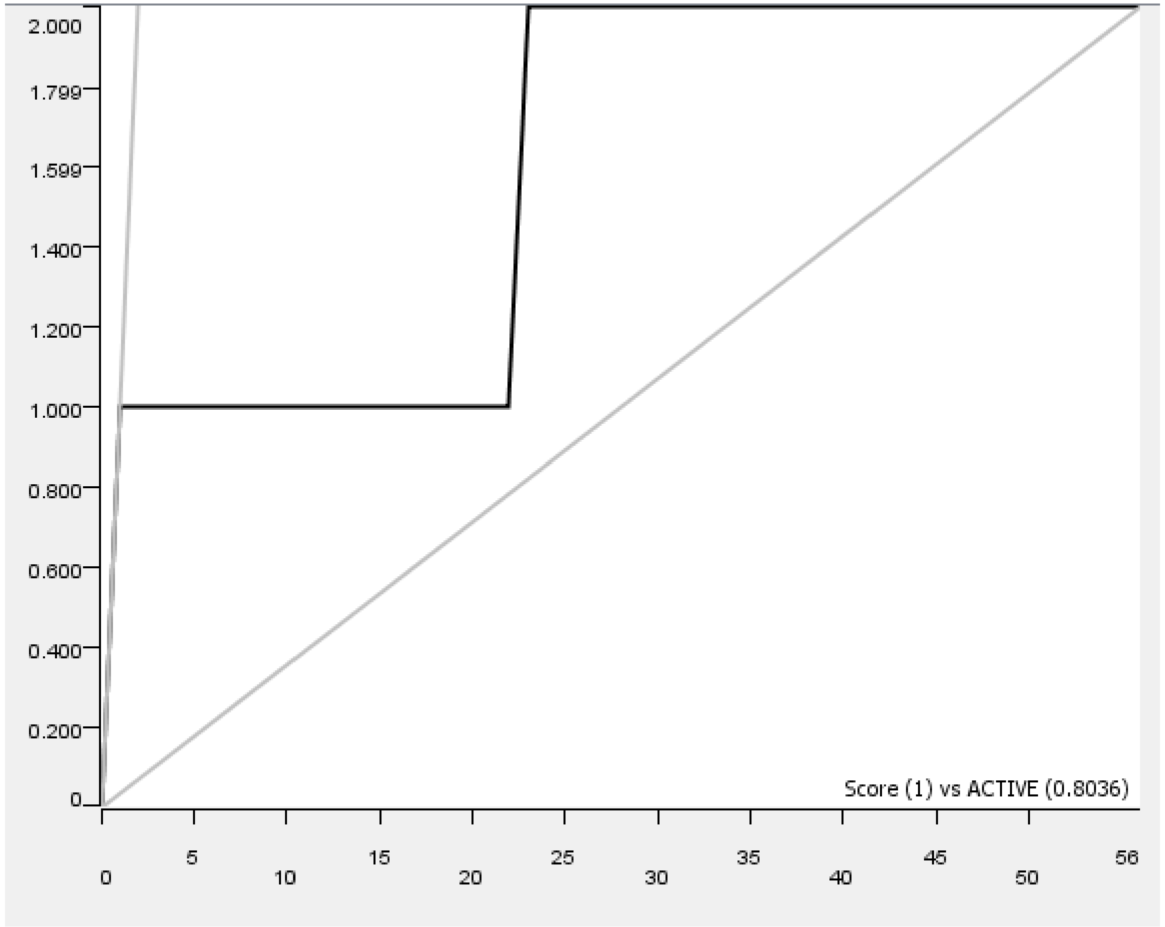

N=56

**Top 10% Mean IC50**	**109 nM**

	**Predicted Active**	**Predicted Inactive**
Active	1	1
Inactive	18	36

<15 nM DefGood in TYRO3

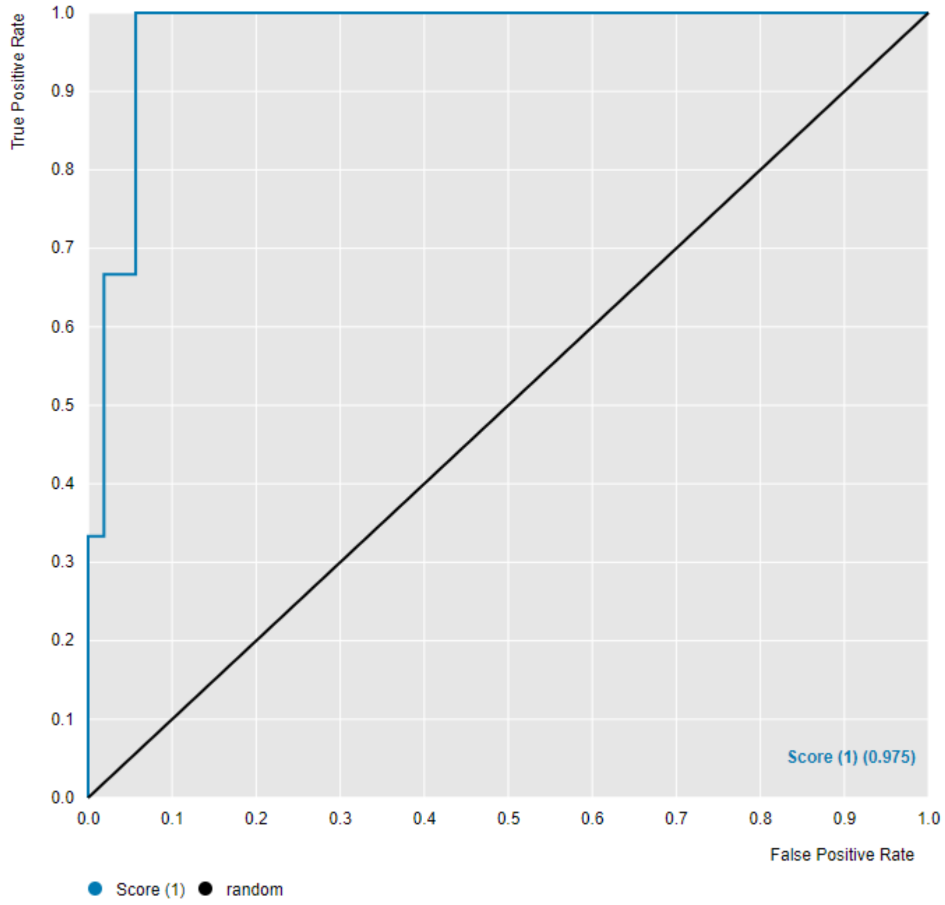

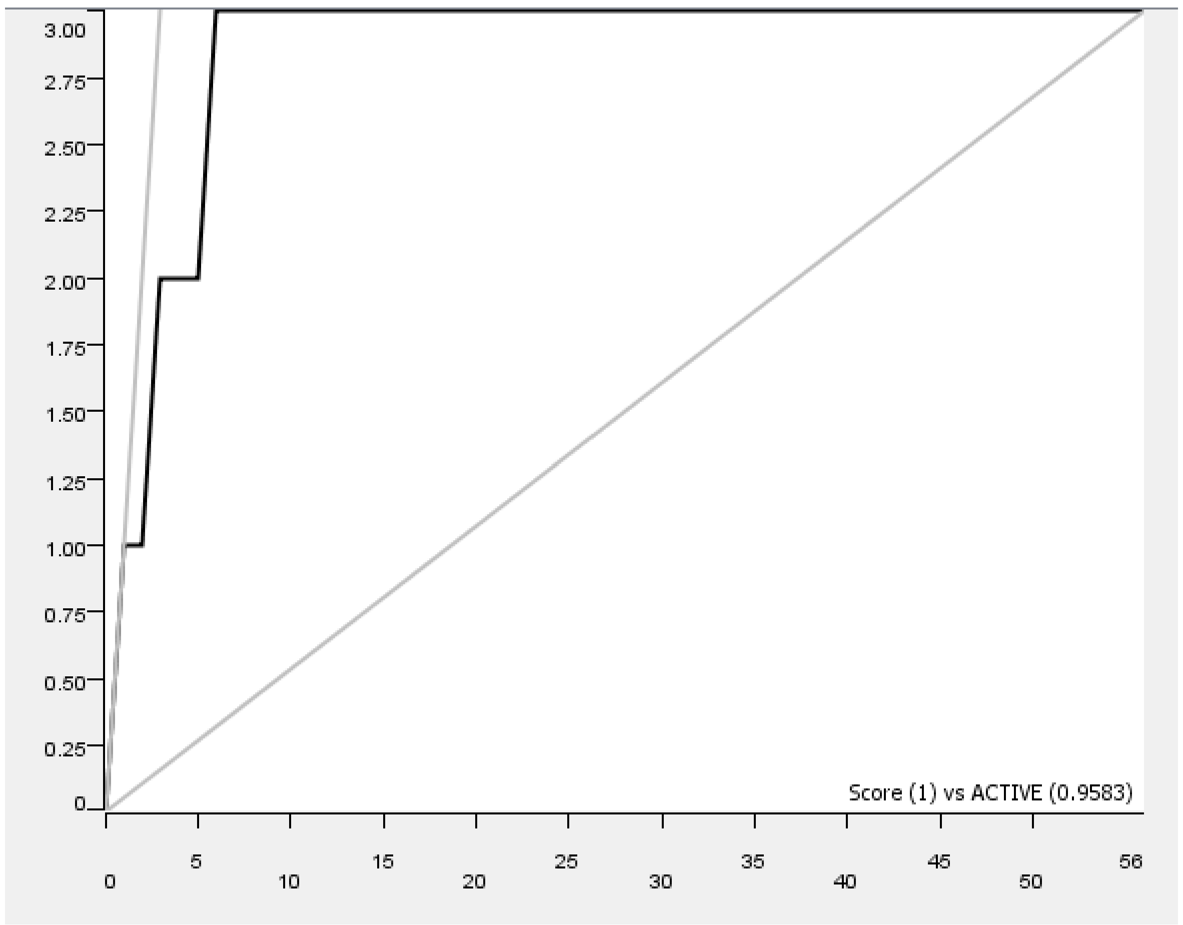

N=56

Top 10% Mean IC50	87.4 nM

	Predicted Active	Predicted Inactive
Active	3	0
Inactive	4	49

<20 nM DefGood in TYRO3

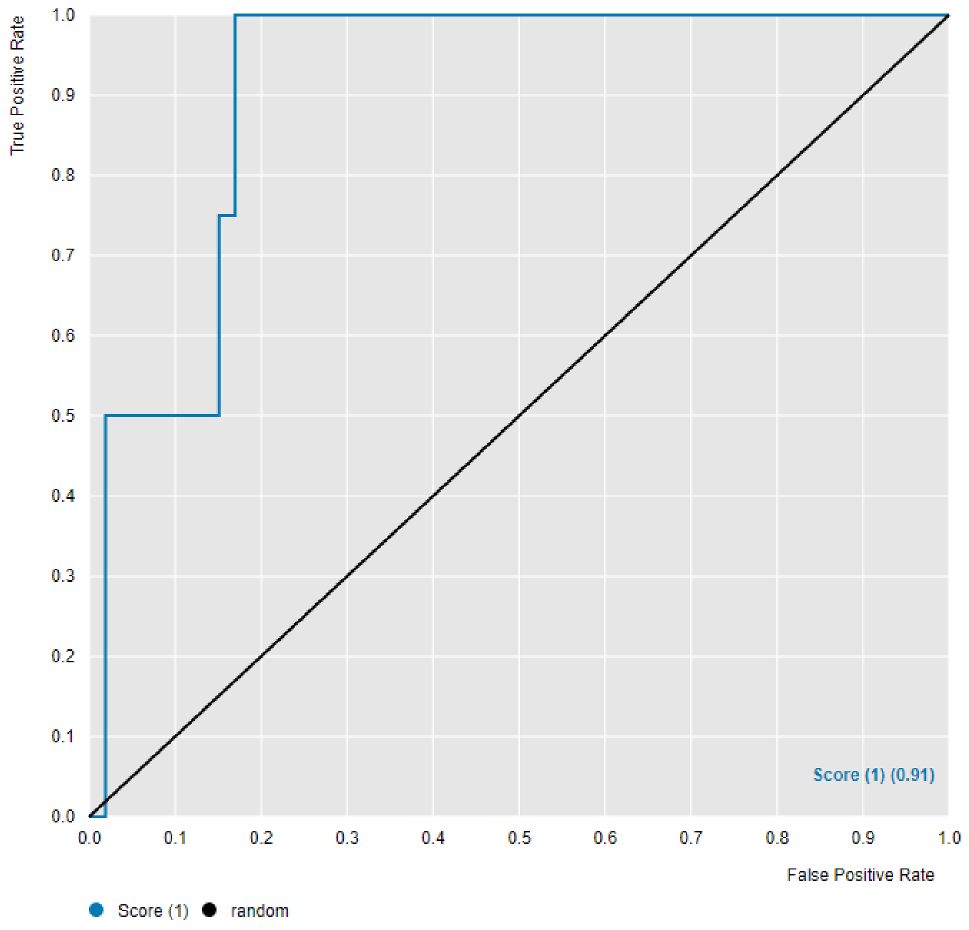

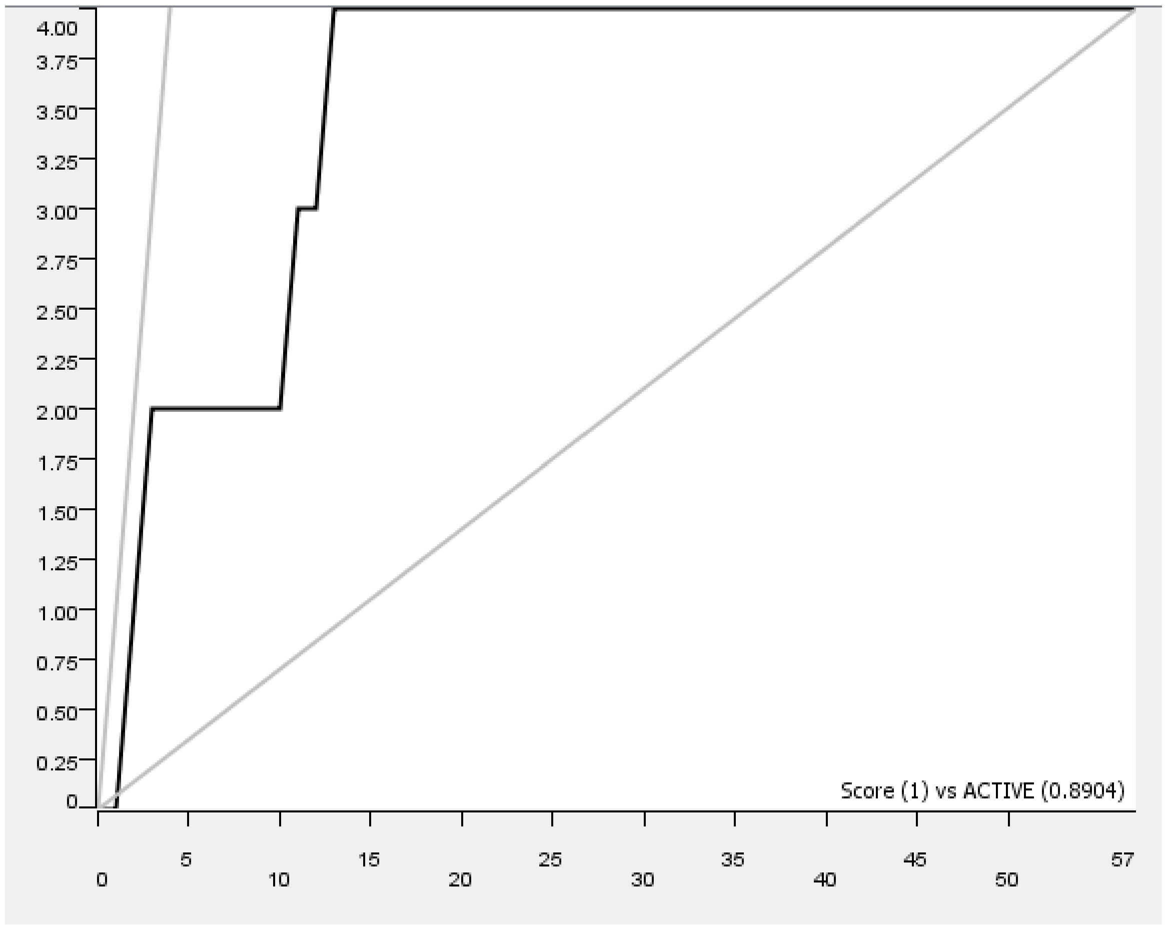

N=56

Top 10% Mean IC50	54.8 nM

	Predicted Active	Predicted Inactive
Active	4	0
Inactive	10	43

<25 nM DefGood in TYRO3

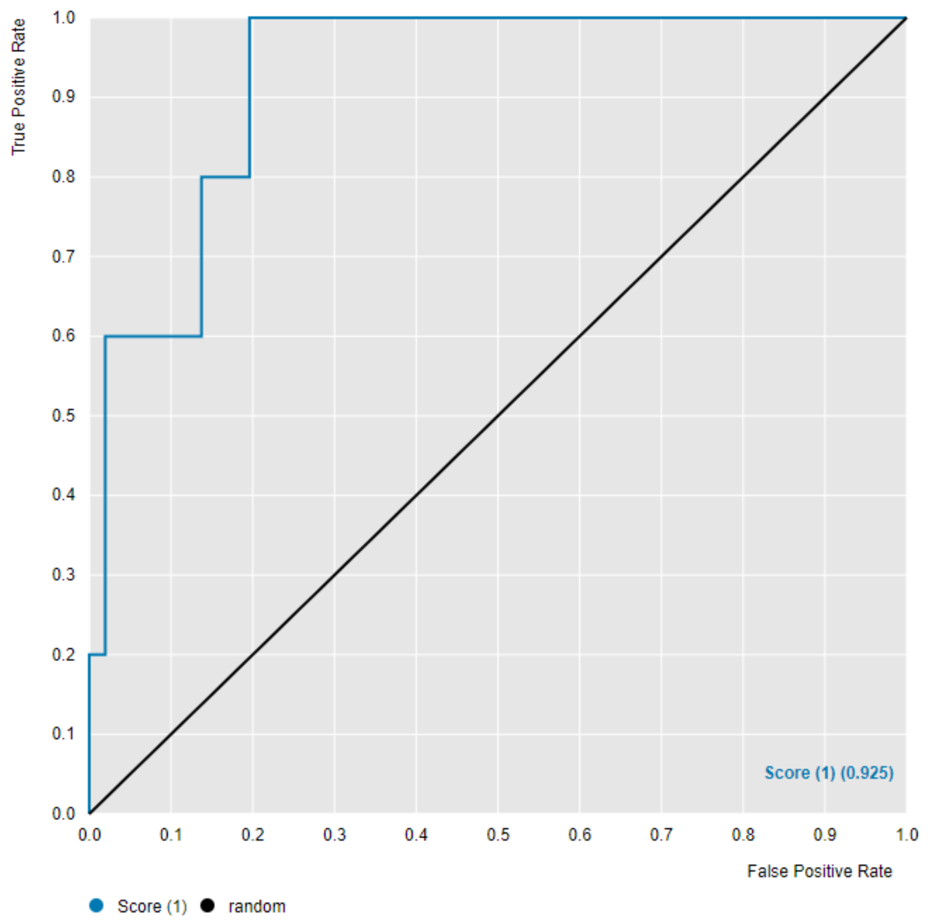

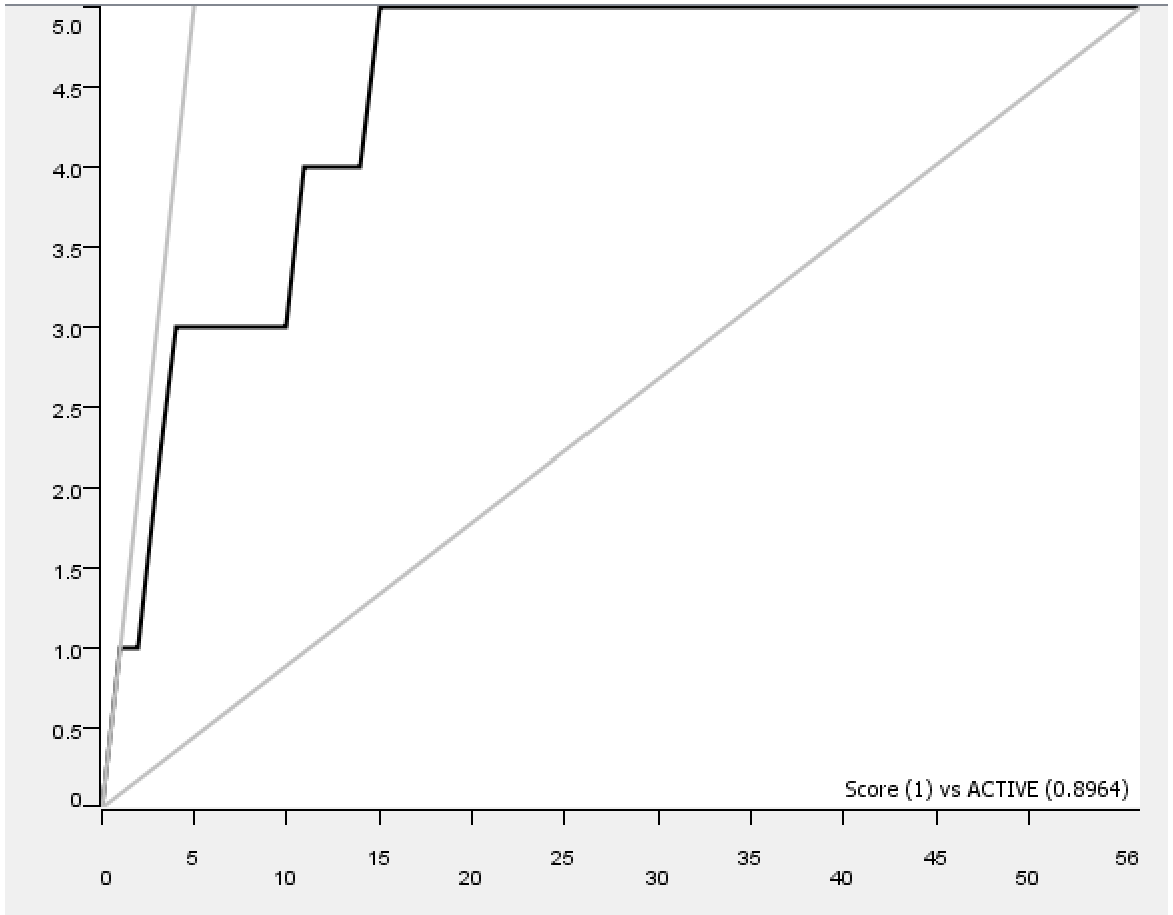

N=56

Top 10% Mean IC50	54.7 nM

	Predicted Active	Predicted Inactive
Active	5	0
Inactive	16	35

<35 nM DefGood in TYRO3

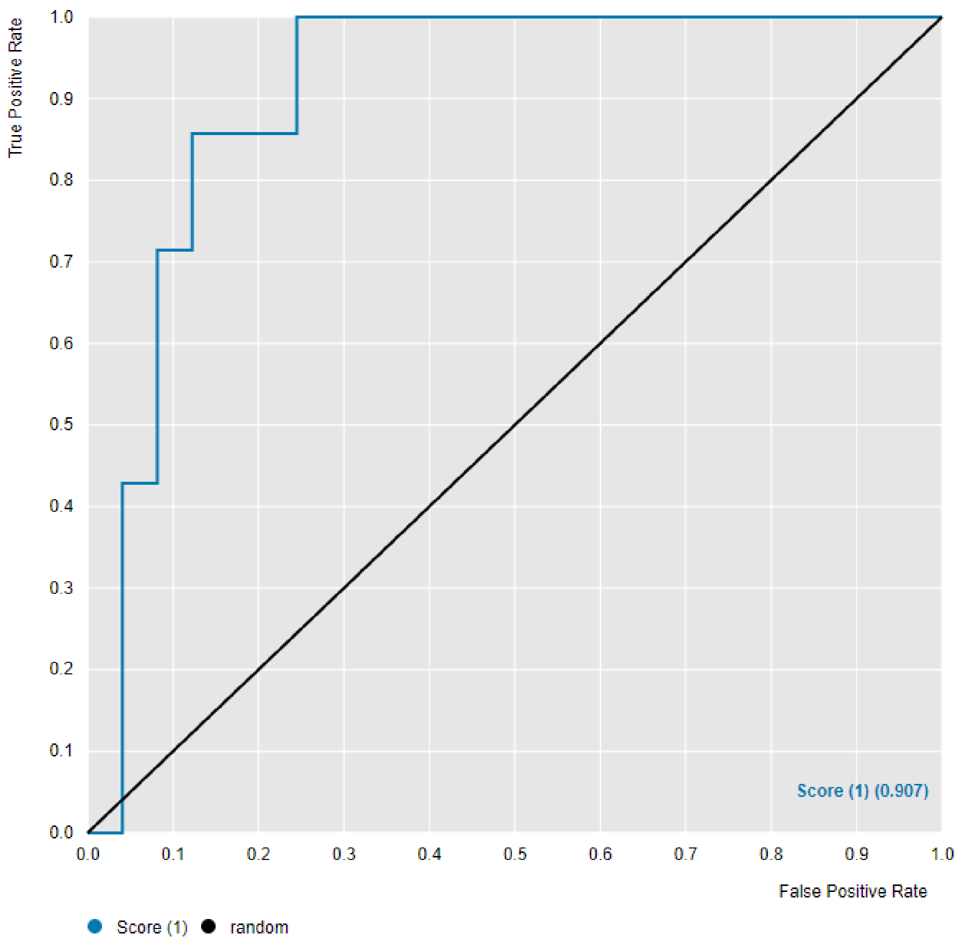

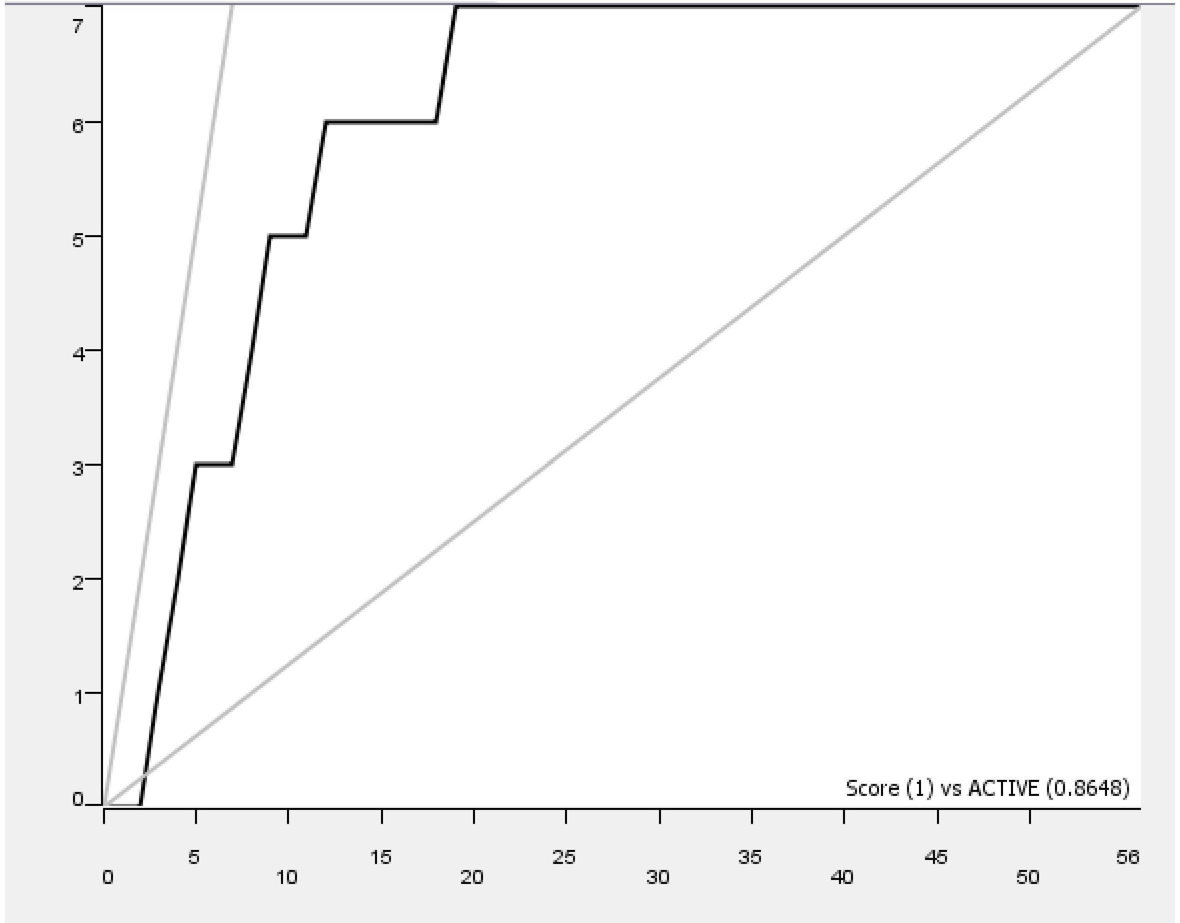

N=56

Top 10% Mean IC50	48.6 nM

	Predicted Active	Predicted Inactive
Active	7	0
Inactive	16	33

Decision on DefGood

- <25 nM has the best performance with regard to enrichment and mean top 10% IC50.

RF - <25 nM DefGood in TYRO3

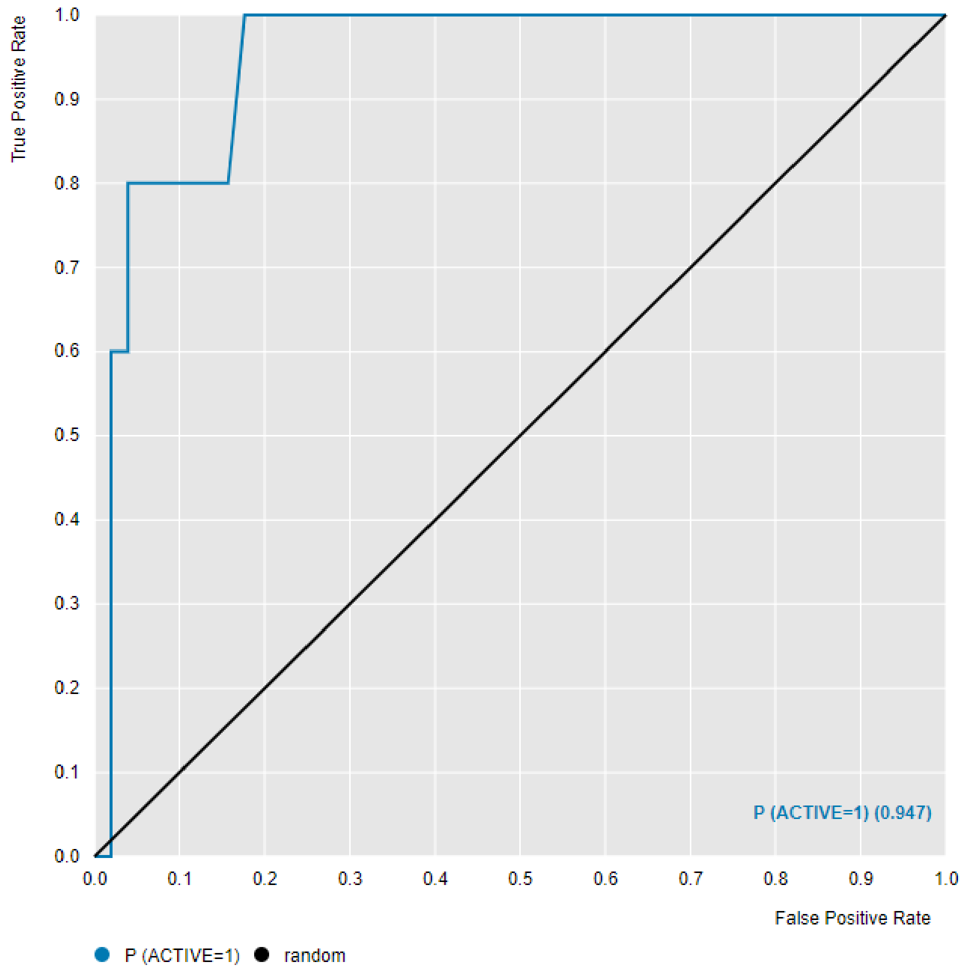

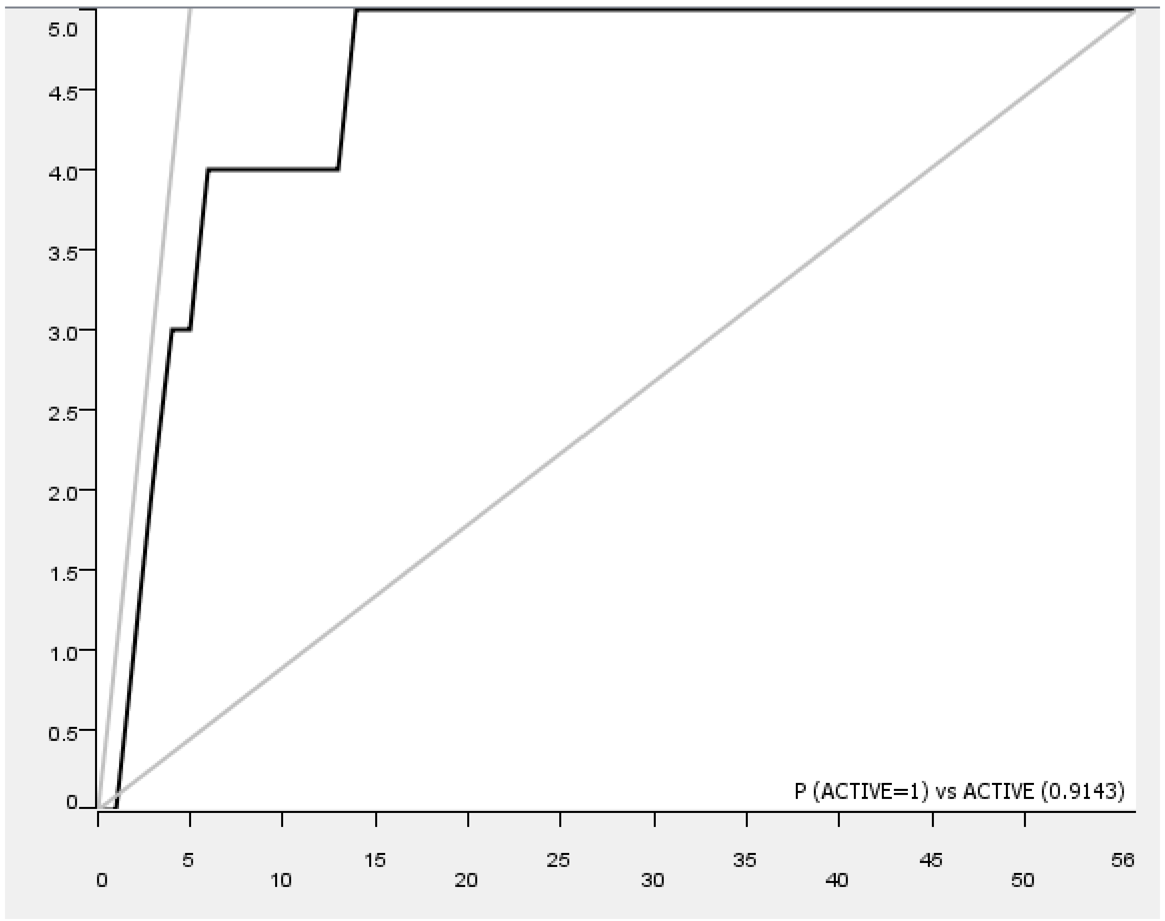

N=56

Top 10% Mean IC50	16.5 nM

	Predicted Active	Predicted Inactive
Active	3	2
Inactive	2	49

Decision on DefGood - RF

- Work in ALK showed DefGood in the NBN was similar to DefGood in an RF. Therefore, this value for DefGood from the NBN design was used as DefGood.

PNN - <25 nM DefGood in TYRO3

- Properties List (46 calculatable properties were used)
- Parameterization (Global Accuracy for Activity at a 25 nM decision value was used, and a 5-fold stratified sampling cross validation was used to parameterize theta minus and theta plus) Theta Minus = 0.75 and Theta Plus = 0.05. This parameterization is not allowed in the PNN algorithm, and PNN was not used as a result

NBN Error Tolerance- <25 nM DefGood in TYRO3

- 0-50% absolute error

<25 nM DefGood in TYRO3, 5% error; Random seed = 1515533876005

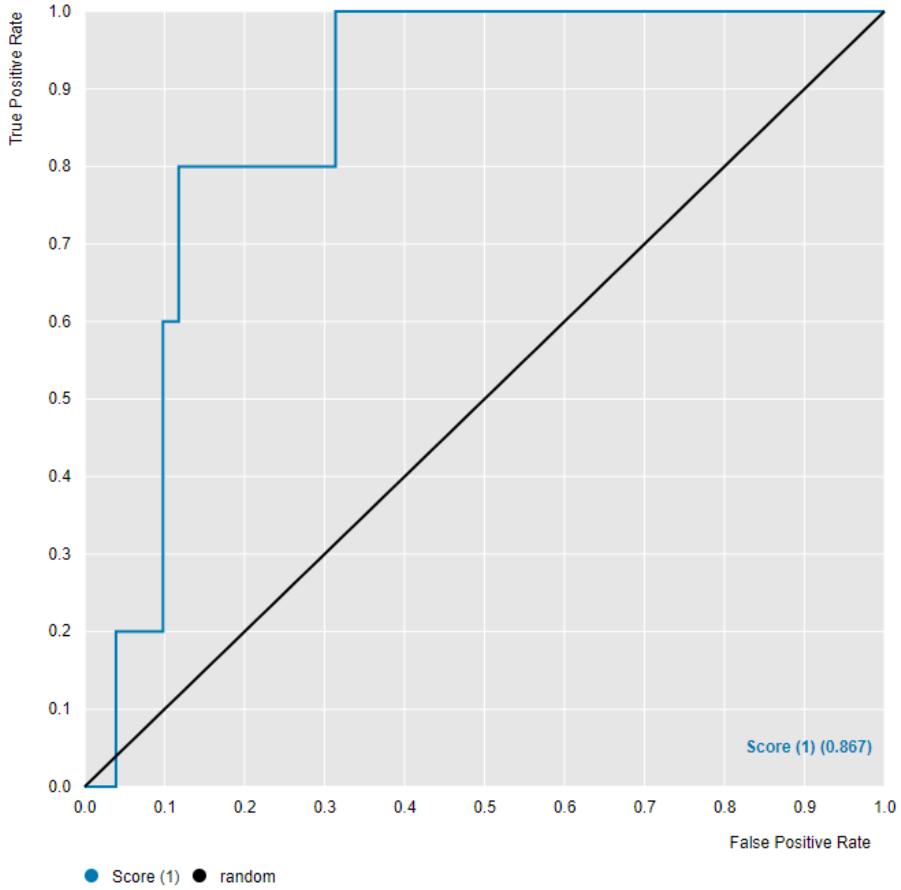

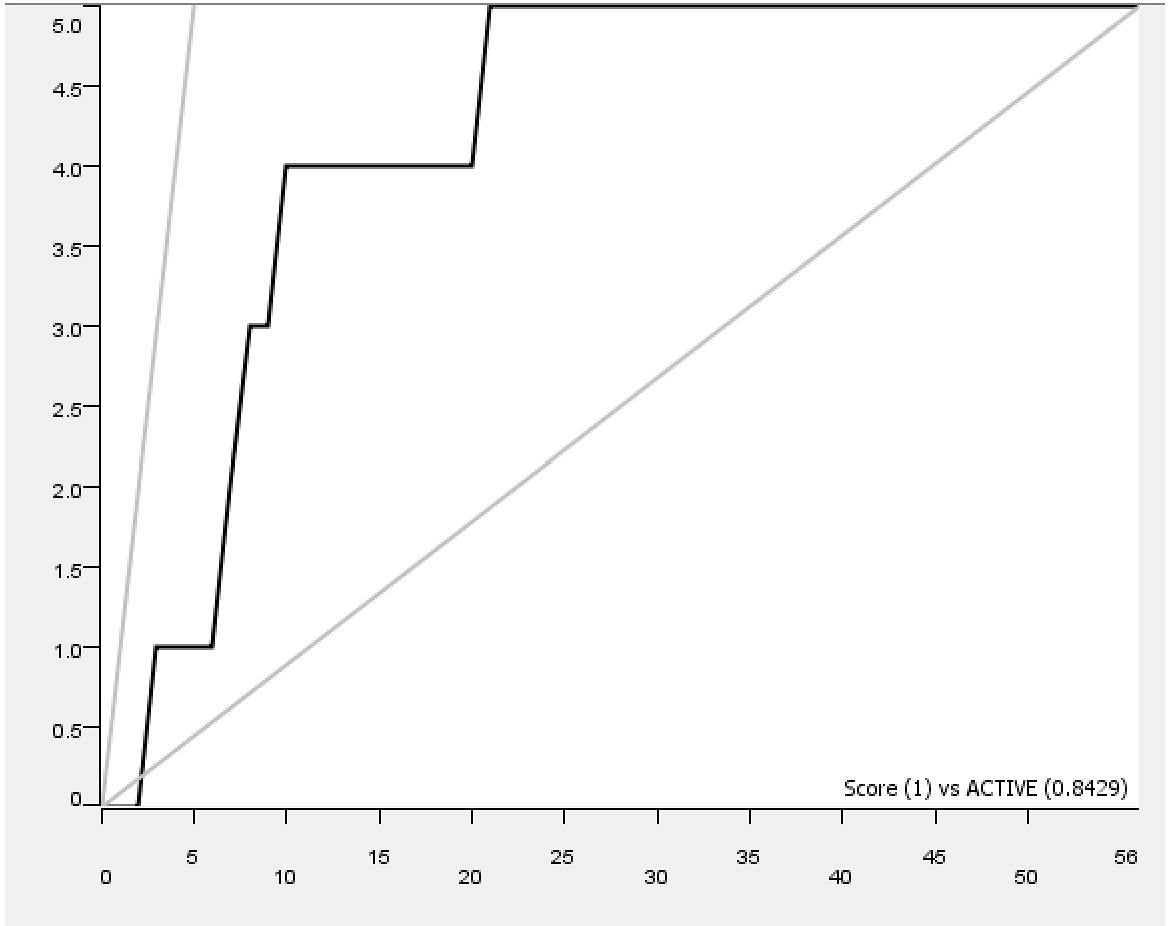

N=56

Top 10% Mean IC50	45.2 nM

	Predicted Active	Predicted Inactive
Active	5	0
Inactive	16	35

<25 nM DefGood in TYRO3, 10% error; Random seed = 1515533876005

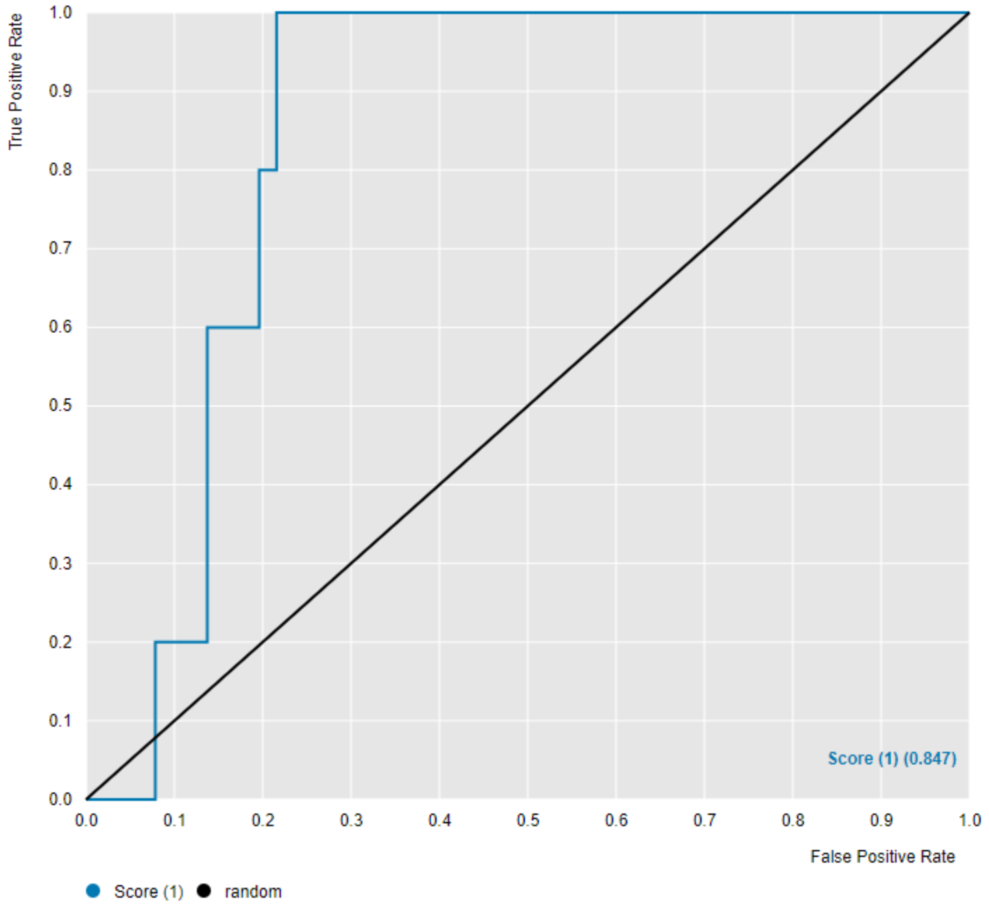

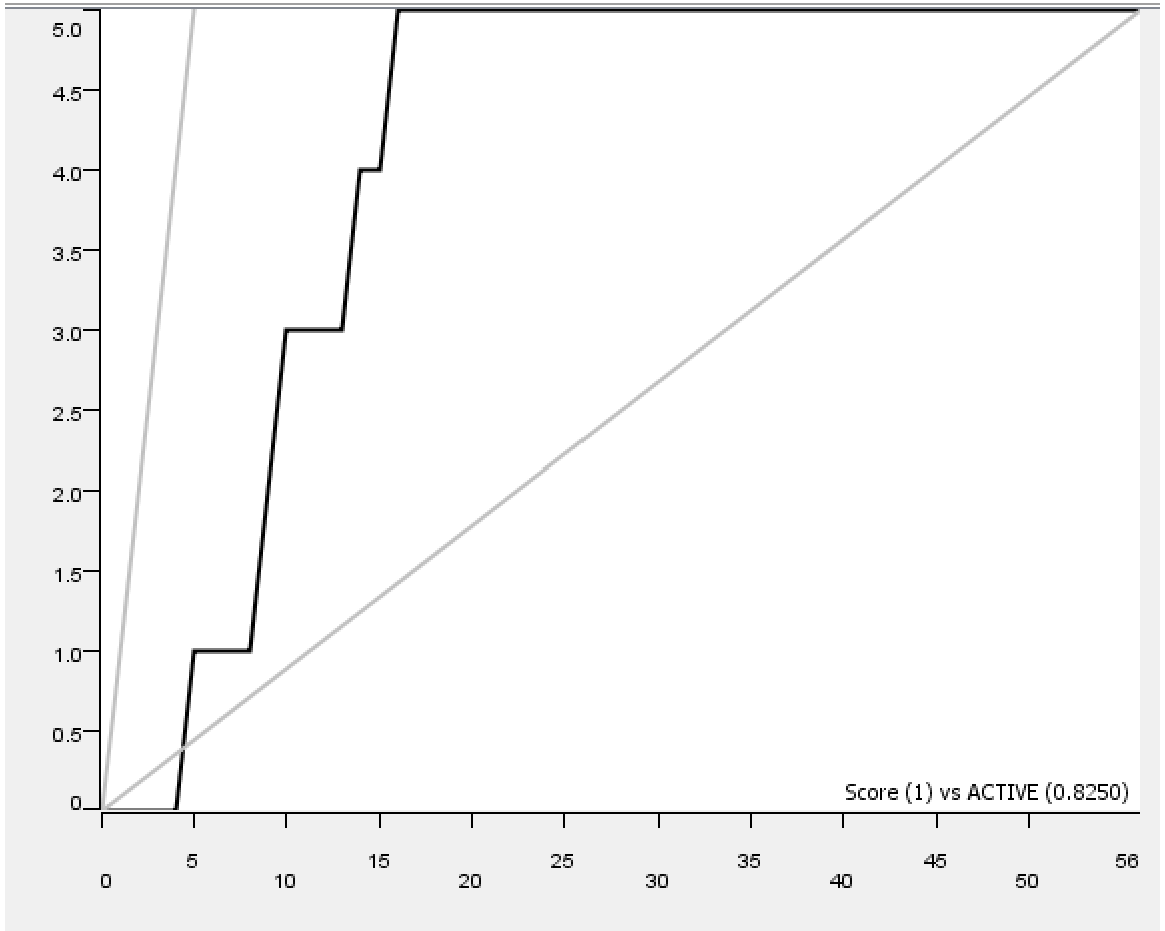

N=56

Top 10% Mean IC50	39.1 nM

	Predicted Active	Predicted Inactive
Active	5	0
Inactive	23	28

<25 nM DefGood in TYRO3, 15% error;

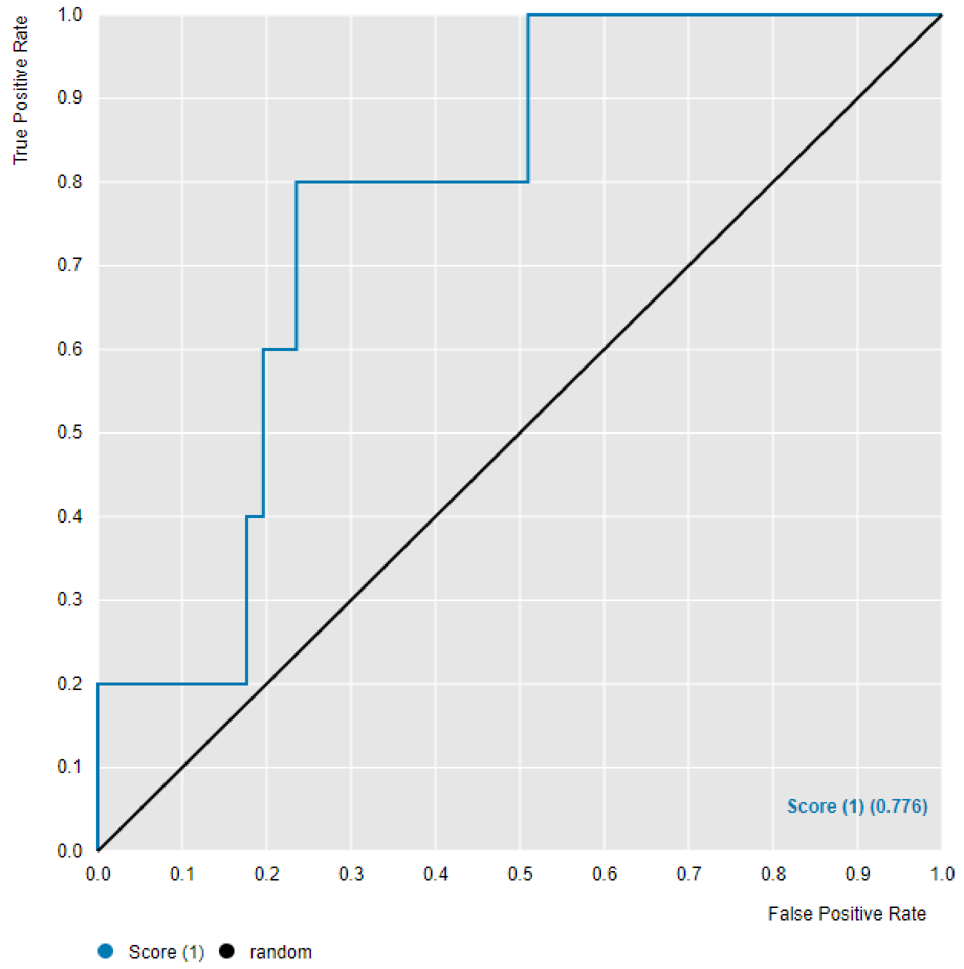

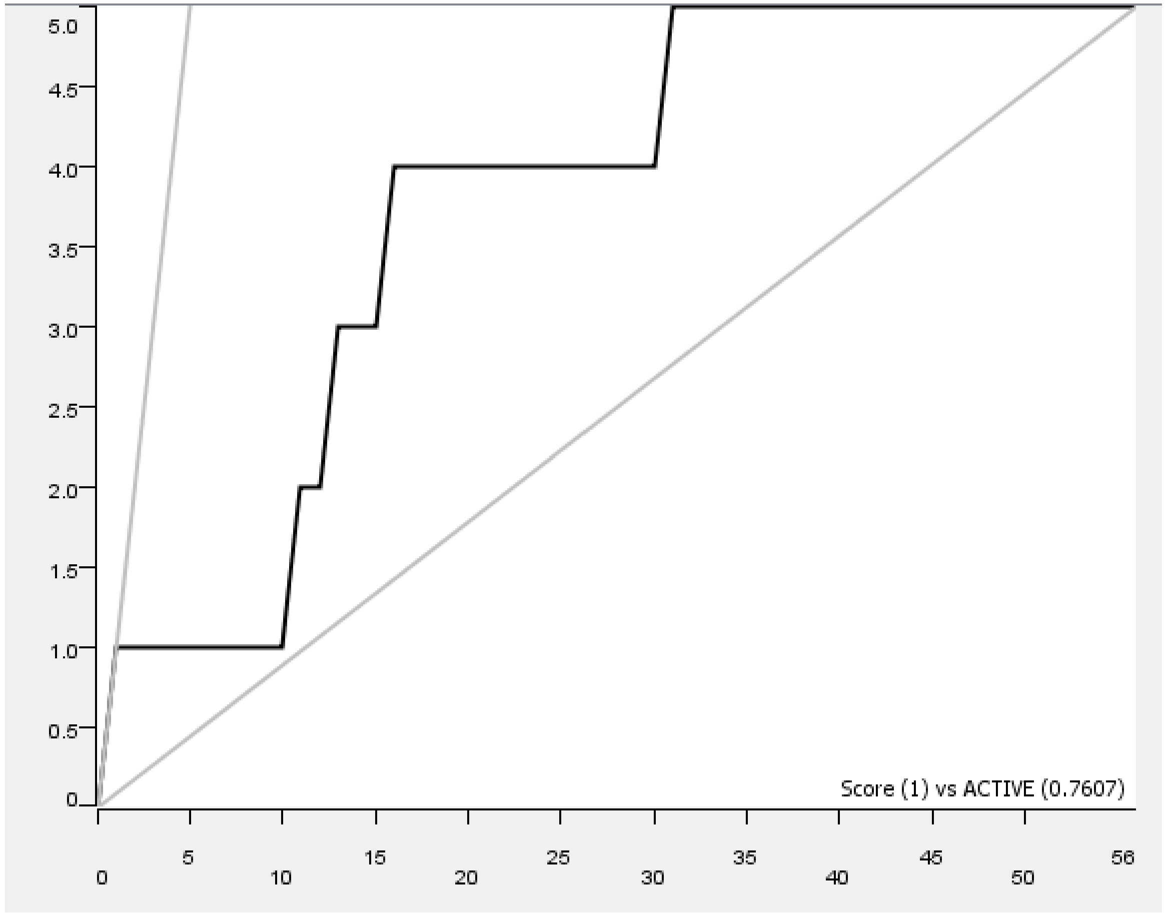

N=56

**Top 10% Mean IC50**	**39.1 nM**

	**Predicted Active**	**Predicted Inactive**
Active	4	1
Inactive	18	33

<25 nM DefGood in TYRO3, 20% error;

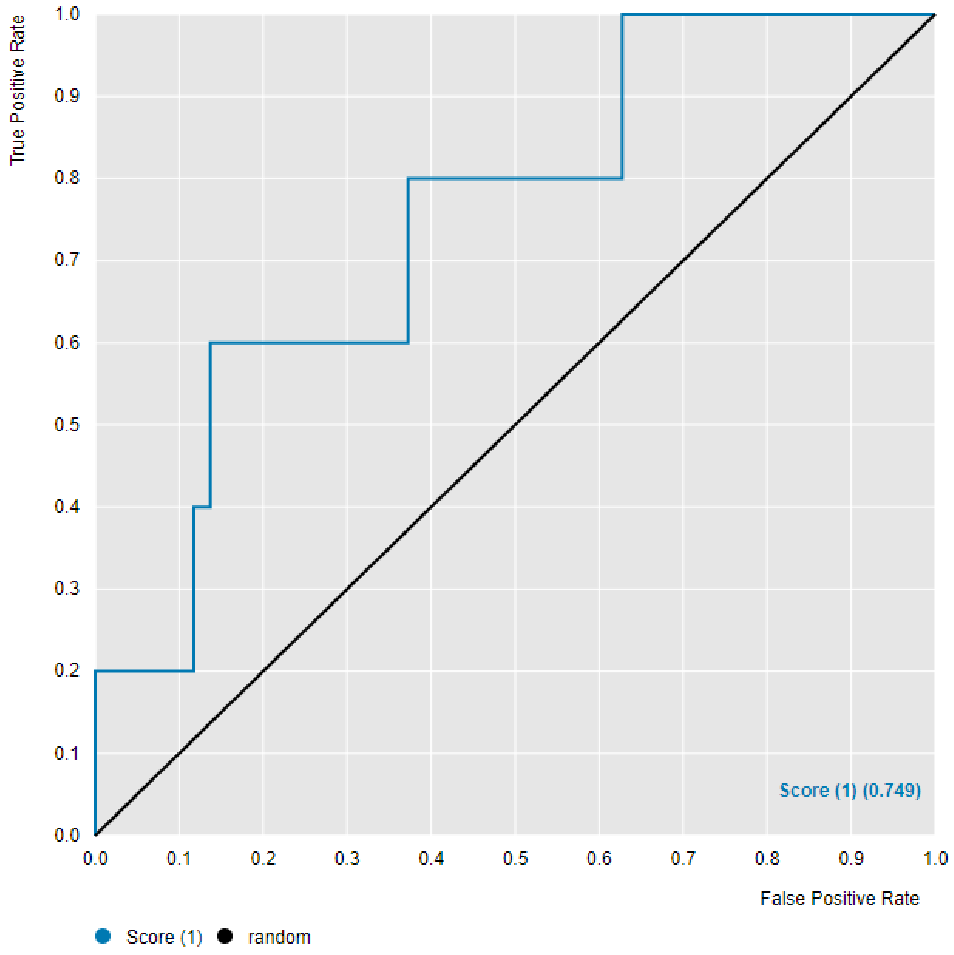

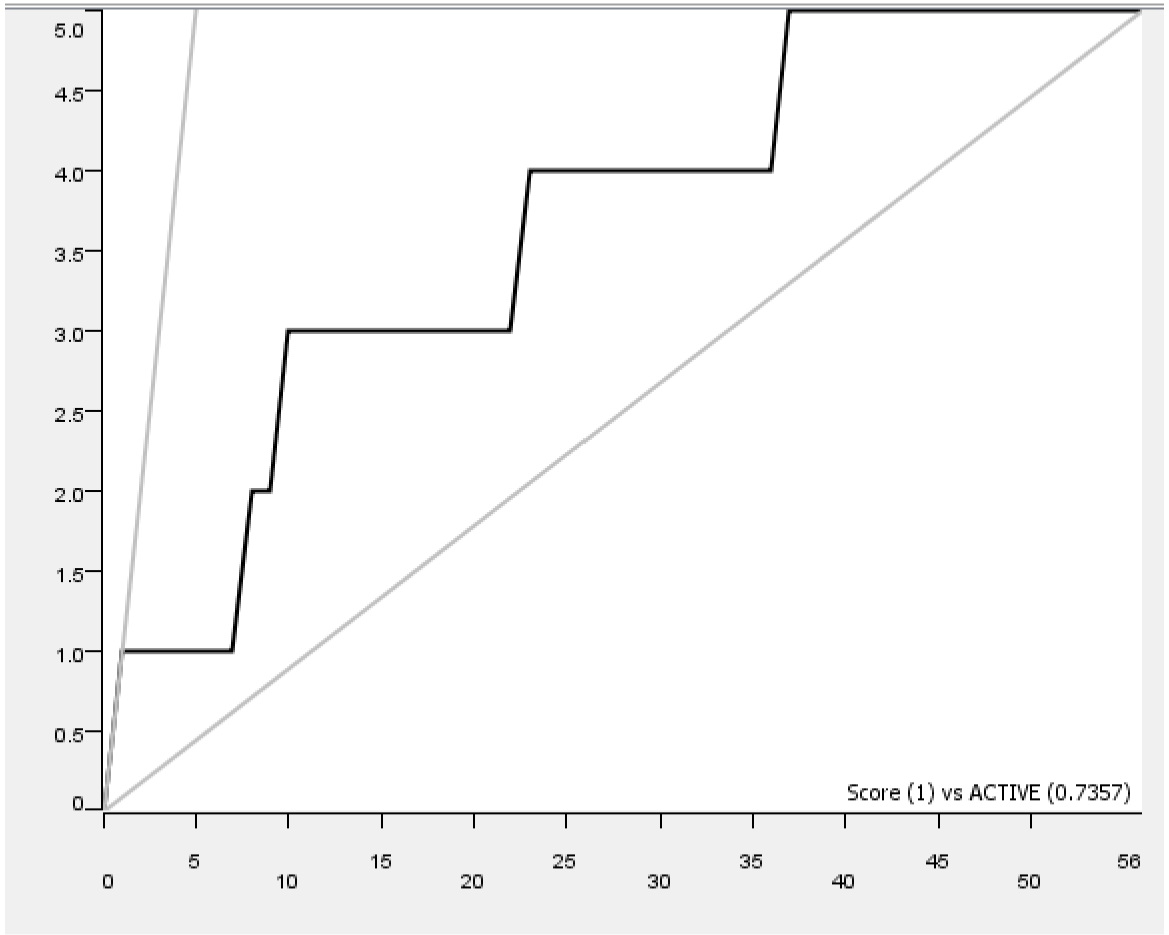

N=56

Top 10% Mean IC50	39.1 nM

	Predicted Active	Predicted Inactive
Active	4	1
Inactive	22	29

<25 nM DefGood in TYRO3, 25% error;

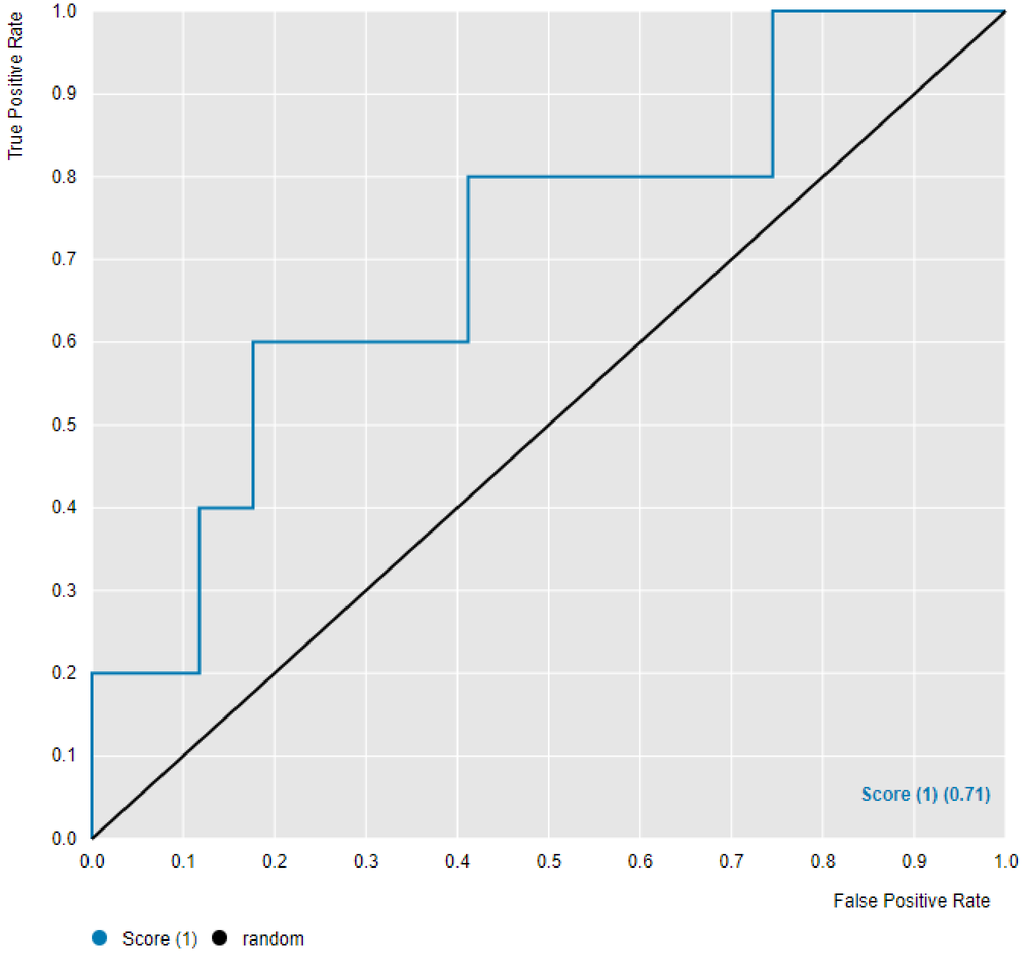

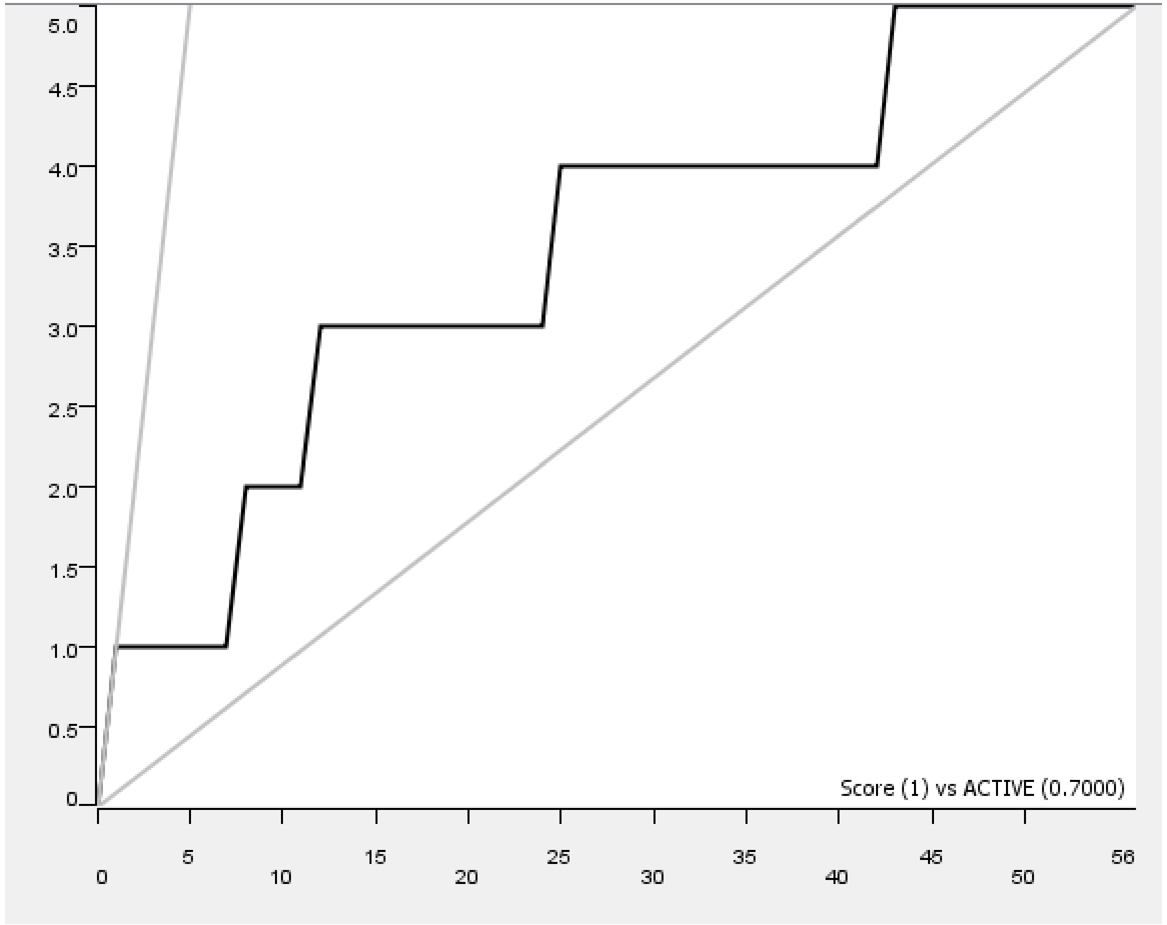

N=56

**Top 10% Mean IC50**	**39.1 nM**

	Predicted Active	Predicted Inactive
Active	4	1
Inactive	22	29

<25 nM DefGood in TYRO3, 30% error;

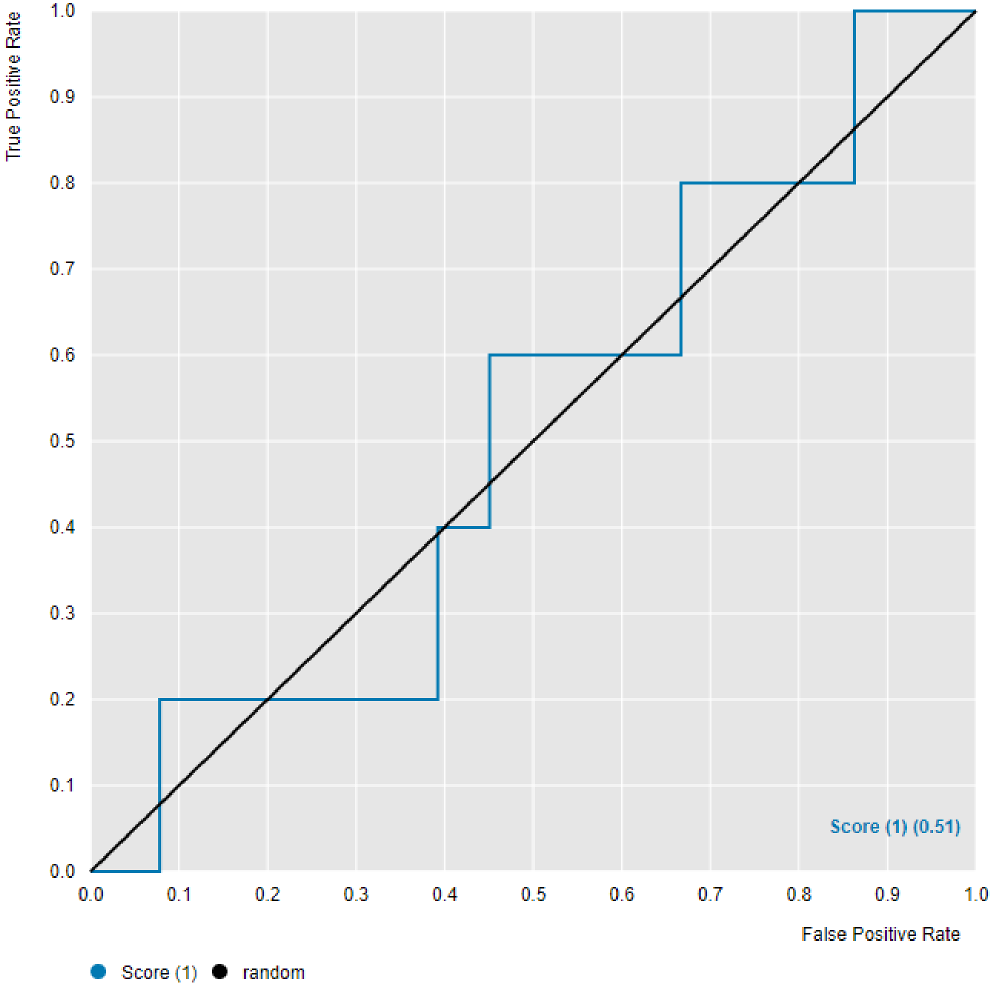

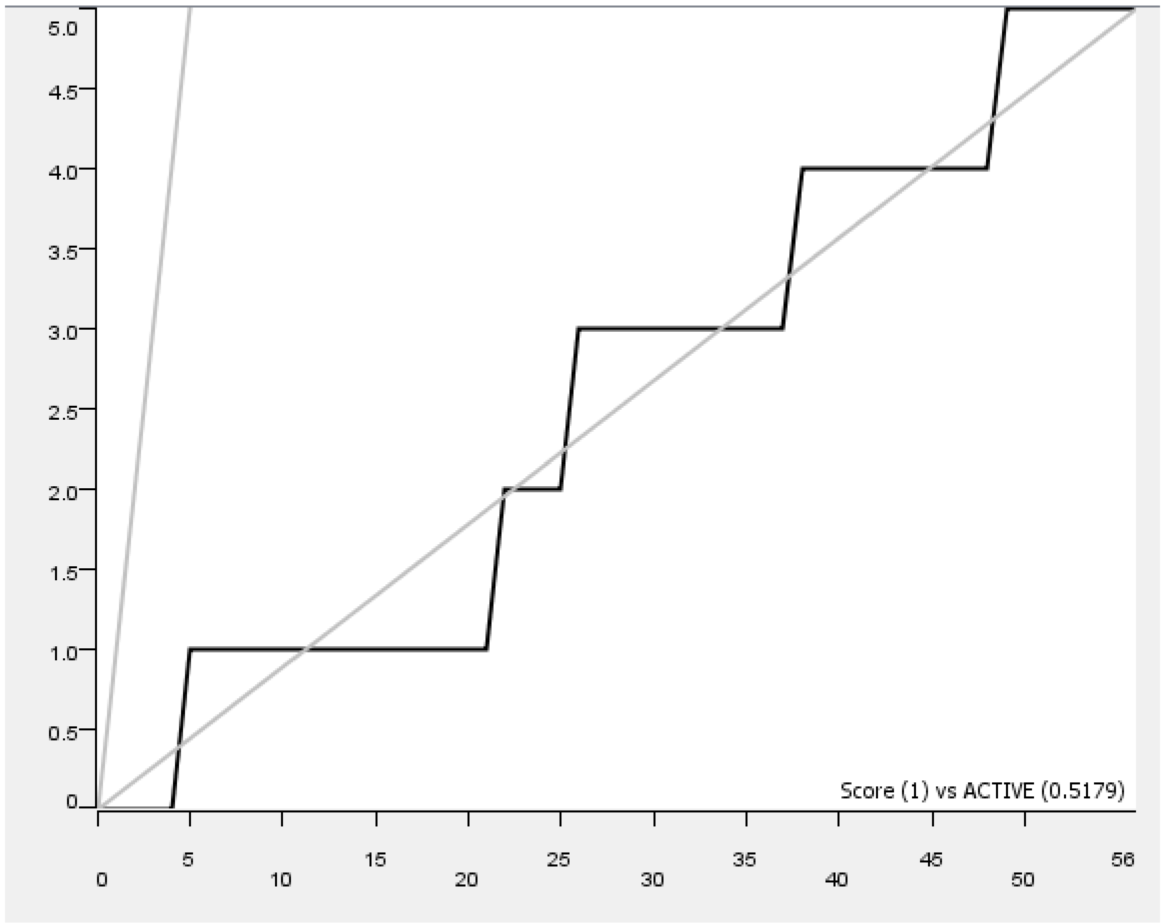

N=56

Top 10% Mean IC50	39.1 nM

	Predicted Active	Predicted Inactive
Active	2	3
Inactive	23	28

<25 nM DefGood in TYRO3, 5% error; Random seed = 429

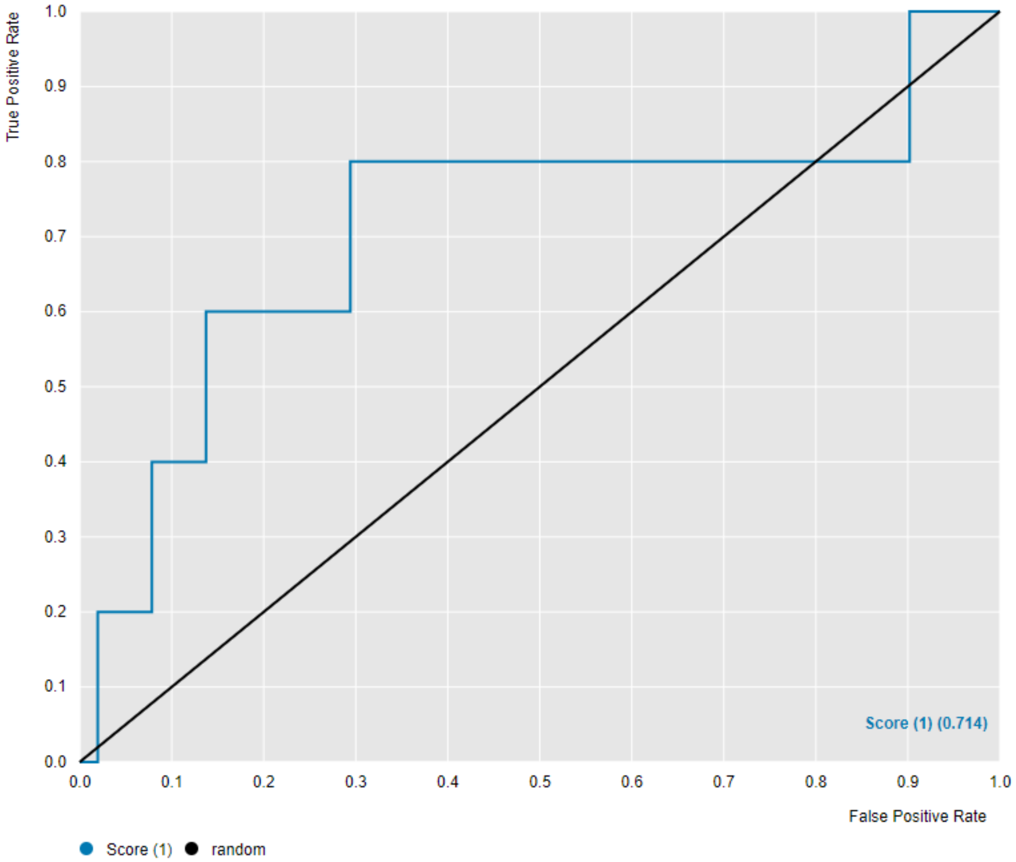

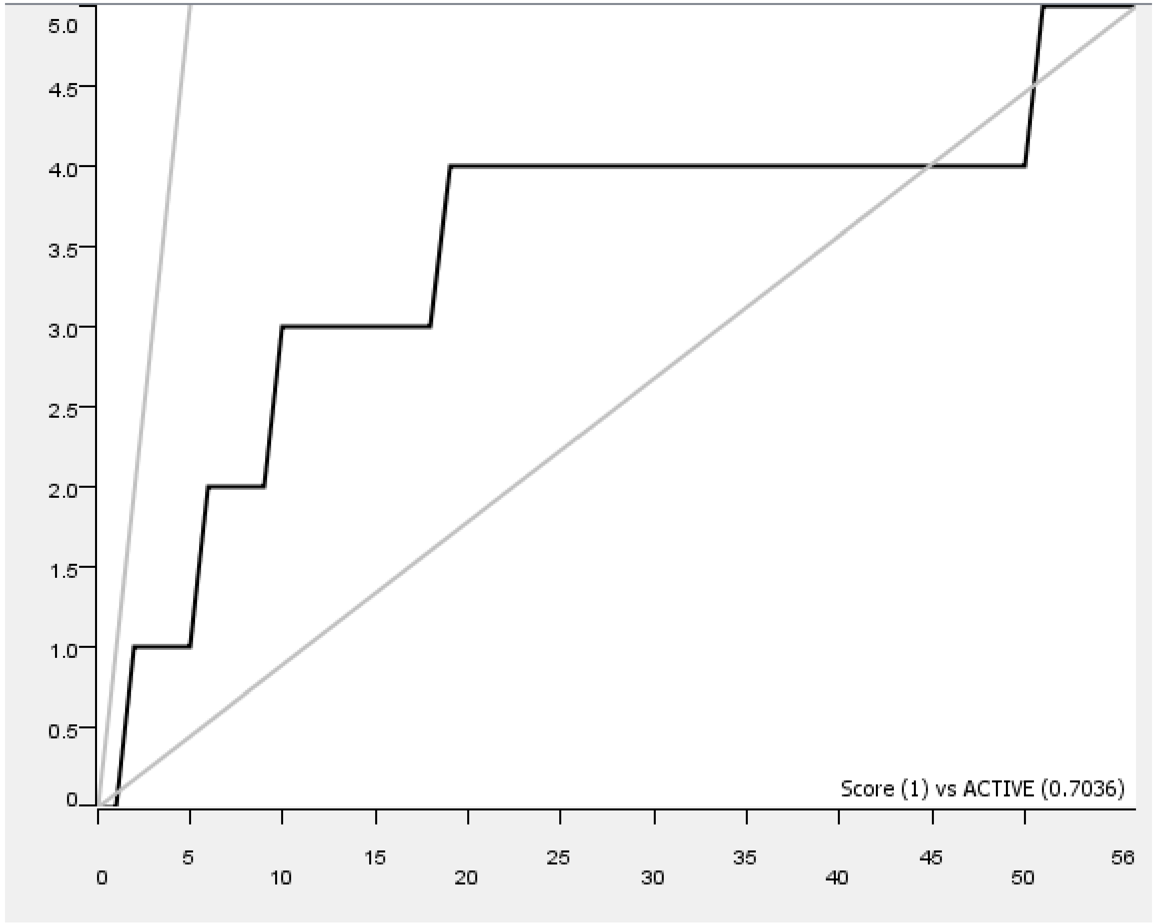

N=56

**Top 10% Mean IC50**	**137 nM**

	Predicted Active	Predicted Inactive
Active	2	3
Inactive	6	45

<25nM DefGood in TYRO3, 10% error; Random seed = 429

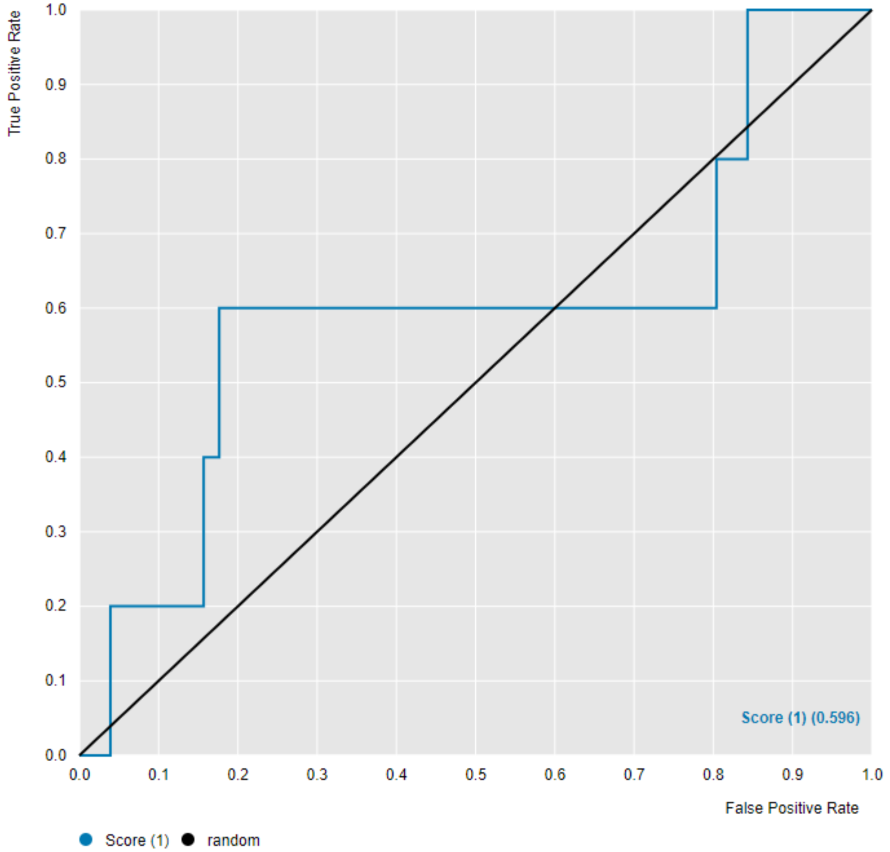

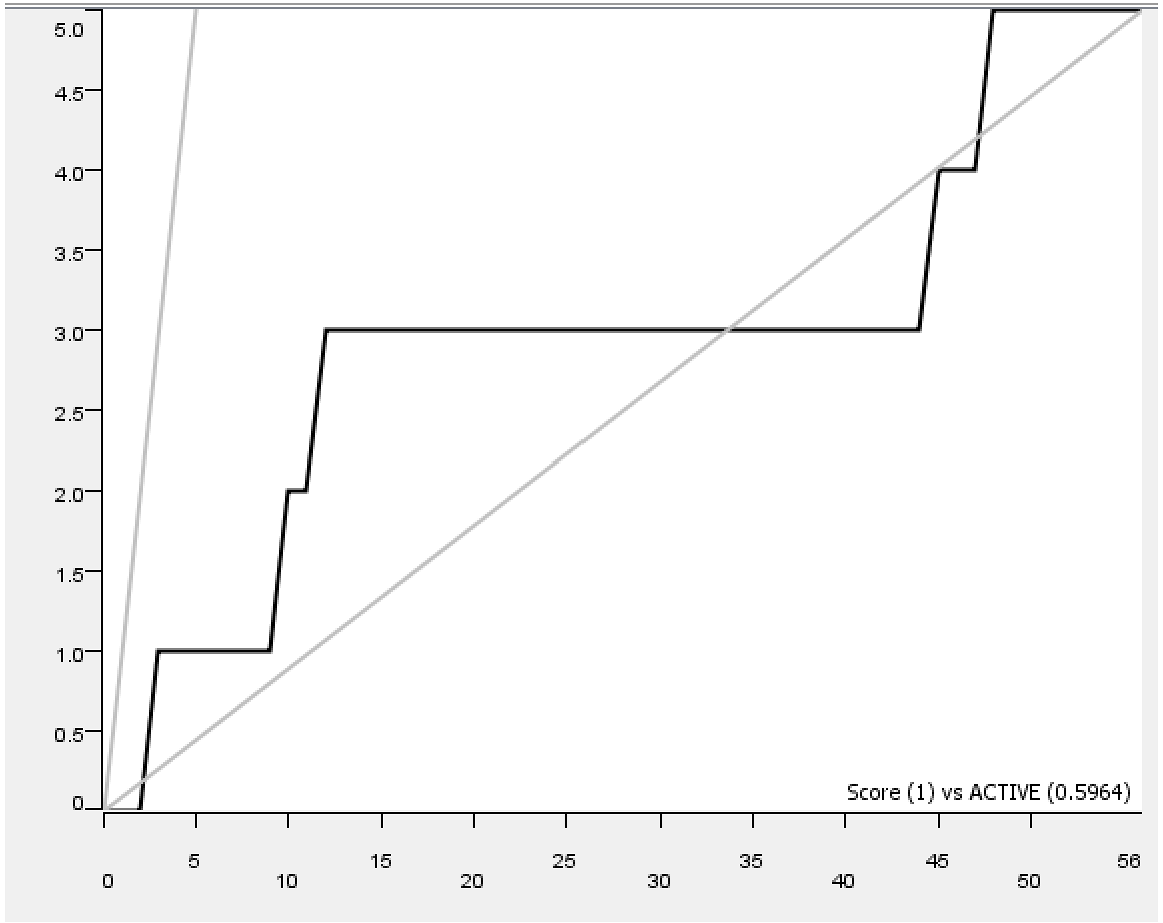

N=56

Top 10% Mean IC50	65.0 nM

	Predicted Active	Predicted Inactive
Active	3	2
Inactive	16	35

<25 nM DefGood in TYRO3, 5% error; Random seed = 121783

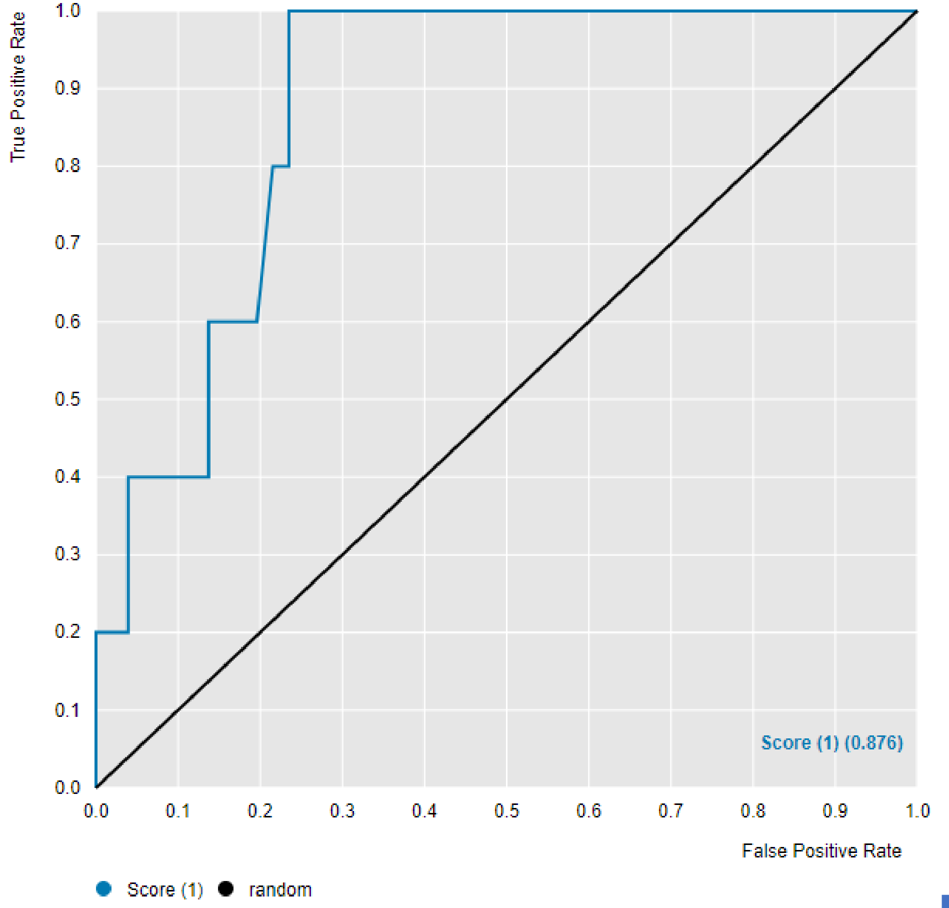

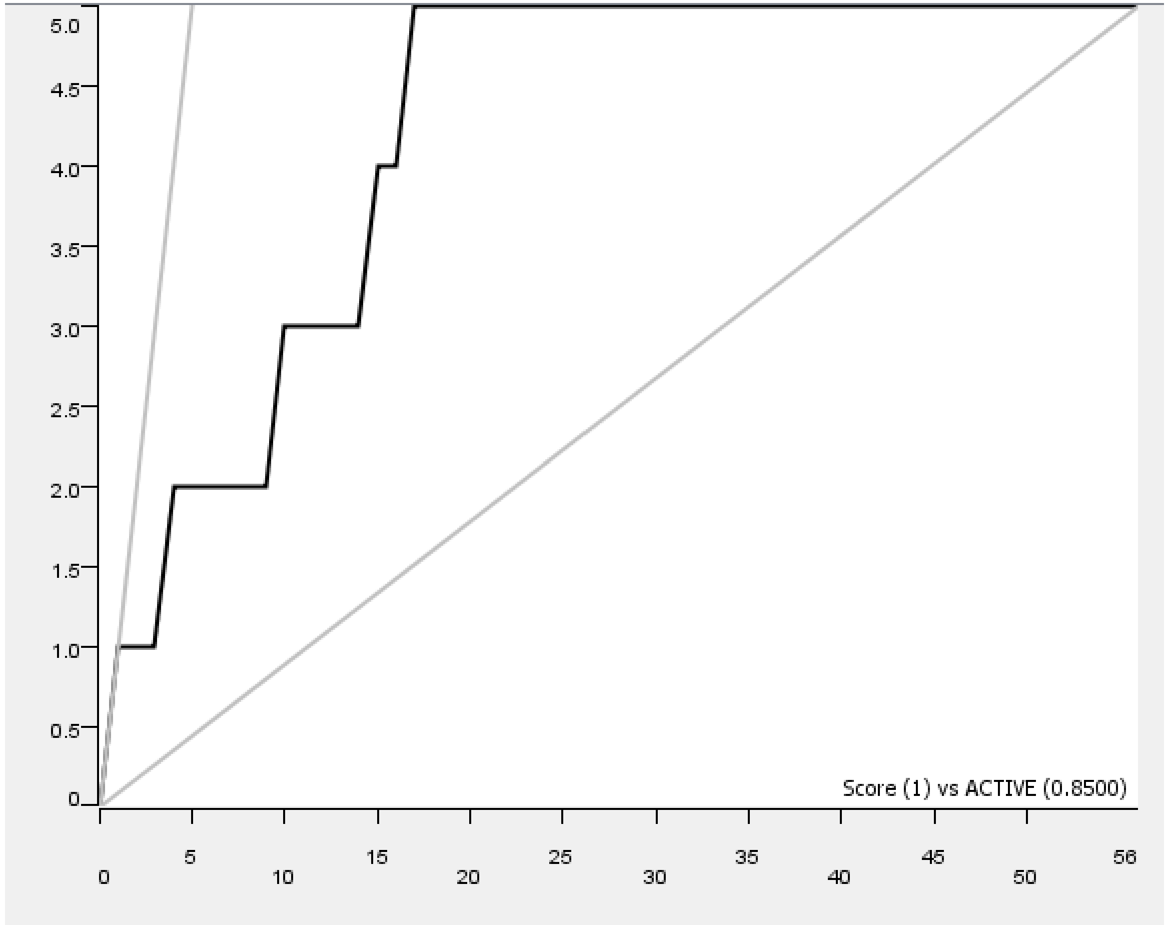

N=56

**Top 10% Mean IC50**	**1,100 nM**

	Predicted Active	Predicted Inactive
Active	5	0
Inactive	21	30

<25nM DefGood in TYRO3, 10% error; Random seed = 121783

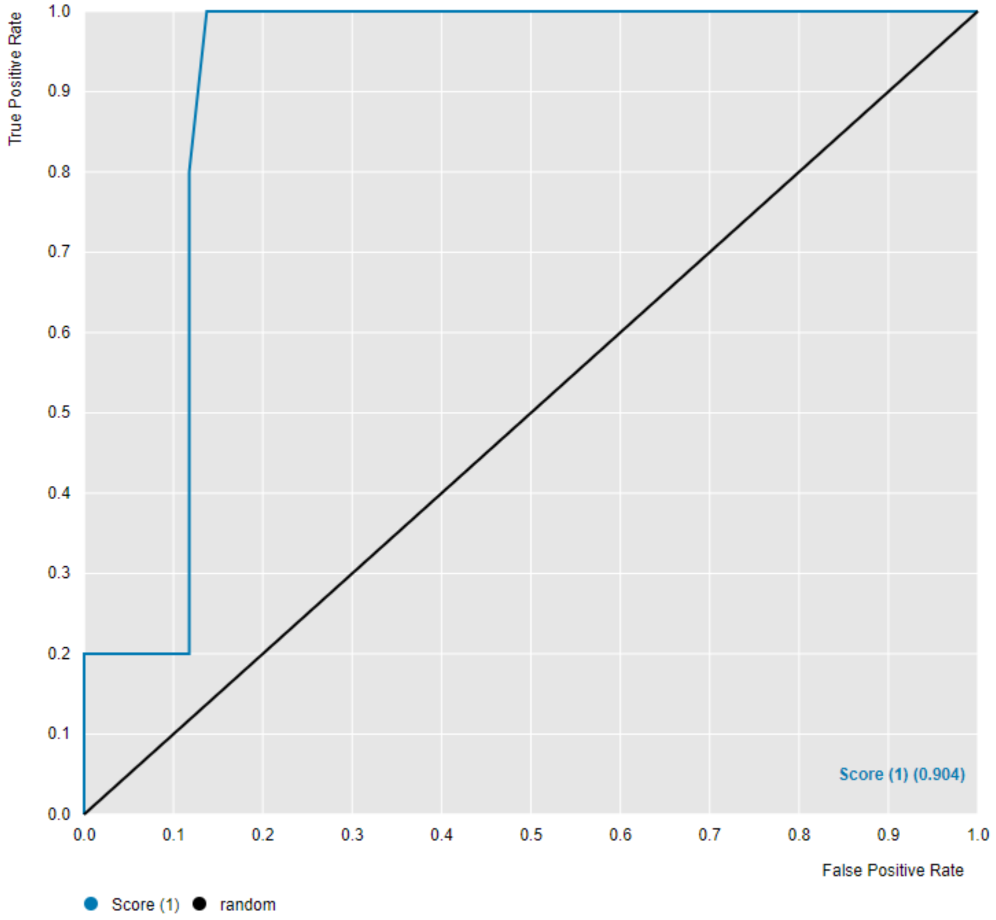

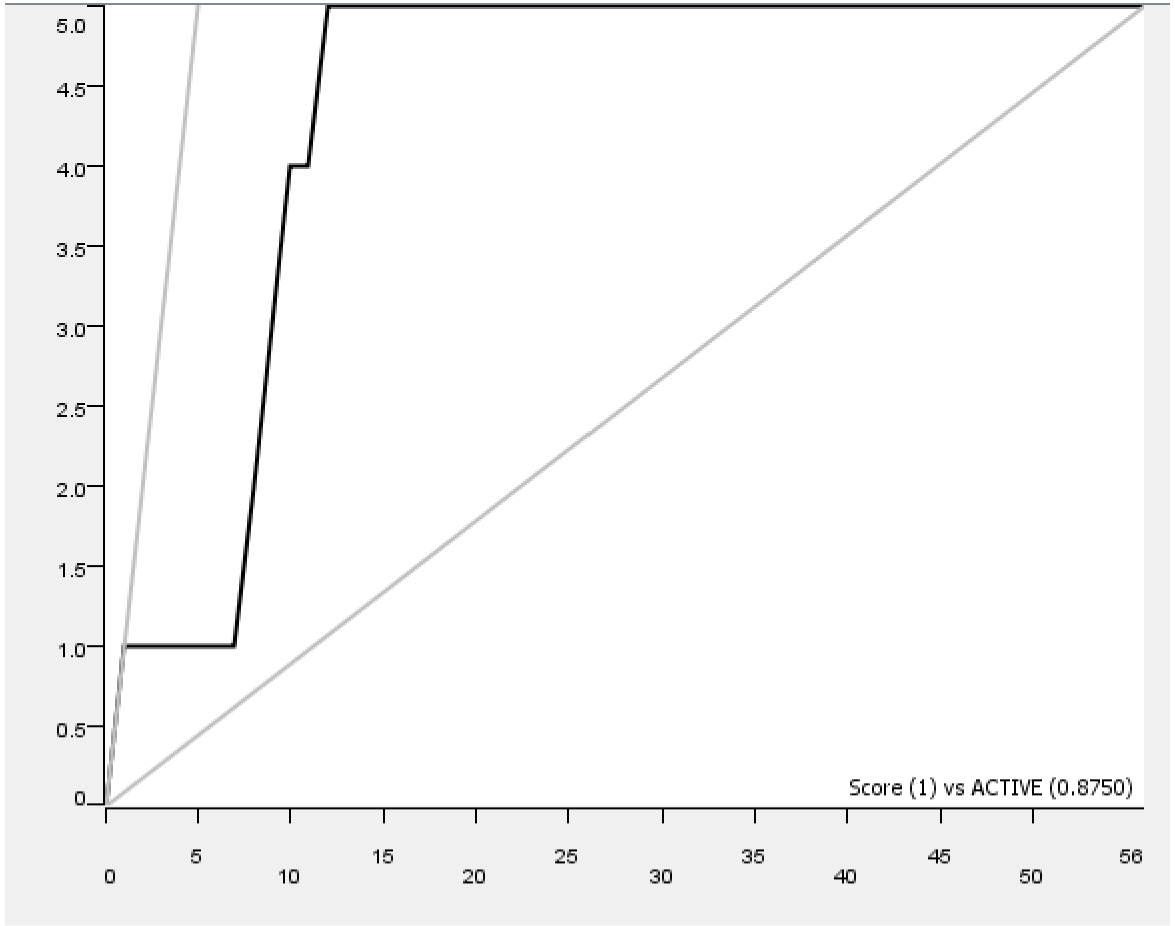

N=56

**Top 10% Mean IC50**	**915 nM One severe micromolar mistake**

	Predicted Active	Predicted Inactive
Active	5	0
Inactive	21	30

Conclusion - NBN

- An NBN could be generated for TYRO3 kinase with a decision value of <25 nM as defined as active. The predictivity of the algorithm could be preserved in three random train/test cycles. The point of failure was 30%, 10% and 5% error.

RF Error Tolerance- <25 nM DefGood in TYRO3

- 0-50% absolute error

RF- <25nM DefGood in TYRO3, 10% error

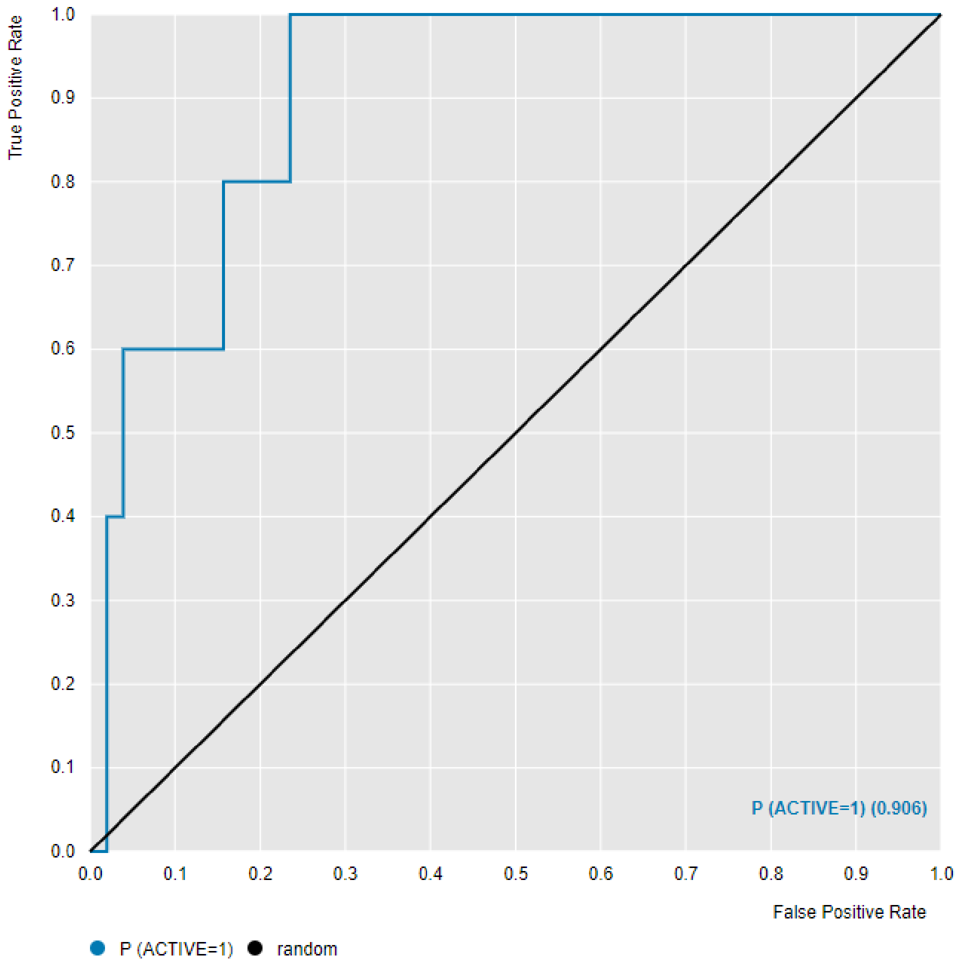

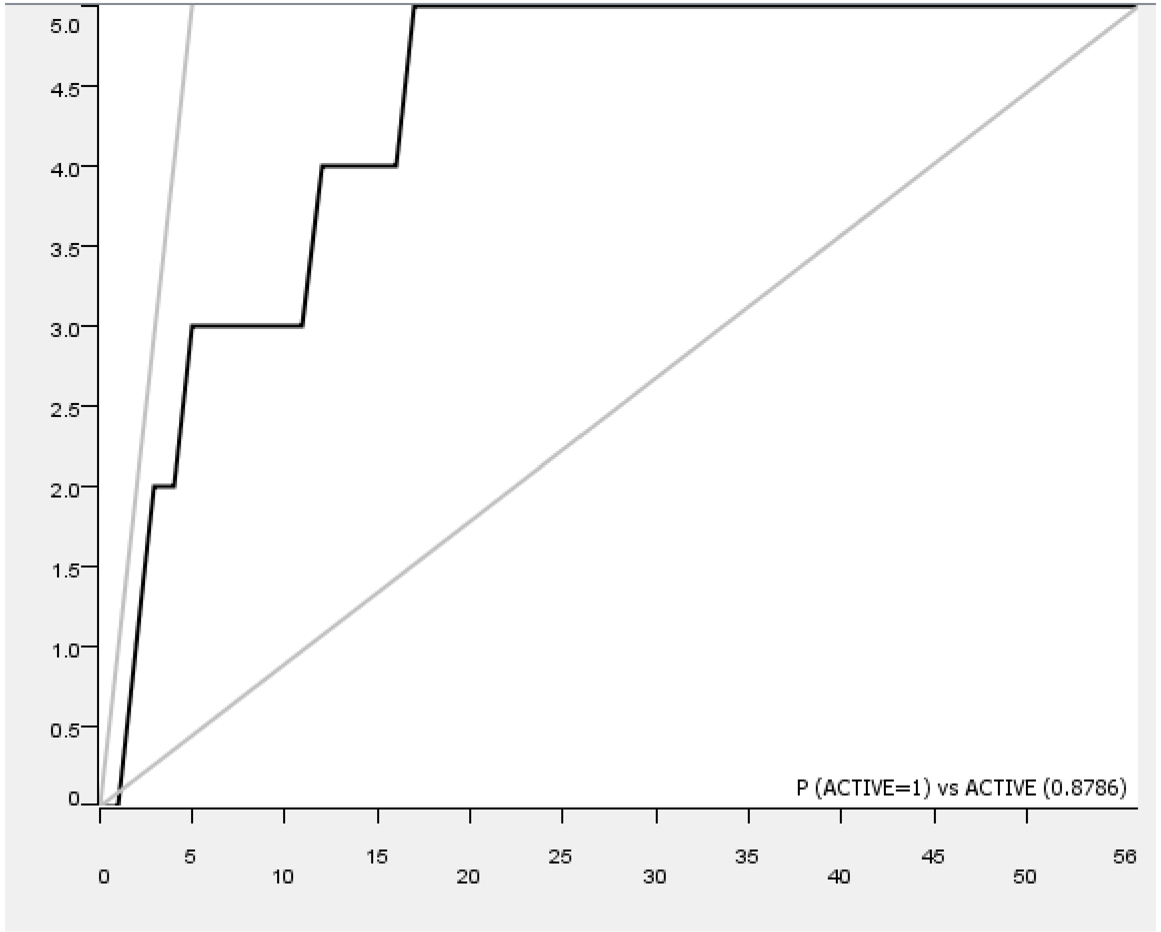

N=56

Top 10% Mean IC50	39.9 nM

	Predicted Active	Predicted Inactive
Active	3	2
Inactive	4	47

RF- <25nM DefGood in TYRO3, 15% error

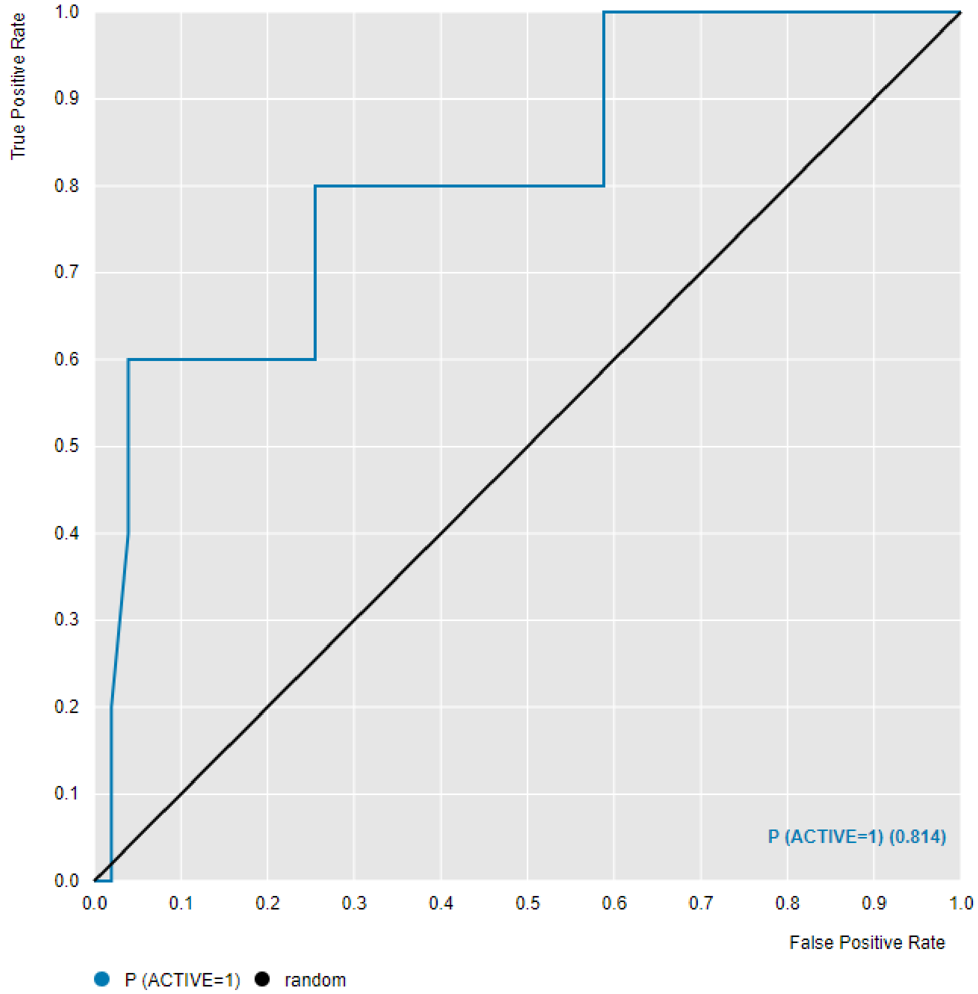

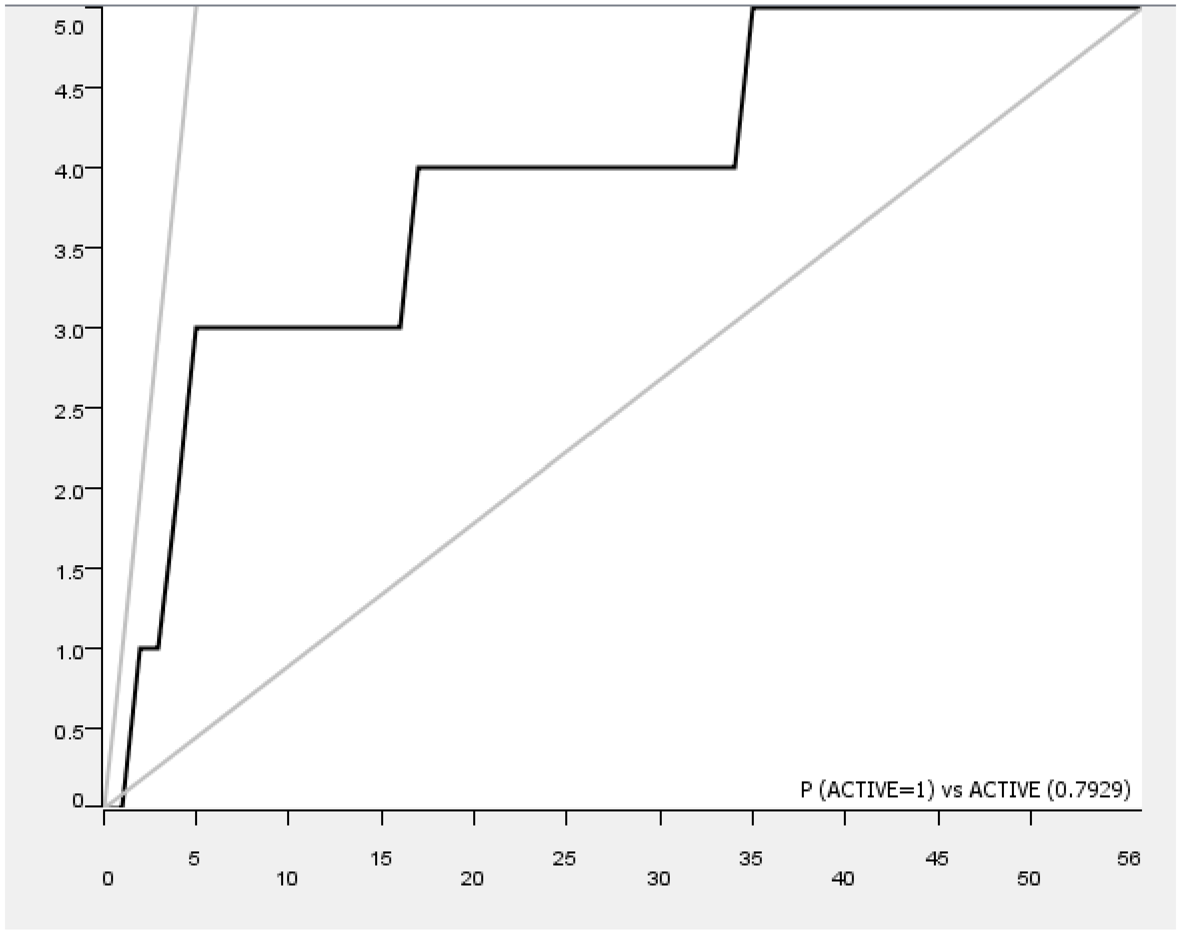

N=56

Top 10% Mean IC50	39.9 nM

	Predicted Active	Predicted Inactive
Active	3	2
Inactive	4	47

RF- <25nM DefGood in TYRO3, 20% error

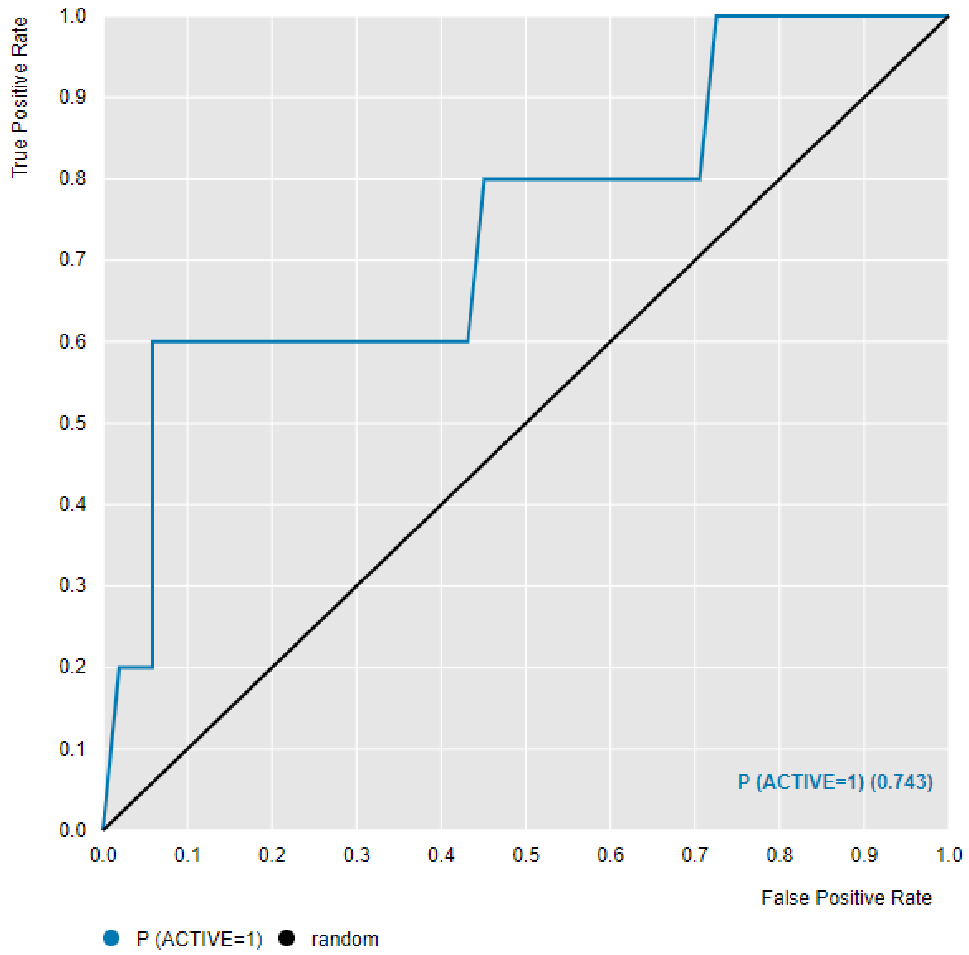

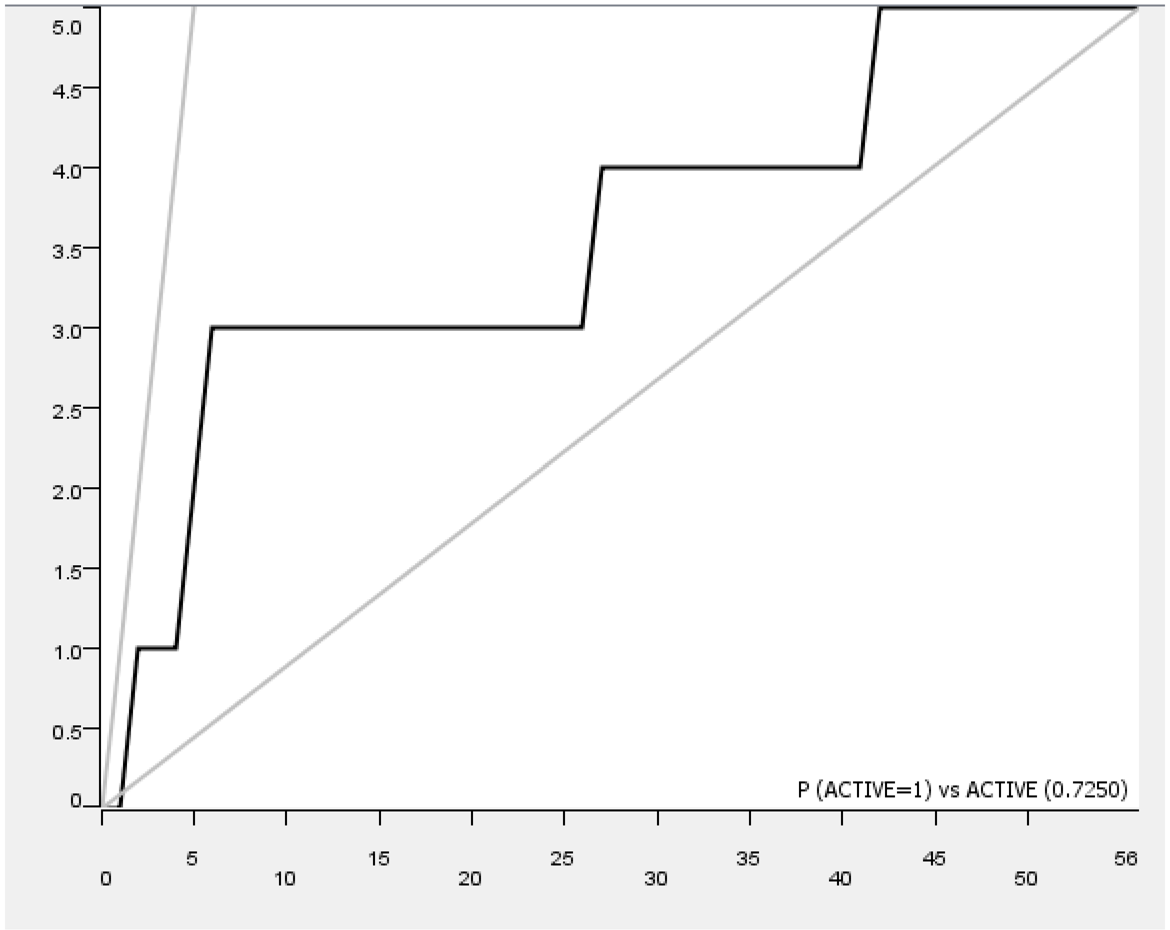

N=56

**Top 10% Mean IC50**	**3,900 nM**

	Predicted Active	Predicted Inactive
Active	3	2
Inactive	4	47

<25nM DefGood in TYRO3, 0% error; Random seed = 429

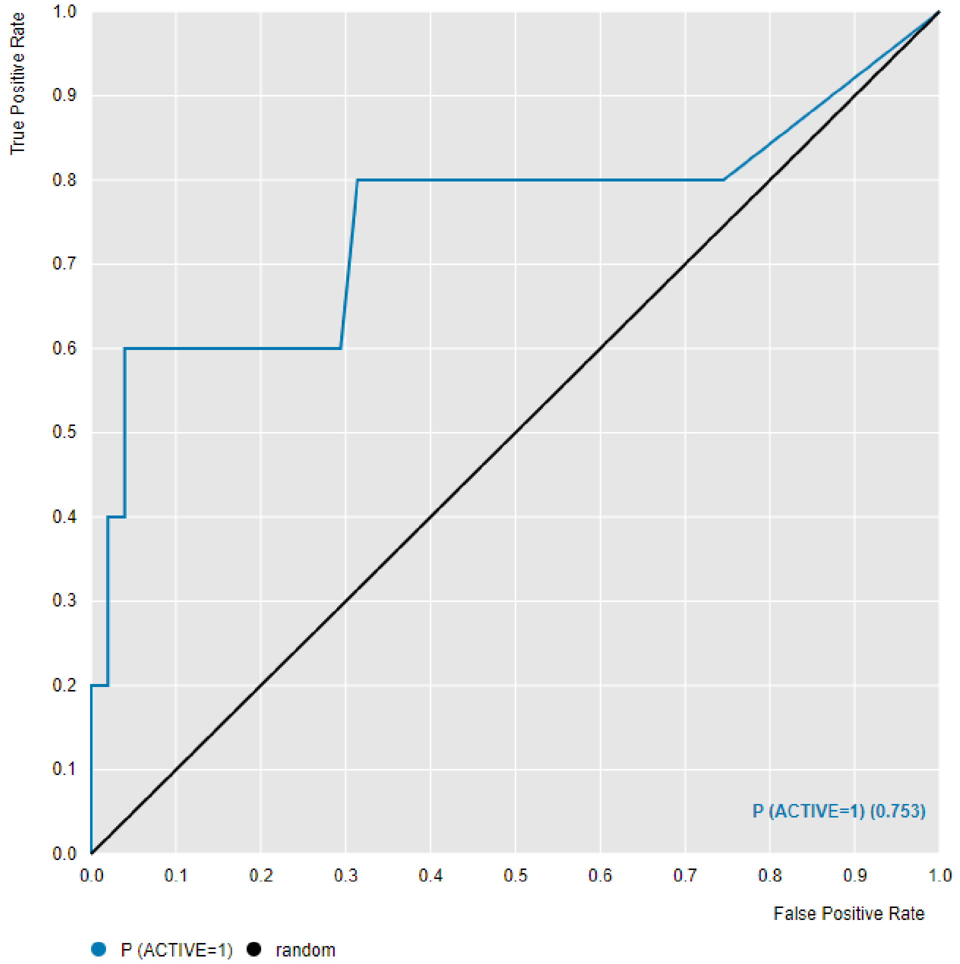

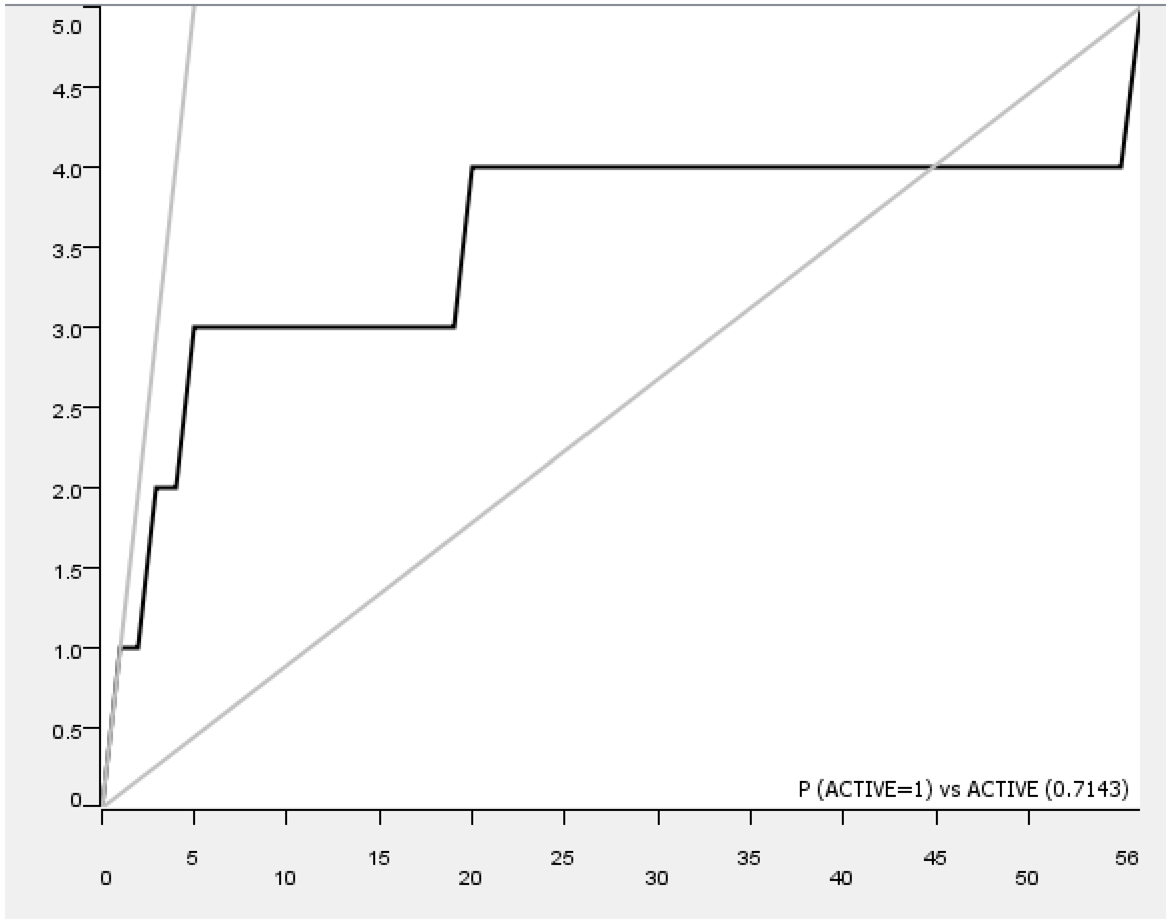

N=56

Top 10% Mean IC50	20.2 nM

	Predicted Active	Predicted Inactive
Active	1	4
Inactive	0	51

<25nM DefGood in TYRO3, 5% error; Random seed = 429

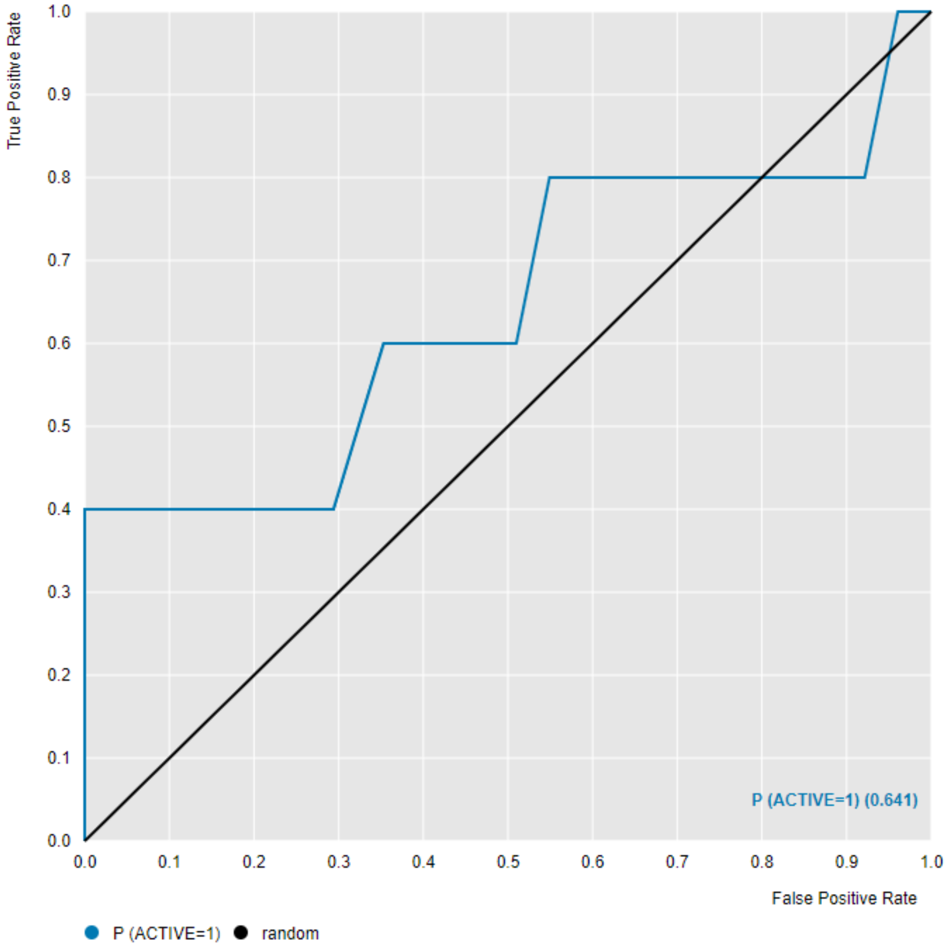

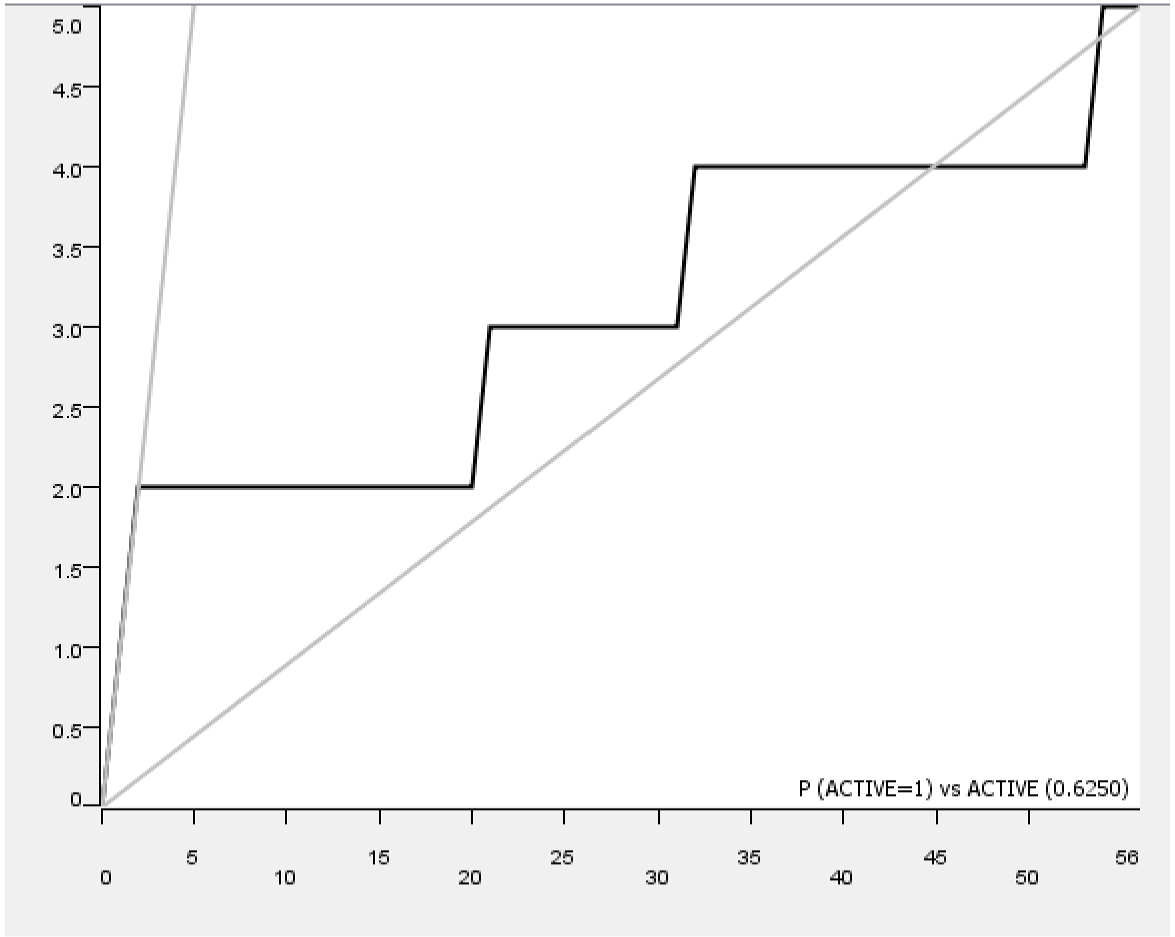

N=56

Top 10% Mean IC50	36.8 nM

	Predicted Active	Predicted Inactive
Active	0	5
Inactive	0	51

<25nM DefGood in TYRO3, 10% error; Random seed = 429

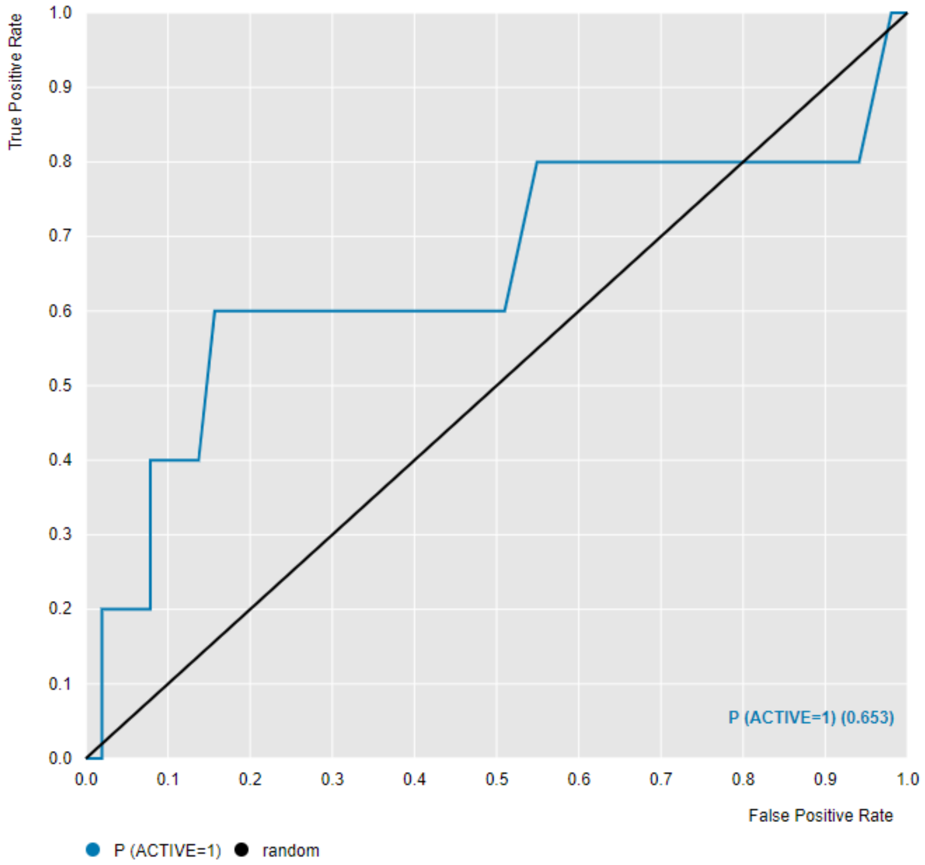

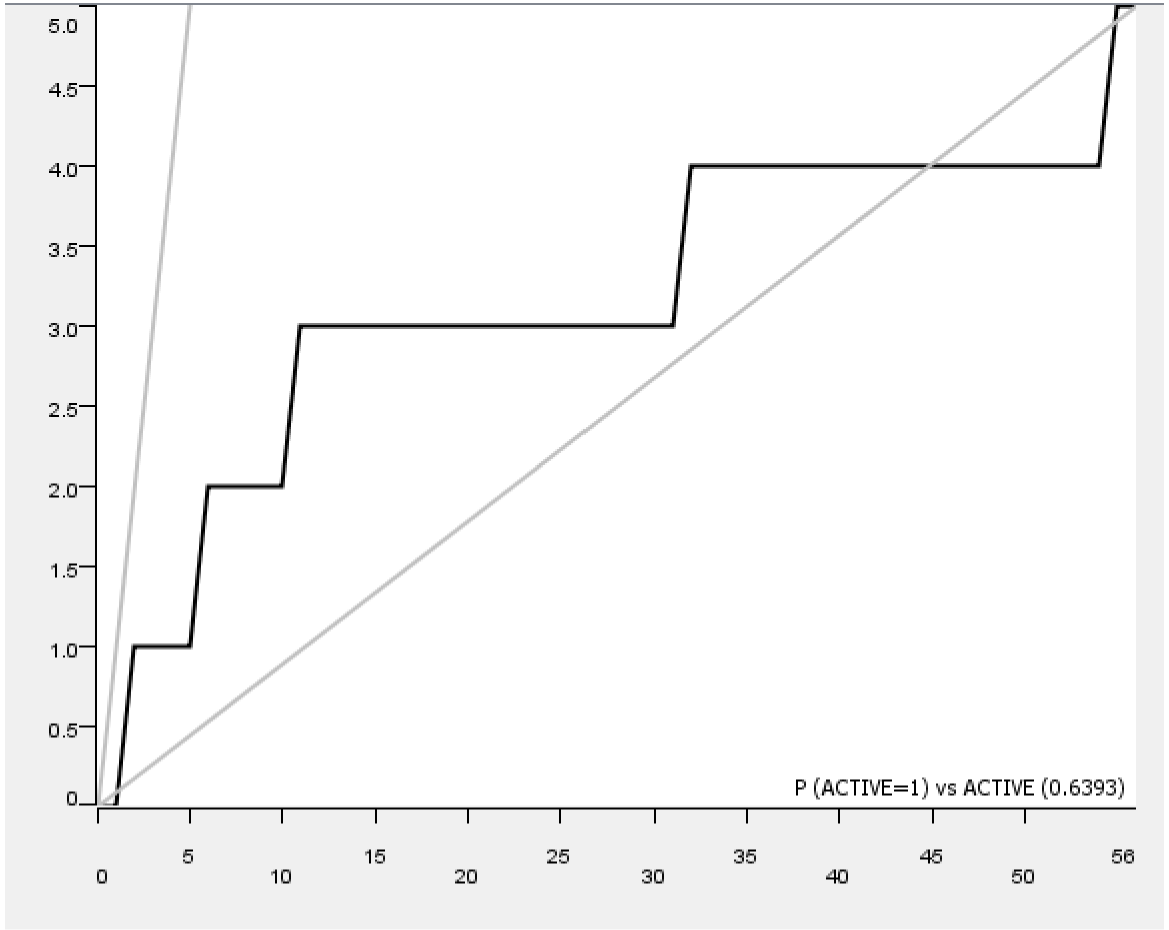

N=56

**Top 10% Mean IC50**	**63.8 nM**

	Predicted Active	Predicted Inactive
Active	0	5
Inactive	1	50

<25nM DefGood in TYRO3, 0% error; Random seed = 121783

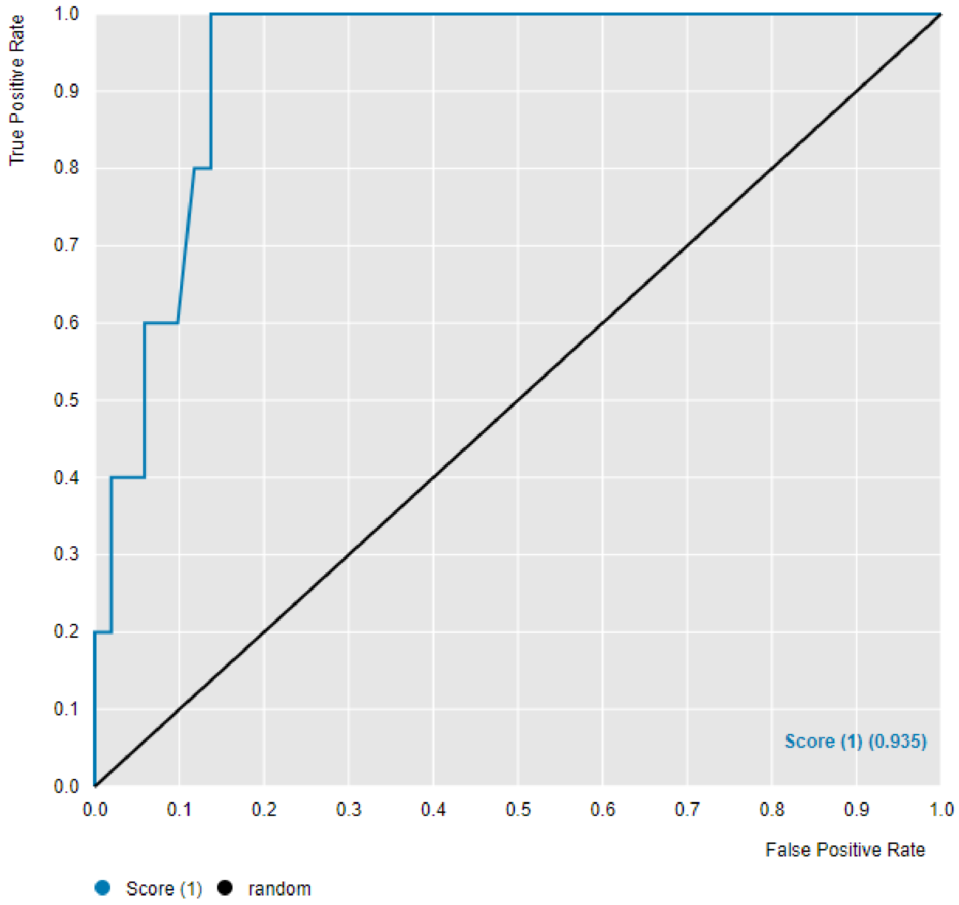

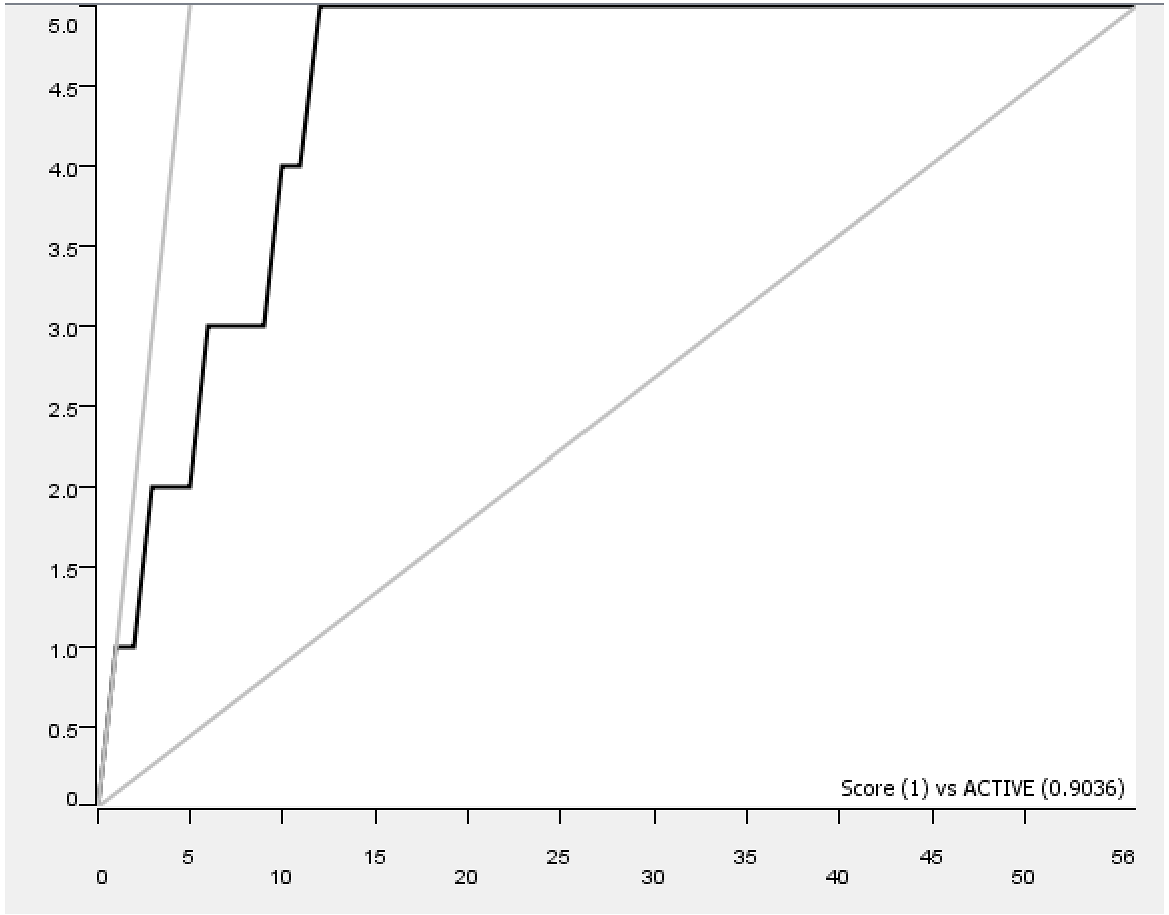

N=56

Top 10% Mean IC50	886 nM (one 4,300 nM mistake)

	Predicted Active	Predicted Inactive
Active	5	0
Inactive	13	38

- 121783 failed to give a good model in the RF, 12178 was used instead

<25nM DefGood in TYRO3, 0% error; Random seed = 12178

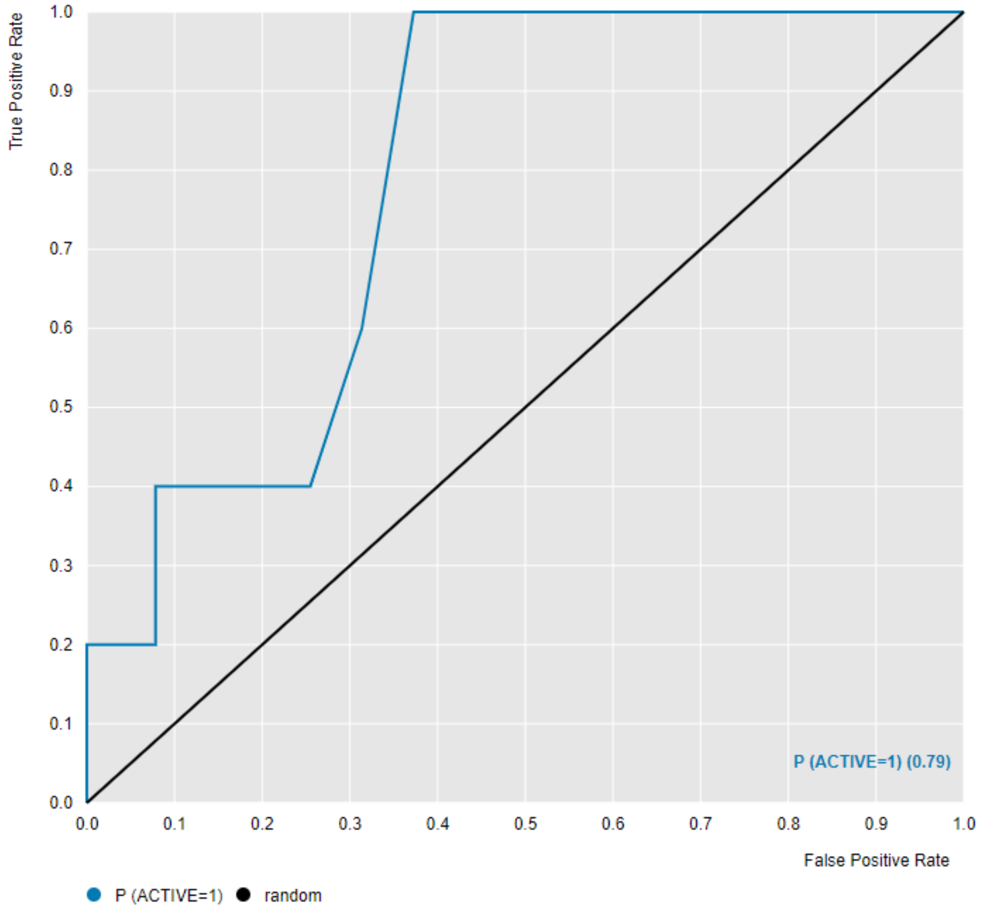

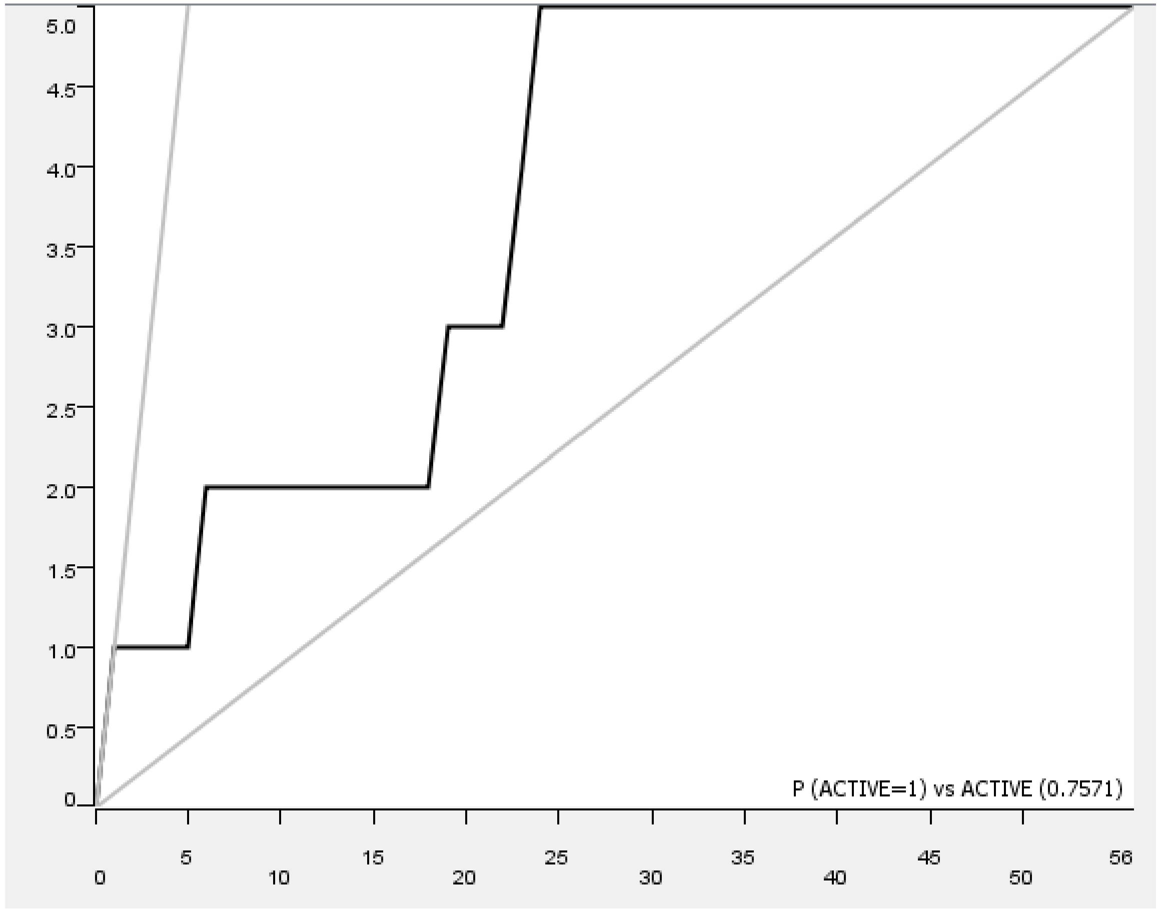

N=56

Top 10% Mean IC50	54.8 nM

	Predicted Active	Predicted Inactive
Active	1	4
Inactive	1	50

<25nM DefGood in TYRO3, 5% error; Random seed = 12178

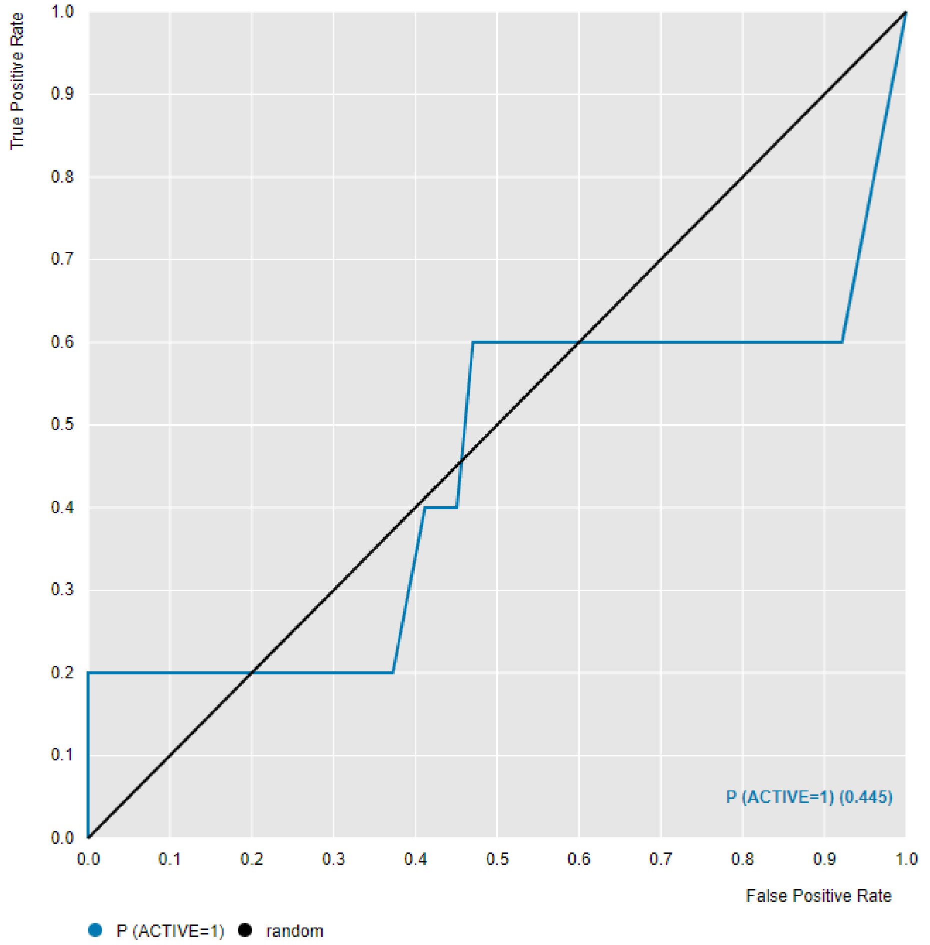

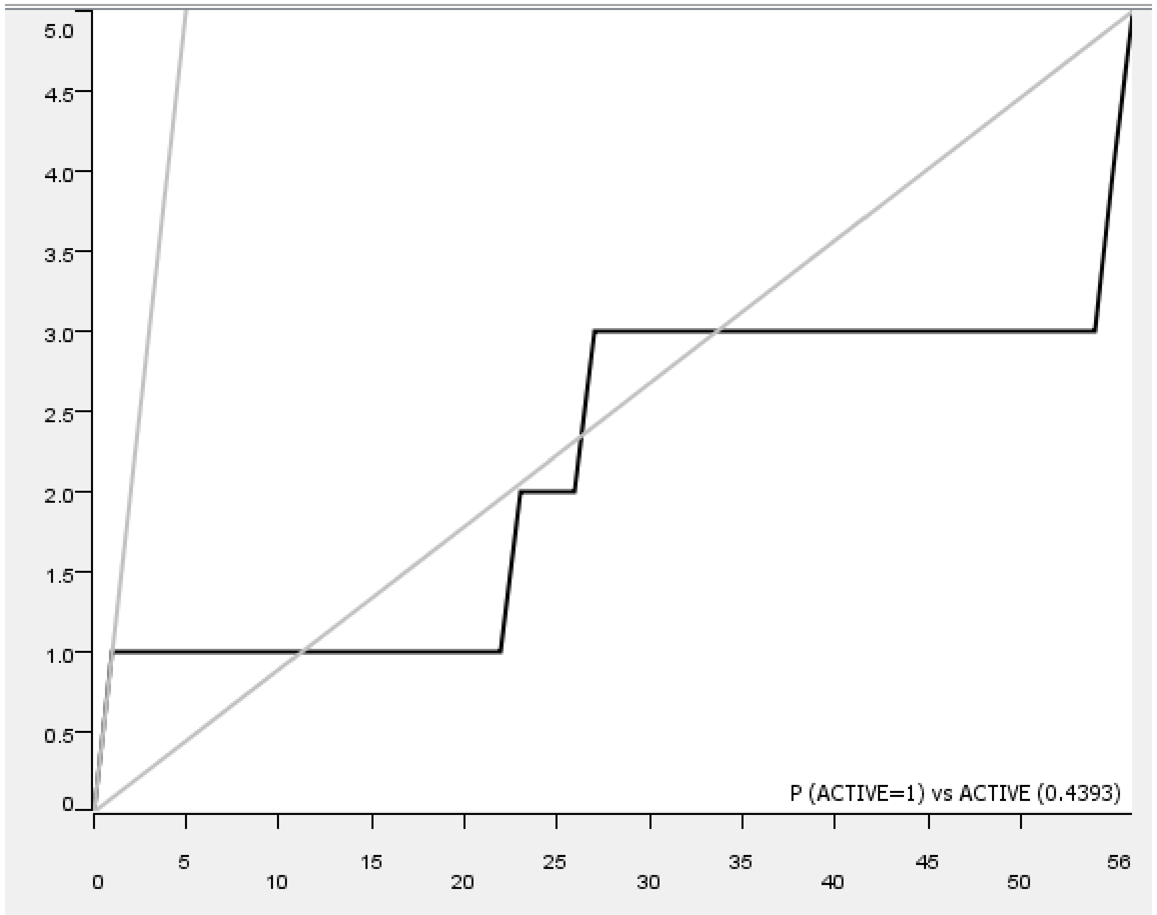

N=56

Top 10% Mean IC50	266.8 nM

	Predicted Active	Predicted Inactive
Active	1	4
Inactive	2	49

Conclusion - RF

- A Random Forrest could be generated for TYRO3 kinase with a decision value of <25 nM as defined as active. In two out of three cases, the algorithm could accommodate small amounts of error. In one case, a successful algorithm could not be generated as the original dataset with a split using 121783 as the seed generated a top 10% IC50 of >800 nM. Therefore, another seed (12178) was used, and this set was found to generate a useful NBN. The point of failure was 20%, 5% and 5% error.

Conclusion - PNN

- A PNN could not be used.
