# Supplementary material for: Error Tolerance of Machine Learning Algorithms across Contemporary Biological Targets
Source: Molecules. 2019 Jun 4;24(11):2115. doi: 10.3390/molecules24112115 (PMC6601015; doi:10.3390/molecules24112115)
Supplement: Supplementary file 1 [file molecules-24-02115-s001.zip › Supporting Information – Target Molecular Biology, Activity Distribution, Support V.pdf]

# Error Tolerance of Machine Learning Algorithms across Contemporary Biological Targets

## Supporting Information – Target Molecular Biology, Activity Distribution, Support Vector Machine Experiments and Calculations

Thomas M. Kaiser [1,*] and Pieter B. Burger [2,3,*]

[1] St Peter's College, University of Oxford, New Inn Hall St, Oxford OX1 2DL, United Kingdom

[2] Department of Drug Discovery and Biomedical Sciences, College of Pharmacy, Medical University of South Carolina, 280 Calhoun St. MSC 141 Charleston, SC 29425-1410, United States

[3] Department of Chemistry, Emory University, 201 Dowman Drive, Atlanta, GA 30322, United States

[*]Correspondence: thomas.kaiser@spc.ox.ac.uk; pieter.burger@gmail.com

# Summary of Potency Distribution for Biological Targets

Table S1: The Activity Distribution (pIC50) of the 10 Targets Studied.

| Targets | Min Activity | 25% | Median | 75% | Max Activity | N |
|---|---|---|---|---|---|---|
| ALK | 9.92 | 8.05 | 6.97 | 5.76 | 3.00 | 1343 |
| Factor Xa | 11.00 | 8.30 | 7.55 | 6.48 | 3.70 | 1657 |
| Aurora B | 9.70 | 7.64 | 6.76 | 5.54 | 4.00 | 1481 |
| B2AR* | 10.30 | 6.82 | 5.96 | 5.10 | 2.81 | 641 |
| c-ABL | 9.70 | 7.92 | 6.36 | 5.00 | 3.14 | 1439 |
| HIV Protease | 10.89 | 8.52 | 7.41 | 6.00 | 2.30 | 2544 |
| JAK2 | 10.26 | 7.80 | 7.13 | 6.35 | 1.30 | 3624 |
| MEK1 | 11.15 | 7.54 | 6.85 | 5.67 | 3.31 | 823 |
| PARP1 | 9.70 | 7.77 | 7.00 | 5.92 | 2.66 | 1933 |
| TYRO3 | 9.15 | 7.15 | 6.40 | 5.59 | 4.12 | 277 |

* β-2 Adrenergic Receptor

# Summary of Molecular Biology

**Activated factor Xa**

Factor $X_a$ is a vitamin-K dependent endopeptidase (serine protease) consisting 2 peptide chains crossed linked by a sulfide bridge [30–32]. The active site of Factor $X_a$ is subdivided into 4 pocket S1, S2, S3 and S4. Direct inhibitors of Factor $X_a$ bind to the S1and S4 pockets forming an L-shape [30–32].

**β-2 Adrenergic Receptor**

The β-2 adrenergic receptors (B2AR) are a 7-helix transmembrane receptor expressed on the cell surface and is a part of the G-protein coupled receptor (GPCR) family. GPCR's bind hormones and other endogenous ligands causing a cascade of signaling events within the cells that regulate cellular processes. The human B2AR is one of the best studied GPCR's and binds catecholamines through which it regulates essential physiological function in the nervous, cardiovascular, pulmonary and other systems. B2AR modulation can occur by applying agonist, antagonist and inverse agonist all three of which have been well studied [33]. The modulators all bind to the same binding pocket with different chemotypes resulting in diverse protein-ligand interactions driving the distinctive outcomes. In this study we look only at antagonists that bind to B2AR.

**c-Abl**

c-Ableson kinase (c-Abl) is a non-receptor tyrosine kinase and member of the larger Abl family of kinases involved in regulation of cell growth, survival, cell migration actin-polymerization and integrin signaling [34]. Until recently c-Abl has been mainly studied as an oncology target; however, its importance in neurodegenerative disease has made it an attractive target against Parkinson's and other diseases [34]. c-Abl is normally inactive in the cell and regulated by a unique mechanism that binds the myristoyl group intramolecularly [34,35]. There have been 16 compounds that entered clinical trials (phase I-IV) all of which bind to the ATP binding site (www.ebi.ac.uk).

**HIV protease**

HIV protease is an essential viral enzyme involved in cleaving polyproteins and play an important role in viral maturation. Inhibition of HIV protease is a fundamental part of the highly active antiretroviral therapy (HAART). There are 10 FDA approved HIV protease inhibitors all of which share similarity in it binding modes [36].

**Aurora B**

Aurora B is part of the Aurora kinase family which plays an important role in mitosis [37]. Together with survivin, borealin and INCENP, Aurora B forms the central component of the chromosomal passenger complex [38]. Aurora B has extensively been targeted in drug discovery efforts with an initial focus on solid tumors and later hematologic cancers [37]. Several kinase inhibitor have made it into the clinic that targets Aroura B and or the Aurora family of kinases [37]. The majority of Aurora B are ATP-competitive inhibitors with several compounds that have entered clinical trials [40].

**JAK2**

The Janus kinase (JAK) family consist of JAK1-3 and TYK2 kinases all of which is non-receptor protein tyrosine kinases [41]. The JAK family play an important role in the regulation of hematopoiesis, the

immune system and cellular metabolism [42]. Interesting is the unique feature of the JAK kinase family in having a pseudokinase domain that regulates function [42]. However, JAK2 is targeted by compounds binding to the kinase binding domain with one approved FDA drug, ruxolitinib [41]. Inhibitors of JAK2 are either type I or II binding to the ATP-binding site [44].

**TYRO3**

Tyrosine protein kinase receptor 3 (TYRO3) is a transmembrane receptor tyrosine kinase and part of the TAM (Tyro3/AXL/MER) family. These kinases are involved in regulations processes involving cell proliferation/survival, migration, platelet aggregation and immune regulation. AXL and MERKT has been extensively studied yet less in known about TYRO3 and it is considered as an emerging therapeutic target [45]. Most inhibitors of TYRO3 targets the kinase binding pocket of the TAM family with some inhibitors showing selectivity for TRYO3 [45].

**ALK**

Anaplastic lymphoma kinase (ALK) is a tyrosine receptor kinase and part of the insulin receptor superfamily [46]. ALK play an important role in development and function of the nervous system and activates multiple pathways and that effect cell growth transformation and anti-apoptotic signaling [46]. ALK is a classical receptor tyrosine kinase with an extracellular ligand binding domain, a transmembrane region and an intracellular tyrosine kinase binding domain. ALK is targeted by tyrosine kinase inhibitors and have been studied in various different cancers including neuroblastoma and non-small cell lung cancer [47].

**PARP1**

Poly (ADP-ribose) polymerase (PARP) is part of a multifunctional family of protein that are involved in cellular homeostasis where it modulates DNA repair, chromatin structure, transcription and replication [48,49]. PARP has a modular architect that binds to DNA [49]. Inhibitors of PARP predominately targets the catalytic domain of PARP1 and PARP2 however there are approximately 15 other PARP proteins that could be targeted [50].

**MEK1**

The dual specific mitogen-activated protein kinase kinase 1 (MEK1) is an enzyme involved in signal transduction pathways that regulates cell motility, proliferation, differentiation, survival and development [51]. MEK1 is a serine/threonine kinase and part of the highly conserved mitogen-activated protein kinases (MAPK) family. The most studied MEK1 inhibitors are non-classical type III kinase inhibitors that binds to the MEK binding pocket adjacent to the ATP binding site [52,53]. Trametinib and Cobimetinib are two MEK1 inhibitors that have been approved in the clinic.

# Properties Employed for PNN

These properties were calculated using the CDK and RDKit Modules in KNIME

Molecular Weight
AlogP
PSA
Number of Rule of 5 Violations
SlogP
SMR
LabuteASA
TPSA
AMW
ExactMW
Number of Lipinski Hydrogen Bond Acceptors
Number of Lipinski Hydrogen Bond Donors
Number of Rotatable Bonds
Number of Amide Bonds
Number of Heteroatoms
Number of Heavy Atoms
Number of Atoms
Number of Stereocenters
Number of Unspecified Stereocenters
Number of Rings
Number of Aromatic Rings
Number of Saturated Rings
Number of Aliphatic Rings
Number of Aromatic Heterocycles
Number of Saturated Heterocycles
Number of Aliphatic Heterocycles
Number of Aromatic Carbocycles
Number of Saturated Carbocycles
Number of Aliphatic Carbocycles
Fraction of C $sp^3$
Mannhold logP
Element Count
Charged Partial Surface Area
Eccentric Connectivity Index
Fragment Complexity
VABC Volumetric Descriptor
Hydrogen Bond Acceptors
Hydrogen Bond Donors
Largest Chain
Larges Pi Chain

Petitjean Number
Lipinski’s Rule of Five
Zagreb Index
Formal Charge
Formal Charge (Positive)
Formal Charge (Negative)

# Support Vector Machine Investigation

Materials and Methods

The dataset used in the exploration of a support vector machine (SVM) approach was the cleaned ALK dataset used in the evaluation of error tolerance for an NBN, PNN and RF in ALK. All cheminformatics processing and analysis, as well as machine learning, was performed in KNIME 3.7.0 and KNIME 3.7.1. The software was run on a Razer Blade 15 with an 8th Gen Intel core i7-8750H 6 core and 16 Gb of DDR4 system RAM. The first step involved filtering the data to ensure all compounds had explicitly defined activities that were reported as nM. Next, molecules that had an exact value for the $IC_{50}$, a value reported as <10 nM or a value >999 nM were retained as these molecules were either exactly known for $IC_{50}$ or were considered potent (<10 nM) or lacking potency (>999 nM). All molecules were sorted in order of increasing $IC_{50}$ and duplicate entries were removed. The molecular structure was generated using the CDK community expansion for KNIME. The molecules were then split according to whether they were <X where X is an $IC_{50}$ value that defines active and inactive categorically (20 nM in the case of the SVM experiment). The independent variables used for learning (46 in total) were calculated from the CDK molecular structure through the use of the RDKit Descriptor Calculation and the CDK Molecular Properties modules. The data generated from the RDKit and CDK modules were normalized through the use of the normalization module applying a min-max normalization step (0-1 as the minimum and maximum). The actives and inactives were each split into an 80% training set and a 20% test set.
The training data generated as above were fed into a 5-fold cross validation a Radial Basis Function (RBF) SVM, a hypertangent SVM and a polynomial SVM could be parameterized. These parameters were used in the SVM algorithm tested on the reserved 20% test data. All $IC_{50}$ values, publication data and other non-molecular data were removed from the training set after the active/inactive split so as to remove confounding variables.
The parameters discovered from the 5-fold cross validation were used to create an SVM with the independent variables calculated above and the category active/inactive as the dependent variable. The algorithm was then fed into the predictor module for the corresponding algorithm, and the performance of the SVM evaluated on the test set using the ROC Curve (Java Script) and Enrichment Plotter modules. Rule based modules were used to sort out the true positive/true negative/false positive/false negative statistics. The definition of good was selected in accordance with which definition of good performed best in the ROC AUC, sensitivity, specificity, top 10% mean $IC_{50}$ and enrichment characteristics.

Results

The SVM kernel initially explored was the RBF kernel. Parameterization of sigma in KNIME resulted in a value of 0.1 when the range to be explored was 0.1-1, a value of 0.01 when the range was 0.01-1 and a value of 0.001 for a range of 0.001 to 1. The sigma values of 0.01 and 0.001 were evaluated on the test data. An RBF SVM with a sigma of 0.001 was found to have a ROC AUC of 0.431 with all molecules in the test set predicted active. Additionally, an RBF SVM with a sigma of 0.01 was found to have a ROC AUC of 0.436 with all molecules in the test set predicted to be active. Next, we evaluated the hypertangent SVM with a kappa of 0.1 and a delta of 0.1. The ROC AUC was found to be 0.623 and all molecules in the test set were predicted to be active. Finally, we evaluated 1st order, 2nd order and 3rd order polynomial SVMs. The 1st order polynomial SVM (with a bias = 3 and gamma = 2.25 from parameterization) generated a ROC AUC of 0.49 with all compounds in the test set predicted to be inactive. The 2nd order

polynomial SVM (with a bias = 4.75 and gamma = 0.25 from parameterization) generated a ROC AUC of 0.49 with all compounds in the test set predicted to be inactive. The 3rd order polynomial SVM (with a bias = 3.5 and gamma = 1.0 from parameterization) generated a ROC AUC of 0.49 with all compounds in the test set predicted to be inactive. We are currently working to understand the failure of SVM for this target and classification problem; however, we did not continue with SVM as all three kernels failed to generate algorithms with retrospective predictive capacity.

# Calculations for Theoretical $IC_{50}$'s

$$\mathrm{IC}_{50} = K_i\left(1 + \frac{[S]}{K_\mathrm{m}}\right)$$

**Equation S1.** The Cheng-Prusoff equation relating $K_i$ *and* $IC_{50}$ for competitive inhibitors.

$$\Delta \mathrm{G}_\mathrm{A} = RT\ln K_i$$

**Equation S2.** Relationship between Gibbs free energy of binding and $K_i$.

$$\Delta \mathrm{G}_\mathrm{B} = \Delta \mathrm{G}_\mathrm{A} - \Delta\Delta \mathrm{G}_\mathrm{A,B}$$

$$\Delta \mathrm{G}_\mathrm{B} = RT\ln K_{i,theor}$$

$$K_{i,theor} = e^{\left(\frac{\Delta \mathrm{G}_\mathrm{B}}{RT}\right)}$$

∴

$$K_{i,theor} = e^{\left(\frac{\Delta \mathrm{G}_\mathrm{A} - \Delta\Delta \mathrm{G}_\mathrm{A,B}}{RT}\right)}$$

**Equation S3.** Relationship between the RBFE derived $\Delta\Delta G_{A,B}$ and the theoretical $K_{i,theor}$ for B.

$$K_i = \frac{\mathrm{IC}_{50}}{\left(1 + \frac{[S]}{K_\mathrm{m}}\right)}$$

Where $[S]$ = 300 µM of ATP
$K_m$ = 134 µM for ALK-ATP
$IC_{50}$ = 2 x $10^{-8}$ M as the definition of good

$$K_i = \frac{2 \times 10^{-8}\ \mathrm{M}}{\left(1 + \frac{300\ \mu\mathrm{M}}{134\ \mu\mathrm{M}}\right)}$$

$K_i$ = 6.18 x $10^{-9}$ M

**Calculation S1.** Determination of approximate $K_i$ value for a 20 nM definition of good.

$$\Delta G_{\text{Good}} = RT\ln K_i$$

$$\Delta G_{\text{Good}} = 1.987 \text{ x } 10^{-3} \frac{\text{kcal}}{\text{K mol}} (298 \text{ K}) \ln(6.18 \text{ x } 10^{-9})$$

$$\Delta G_{\text{Good}} = \text{-11.19 kcal/mol}$$

**Calculation S2.** Determination of approximate ΔG value for a 20 nM definition of good.

$$\text{IC}_{50,theor} = K_{i,theor}\left(1 + \frac{[S]}{K_\text{m}}\right)$$

$$K_{i,theor} = e^{\left(\frac{\Delta G}{RT}\right)}$$

$$\therefore$$

$$\text{IC}_{50,theor} = e^{\left(\frac{\Delta G}{RT}\right)}\left(1 + \frac{[S]}{K_\text{m}}\right)$$

For the minimum value of the ΔG window, -10.19 kcal/mol:

$$\text{IC}_{50,theor} = e^{\left(\frac{-10.19 \text{ kcal/mol}}{1.987 \text{ x } 10^{-3} \frac{\text{kcal}}{\text{K mol}} (298 \text{ K})}\right)} \text{M}\left(1 + \frac{300 \ \mu\text{M}}{134 \ \mu\text{M}}\right)$$

$$\text{IC}_{50,theor} = 109 \text{ nM}$$

For the maximum value of the ΔG window, -12.19 kcal/mol:

$$\text{IC}_{50,theor} = e^{\left(\frac{-12.19 \text{ kcal/mol}}{1.987 \text{ x } 10^{-3} \frac{\text{kcal}}{\text{K mol}} (298 \text{ K})}\right)} \text{M}\left(1 + \frac{300 \ \mu\text{M}}{134 \ \mu\text{M}}\right)$$

$$\text{IC}_{50,theor} = 3.7 \text{ nM}$$

**Calculation S3.** Determination of $\text{IC}_{50}$ minimum and maximum for error introduction.

## Reference:


30. Jyoti Sen, D. Xabans as Direct Factor Xa Inhibitors. *J. Bioanal. Biomed.* **2015**, *07*, 1–3.
31. Patel, N.R.; Patel, D. V.; Murumkar, P.R.; Yadav, M.R. Contemporary developments in the discovery of selective factor Xa inhibitors: A review. *Eur. J. Med. Chem.* **2016**, *121*, 671–698.
32. Yeh, C.H.; Fredenburgh, J.C.; Weitz, J.I. Oral direct factor xa inhibitors. *Circ. Res.* **2012**, *111*, 1069–1078.
33. Chan, H.C.S.; Filipek, S.; Yuan, S. The Principles of Ligand Specificity on beta-2-adrenergic receptor. *Sci. Rep.* **2016**, *6*, 1–11.
34. Brahmachari, S.; Karuppagounder, S.S.; Ge, P.; Lee, S.; Dawson, V.L.; Dawson, T.M.; Ko, H.S. C-Abl and Parkinson's Disease: Mechanisms and Therapeutic Potential. *J. Parkinsons. Dis.* **2017**, *7*, 589–601.
35. Yang, J.; Campobasso, N.; Biju, M.P.; Fisher, K.; Pan, X.Q.; Cottom, J.; Galbraith, S.; Ho, T.; Zhang, H.; Hong, X.; et al. Discovery and characterization of a cell-permeable, small-molecule c-Abl kinase activator that binds to the myristoyl binding site. *Chem. Biol.* **2011**, *18*, 177–186.
36. Lindholm, D.; Pham, D.D.; Cascone, A.; Eriksson, O.; Wennerberg, K.; Saarma, M. C-Abl inhibitors enable insights into the pathophysiology and neuroprotection in Parkinson's disease. *Front. Aging Neurosci.* **2016**, *8*, 6–11.
37. Wang, Y.; Lv, Z.; Chu, Y. HIV protease inhibitors: a review of molecular selectivity and toxicity. *HIV/AIDS - Res. Palliat. Care* **2015**, *7*, 95.
38. Bavetsias, V.; Linardopoulos, S. Aurora Kinase Inhibitors: Current Status and Outlook. *Front. Oncol.* **2015**, *5*, 1–10.
39. Elkins, J.M.; Santaguida, S.; Musacchio, A.; Knapp, S. Crystal structure of human aurora B in complex with INCENP and VX-680. *J. Med. Chem.* **2012**, *55*, 7841–7848.
40. Borisa, A.C.; Bhatt, H.G. A comprehensive review on Aurora kinase: Small molecule inhibitors and clinical trial studies. *Eur. J. Med. Chem.* **2017**, *140*, 1–19.
41. Hubbard, S.R. Mechanistic insights into regulation of JAK2 tyrosine kinase. *Front. Endocrinol. (Lausanne).* **2018**, *8*, 1–7.
42. Hammarén, H.M.; Ungureanu, D.; Grisouard, J.; Skoda, R.C.; Hubbard, S.R.; Silvennoinen, O. ATP binding to the pseudokinase domain of JAK2 is critical for pathogenic activation. *Proc. Natl. Acad. Sci.* **2015**, *112*, 4642–4647.
43. Leroy, E.; Constantinescu, S.N. Rethinking JAK2 inhibition: Towards novel strategies of more specific and versatile janus kinase inhibition. *Leukemia* **2017**, *31*, 1023–1038.
44. Smart, S.K.; Vasileidadi, E.; Wang, X.; DeRychere, D.; Graham, D.K. The Emerging Role of TYRO3 as A Therapeutic Target in Cancer. *Cancers (Basel).* **2018**, 1–27.
45. Powell, N.A.; Hoffman, J.K.; Ciske, F.L.; Kaufman, M.D.; Kohrt, J.T.; Quin, J.; Sheehan, D.J.; Delaney, A.; Baxi, S.M.; Catana, C.; et al. Highly selective 2,4-diaminopyrimidine-5-carboxamide inhibitors of Sky kinase. *Bioorganic Med. Chem. Lett.* **2013**, *23*, 1046–1050.
46. Della Corte, C.M.; Viscardi, G.; Di Liello, R.; Fasano, M.; Martinelli, E.; Troiani, T.; Ciardiello, F.; Morgillo, F. Role and targeting of anaplastic lymphoma kinase in cancer. *Mol. Cancer* **2018**, *17*, 1–9.
47. Zhao, Z.; Verma, V.; Zhang, M. Anaplastic lymphoma kinase: Role in cancer and therapy perspective. *Cancer Biol. Ther.* **2015**.
48. Sonnenblick, A.; De Azambuja, E.; Azim, H.A.; Piccart, M. An update on PARP inhibitors - Moving to the adjuvant setting. *Nat. Rev. Clin. Oncol.* **2015**, *12*, 27–41.
49. Morales, J.C.; Li, L.; Fattah, F.J.; Dong, Y.; Bey, E.A.; Patel, M.; Gao, J.; Boothman, D.A. "Action and

rationale for targeting in cancer and other diseases.” *Crit Rev Eukaryot Gene Expr* **2014**, *24*, 15–28.

50. Langelier, M.; Adp-ribosyl, P.; Planck, J.L.; Roy, S.; Pascal, J.M. Structural Basis for DNA. **2012**, *728*, 728–733.
51. Caunt, C.J.; Sale, M.J.; Smith, P.D.; Cook, S.J. MEK1 and MEK2 inhibitors and cancer therapy: The long and winding road. *Nat. Rev. Cancer* 2015.
52. Zhao, Z.; Xie, L.; Bourne, P.E. Insights into the binding mode of MEK type-III inhibitors. A step towards discovering and designing allosteric kinase inhibitors across the human kinome. *PLoS One* **2017**, *12*, 1–14.
53. Uitdehaag, J.C.M.; Verkaar, F.; Alwan, H.; De Man, J.; Buijsman, R.C.; Zaman, G.J.R. A guide to picking the most selective kinase inhibitor tool compounds for pharmacological validation of drug targets. *Br. J. Pharmacol.* **2012**, *166*, 858–876.
